# Identification, Characterization, and Expression Analysis of Cell Wall Related Genes in *Sorghum bicolor* (L.) Moench, a Food, Fodder, and Biofuel Crop

**DOI:** 10.3389/fpls.2016.01287

**Published:** 2016-08-31

**Authors:** Krishan M. Rai, Sandi W. Thu, Vimal K. Balasubramanian, Christopher J. Cobos, Tesfaye Disasa, Venugopal Mendu

**Affiliations:** ^1^Department of Plant and Soil Science, Fiber and Biopolymer Research Institute, Texas Tech UniversityLubbock, TX, USA; ^2^National Agricultural Biotechnology Research Center, Ethiopian Institute of Agricultural ResearchAddis Ababa, Ethiopia

**Keywords:** cell wall polymers, cellulose, hemicellulose, lignin, pectin, plant biomass, sorghum, abiotic stress

## Abstract

Biomass based alternative fuels offer a solution to the world's ever-increasing energy demand. With the ability to produce high biomass in marginal lands with low inputs, sorghum has a great potential to meet second-generation biofuel needs. Despite the sorghum crop importance in biofuel and fodder industry, there is no comprehensive information available on the cell wall related genes and gene families (biosynthetic and modification). It is important to identify the cell wall related genes to understand the cell wall biosynthetic process as well as to facilitate biomass manipulation. Genome-wide analysis using gene family specific Hidden Markov Model of conserved domains identified 520 genes distributed among 20 gene families related to biosynthesis/modification of various cell wall polymers such as cellulose, hemicellulose, pectin, and lignin. Chromosomal localization analysis of these genes revealed that about 65% of cell wall related genes were confined to four chromosomes (Chr. 1–4). Further, 56 tandem duplication events involving 169 genes were identified in these gene families which could be associated with expansion of genes within families in sorghum. Additionally, we also identified 137 Simple Sequence Repeats related to 112 genes and target sites for 10 miRNAs in some important families such as cellulose synthase, cellulose synthase-like, and laccases, etc. To gain further insight into potential functional roles, expression analysis of these gene families was performed using publically available data sets in various tissues and under abiotic stress conditions. Expression analysis showed tissue specificity as well as differential expression under abiotic stress conditions. Overall, our study provides a comprehensive information on cell wall related genes families in sorghum which offers a valuable resource to develop strategies for altering biomass composition by plant breeding and genetic engineering approaches.

## Introduction

Sorghum (*Sorghum bicolor*), a C4 grass species, is one of the world's important multipurpose cereal crops with uses in food, fodder, and biofuel industries. Sorghum with its relatively smaller genome size (~730 Mbp) makes it an ideal model bioenergy crop compared to other C4 crops such as switch grass, sugarcane or miscanthus with bigger and more complex genomes. Grain sorghum is grown worldwide with an annual production of 62 million tons of grain yield from an estimated area of 42 million hectares (FAOSTAT data 2013; http://faostat3.fao.org). Biomass yield of energy sorghum (fodder sorghum) is twice that of the grain sorghum due to longer vegetative growth period, increased leaf area which helps in greater radiation interception and efficiently converting the synthesized carbon into cell wall polysaccharides (Olson et al., 2012). Apart from food, livestock feed, and biofuel source, sorghum is also a source of malt for brewing and various food industries (Taylor et al., 2006). With a higher biomass yield potential (15–40 Mg/hc), sorghum can be used as high value energy source in second generation biofuel industry (Rooney et al., 2007). Due to its high adaptability to different environmental conditions such as drought, salinity, water-logging, ability to grow in marginal land areas, efficient light to biomass energy conversion rate, and nitrogen utilization rate, sorghum is emerging as favorite multipurpose crop in recent years (Taylor et al., 2010; Byrt et al., 2011).

The focus of second generation biofuels is to produce biofuels from lignocellulosic material, which is derived mainly from plant cell walls. Lignocellulosic material is mainly composed of cellulose (15–40%), hemicellulose (30–40%), lignin (20–30%), and pectins (Mendu et al., 2011b). These structural polymers composition varies between primary and secondary cell walls, different tissues of an individual plant and among different plant species (Mendu et al., 2011b; Welker et al., 2015). Primary cell wall (PCW) is present in all plant cell types whereas secondary cell wall (SCW) is present in specific cell types such as tracheary elements (TE) and sclerenchymal cells. Cellulose, the most abundant polymer on earth, is a homopolymer of β-(1,4)-linked glucose monomers (Cosgrove, 2005; Somerville, 2006) whereas hemicelluloses are branched heteropolymers of pentose and hexose sugar monomers (Burton et al., 2010; Ochoa-Villarreal et al., 2012). Pectin, a complex polymer consists of α-(1–4)-linked D-galacturonic acid backbone, is another polysaccharide which is mainly present in primary cell walls. Three classes of pectins, based on the nature of the sugars on the branches have been known in plants; homogalacturonans (HG), rhamnogalacturonans-I (RG-I), and rhamnogalacturonans-II (RG-II; Burton et al., 2010). The cellulose microfibrils are cross-linked with various matrix polysaccharides such as hemicelluloses and pectins thereby forming a complex polymeric network to maintain the cell wall strength (Cosgrove, 2005; Muthamilarasan et al., 2015). In addition to cellulose, hemicellulose and pectin, plant secondary cell walls are enriched with lignin. Lignin is a complex aromatic heteropolymer synthesized mainly from three canonical hydroxycinnamyl alcohol monomers viz. *p*-coumaryl (H), coniferyl (G), and sinapyl (S) alcohols (Boerjan et al., 2003; Vanholme et al., 2010; Welker et al., 2015). Lignin is ester- and ether-linked with cellulose and hemicellulose polysaccharides in the plant cell walls with the help of ferulic acid (Harris and Trethewey, 2010). Callose, another β-1,3-linked glucan polymer, is present in the cell walls of specialized structures involved in pollen development, cell wall formation during cytokinesis, and plasmodesmatal canals (Nedukha, 2015). Apart from developmental deposition, callose is deposited in response to various external stimuli including biotic and abiotic stresses (Chen and Kim, 2009; Muthamilarasan et al., 2015).

Cell wall biosynthesis, reassembly, and degradation are complex processes, which involves cell wall biosynthetic, modification, and degrading enzymes. Cellulose is synthesized by plasma membrane localized cellulose synthase complexes while other matrix polysaccharides such as hemicelluloses and pectins are synthesized in Golgi complex followed by their transport and cross-linking/embedding which involves cell wall biosynthetic, modifying and degrading enzymes. Cell wall hydrolyzing enzymes produced by bacteria, fungi, and nematodes (Rai et al., 2015) degrade plant cell walls to gain entry into the plant cell and access the sugars for their survival while the cell wall hydrolyzing enzymes produced by plant cells are primarily involved in controlled cleavage of wall polymers to facilitate cell growth and elongation (Cosgrove, 2005). Carbohydrate Active enZymes (CAZy; http://www.CAZy.org/) database broadly classified cell wall enzymes into 135 families of Glycoside Hydrolases (GHs), 98 families of Glycosyl Transferases (GTs), 24 families of Polysaccharide Lyases (PLs), 16 families of Carbohydrate Esterases (CEs), and 13 families of Auxiliary Activities (AAs) enzymes based on the presence of protein catalytic or functional domains (Lombard et al., 2014). Some other web based databases such as Cell Wall Navigator (Girke et al., 2004) and Cell Wall Genomics (https://cellwall.genomics.purdue.edu/families/index.html) further classified these enzymes into different groups based on biological processes in which they are involved.

Most of the enzymes involved directly in polysaccharide biosynthesis belong to the glycosyl transferases. Glycosyl transferases form glycosidic bonds by catalyzing the transfer of sugar moieties from donor to accepter molecules (Scheible and Pauly, 2004). Cellulose microfibrils are synthesized exclusively by cellulose synthases A (CESA) protein complexes, which belong to GT2 family of enzymes. Apart from CesA genes, *Cellulose synthase like* (Csl) genes are also found in plants which are involved in hemicellulose and other glucan biosynthesis (Lerouxel et al., 2006). Among the other hemicellulose biosynthetic enzymes, xyloglucan α-1,6-xylosyltransferases (GT34), xyloglucan fucosyltransferases (GT37), xyloglucan galactosyltransferases (GT47) are involved in synthesis of various xylan and xyloglucan molecules (Zhong and Ye, 2003; Del Bem and Vincentz, 2010; Vuttipongchaikij et al., 2012; Zabotina et al., 2012; Voiniciuc et al., 2015). The pectin biosynthetic galacturonosyltransferases (GT8) genes such as *FRAGILE FIBER8, IRREGULAR XYLEM8*, and *IRREGULAR XYLEM9* are reported to be involved in glucuronoxylan biosynthesis (Lee et al., 2007; Yin et al., 2010). In addition to the regular cell wall polymers, callose, a β-1,3-glucan, which is deposited by the callose synthase (glucan synthase like; Gsls) belongs to the GT48 family (Farrokhi et al., 2006; Muthamilarasan et al., 2015). Integration of new polymers into the cell wall through synergistic action of biosynthesis and wall loosening process is essential in order to maintain the integrity during the cell elongation process (Cosgrove, 2005). This loosening and reassembly is accomplished by the combined action of various degrading enzymes such as glycoside hydrolases (Buchanan et al., 2012; Glass et al., 2015; Wei et al., 2015), pectin lyases (Jiang et al., 2013), xyloglucan endotransglucosylases/hydrolases (XTH; Rose et al., 2002; Nishitani and Vissenberg, 2007), and cell wall loosening proteins such as expansins (Cosgrove, 2015; Marowa et al., 2016), and yieldins (Okamoto-Nakazato et al., 2001). In sorghum, a total of 12 CesA and 36/37 Csl genes have been reported in previous studies (Paterson et al., 2009; Yin et al., 2009). Characterization of sorghum (1,3; 1,4)-β-glucan biosynthetic gene subfamilies CslF and CslH showed that *CslF6* plays an important role in elongating cells while *CslH3* has a major role in cells that has stopped growth and started depositing storage compounds (Ermawar et al., 2015a). In a recent study, genes encoding cellulose, lignin, and glucuroarabinoxylan biosynthetic enzymes were dynamically expressed during the different development stages of sorghum (McKinley et al., 2016). The expansins and XTHs encoding genes were also shown to be differentially expressed in the growing stem internodes of sorghum. One of the glycosyl hydrolases gene families, endo-(1,4)-β-glucanase (GH9) has been studied across 5 grass genomes and 24 members were reported from sorghum (Buchanan et al., 2012).

The focus of second-generation biofuel production from plant biomass is to utilize the sugars from lignocellulosic material for biofuels, in particular for bioethanol production. In order to utilize the lignocellulosic biomass for bioethanol production, the cell wall polysaccharides need to be separated from lignin, hydrolyzed by polysaccharide degrading enzymes to produce fermentable sugars, a process called saccharification (Lin and Tanaka, 2005). The presence of interlinked lignin around cell wall polysaccharides contributes to biomass recalcitrance by hindering the enzyme access to polysaccharides (Ermawar et al., 2015b). Separation of lignin from other cell wall polysaccharides requires pretreatment with concentrated acids at high temperatures. In addition, the presence of hydroxyl groups in the cellulose units allows intra and intermolecular hydrogen bonding which makes the structure more crystalline. Either decreasing the lignin content or reducing the cellulose crystallinity or both will improve saccharification efficiency. A comparative analysis of lignin biosynthesis related gene families have been done across plant kingdom including sorghum (Xu et al., 2009). In sorghum, several mutants (*bmr, brown midrib*, and *rg, red for green*) with reduced lignin content showed increase in saccharification and digestibility compared to control plants (Palmer et al., 2008; Xin et al., 2008; Saballos et al., 2009; Yan et al., 2012; Petti et al., 2013; Sattler et al., 2014). Among these *bmr* mutants, several loci have been identified which includes *bmr2* encoding 4-coumarate: coenzyme A ligase (4CL), *bmr6* encoding cinnamyl alcohol dehydrogenase (CAD), and *bmr12* and *bmr18* encoding caffeic acid O-methyltransferase (COMT) enzymes of monolignol pathways (Saballos et al., 2009; Sattler et al., 2009; Scully et al., 2016). The sorghum biomass digestibility and saccharification efficiency can be further improved by targeting various genes involved in lignin biosynthesis coupled with genes that alter the cellulose crystallinity. Apart from lignin related gene families, CesA, Csls, and Gsls are among the most studied cell wall related gene families in sorghum. As the research on function of cell wall genes in model crop *Arabidopsis* is advancing, it is now essential to identify and characterize the cell wall related genes in sorghum to engineer sorghum biomass for food, feed and biofuel and bioproduct applications.

The present study focuses on mining of publically available *S. bicolor* genome for identification and comprehensive analysis of gene families involved in the biosynthesis of cell wall biopolymers. In addition, various other gene families involved in degradation and reassembly of cell walls have also been analyzed. Further, phylogenetic analysis, physical mapping, and duplication analysis of identified genes have been performed in order to get insight into the relation among the genes and their origin. All the identified genes were also analyzed for the presence of SSR markers and miRNA target sites for molecular breeding and biotechnological applications. Publically available transcriptome datasets from various tissues were analyzed to study the expression pattern of these genes. Furthermore, to understand the expression pattern of cell wall related gene families under abiotic stress condition, differential expression analysis of exogenous abscisic acid (ABA), and polyethylene glycol (PEG) treated tissues were also performed. The identification and analysis of cell wall related gene families in the present study would help the research community in planning effective strategies for more efficient utilization of biomass for various applications.

## Materials and methods

### Data retrieval and identification of cell wall related gene families

Publically available sequences of gene, protein, and chromosomes were downloaded from the Phytozome 11 database (https://phytozome.jgi.doe.gov/pz/portal.html#; Goodstein et al., 2012) for the identification and analysis of cell wall related gene families in sorghum. Protein sequences from other plants were downloaded from the Cell Wall Navigator database (Girke et al., 2004) to build the family specific HMM profile using HMMER v3.1b1 package (http://www.ebi.ac.uk/Tools/hmmer/). We first performed the multiple alignment of downloaded family specific sequences using Clustal Omega (http://www.ebi.ac.uk/Tools/msa/clustalo/) and saved the output alignment as ^*^.stockholm files. Using the family specific ^*^.stockholm alignment file as input for hmmbuild script we built the family specific HMM profiles (Data Sheet 1). Sorghum proteome was screened to identify the protein sequences related to various cell wall related families using HMMER with default parameters. All the identified proteins were screened for presence of their characteristic pfam domains. The successful candidate proteins were further verified for the presence of conserved domains using NCBI's Conserved Domain Database (CDD; (http://www.ncbi.nlm.nih.gov/Structure/cdd/wrpsb.cgi; Marchler-Bauer et al., 2011). Additionally, *Arabidopsis* cell wall related proteins were used as query to search the sorghum genome using blastP with an *e*-value of 10^−5^ and further validated with CDD search. The identified protein sequences from HMMER analysis were compared to blast identified proteins to prepare gene family specific non-redundant gene list. The coding and amino acid sequences of all the identified members were retrieved from sorghum genome dataset obtained from Phytozome database and used for the further analysis. The molecular weight and pI-values of all the identified proteins were calculated using online tool Compute pI/Mw (http://web.expasy.org/compute_pi/; Gasteiger et al., 2005).

### Phylogeny, physical mapping, and duplication analysis of cell wall related genes

The protein sequences of individual families were used for multiple sequence alignment using ClustalW program of MEGA v6 package (Tamura et al., 2013). Individual phylogenetic tree was constructed for the individual gene families with the MEGA v6 using neighbor-joining method. Bootstrap test was performed with 1000 iterations. To map physical locations of the identified cell wall related genes on sorghum chromosomes, their genomic coordinates along with chromosome number were retrieved from the file (*.gff*) downloaded from Phytozome database. The physical localization of genes was performed using the Mapchart 2.30 software (Voorrips, 2002). Furthermore, all the identified genes were analyzed for tandem duplications within the genome using the Plant Genome Duplication Database (http://chibba.agtec.uga.edu/duplication/) dataset (Lee et al., 2012).

### Identification of SSR markers in cell wall related genes

Coding sequences of all the cell wall related genes were used for SSRs identification using microsatellite identification tool (MISA, http://pgrc.ipk-gatersleben.de/misa/misa.html). The criteria for SSR search was repeat stretches having a minimum of five repeat units for dinucleotide (DNRs), trinucleotide (TNRs), tetranucleotide (TtNRs), pentanucleotide (PNRs), and hexanucleotide (HNRs). Mononucleotide repeats (MNRs) were excluded from the analysis. The maximum distance between two markers in a compound microsatellite was set to 100.

### *In silico* prediction of miRNA target sites on cell wall related genes

The identified cell wall related genes from individual families were analyzed for the presence of miRNA target sites using psRNATarget server (http://plantgrn.noble.org/psRNATarget/; Dai and Zhao, 2011). The maximum expectation values of 3.0 with other default parameters were used to perform the analysis.

### Expression analysis of cell wall related genes at various developmental stages of sorghum

Publically available transcriptome datasets from different developmental stages of sorghum (stem, 20 days old leaves, vegetative meristem, floral meristem, spikelet, flowers, embryos, and seeds) were downloaded from NCBI's Short Read Archive (SRA) database (http://www.ncbi.nlm.nih.gov/sra). All the transcriptome datasets were mapped on cell wall related genes using the QSeq program of DNASTAR Lasergene package (http://www.dnastar.com/t-nextgen-qseq.aspx). For the mapping purpose, 520 gene sequences related to cell wall gene families were exclusively used as reference. Transcript abundance was visualized by MeV (http://www.tm4.org/mev.html) generated hierarchical clustered heat map for individual gene families using the self-normalized RPKM (Reads Per Kilobase per Million reads) values calculated by the QSeq program.

### Differential gene expression analysis under various abiotic stress conditions

The role of identified cell wall related sorghum genes in abiotic stress conditions (exogenous ABA and PEG induced osmotic stress) in root and shoot was analyzed using publically available transcriptome datasets (Dugas et al., 2011). In brief, the published experiments were performed by germinating the *S. bicolor* BTx623 seeds and treating the seedlings on the 8th day after germination with 20 μM ABA (dissolved in NaOH), 57.1 μM NaOH (control for ABA), 20% PEG-8000, and Milli-Q (control for PEG treatment). After 27 h of treatment total RNA was extracted from the shoots and roots in three biological replicates and sequenced using the Illumina platform. Respective data sets of stress treated tissues along with controls were downloaded from SRA database of NCBI (Dugas et al., 2011). The expression pattern of cell wall genes was analyzed by using QSeq program of DNASTAR Lasergene package with self-normalized RPKM method. Fold change was calculated by using RPKM values of H_2_O and NaOH treated root and shoot tissues as controls for PEG and ABA, respectively. All the differentially expressed genes were analyzed for statistical significance using the Student's *t*-test with multiple-hypothesis testing at less than 0.05. Significantly differentially expressed genes (fold change ≥2.0, *p* < 0.05) from different stress conditions were used to find commonly up or down regulated genes from root and shoot using the online tool Venny 2.1 (http://bioinfogp.cnb.csic.es/tools/venny/).

## Results

### Identification of cell wall related genes from sorghum

**Figure 1:**
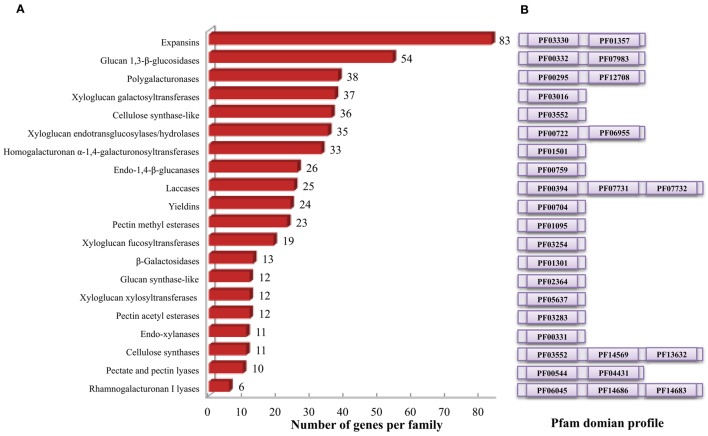
**Cell wall related gene families in sorghum. (A)** Distribution of identified cell wall related genes among various gene families. **(B)** Family specific pfam domains used to identify cell wall related genes using HMMER.

Cell wall related gene families have been shown to play crucial roles in various biological processes related to plant development, biotic and abiotic stress responses (Hamann, 2012; Lombard et al., 2014; Le Gall et al., 2015). Lignin biosynthetic gene families of sorghum have been analyzed elsewhere (Xu et al., 2009), hence, the present study focused on the gene families involved in various cell wall related processes such as polysaccharide synthesis and reassembly and degradation (Girke et al., 2004). Additionally, previously unreported laccase genes that are involved in lignin biosynthesis, were also analyzed in the present study. All 47,205 protein-coding transcripts and proteins from publically available *S. bicolor* genome were downloaded and analyzed. HMMER search identified a total of 520 genes from 20 cell wall related gene families with an average of 26 genes per family (Table 1, Figure 1, and Supplementary Table 1). Among the analyzed gene families, expansin with 83 members was the largest gene family whereas rhamnogalacturonan I lyases (CAZy ID: PL4) was the smallest family with 6 members (Table 1 and Figure 1A). According to the CAZy distribution, the total identified genes were classified into glycosyl transferases (160), glycoside hydrolases (201), pectin/pectate lyases (16), carbohydrate esterases (35), auxiliary activity (25), and expansin (83) families (Table 1).

**Table 1:** **Details of identified cell wall related gene families in ***Sorghum bicolor*****.

**Substrates**	**Gene families**	**Abbre**	**CAZy ID**	**No. of genes**
**POLYSACCHARIDE SYNTHESIS**				
Cellulose	Cellulose synthases	CESA	GT2	11
Hemicellulose	Cellulose synthase-like	CSL	GT2	36
	Xyloglucan xylosyltransferases and galactomannan gal-transferases	XXT	GT34	12
	Xyloglucan fucosyltransferases (MUR2)	XFT	GT37	19
	Xyloglucan galactosyltransferases (MUR3)	XGT	GT47	37
Pectin	Homogalacturonan α-1,4-galacturonosyltransferases	GAUT	GT8	33
Lignin	Laccases	LAC	AA1	25
Callose	Glucan synthase-like (Callose synthases)	GSL	GT48	12
**REASSEMBLY AND DEGRADATION**				
Cell wall loosening	Expansins	EXP		83
	Yieldins		GH18	24
	Xyloglucan endotransglucosylases/hydrolases	XTH	GH16	35
Glycoside hydrolases	Endo-1,4-β-glucanases		GH9	26
	Endo-xylanases		GH10	11
	Glucan 1,3-β-glucosidases		GH17	54
	Polygalacturonases	PGases	GH28	38
	β-Galactosidases	BGAL	GH35	13
Pectin modifying	Pectate and pectin lyases		PL1	10
	Rhamnogalacturonan I lyases		PL4	6
	Pectin methyl esterases	PME	CE8	23
	Pectin acetyl esterases	PAE	CE13	12
Total	20			520

#### I. cell wall polysaccharide biosynthetic gene families of sorghum

Plant cell wall biosynthesis is a complex process involving plethora of enzymes resulting in the biosynthesis of vast variety of cross linked cell wall polysaccharides. Majority of these enzymes belong to glycosyl transferases superfamily that is involved in the synthesis of cellulose, hemicellulose, pectin and callose.

##### Cellulose biosynthetic genes

**Figure 2:**
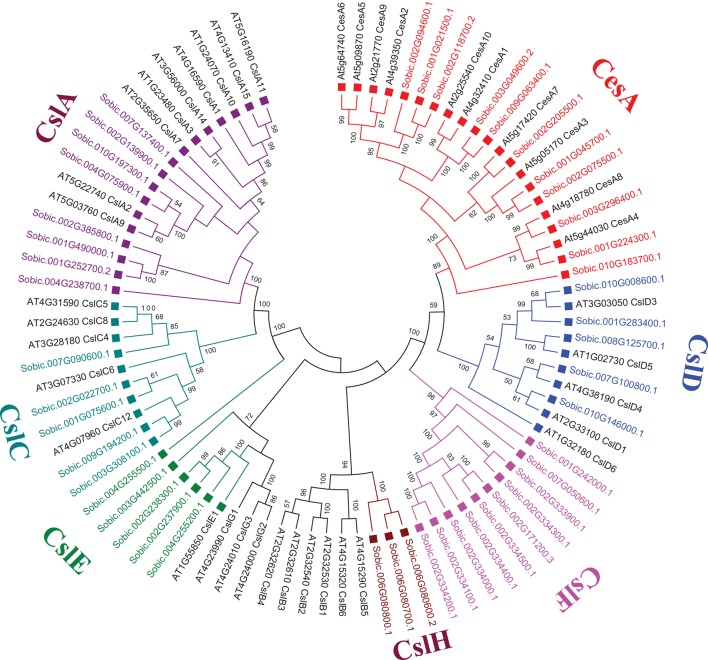
**Unrooted phylogenetic tree representing Cellulase synthase (CesA) and Cellulase synthase-like (Csl) gene family from ***S. bicolor*** and ***A. thaliana*** using MEGA6**. Tree was constructed using Neighbor Joining method with 1000 times bootstrap value. Same colored blocks and text represents the genes from similar gene-subfamilies.

Genome-wide analysis of sorghum showed presence of 11 CesA genes. In contrast to the 12 CesA genes reported in the previous studies (Paterson et al., 2009; Yin et al., 2009), the present study identified only 11 SbCesA genes using conserved domain profiles based HMMER scanning of sorghum genome (Table 1 and Figure 1B). Further investigation showed, according to the updated sorghum genome (v3.1, phytozome), the previously predicted two CesA genes (Sb03g004310.1 and Sb03g004320.1) are indeed a single gene (Sobic.003G049600.2). In addition, domain analysis of these 11 CESA proteins showed the presence of canonical cellulose synthase (CS, PF03552), zinc-binding RING-finger (PF14569), and glycosyl transferase 2 (PF13632) domains (Figure 1B). Majority of the CESAs (9/11) showed presence of all canonical domains, while Sobic.003G296400.1 showed lack of ZF domain and Sobic.010G183700.1 showed lack of ZF and GT2 domains. Eukaryotic CesA genes were first cloned from cotton (Pear et al., 1996) and have been later reported from *Arabidopsis* (10), maize (12), poplar (18), and 14 in foxtail millet (Richmond and Somerville, 2000; Appenzeller et al., 2004; Djerbi et al., 2005; Muthamilarasan et al., 2015). Cluster analysis of CESA proteins found to be clustered with CSLD and CSLF proteins (Figure 2) consistent with earlier reports (Ermawar et al., 2015a). The clustering is due the presence of common conserved domains among cellulose synthase and cellulose synthase like family of proteins. Chromosomal distribution of CesA genes showed presence of 4 and 3 genes on chromosome 2 and 1, respectively while remaining four are present on chromosome 3 (2 genes), 9 (1 gene), and 10 (1 gene; **Figure 4A**).

##### Hemicellulose biosynthetic genes

**Figure 3:**
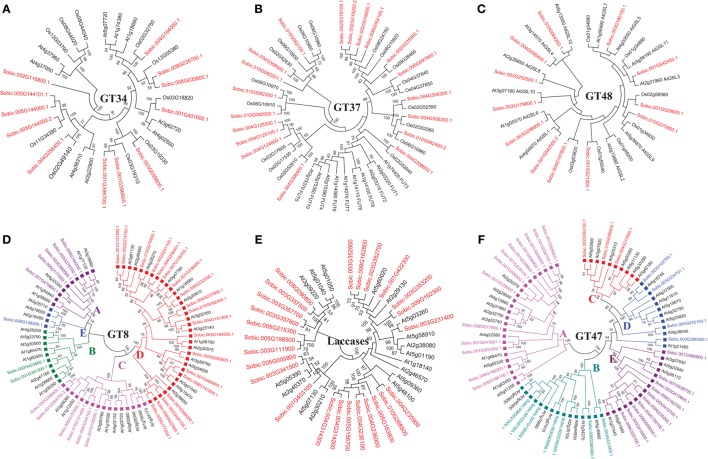
**Unrooted phylogenetic tree representing other cell wall related biosynthetic gene families. (A)** Xyloglucan xylosyltransferases; GT34 **(B)** Xyloglucan fucosyltransferases; GT37 **(C)** Glucan synthase-like; GT48 **(D)** Homogalacturonan α-1,4-galacturonosyltransferases; GT8 **(E)** Laccases; AA1 **(F)** Xyloglucan galactosyltransferases; GT47. Same colored blocks and text represents the genes from similar gene-subfamilies.

**Figure 4:**
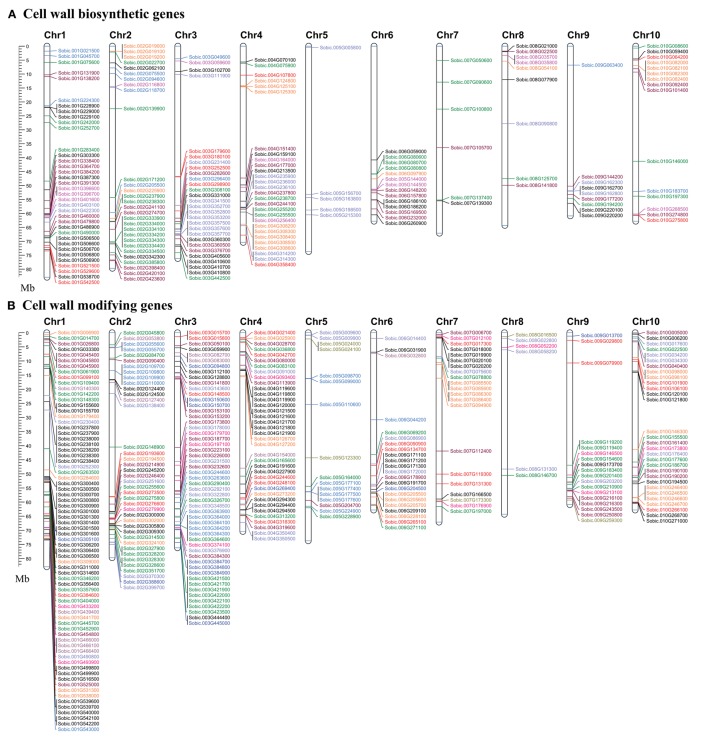
**Map showing the chromosomal location of cell wall related genes in sorghum. (A)** Cell wall biosynthetic genes. **(B)** Cell wall modifying genes. Genes with name in same color represents same gene family. A scale in the left represents length of chromosome in megabases (Mb).

Sorghum genome showed presence of four hemicellulose biosynthetic enzyme families i.e., cellulose synthase like (Csl; GT2), xyloglucan xylosyltransferases (XXT; GT34), xyloglucan fucosyltransferases (MUR2, XFT; GT37), and xyloglucan galactosyltransferases (MUR3, XGT; GT47). A total of 104 genes representing, Csl (36), XXT (12), MUR2 (19), and MUR3 (37) gene families were identified (Table 1, Figure 1A, and Supplementary Table 1). Further, domain analysis of these genes revealed the presence of CS (PF03552) domain in CSL, presence of GT34 (PF05637) domain in XXT, presence of XG_Ftase (PF03254) domain in MUR2, and exostosin (PF03016) domain in MUR3 family proteins (Figure 1B). Phylogenetic analysis of CSL proteins with *Arabidopsis* CSLs clustered them into 6 different sub-families namely, CSLA with 8, CSLC, CSLD, and CSLE with 5 each, CSLF with 10 and CSLH with 3 members (Figure 2). Phylogenetic analysis of other hemicellulose related gene families XXT (GT34) and XFT (GT37) showed their uniform clustering with *Arabidopsis* homologs (Figures 3A,B). Phylogenetic analysis of sorghum XGT (GT47) members with *Arabidopsis* homologs further clustered them into 5 subfamilies, A, B, C, D, and E with 13, 7, 3, 4, and 10 members, respectively (Figure 3F). Physical mapping of Csl family genes showed its distribution over all chromosomes except Chr. 5 (Figure 4A). Majority of the Csl genes, almost one-third (13), were found to be present exclusively on Chr. 2. Physical mapping of GT34 family members showed their distribution over six chromosomes (Chr. 1, 2, 3, 4, 5, and 8) whereas GT37 members were found to be present on five chromosomes (Chr. 2, 4, 6, 8, and 10) with maximum of 8 genes on Chr. 4 (Figure 4A). Another hemicellulose specific family, GT47 members were found to be distributed on all the chromosomes except on Chr. 5. About one-third (12) of GT47 family members were found present on Chr. 1 (Figure 4A).

##### Pectin biosynthetic genes

A total of 33 genes were identified as members of homogalacturonan α-1,4-galacturonosyltransferases (GAUT), a GT8 family involved in pectin biosynthesis. Presence of glycosyl_transferase_8 domain (PF01501) in pfam analysis confirms the annotation of these genes as GT8 members (Figure 1B). Sorghum GAUT members were further clustered into 5 sub-families (A–E) based on phylogenetic analysis with *Arabidopsis* homologs (Figure 3D). Among sub-families, D was the largest one with 19 members followed by C (6), A (5), B (2), and smallest sub-family E with a single member (Figure 3D). GT8 members were found to be distributed on all the chromosomes with 8 members exclusively present on Chr. 1 (Figure 4A).

##### Lignin biosynthetic genes

Sorghum lignin biosynthetic genes were analyzed along with other species (Xu et al., 2009) except laccase family. Here, we analyzed the laccase gene family that is involved in lignin biosynthesis. Laccases are among the CAZy AA1 class of enzyme which play important role particularly in lignin metabolism. A total of 25 sorghum genes were identified as laccase family genes based on the presence of three copper containing conserved domains namely Cu_oxidase (PF00394), Cu_oxidase2 (PF07731), and Cu_oxidase_3 (PF07732) (Table 1 and Figure 1B). Phylogenetic analysis of laccase proteins showed alignment of some of them with *Arabidopsis* proteins whereas some of sorghum laccase proteins clustered in distinct clusters (Figure 3E). Physical mapping of laccase genes showed their presence on all the chromosomes except Chr. 2, 6, and 7 with a maximum of 9 genes on Chr. 3 (Figure 4A).

##### Other cell wall biosynthetic genes

Glucan synthase-like (Gsl) gene family, a GT48 enzyme involved in biosynthesis of specialized polysaccharide callose, were found to have 12 members in sorghum genome based on the glucan_synthase (PF02364) conserved domain (Figure 1B). Phylogenetic analysis of SbGSL proteins showed their uniform distribution with *Arabidopsis* and rice homologs (Figure 3C). Physical mapping of these genes showed their distribution limited to Chr. 1, 3, 4, and 10 with maximum 4 genes on Chr. 3 (Figure 4A).

#### II. gene families involved in cell wall reassembly and degradation

Apart from cell wall biosynthetic enzymes, gene families involved in dynamic and complex cell wall extension and reassembly processes such as controlled degradation, loosening, and reassembly of cell wall polymers were also analyzed in the present study. A total of 335 genes were identified from 12 gene families that are involved in cell wall modifications.

##### Cell wall loosening gene families

A total of 83 and 24 genes were identified as members of expansins and yieldins gene families which are primarily involved in cell wall loosening (Table 1). The identification was based on the presence of conserved domains DPBB_1 (PF03330) and pollen_allerg_1 (PF01357) observed in the pfam domain analysis (Figure 1B). Phylogenetic analysis of these proteins classified them into two major clusters, Expansin-A with 40 proteins and Expansin-B with 43 proteins (Supplementary Figure 1A). Further physical mapping and distribution analysis of these genes showed their presence on all the chromosomes except Chr. 5 and 8 (Figure 4B). Chromosome 1 was observed to have a maximum number of 34 expansins genes. Pfam domain profiling of another cell wall loosening protein family yieldins (GT18) showed presence of glyco_hydro_18 (PF00704) conserved domain (Figure 1B). Phylogenetic analysis of sorghum yieldins showed more similarity with rice yieldins proteins than *Arabidopsis* proteins indicating conservation of yieldins among monocots (Supplementary Figure 1B). Further, yieldins were found distributed on six sorghum chromosomes namely Chr. 1, 2, 3, 5, 6, and 7 with maximum of 8 genes on Chr. 5 (Figure 4B). Xyloglucan endotransglucosylases/hydrolases (XTH), another important cell wall loosening proteins, have dual role of hydrolyzing and extension of existing cell wall. A total of 35 sorghum genes were identified as XTH family members based on the observed conserved domains glyco_hydro_16 (PF00722) and XET_C-term (PF06955) (Table 1 and Figure 1B). Phylogenetic analysis of XTH proteins classified them in 3 sub-families, sub-family A with 6 genes, B with 19 genes, and C with 10 genes (Supplementary Figure 1C). Physical mapping of these genes showed their distribution over six chromosomes (Chr. 1, 2, 4, 6, 7, and 10) with maximum of 7 genes were found to be present on Chr. 10 (Figure 4B).

##### Glycoside hydrolases

Among the identified cell wall modifying genes families in sorghum, there are 7 GH gene families (GH9, GH10, GH16, GH17, GH18, GH28, and GH35) with 201 genes (Table 1). Out of the identified 7 families, GH16 and GH18 have also been classified as cell wall loosening proteins. Among the GH families, GH17 (Glucan 1, 3-β-glucosidases) was the largest with 54 genes followed by GH28 (polygalacturonases) with 38 genes, GH9 (endo-1, 4-β-glucanases) with 26 genes, GH35 (β-galactosidases) with 13 genes, and GH10 (endo-xylanases) with 11 genes (Table 1). Further, Pfam domain analysis showed the presence of conserved Glyco_hydro_9 (PF00759) in GH9, Glyco_hydro_10 (PF00331) and CBM_4_9 in GH10, Glyco_hydro_17 (PF00332) and X8 domain (PF07983) in GH17, Glyco_hydro_28 (PF00295) and pectate_lyase_3 (PF12708) in GH28 and Glyco_hydro_35 (PF01301) domains in GH35 (Figure 1B). Phylogenetic analysis of these gene families showed further classification of GH17 and GH28 into 5 and 7 sub-families, respectively whereas GH9, GH10, and GH35 showed clustering with *Arabidopsis* proteins without any sub-classification (Supplementary Figures 1D–G). GH17 has been further clustered into sub-families A with 13 genes, B with 13 genes, C with 16 genes, D with 9 genes, and sub-family E with 3 genes (Supplementary Figure 1F). GH28 has also been showed sub-clustering into 7 sub-families namely, A with 12 genes, C with 6 genes, D with 7 genes, E with 4 genes, F with 5 genes, and G with 2 genes (Supplementary Figure 1G) however, no sorghum proteins were found in sub-family B. Physical distribution of these genes on the sorghum genome showed the presence of GH9 genes on all chromosomes except Chr. 5 and 8, GH10 genes on five chromosomes (Chr. 1, 2, 3, 4, and 6), GH17 genes on all the chromosomes, GH28 genes on all the chromosomes except Chr. 8 and GH35 genes on all the chromosomes except Chr. 5 and 6 (Figure 4B).

##### Pectin modifying enzymes

Among the identified cell wall related genes, 51 genes were classified as members of 4 gene families (2 pectin lyases and 2 pectin esterases) involved in pectin modification. Two pectin related lyases (PLs) namely pectate and pectin lyases (PL1) and rhamnogalacturonan I lyases (PL4) were found to have 10 and 6 genes respectively (Table 1). Conserved domain analysis of these proteins revealed the presence of Pec_lyase_C (PF00544) and Rhamno_gal_lyase (PF06045) domains in the PL1 and PL4, respectively (Figure 1B). An additional Pec_lyase_N (PF04431) domain was found in PL1 family while CBM_like (PF14683) and Fn3_3 (PF14686) domains were seen in PL4 family members. Phylogenetic analysis of PL1 family members along with *Arabidopsis* and rice homologs showed further clustering into three sub-families. PL1 sub family B was the largest one with 8 genes whereas sub family A and C was found to have one gene each (Supplementary Figure 1I). Phylogenetic analysis of PL4 genes showed more similarity to rice PL4 genes rather than *Arabidopsis* (Supplementary Figure 1J). Chromosomal distribution of these genes showed the presence of PL1 genes over six chromosomes (Chr. 1, 3, 4, 6, 8, and 10) whereas PL4 genes localization was limited to 4 chromosomes (Chr. 5, 7, 8, and 9) (Figure 4B).

Pectin esterases are another class of enzymes, which are involved in the cell wall reassembly. A total of 23 genes were identified as PME (CE8) homologs based on conserved pectin esterase (PF01095) domain whereas 12 genes were identified as PAE (CE13) family members based on pectin acetyl esterase domain (PF03283), respectively (Table 1 and Figure 1B). Phylogenetic analysis of PME protein from sorghum showed their even distribution with *Arabidopsis* PME proteins whereas sorghum PAE proteins showed more similarity to rice PAEs compared to *Arabidopsis* PAE's as expected (Supplementary Figures 1K,L). PME genes were distributed on all the 10 sorghum chromosomes with a maximum of 5 genes on Chr. 3 whereas PAE family members were limited to 6 chromosomes (Chr. 1, 2, 3, 4, 6, and 9) with a maximum of 7 genes on Chr. 3 (Figure 4B).

### Chromosomal localization and duplication analysis

Chromosomal localization of identified cell wall related genes were performed on the 10 sorghum chromosomes using Mapchart 2.30 mapping software (Figures 4A,B). Approximately 65% (336) of cell wall related genes were present on 4 chromosomes namely, chromosome 1 with 22.3% (116), chromosome 3 with ~16% (83), chromosome 2 with 13.2% (69), and chromosome 4 with 13.1% (68). Remaining genes were found to be distributed on remaining six chromosomes with a minimum of 15 genes on chromosome 8 (Figure 4). Further, all the cell wall related families were analyzed for tandem duplication within the respective gene families to study their expansion. Out of 20 gene families analyzed, 56 tandem duplication events involving 169 genes were observed in 17 families (Figures 4A,B). No tandemly duplicated genes were observed in CesA, glucan synthase, and β-galactosidase gene families. Expansins gene family was observed to have highest number of tandem duplication events (14) involving 51 genes. Among other gene families, MUR3 (5 events/15 genes), XTH (5 events/13 genes), MUR2 (4 events/15 genes), GH28 (4 events/9 genes), yieldins (4 events/14 genes), laccases (4 events/10 genes), GH17 (3 events/10 genes), PAE (2 events/5 genes), and XXT with 3 events involving 6 genes were observed with significant number of duplications. Apart from this, Csl gene family was also found to have 2 duplication events involving 9 genes. Chromosome 1 was found to have a maximum number (14) of tandem duplications of the cell wall gene families, followed by chromosome 3 (9 duplications), 4 (8 duplications), and 2 with 6 duplications. Only one tandem duplication event among cell wall genes was observed on chromosome 8.

### Cell wall related genes with SSR markers in sorghum

**Figure 5:**
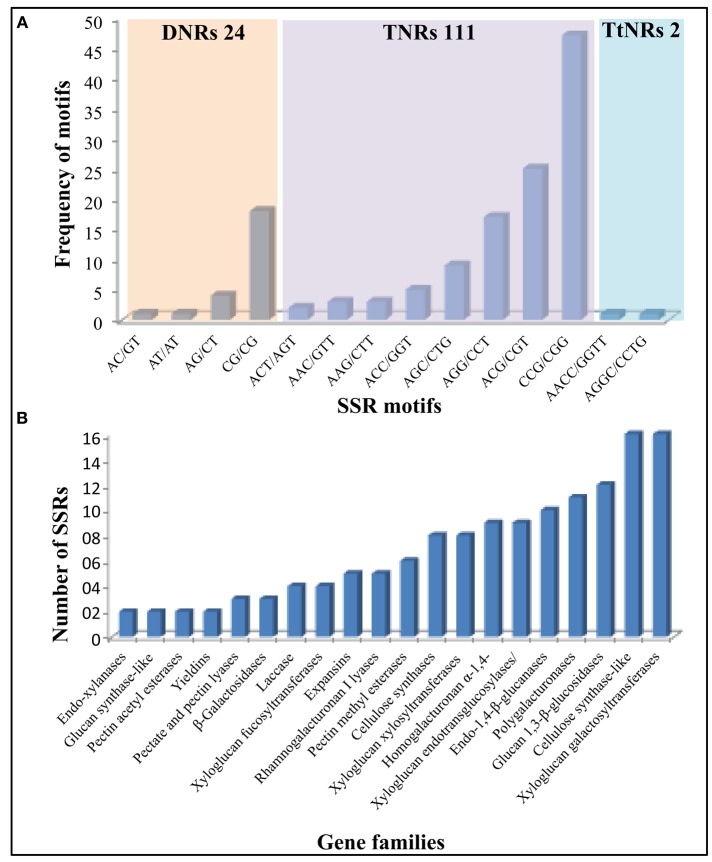
**Details of identified SSR markers (137) in 520 cell wall related genes of sorghum. (A)** Marker distribution based on number and type of repeat motifs. **(B)** Gene family wise distribution of identified SSRs markers.

Microsatellites or SSR markers are short tandem DNA repeats which belongs to comparatively most efficient class of molecular markers with its genome wide distribution and high level of polymorphism. Expressed or coding sequence derived SSRs (ESSRs) have been reported to be comparatively more conserved than the genomic derived SSRs (Guo et al., 2006) which makes ESSRs as an important tool for marker assisted selection for various plant breeding programs. Considering the importance of cell wall related genes in developing sorghum mutants for biofuel applications, we analyzed all of the identified cell wall related genes for the presence of SSR markers. Out of 520 genes, 112 genes were identified with 137 SSRs (125 Simple and 6 compounds; Figure 5A, Supplementary Tables 2, 3). Among the identified SSRs, tri-nucleotide repeats (TNRs) were most abundant with 111 occurrences followed by 24 DNRs. The identified SSRs were found to be present in all the 20 families analyzed with highest representation in Csl (16) and xyloglucan galactosyltransferases (16) gene families (Figure 5B).

### Cell wall related genes with putative miRNA target sites in sorghum

MicroRNAs (miRNAs) are small and conserved non-coding RNA molecules which are known to regulate the gene expression at transcriptional and post-transcriptional levels. To understand the potential roles of miRNAs in regulating the cell wall related gene expression, all the 520 cell wall genes from various families were analyzed for the presence of miRNA target sites. A total of 10 genes were identified to have miRNA target sites out of which 6 belong to laccase (Sobic.001G422300.1, Sobic.003G352700.1, Sobic.003G352800.1, Sobic.003G353200.1, Sobic.005G198500.1, and Sobic.009G162800.1), 2 belong to Gsl (GT48) (Sobic.003G298900.1 and Sobic.004G107800.1) and one each to CesA (Sobic.003G049600.2) and Csl (Sobic.008G125700.1) (Supplementary Table 4). Six different miRNA families (miR156, miR164, miR397, miR528, miR5566, and miR6230) were identified to target these cell wall related genes.

### Expression profile of cell wall related genes in different organs of sorghum plant

**Figure 6:**
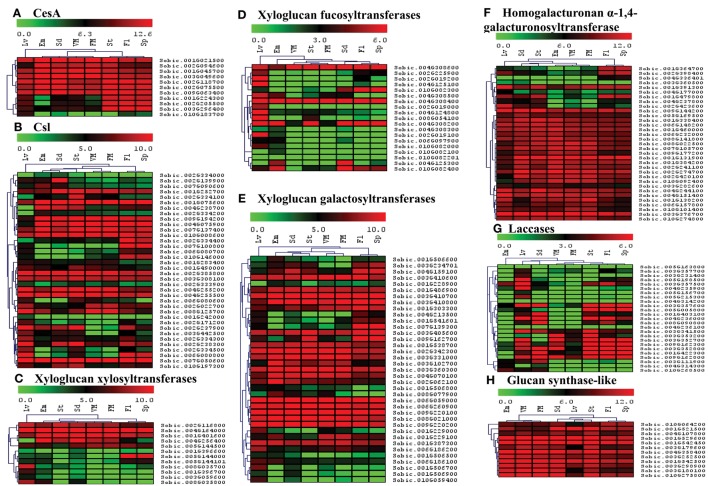
**Heat map showing hierarchical clustering of the sorghum's cell wall related biosynthetic gene families in various developmental stages. (A)** CesA, **(B)** Csl, **(C)** Xyloglucan xylosyltransferases, **(D)** Xyloglucan fucosyltransferases, **(E)** Xyloglucan galactosyltransferases, **(F)** Homogalacturonan α-1,4-galacturonosyltransferase, **(G)** Laccases, **(H)** Glucan synthase-like. RNA-seq data from various developmental stages viz. stem (St), 20 days old leaves (Lv), vegetative meristem (VM), floral meristem (FM), spikelet (Sp), flowers (FL), embryos (Em), and seeds (Sd) were mapped on gene sequences related to above gene families. The respective RPKM values were used to construct heatmap with scale bar on the top showing expression of the genes. Red colors represent high expression whereas green represents low expression.

**Figure 7:**
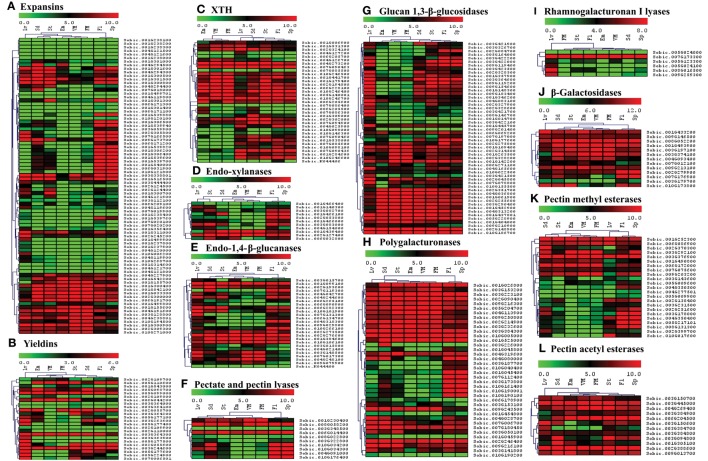
**Heat map showing hierarchical clustering of the sorghum's cell wall related gene families involved in reassembly and degradation in various developmental stages. (A)** Expansins, **(B)** Yieldins, **(C)** XTH, **(D)** Endo-xylanases, **(E)** Endo-1, 4-β-glucanases, **(F)** Pectate and pectin lyases, **(G)** Glucan 1, 3-β-glucosidases, **(H)** Polygalacturonases, **(I)** Rhamnogalacturonan I lyases, **(J)** β-Galactosidases, **(K)** Pectin methyl esterases, **(L)** Pectin acetyl esterases. RNA-seq data from various developmental stages viz. stem (St), 20 days old leaves (Lv), vegetative meristem (VM), floral meristem (FM), spikelet (Sp), flowers (FL), embryos (Em), and seeds (Sd) were mapped on gene sequences related to above gene families. The respective RPKM values were used to construct heatmap with scale bar on the top showing expression of the genes. Red colors represent high expression whereas green represents low expression.

The availability of whole transcriptome data online presented an excellent opportunity to identify candidate genes that play key roles in specific organs during sorghum development. The information on candidate genes can be further used to engineer cell walls in a cell/tissue specific manner to meet various industrial needs particularly in biofuel/feed industry. Publically available whole transcriptome datasets (Supplementary Table 5) were used to analyze spatial expression of the cell wall related genes in 8 different organs (leaves, embryo, seed, stem, spike, flower, vegetative as well as floral meristem) using RPKM values. The relative expression data was represented family wise using individual heat maps in order to better analyze the role of genes from each family (Figures 6, 7, Supplementary Table 6). In case of CesA genes, 7 out of 11 genes were observed to have high expression in all analyzed organs, whereas, 3 genes were highly expressed in stem, flowers and spikes with moderate expression in leaves, seeds, and vegetative meristem (VM; Figure 6A). One CesA gene (Sobic.010G183700) found to be expressed exclusively in leaves. A mixed pattern of expression was observed in Csl gene family with 11 genes showing high expression and 3 genes with very low expression in all the 8 tissues analyzed (Figure 6B). Among hemicellulose biosynthetic genes, 12 xyloglucan xylosyltransferases genes were clustered in two main clusters, first with 5 genes having medium to high expression in all the 8 tissues, whereas second cluster of 7 genes with tissue specific expression mainly in leaves (Figure 6C). Xyloglucan fucosyltransferases gene family members, other than 3 genes (Sobic.004G308200, Sobic.004G308400, Sobic.004G308600) that showed higher expression in all the 8 tissues, expression of remaining genes was mostly limited to leaves (Figure 6D). Half (19) of the xyloglucan galactosyltransferases gene family, showed a higher expression level in all the tissues, whereas the other half (18) showed moderate to low expression in various tissues (Figure 6E). In homogalacturonan α-1,4-galacturonosyltransferase gene family, 25 out of 33 genes showed high expression in all the tissues whereas remaining 8 genes showed tissue specific expression (Figure 6F). Lignin biosynthetic related laccase genes showed high expression in selective tissues like leaves, seeds, stem flower, and spikes (Figure 6G). Other than few laccase genes, most of the laccases were not expressed in embryo and meristematic tissues. All the 12 genes of glucan synthase gene family showed high expression across all the tissue analyzed (Figure 6H).

Apart from polysaccharide biosynthetic gene families, the spatial expression of 12 gene families involved in degradation and reassembly was also analyzed. Among expansin genes, 16 genes from 2 clusters were observed with medium to high expression in all the tissues (Figure 7A). Remaining expansin genes mostly showed expression in flower, spike, leaves, seeds, and/or stem. Yieldins were observed to express consistently in leaves, whereas some of them showed high expression in all the other tissues (Figure 7B). Most of the XTH gene family showed moderate to high expression in almost all the tissues analyzed other than a cluster with no expression in embryo and meristematic tissues (Figure 7C). The five GH family genes (endo-xylanases, endo-1, 4-β-glucanases, glucan 1, 3-β-glucosidases, polygalacturonases and β-galactosidases) were highly expressed in leaves, stem, seed, flowers, and spikes apart from the clusters with high expression in all tissues (Figures 7D,E,G,H,J, respectively). Other than β-galactosidases, a significant proportion of genes from these families were not expressed in the embryo and meristematic tissues. Among the 10 genes encoding pectate lyases, 3 showed expression in all the tissues whereas expression of remaining genes were limited to flower, spike, and seeds (Figure 7F). In the other pectin related rhamnogalacturonan I lyases gene family, only one gene showed consistent expression in all the tissues whereas remaining genes showed leaf specific expression (Figure 7I). Among the two families of esterases, gene encoding PMEs were majorly clustered into two clusters based on expression, first with moderate to high expression in almost all the tissues analyzed whereas second cluster with expression limited to tissues other than embryo and meristem (Figure 7K). All the PAE family genes showed medium to high expression in almost all the tissues analyzed (Figure 7L).

### Differential expression analysis of cell wall related genes under different abiotic stress conditions

**Figure 8:**
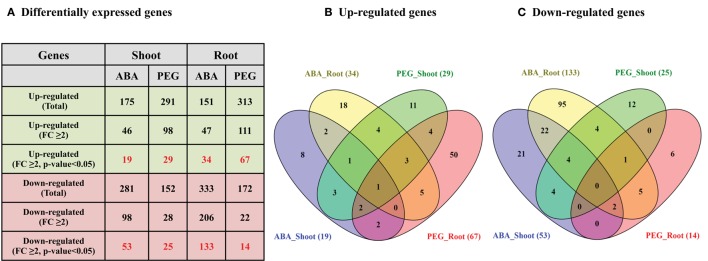
**Differential expression analysis (DEG) of sorghum cell wall related genes in ABA and PEG treated shoot and root. (A)** Details of differentially expressed genes (Fold change ≥ 2.0 and *p* < 0.05) during stress. **(B)** Venn diagram representing up-regulated genes during stress. **(C)** Venn diagram representing down-regulated genes during stress.

Differential expression analysis of cell wall related genes under two abiotic stress treatments (ABA and osmotic stress) was performed to analyze their response in seedling root and shoots. A total of 19 and 29 genes were found to be significantly up-regulated (FC ≥ 2.0 and *p* < 0.05) in the sorghum shoots whereas 34 and 67 genes were found significantly up-regulated in the roots subjected to ABA and PEG treatment, respectively (Figures 8A,B, Supplementary Table 7, Supplementary Figures 2A,C, 3A,C). Similarly, 53 and 25 genes were significantly down-regulated in shoot whereas 133 and 14 genes were found down-regulated in root treated with ABA and PEG, respectively (Figures 8A,C, Supplementary Table 7, Supplementary Figures 2B,D, 3B,D). Relatively higher number of genes was down-regulated in the ABA treated shoot and root than PEG treatment. Comparative analysis of differentially expressed genes in root and shoot subjected to ABA and PEG treatment showed common up-regulation of 1 gene (Figure 8B) whereas no gene was found to be down-regulated in common (Figure 8C).

The ABA treated shoots showed up-regulation of polygalacturonases (4) whereas down-regulation of expansins (12), laccases (9), glucan 1,3-β-glucosidases (8), and polygalacturonases (5) (Supplementary Figures 2A,B). The ABA treated roots showed up-regulation of glucan 1,3-β-glucosidases (5), homogalacturonan α-1,4-galacturonosyltransferases (4), laccases (3), and xyloglucan galactosyltransferases (3) whereas down-regulation of expansins (38), glucan 1,3-β-glucosidases (18), laccases (11), Csls (11), and XTHs (9) (Supplementary Figures 3A,B). Large number of expansins showed down-regulation in ABA treated shoots as well as roots. Glucan 1,3-β-glucosidases was the second most down-regulated family in ABA treated shoot as well as root. Similarly, most of the up-regulated genes in PEG treated roots mainly belong to expansins (21), XTHs (10), and glucan 1,3-β-glucosidases (6) families whereas in PEG treated roots the down-regulated genes mainly belong to yieldins (3) and Csls (3) (Supplementary Figures 3C,D). The major up-regulated cell wall related gene families in PEG-treated shoots were expansins (6), glucan 1, 3-β-glucosidases (4), and XTHs (4). Similar to PEG-treated roots, yieldins were the most down-regulated gene family in PEG treated shoots (Supplementary Figures 2C,D).

## Discussion

Cell wall biogenesis is a dynamic process that involves synergistic action of multiple gene families that are involved in the biosynthesis as well as controlled degradation and reassembly of cell wall polymers. Broadly, glycosyl transferases are the major class of enzymes involved in cell wall polysaccharide biosynthesis while substrate specific glycoside hydrolases, pectin related lyases, various esterases together with cell wall loosening enzymes are responsible for cell wall extension. Among these families, cellulose synthase and cellulose synthase like genes are among the most studied in model, tree, and crop plants (Richmond and Somerville, 2000; Appenzeller et al., 2004; Djerbi et al., 2005; Muthamilarasan et al., 2015). Successful production of renewable biofuels and bioproducts from lignocellulose requires a comprehensive understanding on the genes involved in the biosynthesis of plant lignocellulosic material. A comprehensive report on cell wall related genes was missing in sorghum, which is an important food, fiber, bioproduct, and biofuel crop. Understanding the presence and distribution of cell wall related genes in sorghum would augment the plant breeding and biotechnological approaches to develop sorghum plants with altered cell wall composition for various industrial applications apart from crop improvement. In the present study, 520 genes from 20 cell wall related gene families have been identified and characterized *in silico*. Gene expression analysis of the identified genes was performed in different organs under normal and abiotic stress treated conditions to understand their role in cell wall development and abiotic stress of sorghum. These candidate genes can be putative targets of reverse genetics for crop improvement apart from value addition to sorghum.

Lignocellulosic material deconstruction is important for bioethanol production from plant biomass. Current technology of bioethanol production involves separation of lignin from the lignocellulosic material, saccharification of sugars from wall polysaccharides and fermentation. Lignocellulosic based bioethanol production is technically and economically not competitive compared to fossil based gasoline with the existing conversion technologies. Further, the cost of bioethanol from plant biomass is higher ($1.5/gal) than starch based ($0.9/gal) bioethanol (http://www.nrel.gov/docs/fy01osti/28893.pdf). Improving the efficiency of the lignocellulosic biomass deconstruction, particularly separation of lignin from other wall polymers is essential to make bioethanol production economically feasible. Altering the biomass composition is essential to reduce biomass recalcitrance and improve the conversion technologies. Understanding the composition of lignocellulosic biomass and the genes involved in the lignocellulosic biomass facilitates the biomass engineering to improve the conversion efficiency.

Lignocellulosic material is mainly composed of cellulose, hemicellulose, lignin, and pectin. Cellulose is a linear homopolymer of β-1–4 linked glucose molecules occupying 30–40% of cell wall weight. CesA genes were first identified in bacteria and later in cotton followed by other plants (Saxena et al., 1990; Pear et al., 1996). The cellulose is synthesized by plasma membrane localized cellulose synthase complexes (CSCs) composed of multiple CESA proteins that produce individual glucan chains (Persson et al., 2007; Kumar and Turner, 2015). The individual glucan chains form a cellulose microfibrils and several microfibrils form a cellulose fiber hence the number of cellulose synthases present in individual species is important to understand the cellulose biosynthetic process. Moreover, cellulose synthases serve specific roles in plant development as they have cell, tissue, and developmental specific roles (Taylor et al., 2000; Mendu et al., 2011a). Hence there are multiple CesAs in each species and the number of genes varies based on the plant species; *Arabidopsis* (10), maize (12), poplar (18), and foxtail millet (14) (Richmond and Somerville, 2000; Appenzeller et al., 2004; Djerbi et al., 2005; Muthamilarasan et al., 2015). In the present study we identified 11 CesA genes in contrast to the reports of 12 CesA genes in sorghum, which is due to the removal of errors in the updated sorghum genome assemblies (Table 1, Figure 1). Though the number of CesAs present in sorghum is known, it is important to study the role of individual CesAs in primary and secondary cell wall biosynthesis to modify the biomass composition in specific organ or tissue.

Hemicellulose composition and biosynthesis is complex as they are composed of branched polysaccharides compared to homopolymeric cellulose. Hemicelluloses play an important role in cell wall polymer cross-linking and help in maintaining the cell wall integrity and strength. The hemicellulose content and composition is different in monocot and dicot plants. The cell walls of monocots such as sorghum contain 20–50% of hemicelluloses that makes it an attractive source for pentose sugars (Welker et al., 2015). Bioethanol production from pentose sugars apart from hexoses is currently being heavily investigated (Unrean and Srienc, 2010). Understanding the hemicellulose biosynthesis and genes involved in the biosynthetic process will help to alter the biomass composition for easy deconstruction as well as to improve hexose to pentose ratio. The hemicellulose biosynthesis genes have not been studied very well other than cellulose synthase like genes. In the present investigation, the large family of sorghum Csls has been classified into 8 different groups (CslA to H) based on the phylogenetic studies (Ermawar et al., 2015a; Figure 2). Particularly, two clusters, CslF and CslH were observed be unique to sorghum with no *Arabidopsis* homologs, which is in agreement with the previous reports of these clusters as grass specific (Paterson et al., 2009; Ermawar et al., 2015a) while no sorghum Csl genes were clustered in Clusters CslB and CslG (Figure 2). Similar clustering of SbCsl genes has been reported previously in the sorghum draft genome report (Paterson et al., 2009). Apart from Csls, we also identified additional three hemicellulose gene families including xyloglucan xylosyltransferases (GT34), xyloglucan fucosyltransferases (GT37), and xyloglucan galactosyltransferases (GT47). Homogalacturonan α-1,4-galacturonosyltransferases (GT8), a pectin biosynthesis related gene family was also observed as one of the sorghum's big cell wall biosynthetic gene families (Table 1, Figure 1). Pectin molecules play an important role in cell adhesion and contributes for biomass recalcitrance due to extensive interlinks with other cell wall polymers. Overall, information on cell wall biosynthetic genes will help to design customized biomass production for economical production of biofuels and bioproducts from sorghum. Apart from easier deconstruction and saccharification, enhancing the total sugars in the walls will help to improve the cost effectiveness of bioethanol production from sorghum biomass.

Cell wall biosynthesis is dynamic; it allows cell elongation while maintaining the wall integrity to withstand the internal turgor pressure. The degradation/assembly mechanism plays important roles in the cell wall building process, wall strength and integrity. Altering the process of wall degradation/assembly process will influence the cell wall deconstruction/digestibility hence identification and characterization of genes involved in cell wall degradation/assembly is important. The degradation/assembly related genes identified in this study has been distributed in to 3 cell wall loosening related gene families, 6 family of glycoside hydrolases, 2 pectin related lyases as well as 2 pectin related esterase gene families. The conserved domain analysis of the cell wall related gene families (Figure 1) along with clusters obtained from phylogenetic analysis with *Arabidopsis* and rice proteins suggests the evolutionary conserved nature of these proteins (Supplementary Figure 1). Physical mapping revealed presence of approximately 65% of cell wall related genes mainly confined to chromosomes 1–4 (1 with 22.3%, chromosome 3 with ~16%, chromosome 2 with 13.2%, and chromosome 4 with 13.1%; Figure 4, Supplementary Table 1). These chromosomes with hotspot of cell wall genes can be targeted in breeding and crop improvement programs to alter the cell wall composition. Further, 56 tandem duplication events observed in these genes were found to be distributed across all the 10 chromosomes with maximum duplication observed on first 4 chromosomes. The excessive duplications observed on the chromosomes 1–4 could be the possible reason of presence of ~65% genes on these chromosomes. Further, a total of 137 SSR markers were found on ~22% cell wall related genes with highest representation in Csl (16) and xyloglucan galactosyltransferases (16) gene families (Figure 5, Supplementary Tables 2, 3). Among these, TNRs with ~81% share are the most abundant SSRs which are in agreement with the previous reports of TNRs abundance in plants. These molecular markers will help the breeding programs for selection of genes in a breeding population or introgression of a specific cell wall related genes. Further, *in silico* analysis for the presence of miRNA targets revealed presence of miR156, miR164, miR397, miR528, miR5566, and miR6230 target sites in 10 independent cell wall related genes (Supplementary Table 4). Three of these miRNA families viz. miR156, miR164, and miR528 have been reported to be differentially expressed in stem and leaves during sugar accumulation in sweet sorghum (Yu et al., 2015). Further, Yu et al. (2015) reported miR164 and miR528 as stem specific miRNA whereas miR156 was up-regulated in the leaves at dough stage. Over-expressed miR156 has been reported to cause the *Corngrass1* (*Cg1*) phenotype in maize (Chuck et al., 2007). Further, four of the six sorghum laccase family genes found to have target site of miR397 and showed differential expression during the drought stress conditions (Hamza et al., 2016) indicating a potential change in the sorghum cell wall composition under stress.

The cell wall composition and gene expression varies among different tissues of the plant (stem, root, leaves, etc.) and among the cell types within a tissue (i.e., epidermal, xylem, phloem, fiber cells, etc.; Hatfield et al., 1999; McKinley et al., 2016). Expression analysis across different tissues will provide important insight into the role of cell wall related genes in that particular tissue. In the present study, we found a differential expression of genes among different tissues (Figures 6, 7). This analysis provides information on the tissue specific target genes for bioengineering purposes. A recent study of sorghum gene expression in pre- and post-anthesis stages of stem internodes showed differential expression of genes involved in growth, cell wall development and stem sugar accumulation (McKinley et al., 2016). CesA, Csls, callose synthases, XTHs, glucuroarabinoxylan biosynthetic genes, expansins, glucosyl hydrolases, pectin lyases/esterases, and lignin biosynthetic genes were among the major differentially expressed cell wall related genes (McKinley et al., 2016). In the present study, expression analysis of sorghum cell wall related genes showed that most of the genes from CesA, xyloglucan galactosyltransferases, homogalacturonan α-1,4-galacturonosyltransferase, glucan synthase-like, glucan 1,3-β-glucosidases, polygalacturonases, β-Galactosidases, and pectin acetyl esterases families expressed in all the stages studied. Remaining cell wall gene families showed genes with either stage specific to ubiquitously expressed genes or both.

Environmental conditions including temperature, drought, osmotic, and salinity, etc., have been shown to affect the gene expression and crop productivity (Tenhaken, 2014; Wang et al., 2016). With the fast changing environmental conditions across the globe, studying the effect of stress on plants is important. In addition, to avoid food/fuel competition, the biofuel crops were advocated to be grown on marginal lands with limited irrigation and minimal input. Upon exposure to these adverse environmental conditions, the plants alter their gene expression and biochemical metabolism to survive in these conditions including cell wall composition. Most important component of the cell wall that adds enormous cost of bioethanol production is lignin. It has been reported that abiotic stress results in increased lignin content in plants (Moura et al., 2010). This results in increased cost of bioethanol production hence there is a need to develop bioenergy crops that do not accumulate higher lignin when grown in marginal lands with limited irrigation and low inputs. A better understanding of the cell wall gene expression under abiotic stress is important to design strategies to produce crops in marginal lands with less lignin accumulation. Analysis of sorghum transcriptome under abiotic stress showed differential expression of significant number of cell wall related genes (Figure 8). Comparatively, root was observed to have more altered expression of cell wall genes compared to shoot. Among the differentially expressed gene families, expansins, laccases, and glucan 1, 3-β-glucosidases showed down-regulation in ABA treated root and shoot (Supplementary Figures 2B, 3B). Similarly, following PEG treatment, expansins, and XTHs were among up-regulated genes in root as well as shoot whereas yieldins were among the highly down-regulated genes in both the tissues (Supplementary Figures 2C,D, 3C,D). Since most of the cell wall related gene families are with multiple genes and each with either specific or redundant function, there is a need to characterize function of individual genes in order to develop a fine annotation of their function in normal growth and development as well as under abiotic stress conditions.

## Conclusions

Comprehensive information on cell wall related genes would facilitate biosynthetic pathway engineering for enhanced biomass production as well as efficient deconstruction and saccharification. Lignin content and cellulose crystallinity contribute to the poor separation and saccharification, which are the biggest hurdles in the cost efficient utilization of sorghum biomass for biofuel production. Here we have identified various cell wall related gene families and analyzed the gene expression pattern but the functional role of the individual genes is still not known. Cell wall related gene mutations in sorghum showed higher saccharification efficiency and are being used for animal feed hence further analysis and functional characterization will lead to development of more efficient sorghum lines for animal feed, biofuel and bioproduct industries. Apart from this, analyzing the cell wall composition of sorghum under abiotic stress conditions and their correlation with differentially expressed genes will also shed light on the mechanism involved in regulation of cell wall biosynthesis and degradation. The present study analyzed the gene expression of sorghum seedlings exposed to abiotic stress, which provides valuable information, however a detailed study at different developmental stages that are critical for biomass harvest will provide information necessary to manipulate the biomass through plant breeding and genetic engineering. Overall, the comprehensive information developed in the present study can be used in expanding target genes as well as developing better strategies for the future sorghum crop improvement programs.

## Author contributions

KR designed the work, performed the analysis and wrote the manuscript. ST, VB, CC, and TD helped with bioinformatics analysis, prepared figures and wrote the manuscript. VM conceived the idea, designed work and wrote the manuscript. All the authors have read and approved the manuscript.

### Conflict of interest statement

The authors declare that the research was conducted in the absence of any commercial or financial relationships that could be construed as a potential conflict of interest.
