# Supplementary material for: Identification, Characterization, and Expression Analysis of Cell Wall Related Genes in Sorghum bicolor (L.) Moench, a Food, Fodder, and Biofuel Crop
Source: Front Plant Sci. 2016 Aug 31;7:1287. doi: 10.3389/fpls.2016.01287 (PMC5006623; doi:10.3389/fpls.2016.01287)
Supplement: Supplementary file 1 [file Table1.PDF]

Supplementary Table 1. Family wise distribution list of cell wall related genes from sorghum.

Gene family	Gene Name	Length		Chromosomal location			M/w (in KDa)	pI
		CDS (in bp)	Protein (in aa)	Chr	Start	End		
Cellulose synthases	Sobic.001G021500	3273	1090	Chr01	1763688	1770770	6.64	123.2
	Sobic.001G045700	3243	1080	Chr01	3378992	3385186	7.87	121
	Sobic.001G224300	3177	1058	Chr01	21442414	21446474	7.16	119.6
	Sobic.002G075500	3246	1081	Chr02	7823291	7829588	8.02	121
	Sobic.002G094600	3303	1100	Chr02	9822552	9829338	7.8	122.9
	Sobic.002G118700	3264	1087	Chr02	14797053	14803136	6.52	122.7
	Sobic.002G205500	3150	1049	Chr02	59676825	59681745	6.4	118
	Sobic.003G049600	3225	1074	Chr03	4541759	4550416	6.36	120.9
	Sobic.003G296400	2943	980	Chr03	62874720	62880788	6.22	110.2
	Sobic.009G063400	3222	1073	Chr09	6779024	6785550	6.5	120.8
	Sobic.010G183700	2649	882	Chr10	52077987	52081663	5.94	96.3
Cellulose synthase-like	Sobic.001G075600	2079	692	Chr01	5758870	5763265	8.64	77.7
	Sobic.001G242000	2562	853	Chr01	24977793	24983291	8.72	93.3
	Sobic.001G252700	1857	618	Chr01	27402757	27408552	8.79	69.9
	Sobic.001G283400	3495	1164	Chr01	48424827	48429222	7.49	129.4
	Sobic.001G490000	1644	547	Chr01	68828568	68832876	6.8	61.7
	Sobic.002G022700	2049	682	Chr02	2107016	2109765	9.04	76.8
	Sobic.002G139900	1653	550	Chr02	22430139	22433425	9.35	62
	Sobic.002G171200	2517	838	Chr02	54251558	54256532	7.63	92.6
	Sobic.002G237900	2187	728	Chr02	62786326	62791172	7.53	82.5
	Sobic.002G238300	2268	755	Chr02	62831028	62839678	6.45	83.5
	Sobic.002G333900	2715	904	Chr02	70204031	70207191	7.66	100.4
	Sobic.002G334000	2595	864	Chr02	70208872	70212417	8.11	98
	Sobic.002G334100	2568	855	Chr02	70213957	70217497	8.28	96.1
	Sobic.002G334200	2571	856	Chr02	70222821	70226299	7.34	96.2
	Sobic.002G334300	2640	879	Chr02	70231976	70242620	5.99	96.8
	Sobic.002G334400	2679	892	Chr02	70244172	70247883	6.25	101.2
	Sobic.002G334500	2751	916	Chr02	70259728	70264636	6.35	101.4
	Sobic.002G385800	1722	573	Chr02	74010875	74015985	8.81	64.2
	Sobic.003G308100	2070	689	Chr03	63692979	63697782	8.64	76.8

	Sobic.003G442500	2247	748	Chr03	74115980	74119532	6.58	81.7
	Sobic.004G075900	1566	521	Chr04	6201869	6209399	9.05	59.6
	Sobic.004G238700	1635	544	Chr04	57965231	57969093	8.77	62
	Sobic.004G255200	1878	625	Chr04	59463039	59467085	8.31	71.1
	Sobic.004G255500	1500	499	Chr04	59470411	59476189	8.87	56.2
	Sobic.006G080600	2151	716	Chr06	45804303	45810040	6.42	78.3
	Sobic.006G080700	2328	775	Chr06	45814277	45817780	6.89	84.9
	Sobic.006G080800	2328	775	Chr06	45849099	45853338	6.46	85
	Sobic.007G050600	2886	961	Chr07	5103889	5111325	8.43	106.7
	Sobic.007G090600	2283	760	Chr07	12938747	12943207	8.64	86.5
	Sobic.007G100800	3447	1148	Chr07	22640040	22644196	6.76	127.8
	Sobic.007G137400	1740	579	Chr07	55194661	55205047	8.27	65
	Sobic.008G125700	3678	1225	Chr08	47500226	47504750	7.51	133.4
	Sobic.009G194200	2094	697	Chr09	54569256	54573531	8.62	77.8
	Sobic.010G008600	3540	1179	Chr10	725791	731298	7.49	131
	Sobic.010G146000	3174	1057	Chr10	41254250	41258193	7.05	117.3
	Sobic.010G197300	1611	536	Chr10	53787386	53794028	9.01	60.3
**Xyloglucan xylosyltransferases and galactomannan gal-transferases**	Sobic.001G396600	1341	446	Chr01	61030908	61032663	6.39	50.5
	Sobic.001G396700	1341	446	Chr01	61038233	61039573	6.19	50.6
	Sobic.001G401600	1341	446	Chr01	61518010	61520883	8.34	51.5
	Sobic.002G116800	939	312	Chr02	14461326	14464216	9.85	35.3
	Sobic.003G059600	1362	453	Chr03	5258053	5259649	6.33	50.7
	Sobic.004G164000	1443	480	Chr04	50624115	50629119	6.79	53.3
	Sobic.004G256400	1332	443	Chr04	59541974	59543305	6.2	49
	Sobic.005G144000	1350	449	Chr05	51290857	51292677	9.23	49.8
	Sobic.005G144101	1386	461	Chr05	Nd	Nd	9.44	50.8
	Sobic.005G144500	1440	479	Chr05	51349543	51351602	8.98	52.1
	Sobic.008G035700	1548	515	Chr08	3286596	3288143	9.78	57.1
	Sobic.008G035800	1398	465	Chr08	3288447	3290341	6.29	52.1
	Sobic.002G019000	1719	572	Chr02	1749107	1751113	8.3	63.5
	Sobic.002G019100	1671	556	Chr02	1753583	1755310	8.67	62
	Sobic.002G019200	1569	522	Chr02	1766583	1768694	8.76	58.2
	Sobic.002G225900	1647	548	Chr02	61757191	61759285	6.65	60.8
	Sobic.004G124800	1758	585	Chr04	14239766	14243963	7.92	64.6

**Xyloglucan fucosyltransferases (MUR2)**	Sobic.004G125100	1848	615	Chr04	14358330	14370713	7.92	69.6
	Sobic.004G125300	1713	570	Chr04	14597479	14601779	8.44	63.7
	Sobic.004G308200	1782	593	Chr04	63895836	63900371	9.16	66.3
	Sobic.004G308300	1773	590	Chr04	63900716	63904279	8.36	65.6
	Sobic.004G308400	1821	606	Chr04	63909029	63918173	6.27	68.8
	Sobic.004G308500	1770	589	Chr04	63919885	63922635	7.94	66.7
	Sobic.004G308600	1743	580	Chr04	63924521	63927400	6.75	65.1
	Sobic.006G097900	1752	583	Chr06	47640339	47642659	9.15	64.1
	Sobic.008G054100	1833	610	Chr08	5427503	5429762	8.35	67.4
	Sobic.010G082000	1749	582	Chr10	6964128	6968149	7.58	65.3
	Sobic.010G082100	1662	553	Chr10	6970287	6972544	8.28	61.5
	Sobic.010G082201	1503	500	Chr10	Nd	Nd	9.75	54.8
	Sobic.010G082300	1680	559	Chr10	6991757	6993751	8.77	63.3
	Sobic.010G082400	1851	616	Chr10	6993752	6996092	8.79	69
**Xyloglucan galactosyltransferases (MUR3)**	Sobic.001G228900	1380	459	Chr01	21902831	21904226	8.83	51.1
	Sobic.001G229000	1842	613	Chr01	21907886	21910113	6.1	67.5
	Sobic.001G229100	1473	490	Chr01	21979683	21982535	9.23	54.4
	Sobic.001G303300	1353	450	Chr01	51494553	51496793	8.54	50.4
	Sobic.001G387300	1389	462	Chr01	60265808	60268895	7.66	52
	Sobic.001G486900	2352	783	Chr01	68533511	68538881	6.41	88.6
	Sobic.001G506500	1641	546	Chr01	70264008	70265552	9	62
	Sobic.001G506600	1563	520	Chr01	70266418	70268521	8.69	58.5
	Sobic.001G506700	1539	512	Chr01	70279754	70281390	9.66	57
	Sobic.001G506800	1590	529	Chr01	70287716	70289574	9.54	58.9
	Sobic.001G506900	1542	513	Chr01	70291451	70293177	8.79	57.1
	Sobic.001G538700	1290	429	Chr01	72955323	72958319	9.54	48.5
	Sobic.001G541601	621	206	Chr01	Nd	Nd	6.11	22.7
	Sobic.002G062100	1755	584	Chr02	6022542	6026663	8.89	63.6
	Sobic.002G342300	1371	456	Chr02	70860196	70866563	8.93	50.9
	Sobic.003G102700	1701	566	Chr03	9146679	9150329	9.09	63.9
	Sobic.003G234701	1515	504	Chr03	Nd	Nd	10.06	57.1
	Sobic.003G331000	1500	499	Chr03	65598222	65603459	9.01	56.2
	Sobic.003G360300	1281	426	Chr03	67807330	67811218	9.52	48.5
	Sobic.003G405600	1299	432	Chr03	71296761	71300966	9.22	48.4

	Sobic.003G410600	1248	415	Chr03	71815596	71819830	6.11	46.9
	Sobic.003G410700	1254	417	Chr03	71828059	71831388	6.42	46.9
	Sobic.003G410800	1263	420	Chr03	71834059	71838056	6.42	47.1
	Sobic.004G070100	1212	403	Chr04	5689474	5693600	9.15	45.9
	Sobic.004G159100	1299	432	Chr04	49827888	49831203	8.33	48.5
	Sobic.004G213500	1413	470	Chr04	55611345	55613881	9.51	52.7
	Sobic.006G059000	1293	430	Chr06	40774079	40776804	6.67	48.3
	Sobic.006G186100	1707	568	Chr06	55054652	55057308	8.77	62.7
	Sobic.006G186200	1440	479	Chr06	55063266	55065346	9.21	52.7
	Sobic.006G260900	1371	456	Chr06	60514121	60516998	9.1	51.9
	Sobic.007G139300	1572	523	Chr07	55456905	55459200	9.86	58.9
	Sobic.008G021000	1488	495	Chr08	1824133	1827730	8.92	54.8
	Sobic.008G077900	1572	523	Chr08	12038554	12040495	5.78	58.6
	Sobic.009G162700	1269	422	Chr09	51981567	51983839	6.35	47.2
	Sobic.009G220100	1248	415	Chr09	56360605	56363786	6.37	46.4
	Sobic.009G220200	1251	416	Chr09	56365659	56369212	6.47	47.1
	Sobic.010G059400	1530	509	Chr10	4640050	4642285	9.52	57.1
	Sobic.001G131900	1767	588	Chr01	10338127	10343010	9.02	67.9
	Sobic.001G138200	1062	353	Chr01	10936048	10937633	8.56	38.1
	Sobic.001G338400	1632	543	Chr01	55403813	55408926	8.06	62.6
	Sobic.001G364700	1203	400	Chr01	58163160	58165130	6.96	4.9
	Sobic.001G384200	1887	628	Chr01	59971637	59977394	7.95	72
	Sobic.001G391300	1050	349	Chr01	60573664	60575549	5.55	39.2
	Sobic.001G460000	1683	560	Chr01	66374155	66380727	8.91	63
	Sobic.001G479800	1818	605	Chr01	67962821	67967813	8.6	68.3
	Sobic.002G241100	2361	786	Chr02	63044462	63049000	9.82	87.8
	Sobic.002G274700	2052	683	Chr02	65726175	65731322	9.02	77.7
	Sobic.002G398400	1074	357	Chr02	74928680	74931154	9.06	40
	Sobic.002G420100	1884	627	Chr02	76682290	76687610	9.11	71.9
	Sobic.002G423600	1017	338	Chr02	77036969	77038682	5.8	38.3
	Sobic.003G282600	1614	537	Chr03	61741759	61747104	6.23	59.2
	Sobic.003G360500	1890	629	Chr03	67826511	67828400	7.94	69.5
**Homogalacturonan α-1,4-galacturonosyltransferase**	Sobic.003G376700	1902	633	Chr03	69137096	69142902	8.61	72.9
	Sobic.004G151400	1605	534	Chr04	47286949	47290405	9.49	59.6

galacturonosyltransferase	Sobic.004G177000	1968	655	Chr04	52231128	52235546	7.14	73.5
	Sobic.004G237800	1479	492	Chr04	57904596	57906681	9.45	54.1
	Sobic.004G244100	1107	368	Chr04	58479292	58483614	7.65	39.9
	Sobic.004G336401	1365	454	Chr04	Nd	Nd	9.26	52.8
	Sobic.005G169500	1632	543	Chr05	55241992	55246098	8.94	61
	Sobic.006G148200	1611	536	Chr06	51887174	51894006	8.9	59.8
	Sobic.006G157800	1029	342	Chr06	52559107	52561154	8.67	37.4
	Sobic.006G232000	1668	555	Chr06	58399257	58402470	8.85	63.4
	Sobic.007G105700	1947	648	Chr07	36434038	36439962	9.09	73.4
	Sobic.008G022500	1965	654	Chr08	1979259	1984684	8.55	74.8
	Sobic.008G141800	1608	535	Chr08	49848613	49853619	8.83	61.3
	Sobic.009G144200	1896	631	Chr09	50168727	50174339	8.93	72.9
	Sobic.009G177200	1917	638	Chr09	53227518	53233544	6.5	72
	Sobic.010G092400	1515	504	Chr10	8116625	8120343	8.19	55.6
	Sobic.010G101400	1104	367	Chr10	9290654	9293194	6.46	40
	Sobic.010G274800	2094	697	Chr10	60647640	60651562	8.87	79.7
**Glucan synthase-like (Callose synthase)**	Sobic.001G521500	5865	1954	Chr01	71460239	71479822	8.85	226.3
	Sobic.001G529600	5733	1910	Chr01	72137845	72170639	8.5	218.4
	Sobic.001G542450	900	299	Chr01	34991106	34987795	9.36	34.1
	Sobic.001G542500	4260	1419	Chr01	73446521	73481162	8.57	163.1
	Sobic.003G179600	5805	1934	Chr03	46825124	46851893	8.65	224.1
	Sobic.003G180100	5520	1839	Chr03	46938290	46957823	8.62	212.6
	Sobic.003G252500	5340	1779	Chr03	59117697	59124654	9.34	205.5
	Sobic.003G298900	5373	1790	Chr03	63034808	63042311	9.19	207.6
	Sobic.004G107800	5979	1992	Chr04	10356459	10386005	8.96	228.9
	Sobic.004G358400	5859	1952	Chr04	67807797	67831774	9.14	225
	Sobic.010G064200	5718	1905	Chr10	5026475	5045681	9.08	219.1
	Sobic.010G275800	5898	1965	Chr10	60737555	60752622	9.09	226.6
	Sobic.001G033300	789	262	Chr01	2518142	2522564	9.47	28.1
	Sobic.001G155600	978	325	Chr01	12567056	12570897	4.96	34.2
	Sobic.001G155700	921	306	Chr01	12572494	12576819	5.34	32.1
	Sobic.001G237800	771	256	Chr01	24362263	24363854	8.41	26.9
	Sobic.001G237900	774	257	Chr01	24383130	24384839	9.04	27.4
	Sobic.001G238000	762	253	Chr01	24386991	24388471	8.84	26.7

Sobic.001G238100	762	253	Chr01	24421838	24423190	8.04	26.9
Sobic.001G238200	876	291	Chr01	24426929	24428398	7.96	30.9
Sobic.001G238300	756	251	Chr01	24432854	24434084	8.81	26.6
Sobic.001G238400	762	253	Chr01	24437873	24439068	8.79	26.5
Sobic.001G300400	873	290	Chr01	51003229	51006157	6.99	31.8
Sobic.001G300500	960	319	Chr01	51032339	51034185	8.68	35
Sobic.001G300700	801	266	Chr01	51049401	51051276	5.35	29.1
Sobic.001G300800	801	266	Chr01	51068040	51069913	4.84	28.8
Sobic.001G300900	927	308	Chr01	51077476	51082740	5.75	33.4
Sobic.001G301000	831	276	Chr01	51094363	51096250	9.62	29.9
Sobic.001G301300	810	269	Chr01	51120925	51122485	9.51	28.9
Sobic.001G301400	837	278	Chr01	51133796	51135477	9.56	29.9
Sobic.001G301500	849	282	Chr01	51171906	51173612	7.02	30.8
Sobic.001G301600	849	282	Chr01	51181911	51183494	6.64	30.8
Sobic.001G306200	801	266	Chr01	51849243	51850665	9.15	28.6
Sobic.001G306400	801	266	Chr01	51871602	51873087	9.15	28.7
Sobic.001G306500	801	266	Chr01	51949138	51950841	9.15	28.7
Sobic.001G311000	849	282	Chr01	52621178	52623757	9.05	30.1
Sobic.001G314600	795	264	Chr01	53093995	53095200	9.14	28.3
Sobic.001G356400	786	261	Chr01	57378835	57379893	10.2	27.4
Sobic.001G499701	750	249	Chr01	Nd	Nd	9.03	26.6
Sobic.001G499800	786	261	Chr01	69728984	69736916	8.74	27.7
Sobic.001G499900	765	254	Chr01	69738754	69740350	9.25	27.3
Sobic.001G516500	813	270	Chr01	71127185	71129535	9.03	29.2
Sobic.001G539600	771	256	Chr01	73061655	73063010	6.44	27.7
Sobic.001G539700	801	266	Chr01	73073891	73075162	6.88	28.6
Sobic.001G539760	639	212	Chr01	Nd	Nd	7.69	23
Sobic.001G539820	801	266	Chr01	Nd	Nd	6.88	28.6
Sobic.001G539880	801	266	Chr01	Nd	Nd	6.88	28.6
Sobic.001G539940	801	266	Chr01	Nd	Nd	6.88	28.6
Sobic.001G540000	801	266	Chr01	73106735	73108142	6.44	28.6
Sobic.001G542100	963	320	Chr01	73375648	73377472	5.97	33.8
Sobic.001G542200	894	297	Chr01	73388309	73389880	9.3	32.5
Sobic.002G124400	924	307	Chr02	16624579	16626226	9.44	31.9

**Expansins**	Sobic.002G124500	939	312	Chr02	16660719	16662186	9.55	33
	Sobic.002G245200	609	202	Chr02	63334168	63336239	9.57	21.7
	Sobic.002G300000	825	274	Chr02	67594751	67597346	8.72	29.2
	Sobic.002G305800	819	272	Chr02	68054464	68055997	9.43	29.1
	Sobic.002G309300	678	225	Chr02	68325990	68327575	5.31	24.7
	Sobic.003G059900	762	253	Chr03	5296389	5297767	5.21	26.7
	Sobic.003G112100	765	254	Chr03	10101271	10102475	4.96	26.8
	Sobic.003G128800	735	244	Chr03	11884393	11892299	8.08	25.1
	Sobic.003G338801	759	252	Chr03	Nd	Nd	6.38	26.4
	Sobic.003G444400	849	282	Chr03	74288048	74289733	9.57	30.1
	Sobic.004G119600	774	257	Chr04	12852924	12854072	9.46	27.5
	Sobic.004G119800	813	270	Chr04	12868357	12869693	9.16	28.7
	Sobic.004G119900	765	254	Chr04	12898835	12901007	9.85	27.1
	Sobic.004G120000	741	246	Chr04	12944986	12946010	9.51	26.4
	Sobic.004G121500	573	190	Chr04	13328396	13329454	9.19	20.4
	Sobic.004G121600	789	262	Chr04	13335583	13336955	9.22	28.2
	Sobic.004G121700	768	255	Chr04	13378885	13379925	9.39	27.5
	Sobic.004G121800	780	259	Chr04	13396833	13398175	9.02	27.8
	Sobic.004G121900	777	258	Chr04	13425251	13426897	9.13	27.7
	Sobic.004G191600	957	318	Chr04	53618541	53621643	6.49	34.6
	Sobic.004G227900	822	273	Chr04	57036678	57041683	8.08	28.5
	Sobic.004G238801	879	292	Chr04	Nd	Nd	9.43	30.3
	Sobic.004G294300	888	295	Chr04	62745287	62747348	8.84	30.7
	Sobic.004G294400	885	294	Chr04	62765215	62766991	8.82	30.7
	Sobic.004G294500	795	264	Chr04	62776146	62777961	7.49	27.4
	Sobic.006G031900	792	263	Chr06	6849558	6851202	8.63	27.9
	Sobic.006G171100	867	288	Chr06	53689065	53690823	9.87	30.5
	Sobic.006G171200	792	263	Chr06	53693353	53695031	7.5	26.9
	Sobic.006G171300	876	291	Chr06	53698484	53700078	9.6	30.6
	Sobic.006G191700	774	257	Chr06	55516806	55519853	8.53	27.8
	Sobic.006G209100	225	74	Chr06	56822713	56823206	6.53	7.9
	Sobic.007G018000	801	266	Chr07	1554328	1555546	5.63	28.4
	Sobic.007G019900	837	278	Chr07	1851340	1852657	7.47	30
	Sobic.007G020100	783	260	Chr07	1869542	1870941	6.24	28.1

	Sobic.007G020200	780	259	Chr07	1881643	1883708	6.24	27.9
	Sobic.007G166500	825	274	Chr07	58899108	58899971	9.11	28.8
	Sobic.009G173700	747	248	Chr09	52935907	52938070	6.87	25.8
	Sobic.010G006200	831	276	Chr10	503711	505054	8.51	29.2
	Sobic.010G120100	780	259	Chr10	13583914	13585172	7.45	26.9
	Sobic.010G121800	780	259	Chr10	14054685	14056373	6.79	26.7
	Sobic.010G194500	786	261	Chr10	53449336	53453204	6.88	28
	Sobic.010G268700	813	270	Chr10	60165123	60166338	9.53	28.6
	Sobic.010G271000	1332	443	Chr10	60355385	60357901	9.33	47.3
**Yieldins**	Sobic.001G543000	912	303	Chr01	73517463	73518374	5.04	32.1
	Sobic.002G055600	924	307	Chr02	5311268	5313173	6.59	33.6
	Sobic.002G055700	924	307	Chr02	5317658	5319344	9	33.6
	Sobic.002G109700	894	297	Chr02	13367633	13368526	4.54	31.4
	Sobic.002G109800	894	297	Chr02	13371390	13372283	4.54	31.5
	Sobic.002G109900	894	297	Chr02	13375230	13376123	4.57	31.5
	Sobic.002G110000	762	253	Chr02	13381185	13382078	5.04	26.8
	Sobic.003G244600	1017	338	Chr03	58420673	58422420	5.04	35.2
	Sobic.003G263600	1116	371	Chr03	60135392	60137277	4.82	39
	Sobic.003G363900	903	300	Chr03	68147875	68149144	8.84	31.9
	Sobic.003G364000	900	299	Chr03	68154049	68155143	4.83	31.5
	Sobic.003G364100	900	299	Chr03	68157176	68158448	5.02	31.4
	Sobic.003G364200	894	297	Chr03	68159340	68160753	8.82	31.9
	Sobic.003G364300	912	303	Chr03	68161779	68163386	8.84	31.6
	Sobic.005G098700	921	306	Chr05	16214271	16215781	9.34	33.4
	Sobic.005G099000	939	312	Chr05	16278972	16280400	8.83	34.3
	Sobic.005G110600	1341	446	Chr05	25448795	25450699	4.73	48.2
	Sobic.005G177100	891	296	Chr05	56326620	56327799	8.18	32.1
	Sobic.005G177400	882	293	Chr05	56351932	56352830	8.24	32.2
	Sobic.005G177500	924	307	Chr05	56360364	56361692	6.12	33.3
	Sobic.005G177600	909	302	Chr05	56373524	56374754	7.69	32.9
	Sobic.005G224900	897	298	Chr05	61548910	61550480	7.15	32.3
	Sobic.006G044200	1404	467	Chr06	30761399	30763596	7.15	48.8
	Sobic.007G006501	1884	627	Chr06	Nd	Nd	6.38	68
	Sobic.001G006900	855	284	Chr01	697346	700015	7.09	32.2

Xyloglucan endotransglucosylases /hydrolases	Sobic.001G179400	969	322	Chr01	15145382	15147482	5.3	35.3
	Sobic.001G284600	1056	351	Chr01	48643305	48648643	6.2	38.2
	Sobic.001G309000	930	309	Chr01	52286915	52289432	5.49	34.5
	Sobic.001G441700	1200	399	Chr01	64759977	64764238	7.67	43.1
	Sobic.001G531300	795	264	Chr01	72299812	72301081	4.78	29
	Sobic.001G538000	1023	340	Chr01	72899185	72901801	7.07	37.8
	Sobic.002G194500	1032	343	Chr02	58178836	58181197	6.63	37.4
	Sobic.002G302000	945	314	Chr02	67820294	67822321	8.75	35
	Sobic.002G324100	960	319	Chr02	69471941	69475675	5.23	34.8
	Sobic.004G025900	1116	371	Chr04	2086783	2088615	9.4	41.1
	Sobic.004G126700	888	295	Chr04	15156403	15158280	5.04	32.3
	Sobic.004G127200	873	290	Chr04	15599208	15600956	6.08	32
	Sobic.004G273200	1056	351	Chr04	61013787	61016300	7.7	38.8
	Sobic.005G140001	912	303	Chr05	Nd	Nd	6.17	34.5
	Sobic.006G205500	912	303	Chr06	56462354	56463977	5.12	33.4
	Sobic.006G205600	855	284	Chr06	56466488	56468248	4.94	31
	Sobic.006G205700	897	298	Chr06	56474562	56476629	4.89	34.1
	Sobic.006G228100	978	325	Chr06	58198167	58200279	5.3	35.6
	Sobic.007G085500	882	293	Chr07	10626140	10627479	4.74	33.5
	Sobic.007G085600	861	286	Chr07	10641082	10642567	5.69	32
	Sobic.007G086300	864	287	Chr07	11264293	11265901	8.09	32.9
	Sobic.007G086400	876	291	Chr07	11322673	11323788	5.17	33.3
	Sobic.007G090436	843	280	Chr07	Nd	Nd	4.89	30.8
	Sobic.007G090460	834	277	Chr07	Nd	Nd	5.97	30.9
	Sobic.007G090463	864	287	Chr07	Nd	Nd	5.53	31.9
	Sobic.007G094900	876	291	Chr07	16132743	16134515	4.85	32.1
	Sobic.010G098000	873	290	Chr10	8812597	8814320	6.22	32.1
	Sobic.010G098100	882	293	Chr10	8817467	8819143	4.88	32.5
	Sobic.010G146300	885	294	Chr10	41347815	41349456	5.43	33
	Sobic.010G246400	879	292	Chr10	58406448	58408253	9.2	32.5
	Sobic.010G246500	948	315	Chr10	58415485	58418213	9.05	35.5
	Sobic.010G246600	867	288	Chr10	58418559	58420741	6.51	31.4
	Sobic.010G246700	906	301	Chr10	58474498	58476331	7.01	33.4
	Sobic.K044406	489	162	Scafold	Nd	Nd	6.54	18.8

Endo-1,4-β-glucanases	Sobic.001G099100	1866	621	Chr01	7577941	7581684	9.15	69.2
	Sobic.001G384600	1863	620	Chr01	60010081	60015027	9.27	68.5
	Sobic.002G193600	1629	542	Chr02	57985777	57996449	6.63	59.5
	Sobic.002G273500	1680	559	Chr02	65633945	65638150	5.91	61.1
	Sobic.002G276600	1578	525	Chr02	65892502	65897134	5.94	57
	Sobic.003G015700	1965	654	Chr03	1426531	1429785	7.71	69.7
	Sobic.003G015800	1635	544	Chr03	1430662	1436632	7.74	58
	Sobic.003G148500	1506	501	Chr03	15452546	15454941	9.05	54.4
	Sobic.004G021400	1545	514	Chr04	1681847	1684239	6.22	54.2
	Sobic.004G042700	1608	535	Chr04	3513524	3518030	5.25	57.9
	Sobic.004G244600	2277	758	Chr04	58541365	58545659	9.15	82.9
	Sobic.004G248100	1515	504	Chr04	58849084	58852695	8.3	54.8
	Sobic.004G318300	1494	497	Chr04	64702572	64705325	6.54	54.2
	Sobic.006G090900	1548	515	Chr06	46990664	46992790	8.41	55.3
	Sobic.006G134700	1893	630	Chr06	50705025	50708100	8.61	69.5
	Sobic.006G265100	1893	630	Chr06	60841552	60844677	5.85	68.2
	Sobic.006G282300	1872	623	Chr06	Nd	Nd	9.05	69
	Sobic.007G017300	1509	502	Chr07	1488561	1490891	8.33	53.4
	Sobic.007G119300	1554	517	Chr07	50085403	50089321	5.56	56.1
	Sobic.007G131300	1521	506	Chr07	53495571	53497775	7.71	56.2
	Sobic.009G029800	1170	389	Chr09	2696742	2698194	5.67	41.5
	Sobic.009G079900	1884	627	Chr09	10726507	10729257	8.6	68.7
	Sobic.010G101900	1326	441	Chr10	9363274	9365802	6.01	48.4
	Sobic.010G106100	1689	562	Chr10	10112994	10117093	9.07	60.7
	Sobic.010G266100	1569	522	Chr10	59986970	59990344	5.52	56.9
	Sobic.K044400	228	75	Scafold	Nd	Nd	8.25	7.6
Endo-xylanases	Sobic.001G140300	2247	748	Chr01	11166192	11169469	5.25	81.8
	Sobic.001G439400	1683	560	Chr01	64557271	64560840	5.56	62.8
	Sobic.001G466000	1755	584	Chr01	66790101	66795178	6.38	64.9
	Sobic.001G466100	1719	572	Chr01	66796146	66798756	6.18	63.6
	Sobic.001G466400	1734	577	Chr01	66806878	66809917	5.72	62.9
	Sobic.002G053800	1809	602	Chr02	5134466	5137963	8.75	66.4
	Sobic.002G127400	3297	1098	Chr02	17590856	17597926	6.76	123.4
	Sobic.003G082700	1695	564	Chr03	7091476	7094270	5.18	61.7

	Sobic.003G083000	1785	594	Chr03	7122670	7125955	5.8	65
	Sobic.004G154000	1923	640	Chr04	48031255	48036744	6.76	69.5
	Sobic.006G032800	1725	574	Chr06	7326100	7330447	5.73	63.2
**Glucan 1,3-β-glucosidases**	Sobic.001G014700	1452	483	Chr01	1315812	1317859	8.06	51.2
	Sobic.001G061900	1488	495	Chr01	4575461	4578290	4.77	53.6
	Sobic.001G109400	1338	445	Chr01	8493206	8496413	5.32	45.9
	Sobic.001G142200	1428	475	Chr01	11385147	11388355	4.96	48.5
	Sobic.001G148300	1482	493	Chr01	11913906	11917964	4.8	52.3
	Sobic.001G263500	1305	434	Chr01	41128238	41130968	6.05	45.1
	Sobic.001G346200	1509	502	Chr01	56377094	56378602	6.99	53.6
	Sobic.001G357900	1362	453	Chr01	57569368	57576913	5.67	45.9
	Sobic.001G404000	1464	487	Chr01	61708127	61711499	7.62	52.4
	Sobic.001G437801	3285	1094	Chr01	Nd	Nd	7.82	116
	Sobic.001G445700	1299	432	Chr01	65045419	65049764	4.84	44.7
	Sobic.001G452900	1509	502	Chr01	65747366	65752037	5.46	53.5
	Sobic.002G045800	1473	490	Chr02	4344192	4348862	5.35	52
	Sobic.002G084700	1521	506	Chr02	9036692	9039354	4.86	51.9
	Sobic.002G148900	1479	492	Chr02	40500313	40504434	5.48	52.4
	Sobic.002G255600	1452	483	Chr02	64136334	64140798	8.84	52.4
	Sobic.002G275800	1041	346	Chr02	65828447	65829733	6.23	35.7
	Sobic.002G314500	1626	541	Chr02	68783910	68790375	5.49	57.1
	Sobic.002G327900	1704	567	Chr02	69721953	69724502	5.22	60.5
	Sobic.002G328200	1503	500	Chr02	69742552	69745061	5.84	53
	Sobic.002G328300	1746	581	Chr02	69746565	69757337	5.17	62.1
	Sobic.002G328600	1986	661	Chr02	69774798	69778061	4.96	69.3
	Sobic.002G351700	1485	494	Chr02	71506062	71510843	5.71	52.7
	Sobic.003G290400	1494	497	Chr03	62325921	62329413	5.41	53.9
	Sobic.003G326700	1092	363	Chr03	65278907	65281501	5.8	37.7
	Sobic.003G364600	1227	408	Chr03	68178942	68182058	5.46	43.3
	Sobic.003G421500	1011	336	Chr03	72685094	72686219	4.31	35
	Sobic.003G421700	999	332	Chr03	72688222	72690076	4.84	34.5
	Sobic.003G421900	1056	351	Chr03	72695078	72698897	5.8	37.1
	Sobic.003G422000	1014	337	Chr03	72704280	72705932	9.44	35.6
	Sobic.003G422100	1026	341	Chr03	72716412	72718511	5.34	35.8

	Sobic.003G422200	1011	336	Chr03	72722323	72723949	4.8	34.9
	Sobic.003G423500	954	317	Chr03	72810315	72811268	5.08	33.3
	Sobic.004G036800	1470	489	Chr04	2968510	2973930	5.24	53.3
	Sobic.004G083100	1263	420	Chr04	6858753	6861592	8.39	46.1
	Sobic.004G165600	1230	409	Chr04	50814381	50818619	7.66	43.6
	Sobic.004G313200	1476	491	Chr04	64311087	64314974	8.04	50.9
	Sobic.005G164000	1494	497	Chr05	54352857	54355212	6.19	52.6
	Sobic.005G228900	1404	467	Chr05	62007045	62011417	6.08	49.2
	Sobic.006G069200	1233	410	Chr06	43194284	43199899	5.52	44.5
	Sobic.006G271100	1395	464	Chr06	61234368	61236684	5.15	49.6
	Sobic.007G078800	1485	494	Chr07	9036320	9040471	5.42	52
	Sobic.007G197000	1455	484	Chr07	61622408	61625527	6.59	50.5
	Sobic.008G146700	993	330	Chr08	50505280	50507078	4.45	34.3
	Sobic.009G119200	1008	335	Chr09	46854000	46859416	6.41	35
	Sobic.009G119400	1014	337	Chr09	46933087	46936543	4.84	35
	Sobic.009G154600	1233	410	Chr09	51156082	51162219	5.68	43.7
	Sobic.009G183400	1035	344	Chr09	53702821	53706747	4.72	35.4
	Sobic.009G201400	1035	344	Chr09	55107726	55108736	6.38	36.1
	Sobic.009G210900	1461	486	Chr09	55713257	55716692	8.73	53.1
	Sobic.010G022500	1395	464	Chr10	1856012	1858268	8.95	47.7
	Sobic.010G155500	1227	408	Chr10	45826998	45831189	6.32	43.7
	Sobic.010G177600	1479	492	Chr10	51304038	51306052	5.94	53.7
	Sobic.010G186700	1179	392	Chr10	52480490	52485036	6.71	43.4
	Sobic.001G026800	1428	475	Chr01	2088840	2093170	6.26	51.6
	Sobic.001G045400	1404	467	Chr01	3356260	3358235	9.48	50.3
	Sobic.001G045800	1359	452	Chr01	3386937	3389188	8.28	47.6
	Sobic.001G045900	1311	436	Chr01	3394786	3396286	9.6	46.7
	Sobic.001G454800	1293	430	Chr01	65925339	65929147	5.61	46.8
	Sobic.001G525000	1392	463	Chr01	71732208	71736315	6.01	49.2
	Sobic.002G090400	1500	499	Chr02	9451324	9455165	6.36	54.1
	Sobic.002G214900	1557	518	Chr02	60674506	60678987	9.3	56.4
	Sobic.002G246400	1389	462	Chr02	63421798	63424030	7.19	50.4
	Sobic.003G050100	1275	424	Chr03	4573572	4578203	8.47	46.5
	Sobic.003G141800	1521	506	Chr03	14062451	14065033	6.56	52.6

Polygalacturonases	Sobic.003G153100	2079	692	Chr03	16330373	16333442	8.42	66.2
	Sobic.003G153200	1563	520	Chr03	16370011	16373026	5.05	55.1
	Sobic.003G173800	1242	413	Chr03	40588540	40590578	7.43	42.8
	Sobic.003G187700	1311	436	Chr03	50690711	50693085	9.23	45.8
	Sobic.003G223100	1344	447	Chr03	55919994	55922978	7.06	47.4
	Sobic.003G226000	1215	404	Chr03	56258885	56260919	5.72	41.2
	Sobic.003G232600	1599	532	Chr03	57208067	57213160	8.36	57.2
	Sobic.003G384300	1980	659	Chr03	69727171	69734519	7.27	71
	Sobic.004G028700	1563	520	Chr04	2298817	2302914	4.79	55
	Sobic.004G080000	1122	373	Chr04	6595611	6597440	8.32	39.7
	Sobic.004G113900	1347	448	Chr04	11542051	11545484	9.09	48.1
	Sobic.004G319600	1503	500	Chr04	64776734	64779942	9.22	54.4
	Sobic.005G204700	1356	451	Chr05	59434687	59438872	5.02	48.3
	Sobic.006G178900	1263	420	Chr06	54250418	54253081	6.19	43.9
	Sobic.007G006700	1494	497	Chr07	580875	584935	6.07	53.6
	Sobic.007G112400	1176	391	Chr07	41934156	41936399	8.92	42
	Sobic.007G180450	1968	655	Chr07	Nd	Nd	6.57	69.4
	Sobic.009G216100	1227	408	Chr09	56070704	56079004	7.8	43.2
	Sobic.009G216300	1407	468	Chr09	56083047	56085769	6.22	49.7
	Sobic.009G243500	1614	537	Chr09	57975578	57980534	7.49	57.4
	Sobic.009G250800	1320	439	Chr09	58586031	58588877	8.16	46.6
	Sobic.010G005000	1488	495	Chr10	419980	423864	6.03	52.7
	Sobic.010G040400	1188	395	Chr10	3172505	3174574	9.02	42.2
	Sobic.010G161400	1269	422	Chr10	47645042	47647019	7.37	43.9
	Sobic.010G190001	1233	410	Chr10	Nd	Nd	7.77	42.8
	Sobic.010G190100	1233	410	Chr10	52859840	52861573	7.77	42.8
	Sobic.010G190200	1215	404	Chr10	52867612	52869470	9.11	42.8
β-Galactosidases	Sobic.001G433200	2583	860	Chr01	63988237	63995311	5.44	92.9
	Sobic.001G493900	2529	842	Chr01	69108941	69115502	6.1	92.7
	Sobic.002G279900	2514	837	Chr02	66129522	66136017	6.47	94
	Sobic.003G179700	2493	830	Chr03	46858636	46862978	7.97	91.2
	Sobic.003G197100	2484	827	Chr03	52262414	52271858	5.86	91.8
	Sobic.003G374100	2532	843	Chr03	68937532	68948342	6.46	93.8
	Sobic.004G093400	2223	740	Chr04	7984500	7989429	9.05	81.7

	Sobic.007G012100	306	101	Chr07	1115227	1116028	6.72	12
	Sobic.007G176900	2547	848	Chr07	59794839	59799752	9.01	95.5
	Sobic.008G052200	2772	923	Chr08	5222367	5239297	5.06	101.9
	Sobic.009G146500	2511	836	Chr09	50373803	50379051	6.09	93.2
	Sobic.009G213100	2475	824	Chr09	55874575	55882391	8.27	92.1
	Sobic.010G173800	2178	725	Chr10	50687063	50691941	7.9	79.1
**Pectate and pectin lyases**	Sobic.001G230400	1449	482	Chr01	22215126	22219254	5.88	51
	Sobic.003G322800	1122	373	Chr03	64987613	64989078	10.02	40.5
	Sobic.003G348500	1095	364	Chr03	66901151	66902694	5.53	39.9
	Sobic.004G091000	1338	445	Chr04	7758990	7761156	9.2	49.6
	Sobic.006G014400	1455	484	Chr06	2042084	2049188	7.28	51.6
	Sobic.008G022800	834	277	Chr08	2001682	2003069	5.66	30.1
	Sobic.008G058200	1107	368	Chr08	6042261	6045129	7.02	40.6
	Sobic.010G034200	1398	465	Chr10	2788038	2791467	9.16	51.5
	Sobic.010G034300	1329	442	Chr10	2794362	2795687	9.36	49.3
	Sobic.010G176400	1365	454	Chr10	51190894	51193172	8.96	51.1
**Rhamnogalacturonan I lyases**	Sobic.005G024000	2115	704	Chr05	2160262	2167202	6.29	76.7
	Sobic.005G024100	2109	702	Chr05	2177213	2181475	9.1	76.7
	Sobic.005G123300	2160	719	Chr05	44334382	44336974	5.3	78.9
	Sobic.007G173300	1992	663	Chr07	59571867	59576508	5.05	75.5
	Sobic.008G016500	2202	733	Chr08	1363548	1368749	5.85	79.6
	Sobic.009G259300	2127	708	Chr09	59282435	59286218	5.36	77.4
**Pectin methyl esterases**	Sobic.001G252300	1146	381	Chr01	27372227	27375221	8.78	42.3
	Sobic.001G490800	897	298	Chr01	68868388	68870877	5.64	32.9
	Sobic.002G138400	963	320	Chr02	21503259	21504275	5.69	33.7
	Sobic.002G251600	960	319	Chr02	63788602	63790031	5.71	34.4
	Sobic.002G370300	1089	362	Chr02	72912536	72916286	9.18	38.7
	Sobic.002G399700	1098	365	Chr02	75020920	75022746	5.07	39.2
	Sobic.003G143600	1020	339	Chr03	14333041	14334462	6.44	36.6
	Sobic.003G178000	1041	346	Chr03	45551057	45552930	8.17	37.9
	Sobic.003G231500	900	299	Chr03	57117754	57119775	9	32.2
	Sobic.003G292100	1185	394	Chr03	62488663	62493025	8.6	42.8
	Sobic.003G376900	1620	539	Chr03	69150059	69153095	6.91	57.4
	Sobic.004G277501	897	298	Chr04	Nd	Nd	6.64	31.8

	Sobic.004G350400	1245	414	Chr04	67225698	67227491	5.35	44.4
	Sobic.004G350500	1143	380	Chr04	67236279	67239144	7.7	41.3
	Sobic.005G009600	1122	373	Chr05	837586	839937	5.99	40.3
	Sobic.005G009900	999	332	Chr05	859575	861373	7.26	36.2
	Sobic.005G217101	1887	628	Chr05	Nd	Nd	9.47	66.5
	Sobic.006G086900	1407	468	Chr06	46520446	46523533	9	50.7
	Sobic.006G172000	1053	350	Chr06	53744734	53747178	8.98	38.5
	Sobic.007G075600	1200	399	Chr07	8443656	8446289	5.17	43.7
	Sobic.008G131300	1299	432	Chr08	48280274	48282200	7.6	46.2
	Sobic.009G203200	1221	406	Chr09	55218716	55221278	9.54	44.2
	Sobic.010G017600	630	209	Chr10	1430406	1432835	6.06	21.8
**Pectin acetyl esterases**	Sobic.001G305100	1257	418	Chr01	51725695	51733108	6.45	45.4
	Sobic.002G388600	1245	414	Chr02	74195323	74199442	8.64	45.4
	Sobic.003G094800	1368	455	Chr03	8251085	8254749	7.85	49.7
	Sobic.003G150600	1362	453	Chr03	15873072	15878845	8.85	48.5
	Sobic.003G150700	1254	417	Chr03	15882592	15886034	8.6	44.9
	Sobic.003G384700	1068	355	Chr03	69756168	69760794	8.36	39.1
	Sobic.003G384800	1221	406	Chr03	69762598	69766544	5.33	44.5
	Sobic.003G384900	1188	395	Chr03	69766824	69769796	6.54	43.2
	Sobic.003G445000	1395	464	Chr03	74327807	74332328	9.74	52.7
	Sobic.004G269400	1197	398	Chr04	60684399	60688674	5.51	43.1
	Sobic.006G204500	1185	394	Chr06	56392044	56396526	8.77	43
	Sobic.009G013700	1305	434	Chr09	1191020	1195391	9.03	48.4
	Sobic.001G403100	1803	600	Chr01	61635345	61638454	7.02	64.9
	Sobic.001G422300	1731	576	Chr01	63123730	63127220	8.49	62.6
	Sobic.003G111900	1797	598	Chr03	10082490	10087497	6.31	66.2
	Sobic.003G231400	1476	491	Chr03	57100064	57101932	9.32	53.4
	Sobic.003G341500	1707	568	Chr03	66443112	66445821	6.01	62.8
	Sobic.003G352700	1740	579	Chr03	67179436	67183682	7.67	63.1
	Sobic.003G352800	1740	579	Chr03	67187565	67190664	8.02	63.1
	Sobic.003G353200	1740	579	Chr03	67223693	67226882	8.5	63.5
	Sobic.003G357500	1698	565	Chr03	67584902	67588785	5.66	61.2
	Sobic.003G357600	1950	649	Chr03	67591181	67595377	5.88	68
	Sobic.003G357700	1674	557	Chr03	67596886	67599560	6.49	60.2

**Laccases**	Sobic.004G235900	1776	591	Chr04	57686461	57689009	6.38	65.4
	Sobic.004G236000	1773	590	Chr04	57697569	57700658	6.06	65.4
	Sobic.004G236100	1722	573	Chr04	57706412	57709268	6.23	63.9
	Sobic.004G314200	1812	603	Chr04	64392880	64395597	6.45	67.4
	Sobic.004G314300	1776	591	Chr04	64403091	64405636	7.08	65.4
	Sobic.005G005800	1764	587	Chr05	456102	461051	5.83	63.2
	Sobic.005G156700	1665	554	Chr05	53229146	53231126	7.09	61.3
	Sobic.005G163800	1725	574	Chr05	54327798	54330263	5.75	63.2
	Sobic.005G198500	1806	601	Chr05	58784229	58788152	6.02	66.6
	Sobic.005G215300	1803	600	Chr05	60517452	60521498	5.75	65.4
	Sobic.008G090800	1731	576	Chr08	27803994	27806772	5.19	63.1
	Sobic.009G162300	1758	585	Chr09	51936507	51939258	9.32	63.3
	Sobic.009G162800	1758	585	Chr09	51989967	51993919	5.95	63.2
	Sobic.010G268500	1818	605	Chr10	60152190	60155037	9.08	66.3
