# Supplementary material for: Identification, Characterization, and Expression Analysis of Cell Wall Related Genes in Sorghum bicolor (L.) Moench, a Food, Fodder, and Biofuel Crop
Source: Front Plant Sci. 2016 Aug 31;7:1287. doi: 10.3389/fpls.2016.01287 (PMC5006623; doi:10.3389/fpls.2016.01287)
Supplement: Supplementary file 2 [file Table2.PDF]

Supplementary Table 2. Details of SSRs identification from the 520 cell wall related genes of sorghum.

Parameters	Statistics
Total number of genes examined	520
Total size of examined sequences (bp)	791145
Total number of identified SSRs	137
Number of SSR containing genes	112
Number of genes containing more than 1 SSR	20
Number of SSRs present in compound formation	6
Number of DNRs	24
Number of TNRs	111
Number of TtNRs	2
