# Supplementary material for: Identification, Characterization, and Expression Analysis of Cell Wall Related Genes in Sorghum bicolor (L.) Moench, a Food, Fodder, and Biofuel Crop
Source: Front Plant Sci. 2016 Aug 31;7:1287. doi: 10.3389/fpls.2016.01287 (PMC5006623; doi:10.3389/fpls.2016.01287)
Supplement: Supplementary file 3 [file Table3.PDF]

Supplementary Table 3. Details of SSRs present in cell wall related genes of sorghum.

S. No.	Gene ID	SSR	size	start	end
1	Sobic.001G224300.1-1	(CGA)7	21	513	533
2	Sobic.001G224300.1-2	(TGC)6	18	1792	1809
3	Sobic.001G224300.1-3	(CGG)5	15	1929	1943
4	Sobic.002G094600.1-1	(GGC)5	15	562	576
5	Sobic.002G094600.1-2	(GA)5	10	1024	1033
6	Sobic.002G118700.2-1	(GA)5	10	982	991
7	Sobic.009G063400.1-1	(GAG)5	15	683	697
8	Sobic.010G183700.1-1	(GAG)6	18	1330	1347
9	Sobic.001G283400.1-1	(GGC)5	15	247	261
10	Sobic.002G022700.1-1	(ACG)5….(GGA)5	57	122	178
11	Sobic.002G171200.3-1	(GCG)5	15	257	271
12	Sobic.002G171200.3-2	(TCG)5	15	2015	2029
13	Sobic.002G334300.1-1	(GAG)8	24	130	153
14	Sobic.002G385800.1-1	(CGC)5	15	42	56
15	Sobic.003G308100.1-1	(GGC)6	18	31	48
16	Sobic.003G442500.1-1	(CGT)5	15	1279	1293
17	Sobic.003G442500.1-2	(GC)5	10	1904	1913
18	Sobic.006G080700.1-1	(CGA)5	15	570	584
19	Sobic.006G080800.1-1	(CGG)5	15	147	161
20	Sobic.007G050600.1-1	(CAA)6	18	60	77
21	Sobic.007G090600.1-1	(GCC)5	15	2065	2079
22	Sobic.008G125700.1-1	(CGG)5	15	822	836
23	Sobic.008G125700.1-2	(CGG)5	15	3648	3662
24	Sobic.002G116800.1-1	(AG)5	10	922	931
25	Sobic.004G164000.1-1	(GGA)6	18	37	54
26	Sobic.004G256400.1-1	(GGT)5	15	181	195
27	Sobic.005G144000.1-1	(CG)5	10	82	91
28	Sobic.005G144101.1-1	(CG)5	10	106	115
72	Sobic.008G035700.1-1	(GCG)5	15	53	67
73	Sobic.008G035700.1-2	(GTC)6	18	609	626
74	Sobic.008G035800.1-1	(GCG)6	18	172	189
75	Sobic.002G019200.2-1	(CGA)5	15	1053	1067
76	Sobic.006G097900.1-1	(CGG)5	15	1241	1255
77	Sobic.008G054100.1-1	(GGC)6	18	1432	1449
78	Sobic.010G082400.2-1	(GC)5	10	1534	1543
79	Sobic.001G229100.1-1	(GGC)6	18	406	423
80	Sobic.001G387300.1-1	(CCT)8	24	8	31
81	Sobic.001G506500.1-1	(CGG)5	15	258	272

82	Sobic.001G506600.1-1	(GC)5	10	1016	1025
83	Sobic.002G062100.1-1	(GTC)7……(CGG)6	114	174	287
84	Sobic.003G410600.1-1	(GC)5	10	737	746
85	Sobic.006G059000.1-1	(CGG)5	15	90	104
86	Sobic.006G186200.1-1	(CCG)6	18	143	160
87	Sobic.007G139300.1-1	(TGG)7	21	2	22
88	Sobic.008G077900.1-1	(GC)5	10	913	922
89	Sobic.010G059400.1-1	(TCT)5	15	136	150
90	Sobic.010G059400.1-2	(CGT)5	15	304	318
91	Sobic.010G059400.1-3	(CGG)5	15	597	611
92	Sobic.010G059400.1-4	(GC)5	10	1179	1188
93	Sobic.001G364700.2-1	(TTG)5	15	41	55
94	Sobic.001G391300.1-1	(GCC)5	15	43	57
95	Sobic.001G460000.1-1	(CCG)5	15	103	117
96	Sobic.001G479800.1-1	(CGT)5	15	209	223
97	Sobic.003G360500.1-1	(GCA)6	18	731	748
98	Sobic.004G177000.1-1	(CG)6	12	741	752
99	Sobic.004G237800.1-1	(GC)5	10	345	354
100	Sobic.010G092400.1-1	(CG)5	10	485	494
101	Sobic.010G274800.1-1	(TCC)5	15	7	21
102	Sobic.003G298900.1-1	(GCG)6	18	31	48
103	Sobic.010G275800.1-1	(AG)6	12	2467	2478
104	Sobic.001G311000.1-1	(GTC)5	15	61	75
105	Sobic.001G542100.1-1	(GTG)5	15	248	262
106	Sobic.003G444400.1-1	(CAA)5	15	69	83
107	Sobic.004G121500.1-1	(TA)5	10	429	438
108	Sobic.007G166500.1-1	(GCG)7	21	373	393
109	Sobic.005G177500.1-1	(GCA)6	18	55	72
110	Sobic.007G006501.1-1	(CAG)6	18	1704	1721
111	Sobic.001G179400.1-1	(GC)5	10	886	895
112	Sobic.001G284600.1-1	(CGA)5	15	711	725
113	Sobic.002G194500.1-1	(GCT)5	15	15	29
114	Sobic.002G324100.1-1	(GGC)5	15	574	588
29	Sobic.004G025900.1-1	(GCC)6	18	1051	1068
30	Sobic.004G127200.1-1	(GCG)6	18	671	688
31	Sobic.006G228100.1-1	(GC)5	10	895	904
32	Sobic.007G094900.1-1	(CGG)5	15	684	698
33	Sobic.010G246700.1-1	(GCG)8	24	704	727
34	Sobic.001G099100.1-1	(AAG)5	15	163	177
35	Sobic.002G193600.1-1	(CTC)6	18	64	81
36	Sobic.004G244600.1-1	(CG)6….(CG)5	98	389	486

37	Sobic.006G265100.1-1	(CGG)5	15	918	932
38	Sobic.007G017300.1-1	(CTC)6	18	49	66
39	Sobic.007G119300.1-1	(GTA)5	15	49	63
40	Sobic.010G101900.2-1	(CTC)7….(GGC)5	69	831	899
41	Sobic.010G101900.2-2	(GGC)5	15	1269	1283
42	Sobic.010G106100.1-1	(ACCA)5	20	98	117
43	Sobic.003G083000.1-1	(CGG)5	15	35	49
44	Sobic.004G154000.1-1	(CA)5	10	138	147
45	Sobic.001G109400.1-1	(CG)5	10	846	855
46	Sobic.001G142200.1-1	(GGA)7	21	1102	1122
47	Sobic.001G357900.1-1	(TAC)5	15	673	687
48	Sobic.001G357900.1-2	(CCA)5	15	953	967
49	Sobic.001G404000.1-1	(CCT)6	18	45	62
50	Sobic.002G327900.1-1	(GGC)5	15	853	867
51	Sobic.002G328600.1-1	(CGA)6….(CGA)5	51	681	731
52	Sobic.002G328600.1-2	(CGG)5	15	1164	1178
53	Sobic.005G228900.1-1	(CTC)7	21	10	30
54	Sobic.006G069200.1-1	(CTT)5	15	45	59
55	Sobic.009G210900.1-1	(CG)5	10	292	301
56	Sobic.001G525000.1-1	(CG)5	10	220	229
57	Sobic.002G246400.1-1	(TGC)8	24	38	61
58	Sobic.003G141800.1-1	(CCA)5	15	96	110
59	Sobic.003G153100.1-1	(GGA)6	18	1927	1944
60	Sobic.003G153200.1-1	(CGT)5	15	43	57
61	Sobic.003G153200.1-2	(CGC)5	15	290	304
62	Sobic.004G028700.1-1	(GAC)6	18	223	240
63	Sobic.004G028700.1-2	(GAG)7	21	1495	1515
64	Sobic.004G113900.1-1	(GGC)7	21	66	86
65	Sobic.007G180450.1-1	(TCG)6	18	38	55
66	Sobic.007G180450.1-2	(GAC)5	15	453	467
67	Sobic.001G433200.1-1	(GCG)5	15	46	60
68	Sobic.009G146500.1-1	(CGT)6	18	71	88
69	Sobic.010G173800.1-1	(GGC)5	15	36	50
70	Sobic.001G230400.1-1	(GCC)5	15	55	69
71	Sobic.006G014400.1-1	(CGA)5	15	230	244
115	Sobic.008G058200.3-1	(CCG)6	18	46	63
116	Sobic.005G024100.1-1	(TGC)6	18	71	88
117	Sobic.005G024100.1-2	(CGG)6	18	1887	1904
118	Sobic.008G016500.1-1	(GCG)5	15	1358	1372
119	Sobic.009G259300.1-1	(ACG)5	15	983	997
120	Sobic.009G259300.1-2	(GAC)8	24	1746	1769

121	Sobic.001G252300.1-1	(CCTG)5	20	60	79
122	Sobic.003G231500.1-1	(GCG)5	15	119	133
123	Sobic.005G217101.1-1	(CGA)5….(CTC)5	38	449	486
124	Sobic.008G131300.1-1	(CGT)6	18	125	142
125	Sobic.010G017600.1-1	(CGC)8	24	96	119
126	Sobic.003G150600.1-1	(GCA)7	21	44	64
127	Sobic.003G150600.1-2	(GGA)5	15	176	190
128	Sobic.001G422300.1-1	(CGG)5	15	947	961
129	Sobic.004G235900.1-1	(GCG)5	15	982	996
130	Sobic.005G215300.1-1	(GCA)6	18	4	21
131	Sobic.009G162800.1-1	(GTC)6	18	37	54
