# Supplementary material for: Identification, Characterization, and Expression Analysis of Cell Wall Related Genes in Sorghum bicolor (L.) Moench, a Food, Fodder, and Biofuel Crop
Source: Front Plant Sci. 2016 Aug 31;7:1287. doi: 10.3389/fpls.2016.01287 (PMC5006623; doi:10.3389/fpls.2016.01287)
Supplement: Supplementary file 5 [file Table5.PDF]

Supplementary Table 5. Details of downloaded SRA files source from the different developmental stages expression and various treated samples of *Sorghum bicolor* for use in analysis.	
**Tissues**	**Experiment Accession**
Stem	DRX027774
	DRX027775
	DRX027776
Seed	DRX027768
	DRX027769
	DRX027770
Splikelet	DRX027771
	DRX027772
	DRX027773
Vegetative_Meristem	SRX341657
Flowers	SRX341656
Floral_Meristem	SRX341655
Embryos	SRX341654
Leaves	SRX099190
	**Stress transcriptome data**
NaOH_Sorghum_Shoot	SRX341665
H2O_Sorghum_Shoot	SRX341664
PEG_Sorghum_Shoot	SRX341663
ABA_Sorghum_Shoot	SRX341662
NaOH_Sorghum_Root	SRX341661
H2O_Sorghum_Root	SRX341660
PEG_Sorghum_Root	SRX341659
ABA_Sorghum_Root	SRX341658
