# Supplementary material for: Identification, Characterization, and Expression Analysis of Cell Wall Related Genes in Sorghum bicolor (L.) Moench, a Food, Fodder, and Biofuel Crop
Source: Front Plant Sci. 2016 Aug 31;7:1287. doi: 10.3389/fpls.2016.01287 (PMC5006623; doi:10.3389/fpls.2016.01287)
Supplement: Supplementary file 6 [file Table6.PDF]

**Supplementary Table 6. Self-normalized RPKM values calculated from mapping of transcriptome data from different sorghum tissues.**

| Gene Families | Gene | Self-normalised RPKM values | | | | | | | |
|---|---|---|---|---|---|---|---|---|---|
| | | Embryos | Veg meristem | Leaves | Seed | Stem | Floral meristem | Flowers | Spike |
| Cellulose synthases | Sobic.001G021500 | 13.5175 | 11.91686 | 11.4557 | 13.3607 | 13.1285 | 12.22596 | 11.3052 | 11.1687 |
| | Sobic.001G045700 | 12.9646 | 12.85458 | 10.6499 | 12.1264 | 11.4216 | 13.01303 | 10.6425 | 10.1024 |
| | Sobic.001G224300 | 2.28261 | 7.25519 | 8.58597 | 6.58479 | 11.5122 | 4.38639 | 11.5399 | 11.1558 |
| | Sobic.002G075500 | 12.3431 | 11.34158 | 10.435 | 12.7302 | 13.7452 | 10.94236 | 11.8445 | 11.8888 |
| | Sobic.002G094600 | 12.4722 | 11.04255 | 7.33593 | 11.0719 | 11.1533 | 11.53349 | 9.04716 | 8.57129 |
| | Sobic.002G118700 | 12.5922 | 10.69507 | 11.6964 | 13.2533 | 13.97 | 10.68176 | 12.3151 | 11.9374 |
| | Sobic.002G205500 | 1.29493 | 7.28092 | 8.68808 | 7.39304 | 12.0195 | 4.98427 | 12.127 | 11.9142 |
| | Sobic.003G049600 | 14.0733 | 13.27114 | 10.9397 | 13.5408 | 11.999 | 13.22012 | 10.9216 | 10.7436 |
| | Sobic.003G296400 | 6.35834 | 7.87482 | 8.17565 | 7.78468 | 12.7111 | 7.08055 | 12.502 | 12.3143 |
| | Sobic.009G063400 | 12.9219 | 11.69369 | 11.4473 | 12.4485 | 13.5298 | 11.40953 | 12.0442 | 11.6545 |
| | Sobic.010G183700 | -3.70572 | -3.70572 | 7.70192 | 2.30564 | -0.44244 | -3.70572 | 4.60653 | 3.74676 |
| Cellulose synthase like | Sobic.001G075600 | 13.0873 | 11.51942 | 7.82563 | 11.3517 | 10.5356 | 12.03305 | 11.4569 | 12.074 |
| | Sobic.001G242000 | -3.70572 | -3.70572 | 3.38129 | -0.45354 | -3.70572 | -3.70572 | -3.70572 | -3.70572 |
| | Sobic.001G252700 | 7.37923 | 9.20511 | 7.23791 | 10.5116 | 9.69244 | 9.26133 | 8.44701 | 8.34928 |
| | Sobic.001G283400 | 5.60442 | 5.97601 | 5.80774 | 4.49076 | 4.66551 | 6.05612 | 3.10133 | 3.32044 |
| | Sobic.001G490000 | 5.81803 | 6.93991 | 6.89582 | 5.0445 | 7.67206 | 6.07381 | 5.2743 | 4.25355 |
| | Sobic.002G022700 | 5.72271 | 8.65426 | 8.28189 | 4.56925 | 5.11791 | 8.77046 | 7.02207 | 7.18298 |
| | Sobic.002G139900 | 5.81016 | 2.04126 | 6.14909 | 10.0795 | 3.23792 | 1.42208 | 3.52946 | 2.50374 |
| | Sobic.002G171200 | 3.20354 | 0.69857 | 6.7563 | 1.23765 | 3.09008 | 0.40042 | 5.29057 | 3.02182 |
| | Sobic.002G237900 | 8.03078 | 0.05244 | 14.0426 | 8.35974 | 10.4321 | 1.60317 | 8.7473 | 6.32138 |
| | Sobic.002G238300 | 5.4693 | 7.02234 | 9.69066 | 9.00076 | 10.0116 | 6.48931 | 8.67233 | 7.71102 |
| | Sobic.002G333900 | 1.50933 | 2.81244 | 3.73055 | 3.14612 | 4.09925 | 4.02595 | 2.03952 | 2.40169 |
| | Sobic.002G334000 | 2.57455 | -0.19435 | -3.70572 | 3.33535 | 1.17224 | -3.70572 | -0.5135 | -0.00489 |
| | Sobic.002G334100 | 5.09092 | 5.51554 | 1.79296 | 4.44998 | 9.30608 | 5.10845 | 3.09733 | 3.05459 |
| | Sobic.002G334200 | 6.15332 | 3.54185 | -3.70572 | 4.29629 | 4.00052 | 3.24427 | 2.572 | 2.5631 |
| | Sobic.002G334300 | 4.3571 | 4.80724 | 8.02757 | 5.75402 | 6.03587 | 4.79234 | 7.16116 | 4.26488 |
| | Sobic.002G334400 | 4.33594 | 4.08162 | 1.73191 | 4.76744 | 5.28278 | 4.67967 | 11.3916 | 8.70737 |
| | Sobic.002G334500 | -3.70572 | 2.09964 | 7.74971 | 3.23324 | 6.41 | 0.27217 | 5.01128 | 3.47715 |
| | Sobic.002G385800 | 10.0366 | 10.5938 | 11.1792 | 9.95207 | 11.3078 | 10.73871 | 8.97535 | 8.94925 |

| | | | | | | | | | |
|---|---|---|---|---|---|---|---|---|---|
| **Cellulose synthase-like** | Sobic.003G308100 | 8.45524 | 8.67082 | 8.73279 | 8.78546 | 9.19941 | 8.60533 | 8.50428 | 8.13875 |
| | Sobic.003G442500 | 4.9522 | -3.70572 | 7.47745 | 6.17867 | 6.13486 | -3.70572 | 5.05178 | 3.33213 |
| | Sobic.004G075900 | 10.4221 | 12.06276 | 4.67644 | 9.19916 | 10.9607 | 12.2798 | 10.8999 | 11.1702 |
| | Sobic.004G238700 | 9.52639 | 3.79403 | -3.70572 | 9.11032 | 8.53449 | 3.7598 | 6.82536 | 6.44946 |
| | Sobic.004G255200 | 7.84844 | 9.91244 | 10.3974 | 9.81471 | 10.529 | 9.83602 | 7.68774 | 7.44179 |
| | Sobic.004G255500 | 9.73164 | 10.04351 | 11.1852 | 10.8086 | 10.905 | 10.1952 | 8.92652 | 9.14728 |
| | Sobic.006G080600 | -3.70572 | 5.76942 | 6.26235 | -3.70572 | 6.64906 | 4.04216 | 3.65936 | -0.72604 |
| | Sobic.006G080700 | -3.70572 | 3.56892 | 5.56317 | -0.31536 | 1.4334 | 2.51304 | 7.00577 | 6.31719 |
| | Sobic.006G080800 | -3.70572 | 2.30843 | 14.5968 | 2.2696 | 5.88414 | 0.92807 | 10.3604 | 9.22012 |
| | Sobic.007G050600 | 10.3221 | 9.80714 | 11.6386 | 10.8987 | 12.3535 | 10.62792 | 11.3688 | 10.9368 |
| | Sobic.007G090600 | 7.40321 | 3.31631 | 4.42712 | 5.8989 | 3.44314 | 2.54186 | 2.84474 | 2.14847 |
| | Sobic.007G100800 | -3.70572 | -3.70572 | 5.60853 | 1.70335 | -0.82233 | 0.36182 | 10.3122 | 10.119 |
| | Sobic.007G137400 | 9.72862 | 10.7555 | 7.63992 | 8.69446 | 10.3556 | 10.69228 | 8.82373 | 9.19685 |
| | Sobic.008G125700 | 8.11575 | 11.37962 | 9.83228 | 5.68302 | 7.6988 | 11.01644 | 7.60931 | 6.00565 |
| | Sobic.009G194200 | 10.8382 | 9.80512 | 6.04779 | 9.63026 | 10.6136 | 10.43295 | 9.33124 | 9.03548 |
| | Sobic.010G008600 | 12.2186 | 12.13057 | 8.8416 | 11.4117 | 11.2196 | 12.64942 | 9.9813 | 9.15512 |
| | Sobic.010G146000 | -3.70572 | 3.51509 | 4.74018 | -0.76257 | 0.29671 | 4.43506 | 9.13952 | 8.70605 |
| | Sobic.010G197300 | 4.84729 | 4.66336 | 6.94573 | 7.80074 | 8.53715 | 4.54667 | 6.63371 | 5.94498 |
| **Xyloglucan xylosyltransferases and galactomannan gal-transferases** | Sobic.001G396600 | -3.70572 | -3.70572 | 1.73029 | 2.80235 | -3.70572 | -3.70572 | 1.43892 | 0.94753 |
| | Sobic.001G396700 | 3.52697 | -3.70572 | 6.43073 | 1.48042 | -3.70572 | -3.70572 | 1.02388 | -2.05247 |
| | Sobic.001G401600 | 11.5466 | 11.06756 | 7.88004 | 10.1297 | 9.99913 | 11.6795 | 9.13066 | 8.53999 |
| | Sobic.002G116800 | 7.84844 | 8.28342 | 9.1786 | 8.63839 | 9.42885 | 8.34649 | 6.77321 | 7.06527 |
| | Sobic.003G059600 | 2.50455 | -3.70572 | 4.51523 | 2.04297 | -3.70572 | -3.70572 | -0.5835 | -0.07488 |
| | Sobic.004G164000 | 10.8135 | 11.07122 | 11.4761 | 11.1245 | 11.0939 | 11.2529 | 10.8195 | 10.2701 |
| | Sobic.004G256400 | 10.1876 | 8.61326 | -3.70572 | 12.6839 | 8.52657 | 8.27253 | 4.82578 | 2.61609 |
| | Sobic.005G144000 | 3.53142 | -3.70572 | 3.3056 | 3.08424 | 0.56134 | 0.72192 | 9.92237 | 9.44893 |
| | Sobic.005G144101 | 4.06432 | 0.72574 | 4.0046 | 2.01777 | -0.50792 | 1.69115 | 6.00884 | 6.0041 |
| | Sobic.005G144500 | 6.23157 | 7.98618 | 6.71499 | 7.28455 | 3.82926 | 8.0048 | 7.56978 | 6.67132 |
| | Sobic.008G035700 | 2.31987 | -3.70572 | 9.08951 | 1.85829 | 3.03304 | -3.70572 | 1.55375 | 3.23229 |
| | Sobic.008G035800 | 5.92635 | -3.70572 | 8.15584 | 4.59029 | -3.70572 | 0.6638 | -0.62113 | -2.11252 |
| | Sobic.002G019000 | -3.70572 | -3.70572 | 4.98827 | -3.70572 | -0.81856 | -3.70572 | -3.70572 | -3.70572 |
| | Sobic.002G019100 | 2.20957 | -3.70572 | 6.54612 | 0.16302 | -3.70572 | -3.70572 | -0.45265 | -3.70572 |
| | Sobic.002G019200 | -3.70572 | -3.70572 | 7.85393 | 0.25388 | -3.70572 | -3.70572 | 2.98392 | 0.721 |
| | Sobic.002G225900 | -3.70572 | 0.46155 | 4.60369 | 1.18389 | -3.70572 | -3.70572 | 3.22985 | 3.07727 |

| | | | | | | | | | |
|---|---|---|---|---|---|---|---|---|---|
| **Xyloglucan fucosyltransferases (MUR2)** | Sobic.004G124800 | 2.13634 | 2.36745 | 9.20059 | 1.71468 | 2.73404 | 2.65516 | 1.56152 | 0.7848 |
| | Sobic.004G125100 | -3.70572 | -3.70572 | 9.35789 | 0.09386 | -0.92296 | -3.70572 | 1.78362 | -0.0021 |
| | Sobic.004G125300 | 4.75872 | -3.70572 | -3.70572 | 7.33531 | -0.81352 | -3.70572 | 0.2817 | 1.75805 |
| | Sobic.004G308200 | 9.21481 | 2.66982 | 9.09489 | 9.40785 | 9.47603 | 2.89863 | 8.70116 | 7.92752 |
| | Sobic.004G308300 | 2.12409 | -3.70572 | 1.32741 | 1.07754 | 0.13682 | -3.70572 | 0.03604 | -3.70572 |
| | Sobic.004G308400 | 9.38933 | 7.4356 | 10.3001 | 7.65371 | 9.86646 | 8.25398 | 8.54246 | 7.6685 |
| | Sobic.004G308500 | 4.12653 | 0.35764 | 5.41731 | 2.88734 | 1.72422 | 3.32341 | -3.70572 | -2.45291 |
| | Sobic.004G308600 | 4.47064 | 6.40218 | 5.52195 | 7.09084 | 9.34259 | 6.63099 | 7.6229 | 7.30736 |
| | Sobic.006G097900 | 3.14128 | -3.70572 | 2.92956 | 1.67969 | -3.70572 | -3.70572 | -3.70572 | -2.43816 |
| | Sobic.008G054100 | -3.70572 | -3.70572 | 10.7104 | 0.02952 | 3.73266 | 0.27296 | 0.98802 | -1.50336 |
| | Sobic.010G082000 | 2.14375 | -3.70572 | 4.15443 | 0.0972 | -3.70572 | -3.70572 | -3.70572 | -3.70572 |
| | Sobic.010G082100 | 2.21736 | -3.70572 | -3.70572 | -3.70572 | -3.70572 | -3.70572 | -0.87069 | -3.70572 |
| | Sobic.010G082201 | -3.70572 | -3.70572 | -3.70572 | 0.31589 | -3.70572 | -3.70572 | -3.70572 | -3.70572 |
| | Sobic.010G082300 | -3.70572 | -3.70572 | 2.40514 | 4.6147 | 1.53648 | 0.3987 | 9.68173 | 10.3431 |
| | Sobic.010G082400 | 5.52141 | 0.29308 | 3.6441 | 6.31921 | 2.65967 | 2.25886 | 4.85657 | 4.63229 |
| **Xyloglucan galactosyltransferases** | Sobic.001G228900 | 4.48561 | 3.2169 | -3.70572 | 3.76099 | 1.0833 | 2.6825 | 1.98252 | 3.53425 |
| | Sobic.001G229000 | 3.06901 | 4.88508 | 7.2947 | 1.02246 | -3.70572 | 4.26589 | 1.98096 | 1.07453 |
| | Sobic.001G229100 | 7.29841 | 8.89876 | 5.616 | 4.5149 | 3.65218 | 8.97642 | 7.13002 | 6.53933 |
| | Sobic.001G303300 | 5.83605 | 8.62174 | 6.92689 | 7.80742 | 6.92773 | 8.68255 | 9.51486 | 8.81809 |
| | Sobic.001G387300 | 9.0612 | 8.83146 | 9.15124 | 8.38388 | 8.79274 | 9.19275 | 8.34529 | 8.02994 |
| | Sobic.001G486900 | 9.02927 | 8.84837 | 7.42751 | 9.21094 | 9.2605 | 8.77126 | 8.3357 | 8.56689 |
| | Sobic.001G506500 | 4.2357 | -3.70572 | 4.24638 | 2.77412 | -3.70572 | 0.43259 | 1.46958 | 2.01692 |
| | Sobic.001G506600 | 6.20194 | 1.859 | 2.50928 | 3.35808 | 2.36365 | -0.49715 | 3.8135 | 3.64868 |
| | Sobic.001G506700 | -3.70572 | 2.88132 | 7.35122 | 3.87092 | -3.70572 | -0.47483 | 1.05446 | 1.31931 |
| | Sobic.001G506800 | 4.89313 | 3.73616 | 4.8065 | 4.19554 | 2.84817 | 3.43125 | -0.42178 | 2.03302 |
| | Sobic.001G506900 | -3.70572 | 1.87851 | 3.85073 | 4.35164 | -3.70572 | 0.85096 | -3.70572 | -0.42498 |
| | Sobic.001G538700 | 8.56019 | 9.10404 | 8.66887 | 9.07939 | 9.25563 | 8.89873 | 7.88288 | 7.66703 |
| | Sobic.001G541601 | -3.70572 | -3.70572 | 5.84094 | 3.59106 | 1.65034 | 4.64185 | 7.38246 | 6.83954 |
| | Sobic.002G062100 | 10.3187 | 7.1248 | 7.4575 | 8.46295 | 7.39471 | 6.89028 | 6.36364 | 6.01058 |
| | Sobic.002G342300 | 10.0108 | 9.98825 | 9.55635 | 10.22 | 10.0852 | 9.95872 | 8.89082 | 8.69943 |
| | Sobic.003G102700 | 9.68374 | 8.29152 | 4.70915 | 8.43655 | 7.42063 | 8.34078 | 5.24559 | 5.74912 |
| | Sobic.003G234701 | 5.81039 | 0.58207 | 3.55428 | 6.7138 | 6.28255 | -3.70572 | 7.5758 | 8.45627 |
| | Sobic.003G331000 | 9.55514 | 10.02059 | 8.60206 | 10.2657 | 9.56044 | 9.85682 | 8.08462 | 8.24121 |
| | Sobic.003G360300 | 10.5355 | 10.11414 | 9.02515 | 10.4367 | 10.3879 | 10.05199 | 8.94375 | 9.14157 |

| | | | | | | | | | |
|---|---|---|---|---|---|---|---|---|---|
| (MUR3) | Sobic.003G405600 | 8.45552 | 8.57877 | 8.87423 | 9.18096 | 9.75051 | 8.80319 | 9.61797 | 9.57934 |
| | Sobic.003G410600 | 9.09009 | 9.68514 | 8.78481 | 9.06389 | 9.52603 | 10.18949 | 9.98556 | 8.50553 |
| | Sobic.003G410700 | 12.5396 | 12.5152 | 9.34502 | 12.2363 | 12.7096 | 12.92817 | 12.1603 | 11.5426 |
| | Sobic.003G410800 | 11.5959 | 9.25272 | 7.23955 | 10.4599 | 12.2849 | 10.52383 | 11.4715 | 10.8897 |
| | Sobic.004G070100 | 9.48025 | 9.3716 | 7.08566 | 9.35766 | 9.4849 | 9.18719 | 7.37274 | 7.74988 |
| | Sobic.004G159100 | 6.82081 | 4.61134 | 4.36116 | 8.99393 | 7.28605 | 4.57712 | 9.42881 | 9.4893 |
| | Sobic.004G213500 | -3.70572 | -3.70572 | 5.56332 | 3.21232 | -3.70572 | -3.70572 | 6.91036 | 5.41898 |
| | Sobic.006G059000 | 11.554 | 11.41707 | 12.2571 | 12.2477 | 11.7877 | 11.21731 | 11.1661 | 10.4872 |
| | Sobic.006G186100 | 6.42674 | 3.99489 | 6.84157 | 4.65583 | 4.401 | 4.18306 | 4.22005 | 3.78921 |
| | Sobic.006G186200 | 4.74614 | 6.35576 | 8.09424 | 4.0781 | 5.70373 | 7.01341 | 5.95087 | 5.44469 |
| | Sobic.006G260900 | 9.34054 | 9.64204 | 9.49104 | 10.1262 | 10.2735 | 9.5153 | 9.12296 | 9.31596 |
| | Sobic.007G139300 | -3.70572 | 1.52879 | 5.20144 | 1.83609 | 4.01085 | 3.81649 | 9.93755 | 10.3865 |
| | Sobic.008G021000 | 10.1583 | 9.67679 | 9.27371 | 10.9823 | 10.3248 | 9.57097 | 9.07839 | 8.59837 |
| | Sobic.008G077900 | 7.15566 | 5.43568 | 2.501 | 5.53653 | 3.83397 | 1.49456 | -3.70572 | -2.28176 |
| | Sobic.009G162700 | 2.60659 | 4.74459 | 6.22198 | 5.84544 | 9.44268 | 3.38843 | 10.2801 | 10.106 |
| | Sobic.009G220100 | 10.4131 | 10.18659 | 10.4324 | 11.9722 | 12.1396 | 10.03071 | 10.707 | 10.3369 |
| | Sobic.009G220200 | 10.3754 | 9.44782 | 10.6625 | 11.6983 | 11.7413 | 10.38441 | 9.59443 | 9.02443 |
| | Sobic.010G059400 | -3.70572 | -3.70572 | 6.47892 | 3.09755 | -3.70572 | 1.53363 | 0.2487 | -3.70572 |
| | Sobic.001G131900 | 12.7908 | 12.09649 | 8.13965 | 13.2617 | 11.6225 | 11.84156 | 11.3908 | 11.8171 |
| | Sobic.001G138200 | 9.84078 | 10.21094 | 10.4461 | 6.67493 | 9.68356 | 11.44624 | 8.35287 | 6.80396 |
| | Sobic.001G338400 | 10.9019 | 9.71554 | 9.40696 | 10.9105 | 11.2294 | 9.38597 | 9.1257 | 9.03435 |
| | Sobic.001G364700 | -3.70572 | 0.91705 | 5.07086 | 4.09653 | 3.15721 | 3.68788 | 10.3132 | 10.6154 |
| | Sobic.001G384200 | 10.9351 | 10.19751 | 8.04486 | 10.7087 | 9.65517 | 10.08438 | 9.78849 | 9.76877 |
| | Sobic.001G391300 | 8.00917 | 9.82868 | 12.5368 | 9.21272 | 10.7852 | 10.6804 | 10.5375 | 10.6193 |
| | Sobic.001G460000 | 11.2653 | 10.29299 | 9.9497 | 10.4439 | 10.7365 | 10.13591 | 9.822 | 9.42998 |
| | Sobic.001G479800 | 2.08793 | 5.17702 | 9.39924 | 8.35879 | 10.3138 | 2.86977 | 11.1188 | 10.4809 |
| | Sobic.002G241100 | 10.973 | 10.10186 | 7.21798 | 10.3456 | 8.91714 | 10.07267 | 8.98258 | 8.93638 |
| | Sobic.002G274700 | 11.9118 | 11.24961 | 9.11657 | 12.8746 | 10.8842 | 11.15042 | 10.4436 | 10.4908 |
| | Sobic.002G398400 | 2.84729 | 3.88862 | 8.94467 | 6.77802 | 5.72298 | 4.85152 | 10.3904 | 10.6971 |
| | Sobic.002G420100 | 10.2411 | 10.635 | 6.2842 | 11.0047 | 9.85817 | 10.81174 | 9.30378 | 9.10585 |
| | Sobic.002G423600 | -3.70572 | 3.15707 | 10.9336 | 7.91283 | 7.47007 | 1.12284 | 5.56667 | 3.63193 |
| | Sobic.003G282600 | 8.16653 | 6.62003 | 7.55042 | 5.70494 | 7.92342 | 7.25094 | 7.26962 | 5.46811 |
| | Sobic.003G360500 | -3.70572 | 0.26409 | 2.24023 | 2.98534 | -3.70572 | -3.70572 | -1.05511 | -0.54754 |
| Homogalacturonan α-1,4 | Sobic.003G376700 | 14.2621 | 12.80894 | 13.0015 | 12.0726 | 12.9907 | 13.437 | 11.3679 | 10.5095 |

| | | | | | | | | | |
|---|---|---|---|---|---|---|---|---|---|
| **Homogalacturonan α-1,4-galacturonosyltransferase** | Sobic.004G151400 | 11.3311 | 10.95311 | 12.4291 | 10.2463 | 10.8845 | 10.67404 | 9.78236 | 9.29514 |
| | Sobic.004G177000 | 1.97355 | 4.29212 | 6.03485 | 2.51196 | 4.30821 | 1.7554 | 3.64039 | 3.75167 |
| | Sobic.004G237800 | -3.70572 | 2.20791 | 6.61228 | 5.8937 | 7.82465 | -3.70572 | 5.47297 | 5.38438 |
| | Sobic.004G244100 | 10.1787 | 10.0806 | 11.0867 | 9.78221 | 12.1196 | 10.42873 | 9.77762 | 8.96888 |
| | Sobic.004G336401 | -3.70572 | -3.70572 | -3.70572 | -3.70572 | -0.48589 | -3.70572 | 6.35584 | 6.83184 |
| | Sobic.005G169500 | 8.79823 | 8.17519 | 8.05087 | 9.76884 | 8.48759 | 8.47944 | 8.16682 | 7.66842 |
| | Sobic.006G148200 | 10.462 | 9.97926 | 10.4656 | 10.5897 | 10.9563 | 9.91659 | 9.25775 | 9.22398 |
| | Sobic.006G157800 | 8.30135 | 9.08266 | 9.12611 | 7.14789 | 8.88697 | 9.5068 | 8.0009 | 6.9901 |
| | Sobic.006G232000 | 10.1605 | 9.26982 | 8.88309 | 9.62914 | 10.1075 | 9.27323 | 8.53774 | 8.5767 |
| | Sobic.007G105700 | 11.1311 | 11.02104 | 8.76978 | 10.7426 | 10.2532 | 10.92738 | 9.25412 | 9.01229 |
| | Sobic.008G022500 | 10.6411 | 11.31865 | 7.501 | 9.6157 | 10.5334 | 11.00077 | 8.98046 | 8.45836 |
| | Sobic.008G141800 | 11.1928 | 9.72734 | 9.74446 | 9.87667 | 10.6914 | 10.21845 | 9.31011 | 8.86735 |
| | Sobic.009G144200 | 9.91606 | 10.63455 | 11.4407 | 11.5165 | 11.7614 | 10.26449 | 10.9548 | 10.3483 |
| | Sobic.009G177200 | 10.3288 | 9.84059 | 7.09739 | 9.28455 | 8.93705 | 9.67388 | 7.18547 | 7.31921 |
| | Sobic.010G092400 | 11.129 | 10.80364 | 4.94371 | 9.26731 | 9.72344 | 10.74874 | 9.25121 | 8.61301 |
| | Sobic.010G101400 | 8.50798 | 10.03709 | 9.04323 | 8.40485 | 8.19096 | 9.92049 | 6.91998 | 6.67589 |
| | Sobic.010G274800 | 9.80288 | 9.94802 | 9.33081 | 9.56198 | 10.0293 | 9.96355 | 8.30376 | 8.3088 |
| **Glucan synthase-like (Callose synthase)** | Sobic.001G521500 | 9.79047 | 8.04077 | 11.9237 | 12.3478 | 13.6815 | 7.82144 | 10.2766 | 9.9094 |
| | Sobic.001G529600 | 13.0631 | 12.36719 | 12.3204 | 12.7681 | 12.5658 | 12.16836 | 10.9149 | 10.6957 |
| | Sobic.001G542450 | 13.1977 | 13.02818 | 12.6002 | 13.1534 | 13.0311 | 12.87895 | 11.5476 | 11.2761 |
| | Sobic.001G542500 | 12.8759 | 12.56056 | 11.379 | 12.6786 | 12.4282 | 12.29292 | 10.7121 | 10.5968 |
| | Sobic.003G179600 | 8.87651 | 10.51984 | 6.13987 | 9.27182 | 10.6714 | 9.90908 | 7.47341 | 7.98139 |
| | Sobic.003G180100 | 11.0315 | 11.50115 | 6.30364 | 10.9602 | 9.68322 | 11.56227 | 7.42409 | 7.50609 |
| | Sobic.003G252500 | 11.0639 | 10.71328 | 8.95229 | 10.0075 | 10.0604 | 10.21263 | 8.90277 | 8.4267 |
| | Sobic.003G298900 | 11.1114 | 11.04971 | 8.95437 | 11.0033 | 10.4957 | 10.96996 | 9.09694 | 8.58694 |
| | Sobic.004G107800 | 7.89394 | 9.27214 | 8.11067 | 9.55505 | 10.1753 | 8.44531 | 7.62773 | 8.70962 |
| | Sobic.004G358400 | 11.8834 | 12.03778 | 10.6863 | 12.3955 | 12.2139 | 11.92911 | 10.968 | 10.5527 |
| | Sobic.010G064200 | 8.40055 | 8.1697 | 5.61537 | 9.0677 | 8.59343 | 8.27731 | 10.1661 | 9.87943 |
| | Sobic.010G275800 | 14.0446 | 14.2681 | 8.15236 | 13.1627 | 11.7194 | 14.08089 | 10.1144 | 9.63199 |
| | Sobic.001G033300 | 10.1991 | 11.69422 | 5.95493 | 12.3371 | 10.5822 | 11.58049 | 10.812 | 8.89141 |
| | Sobic.001G155600 | 9.0909 | 9.82819 | 5.99305 | 6.89002 | 7.13465 | 9.51911 | 6.17973 | 5.52188 |
| | Sobic.001G155700 | 9.46132 | 11.0749 | -3.70572 | 6.66631 | 8.07609 | 10.81856 | 6.44039 | 5.91584 |
| | Sobic.001G237800 | -3.70572 | -3.70572 | 2.52999 | -3.70572 | -3.70572 | -3.70572 | -3.70572 | -3.70572 |
| | Sobic.001G237900 | -3.70572 | -3.70572 | 3.5232 | -3.70572 | -3.70572 | -3.70572 | -3.70572 | -3.70572 |

| | | | | | | | | |
|---|---|---|---|---|---|---|---|---|
| Sobic.001G238000 | -3.70572 | -3.70572 | -3.70572 | -3.70572 | -3.70572 | -3.70572 | -3.70572 | -1.23702 |
| Sobic.001G238100 | -3.70572 | -3.70572 | -3.70572 | -3.70572 | -3.70572 | -3.70572 | -3.70572 | -3.70572 |
| Sobic.001G238200 | -3.70572 | -3.70572 | -3.70572 | -3.70572 | -3.70572 | -3.70572 | -3.70572 | -3.70572 |
| Sobic.001G238300 | -3.70572 | -3.70572 | -3.70572 | -3.70572 | -3.70572 | -3.70572 | -3.70572 | -3.70572 |
| Sobic.001G238400 | -3.70572 | -3.70572 | 2.54574 | -3.70572 | -3.70572 | -3.70572 | 0.25437 | -1.23702 |
| Sobic.001G300400 | 12.2096 | 13.17166 | 8.04221 | 12.7483 | 11.4514 | 12.69886 | 12.3054 | 12.297 |
| Sobic.001G300500 | 5.81653 | 4.89557 | 8.23024 | 10.9982 | 7.97461 | 4.02492 | 6.90102 | 5.84278 |
| Sobic.001G300700 | -3.70572 | 2.60349 | 7.72536 | 4.71084 | 10.3726 | 2.46729 | 4.93225 | 3.30283 |
| Sobic.001G300800 | 5.90915 | 6.28426 | 12.505 | 12.3659 | 11.1955 | 4.14076 | 10.7154 | 9.42469 |
| Sobic.001G300900 | 8.86111 | 11.13815 | 10.7925 | 9.70098 | 10.0524 | 11.13736 | 8.24339 | 8.34825 |
| Sobic.001G301000 | 4.21736 | 1.4489 | 2.42068 | 6.30009 | 5.27617 | 1.41424 | 3.83317 | 2.5456 |
| Sobic.001G301300 | 3.27178 | 2.50654 | 4.18675 | 5.68651 | 9.92698 | 2.46666 | 9.8863 | 10.5097 |
| Sobic.001G301400 | -3.70572 | -3.70572 | -3.70572 | -3.70572 | -3.70572 | -3.70572 | 1.70406 | -1.37246 |
| Sobic.001G301500 | 9.29687 | 5.87698 | 11.7447 | 14.2614 | 11.1282 | 5.20954 | 9.11928 | 9.52092 |
| Sobic.001G301600 | 5.97494 | -3.70572 | 11.0223 | 14.3398 | 11.2842 | 4.94626 | 10.2903 | 10.7096 |
| Sobic.001G306200 | -3.70572 | 0.50151 | -3.70572 | 5.28635 | 3.19003 | 2.88233 | 13.7577 | 14.1689 |
| Sobic.001G306400 | -3.70572 | 0.50151 | -3.70572 | 4.42353 | 4.68508 | 1.88233 | 14.1384 | 14.5232 |
| Sobic.001G306500 | -3.70572 | -3.70572 | 2.48219 | 6.0871 | 4.12796 | 3.46729 | 14.7969 | 15.2489 |
| Sobic.001G311000 | 9.33619 | 5.58748 | 12.0277 | 7.54928 | 8.15437 | 6.13821 | 8.91594 | 6.80668 |
| Sobic.001G314600 | -3.70572 | -3.70572 | -3.70572 | -3.70572 | 1.29398 | -3.70572 | -3.70572 | -3.70572 |
| Sobic.001G356400 | -3.70572 | 1.52879 | 6.08596 | -3.70572 | 2.31041 | 3.07953 | 1.20963 | -1.28176 |
| Sobic.001G499701 | -3.70572 | -3.70572 | -3.70572 | -3.70572 | -3.70572 | -3.70572 | 0.27876 | -3.70572 |
| Sobic.001G499800 | 5.61961 | 5.52306 | 2.501 | 4.05848 | 2.63234 | 5.58203 | 3.63215 | 1.88817 |
| Sobic.001G499900 | -3.70572 | -3.70572 | 7.62753 | -3.70572 | 0.34948 | -3.70572 | -3.70572 | -3.70572 |
| Sobic.001G516500 | 4.83392 | 8.09477 | 10.8755 | 10.1768 | 10.4213 | 8.52265 | 11.1402 | 9.66952 |
| Sobic.001G539600 | -3.70572 | -3.70572 | -3.70572 | 6.40078 | 5.36879 | 3.10732 | 14.9106 | 14.9924 |
| Sobic.001G539700 | -3.70572 | -3.70572 | -3.70572 | 5.9683 | 5.44862 | 3.78922 | 15.2129 | 15.4151 |
| Sobic.001G539760 | -3.70572 | -3.70572 | 2.85191 | -3.70572 | -3.70572 | -3.70572 | 9.2483 | 7.26886 |
| Sobic.001G539820 | -3.70572 | -3.70572 | -3.70572 | 5.43235 | -3.70572 | 1.46729 | 12.7923 | 12.4195 |
| Sobic.001G539880 | -3.70572 | -3.70572 | -3.70572 | -3.70572 | 3.76181 | 2.05225 | 13.733 | 14.2885 |
| Sobic.001G539940 | -3.70572 | -3.70572 | 4.79565 | 6.46142 | 5.86216 | 3.46729 | 14.8149 | 14.983 |
| Sobic.001G540000 | -3.70572 | -3.70572 | -3.70572 | 6.31804 | 5.34812 | 0.46729 | 14.9681 | 15.065 |
| Sobic.001G542100 | 8.58963 | 11.67623 | 3.79296 | 12.7819 | 9.04807 | 12.09865 | 7.51654 | 6.39678 |
| Sobic.001G542200 | 10.6894 | 8.4305 | 3.31525 | 9.04838 | 6.93202 | 7.30882 | 8.38144 | 8.48675 |

| | | | | | | | | | |
|---|---|---|---|---|---|---|---|---|---|
| **Expansins** | Sobic.002G124400 | -3.70572 | -3.70572 | 7.07499 | 1.01777 | 8.11047 | -3.70572 | 5.64814 | 3.77028 |
| | Sobic.002G124500 | -3.70572 | -3.70572 | 5.05176 | -3.70572 | 3.22374 | -3.70572 | -0.01939 | -3.70572 |
| | Sobic.002G245200 | 6.25073 | 3.89687 | 11.0931 | 3.61922 | 5.72289 | 5.67 | 6.35908 | 4.40826 |
| | Sobic.002G300000 | 9.45663 | 10.26949 | 5.23849 | 10.308 | 8.46454 | 9.69148 | 8.73222 | 7.92915 |
| | Sobic.002G305800 | -3.70572 | -3.70572 | 9.31203 | 5.83565 | 0.25107 | 1.43523 | 11.9255 | 11.8776 |
| | Sobic.002G309300 | 6.68085 | 8.80812 | 7.96217 | 8.07908 | 8.07824 | 9.19966 | 6.12331 | 6.29781 |
| | Sobic.003G059900 | -3.70572 | -3.70572 | 1.54574 | 1.88177 | 2.35609 | 1.5393 | 5.77425 | 4.21077 |
| | Sobic.003G112100 | -3.70572 | 1.56786 | 1.54007 | 1.87422 | 1.34759 | -3.70572 | 4.64904 | 3.03185 |
| | Sobic.003G128800 | 7.0949 | 9.31906 | 8.09105 | 6.77418 | 6.33793 | 9.7861 | 5.00685 | 5.52927 |
| | Sobic.003G338801 | 4.93307 | 9.59849 | 12.7451 | 12.7219 | 10.5806 | 6.95391 | 9.74087 | 9.35282 |
| | Sobic.003G444400 | -3.70572 | 3.00251 | 7.19712 | 3.94725 | 5.84303 | 4.38333 | 5.3838 | 3.5139 |
| | Sobic.004G119600 | -3.70572 | 1.63968 | 6.85604 | 2.27332 | -3.70572 | 1.51676 | 2.33242 | 4.09799 |
| | Sobic.004G119800 | -3.70572 | -3.70572 | -3.70572 | -3.70572 | -3.70572 | -3.70572 | 0.1609 | -3.70572 |
| | Sobic.004G119900 | -3.70572 | -3.70572 | 4.862 | 2.2902 | 1.34948 | 1.53363 | 0.2487 | -3.70572 |
| | Sobic.004G120000 | -3.70572 | -3.70572 | 2.58606 | -3.70572 | -3.70572 | -3.70572 | -3.70572 | -3.70572 |
| | Sobic.004G121500 | -3.70572 | -3.70572 | -3.70572 | -3.70572 | -3.70572 | -3.70572 | -3.70572 | -3.70572 |
| | Sobic.004G121600 | -3.70572 | -3.70572 | -3.70572 | -3.70572 | -3.70572 | -3.70572 | -3.70572 | -3.70572 |
| | Sobic.004G121700 | -3.70572 | -3.70572 | -3.70572 | -3.70572 | 0.37777 | -3.70572 | -3.70572 | -3.70572 |
| | Sobic.004G121800 | -3.70572 | -3.70572 | -3.70572 | -3.70572 | 1.32146 | -3.70572 | -3.70572 | -3.70572 |
| | Sobic.004G121900 | -3.70572 | 1.5454 | 5.51761 | 1.26774 | 6.76957 | -3.70572 | 2.22624 | 1.54221 |
| | Sobic.004G191600 | 10.4894 | 12.01709 | 8.42364 | 9.38861 | 10.3814 | 12.11294 | 8.85497 | 8.46128 |
| | Sobic.004G227900 | 6.93351 | 7.71211 | 4.75832 | 6.47192 | 9.22879 | 6.82227 | 7.87294 | 7.15348 |
| | Sobic.004G238801 | 9.86427 | 12.36878 | 10.4812 | 9.12322 | 7.1603 | 12.3339 | 8.65192 | 6.27115 |
| | Sobic.004G294300 | 4.12165 | 1.35276 | 7.28363 | 12.0512 | 10.1064 | 2.31853 | 7.84196 | 8.0381 |
| | Sobic.004G294400 | -3.70572 | -3.70572 | 5.63786 | 7.28254 | 8.48665 | -3.70572 | 2.31472 | 2.79368 |
| | Sobic.004G294500 | 8.63881 | -3.70572 | 2.48457 | 12.3895 | 1.87894 | -3.70572 | 2.1932 | 0.70182 |
| | Sobic.006G031900 | -3.70572 | -3.70572 | -3.70572 | 5.77092 | 6.49368 | -3.70572 | 12.0786 | 13.3888 |
| | Sobic.006G171100 | -3.70572 | -3.70572 | -3.70572 | -3.70572 | -3.70572 | -3.70572 | 0.06813 | -1.42326 |
| | Sobic.006G171200 | -3.70572 | -3.70572 | -3.70572 | 8.28455 | 0.29944 | -3.70572 | 7.39833 | 5.48863 |
| | Sobic.006G171300 | -3.70572 | -3.70572 | -3.70572 | -3.70572 | 0.15401 | -3.70572 | 1.05323 | 0.56184 |
| | Sobic.006G191700 | 9.77931 | 10.72341 | 7.5232 | 10.6975 | 11.1351 | 10.80908 | 9.25142 | 9.16355 |
| | Sobic.006G209100 | -3.70572 | 4.42217 | 5.31019 | -3.70572 | 5.5418 | -3.70572 | 4.28925 | 4.28188 |
| | Sobic.007G018000 | 3.27041 | -3.70572 | 3.47373 | 9.3376 | 0.28313 | -3.70572 | 5.46776 | 5.06601 |
| | Sobic.007G019900 | -3.70572 | -3.70572 | 0.82534 | -3.70572 | -3.70572 | -3.70572 | -3.70572 | -0.63549 |

| | | | | | | | | | |
|---|---|---|---|---|---|---|---|---|---|
| | Sobic.007G020100 | -3.70572 | -3.70572 | 0.92155 | -3.70572 | -3.70572 | -3.70572 | 3.40742 | -0.16076 |
| | Sobic.007G020200 | -3.70572 | -3.70572 | 0.92709 | -3.70572 | -3.70572 | -3.70572 | 1.73644 | -1.04831 |
| | Sobic.007G166500 | -3.70572 | -3.70572 | 11.8018 | 4.8817 | 2.24054 | -3.70572 | 11.9285 | 11.7431 |
| | Sobic.009G173700 | 5.3711 | 5.8406 | 10.2919 | 11.2363 | 9.17923 | 4.82603 | 11.271 | 11.5797 |
| | Sobic.010G006200 | -3.70572 | -3.70572 | 7.54996 | -3.70572 | -3.70572 | -3.70572 | -3.70572 | -3.70572 |
| | Sobic.010G120100 | -3.70572 | -3.70572 | -3.70572 | -3.70572 | -3.70572 | -3.70572 | 1.04171 | 1.42647 |
| | Sobic.010G121800 | -3.70572 | -3.70572 | -3.70572 | 4.06954 | 0.32146 | -3.70572 | 9.26572 | 10.1527 |
| | Sobic.010G194500 | 5.61961 | 9.25671 | 6.20144 | 4.42105 | 4.95426 | 9.07953 | 4.45756 | 2.30321 |
| | Sobic.010G268700 | -3.70572 | -3.70572 | 5.6222 | 2.78737 | 1.26168 | 1.44584 | -3.70572 | -0.33048 |
| | Sobic.010G271000 | 8.02854 | 9.10318 | 7.95123 | 7.90799 | 7.97568 | 9.21739 | 5.57792 | 5.40846 |
| **Yieldins** | Sobic.001G543000 | -3.70572 | 1.31428 | 2.28649 | 1.03662 | 1.68087 | 3.60199 | -0.00488 | -3.70572 |
| | Sobic.002G055600 | 3.06432 | 1.29542 | 12.4969 | 8.13671 | 10.8559 | -3.70572 | 9.05841 | 6.84243 |
| | Sobic.002G055700 | 7.23424 | -3.70572 | 13.5039 | 9.78925 | 10.9847 | 5.72063 | 10.5669 | 8.6784 |
| | Sobic.002G109700 | 2.11193 | 2.34304 | 0.73029 | 0.06538 | 0.99913 | 3.30882 | 1.60885 | 2.33985 |
| | Sobic.002G109800 | 2.11193 | 2.34304 | 0.73029 | 0.06538 | 0.99913 | -3.70572 | -3.70572 | -3.70572 |
| | Sobic.002G109900 | -3.70572 | -3.70572 | 0.73029 | -3.70572 | -1.4603 | -3.70572 | -3.70572 | -3.70572 |
| | Sobic.002G110000 | -3.70572 | -3.70572 | 2.54595 | -3.70572 | 0.35515 | -3.70572 | -3.70572 | -3.70572 |
| | Sobic.003G244600 | 5.73332 | 6.36652 | 15.2616 | 12.4464 | 10.142 | 7.29277 | 11.4894 | 9.77698 |
| | Sobic.003G263600 | 3.79194 | -3.70572 | 11.421 | 5.26895 | -0.19533 | 0.98883 | 5.93271 | 4.42196 |
| | Sobic.003G363900 | -3.70572 | -3.70572 | 5.30087 | -3.70572 | -3.70572 | -3.70572 | -3.70572 | -3.70572 |
| | Sobic.003G364000 | -3.70572 | -3.70572 | -3.70572 | 0.05573 | 1.11501 | -3.70572 | -3.70572 | -3.70572 |
| | Sobic.003G364100 | -3.70572 | -3.70572 | -3.70572 | 0.05573 | 1.11501 | -3.70572 | -3.70572 | -1.47715 |
| | Sobic.003G364200 | 3.11193 | -3.70572 | 2.31525 | 4.23531 | 6.42844 | -3.70572 | 1.60885 | 1.70242 |
| | Sobic.003G364300 | 6.08317 | 6.89925 | 9.83397 | 5.79151 | 9.08459 | 6.89477 | 6.31705 | 4.33663 |
| | Sobic.005G098700 | 10.0378 | 4.87763 | 4.44925 | 11.8582 | 4.19019 | 3.40053 | 4.91792 | 3.792 |
| | Sobic.005G099000 | 9.42891 | 8.97306 | 5.99695 | 10.4382 | 8.71826 | 8.289 | 7.26017 | 5.52274 |
| | Sobic.005G110600 | 6.33433 | 8.9479 | -3.70572 | 4.18086 | 9.65084 | 8.2861 | 6.38144 | 5.88417 |
| | Sobic.005G177100 | 4.11678 | -3.70572 | 4.84141 | 2.07023 | -3.70572 | 1.31367 | 2.6137 | -1.46265 |
| | Sobic.005G177400 | -3.70572 | -3.70572 | 8.2882 | 3.40681 | -3.70572 | -3.70572 | 3.04338 | 1.72192 |
| | Sobic.005G177500 | -3.70572 | -3.70572 | 5.8526 | -3.70572 | -3.70572 | -3.70572 | -3.70572 | -3.70572 |
| | Sobic.005G177600 | -3.70572 | -3.70572 | 8.10979 | 5.68523 | 3.56085 | -3.70572 | 4.75477 | 3.20893 |
| | Sobic.005G224900 | -3.70572 | -3.70572 | 8.01685 | 3.86791 | 3.92718 | -3.70572 | 3.01905 | 0.52766 |
| | Sobic.006G044200 | 6.0457 | 7.08416 | 4.83398 | 3.22154 | 8.15646 | 6.35806 | 3.37269 | 2.63619 |
| | Sobic.007G006501 | -3.70572 | 1.26759 | 6.41751 | 3.15986 | 5.19896 | 0.23336 | 2.40786 | 2.2644 |

| | | | | | | | | | |
|---|---|---|---|---|---|---|---|---|---|
| **Xyloglucan endotransglucosylases /hydrolases** | Sobic.001G006900 | 3.17628 | 5.99235 | 7.29766 | 4.7147 | 5.77397 | 6.37317 | 5.41016 | 4.76677 |
| | Sobic.001G179400 | 7.22967 | 12.00252 | 4.01381 | 6.70368 | 9.803 | 11.54732 | 6.4059 | 4.52311 |
| | Sobic.001G284600 | -3.70572 | 1.10296 | 10.5258 | 6.49755 | 6.91782 | 1.06855 | 8.27948 | 7.61068 |
| | Sobic.001G309000 | -3.70572 | 2.28629 | 5.44843 | 13.2113 | 7.01443 | 2.83682 | 4.07955 | 3.75839 |
| | Sobic.001G441700 | 7.44213 | 10.18808 | 3.47553 | 7.40888 | 9.21567 | 10.01093 | 9.71424 | 7.51932 |
| | Sobic.001G531300 | 7.28125 | 8.91324 | 9.1147 | 6.04206 | 7.74519 | 8.65805 | 6.34295 | 6.0147 |
| | Sobic.001G538000 | -3.70572 | -3.70572 | 5.83961 | 8.61171 | 10.4554 | -3.70572 | 8.94165 | 7.19 |
| | Sobic.002G194500 | 10.6463 | 14.05753 | 6.13138 | 8.63965 | 9.74093 | 14.65042 | 8.91218 | 8.14877 |
| | Sobic.002G302000 | 6.35382 | 8.7708 | 9.86243 | 11.9723 | 10.4875 | 8.17129 | 7.56796 | 7.09631 |
| | Sobic.002G324100 | 6.46861 | 9.32774 | 10.7823 | 10.3973 | 8.29803 | 9.28287 | 6.38056 | 5.18463 |
| | Sobic.004G025900 | 4.79194 | 1.02322 | 9.1219 | 5.55275 | 5.84907 | 1.98883 | 10.2077 | 10.8707 |
| | Sobic.004G126700 | 6.29157 | 8.82849 | 3.91012 | 4.39703 | 8.15674 | 7.58532 | 2.0336 | -0.45779 |
| | Sobic.004G127200 | -3.70572 | -3.70572 | 6.75462 | 1.09968 | -3.70572 | -3.70572 | -3.70572 | -3.70572 |
| | Sobic.004G273200 | 10.6919 | 11.37323 | 11.0284 | 10.7154 | 11.3086 | 11.88234 | 9.56826 | 8.38102 |
| | Sobic.005G140001 | 6.99006 | 12.66412 | 7.04634 | 7.10271 | 10.5938 | 12.35487 | 9.9539 | 8.95082 |
| | Sobic.006G205500 | 7.17064 | 1.31428 | 3.28649 | 6.70905 | -3.70572 | -3.70572 | -3.70572 | -3.70572 |
| | Sobic.006G205600 | 10.3562 | 6.36159 | 7.65695 | 7.63753 | 9.0959 | 9.6996 | 8.79214 | 7.60247 |
| | Sobic.006G205700 | -3.70572 | 1.33821 | 3.89538 | 1.06055 | -3.70572 | -3.70572 | -3.70572 | -1.47234 |
| | Sobic.006G228100 | 10.6631 | 14.35302 | 3.99094 | 7.79397 | 11.4794 | 14.25533 | 9.55004 | 8.08256 |
| | Sobic.007G085500 | -3.70572 | 2.36254 | -3.70572 | -3.70572 | 3.31408 | -3.70572 | -3.70572 | -1.44801 |
| | Sobic.007G085600 | -3.70572 | -3.70572 | 7.65492 | 6.87547 | 4.70249 | -3.70572 | 7.6706 | 8.21794 |
| | Sobic.007G086300 | 7.3311 | -3.70572 | 2.3645 | 6.28455 | 0.17391 | -3.70572 | 0.07313 | -3.70572 |
| | Sobic.007G086400 | -3.70572 | -3.70572 | -3.70572 | -3.70572 | -3.70572 | -3.70572 | -3.70572 | -3.70572 |
| | Sobic.007G090436.1 | 3.60913 | 5.58599 | 11.9607 | 9.62237 | 12.1536 | 2.39489 | 10.5216 | 9.82099 |
| | Sobic.007G090460 | 4.63064 | -3.70572 | 7.10784 | 8.98948 | 7.79166 | -3.70572 | 5.55752 | 6.62004 |
| | Sobic.007G090463.1 | 12.178 | 11.51248 | 12.3641 | 11.8832 | 13.0518 | 12.48475 | 11.0017 | 9.89788 |
| | Sobic.007G094900 | 6.72624 | 6.62718 | 7.35695 | 9.69045 | 9.59274 | 6.50872 | 7.84374 | 7.44398 |
| | Sobic.010G098000 | -3.70572 | -3.70572 | 5.67148 | -3.70572 | 0.15896 | -3.70572 | 2.64314 | 2.65425 |
| | Sobic.010G098100 | -3.70572 | -3.70572 | 3.91971 | 6.72874 | 3.46609 | -3.70572 | 8.42708 | 6.30019 |
| | Sobic.010G146300 | -3.70572 | -3.70572 | 3.32985 | -3.70572 | -3.70572 | -3.70572 | 3.79337 | 4.21952 |
| | Sobic.010G246400 | 13.2678 | 13.13222 | 7.01689 | 11.1167 | 13.9336 | 14.56262 | 11.3048 | 10.6103 |
| | Sobic.010G246500 | 9.07172 | 9.40309 | 7.13753 | 6.88766 | 8.07347 | 9.11902 | 8.3946 | 7.08813 |
| | Sobic.010G246600 | -3.70572 | 2.38946 | 15.2034 | 7.73898 | 9.92546 | 2.35337 | 10.6764 | 9.70818 |
| | Sobic.010G246700 | 11.3038 | 13.97424 | 11.604 | 11.3576 | 13.7037 | 14.85946 | 12.485 | 12.0104 |

| | | | | | | | | | |
|---|---|---|---|---|---|---|---|---|---|
| | Sobic.K044406.1 | -3.70572 | -3.70572 | -3.70572 | -3.70572 | -3.70572 | -3.70572 | 4.64921 | -3.70572 |
| **Endo-1,4-β-glucanases** | Sobic.001G099100 | 12.8911 | 10.67804 | 11.2281 | 13.6636 | 13.8563 | 10.30521 | 12.8036 | 12.4515 |
| | Sobic.001G384600 | 11.895 | 12.67312 | 7.38526 | 11.5779 | 9.99094 | 12.78043 | 9.16673 | 8.53459 |
| | Sobic.002G193600 | 8.07918 | 6.70622 | 8.58479 | 10.0054 | 8.88564 | 6.65263 | 7.69319 | 6.54644 |
| | Sobic.002G273500 | 11.0753 | 8.29091 | 3.99404 | 8.09191 | 7.20323 | 7.49728 | 9.22099 | 6.63081 |
| | Sobic.002G276600 | 13.2148 | 12.71497 | 10.6274 | 12.1664 | 12.7896 | 12.74881 | 12.4292 | 11.994 |
| | Sobic.003G015700 | 6.06321 | -3.70572 | 5.94179 | 5.09582 | -3.70572 | 2.69887 | 3.32546 | 3.51923 |
| | Sobic.003G015800 | -3.70572 | 5.33638 | 6.8208 | 4.01026 | 2.57565 | 6.53499 | 3.26797 | 1.16085 |
| | Sobic.003G148500 | 7.26645 | 12.59222 | 12.5741 | 7.32424 | 5.62021 | 12.63794 | 5.87142 | 3.10205 |
| | Sobic.004G021400 | 3.32267 | 3.13909 | 7.28862 | 4.36525 | 1.3354 | 4.10452 | 1.23462 | 0.06517 |
| | Sobic.004G042700 | 3.26501 | 3.304 | 8.00692 | 5.17424 | 7.40321 | 2.04686 | 8.02002 | 6.778 |
| | Sobic.004G244600 | 5.34811 | 1.57922 | 9.22028 | 7.92503 | 8.92066 | 3.44597 | 9.40787 | 8.70744 |
| | Sobic.004G248100 | 8.58026 | 12.72427 | 7.16682 | 4.94827 | 7.65423 | 12.40722 | 6.1583 | 4.11138 |
| | Sobic.004G318300 | -3.70572 | -3.70572 | 6.66188 | 6.60995 | 0.38383 | 0.56798 | 11.8453 | 11.9306 |
| | Sobic.006G090900 | -3.70572 | -3.70572 | 6.98263 | 4.52125 | 2.50253 | -3.70572 | 0.23182 | 0.91036 |
| | Sobic.006G134700 | -3.70572 | 2.26071 | 6.67804 | 5.00198 | -0.94774 | 1.22649 | 6.58631 | 6.13695 |
| | Sobic.006G265100 | 2.02961 | -3.70572 | 5.70069 | 2.30498 | 3.90032 | 0.22649 | 5.20834 | 4.58972 |
| | Sobic.006G282300 | 6.13316 | 5.0317 | 9.55934 | 9.83295 | 10.6111 | 5.45204 | 8.83857 | 8.70641 |
| | Sobic.007G017300 | 6.05713 | 11.91141 | 10.9552 | 4.3976 | 5.76173 | 11.81743 | 3.59057 | 2.58461 |
| | Sobic.007G119300 | -3.70572 | -3.70572 | -3.70572 | 9.89345 | 1.64895 | -3.70572 | 11.4348 | 11.1823 |
| | Sobic.007G131300 | -3.70572 | -3.70572 | 9.81972 | -3.70572 | -3.70572 | -3.70572 | 5.47545 | 3.99708 |
| | Sobic.009G029800 | -3.70572 | -3.70572 | 4.28291 | 0.67722 | -3.70572 | -3.70572 | 5.86454 | 5.89253 |
| | Sobic.009G079900 | 5.49591 | 7.82886 | 5.17631 | 3.89682 | 4.74965 | 6.79991 | 2.40786 | 0.62697 |
| | Sobic.010G101900 | 2.5432 | 3.77431 | 9.93752 | 7.59688 | 9.06796 | 5.42916 | 8.25185 | 7.48863 |
| | Sobic.010G106100 | 9.52481 | 13.15193 | 8.53698 | 11.6632 | 9.457 | 12.74644 | 10.4897 | 9.7736 |
| | Sobic.010G266100 | 3.30043 | 3.70147 | 7.31111 | 5.25388 | 4.92787 | 4.49732 | 10.1155 | 10.3848 |
| | Sobic.K044400 | -3.70572 | -3.70572 | -3.70572 | -3.70572 | -3.70572 | -3.70572 | -3.70572 | -3.70572 |
| **Endo-xylanases** | Sobic.001G140300 | -3.70572 | 1.59835 | 7.62946 | -3.70572 | 6.3028 | 0.97916 | 4.21779 | 4.36272 |
| | Sobic.001G439400 | 11.9608 | 12.33461 | 1.40257 | 10.2468 | 8.09462 | 12.38019 | 7.5788 | 7.27802 |
| | Sobic.001G466000 | 2.13881 | -3.70572 | 4.80156 | 4.61582 | 5.38036 | -3.70572 | 7.35914 | 6.30757 |
| | Sobic.001G466100 | -3.70572 | -3.70572 | 1.37203 | 9.62 | 5.48522 | 0.36559 | 14.1364 | 14.922 |
| | Sobic.001G466400 | -3.70572 | 0.38728 | 2.3595 | 1.11556 | -3.70572 | 1.35306 | 2.97893 | 1.83095 |
| | Sobic.002G053800 | -3.70572 | -3.70572 | 2.88337 | 1.6335 | 2.42974 | 0.29197 | 12.8392 | 12.663 |
| | Sobic.002G127400 | 11.5522 | 11.14936 | 9.22686 | 10.9479 | 10.7866 | 11.19088 | 9.21589 | 9.14654 |

| | | | | | | | | | |
|---|---|---|---|---|---|---|---|---|---|
| | Sobic.003G082700 | -3.70572 | 5.3743 | 5.88233 | 3.31237 | 7.99938 | 2.97084 | 8.53766 | 8.48531 |
| | Sobic.003G083000 | -3.70572 | 4.0459 | 8.62146 | 7.99854 | 10.2692 | -3.70572 | 9.77785 | 8.73582 |
| | Sobic.004G154000 | -3.70572 | -3.70572 | 6.04458 | 2.2823 | 6.47086 | -3.70572 | 6.14769 | 2.01245 |
| | Sobic.006G032800 | 7.77839 | 0.39479 | 2.95197 | 5.81757 | 8.31084 | 1.94553 | 6.21519 | 6.75917 |
| **Glucan 1,3-β-glucosidases** | Sobic.001G014700 | -3.70572 | -3.70572 | 4.42291 | 0.36569 | -3.70572 | -3.70572 | -3.70572 | -3.70572 |
| | Sobic.001G061900 | 9.79476 | 9.62203 | 7.62462 | 9.01686 | 8.9914 | 10.9203 | 8.61529 | 7.81149 |
| | Sobic.001G109400 | 8.41284 | 10.10561 | 9.17647 | 7.43785 | 9.37582 | 10.02133 | 8.65647 | 7.76615 |
| | Sobic.001G142200 | 7.34317 | 10.20267 | 9.29389 | 8.86951 | 10.6202 | 9.89995 | 9.79014 | 8.93366 |
| | Sobic.001G148300 | -3.70572 | 0.61384 | 10.1226 | 8.44993 | 7.70704 | 0.57962 | 7.74809 | 7.03161 |
| | Sobic.001G263500 | 4.56623 | 9.92404 | 4.93948 | 5.37766 | 7.90089 | 9.676 | 6.73557 | 5.62342 |
| | Sobic.001G346200 | 4.94165 | 4.75772 | 7.6261 | 6.3976 | 6.75312 | 3.13853 | 5.88335 | 5.33184 |
| | Sobic.001G357900 | 3.50455 | 1.73566 | 8.92704 | 7.57694 | 8.94564 | 1.70144 | 7.57637 | 7.24253 |
| | Sobic.001G404000 | 9.47718 | 10.1745 | 7.57573 | 8.42528 | 7.66576 | 9.82847 | 6.41035 | 5.93987 |
| | Sobic.001G437801 | 9.44384 | 10.30785 | 9.73462 | 9.72214 | 10.0496 | 10.16852 | 8.53758 | 8.68078 |
| | Sobic.001G445700 | 5.7428 | 6.4187 | 10.08 | 7.33368 | 7.34049 | 7.67863 | 6.39172 | 5.68693 |
| | Sobic.001G452900 | 10.0709 | 12.99586 | 4.36736 | 9.1335 | 9.63971 | 12.47909 | 8.02352 | 6.74304 |
| | Sobic.002G045800 | 4.71345 | 9.42676 | 9.99351 | 6.61176 | 8.60392 | 8.80273 | 7.89966 | 7.02397 |
| | Sobic.002G084700 | -3.70572 | -3.70572 | 3.54858 | 0.29871 | 4.81742 | 1.54214 | 9.53566 | 9.5772 |
| | Sobic.002G148900 | 10.8815 | 10.59976 | 10.0768 | 11.067 | 11.0463 | 10.47281 | 9.82996 | 9.76078 |
| | Sobic.002G255600 | 10.9436 | 11.66641 | 6.37045 | 9.40186 | 8.7514 | 11.53022 | 9.43663 | 8.99834 |
| | Sobic.002G275800 | 5.89231 | 9.0892 | 5.55506 | 5.84576 | 5.49 | 8.59699 | 10.175 | 7.09423 |
| | Sobic.002G314500 | 7.00384 | 9.48288 | 8.31025 | 7.00976 | 8.51671 | 9.15696 | 7.34578 | 6.7248 |
| | Sobic.002G327900 | 3.18135 | 0.42905 | 14.9327 | 1.82757 | 8.44916 | 0.97837 | 5.56765 | 4.79757 |
| | Sobic.002G328200 | 5.53236 | 10.32483 | 7.45807 | 5.01632 | 7.76056 | 10.24054 | 7.47204 | 6.95748 |
| | Sobic.002G328300 | 2.14623 | 0.37733 | 11.0953 | 9.37991 | 6.71038 | 0.92242 | 7.25045 | 5.22204 |
| | Sobic.002G328600 | 3.54538 | 2.51571 | 8.02802 | 2.23579 | 4.58785 | 1.74232 | 2.57322 | 3.28787 |
| | Sobic.002G351700 | 11.8207 | 11.68774 | 4.39049 | 10.3501 | 9.89438 | 11.37274 | 8.42876 | 8.35209 |
| | Sobic.003G290400 | 13.7974 | 14.44259 | 7.00069 | 11.579 | 10.4943 | 14.55258 | 9.25746 | 8.74441 |
| | Sobic.003G326700 | 2.82331 | -3.70572 | 7.80799 | 0.77676 | 6.46539 | 1.02019 | 6.09281 | 4.30996 |
| | Sobic.003G364600 | 6.24011 | 6.37811 | 13.2512 | 10.7368 | 12.683 | 7.20873 | 11.6844 | 10.852 |
| | Sobic.003G421500 | -3.70572 | 1.16561 | 8.25279 | 1.2073 | 1.66285 | 3.71634 | 3.71568 | 1.6944 |
| | Sobic.003G421700 | -3.70572 | -3.70572 | 16.9869 | 2.81292 | 6.2259 | -3.70572 | 4.2823 | 1.52261 |
| | Sobic.003G421900 | 4.45663 | -3.70572 | 6.8422 | 9.05394 | 8.29379 | 1.06855 | 5.73797 | 4.64979 |
| | Sobic.003G422000 | -3.70572 | -3.70572 | 11.365 | 5.97114 | 10.7931 | 2.12711 | 15.3206 | 13.2675 |

| | | | | | | | | | |
|---|---|---|---|---|---|---|---|---|---|
| | Sobic.003G422100 | -3.70572 | -3.70572 | 5.7016 | 2.8667 | -3.70572 | -3.70572 | -0.17477 | -3.70572 |
| | Sobic.003G422200 | -3.70572 | -3.70572 | 8.87664 | 5.28026 | 2.94723 | 2.71634 | 9.54341 | 8.51619 |
| | Sobic.003G423500 | -3.70572 | 3.83429 | 5.22154 | 4.87856 | 6.99673 | -3.70572 | 6.4538 | 4.27167 |
| | Sobic.004G036800 | 11.8102 | 11.90692 | 6.9901 | 12.0557 | 11.0384 | 11.42976 | 9.47132 | 8.80371 |
| | Sobic.004G083100 | 7.47141 | 5.48839 | 9.83911 | 6.85228 | 8.06078 | 3.13224 | 6.50266 | 5.50159 |
| | Sobic.004G165600 | 5.65162 | 5.69008 | 10.3899 | 3.41243 | 7.27168 | 6.05796 | 7.93426 | 6.13288 |
| | Sobic.004G313200 | 9.77229 | 13.76865 | 6.23794 | 10.2474 | 11.0689 | 14.07858 | 10.2634 | 9.25882 |
| | Sobic.005G164000 | -3.70572 | -3.70572 | 4.92289 | 0.32455 | 6.39827 | 1.56798 | 6.58195 | 5.14227 |
| | Sobic.005G228900 | 10.3796 | 11.06253 | 11.5722 | 11.039 | 10.6938 | 10.99747 | 9.73573 | 9.40143 |
| | Sobic.006G069200 | 8.13996 | 5.33865 | 6.66965 | 6.35644 | 8.26817 | 7.88939 | 9.16183 | 7.38155 |
| | Sobic.006G271100 | 2.47001 | 7.16055 | 3.99526 | 2.42346 | 6.8403 | 4.98883 | 4.80823 | 4.01986 |
| | Sobic.007G078800 | -3.70572 | 7.4311 | 9.78281 | 7.69959 | 7.10679 | 5.66416 | 6.85401 | 6.00495 |
| | Sobic.007G197000 | 3.99422 | 2.22533 | 3.93451 | 3.36271 | 1.74392 | 2.19111 | 6.95784 | 6.15625 |
| | Sobic.008G146700 | -3.70572 | -3.70572 | 13.1648 | 1.91386 | 6.03923 | -3.70572 | 2.67988 | 2.70291 |
| | Sobic.009G119200 | -3.70572 | -3.70572 | 16.0874 | 6.53609 | 12.213 | 1.13567 | 11.1258 | 8.03531 |
| | Sobic.009G119400 | -3.70572 | -3.70572 | 9.59459 | 7.0735 | 8.70782 | 1.12711 | 12.9887 | 12.9322 |
| | Sobic.009G154600 | -3.70572 | -3.70572 | 13.3322 | 8.90019 | 11.3836 | 1.87959 | 8.34188 | 8.13723 |
| | Sobic.009G183400 | -3.70572 | 2.71672 | 9.04666 | 8.87091 | 9.59286 | 2.09753 | 8.08872 | 7.49364 |
| | Sobic.009G201400 | -3.70572 | -3.70572 | 5.68893 | -3.70572 | -3.70572 | -3.70572 | -3.70572 | -3.70572 |
| | Sobic.009G210900 | 6.79564 | 4.54132 | 5.30708 | 5.35677 | 4.70145 | 5.30065 | 2.4852 | 2.63124 |
| | Sobic.010G022500 | 4.47001 | 5.83041 | 6.31719 | 3.23082 | 5.65267 | 5.62109 | 5.13685 | 4.69793 |
| | Sobic.010G155500 | -3.70572 | 5.88625 | 6.81266 | 3.93052 | 6.02543 | 3.43699 | 5.05895 | 4.43326 |
| | Sobic.010G177600 | 4.70759 | -3.70572 | 1.58898 | 8.12701 | 3.64631 | 1.58254 | 11.092 | 12.2137 |
| | Sobic.010G186700 | 11.3052 | 11.19885 | 10.0246 | 10.8865 | 11.5939 | 11.08702 | 10.1677 | 9.54002 |
| | Sobic.001G026800 | 11.912 | 12.69115 | 6.28346 | 11.0657 | 11.6453 | 12.36449 | 10.0038 | 9.62916 |
| | Sobic.001G045400 | 3.46074 | 1.69185 | 1.66406 | 3.22154 | 2.47347 | 2.24258 | 10.1736 | 10.0282 |
| | Sobic.001G045800 | -3.70572 | -3.70572 | -3.70572 | 2.04615 | 5.6076 | 0.70462 | 6.95107 | 3.85904 |
| | Sobic.001G045900 | -3.70572 | -3.70572 | 2.76293 | 0.51306 | -0.40584 | -3.70572 | -3.70572 | -3.70572 |
| | Sobic.001G454800 | -3.70572 | -3.70572 | 1.78288 | 2.11797 | 1.59229 | -3.70572 | 2.95094 | 1.32205 |
| | Sobic.001G525000 | 10.4098 | 10.72382 | 7.2 | 12.7664 | 10.3243 | 10.45464 | 11.4725 | 11.2427 |
| | Sobic.002G090400 | 10.7916 | 11.44583 | 10.6347 | 10.8076 | 11.5869 | 11.37518 | 9.6414 | 9.63646 |
| | Sobic.002G214900 | 10.7336 | 9.39749 | 9.42638 | 9.47685 | 10.3727 | 9.44797 | 8.83449 | 8.31139 |
| | Sobic.002G246400 | 8.88562 | 9.75991 | 11.8432 | 10.8706 | 10.8244 | 9.86541 | 10.3026 | 9.57672 |
| | Sobic.003G050100 | 5.40714 | 5.47475 | 8.58446 | 7.07679 | 8.98972 | 5.38163 | 6.87806 | 6.6128 |

| | | | | | | | | | |
|---|---|---|---|---|---|---|---|---|---|
| Polygalacturonases | Sobic.003G141800 | 2.34526 | 7.20572 | 8.89397 | 6.20627 | 7.69338 | 5.82755 | 7.96457 | 7.18578 |
| | Sobic.003G153100 | 2.00221 | 4.98824 | 7.19491 | 5.44096 | 4.04772 | 5.82596 | 3.20591 | 0.07856 |
| | Sobic.003G153200 | 7.69561 | 8.95029 | 7.2334 | 9.13509 | 7.36704 | 9.52452 | 7.03102 | 5.43533 |
| | Sobic.003G173800 | -3.70572 | 0.86872 | -3.70572 | 6.2915 | 2.65034 | 1.8345 | 13.1532 | 13.2783 |
| | Sobic.003G187700 | -3.70572 | -3.70572 | 2.77182 | 0.51306 | -3.70572 | -3.70572 | 6.67123 | 6.56138 |
| | Sobic.003G223100 | 11.3598 | 12.10414 | 9.96547 | 10.9336 | 10.1704 | 11.96559 | 10.3161 | 10.0502 |
| | Sobic.003G226000 | -3.70572 | -3.70572 | -3.70572 | 11.3284 | -3.70572 | 0.86621 | -0.41116 | -1.91011 |
| | Sobic.003G232600 | 11.8841 | 9.11893 | 7.98423 | 9.51888 | 10.5062 | 9.0847 | 6.64449 | 6.90799 |
| | Sobic.003G384300 | 11.0496 | 10.39064 | 10.1425 | 11.4933 | 10.5142 | 10.19095 | 9.51154 | 9.36655 |
| | Sobic.004G028700 | 4.30596 | 4.12203 | 10.1286 | 8.64743 | 6.18344 | 2.08781 | 8.12179 | 6.30781 |
| | Sobic.004G080000 | -3.70572 | 2.60028 | 9.12708 | 9.51244 | 5.25637 | 4.44052 | 13.9974 | 14.4175 |
| | Sobic.004G113900 | 10.7685 | 11.63199 | 8.36388 | 7.92519 | 10.1088 | 12.03596 | 7.17395 | 6.10097 |
| | Sobic.004G319600 | 8.4499 | 8.42643 | 7.49649 | 8.92322 | 9.81914 | 8.31421 | 9.07529 | 8.77381 |
| | Sobic.005G204700 | 10.6708 | 10.44074 | 8.89415 | 9.12258 | 10.5776 | 10.41689 | 9.14055 | 8.29562 |
| | Sobic.006G178900 | 3.61343 | 2.4295 | 3.85848 | 3.56688 | 0.62615 | -3.70572 | 0.52538 | -1.96295 |
| | Sobic.007G006700 | 7.27799 | 4.5091 | 9.08222 | 9.55577 | 8.55125 | 4.9603 | 8.31647 | 8.00841 |
| | Sobic.007G112400 | -3.70572 | -3.70572 | -3.70572 | 4.57673 | 3.05105 | -3.70572 | 12.5068 | 12.2964 |
| | Sobic.007G180450 | 9.24967 | 8.9562 | 10.3543 | 11.5536 | 11.3356 | 8.90175 | 9.42563 | 8.65091 |
| | Sobic.009G216100 | 6.4625 | 8.00519 | 11.0384 | 10.511 | 11.136 | 7.62021 | 9.21095 | 7.68489 |
| | Sobic.009G216300 | 9.02751 | 10.46191 | 8.6944 | 8.6685 | 9.6378 | 10.3305 | 8.51936 | 7.77607 |
| | Sobic.009G243500 | 9.59056 | 10.46085 | 11.9508 | 9.87665 | 9.30717 | 9.80082 | 6.31114 | 6.188 |
| | Sobic.009G250800 | 9.34416 | 9.58175 | 7.75306 | 7.60122 | 8.63661 | 9.2819 | 7.52239 | 6.7185 |
| | Sobic.010G005000 | 12.3783 | 12.00943 | 11.0335 | 13.26 | 11.8501 | 12.24754 | 11.787 | 10.9216 |
| | Sobic.010G040400 | -3.70572 | -3.70572 | 4.22699 | -3.70572 | -3.70572 | -3.70572 | 7.15325 | 6.91543 |
| | Sobic.010G161400 | -3.70572 | 1.8377 | 1.80991 | 7.54872 | 4.70678 | 2.38843 | 14.5106 | 14.8217 |
| | Sobic.010G190001 | -3.70572 | -0.12078 | 2.43639 | 5.7513 | 4.54348 | 2.16692 | 14.3407 | 14.708 |
| | Sobic.010G190100 | -3.70572 | -0.12078 | 2.43639 | 5.7513 | 4.54348 | 2.16692 | 14.3407 | 14.708 |
| | Sobic.010G190200 | -3.70572 | -3.70572 | 1.87264 | -3.70572 | -3.70572 | -3.70572 | -3.70572 | -1.91011 |
| | Sobic.001G433200 | 11.3345 | 9.95189 | 13.9777 | 10.1243 | 10.7284 | 8.79492 | 10.133 | 9.47981 |
| | Sobic.001G493900 | 13.6775 | 14.20819 | 8.36193 | 12.2497 | 12.4211 | 13.73563 | 11.2386 | 10.8778 |
| | Sobic.002G279900 | 8.36176 | 8.89853 | 5.6816 | 9.0855 | 7.67468 | 8.72106 | 12.0796 | 12.7087 |
| | Sobic.003G179700 | -3.70572 | -0.13649 | 2.42068 | 3.04528 | 0.96705 | -0.17072 | 4.93666 | 2.47923 |
| | Sobic.003G197100 | 10.6572 | 11.68611 | 8.10772 | 10.1225 | 10.947 | 11.23164 | 8.80813 | 9.15193 |
| | Sobic.003G374100 | 12.2108 | 12.45697 | 3.13525 | 12.1185 | 10.1131 | 12.27194 | 10.2793 | 10.1616 |

| | | | | | | | | | |
|---|---|---|---|---|---|---|---|---|---|
| **β-Galactosidases** | Sobic.004G093400 | 9.92189 | 10.34855 | 6.62145 | 10.2188 | 8.08895 | 10.34559 | 6.80775 | 6.13422 |
| | Sobic.007G012100 | 8.15589 | 6.02014 | 6.46802 | 6.12161 | 6.18329 | 6.45794 | 3.32612 | 4.68176 |
| | Sobic.007G176900 | 7.02775 | -0.16741 | 7.50524 | 9.67257 | 7.51206 | 3.2578 | 11.4442 | 11.7392 |
| | Sobic.008G052200 | 11.9046 | 11.33022 | 10.2789 | 13.9097 | 12.9021 | 11.32054 | 11.41 | 11.7173 |
| | Sobic.009G146500 | 9.40146 | 8.69938 | 9.2661 | 8.59119 | 8.10477 | 8.91391 | 8.35514 | 7.86455 |
| | Sobic.009G213100 | 11.5163 | 10.03257 | 9.46822 | 11.331 | 11.1477 | 10.13894 | 8.62358 | 8.91633 |
| | Sobic.010G173800 | 3.41224 | 5.61297 | 11.1866 | 10.43 | 11.8123 | 4.11162 | 10.67 | 9.46216 |
| **Pectate and pectin lyases** | Sobic.001G230400 | 7.73715 | 9.99506 | 5.94047 | 8.55355 | 8.02786 | 10.1199 | 4.81903 | 4.35935 |
| | Sobic.003G322800 | -3.70572 | -3.70572 | 2.98753 | 4.19709 | 1.3819 | -3.70572 | 3.69616 | 3.49017 |
| | Sobic.003G348500 | -3.70572 | -3.70572 | -3.70572 | -3.70572 | -3.70572 | -3.70572 | 0.7313 | 1.23991 |
| | Sobic.004G091000 | -3.70572 | -3.70572 | 2.73352 | 5.57111 | 4.00236 | 0.72709 | 13.4792 | 13.6366 |
| | Sobic.006G014400 | 8.57919 | 9.19879 | 9.02403 | 9.01017 | 10.687 | 9.04494 | 10.9972 | 10.9899 |
| | Sobic.008G022800 | -3.70572 | -3.70572 | 2.41548 | 1.16561 | -3.70572 | -3.70572 | 1.12411 | 0.95465 |
| | Sobic.008G058200 | 7.19594 | 9.77957 | 10.5088 | 9.52526 | 10.2603 | 10.82547 | 9.87166 | 9.71253 |
| | Sobic.010G034200 | -3.70572 | 0.69802 | 5.849 | 9.89643 | 5.40203 | 2.85251 | 14.0209 | 14.4796 |
| | Sobic.010G034300 | -3.70572 | -3.70572 | 7.99119 | 9.21393 | 4.52316 | 2.51965 | 13.5344 | 13.695 |
| | Sobic.010G176400 | -3.70572 | -3.70572 | 1.7047 | 7.26218 | 3.76204 | -3.70572 | 12.3807 | 13.1615 |
| **Rhamnogalacturonan I lyases** | Sobic.005G024000 | -3.70572 | -3.70572 | -3.70572 | -3.70572 | -3.70572 | -3.70572 | -3.70572 | -3.70572 |
| | Sobic.005G024100 | -3.70572 | -3.70572 | 5.53647 | -3.70572 | -3.70572 | -3.70572 | -0.21433 | -2.70572 |
| | Sobic.005G123300 | -3.70572 | 3.52979 | 3.3645 | -0.2073 | 3.0999 | 1.03613 | 2.92112 | 2.3891 |
| | Sobic.007G173300 | 13.058 | 10.57519 | 8.06627 | 11.8673 | 9.18311 | 10.22038 | 9.54309 | 10.493 |
| | Sobic.008G016500 | -3.70572 | -3.70572 | 8.51238 | -0.23508 | 3.63155 | 0.00835 | 3.67761 | 2.09001 |
| | Sobic.009G259300 | -3.70572 | 0.09257 | 6.99866 | 1.39987 | -3.70572 | -3.70572 | -1.22659 | 1.08938 |
| | Sobic.001G252300 | 9.84114 | 9.07754 | 9.93427 | 10.2864 | 10.6119 | 8.8393 | 8.30225 | 8.92075 |
| | Sobic.001G490800 | 7.1071 | 8.06613 | 2.31042 | 6.30848 | 7.40523 | 7.55191 | 4.54261 | 5.05123 |
| | Sobic.002G138400 | -3.70572 | 2.23578 | 8.27408 | 3.30929 | 6.54096 | 1.20156 | 7.47898 | 8.46977 |
| | Sobic.002G251600 | -3.70572 | -3.70572 | 4.53442 | 7.59198 | 1.60686 | 2.79102 | 7.65583 | 9.08079 |
| | Sobic.002G370300 | 6.28671 | 3.38031 | -3.70572 | 12.5078 | 4.36357 | 3.34609 | 6.49411 | 5.1667 |
| | Sobic.002G399700 | -3.70572 | -3.70572 | 4.01872 | 7.61434 | 3.73502 | -3.70572 | 4.11967 | 3.28036 |
| | Sobic.003G143600 | 4.50667 | -3.70572 | 4.44696 | 4.04509 | -3.70572 | -3.70572 | 3.00359 | 1.34227 |
| | Sobic.003G178000 | -3.70572 | -3.70572 | 2.09563 | 8.13022 | 4.4286 | 2.08919 | 12.1888 | 13.1151 |
| | Sobic.003G231500 | -3.70572 | 2.33339 | 5.47553 | 2.6407 | 3.92237 | 1.29917 | 5.37179 | 4.38083 |
| | Sobic.003G292100 | 10.7291 | 9.92179 | 3.81775 | 8.94537 | 5.61228 | 10.43485 | 6.42384 | 9.45463 |
| | Sobic.003G376900 | 11.4783 | 12.0065 | 9.71027 | 10.2273 | 9.60129 | 11.84241 | 10.7194 | 9.9159 |

| | | | | | | | | | |
|---|---|---|---|---|---|---|---|---|---|
| **Pectin methyl esterases** | Sobic.004G277501 | -3.70572 | -3.70572 | -3.70572 | -3.70572 | -3.70572 | -3.70572 | 1.60401 | 2.11263 |
| | Sobic.004G350400 | -3.70572 | 1.86524 | 8.23671 | 4.67505 | 3.55375 | 2.41598 | 12.6764 | 13.2299 |
| | Sobic.004G350500 | -3.70572 | 0.98856 | -3.70572 | 2.29587 | -0.22982 | 1.95434 | 7.99583 | 7.43776 |
| | Sobic.005G009600 | 5.10614 | 4.47475 | 1.98753 | 1.73766 | -3.70572 | 3.98109 | 6.35437 | 4.18205 |
| | Sobic.005G009900 | -3.70572 | -3.70572 | -3.70572 | -3.70572 | -3.70572 | -3.70572 | 2.67103 | 1.69422 |
| | Sobic.005G217101 | -3.70572 | -3.70572 | 5.24366 | 4.63149 | 2.74735 | 2.23107 | 12.1756 | 12.6407 |
| | Sobic.006G086900 | 8.70559 | 8.86868 | 9.57387 | 8.56593 | 7.97023 | 9.35498 | 7.56436 | 7.29818 |
| | Sobic.006G172000 | 12.7431 | 13.02798 | 3.0791 | 10.5757 | 10.7566 | 13.0978 | 7.85381 | 7.23337 |
| | Sobic.007G075600 | 6.14668 | 6.75124 | 4.69792 | 6.54759 | 7.23913 | 7.09358 | 5.80865 | 5.82891 |
| | Sobic.008G131300 | -3.70572 | -3.70572 | 6.82059 | 4.43322 | 3.4925 | 0.76976 | 11.1066 | 11.5359 |
| | Sobic.009G203200 | 8.63396 | 10.13219 | 2.18423 | 7.99727 | 8.871 | 10.65739 | 8.32278 | 8.99284 |
| | Sobic.010G017600 | -3.70572 | -3.70572 | 9.92553 | 4.74023 | -3.70572 | -3.70572 | 5.85073 | 6.18717 |
| **Pectin acetyl esterases** | Sobic.001G305100 | 7.52719 | 4.93887 | 4.14554 | 10.5366 | 7.31952 | 4.27661 | 4.43914 | 4.19061 |
| | Sobic.002G388600 | 13.55 | 11.10603 | 10.361 | 13.012 | 12.7459 | 11.38273 | 10.6302 | 10.6548 |
| | Sobic.003G094800 | 4.08317 | 4.81678 | 5.50889 | 11.2671 | 9.73374 | 3.28006 | 8.24305 | 8.47528 |
| | Sobic.003G150600 | -3.70572 | 4.05759 | 13.8905 | 8.44669 | 7.93091 | 4.87136 | 8.17805 | 7.18486 |
| | Sobic.003G150700 | 6.20871 | 7.70034 | 7.63442 | 8.50201 | 6.9126 | 8.22151 | 5.48989 | 5.53616 |
| | Sobic.003G384700 | -3.70572 | 1.08648 | 8.10308 | 0.80882 | 5.67545 | -3.70572 | 3.93724 | 2.91979 |
| | Sobic.003G384800 | 6.36266 | 5.98079 | 7.48025 | 6.74495 | 6.8844 | 5.76599 | 6.41966 | 6.18607 |
| | Sobic.003G384900 | 7.94967 | 6.10278 | 2.90507 | 6.24016 | 6.85403 | 5.59907 | 6.98002 | 7.26697 |
| | Sobic.003G445000 | 10.4924 | 11.24319 | 9.72318 | 10.0852 | 11.0173 | 10.98318 | 8.9893 | 9.23487 |
| | Sobic.004G269400 | 8.54884 | 9.91914 | 6.75216 | 8.64993 | 9.97622 | 9.79463 | 8.76268 | 8.15034 |
| | Sobic.006G204500 | 10.6302 | 11.8628 | 3.90871 | 8.42703 | 11.1439 | 11.53981 | 12.4583 | 12.7675 |
| | Sobic.009G013700 | 9.74614 | 7.55223 | 7.95938 | 10.3839 | 10.6871 | 7.08504 | 8.41776 | 8.71896 |
| | Sobic.001G403100 | -3.70572 | 0.33099 | 3.88816 | 6.52906 | 4.06681 | -3.70572 | 2.71227 | 2.80585 |
| | Sobic.001G422300 | -3.70572 | 3.19714 | 3.94696 | 6.32158 | 10.0362 | 2.67749 | 11.3155 | 11.8289 |
| | Sobic.003G111900 | 3.10469 | 0.3358 | 3.62994 | 5.05814 | 0.70238 | -3.70572 | 1.33857 | 0.11022 |
| | Sobic.003G231400 | -3.70572 | 0.6197 | 3.91384 | 0.34204 | -3.70572 | -3.70572 | -0.69909 | 0.13108 |
| | Sobic.003G341500 | -3.70572 | 3.57985 | 6.18949 | 5.13227 | 9.77307 | 3.54563 | 4.13701 | 3.90316 |
| | Sobic.003G352700 | -3.70572 | 6.18966 | 7.46304 | 0.10464 | 10.7417 | 3.80751 | 8.65015 | 8.61196 |
| | Sobic.003G352800 | 2.15119 | 6.57213 | 5.2614 | 6.05884 | 9.76572 | 2.93304 | 11.2361 | 10.9011 |
| | Sobic.003G353200 | -3.70572 | 6.28919 | 1.35451 | 0.10464 | 7.99997 | 3.34808 | 3.4573 | 2.15686 |
| | Sobic.003G357500 | 4.50837 | 1.41755 | 10.3698 | 2.94725 | 4.59149 | 0.38333 | 5.40217 | 3.91079 |
| | Sobic.003G357600 | 2.98681 | 1.21791 | 12.7152 | 7.02772 | 10.0219 | 2.50562 | 9.92459 | 8.96961 |

| | | | | | | | | | |
|---|---|---|---|---|---|---|---|---|---|
| **Laccases** | Sobic.003G357700 | -3.70572 | 1.43809 | 3.74144 | 0.16043 | -3.70572 | 1.98883 | 3.02582 | 1.94947 |
| | Sobic.004G235900 | 2.12165 | -3.70572 | 6.0331 | 2.66006 | 0.13438 | -3.70572 | 1.0336 | 1.12717 |
| | Sobic.004G236000 | -3.70572 | -3.70572 | 6.91104 | 9.88649 | 7.55041 | -3.70572 | 6.84982 | 4.72456 |
| | Sobic.004G236100 | -3.70572 | -3.70572 | -3.70572 | -3.70572 | -0.80799 | -3.70572 | -1.92185 | -3.70572 |
| | Sobic.004G314200 | -3.70572 | -3.70572 | -3.70572 | -3.70572 | -3.70572 | -3.70572 | -1.99535 | -3.70572 |
| | Sobic.004G314300 | 3.12165 | -3.70572 | -3.70572 | 2.07672 | -3.70572 | -3.70572 | -3.70572 | -3.70572 |
| | Sobic.005G005800 | 2.13143 | -3.70572 | 2.91971 | 1.08488 | 6.0749 | -3.70572 | 7.72637 | 5.99908 |
| | Sobic.005G156700 | -3.70572 | -3.70572 | 2.44965 | -3.70572 | -3.70572 | -3.70572 | -3.70572 | -3.70572 |
| | Sobic.005G163800 | -3.70572 | -3.70572 | -3.70572 | -3.70572 | -3.70572 | -3.70572 | -3.70572 | -3.70572 |
| | Sobic.005G198500 | -3.70572 | -3.70572 | 8.97323 | 1.63589 | -3.70572 | -3.70572 | 2.81679 | 3.7275 |
| | Sobic.005G215300 | -3.70572 | -3.70572 | 4.76263 | -3.70572 | -3.70572 | -3.70572 | 0.59679 | -2.47956 |
| | Sobic.008G090800 | -3.70572 | -3.70572 | 3.68392 | 10.9434 | 3.69496 | -3.70572 | 4.92861 | 2.78869 |
| | Sobic.009G162300 | 2.13634 | 4.89102 | 5.42713 | 2.41172 | 7.6966 | 2.33323 | 6.82358 | 7.12676 |
| | Sobic.009G162800 | -3.70572 | 6.2501 | 5.58759 | 6.81772 | 8.85471 | 4.33323 | 11.7351 | 11.6989 |
| | Sobic.010G268500 | 4.08793 | -3.70572 | 3.29125 | 3.74182 | -3.70572 | 0.28481 | -1.00012 | -0.90655 |
