# Supplementary material for: Identification, Characterization, and Expression Analysis of Cell Wall Related Genes in Sorghum bicolor (L.) Moench, a Food, Fodder, and Biofuel Crop
Source: Front Plant Sci. 2016 Aug 31;7:1287. doi: 10.3389/fpls.2016.01287 (PMC5006623; doi:10.3389/fpls.2016.01287)
Supplement: Supplementary file 11 [file Image3.PDF]

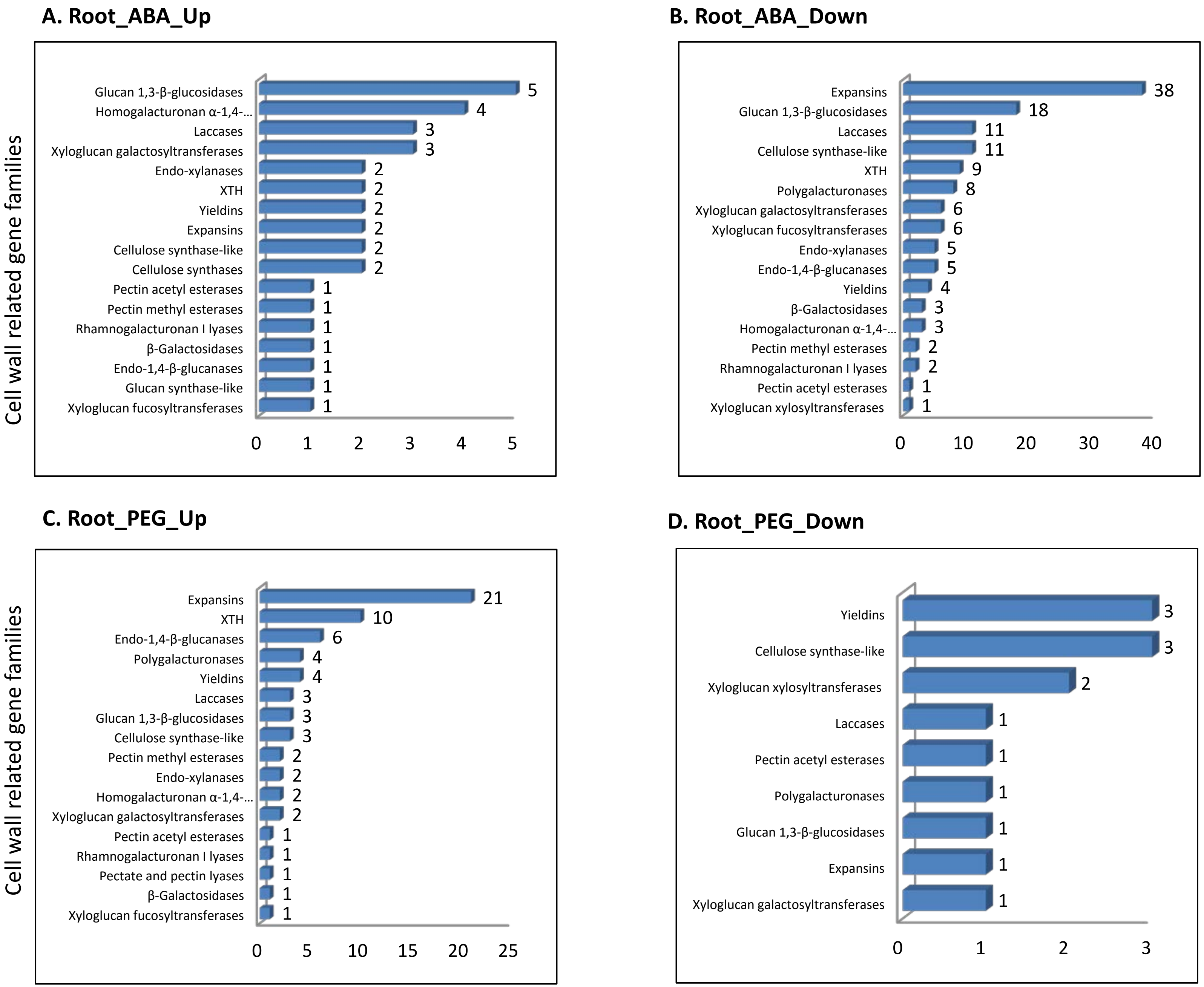

Supplementary Figure 3. Family wise distribution of differentially expressed genes from ABA and PEG treated sorghum root. (A) Root_ABA_UP (B) Root_ABA_Down (C) Root_PEG_UP (D) Root_PEG_Down.
