# Supplementary material for: Identification, Characterization, and Expression Analysis of Cell Wall Related Genes in Sorghum bicolor (L.) Moench, a Food, Fodder, and Biofuel Crop
Source: Front Plant Sci. 2016 Aug 31;7:1287. doi: 10.3389/fpls.2016.01287 (PMC5006623; doi:10.3389/fpls.2016.01287)
Supplement: Supplementary file 8 [file DataSheet1.PDF]

Supplementary Data 1: HMM profiles of 20 cell wall related gene families used to identify the Sorghum's cell wall genes using HMMER.

```
HMMER3/b [3.0 | March 2010]
NAME  CESA_n_CSL
LENG  957
ALPH  amino
RF    no
CS    no
MAP   yes
DATE  Mon Aug  8 15:27:31 2016
NSEQ  39
EFFN  2.194702
CKSUM 3070771411
STATS LOCAL MSV      -12.4787  0.69570
STATS LOCAL VITERBI  -13.8645  0.69570
STATS LOCAL FORWARD   -6.8306  0.69570
HMM       A       C       D       E       F       G       H       I       K       L       M       N       P       Q       R       S       T       V       W       Y
          m->m    m->i    m->d    i->m    i->i    d->m    d->d
  COMPO   2.57599  3.95316  2.99661  2.71710  3.19949  2.91239  3.74094  2.82521  2.69287  2.43780  3.58311  3.10620  3.31063  3.10686  2.96737  2.62478  2.85083  2.61119  4.22525  3.44338
          2.68752  4.42313  2.77531  2.73206  3.46577  2.40651  3.72581  3.29363  2.67947  2.69447  4.24277  2.90264  2.73618  3.18145  2.89761  2.37532  2.77436  2.98452  4.58865  3.61481
          0.52579  1.40210  1.81504  4.19095  0.01525  0.00000        *
      1   2.57314  4.52438  3.14616  2.64215  3.77710  3.06217  3.73642  3.16095  2.58925  2.82635  2.47636  3.10029  3.85607  2.52618  2.98084  2.24665  2.83207  2.88405  5.13711  3.86049    128 - -
          2.68618  4.42225  2.77519  2.73123  3.46354  2.40513  3.72494  3.29354  2.67741  2.69355  4.24690  2.90347  2.73739  3.18146  2.89801  2.37887  2.77519  2.98518  4.58477  3.61503
          0.13761  4.04920  2.19712  0.61958  0.77255  0.66885  0.71805
      2   2.61287  4.58959  3.04934  2.22313  2.94694  3.45192  3.64512  3.16217  2.57267  2.80808  3.68402  2.58474  3.85634  2.91001  2.99158  2.70664  2.84721  2.89666  4.92042  2.84844    129 - -
          2.68611  4.42246  2.77540  2.73144  3.46375  2.40476  3.72515  3.29375  2.67761  2.69358  4.24710  2.90339  2.73760  3.18167  2.89794  2.37851  2.77497  2.98509  4.58498  3.61524
          0.58896  0.95941  2.78096  1.25509  0.33554  0.70595  0.68051
      3   3.20347  4.55912  4.67492  4.11678  3.09783  4.44161  4.74626  1.80075  3.96291  1.05197  2.66567  4.40644  4.65246  4.14519  4.13097  3.78286  3.43058  2.09881  5.14957  4.04460    134 - -
          2.68618  4.42225  2.77519  2.73123  3.46354  2.40513  3.72494  3.29354  2.67741  2.69355  4.24690  2.90347  2.73739  3.18146  2.89801  2.37887  2.77519  2.98518  4.58477  3.61503
          0.02987  3.92172  4.64406  0.61958  0.77255  0.77195  0.62010
      4   2.98240  4.36138  4.51715  3.97149  2.09344  4.18282  4.45408  1.75439  3.85760  1.76338  3.06888  4.18156  4.48000  4.03618  4.02328  3.51419  3.22254  1.72162  4.90974  3.62533    135 - -
          2.68618  4.42225  2.77519  2.73123  3.46354  2.40513  3.72494  3.29354  2.67741  2.69355  4.24690  2.90347  2.73739  3.18146  2.89801  2.37887  2.77519  2.98518  4.58477  3.61503
          0.02987  3.92172  4.64406  0.61958  0.77255  0.77195  0.62010
      5   1.52170  4.13968  3.49069  3.16215  4.06305  3.00127  4.17473  3.17338  3.13958  3.06383  3.99025  3.32615  3.71874  3.46428  3.43328  2.44746  1.76701  2.79667  5.48437  4.27165    136 - -
```

2.68618 4.42225 2.77519 2.73123 3.46354 2.40513 3.72494 3.29354 2.67741 2.69355 4.24690 2.90347 2.73739 3.18146 2.89801 2.37887 2.77519 2.98518 4.58477 3.61503
0.02987 3.92172 4.64406 0.61958 0.77255 0.77195 0.62010
6 2.72642 4.49714 3.41029 3.31587 4.45757 0.65701 4.43327 4.06903 3.50385 3.71703 4.69306 3.57454 3.88986 3.82409 3.73463 2.89972 3.21148 3.58784 5.53986 4.56108 137 - -
2.68618 4.42225 2.77519 2.73123 3.46354 2.40513 3.72494 3.29354 2.67741 2.69355 4.24690 2.90347 2.73739 3.18146 2.89801 2.37887 2.77519 2.98518 4.58477 3.61503
0.02987 3.92172 4.64406 0.61958 0.77255 0.77195 0.62010
7 2.23923 4.20767 3.15010 2.94177 4.31182 1.77571 4.12669 3.78014 3.09849 3.44905 4.27867 3.18032 3.66231 3.38723 3.43301 1.53523 2.72184 3.21977 5.63079 4.38758 138 - -
2.68626 4.42233 2.77528 2.73131 3.46275 2.40521 3.72503 3.29362 2.67731 2.69363 4.24698 2.90327 2.73748 3.18154 2.89809 2.37865 2.77528 2.98526 4.58485 3.61511
0.25371 1.53959 4.64406 0.95031 0.48877 0.77195 0.62010
8 2.67417 4.39558 3.45249 2.93290 3.01094 3.58970 2.66608 2.86525 2.81236 2.54018 3.49813 3.31027 3.98313 3.16851 3.13304 2.86899 2.91185 2.02841 4.55300 2.69881 142 - -
2.68618 4.42225 2.77519 2.73123 3.46354 2.40513 3.72494 3.29354 2.67741 2.69355 4.24690 2.90347 2.73739 3.18146 2.89801 2.37887 2.77519 2.98518 4.58477 3.61503
0.02987 3.92172 4.64406 0.61958 0.77255 0.77195 0.62010
9 2.57881 4.67543 2.95287 2.52622 4.10068 3.30664 3.69123 3.50414 2.40509 3.12275 3.96450 2.32170 3.80188 2.86607 2.45921 2.64322 2.10010 3.15980 5.34391 4.04381 143 - -
2.68618 4.42225 2.77519 2.73123 3.46354 2.40513 3.72494 3.29354 2.67741 2.69355 4.24690 2.90347 2.73739 3.18146 2.89801 2.37887 2.77519 2.98518 4.58477 3.61503
0.02987 3.92172 4.64406 0.61958 0.77255 0.69175 0.69455
10 2.82810 4.83806 3.30996 2.80284 4.30277 3.46531 3.75024 3.73871 2.20748 3.27928 4.17564 3.20581 3.08257 2.92669 1.24753 2.89232 3.09613 3.40922 5.39249 4.18234 144 - -
2.68618 4.42225 2.77519 2.73123 3.46354 2.40513 3.72494 3.29354 2.67741 2.69355 4.24690 2.90347 2.73739 3.18146 2.89801 2.37887 2.77519 2.98518 4.58477 3.61503
0.02833 3.97389 4.69624 0.61958 0.77255 0.73259 0.65520
11 2.18577 4.64583 2.86614 2.48596 4.02376 2.74378 3.72321 3.46147 2.57472 3.09344 3.93118 2.09202 3.79125 2.91096 3.00935 2.61800 2.84410 3.11911 5.33034 3.53969 145 - -
2.68618 4.42225 2.77519 2.73123 3.46354 2.40513 3.72494 3.29354 2.67741 2.69355 4.24690 2.90347 2.73739 3.18146 2.89801 2.37887 2.77519 2.98518 4.58477 3.61503
0.08204 3.97389 2.81397 0.61958 0.77255 0.73259 0.65520
12 2.80418 5.08542 2.53835 1.69740 4.21038 3.33076 2.27182 3.82605 2.43353 3.36629 4.22335 2.84532 3.84347 2.83950 2.83934 2.77380 3.05530 3.47981 5.44740 4.04132 146 - -
2.68618 4.42225 2.77519 2.73123 3.46354 2.40513 3.72494 3.29354 2.67741 2.69355 4.24690 2.90347 2.73739 3.18146 2.89801 2.37887 2.77519 2.98518 4.58477 3.61503
0.02987 3.92172 4.64406 0.61958 0.77255 0.77195 0.62010
13 3.06569 4.45598 4.51305 3.96930 2.19321 4.22880 4.41907 2.16434 3.84910 1.29943 2.97372 4.19591 4.50309 4.01064 4.01338 3.55888 3.29968 1.90786 4.82598 3.51618 147 - -
2.68618 4.42225 2.77519 2.73123 3.46354 2.40513 3.72494 3.29354 2.67741 2.69355 4.24690 2.90347 2.73739 3.18146 2.89801 2.37887 2.77519 2.98518 4.58477 3.61503
0.02987 3.92172 4.64406 0.61958 0.77255 0.71830 0.66862
14 3.01222 4.37928 4.56590 4.01520 3.30738 4.26796 4.66815 1.71142 3.89890 1.82376 2.30868 4.26704 4.55692 4.11773 4.09107 3.60369 3.25518 1.41856 5.20761 4.05748 148 - -
2.68618 4.42225 2.77519 2.73123 3.46354 2.40513 3.72494 3.29354 2.67741 2.69355 4.24690 2.90347 2.73739 3.18146 2.89801 2.37887 2.77519 2.98518 4.58477 3.61503
0.02883 3.95646 4.67880 0.61958 0.77255 0.74615 0.64281
15 2.59984 4.85204 2.40322 2.15656 4.10593 3.37165 3.59798 3.51828 2.37911 2.66417 3.92512 2.88806 3.78465 2.74165 2.59854 2.61418 2.83397 3.00771 5.34191 3.99316 149 - -
2.68618 4.42225 2.77519 2.73123 3.46354 2.40513 3.72494 3.29354 2.67741 2.69355 4.24690 2.90347 2.73739 3.18146 2.89801 2.37887 2.77519 2.98518 4.58477 3.61503
0.02883 3.95646 4.67880 0.61958 0.77255 0.74615 0.64281
16 2.54347 3.33796 3.42431 2.86727 3.46761 3.49145 3.79177 2.23661 2.39356 2.53041 3.42792 3.05706 3.89927 3.10978 3.09106 2.62719 2.79058 2.54961 4.90353 3.66836 150 - -
2.68618 4.42225 2.77519 2.73123 3.46354 2.40513 3.72494 3.29354 2.67741 2.69355 4.24690 2.90347 2.73739 3.18146 2.89801 2.37887 2.77519 2.98518 4.58477 3.61503
0.02883 3.95646 4.67880 0.61958 0.77255 0.66594 0.72111

17 2.58949 4.75805 2.96746 2.35067 4.00442 3.40365 3.36958 3.41521 2.40108 3.02341 3.84477 2.57281 3.80446 2.77687 2.61147 2.39742 2.64144 2.66971 5.26965 3.94594 151 - -

2.68618 4.42225 2.77519 2.73123 3.46354 2.40513 3.72494 3.29354 2.67741 2.69355 4.24690 2.90347 2.73739 3.18146 2.89801 2.37887 2.77519 2.98518 4.58477 3.61503

0.02739 4.00689 4.72924 0.61958 0.77255 0.70574 0.68071

18 2.28809 4.40736 3.06582 2.69421 3.57139 3.25286 3.45737 2.56245 2.66592 2.63436 2.80091 2.98224 3.86976 2.99094 3.06065 2.71805 2.79254 2.69461 4.97664 3.71718 152 - -

2.68618 4.42225 2.77519 2.73123 3.46354 2.40513 3.72494 3.29354 2.67741 2.69355 4.24690 2.90347 2.73739 3.18146 2.89801 2.37887 2.77519 2.98518 4.58477 3.61503

0.02739 4.00689 4.72924 0.61958 0.77255 0.70574 0.68071

19 2.53938 5.31608 1.48990 1.95758 4.59699 3.26367 3.28797 4.08950 2.63250 3.60802 4.42859 2.73256 3.82396 2.85873 3.17885 2.75185 3.09748 3.68589 5.78081 4.32492 153 - -

2.68618 4.42225 2.77519 2.73123 3.46354 2.40513 3.72494 3.29354 2.67741 2.69355 4.24690 2.90347 2.73739 3.18146 2.89801 2.37887 2.77519 2.98518 4.58477 3.61503

0.09548 4.00689 2.61897 0.61958 0.77255 0.70574 0.68071

20 2.61493 4.90180 2.64252 2.13620 4.20967 3.33541 3.60682 3.64197 2.38106 3.20946 4.01352 2.86125 3.77833 2.74712 2.51137 2.13063 2.63000 3.28324 5.41447 4.05749 154 - -

2.68618 4.42225 2.77519 2.73123 3.46354 2.40513 3.72494 3.29354 2.67741 2.69355 4.24690 2.90347 2.73739 3.18146 2.89801 2.37887 2.77519 2.98518 4.58477 3.61503

0.02930 3.94071 4.66305 0.61958 0.77255 0.75804 0.63221

21 2.34511 4.82369 2.40293 2.21285 3.51647 3.34528 3.60941 3.50947 2.42971 3.10556 3.91813 2.76692 3.77505 2.75959 2.90502 2.44635 2.71853 3.17320 5.34014 3.98950 155 - -

2.68618 4.42225 2.77519 2.73123 3.46354 2.40513 3.72494 3.29354 2.67741 2.69355 4.24690 2.90347 2.73739 3.18146 2.89801 2.37887 2.77519 2.98518 4.58477 3.61503

0.11957 3.94071 2.37229 0.61958 0.77255 0.65482 0.73300

22 2.24087 4.71130 2.50452 2.30838 4.02827 3.09946 3.64864 3.41994 2.48734 3.05353 3.88002 2.91451 3.77452 2.81449 2.94927 2.60089 2.47530 2.82243 5.31146 3.98033 156 - -

2.68618 4.42225 2.77519 2.73123 3.46354 2.40513 3.72494 3.29354 2.67741 2.69355 4.24690 2.90347 2.73739 3.18146 2.89801 2.37887 2.77519 2.98518 4.58477 3.61503

0.02976 3.92518 4.64753 0.61958 0.77255 0.76944 0.62226

23 2.47554 4.87992 2.58068 2.24061 4.18991 2.94270 3.59489 3.62606 2.38066 3.19272 3.99066 2.85519 3.76304 2.73190 2.46737 2.41876 2.63003 3.26335 5.39923 4.03829 157 - -

2.68618 4.42225 2.77519 2.73123 3.46354 2.40513 3.72494 3.29354 2.67741 2.69355 4.24690 2.90347 2.73739 3.18146 2.89801 2.37887 2.77519 2.98518 4.58477 3.61503

0.02976 3.92518 4.64753 0.61958 0.77255 0.76944 0.62226

24 2.55759 4.52541 3.09831 2.41349 3.71366 3.20823 2.98311 2.65974 2.52131 2.64583 3.62156 3.04044 3.51811 2.87686 2.93297 2.66627 2.67341 2.82775 5.07138 3.78864 158 - -

2.68618 4.42225 2.77519 2.73123 3.46354 2.40513 3.72494 3.29354 2.67741 2.69355 4.24690 2.90347 2.73739 3.18146 2.89801 2.37887 2.77519 2.98518 4.58477 3.61503

0.02976 3.92518 4.64753 0.61958 0.77255 0.76944 0.62226

25 2.73843 4.96931 3.05326 2.49355 4.29548 3.46719 3.12259 3.70540 1.81432 3.22962 4.05061 2.98070 2.92539 2.70695 2.11069 2.74148 2.94867 3.36497 5.37329 4.08773 159 - -

2.68618 4.42225 2.77519 2.73123 3.46354 2.40513 3.72494 3.29354 2.67741 2.69355 4.24690 2.90347 2.73739 3.18146 2.89801 2.37887 2.77519 2.98518 4.58477 3.61503

0.02976 3.92518 4.64753 0.61958 0.77255 0.64439 0.74440

26 2.62848 4.99949 2.45783 2.30400 4.31310 3.34468 3.60003 3.76601 2.37107 3.30253 4.08675 2.84478 2.87374 2.38340 2.64122 2.30323 2.86954 3.38181 5.48202 4.10173 160 - -

2.68618 4.42225 2.77519 2.73123 3.46354 2.40513 3.72494 3.29354 2.67741 2.69355 4.24690 2.90347 2.73739 3.18146 2.89801 2.37887 2.77519 2.98518 4.58477 3.61503

0.02739 4.00689 4.72924 0.61958 0.77255 0.64468 0.74408

27 2.49220 4.24378 3.54916 2.98180 3.35709 3.57506 3.38001 2.69306 2.90526 2.16361 2.53339 3.35805 3.23679 3.21089 3.22746 2.83325 2.81150 2.28464 4.83381 3.60860 161 - -

2.68618 4.42225 2.77519 2.73123 3.46354 2.40513 3.72494 3.29354 2.67741 2.69355 4.24690 2.90347 2.73739 3.18146 2.89801 2.37887 2.77519 2.98518 4.58477 3.61503

0.02640 4.04331 4.76566 0.61958 0.77255 0.67416 0.71250

28 2.33356 4.91421 2.90037 2.09085 4.19486 3.39805 3.59761 3.62416 2.06269 3.18688 3.59212 2.90573 3.80009 2.56278 2.77899 2.47297 2.85039 3.27088 5.38782 4.03809 162 - -

2.68618 4.42225 2.77519 2.73123 3.46354 2.40513 3.72494 3.29354 2.67741 2.69355 4.24690 2.90347 2.73739 3.18146 2.89801 2.37887 2.77519 2.98518 4.58477 3.61503

0.02640 4.04331 4.76566 0.61958 0.77255 0.67416 0.71250

29 2.71996 5.19166 2.40882 1.89359 4.50153 2.69315 3.64049 3.97893 2.21041 3.48517 4.26803 2.39473 3.80178 2.76787 2.97320 2.46698 2.96783 3.56849 5.64599 4.22395 163 - -

2.68618 4.42225 2.77519 2.73123 3.46354 2.40513 3.72494 3.29354 2.67741 2.69355 4.24690 2.90347 2.73739 3.18146 2.89801 2.37887 2.77519 2.98518 4.58477 3.61503

0.02640 4.04331 4.76566 0.61958 0.77255 0.67416 0.71250

30 2.28735 4.23409 3.55730 2.98755 3.04975 3.58060 3.84561 2.55589 2.60484 2.02986 3.13136 3.36295 3.95800 3.21697 3.24056 2.68785 2.80785 2.46051 4.82903 3.60487 164 - -

2.68618 4.42225 2.77519 2.73123 3.46354 2.40513 3.72494 3.29354 2.67741 2.69355 4.24690 2.90347 2.73739 3.18146 2.89801 2.37887 2.77519 2.98518 4.58477 3.61503

0.02640 4.04331 4.76566 0.61958 0.77255 0.67416 0.71250

31 2.61991 4.93067 2.72346 2.36224 4.23159 3.37800 3.60625 3.66873 2.12243 3.22538 4.01865 2.64405 3.79540 2.73730 2.66767 2.04236 2.85715 3.10441 5.42055 4.06223 165 - -

2.68618 4.42225 2.77519 2.73123 3.46354 2.40513 3.72494 3.29354 2.67741 2.69355 4.24690 2.90347 2.73739 3.18146 2.89801 2.37887 2.77519 2.98518 4.58477 3.61503

0.02640 4.04331 4.76566 0.61958 0.77255 0.67416 0.71250

32 2.03262 4.24249 3.26739 3.01996 4.38282 1.34329 4.18563 3.81191 3.15943 3.47754 4.29386 3.24611 3.15307 3.43638 3.50374 2.21937 2.75088 3.25049 5.70368 4.47435 166 - -

2.68618 4.42225 2.77519 2.73123 3.46354 2.40513 3.72494 3.29354 2.67741 2.69355 4.24690 2.90347 2.73739 3.18146 2.89801 2.37887 2.77519 2.98518 4.58477 3.61503

0.02640 4.04331 4.76566 0.61958 0.77255 0.67416 0.71250

33 2.69784 4.96119 2.83459 2.41728 4.31520 3.38828 3.64962 3.74483 2.18557 3.29361 4.10689 2.93147 3.40552 1.84962 2.72501 2.31430 2.94382 3.38230 5.47164 4.13820 167 - -

2.68618 4.42225 2.77519 2.73123 3.46354 2.40513 3.72494 3.29354 2.67741 2.69355 4.24690 2.90347 2.73739 3.18146 2.89801 2.37887 2.77519 2.98518 4.58477 3.61503

0.02640 4.04331 4.76566 0.61958 0.77255 0.67416 0.71250

34 2.58640 4.20651 3.73271 3.18834 3.12845 3.59102 3.99057 1.88623 3.11176 2.38943 3.35860 3.50466 4.01799 3.39925 3.40264 2.44910 2.32054 2.30309 4.89778 3.67175 168 - -

2.68618 4.42225 2.77519 2.73123 3.46354 2.40513 3.72494 3.29354 2.67741 2.69355 4.24690 2.90347 2.73739 3.18146 2.89801 2.37887 2.77519 2.98518 4.58477 3.61503

0.02640 4.04331 4.76566 0.61958 0.77255 0.67416 0.71250

35 2.79551 0.65115 4.35001 4.13086 4.08655 3.37964 4.74071 3.26180 3.95987 3.16810 4.29606 4.07264 4.09973 4.29913 4.05286 3.06703 3.27689 2.99405 5.46304 4.37218 169 - -

2.68618 4.42225 2.77519 2.73123 3.46354 2.40513 3.72494 3.29354 2.67741 2.69355 4.24690 2.90347 2.73739 3.18146 2.89801 2.37887 2.77519 2.98518 4.58477 3.61503

0.02640 4.04331 4.76566 0.61958 0.77255 0.67416 0.71250

36 2.33285 4.81587 2.98428 2.36040 4.06356 3.42806 3.61772 3.47764 2.17795 3.07189 3.89071 2.79681 3.82291 2.26517 2.77381 2.65596 2.85262 3.15644 3.72126 3.97842 170 - -

2.68618 4.42225 2.77519 2.73123 3.46354 2.40513 3.72494 3.29354 2.67741 2.69355 4.24690 2.90347 2.73739 3.18146 2.89801 2.37887 2.77519 2.98518 4.58477 3.61503

0.02640 4.04331 4.76566 0.61958 0.77255 0.58155 0.81878

37 2.99928 4.34389 4.66257 4.10492 3.40368 4.28692 4.68775 1.45767 3.99343 1.81176 2.86273 4.31372 4.58179 4.20008 4.16108 3.61807 2.93394 1.48494 5.22428 4.06099 171 - -

2.68614 4.42232 2.77527 2.73130 3.46361 2.40498 3.72502 3.29361 2.67730 2.69362 4.24697 2.90354 2.73709 3.18153 2.89808 2.37884 2.77527 2.98525 4.58484 3.61510

0.27483 1.46004 4.81930 0.67158 0.71518 0.62345 0.76807

38 2.53141 1.26212 4.33227 3.94209 3.72187 3.38545 4.56558 2.53122 3.75170 2.60063 3.72733 3.88554 4.06729 4.04365 3.91245 2.84777 2.96815 2.04291 5.35870 4.12517 174 - -

2.68618 4.42225 2.77519 2.73123 3.46354 2.40513 3.72494 3.29354 2.67741 2.69355 4.24690 2.90347 2.73739 3.18146 2.89801 2.37887 2.77519 2.98518 4.58477 3.61503

0.02501 4.09696 4.81930 0.61958 0.77255 0.62345 0.76807

39 2.68385 5.02447 2.02263 2.31969 4.43680 2.18608 3.70820 3.90053 2.54740 3.44364 4.24064 2.85933 3.81792 2.84976 2.74030 2.31291 2.96104 3.49320 5.62654 4.23315 175 - -

2.68618 4.42225 2.77519 2.73123 3.46354 2.40513 3.72494 3.29354 2.67741 2.69355 4.24690 2.90347 2.73739 3.18146 2.89801 2.37887 2.77519 2.98518 4.58477 3.61503

0.02501 4.09696 4.81930 0.61958 0.77255 0.62345 0.76807

40 2.76763 5.19931 1.72856 1.92702 4.51259 2.62847 3.70151 3.97585 2.57588 3.51272 4.31539 2.80457 3.83427 2.84254 3.10112 2.71814 3.02806 3.08985 5.69536 4.27063 176 - -

2.68618 4.42225 2.77519 2.73123 3.46354 2.40513 3.72494 3.29354 2.67741 2.69355 4.24690 2.90347 2.73739 3.18146 2.89801 2.37887 2.77519 2.98518 4.58477 3.61503
0.02501 4.09696 4.81930 0.61958 0.77255 0.62345 0.76807
41 2.78573 5.32108 2.08190 1.76726 4.63560 3.32484 3.67162 4.12990 2.08023 3.60932 4.39014 2.42224 3.82941 2.63963 3.02012 2.71345 3.03586 3.69918 5.75066 4.30704 177 - -
2.68618 4.42225 2.77519 2.73123 3.46354 2.40513 3.72494 3.29354 2.67741 2.69355 4.24690 2.90347 2.73739 3.18146 2.89801 2.37887 2.77519 2.98518 4.58477 3.61503
0.02501 4.09696 4.81930 0.61958 0.77255 0.62345 0.76807
42 3.15335 4.44968 4.91941 4.42938 3.64234 4.53487 5.13937 1.29902 4.32177 2.13692 3.43893 4.63475 4.83998 4.58371 4.51306 3.93094 3.42026 1.07692 5.62671 4.41938 178 - -
2.68618 4.42225 2.77519 2.73123 3.46354 2.40513 3.72494 3.29354 2.67741 2.69355 4.24690 2.90347 2.73739 3.18146 2.89801 2.37887 2.77519 2.98518 4.58477 3.61503
0.02501 4.09696 4.81930 0.61958 0.77255 0.62345 0.76807
43 2.59972 4.57550 3.13513 2.32063 3.77284 2.65541 3.70552 3.13682 2.41976 2.81375 3.00739 3.07969 3.86714 2.91707 2.97614 2.70620 2.83456 2.41608 5.12837 3.84503 179 - -
2.68618 4.42225 2.77519 2.73123 3.46354 2.40513 3.72494 3.29354 2.67741 2.69355 4.24690 2.90347 2.73739 3.18146 2.89801 2.37887 2.77519 2.98518 4.58477 3.61503
0.02501 4.09696 4.81930 0.61958 0.77255 0.62345 0.76807
44 2.60405 4.71325 2.81497 2.49006 3.92812 3.43926 3.12427 3.33368 2.26900 2.34081 3.79621 2.99641 3.83523 2.82851 2.90764 2.35532 2.83628 2.87096 5.22944 3.91485 180 - -
2.68618 4.42225 2.77519 2.73123 3.46354 2.40513 3.72494 3.29354 2.67741 2.69355 4.24690 2.90347 2.73739 3.18146 2.89801 2.37887 2.77519 2.98518 4.58477 3.61503
0.02501 4.09696 4.81930 0.61958 0.77255 0.62345 0.76807
45 2.51938 4.94165 2.20922 2.35224 4.31889 2.57853 3.68111 3.76367 2.37904 3.32654 4.12377 2.88447 3.80741 2.82166 2.99166 2.54617 2.25264 3.37869 5.52833 4.15388 181 - -
2.68618 4.42225 2.77519 2.73123 3.46354 2.40513 3.72494 3.29354 2.67741 2.69355 4.24690 2.90347 2.73739 3.18146 2.89801 2.37887 2.77519 2.98518 4.58477 3.61503
0.10137 4.09696 2.52855 0.61958 0.77255 0.62345 0.76807
46 2.63830 4.92742 2.45858 2.01701 4.19822 3.35644 3.62956 3.62360 2.34889 3.20669 4.01417 2.86738 3.79816 2.77305 2.92162 2.33212 2.87993 2.58869 5.42639 4.06163 182 - -
2.68618 4.42225 2.77519 2.73123 3.46354 2.40513 3.72494 3.29354 2.67741 2.69355 4.24690 2.90347 2.73739 3.18146 2.89801 2.37887 2.77519 2.98518 4.58477 3.61503
0.02696 4.02255 4.74490 0.61958 0.77255 0.69243 0.69387
47 2.63578 5.03693 2.43906 2.21049 4.34741 3.34554 3.59785 3.80542 2.37281 3.33286 4.11127 2.42716 3.78047 2.72002 2.47824 2.35433 2.62077 3.41378 5.50592 4.11675 183 - -
2.68618 4.42225 2.77519 2.73123 3.46354 2.40513 3.72494 3.29354 2.67741 2.69355 4.24690 2.90347 2.73739 3.18146 2.89801 2.37887 2.77519 2.98518 4.58477 3.61503
0.02696 4.02255 4.74490 0.61958 0.77255 0.69243 0.69387
48 2.36127 4.30450 3.19973 3.07864 4.41646 1.00866 4.28438 3.92651 3.28763 3.61767 4.47898 3.30092 3.76199 3.57969 3.58650 2.32777 2.86281 3.35561 5.72710 4.49594 184 - -
2.68618 4.42225 2.77519 2.73123 3.46354 2.40513 3.72494 3.29354 2.67741 2.69355 4.24690 2.90347 2.73739 3.18146 2.89801 2.37887 2.77519 2.98518 4.58477 3.61503
0.02696 4.02255 4.74490 0.61958 0.77255 0.69243 0.69387
49 2.74576 5.25603 2.16616 1.86533 4.57118 3.31149 3.63624 4.05676 2.10726 3.54233 4.32349 2.61946 3.80335 2.33844 2.94451 2.68091 2.99231 3.63428 5.68586 4.25617 185 - -
2.68618 4.42225 2.77519 2.73123 3.46354 2.40513 3.72494 3.29354 2.67741 2.69355 4.24690 2.90347 2.73739 3.18146 2.89801 2.37887 2.77519 2.98518 4.58477 3.61503
0.02696 4.02255 4.74490 0.61958 0.77255 0.69243 0.69387
50 2.56811 4.64740 2.48051 2.49204 3.52260 3.42238 3.64544 3.25320 2.48292 2.68757 3.73525 2.99384 3.39321 2.82617 2.79879 2.56597 2.63240 2.71895 5.18015 3.87176 186 - -
2.68618 4.42225 2.77519 2.73123 3.46354 2.40513 3.72494 3.29354 2.67741 2.69355 4.24690 2.90347 2.73739 3.18146 2.89801 2.37887 2.77519 2.98518 4.58477 3.61503
0.02696 4.02255 4.74490 0.61958 0.77255 0.69243 0.69387
51 2.74326 4.30026 3.87036 3.34348 1.79575 3.74985 3.89663 2.58063 3.24292 2.10570 3.33453 3.05034 4.13635 3.50915 3.49738 3.04936 2.98958 2.24722 4.52443 3.07582 187 - -
2.68618 4.42225 2.77519 2.73123 3.46354 2.40513 3.72494 3.29354 2.67741 2.69355 4.24690 2.90347 2.73739 3.18146 2.89801 2.37887 2.77519 2.98518 4.58477 3.61503
0.02696 4.02255 4.74490 0.61958 0.77255 0.69243 0.69387

52 2.74693 4.28173 3.91273 2.92807 3.37843 3.84255 4.14712 2.14354 3.25260 2.05704 2.93998 3.68839 4.19603 3.54752 3.53638 3.12428 2.98323 1.51974 4.99905 3.79347 188 - -
2.68618 4.42225 2.77519 2.73123 3.46354 2.40513 3.72494 3.29354 2.67741 2.69355 4.24690 2.90347 2.73739 3.18146 2.89801 2.37887 2.77519 2.98518 4.58477 3.61503
0.02696 4.02255 4.74490 0.61958 0.77255 0.56737 0.83705
53 1.62120 4.53042 3.19566 2.73440 4.10091 3.27807 3.85048 3.48731 2.63319 3.13428 3.98051 3.14132 2.51811 3.04778 2.53675 2.62471 2.85028 3.12242 5.39493 4.12161 189 - -
2.68618 4.42225 2.77519 2.73123 3.46354 2.40513 3.72494 3.29354 2.67741 2.69355 4.24690 2.90347 2.73739 3.18146 2.89801 2.37887 2.77519 2.98518 4.58477 3.61503
0.02501 4.09696 4.81930 0.61958 0.77255 0.62345 0.76807
54 2.61737 1.10857 2.72328 3.26379 3.95915 3.32343 4.26679 3.21460 3.32577 3.07609 4.03291 3.53114 3.97340 3.62648 3.61958 2.82685 3.02342 2.91538 5.36527 4.14896 190 - -
2.68623 4.42230 2.77516 2.73128 3.46358 2.40517 3.72444 3.29359 2.67745 2.69359 4.24694 2.90297 2.73744 3.18151 2.89805 2.37891 2.77524 2.98523 4.58482 3.61508
0.37060 1.19864 4.81930 0.19120 1.74851 0.62345 0.76807
55 2.69868 4.68692 3.15153 1.68232 3.83789 3.54846 3.79663 2.76362 2.61377 2.49957 3.71350 3.14290 3.95203 2.98803 3.02622 2.80607 2.93790 2.52080 5.26823 3.96603 192 - -
2.68618 4.42225 2.77519 2.73123 3.46354 2.40513 3.72494 3.29354 2.67741 2.69355 4.24690 2.90347 2.73739 3.18146 2.89801 2.37887 2.77519 2.98518 4.58477 3.61503
0.08005 4.09696 2.80830 0.61958 0.77255 0.62345 0.76807
56 2.79551 0.65115 4.35001 4.13086 4.08655 3.37964 4.74071 3.26180 3.95987 3.16810 4.29606 4.07264 4.09973 4.29913 4.05286 3.06703 3.27689 2.99405 5.46304 4.37218 193 - -
2.68618 4.42225 2.77519 2.73123 3.46354 2.40513 3.72494 3.29354 2.67741 2.69355 4.24690 2.90347 2.73739 3.18146 2.89801 2.37887 2.77519 2.98518 4.58477 3.61503
0.02640 4.04331 4.76566 0.61958 0.77255 0.67416 0.71250
57 2.16949 4.69630 2.72291 2.47283 4.06437 2.59816 3.67951 3.47930 2.49849 3.09163 3.90829 2.96847 3.79075 2.84076 2.72512 2.36993 2.81489 2.86388 5.33695 4.01133 194 - -
2.68618 4.42225 2.77519 2.73123 3.46354 2.40513 3.72494 3.29354 2.67741 2.69355 4.24690 2.90347 2.73739 3.18146 2.89801 2.37887 2.77519 2.98518 4.58477 3.61503
0.02640 4.04331 4.76566 0.61958 0.77255 0.67416 0.71250
58 3.55056 4.83810 4.56087 4.22936 0.92691 4.34174 3.57583 3.15841 4.08086 2.48264 3.77578 4.07962 4.66675 4.10741 4.14585 3.72257 3.78333 3.11581 3.73613 1.81990 195 - -
2.68618 4.42225 2.77519 2.73123 3.46354 2.40513 3.72494 3.29354 2.67741 2.69355 4.24690 2.90347 2.73739 3.18146 2.89801 2.37887 2.77519 2.98518 4.58477 3.61503
0.02640 4.04331 4.76566 0.61958 0.77255 0.67416 0.71250
59 2.78407 4.94308 3.09136 2.58563 4.31940 3.48334 3.65809 3.71766 1.91078 3.26180 4.10189 3.05584 2.03156 2.80657 2.19692 2.80684 3.01451 3.38245 5.41895 4.15095 196 - -
2.68618 4.42225 2.77519 2.73123 3.46354 2.40513 3.72494 3.29354 2.67741 2.69355 4.24690 2.90347 2.73739 3.18146 2.89801 2.37887 2.77519 2.98518 4.58477 3.61503
0.02640 4.04331 4.76566 0.61958 0.77255 0.67416 0.71250
60 2.73845 4.32026 4.54047 4.00367 3.54855 4.22145 4.69876 1.40207 3.90178 2.09713 3.39233 4.24053 4.55812 4.16394 4.11858 3.56322 2.98060 1.34200 5.33965 4.14081 197 - -
2.68618 4.42225 2.77519 2.73123 3.46354 2.40513 3.72494 3.29354 2.67741 2.69355 4.24690 2.90347 2.73739 3.18146 2.89801 2.37887 2.77519 2.98518 4.58477 3.61503
0.02640 4.04331 4.76566 0.61958 0.77255 0.67416 0.71250
61 2.79551 0.65115 4.35001 4.13086 4.08655 3.37964 4.74071 3.26180 3.95987 3.16810 4.29606 4.07264 4.09973 4.29913 4.05286 3.06703 3.27689 2.99405 5.46304 4.37218 198 - -
2.68618 4.42225 2.77519 2.73123 3.46354 2.40513 3.72494 3.29354 2.67741 2.69355 4.24690 2.90347 2.73739 3.18146 2.89801 2.37887 2.77519 2.98518 4.58477 3.61503
0.02640 4.04331 4.76566 0.61958 0.77255 0.67416 0.71250
62 3.08602 5.20281 3.48796 2.81409 4.65527 3.70039 3.61978 3.99447 1.66805 3.42692 4.29506 3.21472 4.05973 2.56387 1.26723 3.07711 3.24349 3.67860 5.46832 4.30560 199 - -
2.68618 4.42225 2.77519 2.73123 3.46354 2.40513 3.72494 3.29354 2.67741 2.69355 4.24690 2.90347 2.73739 3.18146 2.89801 2.37887 2.77519 2.98518 4.58477 3.61503
0.02640 4.04331 4.76566 0.61958 0.77255 0.67416 0.71250
63 2.46993 4.92736 1.91385 2.32950 4.43089 3.23860 3.76248 3.88002 2.65877 3.45687 4.27273 2.85778 2.14524 2.92251 3.16087 2.50008 2.96842 3.46574 5.65884 4.27014 200 - -
2.68618 4.42225 2.77519 2.73123 3.46354 2.40513 3.72494 3.29354 2.67741 2.69355 4.24690 2.90347 2.73739 3.18146 2.89801 2.37887 2.77519 2.98518 4.58477 3.61503

0.02640 4.04331 4.76566 0.61958 0.77255 0.67416 0.71250

64 2.79551 0.65115 4.35001 4.13086 4.08655 3.37964 4.74071 3.26180 3.95987 3.16810 4.29606 4.07264 4.09973 4.29913 4.05286 3.06703 3.27689 2.99405 5.46304 4.37218 201 - -

2.68618 4.42225 2.77519 2.73123 3.46354 2.40513 3.72494 3.29354 2.67741 2.69355 4.24690 2.90347 2.73739 3.18146 2.89801 2.37887 2.77519 2.98518 4.58477 3.61503

0.02640 4.04331 4.76566 0.61958 0.77255 0.67416 0.71250

65 3.48954 4.79168 4.53946 4.15481 1.67035 4.32226 3.54336 3.16365 4.00354 2.29724 3.78100 4.03041 4.63103 4.04147 4.08894 3.66502 3.71091 3.08925 3.71609 1.08226 202 - -

2.68618 4.42225 2.77519 2.73123 3.46354 2.40513 3.72494 3.29354 2.67741 2.69355 4.24690 2.90347 2.73739 3.18146 2.89801 2.37887 2.77519 2.98518 4.58477 3.61503

0.02640 4.04331 4.76566 0.61958 0.77255 0.67416 0.71250

66 2.66178 4.56558 3.20940 1.86620 2.99521 3.54113 3.77270 2.94682 2.67526 2.55093 2.87081 3.16965 3.93551 3.01884 3.07633 2.79536 2.89740 2.75375 5.05245 3.74036 203 - -

2.68618 4.42225 2.77519 2.73123 3.46354 2.40513 3.72494 3.29354 2.67741 2.69355 4.24690 2.90347 2.73739 3.18146 2.89801 2.37887 2.77519 2.98518 4.58477 3.61503

0.02640 4.04331 4.76566 0.61958 0.77255 0.59279 0.80471

67 2.89707 4.97316 1.81066 2.53559 3.35499 3.47942 3.73401 3.61685 2.83005 3.19066 4.13352 3.04472 3.98892 3.09356 3.28597 2.91120 3.16091 3.33673 4.87476 1.75105 204 - -

2.68618 4.42225 2.77519 2.73123 3.46354 2.40513 3.72494 3.29354 2.67741 2.69355 4.24690 2.90347 2.73739 3.18146 2.89801 2.37887 2.77519 2.98518 4.58477 3.61503

0.02517 4.09033 4.81268 0.61958 0.77255 0.63000 0.76055

68 2.06837 3.33433 3.08342 1.75629 3.98929 3.34244 3.79896 3.28911 2.64828 3.01331 3.87423 3.08784 3.84255 2.99628 3.06480 2.65545 2.85237 2.97693 5.32285 4.02915 205 - -

2.68618 4.42225 2.77519 2.73123 3.46354 2.40513 3.72494 3.29354 2.67741 2.69355 4.24690 2.90347 2.73739 3.18146 2.89801 2.37887 2.77519 2.98518 4.58477 3.61503

0.02517 4.09033 4.81268 0.61958 0.77255 0.63000 0.76055

69 2.60827 4.56604 3.19359 2.62723 3.42266 3.49151 3.69945 2.76724 2.53200 2.78775 3.65413 2.86531 3.87684 2.72118 2.12512 2.72024 2.83792 2.66447 5.09832 3.81705 206 - -

2.68618 4.42225 2.77519 2.73123 3.46354 2.40513 3.72494 3.29354 2.67741 2.69355 4.24690 2.90347 2.73739 3.18146 2.89801 2.37887 2.77519 2.98518 4.58477 3.61503

0.02517 4.09033 4.81268 0.61958 0.77255 0.63000 0.76055

70 2.79965 5.01644 3.17715 2.58817 4.39937 3.52699 3.63038 3.78991 1.64078 3.30727 4.12906 3.06137 3.92010 2.76279 1.92048 2.57529 2.60052 3.44308 5.44266 4.17475 207 - -

2.68618 4.42225 2.77519 2.73123 3.46354 2.40513 3.72494 3.29354 2.67741 2.69355 4.24690 2.90347 2.73739 3.18146 2.89801 2.37887 2.77519 2.98518 4.58477 3.61503

0.17802 4.09033 1.92183 0.61958 0.77255 0.63000 0.76055

71 2.79891 5.30483 1.94684 1.66560 4.60876 3.23138 3.67902 4.10315 2.60524 3.61072 4.42484 2.43563 3.79331 2.82776 3.15728 2.31751 3.06864 3.68706 5.77896 4.31745 208 - -

2.68618 4.42225 2.77519 2.73123 3.46354 2.40513 3.72494 3.29354 2.67741 2.69355 4.24690 2.90347 2.73739 3.18146 2.89801 2.37887 2.77519 2.98518 4.58477 3.61503

0.02927 3.94159 4.66393 0.61958 0.77255 0.53019 0.88793

72 2.59889 4.67309 2.77661 2.32116 4.35979 1.40818 3.93365 3.75105 2.81976 3.40443 4.25733 3.03642 3.84115 3.13691 3.23138 2.67821 2.58721 3.34069 5.63991 4.32930 209 - -

2.68618 4.42225 2.77519 2.73123 3.46354 2.40513 3.72494 3.29354 2.67741 2.69355 4.24690 2.90347 2.73739 3.18146 2.89801 2.37887 2.77519 2.98518 4.58477 3.61503

0.02517 4.09033 4.81268 0.61958 0.77255 0.63000 0.76055

73 2.56138 4.72959 2.92140 2.50146 4.23449 2.34613 3.74292 3.66450 2.54145 3.25561 4.06528 2.21283 3.80413 2.90110 2.76516 2.48223 2.37377 3.27539 5.47243 4.14365 210 - -

2.68618 4.42225 2.77519 2.73123 3.46354 2.40513 3.72494 3.29354 2.67741 2.69355 4.24690 2.90347 2.73739 3.18146 2.89801 2.37887 2.77519 2.98518 4.58477 3.61503

0.02517 4.09033 4.81268 0.61958 0.77255 0.63000 0.76055

74 2.76475 5.03517 2.91353 2.46475 4.38442 2.26113 3.64885 3.80513 2.12027 3.33349 4.14880 2.97228 3.88235 2.01585 2.46480 2.75932 2.99488 3.44693 5.48922 4.17444 211 - -

2.68618 4.42225 2.77519 2.73123 3.46354 2.40513 3.72494 3.29354 2.67741 2.69355 4.24690 2.90347 2.73739 3.18146 2.89801 2.37887 2.77519 2.98518 4.58477 3.61503

0.02517 4.09033 4.81268 0.61958 0.77255 0.63000 0.76055

75 2.34235 3.18086 3.47567 2.91117 3.43517 3.17894 3.82324 2.64745 2.86045 2.37081 3.07614 2.74791 3.92821 3.16226 3.20757 2.58350 2.80094 2.55948 4.88279 3.65101 212 - -

2.68618 4.42225 2.77519 2.73123 3.46354 2.40513 3.72494 3.29354 2.67741 2.69355 4.24690 2.90347 2.73739 3.18146 2.89801 2.37887 2.77519 2.98518 4.58477 3.61503
0.07343 4.09033 2.91762 0.61958 0.77255 0.63000 0.76055
76 2.79551 0.65115 4.35001 4.13086 4.08655 3.37964 4.74071 3.26180 3.95987 3.16810 4.29606 4.07264 4.09973 4.29913 4.05286 3.06703 3.27689 2.99405 5.46304 4.37218 213 - -
2.68618 4.42225 2.77519 2.73123 3.46354 2.40513 3.72494 3.29354 2.67741 2.69355 4.24690 2.90347 2.73739 3.18146 2.89801 2.37887 2.77519 2.98518 4.58477 3.61503
0.02640 4.04331 4.76566 0.61958 0.77255 0.67416 0.71250
77 2.68553 4.53466 3.41090 3.11052 3.87112 3.35304 4.13961 3.27720 3.02750 2.55710 4.01216 3.43642 1.15535 3.45244 3.31680 2.85526 3.07492 3.02509 5.32549 4.03187 214 - -
2.68618 4.42225 2.77519 2.73123 3.46354 2.40513 3.72494 3.29354 2.67741 2.69355 4.24690 2.90347 2.73739 3.18146 2.89801 2.37887 2.77519 2.98518 4.58477 3.61503
0.02640 4.04331 4.76566 0.61958 0.77255 0.67416 0.71250
78 2.72419 4.92546 2.87190 2.48451 4.32309 2.31174 3.69241 3.76372 2.34555 3.31462 4.14564 2.97976 3.86573 1.84295 2.51794 2.74115 2.98562 3.40177 5.47932 4.16473 215 - -
2.68618 4.42225 2.77519 2.73123 3.46354 2.40513 3.72494 3.29354 2.67741 2.69355 4.24690 2.90347 2.73739 3.18146 2.89801 2.37887 2.77519 2.98518 4.58477 3.61503
0.02640 4.04331 4.76566 0.61958 0.77255 0.67416 0.71250
79 2.79551 0.65115 4.35001 4.13086 4.08655 3.37964 4.74071 3.26180 3.95987 3.16810 4.29606 4.07264 4.09973 4.29913 4.05286 3.06703 3.27689 2.99405 5.46304 4.37218 216 - -
2.68618 4.42225 2.77519 2.73123 3.46354 2.40513 3.72494 3.29354 2.67741 2.69355 4.24690 2.90347 2.73739 3.18146 2.89801 2.37887 2.77519 2.98518 4.58477 3.61503
0.02640 4.04331 4.76566 0.61958 0.77255 0.67416 0.71250
80 2.79132 5.02493 2.84218 2.48341 4.46696 2.84969 3.68969 3.89499 1.38946 3.41946 4.24920 2.65942 3.88973 2.83643 2.60895 2.79021 3.04713 3.51943 5.54885 4.24414 217 - -
2.68618 4.42225 2.77519 2.73123 3.46354 2.40513 3.72494 3.29354 2.67741 2.69355 4.24690 2.90347 2.73739 3.18146 2.89801 2.37887 2.77519 2.98518 4.58477 3.61503
0.02640 4.04331 4.76566 0.61958 0.77255 0.67416 0.71250
81 2.51539 4.87006 2.70266 1.88032 4.25036 3.31711 3.71441 3.65097 2.53923 3.26352 4.09115 2.70006 3.82246 2.87613 2.99196 2.67706 1.99681 3.29552 5.49368 4.14378 218 - -
2.68618 4.42225 2.77519 2.73123 3.46354 2.40513 3.72494 3.29354 2.67741 2.69355 4.24690 2.90347 2.73739 3.18146 2.89801 2.37887 2.77519 2.98518 4.58477 3.61503
0.02640 4.04331 4.76566 0.61958 0.77255 0.67416 0.71250
82 2.72134 4.85871 3.13246 2.56794 4.14134 3.49052 3.63739 3.54596 2.22854 2.82580 3.96032 3.04612 2.64310 2.57712 1.92440 2.75836 2.94369 3.23473 5.32190 4.03774 219 - -
2.68618 4.42225 2.77519 2.73123 3.46354 2.40513 3.72494 3.29354 2.67741 2.69355 4.24690 2.90347 2.73739 3.18146 2.89801 2.37887 2.77519 2.98518 4.58477 3.61503
0.02640 4.04331 4.76566 0.61958 0.77255 0.67416 0.71250
83 3.63957 4.90401 4.56245 4.24410 1.63983 4.37416 3.49193 3.35368 4.09615 2.69153 3.95904 4.05424 4.69441 4.10270 4.15921 3.74745 3.86626 3.27498 3.64233 0.91913 220 - -
2.68618 4.42225 2.77519 2.73123 3.46354 2.40513 3.72494 3.29354 2.67741 2.69355 4.24690 2.90347 2.73739 3.18146 2.89801 2.37887 2.77519 2.98518 4.58477 3.61503
0.02640 4.04331 4.76566 0.61958 0.77255 0.67416 0.71250
84 2.84014 5.12756 2.80006 2.51966 4.50553 3.51041 3.61102 3.91123 1.54955 3.39318 4.21123 3.00929 3.91305 2.73748 1.85260 2.81980 3.04663 3.55018 5.49515 4.21444 221 - -
2.68618 4.42225 2.77519 2.73123 3.46354 2.40513 3.72494 3.29354 2.67741 2.69355 4.24690 2.90347 2.73739 3.18146 2.89801 2.37887 2.77519 2.98518 4.58477 3.61503
0.02640 4.04331 4.76566 0.61958 0.77255 0.67416 0.71250
85 2.64274 4.97139 2.54491 2.26126 4.26048 3.38951 3.60108 3.29230 2.33522 3.24726 4.04068 2.67577 3.80336 2.73005 2.02417 2.63547 2.87452 3.33315 5.43403 4.07457 222 - -
2.68618 4.42225 2.77519 2.73123 3.46354 2.40513 3.72494 3.29354 2.67741 2.69355 4.24690 2.90347 2.73739 3.18146 2.89801 2.37887 2.77519 2.98518 4.58477 3.61503
0.02640 4.04331 4.76566 0.61958 0.77255 0.67416 0.71250
86 2.57503 4.30710 3.43317 2.86781 3.44533 3.20454 3.00894 2.32516 2.80803 2.28235 3.15160 3.06370 3.92776 3.12375 3.16077 2.79271 2.61909 2.57602 4.88728 3.64950 223 - -
2.68618 4.42225 2.77519 2.73123 3.46354 2.40513 3.72494 3.29354 2.67741 2.69355 4.24690 2.90347 2.73739 3.18146 2.89801 2.37887 2.77519 2.98518 4.58477 3.61503
0.02540 4.08160 4.80395 0.61958 0.77255 0.67416 0.71250

87 2.63524 5.04594 2.47919 2.32367 4.36200 3.37963 3.59025 3.81954 1.95927 3.33651 4.10705 2.48463 3.79162 2.70531 2.57404 2.48756 2.86810 3.18244 5.49758 4.11510 224 - -
2.68618 4.42225 2.77519 2.73123 3.46354 2.40513 3.72494 3.29354 2.67741 2.69355 4.24690 2.90347 2.73739 3.18146 2.89801 2.37887 2.77519 2.98518 4.58477 3.61503
0.06134 4.08160 3.15558 0.61958 0.77255 0.67416 0.71250
88 2.60646 4.63849 2.75311 2.56023 3.84380 2.34572 3.69738 3.22955 2.54335 2.53185 3.05703 3.05271 3.86107 2.89244 2.80349 2.69281 2.84289 2.94935 5.18214 3.88406 225 - -
2.68618 4.42225 2.77519 2.73123 3.46354 2.40513 3.72494 3.29354 2.67741 2.69355 4.24690 2.90347 2.73739 3.18146 2.89801 2.37887 2.77519 2.98518 4.58477 3.61503
0.02401 4.13727 4.85962 0.61958 0.77255 0.70574 0.68071
89 2.01606 4.76268 2.18950 2.49124 4.40222 3.23423 3.85206 3.83546 2.74179 3.44029 4.25341 2.97740 3.82048 3.02057 3.21808 1.64515 2.93182 3.40262 5.65287 4.29926 226 - -
2.68618 4.42225 2.77519 2.73123 3.46354 2.40513 3.72494 3.29354 2.67741 2.69355 4.24690 2.90347 2.73739 3.18146 2.89801 2.37887 2.77519 2.98518 4.58477 3.61503
0.02401 4.13727 4.85962 0.61958 0.77255 0.70574 0.68071
90 2.82000 5.14454 2.09619 2.31617 4.50516 3.32105 3.79631 3.98660 2.69398 3.54213 4.37896 2.86649 1.60979 2.69115 3.19274 2.79021 3.10765 3.59894 5.72690 4.32084 227 - -
2.68618 4.42225 2.77519 2.73123 3.46354 2.40513 3.72494 3.29354 2.67741 2.69355 4.24690 2.90347 2.73739 3.18146 2.89801 2.37887 2.77519 2.98518 4.58477 3.61503
0.02401 4.13727 4.85962 0.61958 0.77255 0.70574 0.68071
91 2.53354 5.01103 2.70818 2.18535 3.69301 3.40226 3.59725 3.76233 2.15149 3.29315 4.06727 2.74079 3.80143 2.71412 2.14758 2.61411 2.85919 3.37843 5.46655 4.09201 228 - -
2.68618 4.42225 2.77519 2.73123 3.46354 2.40513 3.72494 3.29354 2.67741 2.69355 4.24690 2.90347 2.73739 3.18146 2.89801 2.37887 2.77519 2.98518 4.58477 3.61503
0.02401 4.13727 4.85962 0.61958 0.77255 0.70574 0.68071
92 2.45886 4.17103 3.87663 3.04599 2.89234 3.71266 4.01288 2.20995 3.21838 2.24639 3.27797 3.60169 4.08285 3.47892 3.47617 2.98893 2.40138 1.84623 4.82164 3.61602 229 - -
2.68618 4.42225 2.77519 2.73123 3.46354 2.40513 3.72494 3.29354 2.67741 2.69355 4.24690 2.90347 2.73739 3.18146 2.89801 2.37887 2.77519 2.98518 4.58477 3.61503
0.15777 4.13727 2.04029 0.61958 0.77255 0.70574 0.68071
93 2.50407 5.19146 2.15065 1.61117 4.51059 3.28040 3.67448 3.97967 2.55140 3.50832 4.31187 2.76606 2.96639 2.81613 3.07685 2.69459 3.01096 3.57762 5.68694 4.25810 230 - -
2.68618 4.42225 2.77519 2.73123 3.46354 2.40513 3.72494 3.29354 2.67741 2.69355 4.24690 2.90347 2.73739 3.18146 2.89801 2.37887 2.77519 2.98518 4.58477 3.61503
0.02739 4.00689 4.72924 0.61958 0.77255 0.81615 0.58363
94 2.70183 5.05548 2.33257 2.02828 4.37313 2.12265 3.67110 3.82584 2.53365 3.38247 4.19196 2.55131 3.80210 2.81847 3.03923 2.67507 2.96335 3.15815 5.58263 4.18481 231 - -
2.68618 4.42225 2.77519 2.73123 3.46354 2.40513 3.72494 3.29354 2.67741 2.69355 4.24690 2.90347 2.73739 3.18146 2.89801 2.37887 2.77519 2.98518 4.58477 3.61503
0.02739 4.00689 4.72924 0.61958 0.77255 0.81615 0.58363
95 2.77671 5.23512 1.64987 1.95934 4.48940 3.28641 3.67397 3.98969 2.56377 3.51447 4.32358 2.49537 3.81242 2.81998 3.09451 2.71403 3.03371 3.59538 5.68070 3.43747 232 - -
2.68618 4.42225 2.77519 2.73123 3.46354 2.40513 3.72494 3.29354 2.67741 2.69355 4.24690 2.90347 2.73739 3.18146 2.89801 2.37887 2.77519 2.98518 4.58477 3.61503
0.02739 4.00689 4.72924 0.61958 0.77255 0.81615 0.58363
96 2.78153 5.28437 2.04449 1.64703 4.58836 3.28517 3.66563 4.07402 2.20031 3.57368 4.37150 2.74959 3.80960 2.80247 3.04597 2.47362 3.03754 3.65886 5.73143 4.28983 233 - -
2.68618 4.42225 2.77519 2.73123 3.46354 2.40513 3.72494 3.29354 2.67741 2.69355 4.24690 2.90347 2.73739 3.18146 2.89801 2.37887 2.77519 2.98518 4.58477 3.61503
0.02739 4.00689 4.72924 0.61958 0.77255 0.81615 0.58363
97 2.77617 5.28481 2.07555 1.63443 4.58528 3.28627 3.66081 4.07296 2.38895 3.57000 4.36507 2.74830 3.80695 2.52950 3.04313 2.47219 3.03070 3.65660 5.72705 4.28448 234 - -
2.68624 4.42231 2.77525 2.73075 3.46359 2.40510 3.72500 3.29360 2.67746 2.69360 4.24695 2.90339 2.73745 3.18133 2.89806 2.37892 2.77525 2.98524 4.58483 3.61509
0.52079 0.92352 4.72924 0.16660 1.87431 0.81615 0.58363
98 2.51035 5.20671 1.83133 1.98433 4.51238 3.29783 3.64573 3.98985 2.48669 3.49959 4.29050 2.53471 3.79857 2.77835 2.69966 2.67784 2.98661 3.58190 5.66361 4.23678 236 - -
2.68610 4.42235 2.77469 2.73109 3.46363 2.40504 3.72504 3.29364 2.67750 2.69364 4.24644 2.90356 2.73737 3.18156 2.89810 2.37896 2.77529 2.98528 4.58486 3.61513

0.56562 0.86000 4.72924 0.48561 0.95535 0.67836 0.70816

99 2.57927 4.21505 3.60792 3.03747 3.10787 3.22274 3.86628 2.27652 2.64627 2.09225 3.31839 3.39864 3.47250 3.25771 3.27919 2.85460 2.81209 2.28436 4.81403 3.59443 239 - -

2.68618 4.42225 2.77519 2.73123 3.46354 2.40513 3.72494 3.29354 2.67741 2.69355 4.24690 2.90347 2.73739 3.18146 2.89801 2.37887 2.77519 2.98518 4.58477 3.61503

0.02517 4.09033 4.81268 0.61958 0.77255 0.59177 0.80597

100 2.67749 5.36060 1.51096 2.10413 4.68125 3.33002 3.76501 4.17146 2.67832 3.68018 4.48692 2.63261 2.45819 2.90981 3.22696 2.79263 3.13477 3.75268 5.84858 4.39363 240 - -

2.68651 4.42258 2.77421 2.73036 3.46265 2.40541 3.72511 3.29349 2.67766 2.69355 4.24723 2.90316 2.73773 3.18180 2.89834 2.37906 2.77527 2.98531 4.58510 3.61504

0.33613 1.28003 4.90070 1.74965 0.19096 0.66488 0.72224

101 2.59551 4.18178 3.73164 3.15767 2.77896 3.25677 3.64330 2.40523 2.90032 2.09330 3.29131 3.48213 3.48295 3.35001 3.35926 2.90025 2.82733 2.44376 4.76136 2.65564 249 - -

2.68618 4.42225 2.77519 2.73123 3.46354 2.40513 3.72494 3.29354 2.67741 2.69355 4.24690 2.90347 2.73739 3.18146 2.89801 2.37887 2.77519 2.98518 4.58477 3.61503

0.02303 4.17835 4.90070 0.61958 0.77255 0.66488 0.72224

102 2.53893 4.92872 2.88998 2.32099 4.23265 2.97097 3.64945 3.66840 2.30234 3.23507 4.03006 2.92942 2.47626 2.63881 2.89378 2.39718 2.62357 3.30722 5.44182 4.08356 250 - -

2.68618 4.42225 2.77519 2.73123 3.46354 2.40513 3.72494 3.29354 2.67741 2.69355 4.24690 2.90347 2.73739 3.18146 2.89801 2.37887 2.77519 2.98518 4.58477 3.61503

0.11319 4.17835 2.38928 0.61958 0.77255 0.66488 0.72224

103 2.34092 4.80231 3.00944 2.45030 4.04420 3.43319 3.38557 3.45906 2.26896 3.05808 3.43158 2.86196 3.82217 2.20633 2.73301 2.64493 2.83929 3.13807 5.29437 3.50398 251 - -

2.68615 4.42229 2.77524 2.73120 3.46358 2.40506 3.72499 3.29358 2.67745 2.69359 4.24694 2.90342 2.73744 3.18121 2.89791 2.37891 2.77524 2.98523 4.58481 3.61507

0.21135 1.90876 3.16431 0.66326 0.72395 0.74860 0.64060

104 2.60704 4.90828 2.40652 2.35140 4.18465 3.06663 3.60962 3.62083 2.31804 3.19007 3.01119 2.57008 3.37630 2.74411 2.87733 2.53612 2.84407 3.26486 5.40084 4.03809 254 - -

2.68618 4.42225 2.77519 2.73123 3.46354 2.40513 3.72494 3.29354 2.67741 2.69355 4.24690 2.90347 2.73739 3.18146 2.89801 2.37887 2.77519 2.98518 4.58477 3.61503

0.05833 4.05562 3.23565 0.61958 0.77255 0.71689 0.66995

105 2.57821 4.71252 2.87276 2.52811 4.28493 1.81783 3.78566 3.71225 2.18703 3.30657 4.12872 2.99807 3.18304 2.95589 2.97409 2.45730 2.90100 3.31324 5.51721 4.20145 255 - -

2.68609 4.42230 2.77524 2.73098 3.46359 2.40517 3.72479 3.29349 2.67745 2.69354 4.24694 2.90351 2.73744 3.18151 2.89805 2.37883 2.77524 2.98523 4.58482 3.61508

0.40015 1.13506 4.78236 0.18752 1.76615 0.72069 0.66634

106 2.61956 4.95619 2.91113 2.24207 4.24563 3.24896 3.24255 3.39627 2.15657 3.23209 4.01695 2.71384 3.79938 2.71670 2.57515 2.17312 2.84895 3.31774 5.41906 4.05866 257 - -

2.68618 4.42225 2.77519 2.73123 3.46354 2.40513 3.72494 3.29354 2.67741 2.69355 4.24690 2.90347 2.73739 3.18146 2.89801 2.37887 2.77519 2.98518 4.58477 3.61503

0.02517 4.09033 4.81268 0.61958 0.77255 0.74860 0.64060

107 2.51729 4.69332 2.84366 2.48975 3.66827 3.43841 3.22420 3.04774 2.02979 2.81683 3.77225 2.99596 3.82738 2.82126 2.91784 2.57763 2.81849 2.76029 5.21242 3.89878 258 - -

2.68618 4.42225 2.77519 2.73123 3.46354 2.40513 3.72494 3.29354 2.67741 2.69355 4.24690 2.90347 2.73739 3.18146 2.89801 2.37887 2.77519 2.98518 4.58477 3.61503

0.07269 4.09033 2.93041 0.61958 0.77255 0.65599 0.73174

108 2.51269 4.57928 3.01281 2.50557 3.76830 3.46491 3.68626 2.99705 2.55317 2.42304 3.29288 3.06073 3.30867 2.61424 2.98358 2.58499 2.54386 2.88173 5.12045 3.83133 259 - -

2.68618 4.42225 2.77519 2.73123 3.46354 2.40513 3.72494 3.29354 2.67741 2.69355 4.24690 2.90347 2.73739 3.18146 2.89801 2.37887 2.77519 2.98518 4.58477 3.61503

0.02499 4.09764 4.81999 0.61958 0.77255 0.66198 0.72532

109 2.54562 5.09481 2.25380 2.09661 4.41162 3.37930 3.08036 3.88095 2.35899 3.38686 4.14772 2.85496 3.79534 2.62939 2.57251 2.51423 2.65427 3.47116 5.54153 4.14223 260 - -

2.68618 4.42225 2.77519 2.73123 3.46354 2.40513 3.72494 3.29354 2.67741 2.69355 4.24690 2.90347 2.73739 3.18146 2.89801 2.37887 2.77519 2.98518 4.58477 3.61503

0.04547 4.14158 3.55590 0.61958 0.77255 0.70161 0.68475

110 2.48583 4.97272 2.94302 2.24174 4.26763 3.12981 3.37030 3.70546 2.14541 3.24720 4.03198 2.91730 3.81254 2.45950 2.36259 2.63070 2.86286 2.91681 5.42813 4.07260 261 - -

2.68618 4.42225 2.77519 2.73123 3.46354 2.40513 3.72494 3.29354 2.67741 2.69355 4.24690 2.90347 2.73739 3.18146 2.89801 2.37887 2.77519 2.98518 4.58477 3.61503
0.05893 4.12052 3.19450 0.61958 0.77255 0.72148 0.66559
111 2.22266 4.80393 2.99010 2.51122 4.24853 3.11776 3.69477 3.66943 2.39883 3.24392 4.05554 2.52280 3.83038 2.84368 1.93058 2.55342 2.89135 3.29752 5.44176 4.12585 262 - -
2.68618 4.42225 2.77519 2.73123 3.46354 2.40513 3.72494 3.29354 2.67741 2.69355 4.24690 2.90347 2.73739 3.18146 2.89801 2.37887 2.77519 2.98518 4.58477 3.61503
0.07232 4.08686 2.93798 0.61958 0.77255 0.75163 0.63790
112 2.73481 4.27807 3.90968 2.77236 2.89095 3.80683 4.07994 2.24854 3.25140 1.56944 2.76042 3.66398 4.15077 3.50886 3.51732 3.08178 2.96443 2.33626 4.86157 3.67200 263 - -
2.68618 4.42225 2.77519 2.73123 3.46354 2.40513 3.72494 3.29354 2.67741 2.69355 4.24690 2.90347 2.73739 3.18146 2.89801 2.37887 2.77519 2.98518 4.58477 3.61503
0.13799 4.04101 2.19540 0.61958 0.77255 0.57753 0.82390
113 2.61825 4.92856 2.61335 2.12352 4.19671 3.22337 3.61120 3.63516 2.39996 2.88380 3.99671 2.88502 3.79611 2.60528 2.87745 2.10679 2.85321 3.27910 5.40981 3.73991 264 - -
2.68618 4.42225 2.77519 2.73123 3.46354 2.40513 3.72494 3.29354 2.67741 2.69355 4.24690 2.90347 2.73739 3.18146 2.89801 2.37887 2.77519 2.98518 4.58477 3.61503
0.07411 4.07232 2.91160 0.61958 0.77255 0.76406 0.62693
114 2.62019 4.08431 4.07063 3.49509 2.62463 3.37762 3.90123 2.51345 3.36900 2.02196 3.18166 3.67653 4.07506 3.57670 3.54002 3.00070 2.85168 1.99894 3.12845 2.80050 265 - -
2.68618 4.42225 2.77519 2.73123 3.46354 2.40513 3.72494 3.29354 2.67741 2.69355 4.24690 2.90347 2.73739 3.18146 2.89801 2.37887 2.77519 2.98518 4.58477 3.61503
0.02689 4.02511 4.74746 0.61958 0.77255 0.72198 0.66512
115 2.56440 4.13870 3.76488 3.19376 3.24048 3.33450 3.90537 2.60392 3.09358 2.33778 2.54154 3.49289 3.99038 3.36852 3.35475 2.53691 2.80476 2.19409 2.91492 3.51259 266 - -
2.68602 4.42231 2.77525 2.73129 3.46360 2.40519 3.72480 3.29360 2.67728 2.69361 4.24696 2.90341 2.73745 3.18152 2.89792 2.37878 2.77525 2.98524 4.58483 3.61509
0.62542 0.95920 2.50391 0.16285 1.89523 0.64815 0.74026
116 2.58605 4.57204 3.14510 2.58372 3.54483 3.46689 3.68361 3.01535 2.09134 2.52678 3.41024 2.85045 3.85399 2.89173 2.95230 2.36511 2.81737 2.86913 5.11007 3.82247 268 - -
2.68618 4.42225 2.77519 2.73123 3.46354 2.40513 3.72494 3.29354 2.67741 2.69355 4.24690 2.90347 2.73739 3.18146 2.89801 2.37887 2.77519 2.98518 4.58477 3.61503
0.08505 4.06662 2.74266 0.61958 0.77255 0.76885 0.62277
117 2.57677 4.77364 2.76559 2.41906 4.26143 2.95367 3.72029 3.69964 2.54083 3.28353 4.09614 2.23981 3.78508 2.65372 2.99056 1.95547 2.69292 3.30828 5.49533 4.15131 269 - -
2.68618 4.42225 2.77519 2.73123 3.46354 2.40513 3.72494 3.29354 2.67741 2.69355 4.24690 2.90347 2.73739 3.18146 2.89801 2.37887 2.77519 2.98518 4.58477 3.61503
0.02734 4.00891 4.73126 0.61958 0.77255 0.75358 0.63616
118 2.64632 4.80424 3.02601 2.49575 4.06702 3.43350 3.63871 3.49250 2.34154 3.08850 3.91667 2.43345 3.84286 2.79713 2.15857 2.68543 2.36190 3.17194 5.30191 3.60447 270 - -
2.68618 4.42225 2.77519 2.73123 3.46354 2.40513 3.72494 3.29354 2.67741 2.69355 4.24690 2.90347 2.73739 3.18146 2.89801 2.37887 2.77519 2.98518 4.58477 3.61503
0.08979 4.04525 2.68282 0.61958 0.77255 0.51041 0.91691
119 2.48714 5.03777 2.63673 2.27264 4.34710 2.84439 3.59436 3.80651 2.00401 3.32640 3.60700 2.62362 3.79542 2.70612 2.66052 2.60231 2.85446 3.40965 5.49155 4.10568 271 - -
2.68627 4.42234 2.77528 2.73132 3.46363 2.40494 3.72503 3.29363 2.67742 2.69364 4.24698 2.90309 2.73748 3.18155 2.89775 2.37888 2.77528 2.98527 4.58486 3.61484
0.63631 1.32161 1.58937 0.67470 0.71194 0.58432 0.81528
120 2.60822 4.90109 2.78971 2.19839 4.19462 3.03949 3.14140 3.63106 2.40470 3.20002 3.99928 2.56695 3.47457 2.75071 2.87639 2.45075 2.40311 3.27294 5.40997 4.04850 274 - -
2.68618 4.42225 2.77519 2.73123 3.46354 2.40513 3.72494 3.29354 2.67741 2.69355 4.24690 2.90347 2.73739 3.18146 2.89801 2.37887 2.77519 2.98518 4.58477 3.61503
0.07879 4.00447 2.85543 0.61958 0.77255 0.81797 0.58219
121 2.57871 4.84235 2.81630 2.02078 3.81230 3.38618 3.30136 3.52818 2.35761 3.10806 3.54697 2.90067 3.78006 2.72309 2.50184 2.59437 2.67972 3.18617 5.32708 3.97860 275 - -
2.68623 4.42238 2.77505 2.73108 3.46367 2.40496 3.72508 3.29367 2.67739 2.69368 4.24668 2.90335 2.73738 3.18151 2.89806 2.37900 2.77509 2.98524 4.58490 3.61516
0.51418 1.03416 3.06866 0.90499 0.51843 0.46795 0.98425

122 2.62096 4.75527 2.86412 2.50104 3.72492 3.45253 3.68023 3.39676 2.50549 2.91310 3.84677 2.87804 2.99019 2.84939 2.95994 1.87560 2.85737 3.08872 5.27241 3.49798 279 - -
2.68627 4.42288 2.77521 2.73131 3.46363 2.40547 3.72500 3.29374 2.67755 2.69368 4.24606 2.90310 2.73669 3.18141 2.89855 2.37871 2.77573 2.98429 4.58481 3.61476
0.49556 0.95822 4.93466 2.05545 0.13701 0.66299 0.72425
123 2.60144 4.18231 3.75408 3.17856 2.88666 3.25759 3.92379 2.54843 3.09975 1.96052 2.65627 3.49981 3.66876 3.07445 3.17172 2.91128 2.68104 2.36431 4.78164 3.57266 292 - -
2.68618 4.42225 2.77519 2.73123 3.46354 2.40513 3.72494 3.29354 2.67741 2.69355 4.24690 2.90347 2.73739 3.18146 2.89801 2.37887 2.77519 2.98518 4.58477 3.61503
0.06407 4.21231 3.05239 0.61958 0.77255 0.66299 0.72425
124 2.79279 4.31968 4.01112 3.43526 3.31929 3.87367 4.15945 2.39714 2.78340 1.49597 2.91565 3.74685 4.22287 3.58540 3.52473 3.15987 3.02533 1.86437 4.95503 3.76866 293 - -
2.68624 4.42231 2.77526 2.73129 3.46360 2.40519 3.72461 3.29347 2.67747 2.69361 4.24696 2.90353 2.73746 3.18153 2.89807 2.37888 2.77478 2.98525 4.58483 3.61453
0.45468 1.57612 1.84153 0.62027 0.77176 0.70512 0.68132
125 2.25121 3.67540 3.31510 2.75114 3.53902 2.84595 3.74681 2.90019 2.52830 2.44737 3.13351 3.18488 3.87914 3.02595 2.90880 2.72883 2.78989 2.65855 4.95255 3.70280 296 - -
2.68618 4.42225 2.77519 2.73123 3.46354 2.40513 3.72494 3.29354 2.67741 2.69355 4.24690 2.90347 2.73739 3.18146 2.89801 2.37887 2.77519 2.98518 4.58477 3.61503
0.02737 4.00764 4.72999 0.61958 0.77255 0.78329 0.61046
126 2.63745 5.06253 2.51781 2.24721 4.37764 3.16404 3.14162 3.83836 2.31861 3.34908 4.11867 2.84770 3.78452 2.35511 2.31308 2.49154 2.86980 3.43886 5.50547 4.11928 297 - -
2.68618 4.42225 2.77519 2.73123 3.46354 2.40513 3.72494 3.29354 2.67741 2.69355 4.24690 2.90347 2.73739 3.18146 2.89801 2.37887 2.77519 2.98518 4.58477 3.61503
0.02638 4.04403 4.76638 0.61958 0.77255 0.81691 0.58303
127 2.66643 5.10418 2.51054 2.02319 4.41367 3.11184 3.61660 3.88222 2.42089 3.40036 4.17749 2.34879 3.46063 2.59113 2.92347 2.29783 2.76338 3.48024 5.56886 4.16200 298 - -
2.68618 4.42225 2.77519 2.73123 3.46354 2.40513 3.72494 3.29354 2.67741 2.69355 4.24690 2.90347 2.73739 3.18146 2.89801 2.37887 2.77519 2.98518 4.58477 3.61503
0.02638 4.04403 4.76638 0.61958 0.77255 0.72430 0.66294
128 2.64502 5.03028 2.33774 2.31831 4.32414 3.37323 3.35818 3.78024 2.38844 3.31608 4.09679 2.64102 3.79896 2.10842 2.87068 2.62531 2.74778 3.01865 5.49512 4.11006 299 - -
2.68618 4.42225 2.77519 2.73123 3.46354 2.40513 3.72494 3.29354 2.67741 2.69355 4.24690 2.90347 2.73739 3.18146 2.89801 2.37887 2.77519 2.98518 4.58477 3.61503
0.02499 4.09764 4.81999 0.61958 0.77255 0.77309 0.61912
129 2.08005 4.36600 3.33858 2.84548 3.90806 3.25132 3.91075 3.26248 2.82990 2.96754 3.82207 2.85068 3.81421 3.15163 3.21649 1.94187 2.31826 2.38918 5.27822 4.02275 300 - -
2.68618 4.42225 2.77519 2.73123 3.46354 2.40513 3.72494 3.29354 2.67741 2.69355 4.24690 2.90347 2.73739 3.18146 2.89801 2.37887 2.77519 2.98518 4.58477 3.61503
0.07999 4.09764 2.80899 0.61958 0.77255 0.77309 0.61912
130 2.83445 5.16402 2.11822 2.05096 4.58530 1.49954 3.81284 4.05732 2.78478 3.62233 4.47127 2.80331 3.85605 2.99012 3.31649 2.79314 3.14317 3.65478 5.80764 4.39033 301 - -
2.68618 4.42225 2.77519 2.73123 3.46354 2.40513 3.72494 3.29354 2.67741 2.69355 4.24690 2.90347 2.73739 3.18146 2.89801 2.37887 2.77519 2.98518 4.58477 3.61503
0.02638 4.04403 4.76638 0.61958 0.77255 0.55272 0.85657
131 2.66264 4.96597 2.30821 1.74663 4.22870 3.42320 3.65744 3.06001 2.44888 3.21849 3.51576 2.92715 3.83988 2.79181 2.92710 2.66485 2.89834 3.27595 5.45387 4.08776 302 - -
2.68618 4.42225 2.77519 2.73123 3.46354 2.40513 3.72494 3.29354 2.67741 2.69355 4.24690 2.90347 2.73739 3.18146 2.89801 2.37887 2.77519 2.98518 4.58477 3.61503
0.02247 4.20241 4.92475 0.61958 0.77255 0.59331 0.80406
132 2.66380 4.09291 4.27126 3.68717 2.04087 3.77995 3.56136 2.07499 3.53784 2.17039 3.20656 3.80679 4.14406 3.71977 3.65850 2.82696 2.89531 2.26490 3.72669 2.92359 303 - -
2.68618 4.42225 2.77519 2.73123 3.46354 2.40513 3.72494 3.29354 2.67741 2.69355 4.24690 2.90347 2.73739 3.18146 2.89801 2.37887 2.77519 2.98518 4.58477 3.61503
0.05200 4.24206 3.31604 0.61958 0.77255 0.63000 0.76055
133 2.69959 5.17650 2.03395 2.14301 4.49965 3.38586 3.64349 3.97731 2.42293 3.47449 4.23704 2.70159 3.28215 2.75708 2.47704 2.27212 2.93896 3.55822 5.62393 4.21150 304 - -
2.68613 4.42235 2.77529 2.73126 3.46364 2.40506 3.72504 3.29364 2.67751 2.69365 4.24700 2.90357 2.73690 3.18156 2.89811 2.37884 2.77504 2.98519 4.58487 3.61513

0.68544 1.19499 1.64286 0.62072 0.77123 0.60192 0.79353

134 2.33857 5.05957 1.69464 2.26287 4.51120 2.97967 3.73357 3.98322 2.63366 3.52484 4.33067 2.53910 3.80664 2.88418 3.15742 2.34414 2.99973 3.55986 5.71014 4.29550 307 - -

2.68618 4.42225 2.77519 2.73123 3.46354 2.40513 3.72494 3.29354 2.67741 2.69355 4.24690 2.90347 2.73739 3.18146 2.89801 2.37887 2.77519 2.98518 4.58477 3.61503

0.02642 4.04267 4.76501 0.61958 0.77255 0.81797 0.58219

135 2.58623 4.80145 2.96370 2.43575 4.12269 2.90688 3.31113 3.54528 2.38776 3.13053 3.93804 2.94517 3.20620 2.77600 2.34353 2.36175 2.56487 3.19845 5.35084 4.01803 308 - -

2.68616 4.42311 2.77532 2.73211 3.46340 2.40553 3.72348 3.29322 2.67761 2.69362 4.24784 2.90378 2.73646 3.18195 2.89721 2.37847 2.77555 2.98469 4.58603 3.61512

0.62179 0.78857 4.76501 2.72660 0.06768 0.48990 0.94852

136 2.72133 4.99056 2.93744 2.46684 4.27918 3.45881 2.16837 3.73061 2.40052 3.28765 4.09599 2.98594 2.92487 2.18541 2.81535 2.72704 2.73684 3.37689 5.46942 4.12223 345 - -

2.68618 4.42225 2.77519 2.73123 3.46354 2.40513 3.72494 3.29354 2.67741 2.69355 4.24690 2.90347 2.73739 3.18146 2.89801 2.37887 2.77519 2.98518 4.58477 3.61503

0.07471 4.24206 2.85414 0.61958 0.77255 0.63000 0.76055

137 2.52369 4.37160 3.40269 2.83898 3.25802 3.21847 3.80997 2.73595 2.80024 2.37163 3.47884 2.61686 3.48476 3.11173 3.17346 2.79501 2.48748 2.31070 4.95494 3.71075 346 - -

2.68618 4.42225 2.77519 2.73123 3.46354 2.40513 3.72494 3.29354 2.67741 2.69355 4.24690 2.90347 2.73739 3.18146 2.89801 2.37887 2.77519 2.98518 4.58477 3.61503

0.02276 4.19011 4.91246 0.61958 0.77255 0.68630 0.70004

138 2.65350 4.74044 3.24260 2.72097 4.28068 3.40179 3.79549 3.67778 2.41919 3.27177 4.10420 3.15062 3.90126 2.95982 1.61703 2.10152 2.45158 3.30719 5.46921 4.19741 347 - -

2.68618 4.42225 2.77519 2.73123 3.46354 2.40513 3.72494 3.29354 2.67741 2.69355 4.24690 2.90347 2.73739 3.18146 2.89801 2.37887 2.77519 2.98518 4.58477 3.61503

0.02276 4.19011 4.91246 0.61958 0.77255 0.68630 0.70004

139 2.60143 4.24821 3.62731 3.05611 3.37465 3.60561 3.88236 2.52139 2.96225 2.45774 3.36366 3.41437 3.30221 3.27021 3.03680 2.76728 2.69309 2.37812 2.32455 3.61732 348 - -

2.68618 4.42225 2.77519 2.73123 3.46354 2.40513 3.72494 3.29354 2.67741 2.69355 4.24690 2.90347 2.73739 3.18146 2.89801 2.37887 2.77519 2.98518 4.58477 3.61503

0.02276 4.19011 4.91246 0.61958 0.77255 0.68630 0.70004

140 3.23828 4.56952 4.90860 4.33173 2.84075 4.48770 4.79363 2.21971 4.20481 1.06289 2.39757 4.53558 4.70350 4.29133 4.29918 3.82117 3.46008 1.78210 5.13535 4.04793 349 - -

2.68624 4.42231 2.77511 2.73130 3.46360 2.40511 3.72501 3.29360 2.67747 2.69330 4.24696 2.90353 2.73746 3.18153 2.89807 2.37887 2.77526 2.98495 4.58483 3.61486

0.83819 0.99495 1.62067 0.15284 1.95383 0.63264 0.75755

141 2.59661 4.56231 3.11813 2.61243 3.79642 3.43493 3.70764 3.16320 2.36205 2.21843 3.70138 2.89848 2.71076 2.92189 2.90123 2.70570 2.67873 2.89055 5.14082 3.86385 351 - -

2.68618 4.42225 2.77519 2.73123 3.46354 2.40513 3.72494 3.29354 2.67741 2.69355 4.24690 2.90347 2.73739 3.18146 2.89801 2.37887 2.77519 2.98518 4.58477 3.61503

0.02726 4.01172 4.73407 0.61958 0.77255 0.51329 0.91261

142 2.61365 4.40214 3.09703 2.82267 2.50807 3.56268 3.80413 2.76816 2.78119 2.17028 3.49954 3.25519 3.94503 2.69105 2.99657 2.59092 2.84543 2.68009 4.96597 3.71295 352 - -

2.68618 4.42225 2.77519 2.73123 3.46354 2.40513 3.72494 3.29354 2.67741 2.69355 4.24690 2.90347 2.73739 3.18146 2.89801 2.37887 2.77519 2.98518 4.58477 3.61503

0.02215 4.21678 4.93913 0.61958 0.77255 0.65816 0.72941

143 2.67707 5.08193 2.72370 1.91907 3.73877 3.42306 3.62638 3.84649 1.75250 3.36525 4.13835 2.90602 3.83224 2.74232 2.69789 2.65420 2.90805 3.45471 5.52646 4.14688 353 - -

2.68618 4.42225 2.77519 2.73123 3.46354 2.40513 3.72494 3.29354 2.67741 2.69355 4.24690 2.90347 2.73739 3.18146 2.89801 2.37887 2.77519 2.98518 4.58477 3.61503

0.02215 4.21678 4.93913 0.61958 0.77255 0.65816 0.72941

144 2.27868 4.81757 2.46878 2.34332 3.65398 3.44139 3.66194 3.27294 2.46746 3.08565 3.89994 2.97947 3.84043 2.81373 2.93175 2.41908 2.34288 3.15335 5.33178 3.99632 354 - -

2.68618 4.42225 2.77519 2.73123 3.46354 2.40513 3.72494 3.29354 2.67741 2.69355 4.24690 2.90347 2.73739 3.18146 2.89801 2.37887 2.77519 2.98518 4.58477 3.61503

0.02215 4.21678 4.93913 0.61958 0.77255 0.65816 0.72941

145 2.49441 4.81695 3.03716 2.38766 4.05545 3.45992 2.89351 3.31092 2.08984 2.58110 3.88915 2.99360 3.84912 2.64963 2.88236 2.51977 2.86113 2.99851 5.31625 3.98864 355 - -

2.68618 4.42225 2.77519 2.73123 3.46354 2.40513 3.72494 3.29354 2.67741 2.69355 4.24690 2.90347 2.73739 3.18146 2.89801 2.37887 2.77519 2.98518 4.58477 3.61503
0.02215 4.21678 4.93913 0.61958 0.77255 0.60593 0.78870
146 2.44709 5.05209 2.77890 2.25645 4.35932 2.52022 3.61525 3.81823 2.30078 3.14322 4.10550 2.68915 3.81526 2.60615 2.52194 2.53448 2.78005 3.42276 5.50782 4.12209 356 - -
2.68589 4.42061 2.77530 2.73134 3.46365 2.40507 3.72505 3.29349 2.67751 2.69348 4.24700 2.90357 2.73750 3.18157 2.89811 2.37878 2.77530 2.98511 4.58488 3.61514
0.17690 1.86332 4.96441 1.17018 0.37152 0.63000 0.76055
147 2.61958 4.58445 3.19842 2.56569 3.44536 3.51096 3.73415 3.15083 2.47559 2.41280 3.67388 2.95193 3.59977 2.95051 2.83637 2.45986 2.33835 2.63049 5.13022 3.84784 361 - -
2.68618 4.42225 2.77519 2.73123 3.46354 2.40513 3.72494 3.29354 2.67741 2.69355 4.24690 2.90347 2.73739 3.18146 2.89801 2.37887 2.77519 2.98518 4.58477 3.61503
0.02159 4.24206 4.96441 0.61958 0.77255 0.63000 0.76055
148 2.63261 4.60966 2.77814 2.53041 3.78559 3.50480 3.73982 3.17844 2.62136 2.70021 3.70360 3.10909 3.90076 2.95545 3.04821 2.61119 2.53635 2.78368 5.14942 2.21545 362 - -
2.68618 4.42225 2.77519 2.73123 3.46354 2.40513 3.72494 3.29354 2.67741 2.69355 4.24690 2.90347 2.73739 3.18146 2.89801 2.37887 2.77519 2.98518 4.58477 3.61503
0.02159 4.24206 4.96441 0.61958 0.77255 0.63000 0.76055
149 2.50743 4.54907 3.19734 2.58726 3.81386 1.79697 3.83641 3.22368 2.73036 2.89707 3.76404 3.18316 3.92068 3.07920 3.12029 2.59409 2.89942 2.94194 3.10023 3.90988 363 - -
2.68618 4.42225 2.77519 2.73123 3.46354 2.40513 3.72494 3.29354 2.67741 2.69355 4.24690 2.90347 2.73739 3.18146 2.89801 2.37887 2.77519 2.98518 4.58477 3.61503
0.02159 4.24206 4.96441 0.61958 0.77255 0.63000 0.76055
150 2.70683 4.15577 4.21387 3.63412 2.78765 3.47982 4.05058 2.23549 3.50254 1.79278 3.20853 3.80753 4.17581 3.70342 3.65990 3.11075 2.93851 2.25801 4.62887 2.09685 364 - -
2.68618 4.42225 2.77519 2.73123 3.46354 2.40513 3.72494 3.29354 2.67741 2.69355 4.24690 2.90347 2.73739 3.18146 2.89801 2.37887 2.77519 2.98518 4.58477 3.61503
0.02159 4.24206 4.96441 0.61958 0.77255 0.63000 0.76055
151 2.10400 4.28196 3.66872 3.28082 4.25565 1.26813 4.28882 3.59850 3.27950 3.33429 4.17660 3.44791 3.83848 3.56780 3.60129 2.37724 2.49671 2.57468 5.62749 4.41766 365 - -
2.68618 4.42225 2.77519 2.73123 3.46354 2.40513 3.72494 3.29354 2.67741 2.69355 4.24690 2.90347 2.73739 3.18146 2.89801 2.37887 2.77519 2.98518 4.58477 3.61503
0.02159 4.24206 4.96441 0.61958 0.77255 0.63000 0.76055
152 2.77588 5.17921 2.41908 2.23095 4.52760 3.37284 3.74206 3.99523 2.58914 3.52610 4.32029 1.72023 3.87580 2.87907 3.09916 2.12931 2.71563 3.59208 5.70492 4.29432 366 - -
2.68618 4.42225 2.77519 2.73123 3.46354 2.40513 3.72494 3.29354 2.67741 2.69355 4.24690 2.90347 2.73739 3.18146 2.89801 2.37887 2.77519 2.98518 4.58477 3.61503
0.02159 4.24206 4.96441 0.61958 0.77255 0.63000 0.76055
153 1.56629 4.28411 3.64717 3.17346 3.88602 2.99215 4.12206 3.22051 3.14262 2.96094 3.83350 3.42448 3.36002 3.43399 3.47239 2.28784 2.82766 1.87729 5.30371 4.08438 367 - -
2.68618 4.42225 2.77519 2.73123 3.46354 2.40513 3.72494 3.29354 2.67741 2.69355 4.24690 2.90347 2.73739 3.18146 2.89801 2.37887 2.77519 2.98518 4.58477 3.61503
0.05347 4.24206 3.27853 0.61958 0.77255 0.63000 0.76055
154 2.26024 4.32244 3.10025 2.72726 2.55103 3.35140 3.83850 2.68209 2.87249 2.42461 3.42584 3.32247 3.96398 3.17378 3.22534 2.82979 2.83626 2.37530 4.90430 3.30536 368 - -
2.68618 4.42225 2.77519 2.73123 3.46354 2.40513 3.72494 3.29354 2.67741 2.69355 4.24690 2.90347 2.73739 3.18146 2.89801 2.37887 2.77519 2.98518 4.58477 3.61503
0.02228 4.21088 4.93323 0.61958 0.77255 0.66452 0.72262
155 3.50469 4.83898 4.64757 4.22940 2.09025 4.37874 3.76041 3.09232 4.01327 2.20489 3.69682 4.16000 4.69726 4.12654 4.10866 3.73705 3.73466 3.06433 1.12440 2.26971 369 - -
2.68618 4.42225 2.77519 2.73123 3.46354 2.40513 3.72494 3.29354 2.67741 2.69355 4.24690 2.90347 2.73739 3.18146 2.89801 2.37887 2.77519 2.98518 4.58477 3.61503
0.02228 4.21088 4.93323 0.61958 0.77255 0.66452 0.72262
156 2.52498 4.90659 2.99183 2.44097 4.18736 3.44140 3.64147 3.61426 1.87106 3.18693 3.98595 2.96273 3.00174 2.77659 2.72097 2.35811 2.68780 3.26649 5.39739 3.63655 370 - -
2.68618 4.42225 2.77519 2.73123 3.46354 2.40513 3.72494 3.29354 2.67741 2.69355 4.24690 2.90347 2.73739 3.18146 2.89801 2.37887 2.77519 2.98518 4.58477 3.61503
0.02228 4.21088 4.93323 0.61958 0.77255 0.66452 0.72262

157 2.68389 5.13122 2.45798 1.94006 4.44589 3.40155 3.63049 3.91324 2.03693 2.71940 4.18568 2.73073 3.82523 2.57860 2.86888 2.65024 2.91853 3.50654 5.57471 4.17718 371 - -
2.68618 4.42225 2.77519 2.73123 3.46354 2.40513 3.72494 3.29354 2.67741 2.69355 4.24690 2.90347 2.73739 3.18146 2.89801 2.37887 2.77519 2.98518 4.58477 3.61503
0.02228 4.21088 4.93323 0.61958 0.77255 0.66452 0.72262
158 2.65164 5.00679 2.35341 2.26793 4.29988 3.18441 3.62831 3.74291 2.37900 2.98376 4.07025 2.91742 3.82646 2.75068 2.05821 2.64268 2.88397 3.13629 5.47345 4.10354 372 - -
2.68618 4.42225 2.77519 2.73123 3.46354 2.40513 3.72494 3.29354 2.67741 2.69355 4.24690 2.90347 2.73739 3.18146 2.89801 2.37887 2.77519 2.98518 4.58477 3.61503
0.02228 4.21088 4.93323 0.61958 0.77255 0.66452 0.72262
159 2.61170 4.57403 2.95306 2.49351 3.75715 2.63427 3.72892 2.98864 2.61088 2.80710 2.91249 3.11229 3.89007 2.94622 2.86538 2.72451 2.70588 2.41344 5.12243 3.84025 373 - -
2.68618 4.42225 2.77519 2.73123 3.46354 2.40513 3.72494 3.29354 2.67741 2.69355 4.24690 2.90347 2.73739 3.18146 2.89801 2.37887 2.77519 2.98518 4.58477 3.61503
0.02228 4.21088 4.93323 0.61958 0.77255 0.66452 0.72262
160 2.44766 5.01014 2.41234 2.04486 4.30371 3.41041 3.61354 3.75597 2.37718 3.29328 3.60047 2.72028 3.80949 2.63463 2.86105 2.53248 2.72270 3.37304 5.47454 3.40852 374 - -
2.68618 4.42225 2.77519 2.73123 3.46354 2.40513 3.72494 3.29354 2.67741 2.69355 4.24690 2.90347 2.73739 3.18146 2.89801 2.37887 2.77519 2.98518 4.58477 3.61503
0.02228 4.21088 4.93323 0.61958 0.77255 0.60066 0.79507
161 2.50411 4.56211 3.05718 2.51572 3.35282 2.69951 3.74189 2.98739 2.49579 2.79264 3.65244 3.13167 3.90152 2.96594 3.05585 2.62035 2.67797 2.38714 5.11144 3.43682 375 - -
2.68618 4.42225 2.77519 2.73123 3.46354 2.40513 3.72494 3.29354 2.67741 2.69355 4.24690 2.90347 2.73739 3.18146 2.89801 2.37887 2.77519 2.98518 4.58477 3.61503
0.17690 4.24206 1.91216 0.61958 0.77255 0.63000 0.76055
162 2.68028 4.24590 3.81352 3.25965 2.95112 2.88303 3.83089 2.39700 3.14208 2.40437 3.35500 3.54117 4.07135 3.42084 3.39768 2.97470 2.91589 2.51189 2.22916 2.79381 376 - -
2.68618 4.42225 2.77519 2.73123 3.46354 2.40513 3.72494 3.29354 2.67741 2.69355 4.24690 2.90347 2.73739 3.18146 2.89801 2.37887 2.77519 2.98518 4.58477 3.61503
0.07343 4.09033 2.91762 0.61958 0.77255 0.77932 0.61381
163 2.62655 4.91796 2.69463 2.38749 4.21332 2.94325 3.62215 3.64485 1.77872 3.21105 3.44529 2.91461 3.80979 2.75592 2.83081 2.47056 2.86392 3.28746 5.41401 4.06011 377 - -
2.68618 4.42225 2.77519 2.73123 3.46354 2.40513 3.72494 3.29354 2.67741 2.69355 4.24690 2.90347 2.73739 3.18146 2.89801 2.37887 2.77519 2.98518 4.58477 3.61503
0.02503 4.09609 4.81844 0.61958 0.77255 0.81747 0.58258
164 2.45484 4.73295 2.53863 2.46638 3.95951 3.42991 3.64004 3.36299 2.38669 2.64366 3.53163 2.97788 3.82105 2.80337 2.64686 2.42356 2.82138 2.82259 5.24851 3.92712 378 - -
2.68618 4.42225 2.77519 2.73123 3.46354 2.40513 3.72494 3.29354 2.67741 2.69355 4.24690 2.90347 2.73739 3.18146 2.89801 2.37887 2.77519 2.98518 4.58477 3.61503
0.02503 4.09609 4.81844 0.61958 0.77255 0.81747 0.58258
165 2.67914 4.99322 2.99680 2.43239 4.30447 2.96534 3.59859 3.73153 1.80462 3.26326 3.52171 2.70924 3.83807 2.52201 2.40477 2.67453 2.90037 3.36871 5.42930 4.09629 379 - -
2.68618 4.42225 2.77519 2.73123 3.46354 2.40513 3.72494 3.29354 2.67741 2.69355 4.24690 2.90347 2.73739 3.18146 2.89801 2.37887 2.77519 2.98518 4.58477 3.61503
0.02503 4.09609 4.81844 0.61958 0.77255 0.81747 0.58258
166 2.70358 5.15513 2.32447 2.28459 4.46973 2.87174 3.33947 3.93986 2.14038 3.44307 4.21908 2.83640 3.81338 2.02313 2.86802 2.66145 2.94213 3.53363 5.59519 4.19368 380 - -
2.68618 4.42225 2.77519 2.73123 3.46354 2.40513 3.72494 3.29354 2.67741 2.69355 4.24690 2.90347 2.73739 3.18146 2.89801 2.37887 2.77519 2.98518 4.58477 3.61503
0.02503 4.09609 4.81844 0.61958 0.77255 0.81747 0.58258
167 2.73707 5.18853 2.13197 1.85693 4.49274 2.36414 3.66805 3.60651 2.51129 3.48551 4.27429 2.58388 3.82067 2.79985 3.02863 2.68835 2.98802 3.56305 5.65705 4.23582 381 - -
2.68618 4.42225 2.77519 2.73123 3.46354 2.40513 3.72494 3.29354 2.67741 2.69355 4.24690 2.90347 2.73739 3.18146 2.89801 2.37887 2.77519 2.98518 4.58477 3.61503
0.20854 4.09609 1.76264 0.61958 0.77255 0.55328 0.85581
168 2.72328 5.18629 2.47422 2.04913 4.51697 3.37728 3.62178 3.98456 1.74025 3.47190 4.24634 2.54278 3.82322 2.73904 2.63036 2.67689 2.95798 3.57249 5.60846 4.21440 382 - -
2.68618 4.42225 2.77519 2.73123 3.46354 2.40513 3.72494 3.29354 2.67741 2.69355 4.24690 2.90347 2.73739 3.18146 2.89801 2.37887 2.77519 2.98518 4.58477 3.61503

0.05513 4.09549 3.29722 0.61958 0.77255 0.81797 0.58219

169 2.94072 5.17245 3.23085 2.64430 4.59368 3.59634 3.61770 3.97324 1.55590 3.42761 4.26085 2.52538 3.97871 2.74440 1.68872 2.92309 3.12861 3.62408 5.49611 4.26511 383 - -

2.68618 4.42225 2.77519 2.73123 3.46354 2.40513 3.72494 3.29354 2.67741 2.69355 4.24690 2.90347 2.73739 3.18146 2.89801 2.37887 2.77519 2.98518 4.58477 3.61503

0.02580 4.06616 4.78850 0.61958 0.77255 0.78913 0.60558

170 2.60409 4.31114 3.50556 2.93542 3.44600 3.23829 3.83975 2.60488 2.86633 2.24604 2.61003 3.33333 3.96605 3.18003 2.49783 2.69698 2.71355 2.57988 4.89427 3.46574 384 - -

2.68618 4.42225 2.77519 2.73123 3.46354 2.40513 3.72494 3.29354 2.67741 2.69355 4.24690 2.90347 2.73739 3.18146 2.89801 2.37887 2.77519 2.98518 4.58477 3.61503

0.02271 4.19208 4.91443 0.61958 0.77255 0.81797 0.58219

171 2.63249 4.72958 3.09090 2.26196 3.94669 3.09641 3.67728 2.67619 2.37715 2.49720 3.80873 3.03547 3.86572 2.52575 2.69087 2.69534 2.86156 3.04894 5.24330 3.93732 385 - -

2.68618 4.42225 2.77519 2.73123 3.46354 2.40513 3.72494 3.29354 2.67741 2.69355 4.24690 2.90347 2.73739 3.18146 2.89801 2.37887 2.77519 2.98518 4.58477 3.61503

0.02271 4.19208 4.91443 0.61958 0.77255 0.81797 0.58219

172 2.29841 4.66328 3.10651 2.56053 3.88655 3.46400 3.44804 3.28159 2.43799 2.92563 3.76677 3.05465 2.75684 2.77087 2.97402 2.50864 2.69103 2.47886 5.21146 3.91113 386 - -

2.68618 4.42225 2.77519 2.73123 3.46354 2.40513 3.72494 3.29354 2.67741 2.69355 4.24690 2.90347 2.73739 3.18146 2.89801 2.37887 2.77519 2.98518 4.58477 3.61503

0.02271 4.19208 4.91443 0.61958 0.77255 0.81797 0.58219

173 2.48731 4.39561 3.36909 2.69242 2.97192 3.55014 3.22313 2.81367 2.64070 2.61252 3.11282 3.02876 3.93172 2.92414 3.14678 2.78605 2.65864 2.24531 4.96697 3.71861 387 - -

2.68618 4.42225 2.77519 2.73123 3.46354 2.40513 3.72494 3.29354 2.67741 2.69355 4.24690 2.90347 2.73739 3.18146 2.89801 2.37887 2.77519 2.98518 4.58477 3.61503

0.07167 4.19208 2.91784 0.61958 0.77255 0.55291 0.85631

174 2.65460 5.08645 2.76040 2.36714 4.40915 2.70365 3.62540 3.87350 2.06334 3.38410 4.14326 2.72953 3.56369 2.73410 2.45357 2.44441 2.51402 3.46719 5.54093 4.15090 388 - -

2.68626 4.42280 2.77462 2.73063 3.46369 2.40453 3.72440 3.29357 2.67743 2.69404 4.24745 2.90352 2.73771 3.18116 2.89832 2.37919 2.77513 2.98547 4.58532 3.61523

0.55597 1.39074 1.72827 2.21483 0.11560 0.47694 0.96938

175 2.73682 5.24119 2.19002 2.17166 4.56375 2.54499 2.99521 4.04871 2.05671 3.53551 4.29790 2.54997 3.84373 2.77865 2.95712 2.68537 2.97758 3.62177 5.67795 4.25522 408 - -

2.68618 4.42225 2.77519 2.73123 3.46354 2.40513 3.72494 3.29354 2.67741 2.69355 4.24690 2.90347 2.73739 3.18146 2.89801 2.37887 2.77519 2.98518 4.58477 3.61503

0.19686 4.24011 1.80615 0.61958 0.77255 0.69339 0.69291

176 2.59288 4.61968 3.10640 2.28550 3.81615 2.64274 3.30108 3.04165 2.53067 2.67716 3.12737 3.04658 3.85130 2.87717 2.96304 2.49519 2.82497 2.92819 5.15467 3.85880 409 - -

2.68618 4.42225 2.77519 2.73123 3.46354 2.40513 3.72494 3.29354 2.67741 2.69355 4.24690 2.90347 2.73739 3.18146 2.89801 2.37887 2.77519 2.98518 4.58477 3.61503

0.02459 4.11359 4.83594 0.61958 0.77255 0.88382 0.53307

177 2.62596 5.01782 2.36550 2.12525 4.31151 2.85955 3.60045 3.76754 2.37045 3.11739 4.07571 2.64390 3.79247 2.71943 2.67912 2.60805 2.85997 3.38199 5.47909 3.36316 410 - -

2.68618 4.42225 2.77519 2.73123 3.46354 2.40513 3.72494 3.29354 2.67741 2.69355 4.24690 2.90347 2.73739 3.18146 2.89801 2.37887 2.77519 2.98518 4.58477 3.61503

0.02459 4.11359 4.83594 0.61958 0.77255 0.38940 1.13155

178 2.49348 5.13314 2.34919 2.04546 4.45576 2.93605 2.98345 3.92671 2.39190 3.42880 3.67753 2.66359 3.84791 2.75035 2.75444 2.64785 2.90741 3.23185 5.57925 4.18122 411 - -

2.68618 4.42225 2.77519 2.73123 3.46354 2.40513 3.72494 3.29354 2.67741 2.69355 4.24690 2.90347 2.73739 3.18146 2.89801 2.37887 2.77519 2.98518 4.58477 3.61503

0.01831 4.40520 5.12755 0.61958 0.77255 0.57694 0.82466

179 2.41726 5.12392 2.69071 2.12492 4.44583 2.95327 3.66709 3.91206 2.34053 3.42542 4.18551 2.93625 2.84162 2.77737 2.91667 2.27977 2.46283 3.50517 5.58586 4.19118 412 - -

2.68618 4.42225 2.77519 2.73123 3.46354 2.40513 3.72494 3.29354 2.67741 2.69355 4.24690 2.90347 2.73739 3.18146 2.89801 2.37887 2.77519 2.98518 4.58477 3.61503

0.04228 4.40520 3.53415 0.61958 0.77255 0.57694 0.82466

180 2.66854 5.07452 2.33468 2.07757 4.37793 3.30304 3.64967 3.83565 2.30218 3.07227 3.66415 2.80463 2.78143 2.76199 2.88988 2.65146 2.90020 3.06008 5.53294 4.14786 413 - -

2.68618 4.42225 2.77519 2.73123 3.46354 2.40513 3.72494 3.29354 2.67741 2.69355 4.24690 2.90347 2.73739 3.18146 2.89801 2.37887 2.77519 2.98518 4.58477 3.61503

0.01875 4.38167 5.10402 0.61958 0.77255 0.46518 0.98891

181 2.61025 4.56762 3.09377 2.66946 3.54194 2.87409 3.86250 2.72647 2.79603 2.53579 2.48608 3.28062 3.18536 2.90743 3.19831 2.58145 2.93497 2.57638 5.12914 3.86700 414 - -

2.68618 4.42225 2.77519 2.73123 3.46354 2.40513 3.72494 3.29354 2.67741 2.69355 4.24690 2.90347 2.73739 3.18146 2.89801 2.37887 2.77519 2.98518 4.58477 3.61503

0.07842 4.52826 2.73920 0.61958 0.77255 0.52130 0.90079

182 2.19633 5.15690 2.54531 2.01627 4.48067 3.09208 3.66457 3.95276 2.41144 3.27483 4.20401 2.70192 3.22301 2.59599 2.90171 2.66664 2.74243 3.53620 5.60235 3.71681 415 - -

2.68618 4.42225 2.77519 2.73123 3.46354 2.40513 3.72494 3.29354 2.67741 2.69355 4.24690 2.90347 2.73739 3.18146 2.89801 2.37887 2.77519 2.98518 4.58477 3.61503

0.01720 4.46705 5.18940 0.61958 0.77255 0.56022 0.84649

183 2.69090 4.58422 2.56826 2.58585 3.00445 3.60587 3.83684 2.95978 2.75455 2.25039 2.84588 3.04056 3.50958 3.08094 3.16438 2.83160 2.46658 2.87424 5.14133 3.87326 416 - -

2.68618 4.42225 2.77519 2.73123 3.46354 2.40513 3.72494 3.29354 2.67741 2.69355 4.24690 2.90347 2.73739 3.18146 2.89801 2.37887 2.77519 2.98518 4.58477 3.61503

0.01683 4.48867 5.21102 0.61958 0.77255 0.58512 0.81427

184 2.40137 4.66462 3.03363 2.49789 3.84789 3.13413 3.80401 2.91095 2.68337 2.43292 2.92529 3.03943 3.96711 3.01759 3.11123 2.16637 2.80769 2.84991 5.20933 3.92466 417 - -

2.68618 4.42225 2.77519 2.73123 3.46354 2.40513 3.72494 3.29354 2.67741 2.69355 4.24690 2.90347 2.73739 3.18146 2.89801 2.37887 2.77519 2.98518 4.58477 3.61503

0.04153 4.48867 3.52514 0.61958 0.77255 0.58512 0.81427

185 2.56952 4.99680 2.15003 2.30306 3.66694 3.49725 3.71913 3.69352 2.50942 2.80401 4.06858 3.00930 3.90342 2.85464 2.98605 1.89337 2.94481 3.34399 5.48759 4.12853 418 - -

2.68618 4.42225 2.77519 2.73123 3.46354 2.40513 3.72494 3.29354 2.67741 2.69355 4.24690 2.90347 2.73739 3.18146 2.89801 2.37887 2.77519 2.98518 4.58477 3.61503

0.01725 4.46439 5.18673 0.61958 0.77255 0.62120 0.77068

186 2.68899 4.51446 2.75059 1.95705 4.07395 3.26696 3.06686 3.95546 2.12011 3.45247 4.20080 2.94304 3.86186 2.76021 2.52385 2.21194 2.92013 3.53618 5.59837 4.19901 419 - -

2.68618 4.42225 2.77519 2.73123 3.46354 2.40513 3.72494 3.29354 2.67741 2.69355 4.24690 2.90347 2.73739 3.18146 2.89801 2.37887 2.77519 2.98518 4.58477 3.61503

0.01725 4.46439 5.18673 0.61958 0.77255 0.62120 0.77068

187 2.09166 4.62758 3.35528 2.82416 4.12098 2.65400 3.94562 3.09930 2.81168 3.17088 4.00245 3.24984 2.69424 3.14273 3.23583 1.94622 2.22933 3.17148 5.44637 4.16121 420 - -

2.68618 4.42225 2.77519 2.73123 3.46354 2.40513 3.72494 3.29354 2.67741 2.69355 4.24690 2.90347 2.73739 3.18146 2.89801 2.37887 2.77519 2.98518 4.58477 3.61503

0.01725 4.46439 5.18673 0.61958 0.77255 0.62120 0.77068

188 2.68857 3.29540 3.67104 3.09672 3.48012 3.69062 3.94826 2.83648 2.71112 2.05934 3.45755 3.47059 4.06776 3.31973 2.22501 2.73271 2.78016 2.50870 3.22096 3.71607 421 - -

2.68618 4.42225 2.77519 2.73123 3.46354 2.40513 3.72494 3.29354 2.67741 2.69355 4.24690 2.90347 2.73739 3.18146 2.89801 2.37887 2.77519 2.98518 4.58477 3.61503

0.13103 4.46439 2.19558 0.61958 0.77255 0.62120 0.77068

189 2.66441 5.02701 2.83829 2.41711 4.31852 3.23720 3.45709 3.76553 2.29445 2.69995 4.08311 2.95238 3.13989 2.25244 2.38264 2.65677 2.89470 3.38886 5.49076 3.58879 422 - -

2.68618 4.42225 2.77519 2.73123 3.46354 2.40513 3.72494 3.29354 2.67741 2.69355 4.24690 2.90347 2.73739 3.18146 2.89801 2.37887 2.77519 2.98518 4.58477 3.61503

0.01931 4.35267 5.07502 0.61958 0.77255 0.76301 0.62785

190 2.48469 4.42176 3.59853 3.29756 4.38762 3.21021 4.36531 3.77544 3.30947 3.49247 4.35941 3.49700 1.03636 3.63871 3.62034 2.17158 2.95268 2.92399 5.75798 4.51258 423 - -

2.68618 4.42225 2.77519 2.73123 3.46354 2.40513 3.72494 3.29354 2.67741 2.69355 4.24690 2.90347 2.73739 3.18146 2.89801 2.37887 2.77519 2.98518 4.58477 3.61503

0.01931 4.35267 5.07502 0.61958 0.77255 0.76301 0.62785

191 3.68848 4.98115 5.26100 4.72384 3.18823 4.92791 5.21136 2.38412 4.49993 0.57876 2.56516 4.98666 5.05319 4.59152 4.57343 4.32767 3.90886 2.59721 5.37382 4.32186 424 - -

2.68619 4.42226 2.77521 2.73124 3.46355 2.40514 3.72496 3.29355 2.67733 2.69356 4.24691 2.90348 2.73741 3.18133 2.89802 2.37888 2.77521 2.98519 4.58478 3.61504

0.04803 3.20281 5.07502 0.64065 0.74855 0.60857 0.78555

192 2.66773 2.89515 3.46796 2.76178 3.59233 3.62225 3.87402 2.95954 2.86004 2.66345 3.54840 3.32959 4.00690 2.99100 3.23176 1.98805 2.24356 2.72584 4.06299 3.54504 427 - -
2.68618 4.42225 2.77519 2.73123 3.46354 2.40513 3.72494 3.29354 2.67741 2.69355 4.24690 2.90347 2.73739 3.18146 2.89801 2.37887 2.77519 2.98518 4.58477 3.61503
0.01804 4.41997 5.14231 0.61958 0.77255 0.68194 0.70448
193 2.78759 5.21273 2.73810 1.85757 4.54116 3.49544 3.70930 3.99915 2.41916 3.50150 4.27528 2.80785 3.91855 2.82499 1.66216 2.75539 3.01939 3.31994 5.64719 4.26723 428 - -
2.68618 4.42225 2.77519 2.73123 3.46354 2.40513 3.72494 3.29354 2.67741 2.69355 4.24690 2.90347 2.73739 3.18146 2.89801 2.37887 2.77519 2.98518 4.58477 3.61503
0.01804 4.41997 5.14231 0.61958 0.77255 0.68194 0.70448
194 2.82370 5.12266 3.17349 2.58249 4.44833 3.58850 3.38016 3.86658 1.56694 3.38703 4.18565 2.95163 3.56759 2.82768 2.02620 2.81888 3.03811 2.69478 5.53772 4.22384 429 - -
2.68618 4.42225 2.77519 2.73123 3.46354 2.40513 3.72494 3.29354 2.67741 2.69355 4.24690 2.90347 2.73739 3.18146 2.89801 2.37887 2.77519 2.98518 4.58477 3.61503
0.01804 4.41997 5.14231 0.61958 0.77255 0.61547 0.77737
195 2.99719 4.35880 4.70185 4.13542 3.53972 4.24919 4.63855 1.49133 3.50768 1.94181 3.45122 4.29915 4.58060 4.21824 4.14205 3.57264 2.97747 1.30075 5.21602 4.02617 430 - -
2.68618 4.42225 2.77519 2.73123 3.46354 2.40513 3.72494 3.29354 2.67741 2.69355 4.24690 2.90347 2.73739 3.18146 2.89801 2.37887 2.77519 2.98518 4.58477 3.61503
0.01757 4.44640 5.16875 0.61958 0.77255 0.64657 0.74200
196 2.73267 5.10725 2.95198 2.45056 4.42647 3.13675 3.46244 3.87776 2.23226 3.40819 4.18499 2.98636 2.47481 2.51062 2.91027 1.85450 2.96859 3.49069 5.58070 4.20670 431 - -
2.68618 4.42225 2.77519 2.73123 3.46354 2.40513 3.72494 3.29354 2.67741 2.69355 4.24690 2.90347 2.73739 3.18146 2.89801 2.37887 2.77519 2.98518 4.58477 3.61503
0.01757 4.44640 5.16875 0.61958 0.77255 0.39693 1.11591
197 2.71542 4.38068 3.68868 3.11719 3.30497 3.71739 2.62157 1.83528 2.88674 2.58714 3.48665 3.49416 4.09453 3.34722 2.70496 2.81680 2.94680 2.43913 4.96410 3.18083 432 - -
2.68618 4.42225 2.77519 2.73123 3.46354 2.40513 3.72494 3.29354 2.67741 2.69355 4.24690 2.90347 2.73739 3.18146 2.89801 2.37887 2.77519 2.98518 4.58477 3.61503
0.01585 4.54861 5.27096 0.61958 0.77255 0.48576 0.95510
198 2.64096 5.17255 2.77700 2.44064 4.50366 3.28055 3.70076 3.97159 2.00822 3.47628 4.23149 2.98308 2.35205 2.80741 2.60140 2.14193 2.85631 3.55879 5.62799 4.23467 433 - -
2.68618 4.42225 2.77519 2.73123 3.46354 2.40513 3.72494 3.29354 2.67741 2.69355 4.24690 2.90347 2.73739 3.18146 2.89801 2.37887 2.77519 2.98518 4.58477 3.61503
0.01585 4.54861 5.27096 0.61958 0.77255 0.48576 0.95510
199 2.11845 3.53011 3.12435 2.56996 4.19988 3.33934 3.76560 3.62094 2.37790 3.21803 4.02544 2.86494 3.47675 2.91265 2.78399 1.87546 2.75521 3.28328 5.45394 4.11719 434 - -
2.68618 4.42225 2.77519 2.73123 3.46354 2.40513 3.72494 3.29354 2.67741 2.69355 4.24690 2.90347 2.73739 3.18146 2.89801 2.37887 2.77519 2.98518 4.58477 3.61503
0.01585 4.54861 5.27096 0.61958 0.77255 0.48576 0.95510
200 2.22667 4.65253 3.31433 2.75255 3.82870 3.26749 3.83641 2.96407 2.58793 2.75285 3.74266 3.22859 3.99652 2.91003 2.62034 2.23633 2.81314 2.94901 4.05988 2.73234 435 - -
2.68618 4.42225 2.77519 2.73123 3.46354 2.40513 3.72494 3.29354 2.67741 2.69355 4.24690 2.90347 2.73739 3.18146 2.89801 2.37887 2.77519 2.98518 4.58477 3.61503
0.29160 4.54861 1.41736 0.61958 0.77255 0.48576 0.95510
201 2.78939 4.73676 3.40160 2.79005 3.95396 3.63650 3.76814 2.27957 1.99683 2.93275 3.82143 3.23699 4.01038 2.96557 1.88567 2.89492 3.00508 2.74860 5.24384 3.99669 436 - -
2.68618 4.42225 2.77519 2.73123 3.46354 2.40513 3.72494 3.29354 2.67741 2.69355 4.24690 2.90347 2.73739 3.18146 2.89801 2.37887 2.77519 2.98518 4.58477 3.61503
0.02082 4.27784 5.00018 0.61958 0.77255 0.84058 0.56469
202 3.04610 4.39747 4.71032 4.15229 3.48851 4.32298 4.72781 1.19285 4.03572 1.92186 3.07822 4.35451 4.62930 4.24959 4.20330 3.65335 2.58387 1.74503 5.28010 4.10935 437 - -
2.68618 4.42225 2.77519 2.73123 3.46354 2.40513 3.72494 3.29354 2.67741 2.69355 4.24690 2.90347 2.73739 3.18146 2.89801 2.37887 2.77519 2.98518 4.58477 3.61503
0.02082 4.27784 5.00018 0.61958 0.77255 0.84058 0.56469
203 2.40395 4.54079 3.26608 2.74599 3.86732 3.44086 3.84132 2.47158 2.73317 2.91765 3.77654 2.46690 3.90596 3.06570 3.14383 1.96475 2.48685 2.93149 5.23394 3.95958 438 - -
2.68618 4.42225 2.77519 2.73123 3.46354 2.40513 3.72494 3.29354 2.67741 2.69355 4.24690 2.90347 2.73739 3.18146 2.89801 2.37887 2.77519 2.98518 4.58477 3.61503

0.02082 4.27784 5.00018 0.61958 0.77255 0.31450 1.30991
204 2.58399 4.22703 4.26466 3.67692 2.76071 3.87470 4.18293 2.31722 3.55146 2.05121 2.64990 3.87123 1.89981 3.76427 3.71984 2.98752 2.99555 2.37712 4.84286 3.65463 439 - -
2.68618 4.42225 2.77519 2.73123 3.46354 2.40513 3.72494 3.29354 2.67741 2.69355 4.24690 2.90347 2.73739 3.18146 2.89801 2.37887 2.77519 2.98518 4.58477 3.61503
0.01482 4.61473 5.33708 0.61958 0.77255 0.48576 0.95510
205 3.06344 4.50548 4.44958 3.91478 2.49079 4.10782 4.05497 2.93088 3.77928 1.95246 3.59602 4.03208 4.46890 3.95950 3.93258 2.97620 3.09493 2.76228 4.46564 1.17230 440 - -
2.68618 4.42225 2.77519 2.73123 3.46354 2.40513 3.72494 3.29354 2.67741 2.69355 4.24690 2.90347 2.73739 3.18146 2.89801 2.37887 2.77519 2.98518 4.58477 3.61503
0.01482 4.61473 5.33708 0.61958 0.77255 0.48576 0.95510
206 2.92934 5.04262 3.47687 2.90067 4.51591 3.67663 3.92835 3.90588 2.46925 3.25663 4.30292 3.34069 3.40994 3.08053 1.03302 2.66415 2.86712 3.55299 5.64397 4.38502 441 - -
2.68618 4.42225 2.77519 2.73123 3.46354 2.40513 3.72494 3.29354 2.67741 2.69355 4.24690 2.90347 2.73739 3.18146 2.89801 2.37887 2.77519 2.98518 4.58477 3.61503
0.01482 4.61473 5.33708 0.61958 0.77255 0.48576 0.95510
207 2.31806 3.73056 4.76975 4.16527 2.59513 4.05528 4.39351 1.73070 3.97322 1.66553 2.54993 4.19465 4.40153 4.10850 4.00685 3.36934 3.10026 2.18283 4.88157 3.71492 442 - -
2.68618 4.42225 2.77519 2.73123 3.46354 2.40513 3.72494 3.29354 2.67741 2.69355 4.24690 2.90347 2.73739 3.18146 2.89801 2.37887 2.77519 2.98518 4.58477 3.61503
0.01482 4.61473 5.33708 0.61958 0.77255 0.48576 0.95510
208 2.86539 4.24759 4.69212 4.09141 2.96094 4.03824 4.36091 2.15654 3.91166 1.45775 3.25832 4.15154 3.89157 4.05821 3.96851 3.34834 3.09692 1.60193 4.86586 2.95900 443 - -
2.68618 4.42225 2.77519 2.73123 3.46354 2.40513 3.72494 3.29354 2.67741 2.69355 4.24690 2.90347 2.73739 3.18146 2.89801 2.37887 2.77519 2.98518 4.58477 3.61503
0.01482 4.61473 5.33708 0.61958 0.77255 0.48576 0.95510
209 2.46092 4.83918 2.52621 2.65694 4.05126 3.59743 3.81961 1.86417 2.10655 3.08546 3.92341 3.16158 3.99296 2.99892 3.09778 2.65143 2.98568 3.14755 5.36672 3.76214 444 - -
2.68618 4.42225 2.77519 2.73123 3.46354 2.40513 3.72494 3.29354 2.67741 2.69355 4.24690 2.90347 2.73739 3.18146 2.89801 2.37887 2.77519 2.98518 4.58477 3.61503
0.01482 4.61473 5.33708 0.61958 0.77255 0.48576 0.95510
210 2.28045 4.16097 4.47713 3.87788 2.85094 3.53014 4.21574 2.28808 3.71269 1.73479 3.10345 3.97404 3.90648 3.88808 3.80464 3.05148 2.98026 1.68328 4.78620 3.60400 445 - -
2.68618 4.42225 2.77519 2.73123 3.46354 2.40513 3.72494 3.29354 2.67741 2.69355 4.24690 2.90347 2.73739 3.18146 2.89801 2.37887 2.77519 2.98518 4.58477 3.61503
0.01482 4.61473 5.33708 0.61958 0.77255 0.48576 0.95510
211 2.63498 3.33630 4.53678 3.93225 2.29849 3.88704 3.81701 2.21227 3.75193 1.77825 3.06399 3.99423 4.25226 3.91359 3.81771 2.92997 2.28741 2.25509 4.75723 3.57720 446 - -
2.68618 4.42238 2.77533 2.73137 3.46339 2.40526 3.72508 3.29340 2.67754 2.69280 4.24703 2.90360 2.73745 3.18160 2.89805 2.37896 2.77533 2.98477 4.58431 3.61516
0.19319 1.76689 5.33708 0.99627 0.46085 0.48576 0.95510
212 2.75574 4.72444 3.33311 2.76669 3.90742 3.64108 2.90136 2.57147 2.69715 2.95604 3.81291 3.24518 4.02544 2.95080 1.81506 2.47480 2.98529 2.60973 5.26077 3.71226 450 - -
2.68618 4.42225 2.77519 2.73123 3.46354 2.40513 3.72494 3.29354 2.67741 2.69355 4.24690 2.90347 2.73739 3.18146 2.89801 2.37887 2.77519 2.98518 4.58477 3.61503
0.01482 4.61473 5.33708 0.61958 0.77255 0.48576 0.95510
213 2.68429 4.26442 3.40938 3.69297 2.83325 3.91604 4.22611 2.34118 3.57471 1.31462 3.33953 3.90112 4.27907 3.79164 3.75357 3.20570 2.61320 2.18799 4.89458 3.70638 451 - -
2.68610 4.42197 2.77527 2.73131 3.46362 2.40521 3.72502 3.29305 2.67749 2.69323 4.24698 2.90355 2.73747 3.18154 2.89809 2.37885 2.77527 2.98517 4.58485 3.61494
0.19319 1.87758 3.78480 0.67319 0.71351 0.48576 0.95510
214 2.59874 3.19921 4.48411 3.89322 3.31628 2.95672 4.26732 2.25406 3.73643 1.91848 3.32321 4.00020 4.28913 3.92093 3.84252 3.06570 3.01093 1.44103 4.85192 3.66749 454 - -
2.68618 4.42225 2.77519 2.73123 3.46354 2.40513 3.72494 3.29354 2.67741 2.69355 4.24690 2.90347 2.73739 3.18146 2.89801 2.37887 2.77519 2.98518 4.58477 3.61503
0.01510 4.59666 5.31901 0.61958 0.77255 0.46521 0.98887
215 2.35884 3.32807 4.25817 3.66892 3.15786 2.81409 4.15585 1.90271 3.53963 2.00082 3.31363 3.60908 3.86237 3.74981 3.70048 2.74610 2.83818 2.22456 4.81157 3.62220 455 - -

2.68618 4.42225 2.77519 2.73123 3.46354 2.40513 3.72494 3.29354 2.67741 2.69355 4.24690 2.90347 2.73739 3.18146 2.89801 2.37887 2.77519 2.98518 4.58477 3.61503
0.01482 4.61473 5.33708 0.61958 0.77255 0.48576 0.95510
216 3.20750 4.54735 5.04649 4.45637 2.26602 4.42927 4.74053 1.92404 4.28886 0.99352 3.21115 4.54477 4.71083 4.38136 4.33041 3.44761 3.43466 2.28059 5.12704 3.97891 456 - -
2.68618 4.42225 2.77519 2.73123 3.46354 2.40513 3.72494 3.29354 2.67741 2.69355 4.24690 2.90347 2.73739 3.18146 2.89801 2.37887 2.77519 2.98518 4.58477 3.61503
0.01482 4.61473 5.33708 0.61958 0.77255 0.48576 0.95510
217 2.27992 3.01058 3.99767 2.65649 2.50929 2.71314 4.08860 2.42578 3.32816 2.28587 3.38495 3.70141 4.17323 3.57877 3.57192 2.82581 2.96596 2.40799 4.87734 3.46881 457 - -
2.68618 4.42225 2.77519 2.73123 3.46354 2.40513 3.72494 3.29354 2.67741 2.69355 4.24690 2.90347 2.73739 3.18146 2.89801 2.37887 2.77519 2.98518 4.58477 3.61503
0.01482 4.61473 5.33708 0.61958 0.77255 0.48576 0.95510
218 2.44381 4.22420 4.38722 3.79714 2.47969 3.91417 4.23549 2.29994 3.65660 1.57728 3.30624 3.95134 4.27961 3.85257 3.79319 2.18540 3.01949 2.25734 4.84907 3.66357 458 - -
2.68618 4.42225 2.77519 2.73123 3.46354 2.40513 3.72494 3.29354 2.67741 2.69355 4.24690 2.90347 2.73739 3.18146 2.89801 2.37887 2.77519 2.98518 4.58477 3.61503
0.01482 4.61473 5.33708 0.61958 0.77255 0.48576 0.95510
219 2.73299 4.51445 4.97313 4.38631 1.49497 4.38167 4.70295 2.25903 4.22610 1.41908 3.24538 4.48623 4.67674 4.33946 4.28621 3.70610 3.39462 2.05220 5.12241 3.96518 459 - -
2.68618 4.42225 2.77519 2.73123 3.46354 2.40513 3.72494 3.29354 2.67741 2.69355 4.24690 2.90347 2.73739 3.18146 2.89801 2.37887 2.77519 2.98518 4.58477 3.61503
0.01482 4.61473 5.33708 0.61958 0.77255 0.48576 0.95510
220 2.32118 4.32342 4.72473 4.12446 2.44198 4.10074 4.39848 2.58385 3.94899 1.18967 2.49164 4.20116 4.43387 4.08221 4.00839 3.41028 3.16692 2.49981 3.82215 3.69622 460 - -
2.68618 4.42225 2.77519 2.73123 3.46354 2.40513 3.72494 3.29354 2.67741 2.69355 4.24690 2.90347 2.73739 3.18146 2.89801 2.37887 2.77519 2.98518 4.58477 3.61503
0.01482 4.61473 5.33708 0.61958 0.77255 0.48576 0.95510
221 2.73625 4.26811 4.01807 3.43833 2.65844 3.80616 2.82426 2.72675 3.34441 2.11172 3.10213 3.43547 4.17825 3.38055 3.10409 3.08058 2.54839 2.45220 3.28253 2.54432 461 - -
2.68618 4.42225 2.77519 2.73123 3.46354 2.40513 3.72494 3.29354 2.67741 2.69355 4.24690 2.90347 2.73739 3.18146 2.89801 2.37887 2.77519 2.98518 4.58477 3.61503
0.01482 4.61473 5.33708 0.61958 0.77255 0.48576 0.95510
222 3.76442 5.08174 4.86214 4.45321 1.77951 4.61390 2.22349 3.63970 4.31561 3.11634 4.23698 4.32999 4.93230 3.72223 4.40447 3.43560 3.98310 3.48734 2.05182 1.20636 462 - -
2.68618 4.42225 2.77519 2.73123 3.46354 2.40513 3.72494 3.29354 2.67741 2.69355 4.24690 2.90347 2.73739 3.18146 2.89801 2.37887 2.77519 2.98518 4.58477 3.61503
0.01482 4.61473 5.33708 0.61958 0.77255 0.48576 0.95510
223 3.28726 5.46458 3.73994 3.02562 4.90230 3.59879 2.92268 4.25813 1.89692 3.35993 4.53547 3.44503 4.29324 2.99405 0.94416 3.27291 3.44912 3.92608 5.73162 4.54443 463 - -
2.68618 4.42225 2.77519 2.73123 3.46354 2.40513 3.72494 3.29354 2.67741 2.69355 4.24690 2.90347 2.73739 3.18146 2.89801 2.37887 2.77519 2.98518 4.58477 3.61503
0.01482 4.61473 5.33708 0.61958 0.77255 0.48576 0.95510
224 2.75037 4.29141 4.00618 3.26763 3.40505 2.86458 4.10419 1.54720 3.13075 2.41384 3.08619 3.49193 4.18877 3.59025 3.58156 2.94883 2.75474 2.13381 4.89861 3.69604 464 - -
2.68618 4.42225 2.77519 2.73123 3.46354 2.40513 3.72494 3.29354 2.67741 2.69355 4.24690 2.90347 2.73739 3.18146 2.89801 2.37887 2.77519 2.98518 4.58477 3.61503
0.01435 4.64674 5.36909 0.61958 0.77255 0.48576 0.95510
225 2.74898 4.90667 3.04488 2.54940 4.13401 3.26998 3.14706 3.54365 2.47924 2.18470 3.98087 3.13121 3.97796 2.84341 2.44439 2.71274 2.35680 3.23245 3.28768 4.09200 465 - -
2.68618 4.42225 2.77519 2.73123 3.46354 2.40513 3.72494 3.29354 2.67741 2.69355 4.24690 2.90347 2.73739 3.18146 2.89801 2.37887 2.77519 2.98518 4.58477 3.61503
0.02895 4.64674 3.96619 0.61958 0.77255 0.48576 0.95510
226 2.66113 5.06910 3.06185 2.39916 4.34398 3.36308 1.99901 3.78266 2.52419 2.98250 4.13258 2.35306 3.94615 2.88054 2.99866 2.33648 2.81881 3.42252 5.54815 3.89528 466 - -
2.68623 4.42230 2.77511 2.73128 3.46359 2.40508 3.72499 3.29322 2.67746 2.69343 4.24695 2.90336 2.73745 3.18151 2.89806 2.37892 2.77524 2.98523 4.58482 3.61508
0.51468 2.31171 1.19332 0.66906 0.71782 0.51367 0.91204

227 2.70716 4.70865 3.25165 2.69913 3.95494 3.54046 3.76644 3.31821 2.34436 2.81605 3.83633 3.16449 1.82106 2.96148 2.62589 2.80073 2.94630 2.68547 5.26135 3.98649 471 - -
2.68618 4.42225 2.77519 2.73123 3.46354 2.40513 3.72494 3.29354 2.67741 2.69355 4.24690 2.90347 2.73739 3.18146 2.89801 2.37887 2.77519 2.98518 4.58477 3.61503
0.02082 4.27784 5.00018 0.61958 0.77255 0.63340 0.75669
228 2.54054 4.38933 3.62959 3.06673 3.54909 3.68245 3.97120 2.69172 3.01338 2.59664 2.76236 2.25439 4.07264 3.31542 3.35571 2.93999 2.40320 1.94865 5.01300 3.78260 472 - -
2.68618 4.42225 2.77519 2.73123 3.46354 2.40513 3.72494 3.29354 2.67741 2.69355 4.24690 2.90347 2.73739 3.18146 2.89801 2.37887 2.77519 2.98518 4.58477 3.61503
0.01769 4.43923 5.16158 0.61958 0.77255 0.80142 0.59546
229 2.41320 3.75793 2.83349 2.21759 4.42210 3.46622 3.44896 3.88567 2.15476 3.40068 4.15889 2.43743 3.29626 2.76523 2.65464 2.45035 2.91123 3.18531 5.56191 4.17163 473 - -
2.68618 4.42225 2.77519 2.73123 3.46354 2.40513 3.72494 3.29354 2.67741 2.69355 4.24690 2.90347 2.73739 3.18146 2.89801 2.37887 2.77519 2.98518 4.58477 3.61503
0.01769 4.43923 5.16158 0.61958 0.77255 0.80142 0.59546
230 2.78460 5.30183 1.90846 1.91197 4.63060 3.45548 3.71752 4.11935 2.34897 3.20616 4.35419 2.35618 3.90041 2.55159 3.00669 2.60786 3.02404 3.68511 5.73623 4.31117 474 - -
2.68618 4.42225 2.77519 2.73123 3.46354 2.40513 3.72494 3.29354 2.67741 2.69355 4.24690 2.90347 2.73739 3.18146 2.89801 2.37887 2.77519 2.98518 4.58477 3.61503
0.01769 4.43923 5.16158 0.61958 0.77255 0.80142 0.59546
231 1.60112 5.01486 2.70839 2.47744 4.30470 3.48269 3.75090 3.73837 2.47986 3.31583 4.11821 2.23720 3.91679 2.89206 3.03068 2.74407 2.84252 3.38449 5.53203 3.79903 475 - -
2.68618 4.42225 2.77519 2.73123 3.46354 2.40513 3.72494 3.29354 2.67741 2.69355 4.24690 2.90347 2.73739 3.18146 2.89801 2.37887 2.77519 2.98518 4.58477 3.61503
0.01769 4.43923 5.16158 0.61958 0.77255 0.80142 0.59546
232 2.67281 4.42146 3.03769 2.94071 3.19762 3.43868 3.88986 2.59113 2.89932 2.40120 2.86805 2.81699 4.02101 3.20611 2.99223 2.88179 2.54672 2.69727 4.99927 2.65345 476 - -
2.68618 4.42225 2.77519 2.73123 3.46354 2.40513 3.72494 3.29354 2.67741 2.69355 4.24690 2.90347 2.73739 3.18146 2.89801 2.37887 2.77519 2.98518 4.58477 3.61503
0.01769 4.43923 5.16158 0.61958 0.77255 0.80142 0.59546
233 2.37040 4.40721 3.53222 2.96694 3.55274 2.95520 3.90267 2.81090 2.92180 2.22175 3.51637 3.06369 3.34883 3.22731 3.28011 2.79040 2.52360 2.68729 2.68048 3.75673 477 - -
2.68618 4.42225 2.77519 2.73123 3.46354 2.40513 3.72494 3.29354 2.67741 2.69355 4.24690 2.90347 2.73739 3.18146 2.89801 2.37887 2.77519 2.98518 4.58477 3.61503
0.01769 4.43923 5.16158 0.61958 0.77255 0.80142 0.59546
234 3.52788 4.77306 5.41121 4.86853 3.45782 4.94339 5.36847 1.47973 4.74754 0.95332 3.22163 5.05745 5.10296 4.81386 4.81551 4.32661 3.75979 1.80081 5.57922 4.51756 478 - -
2.68618 4.42225 2.77519 2.73123 3.46354 2.40513 3.72494 3.29354 2.67741 2.69355 4.24690 2.90347 2.73739 3.18146 2.89801 2.37887 2.77519 2.98518 4.58477 3.61503
0.01769 4.43923 5.16158 0.61958 0.77255 0.80142 0.59546
235 3.07864 4.72535 4.21241 3.82081 3.22972 3.84273 4.24285 3.35440 3.51783 2.93939 4.03564 3.97300 4.40289 3.94068 3.70710 3.31256 2.47233 3.15960 0.86214 3.28873 479 - -
2.68618 4.42225 2.77519 2.73123 3.46354 2.40513 3.72494 3.29354 2.67741 2.69355 4.24690 2.90347 2.73739 3.18146 2.89801 2.37887 2.77519 2.98518 4.58477 3.61503
0.01769 4.43923 5.16158 0.61958 0.77255 0.80142 0.59546
236 2.68617 3.36854 4.22277 3.63773 2.95529 2.72150 4.12626 2.48110 3.50681 1.42458 3.26552 3.81474 4.17095 3.71774 3.66722 2.92888 2.92525 2.30711 4.77918 3.58781 480 - -
2.68618 4.42225 2.77519 2.73123 3.46354 2.40513 3.72494 3.29354 2.67741 2.69355 4.24690 2.90347 2.73739 3.18146 2.89801 2.37887 2.77519 2.98518 4.58477 3.61503
0.01769 4.43923 5.16158 0.61958 0.77255 0.80142 0.59546
237 2.97601 4.34471 4.70738 4.12684 3.37304 4.19582 4.55182 1.99222 3.98487 1.65346 2.40640 4.26546 4.51836 4.15720 4.09649 3.51314 2.36585 1.57351 5.07450 3.91274 481 - -
2.68618 4.42225 2.77519 2.73123 3.46354 2.40513 3.72494 3.29354 2.67741 2.69355 4.24690 2.90347 2.73739 3.18146 2.89801 2.37887 2.77519 2.98518 4.58477 3.61503
0.01769 4.43923 5.16158 0.61958 0.77255 0.80142 0.59546
238 2.01819 4.46734 3.56838 3.03303 3.76498 3.54350 4.01396 3.06012 2.97972 2.39678 3.22604 3.41195 4.02429 3.30326 3.34150 1.51368 2.94417 2.80773 5.20170 3.95775 482 - -
2.68618 4.42225 2.77519 2.73123 3.46354 2.40513 3.72494 3.29354 2.67741 2.69355 4.24690 2.90347 2.73739 3.18146 2.89801 2.37887 2.77519 2.98518 4.58477 3.61503

0.01769 4.43923 5.16158 0.61958 0.77255 0.80142 0.59546

239 2.98288 4.33613 4.78641 4.20668 2.12650 4.21761 4.57723 1.96473 4.05520 1.90037 3.29746 4.31306 4.53991 4.21622 4.14421 3.54040 2.75972 1.38176 5.07055 3.89535 483 - -

2.68618 4.42225 2.77519 2.73123 3.46354 2.40513 3.72494 3.29354 2.67741 2.69355 4.24690 2.90347 2.73739 3.18146 2.89801 2.37887 2.77519 2.98518 4.58477 3.61503

0.01769 4.43923 5.16158 0.61958 0.77255 0.80142 0.59546

240 3.11399 4.44128 4.94110 4.36927 2.97626 4.39242 4.77301 1.31635 4.22766 1.52950 3.29680 4.49110 4.68539 4.37530 4.31603 3.72573 2.81326 1.81458 5.22194 4.07146 484 - -

2.68618 4.42225 2.77519 2.73123 3.46354 2.40513 3.72494 3.29354 2.67741 2.69355 4.24690 2.90347 2.73739 3.18146 2.89801 2.37887 2.77519 2.98518 4.58477 3.61503

0.01769 4.43923 5.16158 0.61958 0.77255 0.80142 0.59546

241 2.48459 0.95606 4.43145 4.07663 4.14698 3.28389 4.70594 3.30298 3.92093 3.21953 4.15276 3.89482 4.04027 4.16582 4.06901 2.17089 2.97316 2.55346 5.64138 4.45518 485 - -

2.68618 4.42225 2.77519 2.73123 3.46354 2.40513 3.72494 3.29354 2.67741 2.69355 4.24690 2.90347 2.73739 3.18146 2.89801 2.37887 2.77519 2.98518 4.58477 3.61503

0.01769 4.43923 5.16158 0.61958 0.77255 0.80142 0.59546

242 3.27623 5.89267 1.95751 0.76887 5.18716 3.47178 4.06158 4.73860 3.12479 4.21615 5.08839 2.93471 4.11996 3.23525 3.74711 3.13771 3.57567 4.29201 6.34333 4.81734 486 - -

2.68618 4.42225 2.77519 2.73123 3.46354 2.40513 3.72494 3.29354 2.67741 2.69355 4.24690 2.90347 2.73739 3.18146 2.89801 2.37887 2.77519 2.98518 4.58477 3.61503

0.01769 4.43923 5.16158 0.61958 0.77255 0.80142 0.59546

243 2.84178 4.31875 4.28942 3.73514 3.44436 3.94963 4.34587 1.41744 3.60703 1.81547 3.39705 3.95783 4.33855 3.86102 3.80817 2.34784 2.94271 2.21123 5.04726 3.85163 487 - -

2.68618 4.42225 2.77519 2.73123 3.46354 2.40513 3.72494 3.29354 2.67741 2.69355 4.24690 2.90347 2.73739 3.18146 2.89801 2.37887 2.77519 2.98518 4.58477 3.61503

0.01769 4.43923 5.16158 0.61958 0.77255 0.80142 0.59546

244 2.82841 2.61993 4.46460 3.89804 2.90594 3.92460 4.16016 2.59346 3.73354 2.31847 3.33975 3.98941 4.30918 3.91524 3.84781 3.24246 3.07594 2.12215 1.61824 3.29011 488 - -

2.68618 4.42225 2.77519 2.73123 3.46354 2.40513 3.72494 3.29354 2.67741 2.69355 4.24690 2.90347 2.73739 3.18146 2.89801 2.37887 2.77519 2.98518 4.58477 3.61503

0.01769 4.43923 5.16158 0.61958 0.77255 0.80142 0.59546

245 3.81823 5.07794 5.14219 4.77761 0.75515 4.77042 4.24660 2.88850 4.59733 1.75076 3.51828 4.66509 5.03833 4.58773 4.61193 4.18930 4.05566 2.99773 4.37935 2.73561 489 - -

2.68618 4.42225 2.77519 2.73123 3.46354 2.40513 3.72494 3.29354 2.67741 2.69355 4.24690 2.90347 2.73739 3.18146 2.89801 2.37887 2.77519 2.98518 4.58477 3.61503

0.01769 4.43923 5.16158 0.61958 0.77255 0.80142 0.59546

246 1.12227 4.43443 3.67675 3.21948 3.65176 2.57972 4.24649 3.61356 3.21113 3.30119 4.14281 3.47680 3.95064 3.50780 3.56942 2.02267 2.75871 3.19971 5.59069 4.35743 490 - -

2.68618 4.42225 2.77519 2.73123 3.46354 2.40513 3.72494 3.29354 2.67741 2.69355 4.24690 2.90347 2.73739 3.18146 2.89801 2.37887 2.77519 2.98518 4.58477 3.61503

0.01769 4.43923 5.16158 0.61958 0.77255 0.80142 0.59546

247 3.40619 4.71560 5.14214 4.58728 1.49343 4.63691 4.79731 2.39775 4.43857 1.22822 3.13131 4.70382 4.85968 4.48647 4.48638 3.98371 3.63080 1.92224 5.05464 3.77244 491 - -

2.68618 4.42225 2.77519 2.73123 3.46354 2.40513 3.72494 3.29354 2.67741 2.69355 4.24690 2.90347 2.73739 3.18146 2.89801 2.37887 2.77519 2.98518 4.58477 3.61503

0.01769 4.43923 5.16158 0.61958 0.77255 0.80142 0.59546

248 2.70160 4.31833 3.78583 3.21767 3.42153 3.72282 4.01328 2.64554 3.14205 2.47462 2.52291 3.55957 4.10717 3.42558 3.43950 1.65944 2.93998 2.25050 4.91802 3.26835 492 - -

2.68618 4.42225 2.77519 2.73123 3.46354 2.40513 3.72494 3.29354 2.67741 2.69355 4.24690 2.90347 2.73739 3.18146 2.89801 2.37887 2.77519 2.98518 4.58477 3.61503

0.01769 4.43923 5.16158 0.61958 0.77255 0.80142 0.59546

249 3.49723 3.68019 4.79262 4.44050 3.15598 4.12392 4.42019 3.62151 4.13427 3.14635 4.31777 4.43441 4.68142 4.45191 4.23268 3.75510 3.84961 3.46230 0.52830 3.18050 493 - -

2.68618 4.42225 2.77519 2.73123 3.46354 2.40513 3.72494 3.29354 2.67741 2.69355 4.24690 2.90347 2.73739 3.18146 2.89801 2.37887 2.77519 2.98518 4.58477 3.61503

0.01769 4.43923 5.16158 0.61958 0.77255 0.80142 0.59546

250 2.54934 4.49822 4.96560 4.40976 3.52866 4.48067 4.90517 1.54737 4.28339 1.28120 3.37219 4.56636 4.77171 4.45799 4.40513 3.82406 3.41791 1.66989 5.37106 4.21939 494 - -

2.68618 4.42225 2.77519 2.73123 3.46354 2.40513 3.72494 3.29354 2.67741 2.69355 4.24690 2.90347 2.73739 3.18146 2.89801 2.37887 2.77519 2.98518 4.58477 3.61503

0.01769 4.43923 5.16158 0.61958 0.77255 0.35847 1.19979

251 2.75089 4.18476 4.38399 3.54820 2.90209 3.21712 4.19545 2.30867 3.64062 1.36297 3.29477 3.92697 4.24705 3.83217 3.76437 3.17660 2.59549 2.22714 4.80161 3.22487 495 - -

2.68618 4.42225 2.77519 2.73123 3.46354 2.40513 3.72494 3.29354 2.67741 2.69355 4.24690 2.90347 2.73739 3.18146 2.89801 2.37887 2.77519 2.98518 4.58477 3.61503

0.03045 4.63235 3.89930 0.61958 0.77255 0.51367 0.91204

252 2.74557 4.80001 1.72892 2.68710 3.34436 3.34641 3.84040 2.69059 2.69371 3.05877 3.90205 3.18436 3.99931 3.02939 3.13770 2.55620 2.38875 3.11575 5.34924 4.04505 496 - -

2.68618 4.42225 2.77519 2.73123 3.46354 2.40513 3.72494 3.29354 2.67741 2.69355 4.24690 2.90347 2.73739 3.18146 2.89801 2.37887 2.77519 2.98518 4.58477 3.61503

0.01479 4.61670 5.33905 0.61958 0.77255 0.54273 0.87027

253 2.66704 4.94550 3.14867 2.59555 4.19861 3.57231 3.78269 3.30852 2.49389 3.04472 4.02863 3.11144 3.41813 1.74532 3.01832 2.68682 2.04955 3.28706 5.45601 4.12447 497 - -

2.68618 4.42225 2.77519 2.73123 3.46354 2.40513 3.72494 3.29354 2.67741 2.69355 4.24690 2.90347 2.73739 3.18146 2.89801 2.37887 2.77519 2.98518 4.58477 3.61503

0.03645 4.61670 3.65317 0.61958 0.77255 0.54273 0.87027

254 2.63754 2.95411 4.05790 3.47639 2.28751 3.80513 4.09953 2.40936 3.03703 2.07538 3.35642 3.32419 3.73093 3.61694 3.59871 2.28688 2.85003 2.49896 4.85028 3.65196 498 - -

2.68618 4.42225 2.77519 2.73123 3.46354 2.40513 3.72494 3.29354 2.67741 2.69355 4.24690 2.90347 2.73739 3.18146 2.89801 2.37887 2.77519 2.98518 4.58477 3.61503

0.01512 4.59537 5.31771 0.61958 0.77255 0.53223 0.88502

255 2.64878 4.49622 3.55106 2.87916 3.42337 3.69629 3.94721 2.54207 2.80447 2.29832 3.59949 3.40876 1.90193 3.25575 3.31258 2.55003 2.75505 2.77088 5.07554 3.83382 499 - -

2.68618 4.42225 2.77519 2.73123 3.46354 2.40513 3.72494 3.29354 2.67741 2.69355 4.24690 2.90347 2.73739 3.18146 2.89801 2.37887 2.77519 2.98518 4.58477 3.61503

0.03688 4.61135 3.63931 0.61958 0.77255 0.55236 0.85705

256 2.77815 5.19579 3.23458 2.64993 4.54613 3.64676 3.77636 3.96508 1.26031 3.48004 4.27574 3.00289 4.03458 2.89797 2.26237 2.56668 3.10792 2.98910 5.62453 4.30925 500 - -

2.68618 4.42225 2.77519 2.73123 3.46354 2.40513 3.72494 3.29354 2.67741 2.69355 4.24690 2.90347 2.73739 3.18146 2.89801 2.37887 2.77519 2.98518 4.58477 3.61503

0.03842 4.58968 3.59229 0.61958 0.77255 0.59000 0.80817

257 3.08133 4.46423 4.76750 4.18145 3.14436 4.23404 4.43718 2.51447 4.01129 1.27437 2.99329 4.28666 4.54531 4.14474 4.09154 3.54539 3.30713 2.46971 1.73289 3.30066 501 - -

2.68618 4.42225 2.77519 2.73123 3.46354 2.40513 3.72494 3.29354 2.67741 2.69355 4.24690 2.90347 2.73739 3.18146 2.89801 2.37887 2.77519 2.98518 4.58477 3.61503

0.01524 4.58721 5.30955 0.61958 0.77255 0.62739 0.76353

258 2.53977 3.13792 3.13846 2.71660 2.93812 3.60799 3.40501 3.29345 2.53754 2.95341 3.80493 2.57605 3.50896 3.03538 3.13126 2.20094 2.95573 3.02112 5.25886 2.99575 502 - -

2.68618 4.42225 2.77519 2.73123 3.46354 2.40513 3.72494 3.29354 2.67741 2.69355 4.24690 2.90347 2.73739 3.18146 2.89801 2.37887 2.77519 2.98518 4.58477 3.61503

0.01524 4.58721 5.30955 0.61958 0.77255 0.50289 0.92831

259 2.75307 4.67478 3.20616 2.70722 3.58445 3.64991 3.88601 2.82120 2.69651 2.77088 3.77057 3.27794 1.54016 3.11842 3.19662 2.88160 2.75919 2.96031 5.23228 3.95803 503 - -

2.68618 4.42225 2.77519 2.73123 3.46354 2.40513 3.72494 3.29354 2.67741 2.69355 4.24690 2.90347 2.73739 3.18146 2.89801 2.37887 2.77519 2.98518 4.58477 3.61503

0.12686 4.62999 2.21286 0.61958 0.77255 0.55397 0.85488

260 2.15027 4.33553 4.61484 4.04899 3.54596 4.14907 4.55601 1.41974 3.92314 2.24905 3.48581 4.20939 4.50966 4.14082 4.07745 3.24765 2.99518 1.52267 5.16631 3.97102 504 - -

2.68618 4.42225 2.77519 2.73123 3.46354 2.40513 3.72494 3.29354 2.67741 2.69355 4.24690 2.90347 2.73739 3.18146 2.89801 2.37887 2.77519 2.98518 4.58477 3.61503

0.01632 4.51945 5.24179 0.61958 0.77255 0.40812 1.09332

261 2.74385 5.18158 2.83314 2.00906 3.82946 3.52879 3.26861 3.96298 2.46677 3.19832 4.23013 1.93282 3.49501 2.71526 2.84832 2.56584 2.87910 3.55633 4.30238 4.23966 505 - -

2.68618 4.42225 2.77519 2.73123 3.46354 2.40513 3.72494 3.29354 2.67741 2.69355 4.24690 2.90347 2.73739 3.18146 2.89801 2.37887 2.77519 2.98518 4.58477 3.61503

0.01439 4.64441 5.36676 0.61958 0.77255 0.52718 0.89225

262 2.66274 5.15198 2.44742 2.42774 4.45605 3.54045 3.37336 3.91201 2.41979 3.23950 3.91434 3.02661 3.93284 2.84231 1.67999 2.73550 2.63325 3.52058 5.60846 3.72753 506 - -

2.68618 4.42225 2.77519 2.73123 3.46354 2.40513 3.72494 3.29354 2.67741 2.69355 4.24690 2.90347 2.73739 3.18146 2.89801 2.37887 2.77519 2.98518 4.58477 3.61503

0.01439 4.64441 5.36676 0.61958 0.77255 0.52718 0.89225

263 2.32679 5.09386 3.06166 1.98450 3.55820 2.97151 3.73936 3.61929 2.13759 3.02970 4.15128 3.04083 3.63169 2.74441 2.98328 2.47782 2.57729 3.45146 5.56494 4.19414 507 - -

2.68618 4.42225 2.77519 2.73123 3.46354 2.40513 3.72494 3.29354 2.67741 2.69355 4.24690 2.90347 2.73739 3.18146 2.89801 2.37887 2.77519 2.98518 4.58477 3.61503

0.06533 4.64441 2.92564 0.61958 0.77255 0.52718 0.89225

264 2.73000 4.84426 3.18392 2.37248 3.70360 3.57673 3.79602 3.47070 2.54321 2.95757 3.93047 3.13790 2.75482 2.96703 3.07059 2.47048 1.73207 3.04468 5.37114 4.05594 508 - -

2.68618 4.42225 2.77519 2.73123 3.46354 2.40513 3.72494 3.29354 2.67741 2.69355 4.24690 2.90347 2.73739 3.18146 2.89801 2.37887 2.77519 2.98518 4.58477 3.61503

0.17267 4.59421 1.90734 0.61958 0.77255 0.61594 0.77682

265 2.88823 3.79460 2.23459 3.15366 2.48870 3.81380 3.84815 3.10450 3.12406 2.76144 3.70826 3.52084 4.19661 3.40226 3.46917 3.07653 3.12313 2.88938 4.57840 1.58928 509 - -

2.68618 4.42225 2.77519 2.73123 3.46354 2.40513 3.72494 3.29354 2.67741 2.69355 4.24690 2.90347 2.73739 3.18146 2.89801 2.37887 2.77519 2.98518 4.58477 3.61503

0.01769 4.43923 5.16158 0.61958 0.77255 0.82852 0.57393

266 2.75648 4.33556 3.94943 3.39948 3.42944 3.78297 4.14044 2.27958 3.29404 1.49242 3.39990 3.69886 2.32752 3.57951 3.55515 2.80082 3.00887 2.48606 4.97811 3.77074 510 - -

2.68618 4.42225 2.77519 2.73123 3.46354 2.40513 3.72494 3.29354 2.67741 2.69355 4.24690 2.90347 2.73739 3.18146 2.89801 2.37887 2.77519 2.98518 4.58477 3.61503

0.01769 4.43923 5.16158 0.61958 0.77255 0.82852 0.57393

267 2.74182 5.64144 1.47787 1.66914 4.94868 2.84708 3.89515 4.46919 2.82967 3.93743 4.73747 2.18909 4.01155 3.03579 3.40409 2.93930 3.30868 4.02011 6.08131 4.58834 511 - -

2.68618 4.42225 2.77519 2.73123 3.46354 2.40513 3.72494 3.29354 2.67741 2.69355 4.24690 2.90347 2.73739 3.18146 2.89801 2.37887 2.77519 2.98518 4.58477 3.61503

0.01769 4.43923 5.16158 0.61958 0.77255 0.82852 0.57393

268 2.44422 4.90743 3.30591 2.71623 4.19712 3.61639 3.77986 3.55903 2.14274 3.17853 4.02012 3.19105 4.00945 2.94191 1.58133 2.86659 2.90339 2.48930 5.41283 4.13585 512 - -

2.68618 4.42225 2.77519 2.73123 3.46354 2.40513 3.72494 3.29354 2.67741 2.69355 4.24690 2.90347 2.73739 3.18146 2.89801 2.37887 2.77519 2.98518 4.58477 3.61503

0.01769 4.43923 5.16158 0.61958 0.77255 0.82852 0.57393

269 3.48543 4.85542 4.78898 4.31838 2.90796 4.52444 4.43006 2.70973 4.08952 0.70055 3.30695 4.45730 4.80895 4.28610 4.20621 3.90017 3.71578 2.80166 4.72133 2.47993 513 - -

2.68618 4.42225 2.77519 2.73123 3.46354 2.40513 3.72494 3.29354 2.67741 2.69355 4.24690 2.90347 2.73739 3.18146 2.89801 2.37887 2.77519 2.98518 4.58477 3.61503

0.01769 4.43923 5.16158 0.61958 0.77255 0.82852 0.57393

270 2.22599 5.16633 2.35769 2.39376 4.49276 3.46402 3.33757 3.96483 2.06371 3.46371 4.21683 2.84034 3.86792 2.77318 2.73740 2.09057 2.93744 3.54798 5.61145 4.21214 514 - -

2.68618 4.42225 2.77519 2.73123 3.46354 2.40513 3.72494 3.29354 2.67741 2.69355 4.24690 2.90347 2.73739 3.18146 2.89801 2.37887 2.77519 2.98518 4.58477 3.61503

0.01769 4.43923 5.16158 0.61958 0.77255 0.82852 0.57393

271 2.22115 4.93280 2.70594 1.87724 4.18567 3.50008 3.70208 3.40636 2.48810 2.53837 3.57763 3.01840 3.89187 2.84163 2.76483 2.70278 2.91372 3.26966 5.42564 4.07888 515 - -

2.68627 4.42234 2.77529 2.73132 3.46363 2.40522 3.72503 3.29363 2.67716 2.69364 4.24699 2.90356 2.73749 3.18155 2.89701 2.37896 2.77514 2.98527 4.58486 3.61512

0.98456 0.91703 1.48416 0.11739 2.20037 0.31899 1.29786

272 2.56110 4.12390 4.32230 3.72813 1.99930 3.81748 4.11883 2.37825 3.33693 2.18087 3.23840 3.86201 4.18411 3.76838 3.27754 3.11334 2.92225 2.17655 4.02100 2.42493 517 - -

2.68618 4.42225 2.77519 2.73123 3.46354 2.40513 3.72494 3.29354 2.67741 2.69355 4.24690 2.90347 2.73739 3.18146 2.89801 2.37887 2.77519 2.98518 4.58477 3.61503

0.15712 4.47175 2.01013 0.61958 0.77255 0.79003 0.60483

273 2.76910 5.22349 2.18168 1.43608 3.86776 3.42527 3.71010 4.00135 2.51536 3.51454 4.29056 2.89987 3.88280 2.83244 2.87798 2.57994 3.01179 3.59574 5.67588 4.26242 518 - -

2.68618 4.42225 2.77519 2.73123 3.46354 2.40513 3.72494 3.29354 2.67741 2.69355 4.24690 2.90347 2.73739 3.18146 2.89801 2.37887 2.77519 2.98518 4.58477 3.61503

0.01967 4.33430 5.05665 0.61958 0.77255 0.71185 0.67478
274 2.57251 5.05574 2.77103 2.42831 4.34961 3.47480 3.66762 3.38964 2.23062 3.33864 3.66270 2.74205 3.86687 2.64048 2.23789 2.34245 2.58704 3.21464 5.52020 4.14335 519 - -
2.68618 4.42225 2.77519 2.73123 3.46354 2.40513 3.72494 3.29354 2.67741 2.69355 4.24690 2.90347 2.73739 3.18146 2.89801 2.37887 2.77519 2.98518 4.58477 3.61503
0.01775 4.43580 5.15815 0.61958 0.77255 0.83243 0.57092
275 2.69174 5.14352 2.51670 1.93342 4.46289 3.03965 3.66093 3.93240 2.32899 3.43762 3.56348 2.93871 2.56754 2.59789 2.90013 2.66417 2.92385 3.52049 5.59096 3.78730 520 - -
2.68618 4.42225 2.77519 2.73123 3.46354 2.40513 3.72494 3.29354 2.67741 2.69355 4.24690 2.90347 2.73739 3.18146 2.89801 2.37887 2.77519 2.98518 4.58477 3.61503
0.01775 4.43580 5.15815 0.61958 0.77255 0.83243 0.57092
276 2.60489 5.00269 2.76998 2.38688 4.29761 2.20772 3.69944 3.51668 2.47703 3.29891 4.08541 2.55477 3.88236 2.82721 2.95521 2.23296 2.74553 3.03593 5.50137 4.13661 521 - -
2.68640 4.42247 2.77527 2.73145 3.46376 2.40534 3.72516 3.29376 2.67745 2.69359 4.24711 2.90289 2.73645 3.18168 2.89801 2.37884 2.77490 2.98540 4.58499 3.61525
0.61368 1.61948 1.34461 1.42955 0.27367 0.31752 1.30176
277 2.75800 5.17151 3.00193 1.97766 4.50212 2.38979 3.68341 3.95542 2.01982 3.45576 4.22448 2.99432 3.90987 2.79409 2.19778 2.73296 2.98358 3.55667 5.59811 3.62253 527 - -
2.68618 4.42225 2.77519 2.73123 3.46354 2.40513 3.72494 3.29354 2.67741 2.69355 4.24690 2.90347 2.73739 3.18146 2.89801 2.37887 2.77519 2.98518 4.58477 3.61503
0.01769 4.43923 5.16158 0.61958 0.77255 0.82852 0.57393
278 2.73799 4.97566 3.10980 2.54764 4.25135 3.19032 3.71726 3.66879 2.10094 3.24379 4.05104 3.06149 2.38432 2.72609 2.33908 2.75733 2.96539 2.46213 5.45403 4.12571 528 - -
2.68618 4.42225 2.77519 2.73123 3.46354 2.40513 3.72494 3.29354 2.67741 2.69355 4.24690 2.90347 2.73739 3.18146 2.89801 2.37887 2.77519 2.98518 4.58477 3.61503
0.01769 4.43923 5.16158 0.61958 0.77255 0.82852 0.57393
279 2.75774 5.11603 2.88920 2.18771 4.38898 3.48695 2.66016 3.85685 2.49977 3.40006 4.18801 2.67330 3.91345 2.85458 2.97297 1.63790 2.99456 3.48253 5.57623 3.84662 529 - -
2.68618 4.42225 2.77519 2.73123 3.46354 2.40513 3.72494 3.29354 2.67741 2.69355 4.24690 2.90347 2.73739 3.18146 2.89801 2.37887 2.77519 2.98518 4.58477 3.61503
0.01769 4.43923 5.16158 0.61958 0.77255 0.31899 1.29786
280 2.64398 5.08315 1.81470 2.36473 3.92457 3.16606 3.75096 3.12412 2.41593 2.95128 3.83918 3.05513 3.94668 2.52719 2.90044 2.75124 2.98215 3.25513 5.55813 4.19170 530 - -
2.68618 4.42225 2.77519 2.73123 3.46354 2.40513 3.72494 3.29354 2.67741 2.69355 4.24690 2.90347 2.73739 3.18146 2.89801 2.37887 2.77519 2.98518 4.58477 3.61503
0.01408 4.66559 5.38793 0.61958 0.77255 0.48576 0.95510
281 2.97135 4.35543 4.74883 4.15406 2.51909 3.77035 4.47759 2.26995 3.98681 1.04379 3.28049 4.24583 4.48418 4.13534 4.06100 3.45940 2.89107 2.20397 4.98096 3.81714 531 - -
2.68618 4.42225 2.77519 2.73123 3.46354 2.40513 3.72494 3.29354 2.67741 2.69355 4.24690 2.90347 2.73739 3.18146 2.89801 2.37887 2.77519 2.98518 4.58477 3.61503
0.01408 4.66559 5.38793 0.61958 0.77255 0.48576 0.95510
282 2.15590 5.12113 2.91003 2.29243 4.41288 3.19312 3.37419 3.86100 2.51816 3.40581 3.89574 2.94744 1.94431 2.87260 2.99970 2.61206 2.99246 3.31681 5.59359 4.21906 532 - -
2.68618 4.42225 2.77519 2.73123 3.46354 2.40513 3.72494 3.29354 2.67741 2.69355 4.24690 2.90347 2.73739 3.18146 2.89801 2.37887 2.77519 2.98518 4.58477 3.61503
0.01408 4.66559 5.38793 0.61958 0.77255 0.48576 0.95510
283 2.56780 4.81877 3.24404 2.68453 3.76453 2.47464 3.82907 3.26765 2.41160 2.85022 3.67881 3.18521 2.75589 2.81011 2.79990 2.33497 2.98279 2.85022 3.95244 4.04176 533 - -
2.68618 4.42225 2.77519 2.73123 3.46354 2.40513 3.72494 3.29354 2.67741 2.69355 4.24690 2.90347 2.73739 3.18146 2.89801 2.37887 2.77519 2.98518 4.58477 3.61503
0.01408 4.66559 5.38793 0.61958 0.77255 0.48576 0.95510
284 2.88272 4.27136 4.11777 4.05621 3.19695 4.06538 4.40752 1.85928 3.89584 1.84742 3.19992 4.15317 4.41930 4.07238 3.98829 3.00096 3.11729 1.32993 4.96085 3.77796 534 - -
2.68624 4.42231 2.77525 2.73129 3.46360 2.40501 3.72500 3.29360 2.67747 2.69361 4.24696 2.90352 2.73745 3.18152 2.89807 2.37888 2.77512 2.98524 4.58483 3.61391
0.24404 1.55128 5.38793 0.16271 1.89606 0.48576 0.95510
285 2.65190 4.76742 1.48160 2.75334 3.95519 3.64902 3.87394 3.14829 2.73926 2.86176 3.09174 3.24297 4.03728 3.07632 3.17292 2.86882 2.76918 2.81282 5.31657 3.69758 536 - -

2.68618 4.42225 2.77519 2.73123 3.46354 2.40513 3.72494 3.29354 2.67741 2.69355 4.24690 2.90347 2.73739 3.18146 2.89801 2.37887 2.77519 2.98518 4.58477 3.61503

0.01408 4.66559 5.38793 0.61958 0.77255 0.48576 0.95510

286 2.63697 4.16473 4.54382 3.94041 3.06978 3.41729 4.23373 2.42185 3.76319 2.24349 2.32280 4.01081 4.27490 3.92824 3.50619 3.21332 2.99279 1.46320 3.16481 3.60363 537 - -

2.68618 4.42225 2.77519 2.73123 3.46354 2.40513 3.72494 3.29354 2.67741 2.69355 4.24690 2.90347 2.73739 3.18146 2.89801 2.37887 2.77519 2.98518 4.58477 3.61503

0.01408 4.66559 5.38793 0.61958 0.77255 0.48576 0.95510

287 2.98557 4.42083 4.47184 3.90628 1.16937 4.06096 4.11350 2.85714 3.43837 2.34268 3.51478 4.02462 4.42008 3.92995 3.55882 3.36259 3.04529 2.68247 2.60620 3.12473 538 - -

2.68618 4.42225 2.77519 2.73123 3.46354 2.40513 3.72494 3.29354 2.67741 2.69355 4.24690 2.90347 2.73739 3.18146 2.89801 2.37887 2.77519 2.98518 4.58477 3.61503

0.04398 4.66559 3.39280 0.61958 0.77255 0.48576 0.95510

288 2.76958 4.34764 3.64953 2.99906 3.46964 3.81413 4.09374 2.43826 3.25197 2.21924 3.45671 3.66442 4.18834 3.31826 3.34041 3.08376 3.00179 1.29471 4.95916 3.74944 539 - -

2.68618 4.42225 2.77519 2.73123 3.46354 2.40513 3.72494 3.29354 2.67741 2.69355 4.24690 2.90347 2.73739 3.18146 2.89801 2.37887 2.77519 2.98518 4.58477 3.61503

0.01451 4.63611 5.35846 0.61958 0.77255 0.45172 1.01206

289 2.75288 3.24146 3.21395 2.43007 4.09927 3.59894 3.82389 3.50307 2.64724 2.76131 3.96053 3.16526 2.96200 2.69476 3.09711 1.68498 2.72245 3.19939 5.40082 4.08578 540 - -

2.68618 4.42225 2.77519 2.73123 3.46354 2.40513 3.72494 3.29354 2.67741 2.69355 4.24690 2.90347 2.73739 3.18146 2.89801 2.37887 2.77519 2.98518 4.58477 3.61503

0.01408 4.66559 5.38793 0.61958 0.77255 0.48576 0.95510

290 3.03788 4.49264 4.48250 3.91368 3.03857 4.17481 4.51701 2.19793 3.80498 2.05768 3.42861 4.15252 4.52556 4.02988 4.00689 3.47890 1.13897 2.26384 5.16863 3.97552 541 - -

2.68618 4.42225 2.77519 2.73123 3.46354 2.40513 3.72494 3.29354 2.67741 2.69355 4.24690 2.90347 2.73739 3.18146 2.89801 2.37887 2.77519 2.98518 4.58477 3.61503

0.01408 4.66559 5.38793 0.61958 0.77255 0.48576 0.95510

291 1.52425 4.73287 3.53616 2.97224 4.00690 3.69248 3.98443 3.31987 2.65013 3.03362 3.91076 3.40288 4.12045 3.21256 2.05127 2.96672 3.08385 2.12332 5.35753 4.10487 542 - -

2.68618 4.42225 2.77519 2.73123 3.46354 2.40513 3.72494 3.29354 2.67741 2.69355 4.24690 2.90347 2.73739 3.18146 2.89801 2.37887 2.77519 2.98518 4.58477 3.61503

0.01408 4.66559 5.38793 0.61958 0.77255 0.48576 0.95510

292 2.29474 5.00532 1.40825 2.59754 3.86895 3.56772 3.82874 3.49354 2.63514 3.31256 4.12184 3.00253 3.98997 2.97543 3.10564 2.60359 3.02405 3.16066 5.54613 4.19890 543 - -

2.68618 4.42225 2.77519 2.73123 3.46354 2.40513 3.72494 3.29354 2.67741 2.69355 4.24690 2.90347 2.73739 3.18146 2.89801 2.37887 2.77519 2.98518 4.58477 3.61503

0.01408 4.66559 5.38793 0.61958 0.77255 0.48576 0.95510

293 2.59943 4.83455 2.84700 2.57862 3.77673 3.61101 3.83469 3.24860 2.67382 2.97457 3.92301 3.18287 1.60616 3.01544 3.12052 2.70631 2.89151 2.91520 5.36892 4.06105 544 - -

2.68618 4.42225 2.77519 2.73123 3.46354 2.40513 3.72494 3.29354 2.67741 2.69355 4.24690 2.90347 2.73739 3.18146 2.89801 2.37887 2.77519 2.98518 4.58477 3.61503

0.01408 4.66559 5.38793 0.61958 0.77255 0.48576 0.95510

294 2.79055 4.27116 4.22351 2.43077 2.89468 3.89072 4.18339 2.67690 3.52348 1.88320 3.06148 3.85854 4.25664 3.74605 3.71257 3.17593 3.02229 1.80573 4.86570 2.42993 545 - -

2.68618 4.42225 2.77519 2.73123 3.46354 2.40513 3.72494 3.29354 2.67741 2.69355 4.24690 2.90347 2.73739 3.18146 2.89801 2.37887 2.77519 2.98518 4.58477 3.61503

0.01408 4.66559 5.38793 0.61958 0.77255 0.48576 0.95510

295 2.82847 4.75161 3.45330 2.87278 3.65773 3.71917 3.91062 2.36177 1.54663 2.56079 3.82410 3.33581 4.09749 3.13141 2.36885 2.95294 3.05406 2.88293 5.28811 4.02383 546 - -

2.68618 4.42225 2.77519 2.73123 3.46354 2.40513 3.72494 3.29354 2.67741 2.69355 4.24690 2.90347 2.73739 3.18146 2.89801 2.37887 2.77519 2.98518 4.58477 3.61503

0.01408 4.66559 5.38793 0.61958 0.77255 0.48576 0.95510

296 2.31920 4.92164 3.22760 1.25111 4.21923 3.64824 3.95313 3.16679 2.77608 3.17887 4.06620 3.25144 4.08349 3.12357 3.22063 2.91363 3.09351 2.43124 5.55495 4.23562 547 - -

2.68618 4.42225 2.77519 2.73123 3.46354 2.40513 3.72494 3.29354 2.67741 2.69355 4.24690 2.90347 2.73739 3.18146 2.89801 2.37887 2.77519 2.98518 4.58477 3.61503

0.01408 4.66559 5.38793 0.61958 0.77255 0.48576 0.95510

297 2.98191 4.88644 3.92802 3.86668 5.02913 3.58010 5.00547 4.65053 4.02707 4.31049 5.22029 4.00951 0.41877 4.35461 4.25631 2.88999 3.51109 4.04039 6.31686 5.12416 548 - -
2.68618 4.42225 2.77519 2.73123 3.46354 2.40513 3.72494 3.29354 2.67741 2.69355 4.24690 2.90347 2.73739 3.18146 2.89801 2.37887 2.77519 2.98518 4.58477 3.61503
0.01408 4.66559 5.38793 0.61958 0.77255 0.48576 0.95510
298 2.81924 4.59749 4.11045 3.58489 3.73136 3.93623 4.42912 2.47200 3.47426 2.16165 3.64647 3.89708 1.12663 3.79226 3.76357 3.26399 3.24632 2.45052 5.35768 4.14633 549 - -
2.68618 4.42225 2.77519 2.73123 3.46354 2.40513 3.72494 3.29354 2.67741 2.69355 4.24690 2.90347 2.73739 3.18146 2.89801 2.37887 2.77519 2.98518 4.58477 3.61503
0.01408 4.66559 5.38793 0.61958 0.77255 0.48576 0.95510
299 3.77617 5.01866 5.70193 5.11140 2.74890 5.11526 5.41113 1.69677 4.96584 0.76582 2.51696 5.26701 5.20708 4.87313 4.92326 4.48374 3.98318 2.52250 5.50262 4.51679 550 - -
2.68618 4.42225 2.77519 2.73123 3.46354 2.40513 3.72494 3.29354 2.67741 2.69355 4.24690 2.90347 2.73739 3.18146 2.89801 2.37887 2.77519 2.98518 4.58477 3.61503
0.01408 4.66559 5.38793 0.61958 0.77255 0.48576 0.95510
300 2.77298 4.31879 4.01258 3.43137 3.42895 3.83536 4.11715 1.95638 2.51621 2.30771 2.68887 3.72319 4.20640 2.97792 3.37918 3.10840 3.00440 1.80667 4.92532 3.72303 551 - -
2.68618 4.42225 2.77519 2.73123 3.46354 2.40513 3.72494 3.29354 2.67741 2.69355 4.24690 2.90347 2.73739 3.18146 2.89801 2.37887 2.77519 2.98518 4.58477 3.61503
0.01408 4.66559 5.38793 0.61958 0.77255 0.48576 0.95510
301 2.57478 3.43114 4.20590 3.62204 2.99272 3.84981 4.16984 2.68487 3.50521 2.30878 3.36683 3.83441 4.22787 3.72956 3.42443 2.80974 1.69737 1.82297 4.86589 3.67301 552 - -
2.68618 4.42225 2.77519 2.73123 3.46354 2.40513 3.72494 3.29354 2.67741 2.69355 4.24690 2.90347 2.73739 3.18146 2.89801 2.37887 2.77519 2.98518 4.58477 3.61503
0.01408 4.66559 5.38793 0.61958 0.77255 0.48576 0.95510
302 1.64381 4.30729 4.17809 3.61032 3.46893 3.57971 4.23218 2.59625 3.50620 1.89925 3.46274 3.84017 4.25121 3.74777 3.72250 2.77622 3.03534 1.78141 4.98235 3.78530 553 - -
2.68618 4.42225 2.77519 2.73123 3.46354 2.40513 3.72494 3.29354 2.67741 2.69355 4.24690 2.90347 2.73739 3.18146 2.89801 2.37887 2.77519 2.98518 4.58477 3.61503
0.01408 4.66559 5.38793 0.61958 0.77255 0.48576 0.95510
303 2.64198 4.92005 2.99934 2.72956 4.34656 3.54223 3.96696 3.74468 2.81090 3.37701 4.19929 1.29766 4.03632 3.13571 3.26673 2.50264 2.88392 2.46170 5.62801 4.30268 554 - -
2.68618 4.42225 2.77519 2.73123 3.46354 2.40513 3.72494 3.29354 2.67741 2.69355 4.24690 2.90347 2.73739 3.18146 2.89801 2.37887 2.77519 2.98518 4.58477 3.61503
0.01408 4.66559 5.38793 0.61958 0.77255 0.48576 0.95510
304 2.69074 4.81428 3.28553 2.72932 4.02225 3.63627 3.85198 3.41421 2.51875 2.93296 3.90577 2.77373 4.02771 3.04289 2.94009 2.85946 1.39337 3.01692 5.34745 3.54073 555 - -
2.68618 4.42225 2.77519 2.73123 3.46354 2.40513 3.72494 3.29354 2.67741 2.69355 4.24690 2.90347 2.73739 3.18146 2.89801 2.37887 2.77519 2.98518 4.58477 3.61503
0.01408 4.66559 5.38793 0.61958 0.77255 0.48576 0.95510
305 2.50548 4.26498 4.76075 4.16518 2.74609 3.64961 4.44552 1.70900 3.98709 2.05324 3.19548 4.21786 4.44593 4.14560 4.04563 3.40936 3.13043 1.41412 4.96224 3.78270 556 - -
2.68618 4.42225 2.77519 2.73123 3.46354 2.40513 3.72494 3.29354 2.67741 2.69355 4.24690 2.90347 2.73739 3.18146 2.89801 2.37887 2.77519 2.98518 4.58477 3.61503
0.01408 4.66559 5.38793 0.61958 0.77255 0.48576 0.95510
306 3.36290 1.93063 5.15902 4.67101 3.56119 4.40339 5.05456 2.63673 4.44748 0.75688 3.46163 4.71810 4.83724 4.62072 4.51746 3.84307 3.66876 2.63321 5.45432 4.29161 557 - -
2.68618 4.42225 2.77519 2.73123 3.46354 2.40513 3.72494 3.29354 2.67741 2.69355 4.24690 2.90347 2.73739 3.18146 2.89801 2.37887 2.77519 2.98518 4.58477 3.61503
0.01408 4.66559 5.38793 0.61958 0.77255 0.48576 0.95510
307 2.78766 4.52876 3.67661 3.13029 3.70288 3.73431 4.06322 2.51783 3.05626 2.26032 3.66113 3.52244 4.15107 3.17206 3.39907 1.30652 2.87506 2.78222 5.16011 3.92401 558 - -
2.68618 4.42225 2.77519 2.73123 3.46354 2.40513 3.72494 3.29354 2.67741 2.69355 4.24690 2.90347 2.73739 3.18146 2.89801 2.37887 2.77519 2.98518 4.58477 3.61503
0.01408 4.66559 5.38793 0.61958 0.77255 0.48576 0.95510
308 3.49145 4.72076 5.51279 5.00507 3.93728 5.00285 5.58425 1.07493 4.91111 1.77209 3.71236 5.14296 5.23825 5.10534 5.03133 4.39806 3.43104 1.21319 5.93674 4.76163 559 - -
2.68618 4.42225 2.77519 2.73123 3.46354 2.40513 3.72494 3.29354 2.67741 2.69355 4.24690 2.90347 2.73739 3.18146 2.89801 2.37887 2.77519 2.98518 4.58477 3.61503

0.01408 4.66559 5.38793 0.61958 0.77255 0.48576 0.95510

309 3.56364 4.86680 5.34859 4.76344 3.38085 4.83610 5.15657 2.30253 4.61546 0.71946 2.16476 4.93465 5.02265 4.65106 4.65760 4.18086 3.20959 2.45508 5.41948 4.38236 560 - -

2.68618 4.42225 2.77519 2.73123 3.46354 2.40513 3.72494 3.29354 2.67741 2.69355 4.24690 2.90347 2.73739 3.18146 2.89801 2.37887 2.77519 2.98518 4.58477 3.61503

0.01408 4.66559 5.38793 0.61958 0.77255 0.48576 0.95510

310 1.02784 4.49493 3.99651 3.54622 2.58933 3.51087 4.41492 3.43186 3.51203 3.16213 4.06066 3.73948 4.14834 3.78592 3.81003 1.89046 3.08363 3.10691 5.46839 4.20474 561 - -

2.68618 4.42225 2.77519 2.73123 3.46354 2.40513 3.72494 3.29354 2.67741 2.69355 4.24690 2.90347 2.73739 3.18146 2.89801 2.37887 2.77519 2.98518 4.58477 3.61503

0.01408 4.66559 5.38793 0.61958 0.77255 0.48576 0.95510

311 2.75639 4.36878 4.72685 4.14056 3.40242 4.17490 4.51152 2.41375 3.97993 1.64877 3.15965 4.25419 4.51514 4.15538 4.07869 3.48818 3.22013 1.16216 5.04200 3.01661 562 - -

2.68618 4.42225 2.77519 2.73123 3.46354 2.40513 3.72494 3.29354 2.67741 2.69355 4.24690 2.90347 2.73739 3.18146 2.89801 2.37887 2.77519 2.98518 4.58477 3.61503

0.01408 4.66559 5.38793 0.61958 0.77255 0.48576 0.95510

312 2.75690 5.00623 1.81262 2.33351 4.25839 3.57418 3.77775 3.09814 2.56632 2.81299 3.38504 2.44003 3.96658 2.91760 3.03616 2.77775 2.98852 3.03222 5.50048 4.15327 563 - -

2.68618 4.42225 2.77519 2.73123 3.46354 2.40513 3.72494 3.29354 2.67741 2.69355 4.24690 2.90347 2.73739 3.18146 2.89801 2.37887 2.77519 2.98518 4.58477 3.61503

0.01408 4.66559 5.38793 0.61958 0.77255 0.48576 0.95510

313 3.11177 4.65242 4.19556 3.65440 2.78889 4.10354 4.04425 3.06288 3.53732 2.24746 3.68922 3.90500 4.46068 2.62686 3.78489 3.38540 3.34068 2.65562 4.56458 1.11990 564 - -

2.68618 4.42225 2.77519 2.73123 3.46354 2.40513 3.72494 3.29354 2.67741 2.69355 4.24690 2.90347 2.73739 3.18146 2.89801 2.37887 2.77519 2.98518 4.58477 3.61503

0.01408 4.66559 5.38793 0.61958 0.77255 0.48576 0.95510

314 2.79807 4.58017 3.77720 3.29798 2.58078 3.66635 4.17607 3.26169 3.25757 2.79002 3.87746 3.62245 1.23721 3.56359 3.58771 2.31442 3.11765 3.00587 5.16766 3.80691 565 - -

2.68618 4.42225 2.77519 2.73123 3.46354 2.40513 3.72494 3.29354 2.67741 2.69355 4.24690 2.90347 2.73739 3.18146 2.89801 2.37887 2.77519 2.98518 4.58477 3.61503

0.01408 4.66559 5.38793 0.61958 0.77255 0.48576 0.95510

315 2.18253 3.84634 4.34966 3.76700 3.38545 3.90345 4.25755 2.30986 3.63765 2.26532 3.38813 3.93847 3.63719 3.84879 3.79709 2.72742 3.03348 1.33238 4.91088 3.72105 566 - -

2.68618 4.42225 2.77519 2.73123 3.46354 2.40513 3.72494 3.29354 2.67741 2.69355 4.24690 2.90347 2.73739 3.18146 2.89801 2.37887 2.77519 2.98518 4.58477 3.61503

0.01408 4.66559 5.38793 0.61958 0.77255 0.48576 0.95510

316 3.27713 5.94269 1.42814 1.30235 5.22860 3.57748 3.58218 4.77313 3.08274 4.22464 5.04626 1.98370 4.18297 3.23840 3.69027 3.14967 3.55643 4.31114 6.36364 4.83238 567 - -

2.68618 4.42225 2.77519 2.73123 3.46354 2.40513 3.72494 3.29354 2.67741 2.69355 4.24690 2.90347 2.73739 3.18146 2.89801 2.37887 2.77519 2.98518 4.58477 3.61503

0.01408 4.66559 5.38793 0.61958 0.77255 0.48576 0.95510

317 2.90852 5.36613 3.48031 2.84885 4.79621 3.51997 3.85606 4.18508 1.04534 3.65039 4.46268 3.31704 4.18334 2.97593 1.88763 2.94104 3.29670 3.49177 5.73753 4.48105 568 - -

2.68618 4.42225 2.77519 2.73123 3.46354 2.40513 3.72494 3.29354 2.67741 2.69355 4.24690 2.90347 2.73739 3.18146 2.89801 2.37887 2.77519 2.98518 4.58477 3.61503

0.01408 4.66559 5.38793 0.61958 0.77255 0.48576 0.95510

318 3.13970 4.70349 5.36469 4.81003 3.63189 4.80415 5.23502 1.79897 4.67858 1.10085 3.17015 4.93286 5.04327 4.79372 4.74645 4.16386 3.66688 1.35324 5.57720 4.45945 569 - -

2.68618 4.42225 2.77519 2.73123 3.46354 2.40513 3.72494 3.29354 2.67741 2.69355 4.24690 2.90347 2.73739 3.18146 2.89801 2.37887 2.77519 2.98518 4.58477 3.61503

0.01408 4.66559 5.38793 0.61958 0.77255 0.48576 0.95510

319 1.67181 4.41633 3.79708 3.23516 3.57198 3.74601 4.07598 2.66426 3.17390 2.65349 3.55964 3.58748 4.15329 3.46129 3.48998 1.97664 3.00430 2.39220 5.04155 2.68939 570 - -

2.68618 4.42225 2.77519 2.73123 3.46354 2.40513 3.72494 3.29354 2.67741 2.69355 4.24690 2.90347 2.73739 3.18146 2.89801 2.37887 2.77519 2.98518 4.58477 3.61503

0.24404 4.66559 1.57439 0.61958 0.77255 0.48576 0.95510

320 2.44778 1.21362 4.65011 4.22770 3.90754 3.63149 4.79375 2.51055 4.07402 2.76606 3.83473 4.13250 4.28866 4.30478 4.21006 3.06824 3.12050 1.66541 5.53949 4.35384 571 - -

2.68618 4.42225 2.77519 2.73123 3.46354 2.40513 3.72494 3.29354 2.67741 2.69355 4.24690 2.90347 2.73739 3.18146 2.89801 2.37887 2.77519 2.98518 4.58477 3.61503
0.01769 4.43923 5.16158 0.61958 0.77255 0.82852 0.57393
321 4.16234 5.41687 4.72563 4.58941 2.54916 4.52990 4.03481 4.11093 4.43773 3.40970 4.72737 4.48949 5.00313 4.59431 4.48950 4.22751 4.45858 4.01096 4.20357 0.37728 572 - -
2.68618 4.42225 2.77519 2.73123 3.46354 2.40513 3.72494 3.29354 2.67741 2.69355 4.24690 2.90347 2.73739 3.18146 2.89801 2.37887 2.77519 2.98518 4.58477 3.61503
0.01769 4.43923 5.16158 0.61958 0.77255 0.82852 0.57393
322 3.39745 4.64123 5.31666 4.80483 3.73961 4.87373 5.42127 1.66878 4.70416 1.72486 3.51568 4.98312 5.10321 4.89756 4.84313 4.26575 3.64714 0.86492 5.77532 4.61169 573 - -
2.68618 4.42225 2.77519 2.73123 3.46354 2.40513 3.72494 3.29354 2.67741 2.69355 4.24690 2.90347 2.73739 3.18146 2.89801 2.37887 2.77519 2.98518 4.58477 3.61503
0.01769 4.43923 5.16158 0.61958 0.77255 0.82852 0.57393
323 2.70195 4.61115 3.82674 3.70866 4.72121 3.33659 4.77796 4.34378 3.82588 4.02894 4.92575 3.80516 4.12801 4.13001 4.06316 0.48023 3.23013 3.74141 6.04877 4.82114 574 - -
2.68618 4.42225 2.77519 2.73123 3.46354 2.40513 3.72494 3.29354 2.67741 2.69355 4.24690 2.90347 2.73739 3.18146 2.89801 2.37887 2.77519 2.98518 4.58477 3.61503
0.01769 4.43923 5.16158 0.61958 0.77255 0.82852 0.57393
324 3.72053 5.82235 0.36152 2.87896 5.30435 3.75378 4.57979 5.04865 3.79003 4.56099 5.58616 3.45210 4.44567 3.84283 4.35703 3.64143 4.08129 4.63621 6.34279 5.12827 575 - -
2.68618 4.42225 2.77519 2.73123 3.46354 2.40513 3.72494 3.29354 2.67741 2.69355 4.24690 2.90347 2.73739 3.18146 2.89801 2.37887 2.77519 2.98518 4.58477 3.61503
0.01769 4.43923 5.16158 0.61958 0.77255 0.82852 0.57393
325 3.72053 5.82235 0.36152 2.87896 5.30435 3.75378 4.57979 5.04865 3.79003 4.56099 5.58616 3.45210 4.44567 3.84283 4.35703 3.64143 4.08129 4.63621 6.34279 5.12827 576 - -
2.68618 4.42225 2.77519 2.73123 3.46354 2.40513 3.72494 3.29354 2.67741 2.69355 4.24690 2.90347 2.73739 3.18146 2.89801 2.37887 2.77519 2.98518 4.58477 3.61503
0.01769 4.43923 5.16158 0.61958 0.77255 0.82852 0.57393
326 3.51828 5.20009 4.30253 4.28728 5.34898 0.22156 5.33274 5.12505 4.54185 4.68865 5.69448 4.46986 4.60310 4.82206 4.67987 3.71146 4.04057 4.54346 6.25711 5.48224 577 - -
2.68618 4.42225 2.77519 2.73123 3.46354 2.40513 3.72494 3.29354 2.67741 2.69355 4.24690 2.90347 2.73739 3.18146 2.89801 2.37887 2.77519 2.98518 4.58477 3.61503
0.01769 4.43923 5.16158 0.61958 0.77255 0.82852 0.57393
327 1.58098 2.11974 4.22514 3.86055 4.45792 1.37887 4.67271 3.85769 3.79914 3.57378 4.40441 3.75713 3.94850 4.02982 4.02322 2.28854 2.91848 3.31874 5.85401 4.69910 578 - -
2.68618 4.42225 2.77519 2.73123 3.46354 2.40513 3.72494 3.29354 2.67741 2.69355 4.24690 2.90347 2.73739 3.18146 2.89801 2.37887 2.77519 2.98518 4.58477 3.61503
0.01769 4.43923 5.16158 0.61958 0.77255 0.82852 0.57393
328 1.18430 4.38289 3.91026 3.64626 4.70045 3.16700 4.65438 4.10433 3.68803 3.82123 4.63812 3.67101 3.95606 3.94931 3.95996 1.11919 2.96746 3.48650 6.04816 4.86415 579 - -
2.68618 4.42225 2.77519 2.73123 3.46354 2.40513 3.72494 3.29354 2.67741 2.69355 4.24690 2.90347 2.73739 3.18146 2.89801 2.37887 2.77519 2.98518 4.58477 3.61503
0.01769 4.43923 5.16158 0.61958 0.77255 0.82852 0.57393
329 2.68269 4.35178 3.64662 2.84044 3.47805 3.68205 3.94759 2.65124 3.01735 2.03265 2.29620 3.45919 2.68835 3.31466 3.34482 2.55702 2.91560 2.60843 4.94345 3.71951 580 - -
2.68618 4.42225 2.77519 2.73123 3.46354 2.40513 3.72494 3.29354 2.67741 2.69355 4.24690 2.90347 2.73739 3.18146 2.89801 2.37887 2.77519 2.98518 4.58477 3.61503
0.01769 4.43923 5.16158 0.61958 0.77255 0.82852 0.57393
330 3.63942 4.96625 5.23726 4.75627 3.31450 4.82819 5.26204 2.38113 4.54426 0.56543 3.12350 4.97125 5.05779 4.67206 4.62774 4.25952 3.89479 2.26002 5.46938 4.36938 581 - -
2.68618 4.42225 2.77519 2.73123 3.46354 2.40513 3.72494 3.29354 2.67741 2.69355 4.24690 2.90347 2.73739 3.18146 2.89801 2.37887 2.77519 2.98518 4.58477 3.61503
0.01769 4.43923 5.16158 0.61958 0.77255 0.82852 0.57393
331 2.57390 4.45590 3.72263 3.31719 4.15708 3.35606 4.30432 3.44049 3.24646 2.83247 4.12133 3.55280 4.01492 3.59475 3.55703 2.22284 0.99956 3.08682 5.57101 4.33028 582 - -
2.68618 4.42225 2.77519 2.73123 3.46354 2.40513 3.72494 3.29354 2.67741 2.69355 4.24690 2.90347 2.73739 3.18146 2.89801 2.37887 2.77519 2.98518 4.58477 3.61503
0.01769 4.43923 5.16158 0.61958 0.77255 0.82852 0.57393

332 4.04000 5.18691 5.15383 4.88698 0.80823 4.79593 3.75884 3.59224 4.69468 2.41813 4.16956 4.46680 5.07126 4.54789 4.63101 4.16695 4.25049 3.55324 3.86803 1.47989 583 - -
2.68618 4.42225 2.77519 2.73123 3.46354 2.40513 3.72494 3.29354 2.67741 2.69355 4.24690 2.90347 2.73739 3.18146 2.89801 2.37887 2.77519 2.98518 4.58477 3.61503
0.01769 4.43923 5.16158 0.61958 0.77255 0.82852 0.57393
333 2.78702 4.89539 2.81070 1.57200 2.25562 3.56427 3.78024 3.50591 2.68203 3.11860 3.97841 3.10953 3.98613 3.00295 3.13766 2.83356 3.02372 3.21409 5.20956 3.31034 584 - -
2.68618 4.42225 2.77519 2.73123 3.46354 2.40513 3.72494 3.29354 2.67741 2.69355 4.24690 2.90347 2.73739 3.18146 2.89801 2.37887 2.77519 2.98518 4.58477 3.61503
0.01769 4.43923 5.16158 0.61958 0.77255 0.82852 0.57393
334 1.03229 4.37065 3.93410 3.60823 4.62940 3.17229 4.59547 4.01770 3.61491 3.73084 4.54535 3.65161 3.94543 3.88208 3.90115 1.43245 2.58689 3.43142 5.97782 4.79574 585 - -
2.68618 4.42225 2.77519 2.73123 3.46354 2.40513 3.72494 3.29354 2.67741 2.69355 4.24690 2.90347 2.73739 3.18146 2.89801 2.37887 2.77519 2.98518 4.58477 3.61503
0.01769 4.43923 5.16158 0.61958 0.77255 0.82852 0.57393
335 3.76409 5.00828 5.51900 4.94191 3.22802 5.10322 5.37650 2.17476 4.79190 0.63498 2.21542 5.19010 5.15362 4.74419 4.80655 4.48156 3.96830 2.61003 5.44728 4.47739 586 - -
2.68618 4.42225 2.77519 2.73123 3.46354 2.40513 3.72494 3.29354 2.67741 2.69355 4.24690 2.90347 2.73739 3.18146 2.89801 2.37887 2.77519 2.98518 4.58477 3.61503
0.01769 4.43923 5.16158 0.61958 0.77255 0.82852 0.57393
336 2.00274 4.68275 3.23122 2.67185 3.89096 3.55228 3.78748 3.05546 2.13117 2.93374 3.30387 3.15765 3.94679 2.98910 3.07511 2.18258 2.71828 2.84813 5.23773 3.94717 587 - -
2.68618 4.42225 2.77519 2.73123 3.46354 2.40513 3.72494 3.29354 2.67741 2.69355 4.24690 2.90347 2.73739 3.18146 2.89801 2.37887 2.77519 2.98518 4.58477 3.61503
0.01769 4.43923 5.16158 0.61958 0.77255 0.82852 0.57393
337 2.97650 5.46068 2.48454 1.02069 4.73245 3.47665 3.85129 4.23595 2.70305 3.73933 4.54587 2.94182 4.00086 2.59826 3.20978 2.90683 3.23099 3.83083 5.88915 3.92273 588 - -
2.68618 4.42225 2.77519 2.73123 3.46354 2.40513 3.72494 3.29354 2.67741 2.69355 4.24690 2.90347 2.73739 3.18146 2.89801 2.37887 2.77519 2.98518 4.58477 3.61503
0.01769 4.43923 5.16158 0.61958 0.77255 0.82852 0.57393
338 1.01683 4.37556 3.99928 3.68685 4.50701 3.22400 4.62809 3.71319 3.66071 3.55597 4.43836 3.71171 3.99017 3.94334 3.91880 2.68462 1.46832 3.24584 5.90928 4.73019 589 - -
2.68618 4.42225 2.77519 2.73123 3.46354 2.40513 3.72494 3.29354 2.67741 2.69355 4.24690 2.90347 2.73739 3.18146 2.89801 2.37887 2.77519 2.98518 4.58477 3.61503
0.01769 4.43923 5.16158 0.61958 0.77255 0.31899 1.29786
339 1.59079 4.38688 3.92341 3.35933 3.54316 3.78018 4.13997 2.83346 3.28373 2.16393 2.56323 3.67652 4.19163 3.55944 3.57111 2.15379 3.02319 2.48441 5.03611 3.82590 590 - -
2.68618 4.42225 2.77519 2.73123 3.46354 2.40513 3.72494 3.29354 2.67741 2.69355 4.24690 2.90347 2.73739 3.18146 2.89801 2.37887 2.77519 2.98518 4.58477 3.61503
0.01408 4.66559 5.38793 0.61958 0.77255 0.48576 0.95510
340 2.65772 3.51764 2.89211 1.94237 4.53898 3.08927 3.72300 4.01083 1.93183 3.51058 4.26033 2.82771 3.92646 2.82642 2.79842 2.18733 2.98400 3.36329 5.65882 4.26057 591 - -
2.68618 4.42225 2.77519 2.73123 3.46354 2.40513 3.72494 3.29354 2.67741 2.69355 4.24690 2.90347 2.73739 3.18146 2.89801 2.37887 2.77519 2.98518 4.58477 3.61503
0.01408 4.66559 5.38793 0.61958 0.77255 0.48576 0.95510
341 3.77657 5.02720 5.55487 5.02003 0.97943 5.03672 5.02106 2.05289 4.86487 1.41127 3.24305 5.09308 5.18133 4.81375 4.85206 4.40442 3.99026 2.72013 5.15084 3.79738 592 - -
2.68618 4.42225 2.77519 2.73123 3.46354 2.40513 3.72494 3.29354 2.67741 2.69355 4.24690 2.90347 2.73739 3.18146 2.89801 2.37887 2.77519 2.98518 4.58477 3.61503
0.01408 4.66559 5.38793 0.61958 0.77255 0.48576 0.95510
342 1.11548 4.48179 4.10275 3.67156 4.32213 2.35627 4.56744 3.66230 3.63664 3.40945 4.27034 3.78355 4.11830 3.90084 3.92639 2.22364 3.07127 1.87031 5.73689 4.54179 593 - -
2.68618 4.42225 2.77519 2.73123 3.46354 2.40513 3.72494 3.29354 2.67741 2.69355 4.24690 2.90347 2.73739 3.18146 2.89801 2.37887 2.77519 2.98518 4.58477 3.61503
0.01408 4.66559 5.38793 0.61958 0.77255 0.48576 0.95510
343 2.75515 3.26471 2.91384 2.42461 4.35043 3.56065 3.75524 3.40079 1.75446 3.34923 4.13520 2.88207 3.95189 2.88120 2.35491 2.55770 2.98604 2.81315 5.55110 4.18885 594 - -
2.68618 4.42225 2.77519 2.73123 3.46354 2.40513 3.72494 3.29354 2.67741 2.69355 4.24690 2.90347 2.73739 3.18146 2.89801 2.37887 2.77519 2.98518 4.58477 3.61503

0.01408 4.66559 5.38793 0.61958 0.77255 0.48576 0.95510
344 2.64472 4.35604 3.85530 3.28147 2.79862 3.78669 3.47649 2.14326 2.36902 2.19523 3.17577 3.61944 4.16185 3.23265 3.21686 2.93566 2.55316 2.46276 4.95287 3.38484 595 - -
2.68618 4.42225 2.77519 2.73123 3.46354 2.40513 3.72494 3.29354 2.67741 2.69355 4.24690 2.90347 2.73739 3.18146 2.89801 2.37887 2.77519 2.98518 4.58477 3.61503
0.01408 4.66559 5.38793 0.61958 0.77255 0.48576 0.95510
345 3.86200 5.11554 5.13559 4.75430 2.32713 4.74915 3.96952 3.13438 4.56419 2.54634 4.10605 4.51283 5.04643 4.53801 4.57155 4.09351 4.07901 3.43285 0.78080 2.10215 596 - -
2.68618 4.42225 2.77519 2.73123 3.46354 2.40513 3.72494 3.29354 2.67741 2.69355 4.24690 2.90347 2.73739 3.18146 2.89801 2.37887 2.77519 2.98518 4.58477 3.61503
0.01408 4.66559 5.38793 0.61958 0.77255 0.48576 0.95510
346 2.66568 4.65648 5.33180 4.81719 3.96575 4.84076 5.41119 1.64405 4.71892 2.18840 3.77001 4.96292 5.12353 4.94720 4.86625 4.22096 3.64860 0.77934 5.86674 4.66403 597 - -
2.68618 4.42225 2.77519 2.73123 3.46354 2.40513 3.72494 3.29354 2.67741 2.69355 4.24690 2.90347 2.73739 3.18146 2.89801 2.37887 2.77519 2.98518 4.58477 3.61503
0.01408 4.66559 5.38793 0.61958 0.77255 0.48576 0.95510
347 3.23142 5.38819 3.49216 2.99661 4.88306 3.85098 3.99688 4.27387 1.61384 3.75359 4.60377 3.43940 1.13870 3.13850 2.49279 3.23866 3.45749 3.91750 5.83339 4.61088 598 - -
2.68618 4.42225 2.77519 2.73123 3.46354 2.40513 3.72494 3.29354 2.67741 2.69355 4.24690 2.90347 2.73739 3.18146 2.89801 2.37887 2.77519 2.98518 4.58477 3.61503
0.01408 4.66559 5.38793 0.61958 0.77255 0.48576 0.95510
348 3.78108 5.04677 5.28615 4.83971 0.78581 4.83224 4.34252 2.98629 4.66219 1.65567 3.57122 4.72543 5.07428 4.63218 4.66091 4.18532 3.99860 2.87137 4.49684 2.71154 599 - -
2.68618 4.42225 2.77519 2.73123 3.46354 2.40513 3.72494 3.29354 2.67741 2.69355 4.24690 2.90347 2.73739 3.18146 2.89801 2.37887 2.77519 2.98518 4.58477 3.61503
0.24404 4.66559 1.57439 0.61958 0.77255 0.48576 0.95510
349 3.40992 0.27671 4.98841 4.87905 4.80091 3.87936 5.41061 4.09156 4.73311 3.95409 5.10397 4.73322 4.62124 5.05449 4.74292 3.67791 3.92402 3.78379 6.04133 5.07310 600 - -
2.68618 4.42225 2.77519 2.73123 3.46354 2.40513 3.72494 3.29354 2.67741 2.69355 4.24690 2.90347 2.73739 3.18146 2.89801 2.37887 2.77519 2.98518 4.58477 3.61503
0.01769 4.43923 5.16158 0.61958 0.77255 0.82852 0.57393
350 3.45843 5.53934 3.98638 3.17428 5.11100 4.01126 3.83093 4.40225 0.90169 3.75714 4.64067 3.52441 4.34529 2.95730 1.50137 3.43325 3.58068 4.08624 5.72211 4.62501 601 - -
2.68618 4.42225 2.77519 2.73123 3.46354 2.40513 3.72494 3.29354 2.67741 2.69355 4.24690 2.90347 2.73739 3.18146 2.89801 2.37887 2.77519 2.98518 4.58477 3.61503
0.01769 4.43923 5.16158 0.61958 0.77255 0.82852 0.57393
351 3.29109 5.41818 3.38235 2.96243 4.90753 3.80073 3.92955 4.33954 0.71839 3.78750 4.67006 3.04624 4.26287 3.07909 2.48214 3.28059 3.50768 3.98770 5.78110 4.57493 602 - -
2.68618 4.42225 2.77519 2.73123 3.46354 2.40513 3.72494 3.29354 2.67741 2.69355 4.24690 2.90347 2.73739 3.18146 2.89801 2.37887 2.77519 2.98518 4.58477 3.61503
0.01769 4.43923 5.16158 0.61958 0.77255 0.31899 1.29786
352 2.81363 4.68614 3.47251 2.91091 2.31246 3.72295 2.53265 3.23175 2.45294 2.68174 3.78207 2.85387 4.10448 3.19588 3.05225 2.95406 3.04372 2.98355 5.06821 2.23535 603 - -
2.68618 4.42225 2.77519 2.73123 3.46354 2.40513 3.72494 3.29354 2.67741 2.69355 4.24690 2.90347 2.73739 3.18146 2.89801 2.37887 2.77519 2.98518 4.58477 3.61503
0.01408 4.66559 5.38793 0.61958 0.77255 0.48576 0.95510
353 2.75453 4.61935 2.85897 2.41440 4.51778 3.20844 3.72703 3.98496 2.08121 3.49238 4.24688 1.71692 3.93035 2.83289 2.74418 2.43322 2.90551 3.57539 4.69354 4.25347 604 - -
2.68618 4.42225 2.77519 2.73123 3.46354 2.40513 3.72494 3.29354 2.67741 2.69355 4.24690 2.90347 2.73739 3.18146 2.89801 2.37887 2.77519 2.98518 4.58477 3.61503
0.01408 4.66559 5.38793 0.61958 0.77255 0.48576 0.95510
354 2.88840 4.35825 4.31550 3.72751 3.50126 4.00810 4.31552 1.35560 3.30540 2.42197 3.47426 3.96168 4.37011 3.83256 2.71151 3.30286 2.97289 1.69951 5.04431 3.84896 605 - -
2.68618 4.42225 2.77519 2.73123 3.46354 2.40513 3.72494 3.29354 2.67741 2.69355 4.24690 2.90347 2.73739 3.18146 2.89801 2.37887 2.77519 2.98518 4.58477 3.61503
0.01408 4.66559 5.38793 0.61958 0.77255 0.48576 0.95510
355 2.91941 5.36295 3.14319 1.49202 4.73799 3.64691 3.77955 4.18828 1.79033 3.64516 4.41128 3.11790 4.03899 2.52700 2.28854 2.88156 2.85535 3.77301 5.74545 4.39428 606 - -

2.68618 4.42225 2.77519 2.73123 3.46354 2.40513 3.72494 3.29354 2.67741 2.69355 4.24690 2.90347 2.73739 3.18146 2.89801 2.37887 2.77519 2.98518 4.58477 3.61503
0.01408 4.66559 5.38793 0.61958 0.77255 0.48576 0.95510
356 2.89408 4.90422 2.59726 2.92362 4.37722 3.58839 4.20467 3.63153 3.13913 3.40939 4.30535 3.38159 1.10111 3.42359 3.57224 2.99826 3.23077 2.24537 5.74400 4.43441 607 - -
2.68624 4.42231 2.77511 2.73102 3.46360 2.40519 3.72501 3.29360 2.67700 2.69361 4.24696 2.90353 2.73746 3.18139 2.89807 2.37893 2.77526 2.98525 4.58483 3.61509
0.13282 2.12191 5.38793 0.67002 0.71682 0.48576 0.95510
357 2.90381 4.83752 3.54099 2.94419 3.69433 3.75893 3.93382 3.38060 2.49906 3.06237 3.94602 3.38836 4.14704 3.14219 1.21606 3.01918 2.87163 2.40760 5.37037 4.11645 610 - -
2.68618 4.42225 2.77519 2.73123 3.46354 2.40513 3.72494 3.29354 2.67741 2.69355 4.24690 2.90347 2.73739 3.18146 2.89801 2.37887 2.77519 2.98518 4.58477 3.61503
0.01408 4.66559 5.38793 0.61958 0.77255 0.48576 0.95510
358 1.74569 4.67231 3.37623 2.81515 3.43216 3.65885 3.64872 3.08871 2.79212 2.67034 3.76479 2.38803 3.68910 3.12153 3.20794 2.43289 2.98471 2.96697 5.22358 3.16585 611 - -
2.68618 4.42225 2.77519 2.73123 3.46354 2.40513 3.72494 3.29354 2.67741 2.69355 4.24690 2.90347 2.73739 3.18146 2.89801 2.37887 2.77519 2.98518 4.58477 3.61503
0.01408 4.66559 5.38793 0.61958 0.77255 0.48576 0.95510
359 2.88352 5.24321 2.75321 2.56337 4.59272 3.58443 3.84614 4.04139 2.06992 3.56982 4.35682 3.09727 1.41702 2.97262 2.90369 2.71794 2.94539 3.65176 5.73849 4.36857 612 - -
2.68618 4.42225 2.77519 2.73123 3.46354 2.40513 3.72494 3.29354 2.67741 2.69355 4.24690 2.90347 2.73739 3.18146 2.89801 2.37887 2.77519 2.98518 4.58477 3.61503
0.01408 4.66559 5.38793 0.61958 0.77255 0.48576 0.95510
360 2.60306 4.90348 2.67345 1.64704 2.83917 3.59460 3.80238 3.53681 2.61808 3.15545 3.64476 3.13891 3.65762 2.96467 3.07554 2.71359 2.98416 3.22758 3.42440 3.82250 613 - -
2.68618 4.42225 2.77519 2.73123 3.46354 2.40513 3.72494 3.29354 2.67741 2.69355 4.24690 2.90347 2.73739 3.18146 2.89801 2.37887 2.77519 2.98518 4.58477 3.61503
0.01408 4.66559 5.38793 0.61958 0.77255 0.48576 0.95510
361 2.42183 4.81638 3.07477 2.01518 3.09132 3.61591 3.83000 3.41623 2.67298 3.05877 3.47323 3.18668 4.00351 3.01458 2.50244 2.32741 2.98287 2.74420 3.53494 4.04026 614 - -
2.68618 4.42225 2.77519 2.73123 3.46354 2.40513 3.72494 3.29354 2.67741 2.69355 4.24690 2.90347 2.73739 3.18146 2.89801 2.37887 2.77519 2.98518 4.58477 3.61503
0.01408 4.66559 5.38793 0.61958 0.77255 0.48576 0.95510
362 2.51754 4.65971 3.50097 3.02769 3.68749 3.69136 3.67397 3.28217 3.00982 2.96185 3.85053 3.44406 3.11556 3.34006 3.37875 2.55229 3.09258 3.02470 5.14296 1.29983 615 - -
2.68618 4.42225 2.77519 2.73123 3.46354 2.40513 3.72494 3.29354 2.67741 2.69355 4.24690 2.90347 2.73739 3.18146 2.89801 2.37887 2.77519 2.98518 4.58477 3.61503
0.01408 4.66559 5.38793 0.61958 0.77255 0.48576 0.95510
363 3.17979 4.54583 4.92754 4.33930 1.13321 4.36243 4.62883 2.10462 4.17558 1.88276 2.66455 4.44451 3.88282 4.28962 4.24144 3.68039 3.40651 2.55980 5.04580 3.84546 616 - -
2.68618 4.42225 2.77519 2.73123 3.46354 2.40513 3.72494 3.29354 2.67741 2.69355 4.24690 2.90347 2.73739 3.18146 2.89801 2.37887 2.77519 2.98518 4.58477 3.61503
0.01392 4.67731 5.39966 0.61958 0.77255 0.48576 0.95510
364 2.59339 4.23807 2.92395 2.43565 4.42513 2.94464 3.53137 3.87609 2.37367 2.67742 4.18646 2.94014 3.94303 2.64424 2.72249 1.85232 2.76165 3.49430 5.59660 4.21938 617 - -
2.68618 4.42225 2.77519 2.73123 3.46354 2.40513 3.72494 3.29354 2.67741 2.69355 4.24690 2.90347 2.73739 3.18146 2.89801 2.37887 2.77519 2.98518 4.58477 3.61503
0.10679 4.67731 2.38614 0.61958 0.77255 0.48576 0.95510
365 2.64135 5.15611 2.77921 2.35227 4.46815 3.51205 3.39881 3.55082 2.31357 2.52506 4.20587 2.33097 3.90601 2.35491 2.93927 2.26595 2.95917 3.52894 5.60869 4.21859 618 - -
2.68618 4.42225 2.77519 2.73123 3.46354 2.40513 3.72494 3.29354 2.67741 2.69355 4.24690 2.90347 2.73739 3.18146 2.89801 2.37887 2.77519 2.98518 4.58477 3.61503
0.09683 4.58578 2.49994 0.61958 0.77255 0.39521 1.11944
366 2.80411 5.28961 2.44699 2.19266 4.62224 3.52504 3.74693 4.10002 1.46932 3.32533 4.34059 2.99494 2.79827 2.85378 2.97882 2.68190 3.03843 3.67490 5.72527 4.31872 619 - -
2.68618 4.42225 2.77519 2.73123 3.46354 2.40513 3.72494 3.29354 2.67741 2.69355 4.24690 2.90347 2.73739 3.18146 2.89801 2.37887 2.77519 2.98518 4.58477 3.61503
0.01498 4.60432 5.32667 0.61958 0.77255 0.41010 1.08941

367 2.60373 4.77567 2.38315 2.62187 3.72839 3.63250 3.85048 2.48884 2.58000 2.46978 3.23156 3.21880 3.58538 3.04863 2.55425 2.62442 2.98718 2.82571 5.31196 4.01737 620 - -
2.68618 4.42225 2.77519 2.73123 3.46354 2.40513 3.72494 3.29354 2.67741 2.69355 4.24690 2.90347 2.73739 3.18146 2.89801 2.37887 2.77519 2.98518 4.58477 3.61503
0.01392 4.67731 5.39966 0.61958 0.77255 0.48576 0.95510
368 2.61614 5.13475 1.85814 2.31192 4.42894 3.32809 3.28555 3.29800 2.50263 3.17202 3.91515 2.63723 3.94267 2.85990 2.87665 2.66446 2.98655 2.86351 5.59913 4.22107 621 - -
2.68618 4.42225 2.77519 2.73123 3.46354 2.40513 3.72494 3.29354 2.67741 2.69355 4.24690 2.90347 2.73739 3.18146 2.89801 2.37887 2.77519 2.98518 4.58477 3.61503
0.14572 4.67731 2.06914 0.61958 0.77255 0.48576 0.95510
369 2.62597 3.83193 3.39942 2.16957 3.08133 3.63572 3.87307 3.10603 2.80959 2.79480 3.66779 3.29256 2.81876 3.13228 3.21027 2.44024 2.94290 2.75751 5.13271 2.48157 622 - -
2.68618 4.42225 2.77519 2.73123 3.46354 2.40513 3.72494 3.29354 2.67741 2.69355 4.24690 2.90347 2.73739 3.18146 2.89801 2.37887 2.77519 2.98518 4.58477 3.61503
0.01586 4.54746 5.26980 0.61958 0.77255 0.41648 1.07694
370 2.20799 3.99469 3.68622 2.87912 3.57071 3.22755 4.00430 2.92932 3.06379 1.60460 3.54410 3.50637 4.12269 3.36054 3.02444 2.86360 2.65379 2.70900 5.02526 3.11791 623 - -
2.68618 4.42225 2.77519 2.73123 3.46354 2.40513 3.72494 3.29354 2.67741 2.69355 4.24690 2.90347 2.73739 3.18146 2.89801 2.37887 2.77519 2.98518 4.58477 3.61503
0.01420 4.65724 5.37959 0.61958 0.77255 0.52553 0.89463
371 2.52540 5.20595 2.71823 2.46673 4.53158 2.65407 3.72520 4.00144 1.71992 3.50435 4.25646 3.01051 3.92750 2.73880 2.83552 2.46289 2.44447 3.35530 5.65508 4.25900 624 - -
2.68618 4.42225 2.77519 2.73123 3.46354 2.40513 3.72494 3.29354 2.67741 2.69355 4.24690 2.90347 2.73739 3.18146 2.89801 2.37887 2.77519 2.98518 4.58477 3.61503
0.01420 4.65724 5.37959 0.61958 0.77255 0.52553 0.89463
372 2.85989 5.38242 1.80552 2.20190 4.71425 3.53646 3.22170 4.20488 2.57614 3.68220 4.43395 1.97108 3.63509 2.90348 2.91172 2.31594 3.09910 3.76739 5.81661 4.39025 625 - -
2.68618 4.42225 2.77519 2.73123 3.46354 2.40513 3.72494 3.29354 2.67741 2.69355 4.24690 2.90347 2.73739 3.18146 2.89801 2.37887 2.77519 2.98518 4.58477 3.61503
0.12612 4.65724 2.21644 0.61958 0.77255 0.46167 0.99487
373 2.59321 5.23358 2.50231 2.20824 4.56616 2.82032 3.70829 4.04461 1.60534 3.29721 4.28171 2.97265 3.59127 2.81170 2.94819 2.54774 2.98284 3.61834 5.67420 4.26770 626 - -
2.68618 4.42225 2.77519 2.73123 3.46354 2.40513 3.72494 3.29354 2.67741 2.69355 4.24690 2.90347 2.73739 3.18146 2.89801 2.37887 2.77519 2.98518 4.58477 3.61503
0.01552 4.56938 5.29172 0.61958 0.77255 0.38313 1.14484
374 2.21803 4.67503 3.38293 2.62349 3.84758 3.66456 3.89391 2.96507 2.65307 2.90348 3.76782 3.29296 4.05008 2.93253 3.21195 1.96921 2.88612 2.00701 5.22930 3.69271 627 - -
2.68618 4.42225 2.77519 2.73123 3.46354 2.40513 3.72494 3.29354 2.67741 2.69355 4.24690 2.90347 2.73739 3.18146 2.89801 2.37887 2.77519 2.98518 4.58477 3.61503
0.01392 4.67731 5.39966 0.61958 0.77255 0.48576 0.95510
375 2.68139 5.24956 2.79732 1.93334 4.07428 3.53069 3.15872 4.06916 2.35590 3.55190 4.29205 2.58629 3.92424 2.39400 2.37263 2.54677 2.89247 3.05640 5.68613 4.27935 628 - -
2.68618 4.42225 2.77519 2.73123 3.46354 2.40513 3.72494 3.29354 2.67741 2.69355 4.24690 2.90347 2.73739 3.18146 2.89801 2.37887 2.77519 2.98518 4.58477 3.61503
0.01392 4.67731 5.39966 0.61958 0.77255 0.48576 0.95510
376 2.61121 4.58306 3.48539 2.73853 2.45341 3.69526 3.28325 2.91798 2.88968 2.48532 3.68030 2.88583 2.79304 2.99504 3.28094 2.92748 2.71702 2.86674 5.14966 2.69462 629 - -
2.68618 4.42225 2.77519 2.73123 3.46354 2.40513 3.72494 3.29354 2.67741 2.69355 4.24690 2.90347 2.73739 3.18146 2.89801 2.37887 2.77519 2.98518 4.58477 3.61503
0.01392 4.67731 5.39966 0.61958 0.77255 0.48576 0.95510
377 2.50602 5.04210 2.33683 2.55381 4.31898 3.56449 3.77429 3.52522 2.55346 2.99332 4.11766 2.96835 2.36268 2.90639 3.02723 2.09676 2.73442 3.13179 5.53875 4.18273 630 - -
2.68648 4.42255 2.77493 2.73127 3.46339 2.40543 3.72524 3.29335 2.67649 2.69385 4.24437 2.90377 2.73769 3.18176 2.89814 2.37917 2.77539 2.98521 4.58163 3.61429
0.24427 1.90412 2.69165 1.74980 0.19093 0.48576 0.95510
378 2.77500 4.22259 4.33271 3.74652 1.74517 3.88865 4.16509 2.64905 3.60640 2.38000 2.05048 3.50851 4.25573 3.80724 3.75061 3.18290 2.59808 2.38365 4.76727 2.99341 644 - -
2.68618 4.42225 2.77519 2.73123 3.46354 2.40513 3.72494 3.29354 2.67741 2.69355 4.24690 2.90347 2.73739 3.18146 2.89801 2.37887 2.77519 2.98518 4.58477 3.61503

0.09459 4.61197 2.52170 0.61958 0.77255 0.60724 0.78713

379 3.40790 4.70226 5.28323 4.85511 3.98737 4.77656 5.58375 1.82788 4.73346 2.46853 3.78373 4.99791 5.13953 5.02836 4.91132 4.22415 3.70213 0.57879 6.02838 4.77960 645 - -

2.68618 4.42225 2.77519 2.73123 3.46354 2.40513 3.72494 3.29354 2.67741 2.69355 4.24690 2.90347 2.73739 3.18146 2.89801 2.37887 2.77519 2.98518 4.58477 3.61503

0.01609 4.53347 5.25582 0.61958 0.77255 0.72760 0.65984

380 2.77156 4.65793 3.43742 2.85411 3.81885 3.67232 3.86393 2.99920 1.91272 1.90864 3.52425 3.30610 4.04993 3.10121 2.36862 2.90988 2.99663 2.94834 5.19124 3.23241 646 - -

2.68618 4.42225 2.77519 2.73123 3.46354 2.40513 3.72494 3.29354 2.67741 2.69355 4.24690 2.90347 2.73739 3.18146 2.89801 2.37887 2.77519 2.98518 4.58477 3.61503

0.01609 4.53347 5.25582 0.61958 0.77255 0.72760 0.65984

381 2.71671 4.82751 2.43033 1.92976 3.33982 3.56356 3.77868 2.83857 2.49836 3.07077 3.90583 3.11841 3.95505 2.95051 3.05983 2.77751 2.94866 2.31055 5.35090 4.03472 647 - -

2.68618 4.42225 2.77519 2.73123 3.46354 2.40513 3.72494 3.29354 2.67741 2.69355 4.24690 2.90347 2.73739 3.18146 2.89801 2.37887 2.77519 2.98518 4.58477 3.61503

0.01609 4.53347 5.25582 0.61958 0.77255 0.72760 0.65984

382 2.98854 3.13342 3.48982 2.86326 4.46539 3.73585 3.84084 3.85596 2.32053 3.40113 4.24681 3.31145 4.12908 1.79754 1.42366 3.02396 3.20607 3.53539 5.56108 4.31108 648 - -

2.68618 4.42225 2.77519 2.73123 3.46354 2.40513 3.72494 3.29354 2.67741 2.69355 4.24690 2.90347 2.73739 3.18146 2.89801 2.37887 2.77519 2.98518 4.58477 3.61503

0.01609 4.53347 5.25582 0.61958 0.77255 0.72760 0.65984

383 3.00608 4.82830 3.69756 2.85818 4.02758 3.84629 3.97082 1.69865 2.53844 2.97011 3.91796 3.49935 4.22655 3.19640 1.46798 3.15888 3.22042 2.99665 5.35377 4.13461 649 - -

2.68618 4.42225 2.77519 2.73123 3.46354 2.40513 3.72494 3.29354 2.67741 2.69355 4.24690 2.90347 2.73739 3.18146 2.89801 2.37887 2.77519 2.98518 4.58477 3.61503

0.01609 4.53347 5.25582 0.61958 0.77255 0.72760 0.65984

384 2.06390 4.82033 3.17428 2.61471 4.03554 3.56346 3.77189 3.43858 2.47284 3.06825 3.89943 2.78265 2.30597 2.94414 2.61357 2.77365 2.94331 2.96215 4.23376 3.54239 650 - -

2.68618 4.42225 2.77519 2.73123 3.46354 2.40513 3.72494 3.29354 2.67741 2.69355 4.24690 2.90347 2.73739 3.18146 2.89801 2.37887 2.77519 2.98518 4.58477 3.61503

0.01609 4.53347 5.25582 0.61958 0.77255 0.72760 0.65984

385 3.01661 4.42522 4.57055 3.48960 3.44770 4.19840 4.54370 1.99345 3.86182 2.19541 1.36708 4.20602 4.53038 4.08297 4.02785 3.50980 3.25276 1.80923 5.14443 3.97539 651 - -

2.68618 4.42225 2.77519 2.73123 3.46354 2.40513 3.72494 3.29354 2.67741 2.69355 4.24690 2.90347 2.73739 3.18146 2.89801 2.37887 2.77519 2.98518 4.58477 3.61503

0.01609 4.53347 5.25582 0.61958 0.77255 0.35970 1.19694

386 2.61527 3.11225 3.44594 2.88022 3.19058 3.69217 3.91340 3.20086 1.44386 2.88184 3.75457 3.33715 4.07632 2.98111 3.20256 2.92377 3.01010 2.95088 5.19735 3.04942 652 - -

2.68618 4.42225 2.77519 2.73123 3.46354 2.40513 3.72494 3.29354 2.67741 2.69355 4.24690 2.90347 2.73739 3.18146 2.89801 2.37887 2.77519 2.98518 4.58477 3.61503

0.01392 4.67731 5.39966 0.61958 0.77255 0.48576 0.95510

387 3.05509 5.29863 3.42248 2.81470 4.65215 3.78595 3.85252 4.04962 2.20335 3.55402 3.22403 1.97510 4.16246 2.98066 1.29240 3.05271 3.25768 3.70424 5.67975 4.40789 653 - -

2.68618 4.42225 2.77519 2.73123 3.46354 2.40513 3.72494 3.29354 2.67741 2.69355 4.24690 2.90347 2.73739 3.18146 2.89801 2.37887 2.77519 2.98518 4.58477 3.61503

0.01392 4.67731 5.39966 0.61958 0.77255 0.48576 0.95510

388 2.65032 5.20641 2.54035 1.23131 4.49588 3.56474 3.80331 3.92903 2.58559 3.16847 3.31667 3.05477 3.98773 2.92661 3.07296 2.81108 3.06471 3.55440 5.67754 4.29297 654 - -

2.68618 4.42225 2.77519 2.73123 3.46354 2.40513 3.72494 3.29354 2.67741 2.69355 4.24690 2.90347 2.73739 3.18146 2.89801 2.37887 2.77519 2.98518 4.58477 3.61503

0.01392 4.67731 5.39966 0.61958 0.77255 0.48576 0.95510

389 3.29910 5.28463 3.82788 3.16318 4.17271 4.00527 3.93991 3.96419 1.86538 3.48633 4.38133 3.55789 4.36393 3.15177 1.98970 3.34234 3.47760 3.69021 5.40940 1.17918 655 - -

2.68618 4.42225 2.77519 2.73123 3.46354 2.40513 3.72494 3.29354 2.67741 2.69355 4.24690 2.90347 2.73739 3.18146 2.89801 2.37887 2.77519 2.98518 4.58477 3.61503

0.01392 4.67731 5.39966 0.61958 0.77255 0.48576 0.95510

390 2.94771 5.43650 2.05154 1.17828 4.75164 3.56452 3.87122 4.22790 2.68337 3.73059 4.50607 3.03332 4.03835 2.99314 2.91290 2.89473 3.19338 2.99180 5.88447 4.45943 656 - -

2.68618 4.42225 2.77519 2.73123 3.46354 2.40513 3.72494 3.29354 2.67741 2.69355 4.24690 2.90347 2.73739 3.18146 2.89801 2.37887 2.77519 2.98518 4.58477 3.61503

0.01392 4.67731 5.39966 0.61958 0.77255 0.48576 0.95510

391 2.87235 5.30147 2.54721 1.50562 4.60733 3.56660 3.82068 4.05872 2.09332 3.58742 4.36399 3.04492 4.00490 2.94064 3.08747 2.83973 3.11041 2.17228 5.75635 4.35814 657 - -

2.68618 4.42225 2.77519 2.73123 3.46354 2.40513 3.72494 3.29354 2.67741 2.69355 4.24690 2.90347 2.73739 3.18146 2.89801 2.37887 2.77519 2.98518 4.58477 3.61503

0.01392 4.67731 5.39966 0.61958 0.77255 0.48576 0.95510

392 2.93235 3.28141 4.77772 4.18500 1.59387 4.08670 4.30380 2.64888 3.99189 1.78372 2.55555 4.19661 4.43294 4.11296 4.02353 3.39904 3.16244 2.51598 4.73482 2.39975 658 - -

2.68618 4.42225 2.77519 2.73123 3.46354 2.40513 3.72494 3.29354 2.67741 2.69355 4.24690 2.90347 2.73739 3.18146 2.89801 2.37887 2.77519 2.98518 4.58477 3.61503

0.01392 4.67731 5.39966 0.61958 0.77255 0.48576 0.95510

393 2.64329 3.41012 3.08509 2.20975 4.54316 3.57878 3.75201 3.99835 1.47034 3.50595 4.27093 3.06156 3.97056 2.35307 2.80023 2.63432 3.03184 3.59937 5.65664 4.28292 659 - -

2.68618 4.42225 2.77519 2.73123 3.46354 2.40513 3.72494 3.29354 2.67741 2.69355 4.24690 2.90347 2.73739 3.18146 2.89801 2.37887 2.77519 2.98518 4.58477 3.61503

0.01392 4.67731 5.39966 0.61958 0.77255 0.48576 0.95510

394 2.60423 4.51284 3.60818 3.03968 3.65613 3.73472 3.98200 2.76084 2.97667 2.24475 3.61497 3.45470 4.11501 2.47651 2.57761 2.61934 2.99919 1.79109 5.09280 3.85483 660 - -

2.68618 4.42225 2.77519 2.73123 3.46354 2.40513 3.72494 3.29354 2.67741 2.69355 4.24690 2.90347 2.73739 3.18146 2.89801 2.37887 2.77519 2.98518 4.58477 3.61503

0.01392 4.67731 5.39966 0.61958 0.77255 0.48576 0.95510

395 3.21907 5.45881 3.60994 2.96102 4.93942 3.88858 3.89630 4.31072 1.66385 3.74276 4.56910 3.40503 4.26451 3.01699 1.29338 1.85941 3.40869 3.94650 5.79332 4.57402 661 - -

2.68618 4.42225 2.77519 2.73123 3.46354 2.40513 3.72494 3.29354 2.67741 2.69355 4.24690 2.90347 2.73739 3.18146 2.89801 2.37887 2.77519 2.98518 4.58477 3.61503

0.01392 4.67731 5.39966 0.61958 0.77255 0.48576 0.95510

396 3.58313 4.78760 5.63608 5.15659 4.05133 5.17236 5.85445 0.79604 5.08569 2.46503 3.37421 5.32008 5.38367 5.30707 5.23460 4.59682 3.83945 1.23220 6.15781 4.97003 662 - -

2.68618 4.42225 2.77519 2.73123 3.46354 2.40513 3.72494 3.29354 2.67741 2.69355 4.24690 2.90347 2.73739 3.18146 2.89801 2.37887 2.77519 2.98518 4.58477 3.61503

0.01392 4.67731 5.39966 0.61958 0.77255 0.48576 0.95510

397 2.66732 5.44143 2.76556 1.69066 4.83243 2.28884 3.93765 4.32079 2.78295 3.81966 4.59945 1.60457 4.06599 3.06693 3.30304 2.67746 3.24573 3.88393 5.97303 4.54178 663 - -

2.68618 4.42225 2.77519 2.73123 3.46354 2.40513 3.72494 3.29354 2.67741 2.69355 4.24690 2.90347 2.73739 3.18146 2.89801 2.37887 2.77519 2.98518 4.58477 3.61503

0.01392 4.67731 5.39966 0.61958 0.77255 0.48576 0.95510

398 1.58179 5.09533 2.40428 2.54151 4.40900 3.04959 3.13000 3.60493 2.56379 3.40849 4.19152 3.07289 3.96472 2.91320 3.04330 2.43792 2.81420 3.47878 5.60507 4.23699 664 - -

2.68618 4.42225 2.77519 2.73123 3.46354 2.40513 3.72494 3.29354 2.67741 2.69355 4.24690 2.90347 2.73739 3.18146 2.89801 2.37887 2.77519 2.98518 4.58477 3.61503

0.01392 4.67731 5.39966 0.61958 0.77255 0.48576 0.95510

399 1.64422 4.41319 4.29474 3.73849 3.50316 3.97725 4.36771 2.62198 3.61511 1.46873 3.45738 3.97393 4.37428 3.55154 3.82520 3.28759 3.15806 1.92100 5.09279 3.90582 665 - -

2.68618 4.42225 2.77519 2.73123 3.46354 2.40513 3.72494 3.29354 2.67741 2.69355 4.24690 2.90347 2.73739 3.18146 2.89801 2.37887 2.77519 2.98518 4.58477 3.61503

0.01392 4.67731 5.39966 0.61958 0.77255 0.48576 0.95510

400 2.49669 2.16244 4.22137 3.69101 3.68224 3.64884 4.34994 2.98297 3.59387 2.76506 3.67542 3.84284 2.37710 3.83292 3.81470 2.81296 2.20296 1.71684 5.17191 3.98020 666 - -

2.68618 4.42225 2.77519 2.73123 3.46354 2.40513 3.72494 3.29354 2.67741 2.69355 4.24690 2.90347 2.73739 3.18146 2.89801 2.37887 2.77519 2.98518 4.58477 3.61503

0.25577 4.67731 1.53074 0.61958 0.77255 0.48576 0.95510

401 2.13251 5.18543 2.55679 1.95345 4.50834 2.53478 3.29481 3.98188 2.44385 3.48286 4.23897 2.93570 3.87578 2.79089 2.73817 2.52273 2.95654 3.56556 5.63374 4.22983 667 - -

2.68618 4.42225 2.77519 2.73123 3.46354 2.40513 3.72494 3.29354 2.67741 2.69355 4.24690 2.90347 2.73739 3.18146 2.89801 2.37887 2.77519 2.98518 4.58477 3.61503

0.01769 4.43923 5.16158 0.61958 0.77255 0.84528 0.56113

402 2.48567 5.09787 2.39370 2.41477 4.40710 3.46957 3.66747 3.86597 2.03911 3.10925 4.15316 2.95559 3.86647 2.63415 2.90341 2.06806 2.68650 3.31850 5.55726 4.17136 668 - -

2.68618 4.42225 2.77519 2.73123 3.46354 2.40513 3.72494 3.29354 2.67741 2.69355 4.24690 2.90347 2.73739 3.18146 2.89801 2.37887 2.77519 2.98518 4.58477 3.61503

0.01769 4.43923 5.16158 0.61958 0.77255 0.84528 0.56113

403 1.79473 4.39695 3.68243 3.18669 3.86899 2.46543 4.13788 2.03031 3.15532 2.93893 3.82057 3.48681 4.00102 3.45159 3.49539 1.94781 2.92818 2.89376 5.29588 4.06928 669 - -

2.68613 4.42384 2.77570 2.72952 3.46437 2.40617 3.72535 3.29370 2.67543 2.69418 4.24302 2.90368 2.73794 3.18061 2.89585 2.37984 2.77647 2.98502 4.58465 3.61588

1.08731 0.67708 1.86576 2.50512 0.08519 0.84528 0.56113

404 2.51716 3.72364 4.21350 3.64234 3.38526 3.87432 4.24080 1.87934 3.13802 2.36529 3.35034 3.86539 4.25027 3.77081 3.72902 3.17665 2.79709 1.40877 4.94674 3.74799 709 - -

2.68618 4.42225 2.77519 2.73123 3.46354 2.40513 3.72494 3.29354 2.67741 2.69355 4.24690 2.90347 2.73739 3.18146 2.89801 2.37887 2.77519 2.98518 4.58477 3.61503

0.02082 4.27784 5.00018 0.61958 0.77255 1.00009 0.45862

405 2.74127 4.82975 2.93147 2.19636 4.23346 3.42011 3.89311 3.57224 2.71860 3.24596 4.12619 3.10687 1.47399 3.08215 3.12942 2.80590 3.04248 2.88078 5.53765 4.21860 710 - -

2.68618 4.42225 2.77519 2.73123 3.46354 2.40513 3.72494 3.29354 2.67741 2.69355 4.24690 2.90347 2.73739 3.18146 2.89801 2.37887 2.77519 2.98518 4.58477 3.61503

0.02082 4.27784 5.00018 0.61958 0.77255 1.00009 0.45862

406 2.75606 5.11204 2.92289 1.75499 4.43515 3.47760 3.67282 3.86118 1.78023 3.17658 4.18425 2.97117 3.89353 2.79582 2.75973 2.73824 2.53981 3.48653 5.55454 4.20161 711 - -

2.68618 4.42225 2.77519 2.73123 3.46354 2.40513 3.72494 3.29354 2.67741 2.69355 4.24690 2.90347 2.73739 3.18146 2.89801 2.37887 2.77519 2.98518 4.58477 3.61503

0.02082 4.27784 5.00018 0.61958 0.77255 1.00009 0.45862

407 1.65841 5.27675 2.22739 1.63850 4.61772 3.38126 3.80309 4.07362 2.69405 3.62000 4.43112 2.88197 3.91817 2.94973 3.22115 2.81134 3.13040 3.67457 5.80663 4.38017 712 - -

2.68618 4.42225 2.77519 2.73123 3.46354 2.40513 3.72494 3.29354 2.67741 2.69355 4.24690 2.90347 2.73739 3.18146 2.89801 2.37887 2.77519 2.98518 4.58477 3.61503

0.06559 4.27784 3.00360 0.61958 0.77255 1.00009 0.45862

408 2.52440 4.35267 3.53029 3.06772 2.75451 1.92428 4.03249 3.12266 3.04964 2.84962 3.75413 3.38567 3.92968 3.35402 3.39227 2.72252 1.84069 2.83393 5.17575 3.90150 713 - -

2.68618 4.42225 2.77519 2.73123 3.46354 2.40513 3.72494 3.29354 2.67741 2.69355 4.24690 2.90347 2.73739 3.18146 2.89801 2.37887 2.77519 2.98518 4.58477 3.61503

0.02177 4.23402 4.95637 0.61958 0.77255 1.03460 0.43908

409 3.18736 4.62529 4.44579 3.96038 2.73927 4.14031 4.06600 2.15755 3.68534 2.23417 3.48143 4.08242 4.51386 3.97312 3.83762 3.52365 3.43875 2.67667 1.25043 2.86538 714 - -

2.68607 4.42248 2.77542 2.73139 3.46377 2.40536 3.72517 3.29364 2.67764 2.69378 4.24076 2.90370 2.73762 3.18066 2.89824 2.37887 2.77494 2.98517 4.58500 3.61526

0.68874 0.71182 4.95637 0.88600 0.53154 0.94344 0.49313

410 2.34831 5.08625 1.17170 2.42271 4.65079 3.32549 3.93063 4.03425 2.87695 3.66731 4.50669 2.95605 3.93248 3.09906 3.39907 2.81033 2.61971 3.61821 5.88941 4.48132 718 - -

2.68618 4.42225 2.77519 2.73123 3.46354 2.40513 3.72494 3.29354 2.67741 2.69355 4.24690 2.90347 2.73739 3.18146 2.89801 2.37887 2.77519 2.98518 4.58477 3.61503

0.02082 4.27784 5.00018 0.61958 0.77255 0.46758 0.98488

411 2.74724 3.53336 3.14782 2.58502 4.20658 1.83541 3.75786 3.62123 2.35282 3.21481 3.65357 2.93847 3.95687 2.59851 2.45965 2.77839 2.97700 3.29481 5.44586 4.11862 719 - -

2.68618 4.42225 2.77519 2.73123 3.46354 2.40513 3.72494 3.29354 2.67741 2.69355 4.24690 2.90347 2.73739 3.18146 2.89801 2.37887 2.77519 2.98518 4.58477 3.61503

0.01579 4.55235 5.27470 0.61958 0.77255 0.70078 0.68557

412 2.72384 4.51690 2.92287 2.94496 3.68348 3.66729 3.93799 2.86502 2.91272 1.92493 3.62970 3.37568 4.06103 3.22748 3.29243 2.64579 1.82027 2.58974 5.11386 3.86548 720 - -

2.68618 4.42225 2.77519 2.73123 3.46354 2.40513 3.72494 3.29354 2.67741 2.69355 4.24690 2.90347 2.73739 3.18146 2.89801 2.37887 2.77519 2.98518 4.58477 3.61503

0.01579 4.55235 5.27470 0.61958 0.77255 0.37154 1.17012

413 2.76660 5.21852 2.51365 2.47292 4.53962 3.54043 2.15398 3.60016 2.36892 3.51343 4.26704 3.01754 2.76464 2.54936 2.97030 2.25326 2.90569 3.59656 5.66569 4.26917 721 - -

2.68618 4.42225 2.77519 2.73123 3.46354 2.40513 3.72494 3.29354 2.67741 2.69355 4.24690 2.90347 2.73739 3.18146 2.89801 2.37887 2.77519 2.98518 4.58477 3.61503

0.01392 4.67731 5.39966 0.61958 0.77255 0.48576 0.95510
414 3.96925 5.18788 5.22717 4.88053 2.02925 4.82471 3.96064 3.60468 4.68658 2.76155 3.61658 4.56684 5.11468 4.61261 4.66122 4.17632 4.18377 3.53743 0.75820 2.17509 722 - -
2.68618 4.42225 2.77519 2.73123 3.46354 2.40513 3.72494 3.29354 2.67741 2.69355 4.24690 2.90347 2.73739 3.18146 2.89801 2.37887 2.77519 2.98518 4.58477 3.61503
0.01392 4.67731 5.39966 0.61958 0.77255 0.48576 0.95510
415 2.80784 4.73994 3.48400 2.98520 4.08595 3.20661 4.04191 3.47164 2.85715 2.29552 4.00349 3.40701 1.31351 3.27193 2.82565 2.78941 3.09558 3.17734 5.43510 4.17282 723 - -
2.68618 4.42225 2.77519 2.73123 3.46354 2.40513 3.72494 3.29354 2.67741 2.69355 4.24690 2.90347 2.73739 3.18146 2.89801 2.37887 2.77519 2.98518 4.58477 3.61503
0.01392 4.67731 5.39966 0.61958 0.77255 0.48576 0.95510
416 2.76307 4.39580 2.39065 2.53564 4.37107 1.87515 3.76464 3.81137 2.26318 2.89761 3.88234 3.06856 3.95906 2.88942 2.87180 2.47280 2.99514 3.44679 5.56897 4.20393 724 - -
2.68618 4.42225 2.77519 2.73123 3.46354 2.40513 3.72494 3.29354 2.67741 2.69355 4.24690 2.90347 2.73739 3.18146 2.89801 2.37887 2.77519 2.98518 4.58477 3.61503
0.01372 4.69125 5.41360 0.61958 0.77255 0.48576 0.95510
417 2.36817 5.18820 2.24941 2.41395 4.50117 3.06854 3.77183 3.95923 2.53802 3.48741 4.25563 2.18919 3.96133 2.88702 3.02610 2.76936 2.23324 2.81687 5.66035 4.27334 725 - -
2.68603 4.42315 2.77343 2.73092 3.46074 2.40571 3.72417 3.29423 2.67795 2.69408 4.24780 2.90329 2.73781 3.18216 2.89878 2.37882 2.77495 2.98591 4.57891 3.61593
0.86912 1.03999 1.48182 2.05295 0.13737 0.48576 0.95510
418 2.74060 5.07855 2.96661 2.49366 4.47294 3.47365 3.74991 3.92246 1.97604 3.45471 4.23554 2.13655 3.91694 2.87418 2.93264 1.89751 2.52778 3.52288 5.62343 4.25779 736 - -
2.68618 4.42225 2.77519 2.73123 3.46354 2.40513 3.72494 3.29354 2.67741 2.69355 4.24690 2.90347 2.73739 3.18146 2.89801 2.37887 2.77519 2.98518 4.58477 3.61503
0.01769 4.43923 5.16158 0.61958 0.77255 0.86509 0.54648
419 2.72243 4.94804 3.11652 2.55264 4.23264 3.52883 3.72317 3.64863 2.22837 3.23029 4.03830 3.06439 2.65656 2.86437 2.01762 2.66231 2.39659 2.85031 5.44651 4.11830 737 - -
2.68618 4.42225 2.77519 2.73123 3.46354 2.40513 3.72494 3.29354 2.67741 2.69355 4.24690 2.90347 2.73739 3.18146 2.89801 2.37887 2.77519 2.98518 4.58477 3.61503
0.01769 4.43923 5.16158 0.61958 0.77255 0.86509 0.54648
420 2.76152 5.16466 2.98942 2.46446 4.49770 2.50664 3.00887 3.95036 2.30626 3.45558 4.22723 2.29631 3.91347 2.80633 2.00666 2.57576 2.98983 3.55360 5.60220 4.23461 738 - -
2.68618 4.42225 2.77519 2.73123 3.46354 2.40513 3.72494 3.29354 2.67741 2.69355 4.24690 2.90347 2.73739 3.18146 2.89801 2.37887 2.77519 2.98518 4.58477 3.61503
0.01769 4.43923 5.16158 0.61958 0.77255 0.86509 0.54648
421 3.32652 5.87084 0.65840 2.37694 5.20437 3.46524 4.14962 4.84691 3.30074 4.33945 5.25066 2.56746 4.15577 3.34664 3.95732 3.19417 3.66304 4.37940 6.41730 4.86859 739 - -
2.68618 4.42225 2.77519 2.73123 3.46354 2.40513 3.72494 3.29354 2.67741 2.69355 4.24690 2.90347 2.73739 3.18146 2.89801 2.37887 2.77519 2.98518 4.58477 3.61503
0.01769 4.43923 5.16158 0.61958 0.77255 0.86509 0.54648
422 3.83593 5.48018 3.89393 3.74324 3.86969 4.07803 0.36806 4.63815 3.58184 4.03830 5.15471 4.11526 4.67538 4.12757 3.79419 3.91337 4.17371 4.36164 5.31206 3.81191 740 - -
2.68618 4.42225 2.77519 2.73123 3.46354 2.40513 3.72494 3.29354 2.67741 2.69355 4.24690 2.90347 2.73739 3.18146 2.89801 2.37887 2.77519 2.98518 4.58477 3.61503
0.01769 4.43923 5.16158 0.61958 0.77255 0.30559 1.33442
423 2.15616 5.15775 2.95992 2.52601 4.49597 3.24037 3.77332 3.95202 2.43575 3.47989 4.24767 3.06067 2.32270 2.88809 2.12126 2.24465 3.01421 3.55486 5.65050 4.27140 741 - -
2.68618 4.42225 2.77519 2.73123 3.46354 2.40513 3.72494 3.29354 2.67741 2.69355 4.24690 2.90347 2.73739 3.18146 2.89801 2.37887 2.77519 2.98518 4.58477 3.61503
0.01372 4.69125 5.41360 0.61958 0.77255 0.48576 0.95510
424 2.77264 4.35399 3.94993 3.37798 3.48366 2.00269 4.11848 2.34521 3.29718 2.18329 2.93680 3.68984 4.19784 3.56503 3.57007 2.85372 2.02456 2.59593 4.97243 3.76438 742 - -
2.68618 4.42225 2.77519 2.73123 3.46354 2.40513 3.72494 3.29354 2.67741 2.69355 4.24690 2.90347 2.73739 3.18146 2.89801 2.37887 2.77519 2.98518 4.58477 3.61503
0.01372 4.69125 5.41360 0.61958 0.77255 0.48576 0.95510
425 3.58815 4.83406 5.56012 5.00742 3.60709 4.99924 5.42424 1.05999 4.88054 1.60833 2.03668 5.14208 5.18294 4.93740 4.91931 4.37306 3.81947 1.85345 5.66100 4.58732 743 - -

2.68618 4.42225 2.77519 2.73123 3.46354 2.40513 3.72494 3.29354 2.67741 2.69355 4.24690 2.90347 2.73739 3.18146 2.89801 2.37887 2.77519 2.98518 4.58477 3.61503
0.01372 4.69125 5.41360 0.61958 0.77255 0.48576 0.95510
426 3.61301 4.81038 5.68215 5.20135 4.02882 5.21498 5.88326 0.90088 5.13376 1.98897 3.76707 5.36401 5.40931 5.33456 5.27066 4.64093 3.86654 1.20673 6.15242 4.97768 744 - -
2.68618 4.42225 2.77519 2.73123 3.46354 2.40513 3.72494 3.29354 2.67741 2.69355 4.24690 2.90347 2.73739 3.18146 2.89801 2.37887 2.77519 2.98518 4.58477 3.61503
0.01372 4.69125 5.41360 0.61958 0.77255 0.48576 0.95510
427 3.19614 5.42774 3.48104 2.90931 4.79552 3.86493 3.91006 4.19233 1.83293 3.65689 3.89169 3.37292 4.25219 1.00791 2.59096 3.18335 3.39116 3.85535 5.76381 4.51212 745 - -
2.68618 4.42225 2.77519 2.73123 3.46354 2.40513 3.72494 3.29354 2.67741 2.69355 4.24690 2.90347 2.73739 3.18146 2.89801 2.37887 2.77519 2.98518 4.58477 3.61503
0.08706 4.69125 2.60097 0.61958 0.77255 0.48576 0.95510
428 2.81450 4.66783 5.26856 4.80195 4.06799 4.73748 5.50908 1.76675 4.70852 2.60598 3.85048 4.93859 5.10233 4.97897 4.88900 4.15209 3.65132 0.63044 6.01957 4.79818 746 - -
2.68618 4.42225 2.77519 2.73123 3.46354 2.40513 3.72494 3.29354 2.67741 2.69355 4.24690 2.90347 2.73739 3.18146 2.89801 2.37887 2.77519 2.98518 4.58477 3.61503
0.01476 4.61895 5.34130 0.61958 0.77255 0.62067 0.77129
429 3.39502 4.71752 5.21117 4.61942 2.10203 4.60753 4.82677 2.59938 4.45152 0.91818 2.16195 4.71222 4.83702 4.47835 4.46375 3.93843 3.61187 2.59549 5.11197 3.35856 747 - -
2.68618 4.42225 2.77519 2.73123 3.46354 2.40513 3.72494 3.29354 2.67741 2.69355 4.24690 2.90347 2.73739 3.18146 2.89801 2.37887 2.77519 2.98518 4.58477 3.61503
0.01476 4.61895 5.34130 0.61958 0.77255 0.62067 0.77129
430 2.80008 4.89734 1.98072 2.65399 4.10360 3.60229 3.85843 3.49355 2.70533 1.71808 3.98226 3.16476 4.02071 2.64014 3.15473 2.69049 3.03851 2.94721 5.42695 4.10949 748 - -
2.68618 4.42225 2.77519 2.73123 3.46354 2.40513 3.72494 3.29354 2.67741 2.69355 4.24690 2.90347 2.73739 3.18146 2.89801 2.37887 2.77519 2.98518 4.58477 3.61503
0.01476 4.61895 5.34130 0.61958 0.77255 0.62067 0.77129
431 2.60431 5.39459 1.66708 2.43458 4.72297 2.29184 3.50500 4.21106 2.04650 3.69544 4.45708 2.69446 3.98778 2.92761 3.11792 2.83091 3.12700 3.77988 5.83648 4.40954 749 - -
2.68618 4.42225 2.77519 2.73123 3.46354 2.40513 3.72494 3.29354 2.67741 2.69355 4.24690 2.90347 2.73739 3.18146 2.89801 2.37887 2.77519 2.98518 4.58477 3.61503
0.01476 4.61895 5.34130 0.61958 0.77255 0.62067 0.77129
432 2.73575 4.97099 3.11476 2.55770 3.92460 3.24989 2.99916 3.64186 2.44189 2.87926 4.03896 2.87129 2.77163 2.80036 3.01885 1.84672 2.96707 3.08490 5.46650 3.77238 750 - -
2.68618 4.42225 2.77519 2.73123 3.46354 2.40513 3.72494 3.29354 2.67741 2.69355 4.24690 2.90347 2.73739 3.18146 2.89801 2.37887 2.77519 2.98518 4.58477 3.61503
0.01476 4.61895 5.34130 0.61958 0.77255 0.62067 0.77129
433 2.74116 5.18235 2.60669 2.16516 3.58828 3.52024 3.71525 3.96620 2.46445 3.47605 4.23188 2.72938 2.63637 2.82215 2.77477 2.24144 2.31447 3.55813 5.63307 4.23904 751 - -
2.68618 4.42225 2.77519 2.73123 3.46354 2.40513 3.72494 3.29354 2.67741 2.69355 4.24690 2.90347 2.73739 3.18146 2.89801 2.37887 2.77519 2.98518 4.58477 3.61503
0.01476 4.61895 5.34130 0.61958 0.77255 0.62067 0.77129
434 2.81369 5.28940 1.95131 2.16764 4.61327 2.24405 3.76742 4.09079 2.54384 3.06668 4.34718 2.61622 3.95296 2.87831 3.04506 2.48706 2.75762 3.67090 5.73877 4.32748 752 - -
2.68618 4.42225 2.77519 2.73123 3.46354 2.40513 3.72494 3.29354 2.67741 2.69355 4.24690 2.90347 2.73739 3.18146 2.89801 2.37887 2.77519 2.98518 4.58477 3.61503
0.04618 4.61895 3.34472 0.61958 0.77255 0.40980 1.09000
435 2.37494 5.16219 2.51361 2.23922 4.46716 2.96723 3.73431 3.92587 2.30957 3.04071 4.21422 3.02504 2.67662 2.76215 2.97347 2.61053 2.98432 2.63823 5.61995 4.23422 753 - -
2.68675 4.42049 2.77597 2.73056 3.46389 2.40628 3.72594 3.29196 2.67658 2.69331 4.24546 2.90444 2.73863 3.18094 2.89848 2.37944 2.77535 2.98242 4.57600 3.61575
0.47884 1.02740 3.79144 2.37502 0.09763 0.54282 0.87014
436 2.34062 4.49914 2.90871 1.73983 3.58267 3.55243 3.74964 3.75808 2.52311 2.93698 3.50448 3.05774 3.94371 2.58326 2.66413 2.51865 2.97473 3.40129 5.53185 4.17129 777 - -
2.68618 4.42225 2.77519 2.73123 3.46354 2.40513 3.72494 3.29354 2.67741 2.69355 4.24690 2.90347 2.73739 3.18146 2.89801 2.37887 2.77519 2.98518 4.58477 3.61503
0.01439 4.64434 5.36669 0.61958 0.77255 0.57641 0.82533

437 2.78591 5.29427 2.06190 2.35773 4.63230 3.52407 3.73787 4.11785 2.33585 3.59634 4.33953 2.36409 2.60322 2.55716 2.88194 2.31887 2.93457 3.68166 5.72867 4.31420 778 - -
2.68618 4.42225 2.77519 2.73123 3.46354 2.40513 3.72494 3.29354 2.67741 2.69355 4.24690 2.90347 2.73739 3.18146 2.89801 2.37887 2.77519 2.98518 4.58477 3.61503
0.01439 4.64434 5.36669 0.61958 0.77255 0.57641 0.82533
438 2.65649 4.92947 3.02379 2.44073 4.16335 3.57990 3.78413 3.12868 1.79551 2.70394 3.76796 2.95017 3.96934 2.84709 2.90210 2.78397 2.70424 2.44048 5.43490 4.10389 779 - -
2.68618 4.42225 2.77519 2.73123 3.46354 2.40513 3.72494 3.29354 2.67741 2.69355 4.24690 2.90347 2.73739 3.18146 2.89801 2.37887 2.77519 2.98518 4.58477 3.61503
0.01439 4.64434 5.36669 0.61958 0.77255 0.48526 0.95591
439 2.75932 5.11362 2.51623 2.13723 4.39847 1.87078 3.41982 3.50375 2.51835 3.39300 3.71422 3.04994 3.94894 2.87353 2.99890 2.75394 2.75076 3.47171 5.58377 3.54482 780 - -
2.68618 4.42225 2.77519 2.73123 3.46354 2.40513 3.72494 3.29354 2.67741 2.69355 4.24690 2.90347 2.73739 3.18146 2.89801 2.37887 2.77519 2.98518 4.58477 3.61503
0.01397 4.67363 5.39597 0.61958 0.77255 0.52130 0.90079
440 2.64787 4.60965 3.47864 2.92669 3.78944 1.70271 3.94920 2.55067 2.76945 2.85001 3.72694 2.89203 4.08341 3.21525 3.06066 2.93048 3.00567 2.29364 5.19415 3.93846 781 - -
2.68616 4.42300 2.77318 2.73057 3.46356 2.40476 3.72570 3.29356 2.67775 2.69352 4.24765 2.90373 2.73803 3.18222 2.89860 2.37937 2.77506 2.98429 4.58552 3.61578
0.85150 1.07305 1.46420 1.92127 0.15832 0.52130 0.90079
441 2.69410 4.74230 3.18664 2.63209 3.93879 3.55207 3.50803 2.33015 2.13069 2.98206 3.82604 2.22217 3.94065 2.94797 3.00016 2.77335 2.74987 3.05435 5.26218 3.35053 797 - -
2.68618 4.42225 2.77519 2.73123 3.46354 2.40513 3.72494 3.29354 2.67741 2.69355 4.24690 2.90347 2.73739 3.18146 2.89801 2.37887 2.77519 2.98518 4.58477 3.61503
0.01810 4.41650 5.13885 0.61958 0.77255 0.83241 0.57093
442 2.55839 4.98643 3.07237 1.63987 4.25675 3.52446 3.71127 3.17754 2.44429 3.24382 3.70392 3.03942 3.91672 2.84620 2.15435 2.73764 2.95222 3.32178 5.46645 4.12568 798 - -
2.68618 4.42225 2.77519 2.73123 3.46354 2.40513 3.72494 3.29354 2.67741 2.69355 4.24690 2.90347 2.73739 3.18146 2.89801 2.37887 2.77519 2.98518 4.58477 3.61503
0.01769 4.43923 5.16158 0.61958 0.77255 0.86509 0.54648
443 3.45170 4.72579 5.30311 4.73353 3.35092 4.79332 5.15611 1.96309 4.60188 0.98233 2.48409 4.90149 4.97257 4.64709 4.65011 4.15089 3.67565 1.62788 5.40635 4.35468 799 - -
2.68618 4.42225 2.77519 2.73123 3.46354 2.40513 3.72494 3.29354 2.67741 2.69355 4.24690 2.90347 2.73739 3.18146 2.89801 2.37887 2.77519 2.98518 4.58477 3.61503
0.01769 4.43923 5.16158 0.61958 0.77255 0.86509 0.54648
444 3.67816 5.29957 4.34802 4.29028 5.18154 3.95733 5.28456 4.92125 4.40559 4.46215 5.55824 4.52588 0.23060 4.77253 4.55260 3.87056 4.16715 4.49177 6.16841 5.32016 800 - -
2.68618 4.42225 2.77519 2.73123 3.46354 2.40513 3.72494 3.29354 2.67741 2.69355 4.24690 2.90347 2.73739 3.18146 2.89801 2.37887 2.77519 2.98518 4.58477 3.61503
0.01769 4.43923 5.16158 0.61958 0.77255 0.86509 0.54648
445 2.71980 4.83437 3.17834 2.61030 4.05771 3.55817 2.62725 3.46159 2.50589 2.86652 2.66557 2.68779 3.94421 2.91258 2.11947 2.77598 2.74270 3.16106 5.33623 4.02910 801 - -
2.68618 4.42225 2.77519 2.73123 3.46354 2.40513 3.72494 3.29354 2.67741 2.69355 4.24690 2.90347 2.73739 3.18146 2.89801 2.37887 2.77519 2.98518 4.58477 3.61503
0.01769 4.43923 5.16158 0.61958 0.77255 0.86509 0.54648
446 3.66527 4.92331 5.44275 4.87143 2.42286 4.97492 5.19500 2.17915 4.73736 0.66762 3.00676 5.06311 5.07535 4.68957 4.74710 4.34112 3.87432 2.45395 5.31616 4.22412 802 - -
2.68618 4.42225 2.77519 2.73123 3.46354 2.40513 3.72494 3.29354 2.67741 2.69355 4.24690 2.90347 2.73739 3.18146 2.89801 2.37887 2.77519 2.98518 4.58477 3.61503
0.01769 4.43923 5.16158 0.61958 0.77255 0.86509 0.54648
447 2.90132 4.56850 5.18105 4.69653 3.93087 4.70976 5.39259 1.46408 4.60661 2.46558 3.71350 4.85831 5.02948 4.86920 4.78716 4.11153 3.55995 0.80121 5.88335 4.66690 803 - -
2.68618 4.42225 2.77519 2.73123 3.46354 2.40513 3.72494 3.29354 2.67741 2.69355 4.24690 2.90347 2.73739 3.18146 2.89801 2.37887 2.77519 2.98518 4.58477 3.61503
0.01769 4.43923 5.16158 0.61958 0.77255 0.30559 1.33442
448 2.99972 4.97445 3.39208 2.93680 3.76969 3.77524 3.95380 3.61902 2.78044 3.23383 4.12095 2.14840 4.20586 3.23847 2.74532 3.08211 3.03103 3.34309 5.20105 1.28378 804 - -
2.68618 4.42225 2.77519 2.73123 3.46354 2.40513 3.72494 3.29354 2.67741 2.69355 4.24690 2.90347 2.73739 3.18146 2.89801 2.37887 2.77519 2.98518 4.58477 3.61503

0.01372 4.69125 5.41360 0.61958 0.77255 0.48576 0.95510

449 3.61119 4.80230 5.69574 5.23114 4.13318 5.24643 5.98409 1.02580 5.17563 2.33649 3.86132 5.39858 5.44914 5.41906 5.33942 4.68447 3.86977 0.93138 6.27269 5.06341 805 - -

2.68618 4.42225 2.77519 2.73123 3.46354 2.40513 3.72494 3.29354 2.67741 2.69355 4.24690 2.90347 2.73739 3.18146 2.89801 2.37887 2.77519 2.98518 4.58477 3.61503

0.01372 4.69125 5.41360 0.61958 0.77255 0.48576 0.95510

450 2.77270 4.80625 3.29664 2.73540 4.01217 3.64050 3.85860 2.95751 2.41503 3.05164 3.65777 3.10565 4.03035 3.05118 2.89565 1.40546 2.88716 2.82524 5.34372 4.04863 806 - -

2.68618 4.42225 2.77519 2.73123 3.46354 2.40513 3.72494 3.29354 2.67741 2.69355 4.24690 2.90347 2.73739 3.18146 2.89801 2.37887 2.77519 2.98518 4.58477 3.61503

0.01372 4.69125 5.41360 0.61958 0.77255 0.48576 0.95510

451 2.83554 3.61509 3.63961 3.06743 3.38272 3.76729 3.97610 3.15037 2.92737 2.83942 3.73414 3.47768 4.15705 3.30425 1.37399 2.76718 3.07128 2.91525 5.06871 2.51326 807 - -

2.68618 4.42225 2.77519 2.73123 3.46354 2.40513 3.72494 3.29354 2.67741 2.69355 4.24690 2.90347 2.73739 3.18146 2.89801 2.37887 2.77519 2.98518 4.58477 3.61503

0.01372 4.69125 5.41360 0.61958 0.77255 0.48576 0.95510

452 3.21260 5.61502 2.96525 0.86830 4.98487 3.73109 3.97667 4.43558 2.57627 3.89046 4.71300 3.20883 4.21524 2.92423 2.25722 3.15166 3.44187 4.04277 5.98334 4.64114 808 - -

2.68618 4.42225 2.77519 2.73123 3.46354 2.40513 3.72494 3.29354 2.67741 2.69355 4.24690 2.90347 2.73739 3.18146 2.89801 2.37887 2.77519 2.98518 4.58477 3.61503

0.01372 4.69125 5.41360 0.61958 0.77255 0.48576 0.95510

453 3.00580 5.23481 3.41095 2.79083 4.55227 3.76595 2.77043 3.05137 1.12761 3.48197 4.30090 3.27464 4.13695 2.97341 2.37571 3.01232 3.21084 3.34727 5.63125 4.34897 809 - -

2.68618 4.42225 2.77519 2.73123 3.46354 2.40513 3.72494 3.29354 2.67741 2.69355 4.24690 2.90347 2.73739 3.18146 2.89801 2.37887 2.77519 2.98518 4.58477 3.61503

0.01372 4.69125 5.41360 0.61958 0.77255 0.48576 0.95510

454 3.30641 5.29567 3.99490 3.42692 5.00894 3.90578 4.19532 4.46194 2.48122 3.93205 4.82037 3.73576 4.42751 3.36080 0.61066 2.60827 3.60343 4.06467 5.93169 4.76649 810 - -

2.68618 4.42225 2.77519 2.73123 3.46354 2.40513 3.72494 3.29354 2.67741 2.69355 4.24690 2.90347 2.73739 3.18146 2.89801 2.37887 2.77519 2.98518 4.58477 3.61503

0.01372 4.69125 5.41360 0.61958 0.77255 0.48576 0.95510

455 2.80649 5.16383 2.51070 2.39243 4.45125 3.34950 3.78991 3.89726 2.31974 2.83852 4.22767 3.06352 1.67117 2.91397 3.04328 2.79667 3.04088 3.29660 5.63573 4.26056 811 - -

2.68618 4.42225 2.77519 2.73123 3.46354 2.40513 3.72494 3.29354 2.67741 2.69355 4.24690 2.90347 2.73739 3.18146 2.89801 2.37887 2.77519 2.98518 4.58477 3.61503

0.24616 4.69125 1.56527 0.61958 0.77255 0.48576 0.95510

456 2.75035 4.89348 3.01298 2.64333 4.32920 1.54576 3.90649 3.75356 2.73178 3.36601 4.19314 2.21264 3.97581 2.77794 3.16865 2.80305 3.04664 2.72633 5.59763 4.26742 812 - -

2.68618 4.42225 2.77519 2.73123 3.46354 2.40513 3.72494 3.29354 2.67741 2.69355 4.24690 2.90347 2.73739 3.18146 2.89801 2.37887 2.77519 2.98518 4.58477 3.61503

0.01729 4.46237 5.18472 0.61958 0.77255 0.31509 1.30831

457 2.82040 4.42463 3.89460 3.32751 2.28483 3.84103 3.34821 2.63249 3.25798 2.62083 3.53533 2.48917 4.21507 3.52847 3.55083 2.67879 3.05203 2.52110 4.86397 1.95378 813 - -

2.68618 4.42225 2.77519 2.73123 3.46354 2.40513 3.72494 3.29354 2.67741 2.69355 4.24690 2.90347 2.73739 3.18146 2.89801 2.37887 2.77519 2.98518 4.58477 3.61503

0.01372 4.69125 5.41360 0.61958 0.77255 0.48576 0.95510

458 2.63366 5.18806 2.16206 2.48732 4.15236 3.54757 3.39249 3.96292 2.48629 2.79568 4.23782 3.02924 3.40881 2.44642 2.05207 2.65507 2.99239 3.38921 5.64081 4.25135 814 - -

2.68618 4.42225 2.77519 2.73123 3.46354 2.40513 3.72494 3.29354 2.67741 2.69355 4.24690 2.90347 2.73739 3.18146 2.89801 2.37887 2.77519 2.98518 4.58477 3.61503

0.01372 4.69125 5.41360 0.61958 0.77255 0.48576 0.95510

459 2.81917 5.05610 3.11200 2.42272 4.31471 3.60376 1.51708 3.52444 2.59761 3.32999 4.13984 2.56635 4.01306 2.97060 3.04650 2.83897 2.59359 3.23153 5.55080 4.20693 815 - -

2.68618 4.42225 2.77519 2.73123 3.46354 2.40513 3.72494 3.29354 2.67741 2.69355 4.24690 2.90347 2.73739 3.18146 2.89801 2.37887 2.77519 2.98518 4.58477 3.61503

0.01372 4.69125 5.41360 0.61958 0.77255 0.48576 0.95510

460 3.05049 5.35012 2.88314 2.65820 4.70226 1.85710 1.61565 4.29922 2.89547 3.83058 4.65106 1.89747 4.15642 3.22603 3.35205 3.03654 3.34588 3.88376 5.92648 4.50872 816 - -

2.68618 4.42225 2.77519 2.73123 3.46354 2.40513 3.72494 3.29354 2.67741 2.69355 4.24690 2.90347 2.73739 3.18146 2.89801 2.37887 2.77519 2.98518 4.58477 3.61503
0.01372 4.69125 5.41360 0.61958 0.77255 0.48576 0.95510
461 3.13576 4.92825 3.78452 3.23884 2.31181 3.99390 3.91721 3.48662 1.55749 3.07309 4.02974 3.62114 4.36566 3.21415 3.34681 3.25846 3.35861 3.26169 4.68965 1.56521 817 - -
2.68618 4.42225 2.77519 2.73123 3.46354 2.40513 3.72494 3.29354 2.67741 2.69355 4.24690 2.90347 2.73739 3.18146 2.89801 2.37887 2.77519 2.98518 4.58477 3.61503
0.01372 4.69125 5.41360 0.61958 0.77255 0.48576 0.95510
462 4.00044 5.82047 4.22623 3.77023 5.41655 4.30187 4.54371 4.93375 0.30355 4.34015 5.31944 4.12967 4.79441 3.73314 2.98517 4.02751 4.21921 4.61795 6.16465 5.14805 818 - -
2.68618 4.42225 2.77519 2.73123 3.46354 2.40513 3.72494 3.29354 2.67741 2.69355 4.24690 2.90347 2.73739 3.18146 2.89801 2.37887 2.77519 2.98518 4.58477 3.61503
0.01372 4.69125 5.41360 0.61958 0.77255 0.48576 0.95510
463 0.52342 3.74569 4.63472 4.33452 4.76774 3.35578 5.05085 3.97347 4.22389 3.84425 4.70954 4.06136 4.16759 4.44113 4.38057 2.82919 2.39329 3.45309 6.19072 5.06312 819 - -
2.68618 4.42225 2.77519 2.73123 3.46354 2.40513 3.72494 3.29354 2.67741 2.69355 4.24690 2.90347 2.73739 3.18146 2.89801 2.37887 2.77519 2.98518 4.58477 3.61503
0.01372 4.69125 5.41360 0.61958 0.77255 0.48576 0.95510
464 3.95902 5.58125 4.78151 4.80155 5.79700 0.13298 5.78940 5.66883 5.08014 5.17611 6.20714 4.94950 4.98168 5.34176 5.15829 4.16303 4.49355 5.04373 6.60828 5.94871 820 - -
2.68618 4.42225 2.77519 2.73123 3.46354 2.40513 3.72494 3.29354 2.67741 2.69355 4.24690 2.90347 2.73739 3.18146 2.89801 2.37887 2.77519 2.98518 4.58477 3.61503
0.01372 4.69125 5.41360 0.61958 0.77255 0.48576 0.95510
465 0.59419 4.73695 3.64699 3.48647 4.88295 3.43718 4.69866 4.31060 3.68715 4.02081 4.86296 2.36856 4.19723 3.96747 4.01190 2.93358 3.26575 3.74840 6.21287 4.96602 821 - -
2.68618 4.42225 2.77519 2.73123 3.46354 2.40513 3.72494 3.29354 2.67741 2.69355 4.24690 2.90347 2.73739 3.18146 2.89801 2.37887 2.77519 2.98518 4.58477 3.61503
0.01372 4.69125 5.41360 0.61958 0.77255 0.48576 0.95510
466 3.42253 4.81802 4.87358 3.71802 3.40543 4.57154 4.86339 2.62870 4.10731 1.17108 1.20528 4.56488 4.83773 4.32950 4.24315 3.90831 3.64765 2.71150 5.33171 4.22916 822 - -
2.68618 4.42225 2.77519 2.73123 3.46354 2.40513 3.72494 3.29354 2.67741 2.69355 4.24690 2.90347 2.73739 3.18146 2.89801 2.37887 2.77519 2.98518 4.58477 3.61503
0.01372 4.69125 5.41360 0.61958 0.77255 0.48576 0.95510
467 3.12424 5.44888 3.20248 2.76216 4.87209 3.75415 3.91780 4.29082 1.67696 3.75807 4.56989 1.19234 4.18961 3.04356 2.62235 3.09494 3.10472 3.90557 5.85098 4.55192 823 - -
2.68618 4.42225 2.77519 2.73123 3.46354 2.40513 3.72494 3.29354 2.67741 2.69355 4.24690 2.90347 2.73739 3.18146 2.89801 2.37887 2.77519 2.98518 4.58477 3.61503
0.01372 4.69125 5.41360 0.61958 0.77255 0.48576 0.95510
468 1.73371 4.86213 3.22405 2.33842 2.81889 3.31785 3.62746 3.48276 2.67175 3.11659 3.94989 3.18110 4.00926 2.93125 3.12114 2.27215 2.65299 3.18539 5.39260 4.08086 824 - -
2.68618 4.42225 2.77519 2.73123 3.46354 2.40513 3.72494 3.29354 2.67741 2.69355 4.24690 2.90347 2.73739 3.18146 2.89801 2.37887 2.77519 2.98518 4.58477 3.61503
0.01372 4.69125 5.41360 0.61958 0.77255 0.48576 0.95510
469 2.22533 4.38988 4.17320 3.62170 3.50536 2.55661 4.28465 2.77680 3.51289 1.20445 2.90584 3.86628 4.28919 3.76890 3.73970 3.17747 3.09872 2.60611 5.05616 3.86527 825 - -
2.68618 4.42225 2.77519 2.73123 3.46354 2.40513 3.72494 3.29354 2.67741 2.69355 4.24690 2.90347 2.73739 3.18146 2.89801 2.37887 2.77519 2.98518 4.58477 3.61503
0.01372 4.69125 5.41360 0.61958 0.77255 0.48576 0.95510
470 2.91447 4.57853 5.19294 4.62346 3.59050 4.58454 4.98682 1.87124 4.47341 1.71110 1.97042 4.71380 4.87002 4.60876 4.53691 3.92634 3.50984 1.25703 5.40287 4.25713 826 - -
2.68696 4.41865 2.77496 2.73204 3.46230 2.40612 3.72424 3.29302 2.67745 2.69330 4.24563 2.90280 2.73838 3.18202 2.89843 2.37890 2.77580 2.98428 4.58576 3.61093
0.33198 1.27999 5.41360 2.35294 0.09992 0.48576 0.95510
471 3.39952 5.59393 3.83362 2.77585 5.08388 4.02847 3.91729 4.41754 1.53780 3.81001 4.65778 3.51505 4.36917 3.03792 0.95945 3.37135 3.54929 4.07852 5.82088 3.90771 873 - -
2.68618 4.42225 2.77519 2.73123 3.46354 2.40513 3.72494 3.29354 2.67741 2.69355 4.24690 2.90347 2.73739 3.18146 2.89801 2.37887 2.77519 2.98518 4.58477 3.61503
0.26971 4.69125 1.48182 0.61958 0.77255 0.48576 0.95510

472 1.96714 4.38677 4.39871 3.96172 3.93321 3.71659 4.70595 2.41213 3.87159 2.77043 3.83060 4.04879 4.32454 4.13913 4.08870 3.13093 2.63015 0.98637 5.55942 4.36191 874 - -
2.68618 4.42225 2.77519 2.73123 3.46354 2.40513 3.72494 3.29354 2.67741 2.69355 4.24690 2.90347 2.73739 3.18146 2.89801 2.37887 2.77519 2.98518 4.58477 3.61503
0.01769 4.43923 5.16158 0.61958 0.77255 0.86509 0.54648
473 2.33019 4.44609 3.77691 3.62028 4.74958 3.19435 4.69708 4.19788 3.72530 3.92189 4.76787 3.67668 3.99616 4.00586 3.98006 0.60025 3.03864 3.56884 6.10449 4.88759 875 - -
2.68618 4.42225 2.77519 2.73123 3.46354 2.40513 3.72494 3.29354 2.67741 2.69355 4.24690 2.90347 2.73739 3.18146 2.89801 2.37887 2.77519 2.98518 4.58477 3.61503
0.01769 4.43923 5.16158 0.61958 0.77255 0.86509 0.54648
474 1.04250 4.37672 3.87698 3.63526 4.75131 1.47919 4.66838 4.18940 3.72452 3.87404 4.67176 3.65998 3.94612 3.96162 4.00133 2.30823 2.96051 3.53076 6.08694 4.91441 876 - -
2.68618 4.42225 2.77519 2.73123 3.46354 2.40513 3.72494 3.29354 2.67741 2.69355 4.24690 2.90347 2.73739 3.18146 2.89801 2.37887 2.77519 2.98518 4.58477 3.61503
0.01769 4.43923 5.16158 0.61958 0.77255 0.86509 0.54648
475 3.04255 4.39776 4.74399 4.17803 3.52837 4.30244 4.68759 1.55477 3.35220 1.75228 3.42213 4.34861 4.62218 4.25355 4.17526 3.62866 3.28207 1.29927 5.24740 4.06562 877 - -
2.68618 4.42225 2.77519 2.73123 3.46354 2.40513 3.72494 3.29354 2.67741 2.69355 4.24690 2.90347 2.73739 3.18146 2.89801 2.37887 2.77519 2.98518 4.58477 3.61503
0.01769 4.43923 5.16158 0.61958 0.77255 0.86509 0.54648
476 3.67804 4.94456 5.41112 4.83327 3.23312 4.98948 5.27107 2.18018 4.67970 0.98307 1.38276 5.06755 5.08297 4.67109 4.71361 4.35624 3.88663 2.58149 5.40702 4.41230 878 - -
2.68618 4.42225 2.77519 2.73123 3.46354 2.40513 3.72494 3.29354 2.67741 2.69355 4.24690 2.90347 2.73739 3.18146 2.89801 2.37887 2.77519 2.98518 4.58477 3.61503
0.01769 4.43923 5.16158 0.61958 0.77255 0.86509 0.54648
477 2.48578 4.42331 3.79515 3.49599 4.57748 3.21222 4.53015 3.96278 3.50807 3.68284 4.51898 3.61220 3.96891 3.80967 3.80298 1.41088 1.11769 3.41983 5.92929 4.71969 879 - -
2.68618 4.42225 2.77519 2.73123 3.46354 2.40513 3.72494 3.29354 2.67741 2.69355 4.24690 2.90347 2.73739 3.18146 2.89801 2.37887 2.77519 2.98518 4.58477 3.61503
0.01769 4.43923 5.16158 0.61958 0.77255 0.30559 1.33442
478 2.61565 4.15635 2.66553 2.49356 4.49943 3.55154 3.53324 3.96007 2.42795 3.32704 4.24397 1.53003 3.94990 2.55029 2.98909 2.67797 2.92647 3.56222 5.64713 4.25927 880 - -
2.68618 4.42225 2.77519 2.73123 3.46354 2.40513 3.72494 3.29354 2.67741 2.69355 4.24690 2.90347 2.73739 3.18146 2.89801 2.37887 2.77519 2.98518 4.58477 3.61503
0.01372 4.69125 5.41360 0.61958 0.77255 0.48576 0.95510
479 1.68861 2.51350 3.89887 3.37175 3.83563 1.94919 4.24079 3.20553 3.31749 2.92357 3.81023 3.65188 4.13736 3.60084 3.63238 2.49747 3.03387 2.93060 5.28283 2.74761 881 - -
2.68618 4.42225 2.77519 2.73123 3.46354 2.40513 3.72494 3.29354 2.67741 2.69355 4.24690 2.90347 2.73739 3.18146 2.89801 2.37887 2.77519 2.98518 4.58477 3.61503
0.01372 4.69125 5.41360 0.61958 0.77255 0.48576 0.95510
480 2.53184 5.39170 2.55935 1.93369 4.71196 3.55991 3.83513 4.19387 2.47176 3.68751 4.45159 2.90721 1.61752 2.84339 3.13520 2.85176 3.03220 3.77140 5.83573 4.41543 882 - -
2.68618 4.42225 2.77519 2.73123 3.46354 2.40513 3.72494 3.29354 2.67741 2.69355 4.24690 2.90347 2.73739 3.18146 2.89801 2.37887 2.77519 2.98518 4.58477 3.61503
0.01372 4.69125 5.41360 0.61958 0.77255 0.48576 0.95510
481 4.02494 5.21280 5.33684 5.02492 1.06049 4.88564 3.99004 2.38448 4.82815 2.93566 4.19317 4.63722 5.16976 4.70121 4.75856 4.24760 4.24047 3.49468 4.11092 1.15153 883 - -
2.68618 4.42225 2.77519 2.73123 3.46354 2.40513 3.72494 3.29354 2.67741 2.69355 4.24690 2.90347 2.73739 3.18146 2.89801 2.37887 2.77519 2.98518 4.58477 3.61503
0.01372 4.69125 5.41360 0.61958 0.77255 0.48576 0.95510
482 3.37396 4.64849 5.34682 4.79365 3.69134 4.75064 5.19514 1.25241 4.65710 1.83001 2.27064 4.89023 5.01287 4.79354 4.72361 4.10846 3.35153 1.40042 5.57268 4.42833 884 - -
2.68618 4.42225 2.77519 2.73123 3.46354 2.40513 3.72494 3.29354 2.67741 2.69355 4.24690 2.90347 2.73739 3.18146 2.89801 2.37887 2.77519 2.98518 4.58477 3.61503
0.01372 4.69125 5.41360 0.61958 0.77255 0.48576 0.95510
483 2.01118 4.52409 4.62131 4.08793 3.60686 4.11187 4.66718 2.53238 3.95514 0.98068 3.50788 4.24881 4.54720 4.17889 4.12650 3.46350 3.05783 2.14787 5.30836 4.14575 885 - -
2.68618 4.42225 2.77519 2.73123 3.46354 2.40513 3.72494 3.29354 2.67741 2.69355 4.24690 2.90347 2.73739 3.18146 2.89801 2.37887 2.77519 2.98518 4.58477 3.61503

0.01372 4.69125 5.41360 0.61958 0.77255 0.48576 0.95510
484 2.86871 4.63875 3.69983 3.21368 3.92073 3.77802 4.21900 1.84106 3.17255 2.89478 3.85375 1.39007 4.24322 3.50958 3.52782 3.09008 2.65086 2.59036 5.39724 4.14466 886 - -
2.68618 4.42225 2.77519 2.73123 3.46354 2.40513 3.72494 3.29354 2.67741 2.69355 4.24690 2.90347 2.73739 3.18146 2.89801 2.37887 2.77519 2.98518 4.58477 3.61503
0.01372 4.69125 5.41360 0.61958 0.77255 0.48576 0.95510
485 3.58536 4.86754 5.44964 4.88329 1.75374 4.86639 5.10128 2.48433 4.72807 1.06186 3.24720 4.98342 5.06290 4.73840 4.73686 4.22132 3.80768 1.60786 5.32186 4.12881 887 - -
2.68618 4.42225 2.77519 2.73123 3.46354 2.40513 3.72494 3.29354 2.67741 2.69355 4.24690 2.90347 2.73739 3.18146 2.89801 2.37887 2.77519 2.98518 4.58477 3.61503
0.01372 4.69125 5.41360 0.61958 0.77255 0.48576 0.95510
486 4.11833 6.15002 0.23244 3.22676 5.69734 4.06894 4.94997 5.52749 4.21940 4.99136 6.05124 3.80265 4.77227 4.23299 4.79197 4.03199 4.49046 5.09507 6.65939 5.52175 888 - -
2.68618 4.42225 2.77519 2.73123 3.46354 2.40513 3.72494 3.29354 2.67741 2.69355 4.24690 2.90347 2.73739 3.18146 2.89801 2.37887 2.77519 2.98518 4.58477 3.61503
0.01372 4.69125 5.41360 0.61958 0.77255 0.48576 0.95510
487 1.36129 0.83827 4.85802 4.64619 4.75242 3.41918 5.21481 3.87630 4.49004 3.81570 4.73957 4.21095 4.24262 4.68328 4.55838 2.91581 3.21784 3.40966 6.20871 5.09418 889 - -
2.68618 4.42225 2.77519 2.73123 3.46354 2.40513 3.72494 3.29354 2.67741 2.69355 4.24690 2.90347 2.73739 3.18146 2.89801 2.37887 2.77519 2.98518 4.58477 3.61503
0.01372 4.69125 5.41360 0.61958 0.77255 0.48576 0.95510
488 4.11833 6.15002 0.23244 3.22676 5.69734 4.06894 4.94997 5.52749 4.21940 4.99136 6.05124 3.80265 4.77227 4.23299 4.79197 4.03199 4.49046 5.09507 6.65939 5.52175 890 - -
2.68618 4.42225 2.77519 2.73123 3.46354 2.40513 3.72494 3.29354 2.67741 2.69355 4.24690 2.90347 2.73739 3.18146 2.89801 2.37887 2.77519 2.98518 4.58477 3.61503
0.26971 4.69125 1.48182 0.61958 0.77255 0.48576 0.95510
489 3.00404 4.74282 3.70365 3.22700 3.36385 3.84137 1.50784 3.17656 2.95540 2.69386 1.84542 3.59638 4.26581 3.43028 3.22996 3.18562 3.25472 3.01765 4.92207 3.47291 891 - -
2.68618 4.42225 2.77519 2.73123 3.46354 2.40513 3.72494 3.29354 2.67741 2.69355 4.24690 2.90347 2.73739 3.18146 2.89801 2.37887 2.77519 2.98518 4.58477 3.61503
0.01769 4.43923 5.16158 0.61958 0.77255 0.30559 1.33442
490 4.75374 5.68624 5.56004 5.49758 1.24011 5.22464 3.87964 4.30833 5.28007 3.45144 4.83341 4.76791 5.48344 4.94026 5.08606 4.64217 4.95044 4.27311 3.95421 0.63370 892 - -
2.68618 4.42225 2.77519 2.73123 3.46354 2.40513 3.72494 3.29354 2.67741 2.69355 4.24690 2.90347 2.73739 3.18146 2.89801 2.37887 2.77519 2.98518 4.58477 3.61503
0.01372 4.69125 5.41360 0.61958 0.77255 0.48576 0.95510
491 2.16167 4.40288 3.81333 3.24306 3.17439 3.78888 4.06568 1.96423 3.17939 2.60682 3.51436 3.59996 4.16836 2.18153 3.48938 2.66130 2.85748 2.28596 5.00535 3.78696 893 - -
2.68618 4.42225 2.77519 2.73123 3.46354 2.40513 3.72494 3.29354 2.67741 2.69355 4.24690 2.90347 2.73739 3.18146 2.89801 2.37887 2.77519 2.98518 4.58477 3.61503
0.01372 4.69125 5.41360 0.61958 0.77255 0.48576 0.95510
492 2.97730 5.08572 3.15352 2.80039 3.94035 3.67692 3.98504 3.84416 2.89459 3.43181 4.29924 1.48695 2.01512 3.23783 3.32262 3.01977 3.24989 3.52073 5.36344 2.33403 894 - -
2.68618 4.42225 2.77519 2.73123 3.46354 2.40513 3.72494 3.29354 2.67741 2.69355 4.24690 2.90347 2.73739 3.18146 2.89801 2.37887 2.77519 2.98518 4.58477 3.61503
0.01372 4.69125 5.41360 0.61958 0.77255 0.48576 0.95510
493 2.97778 5.51057 2.26120 1.70448 4.83146 3.56463 3.88974 4.32657 2.71878 3.45375 4.57691 1.46686 3.60672 3.01013 3.24358 2.91339 3.06369 3.89173 5.94991 4.50769 895 - -
2.68618 4.42225 2.77519 2.73123 3.46354 2.40513 3.72494 3.29354 2.67741 2.69355 4.24690 2.90347 2.73739 3.18146 2.89801 2.37887 2.77519 2.98518 4.58477 3.61503
0.01372 4.69125 5.41360 0.61958 0.77255 0.48576 0.95510
494 1.96227 3.54932 4.17807 3.76323 4.71442 3.35787 4.71152 4.13436 3.72171 3.81731 4.62805 3.81782 1.47487 3.99325 4.02193 1.21385 3.09755 3.56977 6.06534 4.87809 896 - -
2.68618 4.42225 2.77519 2.73123 3.46354 2.40513 3.72494 3.29354 2.67741 2.69355 4.24690 2.90347 2.73739 3.18146 2.89801 2.37887 2.77519 2.98518 4.58477 3.61503
0.01372 4.69125 5.41360 0.61958 0.77255 0.48576 0.95510
495 2.58396 5.24457 1.76932 2.48640 4.57288 3.55440 3.74512 4.04248 1.77907 3.53971 3.57841 3.02990 3.95202 2.69930 2.77198 2.75694 2.89630 3.62668 5.68486 4.29040 897 - -

2.68618 4.42225 2.77519 2.73123 3.46354 2.40513 3.72494 3.29354 2.67741 2.69355 4.24690 2.90347 2.73739 3.18146 2.89801 2.37887 2.77519 2.98518 4.58477 3.61503
0.01372 4.69125 5.41360 0.61958 0.77255 0.48576 0.95510
496 1.66425 4.25790 4.21761 3.63580 2.24662 3.85965 4.18034 2.70386 3.51959 2.45934 3.38149 3.84652 4.23979 3.74330 3.70709 2.90453 2.61838 2.03929 4.87303 3.04092 898 - -
2.68618 4.42225 2.77519 2.73123 3.46354 2.40513 3.72494 3.29354 2.67741 2.69355 4.24690 2.90347 2.73739 3.18146 2.89801 2.37887 2.77519 2.98518 4.58477 3.61503
0.01372 4.69125 5.41360 0.61958 0.77255 0.48576 0.95510
497 2.97363 4.34584 4.79498 4.20108 3.04023 4.16907 4.51309 1.97081 4.03154 1.29105 3.34058 4.27845 3.59328 4.18398 4.09974 3.48357 2.91458 1.68748 5.01929 3.85268 899 - -
2.68618 4.42225 2.77519 2.73123 3.46354 2.40513 3.72494 3.29354 2.67741 2.69355 4.24690 2.90347 2.73739 3.18146 2.89801 2.37887 2.77519 2.98518 4.58477 3.61503
0.01372 4.69125 5.41360 0.61958 0.77255 0.48576 0.95510
498 2.84779 5.18752 3.18048 2.39275 4.49771 3.62978 3.53033 3.68896 1.93753 3.26357 4.24440 3.12348 4.01408 2.71785 1.53419 2.83987 3.06985 3.13780 4.69971 4.27976 900 - -
2.68618 4.42225 2.77519 2.73123 3.46354 2.40513 3.72494 3.29354 2.67741 2.69355 4.24690 2.90347 2.73739 3.18146 2.89801 2.37887 2.77519 2.98518 4.58477 3.61503
0.01372 4.69125 5.41360 0.61958 0.77255 0.48576 0.95510
499 2.78765 5.28659 2.66741 1.70352 4.63262 3.55047 3.34457 4.11322 2.23175 3.25917 4.32920 3.01872 3.94681 2.29973 2.21106 2.74992 3.01761 3.67823 5.71394 4.31075 901 - -
2.68618 4.42225 2.77519 2.73123 3.46354 2.40513 3.72494 3.29354 2.67741 2.69355 4.24690 2.90347 2.73739 3.18146 2.89801 2.37887 2.77519 2.98518 4.58477 3.61503
0.01372 4.69125 5.41360 0.61958 0.77255 0.48576 0.95510
500 1.10033 4.55374 4.11016 3.73064 4.81046 1.94829 4.72577 4.24822 3.72941 3.91506 4.71181 3.80093 4.11016 3.99381 4.04063 2.19055 1.78687 3.63990 6.14216 4.95281 902 - -
2.68618 4.42225 2.77519 2.73123 3.46354 2.40513 3.72494 3.29354 2.67741 2.69355 4.24690 2.90347 2.73739 3.18146 2.89801 2.37887 2.77519 2.98518 4.58477 3.61503
0.01372 4.69125 5.41360 0.61958 0.77255 0.48576 0.95510
501 3.54261 4.80907 5.46851 4.90458 3.55068 4.90640 5.30027 1.84474 4.76802 1.65300 1.30558 5.03826 5.10641 4.82881 4.80873 4.26793 3.77169 1.60742 5.56864 4.49292 903 - -
2.68618 4.42225 2.77519 2.73123 3.46354 2.40513 3.72494 3.29354 2.67741 2.69355 4.24690 2.90347 2.73739 3.18146 2.89801 2.37887 2.77519 2.98518 4.58477 3.61503
0.01372 4.69125 5.41360 0.61958 0.77255 0.48576 0.95510
502 2.98696 0.71909 4.81619 4.60653 4.89039 3.66004 5.27532 4.26026 4.46976 4.03721 4.97834 4.38869 1.49441 4.74769 4.57463 3.22155 3.51666 3.78523 6.22216 5.15810 904 - -
2.68618 4.42225 2.77519 2.73123 3.46354 2.40513 3.72494 3.29354 2.67741 2.69355 4.24690 2.90347 2.73739 3.18146 2.89801 2.37887 2.77519 2.98518 4.58477 3.61503
0.01372 4.69125 5.41360 0.61958 0.77255 0.48576 0.95510
503 3.07706 4.46987 4.66491 4.11777 1.25653 4.16218 2.73220 2.29424 3.95666 2.57470 3.56827 4.14997 4.51413 4.09158 4.04390 3.46987 3.30665 2.32311 4.48331 2.41757 905 - -
2.68618 4.42225 2.77519 2.73123 3.46354 2.40513 3.72494 3.29354 2.67741 2.69355 4.24690 2.90347 2.73739 3.18146 2.89801 2.37887 2.77519 2.98518 4.58477 3.61503
0.01372 4.69125 5.41360 0.61958 0.77255 0.48576 0.95510
504 3.90721 5.14309 5.75258 5.16990 1.81717 5.21310 5.37443 2.64001 5.01978 0.91799 1.64559 5.33147 5.27069 4.89325 4.96783 4.58547 4.10607 2.92158 5.42486 4.33556 906 - -
2.68618 4.42225 2.77519 2.73123 3.46354 2.40513 3.72494 3.29354 2.67741 2.69355 4.24690 2.90347 2.73739 3.18146 2.89801 2.37887 2.77519 2.98518 4.58477 3.61503
0.01372 4.69125 5.41360 0.61958 0.77255 0.48576 0.95510
505 2.79660 4.27328 4.24749 3.66086 3.36929 3.90106 4.20412 2.36984 2.70213 1.75905 1.88616 3.87585 4.26782 3.76330 3.71945 3.02346 2.73983 2.26880 4.89182 3.70141 907 - -
2.68618 4.42225 2.77519 2.73123 3.46354 2.40513 3.72494 3.29354 2.67741 2.69355 4.24690 2.90347 2.73739 3.18146 2.89801 2.37887 2.77519 2.98518 4.58477 3.61503
0.01372 4.69125 5.41360 0.61958 0.77255 0.48576 0.95510
506 3.08898 5.65261 1.22725 2.36856 4.96099 3.01285 2.41174 4.46844 2.83753 3.94280 4.72843 3.03484 4.10734 2.23657 3.37741 3.00589 3.34553 4.02973 6.08348 4.61991 908 - -
2.68618 4.42225 2.77519 2.73123 3.46354 2.40513 3.72494 3.29354 2.67741 2.69355 4.24690 2.90347 2.73739 3.18146 2.89801 2.37887 2.77519 2.98518 4.58477 3.61503
0.01372 4.69125 5.41360 0.61958 0.77255 0.48576 0.95510

507 2.85353 5.31393 3.05767 2.33489 4.66129 3.59585 3.37014 4.12614 2.22170 3.60461 4.36219 1.88623 2.28100 2.87496 2.29064 2.81817 3.07982 3.70770 5.72978 4.34929 909 - -
2.68618 4.42225 2.77519 2.73123 3.46354 2.40513 3.72494 3.29354 2.67741 2.69355 4.24690 2.90347 2.73739 3.18146 2.89801 2.37887 2.77519 2.98518 4.58477 3.61503
0.17176 4.69125 1.90618 0.61958 0.77255 0.48576 0.95510
508 2.55777 5.17767 2.96969 2.19412 4.50214 3.49462 3.49061 3.97242 2.19484 3.47326 4.22557 2.85850 2.48997 2.35300 2.91352 2.20005 2.95254 3.33905 5.62224 4.22573 910 - -
2.68621 4.42228 2.77523 2.73127 3.46357 2.40516 3.72498 3.29357 2.67718 2.69351 4.24637 2.90350 2.73743 3.18140 2.89804 2.37890 2.77523 2.98522 4.58480 3.61506
0.53033 1.92358 1.32612 0.23349 1.56911 0.35141 1.21637
509 2.68423 4.89115 2.75624 2.38617 4.13267 2.58432 3.71736 2.66782 2.51907 2.83044 3.96420 2.42606 3.90120 2.86691 2.98427 2.45497 2.91669 3.05443 5.39497 4.05769 912 - -
2.68618 4.42225 2.77519 2.73123 3.46354 2.40513 3.72494 3.29354 2.67741 2.69355 4.24690 2.90347 2.73739 3.18146 2.89801 2.37887 2.77519 2.98518 4.58477 3.61503
0.01769 4.43923 5.16158 0.61958 0.77255 0.86509 0.54648
510 2.73277 4.95128 3.02865 2.55269 4.28475 1.67450 3.77552 3.70935 2.33251 3.29325 3.73710 2.90072 3.93405 2.80690 2.97864 2.25828 2.98546 3.35885 5.51174 4.17558 913 - -
2.68618 4.42225 2.77519 2.73123 3.46354 2.40513 3.72494 3.29354 2.67741 2.69355 4.24690 2.90347 2.73739 3.18146 2.89801 2.37887 2.77519 2.98518 4.58477 3.61503
0.01769 4.43923 5.16158 0.61958 0.77255 0.86509 0.54648
511 2.71497 5.20243 2.25823 2.27815 4.02848 3.45918 3.67041 4.01272 1.81720 3.50107 4.24956 2.36404 3.86902 2.66003 2.91386 2.55679 2.82839 3.58605 5.64100 4.23295 914 - -
2.68618 4.42225 2.77519 2.73123 3.46354 2.40513 3.72494 3.29354 2.67741 2.69355 4.24690 2.90347 2.73739 3.18146 2.89801 2.37887 2.77519 2.98518 4.58477 3.61503
0.01769 4.43923 5.16158 0.61958 0.77255 0.30559 1.33442
512 2.78779 5.29667 2.56342 1.87189 4.64189 3.15274 2.72599 4.12644 1.96874 3.59961 4.33920 2.89963 3.94464 2.74098 2.35273 2.74791 3.01925 3.68779 5.72592 4.31651 915 - -
2.68618 4.42225 2.77519 2.73123 3.46354 2.40513 3.72494 3.29354 2.67741 2.69355 4.24690 2.90347 2.73739 3.18146 2.89801 2.37887 2.77519 2.98518 4.58477 3.61503
0.01372 4.69125 5.41360 0.61958 0.77255 0.48576 0.95510
513 3.28523 2.37228 5.27281 4.71807 3.70910 4.64074 5.09252 1.40060 4.57220 1.52689 3.57039 4.79052 4.93312 4.72868 4.63847 3.99441 3.52811 1.54189 5.51233 4.34339 916 - -
2.68618 4.42225 2.77519 2.73123 3.46354 2.40513 3.72494 3.29354 2.67741 2.69355 4.24690 2.90347 2.73739 3.18146 2.89801 2.37887 2.77519 2.98518 4.58477 3.61503
0.01372 4.69125 5.41360 0.61958 0.77255 0.48576 0.95510
514 1.20410 1.48096 4.58819 4.26907 4.72876 2.00318 4.99348 4.13927 4.18858 3.85738 4.68116 4.02141 4.13945 4.38574 4.35743 2.35170 3.10962 3.55537 6.12962 5.00021 917 - -
2.68618 4.42225 2.77519 2.73123 3.46354 2.40513 3.72494 3.29354 2.67741 2.69355 4.24690 2.90347 2.73739 3.18146 2.89801 2.37887 2.77519 2.98518 4.58477 3.61503
0.01372 4.69125 5.41360 0.61958 0.77255 0.48576 0.95510
515 4.07576 5.25157 5.38401 5.07334 1.44622 4.93815 4.02836 3.53350 4.87529 1.46695 4.09315 4.68506 5.20381 4.73089 4.79671 4.30302 4.28699 3.53540 4.14188 1.14497 918 - -
2.68618 4.42225 2.77519 2.73123 3.46354 2.40513 3.72494 3.29354 2.67741 2.69355 4.24690 2.90347 2.73739 3.18146 2.89801 2.37887 2.77519 2.98518 4.58477 3.61503
0.01372 4.69125 5.41360 0.61958 0.77255 0.48576 0.95510
516 3.56809 4.80749 5.56244 5.12815 4.14402 5.06712 5.88966 1.75380 5.03971 2.57842 3.89996 5.27582 5.36481 5.32093 5.21536 4.52004 3.84855 0.52371 6.25330 5.01340 919 - -
2.68618 4.42225 2.77519 2.73123 3.46354 2.40513 3.72494 3.29354 2.67741 2.69355 4.24690 2.90347 2.73739 3.18146 2.89801 2.37887 2.77519 2.98518 4.58477 3.61503
0.01372 4.69125 5.41360 0.61958 0.77255 0.48576 0.95510
517 3.91993 5.75155 3.82828 3.67232 5.05233 4.18515 4.80482 4.91405 3.45259 4.31244 5.39064 4.11169 4.79262 0.29938 3.68601 3.96911 4.25230 4.60469 6.17463 4.98870 920 - -
2.68618 4.42225 2.77519 2.73123 3.46354 2.40513 3.72494 3.29354 2.67741 2.69355 4.24690 2.90347 2.73739 3.18146 2.89801 2.37887 2.77519 2.98518 4.58477 3.61503
0.01372 4.69125 5.41360 0.61958 0.77255 0.48576 0.95510
518 1.94645 3.88771 4.26264 3.70257 1.34501 3.50712 4.24015 2.79440 3.58499 2.54837 3.47414 3.87686 4.24440 3.80863 3.76974 3.12437 2.85443 2.58752 4.93340 3.48303 921 - -
2.68618 4.42225 2.77519 2.73123 3.46354 2.40513 3.72494 3.29354 2.67741 2.69355 4.24690 2.90347 2.73739 3.18146 2.89801 2.37887 2.77519 2.98518 4.58477 3.61503

0.01372 4.69125 5.41360 0.61958 0.77255 0.48576 0.95510

519 3.11473 5.15858 3.65033 3.21110 4.88308 3.32353 4.23903 4.32044 2.75398 3.85645 4.70344 3.60046 1.04561 3.41044 1.59994 3.19136 3.44348 3.90114 5.94602 4.73964 922 - -

2.68618 4.42225 2.77519 2.73123 3.46354 2.40513 3.72494 3.29354 2.67741 2.69355 4.24690 2.90347 2.73739 3.18146 2.89801 2.37887 2.77519 2.98518 4.58477 3.61503

0.01372 4.69125 5.41360 0.61958 0.77255 0.48576 0.95510

520 3.50916 5.33193 3.92891 3.47465 3.51012 4.14651 4.16411 4.01263 3.02121 3.45374 4.49776 3.84700 4.58500 0.92222 3.26743 3.58255 3.74700 3.79858 1.76396 3.46377 923 - -

2.68618 4.42225 2.77519 2.73123 3.46354 2.40513 3.72494 3.29354 2.67741 2.69355 4.24690 2.90347 2.73739 3.18146 2.89801 2.37887 2.77519 2.98518 4.58477 3.61503

0.01372 4.69125 5.41360 0.61958 0.77255 0.48576 0.95510

521 2.58474 4.17825 2.94681 2.26295 4.53141 3.54742 3.73540 3.76547 2.09532 3.50438 4.25847 2.74016 3.94023 2.84086 1.75933 2.54745 2.79911 3.58825 5.65721 4.26435 924 - -

2.68618 4.42225 2.77519 2.73123 3.46354 2.40513 3.72494 3.29354 2.67741 2.69355 4.24690 2.90347 2.73739 3.18146 2.89801 2.37887 2.77519 2.98518 4.58477 3.61503

0.01372 4.69125 5.41360 0.61958 0.77255 0.48576 0.95510

522 4.50756 5.58135 5.41703 5.28366 0.38868 5.08899 3.98630 3.97682 5.09141 3.14898 4.54069 4.76293 5.39082 4.89011 4.97936 4.54068 4.73671 3.98896 4.07545 2.18818 925 - -

2.68618 4.42225 2.77519 2.73123 3.46354 2.40513 3.72494 3.29354 2.67741 2.69355 4.24690 2.90347 2.73739 3.18146 2.89801 2.37887 2.77519 2.98518 4.58477 3.61503

0.01372 4.69125 5.41360 0.61958 0.77255 0.48576 0.95510

523 2.76509 5.02424 2.20341 2.05549 4.27828 3.37062 3.78281 3.70513 2.56957 3.09975 3.82316 3.09582 3.97253 2.71803 3.04041 2.78315 2.99668 2.27902 5.51474 2.86774 926 - -

2.68618 4.42225 2.77519 2.73123 3.46354 2.40513 3.72494 3.29354 2.67741 2.69355 4.24690 2.90347 2.73739 3.18146 2.89801 2.37887 2.77519 2.98518 4.58477 3.61503

0.13866 4.69125 2.11777 0.61958 0.77255 0.48576 0.95510

524 2.96440 5.04242 3.05787 2.98575 5.01917 1.07247 4.45580 4.60263 3.44424 4.18180 5.02391 1.38088 4.19467 3.67984 3.87263 3.04281 3.40909 4.02611 6.24264 4.92679 927 - -

2.68618 4.42225 2.77519 2.73123 3.46354 2.40513 3.72494 3.29354 2.67741 2.69355 4.24690 2.90347 2.73739 3.18146 2.89801 2.37887 2.77519 2.98518 4.58477 3.61503

0.01554 4.56813 5.29047 0.61958 0.77255 0.70078 0.68557

525 2.20717 4.39510 3.83657 3.27554 3.56594 3.78128 4.10014 1.50704 2.48989 2.60455 3.53357 3.62191 4.17733 3.48893 3.47826 2.79996 3.01287 2.28702 5.05128 3.83350 928 - -

2.68618 4.42225 2.77519 2.73123 3.46354 2.40513 3.72494 3.29354 2.67741 2.69355 4.24690 2.90347 2.73739 3.18146 2.89801 2.37887 2.77519 2.98518 4.58477 3.61503

0.01554 4.56813 5.29047 0.61958 0.77255 0.70078 0.68557

526 3.05857 5.61980 1.12358 2.27365 4.93200 3.22912 3.93482 4.43959 2.73169 3.92021 4.71498 2.35470 4.06065 3.07223 3.39922 2.60459 2.94083 4.00153 6.06994 4.59731 929 - -

2.68618 4.42225 2.77519 2.73123 3.46354 2.40513 3.72494 3.29354 2.67741 2.69355 4.24690 2.90347 2.73739 3.18146 2.89801 2.37887 2.77519 2.98518 4.58477 3.61503

0.31112 4.56813 1.35870 0.61958 0.77255 0.70078 0.68557

527 2.75293 4.95131 3.15503 2.57388 4.24118 3.54016 3.68268 3.64214 1.89176 2.68231 4.03216 3.06977 2.38802 2.82510 2.27765 2.77682 2.82842 3.31508 5.41088 4.11048 930 - -

2.68618 4.42225 2.77519 2.73123 3.46354 2.40513 3.72494 3.29354 2.67741 2.69355 4.24690 2.90347 2.73739 3.18146 2.89801 2.37887 2.77519 2.98518 4.58477 3.61503

0.02082 4.27784 5.00018 0.61958 0.77255 1.01708 0.44887

528 2.75243 5.02507 2.79462 2.46007 4.33888 3.40593 2.72492 3.88442 2.57538 3.43745 4.24945 1.82191 3.90568 2.93204 3.02413 1.91974 3.02709 3.49821 5.57206 4.17859 931 - -

2.68618 4.42225 2.77519 2.73123 3.46354 2.40513 3.72494 3.29354 2.67741 2.69355 4.24690 2.90347 2.73739 3.18146 2.89801 2.37887 2.77519 2.98518 4.58477 3.61503

0.02082 4.27784 5.00018 0.61958 0.77255 0.68785 0.69848

529 3.72053 5.82235 0.36152 2.87896 5.30435 3.75378 4.57979 5.04865 3.79003 4.56099 5.58616 3.45210 4.44567 3.84283 4.35703 3.64143 4.08129 4.63621 6.34279 5.12827 932 - -

2.68618 4.42225 2.77519 2.73123 3.46354 2.40513 3.72494 3.29354 2.67741 2.69355 4.24690 2.90347 2.73739 3.18146 2.89801 2.37887 2.77519 2.98518 4.58477 3.61503

0.01769 4.43923 5.16158 0.61958 0.77255 0.86509 0.54648

530 2.77550 4.73686 3.38918 2.80851 3.95477 3.62865 3.83612 2.74506 2.55874 2.73029 3.85259 3.26255 4.02735 3.04161 1.59637 2.18368 3.01078 3.03768 5.28126 4.01150 933 - -

2.68618 4.42225 2.77519 2.73123 3.46354 2.40513 3.72494 3.29354 2.67741 2.69355 4.24690 2.90347 2.73739 3.18146 2.89801 2.37887 2.77519 2.98518 4.58477 3.61503
0.01769 4.43923 5.16158 0.61958 0.77255 0.86509 0.54648
531 3.18534 4.92952 3.57005 3.31039 2.82463 3.87604 3.88357 3.64624 3.32833 3.19211 4.21713 2.83182 4.38299 3.65378 3.61832 3.00897 3.48261 3.40671 4.44067 0.86936 934 - -
2.68618 4.42225 2.77519 2.73123 3.46354 2.40513 3.72494 3.29354 2.67741 2.69355 4.24690 2.90347 2.73739 3.18146 2.89801 2.37887 2.77519 2.98518 4.58477 3.61503
0.01769 4.43923 5.16158 0.61958 0.77255 0.30559 1.33442
532 1.72360 5.14837 3.05275 1.99496 4.45534 3.24914 3.76560 3.90724 2.36082 3.44446 4.21702 3.05395 3.95796 2.78112 3.00691 2.51048 2.44589 3.37090 5.62485 4.24694 935 - -
2.68618 4.42225 2.77519 2.73123 3.46354 2.40513 3.72494 3.29354 2.67741 2.69355 4.24690 2.90347 2.73739 3.18146 2.89801 2.37887 2.77519 2.98518 4.58477 3.61503
0.01372 4.69125 5.41360 0.61958 0.77255 0.48576 0.95510
533 2.78566 2.53339 3.06602 2.61345 4.20230 3.40413 3.90197 3.41351 2.73898 3.23692 4.06595 1.46918 4.02992 3.07567 3.18932 2.56164 3.04495 3.28873 5.50114 4.18416 936 - -
2.68618 4.42225 2.77519 2.73123 3.46354 2.40513 3.72494 3.29354 2.67741 2.69355 4.24690 2.90347 2.73739 3.18146 2.89801 2.37887 2.77519 2.98518 4.58477 3.61503
0.01372 4.69125 5.41360 0.61958 0.77255 0.48576 0.95510
534 2.67828 4.33014 3.14398 2.11619 4.26520 3.58849 2.89626 3.68866 2.38148 2.20221 3.41115 2.92892 3.97824 2.92544 2.25451 2.79042 2.99894 3.35255 5.50304 4.15991 937 - -
2.68618 4.42225 2.77519 2.73123 3.46354 2.40513 3.72494 3.29354 2.67741 2.69355 4.24690 2.90347 2.73739 3.18146 2.89801 2.37887 2.77519 2.98518 4.58477 3.61503
0.01372 4.69125 5.41360 0.61958 0.77255 0.48576 0.95510
535 2.87142 4.46334 3.96647 3.39765 3.46797 3.90593 4.16493 2.70920 3.30672 1.68154 2.35172 1.79658 4.27148 3.58995 3.59084 3.17418 3.10265 2.66053 5.01902 3.51217 938 - -
2.68618 4.42225 2.77519 2.73123 3.46354 2.40513 3.72494 3.29354 2.67741 2.69355 4.24690 2.90347 2.73739 3.18146 2.89801 2.37887 2.77519 2.98518 4.58477 3.61503
0.01372 4.69125 5.41360 0.61958 0.77255 0.48576 0.95510
536 2.70449 4.53319 3.65233 3.08396 3.69396 3.75670 4.01759 2.59966 2.61660 2.74900 3.64807 3.49172 4.14396 3.33165 3.05449 3.00895 1.45286 2.22214 5.13210 3.89648 939 - -
2.68618 4.42225 2.77519 2.73123 3.46354 2.40513 3.72494 3.29354 2.67741 2.69355 4.24690 2.90347 2.73739 3.18146 2.89801 2.37887 2.77519 2.98518 4.58477 3.61503
0.01372 4.69125 5.41360 0.61958 0.77255 0.48576 0.95510
537 3.16957 4.85291 4.23238 3.64086 4.12863 4.02948 4.37718 2.99263 2.95704 3.00854 4.06243 3.93170 4.49027 3.65604 1.52835 3.42620 3.44719 1.07928 5.57688 4.36219 940 - -
2.68618 4.42225 2.77519 2.73123 3.46354 2.40513 3.72494 3.29354 2.67741 2.69355 4.24690 2.90347 2.73739 3.18146 2.89801 2.37887 2.77519 2.98518 4.58477 3.61503
0.01372 4.69125 5.41360 0.61958 0.77255 0.48576 0.95510
538 2.87930 4.27772 4.64456 4.04433 1.74148 3.48529 4.34958 2.42481 3.87188 1.50790 2.49077 4.12841 3.88974 4.02637 3.94553 3.33965 3.11020 2.47373 4.87372 3.69814 941 - -
2.68618 4.42225 2.77519 2.73123 3.46354 2.40513 3.72494 3.29354 2.67741 2.69355 4.24690 2.90347 2.73739 3.18146 2.89801 2.37887 2.77519 2.98518 4.58477 3.61503
0.01372 4.69125 5.41360 0.61958 0.77255 0.48576 0.95510
539 2.97092 4.70298 3.74149 3.20756 1.59191 3.89434 4.04006 2.86013 2.91842 2.73238 3.73396 3.60163 4.27689 1.66953 3.36803 3.16831 3.20230 2.94847 4.98541 3.58229 942 - -
2.68618 4.42225 2.77519 2.73123 3.46354 2.40513 3.72494 3.29354 2.67741 2.69355 4.24690 2.90347 2.73739 3.18146 2.89801 2.37887 2.77519 2.98518 4.58477 3.61503
0.01372 4.69125 5.41360 0.61958 0.77255 0.48576 0.95510
540 2.83045 5.32923 1.66373 1.94065 4.65818 3.54676 3.19900 3.71236 2.54546 3.44946 4.37847 2.47597 3.97064 2.88562 3.04602 2.48257 3.06650 3.71222 5.76742 4.35198 943 - -
2.68618 4.42225 2.77519 2.73123 3.46354 2.40513 3.72494 3.29354 2.67741 2.69355 4.24690 2.90347 2.73739 3.18146 2.89801 2.37887 2.77519 2.98518 4.58477 3.61503
0.01372 4.69125 5.41360 0.61958 0.77255 0.48576 0.95510
541 2.77727 4.19269 4.49745 3.89848 3.28316 3.19888 3.86756 1.55375 3.73193 2.37282 2.37824 3.99719 4.28455 3.90969 3.82583 3.21979 3.00961 1.99429 4.80940 2.76168 944 - -
2.68618 4.42225 2.77519 2.73123 3.46354 2.40513 3.72494 3.29354 2.67741 2.69355 4.24690 2.90347 2.73739 3.18146 2.89801 2.37887 2.77519 2.98518 4.58477 3.61503
0.01372 4.69125 5.41360 0.61958 0.77255 0.48576 0.95510

542 2.79075 4.94739 3.17198 2.50509 4.18817 3.60827 3.83819 3.59584 2.64420 2.78621 3.20186 1.61398 4.01318 2.99997 3.09387 2.07975 3.02750 3.28258 5.46980 4.14379 945 - -
2.68618 4.42225 2.77519 2.73123 3.46354 2.40513 3.72494 3.29354 2.67741 2.69355 4.24690 2.90347 2.73739 3.18146 2.89801 2.37887 2.77519 2.98518 4.58477 3.61503
0.01372 4.69125 5.41360 0.61958 0.77255 0.48576 0.95510
543 2.79236 4.29716 4.15360 3.57182 3.08509 2.99629 4.17278 2.72178 2.97638 1.57731 1.89165 3.81845 4.24621 3.69821 3.66346 3.15875 2.88444 2.53671 4.90350 3.70889 946 - -
2.68618 4.42225 2.77519 2.73123 3.46354 2.40513 3.72494 3.29354 2.67741 2.69355 4.24690 2.90347 2.73739 3.18146 2.89801 2.37887 2.77519 2.98518 4.58477 3.61503
0.01372 4.69125 5.41360 0.61958 0.77255 0.48576 0.95510
544 2.65799 3.74685 2.88256 2.53488 4.36909 3.56681 3.76166 3.57610 2.17029 3.11304 4.15060 2.56630 3.95826 2.88611 1.81929 2.57615 2.99360 3.08504 5.56592 4.20124 947 - -
2.68618 4.42225 2.77519 2.73123 3.46354 2.40513 3.72494 3.29354 2.67741 2.69355 4.24690 2.90347 2.73739 3.18146 2.89801 2.37887 2.77519 2.98518 4.58477 3.61503
0.01372 4.69125 5.41360 0.61958 0.77255 0.48576 0.95510
545 2.05903 4.48852 3.89876 3.37082 2.56457 1.64225 4.09814 3.03136 3.31703 2.74065 3.66222 3.68401 4.23350 3.58817 3.61461 3.09846 3.10256 2.81002 4.90681 2.37635 948 - -
2.68618 4.42225 2.77519 2.73123 3.46354 2.40513 3.72494 3.29354 2.67741 2.69355 4.24690 2.90347 2.73739 3.18146 2.89801 2.37887 2.77519 2.98518 4.58477 3.61503
0.01372 4.69125 5.41360 0.61958 0.77255 0.48576 0.95510
546 2.97187 4.38961 4.58390 3.99606 2.73762 4.11101 2.11867 2.31596 3.84842 1.30520 3.30493 4.14367 4.45051 4.02249 3.96996 3.41230 3.20139 2.22089 4.91756 3.69483 949 - -
2.68618 4.42225 2.77519 2.73123 3.46354 2.40513 3.72494 3.29354 2.67741 2.69355 4.24690 2.90347 2.73739 3.18146 2.89801 2.37887 2.77519 2.98518 4.58477 3.61503
0.01372 4.69125 5.41360 0.61958 0.77255 0.48576 0.95510
547 2.17542 4.99094 1.38808 2.80538 1.99921 3.65602 4.06204 3.62091 3.03710 3.28542 4.19070 3.30162 4.16457 3.30742 3.49511 3.02701 3.23611 3.33751 5.47999 4.07265 950 - -
2.68618 4.42225 2.77519 2.73123 3.46354 2.40513 3.72494 3.29354 2.67741 2.69355 4.24690 2.90347 2.73739 3.18146 2.89801 2.37887 2.77519 2.98518 4.58477 3.61503
0.01372 4.69125 5.41360 0.61958 0.77255 0.48576 0.95510
548 2.67596 4.94675 3.17554 2.24807 4.22512 1.48630 3.85487 3.62951 2.58561 3.24759 3.56838 3.16809 4.01430 3.01491 3.11183 2.83301 2.64110 3.15990 5.50039 4.17485 951 - -
2.68618 4.42225 2.77519 2.73123 3.46354 2.40513 3.72494 3.29354 2.67741 2.69355 4.24690 2.90347 2.73739 3.18146 2.89801 2.37887 2.77519 2.98518 4.58477 3.61503
0.01372 4.69125 5.41360 0.61958 0.77255 0.48576 0.95510
549 2.58261 4.39390 4.80574 4.23135 3.56595 4.25856 4.64187 1.37911 4.08316 1.76625 3.51187 3.60488 4.60333 4.27700 4.19382 3.57997 3.26904 1.55136 5.19336 4.00532 952 - -
2.68618 4.42225 2.77519 2.73123 3.46354 2.40513 3.72494 3.29354 2.67741 2.69355 4.24690 2.90347 2.73739 3.18146 2.89801 2.37887 2.77519 2.98518 4.58477 3.61503
0.01372 4.69125 5.41360 0.61958 0.77255 0.48576 0.95510
550 2.85969 5.23772 3.00709 1.77312 4.54123 3.23917 3.81377 3.99052 2.55642 3.52041 3.05693 3.08085 4.01094 1.54620 3.01504 2.84082 3.09404 3.60922 5.69618 4.32091 953 - -
2.68618 4.42225 2.77519 2.73123 3.46354 2.40513 3.72494 3.29354 2.67741 2.69355 4.24690 2.90347 2.73739 3.18146 2.89801 2.37887 2.77519 2.98518 4.58477 3.61503
0.26971 4.69125 1.48182 0.61958 0.77255 0.48576 0.95510
551 3.51828 5.20009 4.30253 4.28728 5.34898 0.22156 5.33274 5.12505 4.54185 4.68865 5.69448 4.46986 4.60310 4.82206 4.67987 3.71146 4.04057 4.54346 6.25711 5.48224 954 - -
2.68618 4.42225 2.77519 2.73123 3.46354 2.40513 3.72494 3.29354 2.67741 2.69355 4.24690 2.90347 2.73739 3.18146 2.89801 2.37887 2.77519 2.98518 4.58477 3.61503
0.01769 4.43923 5.16158 0.61958 0.77255 0.86509 0.54648
552 3.67816 5.29957 4.34802 4.29028 5.18154 3.95733 5.28456 4.92125 4.40559 4.46215 5.55824 4.52588 0.23060 4.77253 4.55260 3.87056 4.16715 4.49177 6.16841 5.32016 955 - -
2.68618 4.42225 2.77519 2.73123 3.46354 2.40513 3.72494 3.29354 2.67741 2.69355 4.24690 2.90347 2.73739 3.18146 2.89801 2.37887 2.77519 2.98518 4.58477 3.61503
0.01769 4.43923 5.16158 0.61958 0.77255 0.86509 0.54648
553 2.71400 4.14197 4.34731 3.75509 3.08491 3.85419 4.17440 1.93234 3.60894 2.16479 2.63571 3.56845 4.21698 3.55198 3.73681 3.15190 2.66948 1.62198 4.78016 3.59521 956 - -
2.68618 4.42225 2.77519 2.73123 3.46354 2.40513 3.72494 3.29354 2.67741 2.69355 4.24690 2.90347 2.73739 3.18146 2.89801 2.37887 2.77519 2.98518 4.58477 3.61503

0.01769 4.43923 5.16158 0.61958 0.77255 0.86509 0.54648

554 3.65935 5.19313 4.06956 3.75668 2.38923 4.31997 2.61555 3.84708 3.61291 3.23422 4.37853 3.44264 4.70849 3.90993 3.83411 3.70096 3.89166 3.67449 4.04123 0.73572 957 - -

2.68618 4.42225 2.77519 2.73123 3.46354 2.40513 3.72494 3.29354 2.67741 2.69355 4.24690 2.90347 2.73739 3.18146 2.89801 2.37887 2.77519 2.98518 4.58477 3.61503

0.01769 4.43923 5.16158 0.61958 0.77255 0.86509 0.54648

555 2.87203 4.53301 5.12244 4.60230 3.81456 4.66892 5.21980 1.49551 4.50238 2.39228 3.25621 4.76982 4.95940 4.73629 4.66465 4.04198 3.50821 0.86962 5.70604 4.50764 958 - -

2.68618 4.42225 2.77519 2.73123 3.46354 2.40513 3.72494 3.29354 2.67741 2.69355 4.24690 2.90347 2.73739 3.18146 2.89801 2.37887 2.77519 2.98518 4.58477 3.61503

0.01769 4.43923 5.16158 0.61958 0.77255 0.86509 0.54648

556 3.51828 5.20009 4.30253 4.28728 5.34898 0.22156 5.33274 5.12505 4.54185 4.68865 5.69448 4.46986 4.60310 4.82206 4.67987 3.71146 4.04057 4.54346 6.25711 5.48224 959 - -

2.68618 4.42225 2.77519 2.73123 3.46354 2.40513 3.72494 3.29354 2.67741 2.69355 4.24690 2.90347 2.73739 3.18146 2.89801 2.37887 2.77519 2.98518 4.58477 3.61503

0.01769 4.43923 5.16158 0.61958 0.77255 0.30559 1.33442

557 2.81537 4.95067 3.27163 2.84633 4.53597 3.54446 4.05671 3.96434 2.82939 3.55945 4.37383 3.30423 4.08124 1.84162 3.23777 2.20468 1.36210 3.55947 5.76950 4.45367 960 - -

2.68618 4.42225 2.77519 2.73123 3.46354 2.40513 3.72494 3.29354 2.67741 2.69355 4.24690 2.90347 2.73739 3.18146 2.89801 2.37887 2.77519 2.98518 4.58477 3.61503

0.01372 4.69125 5.41360 0.61958 0.77255 0.48576 0.95510

558 3.13875 5.58857 2.76362 2.08067 4.92961 1.18578 3.99738 4.40018 2.62976 3.89437 4.70570 3.11107 4.15779 2.20566 3.23424 3.07386 3.39364 3.99382 6.03940 4.63167 961 - -

2.68618 4.42225 2.77519 2.73123 3.46354 2.40513 3.72494 3.29354 2.67741 2.69355 4.24690 2.90347 2.73739 3.18146 2.89801 2.37887 2.77519 2.98518 4.58477 3.61503

0.01372 4.69125 5.41360 0.61958 0.77255 0.48576 0.95510

559 2.68362 1.15878 4.56964 4.12471 4.00076 3.55428 4.69334 3.14290 3.99260 3.05716 3.98333 4.04366 4.23162 4.20447 4.14228 2.22985 2.61644 1.91527 5.50533 4.33358 962 - -

2.68618 4.42225 2.77519 2.73123 3.46354 2.40513 3.72494 3.29354 2.67741 2.69355 4.24690 2.90347 2.73739 3.18146 2.89801 2.37887 2.77519 2.98518 4.58477 3.61503

0.01372 4.69125 5.41360 0.61958 0.77255 0.48576 0.95510

560 2.76959 3.56977 4.44119 3.84428 2.16918 2.58139 4.22200 2.41924 3.68909 2.16222 2.86138 2.87378 4.27092 3.87407 3.79993 3.20198 3.00190 1.92062 4.81438 3.63017 963 - -

2.68618 4.42225 2.77519 2.73123 3.46354 2.40513 3.72494 3.29354 2.67741 2.69355 4.24690 2.90347 2.73739 3.18146 2.89801 2.37887 2.77519 2.98518 4.58477 3.61503

0.01372 4.69125 5.41360 0.61958 0.77255 0.48576 0.95510

561 2.86233 4.66771 3.59228 3.10300 1.60179 2.85775 2.43700 3.27842 3.08999 2.95198 3.85255 3.50359 4.18570 3.39983 3.45652 2.10642 3.12753 3.02837 5.02111 3.60076 964 - -

2.68618 4.42225 2.77519 2.73123 3.46354 2.40513 3.72494 3.29354 2.67741 2.69355 4.24690 2.90347 2.73739 3.18146 2.89801 2.37887 2.77519 2.98518 4.58477 3.61503

0.01372 4.69125 5.41360 0.61958 0.77255 0.48576 0.95510

562 2.78428 5.06346 3.14120 2.58613 4.35325 3.58682 3.79133 3.78278 2.46898 3.35205 4.14689 2.38057 3.98483 2.92363 1.96587 2.34515 2.23754 2.71985 5.55924 4.21089 965 - -

2.68618 4.42225 2.77519 2.73123 3.46354 2.40513 3.72494 3.29354 2.67741 2.69355 4.24690 2.90347 2.73739 3.18146 2.89801 2.37887 2.77519 2.98518 4.58477 3.61503

0.01372 4.69125 5.41360 0.61958 0.77255 0.48576 0.95510

563 2.91118 4.68588 3.73133 3.14568 2.73166 3.83244 4.04398 2.82518 2.88244 2.78681 3.75379 3.54840 4.22091 3.33979 1.21497 3.10170 2.74065 2.91592 5.19589 3.91625 966 - -

2.68618 4.42225 2.77519 2.73123 3.46354 2.40513 3.72494 3.29354 2.67741 2.69355 4.24690 2.90347 2.73739 3.18146 2.89801 2.37887 2.77519 2.98518 4.58477 3.61503

0.01372 4.69125 5.41360 0.61958 0.77255 0.48576 0.95510

564 2.76051 4.92817 2.96335 2.62373 3.49482 3.59947 2.95008 3.10456 2.61464 2.61946 4.00157 3.13915 3.59713 2.37288 2.22841 2.80429 2.51903 3.25483 5.43707 3.49103 967 - -

2.68618 4.42225 2.77519 2.73123 3.46354 2.40513 3.72494 3.29354 2.67741 2.69355 4.24690 2.90347 2.73739 3.18146 2.89801 2.37887 2.77519 2.98518 4.58477 3.61503

0.01372 4.69125 5.41360 0.61958 0.77255 0.48576 0.95510

565 1.08200 4.60144 3.72305 3.28651 4.24148 2.97898 4.32746 3.57485 3.29781 3.30964 4.17195 2.51097 4.12014 3.59848 3.65867 2.87991 3.08934 2.07175 5.63202 4.39387 968 - -

2.68618 4.42225 2.77519 2.73123 3.46354 2.40513 3.72494 3.29354 2.67741 2.69355 4.24690 2.90347 2.73739 3.18146 2.89801 2.37887 2.77519 2.98518 4.58477 3.61503
0.01372 4.69125 5.41360 0.61958 0.77255 0.48576 0.95510
566 3.84617 5.08916 5.73053 5.13822 1.52798 5.15730 5.37867 2.39101 4.98782 0.93888 2.12005 5.29394 5.23239 4.87151 4.93515 4.52415 4.04725 2.87094 5.44997 4.41465 969 - -
2.68618 4.42225 2.77519 2.73123 3.46354 2.40513 3.72494 3.29354 2.67741 2.69355 4.24690 2.90347 2.73739 3.18146 2.89801 2.37887 2.77519 2.98518 4.58477 3.61503
0.01372 4.69125 5.41360 0.61958 0.77255 0.48576 0.95510
567 4.09397 3.60906 5.32210 5.04344 1.32563 4.88067 3.93060 3.79063 4.84522 3.18610 4.40472 4.61174 5.18813 4.70739 4.77388 4.25097 4.31811 3.68341 4.04818 0.76594 970 - -
2.68618 4.42225 2.77519 2.73123 3.46354 2.40513 3.72494 3.29354 2.67741 2.69355 4.24690 2.90347 2.73739 3.18146 2.89801 2.37887 2.77519 2.98518 4.58477 3.61503
0.01372 4.69125 5.41360 0.61958 0.77255 0.48576 0.95510
568 2.90290 4.81990 3.98904 3.97782 5.25292 0.37604 5.14822 4.87072 4.28968 4.53452 5.37259 4.04101 4.34195 4.50731 4.50500 2.83220 3.45920 4.11446 6.50781 5.37793 971 - -
2.68618 4.42225 2.77519 2.73123 3.46354 2.40513 3.72494 3.29354 2.67741 2.69355 4.24690 2.90347 2.73739 3.18146 2.89801 2.37887 2.77519 2.98518 4.58477 3.61503
0.01372 4.69125 5.41360 0.61958 0.77255 0.48576 0.95510
569 2.73384 4.32112 4.31518 3.73848 1.47001 3.94768 4.15523 2.76059 3.60241 1.98982 3.42264 3.91957 3.46731 3.81791 3.32771 3.23875 3.08829 2.57549 4.74353 2.50946 972 - -
2.68618 4.42225 2.77519 2.73123 3.46354 2.40513 3.72494 3.29354 2.67741 2.69355 4.24690 2.90347 2.73739 3.18146 2.89801 2.37887 2.77519 2.98518 4.58477 3.61503
0.01372 4.69125 5.41360 0.61958 0.77255 0.48576 0.95510
570 2.92461 5.45839 2.12325 2.19471 4.78730 3.55628 3.84784 4.28214 2.48681 3.75813 4.51633 1.75790 3.14927 2.96127 3.16886 2.00529 3.16818 3.84286 5.89383 4.45800 973 - -
2.68618 4.42225 2.77519 2.73123 3.46354 2.40513 3.72494 3.29354 2.67741 2.69355 4.24690 2.90347 2.73739 3.18146 2.89801 2.37887 2.77519 2.98518 4.58477 3.61503
0.01372 4.69125 5.41360 0.61958 0.77255 0.48576 0.95510
571 2.47709 4.73978 3.35787 2.80855 3.99427 2.15164 3.91752 3.23850 2.79188 2.93824 3.89488 3.27904 2.03783 3.12681 3.21835 2.42980 2.87310 3.09784 5.34723 3.28625 974 - -
2.68618 4.42225 2.77519 2.73123 3.46354 2.40513 3.72494 3.29354 2.67741 2.69355 4.24690 2.90347 2.73739 3.18146 2.89801 2.37887 2.77519 2.98518 4.58477 3.61503
0.10301 4.69125 2.42245 0.61958 0.77255 0.48576 0.95510
572 2.76933 4.96713 2.27421 2.64796 4.40090 3.50944 3.91365 3.80215 2.74173 3.41846 4.23120 3.15083 1.89634 3.06714 3.20982 2.80964 1.85936 3.04459 5.65284 4.30813 975 - -
2.68618 4.42225 2.77519 2.73123 3.46354 2.40513 3.72494 3.29354 2.67741 2.69355 4.24690 2.90347 2.73739 3.18146 2.89801 2.37887 2.77519 2.98518 4.58477 3.61503
0.29860 4.60324 1.39381 0.61958 0.77255 0.64657 0.74200
573 2.65570 5.07076 2.25602 2.32513 4.38006 3.44007 3.63071 3.57650 2.12817 3.05442 4.12203 2.75811 3.83383 2.74108 2.37048 2.53836 2.88651 3.17506 5.52411 4.13890 976 - -
2.68618 4.42225 2.77519 2.73123 3.46354 2.40513 3.72494 3.29354 2.67741 2.69355 4.24690 2.90347 2.73739 3.18146 2.89801 2.37887 2.77519 2.98518 4.58477 3.61503
0.01987 4.32450 5.04685 0.61958 0.77255 0.72822 0.65926
574 2.24568 4.60575 3.28783 2.72635 3.53929 3.08032 3.80541 3.16162 2.28697 2.30752 3.39119 3.04725 3.54192 3.03446 3.12205 2.64652 2.59684 2.70837 5.15799 3.88233 977 - -
2.68618 4.42225 2.77519 2.73123 3.46354 2.40513 3.72494 3.29354 2.67741 2.69355 4.24690 2.90347 2.73739 3.18146 2.89801 2.37887 2.77519 2.98518 4.58477 3.61503
0.01769 4.43923 5.16158 0.61958 0.77255 0.86509 0.54648
575 2.68142 5.07508 2.80617 1.83459 3.75048 3.32170 3.66626 3.82924 2.14086 2.88479 4.12845 2.79109 3.37969 2.78005 2.90586 2.66746 2.66930 3.26406 5.53618 4.15454 978 - -
2.68618 4.42225 2.77519 2.73123 3.46354 2.40513 3.72494 3.29354 2.67741 2.69355 4.24690 2.90347 2.73739 3.18146 2.89801 2.37887 2.77519 2.98518 4.58477 3.61503
0.01769 4.43923 5.16158 0.61958 0.77255 0.86509 0.54648
576 2.69293 5.13147 2.39374 1.90809 4.44827 3.46830 3.66234 3.18180 1.99683 3.42325 4.18080 2.94731 3.86512 2.69169 2.76794 2.66823 2.92423 3.05725 5.57982 4.18783 979 - -
2.68618 4.42225 2.77519 2.73123 3.46354 2.40513 3.72494 3.29354 2.67741 2.69355 4.24690 2.90347 2.73739 3.18146 2.89801 2.37887 2.77519 2.98518 4.58477 3.61503
0.01769 4.43923 5.16158 0.61958 0.77255 0.86509 0.54648

577 2.67912 5.03001 2.35202 2.32396 4.31436 3.33807 2.87463 3.49408 2.43859 2.70315 4.08724 2.78762 3.48801 2.79514 2.66710 2.67504 2.83005 3.10762 5.50075 3.66422 980 - -

2.68618 4.42225 2.77519 2.73123 3.46354 2.40513 3.72494 3.29354 2.67741 2.69355 4.24690 2.90347 2.73739 3.18146 2.89801 2.37887 2.77519 2.98518 4.58477 3.61503

0.03877 4.43923 3.64097 0.61958 0.77255 0.86509 0.54648

578 2.69216 5.04356 2.97580 2.20745 4.33135 2.56505 2.96850 3.77628 2.21937 2.72274 4.10341 2.97455 2.84573 2.80019 2.90842 2.54895 2.92374 3.40388 5.51211 4.14230 981 - -

2.68618 4.42225 2.77519 2.73123 3.46354 2.40513 3.72494 3.29354 2.67741 2.69355 4.24690 2.90347 2.73739 3.18146 2.89801 2.37887 2.77519 2.98518 4.58477 3.61503

0.01807 4.41853 5.14087 0.61958 0.77255 0.88741 0.53056

579 2.71595 5.17596 2.88613 1.82060 4.50524 2.67240 3.67390 3.97705 2.24500 3.47565 4.23171 2.83267 3.23216 2.78167 2.73567 2.20086 2.95018 3.56040 5.62325 4.22379 982 - -

2.68618 4.42225 2.77519 2.73123 3.46354 2.40513 3.72494 3.29354 2.67741 2.69355 4.24690 2.90347 2.73739 3.18146 2.89801 2.37887 2.77519 2.98518 4.58477 3.61503

0.01807 4.41853 5.14087 0.61958 0.77255 0.83518 0.56880

580 2.67401 4.47518 3.43490 2.51891 3.62515 3.28000 3.86282 2.53526 2.53336 2.20554 3.13715 3.30715 3.10529 2.98244 3.08704 2.85645 2.90558 2.60333 3.77496 3.79616 983 - -

2.68756 4.40836 2.77562 2.73036 3.46392 2.40620 3.72702 3.29302 2.67311 2.69412 4.24570 2.90360 2.73867 3.18342 2.89765 2.37821 2.77649 2.98656 4.58403 3.61521

0.72120 0.67708 5.16158 3.34065 0.03606 0.86509 0.54648

581 2.70598 5.19007 2.46170 2.11419 4.52189 3.17410 3.66523 4.00038 2.41387 3.48989 4.23759 2.84441 3.86476 2.62081 2.42930 2.07924 2.53436 3.57426 5.63053 4.22392 1064 - -

2.68628 4.42235 2.77511 2.73094 3.46364 2.40522 3.72504 3.29364 2.67750 2.69365 4.24699 2.90335 2.73737 3.18146 2.89810 2.37863 2.77517 2.98528 4.58487 3.61513

0.86974 0.84463 1.88900 0.11049 2.25753 0.86509 0.54648

582 2.49164 4.59306 2.79947 2.43569 3.77453 3.31542 3.74768 3.03041 2.63219 2.45159 3.68280 3.13042 3.90892 2.96523 3.05775 2.45492 2.86258 2.26200 5.14036 3.53811 1066 - -

2.68618 4.42225 2.77519 2.73123 3.46354 2.40513 3.72494 3.29354 2.67741 2.69355 4.24690 2.90347 2.73739 3.18146 2.89801 2.37887 2.77519 2.98518 4.58477 3.61503

0.02074 4.28201 5.00435 0.61958 0.77255 0.92110 0.50763

583 1.99306 5.43071 1.86437 1.76251 4.74533 3.39848 3.79276 4.24249 2.67657 3.73326 4.52315 2.32013 3.92747 2.92682 3.22102 2.82304 3.15835 3.81253 5.88684 4.42952 1067 - -

2.68618 4.42225 2.77519 2.73123 3.46354 2.40513 3.72494 3.29354 2.67741 2.69355 4.24690 2.90347 2.73739 3.18146 2.89801 2.37887 2.77519 2.98518 4.58477 3.61503

0.01987 4.32450 5.04685 0.61958 0.77255 0.97787 0.47179

584 2.64995 5.08204 2.41906 2.25876 3.82327 3.43416 3.62554 3.86007 2.07974 3.37361 3.80648 2.82406 3.82773 2.66187 2.64977 2.40593 2.48643 3.45586 5.53354 4.14268 1068 - -

2.68618 4.42225 2.77519 2.73123 3.46354 2.40513 3.72494 3.29354 2.67741 2.69355 4.24690 2.90347 2.73739 3.18146 2.89801 2.37887 2.77519 2.98518 4.58477 3.61503

0.01987 4.32450 5.04685 0.61958 0.77255 0.97787 0.47179

585 2.61600 5.20081 2.14843 2.14756 4.52781 3.42484 3.67252 4.00408 2.44134 3.50023 4.26128 2.79138 3.85954 2.78468 2.17616 2.16511 2.96544 3.58479 5.64774 4.23888 1069 - -

2.68618 4.42225 2.77519 2.73123 3.46354 2.40513 3.72494 3.29354 2.67741 2.69355 4.24690 2.90347 2.73739 3.18146 2.89801 2.37887 2.77519 2.98518 4.58477 3.61503

0.01987 4.32450 5.04685 0.61958 0.77255 0.97787 0.47179

586 2.64830 5.03715 2.75013 1.99019 4.33479 3.04707 3.42288 3.55111 2.28393 3.02284 3.72855 2.92931 3.08521 2.74888 2.87417 2.39245 2.79715 3.40209 5.49932 4.11895 1070 - -

2.68618 4.42225 2.77519 2.73123 3.46354 2.40513 3.72494 3.29354 2.67741 2.69355 4.24690 2.90347 2.73739 3.18146 2.89801 2.37887 2.77519 2.98518 4.58477 3.61503

0.01987 4.32450 5.04685 0.61958 0.77255 0.97787 0.47179

587 2.65199 4.71711 2.51143 2.22825 2.96113 3.50998 3.72425 3.31470 2.57000 2.27572 3.79737 3.07938 3.90016 2.91195 2.79408 2.72668 2.88363 3.02802 5.23118 3.47488 1071 - -

2.68618 4.42225 2.77519 2.73123 3.46354 2.40513 3.72494 3.29354 2.67741 2.69355 4.24690 2.90347 2.73739 3.18146 2.89801 2.37887 2.77519 2.98518 4.58477 3.61503

0.05765 4.32450 3.15179 0.61958 0.77255 0.97787 0.47179

588 2.53728 4.25904 4.55361 3.96383 2.84329 4.04557 4.37106 2.01847 3.81866 1.48228 2.26467 4.10623 4.36967 3.97742 3.92543 3.35432 2.93483 2.15227 4.89274 3.74200 1072 - -

2.68618 4.42225 2.77519 2.73123 3.46354 2.40513 3.72494 3.29354 2.67741 2.69355 4.24690 2.90347 2.73739 3.18146 2.89801 2.37887 2.77519 2.98518 4.58477 3.61503

0.02062 4.28748 5.00982 0.61958 0.77255 1.00925 0.45333

589 2.26257 4.99284 2.57678 2.23203 4.28801 3.43107 3.64847 3.73252 2.42115 3.28434 3.80206 2.74822 3.83609 2.77193 2.90217 2.00147 2.79484 3.19000 5.47789 4.10620 1073 - -

2.68618 4.42225 2.77519 2.73123 3.46354 2.40513 3.72494 3.29354 2.67741 2.69355 4.24690 2.90347 2.73739 3.18146 2.89801 2.37887 2.77519 2.98518 4.58477 3.61503

0.02062 4.28748 5.00982 0.61958 0.77255 1.00925 0.45333

590 2.65267 4.92502 2.99406 2.00252 4.18839 3.45914 3.65640 3.24601 2.35286 2.95767 3.99069 2.97178 3.85237 2.12044 2.88177 2.45415 2.88329 3.03205 5.40885 4.05965 1074 - -

2.68618 4.42225 2.77519 2.73123 3.46354 2.40513 3.72494 3.29354 2.67741 2.69355 4.24690 2.90347 2.73739 3.18146 2.89801 2.37887 2.77519 2.98518 4.58477 3.61503

0.02062 4.28748 5.00982 0.61958 0.77255 0.92788 0.50317

591 2.64932 4.92794 2.45785 2.32900 4.18936 3.14880 3.66127 3.61972 2.27000 2.50368 3.31669 2.97257 3.85320 2.79542 2.80422 2.52332 2.61286 3.27281 5.41428 4.06093 1075 - -

2.68618 4.42225 2.77519 2.73123 3.46354 2.40513 3.72494 3.29354 2.67741 2.69355 4.24690 2.90347 2.73739 3.18146 2.89801 2.37887 2.77519 2.98518 4.58477 3.61503

0.01987 4.32450 5.04685 0.61958 0.77255 0.97787 0.47179

592 2.67906 4.94961 3.00769 2.46640 4.22138 3.17781 3.67296 3.64865 2.20639 2.52291 4.02057 2.61363 3.87243 2.64861 2.75642 2.01634 2.90973 3.30247 5.43180 4.08645 1076 - -

2.68618 4.42225 2.77519 2.73123 3.46354 2.40513 3.72494 3.29354 2.67741 2.69355 4.24690 2.90347 2.73739 3.18146 2.89801 2.37887 2.77519 2.98518 4.58477 3.61503

0.01987 4.32450 5.04685 0.61958 0.77255 0.97787 0.47179

593 3.43242 4.72257 5.19557 4.62177 2.43645 4.72072 4.99724 2.08511 4.48927 0.84773 2.99746 4.80180 4.88893 4.51164 4.53566 4.07083 3.65000 2.08320 5.22763 4.13103 1077 - -

2.68618 4.42225 2.77519 2.73123 3.46354 2.40513 3.72494 3.29354 2.67741 2.69355 4.24690 2.90347 2.73739 3.18146 2.89801 2.37887 2.77519 2.98518 4.58477 3.61503

0.01987 4.32450 5.04685 0.61958 0.77255 0.97787 0.47179

594 2.49888 4.91982 2.99347 1.88196 4.20397 3.45723 3.68306 3.62879 2.46122 3.21116 4.01126 2.98718 2.65611 2.82096 2.71832 2.59393 2.79934 2.76132 5.43091 4.08206 1078 - -

2.68618 4.42225 2.77519 2.73123 3.46354 2.40513 3.72494 3.29354 2.67741 2.69355 4.24690 2.90347 2.73739 3.18146 2.89801 2.37887 2.77519 2.98518 4.58477 3.61503

0.01987 4.32450 5.04685 0.61958 0.77255 0.97787 0.47179

595 3.21111 5.36567 3.64165 2.94005 4.87351 3.82660 3.74372 4.20534 1.03320 3.61229 4.46941 3.34072 4.18489 2.86977 1.68851 2.97194 3.37072 3.87008 5.62891 4.46891 1079 - -

2.68618 4.42225 2.77519 2.73123 3.46354 2.40513 3.72494 3.29354 2.67741 2.69355 4.24690 2.90347 2.73739 3.18146 2.89801 2.37887 2.77519 2.98518 4.58477 3.61503

0.01987 4.32450 5.04685 0.61958 0.77255 0.97787 0.47179

596 2.86463 5.22324 3.15636 2.39840 4.60470 3.34857 3.67043 4.02322 1.61473 3.48849 4.27933 3.06927 3.96746 2.56025 1.81821 2.83793 2.73397 3.63880 5.58689 4.28292 1080 - -

2.68618 4.42225 2.77519 2.73123 3.46354 2.40513 3.72494 3.29354 2.67741 2.69355 4.24690 2.90347 2.73739 3.18146 2.89801 2.37887 2.77519 2.98518 4.58477 3.61503

0.01987 4.32450 5.04685 0.61958 0.77255 0.97787 0.47179

597 4.04564 5.18350 5.02049 4.79332 0.68530 4.72231 3.66090 3.63725 4.61367 2.89388 4.21395 4.37840 5.01541 4.47671 4.56128 4.11761 4.26376 3.59408 3.76842 1.63401 1081 - -

2.68618 4.42225 2.77519 2.73123 3.46354 2.40513 3.72494 3.29354 2.67741 2.69355 4.24690 2.90347 2.73739 3.18146 2.89801 2.37887 2.77519 2.98518 4.58477 3.61503

0.01987 4.32450 5.04685 0.61958 0.77255 0.97787 0.47179

598 3.32554 5.03153 4.08910 4.05594 5.14353 0.28121 5.12322 4.87799 4.29689 4.46405 5.46026 4.25584 4.43383 4.58604 4.46002 3.51387 3.84064 4.31837 6.09466 5.26883 1082 - -

2.68618 4.42225 2.77519 2.73123 3.46354 2.40513 3.72494 3.29354 2.67741 2.69355 4.24690 2.90347 2.73739 3.18146 2.89801 2.37887 2.77519 2.98518 4.58477 3.61503

0.01987 4.32450 5.04685 0.61958 0.77255 0.97787 0.47179

599 2.67860 4.94309 2.99799 2.47019 4.21706 3.46916 3.68335 3.65007 2.44224 3.03821 3.55224 2.03207 3.87415 2.21376 2.89233 2.29816 2.91381 3.30322 5.43618 4.08717 1083 - -

2.68618 4.42225 2.77519 2.73123 3.46354 2.40513 3.72494 3.29354 2.67741 2.69355 4.24690 2.90347 2.73739 3.18146 2.89801 2.37887 2.77519 2.98518 4.58477 3.61503

0.01987 4.32450 5.04685 0.61958 0.77255 0.97787 0.47179

600 2.60485 4.51870 3.67826 3.54121 4.57471 3.24402 4.62329 4.17314 3.64840 3.86490 4.76590 3.67024 4.02459 3.96289 3.90070 0.57108 3.12295 3.60168 5.91245 4.66905 1084 - -

2.68618 4.42225 2.77519 2.73123 3.46354 2.40513 3.72494 3.29354 2.67741 2.69355 4.24690 2.90347 2.73739 3.18146 2.89801 2.37887 2.77519 2.98518 4.58477 3.61503
0.01987 4.32450 5.04685 0.61958 0.77255 0.97787 0.47179
601 2.56208 4.98138 2.96290 2.44792 4.33469 3.43357 3.68756 3.77830 2.01540 3.32795 4.11100 2.58972 2.67066 2.81355 2.90666 2.41112 2.33122 3.39542 5.51606 4.15320 1085 - -
2.68618 4.42225 2.77519 2.73123 3.46354 2.40513 3.72494 3.29354 2.67741 2.69355 4.24690 2.90347 2.73739 3.18146 2.89801 2.37887 2.77519 2.98518 4.58477 3.61503
0.01987 4.32450 5.04685 0.61958 0.77255 0.97787 0.47179
602 2.65612 4.29684 3.66886 2.31746 3.10612 3.67315 3.94127 2.60567 3.03856 2.34495 2.83865 3.46686 4.04838 3.32562 3.35674 2.63561 2.88841 1.91799 4.89891 3.67808 1086 - -
2.68618 4.42225 2.77519 2.73123 3.46354 2.40513 3.72494 3.29354 2.67741 2.69355 4.24690 2.90347 2.73739 3.18146 2.89801 2.37887 2.77519 2.98518 4.58477 3.61503
0.01987 4.32450 5.04685 0.61958 0.77255 0.97787 0.47179
603 3.48055 4.77949 5.16320 4.59676 1.54614 4.70802 4.82663 2.43196 4.44959 1.08944 2.34961 4.75351 4.87250 4.45470 4.48631 4.05419 3.69213 2.61559 5.02929 3.79693 1087 - -
2.68618 4.42225 2.77519 2.73123 3.46354 2.40513 3.72494 3.29354 2.67741 2.69355 4.24690 2.90347 2.73739 3.18146 2.89801 2.37887 2.77519 2.98518 4.58477 3.61503
0.01987 4.32450 5.04685 0.61958 0.77255 0.72822 0.65926
604 2.83622 4.45989 4.97633 4.43959 3.74879 4.50770 5.00658 1.31864 4.33029 2.43290 3.28770 4.59749 4.82198 4.55930 4.48625 3.86196 3.00436 1.08891 5.53894 4.33747 1088 - -
2.68618 4.42225 2.77519 2.73123 3.46354 2.40513 3.72494 3.29354 2.67741 2.69355 4.24690 2.90347 2.73739 3.18146 2.89801 2.37887 2.77519 2.98518 4.58477 3.61503
0.05553 4.43923 3.16500 0.61958 0.77255 0.86509 0.54648
605 1.90001 5.11877 2.58168 2.21908 4.43073 3.45621 3.66856 3.89279 2.08442 3.41166 4.17583 2.73902 3.86355 2.78087 2.91442 2.67284 2.64587 3.29250 5.57661 4.18544 1089 - -
2.68618 4.42225 2.77519 2.73123 3.46354 2.40513 3.72494 3.29354 2.67741 2.69355 4.24690 2.90347 2.73739 3.18146 2.89801 2.37887 2.77519 2.98518 4.58477 3.61503
0.01837 4.40207 5.12442 0.61958 0.77255 0.90448 0.51878
606 2.39311 4.81321 2.41319 2.67677 4.54400 3.33137 4.00717 3.97852 2.88991 3.58147 4.38651 3.13954 3.93241 3.17595 3.35544 1.16037 2.72014 3.52056 5.79096 4.45110 1090 - -
2.68618 4.42225 2.77519 2.73123 3.46354 2.40513 3.72494 3.29354 2.67741 2.69355 4.24690 2.90347 2.73739 3.18146 2.89801 2.37887 2.77519 2.98518 4.58477 3.61503
0.01837 4.40207 5.12442 0.61958 0.77255 0.90448 0.51878
607 2.48566 4.31643 3.87173 3.30566 3.48433 3.78261 4.09646 1.99115 2.69179 2.49916 3.44794 3.63781 4.16540 3.50956 3.50680 3.05884 2.20000 1.78115 4.99363 3.77903 1091 - -
2.68618 4.42225 2.77519 2.73123 3.46354 2.40513 3.72494 3.29354 2.67741 2.69355 4.24690 2.90347 2.73739 3.18146 2.89801 2.37887 2.77519 2.98518 4.58477 3.61503
0.01837 4.40207 5.12442 0.61958 0.77255 0.90448 0.51878
608 2.66245 4.34675 3.60368 3.03577 3.11131 3.46972 3.91816 2.83563 2.75458 2.14927 3.45457 3.42210 2.48740 3.27605 3.17192 2.74554 2.89543 2.04545 4.93590 3.70844 1092 - -
2.68618 4.42225 2.77519 2.73123 3.46354 2.40513 3.72494 3.29354 2.67741 2.69355 4.24690 2.90347 2.73739 3.18146 2.89801 2.37887 2.77519 2.98518 4.58477 3.61503
0.01837 4.40207 5.12442 0.61958 0.77255 0.90448 0.51878
609 2.70529 4.35609 2.71942 2.87014 3.48768 3.72058 3.98927 2.25609 3.06499 2.37484 2.38287 3.49854 4.09479 3.35656 3.39392 2.97624 2.93795 1.98065 4.97442 3.75071 1093 - -
2.68618 4.42225 2.77519 2.73123 3.46354 2.40513 3.72494 3.29354 2.67741 2.69355 4.24690 2.90347 2.73739 3.18146 2.89801 2.37887 2.77519 2.98518 4.58477 3.61503
0.09744 4.40207 2.51842 0.61958 0.77255 0.90448 0.51878
610 1.56702 4.95916 2.87396 1.86284 4.27182 3.45742 3.78626 3.66504 2.58689 3.09952 4.11503 3.02363 3.92837 2.82190 3.02882 2.78006 3.01519 3.33196 5.52485 4.18246 1094 - -
2.68638 4.42314 2.77553 2.73125 3.46366 2.40439 3.72452 3.29411 2.67773 2.69349 4.24726 2.90325 2.73689 3.18052 2.89716 2.37936 2.77595 2.98545 4.58566 3.61492
0.97258 0.80230 1.75102 2.15561 0.12311 0.41837 1.07329
611 1.17149 4.46086 3.72418 3.41127 4.66511 1.73496 4.50335 4.09772 3.48492 3.76210 4.56732 3.57454 2.21051 3.75683 3.81965 2.66867 2.98663 3.51011 5.98365 4.77403 1114 - -
2.68618 4.42225 2.77519 2.73123 3.46354 2.40513 3.72494 3.29354 2.67741 2.69355 4.24690 2.90347 2.73739 3.18146 2.89801 2.37887 2.77519 2.98518 4.58477 3.61503
0.24040 4.46799 1.59843 0.61958 0.77255 0.83243 0.57092

612 3.20286 4.92360 3.95209 3.90690 5.00951 0.32940 4.98682 4.71788 4.13832 4.31749 5.30814 4.11833 4.32485 4.43337 4.31682 3.38808 3.71278 4.17300 5.98797 5.12987 1115 - -
2.68618 4.42225 2.77519 2.73123 3.46354 2.40513 3.72494 3.29354 2.67741 2.69355 4.24690 2.90347 2.73739 3.18146 2.89801 2.37887 2.77519 2.98518 4.58477 3.61503
0.02144 4.24903 4.97138 0.61958 0.77255 1.03968 0.43629
613 2.12105 4.26018 3.94541 3.41729 3.56570 3.63024 4.20109 2.27103 3.33889 2.54255 3.51690 3.67513 4.11647 3.61722 3.61235 2.34981 2.94277 1.48368 5.09771 3.88681 1116 - -
2.68618 4.42225 2.77519 2.73123 3.46354 2.40513 3.72494 3.29354 2.67741 2.69355 4.24690 2.90347 2.73739 3.18146 2.89801 2.37887 2.77519 2.98518 4.58477 3.61503
0.02144 4.24903 4.97138 0.61958 0.77255 1.03968 0.43629
614 3.31490 4.71075 4.58228 4.09408 2.69452 4.27073 4.10616 2.78877 3.80528 1.35141 3.39376 4.19635 4.60407 4.06092 3.93403 3.66012 3.55068 2.80722 1.51757 2.85378 1117 - -
2.68618 4.42225 2.77519 2.73123 3.46354 2.40513 3.72494 3.29354 2.67741 2.69355 4.24690 2.90347 2.73739 3.18146 2.89801 2.37887 2.77519 2.98518 4.58477 3.61503
0.02144 4.24903 4.97138 0.61958 0.77255 1.03968 0.43629
615 2.41694 4.74257 3.32874 2.75519 4.09110 3.52391 3.78516 3.41476 2.42311 2.79116 3.94084 3.19686 3.95955 2.96551 1.55736 2.81071 2.48418 3.12059 5.34798 4.08639 1118 - -
2.68618 4.42225 2.77519 2.73123 3.46354 2.40513 3.72494 3.29354 2.67741 2.69355 4.24690 2.90347 2.73739 3.18146 2.89801 2.37887 2.77519 2.98518 4.58477 3.61503
0.45866 4.24903 1.03961 0.61958 0.77255 0.38770 1.13511
616 2.88671 4.28257 4.52025 3.94401 3.34838 3.37609 4.43567 1.92969 3.81032 1.49970 3.09397 4.11781 4.41818 4.01201 3.95693 3.39318 3.12379 1.55163 5.01604 3.84089 1119 - -
2.68618 4.42225 2.77519 2.73123 3.46354 2.40513 3.72494 3.29354 2.67741 2.69355 4.24690 2.90347 2.73739 3.18146 2.89801 2.37887 2.77519 2.98518 4.58477 3.61503
0.01987 4.32450 5.04685 0.61958 0.77255 0.97787 0.47179
617 2.48054 4.84609 2.98831 2.60933 4.37665 3.38595 3.86091 3.80755 2.64101 3.39551 4.21318 3.08908 1.73146 2.10403 3.05788 2.48709 2.99415 3.41365 5.60519 4.27880 1120 - -
2.68618 4.42225 2.77519 2.73123 3.46354 2.40513 3.72494 3.29354 2.67741 2.69355 4.24690 2.90347 2.73739 3.18146 2.89801 2.37887 2.77519 2.98518 4.58477 3.61503
0.01987 4.32450 5.04685 0.61958 0.77255 0.97787 0.47179
618 2.76115 5.12684 2.73696 2.30558 4.46017 3.47205 3.69541 3.90542 2.39846 3.42691 4.21385 2.97115 3.11706 2.81909 1.56795 2.53742 2.99623 3.52055 5.58754 4.22515 1121 - -
2.68618 4.42225 2.77519 2.73123 3.46354 2.40513 3.72494 3.29354 2.67741 2.69355 4.24690 2.90347 2.73739 3.18146 2.89801 2.37887 2.77519 2.98518 4.58477 3.61503
0.01987 4.32450 5.04685 0.61958 0.77255 0.97787 0.47179
619 2.53860 5.15799 2.68403 2.09503 4.48729 3.42500 3.63840 3.96421 1.92557 3.45712 4.20802 2.49347 3.43706 2.74368 2.88375 2.38126 2.69667 3.54118 5.60082 4.19491 1122 - -
2.68618 4.42225 2.77519 2.73123 3.46354 2.40513 3.72494 3.29354 2.67741 2.69355 4.24690 2.90347 2.73739 3.18146 2.89801 2.37887 2.77519 2.98518 4.58477 3.61503
0.01987 4.32450 5.04685 0.61958 0.77255 0.97787 0.47179
620 2.23765 4.48347 3.39658 2.86204 3.87403 3.42186 3.91717 3.23055 2.83360 2.68787 3.79221 3.26670 2.14292 3.15887 3.22966 2.21943 2.42980 2.64141 5.25438 3.99397 1123 - -
2.68618 4.42225 2.77519 2.73123 3.46354 2.40513 3.72494 3.29354 2.67741 2.69355 4.24690 2.90347 2.73739 3.18146 2.89801 2.37887 2.77519 2.98518 4.58477 3.61503
0.01987 4.32450 5.04685 0.61958 0.77255 0.72822 0.65926
621 2.69196 4.80445 3.13347 2.58469 3.75612 3.53478 3.34918 3.42642 2.37270 2.24776 3.88552 2.04430 3.92680 2.91784 3.00278 2.46202 2.80712 3.12649 5.31989 4.00448 1124 - -
2.68618 4.42225 2.77519 2.73123 3.46354 2.40513 3.72494 3.29354 2.67741 2.69355 4.24690 2.90347 2.73739 3.18146 2.89801 2.37887 2.77519 2.98518 4.58477 3.61503
0.01769 4.43923 5.16158 0.61958 0.77255 0.86509 0.54648
622 2.42021 5.25484 1.91850 2.24315 4.60470 3.45125 3.81301 4.06999 2.65808 3.60079 4.39118 2.95613 1.86014 2.94722 3.17158 2.80797 2.83942 3.66456 5.77881 4.36658 1125 - -
2.68618 4.42225 2.77519 2.73123 3.46354 2.40513 3.72494 3.29354 2.67741 2.69355 4.24690 2.90347 2.73739 3.18146 2.89801 2.37887 2.77519 2.98518 4.58477 3.61503
0.01769 4.43923 5.16158 0.61958 0.77255 0.86509 0.54648
623 1.91665 4.89089 3.06542 2.44580 3.59172 3.50832 3.72354 3.55563 2.52031 3.01821 3.97100 2.82932 3.90601 2.07544 2.98002 2.48633 2.92310 3.23024 5.39905 4.06338 1126 - -
2.68599 4.42239 2.77533 2.73137 3.46368 2.40527 3.72508 3.29344 2.67716 2.69317 4.24704 2.90330 2.73753 3.18160 2.89815 2.37881 2.77513 2.98532 4.58491 3.61517

0.18222 1.82727 5.16158 1.20705 0.35536 0.86509 0.54648

624 2.34535 4.07775 2.59499 2.49314 4.20976 3.48850 3.70527 3.63797 2.49036 2.97464 4.01839 2.34771 3.49373 2.84234 2.96102 2.32771 2.48047 3.13610 5.44251 4.09305 1131 - -

2.68618 4.42225 2.77519 2.73123 3.46354 2.40513 3.72494 3.29354 2.67741 2.69355 4.24690 2.90347 2.73739 3.18146 2.89801 2.37887 2.77519 2.98518 4.58477 3.61503

0.01769 4.43923 5.16158 0.61958 0.77255 0.86509 0.54648

625 2.69005 4.34018 3.69732 2.87530 3.47821 3.69664 3.98132 2.65063 3.06658 1.72923 3.45171 3.49911 4.08143 3.36083 3.38430 2.56284 2.13142 2.35642 4.95193 3.73090 1132 - -

2.68618 4.42225 2.77519 2.73123 3.46354 2.40513 3.72494 3.29354 2.67741 2.69355 4.24690 2.90347 2.73739 3.18146 2.89801 2.37887 2.77519 2.98518 4.58477 3.61503

0.01769 4.43923 5.16158 0.61958 0.77255 0.86509 0.54648

626 3.06324 4.41984 4.78563 4.21828 3.45295 4.30934 4.69413 1.77470 4.08157 1.43119 3.33608 4.37598 3.60120 4.26724 4.20878 3.63712 3.30237 1.39391 5.21055 4.04803 1133 - -

2.68618 4.42225 2.77519 2.73123 3.46354 2.40513 3.72494 3.29354 2.67741 2.69355 4.24690 2.90347 2.73739 3.18146 2.89801 2.37887 2.77519 2.98518 4.58477 3.61503

0.01769 4.43923 5.16158 0.61958 0.77255 0.86509 0.54648

627 2.06024 5.17865 2.94090 1.90364 4.51032 3.48665 3.68084 3.97392 1.95722 3.47224 4.23323 2.75425 3.89138 2.78944 2.61195 2.57889 2.96767 3.56423 5.61663 4.23104 1134 - -

2.68618 4.42225 2.77519 2.73123 3.46354 2.40513 3.72494 3.29354 2.67741 2.69355 4.24690 2.90347 2.73739 3.18146 2.89801 2.37887 2.77519 2.98518 4.58477 3.61503

0.01769 4.43923 5.16158 0.61958 0.77255 0.86509 0.54648

628 2.20337 4.81025 3.14170 1.59664 4.03687 3.54638 3.78064 3.36049 2.59790 2.62918 3.42647 3.11532 3.94830 2.95252 3.04020 2.77500 2.84420 3.07729 5.35572 4.04316 1135 - -

2.68618 4.42225 2.77519 2.73123 3.46354 2.40513 3.72494 3.29354 2.67741 2.69355 4.24690 2.90347 2.73739 3.18146 2.89801 2.37887 2.77519 2.98518 4.58477 3.61503

0.01769 4.43923 5.16158 0.61958 0.77255 0.86509 0.54648

629 0.85161 4.39046 3.89793 3.50106 4.17331 3.32933 4.43016 2.85143 3.45110 3.21429 4.12817 3.64995 4.02098 3.75370 3.73916 2.36598 2.97975 2.99926 5.61635 4.40044 1136 - -

2.68618 4.42225 2.77519 2.73123 3.46354 2.40513 3.72494 3.29354 2.67741 2.69355 4.24690 2.90347 2.73739 3.18146 2.89801 2.37887 2.77519 2.98518 4.58477 3.61503

0.01769 4.43923 5.16158 0.61958 0.77255 0.86509 0.54648

630 2.71636 4.57327 3.40423 2.83285 3.73826 3.62976 3.08024 1.87496 2.53038 2.79130 3.66795 3.28047 4.01073 2.41523 2.92817 2.86908 2.94403 2.51907 5.12315 3.86849 1137 - -

2.68618 4.42225 2.77519 2.73123 3.46354 2.40513 3.72494 3.29354 2.67741 2.69355 4.24690 2.90347 2.73739 3.18146 2.89801 2.37887 2.77519 2.98518 4.58477 3.61503

0.01769 4.43923 5.16158 0.61958 0.77255 0.86509 0.54648

631 2.31910 5.18847 2.77732 1.97856 4.51865 3.45951 2.68669 3.99639 2.41343 3.48690 4.23527 2.61103 3.86524 2.49375 2.79192 2.35838 2.93926 3.57154 5.62832 4.22247 1138 - -

2.68618 4.42225 2.77519 2.73123 3.46354 2.40513 3.72494 3.29354 2.67741 2.69355 4.24690 2.90347 2.73739 3.18146 2.89801 2.37887 2.77519 2.98518 4.58477 3.61503

0.01769 4.43923 5.16158 0.61958 0.77255 0.86509 0.54648

632 3.34146 4.63988 5.11976 4.61556 3.72633 4.70627 5.26385 1.95145 4.46670 2.04568 3.25661 4.80899 5.00127 4.72435 4.63653 4.09857 3.60488 0.71760 5.71452 4.51347 1139 - -

2.68618 4.42225 2.77519 2.73123 3.46354 2.40513 3.72494 3.29354 2.67741 2.69355 4.24690 2.90347 2.73739 3.18146 2.89801 2.37887 2.77519 2.98518 4.58477 3.61503

0.01769 4.43923 5.16158 0.61958 0.77255 0.86509 0.54648

633 2.07737 4.35276 4.01293 3.56788 3.77030 2.24994 4.37220 1.31428 3.50983 2.74942 3.72614 3.76867 4.16517 3.78731 3.76590 2.97890 3.03247 2.49259 5.29727 4.09108 1140 - -

2.68618 4.42225 2.77519 2.73123 3.46354 2.40513 3.72494 3.29354 2.67741 2.69355 4.24690 2.90347 2.73739 3.18146 2.89801 2.37887 2.77519 2.98518 4.58477 3.61503

0.01769 4.43923 5.16158 0.61958 0.77255 0.86509 0.54648

634 2.70487 4.82135 3.08776 2.74017 4.38319 2.65860 2.35920 3.89978 2.82339 3.49400 4.31383 3.19379 3.97199 3.17331 3.24400 1.27779 3.04838 3.48044 5.65977 4.31444 1141 - -

2.68618 4.42225 2.77519 2.73123 3.46354 2.40513 3.72494 3.29354 2.67741 2.69355 4.24690 2.90347 2.73739 3.18146 2.89801 2.37887 2.77519 2.98518 4.58477 3.61503

0.01769 4.43923 5.16158 0.61958 0.77255 0.86509 0.54648

635 3.40992 0.27671 4.98841 4.87905 4.80091 3.87936 5.41061 4.09156 4.73311 3.95409 5.10397 4.73322 4.62124 5.05449 4.74292 3.67791 3.92402 3.78379 6.04133 5.07310 1142 - -

2.68618 4.42225 2.77519 2.73123 3.46354 2.40513 3.72494 3.29354 2.67741 2.69355 4.24690 2.90347 2.73739 3.18146 2.89801 2.37887 2.77519 2.98518 4.58477 3.61503
0.01769 4.43923 5.16158 0.61958 0.77255 0.86509 0.54648
636 2.67642 4.54309 3.20198 2.79295 3.19170 2.38323 2.85800 2.78312 2.76471 2.76613 3.63822 3.07978 3.98330 2.95884 3.16693 2.82831 2.71384 2.83148 3.01034 3.83290 1143 - -
2.68618 4.42225 2.77519 2.73123 3.46354 2.40513 3.72494 3.29354 2.67741 2.69355 4.24690 2.90347 2.73739 3.18146 2.89801 2.37887 2.77519 2.98518 4.58477 3.61503
0.01769 4.43923 5.16158 0.61958 0.77255 0.86509 0.54648
637 4.18791 5.35092 4.98384 4.78714 2.07644 4.76782 3.75186 3.87746 4.60737 3.13585 4.46929 4.43768 5.10138 4.54482 4.59621 4.20605 4.42508 3.81565 3.86763 0.48855 1144 - -
2.68618 4.42225 2.77519 2.73123 3.46354 2.40513 3.72494 3.29354 2.67741 2.69355 4.24690 2.90347 2.73739 3.18146 2.89801 2.37887 2.77519 2.98518 4.58477 3.61503
0.01769 4.43923 5.16158 0.61958 0.77255 0.86509 0.54648
638 3.59596 5.70859 2.90509 0.42866 5.15137 3.75689 4.44451 4.73592 3.41354 4.27140 5.26223 3.43556 4.39978 3.67429 3.85506 3.54037 3.91760 4.37359 6.20417 4.98506 1145 - -
2.68618 4.42225 2.77519 2.73123 3.46354 2.40513 3.72494 3.29354 2.67741 2.69355 4.24690 2.90347 2.73739 3.18146 2.89801 2.37887 2.77519 2.98518 4.58477 3.61503
0.01769 4.43923 5.16158 0.61958 0.77255 0.86509 0.54648
639 2.60735 4.27210 1.95170 2.13456 4.22864 3.49330 3.70855 3.66208 2.49864 3.08514 4.04191 3.00639 3.89526 2.84608 2.97267 2.62919 2.93131 3.31677 5.46032 2.71344 1146 - -
2.68618 4.42225 2.77519 2.73123 3.46354 2.40513 3.72494 3.29354 2.67741 2.69355 4.24690 2.90347 2.73739 3.18146 2.89801 2.37887 2.77519 2.98518 4.58477 3.61503
0.01769 4.43923 5.16158 0.61958 0.77255 0.30559 1.33442
640 2.80764 5.03036 3.19374 2.62849 4.28609 3.62178 3.80213 2.36529 1.69695 3.17649 3.60518 2.47225 4.00926 2.34011 2.96675 2.83247 3.03471 3.36876 5.51234 4.18241 1147 - -
2.68618 4.42225 2.77519 2.73123 3.46354 2.40513 3.72494 3.29354 2.67741 2.69355 4.24690 2.90347 2.73739 3.18146 2.89801 2.37887 2.77519 2.98518 4.58477 3.61503
0.01372 4.69125 5.41360 0.61958 0.77255 0.48576 0.95510
641 2.39716 4.82866 3.38617 2.86842 4.31590 3.55236 4.01155 3.71008 2.18610 3.34635 4.17225 3.31589 4.05331 3.18884 3.22406 2.43479 1.26682 3.17946 5.60058 4.30513 1148 - -
2.68618 4.42225 2.77519 2.73123 3.46354 2.40513 3.72494 3.29354 2.67741 2.69355 4.24690 2.90347 2.73739 3.18146 2.89801 2.37887 2.77519 2.98518 4.58477 3.61503
0.01372 4.69125 5.41360 0.61958 0.77255 0.48576 0.95510
642 1.89717 5.37838 2.64844 1.61762 4.70717 3.55287 3.81897 4.19224 2.60629 3.68031 4.43809 2.62769 3.99855 2.72894 3.11377 2.22811 3.11856 3.76323 5.82340 4.40173 1149 - -
2.68618 4.42225 2.77519 2.73123 3.46354 2.40513 3.72494 3.29354 2.67741 2.69355 4.24690 2.90347 2.73739 3.18146 2.89801 2.37887 2.77519 2.98518 4.58477 3.61503
0.01372 4.69125 5.41360 0.61958 0.77255 0.48576 0.95510
643 3.44999 4.79447 5.12002 4.54116 3.21242 4.62869 4.76894 2.54269 4.34631 1.40446 2.63979 4.66835 4.87237 4.44813 4.41137 3.95723 3.66900 2.76881 1.18418 3.81184 1150 - -
2.68618 4.42225 2.77519 2.73123 3.46354 2.40513 3.72494 3.29354 2.67741 2.69355 4.24690 2.90347 2.73739 3.18146 2.89801 2.37887 2.77519 2.98518 4.58477 3.61503
0.01372 4.69125 5.41360 0.61958 0.77255 0.48576 0.95510
644 3.25665 5.71416 2.61294 1.76338 5.16800 1.03799 4.17591 4.68529 3.15305 4.18582 5.02565 2.34323 4.22474 3.34274 3.70599 3.17796 3.56882 4.23047 6.33781 4.86466 1151 - -
2.68618 4.42225 2.77519 2.73123 3.46354 2.40513 3.72494 3.29354 2.67741 2.69355 4.24690 2.90347 2.73739 3.18146 2.89801 2.37887 2.77519 2.98518 4.58477 3.61503
0.01372 4.69125 5.41360 0.61958 0.77255 0.48576 0.95510
645 2.69077 5.25511 2.51937 1.93876 3.90569 3.53676 3.72764 4.07171 1.79762 3.55663 3.94955 2.58304 3.93216 2.82720 2.95867 2.36819 2.89088 3.64114 5.69279 4.28646 1152 - -
2.68618 4.42225 2.77519 2.73123 3.46354 2.40513 3.72494 3.29354 2.67741 2.69355 4.24690 2.90347 2.73739 3.18146 2.89801 2.37887 2.77519 2.98518 4.58477 3.61503
0.07653 4.69125 2.74115 0.61958 0.77255 0.48576 0.95510
646 2.24665 4.35521 3.01537 1.88790 4.51147 3.52472 3.71496 3.97924 2.24794 3.48491 4.23865 2.73971 3.91850 2.82038 2.36618 2.33716 2.71522 3.56807 5.63779 4.24414 1153 - -
2.68618 4.42225 2.77519 2.73123 3.46354 2.40513 3.72494 3.29354 2.67741 2.69355 4.24690 2.90347 2.73739 3.18146 2.89801 2.37887 2.77519 2.98518 4.58477 3.61503
0.01461 4.62933 5.35168 0.61958 0.77255 0.41889 1.07229

647 2.85818 4.29980 4.40495 3.50621 3.41643 2.21129 4.32023 1.53211 3.69110 2.10497 3.08177 4.00601 4.35831 3.90598 3.85314 3.29241 3.09259 1.98328 4.96532 3.77613 1154 - -
2.68618 4.42225 2.77519 2.73123 3.46354 2.40513 3.72494 3.29354 2.67741 2.69355 4.24690 2.90347 2.73739 3.18146 2.89801 2.37887 2.77519 2.98518 4.58477 3.61503
0.01372 4.69125 5.41360 0.61958 0.77255 0.48576 0.95510
648 3.95902 5.58125 4.78151 4.80155 5.79700 0.13298 5.78940 5.66883 5.08014 5.17611 6.20714 4.94950 4.98168 5.34176 5.15829 4.16303 4.49355 5.04373 6.60828 5.94871 1155 - -
2.68618 4.42225 2.77519 2.73123 3.46354 2.40513 3.72494 3.29354 2.67741 2.69355 4.24690 2.90347 2.73739 3.18146 2.89801 2.37887 2.77519 2.98518 4.58477 3.61503
0.01372 4.69125 5.41360 0.61958 0.77255 0.48576 0.95510
649 3.94979 5.21104 5.22563 4.87555 2.10082 4.80816 4.07247 3.46898 4.65887 2.77817 4.08437 4.61713 5.12466 4.64118 4.65958 4.18505 4.18719 2.93584 0.70185 2.51328 1156 - -
2.68618 4.42225 2.77519 2.73123 3.46354 2.40513 3.72494 3.29354 2.67741 2.69355 4.24690 2.90347 2.73739 3.18146 2.89801 2.37887 2.77519 2.98518 4.58477 3.61503
0.01372 4.69125 5.41360 0.61958 0.77255 0.48576 0.95510
650 2.80510 4.49431 3.74089 3.15566 3.63025 3.78656 3.73556 1.75069 2.26444 1.92684 3.40856 3.32928 4.16119 3.36006 2.81168 3.04316 3.03288 2.76498 5.07035 3.84717 1157 - -
2.68618 4.42225 2.77519 2.73123 3.46354 2.40513 3.72494 3.29354 2.67741 2.69355 4.24690 2.90347 2.73739 3.18146 2.89801 2.37887 2.77519 2.98518 4.58477 3.61503
0.01372 4.69125 5.41360 0.61958 0.77255 0.48576 0.95510
651 3.13911 5.51551 2.00750 2.04211 4.58001 3.62003 4.02662 4.22991 2.97532 3.78067 4.62200 3.10046 4.16395 3.20645 3.50730 2.92854 3.40146 3.86770 5.87818 1.22444 1158 - -
2.68618 4.42225 2.77519 2.73123 3.46354 2.40513 3.72494 3.29354 2.67741 2.69355 4.24690 2.90347 2.73739 3.18146 2.89801 2.37887 2.77519 2.98518 4.58477 3.61503
0.01372 4.69125 5.41360 0.61958 0.77255 0.48576 0.95510
652 2.98321 5.45534 2.22866 2.43665 4.78889 1.55168 3.88886 4.26450 2.66022 3.75554 4.53484 3.06675 4.06678 2.86238 1.95163 2.93127 3.22721 3.84772 5.89860 4.48894 1159 - -
2.68618 4.42225 2.77519 2.73123 3.46354 2.40513 3.72494 3.29354 2.67741 2.69355 4.24690 2.90347 2.73739 3.18146 2.89801 2.37887 2.77519 2.98518 4.58477 3.61503
0.01372 4.69125 5.41360 0.61958 0.77255 0.48576 0.95510
653 2.59891 3.41949 4.36276 3.98677 4.71371 3.35443 4.84214 4.09388 3.91321 3.82449 4.65166 3.91978 4.13384 4.16997 4.15730 0.86349 1.53899 3.53849 6.09396 4.92944 1160 - -
2.68618 4.42225 2.77519 2.73123 3.46354 2.40513 3.72494 3.29354 2.67741 2.69355 4.24690 2.90347 2.73739 3.18146 2.89801 2.37887 2.77519 2.98518 4.58477 3.61503
0.01372 4.69125 5.41360 0.61958 0.77255 0.48576 0.95510
654 2.92826 4.35072 4.51769 3.93796 3.48798 4.09235 4.43829 2.16942 3.80960 2.18169 3.26666 4.11620 3.68840 4.02306 3.96848 3.39562 1.89142 1.31947 5.06791 3.87865 1161 - -
2.68618 4.42225 2.77519 2.73123 3.46354 2.40513 3.72494 3.29354 2.67741 2.69355 4.24690 2.90347 2.73739 3.18146 2.89801 2.37887 2.77519 2.98518 4.58477 3.61503
0.01372 4.69125 5.41360 0.61958 0.77255 0.48576 0.95510
655 2.12152 4.45218 4.32449 3.86343 4.01974 3.62495 4.60675 2.99572 3.78070 3.02938 3.97455 3.95603 4.25958 4.03524 4.01240 2.86300 1.42734 1.36020 5.52848 4.33772 1162 - -
2.68618 4.42225 2.77519 2.73123 3.46354 2.40513 3.72494 3.29354 2.67741 2.69355 4.24690 2.90347 2.73739 3.18146 2.89801 2.37887 2.77519 2.98518 4.58477 3.61503
0.01372 4.69125 5.41360 0.61958 0.77255 0.48576 0.95510
656 3.99545 6.04291 3.21219 0.27258 5.55080 4.06138 4.81920 5.21630 3.84366 4.71117 5.73204 3.78019 4.72360 4.06935 4.28796 3.93140 4.33090 4.83665 6.53187 5.38164 1163 - -
2.68618 4.42225 2.77519 2.73123 3.46354 2.40513 3.72494 3.29354 2.67741 2.69355 4.24690 2.90347 2.73739 3.18146 2.89801 2.37887 2.77519 2.98518 4.58477 3.61503
0.01372 4.69125 5.41360 0.61958 0.77255 0.48576 0.95510
657 4.11833 6.15002 0.23244 3.22676 5.69734 4.06894 4.94997 5.52749 4.21940 4.99136 6.05124 3.80265 4.77227 4.23299 4.79197 4.03199 4.49046 5.09507 6.65939 5.52175 1164 - -
2.68618 4.42225 2.77519 2.73123 3.46354 2.40513 3.72494 3.29354 2.67741 2.69355 4.24690 2.90347 2.73739 3.18146 2.89801 2.37887 2.77519 2.98518 4.58477 3.61503
0.01372 4.69125 5.41360 0.61958 0.77255 0.48576 0.95510
658 2.76826 4.25112 4.69644 4.09775 3.34631 4.04789 4.38662 1.81913 3.92035 2.27820 1.94649 4.16052 4.40252 4.08063 3.98512 3.35808 2.72019 1.69081 4.91457 3.01234 1165 - -
2.68618 4.42225 2.77519 2.73123 3.46354 2.40513 3.72494 3.29354 2.67741 2.69355 4.24690 2.90347 2.73739 3.18146 2.89801 2.37887 2.77519 2.98518 4.58477 3.61503

0.01372 4.69125 5.41360 0.61958 0.77255 0.48576 0.95510
659 2.76995 4.57888 2.16016 2.95345 3.47625 3.71400 3.95378 2.86864 2.92143 2.10443 3.67476 2.55013 4.09605 3.23739 3.30805 2.94885 3.00175 2.17626 5.14423 3.34523 1166 - -
2.68618 4.42225 2.77519 2.73123 3.46354 2.40513 3.72494 3.29354 2.67741 2.69355 4.24690 2.90347 2.73739 3.18146 2.89801 2.37887 2.77519 2.98518 4.58477 3.61503
0.01372 4.69125 5.41360 0.61958 0.77255 0.48576 0.95510
660 3.15620 4.60681 4.63425 4.08859 3.62232 4.29280 4.72690 1.97543 3.94992 1.74653 3.50802 4.31892 4.66296 4.20284 4.14919 3.62571 1.17066 2.30778 5.37371 4.19802 1167 - -
2.68618 4.42225 2.77519 2.73123 3.46354 2.40513 3.72494 3.29354 2.67741 2.69355 4.24690 2.90347 2.73739 3.18146 2.89801 2.37887 2.77519 2.98518 4.58477 3.61503
0.01372 4.69125 5.41360 0.61958 0.77255 0.48576 0.95510
661 1.37305 4.54747 4.19045 3.95697 5.05639 1.05958 4.95369 4.52212 4.03677 4.19218 4.96831 3.90143 4.13268 4.25333 4.29197 1.86920 3.14507 3.78562 6.38038 5.23102 1168 - -
2.68618 4.42225 2.77519 2.73123 3.46354 2.40513 3.72494 3.29354 2.67741 2.69355 4.24690 2.90347 2.73739 3.18146 2.89801 2.37887 2.77519 2.98518 4.58477 3.61503
0.01372 4.69125 5.41360 0.61958 0.77255 0.48576 0.95510
662 3.01149 4.36963 4.87526 4.28935 1.86815 4.19168 4.41690 2.03658 4.10148 2.15165 3.36443 4.30345 4.52926 4.22267 4.13461 3.50932 3.24291 1.56246 4.83581 2.34576 1169 - -
2.68618 4.42225 2.77519 2.73123 3.46354 2.40513 3.72494 3.29354 2.67741 2.69355 4.24690 2.90347 2.73739 3.18146 2.89801 2.37887 2.77519 2.98518 4.58477 3.61503
0.01372 4.69125 5.41360 0.61958 0.77255 0.48576 0.95510
663 2.96305 5.24153 3.33648 2.73401 4.58662 2.58049 3.82763 3.99586 1.88858 3.51281 3.47879 3.22941 4.10215 2.95223 1.39643 2.96121 2.91223 3.63894 5.65751 4.35796 1170 - -
2.68618 4.42225 2.77519 2.73123 3.46354 2.40513 3.72494 3.29354 2.67741 2.69355 4.24690 2.90347 2.73739 3.18146 2.89801 2.37887 2.77519 2.98518 4.58477 3.61503
0.01372 4.69125 5.41360 0.61958 0.77255 0.48576 0.95510
664 1.96397 4.46489 4.85055 4.26879 3.47856 4.31406 4.67306 1.78143 4.12163 1.98961 1.66167 4.39575 4.63770 4.28669 4.22321 3.63312 3.33249 2.07468 5.18380 4.02532 1171 - -
2.68618 4.42225 2.77519 2.73123 3.46354 2.40513 3.72494 3.29354 2.67741 2.69355 4.24690 2.90347 2.73739 3.18146 2.89801 2.37887 2.77519 2.98518 4.58477 3.61503
0.01372 4.69125 5.41360 0.61958 0.77255 0.48576 0.95510
665 2.79013 4.29260 3.27560 2.73977 4.07807 3.04007 1.40438 3.49328 2.69990 3.13101 3.97169 3.23029 4.04006 2.84860 3.12422 2.67532 2.90391 3.19751 5.39785 3.72987 1172 - -
2.68618 4.42225 2.77519 2.73123 3.46354 2.40513 3.72494 3.29354 2.67741 2.69355 4.24690 2.90347 2.73739 3.18146 2.89801 2.37887 2.77519 2.98518 4.58477 3.61503
0.01372 4.69125 5.41360 0.61958 0.77255 0.48576 0.95510
666 2.49357 2.52222 3.63723 2.85664 3.67008 3.72027 4.02135 3.02843 3.04197 1.71554 3.63408 2.55437 4.12385 3.34849 3.39527 2.48273 3.00786 2.79663 5.11116 3.87619 1173 - -
2.68618 4.42225 2.77519 2.73123 3.46354 2.40513 3.72494 3.29354 2.67741 2.69355 4.24690 2.90347 2.73739 3.18146 2.89801 2.37887 2.77519 2.98518 4.58477 3.61503
0.01372 4.69125 5.41360 0.61958 0.77255 0.48576 0.95510
667 2.87561 4.30115 3.19735 2.34899 4.55510 3.64644 2.53958 3.98980 2.24070 3.18508 4.28566 2.96959 4.03000 2.90045 1.47940 2.86210 3.09514 3.61238 5.65146 4.31132 1174 - -
2.68618 4.42225 2.77519 2.73123 3.46354 2.40513 3.72494 3.29354 2.67741 2.69355 4.24690 2.90347 2.73739 3.18146 2.89801 2.37887 2.77519 2.98518 4.58477 3.61503
0.01372 4.69125 5.41360 0.61958 0.77255 0.48576 0.95510
668 3.95902 5.58125 4.78151 4.80155 5.79700 0.13298 5.78940 5.66883 5.08014 5.17611 6.20714 4.94950 4.98168 5.34176 5.15829 4.16303 4.49355 5.04373 6.60828 5.94871 1175 - -
2.68618 4.42225 2.77519 2.73123 3.46354 2.40513 3.72494 3.29354 2.67741 2.69355 4.24690 2.90347 2.73739 3.18146 2.89801 2.37887 2.77519 2.98518 4.58477 3.61503
0.01372 4.69125 5.41360 0.61958 0.77255 0.48576 0.95510
669 3.71058 5.28239 4.71625 4.49470 3.14079 4.28212 4.43735 4.19551 4.15191 3.62659 4.80648 4.48448 4.88404 4.55488 4.26610 3.29018 4.10496 3.98057 0.42306 3.10070 1176 - -
2.68618 4.42225 2.77519 2.73123 3.46354 2.40513 3.72494 3.29354 2.67741 2.69355 4.24690 2.90347 2.73739 3.18146 2.89801 2.37887 2.77519 2.98518 4.58477 3.61503
0.01372 4.69125 5.41360 0.61958 0.77255 0.48576 0.95510
670 3.09568 5.33377 3.51152 2.86396 4.71234 3.82572 3.86099 3.43561 1.39182 3.58554 4.40711 3.33181 4.19221 2.98607 1.55716 3.09198 2.36160 3.74505 5.69990 4.44483 1177 - -

2.68618 4.42225 2.77519 2.73123 3.46354 2.40513 3.72494 3.29354 2.67741 2.69355 4.24690 2.90347 2.73739 3.18146 2.89801 2.37887 2.77519 2.98518 4.58477 3.61503
0.01372 4.69125 5.41360 0.61958 0.77255 0.48576 0.95510
671 3.00443 4.75272 4.21408 3.87479 1.63346 3.78385 4.36819 3.61413 3.85583 3.21868 4.25895 3.99359 4.41781 4.09481 4.09601 0.91689 3.41057 3.34599 4.94691 3.36816 1178 - -
2.68618 4.42225 2.77519 2.73123 3.46354 2.40513 3.72494 3.29354 2.67741 2.69355 4.24690 2.90347 2.73739 3.18146 2.89801 2.37887 2.77519 2.98518 4.58477 3.61503
0.01372 4.69125 5.41360 0.61958 0.77255 0.48576 0.95510
672 2.70457 4.31285 4.69170 4.10566 3.09546 4.12118 4.47813 1.61554 3.95080 2.20727 3.43150 4.20907 4.47559 4.13353 4.05100 2.44165 3.16532 1.40985 5.03572 3.84967 1179 - -
2.68618 4.42225 2.77519 2.73123 3.46354 2.40513 3.72494 3.29354 2.67741 2.69355 4.24690 2.90347 2.73739 3.18146 2.89801 2.37887 2.77519 2.98518 4.58477 3.61503
0.01372 4.69125 5.41360 0.61958 0.77255 0.48576 0.95510
673 4.74807 5.68500 5.55277 5.48719 1.38864 5.22039 3.88107 4.30471 5.27087 3.44935 4.83112 4.76607 5.48077 4.93723 5.08126 4.63867 4.94576 4.26942 3.95621 0.56193 1180 - -
2.68618 4.42225 2.77519 2.73123 3.46354 2.40513 3.72494 3.29354 2.67741 2.69355 4.24690 2.90347 2.73739 3.18146 2.89801 2.37887 2.77519 2.98518 4.58477 3.61503
0.01372 4.69125 5.41360 0.61958 0.77255 0.48576 0.95510
674 3.26924 1.55514 5.20184 4.64087 3.64061 4.58279 5.01866 1.67884 4.49052 1.60378 3.50644 4.72552 4.88290 4.64106 4.56152 3.93155 3.51225 1.83517 5.44585 4.28656 1181 - -
2.68618 4.42225 2.77519 2.73123 3.46354 2.40513 3.72494 3.29354 2.67741 2.69355 4.24690 2.90347 2.73739 3.18146 2.89801 2.37887 2.77519 2.98518 4.58477 3.61503
0.01372 4.69125 5.41360 0.61958 0.77255 0.48576 0.95510
675 2.68310 4.77308 3.00155 2.73789 3.73533 3.28845 3.63185 2.93467 2.72172 3.01004 2.84165 2.15220 4.02636 3.05918 3.02378 2.33883 2.82829 2.44748 5.31082 4.01788 1182 - -
2.68618 4.42225 2.77519 2.73123 3.46354 2.40513 3.72494 3.29354 2.67741 2.69355 4.24690 2.90347 2.73739 3.18146 2.89801 2.37887 2.77519 2.98518 4.58477 3.61503
0.01372 4.69125 5.41360 0.61958 0.77255 0.48576 0.95510
676 2.94508 5.24085 1.75430 2.63581 4.79577 3.53591 4.06419 4.25994 2.94812 3.81340 4.61376 3.16262 1.53075 3.21518 3.45461 2.80689 2.12867 3.81831 5.99832 4.60293 1183 - -
2.68618 4.42225 2.77519 2.73123 3.46354 2.40513 3.72494 3.29354 2.67741 2.69355 4.24690 2.90347 2.73739 3.18146 2.89801 2.37887 2.77519 2.98518 4.58477 3.61503
0.01372 4.69125 5.41360 0.61958 0.77255 0.48576 0.95510
677 2.76856 5.13529 2.48799 2.29245 4.42583 3.56036 3.75738 3.87322 1.72764 2.64786 4.19193 3.05325 3.70279 2.87590 2.99702 2.67522 2.88300 2.55342 5.60218 4.22743 1184 - -
2.68618 4.42225 2.77519 2.73123 3.46354 2.40513 3.72494 3.29354 2.67741 2.69355 4.24690 2.90347 2.73739 3.18146 2.89801 2.37887 2.77519 2.98518 4.58477 3.61503
0.01372 4.69125 5.41360 0.61958 0.77255 0.48576 0.95510
678 2.80061 5.24006 3.07946 2.24913 4.57176 3.57560 3.74624 3.81233 1.91356 3.22447 4.28800 3.05315 2.71601 2.75515 1.92622 2.70911 2.74398 3.62430 5.67245 4.29155 1185 - -
2.68618 4.42225 2.77519 2.73123 3.46354 2.40513 3.72494 3.29354 2.67741 2.69355 4.24690 2.90347 2.73739 3.18146 2.89801 2.37887 2.77519 2.98518 4.58477 3.61503
0.26971 4.69125 1.48182 0.61958 0.77255 0.48576 0.95510
679 2.19623 5.23169 1.90718 2.41982 4.60480 3.44807 3.83161 4.07100 2.67972 3.60549 4.40029 2.97001 1.88432 2.69791 3.18838 2.81514 3.11297 3.66444 5.78583 4.37819 1186 - -
2.68618 4.42225 2.77519 2.73123 3.46354 2.40513 3.72494 3.29354 2.67741 2.69355 4.24690 2.90347 2.73739 3.18146 2.89801 2.37887 2.77519 2.98518 4.58477 3.61503
0.01769 4.43923 5.16158 0.61958 0.77255 0.86509 0.54648
680 0.58670 4.38016 3.97844 3.79734 4.69417 3.17912 4.76673 4.02762 3.84400 3.82093 4.67667 3.74822 3.98774 4.10455 4.06212 2.37115 3.00318 3.44530 6.07668 4.89962 1187 - -
2.68618 4.42225 2.77519 2.73123 3.46354 2.40513 3.72494 3.29354 2.67741 2.69355 4.24690 2.90347 2.73739 3.18146 2.89801 2.37887 2.77519 2.98518 4.58477 3.61503
0.01769 4.43923 5.16158 0.61958 0.77255 0.30559 1.33442
681 3.13115 3.38758 4.97882 4.43926 1.13794 4.28140 4.62287 2.46666 4.27129 2.37414 3.52630 4.45209 4.66972 4.40974 4.33496 3.63519 3.39651 1.60672 5.03201 3.68239 1188 - -
2.68618 4.42225 2.77519 2.73123 3.46354 2.40513 3.72494 3.29354 2.67741 2.69355 4.24690 2.90347 2.73739 3.18146 2.89801 2.37887 2.77519 2.98518 4.58477 3.61503
0.01372 4.69125 5.41360 0.61958 0.77255 0.48576 0.95510

682 2.80508 4.25704 3.31781 2.74544 4.06210 3.66298 3.85345 3.29685 1.72648 2.16353 3.93538 3.23569 4.04648 2.89100 2.21971 2.88315 2.88691 3.16907 5.37019 3.44014 1189 - -
2.68618 4.42225 2.77519 2.73123 3.46354 2.40513 3.72494 3.29354 2.67741 2.69355 4.24690 2.90347 2.73739 3.18146 2.89801 2.37887 2.77519 2.98518 4.58477 3.61503
0.01372 4.69125 5.41360 0.61958 0.77255 0.48576 0.95510
683 2.62770 2.57282 4.02681 3.67544 4.71667 0.91042 4.68490 4.15179 3.69255 3.82468 4.63803 2.69739 4.12113 3.96371 4.00189 2.18096 3.11876 3.59220 6.06520 4.86645 1190 - -
2.68618 4.42225 2.77519 2.73123 3.46354 2.40513 3.72494 3.29354 2.67741 2.69355 4.24690 2.90347 2.73739 3.18146 2.89801 2.37887 2.77519 2.98518 4.58477 3.61503
0.01372 4.69125 5.41360 0.61958 0.77255 0.48576 0.95510
684 2.69375 4.54291 2.69562 1.82216 4.47148 3.54933 3.76724 3.92489 2.52862 3.45950 4.22870 3.05262 3.95463 2.88291 3.01238 1.75793 2.49469 3.23112 5.63628 4.25552 1191 - -
2.68618 4.42225 2.77519 2.73123 3.46354 2.40513 3.72494 3.29354 2.67741 2.69355 4.24690 2.90347 2.73739 3.18146 2.89801 2.37887 2.77519 2.98518 4.58477 3.61503
0.01372 4.69125 5.41360 0.61958 0.77255 0.48576 0.95510
685 1.59346 4.33384 4.04702 3.47493 3.47035 3.82461 4.16419 2.76857 3.38481 1.82223 2.89687 3.75353 4.22004 3.64045 3.63401 2.68081 2.59870 2.41275 4.97061 3.76866 1192 - -
2.68618 4.42225 2.77519 2.73123 3.46354 2.40513 3.72494 3.29354 2.67741 2.69355 4.24690 2.90347 2.73739 3.18146 2.89801 2.37887 2.77519 2.98518 4.58477 3.61503
0.01372 4.69125 5.41360 0.61958 0.77255 0.48576 0.95510
686 4.15935 5.69933 4.84771 4.83582 5.66285 4.34598 5.77054 5.51687 4.98028 4.99973 6.12415 5.04065 0.13320 5.33195 5.06641 4.36521 4.66460 5.04913 6.53964 5.81294 1193 - -
2.68618 4.42225 2.77519 2.73123 3.46354 2.40513 3.72494 3.29354 2.67741 2.69355 4.24690 2.90347 2.73739 3.18146 2.89801 2.37887 2.77519 2.98518 4.58477 3.61503
0.01372 4.69125 5.41360 0.61958 0.77255 0.48576 0.95510
687 2.67764 4.24404 3.28032 2.31491 3.99701 3.63401 3.85310 1.93314 2.43679 3.03952 3.88413 3.21693 3.57938 2.94844 3.14263 2.14010 2.72456 3.10749 5.33367 4.03625 1194 - -
2.68618 4.42225 2.77519 2.73123 3.46354 2.40513 3.72494 3.29354 2.67741 2.69355 4.24690 2.90347 2.73739 3.18146 2.89801 2.37887 2.77519 2.98518 4.58477 3.61503
0.01372 4.69125 5.41360 0.61958 0.77255 0.48576 0.95510
688 2.76011 3.85877 3.15323 2.59815 4.24122 3.03710 3.79788 3.66234 2.46321 3.13457 4.06473 1.81067 3.97933 2.78372 3.05576 2.18469 2.71482 2.94697 5.49311 4.15321 1195 - -
2.68618 4.42225 2.77519 2.73123 3.46354 2.40513 3.72494 3.29354 2.67741 2.69355 4.24690 2.90347 2.73739 3.18146 2.89801 2.37887 2.77519 2.98518 4.58477 3.61503
0.01372 4.69125 5.41360 0.61958 0.77255 0.48576 0.95510
689 2.92669 4.34519 4.54058 3.96540 2.22215 2.71725 4.21870 2.73368 3.81215 1.28123 3.39779 4.06888 4.39877 3.98102 3.92220 3.34365 3.15863 2.48343 4.70626 2.54700 1196 - -
2.68618 4.42225 2.77519 2.73123 3.46354 2.40513 3.72494 3.29354 2.67741 2.69355 4.24690 2.90347 2.73739 3.18146 2.89801 2.37887 2.77519 2.98518 4.58477 3.61503
0.01372 4.69125 5.41360 0.61958 0.77255 0.48576 0.95510
690 2.76080 5.18105 3.04652 2.17130 4.49070 3.54874 3.39326 3.69061 2.14313 3.20011 4.23139 3.03160 2.99740 2.74530 2.86932 2.12847 2.24211 3.31776 5.63527 4.24758 1197 - -
2.68618 4.42225 2.77519 2.73123 3.46354 2.40513 3.72494 3.29354 2.67741 2.69355 4.24690 2.90347 2.73739 3.18146 2.89801 2.37887 2.77519 2.98518 4.58477 3.61503
0.01372 4.69125 5.41360 0.61958 0.77255 0.48576 0.95510
691 1.78152 5.71552 1.29364 1.89309 5.02357 3.58298 4.01546 4.53231 2.93236 4.01290 4.81204 3.03999 4.13770 2.83710 3.49356 3.05615 3.41290 4.09422 6.16309 4.68550 1198 - -
2.68618 4.42225 2.77519 2.73123 3.46354 2.40513 3.72494 3.29354 2.67741 2.69355 4.24690 2.90347 2.73739 3.18146 2.89801 2.37887 2.77519 2.98518 4.58477 3.61503
0.01372 4.69125 5.41360 0.61958 0.77255 0.48576 0.95510
692 2.26497 4.35606 3.92889 3.35307 2.91592 3.81991 4.09259 2.81379 3.26973 2.35975 3.14747 3.67332 4.19332 3.54046 1.89001 3.08607 3.00795 2.37915 4.94380 2.46360 1199 - -
2.68618 4.42225 2.77519 2.73123 3.46354 2.40513 3.72494 3.29354 2.67741 2.69355 4.24690 2.90347 2.73739 3.18146 2.89801 2.37887 2.77519 2.98518 4.58477 3.61503
0.01372 4.69125 5.41360 0.61958 0.77255 0.48576 0.95510
693 2.82670 4.39662 3.99829 3.41298 3.48278 3.87333 4.13945 2.36446 2.68943 1.47218 3.02195 3.72827 4.24020 3.57576 2.36186 3.14459 3.05642 2.41276 4.99361 3.79328 1200 - -
2.68618 4.42225 2.77519 2.73123 3.46354 2.40513 3.72494 3.29354 2.67741 2.69355 4.24690 2.90347 2.73739 3.18146 2.89801 2.37887 2.77519 2.98518 4.58477 3.61503

0.01372 4.69125 5.41360 0.61958 0.77255 0.48576 0.95510

694 2.76237 4.69053 3.37703 2.81539 2.82822 3.21300 2.21638 3.11954 2.35600 2.73924 3.78149 2.79629 4.05092 3.12275 3.20899 2.68818 2.99386 2.42571 5.24069 3.72058 1201 - -

2.68618 4.42225 2.77519 2.73123 3.46354 2.40513 3.72494 3.29354 2.67741 2.69355 4.24690 2.90347 2.73739 3.18146 2.89801 2.37887 2.77519 2.98518 4.58477 3.61503

0.01372 4.69125 5.41360 0.61958 0.77255 0.48576 0.95510

695 3.91993 5.75155 3.82828 3.67232 5.05233 4.18515 4.80482 4.91405 3.45259 4.31244 5.39064 4.11169 4.79262 0.29938 3.68601 3.96911 4.25230 4.60469 6.17463 4.98870 1202 - -

2.68618 4.42225 2.77519 2.73123 3.46354 2.40513 3.72494 3.29354 2.67741 2.69355 4.24690 2.90347 2.73739 3.18146 2.89801 2.37887 2.77519 2.98518 4.58477 3.61503

0.01372 4.69125 5.41360 0.61958 0.77255 0.48576 0.95510

696 2.94192 4.89568 3.41030 2.91850 4.00856 3.75345 3.20139 3.45194 2.72607 3.11516 4.01111 3.37839 4.17064 1.51656 3.06668 3.04520 3.17914 1.69307 5.36982 4.05345 1203 - -

2.68618 4.42225 2.77519 2.73123 3.46354 2.40513 3.72494 3.29354 2.67741 2.69355 4.24690 2.90347 2.73739 3.18146 2.89801 2.37887 2.77519 2.98518 4.58477 3.61503

0.01372 4.69125 5.41360 0.61958 0.77255 0.48576 0.95510

697 2.82979 4.61287 3.61934 3.03412 3.73912 3.76661 2.28023 2.81348 2.73112 1.69251 3.70754 3.45373 4.14241 3.25656 2.28897 3.01504 3.05551 2.89672 5.15004 3.55146 1204 - -

2.68618 4.42225 2.77519 2.73123 3.46354 2.40513 3.72494 3.29354 2.67741 2.69355 4.24690 2.90347 2.73739 3.18146 2.89801 2.37887 2.77519 2.98518 4.58477 3.61503

0.01372 4.69125 5.41360 0.61958 0.77255 0.48576 0.95510

698 4.20784 5.84117 4.79543 4.21496 5.42824 4.43586 4.77935 5.10368 3.03505 4.46553 5.49640 4.48557 4.94794 4.00896 0.23902 4.29038 4.44972 4.80093 6.20301 5.25030 1205 - -

2.68618 4.42225 2.77519 2.73123 3.46354 2.40513 3.72494 3.29354 2.67741 2.69355 4.24690 2.90347 2.73739 3.18146 2.89801 2.37887 2.77519 2.98518 4.58477 3.61503

0.01372 4.69125 5.41360 0.61958 0.77255 0.48576 0.95510

699 4.96091 5.98022 5.47975 5.41809 3.93634 4.79260 5.22626 5.14153 5.20996 4.36393 5.71406 5.45940 5.37886 5.54048 5.16938 5.16334 5.31464 5.03920 0.14424 3.90935 1206 - -

2.68618 4.42225 2.77519 2.73123 3.46354 2.40513 3.72494 3.29354 2.67741 2.69355 4.24690 2.90347 2.73739 3.18146 2.89801 2.37887 2.77519 2.98518 4.58477 3.61503

0.01372 4.69125 5.41360 0.61958 0.77255 0.48576 0.95510

700 1.12812 4.75232 3.48325 3.03780 4.41388 3.49362 2.71595 3.83869 3.04615 3.47786 4.30383 3.42807 4.07987 3.38529 3.44972 1.89759 2.90484 3.43392 5.72445 4.44031 1207 - -

2.68618 4.42225 2.77519 2.73123 3.46354 2.40513 3.72494 3.29354 2.67741 2.69355 4.24690 2.90347 2.73739 3.18146 2.89801 2.37887 2.77519 2.98518 4.58477 3.61503

0.01372 4.69125 5.41360 0.61958 0.77255 0.48576 0.95510

701 2.76904 2.68440 4.04515 3.14952 3.43082 3.83211 4.13756 2.65299 3.37532 1.92484 3.42988 3.74597 4.21202 3.62717 3.61774 2.50523 1.85299 2.36386 4.92667 3.72489 1208 - -

2.68618 4.42225 2.77519 2.73123 3.46354 2.40513 3.72494 3.29354 2.67741 2.69355 4.24690 2.90347 2.73739 3.18146 2.89801 2.37887 2.77519 2.98518 4.58477 3.61503

0.01372 4.69125 5.41360 0.61958 0.77255 0.48576 0.95510

702 3.95902 5.58125 4.78151 4.80155 5.79700 0.13298 5.78940 5.66883 5.08014 5.17611 6.20714 4.94950 4.98168 5.34176 5.15829 4.16303 4.49355 5.04373 6.60828 5.94871 1209 - -

2.68618 4.42225 2.77519 2.73123 3.46354 2.40513 3.72494 3.29354 2.67741 2.69355 4.24690 2.90347 2.73739 3.18146 2.89801 2.37887 2.77519 2.98518 4.58477 3.61503

0.01372 4.69125 5.41360 0.61958 0.77255 0.48576 0.95510

703 2.68476 4.80956 3.02277 2.73598 4.03345 3.62219 3.87530 3.43372 2.72733 2.55436 3.92472 3.22670 2.26492 3.06789 3.16510 1.62275 3.00918 3.14427 4.54776 4.06597 1210 - -

2.68618 4.42225 2.77519 2.73123 3.46354 2.40513 3.72494 3.29354 2.67741 2.69355 4.24690 2.90347 2.73739 3.18146 2.89801 2.37887 2.77519 2.98518 4.58477 3.61503

0.01372 4.69125 5.41360 0.61958 0.77255 0.48576 0.95510

704 2.16063 4.35535 4.89318 4.30054 2.85954 4.22276 4.57994 1.87279 4.12419 2.03452 2.54494 4.35286 4.55821 4.27003 4.17517 3.54284 3.23621 1.41881 5.06605 3.89742 1211 - -

2.68618 4.42225 2.77519 2.73123 3.46354 2.40513 3.72494 3.29354 2.67741 2.69355 4.24690 2.90347 2.73739 3.18146 2.89801 2.37887 2.77519 2.98518 4.58477 3.61503

0.01372 4.69125 5.41360 0.61958 0.77255 0.48576 0.95510

705 3.18032 5.74312 2.65085 1.03967 5.06021 3.60852 4.01808 4.56979 2.87484 4.03459 4.83822 2.09799 4.15890 2.20587 3.38572 3.08689 3.44100 4.13194 6.16399 4.70272 1212 - -

2.68641 4.42052 2.77563 2.73123 3.46183 2.40556 3.72548 3.29370 2.67728 2.69230 4.24285 2.90401 2.73775 3.18188 2.89734 2.37924 2.77547 2.98563 4.58487 3.61557
0.26971 1.46127 5.41360 1.92460 0.15775 0.48576 0.95510
706 3.59785 4.80518 5.63964 5.15633 4.02462 5.17448 5.83942 0.71581 5.08018 2.43036 3.30530 5.32136 5.38336 5.29008 5.22287 4.59680 3.85318 1.39209 6.13405 4.95739 1221 - -
2.68618 4.42225 2.77519 2.73123 3.46354 2.40513 3.72494 3.29354 2.67741 2.69355 4.24690 2.90347 2.73739 3.18146 2.89801 2.37887 2.77519 2.98518 4.58477 3.61503
0.01372 4.69125 5.41360 0.61958 0.77255 0.48576 0.95510
707 2.65473 4.28732 4.76997 4.16605 1.86214 4.08715 4.41676 1.97738 3.98007 1.52856 2.85189 4.21342 4.43133 4.11696 4.02416 3.13240 3.14163 2.32966 4.90938 3.74440 1222 - -
2.68618 4.42225 2.77519 2.73123 3.46354 2.40513 3.72494 3.29354 2.67741 2.69355 4.24690 2.90347 2.73739 3.18146 2.89801 2.37887 2.77519 2.98518 4.58477 3.61503
0.01372 4.69125 5.41360 0.61958 0.77255 0.48576 0.95510
708 2.78192 4.45023 3.75109 3.17482 1.73577 3.77898 3.76030 2.81908 2.66458 2.10526 3.55477 3.55119 4.15511 3.23292 2.72457 3.03403 2.70535 2.71998 5.03057 3.80484 1223 - -
2.68618 4.42225 2.77519 2.73123 3.46354 2.40513 3.72494 3.29354 2.67741 2.69355 4.24690 2.90347 2.73739 3.18146 2.89801 2.37887 2.77519 2.98518 4.58477 3.61503
0.01372 4.69125 5.41360 0.61958 0.77255 0.48576 0.95510
709 2.86820 4.94244 3.24013 3.08991 4.94227 3.47103 4.46074 4.51109 3.39575 4.10424 4.92002 1.61808 4.18292 3.67320 3.80524 0.90029 3.31635 3.92600 6.21150 4.87094 1224 - -
2.68618 4.42225 2.77519 2.73123 3.46354 2.40513 3.72494 3.29354 2.67741 2.69355 4.24690 2.90347 2.73739 3.18146 2.89801 2.37887 2.77519 2.98518 4.58477 3.61503
0.10922 4.69125 2.36135 0.61958 0.77255 0.48576 0.95510
710 3.49841 5.64590 3.97956 2.95029 5.21865 4.07250 3.88788 4.50224 1.15400 3.84501 4.71106 3.55546 4.39845 2.79177 1.17903 3.46084 3.62022 4.17274 5.81303 4.69984 1225 - -
2.68618 4.42225 2.77519 2.73123 3.46354 2.40513 3.72494 3.29354 2.67741 2.69355 4.24690 2.90347 2.73739 3.18146 2.89801 2.37887 2.77519 2.98518 4.58477 3.61503
0.01509 4.59712 5.31946 0.61958 0.77255 0.39219 1.12571
711 2.83281 5.27675 3.10269 2.53853 4.61716 3.59724 2.48712 4.08076 2.00800 3.56544 4.32396 2.12315 3.98656 2.29005 2.55522 2.80295 3.05724 3.66832 5.69313 3.09380 1226 - -
2.68618 4.42225 2.77519 2.73123 3.46354 2.40513 3.72494 3.29354 2.67741 2.69355 4.24690 2.90347 2.73739 3.18146 2.89801 2.37887 2.77519 2.98518 4.58477 3.61503
0.01372 4.69125 5.41360 0.61958 0.77255 0.48576 0.95510
712 2.76530 2.60194 3.93206 3.36957 3.56029 3.77382 4.14630 2.42836 3.29644 2.63310 3.54575 2.34659 4.19182 3.56989 3.58260 2.01395 3.02246 1.97530 5.04254 3.83078 1227 - -
2.68618 4.42225 2.77519 2.73123 3.46354 2.40513 3.72494 3.29354 2.67741 2.69355 4.24690 2.90347 2.73739 3.18146 2.89801 2.37887 2.77519 2.98518 4.58477 3.61503
0.01372 4.69125 5.41360 0.61958 0.77255 0.48576 0.95510
713 1.90046 4.84421 3.34613 2.83860 4.37548 3.52356 4.01078 3.79498 2.71909 3.40801 4.22055 3.29151 1.65292 3.17866 3.07279 2.12361 2.70727 3.41245 5.64648 4.33851 1228 - -
2.68618 4.42225 2.77519 2.73123 3.46354 2.40513 3.72494 3.29354 2.67741 2.69355 4.24690 2.90347 2.73739 3.18146 2.89801 2.37887 2.77519 2.98518 4.58477 3.61503
0.01372 4.69125 5.41360 0.61958 0.77255 0.48576 0.95510
714 2.84436 4.56952 5.17266 4.58272 2.75735 4.50803 4.86165 1.32126 4.41203 1.35801 2.82305 4.64952 4.78787 4.50256 4.43868 3.84070 3.47831 2.09368 5.24447 4.12341 1229 - -
2.68618 4.42225 2.77519 2.73123 3.46354 2.40513 3.72494 3.29354 2.67741 2.69355 4.24690 2.90347 2.73739 3.18146 2.89801 2.37887 2.77519 2.98518 4.58477 3.61503
0.01372 4.69125 5.41360 0.61958 0.77255 0.48576 0.95510
715 2.64253 4.19441 4.45276 3.85503 2.43622 3.56102 4.22142 2.27760 3.69590 2.21901 3.16022 3.97012 4.27198 3.87945 3.54962 3.20394 2.61674 2.44110 1.81909 3.62131 1230 - -
2.68618 4.42225 2.77519 2.73123 3.46354 2.40513 3.72494 3.29354 2.67741 2.69355 4.24690 2.90347 2.73739 3.18146 2.89801 2.37887 2.77519 2.98518 4.58477 3.61503
0.01372 4.69125 5.41360 0.61958 0.77255 0.48576 0.95510
716 2.12525 4.94798 3.23468 2.71111 4.20299 2.59560 3.87197 3.63279 1.81281 3.24717 4.07653 3.20923 4.04408 3.04062 3.06486 2.87234 3.06298 3.31668 5.48196 2.15693 1231 - -
2.68618 4.42225 2.77519 2.73123 3.46354 2.40513 3.72494 3.29354 2.67741 2.69355 4.24690 2.90347 2.73739 3.18146 2.89801 2.37887 2.77519 2.98518 4.58477 3.61503
0.22806 4.69125 1.63607 0.61958 0.77255 0.48576 0.95510

717 2.58437 4.86374 3.19298 2.63835 4.12674 2.09332 3.77956 3.51231 1.73009 2.87747 3.30870 3.13495 3.96496 2.94361 2.93657 2.79480 2.97672 3.20526 5.39578 4.08880 1232 - -
2.68618 4.42225 2.77519 2.73123 3.46354 2.40513 3.72494 3.29354 2.67741 2.69355 4.24690 2.90347 2.73739 3.18146 2.89801 2.37887 2.77519 2.98518 4.58477 3.61503
0.01698 4.48017 5.20252 0.61958 0.77255 0.67373 0.71295
718 2.32140 3.69544 4.51124 3.90655 2.17257 3.85677 4.17935 2.33851 3.30258 2.12941 3.22298 3.96536 4.22187 3.88528 3.78857 3.16103 2.93772 2.08684 3.59690 2.52645 1233 - -
2.68618 4.42225 2.77519 2.73123 3.46354 2.40513 3.72494 3.29354 2.67741 2.69355 4.24690 2.90347 2.73739 3.18146 2.89801 2.37887 2.77519 2.98518 4.58477 3.61503
0.01606 4.53554 5.25789 0.61958 0.77255 0.35141 1.21637
719 2.76122 3.89544 3.08893 2.53221 4.37101 2.59529 3.25589 3.81200 2.25312 3.36880 4.15188 2.41425 3.95683 2.88475 2.72163 2.37654 2.61893 3.44695 5.56737 2.87074 1234 - -
2.68631 4.42239 2.77533 2.73137 3.46367 2.40457 3.72508 3.29368 2.67737 2.69368 4.24703 2.90360 2.73727 3.18160 2.89731 2.37900 2.77533 2.98532 4.58490 3.61517
1.06053 0.85213 1.48182 0.15502 1.94069 0.48576 0.95510
720 2.86092 5.20578 3.16648 2.59329 4.59559 2.94448 3.72116 4.02107 1.56107 3.50465 4.29310 3.09697 3.60055 2.83658 1.80976 2.55700 3.07968 3.63332 5.62051 4.30497 1237 - -
2.68618 4.42225 2.77519 2.73123 3.46354 2.40513 3.72494 3.29354 2.67741 2.69355 4.24690 2.90347 2.73739 3.18146 2.89801 2.37887 2.77519 2.98518 4.58477 3.61503
0.01769 4.43923 5.16158 0.61958 0.77255 0.86509 0.54648
721 3.65598 4.90347 5.48831 4.91234 3.26619 5.00853 5.32035 1.63114 4.77253 0.85070 2.20413 5.11836 5.10114 4.73793 4.78365 4.38067 3.86716 2.46287 5.43693 4.44580 1238 - -
2.68618 4.42225 2.77519 2.73123 3.46354 2.40513 3.72494 3.29354 2.67741 2.69355 4.24690 2.90347 2.73739 3.18146 2.89801 2.37887 2.77519 2.98518 4.58477 3.61503
0.01769 4.43923 5.16158 0.61958 0.77255 0.86509 0.54648
722 2.82566 5.22375 2.82673 2.50931 4.58649 3.30234 3.71397 4.03136 1.33703 3.51769 4.29539 3.03350 3.95340 2.82745 2.30982 2.48908 3.05045 3.63189 5.64310 4.29306 1239 - -
2.68618 4.42225 2.77519 2.73123 3.46354 2.40513 3.72494 3.29354 2.67741 2.69355 4.24690 2.90347 2.73739 3.18146 2.89801 2.37887 2.77519 2.98518 4.58477 3.61503
0.01769 4.43923 5.16158 0.61958 0.77255 0.30559 1.33442
723 3.12236 4.46999 5.04146 4.43424 1.86808 4.31788 4.61981 2.28879 4.23953 1.24699 2.80511 4.46485 4.61879 4.31654 4.24335 3.63866 3.34767 2.18845 3.18764 3.86284 1240 - -
2.68618 4.42225 2.77519 2.73123 3.46354 2.40513 3.72494 3.29354 2.67741 2.69355 4.24690 2.90347 2.73739 3.18146 2.89801 2.37887 2.77519 2.98518 4.58477 3.61503
0.01372 4.69125 5.41360 0.61958 0.77255 0.48576 0.95510
724 2.85905 4.33553 4.29908 3.71065 3.41405 3.45294 4.26648 1.85123 3.53926 1.38527 3.39479 3.93200 4.32665 3.80454 2.58037 3.25485 3.09229 2.31922 4.96353 3.77617 1241 - -
2.68618 4.42225 2.77519 2.73123 3.46354 2.40513 3.72494 3.29354 2.67741 2.69355 4.24690 2.90347 2.73739 3.18146 2.89801 2.37887 2.77519 2.98518 4.58477 3.61503
0.01372 4.69125 5.41360 0.61958 0.77255 0.48576 0.95510
725 2.76815 4.73712 3.34141 2.17488 3.15814 3.65826 3.87763 3.30054 2.75828 2.60964 3.30888 3.26313 4.04381 1.90261 3.18339 2.87785 2.88269 3.03558 5.27021 2.68480 1242 - -
2.68618 4.42225 2.77519 2.73123 3.46354 2.40513 3.72494 3.29354 2.67741 2.69355 4.24690 2.90347 2.73739 3.18146 2.89801 2.37887 2.77519 2.98518 4.58477 3.61503
0.01372 4.69125 5.41360 0.61958 0.77255 0.48576 0.95510
726 2.79731 4.93072 3.24890 2.68806 4.17929 3.18429 3.83771 3.25100 2.60642 2.58357 4.02656 2.92136 4.02452 3.00101 1.61207 2.17545 3.03120 3.27364 5.45179 4.13964 1243 - -
2.68618 4.42225 2.77519 2.73123 3.46354 2.40513 3.72494 3.29354 2.67741 2.69355 4.24690 2.90347 2.73739 3.18146 2.89801 2.37887 2.77519 2.98518 4.58477 3.61503
0.01372 4.69125 5.41360 0.61958 0.77255 0.48576 0.95510
727 3.56409 4.83976 5.48843 4.90755 1.58532 4.87106 5.17700 1.97068 4.75189 1.13761 3.22493 5.01148 5.05924 4.75486 4.74885 4.22419 3.78442 1.96574 5.40221 4.30410 1244 - -
2.68618 4.42225 2.77519 2.73123 3.46354 2.40513 3.72494 3.29354 2.67741 2.69355 4.24690 2.90347 2.73739 3.18146 2.89801 2.37887 2.77519 2.98518 4.58477 3.61503
0.01372 4.69125 5.41360 0.61958 0.77255 0.48576 0.95510
728 1.24857 4.47046 3.99875 3.53710 1.88541 2.53468 4.39039 3.36514 3.50525 3.08776 3.97417 3.74007 4.15335 3.77068 3.79684 2.47996 3.07168 3.05444 5.41887 4.18985 1245 - -
2.68618 4.42225 2.77519 2.73123 3.46354 2.40513 3.72494 3.29354 2.67741 2.69355 4.24690 2.90347 2.73739 3.18146 2.89801 2.37887 2.77519 2.98518 4.58477 3.61503

0.01372 4.69125 5.41360 0.61958 0.77255 0.48576 0.95510
729 3.99548 5.20296 5.29407 4.95571 1.74442 4.87044 4.01408 3.51581 4.76780 2.07283 4.11422 4.62657 5.15101 4.66818 4.72447 4.22742 4.21053 3.18590 4.14197 0.77076 1246 - -
2.68618 4.42225 2.77519 2.73123 3.46354 2.40513 3.72494 3.29354 2.67741 2.69355 4.24690 2.90347 2.73739 3.18146 2.89801 2.37887 2.77519 2.98518 4.58477 3.61503
0.01372 4.69125 5.41360 0.61958 0.77255 0.48576 0.95510
730 2.83115 2.91683 4.91349 4.31047 2.48175 4.17767 4.52272 1.86906 4.11543 1.37176 3.10409 4.32930 4.51318 4.23865 4.13671 3.49678 3.20373 2.01630 4.98443 3.82426 1247 - -
2.68618 4.42225 2.77519 2.73123 3.46354 2.40513 3.72494 3.29354 2.67741 2.69355 4.24690 2.90347 2.73739 3.18146 2.89801 2.37887 2.77519 2.98518 4.58477 3.61503
0.01372 4.69125 5.41360 0.61958 0.77255 0.48576 0.95510
731 2.79178 3.53062 3.26641 2.70146 4.06809 3.64153 3.56920 3.49904 2.63498 3.12727 3.96338 1.81677 4.02769 2.89453 2.03388 2.85616 3.02069 3.20435 3.60292 3.60216 1248 - -
2.68618 4.42225 2.77519 2.73123 3.46354 2.40513 3.72494 3.29354 2.67741 2.69355 4.24690 2.90347 2.73739 3.18146 2.89801 2.37887 2.77519 2.98518 4.58477 3.61503
0.01372 4.69125 5.41360 0.61958 0.77255 0.48576 0.95510
732 2.68571 4.58622 3.50792 2.94367 3.74343 3.70680 3.94865 2.88430 1.96219 2.80704 3.68709 3.38548 4.09069 3.22567 3.28664 2.52055 2.39718 1.96128 5.15792 3.12119 1249 - -
2.68618 4.42225 2.77519 2.73123 3.46354 2.40513 3.72494 3.29354 2.67741 2.69355 4.24690 2.90347 2.73739 3.18146 2.89801 2.37887 2.77519 2.98518 4.58477 3.61503
0.02796 4.69125 3.99577 0.61958 0.77255 0.48576 0.95510
733 2.56160 3.60382 4.48396 3.88552 3.02347 2.51186 4.23333 1.85153 3.72290 1.81715 3.30414 3.98714 3.76546 3.90075 3.81958 3.20998 2.75823 2.14760 4.80989 3.62715 1250 - -
2.68618 4.42225 2.77519 2.73123 3.46354 2.40513 3.72494 3.29354 2.67741 2.69355 4.24690 2.90347 2.73739 3.18146 2.89801 2.37887 2.77519 2.98518 4.58477 3.61503
0.09461 4.67721 2.51368 0.61958 0.77255 0.46837 0.98355
734 2.72461 3.78399 4.70987 4.10999 2.90872 4.01858 4.36496 1.52862 3.92450 2.21985 2.90622 4.14891 4.37478 4.07931 3.97388 3.02589 2.93879 1.59290 4.88142 3.70130 1251 - -
2.68618 4.42225 2.77519 2.73123 3.46354 2.40513 3.72494 3.29354 2.67741 2.69355 4.24690 2.90347 2.73739 3.18146 2.89801 2.37887 2.77519 2.98518 4.58477 3.61503
0.01485 4.61287 5.33522 0.61958 0.77255 0.40469 1.10016
735 2.49773 4.18674 4.48140 3.88188 3.27959 3.90838 4.22666 2.54399 3.39811 2.03419 2.78164 3.98382 4.27433 3.89562 3.81386 3.20810 2.87959 2.08925 3.09265 1.87803 1252 - -
2.68618 4.42225 2.77519 2.73123 3.46354 2.40513 3.72494 3.29354 2.67741 2.69355 4.24690 2.90347 2.73739 3.18146 2.89801 2.37887 2.77519 2.98518 4.58477 3.61503
0.01372 4.69125 5.41360 0.61958 0.77255 0.48576 0.95510
736 2.41432 2.87548 3.60089 3.09642 4.12046 3.15406 2.46079 3.51515 3.06166 3.19120 4.04637 3.47178 1.41818 3.39425 3.44288 2.87367 3.05490 3.18752 5.49324 4.23593 1253 - -
2.68718 4.41668 2.77641 2.73245 3.45573 2.40634 3.72616 3.29120 2.67862 2.69257 4.24579 2.90468 2.73652 3.18268 2.89922 2.37961 2.77417 2.98389 4.58551 3.61358
0.26971 1.46127 5.41360 2.70697 0.06907 0.48576 0.95510
737 2.78259 4.30590 4.08362 2.10322 1.95870 3.85666 4.13923 2.27491 3.40684 2.23161 3.40484 3.77193 3.63796 3.65117 3.63760 3.13303 3.01439 2.48337 3.56915 3.68105 1272 - -
2.68618 4.42225 2.77519 2.73123 3.46354 2.40513 3.72494 3.29354 2.67741 2.69355 4.24690 2.90347 2.73739 3.18146 2.89801 2.37887 2.77519 2.98518 4.58477 3.61503
0.01372 4.69125 5.41360 0.61958 0.77255 0.48576 0.95510
738 2.31682 4.40986 3.78146 3.20998 3.53732 3.77692 4.04557 2.68424 3.14467 2.54042 3.51883 3.57461 4.15515 3.43338 2.55953 2.82357 1.77545 2.27244 3.54500 3.78340 1273 - -
2.68618 4.42225 2.77519 2.73123 3.46354 2.40513 3.72494 3.29354 2.67741 2.69355 4.24690 2.90347 2.73739 3.18146 2.89801 2.37887 2.77519 2.98518 4.58477 3.61503
0.01372 4.69125 5.41360 0.61958 0.77255 0.48576 0.95510
739 2.78475 4.41497 3.04295 2.18127 4.47001 3.56098 3.56953 3.92263 2.45347 3.45681 4.22948 2.67192 3.96332 2.79325 3.00240 1.42839 2.81157 3.53702 5.63453 4.25581 1274 - -
2.68618 4.42225 2.77519 2.73123 3.46354 2.40513 3.72494 3.29354 2.67741 2.69355 4.24690 2.90347 2.73739 3.18146 2.89801 2.37887 2.77519 2.98518 4.58477 3.61503
0.01372 4.69125 5.41360 0.61958 0.77255 0.48576 0.95510
740 3.63334 4.87021 5.61489 5.06518 3.12983 5.05663 5.47269 1.10300 4.94278 1.20929 3.37045 5.20288 5.22260 4.97885 4.97243 4.43599 3.86338 1.83994 5.67395 4.59974 1275 - -

2.68618 4.42225 2.77519 2.73123 3.46354 2.40513 3.72494 3.29354 2.67741 2.69355 4.24690 2.90347 2.73739 3.18146 2.89801 2.37887 2.77519 2.98518 4.58477 3.61503
0.01372 4.69125 5.41360 0.61958 0.77255 0.48576 0.95510

741 3.41956 5.06819 4.43492 4.15883 1.86408 4.10218 4.59683 3.72688 4.14144 3.19652 4.38378 4.31095 0.67857 4.39459 4.34430 3.64024 3.80334 3.56327 5.08966 3.52832 1276 - -
2.68618 4.42225 2.77519 2.73123 3.46354 2.40513 3.72494 3.29354 2.67741 2.69355 4.24690 2.90347 2.73739 3.18146 2.89801 2.37887 2.77519 2.98518 4.58477 3.61503
0.01372 4.69125 5.41360 0.61958 0.77255 0.48576 0.95510

742 2.57839 4.60773 3.47342 2.31771 3.76615 3.69777 3.93459 2.70980 2.51927 1.65201 3.70471 3.22179 4.08118 3.20105 3.26985 2.71137 2.91400 2.63085 5.17344 3.91617 1277 - -
2.68618 4.42225 2.77519 2.73123 3.46354 2.40513 3.72494 3.29354 2.67741 2.69355 4.24690 2.90347 2.73739 3.18146 2.89801 2.37887 2.77519 2.98518 4.58477 3.61503
0.01372 4.69125 5.41360 0.61958 0.77255 0.48576 0.95510

743 3.02674 4.39317 4.86831 4.27006 3.33474 4.21861 4.54498 1.86158 4.08813 1.37095 3.28237 4.33647 4.54533 4.22159 4.13878 3.53496 3.00733 2.11374 2.13723 3.85068 1278 - -
2.68618 4.42225 2.77519 2.73123 3.46354 2.40513 3.72494 3.29354 2.67741 2.69355 4.24690 2.90347 2.73739 3.18146 2.89801 2.37887 2.77519 2.98518 4.58477 3.61503
0.01372 4.69125 5.41360 0.61958 0.77255 0.48576 0.95510

744 2.17772 4.23051 4.35294 3.76291 3.19254 3.41608 4.22248 2.14960 3.62735 2.31128 3.17086 3.92864 4.27369 3.83144 3.77498 2.75976 2.67649 1.51536 4.85869 3.67046 1279 - -
2.68618 4.42225 2.77519 2.73123 3.46354 2.40513 3.72494 3.29354 2.67741 2.69355 4.24690 2.90347 2.73739 3.18146 2.89801 2.37887 2.77519 2.98518 4.58477 3.61503
0.01372 4.69125 5.41360 0.61958 0.77255 0.48576 0.95510

745 2.59428 4.32034 4.13436 3.56501 3.43352 3.85367 4.19206 2.44279 3.45833 2.52511 3.45559 3.81315 4.24856 3.70787 3.68179 2.99514 2.45806 2.33122 4.94422 1.41434 1280 - -
2.68618 4.42225 2.77519 2.73123 3.46354 2.40513 3.72494 3.29354 2.67741 2.69355 4.24690 2.90347 2.73739 3.18146 2.89801 2.37887 2.77519 2.98518 4.58477 3.61503
0.01372 4.69125 5.41360 0.61958 0.77255 0.48576 0.95510

746 2.77470 1.39729 4.41307 3.83956 3.05102 3.06098 4.29228 2.40720 3.69976 2.48653 3.42131 3.96521 4.27845 3.90123 3.83993 2.90432 3.03704 2.20692 4.93180 3.74236 1281 - -
2.68618 4.42225 2.77519 2.73123 3.46354 2.40513 3.72494 3.29354 2.67741 2.69355 4.24690 2.90347 2.73739 3.18146 2.89801 2.37887 2.77519 2.98518 4.58477 3.61503
0.01372 4.69125 5.41360 0.61958 0.77255 0.48576 0.95510

747 2.77833 4.17893 4.57663 3.97278 2.41241 3.92988 4.25181 2.19067 3.79307 1.87231 3.12237 4.03573 4.29383 3.95401 3.85929 2.80343 2.59524 2.28972 4.79361 2.10893 1282 - -
2.68618 4.42225 2.77519 2.73123 3.46354 2.40513 3.72494 3.29354 2.67741 2.69355 4.24690 2.90347 2.73739 3.18146 2.89801 2.37887 2.77519 2.98518 4.58477 3.61503
0.01372 4.69125 5.41360 0.61958 0.77255 0.48576 0.95510

748 3.77150 5.00350 5.70927 5.14126 3.03437 5.16199 5.50414 1.70488 5.01195 0.72443 3.22451 5.30600 5.26645 4.96241 5.00176 4.54134 3.98814 2.09092 5.61219 4.59464 1283 - -
2.68618 4.42225 2.77519 2.73123 3.46354 2.40513 3.72494 3.29354 2.67741 2.69355 4.24690 2.90347 2.73739 3.18146 2.89801 2.37887 2.77519 2.98518 4.58477 3.61503
0.01372 4.69125 5.41360 0.61958 0.77255 0.48576 0.95510

749 2.93973 4.83926 4.04588 3.94334 5.04472 3.57173 5.02817 4.45924 4.01960 4.16505 5.10268 4.04009 0.45010 4.36193 4.24022 3.12785 2.87055 3.90905 6.35053 5.20596 1284 - -
2.68618 4.42225 2.77519 2.73123 3.46354 2.40513 3.72494 3.29354 2.67741 2.69355 4.24690 2.90347 2.73739 3.18146 2.89801 2.37887 2.77519 2.98518 4.58477 3.61503
0.01372 4.69125 5.41360 0.61958 0.77255 0.48576 0.95510

750 1.47791 4.74344 3.34135 2.78523 3.75618 3.37083 3.89378 3.02777 2.76710 3.01547 3.86670 3.26311 3.54812 2.71063 3.19568 2.27957 2.89137 2.95930 5.32068 4.03421 1285 - -
2.68618 4.42225 2.77519 2.73123 3.46354 2.40513 3.72494 3.29354 2.67741 2.69355 4.24690 2.90347 2.73739 3.18146 2.89801 2.37887 2.77519 2.98518 4.58477 3.61503
0.01372 4.69125 5.41360 0.61958 0.77255 0.48576 0.95510

751 2.84154 4.21813 4.74402 4.13542 2.46364 4.01417 4.34395 1.93795 3.93738 1.69959 3.00468 4.15531 4.36804 4.07299 3.96674 3.32533 2.66297 2.00175 4.83892 2.56101 1286 - -
2.68618 4.42225 2.77519 2.73123 3.46354 2.40513 3.72494 3.29354 2.67741 2.69355 4.24690 2.90347 2.73739 3.18146 2.89801 2.37887 2.77519 2.98518 4.58477 3.61503
0.01372 4.69125 5.41360 0.61958 0.77255 0.48576 0.95510

752 2.44502 1.82027 4.42071 3.84193 3.39985 3.88780 4.29298 1.95943 3.70018 2.46340 2.73358 3.97635 4.29367 3.90273 3.83974 2.35883 3.04370 2.43911 4.92738 3.74116 1287 - -
2.68618 4.42225 2.77519 2.73123 3.46354 2.40513 3.72494 3.29354 2.67741 2.69355 4.24690 2.90347 2.73739 3.18146 2.89801 2.37887 2.77519 2.98518 4.58477 3.61503
0.01372 4.69125 5.41360 0.61958 0.77255 0.48576 0.95510
753 2.80641 4.47661 4.11298 3.63777 3.75765 3.72274 4.39829 3.01253 3.52586 1.05881 3.73288 3.85164 4.26472 3.82462 3.76977 2.26714 2.20644 2.80326 5.29025 4.08580 1288 - -
2.68618 4.42225 2.77519 2.73123 3.46354 2.40513 3.72494 3.29354 2.67741 2.69355 4.24690 2.90347 2.73739 3.18146 2.89801 2.37887 2.77519 2.98518 4.58477 3.61503
0.01372 4.69125 5.41360 0.61958 0.77255 0.48576 0.95510
754 3.83299 5.06826 5.72404 5.15270 1.70850 5.16124 5.34776 1.72561 5.00495 0.89674 3.16298 5.28710 5.25129 4.90864 4.96305 4.53513 4.04054 2.72603 5.42883 4.30052 1289 - -
2.68618 4.42225 2.77519 2.73123 3.46354 2.40513 3.72494 3.29354 2.67741 2.69355 4.24690 2.90347 2.73739 3.18146 2.89801 2.37887 2.77519 2.98518 4.58477 3.61503
0.01372 4.69125 5.41360 0.61958 0.77255 0.48576 0.95510
755 2.76227 4.18164 3.45282 2.88969 3.43006 3.68917 2.94357 3.15407 2.68885 2.06087 3.71594 2.94375 4.07254 3.18338 3.26027 2.14816 2.21226 2.90761 5.18236 3.64719 1290 - -
2.68618 4.42225 2.77519 2.73123 3.46354 2.40513 3.72494 3.29354 2.67741 2.69355 4.24690 2.90347 2.73739 3.18146 2.89801 2.37887 2.77519 2.98518 4.58477 3.61503
0.01372 4.69125 5.41360 0.61958 0.77255 0.48576 0.95510
756 2.87078 5.28695 2.60476 2.50036 4.59301 1.85127 3.59414 3.49549 2.62758 3.58316 4.36055 1.75388 4.00821 2.82965 3.12422 2.74743 3.11225 3.66212 5.75580 4.35718 1291 - -
2.68618 4.42225 2.77519 2.73123 3.46354 2.40513 3.72494 3.29354 2.67741 2.69355 4.24690 2.90347 2.73739 3.18146 2.89801 2.37887 2.77519 2.98518 4.58477 3.61503
0.01372 4.69125 5.41360 0.61958 0.77255 0.48576 0.95510
757 2.30796 4.97521 3.15598 2.59765 4.21744 3.59066 3.41672 2.76031 2.09270 3.11013 4.04501 3.11838 3.98050 2.39829 2.93856 2.21228 2.80191 2.88784 5.47471 4.13737 1292 - -
2.68618 4.42225 2.77519 2.73123 3.46354 2.40513 3.72494 3.29354 2.67741 2.69355 4.24690 2.90347 2.73739 3.18146 2.89801 2.37887 2.77519 2.98518 4.58477 3.61503
0.01372 4.69125 5.41360 0.61958 0.77255 0.48576 0.95510
758 2.25760 4.20942 4.41243 3.81851 1.81250 3.90369 4.22449 2.13573 3.67003 2.06947 3.32489 3.95414 3.65494 3.86147 3.79341 2.74548 2.88554 2.30425 4.83111 3.64581 1293 - -
2.68618 4.42225 2.77519 2.73123 3.46354 2.40513 3.72494 3.29354 2.67741 2.69355 4.24690 2.90347 2.73739 3.18146 2.89801 2.37887 2.77519 2.98518 4.58477 3.61503
0.01372 4.69125 5.41360 0.61958 0.77255 0.48576 0.95510
759 2.32454 4.26661 4.33286 3.74958 3.38756 3.21694 4.25385 1.64930 3.62299 1.90320 3.38889 3.93469 2.94585 3.83772 3.78906 3.21631 3.04345 2.07027 4.91383 3.72368 1294 - -
2.68618 4.42225 2.77519 2.73123 3.46354 2.40513 3.72494 3.29354 2.67741 2.69355 4.24690 2.90347 2.73739 3.18146 2.89801 2.37887 2.77519 2.98518 4.58477 3.61503
0.01372 4.69125 5.41360 0.61958 0.77255 0.48576 0.95510
760 2.45673 4.19974 4.44976 3.85331 2.11283 3.91036 4.23085 2.19337 3.69773 2.38796 3.14784 3.97297 4.27741 3.88292 3.53520 2.90416 2.31991 1.77202 4.82254 3.63846 1295 - -
2.68618 4.42225 2.77519 2.73123 3.46354 2.40513 3.72494 3.29354 2.67741 2.69355 4.24690 2.90347 2.73739 3.18146 2.89801 2.37887 2.77519 2.98518 4.58477 3.61503
0.01372 4.69125 5.41360 0.61958 0.77255 0.48576 0.95510
761 3.00176 5.25963 3.15391 2.51616 4.57486 3.69793 3.89058 3.99437 2.51868 3.29352 4.35475 3.21377 1.25144 2.38798 2.68997 2.99987 3.22880 3.64789 5.70596 4.38901 1296 - -
2.68618 4.42225 2.77519 2.73123 3.46354 2.40513 3.72494 3.29354 2.67741 2.69355 4.24690 2.90347 2.73739 3.18146 2.89801 2.37887 2.77519 2.98518 4.58477 3.61503
0.01372 4.69125 5.41360 0.61958 0.77255 0.48576 0.95510
762 2.81804 5.28033 3.07644 2.19786 4.62672 3.58215 3.74887 4.09326 1.63406 3.57404 4.32674 3.05534 3.69904 2.75081 2.23812 2.45506 2.36982 3.67302 5.70169 4.31875 1297 - -
2.68618 4.42225 2.77519 2.73123 3.46354 2.40513 3.72494 3.29354 2.67741 2.69355 4.24690 2.90347 2.73739 3.18146 2.89801 2.37887 2.77519 2.98518 4.58477 3.61503
0.01372 4.69125 5.41360 0.61958 0.77255 0.48576 0.95510
763 2.53307 4.35343 3.98744 3.42002 3.50378 2.63327 4.14995 2.11875 3.33922 1.92514 3.49398 3.71654 4.20612 3.60281 3.60595 1.91060 3.01966 2.44122 4.99521 3.78871 1298 - -
2.68618 4.42225 2.77519 2.73123 3.46354 2.40513 3.72494 3.29354 2.67741 2.69355 4.24690 2.90347 2.73739 3.18146 2.89801 2.37887 2.77519 2.98518 4.58477 3.61503

0.01372 4.69125 5.41360 0.61958 0.77255 0.48576 0.95510
764 2.76154 4.69903 2.83689 2.80636 2.51529 3.66202 3.88958 2.98213 2.78550 2.58997 3.79043 2.87313 3.22360 3.11628 3.20572 1.92617 2.79942 2.81504 5.25007 3.97283 1299 - -
2.68618 4.42225 2.77519 2.73123 3.46354 2.40513 3.72494 3.29354 2.67741 2.69355 4.24690 2.90347 2.73739 3.18146 2.89801 2.37887 2.77519 2.98518 4.58477 3.61503
0.01372 4.69125 5.41360 0.61958 0.77255 0.48576 0.95510
765 2.76060 3.35366 2.83314 2.69795 4.02501 3.29373 3.12803 2.73135 2.68483 3.06474 3.90431 2.38296 2.98088 3.02596 3.12894 2.67540 2.88284 2.93682 5.35160 2.83497 1300 - -
2.68618 4.42225 2.77519 2.73123 3.46354 2.40513 3.72494 3.29354 2.67741 2.69355 4.24690 2.90347 2.73739 3.18146 2.89801 2.37887 2.77519 2.98518 4.58477 3.61503
0.01372 4.69125 5.41360 0.61958 0.77255 0.48576 0.95510
766 2.76775 4.26179 4.16799 3.58399 2.42603 3.85901 3.64821 2.61662 3.47311 1.59963 3.37558 3.81511 3.28357 3.70297 3.67330 2.56731 2.56725 2.51676 4.86357 2.94513 1301 - -
2.68618 4.42225 2.77519 2.73123 3.46354 2.40513 3.72494 3.29354 2.67741 2.69355 4.24690 2.90347 2.73739 3.18146 2.89801 2.37887 2.77519 2.98518 4.58477 3.61503
0.26971 4.69125 1.48182 0.61958 0.77255 0.48576 0.95510
767 1.93169 3.82662 4.05649 3.48335 2.23172 2.61644 4.06651 2.67435 3.37675 2.41448 3.33528 3.71147 4.13089 3.61199 3.58770 3.02632 2.91972 2.47218 3.01659 3.55267 1302 - -
2.68618 4.42225 2.77519 2.73123 3.46354 2.40513 3.72494 3.29354 2.67741 2.69355 4.24690 2.90347 2.73739 3.18146 2.89801 2.37887 2.77519 2.98518 4.58477 3.61503
0.01769 4.43923 5.16158 0.61958 0.77255 0.86509 0.54648
768 2.68539 4.16964 4.12961 3.54753 2.56059 3.35919 4.09511 2.09672 3.43028 1.81347 3.27986 3.76185 4.15603 3.65524 3.61816 2.22834 2.80712 2.39457 4.78645 3.59023 1303 - -
2.68618 4.42225 2.77519 2.73123 3.46354 2.40513 3.72494 3.29354 2.67741 2.69355 4.24690 2.90347 2.73739 3.18146 2.89801 2.37887 2.77519 2.98518 4.58477 3.61503
0.01769 4.43923 5.16158 0.61958 0.77255 0.30559 1.33442
769 2.81629 4.20088 4.67792 4.07275 3.28942 3.98525 4.31823 1.43086 3.88304 1.96690 2.97091 4.11196 4.34469 4.03440 3.93062 3.03440 2.80991 1.92570 3.43094 3.66079 1304 - -
2.68618 4.42225 2.77519 2.73123 3.46354 2.40513 3.72494 3.29354 2.67741 2.69355 4.24690 2.90347 2.73739 3.18146 2.89801 2.37887 2.77519 2.98518 4.58477 3.61503
0.13866 4.69125 2.11777 0.61958 0.77255 0.48576 0.95510
770 2.61440 3.75336 4.58726 3.97878 2.89949 3.87913 4.20564 2.27643 3.78470 1.80577 3.22492 4.00897 3.61430 3.93349 3.82681 3.18632 2.95240 1.91342 3.38111 2.37741 1305 - -
2.68618 4.42225 2.77519 2.73123 3.46354 2.40513 3.72494 3.29354 2.67741 2.69355 4.24690 2.90347 2.73739 3.18146 2.89801 2.37887 2.77519 2.98518 4.58477 3.61503
0.01554 4.56813 5.29047 0.61958 0.77255 0.70078 0.68557
771 3.10389 4.51522 4.55231 4.01950 1.44920 4.16059 4.05824 2.90157 3.87749 1.76021 3.54062 4.09616 2.72172 4.02546 4.00078 3.46722 3.33334 2.76393 4.42173 2.19901 1306 - -
2.68618 4.42225 2.77519 2.73123 3.46354 2.40513 3.72494 3.29354 2.67741 2.69355 4.24690 2.90347 2.73739 3.18146 2.89801 2.37887 2.77519 2.98518 4.58477 3.61503
0.01554 4.56813 5.29047 0.61958 0.77255 0.37154 1.17012
772 2.78209 4.16890 4.65922 4.05027 3.24707 3.45878 4.26932 2.13518 3.85406 1.65917 2.71298 4.07627 4.30546 3.99969 3.89220 3.25109 3.01406 2.05704 2.73346 2.72581 1307 - -
2.68618 4.42225 2.77519 2.73123 3.46354 2.40513 3.72494 3.29354 2.67741 2.69355 4.24690 2.90347 2.73739 3.18146 2.89801 2.37887 2.77519 2.98518 4.58477 3.61503
0.01372 4.69125 5.41360 0.61958 0.77255 0.48576 0.95510
773 2.48393 3.65668 4.50278 3.90217 3.27769 3.58043 4.23148 1.93037 3.73480 1.82909 3.05378 3.99403 4.27602 3.90838 3.82339 2.65391 2.38139 2.13113 4.80002 3.25220 1308 - -
2.68618 4.42225 2.77519 2.73123 3.46354 2.40513 3.72494 3.29354 2.67741 2.69355 4.24690 2.90347 2.73739 3.18146 2.89801 2.37887 2.77519 2.98518 4.58477 3.61503
0.01372 4.69125 5.41360 0.61958 0.77255 0.48576 0.95510
774 3.88861 5.10107 5.81303 5.24502 3.45464 5.30780 5.64730 1.70077 5.12019 0.64933 2.85129 5.44999 5.35575 5.03229 5.09960 4.70062 4.09964 2.33597 5.67906 4.71402 1309 - -
2.68618 4.42225 2.77519 2.73123 3.46354 2.40513 3.72494 3.29354 2.67741 2.69355 4.24690 2.90347 2.73739 3.18146 2.89801 2.37887 2.77519 2.98518 4.58477 3.61503
0.01372 4.69125 5.41360 0.61958 0.77255 0.48576 0.95510
775 2.68853 4.31231 4.34568 3.79556 1.41527 3.83790 4.32033 2.72415 3.67407 2.54682 3.49800 3.94713 4.28780 3.89307 3.84572 2.90476 2.14149 2.04359 5.00090 3.79022 1310 - -

2.68618 4.42225 2.77519 2.73123 3.46354 2.40513 3.72494 3.29354 2.67741 2.69355 4.24690 2.90347 2.73739 3.18146 2.89801 2.37887 2.77519 2.98518 4.58477 3.61503
0.01372 4.69125 5.41360 0.61958 0.77255 0.48576 0.95510
776 2.67315 4.41926 3.76741 2.04786 3.28370 2.61139 4.04464 2.36117 3.13909 2.04326 3.19426 3.56781 4.15588 3.42627 3.46054 2.93666 3.00034 2.40473 5.01311 3.79134 1311 - -
2.68612 4.42252 2.77546 2.73150 3.46381 2.40540 3.72521 3.29381 2.67768 2.69382 4.24246 2.90250 2.73766 3.18173 2.89828 2.37859 2.77503 2.98475 4.58504 3.61530
0.26971 1.46127 5.41360 1.28088 0.32544 0.48576 0.95510
777 2.59546 3.71489 4.20851 3.62326 3.35873 3.12189 4.17074 2.43666 3.50734 2.24694 2.24440 3.83888 4.23550 3.73116 3.69563 2.12267 2.43845 2.19708 4.86588 3.67294 1316 - -
2.68618 4.42225 2.77519 2.73123 3.46354 2.40513 3.72494 3.29354 2.67741 2.69355 4.24690 2.90347 2.73739 3.18146 2.89801 2.37887 2.77519 2.98518 4.58477 3.61503
0.01372 4.69125 5.41360 0.61958 0.77255 0.48576 0.95510
778 2.41080 4.36005 3.91406 3.34247 3.48241 3.53088 2.19613 2.08841 3.25960 1.94684 3.47397 3.66548 4.18903 3.53484 3.53826 3.07837 2.45278 2.61055 4.96612 3.75562 1317 - -
2.68618 4.42225 2.77519 2.73123 3.46354 2.40513 3.72494 3.29354 2.67741 2.69355 4.24690 2.90347 2.73739 3.18146 2.89801 2.37887 2.77519 2.98518 4.58477 3.61503
0.01372 4.69125 5.41360 0.61958 0.77255 0.48576 0.95510
779 2.42107 2.35540 3.75151 3.18042 3.34874 3.77104 4.03382 2.43400 2.52999 2.63065 3.53051 3.55427 4.14838 3.41028 2.47206 3.02455 2.64455 2.69114 5.01373 2.97915 1318 - -
2.68618 4.42225 2.77519 2.73123 3.46354 2.40513 3.72494 3.29354 2.67741 2.69355 4.24690 2.90347 2.73739 3.18146 2.89801 2.37887 2.77519 2.98518 4.58477 3.61503
0.01372 4.69125 5.41360 0.61958 0.77255 0.48576 0.95510
780 2.10804 4.21562 4.34497 3.75296 2.60948 3.00582 4.20067 2.58178 3.61459 1.78837 2.95333 3.91396 4.25639 3.81579 3.75750 3.01364 2.40214 2.17075 4.83212 3.64487 1319 - -
2.68618 4.42225 2.77519 2.73123 3.46354 2.40513 3.72494 3.29354 2.67741 2.69355 4.24690 2.90347 2.73739 3.18146 2.89801 2.37887 2.77519 2.98518 4.58477 3.61503
0.01372 4.69125 5.41360 0.61958 0.77255 0.48576 0.95510
781 2.76357 4.83535 3.25200 2.69259 4.04061 3.37888 3.83764 3.03787 2.20022 2.41449 3.91634 2.71313 4.01298 2.67113 3.11749 2.74301 2.28658 3.14860 5.36163 2.66094 1320 - -
2.68618 4.42225 2.77519 2.73123 3.46354 2.40513 3.72494 3.29354 2.67741 2.69355 4.24690 2.90347 2.73739 3.18146 2.89801 2.37887 2.77519 2.98518 4.58477 3.61503
0.01372 4.69125 5.41360 0.61958 0.77255 0.48576 0.95510
782 1.58090 4.91127 2.69774 2.80864 4.59604 2.12963 4.04928 4.04546 2.87207 3.62145 4.40536 3.26769 4.02750 3.19769 3.33274 2.01652 2.38266 3.59042 5.81826 4.48213 1321 - -
2.68618 4.42225 2.77519 2.73123 3.46354 2.40513 3.72494 3.29354 2.67741 2.69355 4.24690 2.90347 2.73739 3.18146 2.89801 2.37887 2.77519 2.98518 4.58477 3.61503
0.01372 4.69125 5.41360 0.61958 0.77255 0.48576 0.95510
783 3.16803 4.50968 5.01745 4.43044 3.44644 4.40714 4.76241 1.79070 4.26896 1.25768 2.48219 4.52194 4.70944 4.39674 4.32958 3.73271 2.34843 1.96871 5.20595 4.06655 1322 - -
2.68618 4.42225 2.77519 2.73123 3.46354 2.40513 3.72494 3.29354 2.67741 2.69355 4.24690 2.90347 2.73739 3.18146 2.89801 2.37887 2.77519 2.98518 4.58477 3.61503
0.01372 4.69125 5.41360 0.61958 0.77255 0.48576 0.95510
784 2.60268 3.84647 4.64805 4.03985 2.78265 3.94061 4.26862 2.37984 3.84610 1.37550 3.27522 4.07088 4.30361 3.99420 3.88817 2.89004 3.01256 2.13089 2.85426 3.60931 1323 - -
2.68618 4.42225 2.77519 2.73123 3.46354 2.40513 3.72494 3.29354 2.67741 2.69355 4.24690 2.90347 2.73739 3.18146 2.89801 2.37887 2.77519 2.98518 4.58477 3.61503
0.01372 4.69125 5.41360 0.61958 0.77255 0.48576 0.95510
785 2.78111 5.18350 2.67692 1.50760 4.48450 3.55516 3.75997 2.78937 2.52036 3.46957 3.89095 3.04082 3.95650 2.54929 3.00581 2.34846 3.01368 3.54964 5.64326 4.25795 1324 - -
2.68618 4.42225 2.77519 2.73123 3.46354 2.40513 3.72494 3.29354 2.67741 2.69355 4.24690 2.90347 2.73739 3.18146 2.89801 2.37887 2.77519 2.98518 4.58477 3.61503
0.01372 4.69125 5.41360 0.61958 0.77255 0.48576 0.95510
786 2.86263 4.26089 4.62785 4.02855 2.06420 2.13471 4.34547 2.23989 3.85932 1.93775 2.70198 4.11495 4.37472 4.01996 3.93765 3.32722 3.09441 2.08182 4.88224 3.70762 1325 - -
2.68618 4.42225 2.77519 2.73123 3.46354 2.40513 3.72494 3.29354 2.67741 2.69355 4.24690 2.90347 2.73739 3.18146 2.89801 2.37887 2.77519 2.98518 4.58477 3.61503
0.01372 4.69125 5.41360 0.61958 0.77255 0.48576 0.95510

787 2.81658 4.69921 3.48527 2.90636 3.86447 3.72264 3.92450 3.09837 2.06272 1.79965 2.67349 3.36001 4.10142 2.77693 2.52099 2.95789 3.04310 2.86617 5.24267 3.98663 1326 - -
2.68618 4.42225 2.77519 2.73123 3.46354 2.40513 3.72494 3.29354 2.67741 2.69355 4.24690 2.90347 2.73739 3.18146 2.89801 2.37887 2.77519 2.98518 4.58477 3.61503
0.01372 4.69125 5.41360 0.61958 0.77255 0.48576 0.95510
788 2.79408 3.86043 4.57018 3.97077 2.31747 3.93897 4.23280 2.38325 3.79267 1.86610 3.30939 3.75331 4.30349 3.95448 3.86325 2.85815 3.02633 2.44181 1.89123 3.53644 1327 - -
2.68618 4.42225 2.77519 2.73123 3.46354 2.40513 3.72494 3.29354 2.67741 2.69355 4.24690 2.90347 2.73739 3.18146 2.89801 2.37887 2.77519 2.98518 4.58477 3.61503
0.01372 4.69125 5.41360 0.61958 0.77255 0.48576 0.95510
789 2.76714 4.11635 3.09303 1.92497 4.35842 3.07901 3.76980 3.79608 2.45097 2.57654 4.14597 3.07474 3.96378 2.52690 3.01278 1.88312 2.99909 3.43607 5.56241 4.20057 1328 - -
2.68618 4.42225 2.77519 2.73123 3.46354 2.40513 3.72494 3.29354 2.67741 2.69355 4.24690 2.90347 2.73739 3.18146 2.89801 2.37887 2.77519 2.98518 4.58477 3.61503
0.01372 4.69125 5.41360 0.61958 0.77255 0.48576 0.95510
790 2.60027 4.78894 3.14126 2.74321 3.39416 1.53323 3.87013 3.38326 2.52965 2.70627 3.88564 3.23329 4.03283 3.06899 3.03820 2.86216 2.77004 3.10353 5.33522 4.04136 1329 - -
2.68618 4.42225 2.77519 2.73123 3.46354 2.40513 3.72494 3.29354 2.67741 2.69355 4.24690 2.90347 2.73739 3.18146 2.89801 2.37887 2.77519 2.98518 4.58477 3.61503
0.01372 4.69125 5.41360 0.61958 0.77255 0.48576 0.95510
791 2.76504 4.31093 3.63243 3.42935 2.69336 2.08405 3.81726 2.07000 3.34206 2.51022 3.42295 3.71943 4.20043 3.40374 3.59191 2.94836 2.99712 2.00384 4.91328 3.48315 1330 - -
2.68618 4.42225 2.77519 2.73123 3.46354 2.40513 3.72494 3.29354 2.67741 2.69355 4.24690 2.90347 2.73739 3.18146 2.89801 2.37887 2.77519 2.98518 4.58477 3.61503
0.01372 4.69125 5.41360 0.61958 0.77255 0.48576 0.95510
792 2.61642 4.89012 3.34429 2.82788 4.52058 2.42931 4.01371 3.95770 2.79419 3.53970 4.33094 3.28055 4.02647 3.16297 2.24430 1.47688 2.21795 3.52864 5.74227 4.42142 1331 - -
2.68618 4.42225 2.77519 2.73123 3.46354 2.40513 3.72494 3.29354 2.67741 2.69355 4.24690 2.90347 2.73739 3.18146 2.89801 2.37887 2.77519 2.98518 4.58477 3.61503
0.01372 4.69125 5.41360 0.61958 0.77255 0.48576 0.95510
793 2.46595 4.18803 4.62119 4.01731 3.00159 3.95570 4.28493 1.89694 3.83419 1.81019 3.29969 4.07067 4.31818 3.99223 3.89335 2.81395 3.02758 1.70773 4.81927 2.99003 1332 - -
2.68618 4.42225 2.77519 2.73123 3.46354 2.40513 3.72494 3.29354 2.67741 2.69355 4.24690 2.90347 2.73739 3.18146 2.89801 2.37887 2.77519 2.98518 4.58477 3.61503
0.01372 4.69125 5.41360 0.61958 0.77255 0.48576 0.95510
794 2.76680 5.27370 2.62041 1.93019 4.61699 3.35554 3.27431 4.10169 2.22881 3.57775 4.31531 2.38237 3.93157 2.41463 2.58086 2.72909 2.99816 3.66348 5.70664 3.10503 1333 - -
2.68618 4.42225 2.77519 2.73123 3.46354 2.40513 3.72494 3.29354 2.67741 2.69355 4.24690 2.90347 2.73739 3.18146 2.89801 2.37887 2.77519 2.98518 4.58477 3.61503
0.06159 4.69125 2.98472 0.61958 0.77255 0.48576 0.95510
795 3.03788 5.60661 1.98652 1.20124 4.92509 2.94299 3.92409 4.43268 2.49755 3.90360 4.68201 3.00370 4.06791 3.05147 3.33435 2.41771 3.29365 3.98821 6.04217 4.57838 1334 - -
2.68618 4.42225 2.77519 2.73123 3.46354 2.40513 3.72494 3.29354 2.67741 2.69355 4.24690 2.90347 2.73739 3.18146 2.89801 2.37887 2.77519 2.98518 4.58477 3.61503
0.01439 4.64405 5.36640 0.61958 0.77255 0.57694 0.82466
796 4.05654 5.36371 5.10952 4.77965 2.82869 4.70169 4.31349 3.62279 4.35660 2.34911 4.23392 4.69190 5.11025 4.64333 4.42270 4.27971 4.32407 3.63299 0.53810 2.84189 1335 - -
2.68618 4.42225 2.77519 2.73123 3.46354 2.40513 3.72494 3.29354 2.67741 2.69355 4.24690 2.90347 2.73739 3.18146 2.89801 2.37887 2.77519 2.98518 4.58477 3.61503
0.01439 4.64405 5.36640 0.61958 0.77255 0.57694 0.82466
797 2.94756 4.33082 4.69617 4.11649 2.93986 4.08857 4.25126 2.27803 3.93938 2.25558 3.36637 4.15703 4.43690 4.07797 4.00218 3.39853 3.17822 1.81022 1.58310 2.86388 1336 - -
2.68618 4.42225 2.77519 2.73123 3.46354 2.40513 3.72494 3.29354 2.67741 2.69355 4.24690 2.90347 2.73739 3.18146 2.89801 2.37887 2.77519 2.98518 4.58477 3.61503
0.01439 4.64405 5.36640 0.61958 0.77255 0.57694 0.82466
798 2.77631 4.85673 3.47629 2.92607 4.15310 3.70501 3.95733 3.45976 2.64105 3.16113 4.03206 2.58477 4.13179 3.15641 1.61784 2.99092 3.13455 1.72866 5.44780 4.18668 1337 - -
2.68618 4.42225 2.77519 2.73123 3.46354 2.40513 3.72494 3.29354 2.67741 2.69355 4.24690 2.90347 2.73739 3.18146 2.89801 2.37887 2.77519 2.98518 4.58477 3.61503

0.01439 4.64405 5.36640 0.61958 0.77255 0.57694 0.82466

799 2.84307 5.26815 2.52900 2.34378 4.59548 3.53804 3.81285 4.06202 2.60618 3.58200 4.35460 1.72605 3.98394 2.93304 3.10506 2.54761 1.91658 3.44832 5.74969 4.34812 1338 - -

2.68618 4.42225 2.77519 2.73123 3.46354 2.40513 3.72494 3.29354 2.67741 2.69355 4.24690 2.90347 2.73739 3.18146 2.89801 2.37887 2.77519 2.98518 4.58477 3.61503

0.01439 4.64405 5.36640 0.61958 0.77255 0.57694 0.82466

800 2.84411 5.46426 2.89976 1.28867 4.81622 3.61099 3.84991 4.28212 2.09400 3.74884 4.52736 3.07730 4.06401 1.99632 2.95949 2.93481 3.22176 3.86359 5.86535 4.47927 1339 - -

2.68618 4.42225 2.77519 2.73123 3.46354 2.40513 3.72494 3.29354 2.67741 2.69355 4.24690 2.90347 2.73739 3.18146 2.89801 2.37887 2.77519 2.98518 4.58477 3.61503

0.01439 4.64405 5.36640 0.61958 0.77255 0.57694 0.82466

801 3.08419 5.43135 3.39615 2.77984 4.85584 3.78326 3.82076 4.25918 1.52948 3.69197 4.49035 3.26342 4.15529 1.58281 2.11835 2.49544 3.27906 3.87334 5.75647 4.48283 1340 - -

2.68618 4.42225 2.77519 2.73123 3.46354 2.40513 3.72494 3.29354 2.67741 2.69355 4.24690 2.90347 2.73739 3.18146 2.89801 2.37887 2.77519 2.98518 4.58477 3.61503

0.01439 4.64405 5.36640 0.61958 0.77255 0.57694 0.82466

802 2.58219 4.17328 4.56765 3.96414 1.72662 3.92416 4.24452 2.58441 3.78458 1.75529 2.58888 4.02907 4.28625 3.94427 3.85113 3.03114 2.84539 2.33768 3.95916 3.59922 1341 - -

2.68618 4.42225 2.77519 2.73123 3.46354 2.40513 3.72494 3.29354 2.67741 2.69355 4.24690 2.90347 2.73739 3.18146 2.89801 2.37887 2.77519 2.98518 4.58477 3.61503

0.01439 4.64405 5.36640 0.61958 0.77255 0.57694 0.82466

803 3.14655 4.81690 4.17296 3.75482 3.33481 1.63089 4.27568 3.51779 3.56116 2.81227 4.13913 3.97358 4.46882 3.93152 3.79313 3.36426 3.47273 3.28928 1.13579 3.37360 1342 - -

2.68618 4.42225 2.77519 2.73123 3.46354 2.40513 3.72494 3.29354 2.67741 2.69355 4.24690 2.90347 2.73739 3.18146 2.89801 2.37887 2.77519 2.98518 4.58477 3.61503

0.01439 4.64405 5.36640 0.61958 0.77255 0.57694 0.82466

804 2.74879 4.73042 2.60646 2.64836 3.91589 3.63485 3.85594 3.29658 2.72698 2.28387 3.30135 3.23525 4.02126 3.06621 2.27345 2.66039 2.98022 2.09731 5.27347 3.98742 1343 - -

2.68618 4.42225 2.77519 2.73123 3.46354 2.40513 3.72494 3.29354 2.67741 2.69355 4.24690 2.90347 2.73739 3.18146 2.89801 2.37887 2.77519 2.98518 4.58477 3.61503

0.01439 4.64405 5.36640 0.61958 0.77255 0.57694 0.82466

805 2.51292 4.27736 4.10303 3.52952 3.41037 3.54315 4.15723 1.40973 3.42863 2.49106 3.41183 3.77755 4.20997 3.67132 3.65051 2.39058 2.66638 2.35439 4.91271 3.30570 1344 - -

2.68618 4.42225 2.77519 2.73123 3.46354 2.40513 3.72494 3.29354 2.67741 2.69355 4.24690 2.90347 2.73739 3.18146 2.89801 2.37887 2.77519 2.98518 4.58477 3.61503

0.01439 4.64405 5.36640 0.61958 0.77255 0.57694 0.82466

806 2.75443 4.98284 3.13786 2.57926 4.23349 2.04314 3.77464 3.47379 2.29522 2.65964 4.05286 2.96224 3.96663 2.81468 2.62912 2.26370 2.98521 3.06153 5.47760 4.13850 1345 - -

2.68618 4.42225 2.77519 2.73123 3.46354 2.40513 3.72494 3.29354 2.67741 2.69355 4.24690 2.90347 2.73739 3.18146 2.89801 2.37887 2.77519 2.98518 4.58477 3.61503

0.01439 4.64405 5.36640 0.61958 0.77255 0.57694 0.82466

807 2.48262 5.22480 2.98530 2.09825 4.55122 1.79424 3.75293 4.01694 2.25365 3.52503 4.28600 2.79892 3.95030 2.86317 2.78430 2.66871 2.83286 3.60867 5.67999 4.28771 1346 - -

2.68618 4.42225 2.77519 2.73123 3.46354 2.40513 3.72494 3.29354 2.67741 2.69355 4.24690 2.90347 2.73739 3.18146 2.89801 2.37887 2.77519 2.98518 4.58477 3.61503

0.01439 4.64405 5.36640 0.61958 0.77255 0.57694 0.82466

808 2.47466 4.37901 3.79325 3.22273 3.30644 3.46331 4.04277 2.74720 3.03224 2.31739 3.49368 3.57751 4.14356 3.44133 3.46838 2.51857 1.68539 2.19718 4.98090 3.76297 1347 - -

2.68618 4.42225 2.77519 2.73123 3.46354 2.40513 3.72494 3.29354 2.67741 2.69355 4.24690 2.90347 2.73739 3.18146 2.89801 2.37887 2.77519 2.98518 4.58477 3.61503

0.01439 4.64405 5.36640 0.61958 0.77255 0.43270 1.04627

809 2.79156 4.14088 2.50948 2.53985 4.49308 3.56034 3.79043 3.94516 2.14415 3.48000 4.25397 3.07314 3.97472 2.90926 2.88156 1.38994 3.03245 3.55420 5.65578 4.28075 1348 - -

2.68618 4.42225 2.77519 2.73123 3.46354 2.40513 3.72494 3.29354 2.67741 2.69355 4.24690 2.90347 2.73739 3.18146 2.89801 2.37887 2.77519 2.98518 4.58477 3.61503

0.26971 4.69125 1.48182 0.61958 0.77255 0.48576 0.95510

810 1.14892 2.89523 4.19269 3.80934 4.44381 3.18075 4.64109 3.81050 3.73988 3.55104 4.38869 3.74121 3.95277 3.98565 3.97839 1.35837 2.92268 3.29172 5.84038 4.67579 1349 - -

2.68618 4.42225 2.77519 2.73123 3.46354 2.40513 3.72494 3.29354 2.67741 2.69355 4.24690 2.90347 2.73739 3.18146 2.89801 2.37887 2.77519 2.98518 4.58477 3.61503
0.01769 4.43923 5.16158 0.61958 0.77255 0.86509 0.54648
811 3.83862 5.14606 4.73287 4.35395 1.76781 4.58736 1.15301 3.73302 4.18182 3.10176 4.29370 4.23070 4.90158 4.25047 4.28972 3.92707 4.05476 3.60336 2.06651 2.15616 1350 - -
2.68618 4.42225 2.77519 2.73123 3.46354 2.40513 3.72494 3.29354 2.67741 2.69355 4.24690 2.90347 2.73739 3.18146 2.89801 2.37887 2.77519 2.98518 4.58477 3.61503
0.01769 4.43923 5.16158 0.61958 0.77255 0.33801 1.24893
812 2.70319 4.34485 4.02391 3.25192 3.00948 3.56042 4.16673 2.77575 3.39247 1.08530 3.44048 3.76316 3.44708 3.65356 3.63120 3.13740 3.04084 2.58745 4.95492 3.74889 1351 - -
2.68618 4.42225 2.77519 2.73123 3.46354 2.40513 3.72494 3.29354 2.67741 2.69355 4.24690 2.90347 2.73739 3.18146 2.89801 2.37887 2.77519 2.98518 4.58477 3.61503
0.03289 4.67453 3.77099 0.61958 0.77255 0.51953 0.90337
813 2.20547 4.20289 4.40295 3.80941 1.65575 3.89926 4.21005 2.53439 3.66049 1.98503 3.31121 3.64083 4.26571 3.85158 3.78394 3.19593 2.88742 2.21578 4.81063 3.28588 1352 - -
2.68618 4.42225 2.77519 2.73123 3.46354 2.40513 3.72494 3.29354 2.67741 2.69355 4.24690 2.90347 2.73739 3.18146 2.89801 2.37887 2.77519 2.98518 4.58477 3.61503
0.01422 4.65585 5.37820 0.61958 0.77255 0.55530 0.85308
814 1.22228 4.71938 3.51028 3.04448 4.53387 2.39048 4.20209 3.96665 2.89806 3.58600 4.38817 3.41718 3.18818 3.38642 3.48273 1.90912 2.86804 3.50269 5.81380 4.52872 1353 - -
2.68618 4.42225 2.77519 2.73123 3.46354 2.40513 3.72494 3.29354 2.67741 2.69355 4.24690 2.90347 2.73739 3.18146 2.89801 2.37887 2.77519 2.98518 4.58477 3.61503
0.01422 4.65585 5.37820 0.61958 0.77255 0.49587 0.93915
815 2.75897 4.20702 4.33437 3.74177 2.52694 3.87897 4.18921 2.12383 3.21703 2.06945 3.32278 3.58440 4.24690 3.41849 3.34091 3.17133 2.88051 1.62975 4.82130 3.35461 1354 - -
2.68618 4.42225 2.77519 2.73123 3.46354 2.40513 3.72494 3.29354 2.67741 2.69355 4.24690 2.90347 2.73739 3.18146 2.89801 2.37887 2.77519 2.98518 4.58477 3.61503
0.01396 4.67453 5.39687 0.61958 0.77255 0.51953 0.90337
816 2.45195 4.21480 4.29738 3.70675 2.23317 3.59453 4.18078 2.27508 3.57449 2.02927 3.04589 3.63981 3.91819 3.04237 3.72964 3.03321 2.65904 2.03338 4.82836 3.63965 1355 - -
2.68618 4.42225 2.77519 2.73123 3.46354 2.40513 3.72494 3.29354 2.67741 2.69355 4.24690 2.90347 2.73739 3.18146 2.89801 2.37887 2.77519 2.98518 4.58477 3.61503
0.12312 4.67453 2.23945 0.61958 0.77255 0.46521 0.98887
817 2.82841 5.31710 2.06352 1.93561 4.63848 3.51655 3.76746 3.76313 2.54157 3.37551 4.37097 2.98488 3.95411 1.67994 3.03922 2.78412 3.06633 3.69587 5.75583 4.34106 1356 - -
2.68618 4.42225 2.77519 2.73123 3.46354 2.40513 3.72494 3.29354 2.67741 2.69355 4.24690 2.90347 2.73739 3.18146 2.89801 2.37887 2.77519 2.98518 4.58477 3.61503
0.01527 4.58556 5.30791 0.61958 0.77255 0.67447 0.71218
818 2.72713 4.47319 3.57551 3.01297 3.00196 1.90214 3.95720 2.30160 2.44319 2.69514 3.58623 3.42464 4.07919 3.27535 3.32272 2.79132 2.75955 2.66468 5.06360 3.82494 1357 - -
2.68618 4.42225 2.77519 2.73123 3.46354 2.40513 3.72494 3.29354 2.67741 2.69355 4.24690 2.90347 2.73739 3.18146 2.89801 2.37887 2.77519 2.98518 4.58477 3.61503
0.01527 4.58556 5.30791 0.61958 0.77255 0.38361 1.14381
819 2.76313 4.44629 3.43046 2.76613 3.34494 3.16401 4.01807 1.98915 2.96921 1.87336 3.55148 3.52246 3.79620 3.37697 3.13517 3.00828 2.58947 2.51163 5.03321 3.80581 1358 - -
2.68618 4.42225 2.77519 2.73123 3.46354 2.40513 3.72494 3.29354 2.67741 2.69355 4.24690 2.90347 2.73739 3.18146 2.89801 2.37887 2.77519 2.98518 4.58477 3.61503
0.15848 4.69125 1.98497 0.61958 0.77255 0.48576 0.95510
820 2.81804 4.60771 3.43074 2.65135 3.77712 3.69741 3.96874 3.11656 2.63558 1.24368 3.72254 3.39451 4.11261 3.25449 3.17611 2.70063 2.91584 2.88536 5.18706 3.94688 1359 - -
2.68618 4.42225 2.77519 2.73123 3.46354 2.40513 3.72494 3.29354 2.67741 2.69355 4.24690 2.90347 2.73739 3.18146 2.89801 2.37887 2.77519 2.98518 4.58477 3.61503
0.01585 4.54861 5.27096 0.61958 0.77255 0.72892 0.65861
821 3.15529 5.36501 3.38334 2.70561 4.76945 3.79690 3.86158 4.13734 0.84744 3.24513 4.47062 3.31479 3.73665 2.99500 2.52774 3.14434 3.35347 3.79941 5.72023 4.48111 1360 - -
2.68633 4.42240 2.77535 2.73129 3.46369 2.40528 3.72460 3.29340 2.67727 2.69354 4.24672 2.90342 2.73747 3.18139 2.89794 2.37884 2.77488 2.98521 4.58492 3.61518
0.12707 2.16987 5.27096 1.43885 0.27076 0.72892 0.65861

822 2.75116 4.26396 4.07319 3.23973 3.36836 3.83612 4.13110 2.52906 3.38627 1.78402 3.14251 3.75834 4.20407 3.11570 3.61290 3.11554 2.58186 1.57507 4.88669 3.68953 1367 - -
2.68618 4.42225 2.77519 2.73123 3.46354 2.40513 3.72494 3.29354 2.67741 2.69355 4.24690 2.90347 2.73739 3.18146 2.89801 2.37887 2.77519 2.98518 4.58477 3.61503
0.03907 4.54861 3.58508 0.61958 0.77255 0.72892 0.65861
823 3.03771 4.44649 4.61552 4.02746 3.08683 4.20014 4.50435 1.89161 3.87326 1.10922 3.20746 4.21748 4.51289 4.06002 2.85193 3.50615 3.26580 2.26918 5.04478 3.89603 1368 - -
2.68618 4.42225 2.77519 2.73123 3.46354 2.40513 3.72494 3.29354 2.67741 2.69355 4.24690 2.90347 2.73739 3.18146 2.89801 2.37887 2.77519 2.98518 4.58477 3.61503
0.01621 4.52576 5.24811 0.61958 0.77255 0.76023 0.63028
824 1.45531 4.64056 3.38148 2.91860 3.52798 1.92457 4.05605 3.61949 2.94837 3.27265 4.10746 2.67722 3.98513 3.26622 3.36391 2.58664 2.98313 3.24928 5.54331 4.26156 1369 - -
2.68618 4.42225 2.77519 2.73123 3.46354 2.40513 3.72494 3.29354 2.67741 2.69355 4.24690 2.90347 2.73739 3.18146 2.89801 2.37887 2.77519 2.98518 4.58477 3.61503
0.01621 4.52576 5.24811 0.61958 0.77255 0.76023 0.63028
825 2.76589 4.31060 4.06811 3.50791 3.47360 1.76744 4.20111 2.06952 3.41292 2.14191 3.45181 3.77712 4.22738 3.67482 3.65837 2.91494 3.01975 2.03488 5.00056 3.79583 1370 - -
2.68618 4.42225 2.77519 2.73123 3.46354 2.40513 3.72494 3.29354 2.67741 2.69355 4.24690 2.90347 2.73739 3.18146 2.89801 2.37887 2.77519 2.98518 4.58477 3.61503
0.01621 4.52576 5.24811 0.61958 0.77255 0.76023 0.63028
826 2.89967 4.38679 4.31326 3.76011 3.58086 4.01395 4.43554 1.75240 3.65887 2.36499 3.52204 4.00692 4.40927 3.91861 3.88962 1.69398 3.15849 1.63911 5.18276 3.97604 1371 - -
2.68618 4.42225 2.77519 2.73123 3.46354 2.40513 3.72494 3.29354 2.67741 2.69355 4.24690 2.90347 2.73739 3.18146 2.89801 2.37887 2.77519 2.98518 4.58477 3.61503
0.01621 4.52576 5.24811 0.61958 0.77255 0.76023 0.63028
827 2.67375 5.32861 1.86779 1.81496 4.65942 3.48667 3.74521 4.14845 2.13202 3.62758 4.38077 2.84958 3.92913 2.75186 3.03137 2.67079 2.62725 3.71304 5.76328 4.33858 1372 - -
2.68618 4.42225 2.77519 2.73123 3.46354 2.40513 3.72494 3.29354 2.67741 2.69355 4.24690 2.90347 2.73739 3.18146 2.89801 2.37887 2.77519 2.98518 4.58477 3.61503
0.01621 4.52576 5.24811 0.61958 0.77255 0.69637 0.68993
828 2.71580 4.59027 3.38305 2.83314 3.76793 3.62675 3.59558 2.06345 2.80284 2.82853 3.69960 3.14603 3.35151 2.98065 3.20228 2.32926 2.01030 2.89077 5.16325 3.90021 1373 - -
2.68618 4.42225 2.77519 2.73123 3.46354 2.40513 3.72494 3.29354 2.67741 2.69355 4.24690 2.90347 2.73739 3.18146 2.89801 2.37887 2.77519 2.98518 4.58477 3.61503
0.01585 4.54861 5.27096 0.61958 0.77255 0.72892 0.65861
829 2.57748 5.10681 2.98063 2.26143 4.42089 2.48710 3.73346 3.87275 2.40176 3.41210 4.18743 2.19228 3.91808 2.85344 2.98496 2.09952 2.97598 2.94319 5.59453 4.21588 1374 - -
2.68618 4.42225 2.77519 2.73123 3.46354 2.40513 3.72494 3.29354 2.67741 2.69355 4.24690 2.90347 2.73739 3.18146 2.89801 2.37887 2.77519 2.98518 4.58477 3.61503
0.01585 4.54861 5.27096 0.61958 0.77255 0.72892 0.65861
830 3.38988 4.74842 4.82476 4.33746 0.89892 4.42865 4.14989 2.90941 4.17153 1.90781 3.55495 4.34291 4.74356 4.27064 4.25546 3.75957 3.20118 2.87161 4.40741 2.62793 1375 - -
2.68618 4.42225 2.77519 2.73123 3.46354 2.40513 3.72494 3.29354 2.67741 2.69355 4.24690 2.90347 2.73739 3.18146 2.89801 2.37887 2.77519 2.98518 4.58477 3.61503
0.01585 4.54861 5.27096 0.61958 0.77255 0.72892 0.65861
831 2.72949 4.49611 3.52942 2.69183 3.65168 3.68772 3.94264 2.41503 2.92653 2.46258 3.60183 2.86696 4.07204 3.24342 3.29708 2.93159 1.68112 2.48035 5.08811 3.84390 1376 - -
2.68618 4.42225 2.77519 2.73123 3.46354 2.40513 3.72494 3.29354 2.67741 2.69355 4.24690 2.90347 2.73739 3.18146 2.89801 2.37887 2.77519 2.98518 4.58477 3.61503
0.01585 4.54861 5.27096 0.61958 0.77255 0.72892 0.65861
832 2.78119 4.38173 4.50066 3.06768 3.53161 4.15900 4.51330 1.99229 3.81795 1.72018 3.46123 4.15084 4.50328 4.05227 4.00505 3.46609 3.20676 1.25675 5.16426 3.97247 1377 - -
2.68618 4.42225 2.77519 2.73123 3.46354 2.40513 3.72494 3.29354 2.67741 2.69355 4.24690 2.90347 2.73739 3.18146 2.89801 2.37887 2.77519 2.98518 4.58477 3.61503
0.01585 4.54861 5.27096 0.61958 0.77255 0.72892 0.65861
833 2.37105 4.52464 3.52735 2.82255 3.74719 3.64031 3.98155 2.90040 2.93967 2.45136 3.68999 3.40286 4.06796 3.26836 3.30875 2.79958 1.39793 2.79545 5.17290 3.92690 1378 - -
2.68618 4.42225 2.77519 2.73123 3.46354 2.40513 3.72494 3.29354 2.67741 2.69355 4.24690 2.90347 2.73739 3.18146 2.89801 2.37887 2.77519 2.98518 4.58477 3.61503

0.01585 4.54861 5.27096 0.61958 0.77255 0.72892 0.65861

834 2.51920 4.89585 3.23606 2.77778 4.51723 3.47641 3.96684 3.93585 1.93403 3.52036 4.32541 3.23270 4.00583 3.12000 3.09126 1.23878 2.80245 3.51835 5.71539 4.40481 1379 - -

2.68618 4.42225 2.77519 2.73123 3.46354 2.40513 3.72494 3.29354 2.67741 2.69355 4.24690 2.90347 2.73739 3.18146 2.89801 2.37887 2.77519 2.98518 4.58477 3.61503

0.01585 4.54861 5.27096 0.61958 0.77255 0.72892 0.65861

835 3.46825 5.58825 3.63076 3.10088 5.08304 3.97136 3.94687 4.45578 0.65637 3.84168 4.73520 3.50508 4.37643 2.69522 2.38978 3.43988 3.63329 4.12988 5.83108 4.67494 1380 - -

2.68618 4.42225 2.77519 2.73123 3.46354 2.40513 3.72494 3.29354 2.67741 2.69355 4.24690 2.90347 2.73739 3.18146 2.89801 2.37887 2.77519 2.98518 4.58477 3.61503

0.03932 4.54861 3.57657 0.61958 0.77255 0.72892 0.65861

836 1.90789 4.63386 3.34992 2.80101 3.91837 3.21894 3.89090 3.30158 2.78147 2.58094 3.82641 2.82575 3.98477 3.11184 3.19956 2.10348 2.38191 2.67889 5.28257 4.00584 1381 - -

2.68618 4.42225 2.77519 2.73123 3.46354 2.40513 3.72494 3.29354 2.67741 2.69355 4.24690 2.90347 2.73739 3.18146 2.89801 2.37887 2.77519 2.98518 4.58477 3.61503

0.09569 4.52552 2.52045 0.61958 0.77255 0.76056 0.63000

837 2.15033 4.82590 3.11465 2.41729 4.05344 2.85717 3.73451 3.30056 2.45622 3.08167 2.91622 2.68343 3.91369 2.89760 3.00611 2.28352 2.91308 3.02062 5.34309 4.02075 1382 - -

2.68629 4.42285 2.77580 2.73068 3.46414 2.40459 3.72555 3.29403 2.67695 2.69400 4.24693 2.90396 2.73693 3.18156 2.89836 2.37848 2.77491 2.98538 4.58537 3.61496

1.00499 0.83715 1.60442 1.70245 0.20118 0.66183 0.72548

838 2.75234 5.22191 2.38477 1.95571 4.54318 3.42115 3.69463 4.01902 2.22168 3.52112 4.28898 2.89539 3.87168 2.81178 2.98531 1.81890 2.72239 3.60345 5.67513 4.26205 1397 - -

2.68635 4.42242 2.77431 2.73086 3.46371 2.40504 3.72511 3.29371 2.67758 2.69372 4.24707 2.90364 2.73757 3.18079 2.89795 2.37904 2.77536 2.98535 4.58494 3.61520

0.91535 0.70864 2.23206 0.54124 0.87233 0.62585 0.76530

839 2.87651 5.37725 1.58519 1.78185 4.68814 2.90801 3.79084 4.17401 2.64433 3.67505 4.45822 2.67942 3.93993 2.91986 3.17398 2.81466 3.12926 2.93191 5.83338 4.39445 1401 - -

2.68618 4.42225 2.77519 2.73123 3.46354 2.40513 3.72494 3.29354 2.67741 2.69355 4.24690 2.90347 2.73739 3.18146 2.89801 2.37887 2.77519 2.98518 4.58477 3.61503

0.01837 4.40207 5.12442 0.61958 0.77255 0.81333 0.58587

840 2.74981 5.56873 0.99733 2.02433 4.89038 3.45101 3.94359 4.35408 2.90623 3.89231 4.72341 2.92722 4.03720 3.09906 3.47553 2.97725 3.34393 3.50494 6.07462 4.59922 1402 - -

2.68618 4.42225 2.77519 2.73123 3.46354 2.40513 3.72494 3.29354 2.67741 2.69355 4.24690 2.90347 2.73739 3.18146 2.89801 2.37887 2.77519 2.98518 4.58477 3.61503

0.01769 4.43923 5.16158 0.61958 0.77255 0.86509 0.54648

841 2.70740 4.05182 1.97541 2.24782 4.35056 2.27447 3.70549 3.79521 2.48525 3.14841 4.13093 2.84580 3.88835 2.83068 2.96845 2.70175 2.94521 3.25846 5.54181 4.16780 1403 - -

2.68618 4.42225 2.77519 2.73123 3.46354 2.40513 3.72494 3.29354 2.67741 2.69355 4.24690 2.90347 2.73739 3.18146 2.89801 2.37887 2.77519 2.98518 4.58477 3.61503

0.01769 4.43923 5.16158 0.61958 0.77255 0.86509 0.54648

842 2.63380 5.04983 2.54875 1.64796 4.33111 3.48199 3.69848 3.13859 2.47523 2.90695 4.11241 2.97948 3.02051 2.82372 2.95673 2.70177 2.94069 3.39799 5.52628 4.15418 1404 - -

2.68618 4.42225 2.77519 2.73123 3.46354 2.40513 3.72494 3.29354 2.67741 2.69355 4.24690 2.90347 2.73739 3.18146 2.89801 2.37887 2.77519 2.98518 4.58477 3.61503

0.01769 4.43923 5.16158 0.61958 0.77255 0.86509 0.54648

843 2.54061 4.66309 3.26257 2.71817 1.95928 3.58706 3.80067 3.22944 2.35527 2.89225 3.75422 2.73592 3.97660 2.62648 3.07881 2.81934 2.93862 2.96527 5.16968 3.46098 1405 - -

2.68618 4.42225 2.77519 2.73123 3.46354 2.40513 3.72494 3.29354 2.67741 2.69355 4.24690 2.90347 2.73739 3.18146 2.89801 2.37887 2.77519 2.98518 4.58477 3.61503

0.03611 4.43923 3.74376 0.61958 0.77255 0.86509 0.54648

844 1.82390 4.68127 3.22065 2.66471 3.89479 2.92171 3.78201 3.28432 2.63680 2.55077 3.50813 3.14996 3.93958 2.78464 2.88584 2.33121 2.90887 3.00401 5.23803 3.94719 1406 - -

2.68618 4.42225 2.77519 2.73123 3.46354 2.40513 3.72494 3.29354 2.67741 2.69355 4.24690 2.90347 2.73739 3.18146 2.89801 2.37887 2.77519 2.98518 4.58477 3.61503

0.16413 4.42114 1.97080 0.61958 0.77255 0.88464 0.53249

845 2.99496 5.59095 1.47851 1.53350 4.88980 3.37475 3.82968 4.40562 2.25577 3.87597 4.68675 2.82538 3.94814 2.97438 3.31441 2.88984 3.26339 3.96406 6.01857 4.52992 1407 - -

2.68618 4.42225 2.77519 2.73123 3.46354 2.40513 3.72494 3.29354 2.67741 2.69355 4.24690 2.90347 2.73739 3.18146 2.89801 2.37887 2.77519 2.98518 4.58477 3.61503
0.02082 4.27784 5.00018 0.61958 0.77255 1.01708 0.44887
846 3.05858 4.71499 3.97473 3.39517 3.54572 3.92914 4.12838 2.88313 2.80393 1.08583 3.51264 3.73125 4.31482 3.43691 2.16266 3.30122 3.29330 2.82630 5.15981 3.92602 1408 - -
2.68618 4.42225 2.77519 2.73123 3.46354 2.40513 3.72494 3.29354 2.67741 2.69355 4.24690 2.90347 2.73739 3.18146 2.89801 2.37887 2.77519 2.98518 4.58477 3.61503
0.02082 4.27784 5.00018 0.61958 0.77255 0.63291 0.75724
847 2.61741 4.81049 2.74666 2.61891 3.98477 3.55753 3.79963 3.41377 2.55968 2.88513 3.90014 2.99256 3.97053 2.87155 3.08960 2.71626 2.98051 3.12639 5.31782 1.66494 1409 - -
2.68635 4.42248 2.77543 2.73059 3.46254 2.40505 3.72518 3.29332 2.67714 2.69356 4.24653 2.90370 2.73763 3.18141 2.89824 2.37902 2.77536 2.98525 4.58500 3.61527
0.32425 1.30428 5.19114 1.21601 0.35156 0.83148 0.57164
848 2.59639 4.58458 2.96157 2.21431 3.75276 3.60058 3.83102 2.26128 2.62688 2.36668 3.40356 3.23628 3.98489 2.94648 3.15529 2.82633 2.91878 2.46094 5.14247 3.87348 1415 - -
2.68618 4.42225 2.77519 2.73123 3.46354 2.40513 3.72494 3.29354 2.67741 2.69355 4.24690 2.90347 2.73739 3.18146 2.89801 2.37887 2.77519 2.98518 4.58477 3.61503
0.03686 4.46880 3.69972 0.61958 0.77255 0.59423 0.80294
849 2.72183 4.27567 3.94500 3.37257 1.66015 3.77243 4.06190 2.73558 3.10603 2.47565 3.39149 3.66386 4.15145 3.31221 3.25092 2.92264 2.70092 1.97804 4.87687 3.66824 1416 - -
2.68618 4.42225 2.77519 2.73123 3.46354 2.40513 3.72494 3.29354 2.67741 2.69355 4.24690 2.90347 2.73739 3.18146 2.89801 2.37887 2.77519 2.98518 4.58477 3.61503
0.01589 4.54595 5.26830 0.61958 0.77255 0.47027 0.98038
850 2.77735 5.23889 2.57641 2.46856 4.57023 2.80527 3.73526 4.04149 1.45904 3.53717 4.29013 3.01300 3.09589 2.75584 2.85636 2.74641 3.00932 3.26718 5.68130 4.28472 1417 - -
2.68620 4.42227 2.77522 2.73126 3.46356 2.40515 3.72497 3.29356 2.67743 2.69357 4.24692 2.90349 2.73726 3.18149 2.89803 2.37889 2.77511 2.98504 4.58479 3.61505
0.40969 2.32341 1.43463 0.28092 1.40687 0.57694 0.82466
851 2.70756 4.61016 3.24087 2.57712 3.81285 2.80860 3.89649 3.26705 2.80046 2.94050 3.81522 3.25179 3.99503 3.15690 3.17823 2.33396 2.97926 2.99308 1.75745 3.90801 1419 - -
2.68618 4.42225 2.77519 2.73123 3.46354 2.40513 3.72494 3.29354 2.67741 2.69355 4.24690 2.90347 2.73739 3.18146 2.89801 2.37887 2.77519 2.98518 4.58477 3.61503
0.01882 4.37809 5.10044 0.61958 0.77255 0.92836 0.50286
852 2.45051 4.39190 3.72225 3.41350 4.51976 3.17810 4.45866 3.90363 3.42624 3.62135 4.45813 3.55168 3.92692 3.73172 3.72997 1.29602 1.30028 3.37039 5.86934 4.65386 1420 - -
2.68618 4.42225 2.77519 2.73123 3.46354 2.40513 3.72494 3.29354 2.67741 2.69355 4.24690 2.90347 2.73739 3.18146 2.89801 2.37887 2.77519 2.98518 4.58477 3.61503
0.01882 4.37809 5.10044 0.61958 0.77255 0.92836 0.50286
853 2.38248 4.48102 3.39875 2.84383 3.14614 3.06075 3.86420 3.06845 2.81399 2.42081 3.63617 3.27563 3.44175 3.13261 3.20530 2.00623 2.33947 2.81306 5.10372 3.84868 1421 - -
2.68618 4.42225 2.77519 2.73123 3.46354 2.40513 3.72494 3.29354 2.67741 2.69355 4.24690 2.90347 2.73739 3.18146 2.89801 2.37887 2.77519 2.98518 4.58477 3.61503
0.01882 4.37809 5.10044 0.61958 0.77255 0.92836 0.50286
854 2.94634 3.81073 4.57757 3.98908 3.15912 4.09893 4.38186 2.47000 3.83075 1.08451 2.35486 4.14188 4.41770 3.99338 3.94252 3.40738 3.17497 2.42729 4.88179 3.01274 1422 - -
2.68618 4.42225 2.77519 2.73123 3.46354 2.40513 3.72494 3.29354 2.67741 2.69355 4.24690 2.90347 2.73739 3.18146 2.89801 2.37887 2.77519 2.98518 4.58477 3.61503
0.01882 4.37809 5.10044 0.61958 0.77255 0.92836 0.50286
855 3.51820 4.81018 5.24497 4.67032 1.69925 4.76029 4.91896 2.45271 4.52162 1.09031 1.95162 4.82771 4.91356 4.50951 4.54322 4.10813 3.72816 2.65030 5.10862 3.93034 1423 - -
2.68618 4.42225 2.77519 2.73123 3.46354 2.40513 3.72494 3.29354 2.67741 2.69355 4.24690 2.90347 2.73739 3.18146 2.89801 2.37887 2.77519 2.98518 4.58477 3.61503
0.01882 4.37809 5.10044 0.61958 0.77255 0.36887 1.17609
856 2.62894 4.36254 3.53821 3.25965 3.48471 3.78009 4.05180 1.72858 2.89306 1.92642 3.47116 3.60394 3.41183 3.46624 2.97449 3.04145 2.98339 2.34802 4.96062 3.74624 1424 - -
2.68618 4.42225 2.77519 2.73123 3.46354 2.40513 3.72494 3.29354 2.67741 2.69355 4.24690 2.90347 2.73739 3.18146 2.89801 2.37887 2.77519 2.98518 4.58477 3.61503
0.02903 4.64405 3.96351 0.61958 0.77255 0.57694 0.82466

857 2.74289 4.89440 3.17435 2.61579 4.12042 3.34409 3.52997 3.34532 2.44458 3.14693 3.56936 2.86239 1.75725 2.95139 2.87041 2.62675 2.97401 2.98712 5.40566 3.80480 1425 - -
2.68618 4.42225 2.77519 2.73123 3.46354 2.40513 3.72494 3.29354 2.67741 2.69355 4.24690 2.90347 2.73739 3.18146 2.89801 2.37887 2.77519 2.98518 4.58477 3.61503
0.02965 4.62963 3.93941 0.61958 0.77255 0.60244 0.79291
858 2.73438 4.58426 3.43445 2.87125 3.34081 2.94190 3.89999 2.97272 2.50516 2.44849 3.68049 3.07740 2.00420 3.16307 3.23820 2.89408 2.86121 2.59267 5.14799 3.59175 1426 - -
2.68618 4.42225 2.77519 2.73123 3.46354 2.40513 3.72494 3.29354 2.67741 2.69355 4.24690 2.90347 2.73739 3.18146 2.89801 2.37887 2.77519 2.98518 4.58477 3.61503
0.01482 4.61480 5.33714 0.61958 0.77255 0.58209 0.81810
859 2.74431 4.64796 3.38572 2.82210 3.81852 3.18838 3.88192 2.80555 2.35669 2.38040 3.38045 3.28831 4.03885 3.12090 2.83704 2.68143 1.81281 2.94037 5.20283 3.93373 1427 - -
2.68618 4.42225 2.77519 2.73123 3.46354 2.40513 3.72494 3.29354 2.67741 2.69355 4.24690 2.90347 2.73739 3.18146 2.89801 2.37887 2.77519 2.98518 4.58477 3.61503
0.01460 4.62963 5.35197 0.61958 0.77255 0.60244 0.79291
860 2.74427 4.00932 3.77475 3.20480 2.34839 3.75233 4.03616 2.86655 2.51575 2.51553 3.50670 3.56392 4.13772 3.42554 3.44662 2.86713 1.64595 2.51731 4.99153 3.77001 1428 - -
2.68618 4.42225 2.77519 2.73123 3.46354 2.40513 3.72494 3.29354 2.67741 2.69355 4.24690 2.90347 2.73739 3.18146 2.89801 2.37887 2.77519 2.98518 4.58477 3.61503
0.01460 4.62963 5.35197 0.61958 0.77255 0.55747 0.85016
861 2.74632 4.24743 4.12439 3.54065 3.35387 3.39386 3.71663 1.81455 3.43140 1.86311 3.36119 3.78125 3.35636 3.66658 3.17772 3.11477 2.63766 2.13447 4.85623 3.45830 1429 - -
2.68618 4.42225 2.77519 2.73123 3.46354 2.40513 3.72494 3.29354 2.67741 2.69355 4.24690 2.90347 2.73739 3.18146 2.89801 2.37887 2.77519 2.98518 4.58477 3.61503
0.01439 4.64405 5.36640 0.61958 0.77255 0.57694 0.82466
862 2.36204 4.77063 3.01030 2.41710 3.96492 3.28690 3.83618 2.74597 2.62111 2.26761 2.97970 2.60249 4.00564 2.83351 3.13159 2.83306 2.97542 2.78169 5.30576 4.00970 1430 - -
2.68618 4.42225 2.77519 2.73123 3.46354 2.40513 3.72494 3.29354 2.67741 2.69355 4.24690 2.90347 2.73739 3.18146 2.89801 2.37887 2.77519 2.98518 4.58477 3.61503
0.01439 4.64405 5.36640 0.61958 0.77255 0.57694 0.82466
863 2.82874 4.36046 4.10044 3.50531 3.43946 3.89343 4.15983 1.77244 2.98909 2.01997 2.36176 3.78555 4.25648 3.62716 2.23998 3.17428 3.05755 2.55767 4.96186 3.76909 1431 - -
2.68618 4.42225 2.77519 2.73123 3.46354 2.40513 3.72494 3.29354 2.67741 2.69355 4.24690 2.90347 2.73739 3.18146 2.89801 2.37887 2.77519 2.98518 4.58477 3.61503
0.01439 4.64405 5.36640 0.61958 0.77255 0.57694 0.82466
864 3.52839 4.75627 5.54221 5.02074 3.79298 5.02131 5.54756 1.46343 4.91993 1.59065 3.23588 5.16183 5.22687 5.05639 5.00893 4.41213 3.77165 1.00678 5.83550 4.70571 1432 - -
2.68618 4.42225 2.77519 2.73123 3.46354 2.40513 3.72494 3.29354 2.67741 2.69355 4.24690 2.90347 2.73739 3.18146 2.89801 2.37887 2.77519 2.98518 4.58477 3.61503
0.01439 4.64405 5.36640 0.61958 0.77255 0.57694 0.82466
865 2.95264 4.97822 3.37516 2.85918 3.00821 3.76934 3.31558 3.60117 2.81702 3.18300 4.07440 1.22133 4.16169 3.17568 3.02950 3.01036 3.18315 3.32431 4.92592 2.80384 1433 - -
2.68618 4.42225 2.77519 2.73123 3.46354 2.40513 3.72494 3.29354 2.67741 2.69355 4.24690 2.90347 2.73739 3.18146 2.89801 2.37887 2.77519 2.98518 4.58477 3.61503
0.01439 4.64405 5.36640 0.61958 0.77255 0.57694 0.82466
866 2.74625 4.18380 4.36773 3.77275 3.28231 3.87290 3.57901 2.12789 3.35372 1.54097 2.62426 3.91478 4.24027 3.81918 3.43850 2.83205 2.85306 2.26412 3.81111 3.61342 1434 - -
2.68618 4.42225 2.77519 2.73123 3.46354 2.40513 3.72494 3.29354 2.67741 2.69355 4.24690 2.90347 2.73739 3.18146 2.89801 2.37887 2.77519 2.98518 4.58477 3.61503
0.01439 4.64405 5.36640 0.61958 0.77255 0.57694 0.82466
867 2.37879 4.27509 4.07561 3.49438 3.08556 3.83624 4.12888 2.06507 2.46531 2.05652 3.38690 3.75839 4.20705 3.63874 3.20584 3.11361 2.83226 1.90847 4.88768 3.68865 1435 - -
2.68618 4.42225 2.77519 2.73123 3.46354 2.40513 3.72494 3.29354 2.67741 2.69355 4.24690 2.90347 2.73739 3.18146 2.89801 2.37887 2.77519 2.98518 4.58477 3.61503
0.01439 4.64405 5.36640 0.61958 0.77255 0.43270 1.04627
868 1.63161 3.85054 3.25264 1.64371 4.44934 2.23880 4.00248 3.86958 2.82486 3.47637 4.28101 3.25391 4.03220 3.16032 3.27719 2.81806 3.06700 3.47316 5.70293 4.37669 1436 - -
2.68618 4.42225 2.77519 2.73123 3.46354 2.40513 3.72494 3.29354 2.67741 2.69355 4.24690 2.90347 2.73739 3.18146 2.89801 2.37887 2.77519 2.98518 4.58477 3.61503

0.01372 4.69125 5.41360 0.61958 0.77255 0.48576 0.95510

869 3.48294 4.76164 5.44848 4.86696 2.50317 4.80184 5.15975 1.47212 4.70860 1.21600 2.93540 4.95609 5.01482 4.73962 4.71123 4.15330 3.70777 1.73718 5.42734 4.35047 1437 - -

2.68546 4.42216 2.77577 2.73181 3.46187 2.40481 3.72552 3.29307 2.67798 2.69180 4.24499 2.90404 2.73797 3.18204 2.89858 2.37938 2.77577 2.98471 4.58535 3.61413

0.43424 1.05617 5.41360 1.63628 0.21654 0.48576 0.95510

870 2.19987 3.43725 4.59872 3.99307 2.76130 3.66741 4.25441 2.26221 3.80791 1.98183 3.10643 4.04313 4.29152 3.96517 3.86498 2.96620 2.54010 1.71566 4.79086 3.61137 1445 - -

2.68618 4.42225 2.77519 2.73123 3.46354 2.40513 3.72494 3.29354 2.67741 2.69355 4.24690 2.90347 2.73739 3.18146 2.89801 2.37887 2.77519 2.98518 4.58477 3.61503

0.01372 4.69125 5.41360 0.61958 0.77255 0.48576 0.95510

871 1.84838 2.72751 4.54104 3.93924 2.99704 3.91950 4.24514 2.46657 3.76538 2.36683 3.29726 4.01630 4.28585 3.93371 3.54784 3.22275 3.00528 1.59269 3.71279 3.61975 1446 - -

2.68618 4.42225 2.77519 2.73123 3.46354 2.40513 3.72494 3.29354 2.67741 2.69355 4.24690 2.90347 2.73739 3.18146 2.89801 2.37887 2.77519 2.98518 4.58477 3.61503

0.01372 4.69125 5.41360 0.61958 0.77255 0.48576 0.95510

872 1.59999 4.71172 3.67028 3.27367 4.69080 1.13250 4.39334 4.12163 3.23574 3.75159 4.56076 3.57097 4.10971 3.60142 2.70087 2.63626 3.13572 3.61285 5.96856 4.71910 1447 - -

2.68618 4.42225 2.77519 2.73123 3.46354 2.40513 3.72494 3.29354 2.67741 2.69355 4.24690 2.90347 2.73739 3.18146 2.89801 2.37887 2.77519 2.98518 4.58477 3.61503

0.01372 4.69125 5.41360 0.61958 0.77255 0.48576 0.95510

873 2.51362 3.06689 4.67631 4.06726 2.68969 3.94850 4.28091 2.33917 3.86917 1.61850 3.28192 4.08719 4.31148 4.01442 3.90372 3.13770 2.85624 1.71594 4.79483 3.36336 1448 - -

2.68618 4.42225 2.77519 2.73123 3.46354 2.40513 3.72494 3.29354 2.67741 2.69355 4.24690 2.90347 2.73739 3.18146 2.89801 2.37887 2.77519 2.98518 4.58477 3.61503

0.01372 4.69125 5.41360 0.61958 0.77255 0.48576 0.95510

874 2.42901 3.95936 3.30303 2.74225 3.97235 3.64137 3.86073 3.13085 2.71695 3.01791 3.86617 3.23221 4.02919 2.41119 2.57509 2.00603 2.99671 3.08621 5.31643 2.43344 1449 - -

2.68618 4.42225 2.77519 2.73123 3.46354 2.40513 3.72494 3.29354 2.67741 2.69355 4.24690 2.90347 2.73739 3.18146 2.89801 2.37887 2.77519 2.98518 4.58477 3.61503

0.01372 4.69125 5.41360 0.61958 0.77255 0.48576 0.95510

875 2.79700 5.24254 2.07657 2.48665 4.56511 2.81477 3.76357 4.03519 2.51562 3.54157 4.29977 2.94695 3.96009 2.87257 1.88490 2.29227 3.03128 3.62425 5.69500 3.46321 1450 - -

2.68618 4.42225 2.77519 2.73123 3.46354 2.40513 3.72494 3.29354 2.67741 2.69355 4.24690 2.90347 2.73739 3.18146 2.89801 2.37887 2.77519 2.98518 4.58477 3.61503

0.04098 4.69125 3.47454 0.61958 0.77255 0.48576 0.95510

876 1.99943 4.25698 4.18063 3.59808 2.67098 3.85434 4.16748 2.38842 3.48645 1.88332 3.37231 3.82369 4.22990 3.71509 3.68405 2.51379 2.20092 2.49158 4.87634 3.68197 1451 - -

2.68618 4.42225 2.77519 2.73123 3.46354 2.40513 3.72494 3.29354 2.67741 2.69355 4.24690 2.90347 2.73739 3.18146 2.89801 2.37887 2.77519 2.98518 4.58477 3.61503

0.01410 4.66437 5.38672 0.61958 0.77255 0.53923 0.87515

877 2.65453 4.24470 4.67331 4.07647 2.74706 4.03741 4.37808 1.69415 3.90316 1.74743 3.33553 4.14602 4.39311 4.06621 3.97368 2.57313 3.09362 1.80860 4.91122 3.73201 1452 - -

2.68618 4.42225 2.77519 2.73123 3.46354 2.40513 3.72494 3.29354 2.67741 2.69355 4.24690 2.90347 2.73739 3.18146 2.89801 2.37887 2.77519 2.98518 4.58477 3.61503

0.01410 4.66437 5.38672 0.61958 0.77255 0.53923 0.87515

878 2.50147 4.08294 3.63169 3.06649 2.57831 3.73240 3.69139 2.98334 3.02120 2.69494 3.58738 2.54023 4.11468 3.32326 3.37522 2.29820 2.86113 2.75765 5.04934 2.15554 1453 - -

2.68618 4.42225 2.77519 2.73123 3.46354 2.40513 3.72494 3.29354 2.67741 2.69355 4.24690 2.90347 2.73739 3.18146 2.89801 2.37887 2.77519 2.98518 4.58477 3.61503

0.01410 4.66437 5.38672 0.61958 0.77255 0.53923 0.87515

879 2.39357 4.18682 3.16324 2.60840 4.19328 2.43948 3.79860 3.60954 2.51151 3.21480 4.02832 2.55910 3.97643 2.79174 3.06118 1.82264 2.98623 3.28498 4.08842 4.12755 1454 - -

2.68618 4.42225 2.77519 2.73123 3.46354 2.40513 3.72494 3.29354 2.67741 2.69355 4.24690 2.90347 2.73739 3.18146 2.89801 2.37887 2.77519 2.98518 4.58477 3.61503

0.27818 4.66437 1.45495 0.61958 0.77255 0.45373 1.00856

880 2.41215 4.95369 2.80782 2.48936 4.23279 2.14512 2.71847 3.66288 2.50055 3.24273 4.04125 3.01522 3.31248 2.85150 2.87088 2.70498 2.92615 2.86174 5.46253 4.11081 1455 - -

2.68618 4.42225 2.77519 2.73123 3.46354 2.40513 3.72494 3.29354 2.67741 2.69355 4.24690 2.90347 2.73739 3.18146 2.89801 2.37887 2.77519 2.98518 4.58477 3.61503
0.01769 4.43923 5.16158 0.61958 0.77255 0.86509 0.54648
881 2.67595 4.46433 3.45036 2.57098 3.12863 2.47261 3.86973 2.62566 2.85027 2.68071 3.56417 3.03111 4.00892 3.16465 3.09769 2.86437 2.90774 2.59570 5.03234 2.42072 1456 - -
2.68618 4.42225 2.77519 2.73123 3.46354 2.40513 3.72494 3.29354 2.67741 2.69355 4.24690 2.90347 2.73739 3.18146 2.89801 2.37887 2.77519 2.98518 4.58477 3.61503
0.01769 4.43923 5.16158 0.61958 0.77255 0.86509 0.54648
882 2.86821 5.36122 2.32681 2.15337 4.67915 1.95744 3.78929 4.16411 2.62264 3.66233 4.44083 2.92659 2.33066 2.42350 3.14190 2.81124 3.11832 3.74367 5.81752 4.38607 1457 - -
2.68618 4.42225 2.77519 2.73123 3.46354 2.40513 3.72494 3.29354 2.67741 2.69355 4.24690 2.90347 2.73739 3.18146 2.89801 2.37887 2.77519 2.98518 4.58477 3.61503
0.01769 4.43923 5.16158 0.61958 0.77255 0.86509 0.54648
883 2.59042 5.06216 2.75066 2.43852 4.37685 2.54331 3.69748 3.82793 2.46668 2.99244 4.14515 2.97513 3.58341 2.13026 2.94875 2.08202 2.93709 3.44352 5.55303 4.17576 1458 - -
2.68618 4.42225 2.77519 2.73123 3.46354 2.40513 3.72494 3.29354 2.67741 2.69355 4.24690 2.90347 2.73739 3.18146 2.89801 2.37887 2.77519 2.98518 4.58477 3.61503
0.11347 4.43923 2.34896 0.61958 0.77255 0.86509 0.54648
884 2.76842 4.30622 3.98394 3.42441 3.03075 3.03320 3.94666 2.75754 3.29647 2.28643 3.41240 3.67918 4.17561 3.56335 3.53101 3.08420 3.00445 2.34543 1.72247 2.92300 1459 - -
2.68618 4.42225 2.77519 2.73123 3.46354 2.40513 3.72494 3.29354 2.67741 2.69355 4.24690 2.90347 2.73739 3.18146 2.89801 2.37887 2.77519 2.98518 4.58477 3.61503
0.10344 4.34521 2.46156 0.61958 0.77255 0.74847 0.64073
885 2.63429 4.69874 3.10361 2.84012 4.55842 1.28919 4.11194 4.00527 2.59474 3.61445 4.43470 3.24164 3.94610 3.30735 3.34462 1.83233 3.03650 3.52228 5.81168 4.51806 1460 - -
2.68618 4.42225 2.77519 2.73123 3.46354 2.40513 3.72494 3.29354 2.67741 2.69355 4.24690 2.90347 2.73739 3.18146 2.89801 2.37887 2.77519 2.98518 4.58477 3.61503
0.01908 4.36460 5.08695 0.61958 0.77255 0.94131 0.49449
886 2.69696 5.00639 2.97135 2.48164 4.39426 3.44631 3.72398 3.83798 1.96253 3.38283 4.16819 2.75529 2.34042 2.85184 2.91377 2.09650 2.71956 3.44800 5.56374 4.20530 1461 - -
2.68618 4.42225 2.77519 2.73123 3.46354 2.40513 3.72494 3.29354 2.67741 2.69355 4.24690 2.90347 2.73739 3.18146 2.89801 2.37887 2.77519 2.98518 4.58477 3.61503
0.01908 4.36460 5.08695 0.61958 0.77255 0.94131 0.49449
887 2.38592 4.31067 3.91924 3.37697 3.43002 2.76375 4.12848 2.66563 3.27342 1.34119 3.00639 3.66741 4.14487 3.55909 3.53531 3.02659 2.97803 2.49501 4.98014 3.77940 1462 - -
2.68618 4.42225 2.77519 2.73123 3.46354 2.40513 3.72494 3.29354 2.67741 2.69355 4.24690 2.90347 2.73739 3.18146 2.89801 2.37887 2.77519 2.98518 4.58477 3.61503
0.01908 4.36460 5.08695 0.61958 0.77255 0.94131 0.49449
888 2.80530 4.19967 4.54521 3.94888 2.12702 3.36936 4.28992 2.14533 3.78692 1.56515 2.72222 4.05357 4.31023 3.94217 3.87190 3.27749 3.03589 2.10543 4.81170 3.64474 1463 - -
2.68618 4.42225 2.77519 2.73123 3.46354 2.40513 3.72494 3.29354 2.67741 2.69355 4.24690 2.90347 2.73739 3.18146 2.89801 2.37887 2.77519 2.98518 4.58477 3.61503
0.01908 4.36460 5.08695 0.61958 0.77255 0.76893 0.62270
889 2.04958 4.44898 3.62619 3.10960 3.77333 1.56570 4.06994 3.04546 3.05152 2.37468 3.20966 3.46269 4.04109 3.37428 3.39713 2.85619 2.95933 2.79527 5.22384 3.98717 1464 - -
2.68618 4.42225 2.77519 2.73123 3.46354 2.40513 3.72494 3.29354 2.67741 2.69355 4.24690 2.90347 2.73739 3.18146 2.89801 2.37887 2.77519 2.98518 4.58477 3.61503
0.01769 4.43923 5.16158 0.61958 0.77255 0.30559 1.33442
890 2.81182 5.30391 3.03642 1.92243 4.65493 2.44113 3.32374 4.13128 1.90968 3.60221 4.34732 2.84035 3.96411 2.35734 2.52405 2.77360 3.03994 3.69869 5.72361 4.32822 1465 - -
2.68618 4.42225 2.77519 2.73123 3.46354 2.40513 3.72494 3.29354 2.67741 2.69355 4.24690 2.90347 2.73739 3.18146 2.89801 2.37887 2.77519 2.98518 4.58477 3.61503
0.01372 4.69125 5.41360 0.61958 0.77255 0.48576 0.95510
891 2.23889 4.33275 3.95342 3.37770 3.14269 2.84183 4.10080 2.64241 3.29741 1.84714 3.44641 3.12541 4.19012 3.55920 3.56451 2.96173 2.26802 2.21659 4.93739 3.73046 1466 - -
2.68618 4.42225 2.77519 2.73123 3.46354 2.40513 3.72494 3.29354 2.67741 2.69355 4.24690 2.90347 2.73739 3.18146 2.89801 2.37887 2.77519 2.98518 4.58477 3.61503
0.01372 4.69125 5.41360 0.61958 0.77255 0.48576 0.95510

892 2.76639 2.74099 4.66478 4.05402 1.80137 3.92848 3.90777 2.38148 3.85314 2.14336 2.78777 3.73604 4.29273 3.99647 3.88428 3.23695 2.86751 2.11791 4.24718 3.18488 1467 - -

2.68618 4.42225 2.77519 2.73123 3.46354 2.40513 3.72494 3.29354 2.67741 2.69355 4.24690 2.90347 2.73739 3.18146 2.89801 2.37887 2.77519 2.98518 4.58477 3.61503

0.01372 4.69125 5.41360 0.61958 0.77255 0.48576 0.95510

893 2.71069 4.28667 4.22701 3.64645 1.75308 2.74877 2.91390 2.56341 3.52879 1.92172 3.39821 3.85736 4.26456 3.75059 3.71665 3.18140 3.03671 2.54215 4.80342 3.17057 1468 - -

2.68618 4.42225 2.77519 2.73123 3.46354 2.40513 3.72494 3.29354 2.67741 2.69355 4.24690 2.90347 2.73739 3.18146 2.89801 2.37887 2.77519 2.98518 4.58477 3.61503

0.01372 4.69125 5.41360 0.61958 0.77255 0.48576 0.95510

894 1.91735 2.78547 4.15853 3.57548 2.55198 3.85115 3.84569 2.71885 3.46659 2.46327 3.38090 3.80898 4.22538 3.69782 3.67002 2.32785 2.78801 2.41516 4.87347 2.69300 1469 - -

2.68618 4.42225 2.77519 2.73123 3.46354 2.40513 3.72494 3.29354 2.67741 2.69355 4.24690 2.90347 2.73739 3.18146 2.89801 2.37887 2.77519 2.98518 4.58477 3.61503

0.01372 4.69125 5.41360 0.61958 0.77255 0.48576 0.95510

895 2.50001 4.18026 4.51043 3.90955 2.08090 3.44570 4.23227 2.22921 3.74070 1.93683 3.29703 3.99798 4.27745 3.91281 3.82671 2.58076 3.00030 2.15797 4.79722 2.74017 1470 - -

2.68618 4.42225 2.77519 2.73123 3.46354 2.40513 3.72494 3.29354 2.67741 2.69355 4.24690 2.90347 2.73739 3.18146 2.89801 2.37887 2.77519 2.98518 4.58477 3.61503

0.01372 4.69125 5.41360 0.61958 0.77255 0.48576 0.95510

896 3.15561 4.50571 4.89743 4.35755 1.95449 4.25842 4.16193 2.89303 4.16571 2.09313 2.89678 4.29003 4.59761 4.24357 4.18321 3.57712 3.38359 2.53119 1.70163 2.05523 1471 - -

2.68618 4.42225 2.77519 2.73123 3.46354 2.40513 3.72494 3.29354 2.67741 2.69355 4.24690 2.90347 2.73739 3.18146 2.89801 2.37887 2.77519 2.98518 4.58477 3.61503

0.01372 4.69125 5.41360 0.61958 0.77255 0.48576 0.95510

897 2.69878 4.64239 5.25463 4.71378 3.78000 4.72290 5.20428 1.90179 4.59044 1.43161 3.61148 4.84288 5.00864 4.77254 4.70172 4.08318 3.60045 1.03539 5.64249 4.47491 1472 - -

2.68618 4.42225 2.77519 2.73123 3.46354 2.40513 3.72494 3.29354 2.67741 2.69355 4.24690 2.90347 2.73739 3.18146 2.89801 2.37887 2.77519 2.98518 4.58477 3.61503

0.01372 4.69125 5.41360 0.61958 0.77255 0.48576 0.95510

898 3.22008 4.54678 5.15436 4.55724 2.38882 4.45888 4.79339 1.81336 4.37579 1.29603 3.05623 4.60700 4.74237 4.45465 4.38802 3.78823 3.44736 1.61693 5.16997 3.73757 1473 - -

2.68618 4.42225 2.77519 2.73123 3.46354 2.40513 3.72494 3.29354 2.67741 2.69355 4.24690 2.90347 2.73739 3.18146 2.89801 2.37887 2.77519 2.98518 4.58477 3.61503

0.01372 4.69125 5.41360 0.61958 0.77255 0.48576 0.95510

899 2.28166 3.42882 4.71545 4.10707 2.26207 3.98117 4.31645 2.33880 3.90821 1.89461 2.61318 4.12367 4.34099 4.05048 3.93929 3.29194 2.83442 1.78636 4.82286 3.64686 1474 - -

2.68618 4.42225 2.77519 2.73123 3.46354 2.40513 3.72494 3.29354 2.67741 2.69355 4.24690 2.90347 2.73739 3.18146 2.89801 2.37887 2.77519 2.98518 4.58477 3.61503

0.01372 4.69125 5.41360 0.61958 0.77255 0.48576 0.95510

900 2.79184 5.05419 3.13250 2.58678 4.30776 3.11156 2.00722 3.37607 2.55333 2.78082 4.12241 2.72904 3.99016 1.99500 3.00603 2.80701 3.02229 3.40137 5.53223 4.18342 1475 - -

2.68618 4.42225 2.77519 2.73123 3.46354 2.40513 3.72494 3.29354 2.67741 2.69355 4.24690 2.90347 2.73739 3.18146 2.89801 2.37887 2.77519 2.98518 4.58477 3.61503

0.01372 4.69125 5.41360 0.61958 0.77255 0.48576 0.95510

901 2.40554 3.33057 3.97475 2.95638 2.80895 2.84559 4.10387 2.78571 3.31422 1.71555 2.91859 3.31969 4.19339 3.57250 3.57406 2.72682 2.87431 2.58185 4.92464 3.71929 1476 - -

2.68618 4.42225 2.77519 2.73123 3.46354 2.40513 3.72494 3.29354 2.67741 2.69355 4.24690 2.90347 2.73739 3.18146 2.89801 2.37887 2.77519 2.98518 4.58477 3.61503

0.01372 4.69125 5.41360 0.61958 0.77255 0.48576 0.95510

902 2.76808 3.83355 4.57657 3.97163 3.11681 3.91978 4.24340 2.25703 3.78956 1.82476 3.11898 3.29307 4.28478 3.94943 3.85230 3.22417 2.79155 2.22015 3.89896 1.87183 1477 - -

2.68618 4.42225 2.77519 2.73123 3.46354 2.40513 3.72494 3.29354 2.67741 2.69355 4.24690 2.90347 2.73739 3.18146 2.89801 2.37887 2.77519 2.98518 4.58477 3.61503

0.06159 4.69125 2.98472 0.61958 0.77255 0.48576 0.95510

903 1.80854 4.54323 3.97215 3.62142 4.74831 3.33082 4.65173 4.16872 3.63904 3.84516 4.65215 3.73559 1.01040 3.91641 3.95550 2.25704 2.47575 3.58894 6.08124 4.88273 1478 - -

2.68618 4.42225 2.77519 2.73123 3.46354 2.40513 3.72494 3.29354 2.67741 2.69355 4.24690 2.90347 2.73739 3.18146 2.89801 2.37887 2.77519 2.98518 4.58477 3.61503

0.01439 4.64405 5.36640 0.61958 0.77255 0.57694 0.82466
904 3.81282 5.06529 5.42687 4.96038 0.75541 4.97279 4.67781 2.23292 4.79221 1.84838 3.41385 4.93833 5.16989 4.76659 4.79814 4.34843 4.03586 2.78034 4.81433 3.27274 1479 - -
2.68618 4.42225 2.77519 2.73123 3.46354 2.40513 3.72494 3.29354 2.67741 2.69355 4.24690 2.90347 2.73739 3.18146 2.89801 2.37887 2.77519 2.98518 4.58477 3.61503
0.01439 4.64405 5.36640 0.61958 0.77255 0.57694 0.82466
905 2.08206 4.27661 4.57112 3.97774 2.48532 4.01344 4.30025 2.53096 3.81710 1.35036 3.28240 4.08202 4.36490 3.98122 3.91090 3.31697 3.09996 2.35529 4.82936 2.70721 1480 - -
2.68620 4.42245 2.77540 2.73143 3.46374 2.40458 3.72515 3.29301 2.67761 2.69353 4.24710 2.90367 2.73760 3.18166 2.89821 2.37888 2.77540 2.98414 4.58497 3.61523
0.28478 1.41407 5.36640 1.02484 0.44450 0.57694 0.82466
906 2.75327 4.63913 3.41190 2.64403 3.23230 3.38375 3.89847 3.06759 1.56801 2.31871 3.50916 3.31050 4.05310 3.14413 3.21290 2.89706 2.98444 2.55792 5.19699 3.93103 1484 - -
2.68618 4.42225 2.77519 2.73123 3.46354 2.40513 3.72494 3.29354 2.67741 2.69355 4.24690 2.90347 2.73739 3.18146 2.89801 2.37887 2.77519 2.98518 4.58477 3.61503
0.01439 4.64405 5.36640 0.61958 0.77255 0.57694 0.82466
907 2.39331 5.16260 2.09453 2.78685 5.06763 0.86736 4.34585 4.54845 3.38270 4.13380 4.96390 3.27793 4.17297 3.54100 3.90507 3.02639 3.39895 4.01046 6.31021 4.92526 1485 - -
2.68618 4.42225 2.77519 2.73123 3.46354 2.40513 3.72494 3.29354 2.67741 2.69355 4.24690 2.90347 2.73739 3.18146 2.89801 2.37887 2.77519 2.98518 4.58477 3.61503
0.01439 4.64405 5.36640 0.61958 0.77255 0.57694 0.82466
908 2.97549 4.35571 4.75843 4.16527 2.54640 4.12950 4.36103 2.64088 3.98551 1.09827 2.28972 4.22059 4.46297 3.74841 4.03781 3.44007 3.20368 2.55206 4.11224 3.08302 1486 - -
2.68618 4.42225 2.77519 2.73123 3.46354 2.40513 3.72494 3.29354 2.67741 2.69355 4.24690 2.90347 2.73739 3.18146 2.89801 2.37887 2.77519 2.98518 4.58477 3.61503
0.01439 4.64405 5.36640 0.61958 0.77255 0.57694 0.82466
909 3.37019 4.67042 5.31008 4.71932 2.58051 4.64850 4.99012 1.72843 4.55130 1.51540 1.76212 4.79768 4.88575 4.59507 4.55647 3.98931 3.59440 1.78078 5.29691 4.21010 1487 - -
2.68618 4.42225 2.77519 2.73123 3.46354 2.40513 3.72494 3.29354 2.67741 2.69355 4.24690 2.90347 2.73739 3.18146 2.89801 2.37887 2.77519 2.98518 4.58477 3.61503
0.01439 4.64405 5.36640 0.61958 0.77255 0.43270 1.04627
910 2.82463 5.24490 2.55156 2.33787 4.52102 1.29502 3.91749 3.95631 2.65228 2.67604 4.34652 3.10291 4.06899 3.06724 3.24945 2.93111 3.19091 3.60313 5.75094 4.37550 1488 - -
2.68618 4.42225 2.77519 2.73123 3.46354 2.40513 3.72494 3.29354 2.67741 2.69355 4.24690 2.90347 2.73739 3.18146 2.89801 2.37887 2.77519 2.98518 4.58477 3.61503
0.01372 4.69125 5.41360 0.61958 0.77255 0.48576 0.95510
911 2.82101 5.25424 3.09996 2.18080 4.59074 3.10460 3.55253 4.04904 2.01331 3.54253 3.89484 3.06894 3.98039 2.85998 1.70201 2.79434 2.47321 3.64153 5.68003 4.30607 1489 - -
2.68618 4.42225 2.77519 2.73123 3.46354 2.40513 3.72494 3.29354 2.67741 2.69355 4.24690 2.90347 2.73739 3.18146 2.89801 2.37887 2.77519 2.98518 4.58477 3.61503
0.01450 4.63640 5.35874 0.61958 0.77255 0.48576 0.95510
912 2.74563 5.04855 3.08777 2.53020 3.98487 2.74692 3.74959 3.19988 2.42497 3.32048 4.11044 3.06050 3.65345 1.86123 2.38704 2.52118 2.97649 3.39843 5.52818 3.60410 1490 - -
2.68630 4.42237 2.77463 2.73114 3.46366 2.40525 3.72506 3.29366 2.67709 2.69367 4.24702 2.90359 2.73752 3.18125 2.89813 2.37899 2.77532 2.98530 4.58489 3.61436
0.51872 1.50929 1.69472 0.67859 0.70792 0.48576 0.95510
913 2.98765 5.38172 2.14745 2.41760 4.90037 1.29939 4.00019 4.40660 2.97433 3.92634 4.74806 2.04926 4.02881 3.16310 3.53202 2.76505 3.30893 3.94519 6.09424 4.63924 1493 - -
2.68618 4.42225 2.77519 2.73123 3.46354 2.40513 3.72494 3.29354 2.67741 2.69355 4.24690 2.90347 2.73739 3.18146 2.89801 2.37887 2.77519 2.98518 4.58477 3.61503
0.01769 4.43923 5.16158 0.61958 0.77255 0.78644 0.60782
914 2.88827 4.99858 3.37471 2.75022 4.27421 3.67912 3.76636 2.91281 1.66843 2.75594 4.07067 3.21907 4.04883 2.91992 1.71185 2.93074 2.93525 3.33602 5.43852 4.17661 1494 - -
2.68618 4.42225 2.77519 2.73123 3.46354 2.40513 3.72494 3.29354 2.67741 2.69355 4.24690 2.90347 2.73739 3.18146 2.89801 2.37887 2.77519 2.98518 4.58477 3.61503
0.01769 4.43923 5.16158 0.61958 0.77255 0.33527 1.25577
915 2.75762 4.32795 3.93149 2.91786 3.44572 3.80900 4.09187 2.63023 3.27668 2.26241 2.63323 3.67179 3.11694 3.54229 3.54859 3.07696 2.05599 1.82944 4.93741 3.72959 1495 - -

2.68618 4.42225 2.77519 2.73123 3.46354 2.40513 3.72494 3.29354 2.67741 2.69355 4.24690 2.90347 2.73739 3.18146 2.89801 2.37887 2.77519 2.98518 4.58477 3.61503
0.01450 4.63640 5.35874 0.61958 0.77255 0.48576 0.95510
916 2.75705 4.55672 3.51718 2.81709 3.49196 3.69564 3.94590 3.08017 2.91497 2.31732 3.66297 3.39312 1.62701 3.23926 3.29077 2.40652 2.99278 2.84389 4.47812 3.85198 1496 - -
2.68618 4.42225 2.77519 2.73123 3.46354 2.40513 3.72494 3.29354 2.67741 2.69355 4.24690 2.90347 2.73739 3.18146 2.89801 2.37887 2.77519 2.98518 4.58477 3.61503
0.01604 4.53668 5.25903 0.61958 0.77255 0.48576 0.95510
917 2.61246 4.86666 3.15134 2.60337 4.14397 3.53483 3.78282 3.55543 2.32262 3.16825 3.50501 2.98926 3.26929 2.94158 3.03596 2.24815 1.71306 3.23194 5.41922 4.09534 1497 - -
2.68618 4.42225 2.77519 2.73123 3.46354 2.40513 3.72494 3.29354 2.67741 2.69355 4.24690 2.90347 2.73739 3.18146 2.89801 2.37887 2.77519 2.98518 4.58477 3.61503
0.01635 4.51777 5.24012 0.61958 0.77255 0.48576 0.95510
918 2.71359 4.48348 3.51904 2.94998 3.63271 3.66791 3.65102 1.80703 2.57765 2.69968 3.58608 3.37276 4.04925 3.20884 2.76529 2.61407 2.41474 2.50857 5.05757 3.81626 1498 - -
2.68618 4.42225 2.77519 2.73123 3.46354 2.40513 3.72494 3.29354 2.67741 2.69355 4.24690 2.90347 2.73739 3.18146 2.89801 2.37887 2.77519 2.98518 4.58477 3.61503
0.10834 4.48301 2.39273 0.61958 0.77255 0.48576 0.95510
919 2.80348 4.24702 4.33202 3.76165 3.41482 3.95070 4.32096 1.97137 3.63977 2.13455 3.37743 3.96336 3.76332 3.87308 3.82347 3.04604 2.82329 1.25778 4.99159 3.79632 1499 - -
2.68629 4.42236 2.77531 2.73134 3.46365 2.40508 3.72505 3.29327 2.67731 2.69366 4.24701 2.90332 2.73751 3.18157 2.89786 2.37853 2.77531 2.98529 4.58488 3.61514
0.13619 2.59741 2.94022 1.56200 0.23536 0.40980 1.09000
920 2.70303 3.13332 4.56711 3.96103 2.62241 3.86888 4.19852 2.02157 3.77031 1.87863 3.18944 3.99675 4.22943 3.91971 3.81554 3.17743 2.70742 1.94990 4.71688 2.69507 1508 - -
2.68618 4.42225 2.77519 2.73123 3.46354 2.40513 3.72494 3.29354 2.67741 2.69355 4.24690 2.90347 2.73739 3.18146 2.89801 2.37887 2.77519 2.98518 4.58477 3.61503
0.01769 4.43923 5.16158 0.61958 0.77255 0.55530 0.85308
921 2.83182 4.25560 4.42564 3.84854 2.72436 3.98723 4.32489 2.25566 3.71699 2.08414 3.32695 4.01844 4.34655 3.92389 3.86953 2.59655 3.07074 1.26223 4.93181 3.72505 1509 - -
2.68618 4.42225 2.77519 2.73123 3.46354 2.40513 3.72494 3.29354 2.67741 2.69355 4.24690 2.90347 2.73739 3.18146 2.89801 2.37887 2.77519 2.98518 4.58477 3.61503
0.01769 4.43923 5.16158 0.61958 0.77255 0.55530 0.85308
922 2.72453 4.53695 3.46510 2.57661 3.66563 3.65316 3.86435 2.91113 2.29462 2.49433 3.62730 3.32602 4.03253 3.14096 3.10176 2.89730 2.95241 2.69604 1.95984 3.81797 1510 - -
2.68618 4.42225 2.77519 2.73123 3.46354 2.40513 3.72494 3.29354 2.67741 2.69355 4.24690 2.90347 2.73739 3.18146 2.89801 2.37887 2.77519 2.98518 4.58477 3.61503
0.01769 4.43923 5.16158 0.61958 0.77255 0.55530 0.85308
923 1.75125 4.68659 3.28549 2.87831 4.41844 3.36271 4.05556 3.82857 2.43024 3.45848 4.27889 3.27868 3.95882 3.24156 3.25712 1.30489 2.99903 3.39951 5.68668 4.40007 1511 - -
2.68618 4.42225 2.77519 2.73123 3.46354 2.40513 3.72494 3.29354 2.67741 2.69355 4.24690 2.90347 2.73739 3.18146 2.89801 2.37887 2.77519 2.98518 4.58477 3.61503
0.01769 4.43923 5.16158 0.61958 0.77255 0.55530 0.85308
924 2.29724 4.32951 3.87560 3.33627 3.61109 1.87515 4.15548 2.14397 3.27080 2.64234 3.57485 3.62764 4.11418 3.55064 3.56638 2.95482 2.56152 2.08839 5.10329 3.89003 1512 - -
2.68618 4.42225 2.77519 2.73123 3.46354 2.40513 3.72494 3.29354 2.67741 2.69355 4.24690 2.90347 2.73739 3.18146 2.89801 2.37887 2.77519 2.98518 4.58477 3.61503
0.01769 4.43923 5.16158 0.61958 0.77255 0.44461 1.02464
925 2.83108 4.24359 4.48567 3.89770 2.27498 3.97005 4.23164 2.43876 3.74290 1.27716 3.23315 3.52944 4.31866 3.91330 3.85046 3.27259 3.06181 2.17162 4.75721 3.25595 1513 - -
2.68618 4.42225 2.77519 2.73123 3.46354 2.40513 3.72494 3.29354 2.67741 2.69355 4.24690 2.90347 2.73739 3.18146 2.89801 2.37887 2.77519 2.98518 4.58477 3.61503
0.01693 4.48301 5.20535 0.61958 0.77255 0.48576 0.95510
926 3.23391 4.59327 4.88047 4.30524 3.34194 4.45546 4.78548 2.03629 4.14820 0.93780 3.18278 4.49760 4.72342 3.68285 4.26514 3.78266 3.46246 1.83394 5.22868 4.11582 1514 - -
2.68618 4.42225 2.77519 2.73123 3.46354 2.40513 3.72494 3.29354 2.67741 2.69355 4.24690 2.90347 2.73739 3.18146 2.89801 2.37887 2.77519 2.98518 4.58477 3.61503
0.01693 4.48301 5.20535 0.61958 0.77255 0.48576 0.95510

927 1.10933 4.56371 3.48149 3.04213 4.25342 3.36734 3.49397 3.66008 3.04395 3.32299 4.16053 3.38891 3.97800 3.37258 3.42669 2.12493 2.48435 3.26295 5.59406 4.33339 1515 - -
2.68618 4.42225 2.77519 2.73123 3.46354 2.40513 3.72494 3.29354 2.67741 2.69355 4.24690 2.90347 2.73739 3.18146 2.89801 2.37887 2.77519 2.98518 4.58477 3.61503
0.01729 4.46237 5.18472 0.61958 0.77255 0.48576 0.95510
928 2.63022 3.27400 4.37588 3.78509 2.90825 3.86416 4.19670 1.86643 3.63834 2.18940 3.26787 3.91976 4.23158 3.83088 3.76239 2.06937 2.95835 1.96269 4.79902 3.61275 1516 - -
2.68618 4.42225 2.77519 2.73123 3.46354 2.40513 3.72494 3.29354 2.67741 2.69355 4.24690 2.90347 2.73739 3.18146 2.89801 2.37887 2.77519 2.98518 4.58477 3.61503
0.01729 4.46237 5.18472 0.61958 0.77255 0.48576 0.95510
929 2.68660 3.55469 4.13005 3.29319 3.28305 3.42107 4.09677 1.72840 3.42961 2.12638 3.29007 3.76209 4.15831 3.65516 3.61886 2.72414 2.42566 2.15767 4.79304 3.59973 1517 - -
2.68618 4.42225 2.77519 2.73123 3.46354 2.40513 3.72494 3.29354 2.67741 2.69355 4.24690 2.90347 2.73739 3.18146 2.89801 2.37887 2.77519 2.98518 4.58477 3.61503
0.01769 4.43923 5.16158 0.61958 0.77255 0.48576 0.95510
930 2.58074 4.19551 4.52962 3.93693 2.03323 3.96155 4.29087 2.14894 3.77731 1.63466 3.22498 4.04276 4.31298 3.94726 3.87177 3.00795 3.03022 1.97719 4.83614 3.65528 1518 - -
2.68625 4.42105 2.77526 2.73130 3.46360 2.40513 3.72501 3.29347 2.67747 2.69362 4.24696 2.90353 2.73746 3.18153 2.89807 2.37882 2.77504 2.98502 4.58484 3.61485
0.32156 2.11859 1.86576 0.85827 0.55146 0.48576 0.95510
931 2.48913 3.47835 3.41277 2.90842 4.06773 2.81017 3.98464 3.46355 2.64125 3.12871 3.96778 3.27029 3.87052 3.20868 3.26432 1.41645 2.38785 3.09233 5.41228 4.15260 1522 - -
2.68618 4.42225 2.77519 2.73123 3.46354 2.40513 3.72494 3.29354 2.67741 2.69355 4.24690 2.90347 2.73739 3.18146 2.89801 2.37887 2.77519 2.98518 4.58477 3.61503
0.02082 4.27784 5.00018 0.61958 0.77255 0.70078 0.68557
932 3.33929 4.74421 4.57504 4.09893 2.89967 4.37120 4.36505 2.58863 3.87327 0.78196 3.21271 4.29275 4.67042 4.11472 4.02178 3.74356 3.57319 2.66526 4.71970 2.69777 1523 - -
2.68618 4.42225 2.77519 2.73123 3.46354 2.40513 3.72494 3.29354 2.67741 2.69355 4.24690 2.90347 2.73739 3.18146 2.89801 2.37887 2.77519 2.98518 4.58477 3.61503
0.02082 4.27784 5.00018 0.61958 0.77255 0.37154 1.17012
933 2.59885 4.28824 4.31548 3.74171 3.09581 3.94711 4.29230 2.45111 3.62209 1.34494 3.28282 3.95101 3.26631 3.83861 3.80191 3.24938 3.06842 1.77987 4.94197 3.75991 1524 - -
2.68618 4.42225 2.77519 2.73123 3.46354 2.40513 3.72494 3.29354 2.67741 2.69355 4.24690 2.90347 2.73739 3.18146 2.89801 2.37887 2.77519 2.98518 4.58477 3.61503
0.01769 4.43923 5.16158 0.61958 0.77255 0.48576 0.95510
934 3.03206 4.43385 4.50564 3.97333 1.89411 4.08329 3.93347 2.88182 3.81444 2.52701 3.53395 4.01741 4.43291 3.95983 3.37805 3.38992 3.26069 2.27332 1.64547 2.46529 1525 - -
2.68618 4.42225 2.77519 2.73123 3.46354 2.40513 3.72494 3.29354 2.67741 2.69355 4.24690 2.90347 2.73739 3.18146 2.89801 2.37887 2.77519 2.98518 4.58477 3.61503
0.01769 4.43923 5.16158 0.61958 0.77255 0.48576 0.95510
935 2.83706 4.22912 4.55652 3.96965 2.71149 4.02069 4.36379 2.05312 3.81965 1.91489 3.28130 4.09214 3.71540 4.00083 3.92880 3.33014 2.82351 1.34729 4.92178 3.74182 1526 - -
2.68618 4.42225 2.77519 2.73123 3.46354 2.40513 3.72494 3.29354 2.67741 2.69355 4.24690 2.90347 2.73739 3.18146 2.89801 2.37887 2.77519 2.98518 4.58477 3.61503
0.01837 4.40207 5.12442 0.61958 0.77255 0.48576 0.95510
936 2.42024 4.32440 3.08662 2.52550 4.12051 3.50810 3.70648 3.53527 2.33293 3.13633 3.95026 3.04150 3.89800 2.71403 2.00142 2.42298 2.57485 3.21248 5.37666 3.39872 1527 - -
2.68618 4.42225 2.77519 2.73123 3.46354 2.40513 3.72494 3.29354 2.67741 2.69355 4.24690 2.90347 2.73739 3.18146 2.89801 2.37887 2.77519 2.98518 4.58477 3.61503
0.01925 4.35558 5.07792 0.61958 0.77255 0.48576 0.95510
937 2.95736 4.38228 4.42972 3.91015 3.61352 4.13842 4.58686 1.25029 3.79773 2.42841 3.52750 3.09394 4.51578 4.07610 4.01453 3.47484 3.21644 1.42884 5.28796 4.06770 1528 - -
2.68618 4.42225 2.77519 2.73123 3.46354 2.40513 3.72494 3.29354 2.67741 2.69355 4.24690 2.90347 2.73739 3.18146 2.89801 2.37887 2.77519 2.98518 4.58477 3.61503
0.01968 4.33388 5.05622 0.61958 0.77255 0.48576 0.95510
938 2.72820 5.19778 2.41259 2.34558 4.51899 2.84708 3.06071 3.99495 2.31072 3.49570 4.25820 1.93669 3.86134 2.79113 2.95599 2.52891 2.78567 3.57841 5.64791 4.23772 1529 - -
2.68618 4.42225 2.77519 2.73123 3.46354 2.40513 3.72494 3.29354 2.67741 2.69355 4.24690 2.90347 2.73739 3.18146 2.89801 2.37887 2.77519 2.98518 4.58477 3.61503

0.01968 4.33388 5.05622 0.61958 0.77255 0.48576 0.95510

939 2.78920 5.05911 2.85687 2.50526 4.48459 3.44335 3.79826 3.92752 2.15780 3.47591 4.28512 2.40101 1.69060 2.94326 2.95113 2.59720 3.06018 3.53945 5.65195 4.30225 1530 - -

2.68618 4.42225 2.77519 2.73123 3.46354 2.40513 3.72494 3.29354 2.67741 2.69355 4.24690 2.90347 2.73739 3.18146 2.89801 2.37887 2.77519 2.98518 4.58477 3.61503

0.02136 4.25292 4.97526 0.61958 0.77255 0.48576 0.95510

940 2.60518 4.32357 3.54501 3.01087 2.17557 3.55365 3.90488 2.86757 2.96647 2.58973 3.49076 3.06176 2.55607 3.26548 3.30282 2.60735 2.86606 2.34545 4.92885 3.66785 1531 - -

2.68618 4.42225 2.77519 2.73123 3.46354 2.40513 3.72494 3.29354 2.67741 2.69355 4.24690 2.90347 2.73739 3.18146 2.89801 2.37887 2.77519 2.98518 4.58477 3.61503

0.02136 4.25292 4.97526 0.61958 0.77255 0.48576 0.95510

941 2.48439 4.70808 2.87473 2.40008 3.92185 3.47924 3.70529 3.31540 2.54598 2.63186 3.79617 2.70043 3.87480 2.88657 2.99286 2.47421 2.59495 2.31755 5.24133 3.93318 1532 - -

2.68618 4.42225 2.77519 2.73123 3.46354 2.40513 3.72494 3.29354 2.67741 2.69355 4.24690 2.90347 2.73739 3.18146 2.89801 2.37887 2.77519 2.98518 4.58477 3.61503

0.02136 4.25292 4.97526 0.61958 0.77255 0.48576 0.95510

942 2.44294 4.97603 2.66325 2.20471 4.26096 2.67042 3.62997 3.31442 2.39811 3.10131 4.03799 2.92673 3.44337 2.75267 2.73044 2.32033 2.86526 3.33483 5.45170 4.08190 1533 - -

2.68618 4.42225 2.77519 2.73123 3.46354 2.40513 3.72494 3.29354 2.67741 2.69355 4.24690 2.90347 2.73739 3.18146 2.89801 2.37887 2.77519 2.98518 4.58477 3.61503

0.02136 4.25292 4.97526 0.61958 0.77255 0.48576 0.95510

943 2.55317 4.97415 2.74850 2.40428 4.26238 3.43488 3.63185 3.70282 1.75616 3.04477 4.03843 2.93660 3.44696 2.75520 2.74312 2.49738 2.76156 3.07834 5.44886 4.08433 1534 - -

2.68618 4.42225 2.77519 2.73123 3.46354 2.40513 3.72494 3.29354 2.67741 2.69355 4.24690 2.90347 2.73739 3.18146 2.89801 2.37887 2.77519 2.98518 4.58477 3.61503

0.15487 4.25292 2.04600 0.61958 0.77255 0.48576 0.95510

944 2.46574 4.76857 3.01867 2.46698 4.01261 3.43194 3.64352 3.16004 2.22343 3.03319 3.85355 2.85124 3.82714 2.41439 2.87883 2.49983 2.37808 2.95418 5.28366 3.95977 1535 - -

2.68618 4.42225 2.77519 2.73123 3.46354 2.40513 3.72494 3.29354 2.67741 2.69355 4.24690 2.90347 2.73739 3.18146 2.89801 2.37887 2.77519 2.98518 4.58477 3.61503

0.02437 4.12242 4.84476 0.61958 0.77255 0.48133 0.96224

945 2.51021 5.10393 2.38138 2.32292 4.42045 2.68441 3.30794 3.89005 2.30812 3.39820 4.15860 2.56180 3.53803 2.56448 2.87520 2.62370 2.60440 3.48044 5.55522 4.15486 1536 - -

2.68618 4.42225 2.77519 2.73123 3.46354 2.40513 3.72494 3.29354 2.67741 2.69355 4.24690 2.90347 2.73739 3.18146 2.89801 2.37887 2.77519 2.98518 4.58477 3.61503

0.14999 4.20720 2.08431 0.61958 0.77255 0.54375 0.86885

946 2.39276 4.55161 3.11730 2.60155 3.84582 2.39972 3.73131 3.24000 2.60090 2.89730 3.74477 2.71342 3.83326 2.93685 3.02693 2.47667 2.66032 2.79406 5.19059 3.18820 1537 - -

2.68618 4.42225 2.77519 2.73123 3.46354 2.40513 3.72494 3.29354 2.67741 2.69355 4.24690 2.90347 2.73739 3.18146 2.89801 2.37887 2.77519 2.98518 4.58477 3.61503

0.15559 4.08258 2.06181 0.61958 0.77255 0.53150 0.88606

947 2.34318 4.66617 3.03294 2.50575 3.92586 3.40285 3.02710 3.32941 2.48204 2.96170 3.49619 2.80375 2.58883 2.84303 2.91528 2.64297 2.67636 3.02377 5.22989 3.92063 1538 - -

2.68618 4.42225 2.77519 2.73123 3.46354 2.40513 3.72494 3.29354 2.67741 2.69355 4.24690 2.90347 2.73739 3.18146 2.89801 2.37887 2.77519 2.98518 4.58477 3.61503

0.05711 4.04767 3.26897 0.61958 0.77255 0.70720 0.67929

948 2.57659 4.76446 2.71124 2.30029 3.99862 3.40049 3.04561 3.12817 2.41585 2.81703 3.83828 2.76597 3.79528 2.76576 2.87623 2.42248 2.80869 2.86883 5.26917 3.93621 1539 - -

2.68618 4.42225 2.77519 2.73123 3.46354 2.40513 3.72494 3.29354 2.67741 2.69355 4.24690 2.90347 2.73739 3.18146 2.89801 2.37887 2.77519 2.98518 4.58477 3.61503

0.12459 4.01766 2.31113 0.61958 0.77255 0.68169 0.70474

949 2.53811 4.33762 3.32544 2.80051 3.54744 3.08160 3.78998 2.69475 2.73703 2.05267 3.50054 2.84144 3.88031 3.07990 3.09643 2.72848 2.49178 2.60643 4.97687 3.72672 1540 - -

2.68623 4.42230 2.77524 2.73105 3.46359 2.40518 3.72499 3.29359 2.67735 2.69352 4.24694 2.90343 2.73744 3.18151 2.89805 2.37881 2.77524 2.98508 4.58482 3.61508

0.53496 1.02338 2.90169 0.18649 1.77116 0.69669 0.68962

950 2.55693 4.39879 3.23610 2.57929 3.12589 3.14850 3.71825 2.39727 2.54432 2.62085 3.49868 3.14109 3.86577 2.76892 3.03574 2.58848 2.78955 2.68328 4.96308 3.70520 1542 - -

2.68622 4.41959 2.77524 2.73127 3.46358 2.40517 3.72499 3.29358 2.67745 2.69359 4.24660 2.90342 2.73744 3.18150 2.89805 2.37891 2.77524 2.98522 4.58481 3.61507
0.43616 1.18552 3.03861 0.20821 1.67150 0.61680 0.77581
951 2.33099 4.28290 3.20264 3.06812 4.42689 1.07235 4.27304 3.92507 3.27474 3.60874 4.45564 3.28535 3.74617 3.55939 3.58122 2.19197 2.83237 3.34233 5.74284 4.50871 1544 - -
2.68618 4.42225 2.77519 2.73123 3.46354 2.40513 3.72494 3.29354 2.67741 2.69355 4.24690 2.90347 2.73739 3.18146 2.89801 2.37887 2.77519 2.98518 4.58477 3.61503
0.02680 4.02871 4.75106 0.61958 0.77255 0.36151 1.19278
952 2.62833 4.25513 3.67225 2.94763 3.37943 2.45711 3.92033 2.25345 2.88698 2.12446 2.92865 3.45799 4.02574 3.31746 3.33440 2.90956 2.86071 2.34955 4.85703 3.63978 1545 - -
2.68618 4.42225 2.77519 2.73123 3.46354 2.40513 3.72494 3.29354 2.67741 2.69355 4.24690 2.90347 2.73739 3.18146 2.89801 2.37887 2.77519 2.98518 4.58477 3.61503
0.04677 4.25292 3.45872 0.61958 0.77255 0.48576 0.95510
953 2.65445 5.04707 2.45479 2.35475 4.34664 2.78103 3.63271 3.80444 2.40450 3.33708 4.11083 2.26656 3.82191 2.50902 2.89127 2.34430 2.89005 3.41650 4.28945 4.12795 1546 - -
2.68618 4.42225 2.77519 2.73123 3.46354 2.40513 3.72494 3.29354 2.67741 2.69355 4.24690 2.90347 2.73739 3.18146 2.89801 2.37887 2.77519 2.98518 4.58477 3.61503
0.02190 4.22805 4.95039 0.61958 0.77255 0.51805 0.90555
954 2.67229 2.42338 4.33316 3.75724 2.09232 3.45665 4.14589 2.02858 3.60637 2.22642 3.21763 3.87455 4.17819 3.79815 3.72924 3.10438 2.92101 2.22740 4.72287 3.49112 1547 - -
2.68618 4.42225 2.77519 2.73123 3.46354 2.40513 3.72494 3.29354 2.67741 2.69355 4.24690 2.90347 2.73739 3.18146 2.89801 2.37887 2.77519 2.98518 4.58477 3.61503
0.11926 4.22805 2.32436 0.61958 0.77255 0.51805 0.90555
955 2.59875 4.78809 2.66574 2.44516 4.02477 3.27703 3.63523 3.43867 2.33447 2.63679 3.40090 2.96320 3.82034 2.42053 2.89559 2.49612 2.65741 2.97905 5.29328 3.96119 1548 - -
2.68618 4.42225 2.77519 2.73123 3.46354 2.40513 3.72494 3.29354 2.67741 2.69355 4.24690 2.90347 2.73739 3.18146 2.89801 2.37887 2.77519 2.98518 4.58477 3.61503
0.06876 4.13290 2.98744 0.61958 0.77255 0.62647 0.76459
956 2.60335 4.31179 3.49658 2.92478 2.44601 3.57650 3.80998 2.79160 2.69850 2.24371 3.40956 3.15818 3.95546 3.15328 2.55002 2.83086 2.83415 2.41946 4.85923 3.60539 1549 - -
2.68618 4.42225 2.77519 2.73123 3.46354 2.40513 3.72494 3.29354 2.67741 2.69355 4.24690 2.90347 2.73739 3.18146 2.89801 2.37887 2.77519 2.98518 4.58477 3.61503
0.15800 4.08933 2.04483 0.61958 0.77255 0.66941 0.71746
957 2.57208 4.46043 3.24679 2.73037 3.70747 3.43543 3.77744 2.74356 2.44834 2.70292 3.62773 3.16465 2.25068 3.01620 2.98246 2.72271 2.83809 2.46343 5.11211 3.84602 1550 - -
2.68628 4.42188 2.77521 2.73124 3.46364 2.40523 3.72477 3.29364 2.67742 2.69324 4.24654 2.90307 2.73750 3.18157 2.89811 2.37890 2.77530 2.98516 4.58487 3.61514
0.22440 1.60444 * 1.17360 0.36998 0.00000 *
//

HMMER3/b [3.0 | March 2010]

NAME Xyloglucan xylosyltransferases and galactomannan gal-transferases

LENG 1230

ALPH amino

RF no

CS no

MAP yes

DATE Mon Aug 8 15:41:05 2016

NSEQ 19

EFFN 1.646729

CKSUM 1531952008

STATS LOCAL MSV -13.3360 0.69513

STATS LOCAL VITERBI -14.1104 0.69513

STATS LOCAL FORWARD -7.1802 0.69513

HMM A C D E F G H I K L M N P Q R S T V W Y

m->m m->i m->d i->m i->i d->m d->d

COMPO 2.52377 4.29453 2.92132 2.72232 3.29717 2.81031 3.70701 2.88414 2.70120 2.47676 3.66770 3.08562 3.28284 3.13180 2.82009 2.61364 2.85313 2.67242 4.31570 3.42723

2.68618 4.42225 2.77519 2.73123 3.46354 2.40513 3.72494 3.29354 2.67741 2.69355 4.24690 2.90347 2.73739 3.18146 2.89801 2.37887 2.77519 2.98518 4.58477 3.61503

0.03394 3.79601 4.51836 0.61958 0.77255 0.00000 *

1 2.91157 4.47328 3.96816 3.55750 3.17841 3.77696 4.31044 2.34654 3.32910 1.78626 1.53296 3.84393 4.23864 3.71698 3.54307 3.25192 3.21616 2.34333 5.00802 3.76205 1 - -

2.68618 4.42225 2.77519 2.73123 3.46354 2.40513 3.72494 3.29354 2.67741 2.69355 4.24690 2.90347 2.73739 3.18146 2.89801 2.37887 2.77519 2.98518 4.58477 3.61503

0.03394 3.79601 4.51836 0.61958 0.77255 0.48576 0.95510

2 1.13001 4.15738 3.38757 3.17757 3.97857 2.98348 4.20830 3.09294 3.19189 2.97777 4.01983 3.34570 3.71568 3.54062 3.44647 2.50924 2.78140 2.76077 5.41351 4.20565 2 - -

2.68618 4.42225 2.77519 2.73123 3.46354 2.40513 3.72494 3.29354 2.67741 2.69355 4.24690 2.90347 2.73739 3.18146 2.89801 2.37887 2.77519 2.98518 4.58477 3.61503

0.03394 3.79601 4.51836 0.61958 0.77255 0.48576 0.95510

3 2.82402 4.80362 2.85663 2.61241 3.96136 3.31761 3.76927 3.58235 2.43912 3.09213 4.12436 3.07063 3.87138 1.37681 2.72390 2.87605 3.12210 3.31748 5.24814 3.93742 3 - -

2.68618 4.42225 2.77519 2.73123 3.46354 2.40513 3.72494 3.29354 2.67741 2.69355 4.24690 2.90347 2.73739 3.18146 2.89801 2.37887 2.77519 2.98518 4.58477 3.61503

0.03394 3.79601 4.51836 0.61958 0.77255 0.48576 0.95510

4 2.65642 4.42430 3.25570 3.08531 4.08348 3.11624 4.16757 3.61095 3.13637 3.24548 4.29346 3.39875 0.98293 3.52758 3.38897 2.82198 3.09244 3.26489 5.28934 4.20377 4 - -

2.68618 4.42225 2.77519 2.73123 3.46354 2.40513 3.72494 3.29354 2.67741 2.69355 4.24690 2.90347 2.73739 3.18146 2.89801 2.37887 2.77519 2.98518 4.58477 3.61503

0.03394 3.79601 4.51836 0.61958 0.77255 0.48576 0.95510

5 2.92251 4.76322 3.40570 2.93547 4.09844 3.42264 3.77482 3.63647 2.21535 3.16651 4.17059 3.30011 3.93066 3.00062 1.10219 3.01149 3.19482 3.36420 5.21367 4.04432 5 - -

2.68618 4.42225 2.77519 2.73123 3.46354 2.40513 3.72494 3.29354 2.67741 2.69355 4.24690 2.90347 2.73739 3.18146 2.89801 2.37887 2.77519 2.98518 4.58477 3.61503

0.03394 3.79601 4.51836 0.61958 0.77255 0.48576 0.95510

6 2.92251 4.76322 3.40570 2.93547 4.09844 3.42264 3.77482 3.63647 2.21535 3.16651 4.17059 3.30011 3.93066 3.00062 1.10219 3.01149 3.19482 3.36420 5.21367 4.04432 6 - -
2.68618 4.42225 2.77519 2.73123 3.46354 2.40513 3.72494 3.29354 2.67741 2.69355 4.24690 2.90347 2.73739 3.18146 2.89801 2.37887 2.77519 2.98518 4.58477 3.61503
0.03394 3.79601 4.51836 0.61958 0.77255 0.48576 0.95510
7 2.92251 4.76322 3.40570 2.93547 4.09844 3.42264 3.77482 3.63647 2.21535 3.16651 4.17059 3.30011 3.93066 3.00062 1.10219 3.01149 3.19482 3.36420 5.21367 4.04432 7 - -
2.68618 4.42225 2.77519 2.73123 3.46354 2.40513 3.72494 3.29354 2.67741 2.69355 4.24690 2.90347 2.73739 3.18146 2.89801 2.37887 2.77519 2.98518 4.58477 3.61503
0.03394 3.79601 4.51836 0.61958 0.77255 0.48576 0.95510
8 2.57535 4.35609 3.23321 3.12407 4.26351 0.85754 4.24721 3.84618 3.29467 3.50959 4.48627 3.39802 3.74476 3.62525 3.53733 2.74543 3.05073 3.38948 5.37705 4.36350 8 - -
2.68618 4.42225 2.77519 2.73123 3.46354 2.40513 3.72494 3.29354 2.67741 2.69355 4.24690 2.90347 2.73739 3.18146 2.89801 2.37887 2.77519 2.98518 4.58477 3.61503
0.03394 3.79601 4.51836 0.61958 0.77255 0.48576 0.95510
9 2.57535 4.35609 3.23321 3.12407 4.26351 0.85754 4.24721 3.84618 3.29467 3.50959 4.48627 3.39802 3.74476 3.62525 3.53733 2.74543 3.05073 3.38948 5.37705 4.36350 9 - -
2.68618 4.42225 2.77519 2.73123 3.46354 2.40513 3.72494 3.29354 2.67741 2.69355 4.24690 2.90347 2.73739 3.18146 2.89801 2.37887 2.77519 2.98518 4.58477 3.61503
0.03394 3.79601 4.51836 0.61958 0.77255 0.48576 0.95510
10 2.57535 4.35609 3.23321 3.12407 4.26351 0.85754 4.24721 3.84618 3.29467 3.50959 4.48627 3.39802 3.74476 3.62525 3.53733 2.74543 3.05073 3.38948 5.37705 4.36350 10 - -
2.68618 4.42225 2.77519 2.73123 3.46354 2.40513 3.72494 3.29354 2.67741 2.69355 4.24690 2.90347 2.73739 3.18146 2.89801 2.37887 2.77519 2.98518 4.58477 3.61503
0.03394 3.79601 4.51836 0.61958 0.77255 0.48576 0.95510
11 2.57535 4.35609 3.23321 3.12407 4.26351 0.85754 4.24721 3.84618 3.29467 3.50959 4.48627 3.39802 3.74476 3.62525 3.53733 2.74543 3.05073 3.38948 5.37705 4.36350 11 - -
2.68618 4.42225 2.77519 2.73123 3.46354 2.40513 3.72494 3.29354 2.67741 2.69355 4.24690 2.90347 2.73739 3.18146 2.89801 2.37887 2.77519 2.98518 4.58477 3.61503
0.03394 3.79601 4.51836 0.61958 0.77255 0.48576 0.95510
12 2.91724 4.74509 3.05672 2.81086 3.23540 3.38653 1.31468 3.60804 2.65623 3.11092 4.14942 3.22253 3.93587 3.17665 2.92559 2.98918 3.21880 3.34482 4.70119 3.18882 12 - -
2.68618 4.42225 2.77519 2.73123 3.46354 2.40513 3.72494 3.29354 2.67741 2.69355 4.24690 2.90347 2.73739 3.18146 2.89801 2.37887 2.77519 2.98518 4.58477 3.61503
0.03394 3.79601 4.51836 0.61958 0.77255 0.48576 0.95510
13 2.66315 4.65392 2.67213 2.55089 4.00571 3.13505 3.86476 3.70148 2.76309 3.35772 4.31495 1.30116 3.78410 3.13903 3.11371 2.73434 3.04351 3.33145 5.32076 3.96081 13 - -
2.68618 4.42225 2.77519 2.73123 3.46354 2.40513 3.72494 3.29354 2.67741 2.69355 4.24690 2.90347 2.73739 3.18146 2.89801 2.37887 2.77519 2.98518 4.58477 3.61503
0.03394 3.79601 4.51836 0.61958 0.77255 0.48576 0.95510
14 2.90487 4.86160 3.07558 2.71397 4.23048 3.40048 3.72190 3.65540 1.14833 3.23828 4.20575 3.14113 3.90783 2.91915 2.42439 2.95301 3.16487 3.36817 5.31817 4.12060 14 - -
2.68618 4.42225 2.77519 2.73123 3.46354 2.40513 3.72494 3.29354 2.67741 2.69355 4.24690 2.90347 2.73739 3.18146 2.89801 2.37887 2.77519 2.98518 4.58477 3.61503
0.03394 3.79601 4.51836 0.61958 0.77255 0.48576 0.95510
15 2.50546 1.10990 4.00398 3.72767 3.73846 3.14282 4.39180 2.87127 3.55467 2.78939 3.90900 3.73510 3.84371 3.90416 3.69196 2.77663 2.96608 2.62062 5.16879 4.02616 15 - -
2.68618 4.42225 2.77519 2.73123 3.46354 2.40513 3.72494 3.29354 2.67741 2.69355 4.24690 2.90347 2.73739 3.18146 2.89801 2.37887 2.77519 2.98518 4.58477 3.61503
0.03394 3.79601 4.51836 0.61958 0.77255 0.48576 0.95510
16 2.65642 4.42430 3.25570 3.08531 4.08348 3.11624 4.16757 3.61095 3.13637 3.24548 4.29346 3.39875 0.98293 3.52758 3.38897 2.82198 3.09244 3.26489 5.28934 4.20377 16 - -
2.68618 4.42225 2.77519 2.73123 3.46354 2.40513 3.72494 3.29354 2.67741 2.69355 4.24690 2.90347 2.73739 3.18146 2.89801 2.37887 2.77519 2.98518 4.58477 3.61503
0.03394 3.79601 4.51836 0.61958 0.77255 0.48576 0.95510
17 2.92251 4.76322 3.40570 2.93547 4.09844 3.42264 3.77482 3.63647 2.21535 3.16651 4.17059 3.30011 3.93066 3.00062 1.10219 3.01149 3.19482 3.36420 5.21367 4.04432 17 - -
2.68618 4.42225 2.77519 2.73123 3.46354 2.40513 3.72494 3.29354 2.67741 2.69355 4.24690 2.90347 2.73739 3.18146 2.89801 2.37887 2.77519 2.98518 4.58477 3.61503

0.03394 3.79601 4.51836 0.61958 0.77255 0.48576 0.95510

18 2.65642 4.42430 3.25570 3.08531 4.08348 3.11624 4.16757 3.61095 3.13637 3.24548 4.29346 3.39875 0.98293 3.52758 3.38897 2.82198 3.09244 3.26489 5.28934 4.20377 18 - -

2.68618 4.42225 2.77519 2.73123 3.46354 2.40513 3.72494 3.29354 2.67741 2.69355 4.24690 2.90347 2.73739 3.18146 2.89801 2.37887 2.77519 2.98518 4.58477 3.61503

0.03394 3.79601 4.51836 0.61958 0.77255 0.48576 0.95510

19 1.13001 4.15738 3.38757 3.17757 3.97857 2.98348 4.20830 3.09294 3.19189 2.97777 4.01983 3.34570 3.71568 3.54062 3.44647 2.50924 2.78140 2.76077 5.41351 4.20565 19 - -

2.68618 4.42225 2.77519 2.73123 3.46354 2.40513 3.72494 3.29354 2.67741 2.69355 4.24690 2.90347 2.73739 3.18146 2.89801 2.37887 2.77519 2.98518 4.58477 3.61503

0.03394 3.79601 4.51836 0.61958 0.77255 0.48576 0.95510

20 3.07120 4.55728 4.06560 3.70718 3.10996 3.88305 4.38695 2.31238 3.47558 0.97563 3.16058 3.98980 4.33298 3.86233 3.66334 3.44792 3.36975 2.32945 4.95943 3.66445 20 - -

2.68618 4.42225 2.77519 2.73123 3.46354 2.40513 3.72494 3.29354 2.67741 2.69355 4.24690 2.90347 2.73739 3.18146 2.89801 2.37887 2.77519 2.98518 4.58477 3.61503

0.03394 3.79601 4.51836 0.61958 0.77255 0.48576 0.95510

21 3.07120 4.55728 4.06560 3.70718 3.10996 3.88305 4.38695 2.31238 3.47558 0.97563 3.16058 3.98980 4.33298 3.86233 3.66334 3.44792 3.36975 2.32945 4.95943 3.66445 21 - -

2.68618 4.42225 2.77519 2.73123 3.46354 2.40513 3.72494 3.29354 2.67741 2.69355 4.24690 2.90347 2.73739 3.18146 2.89801 2.37887 2.77519 2.98518 4.58477 3.61503

0.03394 3.79601 4.51836 0.61958 0.77255 0.48576 0.95510

22 2.65642 4.42430 3.25570 3.08531 4.08348 3.11624 4.16757 3.61095 3.13637 3.24548 4.29346 3.39875 0.98293 3.52758 3.38897 2.82198 3.09244 3.26489 5.28934 4.20377 22 - -

2.68618 4.42225 2.77519 2.73123 3.46354 2.40513 3.72494 3.29354 2.67741 2.69355 4.24690 2.90347 2.73739 3.18146 2.89801 2.37887 2.77519 2.98518 4.58477 3.61503

0.03394 3.79601 4.51836 0.61958 0.77255 0.48576 0.95510

23 1.13001 4.15738 3.38757 3.17757 3.97857 2.98348 4.20830 3.09294 3.19189 2.97777 4.01983 3.34570 3.71568 3.54062 3.44647 2.50924 2.78140 2.76077 5.41351 4.20565 23 - -

2.68618 4.42225 2.77519 2.73123 3.46354 2.40513 3.72494 3.29354 2.67741 2.69355 4.24690 2.90347 2.73739 3.18146 2.89801 2.37887 2.77519 2.98518 4.58477 3.61503

0.03394 3.79601 4.51836 0.61958 0.77255 0.48576 0.95510

24 1.13001 4.15738 3.38757 3.17757 3.97857 2.98348 4.20830 3.09294 3.19189 2.97777 4.01983 3.34570 3.71568 3.54062 3.44647 2.50924 2.78140 2.76077 5.41351 4.20565 24 - -

2.68618 4.42225 2.77519 2.73123 3.46354 2.40513 3.72494 3.29354 2.67741 2.69355 4.24690 2.90347 2.73739 3.18146 2.89801 2.37887 2.77519 2.98518 4.58477 3.61503

0.03394 3.79601 4.51836 0.61958 0.77255 0.48576 0.95510

25 1.13001 4.15738 3.38757 3.17757 3.97857 2.98348 4.20830 3.09294 3.19189 2.97777 4.01983 3.34570 3.71568 3.54062 3.44647 2.50924 2.78140 2.76077 5.41351 4.20565 25 - -

2.68618 4.42225 2.77519 2.73123 3.46354 2.40513 3.72494 3.29354 2.67741 2.69355 4.24690 2.90347 2.73739 3.18146 2.89801 2.37887 2.77519 2.98518 4.58477 3.61503

0.03394 3.79601 4.51836 0.61958 0.77255 0.48576 0.95510

26 3.07120 4.55728 4.06560 3.70718 3.10996 3.88305 4.38695 2.31238 3.47558 0.97563 3.16058 3.98980 4.33298 3.86233 3.66334 3.44792 3.36975 2.32945 4.95943 3.66445 26 - -

2.68618 4.42225 2.77519 2.73123 3.46354 2.40513 3.72494 3.29354 2.67741 2.69355 4.24690 2.90347 2.73739 3.18146 2.89801 2.37887 2.77519 2.98518 4.58477 3.61503

0.03394 3.79601 4.51836 0.61958 0.77255 0.48576 0.95510

27 3.07120 4.55728 4.06560 3.70718 3.10996 3.88305 4.38695 2.31238 3.47558 0.97563 3.16058 3.98980 4.33298 3.86233 3.66334 3.44792 3.36975 2.32945 4.95943 3.66445 27 - -

2.68618 4.42225 2.77519 2.73123 3.46354 2.40513 3.72494 3.29354 2.67741 2.69355 4.24690 2.90347 2.73739 3.18146 2.89801 2.37887 2.77519 2.98518 4.58477 3.61503

0.03394 3.79601 4.51836 0.61958 0.77255 0.48576 0.95510

28 3.07120 4.55728 4.06560 3.70718 3.10996 3.88305 4.38695 2.31238 3.47558 0.97563 3.16058 3.98980 4.33298 3.86233 3.66334 3.44792 3.36975 2.32945 4.95943 3.66445 28 - -

2.68618 4.42225 2.77519 2.73123 3.46354 2.40513 3.72494 3.29354 2.67741 2.69355 4.24690 2.90347 2.73739 3.18146 2.89801 2.37887 2.77519 2.98518 4.58477 3.61503

0.03394 3.79601 4.51836 0.61958 0.77255 0.48576 0.95510

29 3.16380 4.58887 4.13307 3.83471 1.14586 3.86813 3.84824 2.69884 3.74404 2.08761 3.44167 3.93408 4.32984 3.93371 3.88264 3.43660 3.46563 2.68355 4.12324 2.50118 29 - -

2.68618 4.42225 2.77519 2.73123 3.46354 2.40513 3.72494 3.29354 2.67741 2.69355 4.24690 2.90347 2.73739 3.18146 2.89801 2.37887 2.77519 2.98518 4.58477 3.61503

0.03394 3.79601 4.51836 0.61958 0.77255 0.48576 0.95510

30 3.07120 4.55728 4.06560 3.70718 3.10996 3.88305 4.38695 2.31238 3.47558 0.97563 3.16058 3.98980 4.33298 3.86233 3.66334 3.44792 3.36975 2.32945 4.95943 3.66445 30 - -

2.68618 4.42225 2.77519 2.73123 3.46354 2.40513 3.72494 3.29354 2.67741 2.69355 4.24690 2.90347 2.73739 3.18146 2.89801 2.37887 2.77519 2.98518 4.58477 3.61503

0.03394 3.79601 4.51836 0.61958 0.77255 0.48576 0.95510

31 3.07120 4.55728 4.06560 3.70718 3.10996 3.88305 4.38695 2.31238 3.47558 0.97563 3.16058 3.98980 4.33298 3.86233 3.66334 3.44792 3.36975 2.32945 4.95943 3.66445 31 - -

2.68618 4.42225 2.77519 2.73123 3.46354 2.40513 3.72494 3.29354 2.67741 2.69355 4.24690 2.90347 2.73739 3.18146 2.89801 2.37887 2.77519 2.98518 4.58477 3.61503

0.03394 3.79601 4.51836 0.61958 0.77255 0.48576 0.95510

32 1.13001 4.15738 3.38757 3.17757 3.97857 2.98348 4.20830 3.09294 3.19189 2.97777 4.01983 3.34570 3.71568 3.54062 3.44647 2.50924 2.78140 2.76077 5.41351 4.20565 32 - -

2.68618 4.42225 2.77519 2.73123 3.46354 2.40513 3.72494 3.29354 2.67741 2.69355 4.24690 2.90347 2.73739 3.18146 2.89801 2.37887 2.77519 2.98518 4.58477 3.61503

0.03394 3.79601 4.51836 0.61958 0.77255 0.48576 0.95510

33 1.13001 4.15738 3.38757 3.17757 3.97857 2.98348 4.20830 3.09294 3.19189 2.97777 4.01983 3.34570 3.71568 3.54062 3.44647 2.50924 2.78140 2.76077 5.41351 4.20565 33 - -

2.68618 4.42225 2.77519 2.73123 3.46354 2.40513 3.72494 3.29354 2.67741 2.69355 4.24690 2.90347 2.73739 3.18146 2.89801 2.37887 2.77519 2.98518 4.58477 3.61503

0.03394 3.79601 4.51836 0.61958 0.77255 0.48576 0.95510

34 2.74814 4.31398 4.01764 3.68039 3.49634 3.64254 4.49596 2.06131 3.54689 2.19820 3.47523 3.88188 4.20873 3.91312 3.76637 3.14284 3.11247 1.12759 5.26714 3.99307 34 - -

2.68618 4.42225 2.77519 2.73123 3.46354 2.40513 3.72494 3.29354 2.67741 2.69355 4.24690 2.90347 2.73739 3.18146 2.89801 2.37887 2.77519 2.98518 4.58477 3.61503

0.03394 3.79601 4.51836 0.61958 0.77255 0.48576 0.95510

35 1.13001 4.15738 3.38757 3.17757 3.97857 2.98348 4.20830 3.09294 3.19189 2.97777 4.01983 3.34570 3.71568 3.54062 3.44647 2.50924 2.78140 2.76077 5.41351 4.20565 35 - -

2.68618 4.42225 2.77519 2.73123 3.46354 2.40513 3.72494 3.29354 2.67741 2.69355 4.24690 2.90347 2.73739 3.18146 2.89801 2.37887 2.77519 2.98518 4.58477 3.61503

0.03394 3.79601 4.51836 0.61958 0.77255 0.48576 0.95510

36 3.07120 4.55728 4.06560 3.70718 3.10996 3.88305 4.38695 2.31238 3.47558 0.97563 3.16058 3.98980 4.33298 3.86233 3.66334 3.44792 3.36975 2.32945 4.95943 3.66445 36 - -

2.68618 4.42225 2.77519 2.73123 3.46354 2.40513 3.72494 3.29354 2.67741 2.69355 4.24690 2.90347 2.73739 3.18146 2.89801 2.37887 2.77519 2.98518 4.58477 3.61503

0.03394 3.79601 4.51836 0.61958 0.77255 0.48576 0.95510

37 3.07120 4.55728 4.06560 3.70718 3.10996 3.88305 4.38695 2.31238 3.47558 0.97563 3.16058 3.98980 4.33298 3.86233 3.66334 3.44792 3.36975 2.32945 4.95943 3.66445 37 - -

2.68618 4.42225 2.77519 2.73123 3.46354 2.40513 3.72494 3.29354 2.67741 2.69355 4.24690 2.90347 2.73739 3.18146 2.89801 2.37887 2.77519 2.98518 4.58477 3.61503

0.03394 3.79601 4.51836 0.61958 0.77255 0.48576 0.95510

38 3.14031 4.66027 3.72681 3.44361 2.30221 3.74216 3.58865 3.16051 3.30332 2.63376 3.84409 3.63185 4.21871 3.62777 3.50199 3.28401 3.42715 3.02772 3.94242 1.10319 38 - -

2.68618 4.42225 2.77519 2.73123 3.46354 2.40513 3.72494 3.29354 2.67741 2.69355 4.24690 2.90347 2.73739 3.18146 2.89801 2.37887 2.77519 2.98518 4.58477 3.61503

0.03394 3.79601 4.51836 0.61958 0.77255 0.48576 0.95510

39 2.74814 4.31398 4.01764 3.68039 3.49634 3.64254 4.49596 2.06131 3.54689 2.19820 3.47523 3.88188 4.20873 3.91312 3.76637 3.14284 3.11247 1.12759 5.26714 3.99307 39 - -

2.68618 4.42225 2.77519 2.73123 3.46354 2.40513 3.72494 3.29354 2.67741 2.69355 4.24690 2.90347 2.73739 3.18146 2.89801 2.37887 2.77519 2.98518 4.58477 3.61503

0.03394 3.79601 4.51836 0.61958 0.77255 0.48576 0.95510

40 2.28079 4.19908 3.14317 2.95737 4.00099 2.93053 4.06839 3.51415 3.02585 3.24109 4.17505 3.19319 3.66344 3.38168 3.31291 1.21257 2.75555 3.06614 5.38010 4.08457 40 - -

2.68618 4.42225 2.77519 2.73123 3.46354 2.40513 3.72494 3.29354 2.67741 2.69355 4.24690 2.90347 2.73739 3.18146 2.89801 2.37887 2.77519 2.98518 4.58477 3.61503

0.03394 3.79601 4.51836 0.61958 0.77255 0.48576 0.95510

41 2.65642 4.42430 3.25570 3.08531 4.08348 3.11624 4.16757 3.61095 3.13637 3.24548 4.29346 3.39875 0.98293 3.52758 3.38897 2.82198 3.09244 3.26489 5.28934 4.20377 41 - -
2.68618 4.42225 2.77519 2.73123 3.46354 2.40513 3.72494 3.29354 2.67741 2.69355 4.24690 2.90347 2.73739 3.18146 2.89801 2.37887 2.77519 2.98518 4.58477 3.61503
0.03394 3.79601 4.51836 0.61958 0.77255 0.48576 0.95510
42 2.65642 4.42430 3.25570 3.08531 4.08348 3.11624 4.16757 3.61095 3.13637 3.24548 4.29346 3.39875 0.98293 3.52758 3.38897 2.82198 3.09244 3.26489 5.28934 4.20377 42 - -
2.68618 4.42225 2.77519 2.73123 3.46354 2.40513 3.72494 3.29354 2.67741 2.69355 4.24690 2.90347 2.73739 3.18146 2.89801 2.37887 2.77519 2.98518 4.58477 3.61503
0.03394 3.79601 4.51836 0.61958 0.77255 0.48576 0.95510
43 2.65642 4.42430 3.25570 3.08531 4.08348 3.11624 4.16757 3.61095 3.13637 3.24548 4.29346 3.39875 0.98293 3.52758 3.38897 2.82198 3.09244 3.26489 5.28934 4.20377 43 - -
2.68618 4.42225 2.77519 2.73123 3.46354 2.40513 3.72494 3.29354 2.67741 2.69355 4.24690 2.90347 2.73739 3.18146 2.89801 2.37887 2.77519 2.98518 4.58477 3.61503
0.03394 3.79601 4.51836 0.61958 0.77255 0.48576 0.95510
44 3.07120 4.55728 4.06560 3.70718 3.10996 3.88305 4.38695 2.31238 3.47558 0.97563 3.16058 3.98980 4.33298 3.86233 3.66334 3.44792 3.36975 2.32945 4.95943 3.66445 44 - -
2.68618 4.42225 2.77519 2.73123 3.46354 2.40513 3.72494 3.29354 2.67741 2.69355 4.24690 2.90347 2.73739 3.18146 2.89801 2.37887 2.77519 2.98518 4.58477 3.61503
0.03394 3.79601 4.51836 0.61958 0.77255 0.48576 0.95510
45 2.97165 4.41429 4.22289 3.83744 3.32476 3.98045 4.58108 1.17818 3.66390 1.92890 3.27450 4.09357 4.42084 4.02132 3.86934 3.47002 3.27299 1.84997 5.19827 3.92855 45 - -
2.68618 4.42225 2.77519 2.73123 3.46354 2.40513 3.72494 3.29354 2.67741 2.69355 4.24690 2.90347 2.73739 3.18146 2.89801 2.37887 2.77519 2.98518 4.58477 3.61503
0.03394 3.79601 4.51836 0.61958 0.77255 0.48576 0.95510
46 2.91056 5.02139 1.03432 2.29423 4.38899 3.15549 3.86877 3.96045 2.93927 3.58342 4.56179 2.84589 3.80315 3.10925 3.44342 2.88302 3.24824 3.60850 5.57234 4.27708 46 - -
2.68618 4.42225 2.77519 2.73123 3.46354 2.40513 3.72494 3.29354 2.67741 2.69355 4.24690 2.90347 2.73739 3.18146 2.89801 2.37887 2.77519 2.98518 4.58477 3.61503
0.03394 3.79601 4.51836 0.61958 0.77255 0.48576 0.95510
47 2.91724 4.74509 3.05672 2.81086 3.23540 3.38653 1.31468 3.60804 2.65623 3.11092 4.14942 3.22253 3.93587 3.17665 2.92559 2.98918 3.21880 3.34482 4.70119 3.18882 47 - -
2.68618 4.42225 2.77519 2.73123 3.46354 2.40513 3.72494 3.29354 2.67741 2.69355 4.24690 2.90347 2.73739 3.18146 2.89801 2.37887 2.77519 2.98518 4.58477 3.61503
0.03394 3.79601 4.51836 0.61958 0.77255 0.48576 0.95510
48 2.65642 4.42430 3.25570 3.08531 4.08348 3.11624 4.16757 3.61095 3.13637 3.24548 4.29346 3.39875 0.98293 3.52758 3.38897 2.82198 3.09244 3.26489 5.28934 4.20377 48 - -
2.68618 4.42225 2.77519 2.73123 3.46354 2.40513 3.72494 3.29354 2.67741 2.69355 4.24690 2.90347 2.73739 3.18146 2.89801 2.37887 2.77519 2.98518 4.58477 3.61503
0.03394 3.79601 4.51836 0.61958 0.77255 0.48576 0.95510
49 1.13001 4.15738 3.38757 3.17757 3.97857 2.98348 4.20830 3.09294 3.19189 2.97777 4.01983 3.34570 3.71568 3.54062 3.44647 2.50924 2.78140 2.76077 5.41351 4.20565 49 - -
2.68618 4.42225 2.77519 2.73123 3.46354 2.40513 3.72494 3.29354 2.67741 2.69355 4.24690 2.90347 2.73739 3.18146 2.89801 2.37887 2.77519 2.98518 4.58477 3.61503
0.03394 3.79601 4.51836 0.61958 0.77255 0.48576 0.95510
50 3.07120 4.55728 4.06560 3.70718 3.10996 3.88305 4.38695 2.31238 3.47558 0.97563 3.16058 3.98980 4.33298 3.86233 3.66334 3.44792 3.36975 2.32945 4.95943 3.66445 50 - -
2.68618 4.42225 2.77519 2.73123 3.46354 2.40513 3.72494 3.29354 2.67741 2.69355 4.24690 2.90347 2.73739 3.18146 2.89801 2.37887 2.77519 2.98518 4.58477 3.61503
0.03394 3.79601 4.51836 0.61958 0.77255 0.48576 0.95510
51 1.13001 4.15738 3.38757 3.17757 3.97857 2.98348 4.20830 3.09294 3.19189 2.97777 4.01983 3.34570 3.71568 3.54062 3.44647 2.50924 2.78140 2.76077 5.41351 4.20565 51 - -
2.68618 4.42225 2.77519 2.73123 3.46354 2.40513 3.72494 3.29354 2.67741 2.69355 4.24690 2.90347 2.73739 3.18146 2.89801 2.37887 2.77519 2.98518 4.58477 3.61503
0.03394 3.79601 4.51836 0.61958 0.77255 0.48576 0.95510
52 2.28079 4.19908 3.14317 2.95737 4.00099 2.93053 4.06839 3.51415 3.02585 3.24109 4.17505 3.19319 3.66344 3.38168 3.31291 1.21257 2.75555 3.06614 5.38010 4.08457 52 - -
2.68618 4.42225 2.77519 2.73123 3.46354 2.40513 3.72494 3.29354 2.67741 2.69355 4.24690 2.90347 2.73739 3.18146 2.89801 2.37887 2.77519 2.98518 4.58477 3.61503

0.03394 3.79601 4.51836 0.61958 0.77255 0.48576 0.95510
53 2.28079 4.19908 3.14317 2.95737 4.00099 2.93053 4.06839 3.51415 3.02585 3.24109 4.17505 3.19319 3.66344 3.38168 3.31291 1.21257 2.75555 3.06614 5.38010 4.08457 53 - -
2.68618 4.42225 2.77519 2.73123 3.46354 2.40513 3.72494 3.29354 2.67741 2.69355 4.24690 2.90347 2.73739 3.18146 2.89801 2.37887 2.77519 2.98518 4.58477 3.61503
0.03394 3.79601 4.51836 0.61958 0.77255 0.48576 0.95510
54 2.92251 4.76322 3.40570 2.93547 4.09844 3.42264 3.77482 3.63647 2.21535 3.16651 4.17059 3.30011 3.93066 3.00062 1.10219 3.01149 3.19482 3.36420 5.21367 4.04432 54 - -
2.68618 4.42225 2.77519 2.73123 3.46354 2.40513 3.72494 3.29354 2.67741 2.69355 4.24690 2.90347 2.73739 3.18146 2.89801 2.37887 2.77519 2.98518 4.58477 3.61503
0.03394 3.79601 4.51836 0.61958 0.77255 0.48576 0.95510
55 2.92251 4.76322 3.40570 2.93547 4.09844 3.42264 3.77482 3.63647 2.21535 3.16651 4.17059 3.30011 3.93066 3.00062 1.10219 3.01149 3.19482 3.36420 5.21367 4.04432 55 - -
2.68618 4.42225 2.77519 2.73123 3.46354 2.40513 3.72494 3.29354 2.67741 2.69355 4.24690 2.90347 2.73739 3.18146 2.89801 2.37887 2.77519 2.98518 4.58477 3.61503
0.03394 3.79601 4.51836 0.61958 0.77255 0.48576 0.95510
56 2.92251 4.76322 3.40570 2.93547 4.09844 3.42264 3.77482 3.63647 2.21535 3.16651 4.17059 3.30011 3.93066 3.00062 1.10219 3.01149 3.19482 3.36420 5.21367 4.04432 56 - -
2.68618 4.42225 2.77519 2.73123 3.46354 2.40513 3.72494 3.29354 2.67741 2.69355 4.24690 2.90347 2.73739 3.18146 2.89801 2.37887 2.77519 2.98518 4.58477 3.61503
0.03394 3.79601 4.51836 0.61958 0.77255 0.48576 0.95510
57 2.28079 4.19908 3.14317 2.95737 4.00099 2.93053 4.06839 3.51415 3.02585 3.24109 4.17505 3.19319 3.66344 3.38168 3.31291 1.21257 2.75555 3.06614 5.38010 4.08457 57 - -
2.68618 4.42225 2.77519 2.73123 3.46354 2.40513 3.72494 3.29354 2.67741 2.69355 4.24690 2.90347 2.73739 3.18146 2.89801 2.37887 2.77519 2.98518 4.58477 3.61503
0.03394 3.79601 4.51836 0.61958 0.77255 0.48576 0.95510
58 2.65642 4.42430 3.25570 3.08531 4.08348 3.11624 4.16757 3.61095 3.13637 3.24548 4.29346 3.39875 0.98293 3.52758 3.38897 2.82198 3.09244 3.26489 5.28934 4.20377 58 - -
2.68618 4.42225 2.77519 2.73123 3.46354 2.40513 3.72494 3.29354 2.67741 2.69355 4.24690 2.90347 2.73739 3.18146 2.89801 2.37887 2.77519 2.98518 4.58477 3.61503
0.03394 3.79601 4.51836 0.61958 0.77255 0.48576 0.95510
59 2.91724 4.74509 3.05672 2.81086 3.23540 3.38653 1.31468 3.60804 2.65623 3.11092 4.14942 3.22253 3.93587 3.17665 2.92559 2.98918 3.21880 3.34482 4.70119 3.18882 59 - -
2.68618 4.42225 2.77519 2.73123 3.46354 2.40513 3.72494 3.29354 2.67741 2.69355 4.24690 2.90347 2.73739 3.18146 2.89801 2.37887 2.77519 2.98518 4.58477 3.61503
0.03394 3.79601 4.51836 0.61958 0.77255 0.48576 0.95510
60 1.13001 4.15738 3.38757 3.17757 3.97857 2.98348 4.20830 3.09294 3.19189 2.97777 4.01983 3.34570 3.71568 3.54062 3.44647 2.50924 2.78140 2.76077 5.41351 4.20565 60 - -
2.68618 4.42225 2.77519 2.73123 3.46354 2.40513 3.72494 3.29354 2.67741 2.69355 4.24690 2.90347 2.73739 3.18146 2.89801 2.37887 2.77519 2.98518 4.58477 3.61503
0.03394 3.79601 4.51836 0.61958 0.77255 0.48576 0.95510
61 2.65642 4.42430 3.25570 3.08531 4.08348 3.11624 4.16757 3.61095 3.13637 3.24548 4.29346 3.39875 0.98293 3.52758 3.38897 2.82198 3.09244 3.26489 5.28934 4.20377 61 - -
2.68618 4.42225 2.77519 2.73123 3.46354 2.40513 3.72494 3.29354 2.67741 2.69355 4.24690 2.90347 2.73739 3.18146 2.89801 2.37887 2.77519 2.98518 4.58477 3.61503
0.03394 3.79601 4.51836 0.61958 0.77255 0.48576 0.95510
62 3.07120 4.55728 4.06560 3.70718 3.10996 3.88305 4.38695 2.31238 3.47558 0.97563 3.16058 3.98980 4.33298 3.86233 3.66334 3.44792 3.36975 2.32945 4.95943 3.66445 62 - -
2.68618 4.42225 2.77519 2.73123 3.46354 2.40513 3.72494 3.29354 2.67741 2.69355 4.24690 2.90347 2.73739 3.18146 2.89801 2.37887 2.77519 2.98518 4.58477 3.61503
0.03394 3.79601 4.51836 0.61958 0.77255 0.48576 0.95510
63 3.07120 4.55728 4.06560 3.70718 3.10996 3.88305 4.38695 2.31238 3.47558 0.97563 3.16058 3.98980 4.33298 3.86233 3.66334 3.44792 3.36975 2.32945 4.95943 3.66445 63 - -
2.68618 4.42225 2.77519 2.73123 3.46354 2.40513 3.72494 3.29354 2.67741 2.69355 4.24690 2.90347 2.73739 3.18146 2.89801 2.37887 2.77519 2.98518 4.58477 3.61503
0.03394 3.79601 4.51836 0.61958 0.77255 0.48576 0.95510
64 2.66315 4.65392 2.67213 2.55089 4.00571 3.13505 3.86476 3.70148 2.76309 3.35772 4.31495 1.30116 3.78410 3.13903 3.11371 2.73434 3.04351 3.33145 5.32076 3.96081 64 - -

2.68618 4.42225 2.77519 2.73123 3.46354 2.40513 3.72494 3.29354 2.67741 2.69355 4.24690 2.90347 2.73739 3.18146 2.89801 2.37887 2.77519 2.98518 4.58477 3.61503

0.03394 3.79601 4.51836 0.61958 0.77255 0.48576 0.95510

65 2.28079 4.19908 3.14317 2.95737 4.00099 2.93053 4.06839 3.51415 3.02585 3.24109 4.17505 3.19319 3.66344 3.38168 3.31291 1.21257 2.75555 3.06614 5.38010 4.08457 65 - -

2.68618 4.42225 2.77519 2.73123 3.46354 2.40513 3.72494 3.29354 2.67741 2.69355 4.24690 2.90347 2.73739 3.18146 2.89801 2.37887 2.77519 2.98518 4.58477 3.61503

0.03394 3.79601 4.51836 0.61958 0.77255 0.48576 0.95510

66 2.28079 4.19908 3.14317 2.95737 4.00099 2.93053 4.06839 3.51415 3.02585 3.24109 4.17505 3.19319 3.66344 3.38168 3.31291 1.21257 2.75555 3.06614 5.38010 4.08457 66 - -

2.68618 4.42225 2.77519 2.73123 3.46354 2.40513 3.72494 3.29354 2.67741 2.69355 4.24690 2.90347 2.73739 3.18146 2.89801 2.37887 2.77519 2.98518 4.58477 3.61503

0.03394 3.79601 4.51836 0.61958 0.77255 0.48576 0.95510

67 2.57535 4.35609 3.23321 3.12407 4.26351 0.85754 4.24721 3.84618 3.29467 3.50959 4.48627 3.39802 3.74476 3.62525 3.53733 2.74543 3.05073 3.38948 5.37705 4.36350 67 - -

2.68618 4.42225 2.77519 2.73123 3.46354 2.40513 3.72494 3.29354 2.67741 2.69355 4.24690 2.90347 2.73739 3.18146 2.89801 2.37887 2.77519 2.98518 4.58477 3.61503

0.03394 3.79601 4.51836 0.61958 0.77255 0.48576 0.95510

68 2.57535 4.35609 3.23321 3.12407 4.26351 0.85754 4.24721 3.84618 3.29467 3.50959 4.48627 3.39802 3.74476 3.62525 3.53733 2.74543 3.05073 3.38948 5.37705 4.36350 68 - -

2.68618 4.42225 2.77519 2.73123 3.46354 2.40513 3.72494 3.29354 2.67741 2.69355 4.24690 2.90347 2.73739 3.18146 2.89801 2.37887 2.77519 2.98518 4.58477 3.61503

0.03394 3.79601 4.51836 0.61958 0.77255 0.48576 0.95510

69 2.57535 4.35609 3.23321 3.12407 4.26351 0.85754 4.24721 3.84618 3.29467 3.50959 4.48627 3.39802 3.74476 3.62525 3.53733 2.74543 3.05073 3.38948 5.37705 4.36350 69 - -

2.68618 4.42225 2.77519 2.73123 3.46354 2.40513 3.72494 3.29354 2.67741 2.69355 4.24690 2.90347 2.73739 3.18146 2.89801 2.37887 2.77519 2.98518 4.58477 3.61503

0.03394 3.79601 4.51836 0.61958 0.77255 0.48576 0.95510

70 2.28079 4.19908 3.14317 2.95737 4.00099 2.93053 4.06839 3.51415 3.02585 3.24109 4.17505 3.19319 3.66344 3.38168 3.31291 1.21257 2.75555 3.06614 5.38010 4.08457 70 - -

2.68618 4.42225 2.77519 2.73123 3.46354 2.40513 3.72494 3.29354 2.67741 2.69355 4.24690 2.90347 2.73739 3.18146 2.89801 2.37887 2.77519 2.98518 4.58477 3.61503

0.03394 3.79601 4.51836 0.61958 0.77255 0.48576 0.95510

71 2.37514 4.21936 3.36356 3.11439 3.90452 3.06054 4.13711 3.02248 3.05582 2.88857 3.94573 3.33472 3.75900 3.45502 3.31699 2.57577 1.30864 2.72567 5.34922 4.12174 71 - -

2.68618 4.42225 2.77519 2.73123 3.46354 2.40513 3.72494 3.29354 2.67741 2.69355 4.24690 2.90347 2.73739 3.18146 2.89801 2.37887 2.77519 2.98518 4.58477 3.61503

0.03394 3.79601 4.51836 0.61958 0.77255 0.48576 0.95510

72 2.74814 4.31398 4.01764 3.68039 3.49634 3.64254 4.49596 2.06131 3.54689 2.19820 3.47523 3.88188 4.20873 3.91312 3.76637 3.14284 3.11247 1.12759 5.26714 3.99307 72 - -

2.68618 4.42225 2.77519 2.73123 3.46354 2.40513 3.72494 3.29354 2.67741 2.69355 4.24690 2.90347 2.73739 3.18146 2.89801 2.37887 2.77519 2.98518 4.58477 3.61503

0.03394 3.79601 4.51836 0.61958 0.77255 0.48576 0.95510

73 2.74814 4.31398 4.01764 3.68039 3.49634 3.64254 4.49596 2.06131 3.54689 2.19820 3.47523 3.88188 4.20873 3.91312 3.76637 3.14284 3.11247 1.12759 5.26714 3.99307 73 - -

2.68618 4.42225 2.77519 2.73123 3.46354 2.40513 3.72494 3.29354 2.67741 2.69355 4.24690 2.90347 2.73739 3.18146 2.89801 2.37887 2.77519 2.98518 4.58477 3.61503

0.03394 3.79601 4.51836 0.61958 0.77255 0.48576 0.95510

74 2.28079 4.19908 3.14317 2.95737 4.00099 2.93053 4.06839 3.51415 3.02585 3.24109 4.17505 3.19319 3.66344 3.38168 3.31291 1.21257 2.75555 3.06614 5.38010 4.08457 74 - -

2.68618 4.42225 2.77519 2.73123 3.46354 2.40513 3.72494 3.29354 2.67741 2.69355 4.24690 2.90347 2.73739 3.18146 2.89801 2.37887 2.77519 2.98518 4.58477 3.61503

0.03394 3.79601 4.51836 0.61958 0.77255 0.48576 0.95510

75 2.89015 4.98001 2.46169 1.13957 4.31725 3.22729 3.81015 3.75600 2.68126 3.38645 4.36600 2.89555 3.82889 3.02881 3.08050 2.88174 3.19454 3.44858 5.50348 4.22803 75 - -

2.68618 4.42225 2.77519 2.73123 3.46354 2.40513 3.72494 3.29354 2.67741 2.69355 4.24690 2.90347 2.73739 3.18146 2.89801 2.37887 2.77519 2.98518 4.58477 3.61503

0.03394 3.79601 4.51836 0.61958 0.77255 0.48576 0.95510

76 2.91724 4.74509 3.05672 2.81086 3.23540 3.38653 1.31468 3.60804 2.65623 3.11092 4.14942 3.22253 3.93587 3.17665 2.92559 2.98918 3.21880 3.34482 4.70119 3.18882 76 - -
2.68618 4.42225 2.77519 2.73123 3.46354 2.40513 3.72494 3.29354 2.67741 2.69355 4.24690 2.90347 2.73739 3.18146 2.89801 2.37887 2.77519 2.98518 4.58477 3.61503
0.03394 3.79601 4.51836 0.61958 0.77255 0.48576 0.95510
77 2.28079 4.19908 3.14317 2.95737 4.00099 2.93053 4.06839 3.51415 3.02585 3.24109 4.17505 3.19319 3.66344 3.38168 3.31291 1.21257 2.75555 3.06614 5.38010 4.08457 77 - -
2.68618 4.42225 2.77519 2.73123 3.46354 2.40513 3.72494 3.29354 2.67741 2.69355 4.24690 2.90347 2.73739 3.18146 2.89801 2.37887 2.77519 2.98518 4.58477 3.61503
0.03394 3.79601 4.51836 0.61958 0.77255 0.48576 0.95510
78 2.89015 4.98001 2.46169 1.13957 4.31725 3.22729 3.81015 3.75600 2.68126 3.38645 4.36600 2.89555 3.82889 3.02881 3.08050 2.88174 3.19454 3.44858 5.50348 4.22803 78 - -
2.68618 4.42225 2.77519 2.73123 3.46354 2.40513 3.72494 3.29354 2.67741 2.69355 4.24690 2.90347 2.73739 3.18146 2.89801 2.37887 2.77519 2.98518 4.58477 3.61503
0.03394 3.79601 4.51836 0.61958 0.77255 0.48576 0.95510
79 2.97165 4.41429 4.22289 3.83744 3.32476 3.98045 4.58108 1.17818 3.66390 1.92890 3.27450 4.09357 4.42084 4.02132 3.86934 3.47002 3.27299 1.84997 5.19827 3.92855 79 - -
2.68618 4.42225 2.77519 2.73123 3.46354 2.40513 3.72494 3.29354 2.67741 2.69355 4.24690 2.90347 2.73739 3.18146 2.89801 2.37887 2.77519 2.98518 4.58477 3.61503
0.03394 3.79601 4.51836 0.61958 0.77255 0.48576 0.95510
80 2.28079 4.19908 3.14317 2.95737 4.00099 2.93053 4.06839 3.51415 3.02585 3.24109 4.17505 3.19319 3.66344 3.38168 3.31291 1.21257 2.75555 3.06614 5.38010 4.08457 80 - -
2.68618 4.42225 2.77519 2.73123 3.46354 2.40513 3.72494 3.29354 2.67741 2.69355 4.24690 2.90347 2.73739 3.18146 2.89801 2.37887 2.77519 2.98518 4.58477 3.61503
0.03394 3.79601 4.51836 0.61958 0.77255 0.48576 0.95510
81 2.92251 4.76322 3.40570 2.93547 4.09844 3.42264 3.77482 3.63647 2.21535 3.16651 4.17059 3.30011 3.93066 3.00062 1.10219 3.01149 3.19482 3.36420 5.21367 4.04432 81 - -
2.68618 4.42225 2.77519 2.73123 3.46354 2.40513 3.72494 3.29354 2.67741 2.69355 4.24690 2.90347 2.73739 3.18146 2.89801 2.37887 2.77519 2.98518 4.58477 3.61503
0.03394 3.79601 4.51836 0.61958 0.77255 0.48576 0.95510
82 2.74814 4.31398 4.01764 3.68039 3.49634 3.64254 4.49596 2.06131 3.54689 2.19820 3.47523 3.88188 4.20873 3.91312 3.76637 3.14284 3.11247 1.12759 5.26714 3.99307 82 - -
2.68618 4.42225 2.77519 2.73123 3.46354 2.40513 3.72494 3.29354 2.67741 2.69355 4.24690 2.90347 2.73739 3.18146 2.89801 2.37887 2.77519 2.98518 4.58477 3.61503
0.03394 3.79601 4.51836 0.61958 0.77255 0.48576 0.95510
83 2.65642 4.42430 3.25570 3.08531 4.08348 3.11624 4.16757 3.61095 3.13637 3.24548 4.29346 3.39875 0.98293 3.52758 3.38897 2.82198 3.09244 3.26489 5.28934 4.20377 83 - -
2.68618 4.42225 2.77519 2.73123 3.46354 2.40513 3.72494 3.29354 2.67741 2.69355 4.24690 2.90347 2.73739 3.18146 2.89801 2.37887 2.77519 2.98518 4.58477 3.61503
0.03394 3.79601 4.51836 0.61958 0.77255 0.48576 0.95510
84 2.97165 4.41429 4.22289 3.83744 3.32476 3.98045 4.58108 1.17818 3.66390 1.92890 3.27450 4.09357 4.42084 4.02132 3.86934 3.47002 3.27299 1.84997 5.19827 3.92855 84 - -
2.68618 4.42225 2.77519 2.73123 3.46354 2.40513 3.72494 3.29354 2.67741 2.69355 4.24690 2.90347 2.73739 3.18146 2.89801 2.37887 2.77519 2.98518 4.58477 3.61503
0.03394 3.79601 4.51836 0.61958 0.77255 0.48576 0.95510
85 2.28079 4.19908 3.14317 2.95737 4.00099 2.93053 4.06839 3.51415 3.02585 3.24109 4.17505 3.19319 3.66344 3.38168 3.31291 1.21257 2.75555 3.06614 5.38010 4.08457 85 - -
2.68618 4.42225 2.77519 2.73123 3.46354 2.40513 3.72494 3.29354 2.67741 2.69355 4.24690 2.90347 2.73739 3.18146 2.89801 2.37887 2.77519 2.98518 4.58477 3.61503
0.03394 3.79601 4.51836 0.61958 0.77255 0.48576 0.95510
86 2.90487 4.86160 3.07558 2.71397 4.23048 3.40048 3.72190 3.65540 1.14833 3.23828 4.20575 3.14113 3.90783 2.91915 2.42439 2.95301 3.16487 3.36817 5.31817 4.12060 86 - -
2.68618 4.42225 2.77519 2.73123 3.46354 2.40513 3.72494 3.29354 2.67741 2.69355 4.24690 2.90347 2.73739 3.18146 2.89801 2.37887 2.77519 2.98518 4.58477 3.61503
0.03394 3.79601 4.51836 0.61958 0.77255 0.48576 0.95510
87 2.89015 4.98001 2.46169 1.13957 4.31725 3.22729 3.81015 3.75600 2.68126 3.38645 4.36600 2.89555 3.82889 3.02881 3.08050 2.88174 3.19454 3.44858 5.50348 4.22803 87 - -
2.68618 4.42225 2.77519 2.73123 3.46354 2.40513 3.72494 3.29354 2.67741 2.69355 4.24690 2.90347 2.73739 3.18146 2.89801 2.37887 2.77519 2.98518 4.58477 3.61503

0.03394 3.79601 4.51836 0.61958 0.77255 0.48576 0.95510

88 1.13001 4.15738 3.38757 3.17757 3.97857 2.98348 4.20830 3.09294 3.19189 2.97777 4.01983 3.34570 3.71568 3.54062 3.44647 2.50924 2.78140 2.76077 5.41351 4.20565 88 - -

2.68618 4.42225 2.77519 2.73123 3.46354 2.40513 3.72494 3.29354 2.67741 2.69355 4.24690 2.90347 2.73739 3.18146 2.89801 2.37887 2.77519 2.98518 4.58477 3.61503

0.03394 3.79601 4.51836 0.61958 0.77255 0.48576 0.95510

89 2.91056 5.02139 1.03432 2.29423 4.38899 3.15549 3.86877 3.96045 2.93927 3.58342 4.56179 2.84589 3.80315 3.10925 3.44342 2.88302 3.24824 3.60850 5.57234 4.27708 89 - -

2.68618 4.42225 2.77519 2.73123 3.46354 2.40513 3.72494 3.29354 2.67741 2.69355 4.24690 2.90347 2.73739 3.18146 2.89801 2.37887 2.77519 2.98518 4.58477 3.61503

0.03394 3.79601 4.51836 0.61958 0.77255 0.48576 0.95510

90 2.57535 4.35609 3.23321 3.12407 4.26351 0.85754 4.24721 3.84618 3.29467 3.50959 4.48627 3.39802 3.74476 3.62525 3.53733 2.74543 3.05073 3.38948 5.37705 4.36350 90 - -

2.68618 4.42225 2.77519 2.73123 3.46354 2.40513 3.72494 3.29354 2.67741 2.69355 4.24690 2.90347 2.73739 3.18146 2.89801 2.37887 2.77519 2.98518 4.58477 3.61503

0.03394 3.79601 4.51836 0.61958 0.77255 0.48576 0.95510

91 3.07120 4.55728 4.06560 3.70718 3.10996 3.88305 4.38695 2.31238 3.47558 0.97563 3.16058 3.98980 4.33298 3.86233 3.66334 3.44792 3.36975 2.32945 4.95943 3.66445 91 - -

2.68618 4.42225 2.77519 2.73123 3.46354 2.40513 3.72494 3.29354 2.67741 2.69355 4.24690 2.90347 2.73739 3.18146 2.89801 2.37887 2.77519 2.98518 4.58477 3.61503

0.03394 3.79601 4.51836 0.61958 0.77255 0.48576 0.95510

92 3.21457 4.61548 3.97805 3.66660 2.59647 3.64757 3.81244 3.23661 3.39107 2.64390 3.87379 3.83150 4.15944 3.78287 3.53232 3.41887 3.51155 3.12484 1.05487 2.60290 92 - -

2.68618 4.42225 2.77519 2.73123 3.46354 2.40513 3.72494 3.29354 2.67741 2.69355 4.24690 2.90347 2.73739 3.18146 2.89801 2.37887 2.77519 2.98518 4.58477 3.61503

0.03394 3.79601 4.51836 0.61958 0.77255 0.48576 0.95510

93 2.57535 4.35609 3.23321 3.12407 4.26351 0.85754 4.24721 3.84618 3.29467 3.50959 4.48627 3.39802 3.74476 3.62525 3.53733 2.74543 3.05073 3.38948 5.37705 4.36350 93 - -

2.68618 4.42225 2.77519 2.73123 3.46354 2.40513 3.72494 3.29354 2.67741 2.69355 4.24690 2.90347 2.73739 3.18146 2.89801 2.37887 2.77519 2.98518 4.58477 3.61503

0.03394 3.79601 4.51836 0.61958 0.77255 0.48576 0.95510

94 2.28079 4.19908 3.14317 2.95737 4.00099 2.93053 4.06839 3.51415 3.02585 3.24109 4.17505 3.19319 3.66344 3.38168 3.31291 1.21257 2.75555 3.06614 5.38010 4.08457 94 - -

2.68618 4.42225 2.77519 2.73123 3.46354 2.40513 3.72494 3.29354 2.67741 2.69355 4.24690 2.90347 2.73739 3.18146 2.89801 2.37887 2.77519 2.98518 4.58477 3.61503

0.03394 3.79601 4.51836 0.61958 0.77255 0.48576 0.95510

95 2.90487 4.86160 3.07558 2.71397 4.23048 3.40048 3.72190 3.65540 1.14833 3.23828 4.20575 3.14113 3.90783 2.91915 2.42439 2.95301 3.16487 3.36817 5.31817 4.12060 95 - -

2.68618 4.42225 2.77519 2.73123 3.46354 2.40513 3.72494 3.29354 2.67741 2.69355 4.24690 2.90347 2.73739 3.18146 2.89801 2.37887 2.77519 2.98518 4.58477 3.61503

0.03394 3.79601 4.51836 0.61958 0.77255 0.48576 0.95510

96 3.16380 4.58887 4.13307 3.83471 1.14586 3.86813 3.84824 2.69884 3.74404 2.08761 3.44167 3.93408 4.32984 3.93371 3.88264 3.43660 3.46563 2.68355 4.12324 2.50118 96 - -

2.68618 4.42225 2.77519 2.73123 3.46354 2.40513 3.72494 3.29354 2.67741 2.69355 4.24690 2.90347 2.73739 3.18146 2.89801 2.37887 2.77519 2.98518 4.58477 3.61503

0.03394 3.79601 4.51836 0.61958 0.77255 0.48576 0.95510

97 1.13001 4.15738 3.38757 3.17757 3.97857 2.98348 4.20830 3.09294 3.19189 2.97777 4.01983 3.34570 3.71568 3.54062 3.44647 2.50924 2.78140 2.76077 5.41351 4.20565 97 - -

2.68618 4.42225 2.77519 2.73123 3.46354 2.40513 3.72494 3.29354 2.67741 2.69355 4.24690 2.90347 2.73739 3.18146 2.89801 2.37887 2.77519 2.98518 4.58477 3.61503

0.03394 3.79601 4.51836 0.61958 0.77255 0.48576 0.95510

98 2.28079 4.19908 3.14317 2.95737 4.00099 2.93053 4.06839 3.51415 3.02585 3.24109 4.17505 3.19319 3.66344 3.38168 3.31291 1.21257 2.75555 3.06614 5.38010 4.08457 98 - -

2.68618 4.42225 2.77519 2.73123 3.46354 2.40513 3.72494 3.29354 2.67741 2.69355 4.24690 2.90347 2.73739 3.18146 2.89801 2.37887 2.77519 2.98518 4.58477 3.61503

0.03394 3.79601 4.51836 0.61958 0.77255 0.48576 0.95510

99 2.92251 4.76322 3.40570 2.93547 4.09844 3.42264 3.77482 3.63647 2.21535 3.16651 4.17059 3.30011 3.93066 3.00062 1.10219 3.01149 3.19482 3.36420 5.21367 4.04432 99 - -

2.68618 4.42225 2.77519 2.73123 3.46354 2.40513 3.72494 3.29354 2.67741 2.69355 4.24690 2.90347 2.73739 3.18146 2.89801 2.37887 2.77519 2.98518 4.58477 3.61503
0.03394 3.79601 4.51836 0.61958 0.77255 0.48576 0.95510
100 3.16380 4.58887 4.13307 3.83471 1.14586 3.86813 3.84824 2.69884 3.74404 2.08761 3.44167 3.93408 4.32984 3.93371 3.88264 3.43660 3.46563 2.68355 4.12324 2.50118 100 - -
2.68618 4.42225 2.77519 2.73123 3.46354 2.40513 3.72494 3.29354 2.67741 2.69355 4.24690 2.90347 2.73739 3.18146 2.89801 2.37887 2.77519 2.98518 4.58477 3.61503
0.03394 3.79601 4.51836 0.61958 0.77255 0.48576 0.95510
101 3.14031 4.66027 3.72681 3.44361 2.30221 3.74216 3.58865 3.16051 3.30332 2.63376 3.84409 3.63185 4.21871 3.62777 3.50199 3.28401 3.42715 3.02772 3.94242 1.10319 101 - -
2.68618 4.42225 2.77519 2.73123 3.46354 2.40513 3.72494 3.29354 2.67741 2.69355 4.24690 2.90347 2.73739 3.18146 2.89801 2.37887 2.77519 2.98518 4.58477 3.61503
0.03394 3.79601 4.51836 0.61958 0.77255 0.48576 0.95510
102 2.57535 4.35609 3.23321 3.12407 4.26351 0.85754 4.24721 3.84618 3.29467 3.50959 4.48627 3.39802 3.74476 3.62525 3.53733 2.74543 3.05073 3.38948 5.37705 4.36350 102 - -
2.68618 4.42225 2.77519 2.73123 3.46354 2.40513 3.72494 3.29354 2.67741 2.69355 4.24690 2.90347 2.73739 3.18146 2.89801 2.37887 2.77519 2.98518 4.58477 3.61503
0.03394 3.79601 4.51836 0.61958 0.77255 0.48576 0.95510
103 2.50546 1.10990 4.00398 3.72767 3.73846 3.14282 4.39180 2.87127 3.55467 2.78939 3.90900 3.73510 3.84371 3.90416 3.69196 2.77663 2.96608 2.62062 5.16879 4.02616 103 - -
2.68618 4.42225 2.77519 2.73123 3.46354 2.40513 3.72494 3.29354 2.67741 2.69355 4.24690 2.90347 2.73739 3.18146 2.89801 2.37887 2.77519 2.98518 4.58477 3.61503
0.03394 3.79601 4.51836 0.61958 0.77255 0.48576 0.95510
104 2.28079 4.19908 3.14317 2.95737 4.00099 2.93053 4.06839 3.51415 3.02585 3.24109 4.17505 3.19319 3.66344 3.38168 3.31291 1.21257 2.75555 3.06614 5.38010 4.08457 104 - -
2.68618 4.42225 2.77519 2.73123 3.46354 2.40513 3.72494 3.29354 2.67741 2.69355 4.24690 2.90347 2.73739 3.18146 2.89801 2.37887 2.77519 2.98518 4.58477 3.61503
0.03394 3.79601 4.51836 0.61958 0.77255 0.48576 0.95510
105 2.66315 4.65392 2.67213 2.55089 4.00571 3.13505 3.86476 3.70148 2.76309 3.35772 4.31495 1.30116 3.78410 3.13903 3.11371 2.73434 3.04351 3.33145 5.32076 3.96081 105 - -
2.68618 4.42225 2.77519 2.73123 3.46354 2.40513 3.72494 3.29354 2.67741 2.69355 4.24690 2.90347 2.73739 3.18146 2.89801 2.37887 2.77519 2.98518 4.58477 3.61503
0.03394 3.79601 4.51836 0.61958 0.77255 0.48576 0.95510
106 2.28079 4.19908 3.14317 2.95737 4.00099 2.93053 4.06839 3.51415 3.02585 3.24109 4.17505 3.19319 3.66344 3.38168 3.31291 1.21257 2.75555 3.06614 5.38010 4.08457 106 - -
2.68618 4.42225 2.77519 2.73123 3.46354 2.40513 3.72494 3.29354 2.67741 2.69355 4.24690 2.90347 2.73739 3.18146 2.89801 2.37887 2.77519 2.98518 4.58477 3.61503
0.03394 3.79601 4.51836 0.61958 0.77255 0.48576 0.95510
107 2.28079 4.19908 3.14317 2.95737 4.00099 2.93053 4.06839 3.51415 3.02585 3.24109 4.17505 3.19319 3.66344 3.38168 3.31291 1.21257 2.75555 3.06614 5.38010 4.08457 107 - -
2.68618 4.42225 2.77519 2.73123 3.46354 2.40513 3.72494 3.29354 2.67741 2.69355 4.24690 2.90347 2.73739 3.18146 2.89801 2.37887 2.77519 2.98518 4.58477 3.61503
0.03394 3.79601 4.51836 0.61958 0.77255 0.48576 0.95510
108 2.28079 4.19908 3.14317 2.95737 4.00099 2.93053 4.06839 3.51415 3.02585 3.24109 4.17505 3.19319 3.66344 3.38168 3.31291 1.21257 2.75555 3.06614 5.38010 4.08457 108 - -
2.68618 4.42225 2.77519 2.73123 3.46354 2.40513 3.72494 3.29354 2.67741 2.69355 4.24690 2.90347 2.73739 3.18146 2.89801 2.37887 2.77519 2.98518 4.58477 3.61503
0.03394 3.79601 4.51836 0.61958 0.77255 0.48576 0.95510
109 2.92251 4.76322 3.40570 2.93547 4.09844 3.42264 3.77482 3.63647 2.21535 3.16651 4.17059 3.30011 3.93066 3.00062 1.10219 3.01149 3.19482 3.36420 5.21367 4.04432 109 - -
2.68618 4.42225 2.77519 2.73123 3.46354 2.40513 3.72494 3.29354 2.67741 2.69355 4.24690 2.90347 2.73739 3.18146 2.89801 2.37887 2.77519 2.98518 4.58477 3.61503
0.03394 3.79601 4.51836 0.61958 0.77255 0.48576 0.95510
110 3.16380 4.58887 4.13307 3.83471 1.14586 3.86813 3.84824 2.69884 3.74404 2.08761 3.44167 3.93408 4.32984 3.93371 3.88264 3.43660 3.46563 2.68355 4.12324 2.50118 110 - -
2.68618 4.42225 2.77519 2.73123 3.46354 2.40513 3.72494 3.29354 2.67741 2.69355 4.24690 2.90347 2.73739 3.18146 2.89801 2.37887 2.77519 2.98518 4.58477 3.61503
0.03394 3.79601 4.51836 0.61958 0.77255 0.48576 0.95510

111 3.07120 4.55728 4.06560 3.70718 3.10996 3.88305 4.38695 2.31238 3.47558 0.97563 3.16058 3.98980 4.33298 3.86233 3.66334 3.44792 3.36975 2.32945 4.95943 3.66445 111 - -
2.68618 4.42225 2.77519 2.73123 3.46354 2.40513 3.72494 3.29354 2.67741 2.69355 4.24690 2.90347 2.73739 3.18146 2.89801 2.37887 2.77519 2.98518 4.58477 3.61503
0.03394 3.79601 4.51836 0.61958 0.77255 0.48576 0.95510
112 2.57535 4.35609 3.23321 3.12407 4.26351 0.85754 4.24721 3.84618 3.29467 3.50959 4.48627 3.39802 3.74476 3.62525 3.53733 2.74543 3.05073 3.38948 5.37705 4.36350 112 - -
2.68618 4.42225 2.77519 2.73123 3.46354 2.40513 3.72494 3.29354 2.67741 2.69355 4.24690 2.90347 2.73739 3.18146 2.89801 2.37887 2.77519 2.98518 4.58477 3.61503
0.03394 3.79601 4.51836 0.61958 0.77255 0.48576 0.95510
113 2.28079 4.19908 3.14317 2.95737 4.00099 2.93053 4.06839 3.51415 3.02585 3.24109 4.17505 3.19319 3.66344 3.38168 3.31291 1.21257 2.75555 3.06614 5.38010 4.08457 113 - -
2.68618 4.42225 2.77519 2.73123 3.46354 2.40513 3.72494 3.29354 2.67741 2.69355 4.24690 2.90347 2.73739 3.18146 2.89801 2.37887 2.77519 2.98518 4.58477 3.61503
0.03394 3.79601 4.51836 0.61958 0.77255 0.48576 0.95510
114 2.28079 4.19908 3.14317 2.95737 4.00099 2.93053 4.06839 3.51415 3.02585 3.24109 4.17505 3.19319 3.66344 3.38168 3.31291 1.21257 2.75555 3.06614 5.38010 4.08457 114 - -
2.68618 4.42225 2.77519 2.73123 3.46354 2.40513 3.72494 3.29354 2.67741 2.69355 4.24690 2.90347 2.73739 3.18146 2.89801 2.37887 2.77519 2.98518 4.58477 3.61503
0.03394 3.79601 4.51836 0.61958 0.77255 0.48576 0.95510
115 2.74814 4.31398 4.01764 3.68039 3.49634 3.64254 4.49596 2.06131 3.54689 2.19820 3.47523 3.88188 4.20873 3.91312 3.76637 3.14284 3.11247 1.12759 5.26714 3.99307 115 - -
2.68618 4.42225 2.77519 2.73123 3.46354 2.40513 3.72494 3.29354 2.67741 2.69355 4.24690 2.90347 2.73739 3.18146 2.89801 2.37887 2.77519 2.98518 4.58477 3.61503
0.03394 3.79601 4.51836 0.61958 0.77255 0.48576 0.95510
116 2.97165 4.41429 4.22289 3.83744 3.32476 3.98045 4.58108 1.17818 3.66390 1.92890 3.27450 4.09357 4.42084 4.02132 3.86934 3.47002 3.27299 1.84997 5.19827 3.92855 116 - -
2.68618 4.42225 2.77519 2.73123 3.46354 2.40513 3.72494 3.29354 2.67741 2.69355 4.24690 2.90347 2.73739 3.18146 2.89801 2.37887 2.77519 2.98518 4.58477 3.61503
0.03394 3.79601 4.51836 0.61958 0.77255 0.48576 0.95510
117 2.37514 4.21936 3.36356 3.11439 3.90452 3.06054 4.13711 3.02248 3.05582 2.88857 3.94573 3.33472 3.75900 3.45502 3.31699 2.57577 1.30864 2.72567 5.34922 4.12174 117 - -
2.68618 4.42225 2.77519 2.73123 3.46354 2.40513 3.72494 3.29354 2.67741 2.69355 4.24690 2.90347 2.73739 3.18146 2.89801 2.37887 2.77519 2.98518 4.58477 3.61503
0.03394 3.79601 4.51836 0.61958 0.77255 0.48576 0.95510
118 2.82402 4.80362 2.85663 2.61241 3.96136 3.31761 3.76927 3.58235 2.43912 3.09213 4.12436 3.07063 3.87138 1.37681 2.72390 2.87605 3.12210 3.31748 5.24814 3.93742 118 - -
2.68618 4.42225 2.77519 2.73123 3.46354 2.40513 3.72494 3.29354 2.67741 2.69355 4.24690 2.90347 2.73739 3.18146 2.89801 2.37887 2.77519 2.98518 4.58477 3.61503
0.03394 3.79601 4.51836 0.61958 0.77255 0.48576 0.95510
119 2.65642 4.42430 3.25570 3.08531 4.08348 3.11624 4.16757 3.61095 3.13637 3.24548 4.29346 3.39875 0.98293 3.52758 3.38897 2.82198 3.09244 3.26489 5.28934 4.20377 119 - -
2.68618 4.42225 2.77519 2.73123 3.46354 2.40513 3.72494 3.29354 2.67741 2.69355 4.24690 2.90347 2.73739 3.18146 2.89801 2.37887 2.77519 2.98518 4.58477 3.61503
0.03394 3.79601 4.51836 0.61958 0.77255 0.48576 0.95510
120 2.91056 5.02139 1.03432 2.29423 4.38899 3.15549 3.86877 3.96045 2.93927 3.58342 4.56179 2.84589 3.80315 3.10925 3.44342 2.88302 3.24824 3.60850 5.57234 4.27708 120 - -
2.68618 4.42225 2.77519 2.73123 3.46354 2.40513 3.72494 3.29354 2.67741 2.69355 4.24690 2.90347 2.73739 3.18146 2.89801 2.37887 2.77519 2.98518 4.58477 3.61503
0.03394 3.79601 4.51836 0.61958 0.77255 0.48576 0.95510
121 2.92251 4.76322 3.40570 2.93547 4.09844 3.42264 3.77482 3.63647 2.21535 3.16651 4.17059 3.30011 3.93066 3.00062 1.10219 3.01149 3.19482 3.36420 5.21367 4.04432 121 - -
2.68618 4.42225 2.77519 2.73123 3.46354 2.40513 3.72494 3.29354 2.67741 2.69355 4.24690 2.90347 2.73739 3.18146 2.89801 2.37887 2.77519 2.98518 4.58477 3.61503
0.03394 3.79601 4.51836 0.61958 0.77255 0.48576 0.95510
122 3.14031 4.66027 3.72681 3.44361 2.30221 3.74216 3.58865 3.16051 3.30332 2.63376 3.84409 3.63185 4.21871 3.62777 3.50199 3.28401 3.42715 3.02772 3.94242 1.10319 122 - -
2.68618 4.42225 2.77519 2.73123 3.46354 2.40513 3.72494 3.29354 2.67741 2.69355 4.24690 2.90347 2.73739 3.18146 2.89801 2.37887 2.77519 2.98518 4.58477 3.61503

0.03394 3.79601 4.51836 0.61958 0.77255 0.48576 0.95510

123 3.07120 4.55728 4.06560 3.70718 3.10996 3.88305 4.38695 2.31238 3.47558 0.97563 3.16058 3.98980 4.33298 3.86233 3.66334 3.44792 3.36975 2.32945 4.95943 3.66445 123 - -

2.68618 4.42225 2.77519 2.73123 3.46354 2.40513 3.72494 3.29354 2.67741 2.69355 4.24690 2.90347 2.73739 3.18146 2.89801 2.37887 2.77519 2.98518 4.58477 3.61503

0.03394 3.79601 4.51836 0.61958 0.77255 0.48576 0.95510

124 2.91157 4.47328 3.96816 3.55750 3.17841 3.77696 4.31044 2.34654 3.32910 1.78626 1.53296 3.84393 4.23864 3.71698 3.54307 3.25192 3.21616 2.34333 5.00802 3.76205 124 - -

2.68618 4.42225 2.77519 2.73123 3.46354 2.40513 3.72494 3.29354 2.67741 2.69355 4.24690 2.90347 2.73739 3.18146 2.89801 2.37887 2.77519 2.98518 4.58477 3.61503

0.03394 3.79601 4.51836 0.61958 0.77255 0.48576 0.95510

125 2.97165 4.41429 4.22289 3.83744 3.32476 3.98045 4.58108 1.17818 3.66390 1.92890 3.27450 4.09357 4.42084 4.02132 3.86934 3.47002 3.27299 1.84997 5.19827 3.92855 125 - -

2.68618 4.42225 2.77519 2.73123 3.46354 2.40513 3.72494 3.29354 2.67741 2.69355 4.24690 2.90347 2.73739 3.18146 2.89801 2.37887 2.77519 2.98518 4.58477 3.61503

0.03394 3.79601 4.51836 0.61958 0.77255 0.48576 0.95510

126 2.74814 4.31398 4.01764 3.68039 3.49634 3.64254 4.49596 2.06131 3.54689 2.19820 3.47523 3.88188 4.20873 3.91312 3.76637 3.14284 3.11247 1.12759 5.26714 3.99307 126 - -

2.68618 4.42225 2.77519 2.73123 3.46354 2.40513 3.72494 3.29354 2.67741 2.69355 4.24690 2.90347 2.73739 3.18146 2.89801 2.37887 2.77519 2.98518 4.58477 3.61503

0.03394 3.79601 4.51836 0.61958 0.77255 0.48576 0.95510

127 2.37514 4.21936 3.36356 3.11439 3.90452 3.06054 4.13711 3.02248 3.05582 2.88857 3.94573 3.33472 3.75900 3.45502 3.31699 2.57577 1.30864 2.72567 5.34922 4.12174 127 - -

2.68618 4.42225 2.77519 2.73123 3.46354 2.40513 3.72494 3.29354 2.67741 2.69355 4.24690 2.90347 2.73739 3.18146 2.89801 2.37887 2.77519 2.98518 4.58477 3.61503

0.03394 3.79601 4.51836 0.61958 0.77255 0.48576 0.95510

128 2.28079 4.19908 3.14317 2.95737 4.00099 2.93053 4.06839 3.51415 3.02585 3.24109 4.17505 3.19319 3.66344 3.38168 3.31291 1.21257 2.75555 3.06614 5.38010 4.08457 128 - -

2.68618 4.42225 2.77519 2.73123 3.46354 2.40513 3.72494 3.29354 2.67741 2.69355 4.24690 2.90347 2.73739 3.18146 2.89801 2.37887 2.77519 2.98518 4.58477 3.61503

0.03394 3.79601 4.51836 0.61958 0.77255 0.48576 0.95510

129 2.57535 4.35609 3.23321 3.12407 4.26351 0.85754 4.24721 3.84618 3.29467 3.50959 4.48627 3.39802 3.74476 3.62525 3.53733 2.74543 3.05073 3.38948 5.37705 4.36350 129 - -

2.68618 4.42225 2.77519 2.73123 3.46354 2.40513 3.72494 3.29354 2.67741 2.69355 4.24690 2.90347 2.73739 3.18146 2.89801 2.37887 2.77519 2.98518 4.58477 3.61503

0.03394 3.79601 4.51836 0.61958 0.77255 0.48576 0.95510

130 2.57535 4.35609 3.23321 3.12407 4.26351 0.85754 4.24721 3.84618 3.29467 3.50959 4.48627 3.39802 3.74476 3.62525 3.53733 2.74543 3.05073 3.38948 5.37705 4.36350 130 - -

2.68618 4.42225 2.77519 2.73123 3.46354 2.40513 3.72494 3.29354 2.67741 2.69355 4.24690 2.90347 2.73739 3.18146 2.89801 2.37887 2.77519 2.98518 4.58477 3.61503

0.03394 3.79601 4.51836 0.61958 0.77255 0.48576 0.95510

131 3.07120 4.55728 4.06560 3.70718 3.10996 3.88305 4.38695 2.31238 3.47558 0.97563 3.16058 3.98980 4.33298 3.86233 3.66334 3.44792 3.36975 2.32945 4.95943 3.66445 131 - -

2.68618 4.42225 2.77519 2.73123 3.46354 2.40513 3.72494 3.29354 2.67741 2.69355 4.24690 2.90347 2.73739 3.18146 2.89801 2.37887 2.77519 2.98518 4.58477 3.61503

0.03394 3.79601 4.51836 0.61958 0.77255 0.48576 0.95510

132 2.66315 4.65392 2.67213 2.55089 4.00571 3.13505 3.86476 3.70148 2.76309 3.35772 4.31495 1.30116 3.78410 3.13903 3.11371 2.73434 3.04351 3.33145 5.32076 3.96081 132 - -

2.68618 4.42225 2.77519 2.73123 3.46354 2.40513 3.72494 3.29354 2.67741 2.69355 4.24690 2.90347 2.73739 3.18146 2.89801 2.37887 2.77519 2.98518 4.58477 3.61503

0.03394 3.79601 4.51836 0.61958 0.77255 0.48576 0.95510

133 2.82402 4.80362 2.85663 2.61241 3.96136 3.31761 3.76927 3.58235 2.43912 3.09213 4.12436 3.07063 3.87138 1.37681 2.72390 2.87605 3.12210 3.31748 5.24814 3.93742 133 - -

2.68618 4.42225 2.77519 2.73123 3.46354 2.40513 3.72494 3.29354 2.67741 2.69355 4.24690 2.90347 2.73739 3.18146 2.89801 2.37887 2.77519 2.98518 4.58477 3.61503

0.03394 3.79601 4.51836 0.61958 0.77255 0.48576 0.95510

134 2.82402 4.80362 2.85663 2.61241 3.96136 3.31761 3.76927 3.58235 2.43912 3.09213 4.12436 3.07063 3.87138 1.37681 2.72390 2.87605 3.12210 3.31748 5.24814 3.93742 134 - -

2.68618 4.42225 2.77519 2.73123 3.46354 2.40513 3.72494 3.29354 2.67741 2.69355 4.24690 2.90347 2.73739 3.18146 2.89801 2.37887 2.77519 2.98518 4.58477 3.61503

0.03394 3.79601 4.51836 0.61958 0.77255 0.48576 0.95510

135 2.92251 4.76322 3.40570 2.93547 4.09844 3.42264 3.77482 3.63647 2.21535 3.16651 4.17059 3.30011 3.93066 3.00062 1.10219 3.01149 3.19482 3.36420 5.21367 4.04432 135 - -

2.68618 4.42225 2.77519 2.73123 3.46354 2.40513 3.72494 3.29354 2.67741 2.69355 4.24690 2.90347 2.73739 3.18146 2.89801 2.37887 2.77519 2.98518 4.58477 3.61503

0.03394 3.79601 4.51836 0.61958 0.77255 0.48576 0.95510

136 2.37514 4.21936 3.36356 3.11439 3.90452 3.06054 4.13711 3.02248 3.05582 2.88857 3.94573 3.33472 3.75900 3.45502 3.31699 2.57577 1.30864 2.72567 5.34922 4.12174 136 - -

2.68618 4.42225 2.77519 2.73123 3.46354 2.40513 3.72494 3.29354 2.67741 2.69355 4.24690 2.90347 2.73739 3.18146 2.89801 2.37887 2.77519 2.98518 4.58477 3.61503

0.03394 3.79601 4.51836 0.61958 0.77255 0.48576 0.95510

137 2.57535 4.35609 3.23321 3.12407 4.26351 0.85754 4.24721 3.84618 3.29467 3.50959 4.48627 3.39802 3.74476 3.62525 3.53733 2.74543 3.05073 3.38948 5.37705 4.36350 137 - -

2.68618 4.42225 2.77519 2.73123 3.46354 2.40513 3.72494 3.29354 2.67741 2.69355 4.24690 2.90347 2.73739 3.18146 2.89801 2.37887 2.77519 2.98518 4.58477 3.61503

0.03394 3.79601 4.51836 0.61958 0.77255 0.48576 0.95510

138 2.97165 4.41429 4.22289 3.83744 3.32476 3.98045 4.58108 1.17818 3.66390 1.92890 3.27450 4.09357 4.42084 4.02132 3.86934 3.47002 3.27299 1.84997 5.19827 3.92855 138 - -

2.68618 4.42225 2.77519 2.73123 3.46354 2.40513 3.72494 3.29354 2.67741 2.69355 4.24690 2.90347 2.73739 3.18146 2.89801 2.37887 2.77519 2.98518 4.58477 3.61503

0.03394 3.79601 4.51836 0.61958 0.77255 0.48576 0.95510

139 2.97165 4.41429 4.22289 3.83744 3.32476 3.98045 4.58108 1.17818 3.66390 1.92890 3.27450 4.09357 4.42084 4.02132 3.86934 3.47002 3.27299 1.84997 5.19827 3.92855 139 - -

2.68618 4.42225 2.77519 2.73123 3.46354 2.40513 3.72494 3.29354 2.67741 2.69355 4.24690 2.90347 2.73739 3.18146 2.89801 2.37887 2.77519 2.98518 4.58477 3.61503

0.03394 3.79601 4.51836 0.61958 0.77255 0.48576 0.95510

140 2.91056 5.02139 1.03432 2.29423 4.38899 3.15549 3.86877 3.96045 2.93927 3.58342 4.56179 2.84589 3.80315 3.10925 3.44342 2.88302 3.24824 3.60850 5.57234 4.27708 140 - -

2.68618 4.42225 2.77519 2.73123 3.46354 2.40513 3.72494 3.29354 2.67741 2.69355 4.24690 2.90347 2.73739 3.18146 2.89801 2.37887 2.77519 2.98518 4.58477 3.61503

0.03394 3.79601 4.51836 0.61958 0.77255 0.48576 0.95510

141 1.13001 4.15738 3.38757 3.17757 3.97857 2.98348 4.20830 3.09294 3.19189 2.97777 4.01983 3.34570 3.71568 3.54062 3.44647 2.50924 2.78140 2.76077 5.41351 4.20565 141 - -

2.68618 4.42225 2.77519 2.73123 3.46354 2.40513 3.72494 3.29354 2.67741 2.69355 4.24690 2.90347 2.73739 3.18146 2.89801 2.37887 2.77519 2.98518 4.58477 3.61503

0.03394 3.79601 4.51836 0.61958 0.77255 0.48576 0.95510

142 2.74814 4.31398 4.01764 3.68039 3.49634 3.64254 4.49596 2.06131 3.54689 2.19820 3.47523 3.88188 4.20873 3.91312 3.76637 3.14284 3.11247 1.12759 5.26714 3.99307 142 - -

2.68618 4.42225 2.77519 2.73123 3.46354 2.40513 3.72494 3.29354 2.67741 2.69355 4.24690 2.90347 2.73739 3.18146 2.89801 2.37887 2.77519 2.98518 4.58477 3.61503

0.03394 3.79601 4.51836 0.61958 0.77255 0.48576 0.95510

143 2.74814 4.31398 4.01764 3.68039 3.49634 3.64254 4.49596 2.06131 3.54689 2.19820 3.47523 3.88188 4.20873 3.91312 3.76637 3.14284 3.11247 1.12759 5.26714 3.99307 143 - -

2.68618 4.42225 2.77519 2.73123 3.46354 2.40513 3.72494 3.29354 2.67741 2.69355 4.24690 2.90347 2.73739 3.18146 2.89801 2.37887 2.77519 2.98518 4.58477 3.61503

0.03394 3.79601 4.51836 0.61958 0.77255 0.48576 0.95510

144 1.13001 4.15738 3.38757 3.17757 3.97857 2.98348 4.20830 3.09294 3.19189 2.97777 4.01983 3.34570 3.71568 3.54062 3.44647 2.50924 2.78140 2.76077 5.41351 4.20565 144 - -

2.68618 4.42225 2.77519 2.73123 3.46354 2.40513 3.72494 3.29354 2.67741 2.69355 4.24690 2.90347 2.73739 3.18146 2.89801 2.37887 2.77519 2.98518 4.58477 3.61503

0.03394 3.79601 4.51836 0.61958 0.77255 0.48576 0.95510

145 1.13001 4.15738 3.38757 3.17757 3.97857 2.98348 4.20830 3.09294 3.19189 2.97777 4.01983 3.34570 3.71568 3.54062 3.44647 2.50924 2.78140 2.76077 5.41351 4.20565 145 - -

2.68618 4.42225 2.77519 2.73123 3.46354 2.40513 3.72494 3.29354 2.67741 2.69355 4.24690 2.90347 2.73739 3.18146 2.89801 2.37887 2.77519 2.98518 4.58477 3.61503

0.03394 3.79601 4.51836 0.61958 0.77255 0.48576 0.95510

146 2.92251 4.76322 3.40570 2.93547 4.09844 3.42264 3.77482 3.63647 2.21535 3.16651 4.17059 3.30011 3.93066 3.00062 1.10219 3.01149 3.19482 3.36420 5.21367 4.04432 146 - -
2.68618 4.42225 2.77519 2.73123 3.46354 2.40513 3.72494 3.29354 2.67741 2.69355 4.24690 2.90347 2.73739 3.18146 2.89801 2.37887 2.77519 2.98518 4.58477 3.61503
0.03394 3.79601 4.51836 0.61958 0.77255 0.48576 0.95510
147 2.97165 4.41429 4.22289 3.83744 3.32476 3.98045 4.58108 1.17818 3.66390 1.92890 3.27450 4.09357 4.42084 4.02132 3.86934 3.47002 3.27299 1.84997 5.19827 3.92855 147 - -
2.68618 4.42225 2.77519 2.73123 3.46354 2.40513 3.72494 3.29354 2.67741 2.69355 4.24690 2.90347 2.73739 3.18146 2.89801 2.37887 2.77519 2.98518 4.58477 3.61503
0.03394 3.79601 4.51836 0.61958 0.77255 0.48576 0.95510
148 3.07120 4.55728 4.06560 3.70718 3.10996 3.88305 4.38695 2.31238 3.47558 0.97563 3.16058 3.98980 4.33298 3.86233 3.66334 3.44792 3.36975 2.32945 4.95943 3.66445 148 - -
2.68618 4.42225 2.77519 2.73123 3.46354 2.40513 3.72494 3.29354 2.67741 2.69355 4.24690 2.90347 2.73739 3.18146 2.89801 2.37887 2.77519 2.98518 4.58477 3.61503
0.03394 3.79601 4.51836 0.61958 0.77255 0.48576 0.95510
149 2.66315 4.65392 2.67213 2.55089 4.00571 3.13505 3.86476 3.70148 2.76309 3.35772 4.31495 1.30116 3.78410 3.13903 3.11371 2.73434 3.04351 3.33145 5.32076 3.96081 149 - -
2.68618 4.42225 2.77519 2.73123 3.46354 2.40513 3.72494 3.29354 2.67741 2.69355 4.24690 2.90347 2.73739 3.18146 2.89801 2.37887 2.77519 2.98518 4.58477 3.61503
0.03394 3.79601 4.51836 0.61958 0.77255 0.48576 0.95510
150 1.13001 4.15738 3.38757 3.17757 3.97857 2.98348 4.20830 3.09294 3.19189 2.97777 4.01983 3.34570 3.71568 3.54062 3.44647 2.50924 2.78140 2.76077 5.41351 4.20565 150 - -
2.68618 4.42225 2.77519 2.73123 3.46354 2.40513 3.72494 3.29354 2.67741 2.69355 4.24690 2.90347 2.73739 3.18146 2.89801 2.37887 2.77519 2.98518 4.58477 3.61503
0.03394 3.79601 4.51836 0.61958 0.77255 0.48576 0.95510
151 2.37514 4.21936 3.36356 3.11439 3.90452 3.06054 4.13711 3.02248 3.05582 2.88857 3.94573 3.33472 3.75900 3.45502 3.31699 2.57577 1.30864 2.72567 5.34922 4.12174 151 - -
2.68618 4.42225 2.77519 2.73123 3.46354 2.40513 3.72494 3.29354 2.67741 2.69355 4.24690 2.90347 2.73739 3.18146 2.89801 2.37887 2.77519 2.98518 4.58477 3.61503
0.03394 3.79601 4.51836 0.61958 0.77255 0.48576 0.95510
152 3.07120 4.55728 4.06560 3.70718 3.10996 3.88305 4.38695 2.31238 3.47558 0.97563 3.16058 3.98980 4.33298 3.86233 3.66334 3.44792 3.36975 2.32945 4.95943 3.66445 152 - -
2.68618 4.42225 2.77519 2.73123 3.46354 2.40513 3.72494 3.29354 2.67741 2.69355 4.24690 2.90347 2.73739 3.18146 2.89801 2.37887 2.77519 2.98518 4.58477 3.61503
0.03394 3.79601 4.51836 0.61958 0.77255 0.48576 0.95510
153 2.74814 4.31398 4.01764 3.68039 3.49634 3.64254 4.49596 2.06131 3.54689 2.19820 3.47523 3.88188 4.20873 3.91312 3.76637 3.14284 3.11247 1.12759 5.26714 3.99307 153 - -
2.68618 4.42225 2.77519 2.73123 3.46354 2.40513 3.72494 3.29354 2.67741 2.69355 4.24690 2.90347 2.73739 3.18146 2.89801 2.37887 2.77519 2.98518 4.58477 3.61503
0.03394 3.79601 4.51836 0.61958 0.77255 0.48576 0.95510
154 2.74814 4.31398 4.01764 3.68039 3.49634 3.64254 4.49596 2.06131 3.54689 2.19820 3.47523 3.88188 4.20873 3.91312 3.76637 3.14284 3.11247 1.12759 5.26714 3.99307 154 - -
2.68618 4.42225 2.77519 2.73123 3.46354 2.40513 3.72494 3.29354 2.67741 2.69355 4.24690 2.90347 2.73739 3.18146 2.89801 2.37887 2.77519 2.98518 4.58477 3.61503
0.03394 3.79601 4.51836 0.61958 0.77255 0.48576 0.95510
155 2.65642 4.42430 3.25570 3.08531 4.08348 3.11624 4.16757 3.61095 3.13637 3.24548 4.29346 3.39875 0.98293 3.52758 3.38897 2.82198 3.09244 3.26489 5.28934 4.20377 155 - -
2.68618 4.42225 2.77519 2.73123 3.46354 2.40513 3.72494 3.29354 2.67741 2.69355 4.24690 2.90347 2.73739 3.18146 2.89801 2.37887 2.77519 2.98518 4.58477 3.61503
0.03394 3.79601 4.51836 0.61958 0.77255 0.48576 0.95510
156 2.90487 4.86160 3.07558 2.71397 4.23048 3.40048 3.72190 3.65540 1.14833 3.23828 4.20575 3.14113 3.90783 2.91915 2.42439 2.95301 3.16487 3.36817 5.31817 4.12060 156 - -
2.68618 4.42225 2.77519 2.73123 3.46354 2.40513 3.72494 3.29354 2.67741 2.69355 4.24690 2.90347 2.73739 3.18146 2.89801 2.37887 2.77519 2.98518 4.58477 3.61503
0.03394 3.79601 4.51836 0.61958 0.77255 0.48576 0.95510
157 3.07120 4.55728 4.06560 3.70718 3.10996 3.88305 4.38695 2.31238 3.47558 0.97563 3.16058 3.98980 4.33298 3.86233 3.66334 3.44792 3.36975 2.32945 4.95943 3.66445 157 - -
2.68618 4.42225 2.77519 2.73123 3.46354 2.40513 3.72494 3.29354 2.67741 2.69355 4.24690 2.90347 2.73739 3.18146 2.89801 2.37887 2.77519 2.98518 4.58477 3.61503

0.03394 3.79601 4.51836 0.61958 0.77255 0.48576 0.95510
158 2.91056 5.02139 1.03432 2.29423 4.38899 3.15549 3.86877 3.96045 2.93927 3.58342 4.56179 2.84589 3.80315 3.10925 3.44342 2.88302 3.24824 3.60850 5.57234 4.27708 158 - -
2.68618 4.42225 2.77519 2.73123 3.46354 2.40513 3.72494 3.29354 2.67741 2.69355 4.24690 2.90347 2.73739 3.18146 2.89801 2.37887 2.77519 2.98518 4.58477 3.61503
0.03394 3.79601 4.51836 0.61958 0.77255 0.48576 0.95510
159 2.82402 4.80362 2.85663 2.61241 3.96136 3.31761 3.76927 3.58235 2.43912 3.09213 4.12436 3.07063 3.87138 1.37681 2.72390 2.87605 3.12210 3.31748 5.24814 3.93742 159 - -
2.68618 4.42225 2.77519 2.73123 3.46354 2.40513 3.72494 3.29354 2.67741 2.69355 4.24690 2.90347 2.73739 3.18146 2.89801 2.37887 2.77519 2.98518 4.58477 3.61503
0.03394 3.79601 4.51836 0.61958 0.77255 0.48576 0.95510
160 2.37514 4.21936 3.36356 3.11439 3.90452 3.06054 4.13711 3.02248 3.05582 2.88857 3.94573 3.33472 3.75900 3.45502 3.31699 2.57577 1.30864 2.72567 5.34922 4.12174 160 - -
2.68618 4.42225 2.77519 2.73123 3.46354 2.40513 3.72494 3.29354 2.67741 2.69355 4.24690 2.90347 2.73739 3.18146 2.89801 2.37887 2.77519 2.98518 4.58477 3.61503
0.03394 3.79601 4.51836 0.61958 0.77255 0.48576 0.95510
161 2.28079 4.19908 3.14317 2.95737 4.00099 2.93053 4.06839 3.51415 3.02585 3.24109 4.17505 3.19319 3.66344 3.38168 3.31291 1.21257 2.75555 3.06614 5.38010 4.08457 161 - -
2.68618 4.42225 2.77519 2.73123 3.46354 2.40513 3.72494 3.29354 2.67741 2.69355 4.24690 2.90347 2.73739 3.18146 2.89801 2.37887 2.77519 2.98518 4.58477 3.61503
0.03394 3.79601 4.51836 0.61958 0.77255 0.48576 0.95510
162 3.16380 4.58887 4.13307 3.83471 1.14586 3.86813 3.84824 2.69884 3.74404 2.08761 3.44167 3.93408 4.32984 3.93371 3.88264 3.43660 3.46563 2.68355 4.12324 2.50118 162 - -
2.68618 4.42225 2.77519 2.73123 3.46354 2.40513 3.72494 3.29354 2.67741 2.69355 4.24690 2.90347 2.73739 3.18146 2.89801 2.37887 2.77519 2.98518 4.58477 3.61503
0.03394 3.79601 4.51836 0.61958 0.77255 0.48576 0.95510
163 3.21457 4.61548 3.97805 3.66660 2.59647 3.64757 3.81244 3.23661 3.39107 2.64390 3.87379 3.83150 4.15944 3.78287 3.53232 3.41887 3.51155 3.12484 1.05487 2.60290 163 - -
2.68618 4.42225 2.77519 2.73123 3.46354 2.40513 3.72494 3.29354 2.67741 2.69355 4.24690 2.90347 2.73739 3.18146 2.89801 2.37887 2.77519 2.98518 4.58477 3.61503
0.03394 3.79601 4.51836 0.61958 0.77255 0.48576 0.95510
164 2.90487 4.86160 3.07558 2.71397 4.23048 3.40048 3.72190 3.65540 1.14833 3.23828 4.20575 3.14113 3.90783 2.91915 2.42439 2.95301 3.16487 3.36817 5.31817 4.12060 164 - -
2.68618 4.42225 2.77519 2.73123 3.46354 2.40513 3.72494 3.29354 2.67741 2.69355 4.24690 2.90347 2.73739 3.18146 2.89801 2.37887 2.77519 2.98518 4.58477 3.61503
0.03394 3.79601 4.51836 0.61958 0.77255 0.48576 0.95510
165 2.91056 5.02139 1.03432 2.29423 4.38899 3.15549 3.86877 3.96045 2.93927 3.58342 4.56179 2.84589 3.80315 3.10925 3.44342 2.88302 3.24824 3.60850 5.57234 4.27708 165 - -
2.68618 4.42225 2.77519 2.73123 3.46354 2.40513 3.72494 3.29354 2.67741 2.69355 4.24690 2.90347 2.73739 3.18146 2.89801 2.37887 2.77519 2.98518 4.58477 3.61503
0.03394 3.79601 4.51836 0.61958 0.77255 0.48576 0.95510
166 1.13001 4.15738 3.38757 3.17757 3.97857 2.98348 4.20830 3.09294 3.19189 2.97777 4.01983 3.34570 3.71568 3.54062 3.44647 2.50924 2.78140 2.76077 5.41351 4.20565 166 - -
2.68618 4.42225 2.77519 2.73123 3.46354 2.40513 3.72494 3.29354 2.67741 2.69355 4.24690 2.90347 2.73739 3.18146 2.89801 2.37887 2.77519 2.98518 4.58477 3.61503
0.03394 3.79601 4.51836 0.61958 0.77255 0.48576 0.95510
167 2.28079 4.19908 3.14317 2.95737 4.00099 2.93053 4.06839 3.51415 3.02585 3.24109 4.17505 3.19319 3.66344 3.38168 3.31291 1.21257 2.75555 3.06614 5.38010 4.08457 167 - -
2.68618 4.42225 2.77519 2.73123 3.46354 2.40513 3.72494 3.29354 2.67741 2.69355 4.24690 2.90347 2.73739 3.18146 2.89801 2.37887 2.77519 2.98518 4.58477 3.61503
0.03394 3.79601 4.51836 0.61958 0.77255 0.48576 0.95510
168 2.66315 4.65392 2.67213 2.55089 4.00571 3.13505 3.86476 3.70148 2.76309 3.35772 4.31495 1.30116 3.78410 3.13903 3.11371 2.73434 3.04351 3.33145 5.32076 3.96081 168 - -
2.68618 4.42225 2.77519 2.73123 3.46354 2.40513 3.72494 3.29354 2.67741 2.69355 4.24690 2.90347 2.73739 3.18146 2.89801 2.37887 2.77519 2.98518 4.58477 3.61503
0.03394 3.79601 4.51836 0.61958 0.77255 0.48576 0.95510
169 3.16380 4.58887 4.13307 3.83471 1.14586 3.86813 3.84824 2.69884 3.74404 2.08761 3.44167 3.93408 4.32984 3.93371 3.88264 3.43660 3.46563 2.68355 4.12324 2.50118 169 - -

2.68618 4.42225 2.77519 2.73123 3.46354 2.40513 3.72494 3.29354 2.67741 2.69355 4.24690 2.90347 2.73739 3.18146 2.89801 2.37887 2.77519 2.98518 4.58477 3.61503

0.03394 3.79601 4.51836 0.61958 0.77255 0.48576 0.95510

170 2.28079 4.19908 3.14317 2.95737 4.00099 2.93053 4.06839 3.51415 3.02585 3.24109 4.17505 3.19319 3.66344 3.38168 3.31291 1.21257 2.75555 3.06614 5.38010 4.08457 170 - -

2.68618 4.42225 2.77519 2.73123 3.46354 2.40513 3.72494 3.29354 2.67741 2.69355 4.24690 2.90347 2.73739 3.18146 2.89801 2.37887 2.77519 2.98518 4.58477 3.61503

0.03394 3.79601 4.51836 0.61958 0.77255 0.48576 0.95510

171 2.89015 4.98001 2.46169 1.13957 4.31725 3.22729 3.81015 3.75600 2.68126 3.38645 4.36600 2.89555 3.82889 3.02881 3.08050 2.88174 3.19454 3.44858 5.50348 4.22803 171 - -

2.68618 4.42225 2.77519 2.73123 3.46354 2.40513 3.72494 3.29354 2.67741 2.69355 4.24690 2.90347 2.73739 3.18146 2.89801 2.37887 2.77519 2.98518 4.58477 3.61503

0.03394 3.79601 4.51836 0.61958 0.77255 0.48576 0.95510

172 2.97165 4.41429 4.22289 3.83744 3.32476 3.98045 4.58108 1.17818 3.66390 1.92890 3.27450 4.09357 4.42084 4.02132 3.86934 3.47002 3.27299 1.84997 5.19827 3.92855 172 - -

2.68618 4.42225 2.77519 2.73123 3.46354 2.40513 3.72494 3.29354 2.67741 2.69355 4.24690 2.90347 2.73739 3.18146 2.89801 2.37887 2.77519 2.98518 4.58477 3.61503

0.03394 3.79601 4.51836 0.61958 0.77255 0.48576 0.95510

173 3.16380 4.58887 4.13307 3.83471 1.14586 3.86813 3.84824 2.69884 3.74404 2.08761 3.44167 3.93408 4.32984 3.93371 3.88264 3.43660 3.46563 2.68355 4.12324 2.50118 173 - -

2.68618 4.42225 2.77519 2.73123 3.46354 2.40513 3.72494 3.29354 2.67741 2.69355 4.24690 2.90347 2.73739 3.18146 2.89801 2.37887 2.77519 2.98518 4.58477 3.61503

0.03394 3.79601 4.51836 0.61958 0.77255 0.48576 0.95510

174 2.91056 5.02139 1.03432 2.29423 4.38899 3.15549 3.86877 3.96045 2.93927 3.58342 4.56179 2.84589 3.80315 3.10925 3.44342 2.88302 3.24824 3.60850 5.57234 4.27708 174 - -

2.68618 4.42225 2.77519 2.73123 3.46354 2.40513 3.72494 3.29354 2.67741 2.69355 4.24690 2.90347 2.73739 3.18146 2.89801 2.37887 2.77519 2.98518 4.58477 3.61503

0.03394 3.79601 4.51836 0.61958 0.77255 0.48576 0.95510

175 2.74814 4.31398 4.01764 3.68039 3.49634 3.64254 4.49596 2.06131 3.54689 2.19820 3.47523 3.88188 4.20873 3.91312 3.76637 3.14284 3.11247 1.12759 5.26714 3.99307 175 - -

2.68618 4.42225 2.77519 2.73123 3.46354 2.40513 3.72494 3.29354 2.67741 2.69355 4.24690 2.90347 2.73739 3.18146 2.89801 2.37887 2.77519 2.98518 4.58477 3.61503

0.03394 3.79601 4.51836 0.61958 0.77255 0.48576 0.95510

176 2.91056 5.02139 1.03432 2.29423 4.38899 3.15549 3.86877 3.96045 2.93927 3.58342 4.56179 2.84589 3.80315 3.10925 3.44342 2.88302 3.24824 3.60850 5.57234 4.27708 176 - -

2.68618 4.42225 2.77519 2.73123 3.46354 2.40513 3.72494 3.29354 2.67741 2.69355 4.24690 2.90347 2.73739 3.18146 2.89801 2.37887 2.77519 2.98518 4.58477 3.61503

0.03394 3.79601 4.51836 0.61958 0.77255 0.48576 0.95510

177 3.21457 4.61548 3.97805 3.66660 2.59647 3.64757 3.81244 3.23661 3.39107 2.64390 3.87379 3.83150 4.15944 3.78287 3.53232 3.41887 3.51155 3.12484 1.05487 2.60290 177 - -

2.68618 4.42225 2.77519 2.73123 3.46354 2.40513 3.72494 3.29354 2.67741 2.69355 4.24690 2.90347 2.73739 3.18146 2.89801 2.37887 2.77519 2.98518 4.58477 3.61503

0.03394 3.79601 4.51836 0.61958 0.77255 0.48576 0.95510

178 3.16380 4.58887 4.13307 3.83471 1.14586 3.86813 3.84824 2.69884 3.74404 2.08761 3.44167 3.93408 4.32984 3.93371 3.88264 3.43660 3.46563 2.68355 4.12324 2.50118 178 - -

2.68618 4.42225 2.77519 2.73123 3.46354 2.40513 3.72494 3.29354 2.67741 2.69355 4.24690 2.90347 2.73739 3.18146 2.89801 2.37887 2.77519 2.98518 4.58477 3.61503

0.03394 3.79601 4.51836 0.61958 0.77255 0.48576 0.95510

179 2.97165 4.41429 4.22289 3.83744 3.32476 3.98045 4.58108 1.17818 3.66390 1.92890 3.27450 4.09357 4.42084 4.02132 3.86934 3.47002 3.27299 1.84997 5.19827 3.92855 179 - -

2.68618 4.42225 2.77519 2.73123 3.46354 2.40513 3.72494 3.29354 2.67741 2.69355 4.24690 2.90347 2.73739 3.18146 2.89801 2.37887 2.77519 2.98518 4.58477 3.61503

0.03394 3.79601 4.51836 0.61958 0.77255 0.48576 0.95510

180 2.28079 4.19908 3.14317 2.95737 4.00099 2.93053 4.06839 3.51415 3.02585 3.24109 4.17505 3.19319 3.66344 3.38168 3.31291 1.21257 2.75555 3.06614 5.38010 4.08457 180 - -

2.68618 4.42225 2.77519 2.73123 3.46354 2.40513 3.72494 3.29354 2.67741 2.69355 4.24690 2.90347 2.73739 3.18146 2.89801 2.37887 2.77519 2.98518 4.58477 3.61503

0.03394 3.79601 4.51836 0.61958 0.77255 0.48576 0.95510

181 2.66315 4.65392 2.67213 2.55089 4.00571 3.13505 3.86476 3.70148 2.76309 3.35772 4.31495 1.30116 3.78410 3.13903 3.11371 2.73434 3.04351 3.33145 5.32076 3.96081 181 - -
2.68618 4.42225 2.77519 2.73123 3.46354 2.40513 3.72494 3.29354 2.67741 2.69355 4.24690 2.90347 2.73739 3.18146 2.89801 2.37887 2.77519 2.98518 4.58477 3.61503
0.03394 3.79601 4.51836 0.61958 0.77255 0.48576 0.95510
182 3.07120 4.55728 4.06560 3.70718 3.10996 3.88305 4.38695 2.31238 3.47558 0.97563 3.16058 3.98980 4.33298 3.86233 3.66334 3.44792 3.36975 2.32945 4.95943 3.66445 182 - -
2.68618 4.42225 2.77519 2.73123 3.46354 2.40513 3.72494 3.29354 2.67741 2.69355 4.24690 2.90347 2.73739 3.18146 2.89801 2.37887 2.77519 2.98518 4.58477 3.61503
0.03394 3.79601 4.51836 0.61958 0.77255 0.48576 0.95510
183 2.28079 4.19908 3.14317 2.95737 4.00099 2.93053 4.06839 3.51415 3.02585 3.24109 4.17505 3.19319 3.66344 3.38168 3.31291 1.21257 2.75555 3.06614 5.38010 4.08457 183 - -
2.68618 4.42225 2.77519 2.73123 3.46354 2.40513 3.72494 3.29354 2.67741 2.69355 4.24690 2.90347 2.73739 3.18146 2.89801 2.37887 2.77519 2.98518 4.58477 3.61503
0.03394 3.79601 4.51836 0.61958 0.77255 0.48576 0.95510
184 2.90487 4.86160 3.07558 2.71397 4.23048 3.40048 3.72190 3.65540 1.14833 3.23828 4.20575 3.14113 3.90783 2.91915 2.42439 2.95301 3.16487 3.36817 5.31817 4.12060 184 - -
2.68618 4.42225 2.77519 2.73123 3.46354 2.40513 3.72494 3.29354 2.67741 2.69355 4.24690 2.90347 2.73739 3.18146 2.89801 2.37887 2.77519 2.98518 4.58477 3.61503
0.03394 3.79601 4.51836 0.61958 0.77255 0.48576 0.95510
185 2.91056 5.02139 1.03432 2.29423 4.38899 3.15549 3.86877 3.96045 2.93927 3.58342 4.56179 2.84589 3.80315 3.10925 3.44342 2.88302 3.24824 3.60850 5.57234 4.27708 185 - -
2.68618 4.42225 2.77519 2.73123 3.46354 2.40513 3.72494 3.29354 2.67741 2.69355 4.24690 2.90347 2.73739 3.18146 2.89801 2.37887 2.77519 2.98518 4.58477 3.61503
0.03394 3.79601 4.51836 0.61958 0.77255 0.48576 0.95510
186 2.74814 4.31398 4.01764 3.68039 3.49634 3.64254 4.49596 2.06131 3.54689 2.19820 3.47523 3.88188 4.20873 3.91312 3.76637 3.14284 3.11247 1.12759 5.26714 3.99307 186 - -
2.68618 4.42225 2.77519 2.73123 3.46354 2.40513 3.72494 3.29354 2.67741 2.69355 4.24690 2.90347 2.73739 3.18146 2.89801 2.37887 2.77519 2.98518 4.58477 3.61503
0.03394 3.79601 4.51836 0.61958 0.77255 0.48576 0.95510
187 2.90487 4.86160 3.07558 2.71397 4.23048 3.40048 3.72190 3.65540 1.14833 3.23828 4.20575 3.14113 3.90783 2.91915 2.42439 2.95301 3.16487 3.36817 5.31817 4.12060 187 - -
2.68618 4.42225 2.77519 2.73123 3.46354 2.40513 3.72494 3.29354 2.67741 2.69355 4.24690 2.90347 2.73739 3.18146 2.89801 2.37887 2.77519 2.98518 4.58477 3.61503
0.03394 3.79601 4.51836 0.61958 0.77255 0.48576 0.95510
188 2.97165 4.41429 4.22289 3.83744 3.32476 3.98045 4.58108 1.17818 3.66390 1.92890 3.27450 4.09357 4.42084 4.02132 3.86934 3.47002 3.27299 1.84997 5.19827 3.92855 188 - -
2.68618 4.42225 2.77519 2.73123 3.46354 2.40513 3.72494 3.29354 2.67741 2.69355 4.24690 2.90347 2.73739 3.18146 2.89801 2.37887 2.77519 2.98518 4.58477 3.61503
0.03394 3.79601 4.51836 0.61958 0.77255 0.48576 0.95510
189 2.74814 4.31398 4.01764 3.68039 3.49634 3.64254 4.49596 2.06131 3.54689 2.19820 3.47523 3.88188 4.20873 3.91312 3.76637 3.14284 3.11247 1.12759 5.26714 3.99307 189 - -
2.68618 4.42225 2.77519 2.73123 3.46354 2.40513 3.72494 3.29354 2.67741 2.69355 4.24690 2.90347 2.73739 3.18146 2.89801 2.37887 2.77519 2.98518 4.58477 3.61503
0.03394 3.79601 4.51836 0.61958 0.77255 0.48576 0.95510
190 2.90487 4.86160 3.07558 2.71397 4.23048 3.40048 3.72190 3.65540 1.14833 3.23828 4.20575 3.14113 3.90783 2.91915 2.42439 2.95301 3.16487 3.36817 5.31817 4.12060 190 - -
2.68618 4.42225 2.77519 2.73123 3.46354 2.40513 3.72494 3.29354 2.67741 2.69355 4.24690 2.90347 2.73739 3.18146 2.89801 2.37887 2.77519 2.98518 4.58477 3.61503
0.03394 3.79601 4.51836 0.61958 0.77255 0.48576 0.95510
191 2.89015 4.98001 2.46169 1.13957 4.31725 3.22729 3.81015 3.75600 2.68126 3.38645 4.36600 2.89555 3.82889 3.02881 3.08050 2.88174 3.19454 3.44858 5.50348 4.22803 191 - -
2.68618 4.42225 2.77519 2.73123 3.46354 2.40513 3.72494 3.29354 2.67741 2.69355 4.24690 2.90347 2.73739 3.18146 2.89801 2.37887 2.77519 2.98518 4.58477 3.61503
0.03394 3.79601 4.51836 0.61958 0.77255 0.48576 0.95510
192 3.07120 4.55728 4.06560 3.70718 3.10996 3.88305 4.38695 2.31238 3.47558 0.97563 3.16058 3.98980 4.33298 3.86233 3.66334 3.44792 3.36975 2.32945 4.95943 3.66445 192 - -
2.68618 4.42225 2.77519 2.73123 3.46354 2.40513 3.72494 3.29354 2.67741 2.69355 4.24690 2.90347 2.73739 3.18146 2.89801 2.37887 2.77519 2.98518 4.58477 3.61503

0.03394 3.79601 4.51836 0.61958 0.77255 0.48576 0.95510

193 2.65642 4.42430 3.25570 3.08531 4.08348 3.11624 4.16757 3.61095 3.13637 3.24548 4.29346 3.39875 0.98293 3.52758 3.38897 2.82198 3.09244 3.26489 5.28934 4.20377 193 - -

2.68618 4.42225 2.77519 2.73123 3.46354 2.40513 3.72494 3.29354 2.67741 2.69355 4.24690 2.90347 2.73739 3.18146 2.89801 2.37887 2.77519 2.98518 4.58477 3.61503

0.03394 3.79601 4.51836 0.61958 0.77255 0.48576 0.95510

194 2.89015 4.98001 2.46169 1.13957 4.31725 3.22729 3.81015 3.75600 2.68126 3.38645 4.36600 2.89555 3.82889 3.02881 3.08050 2.88174 3.19454 3.44858 5.50348 4.22803 194 - -

2.68618 4.42225 2.77519 2.73123 3.46354 2.40513 3.72494 3.29354 2.67741 2.69355 4.24690 2.90347 2.73739 3.18146 2.89801 2.37887 2.77519 2.98518 4.58477 3.61503

0.03394 3.79601 4.51836 0.61958 0.77255 0.48576 0.95510

195 2.97165 4.41429 4.22289 3.83744 3.32476 3.98045 4.58108 1.17818 3.66390 1.92890 3.27450 4.09357 4.42084 4.02132 3.86934 3.47002 3.27299 1.84997 5.19827 3.92855 195 - -

2.68618 4.42225 2.77519 2.73123 3.46354 2.40513 3.72494 3.29354 2.67741 2.69355 4.24690 2.90347 2.73739 3.18146 2.89801 2.37887 2.77519 2.98518 4.58477 3.61503

0.03394 3.79601 4.51836 0.61958 0.77255 0.48576 0.95510

196 2.57535 4.35609 3.23321 3.12407 4.26351 0.85754 4.24721 3.84618 3.29467 3.50959 4.48627 3.39802 3.74476 3.62525 3.53733 2.74543 3.05073 3.38948 5.37705 4.36350 196 - -

2.68618 4.42225 2.77519 2.73123 3.46354 2.40513 3.72494 3.29354 2.67741 2.69355 4.24690 2.90347 2.73739 3.18146 2.89801 2.37887 2.77519 2.98518 4.58477 3.61503

0.03394 3.79601 4.51836 0.61958 0.77255 0.48576 0.95510

197 2.57535 4.35609 3.23321 3.12407 4.26351 0.85754 4.24721 3.84618 3.29467 3.50959 4.48627 3.39802 3.74476 3.62525 3.53733 2.74543 3.05073 3.38948 5.37705 4.36350 197 - -

2.68618 4.42225 2.77519 2.73123 3.46354 2.40513 3.72494 3.29354 2.67741 2.69355 4.24690 2.90347 2.73739 3.18146 2.89801 2.37887 2.77519 2.98518 4.58477 3.61503

0.03394 3.79601 4.51836 0.61958 0.77255 0.48576 0.95510

198 2.90487 4.86160 3.07558 2.71397 4.23048 3.40048 3.72190 3.65540 1.14833 3.23828 4.20575 3.14113 3.90783 2.91915 2.42439 2.95301 3.16487 3.36817 5.31817 4.12060 198 - -

2.68618 4.42225 2.77519 2.73123 3.46354 2.40513 3.72494 3.29354 2.67741 2.69355 4.24690 2.90347 2.73739 3.18146 2.89801 2.37887 2.77519 2.98518 4.58477 3.61503

0.03394 3.79601 4.51836 0.61958 0.77255 0.48576 0.95510

199 3.07120 4.55728 4.06560 3.70718 3.10996 3.88305 4.38695 2.31238 3.47558 0.97563 3.16058 3.98980 4.33298 3.86233 3.66334 3.44792 3.36975 2.32945 4.95943 3.66445 199 - -

2.68618 4.42225 2.77519 2.73123 3.46354 2.40513 3.72494 3.29354 2.67741 2.69355 4.24690 2.90347 2.73739 3.18146 2.89801 2.37887 2.77519 2.98518 4.58477 3.61503

0.03394 3.79601 4.51836 0.61958 0.77255 0.48576 0.95510

200 2.92251 4.76322 3.40570 2.93547 4.09844 3.42264 3.77482 3.63647 2.21535 3.16651 4.17059 3.30011 3.93066 3.00062 1.10219 3.01149 3.19482 3.36420 5.21367 4.04432 200 - -

2.68618 4.42225 2.77519 2.73123 3.46354 2.40513 3.72494 3.29354 2.67741 2.69355 4.24690 2.90347 2.73739 3.18146 2.89801 2.37887 2.77519 2.98518 4.58477 3.61503

0.03394 3.79601 4.51836 0.61958 0.77255 0.48576 0.95510

201 2.37514 4.21936 3.36356 3.11439 3.90452 3.06054 4.13711 3.02248 3.05582 2.88857 3.94573 3.33472 3.75900 3.45502 3.31699 2.57577 1.30864 2.72567 5.34922 4.12174 201 - -

2.68618 4.42225 2.77519 2.73123 3.46354 2.40513 3.72494 3.29354 2.67741 2.69355 4.24690 2.90347 2.73739 3.18146 2.89801 2.37887 2.77519 2.98518 4.58477 3.61503

0.03394 3.79601 4.51836 0.61958 0.77255 0.48576 0.95510

202 2.65642 4.42430 3.25570 3.08531 4.08348 3.11624 4.16757 3.61095 3.13637 3.24548 4.29346 3.39875 0.98293 3.52758 3.38897 2.82198 3.09244 3.26489 5.28934 4.20377 202 - -

2.68618 4.42225 2.77519 2.73123 3.46354 2.40513 3.72494 3.29354 2.67741 2.69355 4.24690 2.90347 2.73739 3.18146 2.89801 2.37887 2.77519 2.98518 4.58477 3.61503

0.03394 3.79601 4.51836 0.61958 0.77255 0.48576 0.95510

203 2.91724 4.74509 3.05672 2.81086 3.23540 3.38653 1.31468 3.60804 2.65623 3.11092 4.14942 3.22253 3.93587 3.17665 2.92559 2.98918 3.21880 3.34482 4.70119 3.18882 203 - -

2.68618 4.42225 2.77519 2.73123 3.46354 2.40513 3.72494 3.29354 2.67741 2.69355 4.24690 2.90347 2.73739 3.18146 2.89801 2.37887 2.77519 2.98518 4.58477 3.61503

0.03394 3.79601 4.51836 0.61958 0.77255 0.48576 0.95510

204 2.92251 4.76322 3.40570 2.93547 4.09844 3.42264 3.77482 3.63647 2.21535 3.16651 4.17059 3.30011 3.93066 3.00062 1.10219 3.01149 3.19482 3.36420 5.21367 4.04432 204 - -

2.68618 4.42225 2.77519 2.73123 3.46354 2.40513 3.72494 3.29354 2.67741 2.69355 4.24690 2.90347 2.73739 3.18146 2.89801 2.37887 2.77519 2.98518 4.58477 3.61503

0.03394 3.79601 4.51836 0.61958 0.77255 0.48576 0.95510

205 2.91157 4.47328 3.96816 3.55750 3.17841 3.77696 4.31044 2.34654 3.32910 1.78626 1.53296 3.84393 4.23864 3.71698 3.54307 3.25192 3.21616 2.34333 5.00802 3.76205 205 - -

2.68618 4.42225 2.77519 2.73123 3.46354 2.40513 3.72494 3.29354 2.67741 2.69355 4.24690 2.90347 2.73739 3.18146 2.89801 2.37887 2.77519 2.98518 4.58477 3.61503

0.03394 3.79601 4.51836 0.61958 0.77255 0.48576 0.95510

206 2.92251 4.76322 3.40570 2.93547 4.09844 3.42264 3.77482 3.63647 2.21535 3.16651 4.17059 3.30011 3.93066 3.00062 1.10219 3.01149 3.19482 3.36420 5.21367 4.04432 206 - -

2.68618 4.42225 2.77519 2.73123 3.46354 2.40513 3.72494 3.29354 2.67741 2.69355 4.24690 2.90347 2.73739 3.18146 2.89801 2.37887 2.77519 2.98518 4.58477 3.61503

0.03394 3.79601 4.51836 0.61958 0.77255 0.48576 0.95510

207 2.74814 4.31398 4.01764 3.68039 3.49634 3.64254 4.49596 2.06131 3.54689 2.19820 3.47523 3.88188 4.20873 3.91312 3.76637 3.14284 3.11247 1.12759 5.26714 3.99307 207 - -

2.68618 4.42225 2.77519 2.73123 3.46354 2.40513 3.72494 3.29354 2.67741 2.69355 4.24690 2.90347 2.73739 3.18146 2.89801 2.37887 2.77519 2.98518 4.58477 3.61503

0.03394 3.79601 4.51836 0.61958 0.77255 0.48576 0.95510

208 2.65642 4.42430 3.25570 3.08531 4.08348 3.11624 4.16757 3.61095 3.13637 3.24548 4.29346 3.39875 0.98293 3.52758 3.38897 2.82198 3.09244 3.26489 5.28934 4.20377 208 - -

2.68618 4.42225 2.77519 2.73123 3.46354 2.40513 3.72494 3.29354 2.67741 2.69355 4.24690 2.90347 2.73739 3.18146 2.89801 2.37887 2.77519 2.98518 4.58477 3.61503

0.03394 3.79601 4.51836 0.61958 0.77255 0.48576 0.95510

209 2.92251 4.76322 3.40570 2.93547 4.09844 3.42264 3.77482 3.63647 2.21535 3.16651 4.17059 3.30011 3.93066 3.00062 1.10219 3.01149 3.19482 3.36420 5.21367 4.04432 209 - -

2.68618 4.42225 2.77519 2.73123 3.46354 2.40513 3.72494 3.29354 2.67741 2.69355 4.24690 2.90347 2.73739 3.18146 2.89801 2.37887 2.77519 2.98518 4.58477 3.61503

0.03394 3.79601 4.51836 0.61958 0.77255 0.48576 0.95510

210 2.90487 4.86160 3.07558 2.71397 4.23048 3.40048 3.72190 3.65540 1.14833 3.23828 4.20575 3.14113 3.90783 2.91915 2.42439 2.95301 3.16487 3.36817 5.31817 4.12060 210 - -

2.68618 4.42225 2.77519 2.73123 3.46354 2.40513 3.72494 3.29354 2.67741 2.69355 4.24690 2.90347 2.73739 3.18146 2.89801 2.37887 2.77519 2.98518 4.58477 3.61503

0.03394 3.79601 4.51836 0.61958 0.77255 0.48576 0.95510

211 2.50546 1.10990 4.00398 3.72767 3.73846 3.14282 4.39180 2.87127 3.55467 2.78939 3.90900 3.73510 3.84371 3.90416 3.69196 2.77663 2.96608 2.62062 5.16879 4.02616 211 - -

2.68618 4.42225 2.77519 2.73123 3.46354 2.40513 3.72494 3.29354 2.67741 2.69355 4.24690 2.90347 2.73739 3.18146 2.89801 2.37887 2.77519 2.98518 4.58477 3.61503

0.03394 3.79601 4.51836 0.61958 0.77255 0.48576 0.95510

212 2.37514 4.21936 3.36356 3.11439 3.90452 3.06054 4.13711 3.02248 3.05582 2.88857 3.94573 3.33472 3.75900 3.45502 3.31699 2.57577 1.30864 2.72567 5.34922 4.12174 212 - -

2.68618 4.42225 2.77519 2.73123 3.46354 2.40513 3.72494 3.29354 2.67741 2.69355 4.24690 2.90347 2.73739 3.18146 2.89801 2.37887 2.77519 2.98518 4.58477 3.61503

0.03394 3.79601 4.51836 0.61958 0.77255 0.48576 0.95510

213 2.82402 4.80362 2.85663 2.61241 3.96136 3.31761 3.76927 3.58235 2.43912 3.09213 4.12436 3.07063 3.87138 1.37681 2.72390 2.87605 3.12210 3.31748 5.24814 3.93742 213 - -

2.68618 4.42225 2.77519 2.73123 3.46354 2.40513 3.72494 3.29354 2.67741 2.69355 4.24690 2.90347 2.73739 3.18146 2.89801 2.37887 2.77519 2.98518 4.58477 3.61503

0.03394 3.79601 4.51836 0.61958 0.77255 0.48576 0.95510

214 2.92251 4.76322 3.40570 2.93547 4.09844 3.42264 3.77482 3.63647 2.21535 3.16651 4.17059 3.30011 3.93066 3.00062 1.10219 3.01149 3.19482 3.36420 5.21367 4.04432 214 - -

2.68618 4.42225 2.77519 2.73123 3.46354 2.40513 3.72494 3.29354 2.67741 2.69355 4.24690 2.90347 2.73739 3.18146 2.89801 2.37887 2.77519 2.98518 4.58477 3.61503

0.03394 3.79601 4.51836 0.61958 0.77255 0.48576 0.95510

215 2.50546 1.10990 4.00398 3.72767 3.73846 3.14282 4.39180 2.87127 3.55467 2.78939 3.90900 3.73510 3.84371 3.90416 3.69196 2.77663 2.96608 2.62062 5.16879 4.02616 215 - -

2.68618 4.42225 2.77519 2.73123 3.46354 2.40513 3.72494 3.29354 2.67741 2.69355 4.24690 2.90347 2.73739 3.18146 2.89801 2.37887 2.77519 2.98518 4.58477 3.61503

0.03394 3.79601 4.51836 0.61958 0.77255 0.48576 0.95510

216 3.14031 4.66027 3.72681 3.44361 2.30221 3.74216 3.58865 3.16051 3.30332 2.63376 3.84409 3.63185 4.21871 3.62777 3.50199 3.28401 3.42715 3.02772 3.94242 1.10319 216 - -
2.68618 4.42225 2.77519 2.73123 3.46354 2.40513 3.72494 3.29354 2.67741 2.69355 4.24690 2.90347 2.73739 3.18146 2.89801 2.37887 2.77519 2.98518 4.58477 3.61503
0.03394 3.79601 4.51836 0.61958 0.77255 0.48576 0.95510
217 2.74814 4.31398 4.01764 3.68039 3.49634 3.64254 4.49596 2.06131 3.54689 2.19820 3.47523 3.88188 4.20873 3.91312 3.76637 3.14284 3.11247 1.12759 5.26714 3.99307 217 - -
2.68618 4.42225 2.77519 2.73123 3.46354 2.40513 3.72494 3.29354 2.67741 2.69355 4.24690 2.90347 2.73739 3.18146 2.89801 2.37887 2.77519 2.98518 4.58477 3.61503
0.03394 3.79601 4.51836 0.61958 0.77255 0.48576 0.95510
218 2.66315 4.65392 2.67213 2.55089 4.00571 3.13505 3.86476 3.70148 2.76309 3.35772 4.31495 1.30116 3.78410 3.13903 3.11371 2.73434 3.04351 3.33145 5.32076 3.96081 218 - -
2.68618 4.42225 2.77519 2.73123 3.46354 2.40513 3.72494 3.29354 2.67741 2.69355 4.24690 2.90347 2.73739 3.18146 2.89801 2.37887 2.77519 2.98518 4.58477 3.61503
0.03394 3.79601 4.51836 0.61958 0.77255 0.48576 0.95510
219 2.92251 4.76322 3.40570 2.93547 4.09844 3.42264 3.77482 3.63647 2.21535 3.16651 4.17059 3.30011 3.93066 3.00062 1.10219 3.01149 3.19482 3.36420 5.21367 4.04432 219 - -
2.68618 4.42225 2.77519 2.73123 3.46354 2.40513 3.72494 3.29354 2.67741 2.69355 4.24690 2.90347 2.73739 3.18146 2.89801 2.37887 2.77519 2.98518 4.58477 3.61503
0.03394 3.79601 4.51836 0.61958 0.77255 0.48576 0.95510
220 2.74814 4.31398 4.01764 3.68039 3.49634 3.64254 4.49596 2.06131 3.54689 2.19820 3.47523 3.88188 4.20873 3.91312 3.76637 3.14284 3.11247 1.12759 5.26714 3.99307 220 - -
2.68618 4.42225 2.77519 2.73123 3.46354 2.40513 3.72494 3.29354 2.67741 2.69355 4.24690 2.90347 2.73739 3.18146 2.89801 2.37887 2.77519 2.98518 4.58477 3.61503
0.03394 3.79601 4.51836 0.61958 0.77255 0.48576 0.95510
221 3.07120 4.55728 4.06560 3.70718 3.10996 3.88305 4.38695 2.31238 3.47558 0.97563 3.16058 3.98980 4.33298 3.86233 3.66334 3.44792 3.36975 2.32945 4.95943 3.66445 221 - -
2.68618 4.42225 2.77519 2.73123 3.46354 2.40513 3.72494 3.29354 2.67741 2.69355 4.24690 2.90347 2.73739 3.18146 2.89801 2.37887 2.77519 2.98518 4.58477 3.61503
0.03394 3.79601 4.51836 0.61958 0.77255 0.48576 0.95510
222 2.65642 4.42430 3.25570 3.08531 4.08348 3.11624 4.16757 3.61095 3.13637 3.24548 4.29346 3.39875 0.98293 3.52758 3.38897 2.82198 3.09244 3.26489 5.28934 4.20377 222 - -
2.68618 4.42225 2.77519 2.73123 3.46354 2.40513 3.72494 3.29354 2.67741 2.69355 4.24690 2.90347 2.73739 3.18146 2.89801 2.37887 2.77519 2.98518 4.58477 3.61503
0.03394 3.79601 4.51836 0.61958 0.77255 0.48576 0.95510
223 1.13001 4.15738 3.38757 3.17757 3.97857 2.98348 4.20830 3.09294 3.19189 2.97777 4.01983 3.34570 3.71568 3.54062 3.44647 2.50924 2.78140 2.76077 5.41351 4.20565 223 - -
2.68618 4.42225 2.77519 2.73123 3.46354 2.40513 3.72494 3.29354 2.67741 2.69355 4.24690 2.90347 2.73739 3.18146 2.89801 2.37887 2.77519 2.98518 4.58477 3.61503
0.03394 3.79601 4.51836 0.61958 0.77255 0.48576 0.95510
224 3.07120 4.55728 4.06560 3.70718 3.10996 3.88305 4.38695 2.31238 3.47558 0.97563 3.16058 3.98980 4.33298 3.86233 3.66334 3.44792 3.36975 2.32945 4.95943 3.66445 224 - -
2.68618 4.42225 2.77519 2.73123 3.46354 2.40513 3.72494 3.29354 2.67741 2.69355 4.24690 2.90347 2.73739 3.18146 2.89801 2.37887 2.77519 2.98518 4.58477 3.61503
0.03394 3.79601 4.51836 0.61958 0.77255 0.48576 0.95510
225 3.07120 4.55728 4.06560 3.70718 3.10996 3.88305 4.38695 2.31238 3.47558 0.97563 3.16058 3.98980 4.33298 3.86233 3.66334 3.44792 3.36975 2.32945 4.95943 3.66445 225 - -
2.68618 4.42225 2.77519 2.73123 3.46354 2.40513 3.72494 3.29354 2.67741 2.69355 4.24690 2.90347 2.73739 3.18146 2.89801 2.37887 2.77519 2.98518 4.58477 3.61503
0.03394 3.79601 4.51836 0.61958 0.77255 0.48576 0.95510
226 2.90487 4.86160 3.07558 2.71397 4.23048 3.40048 3.72190 3.65540 1.14833 3.23828 4.20575 3.14113 3.90783 2.91915 2.42439 2.95301 3.16487 3.36817 5.31817 4.12060 226 - -
2.68618 4.42225 2.77519 2.73123 3.46354 2.40513 3.72494 3.29354 2.67741 2.69355 4.24690 2.90347 2.73739 3.18146 2.89801 2.37887 2.77519 2.98518 4.58477 3.61503
0.03394 3.79601 4.51836 0.61958 0.77255 0.48576 0.95510
227 2.90487 4.86160 3.07558 2.71397 4.23048 3.40048 3.72190 3.65540 1.14833 3.23828 4.20575 3.14113 3.90783 2.91915 2.42439 2.95301 3.16487 3.36817 5.31817 4.12060 227 - -
2.68618 4.42225 2.77519 2.73123 3.46354 2.40513 3.72494 3.29354 2.67741 2.69355 4.24690 2.90347 2.73739 3.18146 2.89801 2.37887 2.77519 2.98518 4.58477 3.61503

0.03394 3.79601 4.51836 0.61958 0.77255 0.48576 0.95510

228 2.91724 4.74509 3.05672 2.81086 3.23540 3.38653 1.31468 3.60804 2.65623 3.11092 4.14942 3.22253 3.93587 3.17665 2.92559 2.98918 3.21880 3.34482 4.70119 3.18882 228 - -

2.68618 4.42225 2.77519 2.73123 3.46354 2.40513 3.72494 3.29354 2.67741 2.69355 4.24690 2.90347 2.73739 3.18146 2.89801 2.37887 2.77519 2.98518 4.58477 3.61503

0.03394 3.79601 4.51836 0.61958 0.77255 0.48576 0.95510

229 2.74814 4.31398 4.01764 3.68039 3.49634 3.64254 4.49596 2.06131 3.54689 2.19820 3.47523 3.88188 4.20873 3.91312 3.76637 3.14284 3.11247 1.12759 5.26714 3.99307 229 - -

2.68618 4.42225 2.77519 2.73123 3.46354 2.40513 3.72494 3.29354 2.67741 2.69355 4.24690 2.90347 2.73739 3.18146 2.89801 2.37887 2.77519 2.98518 4.58477 3.61503

0.03394 3.79601 4.51836 0.61958 0.77255 0.48576 0.95510

230 2.74814 4.31398 4.01764 3.68039 3.49634 3.64254 4.49596 2.06131 3.54689 2.19820 3.47523 3.88188 4.20873 3.91312 3.76637 3.14284 3.11247 1.12759 5.26714 3.99307 230 - -

2.68618 4.42225 2.77519 2.73123 3.46354 2.40513 3.72494 3.29354 2.67741 2.69355 4.24690 2.90347 2.73739 3.18146 2.89801 2.37887 2.77519 2.98518 4.58477 3.61503

0.03394 3.79601 4.51836 0.61958 0.77255 0.48576 0.95510

231 2.92251 4.76322 3.40570 2.93547 4.09844 3.42264 3.77482 3.63647 2.21535 3.16651 4.17059 3.30011 3.93066 3.00062 1.10219 3.01149 3.19482 3.36420 5.21367 4.04432 231 - -

2.68618 4.42225 2.77519 2.73123 3.46354 2.40513 3.72494 3.29354 2.67741 2.69355 4.24690 2.90347 2.73739 3.18146 2.89801 2.37887 2.77519 2.98518 4.58477 3.61503

0.03394 3.79601 4.51836 0.61958 0.77255 0.48576 0.95510

232 3.07120 4.55728 4.06560 3.70718 3.10996 3.88305 4.38695 2.31238 3.47558 0.97563 3.16058 3.98980 4.33298 3.86233 3.66334 3.44792 3.36975 2.32945 4.95943 3.66445 232 - -

2.68618 4.42225 2.77519 2.73123 3.46354 2.40513 3.72494 3.29354 2.67741 2.69355 4.24690 2.90347 2.73739 3.18146 2.89801 2.37887 2.77519 2.98518 4.58477 3.61503

0.03394 3.79601 4.51836 0.61958 0.77255 0.48576 0.95510

233 2.37514 4.21936 3.36356 3.11439 3.90452 3.06054 4.13711 3.02248 3.05582 2.88857 3.94573 3.33472 3.75900 3.45502 3.31699 2.57577 1.30864 2.72567 5.34922 4.12174 233 - -

2.68618 4.42225 2.77519 2.73123 3.46354 2.40513 3.72494 3.29354 2.67741 2.69355 4.24690 2.90347 2.73739 3.18146 2.89801 2.37887 2.77519 2.98518 4.58477 3.61503

0.03394 3.79601 4.51836 0.61958 0.77255 0.48576 0.95510

234 2.90487 4.86160 3.07558 2.71397 4.23048 3.40048 3.72190 3.65540 1.14833 3.23828 4.20575 3.14113 3.90783 2.91915 2.42439 2.95301 3.16487 3.36817 5.31817 4.12060 234 - -

2.68618 4.42225 2.77519 2.73123 3.46354 2.40513 3.72494 3.29354 2.67741 2.69355 4.24690 2.90347 2.73739 3.18146 2.89801 2.37887 2.77519 2.98518 4.58477 3.61503

0.03394 3.79601 4.51836 0.61958 0.77255 0.48576 0.95510

235 3.16380 4.58887 4.13307 3.83471 1.14586 3.86813 3.84824 2.69884 3.74404 2.08761 3.44167 3.93408 4.32984 3.93371 3.88264 3.43660 3.46563 2.68355 4.12324 2.50118 235 - -

2.68618 4.42225 2.77519 2.73123 3.46354 2.40513 3.72494 3.29354 2.67741 2.69355 4.24690 2.90347 2.73739 3.18146 2.89801 2.37887 2.77519 2.98518 4.58477 3.61503

0.03394 3.79601 4.51836 0.61958 0.77255 0.48576 0.95510

236 2.91056 5.02139 1.03432 2.29423 4.38899 3.15549 3.86877 3.96045 2.93927 3.58342 4.56179 2.84589 3.80315 3.10925 3.44342 2.88302 3.24824 3.60850 5.57234 4.27708 236 - -

2.68618 4.42225 2.77519 2.73123 3.46354 2.40513 3.72494 3.29354 2.67741 2.69355 4.24690 2.90347 2.73739 3.18146 2.89801 2.37887 2.77519 2.98518 4.58477 3.61503

0.03394 3.79601 4.51836 0.61958 0.77255 0.48576 0.95510

237 3.14031 4.66027 3.72681 3.44361 2.30221 3.74216 3.58865 3.16051 3.30332 2.63376 3.84409 3.63185 4.21871 3.62777 3.50199 3.28401 3.42715 3.02772 3.94242 1.10319 237 - -

2.68618 4.42225 2.77519 2.73123 3.46354 2.40513 3.72494 3.29354 2.67741 2.69355 4.24690 2.90347 2.73739 3.18146 2.89801 2.37887 2.77519 2.98518 4.58477 3.61503

0.03394 3.79601 4.51836 0.61958 0.77255 0.48576 0.95510

238 2.92251 4.76322 3.40570 2.93547 4.09844 3.42264 3.77482 3.63647 2.21535 3.16651 4.17059 3.30011 3.93066 3.00062 1.10219 3.01149 3.19482 3.36420 5.21367 4.04432 238 - -

2.68618 4.42225 2.77519 2.73123 3.46354 2.40513 3.72494 3.29354 2.67741 2.69355 4.24690 2.90347 2.73739 3.18146 2.89801 2.37887 2.77519 2.98518 4.58477 3.61503

0.03394 3.79601 4.51836 0.61958 0.77255 0.48576 0.95510

239 3.07120 4.55728 4.06560 3.70718 3.10996 3.88305 4.38695 2.31238 3.47558 0.97563 3.16058 3.98980 4.33298 3.86233 3.66334 3.44792 3.36975 2.32945 4.95943 3.66445 239 - -

2.68618 4.42225 2.77519 2.73123 3.46354 2.40513 3.72494 3.29354 2.67741 2.69355 4.24690 2.90347 2.73739 3.18146 2.89801 2.37887 2.77519 2.98518 4.58477 3.61503
0.03394 3.79601 4.51836 0.61958 0.77255 0.48576 0.95510
240 1.13001 4.15738 3.38757 3.17757 3.97857 2.98348 4.20830 3.09294 3.19189 2.97777 4.01983 3.34570 3.71568 3.54062 3.44647 2.50924 2.78140 2.76077 5.41351 4.20565 240 - -
2.68618 4.42225 2.77519 2.73123 3.46354 2.40513 3.72494 3.29354 2.67741 2.69355 4.24690 2.90347 2.73739 3.18146 2.89801 2.37887 2.77519 2.98518 4.58477 3.61503
0.03394 3.79601 4.51836 0.61958 0.77255 0.48576 0.95510
241 2.66315 4.65392 2.67213 2.55089 4.00571 3.13505 3.86476 3.70148 2.76309 3.35772 4.31495 1.30116 3.78410 3.13903 3.11371 2.73434 3.04351 3.33145 5.32076 3.96081 241 - -
2.68618 4.42225 2.77519 2.73123 3.46354 2.40513 3.72494 3.29354 2.67741 2.69355 4.24690 2.90347 2.73739 3.18146 2.89801 2.37887 2.77519 2.98518 4.58477 3.61503
0.03394 3.79601 4.51836 0.61958 0.77255 0.48576 0.95510
242 2.92251 4.76322 3.40570 2.93547 4.09844 3.42264 3.77482 3.63647 2.21535 3.16651 4.17059 3.30011 3.93066 3.00062 1.10219 3.01149 3.19482 3.36420 5.21367 4.04432 242 - -
2.68618 4.42225 2.77519 2.73123 3.46354 2.40513 3.72494 3.29354 2.67741 2.69355 4.24690 2.90347 2.73739 3.18146 2.89801 2.37887 2.77519 2.98518 4.58477 3.61503
0.03394 3.79601 4.51836 0.61958 0.77255 0.48576 0.95510
243 3.07120 4.55728 4.06560 3.70718 3.10996 3.88305 4.38695 2.31238 3.47558 0.97563 3.16058 3.98980 4.33298 3.86233 3.66334 3.44792 3.36975 2.32945 4.95943 3.66445 243 - -
2.68618 4.42225 2.77519 2.73123 3.46354 2.40513 3.72494 3.29354 2.67741 2.69355 4.24690 2.90347 2.73739 3.18146 2.89801 2.37887 2.77519 2.98518 4.58477 3.61503
0.03394 3.79601 4.51836 0.61958 0.77255 0.48576 0.95510
244 2.91056 5.02139 1.03432 2.29423 4.38899 3.15549 3.86877 3.96045 2.93927 3.58342 4.56179 2.84589 3.80315 3.10925 3.44342 2.88302 3.24824 3.60850 5.57234 4.27708 244 - -
2.68618 4.42225 2.77519 2.73123 3.46354 2.40513 3.72494 3.29354 2.67741 2.69355 4.24690 2.90347 2.73739 3.18146 2.89801 2.37887 2.77519 2.98518 4.58477 3.61503
0.03394 3.79601 4.51836 0.61958 0.77255 0.48576 0.95510
245 2.37514 4.21936 3.36356 3.11439 3.90452 3.06054 4.13711 3.02248 3.05582 2.88857 3.94573 3.33472 3.75900 3.45502 3.31699 2.57577 1.30864 2.72567 5.34922 4.12174 245 - -
2.68618 4.42225 2.77519 2.73123 3.46354 2.40513 3.72494 3.29354 2.67741 2.69355 4.24690 2.90347 2.73739 3.18146 2.89801 2.37887 2.77519 2.98518 4.58477 3.61503
0.03394 3.79601 4.51836 0.61958 0.77255 0.48576 0.95510
246 2.91056 5.02139 1.03432 2.29423 4.38899 3.15549 3.86877 3.96045 2.93927 3.58342 4.56179 2.84589 3.80315 3.10925 3.44342 2.88302 3.24824 3.60850 5.57234 4.27708 246 - -
2.68618 4.42225 2.77519 2.73123 3.46354 2.40513 3.72494 3.29354 2.67741 2.69355 4.24690 2.90347 2.73739 3.18146 2.89801 2.37887 2.77519 2.98518 4.58477 3.61503
0.03394 3.79601 4.51836 0.61958 0.77255 0.48576 0.95510
247 3.07120 4.55728 4.06560 3.70718 3.10996 3.88305 4.38695 2.31238 3.47558 0.97563 3.16058 3.98980 4.33298 3.86233 3.66334 3.44792 3.36975 2.32945 4.95943 3.66445 247 - -
2.68618 4.42225 2.77519 2.73123 3.46354 2.40513 3.72494 3.29354 2.67741 2.69355 4.24690 2.90347 2.73739 3.18146 2.89801 2.37887 2.77519 2.98518 4.58477 3.61503
0.03394 3.79601 4.51836 0.61958 0.77255 0.48576 0.95510
248 2.82402 4.80362 2.85663 2.61241 3.96136 3.31761 3.76927 3.58235 2.43912 3.09213 4.12436 3.07063 3.87138 1.37681 2.72390 2.87605 3.12210 3.31748 5.24814 3.93742 248 - -
2.68618 4.42225 2.77519 2.73123 3.46354 2.40513 3.72494 3.29354 2.67741 2.69355 4.24690 2.90347 2.73739 3.18146 2.89801 2.37887 2.77519 2.98518 4.58477 3.61503
0.03394 3.79601 4.51836 0.61958 0.77255 0.48576 0.95510
249 2.90487 4.86160 3.07558 2.71397 4.23048 3.40048 3.72190 3.65540 1.14833 3.23828 4.20575 3.14113 3.90783 2.91915 2.42439 2.95301 3.16487 3.36817 5.31817 4.12060 249 - -
2.68618 4.42225 2.77519 2.73123 3.46354 2.40513 3.72494 3.29354 2.67741 2.69355 4.24690 2.90347 2.73739 3.18146 2.89801 2.37887 2.77519 2.98518 4.58477 3.61503
0.03394 3.79601 4.51836 0.61958 0.77255 0.48576 0.95510
250 3.07120 4.55728 4.06560 3.70718 3.10996 3.88305 4.38695 2.31238 3.47558 0.97563 3.16058 3.98980 4.33298 3.86233 3.66334 3.44792 3.36975 2.32945 4.95943 3.66445 250 - -
2.68618 4.42225 2.77519 2.73123 3.46354 2.40513 3.72494 3.29354 2.67741 2.69355 4.24690 2.90347 2.73739 3.18146 2.89801 2.37887 2.77519 2.98518 4.58477 3.61503
0.03394 3.79601 4.51836 0.61958 0.77255 0.48576 0.95510

251 2.92251 4.76322 3.40570 2.93547 4.09844 3.42264 3.77482 3.63647 2.21535 3.16651 4.17059 3.30011 3.93066 3.00062 1.10219 3.01149 3.19482 3.36420 5.21367 4.04432 251 - -
2.68618 4.42225 2.77519 2.73123 3.46354 2.40513 3.72494 3.29354 2.67741 2.69355 4.24690 2.90347 2.73739 3.18146 2.89801 2.37887 2.77519 2.98518 4.58477 3.61503
0.03394 3.79601 4.51836 0.61958 0.77255 0.48576 0.95510
252 2.50546 1.10990 4.00398 3.72767 3.73846 3.14282 4.39180 2.87127 3.55467 2.78939 3.90900 3.73510 3.84371 3.90416 3.69196 2.77663 2.96608 2.62062 5.16879 4.02616 252 - -
2.68618 4.42225 2.77519 2.73123 3.46354 2.40513 3.72494 3.29354 2.67741 2.69355 4.24690 2.90347 2.73739 3.18146 2.89801 2.37887 2.77519 2.98518 4.58477 3.61503
0.03394 3.79601 4.51836 0.61958 0.77255 0.48576 0.95510
253 2.92251 4.76322 3.40570 2.93547 4.09844 3.42264 3.77482 3.63647 2.21535 3.16651 4.17059 3.30011 3.93066 3.00062 1.10219 3.01149 3.19482 3.36420 5.21367 4.04432 253 - -
2.68618 4.42225 2.77519 2.73123 3.46354 2.40513 3.72494 3.29354 2.67741 2.69355 4.24690 2.90347 2.73739 3.18146 2.89801 2.37887 2.77519 2.98518 4.58477 3.61503
0.03394 3.79601 4.51836 0.61958 0.77255 0.48576 0.95510
254 2.74814 4.31398 4.01764 3.68039 3.49634 3.64254 4.49596 2.06131 3.54689 2.19820 3.47523 3.88188 4.20873 3.91312 3.76637 3.14284 3.11247 1.12759 5.26714 3.99307 254 - -
2.68618 4.42225 2.77519 2.73123 3.46354 2.40513 3.72494 3.29354 2.67741 2.69355 4.24690 2.90347 2.73739 3.18146 2.89801 2.37887 2.77519 2.98518 4.58477 3.61503
0.03394 3.79601 4.51836 0.61958 0.77255 0.48576 0.95510
255 2.66315 4.65392 2.67213 2.55089 4.00571 3.13505 3.86476 3.70148 2.76309 3.35772 4.31495 1.30116 3.78410 3.13903 3.11371 2.73434 3.04351 3.33145 5.32076 3.96081 255 - -
2.68618 4.42225 2.77519 2.73123 3.46354 2.40513 3.72494 3.29354 2.67741 2.69355 4.24690 2.90347 2.73739 3.18146 2.89801 2.37887 2.77519 2.98518 4.58477 3.61503
0.03394 3.79601 4.51836 0.61958 0.77255 0.48576 0.95510
256 3.14031 4.66027 3.72681 3.44361 2.30221 3.74216 3.58865 3.16051 3.30332 2.63376 3.84409 3.63185 4.21871 3.62777 3.50199 3.28401 3.42715 3.02772 3.94242 1.10319 256 - -
2.68618 4.42225 2.77519 2.73123 3.46354 2.40513 3.72494 3.29354 2.67741 2.69355 4.24690 2.90347 2.73739 3.18146 2.89801 2.37887 2.77519 2.98518 4.58477 3.61503
0.03394 3.79601 4.51836 0.61958 0.77255 0.48576 0.95510
257 2.91724 4.74509 3.05672 2.81086 3.23540 3.38653 1.31468 3.60804 2.65623 3.11092 4.14942 3.22253 3.93587 3.17665 2.92559 2.98918 3.21880 3.34482 4.70119 3.18882 257 - -
2.68618 4.42225 2.77519 2.73123 3.46354 2.40513 3.72494 3.29354 2.67741 2.69355 4.24690 2.90347 2.73739 3.18146 2.89801 2.37887 2.77519 2.98518 4.58477 3.61503
0.03394 3.79601 4.51836 0.61958 0.77255 0.48576 0.95510
258 2.57535 4.35609 3.23321 3.12407 4.26351 0.85754 4.24721 3.84618 3.29467 3.50959 4.48627 3.39802 3.74476 3.62525 3.53733 2.74543 3.05073 3.38948 5.37705 4.36350 258 - -
2.68618 4.42225 2.77519 2.73123 3.46354 2.40513 3.72494 3.29354 2.67741 2.69355 4.24690 2.90347 2.73739 3.18146 2.89801 2.37887 2.77519 2.98518 4.58477 3.61503
0.03394 3.79601 4.51836 0.61958 0.77255 0.48576 0.95510
259 3.07120 4.55728 4.06560 3.70718 3.10996 3.88305 4.38695 2.31238 3.47558 0.97563 3.16058 3.98980 4.33298 3.86233 3.66334 3.44792 3.36975 2.32945 4.95943 3.66445 259 - -
2.68618 4.42225 2.77519 2.73123 3.46354 2.40513 3.72494 3.29354 2.67741 2.69355 4.24690 2.90347 2.73739 3.18146 2.89801 2.37887 2.77519 2.98518 4.58477 3.61503
0.03394 3.79601 4.51836 0.61958 0.77255 0.48576 0.95510
260 2.92251 4.76322 3.40570 2.93547 4.09844 3.42264 3.77482 3.63647 2.21535 3.16651 4.17059 3.30011 3.93066 3.00062 1.10219 3.01149 3.19482 3.36420 5.21367 4.04432 260 - -
2.68618 4.42225 2.77519 2.73123 3.46354 2.40513 3.72494 3.29354 2.67741 2.69355 4.24690 2.90347 2.73739 3.18146 2.89801 2.37887 2.77519 2.98518 4.58477 3.61503
0.03394 3.79601 4.51836 0.61958 0.77255 0.48576 0.95510
261 3.16380 4.58887 4.13307 3.83471 1.14586 3.86813 3.84824 2.69884 3.74404 2.08761 3.44167 3.93408 4.32984 3.93371 3.88264 3.43660 3.46563 2.68355 4.12324 2.50118 261 - -
2.68618 4.42225 2.77519 2.73123 3.46354 2.40513 3.72494 3.29354 2.67741 2.69355 4.24690 2.90347 2.73739 3.18146 2.89801 2.37887 2.77519 2.98518 4.58477 3.61503
0.03394 3.79601 4.51836 0.61958 0.77255 0.48576 0.95510
262 2.37514 4.21936 3.36356 3.11439 3.90452 3.06054 4.13711 3.02248 3.05582 2.88857 3.94573 3.33472 3.75900 3.45502 3.31699 2.57577 1.30864 2.72567 5.34922 4.12174 262 - -
2.68618 4.42225 2.77519 2.73123 3.46354 2.40513 3.72494 3.29354 2.67741 2.69355 4.24690 2.90347 2.73739 3.18146 2.89801 2.37887 2.77519 2.98518 4.58477 3.61503

0.03394 3.79601 4.51836 0.61958 0.77255 0.48576 0.95510

263 2.57535 4.35609 3.23321 3.12407 4.26351 0.85754 4.24721 3.84618 3.29467 3.50959 4.48627 3.39802 3.74476 3.62525 3.53733 2.74543 3.05073 3.38948 5.37705 4.36350 263 - -

2.68618 4.42225 2.77519 2.73123 3.46354 2.40513 3.72494 3.29354 2.67741 2.69355 4.24690 2.90347 2.73739 3.18146 2.89801 2.37887 2.77519 2.98518 4.58477 3.61503

0.03394 3.79601 4.51836 0.61958 0.77255 0.48576 0.95510

264 3.07120 4.55728 4.06560 3.70718 3.10996 3.88305 4.38695 2.31238 3.47558 0.97563 3.16058 3.98980 4.33298 3.86233 3.66334 3.44792 3.36975 2.32945 4.95943 3.66445 264 - -

2.68618 4.42225 2.77519 2.73123 3.46354 2.40513 3.72494 3.29354 2.67741 2.69355 4.24690 2.90347 2.73739 3.18146 2.89801 2.37887 2.77519 2.98518 4.58477 3.61503

0.03394 3.79601 4.51836 0.61958 0.77255 0.48576 0.95510

265 2.97165 4.41429 4.22289 3.83744 3.32476 3.98045 4.58108 1.17818 3.66390 1.92890 3.27450 4.09357 4.42084 4.02132 3.86934 3.47002 3.27299 1.84997 5.19827 3.92855 265 - -

2.68618 4.42225 2.77519 2.73123 3.46354 2.40513 3.72494 3.29354 2.67741 2.69355 4.24690 2.90347 2.73739 3.18146 2.89801 2.37887 2.77519 2.98518 4.58477 3.61503

0.03394 3.79601 4.51836 0.61958 0.77255 0.48576 0.95510

266 2.89015 4.98001 2.46169 1.13957 4.31725 3.22729 3.81015 3.75600 2.68126 3.38645 4.36600 2.89555 3.82889 3.02881 3.08050 2.88174 3.19454 3.44858 5.50348 4.22803 266 - -

2.68618 4.42225 2.77519 2.73123 3.46354 2.40513 3.72494 3.29354 2.67741 2.69355 4.24690 2.90347 2.73739 3.18146 2.89801 2.37887 2.77519 2.98518 4.58477 3.61503

0.03394 3.79601 4.51836 0.61958 0.77255 0.48576 0.95510

267 2.89015 4.98001 2.46169 1.13957 4.31725 3.22729 3.81015 3.75600 2.68126 3.38645 4.36600 2.89555 3.82889 3.02881 3.08050 2.88174 3.19454 3.44858 5.50348 4.22803 267 - -

2.68618 4.42225 2.77519 2.73123 3.46354 2.40513 3.72494 3.29354 2.67741 2.69355 4.24690 2.90347 2.73739 3.18146 2.89801 2.37887 2.77519 2.98518 4.58477 3.61503

0.03394 3.79601 4.51836 0.61958 0.77255 0.48576 0.95510

268 2.91157 4.47328 3.96816 3.55750 3.17841 3.77696 4.31044 2.34654 3.32910 1.78626 1.53296 3.84393 4.23864 3.71698 3.54307 3.25192 3.21616 2.34333 5.00802 3.76205 268 - -

2.68618 4.42225 2.77519 2.73123 3.46354 2.40513 3.72494 3.29354 2.67741 2.69355 4.24690 2.90347 2.73739 3.18146 2.89801 2.37887 2.77519 2.98518 4.58477 3.61503

0.03394 3.79601 4.51836 0.61958 0.77255 0.48576 0.95510

269 2.57535 4.35609 3.23321 3.12407 4.26351 0.85754 4.24721 3.84618 3.29467 3.50959 4.48627 3.39802 3.74476 3.62525 3.53733 2.74543 3.05073 3.38948 5.37705 4.36350 269 - -

2.68618 4.42225 2.77519 2.73123 3.46354 2.40513 3.72494 3.29354 2.67741 2.69355 4.24690 2.90347 2.73739 3.18146 2.89801 2.37887 2.77519 2.98518 4.58477 3.61503

0.03394 3.79601 4.51836 0.61958 0.77255 0.48576 0.95510

270 2.89015 4.98001 2.46169 1.13957 4.31725 3.22729 3.81015 3.75600 2.68126 3.38645 4.36600 2.89555 3.82889 3.02881 3.08050 2.88174 3.19454 3.44858 5.50348 4.22803 270 - -

2.68618 4.42225 2.77519 2.73123 3.46354 2.40513 3.72494 3.29354 2.67741 2.69355 4.24690 2.90347 2.73739 3.18146 2.89801 2.37887 2.77519 2.98518 4.58477 3.61503

0.03394 3.79601 4.51836 0.61958 0.77255 0.48576 0.95510

271 2.90487 4.86160 3.07558 2.71397 4.23048 3.40048 3.72190 3.65540 1.14833 3.23828 4.20575 3.14113 3.90783 2.91915 2.42439 2.95301 3.16487 3.36817 5.31817 4.12060 271 - -

2.68618 4.42225 2.77519 2.73123 3.46354 2.40513 3.72494 3.29354 2.67741 2.69355 4.24690 2.90347 2.73739 3.18146 2.89801 2.37887 2.77519 2.98518 4.58477 3.61503

0.03394 3.79601 4.51836 0.61958 0.77255 0.48576 0.95510

272 3.07120 4.55728 4.06560 3.70718 3.10996 3.88305 4.38695 2.31238 3.47558 0.97563 3.16058 3.98980 4.33298 3.86233 3.66334 3.44792 3.36975 2.32945 4.95943 3.66445 272 - -

2.68618 4.42225 2.77519 2.73123 3.46354 2.40513 3.72494 3.29354 2.67741 2.69355 4.24690 2.90347 2.73739 3.18146 2.89801 2.37887 2.77519 2.98518 4.58477 3.61503

0.03394 3.79601 4.51836 0.61958 0.77255 0.48576 0.95510

273 2.97165 4.41429 4.22289 3.83744 3.32476 3.98045 4.58108 1.17818 3.66390 1.92890 3.27450 4.09357 4.42084 4.02132 3.86934 3.47002 3.27299 1.84997 5.19827 3.92855 273 - -

2.68618 4.42225 2.77519 2.73123 3.46354 2.40513 3.72494 3.29354 2.67741 2.69355 4.24690 2.90347 2.73739 3.18146 2.89801 2.37887 2.77519 2.98518 4.58477 3.61503

0.03394 3.79601 4.51836 0.61958 0.77255 0.48576 0.95510

274 2.82402 4.80362 2.85663 2.61241 3.96136 3.31761 3.76927 3.58235 2.43912 3.09213 4.12436 3.07063 3.87138 1.37681 2.72390 2.87605 3.12210 3.31748 5.24814 3.93742 274 - -

2.68618 4.42225 2.77519 2.73123 3.46354 2.40513 3.72494 3.29354 2.67741 2.69355 4.24690 2.90347 2.73739 3.18146 2.89801 2.37887 2.77519 2.98518 4.58477 3.61503
0.03394 3.79601 4.51836 0.61958 0.77255 0.48576 0.95510
275 2.92251 4.76322 3.40570 2.93547 4.09844 3.42264 3.77482 3.63647 2.21535 3.16651 4.17059 3.30011 3.93066 3.00062 1.10219 3.01149 3.19482 3.36420 5.21367 4.04432 275 - -
2.68618 4.42225 2.77519 2.73123 3.46354 2.40513 3.72494 3.29354 2.67741 2.69355 4.24690 2.90347 2.73739 3.18146 2.89801 2.37887 2.77519 2.98518 4.58477 3.61503
0.03394 3.79601 4.51836 0.61958 0.77255 0.48576 0.95510
276 2.91157 4.47328 3.96816 3.55750 3.17841 3.77696 4.31044 2.34654 3.32910 1.78626 1.53296 3.84393 4.23864 3.71698 3.54307 3.25192 3.21616 2.34333 5.00802 3.76205 276 - -
2.68618 4.42225 2.77519 2.73123 3.46354 2.40513 3.72494 3.29354 2.67741 2.69355 4.24690 2.90347 2.73739 3.18146 2.89801 2.37887 2.77519 2.98518 4.58477 3.61503
0.03394 3.79601 4.51836 0.61958 0.77255 0.48576 0.95510
277 2.92251 4.76322 3.40570 2.93547 4.09844 3.42264 3.77482 3.63647 2.21535 3.16651 4.17059 3.30011 3.93066 3.00062 1.10219 3.01149 3.19482 3.36420 5.21367 4.04432 277 - -
2.68618 4.42225 2.77519 2.73123 3.46354 2.40513 3.72494 3.29354 2.67741 2.69355 4.24690 2.90347 2.73739 3.18146 2.89801 2.37887 2.77519 2.98518 4.58477 3.61503
0.03394 3.79601 4.51836 0.61958 0.77255 0.48576 0.95510
278 2.89015 4.98001 2.46169 1.13957 4.31725 3.22729 3.81015 3.75600 2.68126 3.38645 4.36600 2.89555 3.82889 3.02881 3.08050 2.88174 3.19454 3.44858 5.50348 4.22803 278 - -
2.68618 4.42225 2.77519 2.73123 3.46354 2.40513 3.72494 3.29354 2.67741 2.69355 4.24690 2.90347 2.73739 3.18146 2.89801 2.37887 2.77519 2.98518 4.58477 3.61503
0.03394 3.79601 4.51836 0.61958 0.77255 0.48576 0.95510
279 2.92251 4.76322 3.40570 2.93547 4.09844 3.42264 3.77482 3.63647 2.21535 3.16651 4.17059 3.30011 3.93066 3.00062 1.10219 3.01149 3.19482 3.36420 5.21367 4.04432 279 - -
2.68618 4.42225 2.77519 2.73123 3.46354 2.40513 3.72494 3.29354 2.67741 2.69355 4.24690 2.90347 2.73739 3.18146 2.89801 2.37887 2.77519 2.98518 4.58477 3.61503
0.03394 3.79601 4.51836 0.61958 0.77255 0.48576 0.95510
280 2.28079 4.19908 3.14317 2.95737 4.00099 2.93053 4.06839 3.51415 3.02585 3.24109 4.17505 3.19319 3.66344 3.38168 3.31291 1.21257 2.75555 3.06614 5.38010 4.08457 280 - -
2.68618 4.42225 2.77519 2.73123 3.46354 2.40513 3.72494 3.29354 2.67741 2.69355 4.24690 2.90347 2.73739 3.18146 2.89801 2.37887 2.77519 2.98518 4.58477 3.61503
0.03394 3.79601 4.51836 0.61958 0.77255 0.48576 0.95510
281 2.90487 4.86160 3.07558 2.71397 4.23048 3.40048 3.72190 3.65540 1.14833 3.23828 4.20575 3.14113 3.90783 2.91915 2.42439 2.95301 3.16487 3.36817 5.31817 4.12060 281 - -
2.68618 4.42225 2.77519 2.73123 3.46354 2.40513 3.72494 3.29354 2.67741 2.69355 4.24690 2.90347 2.73739 3.18146 2.89801 2.37887 2.77519 2.98518 4.58477 3.61503
0.03394 3.79601 4.51836 0.61958 0.77255 0.48576 0.95510
282 2.91724 4.74509 3.05672 2.81086 3.23540 3.38653 1.31468 3.60804 2.65623 3.11092 4.14942 3.22253 3.93587 3.17665 2.92559 2.98918 3.21880 3.34482 4.70119 3.18882 282 - -
2.68618 4.42225 2.77519 2.73123 3.46354 2.40513 3.72494 3.29354 2.67741 2.69355 4.24690 2.90347 2.73739 3.18146 2.89801 2.37887 2.77519 2.98518 4.58477 3.61503
0.03394 3.79601 4.51836 0.61958 0.77255 0.48576 0.95510
283 3.16380 4.58887 4.13307 3.83471 1.14586 3.86813 3.84824 2.69884 3.74404 2.08761 3.44167 3.93408 4.32984 3.93371 3.88264 3.43660 3.46563 2.68355 4.12324 2.50118 283 - -
2.68618 4.42225 2.77519 2.73123 3.46354 2.40513 3.72494 3.29354 2.67741 2.69355 4.24690 2.90347 2.73739 3.18146 2.89801 2.37887 2.77519 2.98518 4.58477 3.61503
0.03394 3.79601 4.51836 0.61958 0.77255 0.48576 0.95510
284 2.97165 4.41429 4.22289 3.83744 3.32476 3.98045 4.58108 1.17818 3.66390 1.92890 3.27450 4.09357 4.42084 4.02132 3.86934 3.47002 3.27299 1.84997 5.19827 3.92855 284 - -
2.68618 4.42225 2.77519 2.73123 3.46354 2.40513 3.72494 3.29354 2.67741 2.69355 4.24690 2.90347 2.73739 3.18146 2.89801 2.37887 2.77519 2.98518 4.58477 3.61503
0.03394 3.79601 4.51836 0.61958 0.77255 0.48576 0.95510
285 1.13001 4.15738 3.38757 3.17757 3.97857 2.98348 4.20830 3.09294 3.19189 2.97777 4.01983 3.34570 3.71568 3.54062 3.44647 2.50924 2.78140 2.76077 5.41351 4.20565 285 - -
2.68618 4.42225 2.77519 2.73123 3.46354 2.40513 3.72494 3.29354 2.67741 2.69355 4.24690 2.90347 2.73739 3.18146 2.89801 2.37887 2.77519 2.98518 4.58477 3.61503
0.03394 3.79601 4.51836 0.61958 0.77255 0.48576 0.95510

286 3.07120 4.55728 4.06560 3.70718 3.10996 3.88305 4.38695 2.31238 3.47558 0.97563 3.16058 3.98980 4.33298 3.86233 3.66334 3.44792 3.36975 2.32945 4.95943 3.66445 286 - -
2.68618 4.42225 2.77519 2.73123 3.46354 2.40513 3.72494 3.29354 2.67741 2.69355 4.24690 2.90347 2.73739 3.18146 2.89801 2.37887 2.77519 2.98518 4.58477 3.61503
0.03394 3.79601 4.51836 0.61958 0.77255 0.48576 0.95510
287 2.91724 4.74509 3.05672 2.81086 3.23540 3.38653 1.31468 3.60804 2.65623 3.11092 4.14942 3.22253 3.93587 3.17665 2.92559 2.98918 3.21880 3.34482 4.70119 3.18882 287 - -
2.68618 4.42225 2.77519 2.73123 3.46354 2.40513 3.72494 3.29354 2.67741 2.69355 4.24690 2.90347 2.73739 3.18146 2.89801 2.37887 2.77519 2.98518 4.58477 3.61503
0.03394 3.79601 4.51836 0.61958 0.77255 0.48576 0.95510
288 3.07120 4.55728 4.06560 3.70718 3.10996 3.88305 4.38695 2.31238 3.47558 0.97563 3.16058 3.98980 4.33298 3.86233 3.66334 3.44792 3.36975 2.32945 4.95943 3.66445 288 - -
2.68618 4.42225 2.77519 2.73123 3.46354 2.40513 3.72494 3.29354 2.67741 2.69355 4.24690 2.90347 2.73739 3.18146 2.89801 2.37887 2.77519 2.98518 4.58477 3.61503
0.03394 3.79601 4.51836 0.61958 0.77255 0.48576 0.95510
289 2.92251 4.76322 3.40570 2.93547 4.09844 3.42264 3.77482 3.63647 2.21535 3.16651 4.17059 3.30011 3.93066 3.00062 1.10219 3.01149 3.19482 3.36420 5.21367 4.04432 289 - -
2.68618 4.42225 2.77519 2.73123 3.46354 2.40513 3.72494 3.29354 2.67741 2.69355 4.24690 2.90347 2.73739 3.18146 2.89801 2.37887 2.77519 2.98518 4.58477 3.61503
0.03394 3.79601 4.51836 0.61958 0.77255 0.48576 0.95510
290 3.16380 4.58887 4.13307 3.83471 1.14586 3.86813 3.84824 2.69884 3.74404 2.08761 3.44167 3.93408 4.32984 3.93371 3.88264 3.43660 3.46563 2.68355 4.12324 2.50118 290 - -
2.68618 4.42225 2.77519 2.73123 3.46354 2.40513 3.72494 3.29354 2.67741 2.69355 4.24690 2.90347 2.73739 3.18146 2.89801 2.37887 2.77519 2.98518 4.58477 3.61503
0.03394 3.79601 4.51836 0.61958 0.77255 0.48576 0.95510
291 2.89015 4.98001 2.46169 1.13957 4.31725 3.22729 3.81015 3.75600 2.68126 3.38645 4.36600 2.89555 3.82889 3.02881 3.08050 2.88174 3.19454 3.44858 5.50348 4.22803 291 - -
2.68618 4.42225 2.77519 2.73123 3.46354 2.40513 3.72494 3.29354 2.67741 2.69355 4.24690 2.90347 2.73739 3.18146 2.89801 2.37887 2.77519 2.98518 4.58477 3.61503
0.03394 3.79601 4.51836 0.61958 0.77255 0.48576 0.95510
292 2.65642 4.42430 3.25570 3.08531 4.08348 3.11624 4.16757 3.61095 3.13637 3.24548 4.29346 3.39875 0.98293 3.52758 3.38897 2.82198 3.09244 3.26489 5.28934 4.20377 292 - -
2.68618 4.42225 2.77519 2.73123 3.46354 2.40513 3.72494 3.29354 2.67741 2.69355 4.24690 2.90347 2.73739 3.18146 2.89801 2.37887 2.77519 2.98518 4.58477 3.61503
0.03394 3.79601 4.51836 0.61958 0.77255 0.48576 0.95510
293 2.91056 5.02139 1.03432 2.29423 4.38899 3.15549 3.86877 3.96045 2.93927 3.58342 4.56179 2.84589 3.80315 3.10925 3.44342 2.88302 3.24824 3.60850 5.57234 4.27708 293 - -
2.68618 4.42225 2.77519 2.73123 3.46354 2.40513 3.72494 3.29354 2.67741 2.69355 4.24690 2.90347 2.73739 3.18146 2.89801 2.37887 2.77519 2.98518 4.58477 3.61503
0.03394 3.79601 4.51836 0.61958 0.77255 0.48576 0.95510
294 2.91157 4.47328 3.96816 3.55750 3.17841 3.77696 4.31044 2.34654 3.32910 1.78626 1.53296 3.84393 4.23864 3.71698 3.54307 3.25192 3.21616 2.34333 5.00802 3.76205 294 - -
2.68618 4.42225 2.77519 2.73123 3.46354 2.40513 3.72494 3.29354 2.67741 2.69355 4.24690 2.90347 2.73739 3.18146 2.89801 2.37887 2.77519 2.98518 4.58477 3.61503
0.03394 3.79601 4.51836 0.61958 0.77255 0.48576 0.95510
295 3.07120 4.55728 4.06560 3.70718 3.10996 3.88305 4.38695 2.31238 3.47558 0.97563 3.16058 3.98980 4.33298 3.86233 3.66334 3.44792 3.36975 2.32945 4.95943 3.66445 295 - -
2.68618 4.42225 2.77519 2.73123 3.46354 2.40513 3.72494 3.29354 2.67741 2.69355 4.24690 2.90347 2.73739 3.18146 2.89801 2.37887 2.77519 2.98518 4.58477 3.61503
0.03394 3.79601 4.51836 0.61958 0.77255 0.48576 0.95510
296 1.13001 4.15738 3.38757 3.17757 3.97857 2.98348 4.20830 3.09294 3.19189 2.97777 4.01983 3.34570 3.71568 3.54062 3.44647 2.50924 2.78140 2.76077 5.41351 4.20565 296 - -
2.68618 4.42225 2.77519 2.73123 3.46354 2.40513 3.72494 3.29354 2.67741 2.69355 4.24690 2.90347 2.73739 3.18146 2.89801 2.37887 2.77519 2.98518 4.58477 3.61503
0.03394 3.79601 4.51836 0.61958 0.77255 0.48576 0.95510
297 3.16380 4.58887 4.13307 3.83471 1.14586 3.86813 3.84824 2.69884 3.74404 2.08761 3.44167 3.93408 4.32984 3.93371 3.88264 3.43660 3.46563 2.68355 4.12324 2.50118 297 - -
2.68618 4.42225 2.77519 2.73123 3.46354 2.40513 3.72494 3.29354 2.67741 2.69355 4.24690 2.90347 2.73739 3.18146 2.89801 2.37887 2.77519 2.98518 4.58477 3.61503

0.03394 3.79601 4.51836 0.61958 0.77255 0.48576 0.95510

298 2.28079 4.19908 3.14317 2.95737 4.00099 2.93053 4.06839 3.51415 3.02585 3.24109 4.17505 3.19319 3.66344 3.38168 3.31291 1.21257 2.75555 3.06614 5.38010 4.08457 298 - -

2.68618 4.42225 2.77519 2.73123 3.46354 2.40513 3.72494 3.29354 2.67741 2.69355 4.24690 2.90347 2.73739 3.18146 2.89801 2.37887 2.77519 2.98518 4.58477 3.61503

0.03394 3.79601 4.51836 0.61958 0.77255 0.48576 0.95510

299 2.57535 4.35609 3.23321 3.12407 4.26351 0.85754 4.24721 3.84618 3.29467 3.50959 4.48627 3.39802 3.74476 3.62525 3.53733 2.74543 3.05073 3.38948 5.37705 4.36350 299 - -

2.68618 4.42225 2.77519 2.73123 3.46354 2.40513 3.72494 3.29354 2.67741 2.69355 4.24690 2.90347 2.73739 3.18146 2.89801 2.37887 2.77519 2.98518 4.58477 3.61503

0.03394 3.79601 4.51836 0.61958 0.77255 0.48576 0.95510

300 2.50546 1.10990 4.00398 3.72767 3.73846 3.14282 4.39180 2.87127 3.55467 2.78939 3.90900 3.73510 3.84371 3.90416 3.69196 2.77663 2.96608 2.62062 5.16879 4.02616 300 - -

2.68618 4.42225 2.77519 2.73123 3.46354 2.40513 3.72494 3.29354 2.67741 2.69355 4.24690 2.90347 2.73739 3.18146 2.89801 2.37887 2.77519 2.98518 4.58477 3.61503

0.03394 3.79601 4.51836 0.61958 0.77255 0.48576 0.95510

301 3.14031 4.66027 3.72681 3.44361 2.30221 3.74216 3.58865 3.16051 3.30332 2.63376 3.84409 3.63185 4.21871 3.62777 3.50199 3.28401 3.42715 3.02772 3.94242 1.10319 301 - -

2.68618 4.42225 2.77519 2.73123 3.46354 2.40513 3.72494 3.29354 2.67741 2.69355 4.24690 2.90347 2.73739 3.18146 2.89801 2.37887 2.77519 2.98518 4.58477 3.61503

0.03394 3.79601 4.51836 0.61958 0.77255 0.48576 0.95510

302 3.14031 4.66027 3.72681 3.44361 2.30221 3.74216 3.58865 3.16051 3.30332 2.63376 3.84409 3.63185 4.21871 3.62777 3.50199 3.28401 3.42715 3.02772 3.94242 1.10319 302 - -

2.68618 4.42225 2.77519 2.73123 3.46354 2.40513 3.72494 3.29354 2.67741 2.69355 4.24690 2.90347 2.73739 3.18146 2.89801 2.37887 2.77519 2.98518 4.58477 3.61503

0.03394 3.79601 4.51836 0.61958 0.77255 0.48576 0.95510

303 2.57535 4.35609 3.23321 3.12407 4.26351 0.85754 4.24721 3.84618 3.29467 3.50959 4.48627 3.39802 3.74476 3.62525 3.53733 2.74543 3.05073 3.38948 5.37705 4.36350 303 - -

2.68618 4.42225 2.77519 2.73123 3.46354 2.40513 3.72494 3.29354 2.67741 2.69355 4.24690 2.90347 2.73739 3.18146 2.89801 2.37887 2.77519 2.98518 4.58477 3.61503

0.03394 3.79601 4.51836 0.61958 0.77255 0.48576 0.95510

304 2.57535 4.35609 3.23321 3.12407 4.26351 0.85754 4.24721 3.84618 3.29467 3.50959 4.48627 3.39802 3.74476 3.62525 3.53733 2.74543 3.05073 3.38948 5.37705 4.36350 304 - -

2.68618 4.42225 2.77519 2.73123 3.46354 2.40513 3.72494 3.29354 2.67741 2.69355 4.24690 2.90347 2.73739 3.18146 2.89801 2.37887 2.77519 2.98518 4.58477 3.61503

0.03394 3.79601 4.51836 0.61958 0.77255 0.48576 0.95510

305 2.57535 4.35609 3.23321 3.12407 4.26351 0.85754 4.24721 3.84618 3.29467 3.50959 4.48627 3.39802 3.74476 3.62525 3.53733 2.74543 3.05073 3.38948 5.37705 4.36350 305 - -

2.68618 4.42225 2.77519 2.73123 3.46354 2.40513 3.72494 3.29354 2.67741 2.69355 4.24690 2.90347 2.73739 3.18146 2.89801 2.37887 2.77519 2.98518 4.58477 3.61503

0.03394 3.79601 4.51836 0.61958 0.77255 0.48576 0.95510

306 2.89015 4.98001 2.46169 1.13957 4.31725 3.22729 3.81015 3.75600 2.68126 3.38645 4.36600 2.89555 3.82889 3.02881 3.08050 2.88174 3.19454 3.44858 5.50348 4.22803 306 - -

2.68618 4.42225 2.77519 2.73123 3.46354 2.40513 3.72494 3.29354 2.67741 2.69355 4.24690 2.90347 2.73739 3.18146 2.89801 2.37887 2.77519 2.98518 4.58477 3.61503

0.03394 3.79601 4.51836 0.61958 0.77255 0.48576 0.95510

307 2.90487 4.86160 3.07558 2.71397 4.23048 3.40048 3.72190 3.65540 1.14833 3.23828 4.20575 3.14113 3.90783 2.91915 2.42439 2.95301 3.16487 3.36817 5.31817 4.12060 307 - -

2.68618 4.42225 2.77519 2.73123 3.46354 2.40513 3.72494 3.29354 2.67741 2.69355 4.24690 2.90347 2.73739 3.18146 2.89801 2.37887 2.77519 2.98518 4.58477 3.61503

0.03394 3.79601 4.51836 0.61958 0.77255 0.48576 0.95510

308 2.89015 4.98001 2.46169 1.13957 4.31725 3.22729 3.81015 3.75600 2.68126 3.38645 4.36600 2.89555 3.82889 3.02881 3.08050 2.88174 3.19454 3.44858 5.50348 4.22803 308 - -

2.68618 4.42225 2.77519 2.73123 3.46354 2.40513 3.72494 3.29354 2.67741 2.69355 4.24690 2.90347 2.73739 3.18146 2.89801 2.37887 2.77519 2.98518 4.58477 3.61503

0.03394 3.79601 4.51836 0.61958 0.77255 0.48576 0.95510

309 2.92251 4.76322 3.40570 2.93547 4.09844 3.42264 3.77482 3.63647 2.21535 3.16651 4.17059 3.30011 3.93066 3.00062 1.10219 3.01149 3.19482 3.36420 5.21367 4.04432 309 - -

2.68618 4.42225 2.77519 2.73123 3.46354 2.40513 3.72494 3.29354 2.67741 2.69355 4.24690 2.90347 2.73739 3.18146 2.89801 2.37887 2.77519 2.98518 4.58477 3.61503

0.03394 3.79601 4.51836 0.61958 0.77255 0.48576 0.95510

310 2.90487 4.86160 3.07558 2.71397 4.23048 3.40048 3.72190 3.65540 1.14833 3.23828 4.20575 3.14113 3.90783 2.91915 2.42439 2.95301 3.16487 3.36817 5.31817 4.12060 310 - -

2.68618 4.42225 2.77519 2.73123 3.46354 2.40513 3.72494 3.29354 2.67741 2.69355 4.24690 2.90347 2.73739 3.18146 2.89801 2.37887 2.77519 2.98518 4.58477 3.61503

0.03394 3.79601 4.51836 0.61958 0.77255 0.48576 0.95510

311 2.89015 4.98001 2.46169 1.13957 4.31725 3.22729 3.81015 3.75600 2.68126 3.38645 4.36600 2.89555 3.82889 3.02881 3.08050 2.88174 3.19454 3.44858 5.50348 4.22803 311 - -

2.68618 4.42225 2.77519 2.73123 3.46354 2.40513 3.72494 3.29354 2.67741 2.69355 4.24690 2.90347 2.73739 3.18146 2.89801 2.37887 2.77519 2.98518 4.58477 3.61503

0.03394 3.79601 4.51836 0.61958 0.77255 0.48576 0.95510

312 3.07120 4.55728 4.06560 3.70718 3.10996 3.88305 4.38695 2.31238 3.47558 0.97563 3.16058 3.98980 4.33298 3.86233 3.66334 3.44792 3.36975 2.32945 4.95943 3.66445 312 - -

2.68618 4.42225 2.77519 2.73123 3.46354 2.40513 3.72494 3.29354 2.67741 2.69355 4.24690 2.90347 2.73739 3.18146 2.89801 2.37887 2.77519 2.98518 4.58477 3.61503

0.03394 3.79601 4.51836 0.61958 0.77255 0.48576 0.95510

313 2.57535 4.35609 3.23321 3.12407 4.26351 0.85754 4.24721 3.84618 3.29467 3.50959 4.48627 3.39802 3.74476 3.62525 3.53733 2.74543 3.05073 3.38948 5.37705 4.36350 313 - -

2.68618 4.42225 2.77519 2.73123 3.46354 2.40513 3.72494 3.29354 2.67741 2.69355 4.24690 2.90347 2.73739 3.18146 2.89801 2.37887 2.77519 2.98518 4.58477 3.61503

0.03394 3.79601 4.51836 0.61958 0.77255 0.48576 0.95510

314 1.13001 4.15738 3.38757 3.17757 3.97857 2.98348 4.20830 3.09294 3.19189 2.97777 4.01983 3.34570 3.71568 3.54062 3.44647 2.50924 2.78140 2.76077 5.41351 4.20565 314 - -

2.68618 4.42225 2.77519 2.73123 3.46354 2.40513 3.72494 3.29354 2.67741 2.69355 4.24690 2.90347 2.73739 3.18146 2.89801 2.37887 2.77519 2.98518 4.58477 3.61503

0.03394 3.79601 4.51836 0.61958 0.77255 0.48576 0.95510

315 2.97165 4.41429 4.22289 3.83744 3.32476 3.98045 4.58108 1.17818 3.66390 1.92890 3.27450 4.09357 4.42084 4.02132 3.86934 3.47002 3.27299 1.84997 5.19827 3.92855 315 - -

2.68618 4.42225 2.77519 2.73123 3.46354 2.40513 3.72494 3.29354 2.67741 2.69355 4.24690 2.90347 2.73739 3.18146 2.89801 2.37887 2.77519 2.98518 4.58477 3.61503

0.03394 3.79601 4.51836 0.61958 0.77255 0.48576 0.95510

316 2.92251 4.76322 3.40570 2.93547 4.09844 3.42264 3.77482 3.63647 2.21535 3.16651 4.17059 3.30011 3.93066 3.00062 1.10219 3.01149 3.19482 3.36420 5.21367 4.04432 316 - -

2.68618 4.42225 2.77519 2.73123 3.46354 2.40513 3.72494 3.29354 2.67741 2.69355 4.24690 2.90347 2.73739 3.18146 2.89801 2.37887 2.77519 2.98518 4.58477 3.61503

0.03394 3.79601 4.51836 0.61958 0.77255 0.48576 0.95510

317 2.90487 4.86160 3.07558 2.71397 4.23048 3.40048 3.72190 3.65540 1.14833 3.23828 4.20575 3.14113 3.90783 2.91915 2.42439 2.95301 3.16487 3.36817 5.31817 4.12060 317 - -

2.68618 4.42225 2.77519 2.73123 3.46354 2.40513 3.72494 3.29354 2.67741 2.69355 4.24690 2.90347 2.73739 3.18146 2.89801 2.37887 2.77519 2.98518 4.58477 3.61503

0.03394 3.79601 4.51836 0.61958 0.77255 0.48576 0.95510

318 2.92251 4.76322 3.40570 2.93547 4.09844 3.42264 3.77482 3.63647 2.21535 3.16651 4.17059 3.30011 3.93066 3.00062 1.10219 3.01149 3.19482 3.36420 5.21367 4.04432 318 - -

2.68618 4.42225 2.77519 2.73123 3.46354 2.40513 3.72494 3.29354 2.67741 2.69355 4.24690 2.90347 2.73739 3.18146 2.89801 2.37887 2.77519 2.98518 4.58477 3.61503

0.03394 3.79601 4.51836 0.61958 0.77255 0.48576 0.95510

319 3.21457 4.61548 3.97805 3.66660 2.59647 3.64757 3.81244 3.23661 3.39107 2.64390 3.87379 3.83150 4.15944 3.78287 3.53232 3.41887 3.51155 3.12484 1.05487 2.60290 319 - -

2.68618 4.42225 2.77519 2.73123 3.46354 2.40513 3.72494 3.29354 2.67741 2.69355 4.24690 2.90347 2.73739 3.18146 2.89801 2.37887 2.77519 2.98518 4.58477 3.61503

0.03394 3.79601 4.51836 0.61958 0.77255 0.48576 0.95510

320 2.90487 4.86160 3.07558 2.71397 4.23048 3.40048 3.72190 3.65540 1.14833 3.23828 4.20575 3.14113 3.90783 2.91915 2.42439 2.95301 3.16487 3.36817 5.31817 4.12060 320 - -

2.68618 4.42225 2.77519 2.73123 3.46354 2.40513 3.72494 3.29354 2.67741 2.69355 4.24690 2.90347 2.73739 3.18146 2.89801 2.37887 2.77519 2.98518 4.58477 3.61503

0.03394 3.79601 4.51836 0.61958 0.77255 0.48576 0.95510

321 2.37514 4.21936 3.36356 3.11439 3.90452 3.06054 4.13711 3.02248 3.05582 2.88857 3.94573 3.33472 3.75900 3.45502 3.31699 2.57577 1.30864 2.72567 5.34922 4.12174 321 - -
2.68618 4.42225 2.77519 2.73123 3.46354 2.40513 3.72494 3.29354 2.67741 2.69355 4.24690 2.90347 2.73739 3.18146 2.89801 2.37887 2.77519 2.98518 4.58477 3.61503
0.03394 3.79601 4.51836 0.61958 0.77255 0.48576 0.95510
322 3.07120 4.55728 4.06560 3.70718 3.10996 3.88305 4.38695 2.31238 3.47558 0.97563 3.16058 3.98980 4.33298 3.86233 3.66334 3.44792 3.36975 2.32945 4.95943 3.66445 322 - -
2.68618 4.42225 2.77519 2.73123 3.46354 2.40513 3.72494 3.29354 2.67741 2.69355 4.24690 2.90347 2.73739 3.18146 2.89801 2.37887 2.77519 2.98518 4.58477 3.61503
0.03394 3.79601 4.51836 0.61958 0.77255 0.48576 0.95510
323 2.91724 4.74509 3.05672 2.81086 3.23540 3.38653 1.31468 3.60804 2.65623 3.11092 4.14942 3.22253 3.93587 3.17665 2.92559 2.98918 3.21880 3.34482 4.70119 3.18882 323 - -
2.68618 4.42225 2.77519 2.73123 3.46354 2.40513 3.72494 3.29354 2.67741 2.69355 4.24690 2.90347 2.73739 3.18146 2.89801 2.37887 2.77519 2.98518 4.58477 3.61503
0.03394 3.79601 4.51836 0.61958 0.77255 0.48576 0.95510
324 1.13001 4.15738 3.38757 3.17757 3.97857 2.98348 4.20830 3.09294 3.19189 2.97777 4.01983 3.34570 3.71568 3.54062 3.44647 2.50924 2.78140 2.76077 5.41351 4.20565 324 - -
2.68618 4.42225 2.77519 2.73123 3.46354 2.40513 3.72494 3.29354 2.67741 2.69355 4.24690 2.90347 2.73739 3.18146 2.89801 2.37887 2.77519 2.98518 4.58477 3.61503
0.03394 3.79601 4.51836 0.61958 0.77255 0.48576 0.95510
325 2.97165 4.41429 4.22289 3.83744 3.32476 3.98045 4.58108 1.17818 3.66390 1.92890 3.27450 4.09357 4.42084 4.02132 3.86934 3.47002 3.27299 1.84997 5.19827 3.92855 325 - -
2.68618 4.42225 2.77519 2.73123 3.46354 2.40513 3.72494 3.29354 2.67741 2.69355 4.24690 2.90347 2.73739 3.18146 2.89801 2.37887 2.77519 2.98518 4.58477 3.61503
0.03394 3.79601 4.51836 0.61958 0.77255 0.48576 0.95510
326 2.66315 4.65392 2.67213 2.55089 4.00571 3.13505 3.86476 3.70148 2.76309 3.35772 4.31495 1.30116 3.78410 3.13903 3.11371 2.73434 3.04351 3.33145 5.32076 3.96081 326 - -
2.68618 4.42225 2.77519 2.73123 3.46354 2.40513 3.72494 3.29354 2.67741 2.69355 4.24690 2.90347 2.73739 3.18146 2.89801 2.37887 2.77519 2.98518 4.58477 3.61503
0.03394 3.79601 4.51836 0.61958 0.77255 0.48576 0.95510
327 2.65642 4.42430 3.25570 3.08531 4.08348 3.11624 4.16757 3.61095 3.13637 3.24548 4.29346 3.39875 0.98293 3.52758 3.38897 2.82198 3.09244 3.26489 5.28934 4.20377 327 - -
2.68618 4.42225 2.77519 2.73123 3.46354 2.40513 3.72494 3.29354 2.67741 2.69355 4.24690 2.90347 2.73739 3.18146 2.89801 2.37887 2.77519 2.98518 4.58477 3.61503
0.03394 3.79601 4.51836 0.61958 0.77255 0.48576 0.95510
328 2.89015 4.98001 2.46169 1.13957 4.31725 3.22729 3.81015 3.75600 2.68126 3.38645 4.36600 2.89555 3.82889 3.02881 3.08050 2.88174 3.19454 3.44858 5.50348 4.22803 328 - -
2.68618 4.42225 2.77519 2.73123 3.46354 2.40513 3.72494 3.29354 2.67741 2.69355 4.24690 2.90347 2.73739 3.18146 2.89801 2.37887 2.77519 2.98518 4.58477 3.61503
0.03394 3.79601 4.51836 0.61958 0.77255 0.48576 0.95510
329 2.90487 4.86160 3.07558 2.71397 4.23048 3.40048 3.72190 3.65540 1.14833 3.23828 4.20575 3.14113 3.90783 2.91915 2.42439 2.95301 3.16487 3.36817 5.31817 4.12060 329 - -
2.68618 4.42225 2.77519 2.73123 3.46354 2.40513 3.72494 3.29354 2.67741 2.69355 4.24690 2.90347 2.73739 3.18146 2.89801 2.37887 2.77519 2.98518 4.58477 3.61503
0.03394 3.79601 4.51836 0.61958 0.77255 0.48576 0.95510
330 2.57535 4.35609 3.23321 3.12407 4.26351 0.85754 4.24721 3.84618 3.29467 3.50959 4.48627 3.39802 3.74476 3.62525 3.53733 2.74543 3.05073 3.38948 5.37705 4.36350 330 - -
2.68618 4.42225 2.77519 2.73123 3.46354 2.40513 3.72494 3.29354 2.67741 2.69355 4.24690 2.90347 2.73739 3.18146 2.89801 2.37887 2.77519 2.98518 4.58477 3.61503
0.03394 3.79601 4.51836 0.61958 0.77255 0.48576 0.95510
331 2.92251 4.76322 3.40570 2.93547 4.09844 3.42264 3.77482 3.63647 2.21535 3.16651 4.17059 3.30011 3.93066 3.00062 1.10219 3.01149 3.19482 3.36420 5.21367 4.04432 331 - -
2.68618 4.42225 2.77519 2.73123 3.46354 2.40513 3.72494 3.29354 2.67741 2.69355 4.24690 2.90347 2.73739 3.18146 2.89801 2.37887 2.77519 2.98518 4.58477 3.61503
0.03394 3.79601 4.51836 0.61958 0.77255 0.48576 0.95510
332 2.92251 4.76322 3.40570 2.93547 4.09844 3.42264 3.77482 3.63647 2.21535 3.16651 4.17059 3.30011 3.93066 3.00062 1.10219 3.01149 3.19482 3.36420 5.21367 4.04432 332 - -
2.68618 4.42225 2.77519 2.73123 3.46354 2.40513 3.72494 3.29354 2.67741 2.69355 4.24690 2.90347 2.73739 3.18146 2.89801 2.37887 2.77519 2.98518 4.58477 3.61503

0.03394 3.79601 4.51836 0.61958 0.77255 0.48576 0.95510

333 2.82402 4.80362 2.85663 2.61241 3.96136 3.31761 3.76927 3.58235 2.43912 3.09213 4.12436 3.07063 3.87138 1.37681 2.72390 2.87605 3.12210 3.31748 5.24814 3.93742 333 - -

2.68618 4.42225 2.77519 2.73123 3.46354 2.40513 3.72494 3.29354 2.67741 2.69355 4.24690 2.90347 2.73739 3.18146 2.89801 2.37887 2.77519 2.98518 4.58477 3.61503

0.03394 3.79601 4.51836 0.61958 0.77255 0.48576 0.95510

334 2.57535 4.35609 3.23321 3.12407 4.26351 0.85754 4.24721 3.84618 3.29467 3.50959 4.48627 3.39802 3.74476 3.62525 3.53733 2.74543 3.05073 3.38948 5.37705 4.36350 334 - -

2.68618 4.42225 2.77519 2.73123 3.46354 2.40513 3.72494 3.29354 2.67741 2.69355 4.24690 2.90347 2.73739 3.18146 2.89801 2.37887 2.77519 2.98518 4.58477 3.61503

0.03394 3.79601 4.51836 0.61958 0.77255 0.48576 0.95510

335 2.92251 4.76322 3.40570 2.93547 4.09844 3.42264 3.77482 3.63647 2.21535 3.16651 4.17059 3.30011 3.93066 3.00062 1.10219 3.01149 3.19482 3.36420 5.21367 4.04432 335 - -

2.68618 4.42225 2.77519 2.73123 3.46354 2.40513 3.72494 3.29354 2.67741 2.69355 4.24690 2.90347 2.73739 3.18146 2.89801 2.37887 2.77519 2.98518 4.58477 3.61503

0.03394 3.79601 4.51836 0.61958 0.77255 0.48576 0.95510

336 2.50546 1.10990 4.00398 3.72767 3.73846 3.14282 4.39180 2.87127 3.55467 2.78939 3.90900 3.73510 3.84371 3.90416 3.69196 2.77663 2.96608 2.62062 5.16879 4.02616 336 - -

2.68618 4.42225 2.77519 2.73123 3.46354 2.40513 3.72494 3.29354 2.67741 2.69355 4.24690 2.90347 2.73739 3.18146 2.89801 2.37887 2.77519 2.98518 4.58477 3.61503

0.03394 3.79601 4.51836 0.61958 0.77255 0.48576 0.95510

337 2.65642 4.42430 3.25570 3.08531 4.08348 3.11624 4.16757 3.61095 3.13637 3.24548 4.29346 3.39875 0.98293 3.52758 3.38897 2.82198 3.09244 3.26489 5.28934 4.20377 337 - -

2.68618 4.42225 2.77519 2.73123 3.46354 2.40513 3.72494 3.29354 2.67741 2.69355 4.24690 2.90347 2.73739 3.18146 2.89801 2.37887 2.77519 2.98518 4.58477 3.61503

0.03394 3.79601 4.51836 0.61958 0.77255 0.48576 0.95510

338 3.07120 4.55728 4.06560 3.70718 3.10996 3.88305 4.38695 2.31238 3.47558 0.97563 3.16058 3.98980 4.33298 3.86233 3.66334 3.44792 3.36975 2.32945 4.95943 3.66445 338 - -

2.68618 4.42225 2.77519 2.73123 3.46354 2.40513 3.72494 3.29354 2.67741 2.69355 4.24690 2.90347 2.73739 3.18146 2.89801 2.37887 2.77519 2.98518 4.58477 3.61503

0.03394 3.79601 4.51836 0.61958 0.77255 0.48576 0.95510

339 2.37514 4.21936 3.36356 3.11439 3.90452 3.06054 4.13711 3.02248 3.05582 2.88857 3.94573 3.33472 3.75900 3.45502 3.31699 2.57577 1.30864 2.72567 5.34922 4.12174 339 - -

2.68618 4.42225 2.77519 2.73123 3.46354 2.40513 3.72494 3.29354 2.67741 2.69355 4.24690 2.90347 2.73739 3.18146 2.89801 2.37887 2.77519 2.98518 4.58477 3.61503

0.03394 3.79601 4.51836 0.61958 0.77255 0.48576 0.95510

340 2.65642 4.42430 3.25570 3.08531 4.08348 3.11624 4.16757 3.61095 3.13637 3.24548 4.29346 3.39875 0.98293 3.52758 3.38897 2.82198 3.09244 3.26489 5.28934 4.20377 340 - -

2.68618 4.42225 2.77519 2.73123 3.46354 2.40513 3.72494 3.29354 2.67741 2.69355 4.24690 2.90347 2.73739 3.18146 2.89801 2.37887 2.77519 2.98518 4.58477 3.61503

0.03394 3.79601 4.51836 0.61958 0.77255 0.48576 0.95510

341 2.89015 4.98001 2.46169 1.13957 4.31725 3.22729 3.81015 3.75600 2.68126 3.38645 4.36600 2.89555 3.82889 3.02881 3.08050 2.88174 3.19454 3.44858 5.50348 4.22803 341 - -

2.68618 4.42225 2.77519 2.73123 3.46354 2.40513 3.72494 3.29354 2.67741 2.69355 4.24690 2.90347 2.73739 3.18146 2.89801 2.37887 2.77519 2.98518 4.58477 3.61503

0.03394 3.79601 4.51836 0.61958 0.77255 0.48576 0.95510

342 2.89015 4.98001 2.46169 1.13957 4.31725 3.22729 3.81015 3.75600 2.68126 3.38645 4.36600 2.89555 3.82889 3.02881 3.08050 2.88174 3.19454 3.44858 5.50348 4.22803 342 - -

2.68618 4.42225 2.77519 2.73123 3.46354 2.40513 3.72494 3.29354 2.67741 2.69355 4.24690 2.90347 2.73739 3.18146 2.89801 2.37887 2.77519 2.98518 4.58477 3.61503

0.03394 3.79601 4.51836 0.61958 0.77255 0.48576 0.95510

343 2.74814 4.31398 4.01764 3.68039 3.49634 3.64254 4.49596 2.06131 3.54689 2.19820 3.47523 3.88188 4.20873 3.91312 3.76637 3.14284 3.11247 1.12759 5.26714 3.99307 343 - -

2.68618 4.42225 2.77519 2.73123 3.46354 2.40513 3.72494 3.29354 2.67741 2.69355 4.24690 2.90347 2.73739 3.18146 2.89801 2.37887 2.77519 2.98518 4.58477 3.61503

0.03394 3.79601 4.51836 0.61958 0.77255 0.48576 0.95510

344 2.57535 4.35609 3.23321 3.12407 4.26351 0.85754 4.24721 3.84618 3.29467 3.50959 4.48627 3.39802 3.74476 3.62525 3.53733 2.74543 3.05073 3.38948 5.37705 4.36350 344 - -

2.68618 4.42225 2.77519 2.73123 3.46354 2.40513 3.72494 3.29354 2.67741 2.69355 4.24690 2.90347 2.73739 3.18146 2.89801 2.37887 2.77519 2.98518 4.58477 3.61503
0.03394 3.79601 4.51836 0.61958 0.77255 0.48576 0.95510
345 3.07120 4.55728 4.06560 3.70718 3.10996 3.88305 4.38695 2.31238 3.47558 0.97563 3.16058 3.98980 4.33298 3.86233 3.66334 3.44792 3.36975 2.32945 4.95943 3.66445 345 - -
2.68618 4.42225 2.77519 2.73123 3.46354 2.40513 3.72494 3.29354 2.67741 2.69355 4.24690 2.90347 2.73739 3.18146 2.89801 2.37887 2.77519 2.98518 4.58477 3.61503
0.03394 3.79601 4.51836 0.61958 0.77255 0.48576 0.95510
346 2.91157 4.47328 3.96816 3.55750 3.17841 3.77696 4.31044 2.34654 3.32910 1.78626 1.53296 3.84393 4.23864 3.71698 3.54307 3.25192 3.21616 2.34333 5.00802 3.76205 346 - -
2.68618 4.42225 2.77519 2.73123 3.46354 2.40513 3.72494 3.29354 2.67741 2.69355 4.24690 2.90347 2.73739 3.18146 2.89801 2.37887 2.77519 2.98518 4.58477 3.61503
0.03394 3.79601 4.51836 0.61958 0.77255 0.48576 0.95510
347 3.07120 4.55728 4.06560 3.70718 3.10996 3.88305 4.38695 2.31238 3.47558 0.97563 3.16058 3.98980 4.33298 3.86233 3.66334 3.44792 3.36975 2.32945 4.95943 3.66445 347 - -
2.68618 4.42225 2.77519 2.73123 3.46354 2.40513 3.72494 3.29354 2.67741 2.69355 4.24690 2.90347 2.73739 3.18146 2.89801 2.37887 2.77519 2.98518 4.58477 3.61503
0.03394 3.79601 4.51836 0.61958 0.77255 0.48576 0.95510
348 2.92251 4.76322 3.40570 2.93547 4.09844 3.42264 3.77482 3.63647 2.21535 3.16651 4.17059 3.30011 3.93066 3.00062 1.10219 3.01149 3.19482 3.36420 5.21367 4.04432 348 - -
2.68618 4.42225 2.77519 2.73123 3.46354 2.40513 3.72494 3.29354 2.67741 2.69355 4.24690 2.90347 2.73739 3.18146 2.89801 2.37887 2.77519 2.98518 4.58477 3.61503
0.03394 3.79601 4.51836 0.61958 0.77255 0.48576 0.95510
349 1.13001 4.15738 3.38757 3.17757 3.97857 2.98348 4.20830 3.09294 3.19189 2.97777 4.01983 3.34570 3.71568 3.54062 3.44647 2.50924 2.78140 2.76077 5.41351 4.20565 349 - -
2.68618 4.42225 2.77519 2.73123 3.46354 2.40513 3.72494 3.29354 2.67741 2.69355 4.24690 2.90347 2.73739 3.18146 2.89801 2.37887 2.77519 2.98518 4.58477 3.61503
0.03394 3.79601 4.51836 0.61958 0.77255 0.48576 0.95510
350 3.07120 4.55728 4.06560 3.70718 3.10996 3.88305 4.38695 2.31238 3.47558 0.97563 3.16058 3.98980 4.33298 3.86233 3.66334 3.44792 3.36975 2.32945 4.95943 3.66445 350 - -
2.68618 4.42225 2.77519 2.73123 3.46354 2.40513 3.72494 3.29354 2.67741 2.69355 4.24690 2.90347 2.73739 3.18146 2.89801 2.37887 2.77519 2.98518 4.58477 3.61503
0.03394 3.79601 4.51836 0.61958 0.77255 0.48576 0.95510
351 2.57535 4.35609 3.23321 3.12407 4.26351 0.85754 4.24721 3.84618 3.29467 3.50959 4.48627 3.39802 3.74476 3.62525 3.53733 2.74543 3.05073 3.38948 5.37705 4.36350 351 - -
2.68618 4.42225 2.77519 2.73123 3.46354 2.40513 3.72494 3.29354 2.67741 2.69355 4.24690 2.90347 2.73739 3.18146 2.89801 2.37887 2.77519 2.98518 4.58477 3.61503
0.03394 3.79601 4.51836 0.61958 0.77255 0.48576 0.95510
352 3.14031 4.66027 3.72681 3.44361 2.30221 3.74216 3.58865 3.16051 3.30332 2.63376 3.84409 3.63185 4.21871 3.62777 3.50199 3.28401 3.42715 3.02772 3.94242 1.10319 352 - -
2.68618 4.42225 2.77519 2.73123 3.46354 2.40513 3.72494 3.29354 2.67741 2.69355 4.24690 2.90347 2.73739 3.18146 2.89801 2.37887 2.77519 2.98518 4.58477 3.61503
0.03394 3.79601 4.51836 0.61958 0.77255 0.48576 0.95510
353 2.92251 4.76322 3.40570 2.93547 4.09844 3.42264 3.77482 3.63647 2.21535 3.16651 4.17059 3.30011 3.93066 3.00062 1.10219 3.01149 3.19482 3.36420 5.21367 4.04432 353 - -
2.68618 4.42225 2.77519 2.73123 3.46354 2.40513 3.72494 3.29354 2.67741 2.69355 4.24690 2.90347 2.73739 3.18146 2.89801 2.37887 2.77519 2.98518 4.58477 3.61503
0.03394 3.79601 4.51836 0.61958 0.77255 0.48576 0.95510
354 2.66315 4.65392 2.67213 2.55089 4.00571 3.13505 3.86476 3.70148 2.76309 3.35772 4.31495 1.30116 3.78410 3.13903 3.11371 2.73434 3.04351 3.33145 5.32076 3.96081 354 - -
2.68618 4.42225 2.77519 2.73123 3.46354 2.40513 3.72494 3.29354 2.67741 2.69355 4.24690 2.90347 2.73739 3.18146 2.89801 2.37887 2.77519 2.98518 4.58477 3.61503
0.03394 3.79601 4.51836 0.61958 0.77255 0.48576 0.95510
355 2.91056 5.02139 1.03432 2.29423 4.38899 3.15549 3.86877 3.96045 2.93927 3.58342 4.56179 2.84589 3.80315 3.10925 3.44342 2.88302 3.24824 3.60850 5.57234 4.27708 355 - -
2.68618 4.42225 2.77519 2.73123 3.46354 2.40513 3.72494 3.29354 2.67741 2.69355 4.24690 2.90347 2.73739 3.18146 2.89801 2.37887 2.77519 2.98518 4.58477 3.61503
0.03394 3.79601 4.51836 0.61958 0.77255 0.48576 0.95510

356 2.74814 4.31398 4.01764 3.68039 3.49634 3.64254 4.49596 2.06131 3.54689 2.19820 3.47523 3.88188 4.20873 3.91312 3.76637 3.14284 3.11247 1.12759 5.26714 3.99307 356 - -
2.68618 4.42225 2.77519 2.73123 3.46354 2.40513 3.72494 3.29354 2.67741 2.69355 4.24690 2.90347 2.73739 3.18146 2.89801 2.37887 2.77519 2.98518 4.58477 3.61503
0.03394 3.79601 4.51836 0.61958 0.77255 0.48576 0.95510
357 2.91724 4.74509 3.05672 2.81086 3.23540 3.38653 1.31468 3.60804 2.65623 3.11092 4.14942 3.22253 3.93587 3.17665 2.92559 2.98918 3.21880 3.34482 4.70119 3.18882 357 - -
2.68618 4.42225 2.77519 2.73123 3.46354 2.40513 3.72494 3.29354 2.67741 2.69355 4.24690 2.90347 2.73739 3.18146 2.89801 2.37887 2.77519 2.98518 4.58477 3.61503
0.03394 3.79601 4.51836 0.61958 0.77255 0.48576 0.95510
358 2.97165 4.41429 4.22289 3.83744 3.32476 3.98045 4.58108 1.17818 3.66390 1.92890 3.27450 4.09357 4.42084 4.02132 3.86934 3.47002 3.27299 1.84997 5.19827 3.92855 358 - -
2.68618 4.42225 2.77519 2.73123 3.46354 2.40513 3.72494 3.29354 2.67741 2.69355 4.24690 2.90347 2.73739 3.18146 2.89801 2.37887 2.77519 2.98518 4.58477 3.61503
0.03394 3.79601 4.51836 0.61958 0.77255 0.48576 0.95510
359 3.14031 4.66027 3.72681 3.44361 2.30221 3.74216 3.58865 3.16051 3.30332 2.63376 3.84409 3.63185 4.21871 3.62777 3.50199 3.28401 3.42715 3.02772 3.94242 1.10319 359 - -
2.68618 4.42225 2.77519 2.73123 3.46354 2.40513 3.72494 3.29354 2.67741 2.69355 4.24690 2.90347 2.73739 3.18146 2.89801 2.37887 2.77519 2.98518 4.58477 3.61503
0.03394 3.79601 4.51836 0.61958 0.77255 0.48576 0.95510
360 2.74814 4.31398 4.01764 3.68039 3.49634 3.64254 4.49596 2.06131 3.54689 2.19820 3.47523 3.88188 4.20873 3.91312 3.76637 3.14284 3.11247 1.12759 5.26714 3.99307 360 - -
2.68618 4.42225 2.77519 2.73123 3.46354 2.40513 3.72494 3.29354 2.67741 2.69355 4.24690 2.90347 2.73739 3.18146 2.89801 2.37887 2.77519 2.98518 4.58477 3.61503
0.03394 3.79601 4.51836 0.61958 0.77255 0.48576 0.95510
361 1.13001 4.15738 3.38757 3.17757 3.97857 2.98348 4.20830 3.09294 3.19189 2.97777 4.01983 3.34570 3.71568 3.54062 3.44647 2.50924 2.78140 2.76077 5.41351 4.20565 361 - -
2.68618 4.42225 2.77519 2.73123 3.46354 2.40513 3.72494 3.29354 2.67741 2.69355 4.24690 2.90347 2.73739 3.18146 2.89801 2.37887 2.77519 2.98518 4.58477 3.61503
0.03394 3.79601 4.51836 0.61958 0.77255 0.48576 0.95510
362 2.28079 4.19908 3.14317 2.95737 4.00099 2.93053 4.06839 3.51415 3.02585 3.24109 4.17505 3.19319 3.66344 3.38168 3.31291 1.21257 2.75555 3.06614 5.38010 4.08457 362 - -
2.68618 4.42225 2.77519 2.73123 3.46354 2.40513 3.72494 3.29354 2.67741 2.69355 4.24690 2.90347 2.73739 3.18146 2.89801 2.37887 2.77519 2.98518 4.58477 3.61503
0.03394 3.79601 4.51836 0.61958 0.77255 0.48576 0.95510
363 2.57535 4.35609 3.23321 3.12407 4.26351 0.85754 4.24721 3.84618 3.29467 3.50959 4.48627 3.39802 3.74476 3.62525 3.53733 2.74543 3.05073 3.38948 5.37705 4.36350 363 - -
2.68618 4.42225 2.77519 2.73123 3.46354 2.40513 3.72494 3.29354 2.67741 2.69355 4.24690 2.90347 2.73739 3.18146 2.89801 2.37887 2.77519 2.98518 4.58477 3.61503
0.03394 3.79601 4.51836 0.61958 0.77255 0.48576 0.95510
364 2.89015 4.98001 2.46169 1.13957 4.31725 3.22729 3.81015 3.75600 2.68126 3.38645 4.36600 2.89555 3.82889 3.02881 3.08050 2.88174 3.19454 3.44858 5.50348 4.22803 364 - -
2.68618 4.42225 2.77519 2.73123 3.46354 2.40513 3.72494 3.29354 2.67741 2.69355 4.24690 2.90347 2.73739 3.18146 2.89801 2.37887 2.77519 2.98518 4.58477 3.61503
0.03394 3.79601 4.51836 0.61958 0.77255 0.48576 0.95510
365 2.97165 4.41429 4.22289 3.83744 3.32476 3.98045 4.58108 1.17818 3.66390 1.92890 3.27450 4.09357 4.42084 4.02132 3.86934 3.47002 3.27299 1.84997 5.19827 3.92855 365 - -
2.68618 4.42225 2.77519 2.73123 3.46354 2.40513 3.72494 3.29354 2.67741 2.69355 4.24690 2.90347 2.73739 3.18146 2.89801 2.37887 2.77519 2.98518 4.58477 3.61503
0.03394 3.79601 4.51836 0.61958 0.77255 0.48576 0.95510
366 3.14031 4.66027 3.72681 3.44361 2.30221 3.74216 3.58865 3.16051 3.30332 2.63376 3.84409 3.63185 4.21871 3.62777 3.50199 3.28401 3.42715 3.02772 3.94242 1.10319 366 - -
2.68618 4.42225 2.77519 2.73123 3.46354 2.40513 3.72494 3.29354 2.67741 2.69355 4.24690 2.90347 2.73739 3.18146 2.89801 2.37887 2.77519 2.98518 4.58477 3.61503
0.03394 3.79601 4.51836 0.61958 0.77255 0.48576 0.95510
367 2.57535 4.35609 3.23321 3.12407 4.26351 0.85754 4.24721 3.84618 3.29467 3.50959 4.48627 3.39802 3.74476 3.62525 3.53733 2.74543 3.05073 3.38948 5.37705 4.36350 367 - -
2.68618 4.42225 2.77519 2.73123 3.46354 2.40513 3.72494 3.29354 2.67741 2.69355 4.24690 2.90347 2.73739 3.18146 2.89801 2.37887 2.77519 2.98518 4.58477 3.61503

0.03394 3.79601 4.51836 0.61958 0.77255 0.48576 0.95510
368 2.57535 4.35609 3.23321 3.12407 4.26351 0.85754 4.24721 3.84618 3.29467 3.50959 4.48627 3.39802 3.74476 3.62525 3.53733 2.74543 3.05073 3.38948 5.37705 4.36350 368 - -
2.68618 4.42225 2.77519 2.73123 3.46354 2.40513 3.72494 3.29354 2.67741 2.69355 4.24690 2.90347 2.73739 3.18146 2.89801 2.37887 2.77519 2.98518 4.58477 3.61503
0.03394 3.79601 4.51836 0.61958 0.77255 0.48576 0.95510
369 1.13001 4.15738 3.38757 3.17757 3.97857 2.98348 4.20830 3.09294 3.19189 2.97777 4.01983 3.34570 3.71568 3.54062 3.44647 2.50924 2.78140 2.76077 5.41351 4.20565 369 - -
2.68618 4.42225 2.77519 2.73123 3.46354 2.40513 3.72494 3.29354 2.67741 2.69355 4.24690 2.90347 2.73739 3.18146 2.89801 2.37887 2.77519 2.98518 4.58477 3.61503
0.03394 3.79601 4.51836 0.61958 0.77255 0.48576 0.95510
370 2.92251 4.76322 3.40570 2.93547 4.09844 3.42264 3.77482 3.63647 2.21535 3.16651 4.17059 3.30011 3.93066 3.00062 1.10219 3.01149 3.19482 3.36420 5.21367 4.04432 370 - -
2.68618 4.42225 2.77519 2.73123 3.46354 2.40513 3.72494 3.29354 2.67741 2.69355 4.24690 2.90347 2.73739 3.18146 2.89801 2.37887 2.77519 2.98518 4.58477 3.61503
0.03394 3.79601 4.51836 0.61958 0.77255 0.48576 0.95510
371 2.37514 4.21936 3.36356 3.11439 3.90452 3.06054 4.13711 3.02248 3.05582 2.88857 3.94573 3.33472 3.75900 3.45502 3.31699 2.57577 1.30864 2.72567 5.34922 4.12174 371 - -
2.68618 4.42225 2.77519 2.73123 3.46354 2.40513 3.72494 3.29354 2.67741 2.69355 4.24690 2.90347 2.73739 3.18146 2.89801 2.37887 2.77519 2.98518 4.58477 3.61503
0.03394 3.79601 4.51836 0.61958 0.77255 0.48576 0.95510
372 3.07120 4.55728 4.06560 3.70718 3.10996 3.88305 4.38695 2.31238 3.47558 0.97563 3.16058 3.98980 4.33298 3.86233 3.66334 3.44792 3.36975 2.32945 4.95943 3.66445 372 - -
2.68618 4.42225 2.77519 2.73123 3.46354 2.40513 3.72494 3.29354 2.67741 2.69355 4.24690 2.90347 2.73739 3.18146 2.89801 2.37887 2.77519 2.98518 4.58477 3.61503
0.03394 3.79601 4.51836 0.61958 0.77255 0.48576 0.95510
373 1.13001 4.15738 3.38757 3.17757 3.97857 2.98348 4.20830 3.09294 3.19189 2.97777 4.01983 3.34570 3.71568 3.54062 3.44647 2.50924 2.78140 2.76077 5.41351 4.20565 373 - -
2.68618 4.42225 2.77519 2.73123 3.46354 2.40513 3.72494 3.29354 2.67741 2.69355 4.24690 2.90347 2.73739 3.18146 2.89801 2.37887 2.77519 2.98518 4.58477 3.61503
0.03394 3.79601 4.51836 0.61958 0.77255 0.48576 0.95510
374 2.65642 4.42430 3.25570 3.08531 4.08348 3.11624 4.16757 3.61095 3.13637 3.24548 4.29346 3.39875 0.98293 3.52758 3.38897 2.82198 3.09244 3.26489 5.28934 4.20377 374 - -
2.68618 4.42225 2.77519 2.73123 3.46354 2.40513 3.72494 3.29354 2.67741 2.69355 4.24690 2.90347 2.73739 3.18146 2.89801 2.37887 2.77519 2.98518 4.58477 3.61503
0.03394 3.79601 4.51836 0.61958 0.77255 0.48576 0.95510
375 3.07120 4.55728 4.06560 3.70718 3.10996 3.88305 4.38695 2.31238 3.47558 0.97563 3.16058 3.98980 4.33298 3.86233 3.66334 3.44792 3.36975 2.32945 4.95943 3.66445 375 - -
2.68618 4.42225 2.77519 2.73123 3.46354 2.40513 3.72494 3.29354 2.67741 2.69355 4.24690 2.90347 2.73739 3.18146 2.89801 2.37887 2.77519 2.98518 4.58477 3.61503
0.03394 3.79601 4.51836 0.61958 0.77255 0.48576 0.95510
376 2.90487 4.86160 3.07558 2.71397 4.23048 3.40048 3.72190 3.65540 1.14833 3.23828 4.20575 3.14113 3.90783 2.91915 2.42439 2.95301 3.16487 3.36817 5.31817 4.12060 376 - -
2.68618 4.42225 2.77519 2.73123 3.46354 2.40513 3.72494 3.29354 2.67741 2.69355 4.24690 2.90347 2.73739 3.18146 2.89801 2.37887 2.77519 2.98518 4.58477 3.61503
0.03394 3.79601 4.51836 0.61958 0.77255 0.48576 0.95510
377 1.13001 4.15738 3.38757 3.17757 3.97857 2.98348 4.20830 3.09294 3.19189 2.97777 4.01983 3.34570 3.71568 3.54062 3.44647 2.50924 2.78140 2.76077 5.41351 4.20565 377 - -
2.68618 4.42225 2.77519 2.73123 3.46354 2.40513 3.72494 3.29354 2.67741 2.69355 4.24690 2.90347 2.73739 3.18146 2.89801 2.37887 2.77519 2.98518 4.58477 3.61503
0.03394 3.79601 4.51836 0.61958 0.77255 0.48576 0.95510
378 3.16380 4.58887 4.13307 3.83471 1.14586 3.86813 3.84824 2.69884 3.74404 2.08761 3.44167 3.93408 4.32984 3.93371 3.88264 3.43660 3.46563 2.68355 4.12324 2.50118 378 - -
2.68618 4.42225 2.77519 2.73123 3.46354 2.40513 3.72494 3.29354 2.67741 2.69355 4.24690 2.90347 2.73739 3.18146 2.89801 2.37887 2.77519 2.98518 4.58477 3.61503
0.03394 3.79601 4.51836 0.61958 0.77255 0.48576 0.95510
379 3.16380 4.58887 4.13307 3.83471 1.14586 3.86813 3.84824 2.69884 3.74404 2.08761 3.44167 3.93408 4.32984 3.93371 3.88264 3.43660 3.46563 2.68355 4.12324 2.50118 379 - -

2.68618 4.42225 2.77519 2.73123 3.46354 2.40513 3.72494 3.29354 2.67741 2.69355 4.24690 2.90347 2.73739 3.18146 2.89801 2.37887 2.77519 2.98518 4.58477 3.61503

0.03394 3.79601 4.51836 0.61958 0.77255 0.48576 0.95510

380 2.65642 4.42430 3.25570 3.08531 4.08348 3.11624 4.16757 3.61095 3.13637 3.24548 4.29346 3.39875 0.98293 3.52758 3.38897 2.82198 3.09244 3.26489 5.28934 4.20377 380 - -

2.68618 4.42225 2.77519 2.73123 3.46354 2.40513 3.72494 3.29354 2.67741 2.69355 4.24690 2.90347 2.73739 3.18146 2.89801 2.37887 2.77519 2.98518 4.58477 3.61503

0.03394 3.79601 4.51836 0.61958 0.77255 0.48576 0.95510

381 2.66315 4.65392 2.67213 2.55089 4.00571 3.13505 3.86476 3.70148 2.76309 3.35772 4.31495 1.30116 3.78410 3.13903 3.11371 2.73434 3.04351 3.33145 5.32076 3.96081 381 - -

2.68618 4.42225 2.77519 2.73123 3.46354 2.40513 3.72494 3.29354 2.67741 2.69355 4.24690 2.90347 2.73739 3.18146 2.89801 2.37887 2.77519 2.98518 4.58477 3.61503

0.03394 3.79601 4.51836 0.61958 0.77255 0.48576 0.95510

382 3.07120 4.55728 4.06560 3.70718 3.10996 3.88305 4.38695 2.31238 3.47558 0.97563 3.16058 3.98980 4.33298 3.86233 3.66334 3.44792 3.36975 2.32945 4.95943 3.66445 382 - -

2.68618 4.42225 2.77519 2.73123 3.46354 2.40513 3.72494 3.29354 2.67741 2.69355 4.24690 2.90347 2.73739 3.18146 2.89801 2.37887 2.77519 2.98518 4.58477 3.61503

0.03394 3.79601 4.51836 0.61958 0.77255 0.48576 0.95510

383 2.91724 4.74509 3.05672 2.81086 3.23540 3.38653 1.31468 3.60804 2.65623 3.11092 4.14942 3.22253 3.93587 3.17665 2.92559 2.98918 3.21880 3.34482 4.70119 3.18882 383 - -

2.68618 4.42225 2.77519 2.73123 3.46354 2.40513 3.72494 3.29354 2.67741 2.69355 4.24690 2.90347 2.73739 3.18146 2.89801 2.37887 2.77519 2.98518 4.58477 3.61503

0.03394 3.79601 4.51836 0.61958 0.77255 0.48576 0.95510

384 2.37514 4.21936 3.36356 3.11439 3.90452 3.06054 4.13711 3.02248 3.05582 2.88857 3.94573 3.33472 3.75900 3.45502 3.31699 2.57577 1.30864 2.72567 5.34922 4.12174 384 - -

2.68618 4.42225 2.77519 2.73123 3.46354 2.40513 3.72494 3.29354 2.67741 2.69355 4.24690 2.90347 2.73739 3.18146 2.89801 2.37887 2.77519 2.98518 4.58477 3.61503

0.03394 3.79601 4.51836 0.61958 0.77255 0.48576 0.95510

385 2.90487 4.86160 3.07558 2.71397 4.23048 3.40048 3.72190 3.65540 1.14833 3.23828 4.20575 3.14113 3.90783 2.91915 2.42439 2.95301 3.16487 3.36817 5.31817 4.12060 385 - -

2.68618 4.42225 2.77519 2.73123 3.46354 2.40513 3.72494 3.29354 2.67741 2.69355 4.24690 2.90347 2.73739 3.18146 2.89801 2.37887 2.77519 2.98518 4.58477 3.61503

0.03394 3.79601 4.51836 0.61958 0.77255 0.48576 0.95510

386 2.89015 4.98001 2.46169 1.13957 4.31725 3.22729 3.81015 3.75600 2.68126 3.38645 4.36600 2.89555 3.82889 3.02881 3.08050 2.88174 3.19454 3.44858 5.50348 4.22803 386 - -

2.68618 4.42225 2.77519 2.73123 3.46354 2.40513 3.72494 3.29354 2.67741 2.69355 4.24690 2.90347 2.73739 3.18146 2.89801 2.37887 2.77519 2.98518 4.58477 3.61503

0.03394 3.79601 4.51836 0.61958 0.77255 0.48576 0.95510

387 2.37514 4.21936 3.36356 3.11439 3.90452 3.06054 4.13711 3.02248 3.05582 2.88857 3.94573 3.33472 3.75900 3.45502 3.31699 2.57577 1.30864 2.72567 5.34922 4.12174 387 - -

2.68618 4.42225 2.77519 2.73123 3.46354 2.40513 3.72494 3.29354 2.67741 2.69355 4.24690 2.90347 2.73739 3.18146 2.89801 2.37887 2.77519 2.98518 4.58477 3.61503

0.03394 3.79601 4.51836 0.61958 0.77255 0.48576 0.95510

388 2.97165 4.41429 4.22289 3.83744 3.32476 3.98045 4.58108 1.17818 3.66390 1.92890 3.27450 4.09357 4.42084 4.02132 3.86934 3.47002 3.27299 1.84997 5.19827 3.92855 388 - -

2.68618 4.42225 2.77519 2.73123 3.46354 2.40513 3.72494 3.29354 2.67741 2.69355 4.24690 2.90347 2.73739 3.18146 2.89801 2.37887 2.77519 2.98518 4.58477 3.61503

0.03394 3.79601 4.51836 0.61958 0.77255 0.48576 0.95510

389 2.28079 4.19908 3.14317 2.95737 4.00099 2.93053 4.06839 3.51415 3.02585 3.24109 4.17505 3.19319 3.66344 3.38168 3.31291 1.21257 2.75555 3.06614 5.38010 4.08457 389 - -

2.68618 4.42225 2.77519 2.73123 3.46354 2.40513 3.72494 3.29354 2.67741 2.69355 4.24690 2.90347 2.73739 3.18146 2.89801 2.37887 2.77519 2.98518 4.58477 3.61503

0.03394 3.79601 4.51836 0.61958 0.77255 0.48576 0.95510

390 2.28079 4.19908 3.14317 2.95737 4.00099 2.93053 4.06839 3.51415 3.02585 3.24109 4.17505 3.19319 3.66344 3.38168 3.31291 1.21257 2.75555 3.06614 5.38010 4.08457 390 - -

2.68618 4.42225 2.77519 2.73123 3.46354 2.40513 3.72494 3.29354 2.67741 2.69355 4.24690 2.90347 2.73739 3.18146 2.89801 2.37887 2.77519 2.98518 4.58477 3.61503

0.03394 3.79601 4.51836 0.61958 0.77255 0.48576 0.95510

391 2.90487 4.86160 3.07558 2.71397 4.23048 3.40048 3.72190 3.65540 1.14833 3.23828 4.20575 3.14113 3.90783 2.91915 2.42439 2.95301 3.16487 3.36817 5.31817 4.12060 391 - -
2.68618 4.42225 2.77519 2.73123 3.46354 2.40513 3.72494 3.29354 2.67741 2.69355 4.24690 2.90347 2.73739 3.18146 2.89801 2.37887 2.77519 2.98518 4.58477 3.61503
0.03394 3.79601 4.51836 0.61958 0.77255 0.48576 0.95510
392 2.89015 4.98001 2.46169 1.13957 4.31725 3.22729 3.81015 3.75600 2.68126 3.38645 4.36600 2.89555 3.82889 3.02881 3.08050 2.88174 3.19454 3.44858 5.50348 4.22803 392 - -
2.68618 4.42225 2.77519 2.73123 3.46354 2.40513 3.72494 3.29354 2.67741 2.69355 4.24690 2.90347 2.73739 3.18146 2.89801 2.37887 2.77519 2.98518 4.58477 3.61503
0.03394 3.79601 4.51836 0.61958 0.77255 0.48576 0.95510
393 2.89015 4.98001 2.46169 1.13957 4.31725 3.22729 3.81015 3.75600 2.68126 3.38645 4.36600 2.89555 3.82889 3.02881 3.08050 2.88174 3.19454 3.44858 5.50348 4.22803 393 - -
2.68618 4.42225 2.77519 2.73123 3.46354 2.40513 3.72494 3.29354 2.67741 2.69355 4.24690 2.90347 2.73739 3.18146 2.89801 2.37887 2.77519 2.98518 4.58477 3.61503
0.03394 3.79601 4.51836 0.61958 0.77255 0.48576 0.95510
394 3.07120 4.55728 4.06560 3.70718 3.10996 3.88305 4.38695 2.31238 3.47558 0.97563 3.16058 3.98980 4.33298 3.86233 3.66334 3.44792 3.36975 2.32945 4.95943 3.66445 394 - -
2.68618 4.42225 2.77519 2.73123 3.46354 2.40513 3.72494 3.29354 2.67741 2.69355 4.24690 2.90347 2.73739 3.18146 2.89801 2.37887 2.77519 2.98518 4.58477 3.61503
0.03394 3.79601 4.51836 0.61958 0.77255 0.48576 0.95510
395 1.13001 4.15738 3.38757 3.17757 3.97857 2.98348 4.20830 3.09294 3.19189 2.97777 4.01983 3.34570 3.71568 3.54062 3.44647 2.50924 2.78140 2.76077 5.41351 4.20565 395 - -
2.68618 4.42225 2.77519 2.73123 3.46354 2.40513 3.72494 3.29354 2.67741 2.69355 4.24690 2.90347 2.73739 3.18146 2.89801 2.37887 2.77519 2.98518 4.58477 3.61503
0.03394 3.79601 4.51836 0.61958 0.77255 0.48576 0.95510
396 2.65642 4.42430 3.25570 3.08531 4.08348 3.11624 4.16757 3.61095 3.13637 3.24548 4.29346 3.39875 0.98293 3.52758 3.38897 2.82198 3.09244 3.26489 5.28934 4.20377 396 - -
2.68618 4.42225 2.77519 2.73123 3.46354 2.40513 3.72494 3.29354 2.67741 2.69355 4.24690 2.90347 2.73739 3.18146 2.89801 2.37887 2.77519 2.98518 4.58477 3.61503
0.03394 3.79601 4.51836 0.61958 0.77255 0.48576 0.95510
397 3.16380 4.58887 4.13307 3.83471 1.14586 3.86813 3.84824 2.69884 3.74404 2.08761 3.44167 3.93408 4.32984 3.93371 3.88264 3.43660 3.46563 2.68355 4.12324 2.50118 397 - -
2.68618 4.42225 2.77519 2.73123 3.46354 2.40513 3.72494 3.29354 2.67741 2.69355 4.24690 2.90347 2.73739 3.18146 2.89801 2.37887 2.77519 2.98518 4.58477 3.61503
0.03394 3.79601 4.51836 0.61958 0.77255 0.48576 0.95510
398 2.28079 4.19908 3.14317 2.95737 4.00099 2.93053 4.06839 3.51415 3.02585 3.24109 4.17505 3.19319 3.66344 3.38168 3.31291 1.21257 2.75555 3.06614 5.38010 4.08457 398 - -
2.68618 4.42225 2.77519 2.73123 3.46354 2.40513 3.72494 3.29354 2.67741 2.69355 4.24690 2.90347 2.73739 3.18146 2.89801 2.37887 2.77519 2.98518 4.58477 3.61503
0.03394 3.79601 4.51836 0.61958 0.77255 0.48576 0.95510
399 2.90487 4.86160 3.07558 2.71397 4.23048 3.40048 3.72190 3.65540 1.14833 3.23828 4.20575 3.14113 3.90783 2.91915 2.42439 2.95301 3.16487 3.36817 5.31817 4.12060 399 - -
2.68618 4.42225 2.77519 2.73123 3.46354 2.40513 3.72494 3.29354 2.67741 2.69355 4.24690 2.90347 2.73739 3.18146 2.89801 2.37887 2.77519 2.98518 4.58477 3.61503
0.03394 3.79601 4.51836 0.61958 0.77255 0.48576 0.95510
400 3.14031 4.66027 3.72681 3.44361 2.30221 3.74216 3.58865 3.16051 3.30332 2.63376 3.84409 3.63185 4.21871 3.62777 3.50199 3.28401 3.42715 3.02772 3.94242 1.10319 400 - -
2.68618 4.42225 2.77519 2.73123 3.46354 2.40513 3.72494 3.29354 2.67741 2.69355 4.24690 2.90347 2.73739 3.18146 2.89801 2.37887 2.77519 2.98518 4.58477 3.61503
0.03394 3.79601 4.51836 0.61958 0.77255 0.48576 0.95510
401 2.28079 4.19908 3.14317 2.95737 4.00099 2.93053 4.06839 3.51415 3.02585 3.24109 4.17505 3.19319 3.66344 3.38168 3.31291 1.21257 2.75555 3.06614 5.38010 4.08457 401 - -
2.68618 4.42225 2.77519 2.73123 3.46354 2.40513 3.72494 3.29354 2.67741 2.69355 4.24690 2.90347 2.73739 3.18146 2.89801 2.37887 2.77519 2.98518 4.58477 3.61503
0.03394 3.79601 4.51836 0.61958 0.77255 0.48576 0.95510
402 2.28079 4.19908 3.14317 2.95737 4.00099 2.93053 4.06839 3.51415 3.02585 3.24109 4.17505 3.19319 3.66344 3.38168 3.31291 1.21257 2.75555 3.06614 5.38010 4.08457 402 - -
2.68618 4.42225 2.77519 2.73123 3.46354 2.40513 3.72494 3.29354 2.67741 2.69355 4.24690 2.90347 2.73739 3.18146 2.89801 2.37887 2.77519 2.98518 4.58477 3.61503

0.03394 3.79601 4.51836 0.61958 0.77255 0.48576 0.95510

403 2.92251 4.76322 3.40570 2.93547 4.09844 3.42264 3.77482 3.63647 2.21535 3.16651 4.17059 3.30011 3.93066 3.00062 1.10219 3.01149 3.19482 3.36420 5.21367 4.04432 403 - -

2.68618 4.42225 2.77519 2.73123 3.46354 2.40513 3.72494 3.29354 2.67741 2.69355 4.24690 2.90347 2.73739 3.18146 2.89801 2.37887 2.77519 2.98518 4.58477 3.61503

0.03394 3.79601 4.51836 0.61958 0.77255 0.48576 0.95510

404 2.91157 4.47328 3.96816 3.55750 3.17841 3.77696 4.31044 2.34654 3.32910 1.78626 1.53296 3.84393 4.23864 3.71698 3.54307 3.25192 3.21616 2.34333 5.00802 3.76205 404 - -

2.68618 4.42225 2.77519 2.73123 3.46354 2.40513 3.72494 3.29354 2.67741 2.69355 4.24690 2.90347 2.73739 3.18146 2.89801 2.37887 2.77519 2.98518 4.58477 3.61503

0.03394 3.79601 4.51836 0.61958 0.77255 0.48576 0.95510

405 1.13001 4.15738 3.38757 3.17757 3.97857 2.98348 4.20830 3.09294 3.19189 2.97777 4.01983 3.34570 3.71568 3.54062 3.44647 2.50924 2.78140 2.76077 5.41351 4.20565 405 - -

2.68618 4.42225 2.77519 2.73123 3.46354 2.40513 3.72494 3.29354 2.67741 2.69355 4.24690 2.90347 2.73739 3.18146 2.89801 2.37887 2.77519 2.98518 4.58477 3.61503

0.03394 3.79601 4.51836 0.61958 0.77255 0.48576 0.95510

406 1.13001 4.15738 3.38757 3.17757 3.97857 2.98348 4.20830 3.09294 3.19189 2.97777 4.01983 3.34570 3.71568 3.54062 3.44647 2.50924 2.78140 2.76077 5.41351 4.20565 406 - -

2.68618 4.42225 2.77519 2.73123 3.46354 2.40513 3.72494 3.29354 2.67741 2.69355 4.24690 2.90347 2.73739 3.18146 2.89801 2.37887 2.77519 2.98518 4.58477 3.61503

0.03394 3.79601 4.51836 0.61958 0.77255 0.48576 0.95510

407 3.07120 4.55728 4.06560 3.70718 3.10996 3.88305 4.38695 2.31238 3.47558 0.97563 3.16058 3.98980 4.33298 3.86233 3.66334 3.44792 3.36975 2.32945 4.95943 3.66445 407 - -

2.68618 4.42225 2.77519 2.73123 3.46354 2.40513 3.72494 3.29354 2.67741 2.69355 4.24690 2.90347 2.73739 3.18146 2.89801 2.37887 2.77519 2.98518 4.58477 3.61503

0.03394 3.79601 4.51836 0.61958 0.77255 0.48576 0.95510

408 2.91056 5.02139 1.03432 2.29423 4.38899 3.15549 3.86877 3.96045 2.93927 3.58342 4.56179 2.84589 3.80315 3.10925 3.44342 2.88302 3.24824 3.60850 5.57234 4.27708 408 - -

2.68618 4.42225 2.77519 2.73123 3.46354 2.40513 3.72494 3.29354 2.67741 2.69355 4.24690 2.90347 2.73739 3.18146 2.89801 2.37887 2.77519 2.98518 4.58477 3.61503

0.03394 3.79601 4.51836 0.61958 0.77255 0.48576 0.95510

409 3.16380 4.58887 4.13307 3.83471 1.14586 3.86813 3.84824 2.69884 3.74404 2.08761 3.44167 3.93408 4.32984 3.93371 3.88264 3.43660 3.46563 2.68355 4.12324 2.50118 409 - -

2.68618 4.42225 2.77519 2.73123 3.46354 2.40513 3.72494 3.29354 2.67741 2.69355 4.24690 2.90347 2.73739 3.18146 2.89801 2.37887 2.77519 2.98518 4.58477 3.61503

0.03394 3.79601 4.51836 0.61958 0.77255 0.48576 0.95510

410 2.97165 4.41429 4.22289 3.83744 3.32476 3.98045 4.58108 1.17818 3.66390 1.92890 3.27450 4.09357 4.42084 4.02132 3.86934 3.47002 3.27299 1.84997 5.19827 3.92855 410 - -

2.68618 4.42225 2.77519 2.73123 3.46354 2.40513 3.72494 3.29354 2.67741 2.69355 4.24690 2.90347 2.73739 3.18146 2.89801 2.37887 2.77519 2.98518 4.58477 3.61503

0.03394 3.79601 4.51836 0.61958 0.77255 0.48576 0.95510

411 2.74814 4.31398 4.01764 3.68039 3.49634 3.64254 4.49596 2.06131 3.54689 2.19820 3.47523 3.88188 4.20873 3.91312 3.76637 3.14284 3.11247 1.12759 5.26714 3.99307 411 - -

2.68618 4.42225 2.77519 2.73123 3.46354 2.40513 3.72494 3.29354 2.67741 2.69355 4.24690 2.90347 2.73739 3.18146 2.89801 2.37887 2.77519 2.98518 4.58477 3.61503

0.03394 3.79601 4.51836 0.61958 0.77255 0.48576 0.95510

412 2.50546 1.10990 4.00398 3.72767 3.73846 3.14282 4.39180 2.87127 3.55467 2.78939 3.90900 3.73510 3.84371 3.90416 3.69196 2.77663 2.96608 2.62062 5.16879 4.02616 412 - -

2.68618 4.42225 2.77519 2.73123 3.46354 2.40513 3.72494 3.29354 2.67741 2.69355 4.24690 2.90347 2.73739 3.18146 2.89801 2.37887 2.77519 2.98518 4.58477 3.61503

0.03394 3.79601 4.51836 0.61958 0.77255 0.48576 0.95510

413 2.91056 5.02139 1.03432 2.29423 4.38899 3.15549 3.86877 3.96045 2.93927 3.58342 4.56179 2.84589 3.80315 3.10925 3.44342 2.88302 3.24824 3.60850 5.57234 4.27708 413 - -

2.68618 4.42225 2.77519 2.73123 3.46354 2.40513 3.72494 3.29354 2.67741 2.69355 4.24690 2.90347 2.73739 3.18146 2.89801 2.37887 2.77519 2.98518 4.58477 3.61503

0.03394 3.79601 4.51836 0.61958 0.77255 0.48576 0.95510

414 2.57535 4.35609 3.23321 3.12407 4.26351 0.85754 4.24721 3.84618 3.29467 3.50959 4.48627 3.39802 3.74476 3.62525 3.53733 2.74543 3.05073 3.38948 5.37705 4.36350 414 - -

2.68618 4.42225 2.77519 2.73123 3.46354 2.40513 3.72494 3.29354 2.67741 2.69355 4.24690 2.90347 2.73739 3.18146 2.89801 2.37887 2.77519 2.98518 4.58477 3.61503

0.03394 3.79601 4.51836 0.61958 0.77255 0.48576 0.95510

415 2.28079 4.19908 3.14317 2.95737 4.00099 2.93053 4.06839 3.51415 3.02585 3.24109 4.17505 3.19319 3.66344 3.38168 3.31291 1.21257 2.75555 3.06614 5.38010 4.08457 415 - -

2.68618 4.42225 2.77519 2.73123 3.46354 2.40513 3.72494 3.29354 2.67741 2.69355 4.24690 2.90347 2.73739 3.18146 2.89801 2.37887 2.77519 2.98518 4.58477 3.61503

0.03394 3.79601 4.51836 0.61958 0.77255 0.48576 0.95510

416 2.91056 5.02139 1.03432 2.29423 4.38899 3.15549 3.86877 3.96045 2.93927 3.58342 4.56179 2.84589 3.80315 3.10925 3.44342 2.88302 3.24824 3.60850 5.57234 4.27708 416 - -

2.68618 4.42225 2.77519 2.73123 3.46354 2.40513 3.72494 3.29354 2.67741 2.69355 4.24690 2.90347 2.73739 3.18146 2.89801 2.37887 2.77519 2.98518 4.58477 3.61503

0.03394 3.79601 4.51836 0.61958 0.77255 0.48576 0.95510

417 1.13001 4.15738 3.38757 3.17757 3.97857 2.98348 4.20830 3.09294 3.19189 2.97777 4.01983 3.34570 3.71568 3.54062 3.44647 2.50924 2.78140 2.76077 5.41351 4.20565 417 - -

2.68618 4.42225 2.77519 2.73123 3.46354 2.40513 3.72494 3.29354 2.67741 2.69355 4.24690 2.90347 2.73739 3.18146 2.89801 2.37887 2.77519 2.98518 4.58477 3.61503

0.03394 3.79601 4.51836 0.61958 0.77255 0.48576 0.95510

418 3.16380 4.58887 4.13307 3.83471 1.14586 3.86813 3.84824 2.69884 3.74404 2.08761 3.44167 3.93408 4.32984 3.93371 3.88264 3.43660 3.46563 2.68355 4.12324 2.50118 418 - -

2.68618 4.42225 2.77519 2.73123 3.46354 2.40513 3.72494 3.29354 2.67741 2.69355 4.24690 2.90347 2.73739 3.18146 2.89801 2.37887 2.77519 2.98518 4.58477 3.61503

0.03394 3.79601 4.51836 0.61958 0.77255 0.48576 0.95510

419 2.74814 4.31398 4.01764 3.68039 3.49634 3.64254 4.49596 2.06131 3.54689 2.19820 3.47523 3.88188 4.20873 3.91312 3.76637 3.14284 3.11247 1.12759 5.26714 3.99307 419 - -

2.68618 4.42225 2.77519 2.73123 3.46354 2.40513 3.72494 3.29354 2.67741 2.69355 4.24690 2.90347 2.73739 3.18146 2.89801 2.37887 2.77519 2.98518 4.58477 3.61503

0.03394 3.79601 4.51836 0.61958 0.77255 0.48576 0.95510

420 2.37514 4.21936 3.36356 3.11439 3.90452 3.06054 4.13711 3.02248 3.05582 2.88857 3.94573 3.33472 3.75900 3.45502 3.31699 2.57577 1.30864 2.72567 5.34922 4.12174 420 - -

2.68618 4.42225 2.77519 2.73123 3.46354 2.40513 3.72494 3.29354 2.67741 2.69355 4.24690 2.90347 2.73739 3.18146 2.89801 2.37887 2.77519 2.98518 4.58477 3.61503

0.03394 3.79601 4.51836 0.61958 0.77255 0.48576 0.95510

421 2.66315 4.65392 2.67213 2.55089 4.00571 3.13505 3.86476 3.70148 2.76309 3.35772 4.31495 1.30116 3.78410 3.13903 3.11371 2.73434 3.04351 3.33145 5.32076 3.96081 421 - -

2.68618 4.42225 2.77519 2.73123 3.46354 2.40513 3.72494 3.29354 2.67741 2.69355 4.24690 2.90347 2.73739 3.18146 2.89801 2.37887 2.77519 2.98518 4.58477 3.61503

0.03394 3.79601 4.51836 0.61958 0.77255 0.48576 0.95510

422 2.66315 4.65392 2.67213 2.55089 4.00571 3.13505 3.86476 3.70148 2.76309 3.35772 4.31495 1.30116 3.78410 3.13903 3.11371 2.73434 3.04351 3.33145 5.32076 3.96081 422 - -

2.68618 4.42225 2.77519 2.73123 3.46354 2.40513 3.72494 3.29354 2.67741 2.69355 4.24690 2.90347 2.73739 3.18146 2.89801 2.37887 2.77519 2.98518 4.58477 3.61503

0.03394 3.79601 4.51836 0.61958 0.77255 0.48576 0.95510

423 2.66315 4.65392 2.67213 2.55089 4.00571 3.13505 3.86476 3.70148 2.76309 3.35772 4.31495 1.30116 3.78410 3.13903 3.11371 2.73434 3.04351 3.33145 5.32076 3.96081 423 - -

2.68618 4.42225 2.77519 2.73123 3.46354 2.40513 3.72494 3.29354 2.67741 2.69355 4.24690 2.90347 2.73739 3.18146 2.89801 2.37887 2.77519 2.98518 4.58477 3.61503

0.03394 3.79601 4.51836 0.61958 0.77255 0.48576 0.95510

424 2.57535 4.35609 3.23321 3.12407 4.26351 0.85754 4.24721 3.84618 3.29467 3.50959 4.48627 3.39802 3.74476 3.62525 3.53733 2.74543 3.05073 3.38948 5.37705 4.36350 424 - -

2.68618 4.42225 2.77519 2.73123 3.46354 2.40513 3.72494 3.29354 2.67741 2.69355 4.24690 2.90347 2.73739 3.18146 2.89801 2.37887 2.77519 2.98518 4.58477 3.61503

0.03394 3.79601 4.51836 0.61958 0.77255 0.48576 0.95510

425 2.66315 4.65392 2.67213 2.55089 4.00571 3.13505 3.86476 3.70148 2.76309 3.35772 4.31495 1.30116 3.78410 3.13903 3.11371 2.73434 3.04351 3.33145 5.32076 3.96081 425 - -

2.68618 4.42225 2.77519 2.73123 3.46354 2.40513 3.72494 3.29354 2.67741 2.69355 4.24690 2.90347 2.73739 3.18146 2.89801 2.37887 2.77519 2.98518 4.58477 3.61503

0.03394 3.79601 4.51836 0.61958 0.77255 0.48576 0.95510

426 2.91157 4.47328 3.96816 3.55750 3.17841 3.77696 4.31044 2.34654 3.32910 1.78626 1.53296 3.84393 4.23864 3.71698 3.54307 3.25192 3.21616 2.34333 5.00802 3.76205 426 - -
2.68618 4.42225 2.77519 2.73123 3.46354 2.40513 3.72494 3.29354 2.67741 2.69355 4.24690 2.90347 2.73739 3.18146 2.89801 2.37887 2.77519 2.98518 4.58477 3.61503
0.03394 3.79601 4.51836 0.61958 0.77255 0.48576 0.95510
427 1.13001 4.15738 3.38757 3.17757 3.97857 2.98348 4.20830 3.09294 3.19189 2.97777 4.01983 3.34570 3.71568 3.54062 3.44647 2.50924 2.78140 2.76077 5.41351 4.20565 427 - -
2.68618 4.42225 2.77519 2.73123 3.46354 2.40513 3.72494 3.29354 2.67741 2.69355 4.24690 2.90347 2.73739 3.18146 2.89801 2.37887 2.77519 2.98518 4.58477 3.61503
0.03394 3.79601 4.51836 0.61958 0.77255 0.48576 0.95510
428 2.90487 4.86160 3.07558 2.71397 4.23048 3.40048 3.72190 3.65540 1.14833 3.23828 4.20575 3.14113 3.90783 2.91915 2.42439 2.95301 3.16487 3.36817 5.31817 4.12060 428 - -
2.68618 4.42225 2.77519 2.73123 3.46354 2.40513 3.72494 3.29354 2.67741 2.69355 4.24690 2.90347 2.73739 3.18146 2.89801 2.37887 2.77519 2.98518 4.58477 3.61503
0.03394 3.79601 4.51836 0.61958 0.77255 0.48576 0.95510
429 2.97165 4.41429 4.22289 3.83744 3.32476 3.98045 4.58108 1.17818 3.66390 1.92890 3.27450 4.09357 4.42084 4.02132 3.86934 3.47002 3.27299 1.84997 5.19827 3.92855 429 - -
2.68618 4.42225 2.77519 2.73123 3.46354 2.40513 3.72494 3.29354 2.67741 2.69355 4.24690 2.90347 2.73739 3.18146 2.89801 2.37887 2.77519 2.98518 4.58477 3.61503
0.03394 3.79601 4.51836 0.61958 0.77255 0.48576 0.95510
430 3.07120 4.55728 4.06560 3.70718 3.10996 3.88305 4.38695 2.31238 3.47558 0.97563 3.16058 3.98980 4.33298 3.86233 3.66334 3.44792 3.36975 2.32945 4.95943 3.66445 430 - -
2.68618 4.42225 2.77519 2.73123 3.46354 2.40513 3.72494 3.29354 2.67741 2.69355 4.24690 2.90347 2.73739 3.18146 2.89801 2.37887 2.77519 2.98518 4.58477 3.61503
0.03394 3.79601 4.51836 0.61958 0.77255 0.48576 0.95510
431 1.13001 4.15738 3.38757 3.17757 3.97857 2.98348 4.20830 3.09294 3.19189 2.97777 4.01983 3.34570 3.71568 3.54062 3.44647 2.50924 2.78140 2.76077 5.41351 4.20565 431 - -
2.68618 4.42225 2.77519 2.73123 3.46354 2.40513 3.72494 3.29354 2.67741 2.69355 4.24690 2.90347 2.73739 3.18146 2.89801 2.37887 2.77519 2.98518 4.58477 3.61503
0.03394 3.79601 4.51836 0.61958 0.77255 0.48576 0.95510
432 2.57535 4.35609 3.23321 3.12407 4.26351 0.85754 4.24721 3.84618 3.29467 3.50959 4.48627 3.39802 3.74476 3.62525 3.53733 2.74543 3.05073 3.38948 5.37705 4.36350 432 - -
2.68618 4.42225 2.77519 2.73123 3.46354 2.40513 3.72494 3.29354 2.67741 2.69355 4.24690 2.90347 2.73739 3.18146 2.89801 2.37887 2.77519 2.98518 4.58477 3.61503
0.03394 3.79601 4.51836 0.61958 0.77255 0.48576 0.95510
433 2.92251 4.76322 3.40570 2.93547 4.09844 3.42264 3.77482 3.63647 2.21535 3.16651 4.17059 3.30011 3.93066 3.00062 1.10219 3.01149 3.19482 3.36420 5.21367 4.04432 433 - -
2.68618 4.42225 2.77519 2.73123 3.46354 2.40513 3.72494 3.29354 2.67741 2.69355 4.24690 2.90347 2.73739 3.18146 2.89801 2.37887 2.77519 2.98518 4.58477 3.61503
0.03394 3.79601 4.51836 0.61958 0.77255 0.48576 0.95510
434 2.92251 4.76322 3.40570 2.93547 4.09844 3.42264 3.77482 3.63647 2.21535 3.16651 4.17059 3.30011 3.93066 3.00062 1.10219 3.01149 3.19482 3.36420 5.21367 4.04432 434 - -
2.68618 4.42225 2.77519 2.73123 3.46354 2.40513 3.72494 3.29354 2.67741 2.69355 4.24690 2.90347 2.73739 3.18146 2.89801 2.37887 2.77519 2.98518 4.58477 3.61503
0.03394 3.79601 4.51836 0.61958 0.77255 0.48576 0.95510
435 2.92251 4.76322 3.40570 2.93547 4.09844 3.42264 3.77482 3.63647 2.21535 3.16651 4.17059 3.30011 3.93066 3.00062 1.10219 3.01149 3.19482 3.36420 5.21367 4.04432 435 - -
2.68618 4.42225 2.77519 2.73123 3.46354 2.40513 3.72494 3.29354 2.67741 2.69355 4.24690 2.90347 2.73739 3.18146 2.89801 2.37887 2.77519 2.98518 4.58477 3.61503
0.03394 3.79601 4.51836 0.61958 0.77255 0.48576 0.95510
436 3.14031 4.66027 3.72681 3.44361 2.30221 3.74216 3.58865 3.16051 3.30332 2.63376 3.84409 3.63185 4.21871 3.62777 3.50199 3.28401 3.42715 3.02772 3.94242 1.10319 436 - -
2.68618 4.42225 2.77519 2.73123 3.46354 2.40513 3.72494 3.29354 2.67741 2.69355 4.24690 2.90347 2.73739 3.18146 2.89801 2.37887 2.77519 2.98518 4.58477 3.61503
0.03394 3.79601 4.51836 0.61958 0.77255 0.48576 0.95510
437 3.16380 4.58887 4.13307 3.83471 1.14586 3.86813 3.84824 2.69884 3.74404 2.08761 3.44167 3.93408 4.32984 3.93371 3.88264 3.43660 3.46563 2.68355 4.12324 2.50118 437 - -
2.68618 4.42225 2.77519 2.73123 3.46354 2.40513 3.72494 3.29354 2.67741 2.69355 4.24690 2.90347 2.73739 3.18146 2.89801 2.37887 2.77519 2.98518 4.58477 3.61503

0.03394 3.79601 4.51836 0.61958 0.77255 0.48576 0.95510

438 2.57535 4.35609 3.23321 3.12407 4.26351 0.85754 4.24721 3.84618 3.29467 3.50959 4.48627 3.39802 3.74476 3.62525 3.53733 2.74543 3.05073 3.38948 5.37705 4.36350 438 - -

2.68618 4.42225 2.77519 2.73123 3.46354 2.40513 3.72494 3.29354 2.67741 2.69355 4.24690 2.90347 2.73739 3.18146 2.89801 2.37887 2.77519 2.98518 4.58477 3.61503

0.03394 3.79601 4.51836 0.61958 0.77255 0.48576 0.95510

439 2.91724 4.74509 3.05672 2.81086 3.23540 3.38653 1.31468 3.60804 2.65623 3.11092 4.14942 3.22253 3.93587 3.17665 2.92559 2.98918 3.21880 3.34482 4.70119 3.18882 439 - -

2.68618 4.42225 2.77519 2.73123 3.46354 2.40513 3.72494 3.29354 2.67741 2.69355 4.24690 2.90347 2.73739 3.18146 2.89801 2.37887 2.77519 2.98518 4.58477 3.61503

0.03394 3.79601 4.51836 0.61958 0.77255 0.48576 0.95510

440 2.90487 4.86160 3.07558 2.71397 4.23048 3.40048 3.72190 3.65540 1.14833 3.23828 4.20575 3.14113 3.90783 2.91915 2.42439 2.95301 3.16487 3.36817 5.31817 4.12060 440 - -

2.68618 4.42225 2.77519 2.73123 3.46354 2.40513 3.72494 3.29354 2.67741 2.69355 4.24690 2.90347 2.73739 3.18146 2.89801 2.37887 2.77519 2.98518 4.58477 3.61503

0.03394 3.79601 4.51836 0.61958 0.77255 0.48576 0.95510

441 2.92251 4.76322 3.40570 2.93547 4.09844 3.42264 3.77482 3.63647 2.21535 3.16651 4.17059 3.30011 3.93066 3.00062 1.10219 3.01149 3.19482 3.36420 5.21367 4.04432 441 - -

2.68618 4.42225 2.77519 2.73123 3.46354 2.40513 3.72494 3.29354 2.67741 2.69355 4.24690 2.90347 2.73739 3.18146 2.89801 2.37887 2.77519 2.98518 4.58477 3.61503

0.03394 3.79601 4.51836 0.61958 0.77255 0.48576 0.95510

442 2.37514 4.21936 3.36356 3.11439 3.90452 3.06054 4.13711 3.02248 3.05582 2.88857 3.94573 3.33472 3.75900 3.45502 3.31699 2.57577 1.30864 2.72567 5.34922 4.12174 442 - -

2.68618 4.42225 2.77519 2.73123 3.46354 2.40513 3.72494 3.29354 2.67741 2.69355 4.24690 2.90347 2.73739 3.18146 2.89801 2.37887 2.77519 2.98518 4.58477 3.61503

0.03394 3.79601 4.51836 0.61958 0.77255 0.48576 0.95510

443 2.97165 4.41429 4.22289 3.83744 3.32476 3.98045 4.58108 1.17818 3.66390 1.92890 3.27450 4.09357 4.42084 4.02132 3.86934 3.47002 3.27299 1.84997 5.19827 3.92855 443 - -

2.68618 4.42225 2.77519 2.73123 3.46354 2.40513 3.72494 3.29354 2.67741 2.69355 4.24690 2.90347 2.73739 3.18146 2.89801 2.37887 2.77519 2.98518 4.58477 3.61503

0.03394 3.79601 4.51836 0.61958 0.77255 0.48576 0.95510

444 2.92251 4.76322 3.40570 2.93547 4.09844 3.42264 3.77482 3.63647 2.21535 3.16651 4.17059 3.30011 3.93066 3.00062 1.10219 3.01149 3.19482 3.36420 5.21367 4.04432 444 - -

2.68618 4.42225 2.77519 2.73123 3.46354 2.40513 3.72494 3.29354 2.67741 2.69355 4.24690 2.90347 2.73739 3.18146 2.89801 2.37887 2.77519 2.98518 4.58477 3.61503

0.03394 3.79601 4.51836 0.61958 0.77255 0.48576 0.95510

445 2.65642 4.42430 3.25570 3.08531 4.08348 3.11624 4.16757 3.61095 3.13637 3.24548 4.29346 3.39875 0.98293 3.52758 3.38897 2.82198 3.09244 3.26489 5.28934 4.20377 445 - -

2.68618 4.42225 2.77519 2.73123 3.46354 2.40513 3.72494 3.29354 2.67741 2.69355 4.24690 2.90347 2.73739 3.18146 2.89801 2.37887 2.77519 2.98518 4.58477 3.61503

0.03394 3.79601 4.51836 0.61958 0.77255 0.48576 0.95510

446 2.66315 4.65392 2.67213 2.55089 4.00571 3.13505 3.86476 3.70148 2.76309 3.35772 4.31495 1.30116 3.78410 3.13903 3.11371 2.73434 3.04351 3.33145 5.32076 3.96081 446 - -

2.68618 4.42225 2.77519 2.73123 3.46354 2.40513 3.72494 3.29354 2.67741 2.69355 4.24690 2.90347 2.73739 3.18146 2.89801 2.37887 2.77519 2.98518 4.58477 3.61503

0.03394 3.79601 4.51836 0.61958 0.77255 0.48576 0.95510

447 1.13001 4.15738 3.38757 3.17757 3.97857 2.98348 4.20830 3.09294 3.19189 2.97777 4.01983 3.34570 3.71568 3.54062 3.44647 2.50924 2.78140 2.76077 5.41351 4.20565 447 - -

2.68618 4.42225 2.77519 2.73123 3.46354 2.40513 3.72494 3.29354 2.67741 2.69355 4.24690 2.90347 2.73739 3.18146 2.89801 2.37887 2.77519 2.98518 4.58477 3.61503

0.03394 3.79601 4.51836 0.61958 0.77255 0.48576 0.95510

448 2.90487 4.86160 3.07558 2.71397 4.23048 3.40048 3.72190 3.65540 1.14833 3.23828 4.20575 3.14113 3.90783 2.91915 2.42439 2.95301 3.16487 3.36817 5.31817 4.12060 448 - -

2.68618 4.42225 2.77519 2.73123 3.46354 2.40513 3.72494 3.29354 2.67741 2.69355 4.24690 2.90347 2.73739 3.18146 2.89801 2.37887 2.77519 2.98518 4.58477 3.61503

0.03394 3.79601 4.51836 0.61958 0.77255 0.48576 0.95510

449 2.92251 4.76322 3.40570 2.93547 4.09844 3.42264 3.77482 3.63647 2.21535 3.16651 4.17059 3.30011 3.93066 3.00062 1.10219 3.01149 3.19482 3.36420 5.21367 4.04432 449 - -

2.68618 4.42225 2.77519 2.73123 3.46354 2.40513 3.72494 3.29354 2.67741 2.69355 4.24690 2.90347 2.73739 3.18146 2.89801 2.37887 2.77519 2.98518 4.58477 3.61503
0.03394 3.79601 4.51836 0.61958 0.77255 0.48576 0.95510
450 3.16785 4.58562 4.47664 3.94098 3.04219 4.29976 4.59228 2.16744 3.73329 1.02174 2.36676 4.24471 4.55763 3.98696 3.91986 3.65426 3.40406 2.25772 5.06226 3.90831 450 - -
2.68618 4.42225 2.77519 2.73123 3.46354 2.40513 3.72494 3.29354 2.67741 2.69355 4.24690 2.90347 2.73739 3.18146 2.89801 2.37887 2.77519 2.98518 4.58477 3.61503
0.03090 3.88816 4.61050 0.61958 0.77255 0.48576 0.95510
451 2.89402 4.45145 3.86737 3.37402 2.60517 3.82713 3.76102 2.69553 3.19729 2.20314 2.64926 3.62900 4.20264 3.50985 3.43863 3.14950 3.13678 2.59528 4.26262 1.65630 451 - -
2.68618 4.42225 2.77519 2.73123 3.46354 2.40513 3.72494 3.29354 2.67741 2.69355 4.24690 2.90347 2.73739 3.18146 2.89801 2.37887 2.77519 2.98518 4.58477 3.61503
0.03090 3.88816 4.61050 0.61958 0.77255 0.48576 0.95510
452 2.44785 4.32072 3.31044 2.93518 3.66997 3.21639 3.94072 3.04119 2.85386 2.29407 3.78110 3.25304 3.82214 3.24484 3.16870 1.58594 2.82467 2.76480 5.14758 3.81261 452 - -
2.68618 4.42225 2.77519 2.73123 3.46354 2.40513 3.72494 3.29354 2.67741 2.69355 4.24690 2.90347 2.73739 3.18146 2.89801 2.37887 2.77519 2.98518 4.58477 3.61503
0.12608 3.88816 2.32307 0.61958 0.77255 0.48576 0.95510
453 3.07120 4.55728 4.06560 3.70718 3.10996 3.88305 4.38695 2.31238 3.47558 0.97563 3.16058 3.98980 4.33298 3.86233 3.66334 3.44792 3.36975 2.32945 4.95943 3.66445 453 - -
2.68618 4.42225 2.77519 2.73123 3.46354 2.40513 3.72494 3.29354 2.67741 2.69355 4.24690 2.90347 2.73739 3.18146 2.89801 2.37887 2.77519 2.98518 4.58477 3.61503
0.03394 3.79601 4.51836 0.61958 0.77255 0.56425 0.84115
454 2.97165 4.41429 4.22289 3.83744 3.32476 3.98045 4.58108 1.17818 3.66390 1.92890 3.27450 4.09357 4.42084 4.02132 3.86934 3.47002 3.27299 1.84997 5.19827 3.92855 454 - -
2.68618 4.42225 2.77519 2.73123 3.46354 2.40513 3.72494 3.29354 2.67741 2.69355 4.24690 2.90347 2.73739 3.18146 2.89801 2.37887 2.77519 2.98518 4.58477 3.61503
0.03394 3.79601 4.51836 0.61958 0.77255 0.56425 0.84115
455 2.28079 4.19908 3.14317 2.95737 4.00099 2.93053 4.06839 3.51415 3.02585 3.24109 4.17505 3.19319 3.66344 3.38168 3.31291 1.21257 2.75555 3.06614 5.38010 4.08457 455 - -
2.68618 4.42225 2.77519 2.73123 3.46354 2.40513 3.72494 3.29354 2.67741 2.69355 4.24690 2.90347 2.73739 3.18146 2.89801 2.37887 2.77519 2.98518 4.58477 3.61503
0.03394 3.79601 4.51836 0.61958 0.77255 0.56425 0.84115
456 2.66315 4.65392 2.67213 2.55089 4.00571 3.13505 3.86476 3.70148 2.76309 3.35772 4.31495 1.30116 3.78410 3.13903 3.11371 2.73434 3.04351 3.33145 5.32076 3.96081 456 - -
2.68618 4.42225 2.77519 2.73123 3.46354 2.40513 3.72494 3.29354 2.67741 2.69355 4.24690 2.90347 2.73739 3.18146 2.89801 2.37887 2.77519 2.98518 4.58477 3.61503
0.03394 3.79601 4.51836 0.61958 0.77255 0.56425 0.84115
457 2.92251 4.76322 3.40570 2.93547 4.09844 3.42264 3.77482 3.63647 2.21535 3.16651 4.17059 3.30011 3.93066 3.00062 1.10219 3.01149 3.19482 3.36420 5.21367 4.04432 457 - -
2.68618 4.42225 2.77519 2.73123 3.46354 2.40513 3.72494 3.29354 2.67741 2.69355 4.24690 2.90347 2.73739 3.18146 2.89801 2.37887 2.77519 2.98518 4.58477 3.61503
0.03394 3.79601 4.51836 0.61958 0.77255 0.56425 0.84115
458 2.92251 4.76322 3.40570 2.93547 4.09844 3.42264 3.77482 3.63647 2.21535 3.16651 4.17059 3.30011 3.93066 3.00062 1.10219 3.01149 3.19482 3.36420 5.21367 4.04432 458 - -
2.68618 4.42225 2.77519 2.73123 3.46354 2.40513 3.72494 3.29354 2.67741 2.69355 4.24690 2.90347 2.73739 3.18146 2.89801 2.37887 2.77519 2.98518 4.58477 3.61503
0.03394 3.79601 4.51836 0.61958 0.77255 0.56425 0.84115
459 2.66315 4.65392 2.67213 2.55089 4.00571 3.13505 3.86476 3.70148 2.76309 3.35772 4.31495 1.30116 3.78410 3.13903 3.11371 2.73434 3.04351 3.33145 5.32076 3.96081 459 - -
2.68618 4.42225 2.77519 2.73123 3.46354 2.40513 3.72494 3.29354 2.67741 2.69355 4.24690 2.90347 2.73739 3.18146 2.89801 2.37887 2.77519 2.98518 4.58477 3.61503
0.03394 3.79601 4.51836 0.61958 0.77255 0.56425 0.84115
460 2.91157 4.47328 3.96816 3.55750 3.17841 3.77696 4.31044 2.34654 3.32910 1.78626 1.53296 3.84393 4.23864 3.71698 3.54307 3.25192 3.21616 2.34333 5.00802 3.76205 460 - -
2.68618 4.42225 2.77519 2.73123 3.46354 2.40513 3.72494 3.29354 2.67741 2.69355 4.24690 2.90347 2.73739 3.18146 2.89801 2.37887 2.77519 2.98518 4.58477 3.61503
0.03394 3.79601 4.51836 0.61958 0.77255 0.56425 0.84115

461 2.28079 4.19908 3.14317 2.95737 4.00099 2.93053 4.06839 3.51415 3.02585 3.24109 4.17505 3.19319 3.66344 3.38168 3.31291 1.21257 2.75555 3.06614 5.38010 4.08457 461 - -
2.68618 4.42225 2.77519 2.73123 3.46354 2.40513 3.72494 3.29354 2.67741 2.69355 4.24690 2.90347 2.73739 3.18146 2.89801 2.37887 2.77519 2.98518 4.58477 3.61503
0.03394 3.79601 4.51836 0.61958 0.77255 0.56425 0.84115
462 3.21457 4.61548 3.97805 3.66660 2.59647 3.64757 3.81244 3.23661 3.39107 2.64390 3.87379 3.83150 4.15944 3.78287 3.53232 3.41887 3.51155 3.12484 1.05487 2.60290 462 - -
2.68618 4.42225 2.77519 2.73123 3.46354 2.40513 3.72494 3.29354 2.67741 2.69355 4.24690 2.90347 2.73739 3.18146 2.89801 2.37887 2.77519 2.98518 4.58477 3.61503
0.03394 3.79601 4.51836 0.61958 0.77255 0.56425 0.84115
463 2.91056 5.02139 1.03432 2.29423 4.38899 3.15549 3.86877 3.96045 2.93927 3.58342 4.56179 2.84589 3.80315 3.10925 3.44342 2.88302 3.24824 3.60850 5.57234 4.27708 463 - -
2.68618 4.42225 2.77519 2.73123 3.46354 2.40513 3.72494 3.29354 2.67741 2.69355 4.24690 2.90347 2.73739 3.18146 2.89801 2.37887 2.77519 2.98518 4.58477 3.61503
0.03394 3.79601 4.51836 0.61958 0.77255 0.56425 0.84115
464 2.28079 4.19908 3.14317 2.95737 4.00099 2.93053 4.06839 3.51415 3.02585 3.24109 4.17505 3.19319 3.66344 3.38168 3.31291 1.21257 2.75555 3.06614 5.38010 4.08457 464 - -
2.68618 4.42225 2.77519 2.73123 3.46354 2.40513 3.72494 3.29354 2.67741 2.69355 4.24690 2.90347 2.73739 3.18146 2.89801 2.37887 2.77519 2.98518 4.58477 3.61503
0.03394 3.79601 4.51836 0.61958 0.77255 0.56425 0.84115
465 3.16380 4.58887 4.13307 3.83471 1.14586 3.86813 3.84824 2.69884 3.74404 2.08761 3.44167 3.93408 4.32984 3.93371 3.88264 3.43660 3.46563 2.68355 4.12324 2.50118 465 - -
2.68618 4.42225 2.77519 2.73123 3.46354 2.40513 3.72494 3.29354 2.67741 2.69355 4.24690 2.90347 2.73739 3.18146 2.89801 2.37887 2.77519 2.98518 4.58477 3.61503
0.03394 3.79601 4.51836 0.61958 0.77255 0.56425 0.84115
466 2.28079 4.19908 3.14317 2.95737 4.00099 2.93053 4.06839 3.51415 3.02585 3.24109 4.17505 3.19319 3.66344 3.38168 3.31291 1.21257 2.75555 3.06614 5.38010 4.08457 466 - -
2.68618 4.42225 2.77519 2.73123 3.46354 2.40513 3.72494 3.29354 2.67741 2.69355 4.24690 2.90347 2.73739 3.18146 2.89801 2.37887 2.77519 2.98518 4.58477 3.61503
0.03394 3.79601 4.51836 0.61958 0.77255 0.56425 0.84115
467 2.28079 4.19908 3.14317 2.95737 4.00099 2.93053 4.06839 3.51415 3.02585 3.24109 4.17505 3.19319 3.66344 3.38168 3.31291 1.21257 2.75555 3.06614 5.38010 4.08457 467 - -
2.68618 4.42225 2.77519 2.73123 3.46354 2.40513 3.72494 3.29354 2.67741 2.69355 4.24690 2.90347 2.73739 3.18146 2.89801 2.37887 2.77519 2.98518 4.58477 3.61503
0.03394 3.79601 4.51836 0.61958 0.77255 0.56425 0.84115
468 2.92251 4.76322 3.40570 2.93547 4.09844 3.42264 3.77482 3.63647 2.21535 3.16651 4.17059 3.30011 3.93066 3.00062 1.10219 3.01149 3.19482 3.36420 5.21367 4.04432 468 - -
2.68618 4.42225 2.77519 2.73123 3.46354 2.40513 3.72494 3.29354 2.67741 2.69355 4.24690 2.90347 2.73739 3.18146 2.89801 2.37887 2.77519 2.98518 4.58477 3.61503
0.03394 3.79601 4.51836 0.61958 0.77255 0.56425 0.84115
469 2.74814 4.31398 4.01764 3.68039 3.49634 3.64254 4.49596 2.06131 3.54689 2.19820 3.47523 3.88188 4.20873 3.91312 3.76637 3.14284 3.11247 1.12759 5.26714 3.99307 469 - -
2.68618 4.42225 2.77519 2.73123 3.46354 2.40513 3.72494 3.29354 2.67741 2.69355 4.24690 2.90347 2.73739 3.18146 2.89801 2.37887 2.77519 2.98518 4.58477 3.61503
0.03394 3.79601 4.51836 0.61958 0.77255 0.56425 0.84115
470 2.92251 4.76322 3.40570 2.93547 4.09844 3.42264 3.77482 3.63647 2.21535 3.16651 4.17059 3.30011 3.93066 3.00062 1.10219 3.01149 3.19482 3.36420 5.21367 4.04432 470 - -
2.68618 4.42225 2.77519 2.73123 3.46354 2.40513 3.72494 3.29354 2.67741 2.69355 4.24690 2.90347 2.73739 3.18146 2.89801 2.37887 2.77519 2.98518 4.58477 3.61503
0.03394 3.79601 4.51836 0.61958 0.77255 0.56425 0.84115
471 2.91157 4.47328 3.96816 3.55750 3.17841 3.77696 4.31044 2.34654 3.32910 1.78626 1.53296 3.84393 4.23864 3.71698 3.54307 3.25192 3.21616 2.34333 5.00802 3.76205 471 - -
2.68618 4.42225 2.77519 2.73123 3.46354 2.40513 3.72494 3.29354 2.67741 2.69355 4.24690 2.90347 2.73739 3.18146 2.89801 2.37887 2.77519 2.98518 4.58477 3.61503
0.03394 3.79601 4.51836 0.61958 0.77255 0.56425 0.84115
472 2.74814 4.31398 4.01764 3.68039 3.49634 3.64254 4.49596 2.06131 3.54689 2.19820 3.47523 3.88188 4.20873 3.91312 3.76637 3.14284 3.11247 1.12759 5.26714 3.99307 472 - -
2.68618 4.42225 2.77519 2.73123 3.46354 2.40513 3.72494 3.29354 2.67741 2.69355 4.24690 2.90347 2.73739 3.18146 2.89801 2.37887 2.77519 2.98518 4.58477 3.61503

0.03394 3.79601 4.51836 0.61958 0.77255 0.56425 0.84115
473 2.82402 4.80362 2.85663 2.61241 3.96136 3.31761 3.76927 3.58235 2.43912 3.09213 4.12436 3.07063 3.87138 1.37681 2.72390 2.87605 3.12210 3.31748 5.24814 3.93742 473 - -
2.68618 4.42225 2.77519 2.73123 3.46354 2.40513 3.72494 3.29354 2.67741 2.69355 4.24690 2.90347 2.73739 3.18146 2.89801 2.37887 2.77519 2.98518 4.58477 3.61503
0.03394 3.79601 4.51836 0.61958 0.77255 0.56425 0.84115
474 2.90487 4.86160 3.07558 2.71397 4.23048 3.40048 3.72190 3.65540 1.14833 3.23828 4.20575 3.14113 3.90783 2.91915 2.42439 2.95301 3.16487 3.36817 5.31817 4.12060 474 - -
2.68618 4.42225 2.77519 2.73123 3.46354 2.40513 3.72494 3.29354 2.67741 2.69355 4.24690 2.90347 2.73739 3.18146 2.89801 2.37887 2.77519 2.98518 4.58477 3.61503
0.03394 3.79601 4.51836 0.61958 0.77255 0.56425 0.84115
475 2.57535 4.35609 3.23321 3.12407 4.26351 0.85754 4.24721 3.84618 3.29467 3.50959 4.48627 3.39802 3.74476 3.62525 3.53733 2.74543 3.05073 3.38948 5.37705 4.36350 475 - -
2.68618 4.42225 2.77519 2.73123 3.46354 2.40513 3.72494 3.29354 2.67741 2.69355 4.24690 2.90347 2.73739 3.18146 2.89801 2.37887 2.77519 2.98518 4.58477 3.61503
0.03394 3.79601 4.51836 0.61958 0.77255 0.56425 0.84115
476 3.16380 4.58887 4.13307 3.83471 1.14586 3.86813 3.84824 2.69884 3.74404 2.08761 3.44167 3.93408 4.32984 3.93371 3.88264 3.43660 3.46563 2.68355 4.12324 2.50118 476 - -
2.68618 4.42225 2.77519 2.73123 3.46354 2.40513 3.72494 3.29354 2.67741 2.69355 4.24690 2.90347 2.73739 3.18146 2.89801 2.37887 2.77519 2.98518 4.58477 3.61503
0.03394 3.79601 4.51836 0.61958 0.77255 0.56425 0.84115
477 2.91157 4.47328 3.96816 3.55750 3.17841 3.77696 4.31044 2.34654 3.32910 1.78626 1.53296 3.84393 4.23864 3.71698 3.54307 3.25192 3.21616 2.34333 5.00802 3.76205 477 - -
2.68618 4.42225 2.77519 2.73123 3.46354 2.40513 3.72494 3.29354 2.67741 2.69355 4.24690 2.90347 2.73739 3.18146 2.89801 2.37887 2.77519 2.98518 4.58477 3.61503
0.03394 3.79601 4.51836 0.61958 0.77255 0.56425 0.84115
478 2.57535 4.35609 3.23321 3.12407 4.26351 0.85754 4.24721 3.84618 3.29467 3.50959 4.48627 3.39802 3.74476 3.62525 3.53733 2.74543 3.05073 3.38948 5.37705 4.36350 478 - -
2.68618 4.42225 2.77519 2.73123 3.46354 2.40513 3.72494 3.29354 2.67741 2.69355 4.24690 2.90347 2.73739 3.18146 2.89801 2.37887 2.77519 2.98518 4.58477 3.61503
0.03394 3.79601 4.51836 0.61958 0.77255 0.56425 0.84115
479 2.89015 4.98001 2.46169 1.13957 4.31725 3.22729 3.81015 3.75600 2.68126 3.38645 4.36600 2.89555 3.82889 3.02881 3.08050 2.88174 3.19454 3.44858 5.50348 4.22803 479 - -
2.68618 4.42225 2.77519 2.73123 3.46354 2.40513 3.72494 3.29354 2.67741 2.69355 4.24690 2.90347 2.73739 3.18146 2.89801 2.37887 2.77519 2.98518 4.58477 3.61503
0.03394 3.79601 4.51836 0.61958 0.77255 0.56425 0.84115
480 2.65642 4.42430 3.25570 3.08531 4.08348 3.11624 4.16757 3.61095 3.13637 3.24548 4.29346 3.39875 0.98293 3.52758 3.38897 2.82198 3.09244 3.26489 5.28934 4.20377 480 - -
2.68618 4.42225 2.77519 2.73123 3.46354 2.40513 3.72494 3.29354 2.67741 2.69355 4.24690 2.90347 2.73739 3.18146 2.89801 2.37887 2.77519 2.98518 4.58477 3.61503
0.03394 3.79601 4.51836 0.61958 0.77255 0.56425 0.84115
481 2.90487 4.86160 3.07558 2.71397 4.23048 3.40048 3.72190 3.65540 1.14833 3.23828 4.20575 3.14113 3.90783 2.91915 2.42439 2.95301 3.16487 3.36817 5.31817 4.12060 481 - -
2.68618 4.42225 2.77519 2.73123 3.46354 2.40513 3.72494 3.29354 2.67741 2.69355 4.24690 2.90347 2.73739 3.18146 2.89801 2.37887 2.77519 2.98518 4.58477 3.61503
0.03394 3.79601 4.51836 0.61958 0.77255 0.56425 0.84115
482 2.89015 4.98001 2.46169 1.13957 4.31725 3.22729 3.81015 3.75600 2.68126 3.38645 4.36600 2.89555 3.82889 3.02881 3.08050 2.88174 3.19454 3.44858 5.50348 4.22803 482 - -
2.68618 4.42225 2.77519 2.73123 3.46354 2.40513 3.72494 3.29354 2.67741 2.69355 4.24690 2.90347 2.73739 3.18146 2.89801 2.37887 2.77519 2.98518 4.58477 3.61503
0.03394 3.79601 4.51836 0.61958 0.77255 0.56425 0.84115
483 3.07120 4.55728 4.06560 3.70718 3.10996 3.88305 4.38695 2.31238 3.47558 0.97563 3.16058 3.98980 4.33298 3.86233 3.66334 3.44792 3.36975 2.32945 4.95943 3.66445 483 - -
2.68618 4.42225 2.77519 2.73123 3.46354 2.40513 3.72494 3.29354 2.67741 2.69355 4.24690 2.90347 2.73739 3.18146 2.89801 2.37887 2.77519 2.98518 4.58477 3.61503
0.03394 3.79601 4.51836 0.61958 0.77255 0.56425 0.84115
484 2.92251 4.76322 3.40570 2.93547 4.09844 3.42264 3.77482 3.63647 2.21535 3.16651 4.17059 3.30011 3.93066 3.00062 1.10219 3.01149 3.19482 3.36420 5.21367 4.04432 484 - -

2.68618 4.42225 2.77519 2.73123 3.46354 2.40513 3.72494 3.29354 2.67741 2.69355 4.24690 2.90347 2.73739 3.18146 2.89801 2.37887 2.77519 2.98518 4.58477 3.61503
0.03394 3.79601 4.51836 0.61958 0.77255 0.56425 0.84115
485 2.65642 4.42430 3.25570 3.08531 4.08348 3.11624 4.16757 3.61095 3.13637 3.24548 4.29346 3.39875 0.98293 3.52758 3.38897 2.82198 3.09244 3.26489 5.28934 4.20377 485 - -
2.68618 4.42225 2.77519 2.73123 3.46354 2.40513 3.72494 3.29354 2.67741 2.69355 4.24690 2.90347 2.73739 3.18146 2.89801 2.37887 2.77519 2.98518 4.58477 3.61503
0.03394 3.79601 4.51836 0.61958 0.77255 0.56425 0.84115
486 2.57535 4.35609 3.23321 3.12407 4.26351 0.85754 4.24721 3.84618 3.29467 3.50959 4.48627 3.39802 3.74476 3.62525 3.53733 2.74543 3.05073 3.38948 5.37705 4.36350 486 - -
2.68618 4.42225 2.77519 2.73123 3.46354 2.40513 3.72494 3.29354 2.67741 2.69355 4.24690 2.90347 2.73739 3.18146 2.89801 2.37887 2.77519 2.98518 4.58477 3.61503
0.03394 3.79601 4.51836 0.61958 0.77255 0.56425 0.84115
487 2.92251 4.76322 3.40570 2.93547 4.09844 3.42264 3.77482 3.63647 2.21535 3.16651 4.17059 3.30011 3.93066 3.00062 1.10219 3.01149 3.19482 3.36420 5.21367 4.04432 487 - -
2.68618 4.42225 2.77519 2.73123 3.46354 2.40513 3.72494 3.29354 2.67741 2.69355 4.24690 2.90347 2.73739 3.18146 2.89801 2.37887 2.77519 2.98518 4.58477 3.61503
0.03394 3.79601 4.51836 0.61958 0.77255 0.56425 0.84115
488 2.57535 4.35609 3.23321 3.12407 4.26351 0.85754 4.24721 3.84618 3.29467 3.50959 4.48627 3.39802 3.74476 3.62525 3.53733 2.74543 3.05073 3.38948 5.37705 4.36350 488 - -
2.68618 4.42225 2.77519 2.73123 3.46354 2.40513 3.72494 3.29354 2.67741 2.69355 4.24690 2.90347 2.73739 3.18146 2.89801 2.37887 2.77519 2.98518 4.58477 3.61503
0.03394 3.79601 4.51836 0.61958 0.77255 0.43963 1.03359
489 2.75536 4.82670 2.76806 1.60675 3.94751 3.39543 3.74104 3.23926 2.52941 2.41989 3.91387 2.98697 3.88605 2.93964 2.91457 2.80421 3.01410 3.00716 5.32249 3.97956 489 - -
2.68618 4.42225 2.77519 2.73123 3.46354 2.40513 3.72494 3.29354 2.67741 2.69355 4.24690 2.90347 2.73739 3.18146 2.89801 2.37887 2.77519 2.98518 4.58477 3.61503
0.03090 3.88816 4.61050 0.61958 0.77255 0.48576 0.95510
490 3.20741 4.59688 4.43102 3.98203 1.47550 4.20520 4.01887 2.43169 3.83500 1.53165 3.13796 4.10633 4.51796 3.98904 3.98023 3.58092 3.45230 2.47636 4.28802 2.71163 490 - -
2.68618 4.42225 2.77519 2.73123 3.46354 2.40513 3.72494 3.29354 2.67741 2.69355 4.24690 2.90347 2.73739 3.18146 2.89801 2.37887 2.77519 2.98518 4.58477 3.61503
0.03090 3.88816 4.61050 0.61958 0.77255 0.48576 0.95510
491 3.01159 4.82654 3.14060 2.90196 3.29538 3.46046 1.10984 3.71695 2.74457 3.20901 4.25305 3.31072 4.01377 3.26835 3.00942 3.08208 3.31541 3.45176 4.76025 3.24576 491 - -
2.68618 4.42225 2.77519 2.73123 3.46354 2.40513 3.72494 3.29354 2.67741 2.69355 4.24690 2.90347 2.73739 3.18146 2.89801 2.37887 2.77519 2.98518 4.58477 3.61503
0.03090 3.88816 4.61050 0.61958 0.77255 0.48576 0.95510
492 2.80479 5.10423 2.47189 1.50157 4.25174 3.30106 2.79411 3.85084 2.45004 3.38943 4.24933 2.81299 3.82826 2.83659 2.86645 2.76685 3.06056 3.50017 5.48147 4.07319 492 - -
2.68618 4.42225 2.77519 2.73123 3.46354 2.40513 3.72494 3.29354 2.67741 2.69355 4.24690 2.90347 2.73739 3.18146 2.89801 2.37887 2.77519 2.98518 4.58477 3.61503
0.03090 3.88816 4.61050 0.61958 0.77255 0.48576 0.95510
493 2.75458 4.92970 2.61364 2.38593 3.88546 3.30534 2.77832 3.75472 2.48252 3.30591 4.18393 1.72048 3.84083 2.89740 2.86639 2.75736 3.03374 3.40774 5.23499 3.76834 493 - -
2.68618 4.42225 2.77519 2.73123 3.46354 2.40513 3.72494 3.29354 2.67741 2.69355 4.24690 2.90347 2.73739 3.18146 2.89801 2.37887 2.77519 2.98518 4.58477 3.61503
0.03090 3.88816 4.61050 0.61958 0.77255 0.48576 0.95510
494 2.31701 4.28223 3.08435 2.84347 4.15295 2.99497 4.01555 3.60692 2.92579 3.28530 4.15506 3.14289 1.67148 3.27092 3.25999 2.01476 2.76359 3.14207 5.48916 4.21677 494 - -
2.68618 4.42225 2.77519 2.73123 3.46354 2.40513 3.72494 3.29354 2.67741 2.69355 4.24690 2.90347 2.73739 3.18146 2.89801 2.37887 2.77519 2.98518 4.58477 3.61503
0.03090 3.88816 4.61050 0.61958 0.77255 0.48576 0.95510
495 2.32245 4.24316 3.21977 3.03607 4.09179 2.97273 4.14559 3.61643 3.11090 3.33538 4.26003 3.25848 3.71279 3.45895 3.39658 1.06766 2.80372 3.14811 5.46298 4.17535 495 - -
2.68618 4.42225 2.77519 2.73123 3.46354 2.40513 3.72494 3.29354 2.67741 2.69355 4.24690 2.90347 2.73739 3.18146 2.89801 2.37887 2.77519 2.98518 4.58477 3.61503
0.03090 3.88816 4.61050 0.61958 0.77255 0.48576 0.95510

496 2.57187 4.37381 3.33781 2.90718 3.34319 3.39877 3.82542 2.90439 2.81450 2.62849 3.61638 3.27077 3.90536 3.19355 3.13487 2.75707 1.77909 2.67507 4.85783 2.73418 496 - -

2.68618 4.42225 2.77519 2.73123 3.46354 2.40513 3.72494 3.29354 2.67741 2.69355 4.24690 2.90347 2.73739 3.18146 2.89801 2.37887 2.77519 2.98518 4.58477 3.61503

0.03090 3.88816 4.61050 0.61958 0.77255 0.48576 0.95510

497 2.60368 0.90917 4.12658 3.86965 3.85721 3.22349 4.51327 3.00355 3.69662 2.91841 4.04014 3.85245 3.93170 4.04206 3.81828 2.87538 3.07174 2.74712 5.27022 4.14483 497 - -

2.68618 4.42225 2.77519 2.73123 3.46354 2.40513 3.72494 3.29354 2.67741 2.69355 4.24690 2.90347 2.73739 3.18146 2.89801 2.37887 2.77519 2.98518 4.58477 3.61503

0.03090 3.88816 4.61050 0.61958 0.77255 0.48576 0.95510

498 3.06115 4.43541 4.52175 4.01474 2.44936 4.24047 4.47757 1.35397 3.89315 1.73163 3.08137 4.23167 4.53991 4.08704 4.06589 3.59891 3.30870 1.97283 4.88799 3.53546 498 - -

2.68618 4.42225 2.77519 2.73123 3.46354 2.40513 3.72494 3.29354 2.67741 2.69355 4.24690 2.90347 2.73739 3.18146 2.89801 2.37887 2.77519 2.98518 4.58477 3.61503

0.03090 3.88816 4.61050 0.61958 0.77255 0.48576 0.95510

499 2.18740 1.66952 3.83894 3.48665 3.83051 2.96232 4.25525 3.10068 3.35124 2.95329 3.87777 3.46241 3.70096 3.65828 3.55716 1.97129 2.66519 2.72532 5.29259 4.07131 499 - -

2.68618 4.42225 2.77519 2.73123 3.46354 2.40513 3.72494 3.29354 2.67741 2.69355 4.24690 2.90347 2.73739 3.18146 2.89801 2.37887 2.77519 2.98518 4.58477 3.61503

0.03090 3.88816 4.61050 0.61958 0.77255 0.48576 0.95510

500 2.83049 5.17434 2.40325 1.47166 4.45406 3.28126 3.67284 3.92187 2.44881 3.45180 4.31944 2.78415 3.82674 2.35358 2.86545 2.77825 3.09078 3.56440 5.62010 4.23525 500 - -

2.68618 4.42225 2.77519 2.73123 3.46354 2.40513 3.72494 3.29354 2.67741 2.69355 4.24690 2.90347 2.73739 3.18146 2.89801 2.37887 2.77519 2.98518 4.58477 3.61503

0.03090 3.88816 4.61050 0.61958 0.77255 0.48576 0.95510

501 2.73075 4.65906 3.21053 2.71937 2.78583 3.50681 3.69480 3.14038 1.63424 2.75378 3.74607 3.15903 3.93196 2.96254 2.75235 2.83313 2.97299 2.92068 4.95942 3.55833 501 - -

2.68618 4.42225 2.77519 2.73123 3.46354 2.40513 3.72494 3.29354 2.67741 2.69355 4.24690 2.90347 2.73739 3.18146 2.89801 2.37887 2.77519 2.98518 4.58477 3.61503

0.03090 3.88816 4.61050 0.61958 0.77255 0.48576 0.95510

502 2.56930 4.32855 3.56851 3.14004 3.57695 3.41508 4.07604 2.58460 2.98844 2.06904 3.55191 3.44600 3.96921 3.39064 3.27783 2.80979 1.70709 2.40357 5.16603 3.89734 502 - -

2.68618 4.42225 2.77519 2.73123 3.46354 2.40513 3.72494 3.29354 2.67741 2.69355 4.24690 2.90347 2.73739 3.18146 2.89801 2.37887 2.77519 2.98518 4.58477 3.61503

0.03090 3.88816 4.61050 0.61958 0.77255 0.48576 0.95510

503 2.43393 4.18220 3.65724 3.28108 3.66453 3.27625 4.17852 2.53810 3.20073 2.62201 3.66025 3.48659 3.90455 3.54103 3.46987 2.23107 2.82831 1.44873 5.21829 3.95590 503 - -

2.68618 4.42225 2.77519 2.73123 3.46354 2.40513 3.72494 3.29354 2.67741 2.69355 4.24690 2.90347 2.73739 3.18146 2.89801 2.37887 2.77519 2.98518 4.58477 3.61503

0.03090 3.88816 4.61050 0.61958 0.77255 0.48576 0.95510

504 2.30152 4.18231 3.42631 3.07101 3.86815 3.06688 4.07280 3.07845 3.03045 2.93266 3.88363 3.29353 3.74566 3.37350 3.33575 1.47956 2.73448 2.23059 5.32099 4.04658 504 - -

2.68618 4.42225 2.77519 2.73123 3.46354 2.40513 3.72494 3.29354 2.67741 2.69355 4.24690 2.90347 2.73739 3.18146 2.89801 2.37887 2.77519 2.98518 4.58477 3.61503

0.03090 3.88816 4.61050 0.61958 0.77255 0.48576 0.95510

505 2.70540 4.81372 2.94058 2.56013 4.27931 3.34157 3.67807 3.68869 1.48452 3.27072 4.13418 3.01213 3.84903 2.84581 2.52390 2.30703 2.98512 3.33623 5.41692 4.13696 505 - -

2.68618 4.42225 2.77519 2.73123 3.46354 2.40513 3.72494 3.29354 2.67741 2.69355 4.24690 2.90347 2.73739 3.18146 2.89801 2.37887 2.77519 2.98518 4.58477 3.61503

0.03090 3.88816 4.61050 0.61958 0.77255 0.48576 0.95510

506 2.26376 4.18790 3.27086 3.00079 4.13445 2.29084 4.09716 3.45045 3.03928 3.21255 4.09178 3.22867 3.68785 3.37249 3.34885 2.43894 1.45275 3.00857 5.48790 4.26712 506 - -

2.68618 4.42225 2.77519 2.73123 3.46354 2.40513 3.72494 3.29354 2.67741 2.69355 4.24690 2.90347 2.73739 3.18146 2.89801 2.37887 2.77519 2.98518 4.58477 3.61503

0.03090 3.88816 4.61050 0.61958 0.77255 0.48576 0.95510

507 2.76933 4.32053 3.88733 3.40653 3.02583 3.76337 4.02291 2.32714 3.26872 2.20509 3.35039 3.68067 4.18072 3.58638 3.51759 3.10762 3.03797 1.48258 4.68083 2.61789 507 - -

2.68618 4.42225 2.77519 2.73123 3.46354 2.40513 3.72494 3.29354 2.67741 2.69355 4.24690 2.90347 2.73739 3.18146 2.89801 2.37887 2.77519 2.98518 4.58477 3.61503

0.03090 3.88816 4.61050 0.61958 0.77255 0.48576 0.95510
508 1.31290 4.11838 3.47283 3.16988 4.06566 2.97403 4.18602 3.15138 3.15332 3.05694 3.99682 3.31986 3.70184 3.47971 3.43811 2.42958 2.16124 2.77502 5.49469 4.28313 508 - -
2.68618 4.42225 2.77519 2.73123 3.46354 2.40513 3.72494 3.29354 2.67741 2.69355 4.24690 2.90347 2.73739 3.18146 2.89801 2.37887 2.77519 2.98518 4.58477 3.61503
0.03090 3.88816 4.61050 0.61958 0.77255 0.48576 0.95510
509 2.61233 3.06604 3.32082 2.79268 3.91554 3.37942 3.76472 3.20160 1.55548 2.92775 3.84007 3.18587 3.86848 2.98022 2.68954 2.74039 2.90225 2.92081 5.21028 3.96514 509 - -
2.68618 4.42225 2.77519 2.73123 3.46354 2.40513 3.72494 3.29354 2.67741 2.69355 4.24690 2.90347 2.73739 3.18146 2.89801 2.37887 2.77519 2.98518 4.58477 3.61503
0.03090 3.88816 4.61050 0.61958 0.77255 0.48576 0.95510
510 2.32245 4.24316 3.21977 3.03607 4.09179 2.97273 4.14559 3.61643 3.11090 3.33538 4.26003 3.25848 3.71279 3.45895 3.39658 1.06766 2.80372 3.14811 5.46298 4.17535 510 - -
2.68618 4.42225 2.77519 2.73123 3.46354 2.40513 3.72494 3.29354 2.67741 2.69355 4.24690 2.90347 2.73739 3.18146 2.89801 2.37887 2.77519 2.98518 4.58477 3.61503
0.03090 3.88816 4.61050 0.61958 0.77255 0.48576 0.95510
511 2.50832 4.59586 2.66381 2.48984 4.22288 3.09694 3.84057 3.76734 2.72128 3.39658 4.25251 1.63716 3.74475 3.05464 3.12079 2.14012 2.89451 3.32670 5.52500 4.15717 511 - -
2.68618 4.42225 2.77519 2.73123 3.46354 2.40513 3.72494 3.29354 2.67741 2.69355 4.24690 2.90347 2.73739 3.18146 2.89801 2.37887 2.77519 2.98518 4.58477 3.61503
0.03090 3.88816 4.61050 0.61958 0.77255 0.48576 0.95510
512 2.56589 4.72130 2.12064 2.36304 4.30080 3.12367 3.80080 3.77614 2.74554 3.40316 4.25280 2.85952 3.74846 2.99578 3.21988 1.57176 2.92416 3.35686 5.58596 4.21023 512 - -
2.68618 4.42225 2.77519 2.73123 3.46354 2.40513 3.72494 3.29354 2.67741 2.69355 4.24690 2.90347 2.73739 3.18146 2.89801 2.37887 2.77519 2.98518 4.58477 3.61503
0.03090 3.88816 4.61050 0.61958 0.77255 0.48576 0.95510
513 2.74934 4.64061 3.19169 2.75966 2.61602 3.52667 3.71623 3.08144 2.64611 2.57469 3.65363 3.19630 3.96466 1.83973 2.98802 2.86132 3.00354 2.90839 4.77223 3.28922 513 - -
2.68618 4.42225 2.77519 2.73123 3.46354 2.40513 3.72494 3.29354 2.67741 2.69355 4.24690 2.90347 2.73739 3.18146 2.89801 2.37887 2.77519 2.98518 4.58477 3.61503
0.03090 3.88816 4.61050 0.61958 0.77255 0.48576 0.95510
514 2.43698 4.27286 3.40007 2.99278 3.67246 3.24536 3.98050 2.35804 2.92341 2.71057 3.70366 3.29896 3.83961 3.28674 3.24201 1.62932 2.80697 2.59584 5.16190 3.86633 514 - -
2.68618 4.42225 2.77519 2.73123 3.46354 2.40513 3.72494 3.29354 2.67741 2.69355 4.24690 2.90347 2.73739 3.18146 2.89801 2.37887 2.77519 2.98518 4.58477 3.61503
0.03090 3.88816 4.61050 0.61958 0.77255 0.48576 0.95510
515 2.96544 5.08017 2.49372 1.00328 4.42830 3.28393 3.87185 3.88530 2.75433 3.49981 4.47296 2.94132 3.88920 3.08706 3.16494 2.94613 3.26973 3.56745 5.59690 4.31893 515 - -
2.68618 4.42225 2.77519 2.73123 3.46354 2.40513 3.72494 3.29354 2.67741 2.69355 4.24690 2.90347 2.73739 3.18146 2.89801 2.37887 2.77519 2.98518 4.58477 3.61503
0.03090 3.88816 4.61050 0.61958 0.77255 0.48576 0.95510
516 2.72187 4.64332 3.17562 2.72346 3.66963 3.50404 3.77159 2.50205 2.49779 2.59009 3.66157 3.17159 3.94408 1.86746 2.81676 2.84108 2.97515 2.79947 5.13389 3.81752 516 - -
2.68618 4.42225 2.77519 2.73123 3.46354 2.40513 3.72494 3.29354 2.67741 2.69355 4.24690 2.90347 2.73739 3.18146 2.89801 2.37887 2.77519 2.98518 4.58477 3.61503
0.03090 3.88816 4.61050 0.61958 0.77255 0.48576 0.95510
517 2.63856 4.46518 2.51575 2.78342 3.74245 3.42393 3.96273 2.61796 2.92799 2.68160 3.70973 3.22263 3.94875 3.23180 3.31826 2.82625 2.94549 1.56765 5.25139 3.94747 517 - -
2.68618 4.42225 2.77519 2.73123 3.46354 2.40513 3.72494 3.29354 2.67741 2.69355 4.24690 2.90347 2.73739 3.18146 2.89801 2.37887 2.77519 2.98518 4.58477 3.61503
0.03090 3.88816 4.61050 0.61958 0.77255 0.48576 0.95510
518 2.98538 4.49344 4.01185 3.52960 2.70185 3.95111 3.93786 2.51975 3.33091 1.29803 3.21797 3.78552 4.30311 3.64325 3.54977 3.29493 3.22513 2.49337 4.42195 2.37359 518 - -
2.68618 4.42225 2.77519 2.73123 3.46354 2.40513 3.72494 3.29354 2.67741 2.69355 4.24690 2.90347 2.73739 3.18146 2.89801 2.37887 2.77519 2.98518 4.58477 3.61503
0.03090 3.88816 4.61050 0.61958 0.77255 0.48576 0.95510
519 1.82814 4.13624 3.29120 3.03875 4.18782 2.90682 4.13643 3.53721 3.09999 3.29021 4.15551 3.22395 3.65018 3.41247 3.40685 1.34734 2.67883 3.04443 5.55377 4.31512 519 - -

2.68618 4.42225 2.77519 2.73123 3.46354 2.40513 3.72494 3.29354 2.67741 2.69355 4.24690 2.90347 2.73739 3.18146 2.89801 2.37887 2.77519 2.98518 4.58477 3.61503
0.03090 3.88816 4.61050 0.61958 0.77255 0.48576 0.95510
520 2.67635 4.57269 3.08439 2.73974 2.54398 3.41149 3.72120 3.16715 2.78878 2.81297 3.80518 1.79769 3.91785 3.12627 3.15888 2.79068 2.97242 2.92292 4.71221 3.17915 520 - -
2.68618 4.42225 2.77519 2.73123 3.46354 2.40513 3.72494 3.29354 2.67741 2.69355 4.24690 2.90347 2.73739 3.18146 2.89801 2.37887 2.77519 2.98518 4.58477 3.61503
0.03090 3.88816 4.61050 0.61958 0.77255 0.48576 0.95510
521 2.82812 5.23027 1.45199 2.16172 4.53675 3.22281 3.70394 4.02003 2.18250 3.57029 4.42513 2.72799 3.80399 2.87076 3.10793 2.76009 3.10803 3.63366 5.72149 4.29512 521 - -
2.68618 4.42225 2.77519 2.73123 3.46354 2.40513 3.72494 3.29354 2.67741 2.69355 4.24690 2.90347 2.73739 3.18146 2.89801 2.37887 2.77519 2.98518 4.58477 3.61503
0.03090 3.88816 4.61050 0.61958 0.77255 0.48576 0.95510
522 2.56930 4.32855 3.56851 3.14004 3.57695 3.41508 4.07604 2.58460 2.98844 2.06904 3.55191 3.44600 3.96921 3.39064 3.27783 2.80979 1.70709 2.40357 5.16603 3.89734 522 - -
2.68618 4.42225 2.77519 2.73123 3.46354 2.40513 3.72494 3.29354 2.67741 2.69355 4.24690 2.90347 2.73739 3.18146 2.89801 2.37887 2.77519 2.98518 4.58477 3.61503
0.03090 3.88816 4.61050 0.61958 0.77255 0.48576 0.95510
523 2.75536 4.82670 2.76806 1.60675 3.94751 3.39543 3.74104 3.23926 2.52941 2.41989 3.91387 2.98697 3.88605 2.93964 2.91457 2.80421 3.01410 3.00716 5.32249 3.97956 523 - -
2.68618 4.42225 2.77519 2.73123 3.46354 2.40513 3.72494 3.29354 2.67741 2.69355 4.24690 2.90347 2.73739 3.18146 2.89801 2.37887 2.77519 2.98518 4.58477 3.61503
0.03090 3.88816 4.61050 0.61958 0.77255 0.48576 0.95510
524 3.02283 4.86123 3.51505 3.02261 4.21977 3.50806 3.83514 3.76241 2.24300 3.27548 4.27508 3.38124 4.00973 3.05453 0.95470 3.10614 3.28762 3.48564 5.29636 4.14355 524 - -
2.68618 4.42225 2.77519 2.73123 3.46354 2.40513 3.72494 3.29354 2.67741 2.69355 4.24690 2.90347 2.73739 3.18146 2.89801 2.37887 2.77519 2.98518 4.58477 3.61503
0.03090 3.88816 4.61050 0.61958 0.77255 0.48576 0.95510
525 2.46386 4.49344 2.74178 2.59786 4.29705 1.52126 3.94483 3.82125 2.87233 3.45626 4.31321 2.30191 3.73093 3.17846 3.25797 2.56416 2.88611 3.34270 5.57651 4.27148 525 - -
2.68618 4.42225 2.77519 2.73123 3.46354 2.40513 3.72494 3.29354 2.67741 2.69355 4.24690 2.90347 2.73739 3.18146 2.89801 2.37887 2.77519 2.98518 4.58477 3.61503
0.03090 3.88816 4.61050 0.61958 0.77255 0.48576 0.95510
526 3.10980 4.47008 4.57693 4.07375 3.25019 4.34226 4.74082 1.27672 3.89353 1.54474 3.11430 4.34087 4.63205 4.17219 4.08682 3.72137 3.36289 1.82103 5.23196 4.01581 526 - -
2.68618 4.42225 2.77519 2.73123 3.46354 2.40513 3.72494 3.29354 2.67741 2.69355 4.24690 2.90347 2.73739 3.18146 2.89801 2.37887 2.77519 2.98518 4.58477 3.61503
0.03090 3.88816 4.61050 0.61958 0.77255 0.48576 0.95510
527 1.43097 4.16544 3.68648 3.31286 3.80505 3.18571 4.24627 2.61553 3.24570 2.66842 3.73556 3.47991 3.85608 3.57219 3.52250 2.61918 2.79788 1.98981 5.36752 4.13871 527 - -
2.68618 4.42225 2.77519 2.73123 3.46354 2.40513 3.72494 3.29354 2.67741 2.69355 4.24690 2.90347 2.73739 3.18146 2.89801 2.37887 2.77519 2.98518 4.58477 3.61503
0.03090 3.88816 4.61050 0.61958 0.77255 0.48576 0.95510
528 2.87055 4.35125 4.06077 3.56001 2.93334 3.92227 4.07469 1.51483 3.39828 2.03078 3.24153 3.81628 4.28373 3.69485 3.61966 3.25606 3.11671 2.15347 4.63542 2.58929 528 - -
2.68618 4.42225 2.77519 2.73123 3.46354 2.40513 3.72494 3.29354 2.67741 2.69355 4.24690 2.90347 2.73739 3.18146 2.89801 2.37887 2.77519 2.98518 4.58477 3.61503
0.03090 3.88816 4.61050 0.61958 0.77255 0.48576 0.95510
529 2.84438 4.96896 3.06205 2.59429 4.26941 3.47578 3.61816 3.73156 2.07823 3.23362 4.12081 3.04888 3.91074 1.71515 2.02995 2.85928 3.06485 3.41870 5.35701 4.09122 529 - -
2.68618 4.42225 2.77519 2.73123 3.46354 2.40513 3.72494 3.29354 2.67741 2.69355 4.24690 2.90347 2.73739 3.18146 2.89801 2.37887 2.77519 2.98518 4.58477 3.61503
0.03090 3.88816 4.61050 0.61958 0.77255 0.48576 0.95510
530 2.59661 4.62926 2.78882 2.15171 4.07648 3.25265 3.79057 3.33763 2.61108 3.07883 3.98484 2.99116 3.81296 2.99585 2.99270 2.68042 1.73964 3.02908 5.39582 4.09498 530 - -
2.68618 4.42225 2.77519 2.73123 3.46354 2.40513 3.72494 3.29354 2.67741 2.69355 4.24690 2.90347 2.73739 3.18146 2.89801 2.37887 2.77519 2.98518 4.58477 3.61503
0.03090 3.88816 4.61050 0.61958 0.77255 0.48576 0.95510

531 2.77411 5.01711 2.39941 1.37450 4.46188 2.46740 3.76338 3.91821 2.65617 3.50323 4.36918 2.81393 3.80704 2.94607 3.11796 2.75457 3.08179 3.53353 5.65273 4.29570 531 - -
2.68618 4.42225 2.77519 2.73123 3.46354 2.40513 3.72494 3.29354 2.67741 2.69355 4.24690 2.90347 2.73739 3.18146 2.89801 2.37887 2.77519 2.98518 4.58477 3.61503
0.03090 3.88816 4.61050 0.61958 0.77255 0.48576 0.95510
532 2.64297 4.71406 2.89023 2.55008 4.09999 3.30404 3.72906 3.48697 2.41077 3.09634 4.01517 3.00948 3.83179 1.76126 2.74177 2.70735 2.43381 3.17409 5.37163 4.06517 532 - -
2.68618 4.42225 2.77519 2.73123 3.46354 2.40513 3.72494 3.29354 2.67741 2.69355 4.24690 2.90347 2.73739 3.18146 2.89801 2.37887 2.77519 2.98518 4.58477 3.61503
0.03090 3.88816 4.61050 0.61958 0.77255 0.48576 0.95510
533 2.82040 4.35847 4.14715 3.81059 3.56135 3.74026 4.60954 2.04898 3.67862 2.24554 3.52394 3.99795 4.29976 4.03721 3.88964 3.24464 3.18148 1.00534 5.34850 4.08117 533 - -
2.68618 4.42225 2.77519 2.73123 3.46354 2.40513 3.72494 3.29354 2.67741 2.69355 4.24690 2.90347 2.73739 3.18146 2.89801 2.37887 2.77519 2.98518 4.58477 3.61503
0.03090 3.88816 4.61050 0.61958 0.77255 0.48576 0.95510
534 2.22564 4.16716 3.27743 2.99566 4.14610 2.94261 4.08815 3.49235 3.01900 3.23948 4.10887 3.21072 3.66638 3.35549 3.33134 1.38477 2.16260 3.02604 5.50935 4.26405 534 - -
2.68618 4.42225 2.77519 2.73123 3.46354 2.40513 3.72494 3.29354 2.67741 2.69355 4.24690 2.90347 2.73739 3.18146 2.89801 2.37887 2.77519 2.98518 4.58477 3.61503
0.03090 3.88816 4.61050 0.61958 0.77255 0.48576 0.95510
535 2.28233 4.25465 3.11828 2.85258 4.17414 2.97471 4.01584 3.62582 2.93269 3.29645 4.14880 3.14099 2.62670 3.26426 3.27566 1.40121 2.73196 3.14180 5.50467 4.23815 535 - -
2.68618 4.42225 2.77519 2.73123 3.46354 2.40513 3.72494 3.29354 2.67741 2.69355 4.24690 2.90347 2.73739 3.18146 2.89801 2.37887 2.77519 2.98518 4.58477 3.61503
0.03090 3.88816 4.61050 0.61958 0.77255 0.48576 0.95510
536 2.55289 4.64163 2.62053 2.47508 4.31892 2.38131 3.86725 3.85241 2.77204 3.46699 4.32403 1.54782 3.75469 3.08103 3.18361 2.61825 2.94058 3.40121 5.58071 4.24294 536 - -
2.68618 4.42225 2.77519 2.73123 3.46354 2.40513 3.72494 3.29354 2.67741 2.69355 4.24690 2.90347 2.73739 3.18146 2.89801 2.37887 2.77519 2.98518 4.58477 3.61503
0.03090 3.88816 4.61050 0.61958 0.77255 0.48576 0.95510
537 2.84927 5.28353 1.35348 2.10582 4.60141 3.16370 3.74489 4.16011 2.76519 3.70361 4.56951 2.19979 3.79469 2.92618 3.33500 2.76738 3.15845 3.74222 5.82920 4.34670 537 - -
2.68618 4.42225 2.77519 2.73123 3.46354 2.40513 3.72494 3.29354 2.67741 2.69355 4.24690 2.90347 2.73739 3.18146 2.89801 2.37887 2.77519 2.98518 4.58477 3.61503
0.03090 3.88816 4.61050 0.61958 0.77255 0.48576 0.95510
538 2.55550 4.63406 2.23989 2.45979 4.15180 3.18063 3.81204 3.46146 2.72668 3.19418 4.07982 2.93910 3.77493 3.01475 3.16942 2.62740 1.69878 3.11519 5.47368 4.14127 538 - -
2.68618 4.42225 2.77519 2.73123 3.46354 2.40513 3.72494 3.29354 2.67741 2.69355 4.24690 2.90347 2.73739 3.18146 2.89801 2.37887 2.77519 2.98518 4.58477 3.61503
0.03090 3.88816 4.61050 0.61958 0.77255 0.48576 0.95510
539 2.96544 5.08017 2.49372 1.00328 4.42830 3.28393 3.87185 3.88530 2.75433 3.49981 4.47296 2.94132 3.88920 3.08706 3.16494 2.94613 3.26973 3.56745 5.59690 4.31893 539 - -
2.68618 4.42225 2.77519 2.73123 3.46354 2.40513 3.72494 3.29354 2.67741 2.69355 4.24690 2.90347 2.73739 3.18146 2.89801 2.37887 2.77519 2.98518 4.58477 3.61503
0.03090 3.88816 4.61050 0.61958 0.77255 0.48576 0.95510
540 2.66311 4.32380 3.77042 3.29498 3.38916 3.58898 4.14337 2.43681 3.12312 2.09937 1.87261 3.59293 4.07555 3.49353 3.38828 2.95677 2.45460 2.32254 5.07139 3.85763 540 - -
2.68618 4.42225 2.77519 2.73123 3.46354 2.40513 3.72494 3.29354 2.67741 2.69355 4.24690 2.90347 2.73739 3.18146 2.89801 2.37887 2.77519 2.98518 4.58477 3.61503
0.03090 3.88816 4.61050 0.61958 0.77255 0.48576 0.95510
541 1.81465 4.16771 3.21521 3.02437 4.27158 1.41991 4.17476 3.62784 3.16430 3.37381 4.24267 3.22617 3.66112 3.45730 3.47010 2.40511 2.72182 3.11664 5.60488 4.39613 541 - -
2.68618 4.42225 2.77519 2.73123 3.46354 2.40513 3.72494 3.29354 2.67741 2.69355 4.24690 2.90347 2.73739 3.18146 2.89801 2.37887 2.77519 2.98518 4.58477 3.61503
0.12608 3.88816 2.32307 0.61958 0.77255 0.48576 0.95510
542 2.97165 4.41429 4.22289 3.83744 3.32476 3.98045 4.58108 1.17818 3.66390 1.92890 3.27450 4.09357 4.42084 4.02132 3.86934 3.47002 3.27299 1.84997 5.19827 3.92855 542 - -
2.68618 4.42225 2.77519 2.73123 3.46354 2.40513 3.72494 3.29354 2.67741 2.69355 4.24690 2.90347 2.73739 3.18146 2.89801 2.37887 2.77519 2.98518 4.58477 3.61503

0.03394 3.79601 4.51836 0.61958 0.77255 0.56425 0.84115

543 1.13001 4.15738 3.38757 3.17757 3.97857 2.98348 4.20830 3.09294 3.19189 2.97777 4.01983 3.34570 3.71568 3.54062 3.44647 2.50924 2.78140 2.76077 5.41351 4.20565 543 - -

2.68618 4.42225 2.77519 2.73123 3.46354 2.40513 3.72494 3.29354 2.67741 2.69355 4.24690 2.90347 2.73739 3.18146 2.89801 2.37887 2.77519 2.98518 4.58477 3.61503

0.03394 3.79601 4.51836 0.61958 0.77255 0.56425 0.84115

544 2.37514 4.21936 3.36356 3.11439 3.90452 3.06054 4.13711 3.02248 3.05582 2.88857 3.94573 3.33472 3.75900 3.45502 3.31699 2.57577 1.30864 2.72567 5.34922 4.12174 544 - -

2.68618 4.42225 2.77519 2.73123 3.46354 2.40513 3.72494 3.29354 2.67741 2.69355 4.24690 2.90347 2.73739 3.18146 2.89801 2.37887 2.77519 2.98518 4.58477 3.61503

0.03394 3.79601 4.51836 0.61958 0.77255 0.56425 0.84115

545 2.28079 4.19908 3.14317 2.95737 4.00099 2.93053 4.06839 3.51415 3.02585 3.24109 4.17505 3.19319 3.66344 3.38168 3.31291 1.21257 2.75555 3.06614 5.38010 4.08457 545 - -

2.68618 4.42225 2.77519 2.73123 3.46354 2.40513 3.72494 3.29354 2.67741 2.69355 4.24690 2.90347 2.73739 3.18146 2.89801 2.37887 2.77519 2.98518 4.58477 3.61503

0.03394 3.79601 4.51836 0.61958 0.77255 0.56425 0.84115

546 2.89015 4.98001 2.46169 1.13957 4.31725 3.22729 3.81015 3.75600 2.68126 3.38645 4.36600 2.89555 3.82889 3.02881 3.08050 2.88174 3.19454 3.44858 5.50348 4.22803 546 - -

2.68618 4.42225 2.77519 2.73123 3.46354 2.40513 3.72494 3.29354 2.67741 2.69355 4.24690 2.90347 2.73739 3.18146 2.89801 2.37887 2.77519 2.98518 4.58477 3.61503

0.03394 3.79601 4.51836 0.61958 0.77255 0.43963 1.03359

547 2.65995 4.67264 2.90022 2.59428 3.81578 3.29672 2.72360 3.54628 2.56804 3.13619 4.03576 3.04816 1.80906 3.00487 2.91725 2.73396 2.96837 3.21803 5.16973 3.78368 547 - -

2.68618 4.42225 2.77519 2.73123 3.46354 2.40513 3.72494 3.29354 2.67741 2.69355 4.24690 2.90347 2.73739 3.18146 2.89801 2.37887 2.77519 2.98518 4.58477 3.61503

0.03090 3.88816 4.61050 0.61958 0.77255 0.48576 0.95510

548 2.43309 4.21303 3.65327 3.24725 3.74193 3.26733 4.18449 2.56806 3.14485 2.59741 3.67271 3.46905 3.89710 3.50102 3.43020 2.68350 1.63473 1.99075 5.30622 4.06921 548 - -

2.68618 4.42225 2.77519 2.73123 3.46354 2.40513 3.72494 3.29354 2.67741 2.69355 4.24690 2.90347 2.73739 3.18146 2.89801 2.37887 2.77519 2.98518 4.58477 3.61503

0.03090 3.88816 4.61050 0.61958 0.77255 0.48576 0.95510

549 2.97870 4.36525 4.51774 4.03025 3.48168 4.20864 4.78615 1.57531 3.90215 2.02380 3.32103 4.27339 4.57669 4.19879 4.12688 3.59396 3.25840 1.14832 5.40028 4.17499 549 - -

2.68618 4.42225 2.77519 2.73123 3.46354 2.40513 3.72494 3.29354 2.67741 2.69355 4.24690 2.90347 2.73739 3.18146 2.89801 2.37887 2.77519 2.98518 4.58477 3.61503

0.03090 3.88816 4.61050 0.61958 0.77255 0.48576 0.95510

550 2.70582 4.81480 2.61873 2.01765 4.25429 3.24300 3.78553 3.67197 2.62104 3.29532 4.18604 2.92769 1.76371 2.98290 3.02397 2.74033 3.01830 3.32629 5.49365 4.18676 550 - -

2.68618 4.42225 2.77519 2.73123 3.46354 2.40513 3.72494 3.29354 2.67741 2.69355 4.24690 2.90347 2.73739 3.18146 2.89801 2.37887 2.77519 2.98518 4.58477 3.61503

0.03090 3.88816 4.61050 0.61958 0.77255 0.48576 0.95510

551 2.90990 5.42000 1.32546 1.77763 4.70796 3.17541 3.73781 4.21142 2.76466 3.74476 4.61675 2.65451 3.80416 2.91308 3.35095 2.80115 3.20409 3.80420 5.90020 4.41486 551 - -

2.68618 4.42225 2.77519 2.73123 3.46354 2.40513 3.72494 3.29354 2.67741 2.69355 4.24690 2.90347 2.73739 3.18146 2.89801 2.37887 2.77519 2.98518 4.58477 3.61503

0.03090 3.88816 4.61050 0.61958 0.77255 0.48576 0.95510

552 2.82519 4.66519 3.28398 2.85589 3.06090 3.56716 1.87618 3.17353 2.59633 2.26175 3.76308 3.25464 4.00906 3.10044 2.88546 2.93607 3.07691 2.97725 4.60645 3.06691 552 - -

2.68618 4.42225 2.77519 2.73123 3.46354 2.40513 3.72494 3.29354 2.67741 2.69355 4.24690 2.90347 2.73739 3.18146 2.89801 2.37887 2.77519 2.98518 4.58477 3.61503

0.03090 3.88816 4.61050 0.61958 0.77255 0.48576 0.95510

553 2.59661 4.62926 2.78882 2.15171 4.07648 3.25265 3.79057 3.33763 2.61108 3.07883 3.98484 2.99116 3.81296 2.99585 2.99270 2.68042 1.73964 3.02908 5.39582 4.09498 553 - -

2.68618 4.42225 2.77519 2.73123 3.46354 2.40513 3.72494 3.29354 2.67741 2.69355 4.24690 2.90347 2.73739 3.18146 2.89801 2.37887 2.77519 2.98518 4.58477 3.61503

0.03090 3.88816 4.61050 0.61958 0.77255 0.48576 0.95510

554 2.84927 5.28353 1.35348 2.10582 4.60141 3.16370 3.74489 4.16011 2.76519 3.70361 4.56951 2.19979 3.79469 2.92618 3.33500 2.76738 3.15845 3.74222 5.82920 4.34670 554 - -

2.68618 4.42225 2.77519 2.73123 3.46354 2.40513 3.72494 3.29354 2.67741 2.69355 4.24690 2.90347 2.73739 3.18146 2.89801 2.37887 2.77519 2.98518 4.58477 3.61503
0.03090 3.88816 4.61050 0.61958 0.77255 0.48576 0.95510
555 2.96544 5.08017 2.49372 1.00328 4.42830 3.28393 3.87185 3.88530 2.75433 3.49981 4.47296 2.94132 3.88920 3.08706 3.16494 2.94613 3.26973 3.56745 5.59690 4.31893 555 - -
2.68618 4.42225 2.77519 2.73123 3.46354 2.40513 3.72494 3.29354 2.67741 2.69355 4.24690 2.90347 2.73739 3.18146 2.89801 2.37887 2.77519 2.98518 4.58477 3.61503
0.03090 3.88816 4.61050 0.61958 0.77255 0.48576 0.95510
556 2.82422 5.18934 2.29499 1.41386 4.50402 3.22418 3.70655 3.99309 2.58195 3.54934 4.40938 2.27111 3.80961 2.87833 3.05212 2.76542 3.10853 3.61085 5.69829 4.27760 556 - -
2.68618 4.42225 2.77519 2.73123 3.46354 2.40513 3.72494 3.29354 2.67741 2.69355 4.24690 2.90347 2.73739 3.18146 2.89801 2.37887 2.77519 2.98518 4.58477 3.61503
0.03090 3.88816 4.61050 0.61958 0.77255 0.48576 0.95510
557 1.43097 4.16544 3.68648 3.31286 3.80505 3.18571 4.24627 2.61553 3.24570 2.66842 3.73556 3.47991 3.85608 3.57219 3.52250 2.61918 2.79788 1.98981 5.36752 4.13871 557 - -
2.68618 4.42225 2.77519 2.73123 3.46354 2.40513 3.72494 3.29354 2.67741 2.69355 4.24690 2.90347 2.73739 3.18146 2.89801 2.37887 2.77519 2.98518 4.58477 3.61503
0.03090 3.88816 4.61050 0.61958 0.77255 0.48576 0.95510
558 2.32290 4.20089 3.37764 3.07855 3.94507 1.54834 4.11527 3.14797 3.08562 3.00091 3.95635 3.30297 3.74855 3.41969 3.38553 2.51637 2.76623 2.20954 5.37442 4.12625 558 - -
2.68618 4.42225 2.77519 2.73123 3.46354 2.40513 3.72494 3.29354 2.67741 2.69355 4.24690 2.90347 2.73739 3.18146 2.89801 2.37887 2.77519 2.98518 4.58477 3.61503
0.03090 3.88816 4.61050 0.61958 0.77255 0.48576 0.95510
559 2.96544 5.08017 2.49372 1.00328 4.42830 3.28393 3.87185 3.88530 2.75433 3.49981 4.47296 2.94132 3.88920 3.08706 3.16494 2.94613 3.26973 3.56745 5.59690 4.31893 559 - -
2.68618 4.42225 2.77519 2.73123 3.46354 2.40513 3.72494 3.29354 2.67741 2.69355 4.24690 2.90347 2.73739 3.18146 2.89801 2.37887 2.77519 2.98518 4.58477 3.61503
0.03090 3.88816 4.61050 0.61958 0.77255 0.48576 0.95510
560 2.56589 4.72130 2.12064 2.36304 4.30080 3.12367 3.80080 3.77614 2.74554 3.40316 4.25280 2.85952 3.74846 2.99578 3.21988 1.57176 2.92416 3.35686 5.58596 4.21023 560 - -
2.68618 4.42225 2.77519 2.73123 3.46354 2.40513 3.72494 3.29354 2.67741 2.69355 4.24690 2.90347 2.73739 3.18146 2.89801 2.37887 2.77519 2.98518 4.58477 3.61503
0.03090 3.88816 4.61050 0.61958 0.77255 0.48576 0.95510
561 2.71420 4.72206 2.86105 1.65980 2.81226 3.41640 3.72272 3.17913 2.63615 2.81077 3.80418 3.02879 3.88815 2.97945 3.04811 2.78609 2.97161 2.95186 5.06497 3.64459 561 - -
2.68618 4.42225 2.77519 2.73123 3.46354 2.40513 3.72494 3.29354 2.67741 2.69355 4.24690 2.90347 2.73739 3.18146 2.89801 2.37887 2.77519 2.98518 4.58477 3.61503
0.03090 3.88816 4.61050 0.61958 0.77255 0.48576 0.95510
562 1.45138 4.28761 3.63785 3.18682 3.63290 3.37535 4.11502 2.66845 3.08248 2.47623 2.76776 3.46514 3.94807 3.43323 3.38137 2.75980 2.87840 2.46979 5.20258 3.96930 562 - -
2.68618 4.42225 2.77519 2.73123 3.46354 2.40513 3.72494 3.29354 2.67741 2.69355 4.24690 2.90347 2.73739 3.18146 2.89801 2.37887 2.77519 2.98518 4.58477 3.61503
0.02840 3.97121 4.69355 0.61958 0.77255 0.48576 0.95510
563 2.92091 5.50623 1.40990 1.82125 4.78811 3.20985 3.74116 4.31178 2.75130 3.80322 4.64635 2.23697 3.82619 2.90419 3.34763 2.80390 3.20658 3.88013 5.96221 4.45074 563 - -
2.68618 4.42225 2.77519 2.73123 3.46354 2.40513 3.72494 3.29354 2.67741 2.69355 4.24690 2.90347 2.73739 3.18146 2.89801 2.37887 2.77519 2.98518 4.58477 3.61503
0.02840 3.97121 4.69355 0.61958 0.77255 0.48576 0.95510
564 2.64213 4.61750 3.05377 1.88429 3.65126 3.47164 3.69624 2.68123 2.56165 2.76656 3.68630 3.06667 3.88125 2.92851 2.96557 2.74104 2.87766 2.84035 5.06471 3.06497 564 - -
2.68618 4.42225 2.77519 2.73123 3.46354 2.40513 3.72494 3.29354 2.67741 2.69355 4.24690 2.90347 2.73739 3.18146 2.89801 2.37887 2.77519 2.98518 4.58477 3.61503
0.02840 3.97121 4.69355 0.61958 0.77255 0.48576 0.95510
565 2.67087 4.94807 1.59368 2.27646 4.46784 2.59200 3.76553 3.91055 2.69887 3.49885 4.32727 2.81151 3.78731 2.93328 3.21458 2.66632 2.46958 3.49260 5.69946 4.29915 565 - -
2.68618 4.42225 2.77519 2.73123 3.46354 2.40513 3.72494 3.29354 2.67741 2.69355 4.24690 2.90347 2.73739 3.18146 2.89801 2.37887 2.77519 2.98518 4.58477 3.61503
0.11395 3.97121 2.42082 0.61958 0.77255 0.48576 0.95510

566 1.27270 4.10671 3.36642 3.10686 4.15071 2.90564 4.16682 3.45385 3.14942 3.24148 4.11634 3.25957 3.65261 3.45730 3.44205 1.94427 2.66980 2.98172 5.53318 4.30717 566 - -
2.68618 4.42225 2.77519 2.73123 3.46354 2.40513 3.72494 3.29354 2.67741 2.69355 4.24690 2.90347 2.73739 3.18146 2.89801 2.37887 2.77519 2.98518 4.58477 3.61503
0.03090 3.88816 4.61050 0.61958 0.77255 0.56302 0.84277
567 2.77181 4.52677 3.38064 3.22178 4.21596 3.21338 4.29811 3.76521 3.28039 3.39043 4.44081 3.52700 0.79898 3.66869 3.52431 2.93975 3.21457 3.40882 5.39840 4.33677 567 - -
2.68618 4.42225 2.77519 2.73123 3.46354 2.40513 3.72494 3.29354 2.67741 2.69355 4.24690 2.90347 2.73739 3.18146 2.89801 2.37887 2.77519 2.98518 4.58477 3.61503
0.03090 3.88816 4.61050 0.61958 0.77255 0.56302 0.84277
568 1.81465 4.16771 3.21521 3.02437 4.27158 1.41991 4.17476 3.62784 3.16430 3.37381 4.24267 3.22617 3.66112 3.45730 3.47010 2.40511 2.72182 3.11664 5.60488 4.39613 568 - -
2.68618 4.42225 2.77519 2.73123 3.46354 2.40513 3.72494 3.29354 2.67741 2.69355 4.24690 2.90347 2.73739 3.18146 2.89801 2.37887 2.77519 2.98518 4.58477 3.61503
0.03090 3.88816 4.61050 0.61958 0.77255 0.56302 0.84277
569 2.77411 5.01711 2.39941 1.37450 4.46188 2.46740 3.76338 3.91821 2.65617 3.50323 4.36918 2.81393 3.80704 2.94607 3.11796 2.75457 3.08179 3.53353 5.65273 4.29570 569 - -
2.68618 4.42225 2.77519 2.73123 3.46354 2.40513 3.72494 3.29354 2.67741 2.69355 4.24690 2.90347 2.73739 3.18146 2.89801 2.37887 2.77519 2.98518 4.58477 3.61503
0.03090 3.88816 4.61050 0.61958 0.77255 0.56302 0.84277
570 2.98617 4.95093 3.16322 2.78153 4.34094 3.47413 3.76745 3.77115 1.01702 3.33272 4.29316 3.20537 3.97322 2.95792 2.43403 3.02702 3.23818 3.47799 5.38902 4.20398 570 - -
2.68618 4.42225 2.77519 2.73123 3.46354 2.40513 3.72494 3.29354 2.67741 2.69355 4.24690 2.90347 2.73739 3.18146 2.89801 2.37887 2.77519 2.98518 4.58477 3.61503
0.03090 3.88816 4.61050 0.61958 0.77255 0.56302 0.84277
571 2.71852 4.96772 2.42516 1.47321 4.37648 3.21283 3.72667 3.81568 2.58811 3.41946 4.28137 2.80437 3.79493 2.90411 3.03758 2.26340 3.02276 3.44094 5.60899 4.22309 571 - -
2.68618 4.42225 2.77519 2.73123 3.46354 2.40513 3.72494 3.29354 2.67741 2.69355 4.24690 2.90347 2.73739 3.18146 2.89801 2.37887 2.77519 2.98518 4.58477 3.61503
0.12608 3.88816 2.32307 0.61958 0.77255 0.56302 0.84277
572 2.89015 4.98001 2.46169 1.13957 4.31725 3.22729 3.81015 3.75600 2.68126 3.38645 4.36600 2.89555 3.82889 3.02881 3.08050 2.88174 3.19454 3.44858 5.50348 4.22803 572 - -
2.68618 4.42225 2.77519 2.73123 3.46354 2.40513 3.72494 3.29354 2.67741 2.69355 4.24690 2.90347 2.73739 3.18146 2.89801 2.37887 2.77519 2.98518 4.58477 3.61503
0.03394 3.79601 4.51836 0.61958 0.77255 0.63588 0.75389
573 2.97165 4.41429 4.22289 3.83744 3.32476 3.98045 4.58108 1.17818 3.66390 1.92890 3.27450 4.09357 4.42084 4.02132 3.86934 3.47002 3.27299 1.84997 5.19827 3.92855 573 - -
2.68618 4.42225 2.77519 2.73123 3.46354 2.40513 3.72494 3.29354 2.67741 2.69355 4.24690 2.90347 2.73739 3.18146 2.89801 2.37887 2.77519 2.98518 4.58477 3.61503
0.03394 3.79601 4.51836 0.61958 0.77255 0.63588 0.75389
574 2.97165 4.41429 4.22289 3.83744 3.32476 3.98045 4.58108 1.17818 3.66390 1.92890 3.27450 4.09357 4.42084 4.02132 3.86934 3.47002 3.27299 1.84997 5.19827 3.92855 574 - -
2.68618 4.42225 2.77519 2.73123 3.46354 2.40513 3.72494 3.29354 2.67741 2.69355 4.24690 2.90347 2.73739 3.18146 2.89801 2.37887 2.77519 2.98518 4.58477 3.61503
0.03394 3.79601 4.51836 0.61958 0.77255 0.63588 0.75389
575 2.91056 5.02139 1.03432 2.29423 4.38899 3.15549 3.86877 3.96045 2.93927 3.58342 4.56179 2.84589 3.80315 3.10925 3.44342 2.88302 3.24824 3.60850 5.57234 4.27708 575 - -
2.68618 4.42225 2.77519 2.73123 3.46354 2.40513 3.72494 3.29354 2.67741 2.69355 4.24690 2.90347 2.73739 3.18146 2.89801 2.37887 2.77519 2.98518 4.58477 3.61503
0.03394 3.79601 4.51836 0.61958 0.77255 0.63588 0.75389
576 2.65642 4.42430 3.25570 3.08531 4.08348 3.11624 4.16757 3.61095 3.13637 3.24548 4.29346 3.39875 0.98293 3.52758 3.38897 2.82198 3.09244 3.26489 5.28934 4.20377 576 - -
2.68618 4.42225 2.77519 2.73123 3.46354 2.40513 3.72494 3.29354 2.67741 2.69355 4.24690 2.90347 2.73739 3.18146 2.89801 2.37887 2.77519 2.98518 4.58477 3.61503
0.03394 3.79601 4.51836 0.61958 0.77255 0.63588 0.75389
577 2.89015 4.98001 2.46169 1.13957 4.31725 3.22729 3.81015 3.75600 2.68126 3.38645 4.36600 2.89555 3.82889 3.02881 3.08050 2.88174 3.19454 3.44858 5.50348 4.22803 577 - -
2.68618 4.42225 2.77519 2.73123 3.46354 2.40513 3.72494 3.29354 2.67741 2.69355 4.24690 2.90347 2.73739 3.18146 2.89801 2.37887 2.77519 2.98518 4.58477 3.61503

0.03394 3.79601 4.51836 0.61958 0.77255 0.63588 0.75389

578 1.13001 4.15738 3.38757 3.17757 3.97857 2.98348 4.20830 3.09294 3.19189 2.97777 4.01983 3.34570 3.71568 3.54062 3.44647 2.50924 2.78140 2.76077 5.41351 4.20565 578 - -

2.68618 4.42225 2.77519 2.73123 3.46354 2.40513 3.72494 3.29354 2.67741 2.69355 4.24690 2.90347 2.73739 3.18146 2.89801 2.37887 2.77519 2.98518 4.58477 3.61503

0.03394 3.79601 4.51836 0.61958 0.77255 0.63588 0.75389

579 2.91056 5.02139 1.03432 2.29423 4.38899 3.15549 3.86877 3.96045 2.93927 3.58342 4.56179 2.84589 3.80315 3.10925 3.44342 2.88302 3.24824 3.60850 5.57234 4.27708 579 - -

2.68618 4.42225 2.77519 2.73123 3.46354 2.40513 3.72494 3.29354 2.67741 2.69355 4.24690 2.90347 2.73739 3.18146 2.89801 2.37887 2.77519 2.98518 4.58477 3.61503

0.03394 3.79601 4.51836 0.61958 0.77255 0.63588 0.75389

580 2.91056 5.02139 1.03432 2.29423 4.38899 3.15549 3.86877 3.96045 2.93927 3.58342 4.56179 2.84589 3.80315 3.10925 3.44342 2.88302 3.24824 3.60850 5.57234 4.27708 580 - -

2.68618 4.42225 2.77519 2.73123 3.46354 2.40513 3.72494 3.29354 2.67741 2.69355 4.24690 2.90347 2.73739 3.18146 2.89801 2.37887 2.77519 2.98518 4.58477 3.61503

0.03394 3.79601 4.51836 0.61958 0.77255 0.63588 0.75389

581 2.91056 5.02139 1.03432 2.29423 4.38899 3.15549 3.86877 3.96045 2.93927 3.58342 4.56179 2.84589 3.80315 3.10925 3.44342 2.88302 3.24824 3.60850 5.57234 4.27708 581 - -

2.68618 4.42225 2.77519 2.73123 3.46354 2.40513 3.72494 3.29354 2.67741 2.69355 4.24690 2.90347 2.73739 3.18146 2.89801 2.37887 2.77519 2.98518 4.58477 3.61503

0.03394 3.79601 4.51836 0.61958 0.77255 0.51127 0.91563

582 1.56529 4.25715 3.62740 3.20447 2.60624 3.37569 4.03763 2.65879 3.16027 2.42189 3.51857 3.46586 3.94221 3.45979 3.45376 2.76735 2.87642 2.46667 4.93197 3.56852 582 - -

2.68618 4.42225 2.77519 2.73123 3.46354 2.40513 3.72494 3.29354 2.67741 2.69355 4.24690 2.90347 2.73739 3.18146 2.89801 2.37887 2.77519 2.98518 4.58477 3.61503

0.03090 3.88816 4.61050 0.61958 0.77255 0.44031 1.03236

583 2.40450 4.35054 4.09330 3.58137 1.82363 3.86992 4.10139 2.39540 3.47152 1.75795 3.19144 3.82079 4.25543 3.71076 3.69247 3.19901 3.09842 2.32028 4.65876 3.25477 583 - -

2.68618 4.42225 2.77519 2.73123 3.46354 2.40513 3.72494 3.29354 2.67741 2.69355 4.24690 2.90347 2.73739 3.18146 2.89801 2.37887 2.77519 2.98518 4.58477 3.61503

0.02840 3.97121 4.69355 0.61958 0.77255 0.48576 0.95510

584 2.74810 4.28294 3.97612 3.42874 1.98640 3.78820 4.04381 2.52317 3.32396 2.02756 2.33925 3.70281 3.17063 3.57297 3.56016 3.08840 2.99207 2.42838 4.71163 3.40309 584 - -

2.68618 4.42225 2.77519 2.73123 3.46354 2.40513 3.72494 3.29354 2.67741 2.69355 4.24690 2.90347 2.73739 3.18146 2.89801 2.37887 2.77519 2.98518 4.58477 3.61503

0.02618 4.05166 4.77401 0.61958 0.77255 0.48576 0.95510

585 2.90288 4.65697 3.61307 3.53701 4.66925 0.49934 4.64306 4.31567 3.74245 3.94626 4.92592 3.77787 4.05365 4.05269 3.95566 3.08049 3.39808 3.80939 5.71372 4.77837 585 - -

2.68618 4.42225 2.77519 2.73123 3.46354 2.40513 3.72494 3.29354 2.67741 2.69355 4.24690 2.90347 2.73739 3.18146 2.89801 2.37887 2.77519 2.98518 4.58477 3.61503

0.02618 4.05166 4.77401 0.61958 0.77255 0.48576 0.95510

586 2.62363 4.36923 3.43028 2.57649 3.50436 3.57855 3.81797 2.07674 2.67244 2.53874 2.62139 3.29061 3.95951 2.85107 3.11136 2.83255 2.85375 2.60855 4.94581 3.71043 586 - -

2.68618 4.42225 2.77519 2.73123 3.46354 2.40513 3.72494 3.29354 2.67741 2.69355 4.24690 2.90347 2.73739 3.18146 2.89801 2.37887 2.77519 2.98518 4.58477 3.61503

0.05347 4.13150 3.32402 0.61958 0.77255 0.48576 0.95510

587 2.34921 4.92850 2.64794 2.30061 4.26828 3.35625 3.68554 3.69891 2.24720 3.27611 4.08643 2.90753 3.82765 2.83362 2.95252 1.76923 2.92311 3.33693 5.48920 4.13117 587 - -

2.68618 4.42225 2.77519 2.73123 3.46354 2.40513 3.72494 3.29354 2.67741 2.69355 4.24690 2.90347 2.73739 3.18146 2.89801 2.37887 2.77519 2.98518 4.58477 3.61503

0.02486 4.10289 4.82523 0.61958 0.77255 0.51859 0.90475

588 2.11767 4.36842 3.15957 2.82428 3.52645 3.30263 3.81388 2.90661 2.80755 2.63056 3.52191 3.24133 3.91591 3.12018 3.17839 2.76582 2.83053 2.40047 4.96408 2.34375 588 - -

2.68618 4.42225 2.77519 2.73123 3.46354 2.40513 3.72494 3.29354 2.67741 2.69355 4.24690 2.90347 2.73739 3.18146 2.89801 2.37887 2.77519 2.98518 4.58477 3.61503

0.04689 4.10289 3.53081 0.61958 0.77255 0.51859 0.90475

589 1.86812 4.26126 3.39124 3.22332 4.46781 1.07932 4.36492 3.84709 3.38196 3.58316 4.44024 3.38088 3.77262 3.65707 3.67309 2.50490 2.83403 3.28851 5.79157 4.60145 589 - -

2.68618 4.42225 2.77519 2.73123 3.46354 2.40513 3.72494 3.29354 2.67741 2.69355 4.24690 2.90347 2.73739 3.18146 2.89801 2.37887 2.77519 2.98518 4.58477 3.61503
0.02540 4.08140 4.80375 0.61958 0.77255 0.50301 0.92812

590 2.28188 4.32956 3.45223 2.92261 2.16309 3.48441 3.84463 2.90385 2.83992 2.61064 3.51340 3.30283 3.92750 3.17819 2.74748 2.38632 2.83488 2.66357 4.93108 3.65459 590 - -
2.68618 4.42225 2.77519 2.73123 3.46354 2.40513 3.72494 3.29354 2.67741 2.69355 4.24690 2.90347 2.73739 3.18146 2.89801 2.37887 2.77519 2.98518 4.58477 3.61503
0.02486 4.10289 4.82523 0.61958 0.77255 0.51859 0.90475

591 2.26777 4.37500 3.40219 2.85903 3.65787 3.44683 3.84604 2.94328 2.76408 2.70147 3.59280 3.25694 3.61076 3.12270 2.60146 2.73422 2.50035 1.93034 5.07039 3.82721 591 - -
2.68629 4.42236 2.77530 2.73134 3.46364 2.40523 3.72456 3.29365 2.67717 2.69365 4.24700 2.90335 2.73750 3.18157 2.89811 2.37872 2.77511 2.98482 4.58488 3.61514
0.15080 2.43565 2.94805 1.60255 0.22487 0.51859 0.90475

592 2.77155 3.05904 4.42168 3.87402 2.71414 3.91607 4.37133 2.08757 3.74686 2.15853 3.27633 4.02075 4.31462 3.95867 3.90576 3.25662 3.03930 1.31036 4.95488 3.71428 601 - -
2.68618 4.42225 2.77519 2.73123 3.46354 2.40513 3.72494 3.29354 2.67741 2.69355 4.24690 2.90347 2.73739 3.18146 2.89801 2.37887 2.77519 2.98518 4.58477 3.61503
0.02603 4.05745 4.77980 0.61958 0.77255 0.56683 0.83776

593 2.61676 4.38362 3.65487 3.23145 3.67385 3.45363 4.16170 2.69687 3.08273 2.16850 3.65299 3.52077 4.02487 3.48019 3.36890 2.85529 1.39672 2.50593 5.25243 3.98674 602 - -
2.68618 4.42225 2.77519 2.73123 3.46354 2.40513 3.72494 3.29354 2.67741 2.69355 4.24690 2.90347 2.73739 3.18146 2.89801 2.37887 2.77519 2.98518 4.58477 3.61503
0.02603 4.05745 4.77980 0.61958 0.77255 0.56683 0.83776

594 2.74437 5.20785 2.24316 1.64945 4.51523 3.32478 3.65470 3.98538 2.18723 3.49738 4.28718 2.79645 3.81578 2.78531 2.97624 2.69057 2.59003 3.58050 5.66028 4.24109 603 - -
2.68618 4.42225 2.77519 2.73123 3.46354 2.40513 3.72494 3.29354 2.67741 2.69355 4.24690 2.90347 2.73739 3.18146 2.89801 2.37887 2.77519 2.98518 4.58477 3.61503
0.02603 4.05745 4.77980 0.61958 0.77255 0.56683 0.83776

595 2.73051 4.34891 3.71455 3.19279 3.11903 3.70542 3.92014 2.67291 3.06604 1.49316 3.32509 3.52738 3.15367 3.39045 3.34518 3.00358 2.97333 2.53478 4.71235 2.78832 604 - -
2.68618 4.42225 2.77519 2.73123 3.46354 2.40513 3.72494 3.29354 2.67741 2.69355 4.24690 2.90347 2.73739 3.18146 2.89801 2.37887 2.77519 2.98518 4.58477 3.61503
0.02603 4.05745 4.77980 0.61958 0.77255 0.56683 0.83776

596 2.47097 4.38218 3.30153 2.81005 3.81863 3.30123 3.86479 3.16194 2.76839 2.48585 3.74498 3.19554 2.96097 3.11249 3.14367 2.30924 1.88257 2.85652 5.20209 3.94382 605 - -
2.68618 4.42225 2.77519 2.73123 3.46354 2.40513 3.72494 3.29354 2.67741 2.69355 4.24690 2.90347 2.73739 3.18146 2.89801 2.37887 2.77519 2.98518 4.58477 3.61503
0.02603 4.05745 4.77980 0.61958 0.77255 0.56683 0.83776

597 3.33846 4.66682 4.55813 4.12350 1.23795 4.27622 3.69051 2.90007 3.97177 1.89271 3.51707 4.06510 4.57844 4.03259 4.06028 3.61056 3.56069 2.86313 3.89056 1.89704 606 - -
2.68618 4.42225 2.77519 2.73123 3.46354 2.40513 3.72494 3.29354 2.67741 2.69355 4.24690 2.90347 2.73739 3.18146 2.89801 2.37887 2.77519 2.98518 4.58477 3.61503
0.02603 4.05745 4.77980 0.61958 0.77255 0.56683 0.83776

598 2.78893 4.41296 3.77115 3.22464 3.41012 3.77800 4.05652 2.48465 2.54566 1.45666 3.33359 3.58432 4.15721 3.39663 3.23648 3.08348 3.03026 2.11307 5.02622 3.80764 607 - -
2.68618 4.42225 2.77519 2.73123 3.46354 2.40513 3.72494 3.29354 2.67741 2.69355 4.24690 2.90347 2.73739 3.18146 2.89801 2.37887 2.77519 2.98518 4.58477 3.61503
0.02603 4.05745 4.77980 0.61958 0.77255 0.56683 0.83776

599 2.81000 4.77054 2.99561 1.75793 3.81986 3.53221 3.84934 3.07177 2.66691 1.82741 3.76871 3.14894 3.99203 3.06955 3.04043 2.90631 3.06050 2.88882 5.28088 3.97165 608 - -
2.68618 4.42225 2.77519 2.73123 3.46354 2.40513 3.72494 3.29354 2.67741 2.69355 4.24690 2.90347 2.73739 3.18146 2.89801 2.37887 2.77519 2.98518 4.58477 3.61503
0.02603 4.05745 4.77980 0.61958 0.77255 0.56683 0.83776

600 2.57316 4.57258 2.68907 2.59456 3.83690 3.40349 3.74228 3.15706 2.61641 2.51680 3.74730 3.06935 3.84999 2.94990 3.04376 1.94856 2.83745 2.55386 5.20015 3.90015 609 - -
2.68618 4.42225 2.77519 2.73123 3.46354 2.40513 3.72494 3.29354 2.67741 2.69355 4.24690 2.90347 2.73739 3.18146 2.89801 2.37887 2.77519 2.98518 4.58477 3.61503
0.02603 4.05745 4.77980 0.61958 0.77255 0.56683 0.83776

601 2.71252 4.24361 3.94061 3.36899 2.74435 3.78344 4.05444 2.41057 3.23781 1.92536 3.21582 3.66699 4.14320 3.52292 2.90068 3.06848 2.94755 1.68683 4.81605 3.57603 610 - -
2.68618 4.42225 2.77519 2.73123 3.46354 2.40513 3.72494 3.29354 2.67741 2.69355 4.24690 2.90347 2.73739 3.18146 2.89801 2.37887 2.77519 2.98518 4.58477 3.61503
0.02603 4.05745 4.77980 0.61958 0.77255 0.56683 0.83776
602 2.72069 4.31440 3.95256 3.43205 3.52546 3.73893 4.26622 2.23990 3.32104 2.05492 3.43010 3.72780 4.19292 3.63943 3.60344 3.07632 1.91134 1.67462 5.16977 3.95156 611 - -
2.68618 4.42225 2.77519 2.73123 3.46354 2.40513 3.72494 3.29354 2.67741 2.69355 4.24690 2.90347 2.73739 3.18146 2.89801 2.37887 2.77519 2.98518 4.58477 3.61503
0.02603 4.05745 4.77980 0.61958 0.77255 0.56683 0.83776
603 2.27597 4.30085 3.47339 2.92801 3.42766 3.48978 3.83145 2.84983 2.84729 2.30642 3.46712 3.30541 3.92164 3.17531 3.18442 1.97747 2.81799 2.61671 3.50531 3.61195 612 - -
2.68618 4.42225 2.77519 2.73123 3.46354 2.40513 3.72494 3.29354 2.67741 2.69355 4.24690 2.90347 2.73739 3.18146 2.89801 2.37887 2.77519 2.98518 4.58477 3.61503
0.02603 4.05745 4.77980 0.61958 0.77255 0.56683 0.83776
604 2.81632 4.34340 4.03390 3.46268 3.28188 3.89431 4.18472 2.19376 3.33365 2.02229 1.78382 3.77456 4.23986 2.97988 3.58206 3.18050 3.04913 2.09957 4.95120 3.77407 613 - -
2.68618 4.42225 2.77519 2.73123 3.46354 2.40513 3.72494 3.29354 2.67741 2.69355 4.24690 2.90347 2.73739 3.18146 2.89801 2.37887 2.77519 2.98518 4.58477 3.61503
0.02282 4.18713 4.90948 0.61958 0.77255 0.56683 0.83776
605 2.26602 5.00799 2.11792 2.35948 4.31585 2.96581 3.63591 3.76522 2.41244 3.30910 4.09003 2.89845 3.81717 2.50587 2.71780 2.63481 2.88524 3.10401 5.49564 4.11729 614 - -
2.68618 4.42225 2.77519 2.73123 3.46354 2.40513 3.72494 3.29354 2.67741 2.69355 4.24690 2.90347 2.73739 3.18146 2.89801 2.37887 2.77519 2.98518 4.58477 3.61503
0.02282 4.18713 4.90948 0.61958 0.77255 0.56683 0.83776
606 2.29515 4.25357 4.05917 3.53000 3.52384 3.70968 4.25957 2.36488 3.42816 2.46033 3.00494 3.76641 4.17709 3.70623 3.67507 2.76652 2.97753 1.27274 5.08876 3.88283 615 - -
2.68618 4.42225 2.77519 2.73123 3.46354 2.40513 3.72494 3.29354 2.67741 2.69355 4.24690 2.90347 2.73739 3.18146 2.89801 2.37887 2.77519 2.98518 4.58477 3.61503
0.02179 4.23290 4.95524 0.61958 0.77255 0.56683 0.83776
607 2.37236 4.91469 2.91940 2.42841 4.27125 3.05477 3.12780 3.71227 2.46536 3.27642 4.06634 2.15489 2.95860 2.81287 2.93822 2.47463 2.70270 3.33582 5.47899 4.11861 616 - -
2.68618 4.42225 2.77519 2.73123 3.46354 2.40513 3.72494 3.29354 2.67741 2.69355 4.24690 2.90347 2.73739 3.18146 2.89801 2.37887 2.77519 2.98518 4.58477 3.61503
0.02179 4.23290 4.95524 0.61958 0.77255 0.51400 0.91154
608 2.41950 5.06859 2.81274 2.07654 4.38143 3.41183 3.24299 3.83687 2.44339 3.37177 4.15110 2.14089 3.84319 2.78730 2.92775 2.23934 2.74091 3.44888 5.55111 4.16594 617 - -
2.68618 4.42225 2.77519 2.73123 3.46354 2.40513 3.72494 3.29354 2.67741 2.69355 4.24690 2.90347 2.73739 3.18146 2.89801 2.37887 2.77519 2.98518 4.58477 3.61503
0.06436 4.25807 3.03269 0.61958 0.77255 0.53557 0.88028
609 2.65021 4.94756 2.97482 2.44386 4.29358 2.65806 3.65530 3.72951 2.37858 3.28014 4.06975 2.96263 3.35337 2.52166 2.09296 2.39630 2.70792 3.35617 5.46707 4.11803 618 - -
2.68618 4.42225 2.77519 2.73123 3.46354 2.40513 3.72494 3.29354 2.67741 2.69355 4.24690 2.90347 2.73739 3.18146 2.89801 2.37887 2.77519 2.98518 4.58477 3.61503
0.02217 4.21587 4.93822 0.61958 0.77255 0.50726 0.92166
610 2.27648 4.52590 3.28596 2.76995 4.05708 2.80626 3.88908 3.45793 2.75874 3.10855 3.52755 3.18316 2.11508 3.09035 3.17611 2.17416 2.40677 3.09830 5.38393 4.10321 619 - -
2.68618 4.42225 2.77519 2.73123 3.46354 2.40513 3.72494 3.29354 2.67741 2.69355 4.24690 2.90347 2.73739 3.18146 2.89801 2.37887 2.77519 2.98518 4.58477 3.61503
0.17571 4.25478 1.91770 0.61958 0.77255 0.53976 0.87441
611 2.44128 4.80057 2.92420 2.44333 4.06966 3.18532 3.45375 3.48926 2.46036 2.73233 3.91392 2.13370 3.82655 2.71630 2.90896 2.41102 2.86219 3.16270 5.33433 4.00200 620 - -
2.68618 4.42225 2.77519 2.73123 3.46354 2.40513 3.72494 3.29354 2.67741 2.69355 4.24690 2.90347 2.73739 3.18146 2.89801 2.37887 2.77519 2.98518 4.58477 3.61503
0.15690 4.10390 2.05028 0.61958 0.77255 0.70322 0.68317
612 2.71812 4.85113 3.01881 2.53689 4.08678 3.44252 3.63865 3.56431 1.55379 3.14593 3.99531 3.01589 3.87236 2.80687 2.58100 2.40348 2.95361 3.24642 5.29906 3.52037 621 - -
2.68618 4.42225 2.77519 2.73123 3.46354 2.40513 3.72494 3.29354 2.67741 2.69355 4.24690 2.90347 2.73739 3.18146 2.89801 2.37887 2.77519 2.98518 4.58477 3.61503

0.02829 3.97529 4.69763 0.61958 0.77255 0.80916 0.58921

613 2.70420 4.96082 2.87406 2.42543 4.29764 3.40061 3.61587 3.72867 2.25132 3.26812 4.08489 2.75534 3.05973 1.90467 2.41970 2.70529 2.93939 3.37305 5.43163 4.11002 622 - -

2.68618 4.42225 2.77519 2.73123 3.46354 2.40513 3.72494 3.29354 2.67741 2.69355 4.24690 2.90347 2.73739 3.18146 2.89801 2.37887 2.77519 2.98518 4.58477 3.61503

0.02829 3.97529 4.69763 0.61958 0.77255 0.56377 0.84179

614 2.37233 4.93938 2.59476 2.39455 4.26031 3.39513 3.63322 3.69612 2.38301 3.25365 4.04545 2.78466 3.58344 2.76355 1.94836 2.63612 2.87454 3.16635 5.44751 4.09010 623 - -

2.68618 4.42225 2.77519 2.73123 3.46354 2.40513 3.72494 3.29354 2.67741 2.69355 4.24690 2.90347 2.73739 3.18146 2.89801 2.37887 2.77519 2.98518 4.58477 3.61503

0.04554 4.13150 3.55943 0.61958 0.77255 0.67696 0.70960

615 2.44585 4.50316 2.97391 2.72803 4.42937 2.33254 4.01994 3.89663 2.93010 3.51160 4.32430 2.51155 3.78535 3.22279 3.34224 1.35953 2.86843 3.38280 5.70639 4.40515 624 - -

2.68618 4.42225 2.77519 2.73123 3.46354 2.40513 3.72494 3.29354 2.67741 2.69355 4.24690 2.90347 2.73739 3.18146 2.89801 2.37887 2.77519 2.98518 4.58477 3.61503

0.02466 4.11063 4.83297 0.61958 0.77255 0.69695 0.68936

616 2.70033 4.91217 2.94843 2.48055 4.21815 3.43279 2.27437 3.65638 2.05974 3.22503 4.04545 2.98511 3.86587 2.81497 2.73036 2.16861 2.80859 3.31357 5.41058 4.08491 625 - -

2.68618 4.42225 2.77519 2.73123 3.46354 2.40513 3.72494 3.29354 2.67741 2.69355 4.24690 2.90347 2.73739 3.18146 2.89801 2.37887 2.77519 2.98518 4.58477 3.61503

0.02466 4.11063 4.83297 0.61958 0.77255 0.62225 0.76946

617 2.54182 4.28823 3.62481 3.05938 2.90089 3.34649 3.88906 2.72745 2.93076 1.70180 3.36906 3.42180 4.00851 3.26797 2.62555 2.89254 2.86888 2.53041 4.85595 3.61529 626 - -

2.68618 4.42225 2.77519 2.73123 3.46354 2.40513 3.72494 3.29354 2.67741 2.69355 4.24690 2.90347 2.73739 3.18146 2.89801 2.37887 2.77519 2.98518 4.58477 3.61503

0.02367 4.15098 4.87333 0.61958 0.77255 0.65755 0.73006

618 2.59019 4.74982 2.92948 2.51177 4.19901 3.08547 3.28444 3.63220 2.57351 3.23135 4.04649 2.61145 3.82911 2.91902 3.01829 1.69085 2.49268 3.25978 5.45675 4.12554 627 - -

2.68618 4.42225 2.77519 2.73123 3.46354 2.40513 3.72494 3.29354 2.67741 2.69355 4.24690 2.90347 2.73739 3.18146 2.89801 2.37887 2.77519 2.98518 4.58477 3.61503

0.02367 4.15098 4.87333 0.61958 0.77255 0.61859 0.77371

619 2.55724 4.42472 3.33242 2.78822 3.68678 3.21444 3.81909 2.68753 2.75369 2.57389 3.62012 3.21275 3.13779 3.07972 3.14319 2.30687 2.04800 2.77826 5.08685 3.83046 628 - -

2.68618 4.42225 2.77519 2.73123 3.46354 2.40513 3.72494 3.29354 2.67741 2.69355 4.24690 2.90347 2.73739 3.18146 2.89801 2.37887 2.77519 2.98518 4.58477 3.61503

0.02320 4.17104 4.89339 0.61958 0.77255 0.47999 0.96440

620 2.32470 4.84366 2.98170 2.48535 4.20187 2.71302 3.33007 3.63034 2.50376 3.21727 4.02130 2.99516 3.84802 2.53822 2.96231 1.91711 2.67439 3.27113 5.44003 4.09875 629 - -

2.68618 4.42225 2.77519 2.73123 3.46354 2.40513 3.72494 3.29354 2.67741 2.69355 4.24690 2.90347 2.73739 3.18146 2.89801 2.37887 2.77519 2.98518 4.58477 3.61503

0.02137 4.25246 4.97481 0.61958 0.77255 0.54268 0.87033

621 2.43959 5.03691 2.91154 2.38668 4.34541 3.23409 3.09137 3.79611 2.20815 3.32759 4.10225 2.92297 3.45346 2.10809 2.83983 2.26913 2.89181 3.41102 5.50159 4.12741 630 - -

2.68618 4.42225 2.77519 2.73123 3.46354 2.40513 3.72494 3.29354 2.67741 2.69355 4.24690 2.90347 2.73739 3.18146 2.89801 2.37887 2.77519 2.98518 4.58477 3.61503

0.02137 4.25246 4.97481 0.61958 0.77255 0.54268 0.87033

622 1.46174 4.59445 3.14526 2.72614 4.16958 2.50145 3.32588 3.58974 2.77261 3.22461 4.05411 3.15349 2.90991 3.10581 3.19061 2.65296 2.89129 3.20806 5.47691 4.18270 631 - -

2.68618 4.42225 2.77519 2.73123 3.46354 2.40513 3.72494 3.29354 2.67741 2.69355 4.24690 2.90347 2.73739 3.18146 2.89801 2.37887 2.77519 2.98518 4.58477 3.61503

0.09335 4.25246 2.59160 0.61958 0.77255 0.54268 0.87033

623 2.60999 4.46038 3.30816 2.74690 3.20355 2.72095 3.76154 2.99802 2.70730 2.68415 3.56102 3.19613 3.91985 3.03547 2.76191 2.08764 2.84296 2.75239 4.04765 3.33067 632 - -

2.68618 4.42225 2.77519 2.73123 3.46354 2.40513 3.72494 3.29354 2.67741 2.69355 4.24690 2.90347 2.73739 3.18146 2.89801 2.37887 2.77519 2.98518 4.58477 3.61503

0.09127 4.18205 2.63166 0.61958 0.77255 0.48733 0.95261

624 2.39940 5.00070 2.85575 1.85718 4.30622 2.54837 3.64486 3.74847 2.40496 3.29726 4.08557 2.91623 3.83124 2.60598 2.69071 2.65587 2.90103 3.21025 5.48687 4.11930 633 - -

2.68618 4.42225 2.77519 2.73123 3.46354 2.40513 3.72494 3.29354 2.67741 2.69355 4.24690 2.90347 2.73739 3.18146 2.89801 2.37887 2.77519 2.98518 4.58477 3.61503
0.02275 4.19042 4.91277 0.61958 0.77255 0.61582 0.77696
625 1.97938 4.25931 3.66400 3.09489 3.39841 3.62975 3.92372 2.68821 3.01528 2.07461 3.10557 3.45027 4.01739 3.31203 3.05019 2.70468 2.85875 2.13296 4.87646 3.65829 634 - -
2.68618 4.42225 2.77519 2.73123 3.46354 2.40513 3.72494 3.29354 2.67741 2.69355 4.24690 2.90347 2.73739 3.18146 2.89801 2.37887 2.77519 2.98518 4.58477 3.61503
0.06023 4.22512 3.12747 0.61958 0.77255 0.61582 0.77696
626 2.56392 4.36713 3.48021 3.00838 2.12302 3.42487 3.93304 3.06733 2.98941 2.75631 3.66967 3.35378 2.57137 3.29369 3.33441 1.97835 2.88020 2.80165 4.98211 3.63700 635 - -
2.68618 4.42225 2.77519 2.73123 3.46354 2.40513 3.72494 3.29354 2.67741 2.69355 4.24690 2.90347 2.73739 3.18146 2.89801 2.37887 2.77519 2.98518 4.58477 3.61503
0.11203 4.18770 2.39910 0.61958 0.77255 0.45330 1.00930
627 2.65485 5.06426 2.14141 2.16075 4.36591 3.40584 3.12723 3.82465 2.29018 3.35023 3.75642 2.88919 3.81773 2.74016 2.87348 2.63296 2.33796 3.43209 5.52133 4.13299 636 - -
2.68618 4.42225 2.77519 2.73123 3.46354 2.40513 3.72494 3.29354 2.67741 2.69355 4.24690 2.90347 2.73739 3.18146 2.89801 2.37887 2.77519 2.98518 4.58477 3.61503
0.04359 4.21488 3.57980 0.61958 0.77255 0.47062 0.97981
628 2.49183 4.75433 3.11115 2.55604 3.99788 3.48415 3.71309 3.39631 2.15417 2.85191 3.52289 3.05781 2.42912 2.88329 2.96087 2.53628 2.46282 3.09154 5.29403 3.98193 637 - -
2.68618 4.42225 2.77519 2.73123 3.46354 2.40513 3.72494 3.29354 2.67741 2.69355 4.24690 2.90347 2.73739 3.18146 2.89801 2.37887 2.77519 2.98518 4.58477 3.61503
0.02031 4.30264 5.02499 0.61958 0.77255 0.51510 0.90991
629 2.21809 4.24649 3.73840 3.16647 2.35597 3.09148 3.94949 2.72082 3.09576 2.26540 2.78207 3.50453 4.04761 3.12186 3.38871 2.71622 2.69243 2.51114 4.84652 3.63285 638 - -
2.68618 4.42225 2.77519 2.73123 3.46354 2.40513 3.72494 3.29354 2.67741 2.69355 4.24690 2.90347 2.73739 3.18146 2.89801 2.37887 2.77519 2.98518 4.58477 3.61503
0.01945 4.34525 5.06760 0.61958 0.77255 0.53238 0.88481
630 2.26916 4.74819 2.93507 2.46340 3.97154 3.49864 3.72174 3.37070 2.55250 2.91199 3.61803 3.06628 2.86935 2.89625 3.00223 2.55866 2.20070 2.77143 5.28088 3.96854 639 - -
2.68618 4.42225 2.77519 2.73123 3.46354 2.40513 3.72494 3.29354 2.67741 2.69355 4.24690 2.90347 2.73739 3.18146 2.89801 2.37887 2.77519 2.98518 4.58477 3.61503
0.03816 4.34525 3.71017 0.61958 0.77255 0.53238 0.88481
631 2.51459 4.93325 3.08370 2.52742 4.26438 3.48296 3.69905 3.68360 2.09514 3.25329 4.05636 3.03486 3.89270 2.83591 2.32390 1.80284 2.83445 3.13395 5.45471 4.12596 640 - -
2.68618 4.42225 2.77519 2.73123 3.46354 2.40513 3.72494 3.29354 2.67741 2.69355 4.24690 2.90347 2.73739 3.18146 2.89801 2.37887 2.77519 2.98518 4.58477 3.61503
0.01982 4.32691 5.04925 0.61958 0.77255 0.55748 0.85016
632 2.67574 4.92829 2.97029 2.46452 4.18966 2.88968 2.28985 3.63089 2.47874 3.21299 4.01744 2.61948 2.74557 2.83535 2.94146 2.69305 2.78699 3.28723 5.42735 3.59749 641 - -
2.68618 4.42225 2.77519 2.73123 3.46354 2.40513 3.72494 3.29354 2.67741 2.69355 4.24690 2.90347 2.73739 3.18146 2.89801 2.37887 2.77519 2.98518 4.58477 3.61503
0.07846 4.32691 2.77652 0.61958 0.77255 0.55748 0.85016
633 2.04691 4.46711 3.34467 2.95639 4.39180 1.66280 4.10841 3.82248 2.98710 3.45635 4.26687 3.28026 3.84758 3.31509 3.04372 1.68650 2.87313 3.33102 5.68364 4.42061 642 - -
2.68618 4.42225 2.77519 2.73123 3.46354 2.40513 3.72494 3.29354 2.67741 2.69355 4.24690 2.90347 2.73739 3.18146 2.89801 2.37887 2.77519 2.98518 4.58477 3.61503
0.02100 4.26945 4.99179 0.61958 0.77255 0.55488 0.85365
634 2.67735 4.80880 3.08325 2.61547 4.20430 1.79326 3.80256 3.61042 2.56891 3.22040 3.68897 3.09800 3.91402 2.07835 2.97851 2.73532 2.69194 3.26626 5.46331 4.14901 643 - -
2.68618 4.42225 2.77519 2.73123 3.46354 2.40513 3.72494 3.29354 2.67741 2.69355 4.24690 2.90347 2.73739 3.18146 2.89801 2.37887 2.77519 2.98518 4.58477 3.61503
0.01988 4.32401 5.04635 0.61958 0.77255 0.42709 1.05671
635 2.38766 4.89818 3.04944 2.39691 4.14612 2.66616 3.69445 3.56732 2.39466 2.96652 3.96770 3.01666 3.32758 2.19884 2.94553 2.69633 2.74458 3.23586 3.66846 4.05404 644 - -
2.68618 4.42225 2.77519 2.73123 3.46354 2.40513 3.72494 3.29354 2.67741 2.69355 4.24690 2.90347 2.73739 3.18146 2.89801 2.37887 2.77519 2.98518 4.58477 3.61503
0.05136 4.39592 3.27709 0.61958 0.77255 0.48576 0.95510

636 2.55590 4.81634 3.11703 2.55833 4.05061 2.82720 3.72438 3.45634 2.52372 3.07559 3.47459 3.06696 3.90657 2.20824 2.42468 2.56044 2.90791 2.48631 5.33452 4.01588 645 - -

2.68618 4.42225 2.77519 2.73123 3.46354 2.40513 3.72494 3.29354 2.67741 2.69355 4.24690 2.90347 2.73739 3.18146 2.89801 2.37887 2.77519 2.98518 4.58477 3.61503

0.07368 4.39956 2.83447 0.61958 0.77255 0.53354 0.88316

637 2.34042 4.71459 3.14423 2.58734 3.92549 3.21694 3.39667 3.05816 2.57087 2.96404 3.80296 3.08623 3.38893 2.21589 3.01314 2.72517 2.52548 2.55098 5.24897 3.94494 646 - -

2.68618 4.42225 2.77519 2.73123 3.46354 2.40513 3.72494 3.29354 2.67741 2.69355 4.24690 2.90347 2.73739 3.18146 2.89801 2.37887 2.77519 2.98518 4.58477 3.61503

0.01945 4.34533 5.06767 0.61958 0.77255 0.60850 0.78563

638 1.94402 4.77459 3.08917 2.35368 4.01543 2.94806 3.38418 3.42069 2.54920 2.64699 3.87536 3.05763 3.89193 2.89527 2.99982 2.44510 2.60221 3.11367 5.31383 3.99691 647 - -

2.68618 4.42225 2.77519 2.73123 3.46354 2.40513 3.72494 3.29354 2.67741 2.69355 4.24690 2.90347 2.73739 3.18146 2.89801 2.37887 2.77519 2.98518 4.58477 3.61503

0.01945 4.34533 5.06767 0.61958 0.77255 0.60850 0.78563

639 2.51286 4.73072 2.80966 2.58942 3.94098 3.51818 3.73123 3.32959 2.30819 2.66768 3.81520 3.08858 3.90825 2.91375 2.57501 2.63667 2.89354 2.05797 5.25857 3.95536 648 - -

2.68618 4.42225 2.77519 2.73123 3.46354 2.40513 3.72494 3.29354 2.67741 2.69355 4.24690 2.90347 2.73739 3.18146 2.89801 2.37887 2.77519 2.98518 4.58477 3.61503

0.01945 4.34533 5.06767 0.61958 0.77255 0.48388 0.95812

640 2.66462 3.83443 3.33217 2.76837 3.70795 3.42079 3.21080 3.08317 2.51123 2.13551 3.26742 3.22869 3.96732 2.87693 2.59029 2.70539 2.63553 2.61088 5.10180 3.83733 649 - -

2.68618 4.42225 2.77519 2.73123 3.46354 2.40513 3.72494 3.29354 2.67741 2.69355 4.24690 2.90347 2.73739 3.18146 2.89801 2.37887 2.77519 2.98518 4.58477 3.61503

0.07566 4.39956 2.80374 0.61958 0.77255 0.53354 0.88316

641 2.49718 4.50291 3.34163 2.77793 3.66488 3.40790 3.53797 2.92574 2.73980 2.41018 3.60011 3.23129 3.95735 2.92591 2.72079 2.65321 2.18027 2.34424 5.06642 3.80669 650 - -

2.68618 4.42225 2.77519 2.73123 3.46354 2.40513 3.72494 3.29354 2.67741 2.69355 4.24690 2.90347 2.73739 3.18146 2.89801 2.37887 2.77519 2.98518 4.58477 3.61503

0.01949 4.34339 5.06574 0.61958 0.77255 0.54775 0.86335

642 2.68639 5.13333 2.60432 2.37573 4.45538 2.43936 3.65495 3.92445 2.41098 3.29195 4.18935 2.22171 3.62854 2.51454 2.76544 2.50490 2.75897 3.51349 5.58696 4.18926 651 - -

2.68618 4.42225 2.77519 2.73123 3.46354 2.40513 3.72494 3.29354 2.67741 2.69355 4.24690 2.90347 2.73739 3.18146 2.89801 2.37887 2.77519 2.98518 4.58477 3.61503

0.01896 4.37049 5.09284 0.61958 0.77255 0.57491 0.82726

643 2.35461 4.82281 2.61486 2.45763 3.40130 3.49767 2.18156 3.46042 2.51967 2.97822 3.52147 3.03920 3.66385 2.86712 2.97797 2.70676 2.89337 3.14851 5.33152 4.00391 652 - -

2.68618 4.42225 2.77519 2.73123 3.46354 2.40513 3.72494 3.29354 2.67741 2.69355 4.24690 2.90347 2.73739 3.18146 2.89801 2.37887 2.77519 2.98518 4.58477 3.61503

0.26690 4.37049 1.50687 0.61958 0.77255 0.57491 0.82726

644 2.69684 5.04846 2.89983 2.40424 4.38830 3.06481 3.62271 3.82901 2.29196 3.34713 4.13454 2.60414 3.39215 2.00641 2.25081 2.68170 2.92787 3.44689 5.50187 4.15172 653 - -

2.68618 4.42225 2.77519 2.73123 3.46354 2.40513 3.72494 3.29354 2.67741 2.69355 4.24690 2.90347 2.73739 3.18146 2.89801 2.37887 2.77519 2.98518 4.58477 3.61503

0.02424 4.12783 4.85018 0.61958 0.77255 0.75776 0.63246

645 2.67317 5.04480 2.87542 1.85730 4.34365 3.42169 3.61989 3.79451 2.34013 3.32274 4.10473 2.91036 3.42138 2.14977 2.61847 2.65664 2.90291 3.41499 5.49057 3.51830 654 - -

2.68618 4.42225 2.77519 2.73123 3.46354 2.40513 3.72494 3.29354 2.67741 2.69355 4.24690 2.90347 2.73739 3.18146 2.89801 2.37887 2.77519 2.98518 4.58477 3.61503

0.02326 4.16851 4.89086 0.61958 0.77255 0.79915 0.59731

646 2.74056 4.31512 3.93599 3.38596 3.49623 3.77305 4.16328 2.36047 3.18787 2.21639 3.43292 3.69057 4.18556 3.55743 3.06441 3.08813 2.73523 1.31096 5.06603 3.85139 655 - -

2.68618 4.42225 2.77519 2.73123 3.46354 2.40513 3.72494 3.29354 2.67741 2.69355 4.24690 2.90347 2.73739 3.18146 2.89801 2.37887 2.77519 2.98518 4.58477 3.61503

0.02326 4.16851 4.89086 0.61958 0.77255 0.79915 0.59731

647 2.35083 4.70783 3.08164 1.88050 3.92913 3.46758 3.68845 3.29173 2.50061 2.94641 3.15908 3.03757 3.86343 2.86523 2.74920 2.69154 2.85692 2.69911 5.24219 3.93557 656 - -

2.68618 4.42225 2.77519 2.73123 3.46354 2.40513 3.72494 3.29354 2.67741 2.69355 4.24690 2.90347 2.73739 3.18146 2.89801 2.37887 2.77519 2.98518 4.58477 3.61503

0.02326 4.16851 4.89086 0.61958 0.77255 0.33025 1.26849

648 2.56789 4.26248 3.78980 3.21566 3.38122 3.15746 3.45577 1.99576 3.14135 2.27954 3.37252 3.54695 3.26664 3.41363 3.25663 2.78888 2.90032 2.05406 4.86079 3.44907 657 - -

2.68618 4.42225 2.77519 2.73123 3.46354 2.40513 3.72494 3.29354 2.67741 2.69355 4.24690 2.90347 2.73739 3.18146 2.89801 2.37887 2.77519 2.98518 4.58477 3.61503

0.01785 4.43070 5.15305 0.61958 0.77255 0.48576 0.95510

649 2.57493 5.16656 2.46787 2.43708 4.49981 3.49530 3.43693 3.95976 1.82725 3.45611 4.21727 2.97349 3.89004 2.77659 1.98977 2.70496 2.95939 3.25884 5.59679 4.21898 658 - -

2.68618 4.42225 2.77519 2.73123 3.46354 2.40513 3.72494 3.29354 2.67741 2.69355 4.24690 2.90347 2.73739 3.18146 2.89801 2.37887 2.77519 2.98518 4.58477 3.61503

0.01785 4.43070 5.15305 0.61958 0.77255 0.48576 0.95510

650 2.67369 4.27873 3.15880 2.60157 3.73961 2.49087 3.75432 3.39808 2.58026 3.03346 3.62934 1.93382 3.92204 2.93033 2.63297 2.74145 2.91111 2.90474 5.30766 3.99711 659 - -

2.68618 4.42225 2.77519 2.73123 3.46354 2.40513 3.72494 3.29354 2.67741 2.69355 4.24690 2.90347 2.73739 3.18146 2.89801 2.37887 2.77519 2.98518 4.58477 3.61503

0.01785 4.43070 5.15305 0.61958 0.77255 0.48576 0.95510

651 2.56739 3.98348 3.20953 2.64627 3.94393 2.04096 3.76687 3.33703 2.49745 2.98104 3.82455 3.13738 3.94314 2.81208 2.33202 2.67142 2.92282 3.05207 3.83780 3.97150 660 - -

2.68618 4.42225 2.77519 2.73123 3.46354 2.40513 3.72494 3.29354 2.67741 2.69355 4.24690 2.90347 2.73739 3.18146 2.89801 2.37887 2.77519 2.98518 4.58477 3.61503

0.01785 4.43070 5.15305 0.61958 0.77255 0.48576 0.95510

652 2.70724 4.53320 3.43556 2.76230 3.69118 3.63444 3.51509 1.80739 2.73622 2.60059 3.62850 3.30152 4.01427 2.53131 2.61435 2.87475 2.93535 2.63701 5.08989 3.84060 661 - -

2.68618 4.42225 2.77519 2.73123 3.46354 2.40513 3.72494 3.29354 2.67741 2.69355 4.24690 2.90347 2.73739 3.18146 2.89801 2.37887 2.77519 2.98518 4.58477 3.61503

0.01785 4.43070 5.15305 0.61958 0.77255 0.48576 0.95510

653 2.52689 4.15047 4.10437 3.51837 2.19300 3.44343 3.24497 2.05718 3.20351 2.28462 2.97422 3.73600 4.13486 3.62629 3.59017 2.84571 2.89891 2.20435 4.76089 3.56744 662 - -

2.68618 4.42225 2.77519 2.73123 3.46354 2.40513 3.72494 3.29354 2.67741 2.69355 4.24690 2.90347 2.73739 3.18146 2.89801 2.37887 2.77519 2.98518 4.58477 3.61503

0.01785 4.43070 5.15305 0.61958 0.77255 0.48576 0.95510

654 2.84109 5.35152 2.06991 1.72740 4.66862 3.45689 3.75784 3.72989 2.56470 3.64369 4.41343 2.92055 3.92856 1.81811 3.07454 2.78383 3.08537 3.72946 5.78837 4.35860 663 - -

2.68600 4.42229 2.77523 2.73127 3.46358 2.40516 3.72478 3.29358 2.67744 2.69359 4.24693 2.90329 2.73743 3.18150 2.89804 2.37891 2.77523 2.98508 4.58481 3.61507

0.69602 1.71859 1.13285 0.21815 1.62967 0.48576 0.95510

655 2.47852 4.65620 3.17621 2.60046 3.89293 3.47855 3.67284 3.24800 2.26743 2.67677 3.76058 3.07483 3.87074 2.85991 2.10116 2.71912 2.55556 2.75321 5.18884 3.91154 665 - -

2.68618 4.42225 2.77519 2.73123 3.46354 2.40513 3.72494 3.29354 2.67741 2.69355 4.24690 2.90347 2.73739 3.18146 2.89801 2.37887 2.77519 2.98518 4.58477 3.61503

0.02621 4.05042 4.77277 0.61958 0.77255 0.89442 0.52568

656 2.53329 3.58789 4.05398 3.48048 2.89049 3.73578 4.08792 2.33192 3.37334 1.73554 3.20166 3.71526 4.11513 3.60835 3.58238 3.03289 2.40542 2.03389 4.79904 3.60483 666 - -

2.68618 4.42225 2.77519 2.73123 3.46354 2.40513 3.72494 3.29354 2.67741 2.69355 4.24690 2.90347 2.73739 3.18146 2.89801 2.37887 2.77519 2.98518 4.58477 3.61503

0.02621 4.05042 4.77277 0.61958 0.77255 0.89442 0.52568

657 2.57818 4.22455 3.98673 3.43123 1.84903 3.75839 4.01394 2.48011 3.31793 1.96086 3.02072 3.68674 4.13591 3.56815 3.54526 2.81842 2.94735 2.34311 4.67879 3.36098 667 - -

2.68618 4.42225 2.77519 2.73123 3.46354 2.40513 3.72494 3.29354 2.67741 2.69355 4.24690 2.90347 2.73739 3.18146 2.89801 2.37887 2.77519 2.98518 4.58477 3.61503

0.02621 4.05042 4.77277 0.61958 0.77255 0.89442 0.52568

658 2.31328 4.67482 3.06655 2.53814 3.95182 3.41554 3.67645 3.33430 2.45206 2.59275 3.81722 2.30507 3.83681 2.85428 2.54298 2.66766 2.65413 3.03150 5.24456 3.94470 668 - -

2.68618 4.42225 2.77519 2.73123 3.46354 2.40513 3.72494 3.29354 2.67741 2.69355 4.24690 2.90347 2.73739 3.18146 2.89801 2.37887 2.77519 2.98518 4.58477 3.61503

0.02621 4.05042 4.77277 0.61958 0.77255 0.89442 0.52568

659 2.22540 4.80692 2.77250 2.41654 4.27883 2.93136 3.72137 3.71790 2.54411 3.29802 4.10675 2.06054 3.79626 2.60552 3.00287 2.30480 2.88720 3.32675 5.50884 4.15935 669 - -

2.68604 4.42229 2.77514 2.73127 3.46337 2.40516 3.72498 3.29357 2.67744 2.69358 4.24693 2.90350 2.73724 3.18150 2.89804 2.37890 2.77523 2.98522 4.58480 3.61507
0.27807 2.35540 1.91123 0.85139 0.55656 0.29531 1.36376
660 2.77990 4.31333 4.58193 4.01869 3.45163 3.72658 4.54245 1.86868 3.88901 1.56720 3.36392 4.19372 4.48892 4.10644 4.04558 3.46794 3.17252 1.37299 5.13268 3.94771 674 - -
2.68618 4.42225 2.77519 2.73123 3.46354 2.40513 3.72494 3.29354 2.67741 2.69355 4.24690 2.90347 2.73739 3.18146 2.89801 2.37887 2.77519 2.98518 4.58477 3.61503
0.01975 4.33052 5.05286 0.61958 0.77255 0.62738 0.76355
661 2.65188 4.69123 3.18823 2.63835 3.53269 3.50249 3.76320 3.31799 1.98157 2.97658 3.82358 3.12174 3.91455 2.95352 3.00586 2.46915 1.92121 2.85952 5.26533 3.96874 675 - -
2.68618 4.42225 2.77519 2.73123 3.46354 2.40513 3.72494 3.29354 2.67741 2.69355 4.24690 2.90347 2.73739 3.18146 2.89801 2.37887 2.77519 2.98518 4.58477 3.61503
0.01975 4.33052 5.05286 0.61958 0.77255 0.62738 0.76355
662 2.95072 4.33284 4.65332 4.06179 2.40773 4.13227 4.44611 2.11576 3.91287 1.23379 2.92347 4.20005 3.68748 4.05064 4.00535 3.44392 3.17839 2.07654 4.92648 3.78510 676 - -
2.68618 4.42225 2.77519 2.73123 3.46354 2.40513 3.72494 3.29354 2.67741 2.69355 4.24690 2.90347 2.73739 3.18146 2.89801 2.37887 2.77519 2.98518 4.58477 3.61503
0.01975 4.33052 5.05286 0.61958 0.77255 0.62738 0.76355
663 2.63205 4.39924 3.54792 2.99480 3.62648 3.56875 3.94821 2.70169 2.94234 2.65798 3.02984 3.38440 4.00551 3.25659 3.30019 1.69571 2.25218 2.51684 5.07682 3.83905 677 - -
2.68618 4.42225 2.77519 2.73123 3.46354 2.40513 3.72494 3.29354 2.67741 2.69355 4.24690 2.90347 2.73739 3.18146 2.89801 2.37887 2.77519 2.98518 4.58477 3.61503
0.01975 4.33052 5.05286 0.61958 0.77255 0.62738 0.76355
664 2.65716 4.54968 3.30203 2.74624 3.72782 3.56505 3.80244 2.44736 2.44779 2.66593 3.65114 2.19426 3.95438 3.04405 3.10077 2.55125 2.89106 2.39595 5.11697 3.84922 678 - -
2.68618 4.42225 2.77519 2.73123 3.46354 2.40513 3.72494 3.29354 2.67741 2.69355 4.24690 2.90347 2.73739 3.18146 2.89801 2.37887 2.77519 2.98518 4.58477 3.61503
0.01975 4.33052 5.05286 0.61958 0.77255 0.62738 0.76355
665 2.88862 4.46355 4.03618 3.48796 3.42818 3.92797 4.28616 2.24685 3.37880 1.62834 3.33429 3.81780 1.80499 3.67331 3.65590 3.22984 3.14186 2.29595 5.11648 3.91787 679 - -
2.68618 4.42225 2.77519 2.73123 3.46354 2.40513 3.72494 3.29354 2.67741 2.69355 4.24690 2.90347 2.73739 3.18146 2.89801 2.37887 2.77519 2.98518 4.58477 3.61503
0.01975 4.33052 5.05286 0.61958 0.77255 0.40642 1.09671
666 2.69444 2.69500 2.14526 2.55988 4.10523 2.51012 3.76309 3.51425 2.58679 2.79221 3.96000 3.07079 3.92108 2.65322 3.04374 2.74274 2.93889 3.19696 5.39248 4.06582 680 - -
2.68618 4.42225 2.77519 2.73123 3.46354 2.40513 3.72494 3.29354 2.67741 2.69355 4.24690 2.90347 2.73739 3.18146 2.89801 2.37887 2.77519 2.98518 4.58477 3.61503
0.01785 4.43070 5.15305 0.61958 0.77255 0.48576 0.95510
667 2.16832 3.32752 3.86481 3.32529 3.61211 1.61015 4.13553 2.68418 3.24974 2.25826 3.58614 3.59828 4.06896 3.53119 3.54053 2.89393 2.93314 2.62359 5.09125 3.87954 681 - -
2.68618 4.42225 2.77519 2.73123 3.46354 2.40513 3.72494 3.29354 2.67741 2.69355 4.24690 2.90347 2.73739 3.18146 2.89801 2.37887 2.77519 2.98518 4.58477 3.61503
0.01785 4.43070 5.15305 0.61958 0.77255 0.48576 0.95510
668 2.80181 4.20128 4.52442 3.93091 2.33913 3.95849 3.29267 1.77194 3.76759 1.76476 3.19970 4.03279 4.30585 3.93145 3.85787 3.26411 3.03301 1.95682 4.78129 3.57384 682 - -
2.68618 4.42225 2.77519 2.73123 3.46354 2.40513 3.72494 3.29354 2.67741 2.69355 4.24690 2.90347 2.73739 3.18146 2.89801 2.37887 2.77519 2.98518 4.58477 3.61503
0.01785 4.43070 5.15305 0.61958 0.77255 0.48576 0.95510
669 2.43452 4.42228 4.90432 4.34726 3.58564 4.41366 4.83877 1.62364 4.22400 1.95359 3.06980 4.49394 4.72056 4.42018 4.35349 3.75160 3.34236 1.17698 5.34923 4.16947 683 - -
2.68618 4.42225 2.77519 2.73123 3.46354 2.40513 3.72494 3.29354 2.67741 2.69355 4.24690 2.90347 2.73739 3.18146 2.89801 2.37887 2.77519 2.98518 4.58477 3.61503
0.01785 4.43070 5.15305 0.61958 0.77255 0.48576 0.95510
670 2.09351 4.41300 3.73738 3.35630 4.45689 2.82655 4.39779 3.86923 3.37251 3.54541 4.36619 3.53541 3.28821 3.65906 3.70775 2.40043 0.99249 3.35765 5.80245 4.58846 684 - -
2.68618 4.42225 2.77519 2.73123 3.46354 2.40513 3.72494 3.29354 2.67741 2.69355 4.24690 2.90347 2.73739 3.18146 2.89801 2.37887 2.77519 2.98518 4.58477 3.61503
0.01785 4.43070 5.15305 0.61958 0.77255 0.48576 0.95510

671 2.67106 3.72999 3.85486 3.28031 3.37392 2.14410 4.01291 2.26712 3.19543 2.18012 3.36670 3.59046 4.09670 3.46378 3.01948 2.79877 2.90772 2.18084 4.86090 3.65316 685 - -
2.68618 4.42225 2.77519 2.73123 3.46354 2.40513 3.72494 3.29354 2.67741 2.69355 4.24690 2.90347 2.73739 3.18146 2.89801 2.37887 2.77519 2.98518 4.58477 3.61503
0.01785 4.43070 5.15305 0.61958 0.77255 0.48576 0.95510

672 3.15670 4.46454 5.00955 4.45067 3.17155 4.48124 4.89764 1.71736 4.32069 1.62806 3.38364 4.58083 4.76836 4.48566 4.42317 3.82432 3.05051 1.16271 5.34714 4.18523 686 - -
2.68758 4.42385 2.77364 2.73095 3.46471 2.40494 3.72654 3.29351 2.67800 2.69297 4.24509 2.89987 2.73879 3.18246 2.89690 2.38032 2.77679 2.98558 4.57915 3.61115
0.40650 1.11402 5.15305 2.90370 0.05638 0.48576 0.95510

673 3.42423 4.67716 5.31961 4.78628 3.56427 4.85112 5.32067 1.72370 4.67201 1.19663 3.34174 4.96139 5.05650 4.79999 4.77152 4.23111 3.66431 1.22546 5.61812 4.50707 709 - -
2.68618 4.42225 2.77519 2.73123 3.46354 2.40513 3.72494 3.29354 2.67741 2.69355 4.24690 2.90347 2.73739 3.18146 2.89801 2.37887 2.77519 2.98518 4.58477 3.61503
0.01785 4.43070 5.15305 0.61958 0.77255 0.48576 0.95510

674 3.35805 4.64031 5.19885 4.64479 3.05397 4.70867 5.09455 2.00179 4.52132 1.27044 3.25538 4.80497 4.93170 4.62720 4.60579 4.06609 3.59168 1.15571 5.41866 4.28977 710 - -
2.68618 4.42225 2.77519 2.73123 3.46354 2.40513 3.72494 3.29354 2.67741 2.69355 4.24690 2.90347 2.73739 3.18146 2.89801 2.37887 2.77519 2.98518 4.58477 3.61503
0.01785 4.43070 5.15305 0.61958 0.77255 0.48576 0.95510

675 2.92183 4.45463 4.09874 3.52461 2.77218 3.93858 3.98716 2.84999 3.30087 1.88313 3.46826 3.77820 4.29468 3.62714 2.44605 3.22508 3.14902 2.69988 1.79012 3.13691 711 - -
2.68618 4.42225 2.77519 2.73123 3.46354 2.40513 3.72494 3.29354 2.67741 2.69355 4.24690 2.90347 2.73739 3.18146 2.89801 2.37887 2.77519 2.98518 4.58477 3.61503
0.01785 4.43070 5.15305 0.61958 0.77255 0.48576 0.95510

676 2.51110 4.71783 3.18608 2.64414 3.57125 2.39141 3.78833 3.37008 2.63857 3.01418 3.85355 2.77266 3.93007 2.97721 3.07913 1.90151 2.74536 3.07260 5.29684 3.34832 712 - -
2.68618 4.42225 2.77519 2.73123 3.46354 2.40513 3.72494 3.29354 2.67741 2.69355 4.24690 2.90347 2.73739 3.18146 2.89801 2.37887 2.77519 2.98518 4.58477 3.61503
0.01785 4.43070 5.15305 0.61958 0.77255 0.48576 0.95510

677 2.69969 4.68292 3.26325 2.70508 2.99815 3.57353 3.79818 3.25404 1.84671 2.74769 3.77939 3.18371 3.60118 3.00698 3.04278 2.80362 2.01815 2.98549 5.22482 3.93703 713 - -
2.68618 4.42225 2.77519 2.73123 3.46354 2.40513 3.72494 3.29354 2.67741 2.69355 4.24690 2.90347 2.73739 3.18146 2.89801 2.37887 2.77519 2.98518 4.58477 3.61503
0.04652 4.43070 3.39477 0.61958 0.77255 0.48576 0.95510

678 2.40468 4.18515 4.20780 3.62405 3.00948 3.84017 4.15681 1.99320 3.50443 1.70917 3.26622 3.82992 2.88883 3.72229 3.68270 3.13146 2.78751 2.16548 4.82479 3.63658 714 - -
2.68618 4.42225 2.77519 2.73123 3.46354 2.40513 3.72494 3.29354 2.67741 2.69355 4.24690 2.90347 2.73739 3.18146 2.89801 2.37887 2.77519 2.98518 4.58477 3.61503
0.12358 4.40254 2.26335 0.61958 0.77255 0.52912 0.88946

679 2.86076 4.80053 3.43211 3.17603 4.63257 0.78569 4.29964 4.15369 3.03096 3.75037 4.63043 3.52033 4.10369 3.54078 2.48978 2.98520 3.27268 3.69661 5.79676 4.62179 715 - -
2.68618 4.42225 2.77519 2.73123 3.46354 2.40513 3.72494 3.29354 2.67741 2.69355 4.24690 2.90347 2.73739 3.18146 2.89801 2.37887 2.77519 2.98518 4.58477 3.61503
0.02038 4.29934 5.02169 0.61958 0.77255 0.38802 1.13445

680 2.47262 4.87433 3.07625 1.93901 4.11625 3.50874 3.71890 3.18997 2.51849 2.99804 3.95065 3.04427 3.90228 2.63058 2.97888 2.05020 2.91396 2.79797 5.38311 4.04979 716 - -
2.68618 4.42225 2.77519 2.73123 3.46354 2.40513 3.72494 3.29354 2.67741 2.69355 4.24690 2.90347 2.73739 3.18146 2.89801 2.37887 2.77519 2.98518 4.58477 3.61503
0.27903 4.43070 1.46285 0.61958 0.77255 0.48576 0.95510

681 2.57454 4.52894 3.19741 2.66974 3.03268 2.41984 3.76782 3.17394 2.66348 2.84508 3.70264 2.49630 3.42980 2.99261 3.08134 2.56123 2.83593 2.59054 5.15224 3.87053 717 - -
2.68618 4.42225 2.77519 2.73123 3.46354 2.40513 3.72494 3.29354 2.67741 2.69355 4.24690 2.90347 2.73739 3.18146 2.89801 2.37887 2.77519 2.98518 4.58477 3.61503
0.02311 4.17479 4.89713 0.61958 0.77255 0.75111 0.63836

682 2.62534 4.83668 2.80833 2.44579 3.61348 3.43759 3.65293 3.49990 2.26746 2.64642 3.91266 2.31969 3.83678 2.80275 2.91370 2.24589 2.85870 3.17322 5.33728 3.99721 718 - -
2.68618 4.42225 2.77519 2.73123 3.46354 2.40513 3.72494 3.29354 2.67741 2.69355 4.24690 2.90347 2.73739 3.18146 2.89801 2.37887 2.77519 2.98518 4.58477 3.61503

0.17190 4.19611 1.94571 0.61958 0.77255 0.77371 0.61859

683 2.65275 4.23175 3.82809 3.27586 2.43868 3.67617 3.97205 2.54063 3.18819 1.90241 3.27486 3.57387 4.07212 3.45595 3.45599 2.23196 2.90317 2.24477 4.74309 3.44441 719 - -

2.68618 4.42225 2.77519 2.73123 3.46354 2.40513 3.72494 3.29354 2.67741 2.69355 4.24690 2.90347 2.73739 3.18146 2.89801 2.37887 2.77519 2.98518 4.58477 3.61503

0.02621 4.05042 4.77277 0.61958 0.77255 0.36973 1.17417

684 2.66473 4.82306 3.11168 2.55616 4.09581 2.59144 3.72715 3.21579 2.51525 2.97254 3.93646 3.06172 2.82038 2.88505 2.11381 2.57444 2.65802 3.18362 5.36567 4.04404 720 - -

2.68646 4.42253 2.77547 2.73139 3.46382 2.40438 3.72522 3.29382 2.67769 2.69373 4.24661 2.90365 2.73728 3.18152 2.89804 2.37890 2.77443 2.98508 4.58505 3.61512

1.02714 0.50353 3.28141 0.74064 0.64781 0.44031 1.03236

685 2.66470 4.46209 3.43194 2.86962 3.61696 3.60989 3.85829 2.12002 2.73343 2.68263 3.05524 3.30343 2.93727 2.90908 3.20273 2.21419 2.70857 2.74332 5.03812 3.79010 729 - -

2.68618 4.42225 2.77519 2.73123 3.46354 2.40513 3.72494 3.29354 2.67741 2.69355 4.24690 2.90347 2.73739 3.18146 2.89801 2.37887 2.77519 2.98518 4.58477 3.61503

0.32794 4.40035 1.31931 0.61958 0.77255 0.53238 0.88481

686 2.64331 4.23441 3.40054 3.19772 2.03519 3.66785 3.96105 2.26483 3.13507 2.32877 3.31614 3.52505 4.05725 3.40777 3.42131 2.95191 2.43903 2.35936 4.80130 3.53438 730 - -

2.68618 4.42225 2.77519 2.73123 3.46354 2.40513 3.72494 3.29354 2.67741 2.69355 4.24690 2.90347 2.73739 3.18146 2.89801 2.37887 2.77519 2.98518 4.58477 3.61503

0.02499 4.09740 4.81975 0.61958 0.77255 0.85892 0.55098

687 2.64602 5.25431 0.97320 2.28887 4.71248 3.25855 3.93631 4.15137 2.99338 3.78853 4.70164 2.84884 3.91810 3.13188 3.55974 2.88876 3.28074 3.75180 5.96555 4.51781 731 - -

2.68618 4.42225 2.77519 2.73123 3.46354 2.40513 3.72494 3.29354 2.67741 2.69355 4.24690 2.90347 2.73739 3.18146 2.89801 2.37887 2.77519 2.98518 4.58477 3.61503

0.02499 4.09740 4.81975 0.61958 0.77255 0.49374 0.94248

688 2.54682 4.52511 2.80767 2.73403 3.69138 3.55288 3.79087 3.06030 2.71369 2.75131 3.08515 2.66522 3.94070 3.03899 3.12100 2.70484 2.87317 1.98581 5.08594 3.24996 732 - -

2.68618 4.42225 2.77519 2.73123 3.46354 2.40513 3.72494 3.29354 2.67741 2.69355 4.24690 2.90347 2.73739 3.18146 2.89801 2.37887 2.77519 2.98518 4.58477 3.61503

0.02015 4.31022 5.03256 0.61958 0.77255 0.65224 0.73580

689 2.23498 4.30145 3.64325 3.52923 3.42464 3.89725 4.24289 1.62651 3.44489 1.73657 3.36725 3.81715 4.26949 3.70657 3.69264 3.19478 3.04030 2.16519 5.01821 3.81592 733 - -

2.68618 4.42225 2.77519 2.73123 3.46354 2.40513 3.72494 3.29354 2.67741 2.69355 4.24690 2.90347 2.73739 3.18146 2.89801 2.37887 2.77519 2.98518 4.58477 3.61503

0.02015 4.31022 5.03256 0.61958 0.77255 0.65224 0.73580

690 2.48722 5.01118 2.25955 2.35022 4.29867 3.44782 3.65743 3.51025 1.85347 3.28998 4.07550 2.94624 3.85103 2.78151 2.89522 2.66423 2.90192 2.77409 5.48683 4.11739 734 - -

2.68618 4.42225 2.77519 2.73123 3.46354 2.40513 3.72494 3.29354 2.67741 2.69355 4.24690 2.90347 2.73739 3.18146 2.89801 2.37887 2.77519 2.98518 4.58477 3.61503

0.02015 4.31022 5.03256 0.61958 0.77255 0.65224 0.73580

691 2.28029 4.72964 3.08445 2.35116 4.12904 1.97429 3.79862 3.53465 2.63069 2.97859 3.98572 3.08432 3.88136 2.97014 3.07593 2.18775 2.90082 3.03738 5.41775 4.10256 735 - -

2.68618 4.42225 2.77519 2.73123 3.46354 2.40513 3.72494 3.29354 2.67741 2.69355 4.24690 2.90347 2.73739 3.18146 2.89801 2.37887 2.77519 2.98518 4.58477 3.61503

0.02015 4.31022 5.03256 0.61958 0.77255 0.65224 0.73580

692 2.57233 4.27313 3.79855 3.04125 3.07219 3.72697 4.02149 2.40972 3.15818 2.35591 3.37888 3.56821 4.10379 3.43697 3.45184 2.99588 1.74694 2.07896 4.91463 3.70030 736 - -

2.68618 4.42225 2.77519 2.73123 3.46354 2.40513 3.72494 3.29354 2.67741 2.69355 4.24690 2.90347 2.73739 3.18146 2.89801 2.37887 2.77519 2.98518 4.58477 3.61503

0.02015 4.31022 5.03256 0.61958 0.77255 0.52356 0.89748

693 2.80369 5.25513 2.88001 1.61697 4.60373 3.25947 3.69231 4.06419 2.17827 3.54157 4.31197 2.48408 3.91625 2.80429 2.16227 2.76255 3.03170 3.65144 5.66514 4.28900 737 - -

2.68618 4.42225 2.77519 2.73123 3.46354 2.40513 3.72494 3.29354 2.67741 2.69355 4.24690 2.90347 2.73739 3.18146 2.89801 2.37887 2.77519 2.98518 4.58477 3.61503

0.01901 4.36822 5.09057 0.61958 0.77255 0.57803 0.82327

694 2.47276 4.40119 3.49222 2.83162 1.98416 3.62593 3.61797 2.61887 2.88478 2.61276 3.50478 3.34956 4.00915 2.59017 3.24058 2.68338 2.89419 2.67716 4.96919 3.72305 738 - -

2.68618 4.42225 2.77519 2.73123 3.46354 2.40513 3.72494 3.29354 2.67741 2.69355 4.24690 2.90347 2.73739 3.18146 2.89801 2.37887 2.77519 2.98518 4.58477 3.61503
0.05745 4.36822 3.14285 0.61958 0.77255 0.57803 0.82327
695 2.73516 5.17022 2.64706 2.41542 4.50867 3.47468 3.27222 3.96733 2.08377 3.45743 4.22285 2.75653 3.87742 2.76171 1.77867 2.46638 2.96171 3.55998 5.59162 4.21762 739 - -
2.68618 4.42225 2.77519 2.73123 3.46354 2.40513 3.72494 3.29354 2.67741 2.69355 4.24690 2.90347 2.73739 3.18146 2.89801 2.37887 2.77519 2.98518 4.58477 3.61503
0.01975 4.33052 5.05286 0.61958 0.77255 0.62738 0.76355
696 2.87677 4.41390 4.90792 4.35498 2.46184 4.39572 4.80793 1.19649 4.22723 2.12797 3.35205 4.49028 4.69707 4.40159 4.34123 3.73946 3.33632 1.57188 5.27096 4.08118 740 - -
2.68618 4.42225 2.77519 2.73123 3.46354 2.40513 3.72494 3.29354 2.67741 2.69355 4.24690 2.90347 2.73739 3.18146 2.89801 2.37887 2.77519 2.98518 4.58477 3.61503
0.01975 4.33052 5.05286 0.61958 0.77255 0.62738 0.76355
697 2.73214 4.20226 4.15068 3.24181 3.12902 3.84862 3.79439 1.53545 3.45774 2.31091 3.29595 3.81015 4.21239 3.70009 3.65583 3.14118 2.96680 1.72367 4.85322 3.64768 741 - -
2.68618 4.42225 2.77519 2.73123 3.46354 2.40513 3.72494 3.29354 2.67741 2.69355 4.24690 2.90347 2.73739 3.18146 2.89801 2.37887 2.77519 2.98518 4.58477 3.61503
0.19515 4.33052 1.80709 0.61958 0.77255 0.62738 0.76355
698 2.76576 4.52597 3.46636 2.97497 2.74047 3.64919 3.73108 3.08941 2.94551 2.73584 3.67049 2.80916 4.05800 3.24786 3.29761 2.39549 3.01214 2.85847 4.51789 1.71986 742 - -
2.68618 4.42225 2.77519 2.73123 3.46354 2.40513 3.72494 3.29354 2.67741 2.69355 4.24690 2.90347 2.73739 3.18146 2.89801 2.37887 2.77519 2.98518 4.58477 3.61503
0.04772 4.15885 3.47455 0.61958 0.77255 0.76457 0.62649
699 2.38231 4.85802 2.88284 2.47640 4.30147 3.35378 3.73316 3.73158 2.50004 3.30854 4.11796 2.76838 3.84396 1.72254 2.92927 2.34318 2.78314 3.35109 5.51185 4.17315 743 - -
2.68618 4.42225 2.77519 2.73123 3.46354 2.40513 3.72494 3.29354 2.67741 2.69355 4.24690 2.90347 2.73739 3.18146 2.89801 2.37887 2.77519 2.98518 4.58477 3.61503
0.02351 4.15778 4.88013 0.61958 0.77255 0.80868 0.58959
700 2.69965 5.11862 1.86571 2.20890 4.43712 3.37127 3.65365 3.90026 2.44553 3.42270 4.20278 2.85896 3.82571 2.77794 2.54134 2.37478 2.60991 3.50161 5.59219 4.19303 744 - -
2.68618 4.42225 2.77519 2.73123 3.46354 2.40513 3.72494 3.29354 2.67741 2.69355 4.24690 2.90347 2.73739 3.18146 2.89801 2.37887 2.77519 2.98518 4.58477 3.61503
0.02351 4.15778 4.88013 0.61958 0.77255 0.80868 0.58959
701 2.72615 5.11638 2.76275 1.57152 4.44032 3.40599 3.64978 3.89116 2.23051 3.40870 4.19603 2.89490 3.44156 2.77459 2.63685 2.42242 2.96268 3.50250 5.56869 4.19435 745 - -
2.68618 4.42225 2.77519 2.73123 3.46354 2.40513 3.72494 3.29354 2.67741 2.69355 4.24690 2.90347 2.73739 3.18146 2.89801 2.37887 2.77519 2.98518 4.58477 3.61503
0.02351 4.15778 4.88013 0.61958 0.77255 0.49223 0.94485
702 2.70131 5.19173 2.42943 1.98230 4.52248 3.42764 3.44159 4.00232 2.22769 3.48905 4.24027 2.07553 3.84548 2.75450 2.70428 2.66127 2.93567 3.31148 5.62794 4.21854 746 - -
2.68624 4.42241 2.77512 2.73089 3.46370 2.40520 3.72436 3.29293 2.67757 2.69371 4.24706 2.90349 2.73727 3.18162 2.89817 2.37903 2.77494 2.98534 4.58493 3.61519
0.32089 1.31638 5.05286 1.00200 0.45752 0.62738 0.76355
703 2.71047 5.04131 3.03005 2.46756 4.34424 3.49227 2.47638 3.78075 2.34581 3.31738 4.10426 2.99105 3.27279 2.20166 2.24791 2.70564 2.77319 3.18287 5.49077 4.14239 751 - -
2.68618 4.42225 2.77519 2.73123 3.46354 2.40513 3.72494 3.29354 2.67741 2.69355 4.24690 2.90347 2.73739 3.18146 2.89801 2.37887 2.77519 2.98518 4.58477 3.61503
0.01975 4.33052 5.05286 0.61958 0.77255 0.54104 0.87261
704 1.67556 4.39468 4.55601 4.00509 3.58069 4.20883 4.64576 1.58548 3.90251 2.09337 3.46443 4.22863 4.56126 4.14462 4.10693 3.53611 3.24531 1.64594 5.29543 4.10174 752 - -
2.68618 4.42225 2.77519 2.73123 3.46354 2.40513 3.72494 3.29354 2.67741 2.69355 4.24690 2.90347 2.73739 3.18146 2.89801 2.37887 2.77519 2.98518 4.58477 3.61503
0.01901 4.36822 5.09057 0.61958 0.77255 0.57803 0.82327
705 2.72079 4.73514 3.16246 1.68787 3.92139 3.56074 3.81416 3.21592 2.65689 2.12026 3.80573 3.15695 3.45644 3.01088 3.08008 2.81332 2.96006 2.64734 5.29304 3.99771 753 - -
2.68618 4.42225 2.77519 2.73123 3.46354 2.40513 3.72494 3.29354 2.67741 2.69355 4.24690 2.90347 2.73739 3.18146 2.89801 2.37887 2.77519 2.98518 4.58477 3.61503
0.04670 4.36822 3.41262 0.61958 0.77255 0.57803 0.82327

706 2.15260 4.26256 3.84691 3.11852 3.39783 3.74292 4.03263 2.34886 3.17860 2.21524 3.37360 3.59800 4.11707 3.46467 3.04661 3.01451 2.92468 1.65458 4.90095 3.69073 754 - -
2.68618 4.42225 2.77519 2.73123 3.46354 2.40513 3.72494 3.29354 2.67741 2.69355 4.24690 2.90347 2.73739 3.18146 2.89801 2.37887 2.77519 2.98518 4.58477 3.61503
0.01954 4.34106 5.06340 0.61958 0.77255 0.53747 0.87761
707 2.61444 5.14721 2.70332 1.76036 4.58822 3.41057 3.84348 4.04926 2.68771 3.59402 4.39560 2.72384 3.93998 2.98862 3.17808 1.47862 3.09873 3.63626 5.77599 4.38170 755 - -
2.68618 4.42225 2.77519 2.73123 3.46354 2.40513 3.72494 3.29354 2.67741 2.69355 4.24690 2.90347 2.73739 3.18146 2.89801 2.37887 2.77519 2.98518 4.58477 3.61503
0.01890 4.37405 5.09640 0.61958 0.77255 0.57001 0.83361
708 2.70984 4.18127 3.63540 3.57525 1.81412 3.81192 3.74151 2.01963 3.45656 2.18436 3.26289 3.78308 4.17661 3.67502 3.63874 3.10066 2.94208 2.18115 4.72979 3.48465 756 - -
2.68618 4.42225 2.77519 2.73123 3.46354 2.40513 3.72494 3.29354 2.67741 2.69355 4.24690 2.90347 2.73739 3.18146 2.89801 2.37887 2.77519 2.98518 4.58477 3.61503
0.01890 4.37405 5.09640 0.61958 0.77255 0.57001 0.83361
709 2.51405 4.70840 3.17768 2.61990 3.92166 3.52603 3.53094 3.31519 2.58405 2.96078 3.80343 3.11288 3.31359 2.84055 2.39118 2.45362 2.60347 2.15490 5.24864 3.95103 757 - -
2.68618 4.42225 2.77519 2.73123 3.46354 2.40513 3.72494 3.29354 2.67741 2.69355 4.24690 2.90347 2.73739 3.18146 2.89801 2.37887 2.77519 2.98518 4.58477 3.61503
0.01890 4.37405 5.09640 0.61958 0.77255 0.57001 0.83361
710 2.68510 4.89058 2.95300 2.56677 4.26414 3.46669 3.75430 3.68153 2.49566 3.26865 4.07653 3.06624 3.90604 2.89952 1.88769 1.80569 2.82995 3.04652 5.48256 4.15371 758 - -
2.68618 4.42225 2.77519 2.73123 3.46354 2.40513 3.72494 3.29354 2.67741 2.69355 4.24690 2.90347 2.73739 3.18146 2.89801 2.37887 2.77519 2.98518 4.58477 3.61503
0.01890 4.37405 5.09640 0.61958 0.77255 0.49531 0.94003
711 2.62708 5.07000 2.28860 2.43524 4.41435 3.27227 3.73132 3.86630 2.32880 3.41104 4.19367 2.97193 3.49402 2.85857 3.00866 2.56184 1.73028 3.47720 5.59810 4.21815 759 - -
2.68618 4.42225 2.77519 2.73123 3.46354 2.40513 3.72494 3.29354 2.67741 2.69355 4.24690 2.90347 2.73739 3.18146 2.89801 2.37887 2.77519 2.98518 4.58477 3.61503
0.01831 4.40520 5.12755 0.61958 0.77255 0.52515 0.89517
712 2.66356 4.34939 3.59978 3.03195 3.03462 3.25520 3.91392 2.84125 2.97959 2.48449 3.45598 3.14583 3.61172 3.27469 3.31715 2.25999 2.68411 2.62120 2.55688 3.12133 760 - -
2.68618 4.42225 2.77519 2.73123 3.46354 2.40513 3.72494 3.29354 2.67741 2.69355 4.24690 2.90347 2.73739 3.18146 2.89801 2.37887 2.77519 2.98518 4.58477 3.61503
0.03711 4.40520 3.72090 0.61958 0.77255 0.52515 0.89517
713 2.56999 5.23123 1.82538 2.24600 4.55519 3.23158 3.14722 4.03578 2.47386 3.52996 4.28829 2.59546 3.48864 2.80356 2.97807 2.34983 2.87056 3.61266 5.67737 4.26159 761 - -
2.68618 4.42225 2.77519 2.73123 3.46354 2.40513 3.72494 3.29354 2.67741 2.69355 4.24690 2.90347 2.73739 3.18146 2.89801 2.37887 2.77519 2.98518 4.58477 3.61503
0.01866 4.38675 5.10910 0.61958 0.77255 0.55213 0.85737
714 2.46774 5.11237 2.67295 2.29288 4.43337 3.44829 3.74689 3.88461 2.55394 3.43217 4.22356 2.95992 1.85188 2.58364 3.04241 2.33149 3.00853 3.50307 5.62387 4.24022 762 - -
2.68618 4.42225 2.77519 2.73123 3.46354 2.40513 3.72494 3.29354 2.67741 2.69355 4.24690 2.90347 2.73739 3.18146 2.89801 2.37887 2.77519 2.98518 4.58477 3.61503
0.07373 4.38675 2.83637 0.61958 0.77255 0.55213 0.85737
715 2.62105 4.73229 2.59245 2.69562 4.46747 3.29322 4.00965 3.89520 2.90262 3.51887 4.33585 3.14189 3.90735 3.19150 3.35250 1.17922 2.32975 3.44739 5.73788 4.41058 763 - -
2.68618 4.42225 2.77519 2.73123 3.46354 2.40513 3.72494 3.29354 2.67741 2.69355 4.24690 2.90347 2.73739 3.18146 2.89801 2.37887 2.77519 2.98518 4.58477 3.61503
0.01970 4.33272 5.05507 0.61958 0.77255 0.62460 0.76674
716 2.51653 4.92027 2.39416 2.39535 4.17899 3.46557 3.68209 3.60468 2.47139 2.22046 3.99360 2.98500 3.51118 2.82281 2.64200 2.37177 2.89973 3.26478 5.41633 4.06769 764 - -
2.68618 4.42225 2.77519 2.73123 3.46354 2.40513 3.72494 3.29354 2.67741 2.69355 4.24690 2.90347 2.73739 3.18146 2.89801 2.37887 2.77519 2.98518 4.58477 3.61503
0.01970 4.33272 5.05507 0.61958 0.77255 0.46182 0.99462
717 2.54127 5.14134 2.62577 1.86150 4.46364 3.08245 3.65237 3.93473 2.40226 2.95258 4.19043 2.76839 3.06258 2.75812 2.65039 2.51719 2.91663 3.52017 5.58851 4.18956 765 - -
2.68597 4.42229 2.77509 2.73127 3.46357 2.40516 3.72498 3.29357 2.67744 2.69358 4.24693 2.90350 2.73743 3.18150 2.89797 2.37883 2.77523 2.98522 4.58480 3.61507

0.21826 1.75714 3.74878 0.22789 1.59066 0.52515 0.89517

718 2.59174 5.02079 2.86073 2.19906 4.30458 2.08969 3.67920 3.36663 2.34748 3.30238 4.08678 2.59521 3.86910 2.80473 2.93316 2.56073 2.91759 3.13595 5.49976 4.13024 767 - -

2.68618 4.42225 2.77519 2.73123 3.46354 2.40513 3.72494 3.29354 2.67741 2.69355 4.24690 2.90347 2.73739 3.18146 2.89801 2.37887 2.77519 2.98518 4.58477 3.61503

0.19798 4.38743 1.78866 0.61958 0.77255 0.50758 0.92118

719 2.56337 5.01885 2.87809 2.31616 4.36866 2.75051 3.66383 3.81655 1.77988 3.35312 4.13531 2.93319 3.20048 2.78816 2.86762 2.31111 2.91651 3.42816 5.52976 4.16099 768 - -

2.68618 4.42225 2.77519 2.73123 3.46354 2.40513 3.72494 3.29354 2.67741 2.69355 4.24690 2.90347 2.73739 3.18146 2.89801 2.37887 2.77519 2.98518 4.58477 3.61503

0.02179 4.23286 4.95521 0.61958 0.77255 0.52436 0.89632

720 1.83653 4.96302 2.67026 2.36590 4.36889 2.61047 3.75099 3.81558 2.56041 3.37697 4.16699 2.98351 2.95959 2.88746 3.04321 2.28034 2.94508 3.42385 5.57666 4.20925 769 - -

2.68618 4.42225 2.77519 2.73123 3.46354 2.40513 3.72494 3.29354 2.67741 2.69355 4.24690 2.90347 2.73739 3.18146 2.89801 2.37887 2.77519 2.98518 4.58477 3.61503

0.03897 4.33876 3.68233 0.61958 0.77255 0.54041 0.87349

721 2.49134 3.78142 3.49138 2.79863 3.53485 3.11922 3.87027 2.62695 2.61284 2.60705 3.49624 3.18949 3.58341 3.18964 3.24353 2.75677 2.87858 1.83590 4.97314 3.73499 770 - -

2.68618 4.42225 2.77519 2.73123 3.46354 2.40513 3.72494 3.29354 2.67741 2.69355 4.24690 2.90347 2.73739 3.18146 2.89801 2.37887 2.77519 2.98518 4.58477 3.61503

0.01929 4.35344 5.07579 0.61958 0.77255 0.59788 0.79845

722 2.47034 5.09275 2.55733 2.28932 4.41277 3.44029 3.69415 3.86801 2.47188 3.40245 4.18077 2.94121 2.03938 2.81679 2.62872 2.39937 2.95321 3.47816 5.58116 4.19652 771 - -

2.68618 4.42225 2.77519 2.73123 3.46354 2.40513 3.72494 3.29354 2.67741 2.69355 4.24690 2.90347 2.73739 3.18146 2.89801 2.37887 2.77519 2.98518 4.58477 3.61503

0.14457 4.35344 2.10583 0.61958 0.77255 0.59788 0.79845

723 2.87746 4.39575 4.25106 3.74126 3.36831 3.92410 4.40715 2.35523 3.58345 1.11559 3.30111 3.97229 4.34786 3.87032 3.79268 2.90356 3.15828 1.96049 5.09974 3.89031 772 - -

2.68618 4.42225 2.77519 2.73123 3.46354 2.40513 3.72494 3.29354 2.67741 2.69355 4.24690 2.90347 2.73739 3.18146 2.89801 2.37887 2.77519 2.98518 4.58477 3.61503

0.09655 4.23071 2.55751 0.61958 0.77255 0.73983 0.64855

724 2.70429 4.81475 2.73269 2.60655 4.56925 2.20629 4.03672 4.11742 2.96200 3.70975 4.55698 1.19361 3.90085 3.24486 3.39101 2.76309 3.10211 3.63004 5.80952 4.46821 773 - -

2.68618 4.42225 2.77519 2.73123 3.46354 2.40513 3.72494 3.29354 2.67741 2.69355 4.24690 2.90347 2.73739 3.18146 2.89801 2.37887 2.77519 2.98518 4.58477 3.61503

0.02352 4.15767 4.88002 0.61958 0.77255 0.80877 0.58951

725 3.20878 4.49492 5.00366 4.51955 3.65991 4.60422 5.22156 1.01603 4.40891 2.13075 3.45035 4.71802 4.90074 4.66401 4.59067 4.00809 3.47598 1.32768 5.67340 4.47216 774 - -

2.68618 4.42225 2.77519 2.73123 3.46354 2.40513 3.72494 3.29354 2.67741 2.69355 4.24690 2.90347 2.73739 3.18146 2.89801 2.37887 2.77519 2.98518 4.58477 3.61503

0.02352 4.15767 4.88002 0.61958 0.77255 0.68416 0.70222

726 3.05232 5.42430 0.92043 2.36775 4.75491 3.39511 3.93837 4.31223 2.88107 3.83896 4.71181 2.93358 4.00162 3.11743 2.82103 2.98287 3.34923 3.90977 5.93295 4.51592 775 - -

2.68618 4.42225 2.77519 2.73123 3.46354 2.40513 3.72494 3.29354 2.67741 2.69355 4.24690 2.90347 2.73739 3.18146 2.89801 2.37887 2.77519 2.98518 4.58477 3.61503

0.02202 4.22274 4.94509 0.61958 0.77255 0.69502 0.69128

727 2.56123 4.68406 3.20529 2.64098 3.90226 3.17593 3.74217 3.24361 1.65220 2.79099 3.18968 3.12379 3.91970 2.93461 2.93542 2.75713 2.89950 2.66652 5.22982 3.94297 776 - -

2.68618 4.42225 2.77519 2.73123 3.46354 2.40513 3.72494 3.29354 2.67741 2.69355 4.24690 2.90347 2.73739 3.18146 2.89801 2.37887 2.77519 2.98518 4.58477 3.61503

0.18024 4.27313 1.89055 0.61958 0.77255 0.48107 0.96265

728 2.33638 4.73307 3.10998 2.63586 4.29457 3.13981 3.81844 3.72025 2.57454 3.31129 4.12037 2.92713 3.86501 2.97744 2.28082 1.56461 2.90863 3.32234 5.52178 4.21183 777 - -

2.68650 4.41558 2.77641 2.73167 3.46239 2.40642 3.72605 3.29214 2.67828 2.69072 4.24654 2.90283 2.73804 3.18339 2.89843 2.37978 2.77477 2.98541 4.58060 3.61123

0.89961 0.64813 2.65571 2.97155 0.05258 0.65363 0.73430

729 2.72954 5.14026 2.57744 2.25979 4.47220 3.44850 3.64108 3.92347 2.14347 3.42415 4.20010 2.92900 3.86232 2.75637 1.75527 2.58752 2.80942 3.52694 5.56720 4.19914 833 - -

2.68618 4.42225 2.77519 2.73123 3.46354 2.40513 3.72494 3.29354 2.67741 2.69355 4.24690 2.90347 2.73739 3.18146 2.89801 2.37887 2.77519 2.98518 4.58477 3.61503
0.11808 4.23191 2.33455 0.61958 0.77255 0.76437 0.62666
730 3.19893 4.61372 4.58068 4.14691 1.15490 4.20238 4.18644 2.48881 4.01482 1.98567 3.32644 4.21906 4.58096 4.15592 4.14297 3.59118 3.47490 2.10004 4.47044 2.90452 834 - -
2.68618 4.42225 2.77519 2.73123 3.46354 2.40513 3.72494 3.29354 2.67741 2.69355 4.24690 2.90347 2.73739 3.18146 2.89801 2.37887 2.77519 2.98518 4.58477 3.61503
0.02399 4.13782 4.86017 0.61958 0.77255 0.84954 0.55793
731 2.84357 5.24682 2.18811 2.27898 4.41943 3.34985 1.77667 4.02502 2.61249 3.55093 4.37481 2.28327 3.88196 2.89942 3.11029 2.79024 3.10563 3.63834 5.65324 4.20254 835 - -
2.68539 4.42254 2.77549 2.73153 3.46346 2.40542 3.72524 3.29348 2.67770 2.69312 4.24204 2.90376 2.73769 3.18176 2.89830 2.37916 2.77549 2.98524 4.58506 3.61302
0.59363 0.82113 4.86017 1.28876 0.32243 0.84954 0.55793
732 1.21079 4.21733 3.58581 3.30334 4.37701 3.00771 4.34567 3.74353 3.34530 3.48031 4.32030 3.41522 3.76841 3.63031 3.64079 1.51589 2.78061 3.20540 5.73670 4.52799 840 - -
2.68618 4.42225 2.77519 2.73123 3.46354 2.40513 3.72494 3.29354 2.67741 2.69355 4.24690 2.90347 2.73739 3.18146 2.89801 2.37887 2.77519 2.98518 4.58477 3.61503
0.05313 4.13782 3.33034 0.61958 0.77255 0.84954 0.55793
733 2.85201 5.14969 1.12862 2.31293 4.62314 3.26578 3.89060 4.02569 2.88400 3.66805 4.54956 2.86282 3.89500 3.07322 3.42149 2.82464 2.53818 3.63133 5.87549 4.44618 841 - -
2.68618 4.42225 2.77519 2.73123 3.46354 2.40513 3.72494 3.29354 2.67741 2.69355 4.24690 2.90347 2.73739 3.18146 2.89801 2.37887 2.77519 2.98518 4.58477 3.61503
0.02469 4.10939 4.83174 0.61958 0.77255 0.87248 0.54114
734 3.01923 5.51736 1.03139 2.18264 4.83779 3.26314 3.87842 4.42284 2.94490 3.94044 4.81676 2.28355 3.91619 3.06236 3.54965 2.91334 3.33556 3.98386 6.05324 4.54018 842 - -
2.68618 4.42225 2.77519 2.73123 3.46354 2.40513 3.72494 3.29354 2.67741 2.69355 4.24690 2.90347 2.73739 3.18146 2.89801 2.37887 2.77519 2.98518 4.58477 3.61503
0.02469 4.10939 4.83174 0.61958 0.77255 0.87248 0.54114
735 2.82640 4.63109 3.58506 2.97158 3.66982 3.70799 3.85560 2.46486 2.50506 2.24256 3.59654 3.37812 4.08020 3.09597 1.63536 2.99789 3.04891 2.72271 5.13345 3.88327 843 - -
2.68618 4.42225 2.77519 2.73123 3.46354 2.40513 3.72494 3.29354 2.67741 2.69355 4.24690 2.90347 2.73739 3.18146 2.89801 2.37887 2.77519 2.98518 4.58477 3.61503
0.02469 4.10939 4.83174 0.61958 0.77255 0.87248 0.54114
736 2.76919 4.89963 3.06481 2.58203 4.17547 3.49838 3.69349 3.54684 2.13744 3.14190 4.01487 3.06461 3.92336 1.63426 2.63233 2.80583 3.00157 2.74844 5.38092 4.08714 844 - -
2.68618 4.42225 2.77519 2.73123 3.46354 2.40513 3.72494 3.29354 2.67741 2.69355 4.24690 2.90347 2.73739 3.18146 2.89801 2.37887 2.77519 2.98518 4.58477 3.61503
0.02469 4.10939 4.83174 0.61958 0.77255 0.87248 0.54114
737 3.01458 5.22174 3.26177 2.32757 4.64852 3.64146 3.64397 4.00842 1.80090 3.45862 4.30389 3.14083 4.02409 2.77339 1.33976 2.99451 3.19358 3.67062 5.52319 4.31122 845 - -
2.68618 4.42225 2.77519 2.73123 3.46354 2.40513 3.72494 3.29354 2.67741 2.69355 4.24690 2.90347 2.73739 3.18146 2.89801 2.37887 2.77519 2.98518 4.58477 3.61503
0.02469 4.10939 4.83174 0.61958 0.77255 0.74380 0.64494
738 2.78406 4.40036 4.70956 4.14844 2.44042 4.28286 4.60913 1.31309 4.01917 1.70085 3.15202 4.32554 4.57302 4.18328 4.14894 3.61274 3.28147 2.02770 5.06654 3.86651 846 - -
2.68618 4.42225 2.77519 2.73123 3.46354 2.40513 3.72494 3.29354 2.67741 2.69355 4.24690 2.90347 2.73739 3.18146 2.89801 2.37887 2.77519 2.98518 4.58477 3.61503
0.02299 4.17985 4.90220 0.61958 0.77255 0.81337 0.58584
739 2.74487 4.45535 3.73355 3.28384 3.54616 3.60963 4.16566 2.81721 3.14570 2.35948 1.46599 3.60052 2.49566 3.51501 3.42378 2.99280 3.05829 2.68291 5.14874 3.92125 847 - -
2.68618 4.42225 2.77519 2.73123 3.46354 2.40513 3.72494 3.29354 2.67741 2.69355 4.24690 2.90347 2.73739 3.18146 2.89801 2.37887 2.77519 2.98518 4.58477 3.61503
0.02299 4.17985 4.90220 0.61958 0.77255 0.81337 0.58584
740 2.85161 4.87054 3.07311 2.79913 4.41611 3.43204 3.98666 3.85473 2.20106 3.45916 4.35673 3.23899 1.15581 3.19083 2.97119 2.92503 3.18254 3.49494 5.60773 4.35955 848 - -
2.68618 4.42225 2.77519 2.73123 3.46354 2.40513 3.72494 3.29354 2.67741 2.69355 4.24690 2.90347 2.73739 3.18146 2.89801 2.37887 2.77519 2.98518 4.58477 3.61503
0.02299 4.17985 4.90220 0.61958 0.77255 0.81337 0.58584

741 2.34501 4.23878 3.63863 3.08149 2.92305 3.57471 3.91295 2.73180 3.01675 2.45518 2.14183 3.42750 3.22742 3.30397 3.32628 2.52114 2.83506 2.51729 4.86567 3.64332 849 - -
2.68618 4.42225 2.77519 2.73123 3.46354 2.40513 3.72494 3.29354 2.67741 2.69355 4.24690 2.90347 2.73739 3.18146 2.89801 2.37887 2.77519 2.98518 4.58477 3.61503
0.02299 4.17985 4.90220 0.61958 0.77255 0.81337 0.58584
742 2.29669 4.93563 2.02831 2.42646 4.48096 3.28698 3.85579 3.91270 2.76235 3.51148 4.33771 2.94459 1.73615 3.02313 3.25742 2.72278 3.03005 3.49950 5.72210 4.34488 850 - -
2.68618 4.42225 2.77519 2.73123 3.46354 2.40513 3.72494 3.29354 2.67741 2.69355 4.24690 2.90347 2.73739 3.18146 2.89801 2.37887 2.77519 2.98518 4.58477 3.61503
0.09516 4.17985 2.58404 0.61958 0.77255 0.81337 0.58584
743 2.65957 4.73121 2.98723 1.77329 3.99283 3.44682 3.74523 2.76767 2.56068 2.96842 3.85857 3.04378 3.88665 2.92520 2.98669 2.73213 2.29102 2.93560 5.32694 4.01410 851 - -
2.68618 4.42225 2.77519 2.73123 3.46354 2.40513 3.72494 3.29354 2.67741 2.69355 4.24690 2.90347 2.73739 3.18146 2.89801 2.37887 2.77519 2.98518 4.58477 3.61503
0.02469 4.10939 4.83174 0.61958 0.77255 0.87248 0.54114
744 2.88988 5.24710 1.08364 2.26006 4.71652 3.24206 3.89373 4.24854 2.92949 3.81441 4.68027 2.81948 3.89252 3.07966 3.49714 2.33220 3.22899 3.80687 5.95760 4.48821 852 - -
2.68618 4.42225 2.77519 2.73123 3.46354 2.40513 3.72494 3.29354 2.67741 2.69355 4.24690 2.90347 2.73739 3.18146 2.89801 2.37887 2.77519 2.98518 4.58477 3.61503
0.02469 4.10939 4.83174 0.61958 0.77255 0.87248 0.54114
745 2.90562 4.93075 3.39032 2.86922 4.42067 3.54118 3.81145 3.85475 2.25309 3.38237 4.26987 3.27269 2.86656 2.98163 1.12882 2.96382 3.17047 3.51620 5.48096 4.27642 853 - -
2.68618 4.42225 2.77519 2.73123 3.46354 2.40513 3.72494 3.29354 2.67741 2.69355 4.24690 2.90347 2.73739 3.18146 2.89801 2.37887 2.77519 2.98518 4.58477 3.61503
0.02469 4.10939 4.83174 0.61958 0.77255 0.87248 0.54114
746 2.68883 4.78383 2.14632 2.47131 4.06630 3.38035 3.78492 3.34627 2.68552 3.10401 3.97595 2.57203 3.88081 2.97854 3.14837 2.74554 2.96193 1.87753 5.40558 4.06429 854 - -
2.68618 4.42225 2.77519 2.73123 3.46354 2.40513 3.72494 3.29354 2.67741 2.69355 4.24690 2.90347 2.73739 3.18146 2.89801 2.37887 2.77519 2.98518 4.58477 3.61503
0.02469 4.10939 4.83174 0.61958 0.77255 0.74380 0.64494
747 1.95224 4.37387 3.42323 2.94241 3.87705 3.31149 3.97691 2.67624 2.91441 2.92723 3.80659 3.29166 1.94622 3.23988 3.28134 2.40725 2.83286 2.86898 5.27514 4.02737 855 - -
2.68618 4.42225 2.77519 2.73123 3.46354 2.40513 3.72494 3.29354 2.67741 2.69355 4.24690 2.90347 2.73739 3.18146 2.89801 2.37887 2.77519 2.98518 4.58477 3.61503
0.02299 4.17985 4.90220 0.61958 0.77255 0.81337 0.58584
748 2.46358 4.39762 3.42971 3.14518 4.31053 3.16770 4.24759 3.63620 3.15718 3.36400 4.26447 3.39340 1.27084 3.51090 3.47073 2.63199 1.98939 3.20565 5.66249 4.43519 856 - -
2.68618 4.42225 2.77519 2.73123 3.46354 2.40513 3.72494 3.29354 2.67741 2.69355 4.24690 2.90347 2.73739 3.18146 2.89801 2.37887 2.77519 2.98518 4.58477 3.61503
0.02299 4.17985 4.90220 0.61958 0.77255 0.81337 0.58584
749 2.79500 4.96713 3.19960 2.60731 4.24557 3.55887 3.65885 3.66389 2.02704 3.21701 4.04794 3.08691 3.15294 2.80490 1.62567 2.81761 3.00545 3.34309 5.38225 3.24142 857 - -
2.68616 4.42231 2.77526 2.73130 3.46360 2.40506 3.72501 3.29360 2.67747 2.69344 4.24696 2.90353 2.73746 3.18153 2.89786 2.37879 2.77507 2.98525 4.58483 3.61510
0.25002 1.54280 4.90220 0.61159 0.78196 0.81337 0.58584
750 2.17500 4.55774 3.03366 2.74590 4.33953 1.39701 3.99793 3.76442 2.87701 3.39490 4.22501 3.14645 3.22919 2.86317 3.27704 2.62480 2.90843 3.32014 5.62954 4.34141 860 - -
2.68618 4.42225 2.77519 2.73123 3.46354 2.40513 3.72494 3.29354 2.67741 2.69355 4.24690 2.90347 2.73739 3.18146 2.89801 2.37887 2.77519 2.98518 4.58477 3.61503
0.05666 4.17985 3.22425 0.61958 0.77255 0.81337 0.58584
751 2.29489 4.79449 2.93127 2.50049 4.26049 3.33592 3.73925 3.68931 2.51789 3.27436 4.08436 2.99286 2.83227 1.87122 2.95036 2.35718 2.89351 3.30740 5.48655 4.15322 861 - -
2.68618 4.42225 2.77519 2.73123 3.46354 2.40513 3.72494 3.29354 2.67741 2.69355 4.24690 2.90347 2.73739 3.18146 2.89801 2.37887 2.77519 2.98518 4.58477 3.61503
0.02377 4.14696 4.86931 0.61958 0.77255 0.84192 0.56367
752 2.77704 5.01713 3.01512 2.52880 4.37071 3.48094 3.67380 3.79945 2.26469 3.32755 4.15049 3.02449 2.46353 1.82732 2.31784 2.78198 3.00984 3.44538 5.48238 4.17589 862 - -
2.68618 4.42225 2.77519 2.73123 3.46354 2.40513 3.72494 3.29354 2.67741 2.69355 4.24690 2.90347 2.73739 3.18146 2.89801 2.37887 2.77519 2.98518 4.58477 3.61503

0.02377 4.14696 4.86931 0.61958 0.77255 0.84192 0.56367

753 3.00063 4.47385 4.31378 3.77724 3.24096 4.09785 4.45052 2.04637 3.61836 1.05506 3.11869 4.05705 3.57386 3.88497 3.82750 3.42817 3.24763 2.28800 5.07735 3.90841 863 - -

2.68618 4.42225 2.77519 2.73123 3.46354 2.40513 3.72494 3.29354 2.67741 2.69355 4.24690 2.90347 2.73739 3.18146 2.89801 2.37887 2.77519 2.98518 4.58477 3.61503

0.02377 4.14696 4.86931 0.61958 0.77255 0.77866 0.61438

754 1.02694 4.34481 3.76475 3.36210 3.88156 3.33987 4.29304 2.92121 3.26049 2.76640 3.23499 3.56942 3.98717 3.61142 3.54389 2.76085 2.94355 2.67728 5.42445 4.18867 864 - -

2.68618 4.42225 2.77519 2.73123 3.46354 2.40513 3.72494 3.29354 2.67741 2.69355 4.24690 2.90347 2.73739 3.18146 2.89801 2.37887 2.77519 2.98518 4.58477 3.61503

0.02299 4.17985 4.90220 0.61958 0.77255 0.72843 0.65907

755 2.69204 4.58104 3.22510 2.45766 3.63209 3.55674 3.77179 3.12109 2.65440 2.62472 3.67740 3.18839 3.95784 2.84716 3.02692 2.82381 2.69869 2.87347 5.04241 1.89728 865 - -

2.68618 4.42225 2.77519 2.73123 3.46354 2.40513 3.72494 3.29354 2.67741 2.69355 4.24690 2.90347 2.73739 3.18146 2.89801 2.37887 2.77519 2.98518 4.58477 3.61503

0.02202 4.22284 4.94519 0.61958 0.77255 0.77327 0.61897

756 2.51449 4.56340 3.24490 2.69343 3.32461 3.52256 3.76602 2.75181 2.20221 2.78486 3.66530 3.16055 2.24737 2.99352 3.03578 2.76058 2.87179 2.84061 5.12687 3.84979 866 - -

2.68618 4.42225 2.77519 2.73123 3.46354 2.40513 3.72494 3.29354 2.67741 2.69355 4.24690 2.90347 2.73739 3.18146 2.89801 2.37887 2.77519 2.98518 4.58477 3.61503

0.02202 4.22284 4.94519 0.61958 0.77255 0.77327 0.61897

757 2.74978 5.16906 2.44489 1.43744 4.48839 3.09827 3.70385 3.95185 2.53127 3.48153 4.26936 2.86936 3.13386 2.83462 3.03806 2.71296 3.00117 3.19920 5.65810 4.24953 867 - -

2.68618 4.42225 2.77519 2.73123 3.46354 2.40513 3.72494 3.29354 2.67741 2.69355 4.24690 2.90347 2.73739 3.18146 2.89801 2.37887 2.77519 2.98518 4.58477 3.61503

0.02202 4.22284 4.94519 0.61958 0.77255 0.77327 0.61897

758 1.27816 4.29708 3.71189 3.22332 3.81297 3.35856 4.14739 2.61157 3.16343 2.82534 3.75609 3.48329 3.94940 3.46980 3.48293 2.35546 2.86848 2.44626 5.27685 4.05257 868 - -

2.68618 4.42225 2.77519 2.73123 3.46354 2.40513 3.72494 3.29354 2.67741 2.69355 4.24690 2.90347 2.73739 3.18146 2.89801 2.37887 2.77519 2.98518 4.58477 3.61503

0.02202 4.22284 4.94519 0.61958 0.77255 0.71874 0.66820

759 2.69120 4.69266 2.44961 2.61717 3.97867 3.45725 3.84796 3.22216 2.75552 3.00739 3.88500 2.63955 3.93664 3.06027 3.19484 2.78828 2.78988 1.63641 5.34536 4.04093 869 - -

2.68618 4.42225 2.77519 2.73123 3.46354 2.40513 3.72494 3.29354 2.67741 2.69355 4.24690 2.90347 2.73739 3.18146 2.89801 2.37887 2.77519 2.98518 4.58477 3.61503

0.02144 4.24910 4.97144 0.61958 0.77255 0.65133 0.73680

760 2.46171 4.65489 2.95476 2.61667 3.85113 3.51250 3.20311 3.23987 2.45669 1.93260 3.74729 3.10635 3.90776 2.94571 3.02859 2.50533 2.65390 2.96396 5.19672 3.90537 870 - -

2.68613 4.42234 2.77528 2.73132 3.46337 2.40521 3.72427 3.29352 2.67749 2.69340 4.24698 2.90346 2.73748 3.18145 2.89809 2.37870 2.77528 2.98527 4.58486 3.61512

0.28487 1.42186 5.01685 0.63422 0.75577 0.43447 1.04301

761 2.67867 4.72177 2.80752 2.51828 3.92213 3.21985 3.76300 3.31547 2.61402 1.84596 3.80765 3.11900 3.93233 2.83485 2.94094 2.61896 2.55957 2.83067 5.25577 3.95608 873 - -

2.68618 4.42225 2.77519 2.73123 3.46354 2.40513 3.72494 3.29354 2.67741 2.69355 4.24690 2.90347 2.73739 3.18146 2.89801 2.37887 2.77519 2.98518 4.58477 3.61503

0.01805 4.41970 5.14205 0.61958 0.77255 0.53557 0.88028

762 2.67351 4.80325 2.57875 2.46479 4.02270 3.51943 3.72950 3.04027 2.55021 2.92545 3.88013 2.94110 3.48474 2.74013 2.83511 2.72844 2.66199 2.07843 5.31982 4.00098 874 - -

2.68618 4.42225 2.77519 2.73123 3.46354 2.40513 3.72494 3.29354 2.67741 2.69355 4.24690 2.90347 2.73739 3.18146 2.89801 2.37887 2.77519 2.98518 4.58477 3.61503

0.01805 4.41970 5.14205 0.61958 0.77255 0.53557 0.88028

763 2.65894 5.21471 1.64531 2.25477 4.51836 3.12127 3.72456 3.98636 2.52149 2.82291 4.27748 2.63925 3.90203 2.68542 3.02341 2.73757 2.86573 3.58482 5.67118 4.26556 875 - -

2.68618 4.42225 2.77519 2.73123 3.46354 2.40513 3.72494 3.29354 2.67741 2.69355 4.24690 2.90347 2.73739 3.18146 2.89801 2.37887 2.77519 2.98518 4.58477 3.61503

0.01805 4.41970 5.14205 0.61958 0.77255 0.53557 0.88028

764 2.51463 4.78491 3.13572 2.52552 4.06898 2.95449 3.77357 3.47387 2.58992 2.77242 3.92800 3.10118 1.92855 2.94469 2.85199 2.61221 2.68488 3.15862 5.36353 4.05077 876 - -

2.68618 4.42225 2.77519 2.73123 3.46354 2.40513 3.72494 3.29354 2.67741 2.69355 4.24690 2.90347 2.73739 3.18146 2.89801 2.37887 2.77519 2.98518 4.58477 3.61503

0.01805 4.41970 5.14205 0.61958 0.77255 0.45583 1.00491

765 2.46882 5.01325 2.90511 2.10693 4.28919 3.06058 3.68851 2.45444 2.27987 3.28868 4.07404 2.98974 3.43874 2.59239 2.93693 2.57545 2.76445 3.36637 5.49022 4.12522 877 - -

2.68618 4.42225 2.77519 2.73123 3.46354 2.40513 3.72494 3.29354 2.67741 2.69355 4.24690 2.90347 2.73739 3.18146 2.89801 2.37887 2.77519 2.98518 4.58477 3.61503

0.01747 4.45155 5.17389 0.61958 0.77255 0.48576 0.95510

766 2.62947 5.18050 2.63189 2.06324 4.49982 2.95866 3.69025 3.97081 2.45590 3.04121 4.23618 1.99900 3.05476 2.80046 2.95063 2.69511 2.83699 3.55882 5.63264 4.23061 878 - -

2.68618 4.42225 2.77519 2.73123 3.46354 2.40513 3.72494 3.29354 2.67741 2.69355 4.24690 2.90347 2.73739 3.18146 2.89801 2.37887 2.77519 2.98518 4.58477 3.61503

0.01747 4.45155 5.17389 0.61958 0.77255 0.48576 0.95510

767 2.57306 5.09890 3.03169 2.38405 4.40105 3.54294 3.17383 3.83155 2.41582 3.37484 4.17298 3.04729 1.76387 2.76975 2.38486 2.78998 3.02128 3.47198 5.55362 4.21237 879 - -

2.68618 4.42225 2.77519 2.73123 3.46354 2.40513 3.72494 3.29354 2.67741 2.69355 4.24690 2.90347 2.73739 3.18146 2.89801 2.37887 2.77519 2.98518 4.58477 3.61503

0.01747 4.45155 5.17389 0.61958 0.77255 0.48576 0.95510

768 2.51950 5.12652 2.14535 2.35601 4.44400 3.29008 3.65883 3.91076 2.16400 3.41985 3.51040 2.94694 3.39827 2.53363 2.75732 2.66048 2.62014 3.24506 5.57601 4.18256 880 - -

2.68618 4.42225 2.77519 2.73123 3.46354 2.40513 3.72494 3.29354 2.67741 2.69355 4.24690 2.90347 2.73739 3.18146 2.89801 2.37887 2.77519 2.98518 4.58477 3.61503

0.01747 4.45155 5.17389 0.61958 0.77255 0.48576 0.95510

769 2.36976 4.22848 3.90382 3.32762 2.47320 3.53529 4.02042 2.69162 3.23916 1.67949 3.34141 3.62180 3.29771 3.49712 3.27521 2.79548 2.90902 2.48862 4.82938 3.29092 881 - -

2.68618 4.42225 2.77519 2.73123 3.46354 2.40513 3.72494 3.29354 2.67741 2.69355 4.24690 2.90347 2.73739 3.18146 2.89801 2.37887 2.77519 2.98518 4.58477 3.61503

0.03457 4.45155 3.80223 0.61958 0.77255 0.48576 0.95510

770 2.79545 5.11841 2.56180 2.52153 4.38993 3.54698 3.71374 3.85538 2.20579 3.38691 4.18260 3.04305 3.94662 2.84212 1.63363 2.78675 3.01942 3.49148 5.53752 2.74809 882 - -

2.68618 4.42225 2.77519 2.73123 3.46354 2.40513 3.72494 3.29354 2.67741 2.69355 4.24690 2.90347 2.73739 3.18146 2.89801 2.37887 2.77519 2.98518 4.58477 3.61503

0.01777 4.43475 5.15709 0.61958 0.77255 0.51255 0.91372

771 2.59514 4.95795 2.42287 2.47876 4.22114 2.07490 3.69697 3.18478 2.47754 3.10930 3.79612 3.00682 3.88714 2.59268 2.83140 2.69718 2.82112 3.30362 5.44771 4.09507 883 - -

2.68618 4.42225 2.77519 2.73123 3.46354 2.40513 3.72494 3.29354 2.67741 2.69355 4.24690 2.90347 2.73739 3.18146 2.89801 2.37887 2.77519 2.98518 4.58477 3.61503

0.01777 4.43475 5.15709 0.61958 0.77255 0.46937 0.98188

772 2.74332 5.22481 2.23104 1.71815 4.54888 3.46405 3.69446 4.02691 2.45921 2.94226 4.27570 2.84432 3.88646 2.56235 2.85312 2.32705 2.97896 3.60507 5.66633 4.25653 884 - -

2.68618 4.42225 2.77519 2.73123 3.46354 2.40513 3.72494 3.29354 2.67741 2.69355 4.24690 2.90347 2.73739 3.18146 2.89801 2.37887 2.77519 2.98518 4.58477 3.61503

0.01747 4.45155 5.17389 0.61958 0.77255 0.48576 0.95510

773 2.55008 4.41643 3.58410 3.03421 3.64721 3.59015 3.97271 2.97931 2.97920 2.62444 3.60525 3.41641 2.53225 3.29208 3.15923 2.55360 2.52651 1.69813 5.08452 3.85048 885 - -

2.68618 4.42225 2.77519 2.73123 3.46354 2.40513 3.72494 3.29354 2.67741 2.69355 4.24690 2.90347 2.73739 3.18146 2.89801 2.37887 2.77519 2.98518 4.58477 3.61503

0.01747 4.45155 5.17389 0.61958 0.77255 0.48576 0.95510

774 2.88039 5.34917 1.35708 2.38259 4.68482 3.11938 3.81338 4.16875 2.65134 3.67417 4.45812 2.18925 3.95983 2.94303 2.86500 2.82805 3.13666 3.42770 5.83179 4.40368 886 - -

2.68633 4.42240 2.77416 2.73138 3.46368 2.40527 3.72509 3.29369 2.67648 2.69369 4.24704 2.90361 2.73754 3.18161 2.89815 2.37901 2.77534 2.98533 4.58492 3.61518

0.39620 1.13486 5.17389 0.68076 0.70569 0.48576 0.95510

775 2.40769 4.46467 3.46005 2.78022 3.61171 3.63185 3.58592 2.97888 2.67919 2.46334 3.39072 3.32669 3.44458 3.17010 3.23051 2.72290 2.91080 2.46384 5.03689 2.08391 889 - -

2.68618 4.42225 2.77519 2.73123 3.46354 2.40513 3.72494 3.29354 2.67741 2.69355 4.24690 2.90347 2.73739 3.18146 2.89801 2.37887 2.77519 2.98518 4.58477 3.61503

0.01747 4.45155 5.17389 0.61958 0.77255 0.48576 0.95510

776 2.56601 5.02654 3.00632 2.27497 4.31084 3.48730 3.68430 3.75320 2.44640 3.30615 3.45300 2.98730 2.96337 2.10238 2.81252 2.46974 2.36481 3.38485 5.50087 4.13383 890 - -
2.68618 4.42225 2.77519 2.73123 3.46354 2.40513 3.72494 3.29354 2.67741 2.69355 4.24690 2.90347 2.73739 3.18146 2.89801 2.37887 2.77519 2.98518 4.58477 3.61503
0.01747 4.45155 5.17389 0.61958 0.77255 0.48576 0.95510
777 4.18366 5.43309 4.74563 4.61262 2.55917 4.54565 4.04677 4.13151 4.46104 3.42685 4.74656 4.50719 5.01904 4.61403 4.50971 4.24703 4.48017 4.03217 4.21361 0.36989 891 - -
2.68618 4.42225 2.77519 2.73123 3.46354 2.40513 3.72494 3.29354 2.67741 2.69355 4.24690 2.90347 2.73739 3.18146 2.89801 2.37887 2.77519 2.98518 4.58477 3.61503
0.01747 4.45155 5.17389 0.61958 0.77255 0.48576 0.95510
778 2.12699 4.41363 3.82883 3.41037 4.44622 3.23748 4.41850 3.80622 3.38688 3.52467 4.35541 3.57170 3.95487 3.68156 3.71631 1.17210 1.67710 3.08019 5.80109 4.58884 892 - -
2.68618 4.42225 2.77519 2.73123 3.46354 2.40513 3.72494 3.29354 2.67741 2.69355 4.24690 2.90347 2.73739 3.18146 2.89801 2.37887 2.77519 2.98518 4.58477 3.61503
0.01747 4.45155 5.17389 0.61958 0.77255 0.48576 0.95510
779 3.08895 2.18683 4.94636 4.37665 3.45011 4.35991 4.76536 1.84747 4.22599 1.35626 3.33139 4.47958 4.66997 4.38124 4.30950 3.69747 3.32955 1.74838 5.22347 4.06596 893 - -
2.68618 4.42225 2.77519 2.73123 3.46354 2.40513 3.72494 3.29354 2.67741 2.69355 4.24690 2.90347 2.73739 3.18146 2.89801 2.37887 2.77519 2.98518 4.58477 3.61503
0.01747 4.45155 5.17389 0.61958 0.77255 0.48576 0.95510
780 3.13916 5.73466 1.97143 1.23469 5.05385 1.82874 3.97704 4.58232 2.97349 4.05775 4.88608 2.91557 4.06234 3.13320 3.57096 3.02608 3.42464 4.13289 6.20676 4.69694 894 - -
2.68621 4.42228 2.77523 2.73126 3.46357 2.40507 3.72498 3.29357 2.67744 2.69358 4.24693 2.90350 2.73743 3.18149 2.89774 2.37890 2.77523 2.98505 4.58480 3.61506
0.44813 3.08550 1.15369 0.95320 0.48696 0.48576 0.95510
781 2.70361 4.90090 2.99913 2.51389 4.27555 3.01387 3.66606 3.68714 2.07776 3.24688 4.06791 3.00523 2.12301 2.81227 2.33490 2.72686 2.94859 3.33718 5.42956 4.12764 899 - -
2.68618 4.42225 2.77519 2.73123 3.46354 2.40513 3.72494 3.29354 2.67741 2.69355 4.24690 2.90347 2.73739 3.18146 2.89801 2.37887 2.77519 2.98518 4.58477 3.61503
0.02542 4.08076 4.80311 0.61958 0.77255 0.89442 0.52568
782 2.43570 4.80192 2.95035 2.46621 4.23058 2.98142 3.67892 3.65647 1.79028 3.23247 4.03545 2.96413 3.01528 2.82311 2.85457 2.49986 2.68175 3.28030 5.43772 4.10523 900 - -
2.68618 4.42225 2.77519 2.73123 3.46354 2.40513 3.72494 3.29354 2.67741 2.69355 4.24690 2.90347 2.73739 3.18146 2.89801 2.37887 2.77519 2.98518 4.58477 3.61503
0.02542 4.08076 4.80311 0.61958 0.77255 0.89442 0.52568
783 3.15492 4.46284 4.88349 4.39763 3.58800 4.50687 5.10419 1.05199 4.27870 2.06738 3.38990 4.60773 4.81757 4.54009 4.46762 3.90579 3.42388 1.39149 5.58507 4.38326 901 - -
2.68618 4.42225 2.77519 2.73123 3.46354 2.40513 3.72494 3.29354 2.67741 2.69355 4.24690 2.90347 2.73739 3.18146 2.89801 2.37887 2.77519 2.98518 4.58477 3.61503
0.02542 4.08076 4.80311 0.61958 0.77255 0.89442 0.52568
784 2.55423 4.46904 3.25630 2.72546 3.76275 3.41774 3.78409 3.08622 2.47648 2.63685 3.67861 3.16018 3.86956 3.01887 3.03322 2.30762 1.95299 2.61532 5.13828 3.87333 902 - -
2.68618 4.42225 2.77519 2.73123 3.46354 2.40513 3.72494 3.29354 2.67741 2.69355 4.24690 2.90347 2.73739 3.18146 2.89801 2.37887 2.77519 2.98518 4.58477 3.61503
0.02542 4.08076 4.80311 0.61958 0.77255 0.29531 1.36376
785 2.99504 5.52258 1.56712 2.35396 4.86698 2.41303 3.89067 4.37437 2.79595 3.86102 4.65934 1.69719 4.00637 3.03100 3.08150 2.91585 3.26616 3.93469 6.01068 4.54507 903 - -
2.68618 4.42225 2.77519 2.73123 3.46354 2.40513 3.72494 3.29354 2.67741 2.69355 4.24690 2.90347 2.73739 3.18146 2.89801 2.37887 2.77519 2.98518 4.58477 3.61503
0.01747 4.45155 5.17389 0.61958 0.77255 0.48576 0.95510
786 2.85141 4.86578 3.35999 2.78273 3.77399 3.67701 3.77942 3.47576 2.09834 3.08171 3.95118 3.24667 4.05577 2.04164 2.84863 2.92423 3.06974 3.20018 2.12649 3.12340 904 - -
2.68618 4.42225 2.77519 2.73123 3.46354 2.40513 3.72494 3.29354 2.67741 2.69355 4.24690 2.90347 2.73739 3.18146 2.89801 2.37887 2.77519 2.98518 4.58477 3.61503
0.01747 4.45155 5.17389 0.61958 0.77255 0.48576 0.95510
787 3.06680 5.38193 1.20376 2.47752 4.05510 3.53440 3.91419 4.11119 2.93458 3.65004 4.52876 2.82077 4.08767 3.15628 3.45742 3.01771 3.33716 3.76254 5.48278 2.04207 905 - -
2.68618 4.42225 2.77519 2.73123 3.46354 2.40513 3.72494 3.29354 2.67741 2.69355 4.24690 2.90347 2.73739 3.18146 2.89801 2.37887 2.77519 2.98518 4.58477 3.61503

0.01747 4.45155 5.17389 0.61958 0.77255 0.48576 0.95510

788 2.27811 5.24229 2.75867 1.95161 4.58255 3.23308 3.76995 4.05378 2.57581 3.56861 4.34566 1.74725 3.93004 2.78387 3.07663 2.42891 3.05954 3.64320 5.73315 4.32517 906 - -

2.68618 4.42225 2.77519 2.73123 3.46354 2.40513 3.72494 3.29354 2.67741 2.69355 4.24690 2.90347 2.73739 3.18146 2.89801 2.37887 2.77519 2.98518 4.58477 3.61503

0.01747 4.45155 5.17389 0.61958 0.77255 0.48576 0.95510

789 2.97467 5.34386 3.24032 2.65346 4.75671 3.67084 3.72973 4.16960 1.70351 3.60538 4.39880 2.03875 4.04992 2.14959 1.95235 2.93917 3.17375 3.77827 5.67525 4.38645 907 - -

2.68618 4.42225 2.77519 2.73123 3.46354 2.40513 3.72494 3.29354 2.67741 2.69355 4.24690 2.90347 2.73739 3.18146 2.89801 2.37887 2.77519 2.98518 4.58477 3.61503

0.01747 4.45155 5.17389 0.61958 0.77255 0.48576 0.95510

790 2.98627 4.75091 3.89548 3.30469 4.00866 3.82088 4.11800 3.02425 2.69597 2.91445 3.93738 3.64426 4.26970 3.36789 1.43989 3.18867 3.25331 1.48470 5.41078 4.18426 908 - -

2.68618 4.42225 2.77519 2.73123 3.46354 2.40513 3.72494 3.29354 2.67741 2.69355 4.24690 2.90347 2.73739 3.18146 2.89801 2.37887 2.77519 2.98518 4.58477 3.61503

0.01747 4.45155 5.17389 0.61958 0.77255 0.48576 0.95510

791 2.30457 5.10338 3.12807 2.56844 4.42388 3.57117 1.93139 3.84893 2.08082 3.38159 4.17975 3.07923 3.96416 2.84962 2.32912 2.58343 3.03041 3.48869 5.54542 4.21995 909 - -

2.68618 4.42225 2.77519 2.73123 3.46354 2.40513 3.72494 3.29354 2.67741 2.69355 4.24690 2.90347 2.73739 3.18146 2.89801 2.37887 2.77519 2.98518 4.58477 3.61503

0.01747 4.45155 5.17389 0.61958 0.77255 0.48576 0.95510

792 2.39770 5.03357 2.79878 2.20779 4.31653 2.06812 3.69307 3.75844 2.46203 3.18334 4.09713 2.98718 3.88608 2.71378 2.68018 2.69546 2.93035 2.92242 5.51023 4.14231 910 - -

2.68618 4.42225 2.77519 2.73123 3.46354 2.40513 3.72494 3.29354 2.67741 2.69355 4.24690 2.90347 2.73739 3.18146 2.89801 2.37887 2.77519 2.98518 4.58477 3.61503

0.01747 4.45155 5.17389 0.61958 0.77255 0.48576 0.95510

793 4.45925 5.59199 5.06990 4.94203 3.49588 4.46748 4.79345 4.56509 4.68988 3.84196 5.16132 4.99588 5.03651 5.04140 4.70181 4.65845 4.79682 4.46620 0.24156 3.47448 911 - -

2.68618 4.42225 2.77519 2.73123 3.46354 2.40513 3.72494 3.29354 2.67741 2.69355 4.24690 2.90347 2.73739 3.18146 2.89801 2.37887 2.77519 2.98518 4.58477 3.61503

0.01747 4.45155 5.17389 0.61958 0.77255 0.48576 0.95510

794 3.04766 4.42059 4.76756 4.17618 2.99355 4.23348 3.96098 1.45255 4.01593 1.27486 2.81410 4.30556 4.53200 4.14674 4.09774 3.54827 3.27400 2.41296 4.98046 3.82432 912 - -

2.68618 4.42225 2.77519 2.73123 3.46354 2.40513 3.72494 3.29354 2.67741 2.69355 4.24690 2.90347 2.73739 3.18146 2.89801 2.37887 2.77519 2.98518 4.58477 3.61503

0.01747 4.45155 5.17389 0.61958 0.77255 0.48576 0.95510

795 2.53594 5.07432 3.02667 2.49713 4.42463 3.49970 3.71815 3.86790 2.29274 3.40100 4.18188 2.50602 3.91336 2.83943 2.29065 1.71756 2.86934 3.48228 5.57194 4.21385 913 - -

2.68618 4.42225 2.77519 2.73123 3.46354 2.40513 3.72494 3.29354 2.67741 2.69355 4.24690 2.90347 2.73739 3.18146 2.89801 2.37887 2.77519 2.98518 4.58477 3.61503

0.01747 4.45155 5.17389 0.61958 0.77255 0.48576 0.95510

796 2.46380 4.75816 2.98269 2.61054 2.48881 3.54389 3.75777 3.36220 2.52683 2.66324 3.83979 3.11195 3.93280 2.40806 2.81292 2.27557 2.91611 3.07213 5.28422 3.97802 914 - -

2.68618 4.42225 2.77519 2.73123 3.46354 2.40513 3.72494 3.29354 2.67741 2.69355 4.24690 2.90347 2.73739 3.18146 2.89801 2.37887 2.77519 2.98518 4.58477 3.61503

0.01747 4.45155 5.17389 0.61958 0.77255 0.48576 0.95510

797 2.88230 5.38890 1.73219 2.37149 4.69270 3.47001 2.45615 4.19549 2.60189 3.68272 4.45927 1.94540 3.95467 2.91215 2.82916 2.82172 3.12918 3.77100 5.81974 4.38573 915 - -

2.68618 4.42225 2.77519 2.73123 3.46354 2.40513 3.72494 3.29354 2.67741 2.69355 4.24690 2.90347 2.73739 3.18146 2.89801 2.37887 2.77519 2.98518 4.58477 3.61503

0.31665 4.45155 1.34802 0.61958 0.77255 0.48576 0.95510

798 2.66426 4.67150 3.10295 2.62514 3.73839 3.47413 3.02655 3.31268 2.57543 2.94909 3.81525 3.10005 1.94808 2.96047 2.97086 2.75209 2.71457 3.02883 5.11923 3.25988 916 - -

2.68625 4.42232 2.77521 2.73081 3.46361 2.40509 3.72502 3.29361 2.67748 2.69362 4.24697 2.90354 2.73714 3.18154 2.89808 2.37885 2.77527 2.98526 4.58484 3.61510

0.80647 0.82371 2.16483 0.13684 2.05658 0.31761 1.30153

799 2.68062 4.69849 3.17886 2.64246 2.38220 3.53696 3.76448 3.29574 2.60373 2.94543 3.79579 2.76626 2.40591 2.68020 2.80676 2.77050 2.80986 3.01634 5.22943 3.93075 918 - -

2.68618 4.42225 2.77519 2.73123 3.46354 2.40513 3.72494 3.29354 2.67741 2.69355 4.24690 2.90347 2.73739 3.18146 2.89801 2.37887 2.77519 2.98518 4.58477 3.61503
0.01901 4.36822 5.09057 0.61958 0.77255 0.60821 0.78597

800 2.76509 4.77774 3.30733 2.97347 4.51380 2.88617 4.12100 3.96337 2.84187 3.56599 4.41436 3.36142 1.05032 3.32437 2.57608 2.87061 3.13868 3.53797 5.72503 4.48336 919 - -
2.68618 4.42225 2.77519 2.73123 3.46354 2.40513 3.72494 3.29354 2.67741 2.69355 4.24690 2.90347 2.73739 3.18146 2.89801 2.37887 2.77519 2.98518 4.58477 3.61503
0.01901 4.36822 5.09057 0.61958 0.77255 0.60821 0.78597

801 2.42005 4.30198 3.66514 3.09785 3.43694 3.66095 3.94027 1.87448 3.03479 2.37127 2.94310 2.79476 3.74029 3.32448 3.35203 2.52677 2.88853 2.55226 4.90487 3.68393 920 - -
2.68618 4.42225 2.77519 2.73123 3.46354 2.40513 3.72494 3.29354 2.67741 2.69355 4.24690 2.90347 2.73739 3.18146 2.89801 2.37887 2.77519 2.98518 4.58477 3.61503
0.01901 4.36822 5.09057 0.61958 0.77255 0.41616 1.07755

802 2.68268 4.72181 3.19293 2.51683 3.19288 2.06424 3.77301 2.95506 2.62333 2.96766 3.81148 2.91240 3.94081 2.96535 2.79122 2.43622 2.72744 3.03544 5.25992 3.96144 921 - -
2.68618 4.42225 2.77519 2.73123 3.46354 2.40513 3.72494 3.29354 2.67741 2.69355 4.24690 2.90347 2.73739 3.18146 2.89801 2.37887 2.77519 2.98518 4.58477 3.61503
0.01747 4.45155 5.17389 0.61958 0.77255 0.48576 0.95510

803 2.59508 4.23737 3.83502 3.75914 1.70401 3.93759 4.25513 2.27420 3.63511 2.18510 3.30862 3.94579 4.29683 3.84487 3.79856 3.23625 3.03375 1.74289 4.88312 3.67681 922 - -
2.68684 4.42291 2.77489 2.73076 3.46184 2.40579 3.72561 3.29221 2.67807 2.69421 4.24674 2.90389 2.73692 3.18095 2.89867 2.37873 2.77467 2.98558 4.57822 3.61569
0.39620 1.13486 5.17389 2.04906 0.13795 0.48576 0.95510

804 2.28805 5.08228 3.00196 2.44432 4.38530 3.12596 3.20918 3.48159 2.17959 3.36626 4.13786 2.51330 3.88180 2.79050 2.12956 2.68852 2.75270 3.45088 5.53941 4.16618 932 - -
2.68618 4.42225 2.77519 2.73123 3.46354 2.40513 3.72494 3.29354 2.67741 2.69355 4.24690 2.90347 2.73739 3.18146 2.89801 2.37887 2.77519 2.98518 4.58477 3.61503
0.24660 4.45155 1.57559 0.61958 0.77255 0.48576 0.95510

805 1.93575 4.38504 3.44121 2.99185 4.24577 2.79779 4.08781 3.66083 3.00010 3.31519 4.13460 3.29256 2.65365 3.30945 3.38525 2.17219 1.65282 3.20358 5.57081 4.31932 933 - -
2.68618 4.42225 2.77519 2.73123 3.46354 2.40513 3.72494 3.29354 2.67741 2.69355 4.24690 2.90347 2.73739 3.18146 2.89801 2.37887 2.77519 2.98518 4.58477 3.61503
0.02193 4.22687 4.94922 0.61958 0.77255 0.49383 0.94234

806 2.31182 3.49782 3.06577 2.71569 4.07957 1.69107 3.85839 3.48353 2.70496 3.12307 3.95397 3.16708 3.37477 3.04652 2.75127 2.69473 2.89708 3.14277 5.39348 4.09895 934 - -
2.68618 4.42225 2.77519 2.73123 3.46354 2.40513 3.72494 3.29354 2.67741 2.69355 4.24690 2.90347 2.73739 3.18146 2.89801 2.37887 2.77519 2.98518 4.58477 3.61503
0.01904 4.36658 5.08893 0.61958 0.77255 0.41505 1.07972

807 2.30369 4.50589 3.50742 3.26200 4.64034 0.89088 4.43503 4.07232 3.40768 3.73781 4.55277 2.93767 3.97057 3.68397 3.75543 2.50461 3.00988 3.17789 5.96061 4.72656 935 - -
2.68618 4.42225 2.77519 2.73123 3.46354 2.40513 3.72494 3.29354 2.67741 2.69355 4.24690 2.90347 2.73739 3.18146 2.89801 2.37887 2.77519 2.98518 4.58477 3.61503
0.01747 4.45155 5.17389 0.61958 0.77255 0.48576 0.95510

808 2.83691 5.22064 3.05034 2.31264 4.57292 3.23437 3.71726 4.01180 1.81804 3.50299 4.28490 3.04818 1.99812 2.83219 2.32033 2.81144 3.05829 3.62073 5.63204 4.28919 936 - -
2.68618 4.42225 2.77519 2.73123 3.46354 2.40513 3.72494 3.29354 2.67741 2.69355 4.24690 2.90347 2.73739 3.18146 2.89801 2.37887 2.77519 2.98518 4.58477 3.61503
0.01747 4.45155 5.17389 0.61958 0.77255 0.48576 0.95510

809 2.35213 4.51159 3.40700 2.53972 3.67238 3.61956 3.86392 2.72949 2.81538 2.48324 3.61282 2.92161 2.71990 3.13620 3.20643 2.85478 2.91715 2.04142 5.08619 3.83099 937 - -
2.68618 4.42225 2.77519 2.73123 3.46354 2.40513 3.72494 3.29354 2.67741 2.69355 4.24690 2.90347 2.73739 3.18146 2.89801 2.37887 2.77519 2.98518 4.58477 3.61503
0.01747 4.45155 5.17389 0.61958 0.77255 0.48576 0.95510

810 3.49060 5.56039 4.04824 3.20717 5.14457 4.03712 3.83777 4.42547 1.10751 3.77246 4.65868 3.54810 4.36513 2.96372 1.18059 3.46440 3.60586 4.11245 5.73116 4.64383 938 - -
2.68618 4.42225 2.77519 2.73123 3.46354 2.40513 3.72494 3.29354 2.67741 2.69355 4.24690 2.90347 2.73739 3.18146 2.89801 2.37887 2.77519 2.98518 4.58477 3.61503
0.01747 4.45155 5.17389 0.61958 0.77255 0.48576 0.95510

811 2.72293 4.52009 3.48710 2.91824 3.67953 3.66299 3.89804 2.34262 2.80868 2.71157 3.61576 3.35023 4.04558 2.03437 2.78617 2.90794 2.77070 2.05178 5.10143 3.85589 939 - -
2.68618 4.42225 2.77519 2.73123 3.46354 2.40513 3.72494 3.29354 2.67741 2.69355 4.24690 2.90347 2.73739 3.18146 2.89801 2.37887 2.77519 2.98518 4.58477 3.61503
0.01747 4.45155 5.17389 0.61958 0.77255 0.48576 0.95510
812 2.72948 4.87126 2.73090 2.33638 4.09228 3.52837 3.77135 3.48417 2.60459 1.97871 3.95359 2.15307 3.94171 2.94058 3.06254 2.77598 2.96520 2.75839 5.39006 4.06372 940 - -
2.68618 4.42225 2.77519 2.73123 3.46354 2.40513 3.72494 3.29354 2.67741 2.69355 4.24690 2.90347 2.73739 3.18146 2.89801 2.37887 2.77519 2.98518 4.58477 3.61503
0.01747 4.45155 5.17389 0.61958 0.77255 0.48576 0.95510
813 3.71382 4.98256 5.43065 4.85846 3.23929 5.01648 5.30869 2.41015 4.69858 0.67929 2.01025 5.10009 5.10806 4.69122 4.73398 4.39035 3.92467 2.43485 5.43099 4.44286 941 - -
2.68618 4.42225 2.77519 2.73123 3.46354 2.40513 3.72494 3.29354 2.67741 2.69355 4.24690 2.90347 2.73739 3.18146 2.89801 2.37887 2.77519 2.98518 4.58477 3.61503
0.01747 4.45155 5.17389 0.61958 0.77255 0.48576 0.95510
814 2.95026 4.43755 4.40723 3.87712 3.64489 4.05855 4.58171 2.24270 3.75917 2.04696 3.54540 4.10891 4.48004 4.03673 3.98807 3.40118 1.65129 1.37955 5.32438 4.12237 942 - -
2.68618 4.42225 2.77519 2.73123 3.46354 2.40513 3.72494 3.29354 2.67741 2.69355 4.24690 2.90347 2.73739 3.18146 2.89801 2.37887 2.77519 2.98518 4.58477 3.61503
0.01747 4.45155 5.17389 0.61958 0.77255 0.48576 0.95510
815 2.49057 4.43341 3.80048 3.50050 4.61084 3.21535 4.54097 4.00450 3.51636 3.71761 4.54849 3.61703 3.97392 3.81611 3.81452 1.10425 1.40694 3.44790 5.95649 4.74653 943 - -
2.68618 4.42225 2.77519 2.73123 3.46354 2.40513 3.72494 3.29354 2.67741 2.69355 4.24690 2.90347 2.73739 3.18146 2.89801 2.37887 2.77519 2.98518 4.58477 3.61503
0.01747 4.45155 5.17389 0.61958 0.77255 0.48576 0.95510
816 3.03512 5.05496 3.19257 2.98629 4.34282 1.28099 1.41673 4.20855 3.09018 3.77159 4.65669 3.43068 4.19901 3.48744 3.44546 3.10562 3.39566 3.80085 5.66359 4.27671 944 - -
2.68618 4.42225 2.77519 2.73123 3.46354 2.40513 3.72494 3.29354 2.67741 2.69355 4.24690 2.90347 2.73739 3.18146 2.89801 2.37887 2.77519 2.98518 4.58477 3.61503
0.01747 4.45155 5.17389 0.61958 0.77255 0.48576 0.95510
817 2.61925 4.40758 4.02223 3.65181 4.07536 3.43597 4.52551 3.03306 3.59703 3.07771 4.05699 3.77435 4.11733 3.89467 3.85549 1.31065 3.05199 1.35517 5.58649 4.35523 945 - -
2.68618 4.42225 2.77519 2.73123 3.46354 2.40513 3.72494 3.29354 2.67741 2.69355 4.24690 2.90347 2.73739 3.18146 2.89801 2.37887 2.77519 2.98518 4.58477 3.61503
0.01747 4.45155 5.17389 0.61958 0.77255 0.48576 0.95510
818 2.23822 4.86707 3.07180 2.63222 4.43724 1.78107 3.88278 3.88026 2.68797 3.45513 4.24822 3.11461 3.39758 2.15210 3.14298 2.34677 2.98016 3.45992 5.65683 4.31617 946 - -
2.68618 4.42225 2.77519 2.73123 3.46354 2.40513 3.72494 3.29354 2.67741 2.69355 4.24690 2.90347 2.73739 3.18146 2.89801 2.37887 2.77519 2.98518 4.58477 3.61503
0.01747 4.45155 5.17389 0.61958 0.77255 0.48576 0.95510
819 2.25142 4.47073 3.64839 3.45226 4.71548 3.21269 4.57765 4.13964 3.56718 3.83159 4.66033 3.59558 0.84570 3.85566 3.86153 2.26065 3.02768 3.54297 6.04900 4.83804 947 - -
2.68618 4.42225 2.77519 2.73123 3.46354 2.40513 3.72494 3.29354 2.67741 2.69355 4.24690 2.90347 2.73739 3.18146 2.89801 2.37887 2.77519 2.98518 4.58477 3.61503
0.01747 4.45155 5.17389 0.61958 0.77255 0.48576 0.95510
820 2.39275 5.01678 3.01032 2.36702 4.31570 2.94046 3.70113 3.75563 2.19660 3.31214 4.09754 3.00084 3.46804 2.82722 2.78179 2.60662 1.89049 3.38808 5.50919 4.14677 948 - -
2.68618 4.42225 2.77519 2.73123 3.46354 2.40513 3.72494 3.29354 2.67741 2.69355 4.24690 2.90347 2.73739 3.18146 2.89801 2.37887 2.77519 2.98518 4.58477 3.61503
0.01747 4.45155 5.17389 0.61958 0.77255 0.48576 0.95510
821 2.68220 4.59572 3.36346 2.81318 3.83689 3.56309 3.86707 3.21165 2.73948 2.88756 2.32439 3.26028 2.09352 3.10260 2.76988 2.52684 2.64590 2.94313 5.21055 3.94564 949 - -
2.68618 4.42225 2.77519 2.73123 3.46354 2.40513 3.72494 3.29354 2.67741 2.69355 4.24690 2.90347 2.73739 3.18146 2.89801 2.37887 2.77519 2.98518 4.58477 3.61503
0.01747 4.45155 5.17389 0.61958 0.77255 0.48576 0.95510
822 3.09195 1.52071 4.80371 4.31503 3.48311 4.10411 4.76734 2.50912 4.09584 1.18383 3.43583 4.38655 4.57793 4.32970 4.21030 3.53100 3.40776 2.43954 5.27816 4.07258 950 - -
2.68618 4.42225 2.77519 2.73123 3.46354 2.40513 3.72494 3.29354 2.67741 2.69355 4.24690 2.90347 2.73739 3.18146 2.89801 2.37887 2.77519 2.98518 4.58477 3.61503

0.01747 4.45155 5.17389 0.61958 0.77255 0.48576 0.95510

823 2.04518 5.16190 2.39950 2.23734 4.48904 3.21500 3.66296 3.96259 2.17732 3.46038 4.21054 2.93910 3.56004 2.76709 2.72883 2.47151 2.65469 3.54355 5.60739 4.20649 951 - -

2.68618 4.42225 2.77519 2.73123 3.46354 2.40513 3.72494 3.29354 2.67741 2.69355 4.24690 2.90347 2.73739 3.18146 2.89801 2.37887 2.77519 2.98518 4.58477 3.61503

0.01747 4.45155 5.17389 0.61958 0.77255 0.48576 0.95510

824 2.72987 5.02179 2.56242 2.47076 4.29254 3.17386 3.73040 3.72662 2.33212 3.29937 2.11333 2.29092 3.91119 2.86742 2.99782 2.59354 2.96658 3.37478 5.51348 4.15303 952 - -

2.68618 4.42225 2.77519 2.73123 3.46354 2.40513 3.72494 3.29354 2.67741 2.69355 4.24690 2.90347 2.73739 3.18146 2.89801 2.37887 2.77519 2.98518 4.58477 3.61503

0.01747 4.45155 5.17389 0.61958 0.77255 0.48576 0.95510

825 2.61689 4.52547 3.42815 2.74215 1.83910 3.62346 3.98733 3.06810 3.01345 2.76313 3.68922 3.40356 1.97950 3.32529 3.36762 2.94212 3.01657 2.83651 5.08170 3.77416 953 - -

2.68618 4.42225 2.77519 2.73123 3.46354 2.40513 3.72494 3.29354 2.67741 2.69355 4.24690 2.90347 2.73739 3.18146 2.89801 2.37887 2.77519 2.98518 4.58477 3.61503

0.01747 4.45155 5.17389 0.61958 0.77255 0.48576 0.95510

826 2.45592 4.28825 3.46009 2.99888 3.41357 3.32039 3.99610 2.19608 3.14110 1.71109 3.39978 3.54919 4.09389 3.41642 3.43512 2.98072 2.64240 2.32161 4.89400 3.68021 954 - -

2.68618 4.42225 2.77519 2.73123 3.46354 2.40513 3.72494 3.29354 2.67741 2.69355 4.24690 2.90347 2.73739 3.18146 2.89801 2.37887 2.77519 2.98518 4.58477 3.61503

0.01747 4.45155 5.17389 0.61958 0.77255 0.48576 0.95510

827 2.52181 4.48027 3.67612 3.52120 4.86854 1.05973 4.67510 4.37466 3.71989 4.03077 4.82985 3.63434 3.99483 3.95936 4.01470 1.33294 3.04943 3.67566 6.17205 4.97779 955 - -

2.68618 4.42225 2.77519 2.73123 3.46354 2.40513 3.72494 3.29354 2.67741 2.69355 4.24690 2.90347 2.73739 3.18146 2.89801 2.37887 2.77519 2.98518 4.58477 3.61503

0.01747 4.45155 5.17389 0.61958 0.77255 0.48576 0.95510

828 2.77967 5.12966 1.72186 2.45238 4.51886 2.15890 3.80597 3.97364 2.63168 3.52073 4.30899 2.64964 3.93317 2.93999 3.13030 2.76775 2.65539 3.03142 5.70710 4.31623 956 - -

2.68618 4.42225 2.77519 2.73123 3.46354 2.40513 3.72494 3.29354 2.67741 2.69355 4.24690 2.90347 2.73739 3.18146 2.89801 2.37887 2.77519 2.98518 4.58477 3.61503

0.01747 4.45155 5.17389 0.61958 0.77255 0.48576 0.95510

829 3.15166 5.01579 3.47891 3.10433 2.98678 3.86074 1.06573 3.69771 2.97459 3.22060 4.20662 3.49488 4.30801 3.40134 3.28908 2.87185 3.40529 3.44838 4.56941 2.50283 957 - -

2.68618 4.42225 2.77519 2.73123 3.46354 2.40513 3.72494 3.29354 2.67741 2.69355 4.24690 2.90347 2.73739 3.18146 2.89801 2.37887 2.77519 2.98518 4.58477 3.61503

0.01747 4.45155 5.17389 0.61958 0.77255 0.48576 0.95510

830 3.67156 5.00876 4.74980 4.36587 2.46413 4.53051 3.95172 3.21072 4.13180 1.65607 3.85791 4.32119 4.86015 4.28733 4.23057 3.91153 3.90511 3.18177 4.15623 0.87606 958 - -

2.68618 4.42225 2.77519 2.73123 3.46354 2.40513 3.72494 3.29354 2.67741 2.69355 4.24690 2.90347 2.73739 3.18146 2.89801 2.37887 2.77519 2.98518 4.58477 3.61503

0.01747 4.45155 5.17389 0.61958 0.77255 0.48576 0.95510

831 1.70782 4.46829 4.40343 3.88076 3.53040 4.00472 4.55881 2.41529 3.76162 1.26810 3.42052 4.09537 4.44309 4.01339 3.97412 3.35610 3.23820 2.05279 5.26002 4.08615 959 - -

2.68640 4.42247 2.77345 2.73145 3.46376 2.40453 3.72516 3.29376 2.67746 2.69377 4.24712 2.90344 2.73762 3.18168 2.89823 2.37909 2.77542 2.98540 4.58499 3.61525

1.03953 1.13486 1.12421 1.03028 0.44147 0.48576 0.95510

832 3.33087 4.64959 4.91714 4.34940 3.10912 4.60238 4.90365 1.85508 4.20675 0.94178 2.41143 4.61303 4.77194 4.31250 4.32506 3.94917 3.55034 2.23372 5.20391 4.14998 963 - -

2.68618 4.42225 2.77519 2.73123 3.46354 2.40513 3.72494 3.29354 2.67741 2.69355 4.24690 2.90347 2.73739 3.18146 2.89801 2.37887 2.77519 2.98518 4.58477 3.61503

0.02578 4.06699 4.78934 0.61958 0.77255 0.29184 1.37393

833 2.72029 4.28049 3.93178 3.36317 3.06233 3.77174 4.07894 2.64824 3.27506 1.64504 3.37258 3.12167 4.15339 3.53998 3.53602 3.05079 1.97026 2.24197 4.89796 3.68603 964 - -

2.68618 4.42225 2.77519 2.73123 3.46354 2.40513 3.72494 3.29354 2.67741 2.69355 4.24690 2.90347 2.73739 3.18146 2.89801 2.37887 2.77519 2.98518 4.58477 3.61503

0.01747 4.45155 5.17389 0.61958 0.77255 0.48576 0.95510

834 2.81699 5.08864 3.18427 2.59720 4.40511 3.59114 3.72248 3.81853 1.82680 2.89562 4.16060 3.10118 2.36664 2.85078 2.05413 2.82102 2.71631 3.46765 5.52522 4.21199 965 - -

2.68618 4.42225 2.77519 2.73123 3.46354 2.40513 3.72494 3.29354 2.67741 2.69355 4.24690 2.90347 2.73739 3.18146 2.89801 2.37887 2.77519 2.98518 4.58477 3.61503
0.01747 4.45155 5.17389 0.61958 0.77255 0.48576 0.95510
835 2.23158 4.18645 3.18100 2.62229 3.23627 3.54190 3.76429 3.35342 1.85701 2.82682 3.83530 2.81870 3.93385 2.94871 3.04498 2.41443 2.80193 3.06359 5.28112 3.97740 966 - -
2.68618 4.42225 2.77519 2.73123 3.46354 2.40513 3.72494 3.29354 2.67741 2.69355 4.24690 2.90347 2.73739 3.18146 2.89801 2.37887 2.77519 2.98518 4.58477 3.61503
0.01747 4.45155 5.17389 0.61958 0.77255 0.48576 0.95510
836 2.79876 4.18815 4.58123 3.98422 1.86427 3.96413 4.27219 2.04140 3.81226 1.88187 3.20217 4.06054 4.31240 3.96587 3.88326 3.27228 2.73028 2.01861 4.78422 3.04230 967 - -
2.68618 4.42225 2.77519 2.73123 3.46354 2.40513 3.72494 3.29354 2.67741 2.69355 4.24690 2.90347 2.73739 3.18146 2.89801 2.37887 2.77519 2.98518 4.58477 3.61503
0.01747 4.45155 5.17389 0.61958 0.77255 0.48576 0.95510
837 2.81821 4.92282 3.26326 2.42298 4.14824 3.62951 3.77792 3.50863 1.45997 2.22930 3.43922 3.17711 4.01182 2.94061 2.79333 2.87051 3.03751 3.22737 5.39903 4.11298 968 - -
2.68618 4.42225 2.77519 2.73123 3.46354 2.40513 3.72494 3.29354 2.67741 2.69355 4.24690 2.90347 2.73739 3.18146 2.89801 2.37887 2.77519 2.98518 4.58477 3.61503
0.01747 4.45155 5.17389 0.61958 0.77255 0.48576 0.95510
838 2.81009 5.12297 2.80913 2.22894 4.52430 3.45835 3.83957 3.97358 2.65962 3.52882 4.32924 1.62621 3.95817 2.98217 3.13852 2.50132 2.00361 3.57938 5.72046 4.34121 969 - -
2.68618 4.42225 2.77519 2.73123 3.46354 2.40513 3.72494 3.29354 2.67741 2.69355 4.24690 2.90347 2.73739 3.18146 2.89801 2.37887 2.77519 2.98518 4.58477 3.61503
0.01747 4.45155 5.17389 0.61958 0.77255 0.48576 0.95510
839 3.05720 5.19697 3.22805 2.87501 4.81744 1.65619 4.02490 4.25772 1.13252 3.77040 4.61537 3.33229 4.16212 3.18264 2.85972 3.08272 3.34935 3.85055 5.83745 4.59631 970 - -
2.68618 4.42225 2.77519 2.73123 3.46354 2.40513 3.72494 3.29354 2.67741 2.69355 4.24690 2.90347 2.73739 3.18146 2.89801 2.37887 2.77519 2.98518 4.58477 3.61503
0.01747 4.45155 5.17389 0.61958 0.77255 0.48576 0.95510
840 2.86777 4.50047 3.82255 1.80866 3.64445 3.91182 4.19338 1.90394 3.20980 2.35992 3.32638 3.66597 4.27357 3.51884 3.55484 3.17289 3.10302 1.85217 5.21569 3.98785 971 - -
2.68618 4.42225 2.77519 2.73123 3.46354 2.40513 3.72494 3.29354 2.67741 2.69355 4.24690 2.90347 2.73739 3.18146 2.89801 2.37887 2.77519 2.98518 4.58477 3.61503
0.01747 4.45155 5.17389 0.61958 0.77255 0.48576 0.95510
841 2.93799 5.18961 1.48137 2.51156 4.09950 3.52791 3.87275 3.90363 2.81321 3.47315 4.32892 3.05294 3.18419 3.08320 3.30902 2.92607 3.19933 3.56597 5.47470 1.93793 972 - -
2.68618 4.42225 2.77519 2.73123 3.46354 2.40513 3.72494 3.29354 2.67741 2.69355 4.24690 2.90347 2.73739 3.18146 2.89801 2.37887 2.77519 2.98518 4.58477 3.61503
0.01747 4.45155 5.17389 0.61958 0.77255 0.48576 0.95510
842 4.30522 5.38099 5.16496 4.96892 2.13597 4.86989 3.70938 4.01830 4.70965 3.24896 4.55848 4.48210 5.16230 4.59490 4.64672 4.28581 4.51380 3.93821 1.32228 0.92839 973 - -
2.68618 4.42225 2.77519 2.73123 3.46354 2.40513 3.72494 3.29354 2.67741 2.69355 4.24690 2.90347 2.73739 3.18146 2.89801 2.37887 2.77519 2.98518 4.58477 3.61503
0.01747 4.45155 5.17389 0.61958 0.77255 0.48576 0.95510
843 2.61573 1.72648 3.64678 3.11108 4.18958 3.56125 4.05175 3.46415 1.46423 3.19387 4.08602 3.46038 4.08982 3.27369 2.96656 2.92500 3.09876 3.15849 5.48739 4.26003 974 - -
2.68618 4.42225 2.77519 2.73123 3.46354 2.40513 3.72494 3.29354 2.67741 2.69355 4.24690 2.90347 2.73739 3.18146 2.89801 2.37887 2.77519 2.98518 4.58477 3.61503
0.01747 4.45155 5.17389 0.61958 0.77255 0.48576 0.95510
844 3.49274 5.56086 4.05211 3.20967 5.14553 4.03850 3.83827 4.42596 1.13656 3.77281 4.65946 3.54978 4.36636 2.96432 1.14858 3.46668 3.60762 4.11335 5.73125 4.64464 975 - -
2.68618 4.42225 2.77519 2.73123 3.46354 2.40513 3.72494 3.29354 2.67741 2.69355 4.24690 2.90347 2.73739 3.18146 2.89801 2.37887 2.77519 2.98518 4.58477 3.61503
0.01747 4.45155 5.17389 0.61958 0.77255 0.48576 0.95510
845 3.41294 4.65022 5.36522 4.84906 3.71506 4.89582 5.43181 1.51748 4.74960 1.56829 3.48774 5.01539 5.11359 4.91854 4.86945 4.28691 3.65958 1.01711 5.75668 4.60932 976 - -
2.68618 4.42225 2.77519 2.73123 3.46354 2.40513 3.72494 3.29354 2.67741 2.69355 4.24690 2.90347 2.73739 3.18146 2.89801 2.37887 2.77519 2.98518 4.58477 3.61503
0.01747 4.45155 5.17389 0.61958 0.77255 0.48576 0.95510

846 2.86274 4.64909 3.55806 3.03003 2.79096 3.75878 1.42296 3.17575 2.93923 2.81205 3.05210 3.10685 4.14833 3.28718 3.28915 3.02153 3.09745 2.95186 4.74436 3.24895 977 - -
2.68618 4.42225 2.77519 2.73123 3.46354 2.40513 3.72494 3.29354 2.67741 2.69355 4.24690 2.90347 2.73739 3.18146 2.89801 2.37887 2.77519 2.98518 4.58477 3.61503
0.01747 4.45155 5.17389 0.61958 0.77255 0.48576 0.95510
847 3.53934 5.21843 4.32571 4.31233 5.37106 0.21600 5.35526 5.15171 4.56828 4.71276 5.71970 4.49308 4.62145 4.84753 4.70350 3.73304 4.06234 4.56781 6.27450 5.50520 978 - -
2.68618 4.42225 2.77519 2.73123 3.46354 2.40513 3.72494 3.29354 2.67741 2.69355 4.24690 2.90347 2.73739 3.18146 2.89801 2.37887 2.77519 2.98518 4.58477 3.61503
0.01747 4.45155 5.17389 0.61958 0.77255 0.48576 0.95510
848 2.70956 4.26846 3.90750 3.33285 3.10258 3.76481 3.32872 2.07477 3.24530 2.25323 3.35580 3.63831 2.05654 3.50888 3.50778 3.03620 2.94230 2.24053 4.85941 3.64132 979 - -
2.68618 4.42225 2.77519 2.73123 3.46354 2.40513 3.72494 3.29354 2.67741 2.69355 4.24690 2.90347 2.73739 3.18146 2.89801 2.37887 2.77519 2.98518 4.58477 3.61503
0.01747 4.45155 5.17389 0.61958 0.77255 0.48576 0.95510
849 2.84250 5.17765 2.24935 1.75772 4.44316 3.48972 3.80293 3.86092 2.64462 3.44698 4.25982 2.98337 3.96083 2.74298 3.14175 2.82347 3.08814 1.85794 5.66475 4.27922 980 - -
2.68618 4.42225 2.77519 2.73123 3.46354 2.40513 3.72494 3.29354 2.67741 2.69355 4.24690 2.90347 2.73739 3.18146 2.89801 2.37887 2.77519 2.98518 4.58477 3.61503
0.01747 4.45155 5.17389 0.61958 0.77255 0.48576 0.95510
850 3.37331 4.66424 5.20769 4.64514 1.57167 4.66578 4.92808 1.41925 4.50074 1.61824 3.17912 4.76207 4.88653 4.56093 4.54571 4.01518 3.60000 2.14016 5.20589 3.99790 981 - -
2.68618 4.42225 2.77519 2.73123 3.46354 2.40513 3.72494 3.29354 2.67741 2.69355 4.24690 2.90347 2.73739 3.18146 2.89801 2.37887 2.77519 2.98518 4.58477 3.61503
0.01747 4.45155 5.17389 0.61958 0.77255 0.48576 0.95510
851 3.19319 4.51712 5.05711 4.46633 2.15207 4.44054 4.76867 2.07707 4.30414 1.62365 1.90911 4.56296 4.70037 4.37904 4.33777 3.77194 3.41844 1.68940 5.12718 4.01729 982 - -
2.68618 4.42225 2.77519 2.73123 3.46354 2.40513 3.72494 3.29354 2.67741 2.69355 4.24690 2.90347 2.73739 3.18146 2.89801 2.37887 2.77519 2.98518 4.58477 3.61503
0.01747 4.45155 5.17389 0.61958 0.77255 0.48576 0.95510
852 4.05084 5.35520 4.54595 4.30959 2.25270 4.60921 2.88692 4.03561 4.09481 3.30869 4.56606 4.23382 4.97336 4.28071 4.20701 4.04469 4.28121 3.91382 3.91920 0.54404 983 - -
2.68618 4.42225 2.77519 2.73123 3.46354 2.40513 3.72494 3.29354 2.67741 2.69355 4.24690 2.90347 2.73739 3.18146 2.89801 2.37887 2.77519 2.98518 4.58477 3.61503
0.01747 4.45155 5.17389 0.61958 0.77255 0.48576 0.95510
853 2.73789 4.65901 3.27352 2.86062 3.91298 3.53833 3.96888 3.32193 2.86470 1.67063 3.90169 1.93269 4.03232 3.22056 3.24409 2.29342 3.02783 3.04588 5.31233 4.01183 984 - -
2.68618 4.42225 2.77519 2.73123 3.46354 2.40513 3.72494 3.29354 2.67741 2.69355 4.24690 2.90347 2.73739 3.18146 2.89801 2.37887 2.77519 2.98518 4.58477 3.61503
0.01747 4.45155 5.17389 0.61958 0.77255 0.48576 0.95510
854 2.73119 4.65029 3.34327 2.83180 3.86066 3.59186 3.90801 3.17399 2.77297 2.74230 2.49521 1.59996 4.02491 3.14088 3.16003 2.86563 2.64028 2.92597 5.26143 3.98197 985 - -
2.68618 4.42225 2.77519 2.73123 3.46354 2.40513 3.72494 3.29354 2.67741 2.69355 4.24690 2.90347 2.73739 3.18146 2.89801 2.37887 2.77519 2.98518 4.58477 3.61503
0.01747 4.45155 5.17389 0.61958 0.77255 0.48576 0.95510
855 1.21975 4.38307 3.93292 3.61071 4.65954 3.17841 4.60614 4.05268 3.62154 3.76191 4.57327 3.65675 3.95303 3.88913 3.90994 1.17870 2.63792 3.45597 6.00316 4.82026 986 - -
2.68618 4.42225 2.77519 2.73123 3.46354 2.40513 3.72494 3.29354 2.67741 2.69355 4.24690 2.90347 2.73739 3.18146 2.89801 2.37887 2.77519 2.98518 4.58477 3.61503
0.01747 4.45155 5.17389 0.61958 0.77255 0.48576 0.95510
856 2.47179 4.60692 3.29791 2.74007 3.48008 3.58200 3.08729 3.16504 2.71607 2.19164 3.70387 2.59654 3.97088 3.04789 3.13172 2.06730 2.91473 2.75647 5.15974 3.88226 987 - -
2.68618 4.42225 2.77519 2.73123 3.46354 2.40513 3.72494 3.29354 2.67741 2.69355 4.24690 2.90347 2.73739 3.18146 2.89801 2.37887 2.77519 2.98518 4.58477 3.61503
0.01747 4.45155 5.17389 0.61958 0.77255 0.48576 0.95510
857 2.73465 4.28185 3.99411 3.43378 3.27446 3.77018 4.05594 2.73749 3.29349 1.50242 3.38742 3.69593 3.27960 3.57730 3.52347 2.71461 2.97845 2.54743 2.41437 3.52654 988 - -
2.68618 4.42225 2.77519 2.73123 3.46354 2.40513 3.72494 3.29354 2.67741 2.69355 4.24690 2.90347 2.73739 3.18146 2.89801 2.37887 2.77519 2.98518 4.58477 3.61503

0.01747 4.45155 5.17389 0.61958 0.77255 0.48576 0.95510

858 2.82130 5.29413 1.47996 2.39592 4.61250 3.47914 3.14452 4.09205 2.51373 3.58661 4.35687 2.94979 3.93009 2.65620 2.49452 2.51034 3.06062 3.67647 5.73079 4.32112 989 - -

2.68624 4.42231 2.77526 2.73129 3.46360 2.40465 3.72501 3.29360 2.67747 2.69361 4.24696 2.90353 2.73746 3.18152 2.89783 2.37893 2.77526 2.98524 4.58483 3.61509

0.39620 1.30133 2.90116 0.15827 1.92156 0.48576 0.95510

859 2.28592 5.03300 2.96561 2.26656 4.34848 3.00907 3.68181 3.79712 2.33503 3.34065 4.11745 2.96943 2.85509 2.80250 2.80327 2.00376 2.80226 3.41518 5.52619 4.15316 991 - -

2.68618 4.42225 2.77519 2.73123 3.46354 2.40513 3.72494 3.29354 2.67741 2.69355 4.24690 2.90347 2.73739 3.18146 2.89801 2.37887 2.77519 2.98518 4.58477 3.61503

0.01839 4.40099 5.12334 0.61958 0.77255 0.56302 0.84277

860 2.83478 5.37050 1.81397 1.66059 4.69612 3.44136 3.74541 4.19072 2.31461 3.66593 4.42982 2.66175 3.91578 2.86202 2.80257 2.59509 3.07945 3.75326 5.80149 4.36437 992 - -

2.68618 4.42225 2.77519 2.73123 3.46354 2.40513 3.72494 3.29354 2.67741 2.69355 4.24690 2.90347 2.73739 3.18146 2.89801 2.37887 2.77519 2.98518 4.58477 3.61503

0.01839 4.40099 5.12334 0.61958 0.77255 0.44031 1.03236

861 2.76693 4.41302 3.77501 3.20114 3.47410 3.76556 4.02852 2.80744 3.05024 1.84775 2.16019 3.56365 2.36304 3.39678 2.91655 3.03475 3.00052 2.64025 4.99286 3.78183 993 - -

2.68618 4.42225 2.77519 2.73123 3.46354 2.40513 3.72494 3.29354 2.67741 2.69355 4.24690 2.90347 2.73739 3.18146 2.89801 2.37887 2.77519 2.98518 4.58477 3.61503

0.01747 4.45155 5.17389 0.61958 0.77255 0.48576 0.95510

862 2.23969 4.98573 2.84130 2.47439 4.26734 3.48804 3.17101 3.70284 2.48382 3.02568 4.06361 2.51694 3.88973 2.83576 2.95853 2.31203 2.14207 3.34592 5.48215 4.12316 994 - -

2.68618 4.42225 2.77519 2.73123 3.46354 2.40513 3.72494 3.29354 2.67741 2.69355 4.24690 2.90347 2.73739 3.18146 2.89801 2.37887 2.77519 2.98518 4.58477 3.61503

0.01747 4.45155 5.17389 0.61958 0.77255 0.48576 0.95510

863 2.47518 4.52853 3.43970 2.92232 3.83985 2.10663 3.95296 3.21160 2.87005 1.84135 3.77412 3.33123 3.99749 3.21425 2.97672 2.27179 2.94259 2.93464 5.23302 3.98010 995 - -

2.68618 4.42225 2.77519 2.73123 3.46354 2.40513 3.72494 3.29354 2.67741 2.69355 4.24690 2.90347 2.73739 3.18146 2.89801 2.37887 2.77519 2.98518 4.58477 3.61503

0.01747 4.45155 5.17389 0.61958 0.77255 0.48576 0.95510

864 3.59295 4.87150 4.99995 4.57264 1.87731 4.57447 3.98170 3.06310 4.39901 1.21938 3.64261 4.41639 4.85614 4.38615 4.41203 3.91674 3.81122 3.06263 4.15041 1.44320 996 - -

2.68618 4.42225 2.77519 2.73123 3.46354 2.40513 3.72494 3.29354 2.67741 2.69355 4.24690 2.90347 2.73739 3.18146 2.89801 2.37887 2.77519 2.98518 4.58477 3.61503

0.01747 4.45155 5.17389 0.61958 0.77255 0.48576 0.95510

865 4.45925 5.59199 5.06990 4.94203 3.49588 4.46748 4.79345 4.56509 4.68988 3.84196 5.16132 4.99588 5.03651 5.04140 4.70181 4.65845 4.79682 4.46620 0.24156 3.47448 997 - -

2.68618 4.42225 2.77519 2.73123 3.46354 2.40513 3.72494 3.29354 2.67741 2.69355 4.24690 2.90347 2.73739 3.18146 2.89801 2.37887 2.77519 2.98518 4.58477 3.61503

0.01747 4.45155 5.17389 0.61958 0.77255 0.48576 0.95510

866 1.86613 4.89323 3.06879 2.00523 4.16993 3.50729 3.75114 3.56725 2.40484 3.18526 4.00170 3.06179 3.91981 2.90320 3.00277 2.73720 2.35437 2.85174 5.43100 4.09751 998 - -

2.68524 4.42240 2.77416 2.73138 3.46368 2.40527 3.72509 3.29369 2.67755 2.69369 4.24704 2.90361 2.73754 3.18161 2.89815 2.37901 2.77534 2.98533 4.58492 3.61518

0.39620 1.13486 5.17389 0.68076 0.70569 0.48576 0.95510

867 3.63416 5.52892 3.84445 3.40077 5.05715 4.01036 4.24283 4.53859 0.45175 3.99052 4.95531 3.78421 4.49772 3.42591 2.73971 3.65910 3.85947 4.22655 5.89229 4.81374 1001 - -

2.68618 4.42225 2.77519 2.73123 3.46354 2.40513 3.72494 3.29354 2.67741 2.69355 4.24690 2.90347 2.73739 3.18146 2.89801 2.37887 2.77519 2.98518 4.58477 3.61503

0.01747 4.45155 5.17389 0.61958 0.77255 0.48576 0.95510

868 3.00129 4.40285 4.58914 4.00719 3.37009 4.18403 4.51494 2.05539 3.83758 1.28135 3.27233 4.20053 4.50646 4.06068 3.18718 3.49598 3.23458 1.59034 5.08861 3.92786 1002 - -

2.68618 4.42225 2.77519 2.73123 3.46354 2.40513 3.72494 3.29354 2.67741 2.69355 4.24690 2.90347 2.73739 3.18146 2.89801 2.37887 2.77519 2.98518 4.58477 3.61503

0.05474 4.45155 3.17944 0.61958 0.77255 0.48576 0.95510

869 2.96574 5.10787 3.04586 2.75998 4.56743 3.54824 4.01522 4.07947 2.71615 3.61329 4.48907 3.24200 1.47692 1.55268 3.06807 2.99669 3.27154 3.70240 5.75266 4.44079 1003 - -

2.68618 4.42225 2.77519 2.73123 3.46354 2.40513 3.72494 3.29354 2.67741 2.69355 4.24690 2.90347 2.73739 3.18146 2.89801 2.37887 2.77519 2.98518 4.58477 3.61503
0.01813 4.41494 5.13728 0.61958 0.77255 0.54268 0.87033
870 2.22628 4.35445 4.56521 3.99163 3.38225 4.13657 4.50153 2.26115 3.86155 1.68458 2.32292 4.17414 4.47409 4.06046 4.01297 3.45123 3.19085 1.51699 5.07905 3.91313 1004 - -
2.68618 4.42225 2.77519 2.73123 3.46354 2.40513 3.72494 3.29354 2.67741 2.69355 4.24690 2.90347 2.73739 3.18146 2.89801 2.37887 2.77519 2.98518 4.58477 3.61503
0.13570 4.41494 2.16454 0.61958 0.77255 0.54268 0.87033
871 3.33364 4.58317 5.22473 4.70330 3.59701 4.78861 5.29897 1.12262 4.59938 1.61508 3.37313 4.88859 5.00824 4.77068 4.72925 4.17358 3.57908 1.42385 5.63913 4.49727 1005 - -
2.68618 4.42225 2.77519 2.73123 3.46354 2.40513 3.72494 3.29354 2.67741 2.69355 4.24690 2.90347 2.73739 3.18146 2.89801 2.37887 2.77519 2.98518 4.58477 3.61503
0.02037 4.29961 5.02195 0.61958 0.77255 0.69252 0.69378
872 2.95229 5.09231 3.39857 2.78158 4.49906 3.66283 3.73779 3.86241 1.95884 3.38562 4.22967 3.22362 4.05565 2.87823 1.44501 2.97380 2.05524 3.53402 5.51681 4.28691 1006 - -
2.68618 4.42225 2.77519 2.73123 3.46354 2.40513 3.72494 3.29354 2.67741 2.69355 4.24690 2.90347 2.73739 3.18146 2.89801 2.37887 2.77519 2.98518 4.58477 3.61503
0.02037 4.29961 5.02195 0.61958 0.77255 0.69252 0.69378
873 2.05819 4.41169 3.44344 2.87701 2.42737 3.59182 3.84016 2.92185 2.63054 2.62693 3.25802 3.30027 3.97618 3.14068 2.69255 2.83517 2.59902 2.68986 4.98877 3.74537 1007 - -
2.68618 4.42225 2.77519 2.73123 3.46354 2.40513 3.72494 3.29354 2.67741 2.69355 4.24690 2.90347 2.73739 3.18146 2.89801 2.37887 2.77519 2.98518 4.58477 3.61503
0.02037 4.29961 5.02195 0.61958 0.77255 0.69252 0.69378
874 2.15840 4.56736 3.54175 2.97192 3.74343 3.63588 3.90421 3.03400 2.66457 1.77184 3.66652 3.37401 4.05186 3.16237 2.05326 2.92767 3.00132 2.82237 5.16347 3.92725 1008 - -
2.68618 4.42225 2.77519 2.73123 3.46354 2.40513 3.72494 3.29354 2.67741 2.69355 4.24690 2.90347 2.73739 3.18146 2.89801 2.37887 2.77519 2.98518 4.58477 3.61503
0.02037 4.29961 5.02195 0.61958 0.77255 0.69252 0.69378
875 3.53608 4.85773 5.09112 4.53893 3.16372 4.75719 5.04341 2.35980 4.33755 1.00475 1.43364 4.79468 4.92094 4.45021 4.43170 4.12928 3.75694 2.54236 5.28964 4.22907 1009 - -
2.68618 4.42225 2.77519 2.73123 3.46354 2.40513 3.72494 3.29354 2.67741 2.69355 4.24690 2.90347 2.73739 3.18146 2.89801 2.37887 2.77519 2.98518 4.58477 3.61503
0.02037 4.29961 5.02195 0.61958 0.77255 0.69252 0.69378
876 3.40054 4.66188 5.23200 4.69136 3.41424 4.79770 5.22684 1.36137 4.57030 1.12586 3.18820 4.89025 4.98473 4.67863 4.66762 4.17397 3.63586 1.75521 5.50003 4.41313 1010 - -
2.68658 4.42265 2.77495 2.73163 3.46394 2.40553 3.72535 3.29286 2.67781 2.69335 4.24449 2.90313 2.73780 3.18186 2.89841 2.37840 2.77430 2.98479 4.58517 3.61543
0.24426 1.56004 5.02195 2.04582 0.13843 0.69252 0.69378
877 0.97127 4.29434 3.77219 3.50428 4.53528 3.08699 4.51859 3.91481 3.54436 3.64804 4.47929 3.55851 3.86560 3.81498 3.82103 1.58870 2.87577 3.34049 5.89412 4.70127 1021 - -
2.68618 4.42225 2.77519 2.73123 3.46354 2.40513 3.72494 3.29354 2.67741 2.69355 4.24690 2.90347 2.73739 3.18146 2.89801 2.37887 2.77519 2.98518 4.58477 3.61503
0.02037 4.29961 5.02195 0.61958 0.77255 0.60158 0.79395
878 3.04779 5.23957 3.03349 2.68125 4.14428 3.63209 1.53137 4.01855 2.42763 3.50596 4.38702 3.17138 4.09616 1.79493 2.73619 3.03551 3.27926 3.68785 5.41233 3.98235 1022 - -
2.68618 4.42225 2.77519 2.73123 3.46354 2.40513 3.72494 3.29354 2.67741 2.69355 4.24690 2.90347 2.73739 3.18146 2.89801 2.37887 2.77519 2.98518 4.58477 3.61503
0.01955 4.34060 5.06294 0.61958 0.77255 0.64371 0.74515
879 2.80814 4.55982 3.76873 3.33534 3.71405 3.63802 4.25173 3.00194 3.20645 2.55135 1.87524 3.65219 1.47710 3.58373 3.49330 3.03565 3.13944 2.84694 5.29170 4.05604 1023 - -
2.68618 4.42225 2.77519 2.73123 3.46354 2.40513 3.72494 3.29354 2.67741 2.69355 4.24690 2.90347 2.73739 3.18146 2.89801 2.37887 2.77519 2.98518 4.58477 3.61503
0.01955 4.34060 5.06294 0.61958 0.77255 0.39832 1.11305
880 2.70519 4.77672 2.92177 1.69425 3.66646 3.55488 3.77888 3.33174 2.62088 2.98541 2.43142 3.12225 3.94941 2.96149 3.06664 2.77831 2.93829 2.76178 5.30654 3.99744 1024 - -
2.68618 4.42225 2.77519 2.73123 3.46354 2.40513 3.72494 3.29354 2.67741 2.69355 4.24690 2.90347 2.73739 3.18146 2.89801 2.37887 2.77519 2.98518 4.58477 3.61503
0.01747 4.45155 5.17389 0.61958 0.77255 0.48576 0.95510

881 2.01758 4.24496 4.23108 3.65942 3.41185 3.87906 4.25002 1.60940 3.55008 2.41936 3.16815 3.87804 4.26445 3.78524 3.75108 2.78937 2.81223 1.88430 4.95781 3.76113 1025 - -
2.68618 4.42225 2.77519 2.73123 3.46354 2.40513 3.72494 3.29354 2.67741 2.69355 4.24690 2.90347 2.73739 3.18146 2.89801 2.37887 2.77519 2.98518 4.58477 3.61503
0.01747 4.45155 5.17389 0.61958 0.77255 0.48576 0.95510
882 3.31722 5.82421 2.29167 0.65602 5.15233 3.52019 4.12128 4.69674 3.15912 4.20312 5.09323 3.01477 4.16763 3.30357 3.73862 3.19675 3.62138 4.27105 6.30186 4.83147 1026 - -
2.68618 4.42225 2.77519 2.73123 3.46354 2.40513 3.72494 3.29354 2.67741 2.69355 4.24690 2.90347 2.73739 3.18146 2.89801 2.37887 2.77519 2.98518 4.58477 3.61503
0.01785 4.43070 5.15305 0.61958 0.77255 0.48576 0.95510
883 2.43721 4.33044 3.71502 3.14210 2.97794 3.70455 3.95795 2.79318 2.12525 2.30560 3.42721 3.50201 4.08010 3.35627 3.36425 2.96306 2.92396 2.37780 2.47734 3.65454 1027 - -
2.68618 4.42225 2.77519 2.73123 3.46354 2.40513 3.72494 3.29354 2.67741 2.69355 4.24690 2.90347 2.73739 3.18146 2.89801 2.37887 2.77519 2.98518 4.58477 3.61503
0.01785 4.43070 5.15305 0.61958 0.77255 0.48576 0.95510
884 2.69090 4.62378 3.29594 2.02287 3.79826 3.58915 3.80956 2.36352 2.68426 2.47374 3.70904 3.20738 3.97346 2.74441 2.75315 2.81469 2.92142 2.49185 5.17367 3.89951 1028 - -
2.68618 4.42225 2.77519 2.73123 3.46354 2.40513 3.72494 3.29354 2.67741 2.69355 4.24690 2.90347 2.73739 3.18146 2.89801 2.37887 2.77519 2.98518 4.58477 3.61503
0.01785 4.43070 5.15305 0.61958 0.77255 0.48576 0.95510
885 2.79256 4.67712 3.43397 2.92693 3.73152 3.62173 3.89671 3.32950 2.39439 2.98373 3.87184 3.34429 4.06651 3.17668 3.03566 1.79020 3.05344 3.06207 1.90791 3.78746 1029 - -
2.68618 4.42225 2.77519 2.73123 3.46354 2.40513 3.72494 3.29354 2.67741 2.69355 4.24690 2.90347 2.73739 3.18146 2.89801 2.37887 2.77519 2.98518 4.58477 3.61503
0.01785 4.43070 5.15305 0.61958 0.77255 0.48576 0.95510
886 4.41538 5.55787 5.03341 4.89935 3.45933 4.43909 4.75660 4.51583 4.64435 3.79743 5.11391 4.95527 5.00644 4.99755 4.66087 4.61454 4.75147 4.41693 0.25288 3.43853 1030 - -
2.68618 4.42225 2.77519 2.73123 3.46354 2.40513 3.72494 3.29354 2.67741 2.69355 4.24690 2.90347 2.73739 3.18146 2.89801 2.37887 2.77519 2.98518 4.58477 3.61503
0.01785 4.43070 5.15305 0.61958 0.77255 0.48576 0.95510
887 2.80976 4.40631 4.02587 3.48864 3.51364 3.81827 4.26280 2.59734 3.38224 2.35691 2.19345 3.78126 1.85663 3.67397 3.64913 3.13619 3.08353 1.87661 5.11624 3.91539 1031 - -
2.68618 4.42225 2.77519 2.73123 3.46354 2.40513 3.72494 3.29354 2.67741 2.69355 4.24690 2.90347 2.73739 3.18146 2.89801 2.37887 2.77519 2.98518 4.58477 3.61503
0.01785 4.43070 5.15305 0.61958 0.77255 0.48576 0.95510
888 3.19601 5.32119 1.34664 2.69189 3.50012 3.66773 3.94355 4.01711 3.12535 3.54162 4.51014 3.21404 4.21504 3.34092 3.60801 3.17184 3.47028 3.72504 5.04312 1.47732 1032 - -
2.68618 4.42225 2.77519 2.73123 3.46354 2.40513 3.72494 3.29354 2.67741 2.69355 4.24690 2.90347 2.73739 3.18146 2.89801 2.37887 2.77519 2.98518 4.58477 3.61503
0.01785 4.43070 5.15305 0.61958 0.77255 0.48576 0.95510
889 2.47756 2.59154 2.90945 2.81012 3.85212 3.51226 3.88864 3.23690 2.80311 2.91793 3.77989 3.25165 3.96536 3.12674 3.21167 1.64943 2.91986 2.95539 5.23291 3.41695 1033 - -
2.68618 4.42225 2.77519 2.73123 3.46354 2.40513 3.72494 3.29354 2.67741 2.69355 4.24690 2.90347 2.73739 3.18146 2.89801 2.37887 2.77519 2.98518 4.58477 3.61503
0.01785 4.43070 5.15305 0.61958 0.77255 0.48576 0.95510
890 3.18799 5.10814 1.37165 2.92722 1.43523 3.76419 4.02131 3.63459 3.28715 3.18807 4.25008 3.40537 4.29018 3.49877 3.72465 3.24442 3.46846 3.42368 4.92781 3.33054 1034 - -
2.68618 4.42225 2.77519 2.73123 3.46354 2.40513 3.72494 3.29354 2.67741 2.69355 4.24690 2.90347 2.73739 3.18146 2.89801 2.37887 2.77519 2.98518 4.58477 3.61503
0.01785 4.43070 5.15305 0.61958 0.77255 0.48576 0.95510
891 0.93859 4.38180 3.89912 3.48583 4.17117 3.32337 4.41884 3.33609 3.44375 3.19362 4.10816 3.63973 2.93706 3.73656 3.73977 2.74205 2.96704 2.31972 5.61230 4.40549 1035 - -
2.68618 4.42225 2.77519 2.73123 3.46354 2.40513 3.72494 3.29354 2.67741 2.69355 4.24690 2.90347 2.73739 3.18146 2.89801 2.37887 2.77519 2.98518 4.58477 3.61503
0.01785 4.43070 5.15305 0.61958 0.77255 0.48576 0.95510
892 2.93302 4.37978 4.38511 3.80285 3.33746 4.06932 4.37645 2.41987 3.02854 1.25816 2.78835 4.03708 4.40297 3.88678 3.81944 3.36968 3.16449 1.74583 5.01925 3.85486 1036 - -
2.68618 4.42225 2.77519 2.73123 3.46354 2.40513 3.72494 3.29354 2.67741 2.69355 4.24690 2.90347 2.73739 3.18146 2.89801 2.37887 2.77519 2.98518 4.58477 3.61503

0.01785 4.43070 5.15305 0.61958 0.77255 0.48576 0.95510

893 2.87895 4.51067 4.00148 3.52061 1.60507 3.79657 4.04579 2.99517 3.44100 2.43375 3.67059 3.75635 4.26705 3.70190 3.70088 1.57314 3.17298 2.81132 4.65353 3.14188 1037 - -

2.68618 4.42225 2.77519 2.73123 3.46354 2.40513 3.72494 3.29354 2.67741 2.69355 4.24690 2.90347 2.73739 3.18146 2.89801 2.37887 2.77519 2.98518 4.58477 3.61503

0.01785 4.43070 5.15305 0.61958 0.77255 0.48576 0.95510

894 2.69342 4.91294 2.04877 2.54318 4.29906 3.44912 3.79916 3.71382 2.61620 3.31562 4.12213 3.05428 3.18417 2.94750 3.08697 2.62799 1.78501 2.91716 5.54334 4.19572 1038 - -

2.68618 4.42225 2.77519 2.73123 3.46354 2.40513 3.72494 3.29354 2.67741 2.69355 4.24690 2.90347 2.73739 3.18146 2.89801 2.37887 2.77519 2.98518 4.58477 3.61503

0.01785 4.43070 5.15305 0.61958 0.77255 0.48576 0.95510

895 3.70767 5.81134 0.36689 2.86814 5.29130 3.74379 4.56793 5.03282 3.77612 4.54671 5.57078 3.44114 4.43519 3.83036 4.34272 3.62893 4.06803 4.62109 6.33213 5.11541 1039 - -

2.68618 4.42225 2.77519 2.73123 3.46354 2.40513 3.72494 3.29354 2.67741 2.69355 4.24690 2.90347 2.73739 3.18146 2.89801 2.37887 2.77519 2.98518 4.58477 3.61503

0.01785 4.43070 5.15305 0.61958 0.77255 0.48576 0.95510

896 3.04944 4.41879 4.74948 4.17584 1.72034 4.19951 4.32987 2.21001 4.01115 1.96545 1.78992 4.24875 4.50837 4.12091 4.07637 3.51478 3.27541 2.52235 4.70806 2.82774 1040 - -

2.68618 4.42225 2.77519 2.73123 3.46354 2.40513 3.72494 3.29354 2.67741 2.69355 4.24690 2.90347 2.73739 3.18146 2.89801 2.37887 2.77519 2.98518 4.58477 3.61503

0.01785 4.43070 5.15305 0.61958 0.77255 0.48576 0.95510

897 2.39184 5.00653 2.54640 2.45158 4.28241 3.48268 3.16684 3.72202 2.45342 2.61425 4.06795 2.98488 3.33148 2.09795 2.92954 2.68475 2.91481 3.10435 5.48370 4.11912 1041 - -

2.68618 4.42225 2.77519 2.73123 3.46354 2.40513 3.72494 3.29354 2.67741 2.69355 4.24690 2.90347 2.73739 3.18146 2.89801 2.37887 2.77519 2.98518 4.58477 3.61503

0.01785 4.43070 5.15305 0.61958 0.77255 0.48576 0.95510

898 2.96106 4.67478 3.75964 3.20468 1.66970 3.87210 3.95015 3.03013 1.73885 2.32116 3.65604 3.57783 4.24577 3.37985 3.21630 3.15779 3.18927 2.87591 4.82838 3.37708 1042 - -

2.68618 4.42225 2.77519 2.73123 3.46354 2.40513 3.72494 3.29354 2.67741 2.69355 4.24690 2.90347 2.73739 3.18146 2.89801 2.37887 2.77519 2.98518 4.58477 3.61503

0.01785 4.43070 5.15305 0.61958 0.77255 0.48576 0.95510

899 2.47044 5.07952 2.95724 1.86426 4.37881 3.47977 3.19780 3.82842 2.43785 3.36539 4.14170 2.97282 3.88298 2.72830 2.75124 2.54568 2.05588 3.44726 5.54521 4.17030 1043 - -

2.68618 4.42225 2.77519 2.73123 3.46354 2.40513 3.72494 3.29354 2.67741 2.69355 4.24690 2.90347 2.73739 3.18146 2.89801 2.37887 2.77519 2.98518 4.58477 3.61503

0.01785 4.43070 5.15305 0.61958 0.77255 0.48576 0.95510

900 2.68278 4.70788 3.19196 1.98839 3.90196 3.55123 3.24826 2.64210 2.62428 2.27679 3.79331 3.13558 3.27571 2.96809 3.05794 2.52022 2.91461 3.01395 5.24235 3.94646 1044 - -

2.68618 4.42225 2.77519 2.73123 3.46354 2.40513 3.72494 3.29354 2.67741 2.69355 4.24690 2.90347 2.73739 3.18146 2.89801 2.37887 2.77519 2.98518 4.58477 3.61503

0.01785 4.43070 5.15305 0.61958 0.77255 0.48576 0.95510

901 2.97613 5.12408 3.05329 2.76470 4.58368 3.55855 4.02003 4.09634 2.71803 3.62757 4.50143 3.24795 1.54707 1.46301 3.07046 3.00505 3.28037 3.71778 5.76414 4.45164 1045 - -

2.68640 4.41912 2.77542 2.73145 3.46376 2.40453 3.72516 3.29376 2.67763 2.69377 4.24712 2.90369 2.73762 3.18168 2.89673 2.37894 2.77523 2.98540 4.58499 3.61525

1.07878 1.11402 1.10337 1.03028 0.44147 0.48576 0.95510

902 3.13904 4.44081 4.87826 4.34137 3.46527 4.49982 4.96242 1.23339 4.23028 1.95328 2.74423 4.55382 4.76302 4.43402 4.39377 3.85919 3.38473 1.40168 5.41843 4.26480 1049 - -

2.68618 4.42225 2.77519 2.73123 3.46354 2.40513 3.72494 3.29354 2.67741 2.69355 4.24690 2.90347 2.73739 3.18146 2.89801 2.37887 2.77519 2.98518 4.58477 3.61503

0.02502 4.09652 4.81887 0.61958 0.77255 0.90459 0.51870

903 2.64943 4.83650 2.93752 2.48375 4.20539 3.38769 3.68697 3.62041 2.18279 3.20545 4.02543 2.98298 3.84350 2.55670 2.81131 1.80798 2.51887 3.27066 5.41829 4.09809 1050 - -

2.68618 4.42225 2.77519 2.73123 3.46354 2.40513 3.72494 3.29354 2.67741 2.69355 4.24690 2.90347 2.73739 3.18146 2.89801 2.37887 2.77519 2.98518 4.58477 3.61503

0.02502 4.09652 4.81887 0.61958 0.77255 0.90459 0.51870

904 2.72954 4.74239 2.95673 2.75766 4.45456 1.12149 4.02261 3.91370 2.31024 3.53312 4.40972 3.18451 3.92609 3.24063 3.12528 2.81651 3.10792 3.49576 5.65125 4.40853 1051 - -

2.68618 4.42225 2.77519 2.73123 3.46354 2.40513 3.72494 3.29354 2.67741 2.69355 4.24690 2.90347 2.73739 3.18146 2.89801 2.37887 2.77519 2.98518 4.58477 3.61503

0.02502 4.09652 4.81887 0.61958 0.77255 0.90459 0.51870

905 3.08393 5.15870 3.45366 2.85222 4.54413 3.68495 3.68801 3.96309 1.98722 3.41159 4.30679 3.25259 4.08103 2.37773 1.13914 3.09269 3.26848 3.65567 5.46808 4.27566 1052 - -

2.68618 4.42225 2.77519 2.73123 3.46354 2.40513 3.72494 3.29354 2.67741 2.69355 4.24690 2.90347 2.73739 3.18146 2.89801 2.37887 2.77519 2.98518 4.58477 3.61503

0.02502 4.09652 4.81887 0.61958 0.77255 0.90459 0.51870

906 3.25499 4.65124 4.69924 4.20889 3.19780 4.39529 4.82512 2.16621 4.01274 0.86367 3.04594 4.45674 4.69922 4.24551 4.17265 3.79210 3.51827 1.96720 5.23809 4.07268 1053 - -

2.68618 4.42225 2.77519 2.73123 3.46354 2.40513 3.72494 3.29354 2.67741 2.69355 4.24690 2.90347 2.73739 3.18146 2.89801 2.37887 2.77519 2.98518 4.58477 3.61503

0.02502 4.09652 4.81887 0.61958 0.77255 0.90459 0.51870

907 3.31572 4.63361 4.91918 4.36406 2.08883 4.54271 4.68882 2.00797 4.23085 1.00941 2.91939 4.55502 4.73414 4.29421 4.31782 3.88608 3.53451 2.35234 4.94842 3.69509 1054 - -

2.68618 4.42225 2.77519 2.73123 3.46354 2.40513 3.72494 3.29354 2.67741 2.69355 4.24690 2.90347 2.73739 3.18146 2.89801 2.37887 2.77519 2.98518 4.58477 3.61503

0.02502 4.09652 4.81887 0.61958 0.77255 0.90459 0.51870

908 3.03322 4.43657 4.56634 4.08615 2.72873 4.19050 4.61793 2.01710 3.97553 2.05511 3.34017 4.27164 4.57180 4.20464 4.16121 3.57257 3.31134 1.02603 5.10717 3.75826 1055 - -

2.68618 4.42225 2.77519 2.73123 3.46354 2.40513 3.72494 3.29354 2.67741 2.69355 4.24690 2.90347 2.73739 3.18146 2.89801 2.37887 2.77519 2.98518 4.58477 3.61503

0.02502 4.09652 4.81887 0.61958 0.77255 0.29184 1.37393

909 2.76747 4.30556 4.01599 3.42740 3.33268 3.82767 4.08554 2.44478 3.25507 1.79092 3.33885 3.71339 4.19052 3.56161 2.04772 3.10601 2.99693 2.34486 2.84927 3.65391 1056 - -

2.68618 4.42225 2.77519 2.73123 3.46354 2.40513 3.72494 3.29354 2.67741 2.69355 4.24690 2.90347 2.73739 3.18146 2.89801 2.37887 2.77519 2.98518 4.58477 3.61503

0.01712 4.47174 5.19409 0.61958 0.77255 0.48576 0.95510

910 2.55156 5.15967 1.92491 2.17836 4.48221 3.23845 3.33309 3.95358 2.41846 3.45551 3.60561 2.94719 3.32811 2.77497 2.75596 2.42034 2.93338 3.53845 5.60608 4.20708 1057 - -

2.68618 4.42225 2.77519 2.73123 3.46354 2.40513 3.72494 3.29354 2.67741 2.69355 4.24690 2.90347 2.73739 3.18146 2.89801 2.37887 2.77519 2.98518 4.58477 3.61503

0.01712 4.47174 5.19409 0.61958 0.77255 0.48576 0.95510

911 3.53845 5.52011 4.06471 3.30802 5.07235 4.04857 3.92928 4.40605 1.92589 3.78997 4.70294 3.63209 4.41782 3.07090 0.66961 3.53829 3.67878 4.10519 5.75304 4.66618 1058 - -

2.68618 4.42225 2.77519 2.73123 3.46354 2.40513 3.72494 3.29354 2.67741 2.69355 4.24690 2.90347 2.73739 3.18146 2.89801 2.37887 2.77519 2.98518 4.58477 3.61503

0.01712 4.47174 5.19409 0.61958 0.77255 0.48576 0.95510

912 4.31016 5.40272 5.14472 4.96927 1.91992 4.88329 3.74412 3.94307 4.78337 3.16934 4.51275 4.50444 5.18336 4.62822 4.71882 4.30091 4.53052 3.89107 3.84379 0.49569 1059 - -

2.68618 4.42225 2.77519 2.73123 3.46354 2.40513 3.72494 3.29354 2.67741 2.69355 4.24690 2.90347 2.73739 3.18146 2.89801 2.37887 2.77519 2.98518 4.58477 3.61503

0.01712 4.47174 5.19409 0.61958 0.77255 0.48576 0.95510

913 2.58273 4.79128 3.01239 2.35329 4.00399 3.37265 3.75248 3.03009 2.24695 2.20023 3.27127 3.10082 3.93054 2.74138 2.72252 2.75161 2.91944 2.86026 5.31215 3.99966 1060 - -

2.68618 4.42225 2.77519 2.73123 3.46354 2.40513 3.72494 3.29354 2.67741 2.69355 4.24690 2.90347 2.73739 3.18146 2.89801 2.37887 2.77519 2.98518 4.58477 3.61503

0.01712 4.47174 5.19409 0.61958 0.77255 0.48576 0.95510

914 2.70573 5.19602 1.96551 2.29574 4.53089 3.46767 3.31296 4.01085 2.10645 3.49651 4.24065 2.61753 3.64401 2.76680 2.76121 2.45322 2.82429 3.34501 5.63357 4.22667 1061 - -

2.68618 4.42225 2.77519 2.73123 3.46354 2.40513 3.72494 3.29354 2.67741 2.69355 4.24690 2.90347 2.73739 3.18146 2.89801 2.37887 2.77519 2.98518 4.58477 3.61503

0.01712 4.47174 5.19409 0.61958 0.77255 0.48576 0.95510

915 2.72384 5.17601 2.12781 2.40464 4.49006 3.47545 2.33891 3.96351 2.44159 3.46880 4.22666 2.69140 3.88393 2.60344 2.81671 2.38788 2.95714 3.55259 5.62081 3.48293 1062 - -

2.68618 4.42225 2.77519 2.73123 3.46354 2.40513 3.72494 3.29354 2.67741 2.69355 4.24690 2.90347 2.73739 3.18146 2.89801 2.37887 2.77519 2.98518 4.58477 3.61503

0.01712 4.47174 5.19409 0.61958 0.77255 0.48576 0.95510

916 1.99455 4.78695 3.17895 2.64081 4.04125 3.54272 3.79531 3.05263 2.38427 3.06731 3.90816 1.88888 3.95301 2.97288 3.04662 2.77611 2.94779 2.78801 5.35163 4.04435 1063 - -
2.68618 4.42225 2.77519 2.73123 3.46354 2.40513 3.72494 3.29354 2.67741 2.69355 4.24690 2.90347 2.73739 3.18146 2.89801 2.37887 2.77519 2.98518 4.58477 3.61503
0.01712 4.47174 5.19409 0.61958 0.77255 0.48576 0.95510
917 2.73002 4.56546 2.09232 2.86407 3.30239 3.65147 3.88177 3.04609 2.84889 1.79280 3.63460 3.31386 4.03860 3.16511 3.24341 2.89358 2.67802 2.82117 5.07270 3.29358 1064 - -
2.68620 4.42146 2.77522 2.73125 3.46356 2.40515 3.72496 3.29356 2.67743 2.69357 4.24692 2.90349 2.73742 3.18148 2.89803 2.37889 2.77522 2.98520 4.58479 3.61461
0.54190 2.30311 1.14441 0.30697 1.33058 0.48576 0.95510
918 3.04427 4.47706 4.40666 3.84164 3.00201 4.16282 4.34263 2.39249 3.68021 1.07451 2.48385 4.09198 4.44697 3.89123 3.85705 3.47243 3.26849 2.43849 4.83340 2.93254 1066 - -
2.68618 4.42225 2.77519 2.73123 3.46354 2.40513 3.72494 3.29354 2.67741 2.69355 4.24690 2.90347 2.73739 3.18146 2.89801 2.37887 2.77519 2.98518 4.58477 3.61503
0.02502 4.09652 4.81887 0.61958 0.77255 0.90459 0.51870
919 2.42669 4.47495 4.09898 3.61658 1.91370 3.93453 3.72071 2.89546 3.51556 2.48836 3.56008 3.76523 4.30931 3.70798 3.71932 3.24826 3.22803 2.73870 4.09001 1.49714 1067 - -
2.68618 4.42225 2.77519 2.73123 3.46354 2.40513 3.72494 3.29354 2.67741 2.69355 4.24690 2.90347 2.73739 3.18146 2.89801 2.37887 2.77519 2.98518 4.58477 3.61503
0.02502 4.09652 4.81887 0.61958 0.77255 0.90459 0.51870
920 1.50048 4.50644 3.07942 2.71975 4.08279 2.37574 3.07924 3.52311 2.77404 3.17259 4.01805 3.12725 3.82093 3.11808 3.16542 2.61653 2.85830 3.14393 5.41224 4.11785 1068 - -
2.68561 4.42277 2.77571 2.73175 3.46288 2.40468 3.72546 3.29406 2.67792 2.69297 4.24648 2.90398 2.73791 3.18170 2.89852 2.37886 2.77493 2.98424 4.58528 3.61373
0.74460 0.77983 2.70917 1.80954 0.17880 0.29184 1.37393
921 1.53580 4.43854 4.51701 4.01176 3.77930 4.05753 4.73650 1.98989 3.91698 2.53039 3.65632 4.20159 4.52506 4.18561 4.14540 3.42768 3.25737 1.24808 5.48374 4.27611 1076 - -
2.68647 4.42254 2.77471 2.73135 3.46383 2.40460 3.72524 3.29383 2.67770 2.69295 4.24719 2.90376 2.73655 3.18144 2.89830 2.37916 2.77528 2.98548 4.58506 3.61532
0.40650 1.11402 5.15305 1.28876 0.32243 0.55048 0.85961
922 2.38293 4.24163 4.40846 3.82897 3.37567 3.98673 4.33137 1.81259 3.70221 2.22610 2.87140 4.00881 3.31898 3.91480 3.86169 3.28980 3.05767 1.46345 4.95979 3.77110 1081 - -
2.68618 4.42225 2.77519 2.73123 3.46354 2.40513 3.72494 3.29354 2.67741 2.69355 4.24690 2.90347 2.73739 3.18146 2.89801 2.37887 2.77519 2.98518 4.58477 3.61503
0.01785 4.43070 5.15305 0.61958 0.77255 0.55048 0.85961
923 2.89285 4.92690 3.15832 2.77470 3.62260 1.71916 1.82442 3.65342 2.77149 3.24030 4.12877 3.24461 4.08420 3.15777 3.15910 2.95217 3.15762 3.35281 5.08274 3.10659 1082 - -
2.68618 4.42225 2.77519 2.73123 3.46354 2.40513 3.72494 3.29354 2.67741 2.69355 4.24690 2.90347 2.73739 3.18146 2.89801 2.37887 2.77519 2.98518 4.58477 3.61503
0.01785 4.43070 5.15305 0.61958 0.77255 0.44732 1.01981
924 2.16825 4.43191 3.79473 3.56475 4.84167 1.29444 4.65844 4.30355 3.68956 3.96499 4.75103 3.64099 3.96837 3.93048 3.99040 1.21731 2.99488 3.61226 6.15457 4.96810 1083 - -
2.68618 4.42225 2.77519 2.73123 3.46354 2.40513 3.72494 3.29354 2.67741 2.69355 4.24690 2.90347 2.73739 3.18146 2.89801 2.37887 2.77519 2.98518 4.58477 3.61503
0.01712 4.47174 5.19409 0.61958 0.77255 0.48576 0.95510
925 3.42014 4.84486 4.51720 4.07068 2.46046 4.29980 3.83884 3.35915 3.84880 2.88282 3.97755 4.09629 4.66524 4.05823 3.99534 3.63835 2.96490 3.20185 1.09468 1.80031 1084 - -
2.68618 4.42225 2.77519 2.73123 3.46354 2.40513 3.72494 3.29354 2.67741 2.69355 4.24690 2.90347 2.73739 3.18146 2.89801 2.37887 2.77519 2.98518 4.58477 3.61503
0.01712 4.47174 5.19409 0.61958 0.77255 0.48576 0.95510
926 2.74923 5.19097 2.63701 2.32021 4.50511 3.47741 3.70893 3.97251 2.47621 3.48414 4.24894 2.59604 2.59963 1.80310 2.96678 2.71864 2.98520 3.06091 5.64393 4.24513 1085 - -
2.68618 4.42225 2.77519 2.73123 3.46354 2.40513 3.72494 3.29354 2.67741 2.69355 4.24690 2.90347 2.73739 3.18146 2.89801 2.37887 2.77519 2.98518 4.58477 3.61503
0.01712 4.47174 5.19409 0.61958 0.77255 0.48576 0.95510
927 2.59361 5.16078 2.49564 2.19052 4.48731 3.03770 3.37281 3.96000 2.29901 3.45753 4.20695 2.94647 3.86697 2.76632 2.15526 2.66657 2.47076 3.54155 5.60389 3.61096 1086 - -
2.68618 4.42225 2.77519 2.73123 3.46354 2.40513 3.72494 3.29354 2.67741 2.69355 4.24690 2.90347 2.73739 3.18146 2.89801 2.37887 2.77519 2.98518 4.58477 3.61503

0.01712 4.47174 5.19409 0.61958 0.77255 0.48576 0.95510

928 2.58329 4.59534 3.36061 1.74953 3.75530 3.62631 3.85703 2.81219 2.64402 2.18513 3.16628 3.26440 4.00907 3.09878 3.17062 2.85448 2.94079 2.67415 5.16119 3.89540 1087 - -

2.68618 4.42225 2.77519 2.73123 3.46354 2.40513 3.72494 3.29354 2.67741 2.69355 4.24690 2.90347 2.73739 3.18146 2.89801 2.37887 2.77519 2.98518 4.58477 3.61503

0.01712 4.47174 5.19409 0.61958 0.77255 0.48576 0.95510

929 2.69525 2.40375 4.14344 3.56268 3.30895 3.78698 4.12047 2.44659 3.44524 1.98572 3.31360 3.19416 4.16965 3.67393 3.63640 2.50710 2.93522 1.87845 4.82194 3.62782 1088 - -

2.68618 4.42225 2.77519 2.73123 3.46354 2.40513 3.72494 3.29354 2.67741 2.69355 4.24690 2.90347 2.73739 3.18146 2.89801 2.37887 2.77519 2.98518 4.58477 3.61503

0.01712 4.47174 5.19409 0.61958 0.77255 0.48576 0.95510

930 2.83923 4.66891 3.50804 2.95939 2.34726 3.73327 3.83182 3.21518 1.70515 2.86365 3.77301 3.38219 4.11622 3.21764 3.21147 2.70156 3.06939 2.97691 4.81693 2.26364 1089 - -

2.68618 4.42225 2.77519 2.73123 3.46354 2.40513 3.72494 3.29354 2.67741 2.69355 4.24690 2.90347 2.73739 3.18146 2.89801 2.37887 2.77519 2.98518 4.58477 3.61503

0.01712 4.47174 5.19409 0.61958 0.77255 0.48576 0.95510

931 2.84145 5.60219 1.97727 2.11059 4.92529 1.53979 3.92201 4.43521 2.85937 3.92047 4.72671 2.09278 4.03099 3.06672 3.42803 2.95682 3.32060 3.99639 6.07565 4.59500 1090 - -

2.68618 4.42225 2.77519 2.73123 3.46354 2.40513 3.72494 3.29354 2.67741 2.69355 4.24690 2.90347 2.73739 3.18146 2.89801 2.37887 2.77519 2.98518 4.58477 3.61503

0.01712 4.47174 5.19409 0.61958 0.77255 0.48576 0.95510

932 2.61262 5.11252 2.66228 2.23964 4.39814 3.49235 3.71693 3.84872 2.33592 3.39110 4.17310 2.98226 3.90774 2.33942 2.96931 2.59082 2.97754 3.47243 5.57679 2.03240 1091 - -

2.68618 4.42225 2.77519 2.73123 3.46354 2.40513 3.72494 3.29354 2.67741 2.69355 4.24690 2.90347 2.73739 3.18146 2.89801 2.37887 2.77519 2.98518 4.58477 3.61503

0.01712 4.47174 5.19409 0.61958 0.77255 0.48576 0.95510

933 3.16325 5.43473 3.53210 2.85728 4.90066 3.82182 3.41504 4.26506 1.55473 3.66808 4.49345 3.30241 4.17569 1.49296 1.88790 3.13046 3.33275 3.90287 5.69617 4.48466 1092 - -

2.68618 4.42225 2.77519 2.73123 3.46354 2.40513 3.72494 3.29354 2.67741 2.69355 4.24690 2.90347 2.73739 3.18146 2.89801 2.37887 2.77519 2.98518 4.58477 3.61503

0.01712 4.47174 5.19409 0.61958 0.77255 0.48576 0.95510

934 2.68382 4.47859 3.56273 3.10262 1.68598 3.54957 4.04239 3.16997 3.09743 2.87327 3.77008 2.90808 4.05887 3.39855 3.44335 1.80023 2.77275 2.90314 5.10486 3.77799 1093 - -

2.68618 4.42225 2.77519 2.73123 3.46354 2.40513 3.72494 3.29354 2.67741 2.69355 4.24690 2.90347 2.73739 3.18146 2.89801 2.37887 2.77519 2.98518 4.58477 3.61503

0.01712 4.47174 5.19409 0.61958 0.77255 0.48576 0.95510

935 4.50179 5.62504 5.10517 4.98327 3.53162 4.49500 4.82933 4.61303 4.73401 3.88530 5.20744 5.03524 5.06565 5.08388 4.74150 4.70107 4.84079 4.51410 0.23110 3.50965 1094 - -

2.68618 4.42225 2.77519 2.73123 3.46354 2.40513 3.72494 3.29354 2.67741 2.69355 4.24690 2.90347 2.73739 3.18146 2.89801 2.37887 2.77519 2.98518 4.58477 3.61503

0.01712 4.47174 5.19409 0.61958 0.77255 0.48576 0.95510

936 2.88236 2.27520 4.59521 4.02267 3.45769 4.07322 4.47701 1.75162 3.88409 2.26060 3.41097 4.15656 4.43967 4.08735 4.01673 3.39407 2.01807 1.79458 5.06594 3.87656 1095 - -

2.68618 4.42225 2.77519 2.73123 3.46354 2.40513 3.72494 3.29354 2.67741 2.69355 4.24690 2.90347 2.73739 3.18146 2.89801 2.37887 2.77519 2.98518 4.58477 3.61503

0.40024 4.47174 1.14441 0.61958 0.77255 0.48576 0.95510

937 3.27534 5.08730 3.79049 3.26179 4.49964 3.71266 4.01132 4.06195 2.36107 3.53729 4.53657 3.60176 4.20883 3.22485 0.68436 3.35179 3.52888 3.77741 5.49442 4.38373 1096 - -

2.68618 4.42225 2.77519 2.73123 3.46354 2.40513 3.72494 3.29354 2.67741 2.69355 4.24690 2.90347 2.73739 3.18146 2.89801 2.37887 2.77519 2.98518 4.58477 3.61503

0.02502 4.09652 4.81887 0.61958 0.77255 0.29184 1.37393

938 0.78089 4.39076 3.94113 3.71227 4.78016 1.94506 4.72614 4.21398 3.79808 3.90677 4.70657 3.70705 3.97265 4.03023 4.05986 2.38178 2.98550 3.55218 6.12167 4.95693 1097 - -

2.68618 4.42225 2.77519 2.73123 3.46354 2.40513 3.72494 3.29354 2.67741 2.69355 4.24690 2.90347 2.73739 3.18146 2.89801 2.37887 2.77519 2.98518 4.58477 3.61503

0.01712 4.47174 5.19409 0.61958 0.77255 0.48576 0.95510

939 2.71420 4.94456 2.55882 2.00258 4.18872 3.51454 3.34254 3.60860 2.53409 2.00716 4.01542 3.03641 3.91732 2.88047 3.00188 2.73751 2.65619 3.08924 5.44161 4.09733 1098 - -

2.68618 4.42225 2.77519 2.73123 3.46354 2.40513 3.72494 3.29354 2.67741 2.69355 4.24690 2.90347 2.73739 3.18146 2.89801 2.37887 2.77519 2.98518 4.58477 3.61503

0.01712 4.47174 5.19409 0.61958 0.77255 0.48576 0.95510

940 2.93304 5.46649 1.80240 2.36463 4.79055 3.47070 3.82956 4.28833 2.21395 3.76906 4.54897 1.63154 3.13393 2.95669 3.19842 2.86196 3.18682 3.85367 5.91120 4.46366 1099 - -

2.68618 4.42225 2.77519 2.73123 3.46354 2.40513 3.72494 3.29354 2.67741 2.69355 4.24690 2.90347 2.73739 3.18146 2.89801 2.37887 2.77519 2.98518 4.58477 3.61503

0.01712 4.47174 5.19409 0.61958 0.77255 0.48576 0.95510

941 2.21206 3.57069 2.35627 2.71490 3.89982 3.19617 3.82531 3.28132 2.70035 2.94800 2.94357 3.18989 3.95847 3.03410 3.12900 2.78049 2.11640 3.00001 5.25416 3.96787 1100 - -

2.68618 4.42225 2.77519 2.73123 3.46354 2.40513 3.72494 3.29354 2.67741 2.69355 4.24690 2.90347 2.73739 3.18146 2.89801 2.37887 2.77519 2.98518 4.58477 3.61503

0.01712 4.47174 5.19409 0.61958 0.77255 0.48576 0.95510

942 3.57401 5.24861 4.36381 4.35348 5.40728 0.20721 5.39221 5.19546 4.61166 4.75230 5.76109 4.53127 4.65161 4.88936 4.74225 3.76858 4.09816 4.60781 6.30299 5.54286 1101 - -

2.68618 4.42225 2.77519 2.73123 3.46354 2.40513 3.72494 3.29354 2.67741 2.69355 4.24690 2.90347 2.73739 3.18146 2.89801 2.37887 2.77519 2.98518 4.58477 3.61503

0.01712 4.47174 5.19409 0.61958 0.77255 0.48576 0.95510

943 2.76750 4.36481 3.93823 3.37852 3.51106 3.79617 4.16624 2.36576 3.29713 2.46733 2.69393 3.69633 4.20213 3.57851 3.58332 1.50689 3.02307 2.07059 5.05041 3.83923 1102 - -

2.68618 4.42225 2.77519 2.73123 3.46354 2.40513 3.72494 3.29354 2.67741 2.69355 4.24690 2.90347 2.73739 3.18146 2.89801 2.37887 2.77519 2.98518 4.58477 3.61503

0.01712 4.47174 5.19409 0.61958 0.77255 0.48576 0.95510

944 4.13482 5.36638 5.08039 4.93993 0.38366 4.60565 4.29768 3.63487 4.84583 2.87738 4.29054 4.75773 5.07468 4.85450 4.81517 4.34644 4.44809 3.66349 4.43269 2.75127 1103 - -

2.68618 4.42225 2.77519 2.73123 3.46354 2.40513 3.72494 3.29354 2.67741 2.69355 4.24690 2.90347 2.73739 3.18146 2.89801 2.37887 2.77519 2.98518 4.58477 3.61503

0.01712 4.47174 5.19409 0.61958 0.77255 0.48576 0.95510

945 2.69372 2.51341 4.18692 3.61412 3.34603 3.76414 4.16616 2.46763 3.48967 1.58651 3.34559 3.80481 4.17619 3.71879 3.67475 2.49952 2.43473 2.40655 4.86700 3.67536 1104 - -

2.68618 4.42225 2.77519 2.73123 3.46354 2.40513 3.72494 3.29354 2.67741 2.69355 4.24690 2.90347 2.73739 3.18146 2.89801 2.37887 2.77519 2.98518 4.58477 3.61503

0.01712 4.47174 5.19409 0.61958 0.77255 0.48576 0.95510

946 3.54296 4.79427 5.40033 4.85644 2.90471 4.92190 5.24883 0.93241 4.72925 1.41221 3.21446 5.02245 5.08571 4.77745 4.78321 4.29773 3.77113 2.18005 5.45343 4.30660 1105 - -

2.68618 4.42225 2.77519 2.73123 3.46354 2.40513 3.72494 3.29354 2.67741 2.69355 4.24690 2.90347 2.73739 3.18146 2.89801 2.37887 2.77519 2.98518 4.58477 3.61503

0.01712 4.47174 5.19409 0.61958 0.77255 0.48576 0.95510

947 2.22709 4.87258 3.50204 2.98338 4.55503 3.53344 3.99240 3.94579 2.53127 3.52382 4.36155 3.36722 4.07096 3.15829 1.08258 2.47133 3.14152 3.54438 5.68767 4.44765 1106 - -

2.68618 4.42225 2.77519 2.73123 3.46354 2.40513 3.72494 3.29354 2.67741 2.69355 4.24690 2.90347 2.73739 3.18146 2.89801 2.37887 2.77519 2.98518 4.58477 3.61503

0.01712 4.47174 5.19409 0.61958 0.77255 0.48576 0.95510

948 3.37769 5.32050 3.25444 3.21961 4.79844 3.71978 4.60919 4.69124 3.59577 4.27097 5.25410 0.42645 4.41503 3.91817 3.94201 3.44254 3.79880 4.24493 6.02552 4.73074 1107 - -

2.68618 4.42225 2.77519 2.73123 3.46354 2.40513 3.72494 3.29354 2.67741 2.69355 4.24690 2.90347 2.73739 3.18146 2.89801 2.37887 2.77519 2.98518 4.58477 3.61503

0.01712 4.47174 5.19409 0.61958 0.77255 0.48576 0.95510

949 2.79473 1.99200 4.51902 3.95022 3.41209 3.93391 4.37870 1.69020 3.79836 2.41021 3.39378 3.71329 4.33659 4.00164 3.92182 3.09038 3.05370 1.71899 4.98045 3.79019 1108 - -

2.68618 4.42225 2.77519 2.73123 3.46354 2.40513 3.72494 3.29354 2.67741 2.69355 4.24690 2.90347 2.73739 3.18146 2.89801 2.37887 2.77519 2.98518 4.58477 3.61503

0.01712 4.47174 5.19409 0.61958 0.77255 0.48576 0.95510

950 2.89257 4.97274 3.36417 2.78144 4.23943 3.67659 3.81362 3.59565 2.39334 3.20183 4.07018 3.24705 4.06800 1.70214 2.06351 2.94732 3.11281 2.11302 5.44665 4.17724 1109 - -

2.68618 4.42225 2.77519 2.73123 3.46354 2.40513 3.72494 3.29354 2.67741 2.69355 4.24690 2.90347 2.73739 3.18146 2.89801 2.37887 2.77519 2.98518 4.58477 3.61503

0.01712 4.47174 5.19409 0.61958 0.77255 0.48576 0.95510

951 1.62224 4.45033 3.73980 3.25203 3.68934 3.54425 4.12454 3.17890 3.16160 2.87740 3.78900 3.55334 2.78749 3.49363 3.46936 2.89630 3.00775 2.91095 1.81002 3.86129 1110 - -
2.68618 4.42225 2.77519 2.73123 3.46354 2.40513 3.72494 3.29354 2.67741 2.69355 4.24690 2.90347 2.73739 3.18146 2.89801 2.37887 2.77519 2.98518 4.58477 3.61503
0.01712 4.47174 5.19409 0.61958 0.77255 0.48576 0.95510
952 2.34736 4.44625 3.74616 3.55155 4.86677 1.20473 4.67212 4.33718 3.71041 3.99858 4.78704 3.63778 3.97677 3.94784 4.00818 1.22968 3.01176 3.63698 6.17903 4.98947 1111 - -
2.68618 4.42225 2.77519 2.73123 3.46354 2.40513 3.72494 3.29354 2.67741 2.69355 4.24690 2.90347 2.73739 3.18146 2.89801 2.37887 2.77519 2.98518 4.58477 3.61503
0.01712 4.47174 5.19409 0.61958 0.77255 0.48576 0.95510
953 2.74189 4.53931 2.27477 2.93338 3.67374 3.68801 3.94599 2.89714 2.90533 1.75416 3.27108 3.37492 4.07261 3.22214 3.29137 2.93165 2.49210 2.43667 5.13713 3.88880 1112 - -
2.68618 4.42225 2.77519 2.73123 3.46354 2.40513 3.72494 3.29354 2.67741 2.69355 4.24690 2.90347 2.73739 3.18146 2.89801 2.37887 2.77519 2.98518 4.58477 3.61503
0.01712 4.47174 5.19409 0.61958 0.77255 0.48576 0.95510
954 3.03939 5.40242 1.55845 2.17535 4.37306 3.51653 3.91480 4.10826 2.87456 3.65890 4.51062 2.99871 4.06074 3.10639 3.40286 2.98631 3.30163 3.75246 5.70313 1.64771 1113 - -
2.68618 4.42225 2.77519 2.73123 3.46354 2.40513 3.72494 3.29354 2.67741 2.69355 4.24690 2.90347 2.73739 3.18146 2.89801 2.37887 2.77519 2.98518 4.58477 3.61503
0.01712 4.47174 5.19409 0.61958 0.77255 0.48576 0.95510
955 2.72862 4.47995 3.53715 3.00688 2.81681 3.66764 3.93388 2.98477 2.96856 1.85154 3.59674 2.04274 4.07211 3.27990 3.32484 2.44492 2.97307 2.76055 4.98555 3.67776 1114 - -
2.68618 4.42225 2.77519 2.73123 3.46354 2.40513 3.72494 3.29354 2.67741 2.69355 4.24690 2.90347 2.73739 3.18146 2.89801 2.37887 2.77519 2.98518 4.58477 3.61503
0.01712 4.47174 5.19409 0.61958 0.77255 0.48576 0.95510
956 3.82181 5.05877 5.57262 4.99833 3.24717 5.16853 5.43681 2.22969 4.84168 0.56686 2.43172 5.25551 5.20313 4.78950 4.85372 4.55387 4.02516 2.64026 5.48429 4.51747 1115 - -
2.68618 4.42225 2.77519 2.73123 3.46354 2.40513 3.72494 3.29354 2.67741 2.69355 4.24690 2.90347 2.73739 3.18146 2.89801 2.37887 2.77519 2.98518 4.58477 3.61503
0.01712 4.47174 5.19409 0.61958 0.77255 0.48576 0.95510
957 3.24872 5.35953 1.36061 2.73856 3.47581 3.70917 3.97371 4.05920 3.18560 3.57598 4.55523 3.25784 4.25979 3.39426 3.66662 3.22297 3.52451 3.77071 5.02874 1.37302 1116 - -
2.68618 4.42225 2.77519 2.73123 3.46354 2.40513 3.72494 3.29354 2.67741 2.69355 4.24690 2.90347 2.73739 3.18146 2.89801 2.37887 2.77519 2.98518 4.58477 3.61503
0.01712 4.47174 5.19409 0.61958 0.77255 0.48576 0.95510
958 0.63617 4.39423 4.09257 3.84909 4.63536 3.22950 4.77211 3.80686 3.83273 3.67908 4.57457 3.79478 4.02375 4.10597 4.05432 2.70926 2.34286 3.31345 6.04468 4.88272 1117 - -
2.68618 4.42225 2.77519 2.73123 3.46354 2.40513 3.72494 3.29354 2.67741 2.69355 4.24690 2.90347 2.73739 3.18146 2.89801 2.37887 2.77519 2.98518 4.58477 3.61503
0.01712 4.47174 5.19409 0.61958 0.77255 0.48576 0.95510
959 4.50179 5.62504 5.10517 4.98327 3.53162 4.49500 4.82933 4.61303 4.73401 3.88530 5.20744 5.03524 5.06565 5.08388 4.74150 4.70107 4.84079 4.51410 0.23110 3.50965 1118 - -
2.68618 4.42225 2.77519 2.73123 3.46354 2.40513 3.72494 3.29354 2.67741 2.69355 4.24690 2.90347 2.73739 3.18146 2.89801 2.37887 2.77519 2.98518 4.58477 3.61503
0.01712 4.47174 5.19409 0.61958 0.77255 0.48576 0.95510
960 1.45688 4.39312 4.35112 3.92042 3.98334 3.64166 4.68243 2.55731 3.83544 2.85001 3.89012 3.99785 4.27539 4.10178 4.05948 3.05999 2.79177 1.20402 5.58085 4.38582 1119 - -
2.68618 4.42225 2.77519 2.73123 3.46354 2.40513 3.72494 3.29354 2.67741 2.69355 4.24690 2.90347 2.73739 3.18146 2.89801 2.37887 2.77519 2.98518 4.58477 3.61503
0.01712 4.47174 5.19409 0.61958 0.77255 0.48576 0.95510
961 2.41825 4.65877 3.35063 2.94484 4.45878 2.97019 4.11540 3.88663 2.94898 3.50679 4.32035 3.32293 1.26940 3.30640 2.92336 2.17140 3.00080 3.43298 5.73221 4.45324 1120 - -
2.68618 4.42225 2.77519 2.73123 3.46354 2.40513 3.72494 3.29354 2.67741 2.69355 4.24690 2.90347 2.73739 3.18146 2.89801 2.37887 2.77519 2.98518 4.58477 3.61503
0.01712 4.47174 5.19409 0.61958 0.77255 0.48576 0.95510
962 2.78757 4.56878 3.85950 3.49967 3.98032 1.36264 4.41824 3.29971 3.42729 2.95920 1.48886 3.74306 4.18860 3.77599 3.69768 3.01075 3.17590 3.05364 5.47708 4.24957 1121 - -
2.68618 4.42225 2.77519 2.73123 3.46354 2.40513 3.72494 3.29354 2.67741 2.69355 4.24690 2.90347 2.73739 3.18146 2.89801 2.37887 2.77519 2.98518 4.58477 3.61503

0.01712 4.47174 5.19409 0.61958 0.77255 0.48576 0.95510

963 3.06353 4.74282 4.12313 3.83473 1.18985 1.32568 4.33499 3.52531 3.85814 3.12640 4.19017 3.98940 4.40001 4.09029 4.07829 3.28578 3.44895 3.28914 4.88298 3.33279 1122 - -

2.68618 4.42225 2.77519 2.73123 3.46354 2.40513 3.72494 3.29354 2.67741 2.69355 4.24690 2.90347 2.73739 3.18146 2.89801 2.37887 2.77519 2.98518 4.58477 3.61503

0.01712 4.47174 5.19409 0.61958 0.77255 0.48576 0.95510

964 3.00722 4.51173 4.30215 3.77808 3.53496 4.08814 4.50141 1.44594 3.64276 2.03202 3.45190 4.05593 1.79284 3.93847 3.87933 3.41981 3.26887 2.26287 5.24428 4.03664 1123 - -

2.68618 4.42225 2.77519 2.73123 3.46354 2.40513 3.72494 3.29354 2.67741 2.69355 4.24690 2.90347 2.73739 3.18146 2.89801 2.37887 2.77519 2.98518 4.58477 3.61503

0.01712 4.47174 5.19409 0.61958 0.77255 0.48576 0.95510

965 2.56378 5.07734 2.81096 2.46162 4.39589 1.93918 3.70897 3.84421 1.95499 3.38329 4.16060 2.99555 3.53884 2.82933 2.79086 2.70979 2.95522 3.19166 5.56382 4.19174 1124 - -

2.68618 4.42225 2.77519 2.73123 3.46354 2.40513 3.72494 3.29354 2.67741 2.69355 4.24690 2.90347 2.73739 3.18146 2.89801 2.37887 2.77519 2.98518 4.58477 3.61503

0.01712 4.47174 5.19409 0.61958 0.77255 0.48576 0.95510

966 2.98814 5.35169 1.26746 2.46571 4.96719 1.80654 4.05896 4.48024 3.05218 4.00067 4.82115 3.01569 4.04993 3.22535 3.61539 2.23982 3.32997 3.99034 6.16162 4.70742 1125 - -

2.68618 4.42225 2.77519 2.73123 3.46354 2.40513 3.72494 3.29354 2.67741 2.69355 4.24690 2.90347 2.73739 3.18146 2.89801 2.37887 2.77519 2.98518 4.58477 3.61503

0.01712 4.47174 5.19409 0.61958 0.77255 0.48576 0.95510

967 2.75789 4.69411 3.33696 2.78383 3.45357 3.63321 3.81687 3.26339 2.31892 2.91801 3.79008 3.24811 2.44445 3.06781 2.91184 2.87205 2.98777 3.00308 5.11509 1.91322 1126 - -

2.68618 4.42225 2.77519 2.73123 3.46354 2.40513 3.72494 3.29354 2.67741 2.69355 4.24690 2.90347 2.73739 3.18146 2.89801 2.37887 2.77519 2.98518 4.58477 3.61503

0.01712 4.47174 5.19409 0.61958 0.77255 0.48576 0.95510

968 2.72204 4.66181 3.31876 2.35900 3.84476 3.61811 3.83501 2.42681 1.79833 2.86356 3.16818 3.23052 4.00145 3.05327 3.09554 2.84429 2.95227 2.45600 5.21992 3.94404 1127 - -

2.68618 4.42225 2.77519 2.73123 3.46354 2.40513 3.72494 3.29354 2.67741 2.69355 4.24690 2.90347 2.73739 3.18146 2.89801 2.37887 2.77519 2.98518 4.58477 3.61503

0.01712 4.47174 5.19409 0.61958 0.77255 0.48576 0.95510

969 2.96424 4.63979 3.86030 3.26760 3.24634 3.88843 3.93011 3.08630 2.88277 1.82128 3.68189 3.60998 4.25351 3.38375 1.92341 3.17487 3.18510 2.90591 4.78470 1.93532 1128 - -

2.68618 4.42225 2.77519 2.73123 3.46354 2.40513 3.72494 3.29354 2.67741 2.69355 4.24690 2.90347 2.73739 3.18146 2.89801 2.37887 2.77519 2.98518 4.58477 3.61503

0.01712 4.47174 5.19409 0.61958 0.77255 0.48576 0.95510

970 2.44788 5.04397 1.79403 2.28965 4.32399 3.49306 3.71173 3.54465 2.49178 3.32250 4.11063 2.99479 3.89952 2.69228 2.97236 2.71319 2.65932 2.77851 5.52580 4.15713 1129 - -

2.68618 4.42225 2.77519 2.73123 3.46354 2.40513 3.72494 3.29354 2.67741 2.69355 4.24690 2.90347 2.73739 3.18146 2.89801 2.37887 2.77519 2.98518 4.58477 3.61503

0.01712 4.47174 5.19409 0.61958 0.77255 0.48576 0.95510

971 2.04866 5.09731 3.00034 1.96945 4.40178 3.49476 3.37853 3.85375 2.31901 3.38165 3.72939 2.98297 3.88763 2.79551 2.28989 2.47080 2.93864 3.46657 5.55249 4.17719 1130 - -

2.68618 4.42225 2.77519 2.73123 3.46354 2.40513 3.72494 3.29354 2.67741 2.69355 4.24690 2.90347 2.73739 3.18146 2.89801 2.37887 2.77519 2.98518 4.58477 3.61503

0.40024 4.47174 1.14441 0.61958 0.77255 0.48576 0.95510

972 2.79569 5.24107 2.11696 2.30023 4.56761 3.37699 3.66160 4.03469 1.62263 3.52515 4.32071 2.84994 3.85531 2.40612 2.82425 2.74204 3.03546 3.63191 5.66033 4.26891 1131 - -

2.68618 4.42225 2.77519 2.73123 3.46354 2.40513 3.72494 3.29354 2.67741 2.69355 4.24690 2.90347 2.73739 3.18146 2.89801 2.37887 2.77519 2.98518 4.58477 3.61503

0.02502 4.09652 4.81887 0.61958 0.77255 0.90459 0.51870

973 3.24746 4.55072 4.93174 4.38653 3.29626 4.57786 4.97678 1.60164 4.26189 1.13973 3.08690 4.62049 4.79648 4.41539 4.40163 3.93767 3.48396 1.69519 5.33963 4.23286 1132 - -

2.68618 4.42225 2.77519 2.73123 3.46354 2.40513 3.72494 3.29354 2.67741 2.69355 4.24690 2.90347 2.73739 3.18146 2.89801 2.37887 2.77519 2.98518 4.58477 3.61503

0.02502 4.09652 4.81887 0.61958 0.77255 0.90459 0.51870

974 2.54951 4.39302 3.64478 3.41472 4.18432 3.22305 4.41266 3.33963 3.36216 3.19498 4.23571 3.58273 3.94666 3.74691 3.60593 2.75390 0.84170 3.00969 5.60184 4.40281 1133 - -

2.68618 4.42225 2.77519 2.73123 3.46354 2.40513 3.72494 3.29354 2.67741 2.69355 4.24690 2.90347 2.73739 3.18146 2.89801 2.37887 2.77519 2.98518 4.58477 3.61503
0.02502 4.09652 4.81887 0.61958 0.77255 0.90459 0.51870
975 3.16663 4.48000 4.88184 4.40179 3.58075 4.50366 5.10684 0.99770 4.27566 2.05566 3.38670 4.61084 4.82070 4.54025 4.46286 3.90698 3.43815 1.47603 5.58412 4.38065 1134 - -
2.68618 4.42225 2.77519 2.73123 3.46354 2.40513 3.72494 3.29354 2.67741 2.69355 4.24690 2.90347 2.73739 3.18146 2.89801 2.37887 2.77519 2.98518 4.58477 3.61503
0.02502 4.09652 4.81887 0.61958 0.77255 0.90459 0.51870
976 2.31209 4.25553 3.45569 3.13794 4.27307 3.04142 4.20857 3.63465 3.15076 3.36338 4.21426 3.33482 3.76587 3.47270 3.46897 1.37252 1.69609 3.14925 5.62761 4.39515 1135 - -
2.68618 4.42225 2.77519 2.73123 3.46354 2.40513 3.72494 3.29354 2.67741 2.69355 4.24690 2.90347 2.73739 3.18146 2.89801 2.37887 2.77519 2.98518 4.58477 3.61503
0.02502 4.09652 4.81887 0.61958 0.77255 0.90459 0.51870
977 2.37728 4.35897 3.24956 2.97486 4.33897 3.07158 4.14119 3.80014 3.06063 3.45761 4.29583 3.25552 1.92798 3.38271 3.41006 1.37875 2.83280 3.28814 5.65597 4.39466 1136 - -
2.68618 4.42225 2.77519 2.73123 3.46354 2.40513 3.72494 3.29354 2.67741 2.69355 4.24690 2.90347 2.73739 3.18146 2.89801 2.37887 2.77519 2.98518 4.58477 3.61503
0.02502 4.09652 4.81887 0.61958 0.77255 0.90459 0.51870
978 2.96799 4.71525 3.68718 3.61793 4.74485 0.45423 4.71894 4.40446 3.82935 4.02854 5.01019 3.85228 4.11316 4.13617 4.03545 3.14723 3.46664 3.88946 5.77513 4.85627 1137 - -
2.68618 4.42225 2.77519 2.73123 3.46354 2.40513 3.72494 3.29354 2.67741 2.69355 4.24690 2.90347 2.73739 3.18146 2.89801 2.37887 2.77519 2.98518 4.58477 3.61503
0.02502 4.09652 4.81887 0.61958 0.77255 0.90459 0.51870
979 2.83486 5.18281 1.59493 2.35803 4.51260 3.37516 3.73756 4.00569 2.50543 3.52963 4.35671 2.90129 3.88756 2.88649 1.92767 2.79753 3.09393 3.61954 5.65350 4.27881 1138 - -
2.68618 4.42225 2.77519 2.73123 3.46354 2.40513 3.72494 3.29354 2.67741 2.69355 4.24690 2.90347 2.73739 3.18146 2.89801 2.37887 2.77519 2.98518 4.58477 3.61503
0.02502 4.09652 4.81887 0.61958 0.77255 0.90459 0.51870
980 2.96320 5.53946 1.37412 1.71342 4.84699 2.56418 3.79910 4.36742 2.80627 3.85682 4.69391 2.73102 3.88098 2.95980 3.40441 2.85120 3.25006 3.93000 6.01478 4.50894 1139 - -
2.68618 4.42225 2.77519 2.73123 3.46354 2.40513 3.72494 3.29354 2.67741 2.69355 4.24690 2.90347 2.73739 3.18146 2.89801 2.37887 2.77519 2.98518 4.58477 3.61503
0.02502 4.09652 4.81887 0.61958 0.77255 0.90459 0.51870
981 3.20918 4.57349 4.64253 4.27155 3.53493 4.29975 4.96344 0.83672 4.10413 2.08132 3.44180 4.48639 4.71477 4.43298 4.27591 3.82186 3.50654 1.87870 5.46804 4.22192 1140 - -
2.68618 4.42225 2.77519 2.73123 3.46354 2.40513 3.72494 3.29354 2.67741 2.69355 4.24690 2.90347 2.73739 3.18146 2.89801 2.37887 2.77519 2.98518 4.58477 3.61503
0.02502 4.09652 4.81887 0.61958 0.77255 0.90459 0.51870
982 2.77824 4.97349 2.13157 2.39612 3.78069 3.40167 3.70295 3.64831 2.65208 3.23252 4.09702 2.48157 3.89380 2.93707 3.12941 2.78085 3.03265 3.33006 5.19196 1.99844 1141 - -
2.68618 4.42225 2.77519 2.73123 3.46354 2.40513 3.72494 3.29354 2.67741 2.69355 4.24690 2.90347 2.73739 3.18146 2.89801 2.37887 2.77519 2.98518 4.58477 3.61503
0.02502 4.09652 4.81887 0.61958 0.77255 0.29184 1.37393
983 2.18692 4.14272 4.26815 3.67686 3.22702 3.81463 4.12294 2.56714 3.53595 1.64435 3.24839 3.83884 4.18216 3.73894 3.67949 3.10801 2.63650 2.07877 3.16198 3.05241 1142 - -
2.68618 4.42225 2.77519 2.73123 3.46354 2.40513 3.72494 3.29354 2.67741 2.69355 4.24690 2.90347 2.73739 3.18146 2.89801 2.37887 2.77519 2.98518 4.58477 3.61503
0.01712 4.47174 5.19409 0.61958 0.77255 0.48576 0.95510
984 3.57401 5.24861 4.36381 4.35348 5.40728 0.20721 5.39221 5.19546 4.61166 4.75230 5.76109 4.53127 4.65161 4.88936 4.74225 3.76858 4.09816 4.60781 6.30299 5.54286 1143 - -
2.68618 4.42225 2.77519 2.73123 3.46354 2.40513 3.72494 3.29354 2.67741 2.69355 4.24690 2.90347 2.73739 3.18146 2.89801 2.37887 2.77519 2.98518 4.58477 3.61503
0.01712 4.47174 5.19409 0.61958 0.77255 0.48576 0.95510
985 2.62219 5.27181 1.90537 2.29868 4.60362 3.47373 2.89029 4.08628 1.87131 3.56897 4.32211 2.94037 3.90258 2.65768 2.96119 2.56202 3.00744 3.65657 5.70534 4.29143 1144 - -
2.68618 4.42225 2.77519 2.73123 3.46354 2.40513 3.72494 3.29354 2.67741 2.69355 4.24690 2.90347 2.73739 3.18146 2.89801 2.37887 2.77519 2.98518 4.58477 3.61503
0.01712 4.47174 5.19409 0.61958 0.77255 0.48576 0.95510

986 3.47442 4.71197 5.41977 4.89037 3.60080 4.93211 5.41710 1.33115 4.78133 1.22102 3.36879 5.05706 5.12199 4.89365 4.86792 4.31953 3.71400 1.47706 5.67904 4.57635 1145 - -
2.68618 4.42225 2.77519 2.73123 3.46354 2.40513 3.72494 3.29354 2.67741 2.69355 4.24690 2.90347 2.73739 3.18146 2.89801 2.37887 2.77519 2.98518 4.58477 3.61503
0.01712 4.47174 5.19409 0.61958 0.77255 0.48576 0.95510
987 3.01901 4.41709 4.62897 4.05136 2.78311 4.18840 4.48919 2.41342 3.90331 1.40937 3.21547 4.22130 4.50646 3.48143 4.03075 3.50055 3.25021 1.35598 5.00481 3.81828 1146 - -
2.68618 4.42225 2.77519 2.73123 3.46354 2.40513 3.72494 3.29354 2.67741 2.69355 4.24690 2.90347 2.73739 3.18146 2.89801 2.37887 2.77519 2.98518 4.58477 3.61503
0.01712 4.47174 5.19409 0.61958 0.77255 0.48576 0.95510
988 2.46934 4.40604 3.61577 3.05170 2.97494 3.68958 3.45508 2.90345 2.98928 2.61403 3.51363 3.44136 4.07016 3.29466 3.07245 2.94121 2.09228 2.68217 4.92033 2.04967 1147 - -
2.68618 4.42225 2.77519 2.73123 3.46354 2.40513 3.72494 3.29354 2.67741 2.69355 4.24690 2.90347 2.73739 3.18146 2.89801 2.37887 2.77519 2.98518 4.58477 3.61503
0.01712 4.47174 5.19409 0.61958 0.77255 0.48576 0.95510
989 2.25724 5.10042 2.76923 1.68976 4.40118 3.48315 3.69190 3.85504 2.39289 3.38829 4.15967 2.97265 3.88656 2.80837 2.75862 2.45449 2.94358 2.82766 5.56549 4.18327 1148 - -
2.68618 4.42225 2.77519 2.73123 3.46354 2.40513 3.72494 3.29354 2.67741 2.69355 4.24690 2.90347 2.73739 3.18146 2.89801 2.37887 2.77519 2.98518 4.58477 3.61503
0.01712 4.47174 5.19409 0.61958 0.77255 0.48576 0.95510
990 2.69841 4.80109 3.13689 2.37619 4.01936 3.54069 3.76375 3.16593 2.59235 3.05464 3.88775 2.87209 2.03326 2.93884 3.03968 2.66011 2.50086 3.12392 5.32738 3.11725 1149 - -
2.68618 4.42225 2.77519 2.73123 3.46354 2.40513 3.72494 3.29354 2.67741 2.69355 4.24690 2.90347 2.73739 3.18146 2.89801 2.37887 2.77519 2.98518 4.58477 3.61503
0.01712 4.47174 5.19409 0.61958 0.77255 0.48576 0.95510
991 3.03458 4.57085 4.23036 3.70464 2.68157 4.05799 4.32806 2.69580 3.60018 1.38161 3.33561 3.98384 1.68492 3.84777 3.83548 3.37663 3.28584 2.65642 4.96156 3.64466 1150 - -
2.68618 4.42225 2.77519 2.73123 3.46354 2.40513 3.72494 3.29354 2.67741 2.69355 4.24690 2.90347 2.73739 3.18146 2.89801 2.37887 2.77519 2.98518 4.58477 3.61503
0.01712 4.47174 5.19409 0.61958 0.77255 0.48576 0.95510
992 3.13450 5.34534 3.55871 2.88612 4.81089 3.80769 3.79105 4.16837 1.50634 3.61064 4.44432 3.32182 4.17411 2.91432 1.27244 2.88222 2.81811 3.81848 5.66865 4.46151 1151 - -
2.68618 4.42225 2.77519 2.73123 3.46354 2.40513 3.72494 3.29354 2.67741 2.69355 4.24690 2.90347 2.73739 3.18146 2.89801 2.37887 2.77519 2.98518 4.58477 3.61503
0.01712 4.47174 5.19409 0.61958 0.77255 0.48576 0.95510
993 3.03478 5.54099 1.38377 2.37392 4.95366 2.04913 3.95876 4.46892 2.91281 3.95811 4.76693 2.16106 4.03624 3.10832 3.48370 2.50463 3.32767 4.01129 6.11222 4.63329 1152 - -
2.68618 4.42225 2.77519 2.73123 3.46354 2.40513 3.72494 3.29354 2.67741 2.69355 4.24690 2.90347 2.73739 3.18146 2.89801 2.37887 2.77519 2.98518 4.58477 3.61503
0.01712 4.47174 5.19409 0.61958 0.77255 0.48576 0.95510
994 3.06147 5.26930 3.37947 2.82202 4.73615 1.62949 3.82623 4.12282 1.88599 3.59777 4.42838 3.27694 4.13709 2.95756 1.65034 3.06006 3.27557 3.76175 5.68375 4.44593 1153 - -
2.68618 4.42225 2.77519 2.73123 3.46354 2.40513 3.72494 3.29354 2.67741 2.69355 4.24690 2.90347 2.73739 3.18146 2.89801 2.37887 2.77519 2.98518 4.58477 3.61503
0.01712 4.47174 5.19409 0.61958 0.77255 0.48576 0.95510
995 2.63616 4.51704 3.67672 3.26507 4.09549 3.42322 4.26545 3.39457 3.18908 2.81710 4.08251 3.54295 1.06761 3.55157 3.50798 2.54146 3.03266 2.76709 5.52730 4.26295 1154 - -
2.68618 4.42225 2.77519 2.73123 3.46354 2.40513 3.72494 3.29354 2.67741 2.69355 4.24690 2.90347 2.73739 3.18146 2.89801 2.37887 2.77519 2.98518 4.58477 3.61503
0.40024 4.47174 1.14441 0.61958 0.77255 0.48576 0.95510
996 2.54951 4.39302 3.64478 3.41472 4.18432 3.22305 4.41266 3.33963 3.36216 3.19498 4.23571 3.58273 3.94666 3.74691 3.60593 2.75390 0.84170 3.00969 5.60184 4.40281 1155 - -
2.68618 4.42225 2.77519 2.73123 3.46354 2.40513 3.72494 3.29354 2.67741 2.69355 4.24690 2.90347 2.73739 3.18146 2.89801 2.37887 2.77519 2.98518 4.58477 3.61503
0.02502 4.09652 4.81887 0.61958 0.77255 0.29184 1.37393
997 2.14952 4.24719 3.44458 3.33387 2.88872 3.75081 4.03712 2.68883 3.24984 1.96759 2.23828 3.63338 3.37318 3.50689 3.50773 3.02184 2.92511 2.35696 4.85163 3.64750 1156 - -
2.68618 4.42225 2.77519 2.73123 3.46354 2.40513 3.72494 3.29354 2.67741 2.69355 4.24690 2.90347 2.73739 3.18146 2.89801 2.37887 2.77519 2.98518 4.58477 3.61503

0.40024 4.47174 1.14441 0.61958 0.77255 0.48576 0.95510

998 3.73511 5.02498 4.45117 4.21677 2.93235 3.99608 4.20435 3.77473 3.93763 3.12831 4.39709 4.32223 4.53360 4.31344 4.02452 3.93646 4.04815 3.67102 0.53344 2.92368 1157 - -

2.68618 4.42225 2.77519 2.73123 3.46354 2.40513 3.72494 3.29354 2.67741 2.69355 4.24690 2.90347 2.73739 3.18146 2.89801 2.37887 2.77519 2.98518 4.58477 3.61503

0.02502 4.09652 4.81887 0.61958 0.77255 0.90459 0.51870

999 3.16393 5.30333 2.60663 0.74394 4.68056 3.43230 4.04645 4.18022 2.95681 3.76379 4.73428 3.08464 4.04944 3.26144 3.38441 3.12707 3.47193 3.84321 5.80957 4.53968 1158 - -

2.68618 4.42225 2.77519 2.73123 3.46354 2.40513 3.72494 3.29354 2.67741 2.69355 4.24690 2.90347 2.73739 3.18146 2.89801 2.37887 2.77519 2.98518 4.58477 3.61503

0.02502 4.09652 4.81887 0.61958 0.77255 0.29184 1.37393

1000 2.68770 4.15711 4.17365 3.58611 2.21262 3.79369 4.09424 1.71823 3.13324 2.34397 2.85017 3.40673 3.64045 3.67702 3.63033 3.08154 2.91973 2.40429 4.76199 3.27121 1159 - -

2.68618 4.42225 2.77519 2.73123 3.46354 2.40513 3.72494 3.29354 2.67741 2.69355 4.24690 2.90347 2.73739 3.18146 2.89801 2.37887 2.77519 2.98518 4.58477 3.61503

0.01712 4.47174 5.19409 0.61958 0.77255 0.48576 0.95510

1001 3.15684 5.46297 2.65458 1.23667 5.01005 1.32793 4.15055 4.52232 3.10562 4.05437 4.91118 3.12844 4.15452 3.33121 3.61264 3.11532 3.48558 4.07792 6.15796 4.77608 1160 - -

2.68618 4.42225 2.77519 2.73123 3.46354 2.40513 3.72494 3.29354 2.67741 2.69355 4.24690 2.90347 2.73739 3.18146 2.89801 2.37887 2.77519 2.98518 4.58477 3.61503

0.01712 4.47174 5.19409 0.61958 0.77255 0.48576 0.95510

1002 1.90660 4.42361 3.58355 3.02793 3.60156 3.65004 3.96014 2.05547 2.97534 2.65992 3.56088 3.42449 4.05587 2.66414 3.32641 2.43394 2.93702 2.42110 5.04910 3.81446 1161 - -

2.68618 4.42225 2.77519 2.73123 3.46354 2.40513 3.72494 3.29354 2.67741 2.69355 4.24690 2.90347 2.73739 3.18146 2.89801 2.37887 2.77519 2.98518 4.58477 3.61503

0.01712 4.47174 5.19409 0.61958 0.77255 0.48576 0.95510

1003 3.21505 5.54161 1.21105 2.56632 5.05607 3.52996 4.19727 4.61216 3.23657 4.14054 5.01410 3.12809 1.33946 3.38674 3.79932 3.15990 3.55154 4.16240 6.22289 4.81984 1162 - -

2.68618 4.42225 2.77519 2.73123 3.46354 2.40513 3.72494 3.29354 2.67741 2.69355 4.24690 2.90347 2.73739 3.18146 2.89801 2.37887 2.77519 2.98518 4.58477 3.61503

0.01712 4.47174 5.19409 0.61958 0.77255 0.48576 0.95510

1004 3.76994 5.86439 0.34171 2.92082 5.35427 3.79231 4.62543 5.10926 3.84350 4.61566 5.64508 3.49443 4.48598 3.89084 4.41191 3.68955 4.13224 4.69415 6.38349 5.17761 1163 - -

2.68618 4.42225 2.77519 2.73123 3.46354 2.40513 3.72494 3.29354 2.67741 2.69355 4.24690 2.90347 2.73739 3.18146 2.89801 2.37887 2.77519 2.98518 4.58477 3.61503

0.01712 4.47174 5.19409 0.61958 0.77255 0.48576 0.95510

1005 3.30915 5.45057 3.66439 3.02348 4.90385 3.90016 3.85358 4.29426 2.12494 3.69736 4.57066 3.42979 4.27667 1.47771 1.22991 3.29285 3.47681 3.96476 5.71163 4.53515 1164 - -

2.68618 4.42225 2.77519 2.73123 3.46354 2.40513 3.72494 3.29354 2.67741 2.69355 4.24690 2.90347 2.73739 3.18146 2.89801 2.37887 2.77519 2.98518 4.58477 3.61503

0.01712 4.47174 5.19409 0.61958 0.77255 0.48576 0.95510

1006 2.50102 4.44417 3.83543 3.57232 4.69342 3.21627 4.61468 4.09698 3.60188 3.81172 4.64017 3.65741 3.99164 3.89692 3.88552 0.77423 2.00073 3.50724 6.03655 4.83225 1165 - -

2.68618 4.42225 2.77519 2.73123 3.46354 2.40513 3.72494 3.29354 2.67741 2.69355 4.24690 2.90347 2.73739 3.18146 2.89801 2.37887 2.77519 2.98518 4.58477 3.61503

0.01712 4.47174 5.19409 0.61958 0.77255 0.48576 0.95510

1007 0.41286 4.61633 4.19984 4.06824 4.77770 3.41735 4.99816 4.00726 4.10417 3.85501 4.84607 4.02564 4.21468 4.38983 4.27943 2.97576 3.29622 3.54376 6.13289 5.02381 1166 - -

2.68618 4.42225 2.77519 2.73123 3.46354 2.40513 3.72494 3.29354 2.67741 2.69355 4.24690 2.90347 2.73739 3.18146 2.89801 2.37887 2.77519 2.98518 4.58477 3.61503

0.01712 4.47174 5.19409 0.61958 0.77255 0.48576 0.95510

1008 2.08496 4.34940 3.81393 3.26004 3.51202 3.68959 4.07589 2.79732 3.18190 1.68675 2.98174 3.58680 4.11580 3.47132 3.47964 2.03243 2.96462 2.59499 5.01102 3.79765 1167 - -

2.68618 4.42225 2.77519 2.73123 3.46354 2.40513 3.72494 3.29354 2.67741 2.69355 4.24690 2.90347 2.73739 3.18146 2.89801 2.37887 2.77519 2.98518 4.58477 3.61503

0.01712 4.47174 5.19409 0.61958 0.77255 0.48576 0.95510

1009 2.90065 4.57198 3.75388 1.69084 3.74376 3.92079 4.20629 1.92586 3.17054 2.42355 3.63053 3.64097 4.29017 3.49653 3.53473 3.18530 3.13855 1.83547 5.31318 4.06995 1168 - -

2.68618 4.42225 2.77519 2.73123 3.46354 2.40513 3.72494 3.29354 2.67741 2.69355 4.24690 2.90347 2.73739 3.18146 2.89801 2.37887 2.77519 2.98518 4.58477 3.61503
0.01712 4.47174 5.19409 0.61958 0.77255 0.48576 0.95510
1010 4.35404 5.39616 5.23188 5.06341 0.88986 4.93345 3.19290 4.03922 4.86617 3.26802 4.57697 4.50547 5.20498 4.64264 4.75958 4.32057 4.55482 3.95730 3.78767 1.08475 1169 - -
2.68618 4.42225 2.77519 2.73123 3.46354 2.40513 3.72494 3.29354 2.67741 2.69355 4.24690 2.90347 2.73739 3.18146 2.89801 2.37887 2.77519 2.98518 4.58477 3.61503
0.01712 4.47174 5.19409 0.61958 0.77255 0.48576 0.95510
1011 3.10925 4.46437 4.85853 4.28306 1.82772 4.26827 4.38863 2.35633 4.11211 1.31309 2.87901 4.33367 4.56597 4.19735 4.15428 3.58700 3.33348 2.57168 4.73674 2.34348 1170 - -
2.68618 4.42225 2.77519 2.73123 3.46354 2.40513 3.72494 3.29354 2.67741 2.69355 4.24690 2.90347 2.73739 3.18146 2.89801 2.37887 2.77519 2.98518 4.58477 3.61503
0.01712 4.47174 5.19409 0.61958 0.77255 0.48576 0.95510
1012 3.46769 4.71007 5.40034 4.86782 3.58446 4.91270 5.38743 1.54566 4.75604 1.15439 3.35550 5.03488 5.10553 4.86696 4.84200 4.29693 3.70661 1.36390 5.65542 4.55405 1171 - -
2.68618 4.42225 2.77519 2.73123 3.46354 2.40513 3.72494 3.29354 2.67741 2.69355 4.24690 2.90347 2.73739 3.18146 2.89801 2.37887 2.77519 2.98518 4.58477 3.61503
0.01712 4.47174 5.19409 0.61958 0.77255 0.48576 0.95510
1013 2.45078 4.37569 3.65586 3.09744 3.52647 3.66812 3.97622 2.86451 3.03674 2.04798 3.50407 3.47166 1.94929 3.33695 3.36663 2.72845 2.93298 2.41296 4.98910 3.38086 1172 - -
2.68618 4.42225 2.77519 2.73123 3.46354 2.40513 3.72494 3.29354 2.67741 2.69355 4.24690 2.90347 2.73739 3.18146 2.89801 2.37887 2.77519 2.98518 4.58477 3.61503
0.01712 4.47174 5.19409 0.61958 0.77255 0.48576 0.95510
1014 2.83229 5.31924 1.60479 2.24804 4.63578 3.47170 3.76391 4.11677 2.42864 3.61463 4.38438 2.94078 3.93510 2.69879 3.07942 2.28974 2.36372 3.69770 5.76722 4.34521 1173 - -
2.68618 4.42225 2.77519 2.73123 3.46354 2.40513 3.72494 3.29354 2.67741 2.69355 4.24690 2.90347 2.73739 3.18146 2.89801 2.37887 2.77519 2.98518 4.58477 3.61503
0.01712 4.47174 5.19409 0.61958 0.77255 0.48576 0.95510
1015 2.81888 5.19895 2.88656 2.32376 4.50735 3.51448 3.04674 3.96224 2.49004 3.48703 4.27458 3.00446 1.88977 2.05437 2.93816 2.62918 3.05480 3.57852 5.65214 4.27686 1174 - -
2.68618 4.42225 2.77519 2.73123 3.46354 2.40513 3.72494 3.29354 2.67741 2.69355 4.24690 2.90347 2.73739 3.18146 2.89801 2.37887 2.77519 2.98518 4.58477 3.61503
0.01712 4.47174 5.19409 0.61958 0.77255 0.48576 0.95510
1016 2.77009 5.27153 2.42578 2.27565 4.61061 3.19931 3.70283 4.09225 2.04920 3.56927 4.32051 1.95648 3.90290 2.80843 2.31299 2.72557 3.00411 3.66033 5.69947 4.29009 1175 - -
2.68618 4.42225 2.77519 2.73123 3.46354 2.40513 3.72494 3.29354 2.67741 2.69355 4.24690 2.90347 2.73739 3.18146 2.89801 2.37887 2.77519 2.98518 4.58477 3.61503
0.01712 4.47174 5.19409 0.61958 0.77255 0.48576 0.95510
1017 2.86816 5.35469 2.51681 1.73377 4.66404 3.47526 3.38972 4.14592 2.60750 3.64592 4.42324 2.94294 1.81906 2.74264 3.11882 2.63253 3.11422 3.73020 5.80119 4.37586 1176 - -
2.68618 4.42225 2.77519 2.73123 3.46354 2.40513 3.72494 3.29354 2.67741 2.69355 4.24690 2.90347 2.73739 3.18146 2.89801 2.37887 2.77519 2.98518 4.58477 3.61503
0.01712 4.47174 5.19409 0.61958 0.77255 0.48576 0.95510
1018 2.64452 5.10863 3.01498 2.45951 4.42658 3.50165 3.68667 3.87751 1.88964 3.40022 4.17040 2.17009 3.52412 2.80026 2.52449 2.70589 2.40416 3.21950 5.56473 4.19322 1177 - -
2.68618 4.42225 2.77519 2.73123 3.46354 2.40513 3.72494 3.29354 2.67741 2.69355 4.24690 2.90347 2.73739 3.18146 2.89801 2.37887 2.77519 2.98518 4.58477 3.61503
0.01712 4.47174 5.19409 0.61958 0.77255 0.48576 0.95510
1019 3.89228 5.10892 5.02890 4.69411 2.23274 4.68878 3.76802 3.60760 4.47184 2.16136 4.19122 4.38915 4.98618 4.44077 4.47125 4.04500 4.10646 3.51107 1.49256 1.14446 1178 - -
2.68618 4.42225 2.77519 2.73123 3.46354 2.40513 3.72494 3.29354 2.67741 2.69355 4.24690 2.90347 2.73739 3.18146 2.89801 2.37887 2.77519 2.98518 4.58477 3.61503
0.01712 4.47174 5.19409 0.61958 0.77255 0.48576 0.95510
1020 2.68779 4.12494 4.30687 3.71257 2.62605 2.64452 4.12830 2.34579 3.56630 2.12080 2.72554 3.85613 4.18177 3.76009 3.34262 3.11024 2.91986 1.78840 4.73992 3.23456 1179 - -
2.68618 4.42225 2.77519 2.73123 3.46354 2.40513 3.72494 3.29354 2.67741 2.69355 4.24690 2.90347 2.73739 3.18146 2.89801 2.37887 2.77519 2.98518 4.58477 3.61503
0.01712 4.47174 5.19409 0.61958 0.77255 0.48576 0.95510

1021 2.47949 5.26548 2.44213 2.04945 4.59398 3.29044 3.73211 4.07447 2.51955 3.57026 4.33080 1.83608 3.48071 2.84524 3.02510 2.74024 2.51401 3.65129 5.71918 4.30267 1180 - -
2.68618 4.42225 2.77519 2.73123 3.46354 2.40513 3.72494 3.29354 2.67741 2.69355 4.24690 2.90347 2.73739 3.18146 2.89801 2.37887 2.77519 2.98518 4.58477 3.61503
0.01712 4.47174 5.19409 0.61958 0.77255 0.48576 0.95510
1022 3.49467 5.56987 4.03727 3.20929 5.15137 4.04210 3.85059 4.43643 0.89880 3.78479 4.66997 3.55399 4.37289 2.97657 1.45711 3.46827 3.61262 4.12163 5.74441 4.65364 1181 - -
2.68618 4.42225 2.77519 2.73123 3.46354 2.40513 3.72494 3.29354 2.67741 2.69355 4.24690 2.90347 2.73739 3.18146 2.89801 2.37887 2.77519 2.98518 4.58477 3.61503
0.01712 4.47174 5.19409 0.61958 0.77255 0.48576 0.95510
1023 3.24694 4.54479 5.09130 4.54328 3.60114 4.59684 5.04551 1.73724 4.41849 1.31891 3.42835 4.69439 4.87277 4.59354 4.53550 3.95050 3.03604 1.27400 5.48196 4.32907 1182 - -
2.68618 4.42225 2.77519 2.73123 3.46354 2.40513 3.72494 3.29354 2.67741 2.69355 4.24690 2.90347 2.73739 3.18146 2.89801 2.37887 2.77519 2.98518 4.58477 3.61503
0.01712 4.47174 5.19409 0.61958 0.77255 0.48576 0.95510
1024 4.39535 5.42306 5.26633 5.12425 1.41650 4.95515 3.71947 3.99177 4.92642 3.19286 4.54092 4.54061 5.22966 4.68282 4.80260 4.36089 4.60185 3.94376 3.80694 0.64823 1183 - -
2.68618 4.42225 2.77519 2.73123 3.46354 2.40513 3.72494 3.29354 2.67741 2.69355 4.24690 2.90347 2.73739 3.18146 2.89801 2.37887 2.77519 2.98518 4.58477 3.61503
0.01712 4.47174 5.19409 0.61958 0.77255 0.48576 0.95510
1025 3.49099 4.72644 5.43265 4.90436 3.59560 4.95200 5.43705 1.19148 4.79540 1.25576 3.36067 5.07612 5.13567 4.90418 4.88190 4.34168 3.73033 1.60575 5.68769 4.58930 1184 - -
2.68618 4.42225 2.77519 2.73123 3.46354 2.40513 3.72494 3.29354 2.67741 2.69355 4.24690 2.90347 2.73739 3.18146 2.89801 2.37887 2.77519 2.98518 4.58477 3.61503
0.01712 4.47174 5.19409 0.61958 0.77255 0.48576 0.95510
1026 2.98375 5.44383 2.59916 1.39875 4.80224 3.47724 3.89594 4.28181 2.75761 3.79413 4.60012 1.62851 4.01965 3.03826 3.26693 2.92196 2.71825 3.86219 5.95213 4.51647 1185 - -
2.68631 4.42238 2.77533 2.73137 3.46367 2.40526 3.72305 3.29367 2.67754 2.69368 4.24703 2.90321 2.73720 3.18160 2.89814 2.37877 2.77533 2.98532 4.58490 3.61436
0.96511 1.20182 1.14441 0.64572 0.74294 0.48576 0.95510
1027 2.78313 5.02329 2.88658 2.47920 4.37070 3.44526 3.67698 3.79405 2.04172 3.33219 4.16166 2.98421 2.05421 2.23433 2.64772 2.78319 3.02087 3.44333 5.49685 4.18370 1190 - -
2.68618 4.42225 2.77519 2.73123 3.46354 2.40513 3.72494 3.29354 2.67741 2.69355 4.24690 2.90347 2.73739 3.18146 2.89801 2.37887 2.77519 2.98518 4.58477 3.61503
0.02502 4.09652 4.81887 0.61958 0.77255 0.29184 1.37393
1028 2.98761 5.53027 1.35401 2.35516 4.86117 3.08308 3.87880 4.36634 2.54001 3.84902 4.64075 2.08684 4.00473 3.01471 3.32239 2.33560 3.25265 3.92698 5.99776 4.53256 1191 - -
2.68618 4.42225 2.77519 2.73123 3.46354 2.40513 3.72494 3.29354 2.67741 2.69355 4.24690 2.90347 2.73739 3.18146 2.89801 2.37887 2.77519 2.98518 4.58477 3.61503
0.01712 4.47174 5.19409 0.61958 0.77255 0.48576 0.95510
1029 2.74260 5.20962 2.63845 2.42415 4.54951 2.85216 3.68199 4.01976 2.15311 3.50555 4.25901 2.96644 3.89520 2.43243 1.88070 2.40921 2.97233 3.59909 5.63961 4.24805 1192 - -
2.68618 4.42225 2.77519 2.73123 3.46354 2.40513 3.72494 3.29354 2.67741 2.69355 4.24690 2.90347 2.73739 3.18146 2.89801 2.37887 2.77519 2.98518 4.58477 3.61503
0.01712 4.47174 5.19409 0.61958 0.77255 0.48576 0.95510
1030 4.40346 5.42121 5.29054 5.15571 0.99105 4.96514 3.71727 3.99118 4.95410 3.18983 4.53745 4.54804 5.23563 4.69345 4.81697 4.36938 4.60790 3.94422 3.80274 0.92837 1193 - -
2.68618 4.42225 2.77519 2.73123 3.46354 2.40513 3.72494 3.29354 2.67741 2.69355 4.24690 2.90347 2.73739 3.18146 2.89801 2.37887 2.77519 2.98518 4.58477 3.61503
0.01712 4.47174 5.19409 0.61958 0.77255 0.48576 0.95510
1031 2.77064 4.78654 3.05828 2.71975 3.09837 3.62193 3.77879 3.38357 2.65826 3.01232 3.87642 2.98777 4.01071 3.02823 1.76491 2.85137 3.00061 3.10728 5.08583 2.29240 1194 - -
2.68618 4.42225 2.77519 2.73123 3.46354 2.40513 3.72494 3.29354 2.67741 2.69355 4.24690 2.90347 2.73739 3.18146 2.89801 2.37887 2.77519 2.98518 4.58477 3.61503
0.01712 4.47174 5.19409 0.61958 0.77255 0.48576 0.95510
1032 3.03984 4.68886 3.91592 3.37628 2.49897 4.00937 4.14625 2.85736 3.24699 1.53015 3.41911 3.75277 4.35826 1.65253 3.54859 3.28851 3.26876 2.81906 4.93700 3.56188 1195 - -
2.68618 4.42225 2.77519 2.73123 3.46354 2.40513 3.72494 3.29354 2.67741 2.69355 4.24690 2.90347 2.73739 3.18146 2.89801 2.37887 2.77519 2.98518 4.58477 3.61503

0.01712 4.47174 5.19409 0.61958 0.77255 0.48576 0.95510

1033 2.83775 5.01719 3.03101 2.32435 3.99595 3.58265 2.29722 3.67572 2.57743 3.25879 4.09833 2.91295 4.00352 2.74266 2.99752 2.85956 3.06901 3.36083 5.33001 1.86141 1196 - -

2.68618 4.42225 2.77519 2.73123 3.46354 2.40513 3.72494 3.29354 2.67741 2.69355 4.24690 2.90347 2.73739 3.18146 2.89801 2.37887 2.77519 2.98518 4.58477 3.61503

0.01712 4.47174 5.19409 0.61958 0.77255 0.48576 0.95510

1034 3.57401 5.24861 4.36381 4.35348 5.40728 0.20721 5.39221 5.19546 4.61166 4.75230 5.76109 4.53127 4.65161 4.88936 4.74225 3.76858 4.09816 4.60781 6.30299 5.54286 1197 - -

2.68618 4.42225 2.77519 2.73123 3.46354 2.40513 3.72494 3.29354 2.67741 2.69355 4.24690 2.90347 2.73739 3.18146 2.89801 2.37887 2.77519 2.98518 4.58477 3.61503

0.01712 4.47174 5.19409 0.61958 0.77255 0.48576 0.95510

1035 3.58504 4.86288 5.04303 4.60598 1.64653 4.58965 4.03873 3.01848 4.42973 1.13075 3.58849 4.45490 4.86546 4.41179 4.43406 3.93228 3.80295 3.03444 4.20786 1.78284 1198 - -

2.68618 4.42225 2.77519 2.73123 3.46354 2.40513 3.72494 3.29354 2.67741 2.69355 4.24690 2.90347 2.73739 3.18146 2.89801 2.37887 2.77519 2.98518 4.58477 3.61503

0.01712 4.47174 5.19409 0.61958 0.77255 0.48576 0.95510

1036 4.50179 5.62504 5.10517 4.98327 3.53162 4.49500 4.82933 4.61303 4.73401 3.88530 5.20744 5.03524 5.06565 5.08388 4.74150 4.70107 4.84079 4.51410 0.23110 3.50965 1199 - -

2.68618 4.42225 2.77519 2.73123 3.46354 2.40513 3.72494 3.29354 2.67741 2.69355 4.24690 2.90347 2.73739 3.18146 2.89801 2.37887 2.77519 2.98518 4.58477 3.61503

0.01712 4.47174 5.19409 0.61958 0.77255 0.48576 0.95510

1037 2.43540 5.12480 2.47224 1.56777 4.42674 3.04728 3.71341 3.87663 2.49071 2.95042 4.19147 2.97160 3.89922 2.83313 2.97999 2.71595 2.97062 3.17811 5.59659 4.21007 1200 - -

2.68618 4.42225 2.77519 2.73123 3.46354 2.40513 3.72494 3.29354 2.67741 2.69355 4.24690 2.90347 2.73739 3.18146 2.89801 2.37887 2.77519 2.98518 4.58477 3.61503

0.01712 4.47174 5.19409 0.61958 0.77255 0.48576 0.95510

1038 2.74257 5.11017 2.81096 2.15436 4.41286 2.54310 3.70890 3.27479 2.44696 3.39481 4.17465 2.99718 3.91044 2.82934 1.77226 2.72964 2.97464 3.47939 5.57160 4.20110 1201 - -

2.68618 4.42225 2.77519 2.73123 3.46354 2.40513 3.72494 3.29354 2.67741 2.69355 4.24690 2.90347 2.73739 3.18146 2.89801 2.37887 2.77519 2.98518 4.58477 3.61503

0.01712 4.47174 5.19409 0.61958 0.77255 0.48576 0.95510

1039 3.05942 4.44119 4.69402 4.12284 3.14263 4.22470 4.42078 1.96968 3.96667 1.55336 3.25935 4.25218 4.53921 4.12465 4.07555 3.53931 3.28928 2.21443 4.86159 1.62756 1202 - -

2.68618 4.42225 2.77519 2.73123 3.46354 2.40513 3.72494 3.29354 2.67741 2.69355 4.24690 2.90347 2.73739 3.18146 2.89801 2.37887 2.77519 2.98518 4.58477 3.61503

0.01712 4.47174 5.19409 0.61958 0.77255 0.48576 0.95510

1040 1.39874 4.34875 4.08223 3.65106 4.02047 3.41185 4.47410 3.08076 3.59157 3.04667 3.97403 3.75681 4.07956 3.85767 3.84862 2.18560 2.99421 1.45907 5.50155 4.30523 1203 - -

2.68618 4.42225 2.77519 2.73123 3.46354 2.40513 3.72494 3.29354 2.67741 2.69355 4.24690 2.90347 2.73739 3.18146 2.89801 2.37887 2.77519 2.98518 4.58477 3.61503

0.01712 4.47174 5.19409 0.61958 0.77255 0.48576 0.95510

1041 2.84736 5.31828 1.76777 1.62785 4.63334 3.15067 3.78395 4.10861 2.60835 3.61865 4.39578 2.94466 3.46222 2.90803 3.12410 2.79908 3.09500 2.79763 5.78059 4.35816 1204 - -

2.68618 4.42225 2.77519 2.73123 3.46354 2.40513 3.72494 3.29354 2.67741 2.69355 4.24690 2.90347 2.73739 3.18146 2.89801 2.37887 2.77519 2.98518 4.58477 3.61503

0.01712 4.47174 5.19409 0.61958 0.77255 0.48576 0.95510

1042 2.95002 4.92789 3.52592 2.89904 4.18068 3.31130 3.83014 3.54258 2.03572 1.64249 4.02101 3.33282 4.12018 3.01234 1.82377 3.03183 3.15565 3.27317 5.38628 4.15609 1205 - -

2.68618 4.42225 2.77519 2.73123 3.46354 2.40513 3.72494 3.29354 2.67741 2.69355 4.24690 2.90347 2.73739 3.18146 2.89801 2.37887 2.77519 2.98518 4.58477 3.61503

0.27226 4.47174 1.48318 0.61958 0.77255 0.48576 0.95510

1043 3.42574 4.82782 4.41431 4.00820 2.36218 4.27840 3.79004 3.07094 3.78889 1.85929 3.74251 4.05569 4.62799 4.00577 3.93710 3.64849 3.66342 3.00812 4.04322 1.00338 1206 - -

2.68618 4.42225 2.77519 2.73123 3.46354 2.40513 3.72494 3.29354 2.67741 2.69355 4.24690 2.90347 2.73739 3.18146 2.89801 2.37887 2.77519 2.98518 4.58477 3.61503

0.02204 4.22153 4.94388 0.61958 0.77255 0.79915 0.59731

1044 2.66011 4.81781 2.44246 2.53362 4.50210 2.86154 3.94731 3.95881 2.87474 3.55409 4.37693 3.01919 1.40951 3.12988 3.35069 2.45742 3.02301 3.51102 5.76106 4.40749 1207 - -

2.68618 4.42225 2.77519 2.73123 3.46354 2.40513 3.72494 3.29354 2.67741 2.69355 4.24690 2.90347 2.73739 3.18146 2.89801 2.37887 2.77519 2.98518 4.58477 3.61503
0.02204 4.22153 4.94388 0.61958 0.77255 0.79915 0.59731
1045 2.86350 5.33563 1.43395 2.29278 4.65639 3.37670 3.75498 4.15290 2.58834 3.65226 4.45309 2.41721 3.90042 2.89341 2.36442 2.79956 3.12242 3.73707 5.79562 4.36462 1208 - -
2.68618 4.42225 2.77519 2.73123 3.46354 2.40513 3.72494 3.29354 2.67741 2.69355 4.24690 2.90347 2.73739 3.18146 2.89801 2.37887 2.77519 2.98518 4.58477 3.61503
0.02204 4.22153 4.94388 0.61958 0.77255 0.79915 0.59731
1046 2.58437 4.51194 3.25907 2.78282 3.85922 1.86281 3.87680 2.63368 2.77523 2.91182 3.78942 2.49110 3.90992 3.11399 3.16980 2.72552 2.88164 2.65852 5.24352 3.96667 1209 - -
2.68618 4.42225 2.77519 2.73123 3.46354 2.40513 3.72494 3.29354 2.67741 2.69355 4.24690 2.90347 2.73739 3.18146 2.89801 2.37887 2.77519 2.98518 4.58477 3.61503
0.02204 4.22153 4.94388 0.61958 0.77255 0.79915 0.59731
1047 2.49688 5.07566 1.20399 2.38818 4.62059 3.30273 3.90164 3.99866 2.84994 3.63665 4.48075 2.92425 3.90906 3.07097 3.37276 2.79243 2.51203 3.58943 5.86212 4.45170 1210 - -
2.68618 4.42225 2.77519 2.73123 3.46354 2.40513 3.72494 3.29354 2.67741 2.69355 4.24690 2.90347 2.73739 3.18146 2.89801 2.37887 2.77519 2.98518 4.58477 3.61503
0.02204 4.22153 4.94388 0.61958 0.77255 0.79915 0.59731
1048 2.54542 4.53070 3.41417 2.53095 3.60775 3.67394 4.02867 2.76565 2.95455 1.32325 3.53540 3.42960 4.11223 3.33139 3.27493 3.01779 3.05486 2.60942 5.15603 3.91986 1211 - -
2.68618 4.42225 2.77519 2.73123 3.46354 2.40513 3.72494 3.29354 2.67741 2.69355 4.24690 2.90347 2.73739 3.18146 2.89801 2.37887 2.77519 2.98518 4.58477 3.61503
0.02204 4.22153 4.94388 0.61958 0.77255 0.79915 0.59731
1049 2.81596 4.49052 3.84521 3.31710 3.71191 3.71948 4.13448 2.63234 2.90842 2.61522 3.66960 3.63266 4.18437 3.45183 2.31900 3.08739 3.10026 1.22012 5.22511 3.98840 1212 - -
2.68618 4.42225 2.77519 2.73123 3.46354 2.40513 3.72494 3.29354 2.67741 2.69355 4.24690 2.90347 2.73739 3.18146 2.89801 2.37887 2.77519 2.98518 4.58477 3.61503
0.02204 4.22153 4.94388 0.61958 0.77255 0.33025 1.26849
1050 4.39167 5.42221 5.26106 5.11709 1.46080 4.95227 3.72037 3.98948 4.91999 3.19157 4.53945 4.53899 5.22772 4.68031 4.79901 4.35830 4.59870 3.94137 3.80834 0.62915 1213 - -
2.68618 4.42225 2.77519 2.73123 3.46354 2.40513 3.72494 3.29354 2.67741 2.69355 4.24690 2.90347 2.73739 3.18146 2.89801 2.37887 2.77519 2.98518 4.58477 3.61503
0.01712 4.47174 5.19409 0.61958 0.77255 0.48576 0.95510
1051 2.81532 4.99570 2.98301 1.63036 4.31332 3.52912 3.86512 3.64119 2.65970 3.00824 4.16126 3.11224 4.00024 3.02635 3.09557 2.84852 1.64293 3.32416 5.58346 4.24382 1214 - -
2.68618 4.42225 2.77519 2.73123 3.46354 2.40513 3.72494 3.29354 2.67741 2.69355 4.24690 2.90347 2.73739 3.18146 2.89801 2.37887 2.77519 2.98518 4.58477 3.61503
0.01712 4.47174 5.19409 0.61958 0.77255 0.48576 0.95510
1052 2.09916 4.67887 3.30815 1.83351 3.91360 3.65849 3.98659 1.85499 2.88492 2.87602 3.81429 3.32679 4.09154 3.22094 3.28844 2.95074 3.05132 2.72832 5.34271 4.06706 1215 - -
2.68618 4.42225 2.77519 2.73123 3.46354 2.40513 3.72494 3.29354 2.67741 2.69355 4.24690 2.90347 2.73739 3.18146 2.89801 2.37887 2.77519 2.98518 4.58477 3.61503
0.01712 4.47174 5.19409 0.61958 0.77255 0.48576 0.95510
1053 2.48287 4.42641 3.76594 3.26783 3.70621 1.66377 4.15672 2.96887 3.18515 2.54002 1.92543 3.57967 4.10430 3.50681 3.49162 2.93227 3.00442 2.74478 5.19808 3.97509 1216 - -
2.68618 4.42225 2.77519 2.73123 3.46354 2.40513 3.72494 3.29354 2.67741 2.69355 4.24690 2.90347 2.73739 3.18146 2.89801 2.37887 2.77519 2.98518 4.58477 3.61503
0.01712 4.47174 5.19409 0.61958 0.77255 0.48576 0.95510
1054 2.69596 4.74668 3.20079 2.13639 3.94632 3.56220 3.77352 2.70879 2.60944 2.87647 3.21964 3.13676 3.94888 2.15481 2.75102 2.77630 2.92669 2.58142 5.27600 3.97631 1217 - -
2.68618 4.42225 2.77519 2.73123 3.46354 2.40513 3.72494 3.29354 2.67741 2.69355 4.24690 2.90347 2.73739 3.18146 2.89801 2.37887 2.77519 2.98518 4.58477 3.61503
0.01712 4.47174 5.19409 0.61958 0.77255 0.48576 0.95510
1055 2.83795 5.19638 2.95267 1.89659 4.56645 3.52874 3.77364 4.01470 2.46087 3.52909 4.31829 3.04412 3.97150 2.89858 2.16890 1.61174 3.08001 3.62095 5.67834 4.31943 1218 - -
2.68618 4.42225 2.77519 2.73123 3.46354 2.40513 3.72494 3.29354 2.67741 2.69355 4.24690 2.90347 2.73739 3.18146 2.89801 2.37887 2.77519 2.98518 4.58477 3.61503
0.01712 4.47174 5.19409 0.61958 0.77255 0.48576 0.95510

1056 2.64913 5.29057 1.69889 2.27266 4.62390 3.18642 3.71450 4.11018 2.18781 3.58897 4.34088 2.55059 3.90456 2.67392 2.65667 2.73100 3.01599 3.67616 5.72302 4.30404 1219 - -
2.68618 4.42225 2.77519 2.73123 3.46354 2.40513 3.72494 3.29354 2.67741 2.69355 4.24690 2.90347 2.73739 3.18146 2.89801 2.37887 2.77519 2.98518 4.58477 3.61503
0.40024 4.47174 1.14441 0.61958 0.77255 0.48576 0.95510
1057 3.13505 4.60407 4.17796 3.73031 2.48623 4.05713 3.80847 2.26685 3.55058 2.37899 3.57453 3.87750 4.42793 3.80608 3.74274 3.40543 3.38121 2.67485 4.15687 1.20026 1220 - -
2.68618 4.42225 2.77519 2.73123 3.46354 2.40513 3.72494 3.29354 2.67741 2.69355 4.24690 2.90347 2.73739 3.18146 2.89801 2.37887 2.77519 2.98518 4.58477 3.61503
0.02502 4.09652 4.81887 0.61958 0.77255 0.90459 0.51870
1058 2.59595 4.67050 3.07256 2.62714 4.15784 3.34034 3.77658 3.53944 2.03398 3.17228 4.01147 3.07720 3.85026 2.95415 2.86845 2.31133 1.79432 3.18703 5.40903 4.12070 1221 - -
2.68618 4.42225 2.77519 2.73123 3.46354 2.40513 3.72494 3.29354 2.67741 2.69355 4.24690 2.90347 2.73739 3.18146 2.89801 2.37887 2.77519 2.98518 4.58477 3.61503
0.02502 4.09652 4.81887 0.61958 0.77255 0.81826 0.58196
1059 2.66629 4.79524 3.06536 2.59100 4.22306 3.40807 3.73928 3.60651 1.81766 3.21334 4.04788 3.06309 3.27660 2.90000 2.78028 2.71971 1.85955 3.26052 5.43308 4.13777 1222 - -
2.68618 4.42225 2.77519 2.73123 3.46354 2.40513 3.72494 3.29354 2.67741 2.69355 4.24690 2.90347 2.73739 3.18146 2.89801 2.37887 2.77519 2.98518 4.58477 3.61503
0.02384 4.14394 4.86628 0.61958 0.77255 0.86741 0.54479
1060 2.94663 5.31721 0.97962 2.26922 4.77062 3.26199 3.93002 4.31703 2.99005 3.88363 4.76214 2.83292 3.92355 3.12084 3.56966 2.49569 3.28948 3.87440 6.01541 4.53400 1223 - -
2.68618 4.42225 2.77519 2.73123 3.46354 2.40513 3.72494 3.29354 2.67741 2.69355 4.24690 2.90347 2.73739 3.18146 2.89801 2.37887 2.77519 2.98518 4.58477 3.61503
0.02384 4.14394 4.86628 0.61958 0.77255 0.86741 0.54479
1061 2.47766 4.56803 3.96784 3.58082 2.98436 3.69086 4.01830 3.13931 3.32199 2.70914 3.82380 3.76694 4.22940 3.72633 3.52428 3.16003 3.26632 2.96102 1.12049 3.03897 1224 - -
2.68618 4.42225 2.77519 2.73123 3.46354 2.40513 3.72494 3.29354 2.67741 2.69355 4.24690 2.90347 2.73739 3.18146 2.89801 2.37887 2.77519 2.98518 4.58477 3.61503
0.02384 4.14394 4.86628 0.61958 0.77255 0.86741 0.54479
1062 2.55787 4.53999 4.19299 3.83733 1.07955 3.85426 4.09260 2.74743 3.75645 2.28875 3.54246 3.94897 4.36107 3.96737 3.93276 3.28192 3.31710 2.65744 4.49055 2.91745 1225 - -
2.68618 4.42225 2.77519 2.73123 3.46354 2.40513 3.72494 3.29354 2.67741 2.69355 4.24690 2.90347 2.73739 3.18146 2.89801 2.37887 2.77519 2.98518 4.58477 3.61503
0.02384 4.14394 4.86628 0.61958 0.77255 0.72150 0.66558
1063 2.36844 4.37701 3.24374 3.19480 1.73402 3.72911 3.83293 2.85142 3.15662 2.53794 3.48989 3.51808 4.11643 3.41654 3.45486 3.01154 2.99752 2.64941 4.52347 2.40051 1226 - -
2.68618 4.42225 2.77519 2.73123 3.46354 2.40513 3.72494 3.29354 2.67741 2.69355 4.24690 2.90347 2.73739 3.18146 2.89801 2.37887 2.77519 2.98518 4.58477 3.61503
0.02204 4.22153 4.94388 0.61958 0.77255 0.79915 0.59731
1064 0.60842 4.40663 3.85705 3.68225 4.43954 3.21877 4.65704 3.62061 3.70874 3.48458 4.48877 3.73078 3.99231 4.01916 3.92399 2.75963 3.06205 3.21164 5.82967 4.67539 1227 - -
2.68618 4.42225 2.77519 2.73123 3.46354 2.40513 3.72494 3.29354 2.67741 2.69355 4.24690 2.90347 2.73739 3.18146 2.89801 2.37887 2.77519 2.98518 4.58477 3.61503
0.02204 4.22153 4.94388 0.61958 0.77255 0.79915 0.59731
1065 2.73578 5.00670 2.93211 2.46908 4.29428 3.46124 2.28507 3.75141 2.37823 3.30204 4.11274 2.98640 3.41364 1.92059 2.78037 2.73863 2.74927 3.39706 5.47363 4.12796 1228 - -
2.68618 4.42225 2.77519 2.73123 3.46354 2.40513 3.72494 3.29354 2.67741 2.69355 4.24690 2.90347 2.73739 3.18146 2.89801 2.37887 2.77519 2.98518 4.58477 3.61503
0.02204 4.22153 4.94388 0.61958 0.77255 0.79915 0.59731
1066 3.19487 4.53918 4.84046 4.35385 3.54585 4.46196 5.00957 1.92807 4.19986 1.67683 3.39151 4.56056 4.79641 4.46765 4.37921 3.85884 3.46635 0.95028 5.50273 4.28895 1229 - -
2.68618 4.42225 2.77519 2.73123 3.46354 2.40513 3.72494 3.29354 2.67741 2.69355 4.24690 2.90347 2.73739 3.18146 2.89801 2.37887 2.77519 2.98518 4.58477 3.61503
0.02204 4.22153 4.94388 0.61958 0.77255 0.79915 0.59731
1067 2.74641 4.95168 3.00187 2.56388 4.38059 3.44375 3.74700 3.80606 2.39464 3.36615 4.18738 1.63609 3.91554 2.89456 2.20463 2.76983 2.56321 3.43592 5.53455 4.21907 1230 - -
2.68618 4.42225 2.77519 2.73123 3.46354 2.40513 3.72494 3.29354 2.67741 2.69355 4.24690 2.90347 2.73739 3.18146 2.89801 2.37887 2.77519 2.98518 4.58477 3.61503

0.02204 4.22153 4.94388 0.61958 0.77255 0.79915 0.59731

1068 3.44665 5.23182 3.97719 3.43218 4.67816 3.84777 4.14227 4.25755 2.46410 3.70990 4.71303 3.75859 4.34438 3.35590 0.55538 3.52174 3.69575 3.96918 5.62543 4.54339 1231 - -

2.68618 4.42225 2.77519 2.73123 3.46354 2.40513 3.72494 3.29354 2.67741 2.69355 4.24690 2.90347 2.73739 3.18146 2.89801 2.37887 2.77519 2.98518 4.58477 3.61503

0.02204 4.22153 4.94388 0.61958 0.77255 0.79915 0.59731

1069 3.27198 5.35524 3.76221 3.00592 4.87598 3.84861 3.71438 4.19098 1.41298 3.58388 4.46474 3.37271 4.19608 2.84496 1.14430 3.25746 3.41159 3.87675 5.57949 4.45778 1232 - -

2.68618 4.42225 2.77519 2.73123 3.46354 2.40513 3.72494 3.29354 2.67741 2.69355 4.24690 2.90347 2.73739 3.18146 2.89801 2.37887 2.77519 2.98518 4.58477 3.61503

0.02204 4.22153 4.94388 0.61958 0.77255 0.79915 0.59731

1070 2.23704 4.77728 3.03831 2.54242 4.06435 3.44486 2.78545 3.47692 2.50128 3.09330 3.92446 3.03986 3.87640 2.55093 2.92141 2.70621 1.98730 3.15811 5.34098 4.02535 1233 - -

2.68618 4.42225 2.77519 2.73123 3.46354 2.40513 3.72494 3.29354 2.67741 2.69355 4.24690 2.90347 2.73739 3.18146 2.89801 2.37887 2.77519 2.98518 4.58477 3.61503

0.02204 4.22153 4.94388 0.61958 0.77255 0.79915 0.59731

1071 2.10434 5.03003 1.58768 2.40145 4.63616 2.05177 3.89419 4.09790 2.83269 3.66593 4.48209 2.92979 3.88821 3.05878 3.35448 2.75360 3.08881 3.64623 5.85434 4.45134 1234 - -

2.68618 4.42225 2.77519 2.73123 3.46354 2.40513 3.72494 3.29354 2.67741 2.69355 4.24690 2.90347 2.73739 3.18146 2.89801 2.37887 2.77519 2.98518 4.58477 3.61503

0.09643 4.22153 2.56067 0.61958 0.77255 0.79915 0.59731

1072 2.85303 5.30663 2.52880 1.88281 4.62194 3.37526 3.71337 4.09886 2.48869 3.59477 4.40110 2.23252 3.88474 1.77449 2.94281 2.79064 3.10176 3.69629 5.73666 4.32866 1235 - -

2.68618 4.42225 2.77519 2.73123 3.46354 2.40513 3.72494 3.29354 2.67741 2.69355 4.24690 2.90347 2.73739 3.18146 2.89801 2.37887 2.77519 2.98518 4.58477 3.61503

0.02373 4.14882 4.87117 0.61958 0.77255 0.72576 0.66156

1073 2.51248 5.02321 2.51993 2.37328 4.36316 2.68620 3.66137 3.81658 2.31544 3.35512 4.13504 2.91042 3.83041 2.78583 2.70342 1.90388 2.90817 3.42577 5.53635 4.15631 1236 - -

2.68618 4.42225 2.77519 2.73123 3.46354 2.40513 3.72494 3.29354 2.67741 2.69355 4.24690 2.90347 2.73739 3.18146 2.89801 2.37887 2.77519 2.98518 4.58477 3.61503

0.15003 4.22153 2.08234 0.61958 0.77255 0.79915 0.59731

1074 2.35806 4.28012 3.44890 3.18602 4.29719 2.32890 4.26604 3.63140 3.22735 3.38442 4.25433 3.37750 3.79799 3.54885 3.52727 2.53809 1.11563 3.16095 5.64590 4.43821 1237 - -

2.68618 4.42225 2.77519 2.73123 3.46354 2.40513 3.72494 3.29354 2.67741 2.69355 4.24690 2.90347 2.73739 3.18146 2.89801 2.37887 2.77519 2.98518 4.58477 3.61503

0.02502 4.09652 4.81887 0.61958 0.77255 0.90459 0.51870

1075 3.58653 4.98405 4.17677 3.95328 2.33811 4.09723 3.74683 3.56642 3.80478 2.96102 4.21898 4.01036 4.56820 4.05581 3.94014 3.69866 3.87563 3.44823 3.98866 0.66145 1238 - -

2.68618 4.42225 2.77519 2.73123 3.46354 2.40513 3.72494 3.29354 2.67741 2.69355 4.24690 2.90347 2.73739 3.18146 2.89801 2.37887 2.77519 2.98518 4.58477 3.61503

0.02502 4.09652 4.81887 0.61958 0.77255 0.90459 0.51870

1076 2.40710 5.03825 2.95774 2.50348 4.40045 3.46924 3.66003 3.80506 1.87536 3.33666 4.16387 3.00152 3.90272 1.79340 2.57064 2.78910 3.02059 3.45379 5.49328 4.19116 1239 - -

2.68618 4.42225 2.77519 2.73123 3.46354 2.40513 3.72494 3.29354 2.67741 2.69355 4.24690 2.90347 2.73739 3.18146 2.89801 2.37887 2.77519 2.98518 4.58477 3.61503

0.02502 4.09652 4.81887 0.61958 0.77255 0.90459 0.51870

1077 1.50564 4.30339 3.41148 3.12672 4.27916 3.08008 4.22576 3.60446 3.17371 3.34006 4.22474 3.34909 1.59827 3.49344 3.49291 2.54182 2.83322 3.15106 5.63452 4.41337 1240 - -

2.68618 4.42225 2.77519 2.73123 3.46354 2.40513 3.72494 3.29354 2.67741 2.69355 4.24690 2.90347 2.73739 3.18146 2.89801 2.37887 2.77519 2.98518 4.58477 3.61503

0.02502 4.09652 4.81887 0.61958 0.77255 0.90459 0.51870

1078 2.54951 4.39302 3.64478 3.41472 4.18432 3.22305 4.41266 3.33963 3.36216 3.19498 4.23571 3.58273 3.94666 3.74691 3.60593 2.75390 0.84170 3.00969 5.60184 4.40281 1241 - -

2.68618 4.42225 2.77519 2.73123 3.46354 2.40513 3.72494 3.29354 2.67741 2.69355 4.24690 2.90347 2.73739 3.18146 2.89801 2.37887 2.77519 2.98518 4.58477 3.61503

0.02502 4.09652 4.81887 0.61958 0.77255 0.90459 0.51870

1079 2.54951 4.39302 3.64478 3.41472 4.18432 3.22305 4.41266 3.33963 3.36216 3.19498 4.23571 3.58273 3.94666 3.74691 3.60593 2.75390 0.84170 3.00969 5.60184 4.40281 1242 - -

2.68618 4.42225 2.77519 2.73123 3.46354 2.40513 3.72494 3.29354 2.67741 2.69355 4.24690 2.90347 2.73739 3.18146 2.89801 2.37887 2.77519 2.98518 4.58477 3.61503
0.02502 4.09652 4.81887 0.61958 0.77255 0.90459 0.51870
1080 3.05498 4.71469 3.85441 3.31580 3.06893 3.78433 3.83328 3.31387 2.74082 2.85085 3.88513 3.60834 4.21177 3.36451 2.28583 3.22289 3.29663 3.12045 1.30544 3.07621 1243 - -
2.68618 4.42225 2.77519 2.73123 3.46354 2.40513 3.72494 3.29354 2.67741 2.69355 4.24690 2.90347 2.73739 3.18146 2.89801 2.37887 2.77519 2.98518 4.58477 3.61503
0.02502 4.09652 4.81887 0.61958 0.77255 0.90459 0.51870
1081 3.07974 5.05966 3.07175 2.83627 4.25706 3.52758 3.99436 3.92853 2.64276 3.40778 4.43534 3.29573 4.08807 0.88232 2.91989 3.12079 3.37936 3.64597 5.49162 4.20297 1244 - -
2.68618 4.42225 2.77519 2.73123 3.46354 2.40513 3.72494 3.29354 2.67741 2.69355 4.24690 2.90347 2.73739 3.18146 2.89801 2.37887 2.77519 2.98518 4.58477 3.61503
0.02502 4.09652 4.81887 0.61958 0.77255 0.90459 0.51870
1082 3.20918 4.57349 4.64253 4.27155 3.53493 4.29975 4.96344 0.83672 4.10413 2.08132 3.44180 4.48639 4.71477 4.43298 4.27591 3.82186 3.50654 1.87870 5.46804 4.22192 1245 - -
2.68618 4.42225 2.77519 2.73123 3.46354 2.40513 3.72494 3.29354 2.67741 2.69355 4.24690 2.90347 2.73739 3.18146 2.89801 2.37887 2.77519 2.98518 4.58477 3.61503
0.02502 4.09652 4.81887 0.61958 0.77255 0.90459 0.51870
1083 3.11963 5.22561 3.50612 2.85577 4.69231 3.71424 3.66649 4.04315 1.05819 3.47730 4.35378 3.25398 4.08994 2.80246 1.85520 3.11306 3.28668 3.72408 5.50704 4.34532 1246 - -
2.68618 4.42225 2.77519 2.73123 3.46354 2.40513 3.72494 3.29354 2.67741 2.69355 4.24690 2.90347 2.73739 3.18146 2.89801 2.37887 2.77519 2.98518 4.58477 3.61503
0.02502 4.09652 4.81887 0.61958 0.77255 0.90459 0.51870
1084 3.55768 4.89539 4.54704 4.30922 0.70572 4.18416 3.99699 3.05980 4.21210 2.38042 3.75988 4.27904 4.64157 4.31940 4.27613 3.81243 3.86089 3.06610 4.18921 2.53208 1247 - -
2.68618 4.42225 2.77519 2.73123 3.46354 2.40513 3.72494 3.29354 2.67741 2.69355 4.24690 2.90347 2.73739 3.18146 2.89801 2.37887 2.77519 2.98518 4.58477 3.61503
0.02502 4.09652 4.81887 0.61958 0.77255 0.90459 0.51870
1085 2.82650 4.98653 2.88113 2.56907 4.35722 3.41307 3.77556 3.89155 2.40329 3.43883 4.29801 1.39010 3.93405 2.94898 2.26719 2.84479 3.10647 3.52407 5.52306 4.19462 1248 - -
2.68618 4.42225 2.77519 2.73123 3.46354 2.40513 3.72494 3.29354 2.67741 2.69355 4.24690 2.90347 2.73739 3.18146 2.89801 2.37887 2.77519 2.98518 4.58477 3.61503
0.02502 4.09652 4.81887 0.61958 0.77255 0.90459 0.51870
1086 3.41588 4.81501 4.54845 4.19505 3.23618 4.26421 4.78108 2.45815 3.96075 0.64134 3.19931 4.44883 4.66468 4.27484 4.09780 3.87006 3.70194 2.55258 5.19071 3.96384 1249 - -
2.68618 4.42225 2.77519 2.73123 3.46354 2.40513 3.72494 3.29354 2.67741 2.69355 4.24690 2.90347 2.73739 3.18146 2.89801 2.37887 2.77519 2.98518 4.58477 3.61503
0.02502 4.09652 4.81887 0.61958 0.77255 0.90459 0.51870
1087 2.88956 5.43454 1.50056 1.92037 4.73473 3.30603 3.73480 4.23233 2.05678 3.71929 4.53145 2.76337 3.86451 2.87911 3.15565 2.79765 3.15317 3.80806 5.86817 4.40499 1250 - -
2.68618 4.42225 2.77519 2.73123 3.46354 2.40513 3.72494 3.29354 2.67741 2.69355 4.24690 2.90347 2.73739 3.18146 2.89801 2.37887 2.77519 2.98518 4.58477 3.61503
0.02502 4.09652 4.81887 0.61958 0.77255 0.90459 0.51870
1088 1.96507 4.49410 3.21697 2.76772 4.16659 3.22618 3.88631 3.56844 2.67288 3.21002 4.04380 3.15256 3.80896 3.08270 2.49757 1.60265 2.83325 3.16815 5.45095 4.17785 1251 - -
2.68618 4.42225 2.77519 2.73123 3.46354 2.40513 3.72494 3.29354 2.67741 2.69355 4.24690 2.90347 2.73739 3.18146 2.89801 2.37887 2.77519 2.98518 4.58477 3.61503
0.02502 4.09652 4.81887 0.61958 0.77255 0.90459 0.51870
1089 2.98877 4.47595 4.43109 4.10674 3.72428 3.95068 4.87313 2.06701 3.97813 2.37578 3.66058 4.25914 4.50281 4.32432 4.16830 3.47278 3.34743 0.78272 5.54475 4.28797 1252 - -
2.68618 4.42225 2.77519 2.73123 3.46354 2.40513 3.72494 3.29354 2.67741 2.69355 4.24690 2.90347 2.73739 3.18146 2.89801 2.37887 2.77519 2.98518 4.58477 3.61503
0.02502 4.09652 4.81887 0.61958 0.77255 0.90459 0.51870
1090 2.60253 4.28569 3.74421 3.23169 3.56757 3.55149 4.10809 2.02833 3.15365 2.50658 3.51842 3.53913 4.04451 3.46589 3.46108 1.77900 2.90974 2.03354 5.10809 3.87275 1253 - -
2.68618 4.42225 2.77519 2.73123 3.46354 2.40513 3.72494 3.29354 2.67741 2.69355 4.24690 2.90347 2.73739 3.18146 2.89801 2.37887 2.77519 2.98518 4.58477 3.61503
0.02502 4.09652 4.81887 0.61958 0.77255 0.90459 0.51870

1091 2.24952 4.71831 2.93034 2.56416 4.21145 3.31010 3.79392 3.62198 2.57272 3.23120 4.07702 3.03318 1.97861 2.27483 2.96888 2.67320 2.91960 3.25735 5.47198 4.15987 1254 - -
2.68618 4.42225 2.77519 2.73123 3.46354 2.40513 3.72494 3.29354 2.67741 2.69355 4.24690 2.90347 2.73739 3.18146 2.89801 2.37887 2.77519 2.98518 4.58477 3.61503
0.02502 4.09652 4.81887 0.61958 0.77255 0.90459 0.51870
1092 2.73679 5.06899 2.18267 2.30130 4.47393 3.29195 3.75115 3.93001 2.63505 3.49624 4.31246 1.73662 3.83864 2.90550 3.14133 2.71582 2.29603 3.53010 5.68642 4.28062 1255 - -
2.68618 4.42225 2.77519 2.73123 3.46354 2.40513 3.72494 3.29354 2.67741 2.69355 4.24690 2.90347 2.73739 3.18146 2.89801 2.37887 2.77519 2.98518 4.58477 3.61503
0.02502 4.09652 4.81887 0.61958 0.77255 0.90459 0.51870
1093 2.17400 4.56539 3.18640 2.72076 4.19054 3.26771 3.83833 3.59462 2.57563 3.22019 4.05240 3.12768 3.82370 3.02202 2.23762 1.63896 2.85808 3.20316 5.44680 4.16842 1256 - -
2.68618 4.42225 2.77519 2.73123 3.46354 2.40513 3.72494 3.29354 2.67741 2.69355 4.24690 2.90347 2.73739 3.18146 2.89801 2.37887 2.77519 2.98518 4.58477 3.61503
0.02502 4.09652 4.81887 0.61958 0.77255 0.90459 0.51870
1094 2.69688 4.34900 3.89110 3.42501 3.62626 3.64027 4.29743 1.88279 3.30512 2.41715 3.54419 3.70070 4.15951 3.65106 3.58442 3.01586 1.53766 2.18531 5.26238 4.03125 1257 - -
2.68618 4.42225 2.77519 2.73123 3.46354 2.40513 3.72494 3.29354 2.67741 2.69355 4.24690 2.90347 2.73739 3.18146 2.89801 2.37887 2.77519 2.98518 4.58477 3.61503
0.02502 4.09652 4.81887 0.61958 0.77255 0.90459 0.51870
1095 3.58653 4.98405 4.17677 3.95328 2.33811 4.09723 3.74683 3.56642 3.80478 2.96102 4.21898 4.01036 4.56820 4.05581 3.94014 3.69866 3.87563 3.44823 3.98866 0.66145 1258 - -
2.68618 4.42225 2.77519 2.73123 3.46354 2.40513 3.72494 3.29354 2.67741 2.69355 4.24690 2.90347 2.73739 3.18146 2.89801 2.37887 2.77519 2.98518 4.58477 3.61503
0.02502 4.09652 4.81887 0.61958 0.77255 0.90459 0.51870
1096 2.75078 4.84192 3.13626 2.67288 4.29953 3.44793 3.75285 3.62381 1.50908 3.25078 4.11919 3.12020 3.92749 2.91586 2.61466 2.80642 1.99469 3.29428 5.45946 4.19442 1259 - -
2.68618 4.42225 2.77519 2.73123 3.46354 2.40513 3.72494 3.29354 2.67741 2.69355 4.24690 2.90347 2.73739 3.18146 2.89801 2.37887 2.77519 2.98518 4.58477 3.61503
0.02502 4.09652 4.81887 0.61958 0.77255 0.68363 0.70275
1097 2.79339 4.32634 3.97470 2.77607 1.98822 3.85403 4.09664 2.52919 3.32803 1.66910 3.23764 3.72474 4.20850 3.58578 3.57914 3.13907 3.02573 2.27000 4.82048 3.55227 1260 - -
2.68618 4.42225 2.77519 2.73123 3.46354 2.40513 3.72494 3.29354 2.67741 2.69355 4.24690 2.90347 2.73739 3.18146 2.89801 2.37887 2.77519 2.98518 4.58477 3.61503
0.02204 4.22153 4.94388 0.61958 0.77255 0.79915 0.59731
1098 2.96886 5.10029 3.34642 2.76776 4.39279 3.64683 2.82146 3.89124 2.12805 3.39170 4.24457 3.20541 4.04792 2.86492 1.24769 2.69991 3.17572 3.56276 5.44927 4.18013 1261 - -
2.68618 4.42225 2.77519 2.73123 3.46354 2.40513 3.72494 3.29354 2.67741 2.69355 4.24690 2.90347 2.73739 3.18146 2.89801 2.37887 2.77519 2.98518 4.58477 3.61503
0.02204 4.22153 4.94388 0.61958 0.77255 0.79915 0.59731
1099 2.59875 4.34471 3.94593 2.75611 3.56121 3.89958 4.25591 1.87427 3.34607 2.43532 3.47479 3.75809 4.27721 3.64753 3.64099 3.19707 3.05512 1.35934 5.15210 3.93140 1262 - -
2.68618 4.42225 2.77519 2.73123 3.46354 2.40513 3.72494 3.29354 2.67741 2.69355 4.24690 2.90347 2.73739 3.18146 2.89801 2.37887 2.77519 2.98518 4.58477 3.61503
0.02204 4.22153 4.94388 0.61958 0.77255 0.79915 0.59731
1100 1.60720 4.83742 2.98520 2.26255 4.20588 3.42056 3.74082 3.61344 2.49173 3.21442 4.03799 3.03155 3.88152 2.89998 2.48284 2.53571 2.93780 3.26920 5.44172 4.12333 1263 - -
2.68618 4.42225 2.77519 2.73123 3.46354 2.40513 3.72494 3.29354 2.67741 2.69355 4.24690 2.90347 2.73739 3.18146 2.89801 2.37887 2.77519 2.98518 4.58477 3.61503
0.02204 4.22153 4.94388 0.61958 0.77255 0.79915 0.59731
1101 3.23944 4.65932 4.44676 3.96628 2.74266 4.24727 4.13835 2.65210 3.75833 0.97689 3.29086 4.13245 4.56609 3.99378 3.91617 3.59969 3.47245 2.67265 4.49091 2.16228 1264 - -
2.68618 4.42225 2.77519 2.73123 3.46354 2.40513 3.72494 3.29354 2.67741 2.69355 4.24690 2.90347 2.73739 3.18146 2.89801 2.37887 2.77519 2.98518 4.58477 3.61503
0.02204 4.22153 4.94388 0.61958 0.77255 0.79915 0.59731
1102 0.60842 4.40663 3.85705 3.68225 4.43954 3.21877 4.65704 3.62061 3.70874 3.48458 4.48877 3.73078 3.99231 4.01916 3.92399 2.75963 3.06205 3.21164 5.82967 4.67539 1265 - -
2.68618 4.42225 2.77519 2.73123 3.46354 2.40513 3.72494 3.29354 2.67741 2.69355 4.24690 2.90347 2.73739 3.18146 2.89801 2.37887 2.77519 2.98518 4.58477 3.61503

0.02204 4.22153 4.94388 0.61958 0.77255 0.79915 0.59731

1103 1.21222 4.26058 3.66637 3.38464 4.46092 3.04826 4.42027 3.83975 3.42635 3.56833 4.40105 3.47807 3.81559 3.70555 3.71848 1.37842 2.82749 3.28065 5.81595 4.61271 1266 - -

2.68618 4.42225 2.77519 2.73123 3.46354 2.40513 3.72494 3.29354 2.67741 2.69355 4.24690 2.90347 2.73739 3.18146 2.89801 2.37887 2.77519 2.98518 4.58477 3.61503

0.02204 4.22153 4.94388 0.61958 0.77255 0.79915 0.59731

1104 1.53313 4.33676 3.54498 3.09342 3.26083 3.25442 4.09403 3.29972 3.07953 3.02979 3.91238 3.37471 3.87737 3.38327 3.42901 1.61693 2.84192 2.95358 5.34085 4.07640 1267 - -

2.68618 4.42225 2.77519 2.73123 3.46354 2.40513 3.72494 3.29354 2.67741 2.69355 4.24690 2.90347 2.73739 3.18146 2.89801 2.37887 2.77519 2.98518 4.58477 3.61503

0.02204 4.22153 4.94388 0.61958 0.77255 0.79915 0.59731

1105 1.85444 4.83592 3.06339 2.54588 4.20900 3.43149 3.71393 3.62450 2.43076 3.21008 4.02533 3.03803 3.87269 2.51931 2.31941 2.24907 2.91506 3.27418 5.42439 4.10858 1268 - -

2.68618 4.42225 2.77519 2.73123 3.46354 2.40513 3.72494 3.29354 2.67741 2.69355 4.24690 2.90347 2.73739 3.18146 2.89801 2.37887 2.77519 2.98518 4.58477 3.61503

0.02204 4.22153 4.94388 0.61958 0.77255 0.79915 0.59731

1106 2.66318 4.94062 2.69672 2.29063 4.21117 3.42308 3.66791 3.63724 2.46476 3.21963 3.25779 1.95814 3.84489 2.80821 2.93778 2.67140 2.42698 3.29018 5.43968 4.08187 1269 - -

2.68618 4.42225 2.77519 2.73123 3.46354 2.40513 3.72494 3.29354 2.67741 2.69355 4.24690 2.90347 2.73739 3.18146 2.89801 2.37887 2.77519 2.98518 4.58477 3.61503

0.02204 4.22153 4.94388 0.61958 0.77255 0.79915 0.59731

1107 1.47468 4.43868 3.45962 2.73941 3.71552 3.56167 4.01210 2.21901 2.96333 2.67908 3.64502 3.39339 4.03381 3.30484 3.31204 2.88554 2.95282 2.52586 5.19148 3.94619 1270 - -

2.68618 4.42225 2.77519 2.73123 3.46354 2.40513 3.72494 3.29354 2.67741 2.69355 4.24690 2.90347 2.73739 3.18146 2.89801 2.37887 2.77519 2.98518 4.58477 3.61503

0.02204 4.22153 4.94388 0.61958 0.77255 0.79915 0.59731

1108 2.39344 5.21146 2.22633 1.67910 4.53194 3.38880 3.67226 4.00597 2.46121 3.50800 4.28099 2.86197 3.84831 2.79210 2.42586 2.69603 2.98451 3.59215 5.66105 4.24806 1271 - -

2.68618 4.42225 2.77519 2.73123 3.46354 2.40513 3.72494 3.29354 2.67741 2.69355 4.24690 2.90347 2.73739 3.18146 2.89801 2.37887 2.77519 2.98518 4.58477 3.61503

0.02204 4.22153 4.94388 0.61958 0.77255 0.79915 0.59731

1109 3.33558 4.66031 4.96674 4.41424 2.78428 4.58587 4.86983 2.20905 4.28583 0.89594 3.01580 4.62695 4.79740 4.38033 4.39181 3.93667 3.56423 1.82688 5.17362 4.01263 1272 - -

2.68618 4.42225 2.77519 2.73123 3.46354 2.40513 3.72494 3.29354 2.67741 2.69355 4.24690 2.90347 2.73739 3.18146 2.89801 2.37887 2.77519 2.98518 4.58477 3.61503

0.02204 4.22153 4.94388 0.61958 0.77255 0.79915 0.59731

1110 2.56979 4.80107 3.21197 2.66106 4.01678 3.55434 3.72948 3.39689 2.39290 2.28214 3.87992 3.13506 3.95163 1.79084 2.52927 2.81873 2.97659 3.12526 5.29458 4.01466 1273 - -

2.68618 4.42225 2.77519 2.73123 3.46354 2.40513 3.72494 3.29354 2.67741 2.69355 4.24690 2.90347 2.73739 3.18146 2.89801 2.37887 2.77519 2.98518 4.58477 3.61503

0.02204 4.22153 4.94388 0.61958 0.77255 0.72960 0.65797

1111 2.63927 4.26136 3.72123 3.16896 3.38981 2.94865 3.95035 2.72110 2.80460 2.48046 3.39891 3.49693 4.04044 3.36301 3.31036 2.92439 2.88542 1.48843 3.96971 3.63964 1274 - -

2.68602 4.42232 2.77526 2.73130 3.46361 2.40495 3.72501 3.29361 2.67747 2.69351 4.24696 2.90353 2.73721 3.18153 2.89807 2.37893 2.77526 2.98509 4.58484 3.61510

0.14468 2.05718 4.97753 0.94847 0.48993 0.38717 1.13624

1112 2.42532 4.60176 3.32546 2.61599 3.75677 3.59755 3.19095 3.15224 2.70477 2.82764 3.69393 3.22647 3.98099 3.05409 2.11527 2.82412 2.92223 2.61151 3.34154 2.83358 1280 - -

2.68618 4.42225 2.77519 2.73123 3.46354 2.40513 3.72494 3.29354 2.67741 2.69355 4.24690 2.90347 2.73739 3.18146 2.89801 2.37887 2.77519 2.98518 4.58477 3.61503

0.01755 4.44728 5.16963 0.61958 0.77255 0.52515 0.89517

1113 2.68787 4.48677 3.44068 2.72015 2.35511 3.27342 2.49538 3.00543 2.73634 2.70403 3.58649 3.31622 4.01335 3.15686 2.98121 2.87019 2.91919 2.19371 5.05068 3.79850 1281 - -

2.68618 4.42225 2.77519 2.73123 3.46354 2.40513 3.72494 3.29354 2.67741 2.69355 4.24690 2.90347 2.73739 3.18146 2.89801 2.37887 2.77519 2.98518 4.58477 3.61503

0.01755 4.44728 5.16963 0.61958 0.77255 0.52515 0.89517

1114 2.72343 4.90391 2.93243 2.67178 4.66422 2.66303 4.03664 4.13439 2.90877 3.70201 4.50070 1.45115 2.05113 3.20129 3.37223 2.54710 3.08819 3.64897 5.88346 4.53034 1282 - -

2.68618 4.42225 2.77519 2.73123 3.46354 2.40513 3.72494 3.29354 2.67741 2.69355 4.24690 2.90347 2.73739 3.18146 2.89801 2.37887 2.77519 2.98518 4.58477 3.61503
0.01755 4.44728 5.16963 0.61958 0.77255 0.46189 0.99449
1115 3.16612 5.78378 1.67437 1.90582 5.08886 1.41155 3.99211 4.62150 3.00303 4.09254 4.92406 2.59752 4.07619 3.14961 3.61135 3.04622 3.45227 4.17038 6.24551 4.72343 1283 - -
2.68618 4.42225 2.77519 2.73123 3.46354 2.40513 3.72494 3.29354 2.67741 2.69355 4.24690 2.90347 2.73739 3.18146 2.89801 2.37887 2.77519 2.98518 4.58477 3.61503
0.01712 4.47174 5.19409 0.61958 0.77255 0.48576 0.95510
1116 2.72146 4.63449 3.35276 2.78216 3.57869 3.62081 3.83001 3.17454 2.54305 2.12129 3.71800 3.24642 4.00261 2.06750 2.61034 2.85131 2.94989 2.36821 5.17713 3.91022 1284 - -
2.68618 4.42225 2.77519 2.73123 3.46354 2.40513 3.72494 3.29354 2.67741 2.69355 4.24690 2.90347 2.73739 3.18146 2.89801 2.37887 2.77519 2.98518 4.58477 3.61503
0.11149 4.47174 2.36372 0.61958 0.77255 0.48576 0.95510
1117 2.38575 5.08141 1.63682 2.48111 4.71360 1.81917 3.94551 4.19120 2.85784 3.73577 4.53522 3.00525 3.94591 3.10065 3.37541 2.34327 3.12454 3.72140 5.91389 4.51074 1285 - -
2.68618 4.42225 2.77519 2.73123 3.46354 2.40513 3.72494 3.29354 2.67741 2.69355 4.24690 2.90347 2.73739 3.18146 2.89801 2.37887 2.77519 2.98518 4.58477 3.61503
0.01880 4.37906 5.10140 0.61958 0.77255 0.40858 1.09240
1118 2.86774 5.30719 1.49388 2.42436 4.68288 2.84499 3.81744 4.16208 2.61876 3.66500 4.44653 2.97616 3.96657 2.94493 2.02143 2.62909 3.12646 3.73852 5.81568 4.40356 1286 - -
2.68618 4.42225 2.77519 2.73123 3.46354 2.40513 3.72494 3.29354 2.67741 2.69355 4.24690 2.90347 2.73739 3.18146 2.89801 2.37887 2.77519 2.98518 4.58477 3.61503
0.01712 4.47174 5.19409 0.61958 0.77255 0.48576 0.95510
1119 2.69633 5.07381 2.83328 1.95993 4.36728 3.26961 3.20940 3.81855 2.34475 3.35677 4.12947 2.97610 3.88084 2.80014 2.92488 2.35158 2.16476 3.43669 3.77745 4.16067 1287 - -
2.68618 4.42225 2.77519 2.73123 3.46354 2.40513 3.72494 3.29354 2.67741 2.69355 4.24690 2.90347 2.73739 3.18146 2.89801 2.37887 2.77519 2.98518 4.58477 3.61503
0.01712 4.47174 5.19409 0.61958 0.77255 0.48576 0.95510
1120 1.77412 5.17701 3.42543 2.81792 4.60330 3.71095 3.80693 3.97809 1.91749 3.49032 4.32280 3.27007 4.10965 2.94188 1.48246 3.01310 3.21386 3.63363 5.61373 4.37053 1288 - -
2.68618 4.42225 2.77519 2.73123 3.46354 2.40513 3.72494 3.29354 2.67741 2.69355 4.24690 2.90347 2.73739 3.18146 2.89801 2.37887 2.77519 2.98518 4.58477 3.61503
0.07427 4.47174 2.81088 0.61958 0.77255 0.48576 0.95510
1121 2.86868 4.74200 3.54185 2.97901 3.74377 3.69324 3.89118 3.37110 2.63413 3.00089 3.90162 3.39149 1.83943 3.16671 2.31778 2.99897 3.11064 3.11470 2.16318 3.78640 1289 - -
2.68618 4.42225 2.77519 2.73123 3.46354 2.40513 3.72494 3.29354 2.67741 2.69355 4.24690 2.90347 2.73739 3.18146 2.89801 2.37887 2.77519 2.98518 4.58477 3.61503
0.01812 4.41559 5.13794 0.61958 0.77255 0.43502 1.04200
1122 2.83164 4.74100 3.37162 2.88581 3.71356 3.65801 2.22543 3.32177 2.76197 2.15873 3.86583 3.33033 1.87264 3.17912 3.11584 2.95360 3.08179 3.07577 5.14798 3.77726 1290 - -
2.68618 4.42225 2.77519 2.73123 3.46354 2.40513 3.72494 3.29354 2.67741 2.69355 4.24690 2.90347 2.73739 3.18146 2.89801 2.37887 2.77519 2.98518 4.58477 3.61503
0.01712 4.47174 5.19409 0.61958 0.77255 0.48576 0.95510
1123 3.20980 4.63354 4.66385 4.16121 1.06465 4.27207 4.36457 2.64205 4.00879 1.79584 3.37439 4.28163 4.62732 4.17763 4.14201 3.61811 2.78202 2.61331 4.73534 3.26671 1291 - -
2.68618 4.42225 2.77519 2.73123 3.46354 2.40513 3.72494 3.29354 2.67741 2.69355 4.24690 2.90347 2.73739 3.18146 2.89801 2.37887 2.77519 2.98518 4.58477 3.61503
0.01712 4.47174 5.19409 0.61958 0.77255 0.48576 0.95510
1124 3.09358 4.47093 4.76618 4.23742 3.75712 4.34348 4.88189 1.60989 4.13071 2.46954 3.61952 4.42763 4.71399 4.38381 4.32559 3.69716 1.80125 1.22114 5.50956 4.30517 1292 - -
2.68618 4.42225 2.77519 2.73123 3.46354 2.40513 3.72494 3.29354 2.67741 2.69355 4.24690 2.90347 2.73739 3.18146 2.89801 2.37887 2.77519 2.98518 4.58477 3.61503
0.01712 4.47174 5.19409 0.61958 0.77255 0.48576 0.95510
1125 2.12575 4.43089 4.00426 3.75885 4.57462 3.26999 4.71510 3.70824 3.73349 3.59649 4.51916 3.77249 4.04775 4.03944 3.96833 2.74959 0.70658 3.26160 5.99333 4.81504 1293 - -
2.68618 4.42225 2.77519 2.73123 3.46354 2.40513 3.72494 3.29354 2.67741 2.69355 4.24690 2.90347 2.73739 3.18146 2.89801 2.37887 2.77519 2.98518 4.58477 3.61503
0.01712 4.47174 5.19409 0.61958 0.77255 0.48576 0.95510

1126 2.35867 4.81578 3.13382 2.83235 4.38882 1.63830 1.65043 3.90705 2.92044 3.51990 4.35811 3.26745 4.02303 3.28029 3.31609 2.83618 3.10803 3.49580 5.68753 4.35392 1294 - -
2.68618 4.42225 2.77519 2.73123 3.46354 2.40513 3.72494 3.29354 2.67741 2.69355 4.24690 2.90347 2.73739 3.18146 2.89801 2.37887 2.77519 2.98518 4.58477 3.61503
0.01712 4.47174 5.19409 0.61958 0.77255 0.48576 0.95510
1127 2.67418 4.62008 4.82175 4.28285 1.54165 4.36939 4.56763 2.53435 4.13654 1.17288 3.20356 4.41782 4.67530 4.26082 4.23734 3.70784 3.47393 2.56077 4.93594 3.61266 1295 - -
2.68618 4.42225 2.77519 2.73123 3.46354 2.40513 3.72494 3.29354 2.67741 2.69355 4.24690 2.90347 2.73739 3.18146 2.89801 2.37887 2.77519 2.98518 4.58477 3.61503
0.01712 4.47174 5.19409 0.61958 0.77255 0.48576 0.95510
1128 3.07543 4.40563 4.89823 4.34011 2.90815 4.36916 4.79401 1.30673 4.20894 2.33288 3.47513 4.46566 4.69084 4.40187 4.32698 3.70674 2.51533 1.34988 5.30633 4.11629 1296 - -
2.68618 4.42225 2.77519 2.73123 3.46354 2.40513 3.72494 3.29354 2.67741 2.69355 4.24690 2.90347 2.73739 3.18146 2.89801 2.37887 2.77519 2.98518 4.58477 3.61503
0.01712 4.47174 5.19409 0.61958 0.77255 0.48576 0.95510
1129 3.57401 5.24861 4.36381 4.35348 5.40728 0.20721 5.39221 5.19546 4.61166 4.75230 5.76109 4.53127 4.65161 4.88936 4.74225 3.76858 4.09816 4.60781 6.30299 5.54286 1297 - -
2.68618 4.42225 2.77519 2.73123 3.46354 2.40513 3.72494 3.29354 2.67741 2.69355 4.24690 2.90347 2.73739 3.18146 2.89801 2.37887 2.77519 2.98518 4.58477 3.61503
0.01712 4.47174 5.19409 0.61958 0.77255 0.48576 0.95510
1130 2.78369 2.11321 2.76205 2.59321 4.29963 3.56707 3.75037 3.71183 1.82578 3.28596 4.09873 3.10018 3.96667 2.89180 2.39021 2.80448 3.01292 3.37492 5.48929 4.16998 1298 - -
2.68618 4.42225 2.77519 2.73123 3.46354 2.40513 3.72494 3.29354 2.67741 2.69355 4.24690 2.90347 2.73739 3.18146 2.89801 2.37887 2.77519 2.98518 4.58477 3.61503
0.01712 4.47174 5.19409 0.61958 0.77255 0.48576 0.95510
1131 2.89236 5.41024 1.65432 2.38430 4.74076 2.91864 3.79069 4.22962 1.77192 3.70660 4.47883 2.94620 3.96351 2.37026 3.07728 2.82992 3.13744 3.79833 5.84122 4.41308 1299 - -
2.68623 4.41644 2.77529 2.73151 3.46366 2.40440 3.72487 3.29405 2.67752 2.69406 4.24741 2.90357 2.73661 3.18198 2.89818 2.37896 2.77571 2.98570 4.58528 3.61423
0.40024 1.12611 5.19409 1.77828 0.18504 0.48576 0.95510
1132 2.71644 5.09020 2.23252 2.43825 4.37977 3.48587 3.38350 3.43145 2.47053 3.37494 3.68127 1.82429 3.89259 2.82105 2.95410 2.48594 2.95047 3.45380 5.55728 4.17685 1308 - -
2.68618 4.42225 2.77519 2.73123 3.46354 2.40513 3.72494 3.29354 2.67741 2.69355 4.24690 2.90347 2.73739 3.18146 2.89801 2.37887 2.77519 2.98518 4.58477 3.61503
0.01712 4.47174 5.19409 0.61958 0.77255 0.48576 0.95510
1133 2.00381 4.43713 3.68703 3.19732 3.62155 3.57328 4.08053 3.06808 3.13963 2.80667 3.71997 3.52290 4.07963 3.45323 3.45984 2.90526 1.94220 2.81588 5.09817 1.78564 1309 - -
2.68618 4.42225 2.77519 2.73123 3.46354 2.40513 3.72494 3.29354 2.67741 2.69355 4.24690 2.90347 2.73739 3.18146 2.89801 2.37887 2.77519 2.98518 4.58477 3.61503
0.01712 4.47174 5.19409 0.61958 0.77255 0.48576 0.95510
1134 2.32810 4.55330 2.89827 2.84703 3.77624 3.57947 3.90313 1.76809 2.84509 2.83261 3.70883 3.29072 2.80688 3.16182 3.24455 2.65204 2.75904 2.85782 5.17909 3.91813 1310 - -
2.68618 4.42225 2.77519 2.73123 3.46354 2.40513 3.72494 3.29354 2.67741 2.69355 4.24690 2.90347 2.73739 3.18146 2.89801 2.37887 2.77519 2.98518 4.58477 3.61503
0.01712 4.47174 5.19409 0.61958 0.77255 0.48576 0.95510
1135 1.53130 4.57218 3.40800 2.88022 3.87598 2.76522 3.93055 3.23520 2.62077 2.93039 3.79795 3.30602 3.99855 3.17530 3.02038 2.82024 2.95006 2.19039 5.25608 3.99677 1311 - -
2.68618 4.42225 2.77519 2.73123 3.46354 2.40513 3.72494 3.29354 2.67741 2.69355 4.24690 2.90347 2.73739 3.18146 2.89801 2.37887 2.77519 2.98518 4.58477 3.61503
0.01712 4.47174 5.19409 0.61958 0.77255 0.48576 0.95510
1136 2.85340 5.33045 2.43555 1.86965 4.67950 3.52233 3.73868 4.14782 2.18461 3.61779 4.38538 2.98921 3.95544 2.85029 1.65145 2.80536 3.08371 3.72652 5.73719 4.34950 1312 - -
2.68618 4.42225 2.77519 2.73123 3.46354 2.40513 3.72494 3.29354 2.67741 2.69355 4.24690 2.90347 2.73739 3.18146 2.89801 2.37887 2.77519 2.98518 4.58477 3.61503
0.01712 4.47174 5.19409 0.61958 0.77255 0.48576 0.95510
1137 2.88625 5.09154 3.25254 2.69858 4.43133 3.62684 1.77974 3.86557 2.35319 3.40480 4.22563 3.18054 4.03661 2.92788 1.81681 2.51096 2.86672 3.51877 5.55220 4.25461 1313 - -
2.68618 4.42225 2.77519 2.73123 3.46354 2.40513 3.72494 3.29354 2.67741 2.69355 4.24690 2.90347 2.73739 3.18146 2.89801 2.37887 2.77519 2.98518 4.58477 3.61503

0.01712 4.47174 5.19409 0.61958 0.77255 0.48576 0.95510
1138 2.65835 1.22642 4.50768 4.29460 4.72637 1.06359 5.01746 4.12028 4.21707 3.89332 4.77080 4.07049 4.15449 4.45682 4.34285 2.88510 3.19571 3.57373 6.05256 4.98344 1314 - -
2.68618 4.42225 2.77519 2.73123 3.46354 2.40513 3.72494 3.29354 2.67741 2.69355 4.24690 2.90347 2.73739 3.18146 2.89801 2.37887 2.77519 2.98518 4.58477 3.61503
0.01712 4.47174 5.19409 0.61958 0.77255 0.48576 0.95510
1139 2.30472 4.17102 3.62782 3.53529 2.88712 3.78510 4.08449 1.72746 3.41827 1.97578 3.28065 3.75440 4.15441 3.64383 3.09167 3.06950 2.91912 2.41762 3.96273 3.58421 1315 - -
2.68618 4.42225 2.77519 2.73123 3.46354 2.40513 3.72494 3.29354 2.67741 2.69355 4.24690 2.90347 2.73739 3.18146 2.89801 2.37887 2.77519 2.98518 4.58477 3.61503
0.01712 4.47174 5.19409 0.61958 0.77255 0.48576 0.95510
1140 2.77111 5.23172 2.74129 2.32838 4.56981 3.29155 1.94401 4.03675 2.04128 3.52228 4.28140 2.79552 3.91249 2.80350 2.43085 2.73540 3.00010 3.62026 5.65532 4.26819 1316 - -
2.68618 4.42225 2.77519 2.73123 3.46354 2.40513 3.72494 3.29354 2.67741 2.69355 4.24690 2.90347 2.73739 3.18146 2.89801 2.37887 2.77519 2.98518 4.58477 3.61503
0.01712 4.47174 5.19409 0.61958 0.77255 0.48576 0.95510
1141 2.39223 4.71384 3.19459 2.91101 4.59740 1.15494 4.17900 4.04164 3.05834 3.65401 4.46690 3.30379 3.98529 2.75176 3.46660 2.09866 3.05885 3.55043 5.86520 4.57108 1317 - -
2.68618 4.42225 2.77519 2.73123 3.46354 2.40513 3.72494 3.29354 2.67741 2.69355 4.24690 2.90347 2.73739 3.18146 2.89801 2.37887 2.77519 2.98518 4.58477 3.61503
0.01712 4.47174 5.19409 0.61958 0.77255 0.48576 0.95510
1142 3.58354 4.85825 5.37390 4.79411 3.27995 4.89771 5.21179 2.19677 4.65709 0.99869 1.55291 4.99343 5.03229 4.65710 4.69013 4.25534 3.79768 2.16441 5.39882 4.38900 1318 - -
2.68618 4.42225 2.77519 2.73123 3.46354 2.40513 3.72494 3.29354 2.67741 2.69355 4.24690 2.90347 2.73739 3.18146 2.89801 2.37887 2.77519 2.98518 4.58477 3.61503
0.01712 4.47174 5.19409 0.61958 0.77255 0.48576 0.95510
1143 2.71269 5.09340 2.64985 1.97622 4.38417 3.48758 3.69275 3.47944 2.45449 3.37528 4.14966 2.78864 3.88936 2.55886 2.77756 2.69926 2.94494 3.45636 5.55455 2.23708 1319 - -
2.68618 4.42225 2.77519 2.73123 3.46354 2.40513 3.72494 3.29354 2.67741 2.69355 4.24690 2.90347 2.73739 3.18146 2.89801 2.37887 2.77519 2.98518 4.58477 3.61503
0.01712 4.47174 5.19409 0.61958 0.77255 0.48576 0.95510
1144 3.21863 5.23716 3.81402 3.06628 4.47593 3.91296 3.82656 3.94365 1.71970 3.44488 4.32351 3.45353 4.25516 2.98675 1.41297 3.25104 3.38217 3.65936 1.98587 4.28271 1320 - -
2.68618 4.42225 2.77519 2.73123 3.46354 2.40513 3.72494 3.29354 2.67741 2.69355 4.24690 2.90347 2.73739 3.18146 2.89801 2.37887 2.77519 2.98518 4.58477 3.61503
0.01712 4.47174 5.19409 0.61958 0.77255 0.48576 0.95510
1145 1.45232 4.38503 3.90290 3.43899 3.76662 3.53518 4.27812 2.99569 3.35953 1.54183 3.74004 3.67083 4.10794 3.66119 3.63538 2.48845 3.00872 2.75381 5.26834 4.05263 1321 - -
2.68618 4.42225 2.77519 2.73123 3.46354 2.40513 3.72494 3.29354 2.67741 2.69355 4.24690 2.90347 2.73739 3.18146 2.89801 2.37887 2.77519 2.98518 4.58477 3.61503
0.01712 4.47174 5.19409 0.61958 0.77255 0.48576 0.95510
1146 3.44091 4.74704 4.96523 4.49117 1.09537 4.48622 4.07449 2.62565 4.31989 1.73991 3.52387 4.39496 4.77552 4.33885 4.34353 3.81950 3.66183 2.89341 4.27693 2.08750 1322 - -
2.68618 4.42225 2.77519 2.73123 3.46354 2.40513 3.72494 3.29354 2.67741 2.69355 4.24690 2.90347 2.73739 3.18146 2.89801 2.37887 2.77519 2.98518 4.58477 3.61503
0.01712 4.47174 5.19409 0.61958 0.77255 0.48576 0.95510
1147 3.10286 5.49893 2.53180 2.13240 4.92386 3.47620 4.02723 4.43447 2.94754 3.95894 4.80655 0.94611 4.09201 3.19784 3.44815 2.73661 3.41072 4.00584 6.10743 4.66073 1323 - -
2.68618 4.42225 2.77519 2.73123 3.46354 2.40513 3.72494 3.29354 2.67741 2.69355 4.24690 2.90347 2.73739 3.18146 2.89801 2.37887 2.77519 2.98518 4.58477 3.61503
0.01712 4.47174 5.19409 0.61958 0.77255 0.48576 0.95510
1148 3.49073 4.77952 5.18644 4.69749 1.32270 4.70179 4.71028 1.32508 4.54772 1.95771 3.33577 4.74818 4.95420 4.60809 4.59731 4.07877 3.72844 2.36851 4.92710 3.46806 1324 - -
2.68618 4.42225 2.77519 2.73123 3.46354 2.40513 3.72494 3.29354 2.67741 2.69355 4.24690 2.90347 2.73739 3.18146 2.89801 2.37887 2.77519 2.98518 4.58477 3.61503
0.01712 4.47174 5.19409 0.61958 0.77255 0.48576 0.95510
1149 1.43456 5.12168 1.46513 2.56200 4.78687 2.78330 4.05520 4.24885 3.00295 3.82095 4.63662 3.08168 4.02070 3.22312 3.52289 2.87864 3.21749 3.78269 6.00847 4.61112 1325 - -

2.68618 4.42225 2.77519 2.73123 3.46354 2.40513 3.72494 3.29354 2.67741 2.69355 4.24690 2.90347 2.73739 3.18146 2.89801 2.37887 2.77519 2.98518 4.58477 3.61503
0.01712 4.47174 5.19409 0.61958 0.77255 0.48576 0.95510
1150 2.92579 4.66811 1.76906 3.11005 3.90991 3.86247 4.22870 2.24712 3.17654 2.74460 3.79467 3.56507 4.28428 3.48412 3.57939 3.17178 3.18317 1.47557 5.46921 4.18843 1326 - -
2.68618 4.42225 2.77519 2.73123 3.46354 2.40513 3.72494 3.29354 2.67741 2.69355 4.24690 2.90347 2.73739 3.18146 2.89801 2.37887 2.77519 2.98518 4.58477 3.61503
0.01712 4.47174 5.19409 0.61958 0.77255 0.48576 0.95510
1151 2.79469 5.28883 2.26838 2.38369 4.61387 3.47126 2.87166 4.09752 2.29107 3.58710 4.34691 2.06605 3.91516 2.84491 3.01450 1.97967 3.03434 3.67194 5.73017 4.31201 1327 - -
2.68618 4.42225 2.77519 2.73123 3.46354 2.40513 3.72494 3.29354 2.67741 2.69355 4.24690 2.90347 2.73739 3.18146 2.89801 2.37887 2.77519 2.98518 4.58477 3.61503
0.01712 4.47174 5.19409 0.61958 0.77255 0.48576 0.95510
1152 2.98732 5.12144 3.02425 2.81258 4.75037 1.35621 4.14147 4.27651 2.90662 3.82167 4.67516 3.28923 4.13485 1.41183 3.27784 3.02597 3.33353 3.83960 5.91831 4.61007 1328 - -
2.68618 4.42225 2.77519 2.73123 3.46354 2.40513 3.72494 3.29354 2.67741 2.69355 4.24690 2.90347 2.73739 3.18146 2.89801 2.37887 2.77519 2.98518 4.58477 3.61503
0.01712 4.47174 5.19409 0.61958 0.77255 0.48576 0.95510
1153 1.97196 4.29115 3.85824 3.28526 3.41710 3.75714 4.04100 2.24924 2.61762 2.47921 3.40341 3.60953 4.13236 3.47675 3.47494 3.02607 2.94486 1.82462 4.90916 3.11063 1329 - -
2.68618 4.42225 2.77519 2.73123 3.46354 2.40513 3.72494 3.29354 2.67741 2.69355 4.24690 2.90347 2.73739 3.18146 2.89801 2.37887 2.77519 2.98518 4.58477 3.61503
0.01712 4.47174 5.19409 0.61958 0.77255 0.48576 0.95510
1154 3.30382 4.65786 4.95286 4.37229 3.10018 4.47454 4.63612 2.56205 4.17725 0.97785 2.40225 4.51192 4.72355 4.29029 4.25035 3.80149 3.52308 2.66353 2.16090 3.71963 1330 - -
2.68618 4.42225 2.77519 2.73123 3.46354 2.40513 3.72494 3.29354 2.67741 2.69355 4.24690 2.90347 2.73739 3.18146 2.89801 2.37887 2.77519 2.98518 4.58477 3.61503
0.01712 4.47174 5.19409 0.61958 0.77255 0.48576 0.95510
1155 2.95419 5.09017 3.39972 2.60987 4.39189 3.71560 3.77677 3.77081 1.91747 1.70194 4.16192 3.24233 4.08423 2.59048 2.01584 2.98209 3.15421 3.46204 5.49749 4.24470 1331 - -
2.68618 4.42225 2.77519 2.73123 3.46354 2.40513 3.72494 3.29354 2.67741 2.69355 4.24690 2.90347 2.73739 3.18146 2.89801 2.37887 2.77519 2.98518 4.58477 3.61503
0.01712 4.47174 5.19409 0.61958 0.77255 0.48576 0.95510
1156 2.55513 4.31259 3.75963 3.18436 3.43630 3.71777 3.98494 2.68290 2.88935 1.95710 3.11260 3.19663 4.09293 3.39019 2.73849 2.97857 2.92527 1.79147 4.90966 3.69445 1332 - -
2.68618 4.42225 2.77519 2.73123 3.46354 2.40513 3.72494 3.29354 2.67741 2.69355 4.24690 2.90347 2.73739 3.18146 2.89801 2.37887 2.77519 2.98518 4.58477 3.61503
0.01712 4.47174 5.19409 0.61958 0.77255 0.48576 0.95510
1157 2.75950 4.75108 3.22708 2.45284 3.84446 3.60560 3.81365 3.32087 2.65712 2.97170 3.83928 3.19656 4.00385 2.11564 3.05908 2.85333 2.99082 2.53592 5.21524 2.01347 1333 - -
2.68618 4.42225 2.77519 2.73123 3.46354 2.40513 3.72494 3.29354 2.67741 2.69355 4.24690 2.90347 2.73739 3.18146 2.89801 2.37887 2.77519 2.98518 4.58477 3.61503
0.01712 4.47174 5.19409 0.61958 0.77255 0.48576 0.95510
1158 3.57401 5.24861 4.36381 4.35348 5.40728 0.20721 5.39221 5.19546 4.61166 4.75230 5.76109 4.53127 4.65161 4.88936 4.74225 3.76858 4.09816 4.60781 6.30299 5.54286 1334 - -
2.68618 4.42225 2.77519 2.73123 3.46354 2.40513 3.72494 3.29354 2.67741 2.69355 4.24690 2.90347 2.73739 3.18146 2.89801 2.37887 2.77519 2.98518 4.58477 3.61503
0.01712 4.47174 5.19409 0.61958 0.77255 0.48576 0.95510
1159 2.72004 4.35261 3.72574 2.78179 1.99359 3.72767 3.96389 2.82588 3.10391 2.53864 2.25766 2.78598 4.10510 3.38964 3.41182 2.98989 2.95194 2.61700 4.85828 3.21600 1335 - -
2.68618 4.42225 2.77519 2.73123 3.46354 2.40513 3.72494 3.29354 2.67741 2.69355 4.24690 2.90347 2.73739 3.18146 2.89801 2.37887 2.77519 2.98518 4.58477 3.61503
0.36557 4.47174 1.22157 0.61958 0.77255 0.48576 0.95510
1160 2.29395 4.79366 2.99474 2.24332 4.08076 3.24286 3.64924 3.49718 2.43536 3.09706 3.90850 2.96728 3.81860 2.79895 2.44835 2.34766 2.56387 2.93353 5.33384 4.00128 1336 - -
2.68618 4.42225 2.77519 2.73123 3.46354 2.40513 3.72494 3.29354 2.67741 2.69355 4.24690 2.90347 2.73739 3.18146 2.89801 2.37887 2.77519 2.98518 4.58477 3.61503
0.02417 4.13035 4.85270 0.61958 0.77255 0.87839 0.53692

1161 2.95858 5.15475 2.69759 2.10191 4.16409 3.45883 1.40817 3.90889 2.53306 3.44855 4.33524 3.00462 3.98115 2.99235 2.89560 2.94028 3.21307 3.58036 5.44381 4.02594 1337 - -
2.68618 4.42225 2.77519 2.73123 3.46354 2.40513 3.72494 3.29354 2.67741 2.69355 4.24690 2.90347 2.73739 3.18146 2.89801 2.37887 2.77519 2.98518 4.58477 3.61503
0.02417 4.13035 4.85270 0.61958 0.77255 0.30088 1.34773
1162 2.64211 5.23465 2.97191 2.27269 4.57924 3.52620 3.70806 4.03689 2.04391 3.52356 4.29049 1.75437 3.93362 2.81790 2.33936 2.61025 3.02523 3.62827 5.65462 4.28201 1338 - -
2.68618 4.42225 2.77519 2.73123 3.46354 2.40513 3.72494 3.29354 2.67741 2.69355 4.24690 2.90347 2.73739 3.18146 2.89801 2.37887 2.77519 2.98518 4.58477 3.61503
0.01712 4.47174 5.19409 0.61958 0.77255 0.48576 0.95510
1163 2.65845 4.80544 2.67603 2.65380 4.26671 2.47262 3.86436 3.68530 2.69213 3.29628 4.10691 3.13449 3.92796 3.02510 3.14812 2.16135 1.77398 2.83786 5.53478 4.20962 1339 - -
2.68618 4.42225 2.77519 2.73123 3.46354 2.40513 3.72494 3.29354 2.67741 2.69355 4.24690 2.90347 2.73739 3.18146 2.89801 2.37887 2.77519 2.98518 4.58477 3.61503
0.01712 4.47174 5.19409 0.61958 0.77255 0.48576 0.95510
1164 3.08113 4.45192 4.75588 4.18669 3.42573 4.30396 4.67534 1.39241 4.04917 1.39944 3.30562 4.35847 3.21505 4.23345 4.18215 3.62848 3.31900 1.92846 5.19548 4.04135 1340 - -
2.68618 4.42225 2.77519 2.73123 3.46354 2.40513 3.72494 3.29354 2.67741 2.69355 4.24690 2.90347 2.73739 3.18146 2.89801 2.37887 2.77519 2.98518 4.58477 3.61503
0.40024 4.47174 1.14441 0.61958 0.77255 0.48576 0.95510
1165 3.76328 4.96455 4.73516 4.44889 1.26849 4.48324 3.50296 3.43600 4.28800 2.74595 4.02503 4.14785 4.78584 4.22044 4.29506 3.85847 3.98281 3.36477 3.63059 1.06298 1341 - -
2.68618 4.42225 2.77519 2.73123 3.46354 2.40513 3.72494 3.29354 2.67741 2.69355 4.24690 2.90347 2.73739 3.18146 2.89801 2.37887 2.77519 2.98518 4.58477 3.61503
0.02502 4.09652 4.81887 0.61958 0.77255 0.90459 0.51870
1166 3.36778 4.69546 4.89050 4.36764 3.15690 4.60589 4.93607 1.83476 4.17878 0.85167 2.97796 4.62919 4.81344 4.35443 4.31282 3.98821 3.60326 2.18465 5.25016 4.12043 1342 - -
2.68618 4.42225 2.77519 2.73123 3.46354 2.40513 3.72494 3.29354 2.67741 2.69355 4.24690 2.90347 2.73739 3.18146 2.89801 2.37887 2.77519 2.98518 4.58477 3.61503
0.02502 4.09652 4.81887 0.61958 0.77255 0.90459 0.51870
1167 2.75078 4.84192 3.13626 2.67288 4.29953 3.44793 3.75285 3.62381 1.50908 3.25078 4.11919 3.12020 3.92749 2.91586 2.61466 2.80642 1.99469 3.29428 5.45946 4.19442 1343 - -
2.68618 4.42225 2.77519 2.73123 3.46354 2.40513 3.72494 3.29354 2.67741 2.69355 4.24690 2.90347 2.73739 3.18146 2.89801 2.37887 2.77519 2.98518 4.58477 3.61503
0.02502 4.09652 4.81887 0.61958 0.77255 0.90459 0.51870
1168 2.89147 5.08990 2.91105 2.54346 4.07828 3.50074 2.52547 3.84942 2.31208 3.35239 4.22505 3.03778 3.95889 1.53692 2.63176 2.88425 3.12323 3.51985 5.32802 3.92001 1344 - -
2.68618 4.42225 2.77519 2.73123 3.46354 2.40513 3.72494 3.29354 2.67741 2.69355 4.24690 2.90347 2.73739 3.18146 2.89801 2.37887 2.77519 2.98518 4.58477 3.61503
0.02502 4.09652 4.81887 0.61958 0.77255 0.90459 0.51870
1169 2.36301 4.34610 3.26010 2.94418 4.31006 2.14748 4.10195 3.71797 3.02000 3.38443 4.21382 3.23659 1.69564 3.33360 3.38355 2.51233 2.26207 3.22866 5.62439 4.37796 1345 - -
2.68618 4.42225 2.77519 2.73123 3.46354 2.40513 3.72494 3.29354 2.67741 2.69355 4.24690 2.90347 2.73739 3.18146 2.89801 2.37887 2.77519 2.98518 4.58477 3.61503
0.02502 4.09652 4.81887 0.61958 0.77255 0.90459 0.51870
1170 3.27534 5.08730 3.79049 3.26179 4.49964 3.71266 4.01132 4.06195 2.36107 3.53729 4.53657 3.60176 4.20883 3.22485 0.68436 3.35179 3.52888 3.77741 5.49442 4.38373 1346 - -
2.68618 4.42225 2.77519 2.73123 3.46354 2.40513 3.72494 3.29354 2.67741 2.69355 4.24690 2.90347 2.73739 3.18146 2.89801 2.37887 2.77519 2.98518 4.58477 3.61503
0.02502 4.09652 4.81887 0.61958 0.77255 0.90459 0.51870
1171 2.13026 4.65214 2.82192 2.64605 4.37298 3.18772 3.99046 3.80150 2.87746 3.48716 4.35752 1.28484 3.84792 3.20408 3.27380 2.67458 2.98652 3.36941 5.67400 4.33311 1347 - -
2.68618 4.42225 2.77519 2.73123 3.46354 2.40513 3.72494 3.29354 2.67741 2.69355 4.24690 2.90347 2.73739 3.18146 2.89801 2.37887 2.77519 2.98518 4.58477 3.61503
0.02502 4.09652 4.81887 0.61958 0.77255 0.90459 0.51870
1172 2.66697 4.75150 3.04036 2.57575 3.99878 3.43015 3.71936 3.43470 2.45639 2.59604 3.91877 3.05551 3.88083 1.87074 2.83101 2.21702 2.92506 3.13388 5.29771 3.97590 1348 - -
2.68618 4.42225 2.77519 2.73123 3.46354 2.40513 3.72494 3.29354 2.67741 2.69355 4.24690 2.90347 2.73739 3.18146 2.89801 2.37887 2.77519 2.98518 4.58477 3.61503

0.02502 4.09652 4.81887 0.61958 0.77255 0.90459 0.51870

1173 2.69792 4.91996 1.94471 2.39163 4.50457 3.22232 3.87460 3.99653 2.82451 3.58878 4.42545 2.90812 3.84407 3.05598 3.32626 1.38265 3.04761 3.55635 5.75827 4.36048 1349 - -

2.68618 4.42225 2.77519 2.73123 3.46354 2.40513 3.72494 3.29354 2.67741 2.69355 4.24690 2.90347 2.73739 3.18146 2.89801 2.37887 2.77519 2.98518 4.58477 3.61503

0.02502 4.09652 4.81887 0.61958 0.77255 0.90459 0.51870

1174 2.39431 4.36367 3.24110 3.00181 4.33648 3.07541 4.17157 3.80320 3.09755 3.46973 4.32145 3.27448 1.38467 3.42584 3.43195 1.84949 2.85621 3.29588 5.66159 4.40110 1350 - -

2.68618 4.42225 2.77519 2.73123 3.46354 2.40513 3.72494 3.29354 2.67741 2.69355 4.24690 2.90347 2.73739 3.18146 2.89801 2.37887 2.77519 2.98518 4.58477 3.61503

0.02502 4.09652 4.81887 0.61958 0.77255 0.90459 0.51870

1175 3.55768 4.89539 4.54704 4.30922 0.70572 4.18416 3.99699 3.05980 4.21210 2.38042 3.75988 4.27904 4.64157 4.31940 4.27613 3.81243 3.86089 3.06610 4.18921 2.53208 1351 - -

2.68618 4.42225 2.77519 2.73123 3.46354 2.40513 3.72494 3.29354 2.67741 2.69355 4.24690 2.90347 2.73739 3.18146 2.89801 2.37887 2.77519 2.98518 4.58477 3.61503

0.02502 4.09652 4.81887 0.61958 0.77255 0.90459 0.51870

1176 2.63586 4.65952 3.08333 2.60604 3.00667 3.44016 3.71980 3.30756 2.56968 2.93128 3.81302 3.08016 3.88137 1.92132 2.97266 2.23283 2.89344 3.02378 5.12158 3.74815 1352 - -

2.68618 4.42225 2.77519 2.73123 3.46354 2.40513 3.72494 3.29354 2.67741 2.69355 4.24690 2.90347 2.73739 3.18146 2.89801 2.37887 2.77519 2.98518 4.58477 3.61503

0.02502 4.09652 4.81887 0.61958 0.77255 0.90459 0.51870

1177 2.96799 4.71525 3.68718 3.61793 4.74485 0.45423 4.71894 4.40446 3.82935 4.02854 5.01019 3.85228 4.11316 4.13617 4.03545 3.14723 3.46664 3.88946 5.77513 4.85627 1353 - -

2.68618 4.42225 2.77519 2.73123 3.46354 2.40513 3.72494 3.29354 2.67741 2.69355 4.24690 2.90347 2.73739 3.18146 2.89801 2.37887 2.77519 2.98518 4.58477 3.61503

0.02502 4.09652 4.81887 0.61958 0.77255 0.90459 0.51870

1178 3.16457 4.53565 4.70731 4.19878 3.31784 4.37729 4.83284 2.01716 4.03868 1.18050 3.15799 4.43415 4.68544 4.27297 4.21126 3.75558 3.42437 1.43111 5.30003 4.12851 1354 - -

2.68618 4.42225 2.77519 2.73123 3.46354 2.40513 3.72494 3.29354 2.67741 2.69355 4.24690 2.90347 2.73739 3.18146 2.89801 2.37887 2.77519 2.98518 4.58477 3.61503

0.02502 4.09652 4.81887 0.61958 0.77255 0.90459 0.51870

1179 3.16039 4.67678 4.29805 3.91421 3.32640 4.00582 4.59764 2.52534 3.66841 1.89210 1.00525 4.16228 4.47259 4.03829 3.84622 3.53024 3.47272 2.55636 5.19371 3.97266 1355 - -

2.68618 4.42225 2.77519 2.73123 3.46354 2.40513 3.72494 3.29354 2.67741 2.69355 4.24690 2.90347 2.73739 3.18146 2.89801 2.37887 2.77519 2.98518 4.58477 3.61503

0.02502 4.09652 4.81887 0.61958 0.77255 0.90459 0.51870

1180 3.58653 4.98405 4.17677 3.95328 2.33811 4.09723 3.74683 3.56642 3.80478 2.96102 4.21898 4.01036 4.56820 4.05581 3.94014 3.69866 3.87563 3.44823 3.98866 0.66145 1356 - -

2.68618 4.42225 2.77519 2.73123 3.46354 2.40513 3.72494 3.29354 2.67741 2.69355 4.24690 2.90347 2.73739 3.18146 2.89801 2.37887 2.77519 2.98518 4.58477 3.61503

0.02502 4.09652 4.81887 0.61958 0.77255 0.90459 0.51870

1181 3.24651 5.39095 0.64312 2.50354 4.80187 3.39506 4.15009 4.44451 3.27990 4.01494 5.00362 3.06907 4.06413 3.39356 3.82228 3.18742 3.59179 4.06160 5.92512 4.64506 1357 - -

2.68618 4.42225 2.77519 2.73123 3.46354 2.40513 3.72494 3.29354 2.67741 2.69355 4.24690 2.90347 2.73739 3.18146 2.89801 2.37887 2.77519 2.98518 4.58477 3.61503

0.02502 4.09652 4.81887 0.61958 0.77255 0.90459 0.51870

1182 3.58653 4.98405 4.17677 3.95328 2.33811 4.09723 3.74683 3.56642 3.80478 2.96102 4.21898 4.01036 4.56820 4.05581 3.94014 3.69866 3.87563 3.44823 3.98866 0.66145 1358 - -

2.68618 4.42225 2.77519 2.73123 3.46354 2.40513 3.72494 3.29354 2.67741 2.69355 4.24690 2.90347 2.73739 3.18146 2.89801 2.37887 2.77519 2.98518 4.58477 3.61503

0.02502 4.09652 4.81887 0.61958 0.77255 0.90459 0.51870

1183 3.36778 4.69546 4.89050 4.36764 3.15690 4.60589 4.93607 1.83476 4.17878 0.85167 2.97796 4.62919 4.81344 4.35443 4.31282 3.98821 3.60326 2.18465 5.25016 4.12043 1359 - -

2.68618 4.42225 2.77519 2.73123 3.46354 2.40513 3.72494 3.29354 2.67741 2.69355 4.24690 2.90347 2.73739 3.18146 2.89801 2.37887 2.77519 2.98518 4.58477 3.61503

0.02502 4.09652 4.81887 0.61958 0.77255 0.90459 0.51870

1184 3.27534 5.08730 3.79049 3.26179 4.49964 3.71266 4.01132 4.06195 2.36107 3.53729 4.53657 3.60176 4.20883 3.22485 0.68436 3.35179 3.52888 3.77741 5.49442 4.38373 1360 - -

2.68618 4.42225 2.77519 2.73123 3.46354 2.40513 3.72494 3.29354 2.67741 2.69355 4.24690 2.90347 2.73739 3.18146 2.89801 2.37887 2.77519 2.98518 4.58477 3.61503
0.02502 4.09652 4.81887 0.61958 0.77255 0.90459 0.51870

1185 3.31649 4.69010 4.74826 4.20151 3.09503 4.48941 4.78102 2.26089 4.00157 1.10812 1.62465 4.47842 4.70973 4.19086 4.14746 3.84695 3.54510 2.39094 5.15383 4.04695 1361 - -
2.68618 4.42225 2.77519 2.73123 3.46354 2.40513 3.72494 3.29354 2.67741 2.69355 4.24690 2.90347 2.73739 3.18146 2.89801 2.37887 2.77519 2.98518 4.58477 3.61503
0.02502 4.09652 4.81887 0.61958 0.77255 0.90459 0.51870

1186 3.16393 5.30333 2.60663 0.74394 4.68056 3.43230 4.04645 4.18022 2.95681 3.76379 4.73428 3.08464 4.04944 3.26144 3.38441 3.12707 3.47193 3.84321 5.80957 4.53968 1362 - -
2.68618 4.42225 2.77519 2.73123 3.46354 2.40513 3.72494 3.29354 2.67741 2.69355 4.24690 2.90347 2.73739 3.18146 2.89801 2.37887 2.77519 2.98518 4.58477 3.61503
0.02502 4.09652 4.81887 0.61958 0.77255 0.90459 0.51870

1187 2.85919 5.11856 2.49418 1.48371 4.61218 1.71134 3.87528 4.08477 2.78493 3.65209 4.50628 2.91112 3.90303 3.05449 3.26275 2.83942 3.17562 3.67702 5.79556 4.43026 1363 - -
2.68618 4.42225 2.77519 2.73123 3.46354 2.40513 3.72494 3.29354 2.67741 2.69355 4.24690 2.90347 2.73739 3.18146 2.89801 2.37887 2.77519 2.98518 4.58477 3.61503
0.02502 4.09652 4.81887 0.61958 0.77255 0.29184 1.37393

1188 2.54254 4.80186 2.89995 2.51819 4.02644 2.97269 3.80085 3.42503 2.65624 1.86823 3.90605 2.94025 2.50516 2.98652 3.10512 2.79110 2.96387 3.13055 5.34803 4.03782 1364 - -
2.68618 4.42225 2.77519 2.73123 3.46354 2.40513 3.72494 3.29354 2.67741 2.69355 4.24690 2.90347 2.73739 3.18146 2.89801 2.37887 2.77519 2.98518 4.58477 3.61503
0.35726 4.47174 1.24140 0.61958 0.77255 0.48576 0.95510

1189 2.79293 4.95646 3.16772 2.63444 4.37422 3.50454 3.69100 3.77599 2.07520 3.31450 4.14642 3.09658 3.93610 2.83599 1.76881 1.86015 3.03131 3.42721 5.46182 4.19385 1365 - -
2.68618 4.42225 2.77519 2.73123 3.46354 2.40513 3.72494 3.29354 2.67741 2.69355 4.24690 2.90347 2.73739 3.18146 2.89801 2.37887 2.77519 2.98518 4.58477 3.61503
0.02398 4.13846 4.86081 0.61958 0.77255 0.30317 1.34121

1190 2.48125 4.44946 3.75989 3.46004 4.73716 2.05377 4.54726 4.18494 3.52889 3.84275 4.63647 3.59343 3.96352 3.80077 3.85374 0.94064 2.23155 3.55365 6.04808 4.84244 1366 - -
2.68618 4.42225 2.77519 2.73123 3.46354 2.40513 3.72494 3.29354 2.67741 2.69355 4.24690 2.90347 2.73739 3.18146 2.89801 2.37887 2.77519 2.98518 4.58477 3.61503
0.01712 4.47174 5.19409 0.61958 0.77255 0.48576 0.95510

1191 2.59574 4.88734 2.73296 2.53622 4.13987 3.51399 3.73053 3.55598 2.53001 3.15960 3.60391 3.05400 2.26907 2.88047 2.72064 2.27115 2.79032 2.69734 5.40266 4.06758 1367 - -
2.68618 4.42225 2.77519 2.73123 3.46354 2.40513 3.72494 3.29354 2.67741 2.69355 4.24690 2.90347 2.73739 3.18146 2.89801 2.37887 2.77519 2.98518 4.58477 3.61503
0.01956 4.33964 5.06199 0.61958 0.77255 0.48576 0.95510

1192 2.33696 4.68637 3.19945 2.48665 3.88402 3.53757 3.74259 3.27332 2.09896 2.20362 3.77121 3.12245 3.51138 2.93861 2.75489 2.75763 2.89688 2.99562 3.72480 3.92559 1368 - -
2.68618 4.42225 2.77519 2.73123 3.46354 2.40513 3.72494 3.29354 2.67741 2.69355 4.24690 2.90347 2.73739 3.18146 2.89801 2.37887 2.77519 2.98518 4.58477 3.61503
0.01956 4.33964 5.06199 0.61958 0.77255 0.48576 0.95510

1193 2.67862 4.28729 3.77438 3.20365 3.42660 3.70693 4.00117 2.20442 3.10711 2.34033 3.40178 3.54440 4.08990 3.40680 2.90590 2.15803 2.91670 1.83935 4.91815 3.70237 1369 - -
2.68618 4.42225 2.77519 2.73123 3.46354 2.40513 3.72494 3.29354 2.67741 2.69355 4.24690 2.90347 2.73739 3.18146 2.89801 2.37887 2.77519 2.98518 4.58477 3.61503
0.01956 4.33964 5.06199 0.61958 0.77255 0.48576 0.95510

1194 2.69916 5.12725 2.63779 2.40326 4.45512 3.46085 3.64484 3.91467 1.92283 3.41769 4.18059 2.94112 3.85922 2.60746 2.34132 2.54082 2.20772 3.51033 5.56531 4.18536 1370 - -
2.68618 4.42225 2.77519 2.73123 3.46354 2.40513 3.72494 3.29354 2.67741 2.69355 4.24690 2.90347 2.73739 3.18146 2.89801 2.37887 2.77519 2.98518 4.58477 3.61503
0.01956 4.33964 5.06199 0.61958 0.77255 0.48576 0.95510

1195 2.69868 4.85791 3.18221 2.68085 4.38974 3.44035 3.82455 3.81864 2.49228 3.38561 4.19755 3.14137 2.31568 2.97485 1.85088 1.93509 2.99228 3.42608 5.56549 4.26289 1371 - -
2.68618 4.42225 2.77519 2.73123 3.46354 2.40513 3.72494 3.29354 2.67741 2.69355 4.24690 2.90347 2.73739 3.18146 2.89801 2.37887 2.77519 2.98518 4.58477 3.61503
0.01956 4.33964 5.06199 0.61958 0.77255 0.48576 0.95510

1196 2.75486 4.29106 3.97734 3.40212 3.35514 3.83353 4.12283 2.10366 3.30902 2.04433 2.82315 3.71021 4.19234 2.33472 3.56820 3.10883 2.85965 1.87973 4.92423 3.72960 1372 - -
2.68618 4.42225 2.77519 2.73123 3.46354 2.40513 3.72494 3.29354 2.67741 2.69355 4.24690 2.90347 2.73739 3.18146 2.89801 2.37887 2.77519 2.98518 4.58477 3.61503
0.01956 4.33964 5.06199 0.61958 0.77255 0.48576 0.95510
1197 1.86962 4.74990 3.20762 2.67536 4.21834 3.42441 3.81748 3.62169 2.56495 3.23246 4.05259 3.13640 2.80221 2.98048 1.96257 2.70639 2.55406 3.26035 5.46586 4.16493 1373 - -
2.68618 4.42225 2.77519 2.73123 3.46354 2.40513 3.72494 3.29354 2.67741 2.69355 4.24690 2.90347 2.73739 3.18146 2.89801 2.37887 2.77519 2.98518 4.58477 3.61503
0.13215 4.33964 2.20045 0.61958 0.77255 0.48576 0.95510
1198 2.61287 4.74424 2.92765 2.61352 4.39121 3.28737 3.90434 3.82490 2.74075 3.43411 4.25483 1.72627 2.12582 3.07858 3.16998 2.67929 2.47381 3.40029 5.64230 4.31264 1374 - -
2.68618 4.42225 2.77519 2.73123 3.46354 2.40513 3.72494 3.29354 2.67741 2.69355 4.24690 2.90347 2.73739 3.18146 2.89801 2.37887 2.77519 2.98518 4.58477 3.61503
0.02187 4.22936 4.95170 0.61958 0.77255 0.62738 0.76355
1199 2.80446 5.27531 2.48585 1.42559 4.59791 3.41007 3.69344 4.07058 2.44934 3.56019 4.34305 2.87808 3.88048 2.56417 2.41033 2.75069 3.04457 3.65909 5.70067 4.29602 1375 - -
2.68618 4.42225 2.77519 2.73123 3.46354 2.40513 3.72494 3.29354 2.67741 2.69355 4.24690 2.90347 2.73739 3.18146 2.89801 2.37887 2.77519 2.98518 4.58477 3.61503
0.02187 4.22936 4.95170 0.61958 0.77255 0.62738 0.76355
1200 1.49131 4.27314 3.65992 3.30121 4.33086 2.43063 4.32246 3.68769 3.31281 3.41330 4.24846 3.44859 3.82423 3.59862 3.62909 2.52974 1.45922 3.19216 5.69147 4.48636 1376 - -
2.68618 4.42225 2.77519 2.73123 3.46354 2.40513 3.72494 3.29354 2.67741 2.69355 4.24690 2.90347 2.73739 3.18146 2.89801 2.37887 2.77519 2.98518 4.58477 3.61503
0.02187 4.22936 4.95170 0.61958 0.77255 0.62738 0.76355
1201 2.61142 4.49753 2.96728 2.71610 2.46512 3.03966 3.77069 3.04913 2.69230 2.73411 3.60352 3.17585 3.91466 3.01887 2.73638 2.58243 2.64544 2.47634 5.06634 3.79954 1377 - -
2.68618 4.42225 2.77519 2.73123 3.46354 2.40513 3.72494 3.29354 2.67741 2.69355 4.24690 2.90347 2.73739 3.18146 2.89801 2.37887 2.77519 2.98518 4.58477 3.61503
0.02353 4.15724 4.87958 0.61958 0.77255 0.62738 0.76355
1202 2.65076 5.08970 2.09115 2.22586 4.00488 3.38265 3.61086 3.86714 2.30033 3.38020 4.14541 2.55916 3.80232 2.72449 2.61935 2.33873 2.88602 3.46308 5.54121 4.14349 1378 - -
2.68618 4.42225 2.77519 2.73123 3.46354 2.40513 3.72494 3.29354 2.67741 2.69355 4.24690 2.90347 2.73739 3.18146 2.89801 2.37887 2.77519 2.98518 4.58477 3.61503
0.02353 4.15724 4.87958 0.61958 0.77255 0.62738 0.76355
1203 3.17587 4.87516 3.81524 3.70218 4.65734 3.54705 4.74775 4.28704 3.78609 3.87814 4.94631 3.97581 0.43543 4.16570 3.99088 3.35433 3.64143 3.89781 5.75529 4.78482 1379 - -
2.68618 4.42225 2.77519 2.73123 3.46354 2.40513 3.72494 3.29354 2.67741 2.69355 4.24690 2.90347 2.73739 3.18146 2.89801 2.37887 2.77519 2.98518 4.58477 3.61503
0.02353 4.15724 4.87958 0.61958 0.77255 0.40642 1.09671
1204 3.48362 4.77861 5.09959 4.56517 1.48931 4.68663 4.67126 2.26988 4.41732 1.08603 3.02637 4.69101 4.86769 4.43761 4.46755 4.03999 3.70028 2.54896 4.86449 3.49056 1380 - -
2.68618 4.42225 2.77519 2.73123 3.46354 2.40513 3.72494 3.29354 2.67741 2.69355 4.24690 2.90347 2.73739 3.18146 2.89801 2.37887 2.77519 2.98518 4.58477 3.61503
0.02088 4.27530 4.99765 0.61958 0.77255 0.48576 0.95510
1205 2.74614 5.16320 1.90008 2.03942 4.46688 3.40812 3.69883 3.92239 2.42897 3.45640 4.24090 2.89194 3.86705 2.82577 3.00876 2.71274 2.12206 3.27002 5.63455 4.23219 1381 - -
2.68618 4.42225 2.77519 2.73123 3.46354 2.40513 3.72494 3.29354 2.67741 2.69355 4.24690 2.90347 2.73739 3.18146 2.89801 2.37887 2.77519 2.98518 4.58477 3.61503
0.02088 4.27530 4.99765 0.61958 0.77255 0.48576 0.95510
1206 2.35194 4.59659 3.04632 2.64321 3.39740 3.51831 3.39615 3.16456 2.62453 2.83014 3.48549 2.97570 3.90517 2.95854 3.05097 1.98105 2.86017 2.79671 5.13830 3.15396 1382 - -
2.68618 4.42225 2.77519 2.73123 3.46354 2.40513 3.72494 3.29354 2.67741 2.69355 4.24690 2.90347 2.73739 3.18146 2.89801 2.37887 2.77519 2.98518 4.58477 3.61503
0.02088 4.27530 4.99765 0.61958 0.77255 0.48576 0.95510
1207 2.64185 4.73039 2.90366 2.54861 3.94061 3.24580 3.70806 3.34364 2.20465 2.97974 3.81597 3.05245 2.95252 2.88726 2.98228 2.70868 2.87502 2.59077 5.25472 2.57000 1383 - -
2.68618 4.42225 2.77519 2.73123 3.46354 2.40513 3.72494 3.29354 2.67741 2.69355 4.24690 2.90347 2.73739 3.18146 2.89801 2.37887 2.77519 2.98518 4.58477 3.61503

0.02088 4.27530 4.99765 0.61958 0.77255 0.48576 0.95510

1208 2.55940 5.23471 1.85159 2.14132 4.56845 3.38768 3.72652 4.04568 2.56193 3.55766 4.33881 2.67801 3.87568 2.85435 3.07920 1.96073 2.79224 3.63114 5.72253 4.30063 1384 - -

2.68618 4.42225 2.77519 2.73123 3.46354 2.40513 3.72494 3.29354 2.67741 2.69355 4.24690 2.90347 2.73739 3.18146 2.89801 2.37887 2.77519 2.98518 4.58477 3.61503

0.02088 4.27530 4.99765 0.61958 0.77255 0.48576 0.95510

1209 2.45776 5.17359 2.87712 2.02795 4.50957 3.45816 3.65096 3.96608 2.10471 3.45822 4.22940 2.79612 3.87235 1.89921 2.66963 2.70915 2.97051 3.56203 5.59474 4.22095 1385 - -

2.68618 4.42225 2.77519 2.73123 3.46354 2.40513 3.72494 3.29354 2.67741 2.69355 4.24690 2.90347 2.73739 3.18146 2.89801 2.37887 2.77519 2.98518 4.58477 3.61503

0.02088 4.27530 4.99765 0.61958 0.77255 0.48576 0.95510

1210 2.54457 4.74703 3.09252 2.34091 2.96795 3.48637 3.69505 3.36472 2.51260 2.68680 3.82554 3.04275 3.48542 2.18460 2.76154 2.69836 2.71164 3.06619 5.26512 3.95208 1386 - -

2.68618 4.42225 2.77519 2.73123 3.46354 2.40513 3.72494 3.29354 2.67741 2.69355 4.24690 2.90347 2.73739 3.18146 2.89801 2.37887 2.77519 2.98518 4.58477 3.61503

0.27167 4.23385 1.49881 0.61958 0.77255 0.48576 0.95510

1211 2.46771 4.51513 3.27183 2.71648 3.54164 3.51736 3.71241 3.02636 2.55563 2.36663 3.57938 3.16324 3.90698 2.07332 2.90946 2.77082 2.86517 2.79869 3.90055 3.65033 1387 - -

2.68618 4.42225 2.77519 2.73123 3.46354 2.40513 3.72494 3.29354 2.67741 2.69355 4.24690 2.90347 2.73739 3.18146 2.89801 2.37887 2.77519 2.98518 4.58477 3.61503

0.03081 3.89121 4.61356 0.61958 0.77255 0.73540 0.65260

1212 2.67442 4.78817 2.73179 2.47684 4.24271 2.50776 3.75121 3.73758 2.48664 3.31267 4.18542 2.95770 3.81555 1.64919 2.84332 2.71471 2.98707 3.36759 5.45755 4.14311 1388 - -

2.68618 4.42225 2.77519 2.73123 3.46354 2.40513 3.72494 3.29354 2.67741 2.69355 4.24690 2.90347 2.73739 3.18146 2.89801 2.37887 2.77519 2.98518 4.58477 3.61503

0.03081 3.89121 4.61356 0.61958 0.77255 0.35635 1.20474

1213 1.97876 4.89826 2.34241 2.40666 4.21974 2.92377 3.66180 3.65035 2.45242 3.03832 4.02740 2.93176 3.82132 2.80085 2.60183 2.64044 2.87685 3.28990 5.44077 4.08445 1389 - -

2.68618 4.42225 2.77519 2.73123 3.46354 2.40513 3.72494 3.29354 2.67741 2.69355 4.24690 2.90347 2.73739 3.18146 2.89801 2.37887 2.77519 2.98518 4.58477 3.61503

0.02353 4.15724 4.87958 0.61958 0.77255 0.48576 0.95510

1214 2.91371 4.31430 4.43026 3.86731 3.41184 4.12625 4.48888 1.74366 3.34095 1.75399 3.30187 4.10496 4.45355 3.98680 3.92601 3.44201 3.15335 1.37602 5.13068 3.94376 1390 - -

2.68618 4.42225 2.77519 2.73123 3.46354 2.40513 3.72494 3.29354 2.67741 2.69355 4.24690 2.90347 2.73739 3.18146 2.89801 2.37887 2.77519 2.98518 4.58477 3.61503

0.02353 4.15724 4.87958 0.61958 0.77255 0.48576 0.95510

1215 2.75905 4.48992 3.61701 3.00919 3.56483 3.68731 3.85754 2.88204 2.48758 1.56331 3.21374 3.39669 4.05467 3.14320 2.36305 2.96692 2.97946 2.70753 5.02086 3.80283 1391 - -

2.68618 4.42225 2.77519 2.73123 3.46354 2.40513 3.72494 3.29354 2.67741 2.69355 4.24690 2.90347 2.73739 3.18146 2.89801 2.37887 2.77519 2.98518 4.58477 3.61503

0.02353 4.15724 4.87958 0.61958 0.77255 0.48576 0.95510

1216 2.54255 4.88605 2.81367 2.44185 4.16558 3.42881 2.73505 3.59127 2.39606 3.17142 3.97937 2.96047 3.83865 2.79011 2.41124 2.66518 2.12172 3.25003 5.38572 4.04677 1392 - -

2.68630 4.42237 2.77467 2.73135 3.46366 2.40525 3.72506 3.29366 2.67753 2.69367 4.24702 2.90359 2.73751 3.18061 2.89813 2.37899 2.77467 2.98530 4.58489 3.61515

0.28752 1.41768 4.87958 0.98672 0.46649 0.48576 0.95510

1217 2.47170 4.52022 3.24943 2.73303 3.81623 3.43997 3.80528 3.14969 2.53424 2.84467 3.06998 3.17274 1.95745 3.03334 3.03707 2.72347 2.64420 2.87557 5.18589 3.92012 1396 - -

2.68618 4.42225 2.77519 2.73123 3.46354 2.40513 3.72494 3.29354 2.67741 2.69355 4.24690 2.90347 2.73739 3.18146 2.89801 2.37887 2.77519 2.98518 4.58477 3.61503

0.02353 4.15724 4.87958 0.61958 0.77255 0.48576 0.95510

1218 2.45066 4.68151 3.08635 2.55208 3.92673 3.44831 3.13113 3.12814 2.41588 2.96086 3.80069 3.04456 2.33669 2.87935 2.93329 2.68614 2.48546 3.02484 5.23700 3.93497 1397 - -

2.68618 4.42225 2.77519 2.73123 3.46354 2.40513 3.72494 3.29354 2.67741 2.69355 4.24690 2.90347 2.73739 3.18146 2.89801 2.37887 2.77519 2.98518 4.58477 3.61503

0.02353 4.15724 4.87958 0.61958 0.77255 0.48576 0.95510

1219 2.65013 4.10915 4.15066 3.56787 2.45720 3.76917 4.08567 2.29682 3.44366 2.18549 2.76174 3.76651 3.77001 3.65957 3.61641 2.89032 2.88522 1.67426 4.73971 3.54454 1398 - -

2.68618 4.42225 2.77519 2.73123 3.46354 2.40513 3.72494 3.29354 2.67741 2.69355 4.24690 2.90347 2.73739 3.18146 2.89801 2.37887 2.77519 2.98518 4.58477 3.61503

0.02353 4.15724 4.87958 0.61958 0.77255 0.48576 0.95510

1220 2.66944 4.99567 2.92597 2.09750 4.29943 3.43136 3.62371 3.73386 2.06450 3.12485 4.06716 2.93343 3.83553 2.74948 2.62149 2.01301 2.76733 3.36727 5.45674 4.10521 1399 - -

2.68618 4.42225 2.77519 2.73123 3.46354 2.40513 3.72494 3.29354 2.67741 2.69355 4.24690 2.90347 2.73739 3.18146 2.89801 2.37887 2.77519 2.98518 4.58477 3.61503

0.02353 4.15724 4.87958 0.61958 0.77255 0.48576 0.95510

1221 1.32371 4.37313 3.36516 2.95889 4.10708 3.19672 4.04213 3.44969 2.75496 3.16378 4.01599 3.27462 3.82993 3.28454 3.29415 2.41908 2.16074 3.06056 5.45869 4.21475 1400 - -

2.68618 4.42225 2.77519 2.73123 3.46354 2.40513 3.72494 3.29354 2.67741 2.69355 4.24690 2.90347 2.73739 3.18146 2.89801 2.37887 2.77519 2.98518 4.58477 3.61503

0.29730 4.12842 1.42265 0.61958 0.77255 0.48576 0.95510

1222 2.35903 4.53300 3.13535 2.56895 3.74348 3.42754 3.64412 2.96230 2.17652 2.77447 3.39617 3.04143 3.82000 2.85045 2.65722 2.54934 2.79044 2.72265 5.08196 3.80560 1401 - -

2.68618 4.42225 2.77519 2.73123 3.46354 2.40513 3.72494 3.29354 2.67741 2.69355 4.24690 2.90347 2.73739 3.18146 2.89801 2.37887 2.77519 2.98518 4.58477 3.61503

0.03294 3.82522 4.54757 0.61958 0.77255 0.35919 1.19814

1223 2.79406 5.01822 2.96481 2.41291 4.36012 3.47868 3.66194 3.76464 1.92535 3.30502 4.13293 3.00829 1.96913 2.80482 2.42396 2.79754 3.02017 3.42274 5.46849 4.17283 1402 - -

2.68618 4.42225 2.77519 2.73123 3.46354 2.40513 3.72494 3.29354 2.67741 2.69355 4.24690 2.90347 2.73739 3.18146 2.89801 2.37887 2.77519 2.98518 4.58477 3.61503

0.02687 4.02581 4.74816 0.61958 0.77255 0.48576 0.95510

1224 2.50206 4.38849 3.33823 2.79379 3.55822 3.51004 3.77404 2.88976 2.52503 1.87850 3.49794 3.21884 3.49337 2.92983 3.02768 2.77278 2.71192 2.56380 4.97758 3.73500 1403 - -

2.68618 4.42225 2.77519 2.73123 3.46354 2.40513 3.72494 3.29354 2.67741 2.69355 4.24690 2.90347 2.73739 3.18146 2.89801 2.37887 2.77519 2.98518 4.58477 3.61503

0.02687 4.02581 4.74816 0.61958 0.77255 0.48576 0.95510

1225 2.62490 4.54639 3.20210 2.65887 3.74392 3.48900 3.70035 3.11709 2.35515 2.01745 3.65181 2.98389 3.88417 2.80180 2.72576 2.64192 2.58631 2.85737 5.08853 3.82737 1404 - -

2.68618 4.42225 2.77519 2.73123 3.46354 2.40513 3.72494 3.29354 2.67741 2.69355 4.24690 2.90347 2.73739 3.18146 2.89801 2.37887 2.77519 2.98518 4.58477 3.61503

0.02864 3.96319 4.68553 0.61958 0.77255 0.48576 0.95510

1226 2.25309 4.19673 3.33748 3.04818 4.20224 2.97242 4.13571 3.55671 3.07107 3.29608 4.15783 3.25434 3.69926 3.40174 3.38474 1.29963 2.11339 3.07804 5.56083 4.32018 1405 - -

2.68618 4.42225 2.77519 2.73123 3.46354 2.40513 3.72494 3.29354 2.67741 2.69355 4.24690 2.90347 2.73739 3.18146 2.89801 2.37887 2.77519 2.98518 4.58477 3.61503

0.02943 3.93619 4.65854 0.61958 0.77255 0.48576 0.95510

1227 2.30861 4.29110 3.10961 2.82870 4.19842 3.01139 4.00162 3.61442 2.90216 3.29714 4.13648 2.85588 3.70129 3.23845 3.25419 1.38076 2.37459 3.14144 5.52380 4.25567 1406 - -

2.68618 4.42225 2.77519 2.73123 3.46354 2.40513 3.72494 3.29354 2.67741 2.69355 4.24690 2.90347 2.73739 3.18146 2.89801 2.37887 2.77519 2.98518 4.58477 3.61503

0.03033 3.90654 4.62888 0.61958 0.77255 0.48576 0.95510

1228 2.53945 4.59728 2.98899 2.57175 4.07421 3.26924 3.71939 3.48531 2.44248 3.10292 3.94920 3.01657 3.78929 2.73587 2.55655 1.74284 2.68362 3.13396 5.33299 4.04538 1407 - -

2.68618 4.42225 2.77519 2.73123 3.46354 2.40513 3.72494 3.29354 2.67741 2.69355 4.24690 2.90347 2.73739 3.18146 2.89801 2.37887 2.77519 2.98518 4.58477 3.61503

0.03033 3.90654 4.62888 0.61958 0.77255 0.48576 0.95510

1229 2.47407 4.31299 3.92632 3.43059 3.39938 3.70877 4.25710 2.24143 3.28891 1.43414 3.28247 3.71902 4.16797 3.62178 3.55246 3.06398 2.80901 2.03306 5.12217 3.92371 1408 - -

2.68618 4.42225 2.77519 2.73123 3.46354 2.40513 3.72494 3.29354 2.67741 2.69355 4.24690 2.90347 2.73739 3.18146 2.89801 2.37887 2.77519 2.98518 4.58477 3.61503

0.03222 3.84707 4.56941 0.61958 0.77255 0.48576 0.95510

1230 1.05100 4.17994 3.43728 3.22777 4.02753 3.00509 4.25276 3.14927 3.24339 3.03202 4.06692 3.38366 3.74194 3.58696 3.49546 2.53060 2.80621 2.80809 5.45723 4.25473 1409 - -

2.68620 4.42227 2.77521 2.73125 3.46356 2.40515 3.72496 3.29356 2.67743 2.69357 4.24646 2.90348 2.73731 3.18148 2.89791 2.37889 2.77521 2.98520 4.58479 3.61505

0.07457 2.63304 * 0.78607 0.60813 0.00000 *

//

HMMER3/b [3.0 | March 2010]

NAME Xyloglucan fucosyltransferases

LENG 954

ALPH amino

RF no

CS no

MAP yes

DATE Mon Aug 8 15:41:28 2016

NSEQ 28

EFFN 1.356934

CKSUM 2760911016

STATS LOCAL MSV -12.9288 0.69570

STATS LOCAL VITERBI -13.9626 0.69570

STATS LOCAL FORWARD -6.7631 0.69570

HMM A C D E F G H I K L M N P Q R S T V W Y

m->m m->i m->d i->m i->i d->m d->d

COMPO 2.57106 3.93416 2.96604 2.71825 3.23182 2.97160 3.63259 2.91982 2.71170 2.36666 3.64502 3.15250 3.22002 3.09782 2.94161 2.60713 2.85022 2.67057 4.45230 3.32476

2.68610 4.41779 2.77683 2.73176 3.46479 2.40497 3.72462 3.29287 2.67792 2.69323 4.24628 2.90368 2.73767 3.18226 2.89676 2.37940 2.77520 2.98430 4.58549 3.61201

0.42738 2.01231 1.54128 4.01246 0.01825 0.00000 *

1 2.60761 4.26591 3.92337 3.36110 3.25123 3.78780 4.10763 1.93900 3.19946 2.00043 2.20428 3.67506 4.15194 3.51744 3.25115 3.08597 2.96675 2.19266 4.91407 3.72484 141 - -

2.68618 4.42225 2.77519 2.73123 3.46354 2.40513 3.72494 3.29354 2.67741 2.69355 4.24690 2.90347 2.73739 3.18146 2.89801 2.37887 2.77519 2.98518 4.58477 3.61503

0.03034 3.90622 4.62857 0.61958 0.77255 0.65956 0.72790

2 2.49279 4.92485 2.63932 1.92215 4.26916 2.54820 3.63241 3.70274 2.41419 3.26829 4.08372 2.71556 3.78178 2.78175 2.63067 2.64534 2.90633 3.33817 5.46899 4.11116 142 - -

2.68618 4.42225 2.77519 2.73123 3.46354 2.40513 3.72494 3.29354 2.67741 2.69355 4.24690 2.90347 2.73739 3.18146 2.89801 2.37887 2.77519 2.98518 4.58477 3.61503

0.05682 3.90622 3.34898 0.61958 0.77255 0.65956 0.72790

3 2.51972 4.55415 3.09827 2.56692 3.90762 2.69922 3.66886 3.29449 2.42980 2.93573 3.44564 3.02385 3.78757 2.85727 2.29858 2.44509 2.62788 2.98108 5.20264 3.91983 143 - -

2.68618 4.42225 2.77519 2.73123 3.46354 2.40513 3.72494 3.29354 2.67741 2.69355 4.24690 2.90347 2.73739 3.18146 2.89801 2.37887 2.77519 2.98518 4.58477 3.61503

0.05819 3.88055 3.32717 0.61958 0.77255 0.67948 0.70700

4 2.66138 5.02959 2.12164 2.06489 4.34368 3.08678 3.61417 3.79977 2.45715 3.34648 4.15347 2.75776 3.75553 2.75844 2.74044 2.19785 2.91885 3.41865 5.53835 4.14086 144 - -

2.68618 4.42225 2.77519 2.73123 3.46354 2.40513 3.72494 3.29354 2.67741 2.69355 4.24690 2.90347 2.73739 3.18146 2.89801 2.37887 2.77519 2.98518 4.58477 3.61503

0.03198 3.85433 4.57668 0.61958 0.77255 0.66093 0.72643

5 2.23961 4.19939 3.08879 2.91927 4.28320 1.42451 4.12032 3.75897 3.09939 3.44091 4.28507 3.16176 3.65103 3.39289 3.42198 1.98694 2.72605 3.20672 5.60613 4.36008 145 - -

2.68618 4.42225 2.77519 2.73123 3.46354 2.40513 3.72494 3.29354 2.67741 2.69355 4.24690 2.90347 2.73739 3.18146 2.89801 2.37887 2.77519 2.98518 4.58477 3.61503

0.10524 3.88055 2.53515 0.61958 0.77255 0.67948 0.70700

6 1.84573 4.39996 2.96224 2.59533 4.01341 2.91865 3.77661 3.40585 2.43309 3.06086 3.90281 3.00571 3.13724 2.98530 3.01108 2.37901 2.74229 3.02739 5.32388 4.04656 146 - -
2.68618 4.42225 2.77519 2.73123 3.46354 2.40513 3.72494 3.29354 2.67741 2.69355 4.24690 2.90347 2.73739 3.18146 2.89801 2.37887 2.77519 2.98518 4.58477 3.61503
0.03350 3.80881 4.53115 0.61958 0.77255 0.59743 0.79900
7 2.07942 4.40584 3.16058 2.66267 3.78495 2.70815 3.75237 2.86090 2.48436 2.83426 3.69410 3.08850 3.78590 2.98035 3.02500 2.49909 2.54057 2.83847 5.14710 3.87939 147 - -
2.68548 4.42241 2.77520 2.73139 3.46370 2.40497 3.72510 3.29370 2.67756 2.69358 4.24705 2.90362 2.73716 3.18154 2.89799 2.37902 2.77515 2.98528 4.58493 3.61519
0.55430 1.01054 2.78862 1.08921 0.41020 0.65956 0.72790
8 2.57660 4.40977 3.23779 2.77895 3.45255 2.16940 3.73899 3.06884 2.52900 2.73545 3.64097 3.17988 3.86128 3.05850 2.96426 2.73429 2.85313 2.80854 2.99201 3.53394 157 - -
2.68618 4.42225 2.77519 2.73123 3.46354 2.40513 3.72494 3.29354 2.67741 2.69355 4.24690 2.90347 2.73739 3.18146 2.89801 2.37887 2.77519 2.98518 4.58477 3.61503
0.03202 3.85310 4.57545 0.61958 0.77255 0.69981 0.68653
9 2.49519 4.75740 2.66895 2.28160 4.00065 3.24253 3.59198 3.40597 2.15233 3.01770 3.41181 2.90730 3.77518 2.74545 2.84175 2.59854 2.79804 2.79535 5.26440 3.93157 158 - -
2.68618 4.42225 2.77519 2.73123 3.46354 2.40513 3.72494 3.29354 2.67741 2.69355 4.24690 2.90347 2.73739 3.18146 2.89801 2.37887 2.77519 2.98518 4.58477 3.61503
0.07073 3.90666 3.03274 0.61958 0.77255 0.62338 0.76815
10 2.68214 5.06961 2.39920 1.75803 4.37995 3.08944 3.62867 3.83884 2.39474 3.37934 4.18228 2.77092 3.18833 2.76997 2.97368 2.36574 2.93753 3.45365 5.56801 4.16548 159 - -
2.68602 4.42216 2.77534 2.73129 3.46374 2.40526 3.72413 3.29375 2.67757 2.69325 4.24710 2.90362 2.73746 3.18060 2.89799 2.37880 2.77540 2.98533 4.58497 3.61524
0.43115 1.23385 2.82902 1.48370 0.25721 0.68961 0.69670
11 2.57288 4.67097 3.02253 2.47715 3.66704 3.39398 3.13226 3.32235 2.35755 2.94137 3.77834 2.96959 3.34775 2.77961 2.27980 2.54630 2.68614 3.01976 5.18405 3.87505 166 - -
2.68618 4.42225 2.77519 2.73123 3.46354 2.40513 3.72494 3.29354 2.67741 2.69355 4.24690 2.90347 2.73739 3.18146 2.89801 2.37887 2.77519 2.98518 4.58477 3.61503
0.05528 3.86917 3.41410 0.61958 0.77255 0.72749 0.65995
12 2.55145 4.43888 3.18392 2.65157 3.62550 2.98780 3.67815 3.02769 2.41184 2.70076 3.57369 3.09606 3.56521 2.92428 2.75140 2.59482 2.79362 2.76887 3.01594 3.72887 167 - -
2.68618 4.42225 2.77519 2.73123 3.46354 2.40513 3.72494 3.29354 2.67741 2.69355 4.24690 2.90347 2.73739 3.18146 2.89801 2.37887 2.77519 2.98518 4.58477 3.61503
0.03225 3.84614 4.56848 0.61958 0.77255 0.70612 0.68034
13 2.51744 4.24677 3.63425 3.07556 2.86563 3.62013 3.91938 2.40166 3.00497 1.99683 3.24823 3.43872 2.87791 3.29748 3.31968 2.89351 2.85303 2.25732 4.84534 3.60762 168 - -
2.68618 4.42225 2.77519 2.73123 3.46354 2.40513 3.72494 3.29354 2.67741 2.69355 4.24690 2.90347 2.73739 3.18146 2.89801 2.37887 2.77519 2.98518 4.58477 3.61503
0.03141 3.87223 4.59458 0.61958 0.77255 0.72529 0.66200
14 2.71899 4.99278 2.57324 1.62811 4.34325 2.87496 3.64396 3.77944 2.38513 3.33536 4.16915 2.83347 3.80078 2.79963 2.51492 2.70398 2.97812 3.41713 5.50907 4.16076 169 - -
2.68618 4.42225 2.77519 2.73123 3.46354 2.40513 3.72494 3.29354 2.67741 2.69355 4.24690 2.90347 2.73739 3.18146 2.89801 2.37887 2.77519 2.98518 4.58477 3.61503
0.03141 3.87223 4.59458 0.61958 0.77255 0.72529 0.66200
15 2.19364 4.23164 3.51704 2.95341 3.37165 3.54002 3.52309 2.48991 2.87952 2.40564 2.68931 3.33137 3.93171 3.18850 3.20728 2.80314 2.55336 2.38690 4.84613 3.62018 170 - -
2.68618 4.42225 2.77519 2.73123 3.46354 2.40513 3.72494 3.29354 2.67741 2.69355 4.24690 2.90347 2.73739 3.18146 2.89801 2.37887 2.77519 2.98518 4.58477 3.61503
0.02897 3.95173 4.67407 0.61958 0.77255 0.60184 0.79364
16 2.44573 5.04676 2.29450 2.08976 4.34730 3.18472 3.60275 3.80650 2.39484 3.18085 4.11596 2.82738 3.78110 2.38608 2.88863 2.46818 2.77967 3.41613 5.51398 4.11967 171 - -
2.68532 4.42238 2.77544 2.73101 3.46383 2.40529 3.72519 3.29395 2.67750 2.69361 4.24697 2.90382 2.73697 3.18171 2.89756 2.37885 2.77543 2.98529 4.58391 3.61544
0.61366 0.79850 4.75269 1.61470 0.22183 0.65956 0.72790
17 2.00945 3.96090 3.26401 2.70492 3.48728 2.87253 3.74197 2.99144 2.66804 2.68014 3.55166 3.15281 3.87090 2.90463 2.83426 2.70931 2.70596 2.66504 5.01630 3.59782 183 - -
2.68618 4.42225 2.77519 2.73123 3.46354 2.40513 3.72494 3.29354 2.67741 2.69355 4.24690 2.90347 2.73739 3.18146 2.89801 2.37887 2.77519 2.98518 4.58477 3.61503

0.19740 4.06089 1.82076 0.61958 0.77255 0.65956 0.72790

18 2.49163 4.67856 2.98458 2.38795 3.90924 3.01360 3.25741 3.31212 2.29304 2.93694 3.57765 2.95162 3.05609 2.78053 2.84989 2.61664 2.79370 2.81168 5.20118 3.88781 184 - -

2.68618 4.42225 2.77519 2.73123 3.46354 2.40513 3.72494 3.29354 2.67741 2.69355 4.24690 2.90347 2.73739 3.18146 2.89801 2.37887 2.77519 2.98518 4.58477 3.61503

0.03003 3.91624 4.63858 0.61958 0.77255 0.79257 0.60272

19 1.93934 4.56526 2.53634 2.48323 4.17269 2.44270 3.76700 3.59069 2.64866 3.21364 4.03411 2.94538 3.19060 2.94678 3.10203 2.53008 2.67396 3.18644 5.45357 4.13241 185 - -

2.68588 4.42230 2.77524 2.73123 3.46309 2.40517 3.72499 3.29359 2.67741 2.69360 4.24694 2.90351 2.73744 3.18151 2.89805 2.37891 2.77519 2.98523 4.58482 3.61508

0.37320 1.38570 2.79157 0.48892 0.95008 0.60719 0.78719

20 2.03506 4.61357 2.90404 2.50865 3.90149 2.96893 3.67365 3.29708 2.51483 2.84440 3.57053 2.99714 3.01596 2.78512 2.95373 2.62008 2.80017 2.98968 5.21790 3.91271 188 - -

2.68618 4.42225 2.77519 2.73123 3.46354 2.40513 3.72494 3.29354 2.67741 2.69355 4.24690 2.90347 2.73739 3.18146 2.89801 2.37887 2.77519 2.98518 4.58477 3.61503

0.08967 3.99292 2.69825 0.61958 0.77255 0.73500 0.65298

21 2.36964 4.82687 2.79992 2.22745 4.08538 3.23374 3.58649 3.51065 2.24101 3.09551 3.58340 2.89356 3.07522 2.65894 2.83578 2.59043 2.80550 3.05233 5.32051 3.97299 189 - -

2.68618 4.42225 2.77519 2.73123 3.46354 2.40513 3.72494 3.29354 2.67741 2.69355 4.24690 2.90347 2.73739 3.18146 2.89801 2.37887 2.77519 2.98518 4.58477 3.61503

0.02852 3.96726 4.68961 0.61958 0.77255 0.62890 0.76181

22 2.41185 4.79310 2.70264 2.41505 3.85319 3.40627 3.61342 3.45566 2.30136 3.05543 3.57358 2.93584 3.36586 2.76140 2.79961 2.17473 2.81376 3.12884 5.29551 3.75351 190 - -

2.68621 4.42234 2.77517 2.73132 3.46351 2.40522 3.72490 3.29356 2.67724 2.69359 4.24699 2.90356 2.73710 3.18149 2.89794 2.37892 2.77517 2.98527 4.58486 3.61499

0.43749 1.06133 4.78540 0.56127 0.84509 0.70268 0.68370

23 2.40798 4.82201 2.93845 2.28434 4.08855 3.39694 3.46006 3.51023 2.23377 3.10109 3.90861 2.92980 3.04570 2.76305 2.85943 2.42059 2.49609 3.04828 5.33117 3.99060 193 - -

2.68618 4.42225 2.77519 2.73123 3.46354 2.40513 3.72494 3.29354 2.67741 2.69355 4.24690 2.90347 2.73739 3.18146 2.89801 2.37887 2.77519 2.98518 4.58477 3.61503

0.02588 4.06305 4.78540 0.61958 0.77255 0.70268 0.68370

24 2.51097 4.59649 3.10791 2.41332 3.79615 3.00102 3.67141 3.18562 2.52156 2.63139 3.03588 3.04056 3.34069 2.86931 2.68107 2.52486 2.81033 2.82722 5.13642 3.84342 194 - -

2.68618 4.42225 2.77519 2.73123 3.46354 2.40513 3.72494 3.29354 2.67741 2.69355 4.24690 2.90347 2.73739 3.18146 2.89801 2.37887 2.77519 2.98518 4.58477 3.61503

0.04916 4.06305 3.48103 0.61958 0.77255 0.70268 0.68370

25 2.46979 4.88564 2.92443 2.33953 4.19021 3.11310 3.60795 3.61755 2.22755 3.18278 3.98237 2.92066 2.89001 2.74289 2.31448 2.51952 2.76873 3.26346 5.38467 4.04251 195 - -

2.68618 4.42225 2.77519 2.73123 3.46354 2.40513 3.72494 3.29354 2.67741 2.69355 4.24690 2.90347 2.73739 3.18146 2.89801 2.37887 2.77519 2.98518 4.58477 3.61503

0.02648 4.04037 4.76272 0.61958 0.77255 0.68295 0.70345

26 2.51360 4.24182 3.06037 2.37044 3.88534 3.43844 3.64541 3.19156 2.35688 2.91846 3.57986 3.00106 3.33975 2.82207 2.32707 2.47845 2.81394 2.99044 4.51175 3.88648 196 - -

2.68618 4.42225 2.77519 2.73123 3.46354 2.40513 3.72494 3.29354 2.67741 2.69355 4.24690 2.90347 2.73739 3.18146 2.89801 2.37887 2.77519 2.98518 4.58477 3.61503

0.07439 4.06305 2.90965 0.61958 0.77255 0.70268 0.68370

27 2.52199 4.65579 3.06670 2.43794 3.86918 3.28280 3.63962 3.26450 2.32413 2.54554 3.46231 3.00300 3.28687 2.81948 2.50412 2.57780 2.81142 2.90202 5.17751 3.87582 197 - -

2.68618 4.42225 2.77519 2.73123 3.46354 2.40513 3.72494 3.29354 2.67741 2.69355 4.24690 2.90347 2.73739 3.18146 2.89801 2.37887 2.77519 2.98518 4.58477 3.61503

0.05217 4.01581 3.41707 0.61958 0.77255 0.74290 0.64575

28 2.55551 4.36780 3.31125 2.75081 3.53524 2.96049 3.74086 2.90536 2.50894 2.60183 3.48256 3.18314 3.87900 3.02101 2.93528 2.56443 2.68820 2.35250 3.35826 3.69665 198 - -

2.68618 4.42225 2.77519 2.73123 3.46354 2.40513 3.72494 3.29354 2.67741 2.69355 4.24690 2.90347 2.73739 3.18146 2.89801 2.37887 2.77519 2.98518 4.58477 3.61503

0.02783 3.99146 4.71381 0.61958 0.77255 0.76231 0.62846

29 2.58720 4.54141 3.18172 2.62212 3.72061 3.45754 3.68156 3.00069 2.49702 2.79005 3.65445 3.08717 3.85532 2.90159 2.29630 2.38040 2.62818 2.85928 5.07562 3.01559 199 - -

2.68618 4.42225 2.77519 2.73123 3.46354 2.40513 3.72494 3.29354 2.67741 2.69355 4.24690 2.90347 2.73739 3.18146 2.89801 2.37887 2.77519 2.98518 4.58477 3.61503
0.02783 3.99146 4.71381 0.61958 0.77255 0.76231 0.62846

30 2.53726 4.02544 3.31045 2.75002 3.10920 3.01341 3.74781 2.89449 2.62938 2.54132 3.24145 3.18172 3.45970 3.03066 3.09407 2.21850 2.77922 2.65145 4.94414 3.69299 200 - -
2.68618 4.42225 2.77519 2.73123 3.46354 2.40513 3.72494 3.29354 2.67741 2.69355 4.24690 2.90347 2.73739 3.18146 2.89801 2.37887 2.77519 2.98518 4.58477 3.61503
0.02783 3.99146 4.71381 0.61958 0.77255 0.34504 1.23165

31 2.10558 4.14028 3.42557 2.86265 3.56928 2.60224 3.83584 2.92779 2.65437 2.63621 2.63022 3.28380 3.95503 3.13285 3.03204 2.80585 2.85400 2.47163 4.99493 3.75094 201 - -
2.68616 4.42254 2.77548 2.73147 3.46383 2.40542 3.72514 3.29278 2.67695 2.69349 4.24664 2.90376 2.73763 3.18175 2.89784 2.37903 2.77535 2.98371 4.58466 3.61532
0.57354 0.90546 3.43850 1.18316 0.36573 0.48576 0.95510

32 2.03758 4.48296 3.31564 2.75129 3.64501 3.54954 3.78330 3.01766 2.63926 2.59331 3.43312 3.20548 3.67059 2.97269 2.73524 2.77969 2.32670 2.57773 4.35567 3.78512 210 - -
2.68618 4.42225 2.77519 2.73123 3.46354 2.40513 3.72494 3.29354 2.67741 2.69355 4.24690 2.90347 2.73739 3.18146 2.89801 2.37887 2.77519 2.98518 4.58477 3.61503
0.32238 4.26287 1.34134 0.61958 0.77255 0.51923 0.90381

33 2.57552 4.71357 2.97680 2.44754 3.95667 3.39517 3.62139 3.36560 2.35417 2.98342 3.81062 2.43249 3.09522 2.78667 2.73340 2.49014 2.81181 2.77652 5.23757 3.66882 211 - -
2.68618 4.42225 2.77519 2.73123 3.46354 2.40513 3.72494 3.29354 2.67741 2.69355 4.24690 2.90347 2.73739 3.18146 2.89801 2.37887 2.77519 2.98518 4.58477 3.61503
0.02847 3.96896 4.69130 0.61958 0.77255 0.80612 0.59165

34 2.03130 4.46839 3.19182 2.64068 3.69991 3.26163 3.47259 2.94993 2.57122 2.75023 3.61449 3.09608 3.83653 2.93460 2.68216 2.67027 2.61817 2.63087 5.06827 3.79864 212 - -
2.68618 4.42225 2.77519 2.73123 3.46354 2.40513 3.72494 3.29354 2.67741 2.69355 4.24690 2.90347 2.73739 3.18146 2.89801 2.37887 2.77519 2.98518 4.58477 3.61503
0.02847 3.96896 4.69130 0.61958 0.77255 0.75228 0.63732

35 2.14630 4.24710 3.86975 3.31468 3.41226 3.73024 4.12246 2.26649 3.22842 1.97680 3.12748 3.63542 4.13026 3.51847 3.51663 3.02807 2.67782 1.80357 5.00434 3.79696 213 - -
2.68618 4.42225 2.77519 2.73123 3.46354 2.40513 3.72494 3.29354 2.67741 2.69355 4.24690 2.90347 2.73739 3.18146 2.89801 2.37887 2.77519 2.98518 4.58477 3.61503
0.02752 4.00224 4.72459 0.61958 0.77255 0.35437 1.20938

36 2.32592 3.82828 4.24374 3.68186 3.38639 3.81065 4.26009 2.31954 3.56230 1.61787 3.35051 3.87086 4.22460 3.79690 3.75238 3.02572 2.86748 1.64057 4.95586 3.76298 214 - -
2.68618 4.42225 2.77519 2.73123 3.46354 2.40513 3.72494 3.29354 2.67741 2.69355 4.24690 2.90347 2.73739 3.18146 2.89801 2.37887 2.77519 2.98518 4.58477 3.61503
0.02086 4.27605 4.99840 0.61958 0.77255 0.50177 0.93004

37 2.46071 4.17182 4.17902 3.60642 2.87581 3.45397 4.16927 2.37165 3.49066 1.92706 3.28233 3.81612 4.19344 3.71913 3.67984 3.11478 2.83906 1.49911 4.85400 3.65819 215 - -
2.68618 4.42225 2.77519 2.73123 3.46354 2.40513 3.72494 3.29354 2.67741 2.69355 4.24690 2.90347 2.73739 3.18146 2.89801 2.37887 2.77519 2.98518 4.58477 3.61503
0.02086 4.27605 4.99840 0.61958 0.77255 0.50177 0.93004

38 2.06876 4.24701 3.81354 3.25303 3.44425 3.49542 4.03989 2.43334 3.17915 2.48426 3.25550 3.56497 4.07147 3.45645 3.46765 2.87663 2.21184 1.81791 4.94105 3.72842 216 - -
2.68618 4.42225 2.77519 2.73123 3.46354 2.40513 3.72494 3.29354 2.67741 2.69355 4.24690 2.90347 2.73739 3.18146 2.89801 2.37887 2.77519 2.98518 4.58477 3.61503
0.02086 4.27605 4.99840 0.61958 0.77255 0.50177 0.93004

39 2.40479 2.77239 4.20323 3.61462 2.48424 3.20555 4.06897 2.41890 3.47728 2.15916 3.19765 3.77318 4.11275 3.68007 3.62218 2.95198 2.77485 1.94174 4.70385 3.51591 217 - -
2.68618 4.42225 2.77519 2.73123 3.46354 2.40513 3.72494 3.29354 2.67741 2.69355 4.24690 2.90347 2.73739 3.18146 2.89801 2.37887 2.77519 2.98518 4.58477 3.61503
0.03367 4.27605 3.95214 0.61958 0.77255 0.50177 0.93004

40 2.92953 2.47259 4.73181 4.15316 3.10846 4.16801 4.53667 1.82497 4.00398 1.52713 3.21377 4.26487 4.48667 4.16286 4.09464 3.49282 3.16587 1.75890 5.02520 3.86025 218 - -
2.68618 4.42225 2.77519 2.73123 3.46354 2.40513 3.72494 3.29354 2.67741 2.69355 4.24690 2.90347 2.73739 3.18146 2.89801 2.37887 2.77519 2.98518 4.58477 3.61503
0.02113 4.26351 4.98585 0.61958 0.77255 0.51840 0.90504

41 2.62782 4.15351 4.30350 3.71534 2.15438 3.85220 4.16729 2.42793 3.57646 1.84608 2.26984 3.88119 4.20673 3.76970 3.71669 3.01042 2.83389 2.14645 4.76642 3.58453 219 - -
2.68618 4.42225 2.77519 2.73123 3.46354 2.40513 3.72494 3.29354 2.67741 2.69355 4.24690 2.90347 2.73739 3.18146 2.89801 2.37887 2.77519 2.98518 4.58477 3.61503
0.02113 4.26351 4.98585 0.61958 0.77255 0.51840 0.90504
42 2.52810 4.18121 4.15174 3.57293 3.29237 3.83528 4.15783 2.15319 3.46241 1.77343 3.04314 3.80362 3.87266 3.69354 3.66209 3.12642 2.38882 1.83864 4.85302 3.66170 220 - -
2.68618 4.42225 2.77519 2.73123 3.46354 2.40513 3.72494 3.29354 2.67741 2.69355 4.24690 2.90347 2.73739 3.18146 2.89801 2.37887 2.77519 2.98518 4.58477 3.61503
0.03941 4.26351 3.70627 0.61958 0.77255 0.51840 0.90504
43 2.04892 4.15355 4.14283 3.55938 3.22806 3.80109 4.11217 2.36075 3.44136 1.83823 2.83811 3.77794 4.16199 3.66355 3.62783 3.09077 2.80511 1.87779 4.43290 3.60122 221 - -
2.68618 4.42225 2.77519 2.73123 3.46354 2.40513 3.72494 3.29354 2.67741 2.69355 4.24690 2.90347 2.73739 3.18146 2.89801 2.37887 2.77519 2.98518 4.58477 3.61503
0.25524 4.24561 1.55619 0.61958 0.77255 0.54131 0.87224
44 2.47968 4.27259 3.51274 2.95827 3.45363 3.53156 3.87008 2.44784 2.89554 2.18255 3.40878 3.33944 2.72198 3.20649 3.23521 2.57620 2.81692 2.28042 4.92666 3.69556 222 - -
2.68618 4.42225 2.77519 2.73123 3.46354 2.40513 3.72494 3.29354 2.67741 2.69355 4.24690 2.90347 2.73739 3.18146 2.89801 2.37887 2.77519 2.98518 4.58477 3.61503
0.02710 4.01748 4.73983 0.61958 0.77255 0.76915 0.62251
45 2.56514 4.37555 3.51222 3.06349 3.66796 3.40415 4.04248 2.77755 2.96378 2.42612 3.64383 3.39569 1.77016 3.33679 3.28518 2.77596 2.90825 2.20280 5.20462 3.93022 223 - -
2.68618 4.42225 2.77519 2.73123 3.46354 2.40513 3.72494 3.29354 2.67741 2.69355 4.24690 2.90347 2.73739 3.18146 2.89801 2.37887 2.77519 2.98518 4.58477 3.61503
0.10346 4.01748 2.52207 0.61958 0.77255 0.36796 1.17812
46 2.43628 4.21771 3.75403 3.18114 3.19490 3.66236 3.94724 2.56625 3.10110 1.95073 2.55438 3.51074 4.03904 3.37632 3.27823 2.23624 2.85982 2.44691 4.82129 3.61101 224 - -
2.68618 4.42225 2.77519 2.73123 3.46354 2.40513 3.72494 3.29354 2.67741 2.69355 4.24690 2.90347 2.73739 3.18146 2.89801 2.37887 2.77519 2.98518 4.58477 3.61503
0.02200 4.22354 4.94589 0.61958 0.77255 0.46480 0.98957
47 2.94010 4.31983 4.60447 4.03362 2.67393 4.14479 4.47790 2.13641 3.89786 1.61858 3.23299 4.19266 4.46973 4.08049 4.02942 3.27626 3.17725 1.33205 4.99864 3.79165 225 - -
2.68618 4.42225 2.77519 2.73123 3.46354 2.40513 3.72494 3.29354 2.67741 2.69355 4.24690 2.90347 2.73739 3.18146 2.89801 2.37887 2.77519 2.98518 4.58477 3.61503
0.03865 4.28014 3.72677 0.61958 0.77255 0.47125 0.97876
48 2.47475 4.12287 4.32379 3.73415 2.96370 3.84496 4.16934 2.20116 3.59303 1.96618 2.26669 3.88523 4.20339 3.78737 3.72583 2.96192 2.82377 1.81537 4.77273 3.59026 226 - -
2.68618 4.42225 2.77519 2.73123 3.46354 2.40513 3.72494 3.29354 2.67741 2.69355 4.24690 2.90347 2.73739 3.18146 2.89801 2.37887 2.77519 2.98518 4.58477 3.61503
0.09085 4.28881 2.61558 0.61958 0.77255 0.47132 0.97864
49 2.57270 4.42370 3.71125 3.14976 3.43401 3.76327 4.03046 2.43546 3.03344 1.53094 3.34886 3.53591 4.13179 2.41960 3.34780 3.03176 2.99891 2.43290 5.01672 3.80808 227 - -
2.68618 4.42225 2.77519 2.73123 3.46354 2.40513 3.72494 3.29354 2.67741 2.69355 4.24690 2.90347 2.73739 3.18146 2.89801 2.37887 2.77519 2.98518 4.58477 3.61503
0.04845 4.23830 3.41550 0.61958 0.77255 0.57380 0.82869
50 2.68989 4.25390 4.33198 3.75547 2.83090 3.95066 4.19969 2.40973 3.62549 1.36043 3.14428 3.94306 4.28795 3.81637 3.78139 3.25085 3.05202 1.98400 4.75127 2.78122 228 - -
2.68618 4.42225 2.77519 2.73123 3.46354 2.40513 3.72494 3.29354 2.67741 2.69355 4.24690 2.90347 2.73739 3.18146 2.89801 2.37887 2.77519 2.98518 4.58477 3.61503
0.02226 4.21211 4.93445 0.61958 0.77255 0.51217 0.91427
51 2.37285 4.71907 2.99219 2.55109 3.99278 2.80404 3.71950 3.39562 2.54920 2.81362 3.85598 3.04856 3.86538 2.66733 2.99735 1.96891 2.80291 2.78070 5.29573 3.98268 229 - -
2.68618 4.42225 2.77519 2.73123 3.46354 2.40513 3.72494 3.29354 2.67741 2.69355 4.24690 2.90347 2.73739 3.18146 2.89801 2.37887 2.77519 2.98518 4.58477 3.61503
0.29730 4.25746 1.41460 0.61958 0.77255 0.44748 1.01954
52 2.24064 4.29422 3.42425 2.85794 2.87165 3.09221 3.78970 2.79587 2.80859 2.40997 3.25652 3.26690 3.91614 3.11625 2.86510 2.60383 2.79471 2.49937 4.87563 3.45876 230 - -
2.68618 4.42225 2.77519 2.73123 3.46354 2.40513 3.72494 3.29354 2.67741 2.69355 4.24690 2.90347 2.73739 3.18146 2.89801 2.37887 2.77519 2.98518 4.58477 3.61503

0.02620 4.05099 4.77334 0.61958 0.77255 0.38276 1.14562

53 2.65873 4.24026 3.79372 3.22051 2.11097 3.02955 3.92031 2.70885 3.12657 2.27738 3.34752 3.53558 4.06566 3.40297 2.51911 2.96001 2.89013 2.43333 3.72806 3.31474 231 - -

2.68618 4.42225 2.77519 2.73123 3.46354 2.40513 3.72494 3.29354 2.67741 2.69355 4.24690 2.90347 2.73739 3.18146 2.89801 2.37887 2.77519 2.98518 4.58477 3.61503

0.02075 4.28141 5.00376 0.61958 0.77255 0.46539 0.98857

54 2.48581 4.40964 3.42863 2.86576 2.46102 3.04740 3.83909 2.84842 2.82421 2.63261 3.51740 3.29183 3.96977 2.86449 2.96893 2.29275 2.86561 2.41687 4.99020 3.74503 232 - -

2.68618 4.42225 2.77519 2.73123 3.46354 2.40513 3.72494 3.29354 2.67741 2.69355 4.24690 2.90347 2.73739 3.18146 2.89801 2.37887 2.77519 2.98518 4.58477 3.61503

0.15863 4.30587 2.01589 0.61958 0.77255 0.48576 0.95510

55 2.27341 4.56499 3.17808 2.62055 3.61637 2.97558 3.71932 3.13655 2.59990 2.59367 3.50273 2.32550 3.64408 2.93529 3.02478 2.70923 2.83235 2.74769 5.11789 3.63480 233 - -

2.68618 4.42225 2.77519 2.73123 3.46354 2.40513 3.72494 3.29354 2.67741 2.69355 4.24690 2.90347 2.73739 3.18146 2.89801 2.37887 2.77519 2.98518 4.58477 3.61503

0.09763 4.17045 2.55655 0.61958 0.77255 0.64991 0.73834

56 2.50792 4.48969 2.86887 2.66657 3.70368 2.57208 3.75969 2.24550 2.67907 2.63324 3.62988 3.03590 3.87920 3.00193 3.08851 2.63548 2.83331 2.79028 5.09259 3.82077 234 - -

2.68618 4.42225 2.77519 2.73123 3.46354 2.40513 3.72494 3.29354 2.67741 2.69355 4.24690 2.90347 2.73739 3.18146 2.89801 2.37887 2.77519 2.98518 4.58477 3.61503

0.02498 4.09780 4.82015 0.61958 0.77255 0.41405 1.08166

57 2.63505 4.59189 3.22300 2.66933 2.52081 2.95751 3.75628 3.17367 2.62462 2.76039 3.69801 3.14326 3.68724 2.88621 2.42606 2.29820 2.87163 2.90568 5.14534 3.86282 235 - -

2.68618 4.42225 2.77519 2.73123 3.46354 2.40513 3.72494 3.29354 2.67741 2.69355 4.24690 2.90347 2.73739 3.18146 2.89801 2.37887 2.77519 2.98518 4.58477 3.61503

0.02090 4.27416 4.99651 0.61958 0.77255 0.52879 0.88994

58 2.46793 4.91157 2.80097 2.37209 3.91326 3.32483 3.50817 3.60109 2.40362 3.17960 3.76669 2.33827 3.84329 2.78641 2.74885 2.27140 2.86858 3.25555 3.65080 4.04651 236 - -

2.68618 4.42225 2.77519 2.73123 3.46354 2.40513 3.72494 3.29354 2.67741 2.69355 4.24690 2.90347 2.73739 3.18146 2.89801 2.37887 2.77519 2.98518 4.58477 3.61503

0.34500 4.29060 1.27983 0.61958 0.77255 0.52879 0.88994

59 2.30259 4.59488 2.96046 2.52932 4.04858 3.07566 3.72818 3.45600 2.46302 3.08261 3.91313 2.99595 2.32385 2.79385 2.97338 2.46844 2.57845 3.10397 5.33715 4.03381 237 - -

2.68613 4.42234 2.77523 2.73125 3.46351 2.40522 3.72504 3.29363 2.67750 2.69359 4.24675 2.90356 2.73735 3.18146 2.89810 2.37851 2.77511 2.98527 4.58486 3.61512

0.31344 1.34729 4.69627 0.86196 0.54876 0.33161 1.26503

60 2.16865 4.85342 2.90241 2.47711 4.09513 3.20542 3.57654 3.51369 2.42142 3.11380 3.92426 2.80938 3.39735 2.75695 2.83971 2.54603 2.75620 2.96621 5.35469 2.85998 244 - -

2.68618 4.42225 2.77519 2.73123 3.46354 2.40513 3.72494 3.29354 2.67741 2.69355 4.24690 2.90347 2.73739 3.18146 2.89801 2.37887 2.77519 2.98518 4.58477 3.61503

0.03818 4.31046 3.72844 0.61958 0.77255 0.50177 0.93004

61 2.64240 4.89479 2.87418 2.31460 4.14843 3.09508 3.51349 3.57388 2.40694 3.02891 3.76767 2.97551 3.45083 2.20373 2.91220 2.66204 2.87378 2.41213 5.38617 4.03926 245 - -

2.68618 4.42225 2.77519 2.73123 3.46354 2.40513 3.72494 3.29354 2.67741 2.69355 4.24690 2.90347 2.73739 3.18146 2.89801 2.37887 2.77519 2.98518 4.58477 3.61503

0.02051 4.29280 5.01514 0.61958 0.77255 0.52586 0.89416

62 2.63052 4.23376 3.72923 3.15483 3.08288 3.66126 3.68207 2.70649 3.07119 1.94745 3.34256 3.32029 3.80316 3.06261 3.22643 2.92404 2.75868 2.40916 2.53061 3.59410 246 - -

2.68618 4.42225 2.77519 2.73123 3.46354 2.40513 3.72494 3.29354 2.67741 2.69355 4.24690 2.90347 2.73739 3.18146 2.89801 2.37887 2.77519 2.98518 4.58477 3.61503

0.08695 4.29280 2.66489 0.61958 0.77255 0.52586 0.89416

63 2.57513 4.14738 4.04043 3.45945 3.24694 3.52200 4.05554 1.91437 3.34138 1.70759 3.08161 3.70087 4.11832 3.30515 3.54840 2.93474 2.88789 2.19153 4.77649 3.58288 247 - -

2.68618 4.42225 2.77519 2.73123 3.46354 2.40513 3.72494 3.29354 2.67741 2.69355 4.24690 2.90347 2.73739 3.18146 2.89801 2.37887 2.77519 2.98518 4.58477 3.61503

0.05654 4.24810 3.20200 0.61958 0.77255 0.56713 0.83737

64 2.52589 5.15394 2.09892 2.17984 4.46918 2.90744 3.65520 3.94076 2.32545 3.45102 4.21948 2.86974 3.83401 2.69108 2.94560 2.11648 2.94221 3.42459 5.61180 4.20431 248 - -

2.68618 4.42225 2.77519 2.73123 3.46354 2.40513 3.72494 3.29354 2.67741 2.69355 4.24690 2.90347 2.73739 3.18146 2.89801 2.37887 2.77519 2.98518 4.58477 3.61503
0.06270 4.23288 3.07348 0.61958 0.77255 0.62425 0.76715
65 1.30852 4.25938 3.60793 3.23840 4.38132 2.48587 4.28572 3.79744 3.26257 3.47289 4.28507 3.39784 3.78383 3.54323 3.59620 1.71106 2.42990 3.25204 5.71727 4.50625 249 - -
2.68618 4.42225 2.77519 2.73123 3.46354 2.40513 3.72494 3.29354 2.67741 2.69355 4.24690 2.90347 2.73739 3.18146 2.89801 2.37887 2.77519 2.98518 4.58477 3.61503
0.02269 4.19288 4.91523 0.61958 0.77255 0.66798 0.71896
66 2.09881 4.65750 3.09719 2.47443 4.00000 3.22639 3.74346 3.40290 2.57666 3.03690 3.86817 2.95568 3.84822 2.92354 2.89135 2.40829 2.02756 2.97271 5.30601 4.00131 250 - -
2.68618 4.42225 2.77519 2.73123 3.46354 2.40513 3.72494 3.29354 2.67741 2.69355 4.24690 2.90347 2.73739 3.18146 2.89801 2.37887 2.77519 2.98518 4.58477 3.61503
0.02269 4.19288 4.91523 0.61958 0.77255 0.50028 0.93233
67 2.54923 4.99676 2.69846 2.39211 4.28518 3.43392 3.21745 3.63016 2.28140 3.14964 4.05351 2.55516 3.63813 2.74753 2.63063 2.59373 2.31461 3.06182 5.46508 4.09220 251 - -
2.68618 4.42225 2.77519 2.73123 3.46354 2.40513 3.72494 3.29354 2.67741 2.69355 4.24690 2.90347 2.73739 3.18146 2.89801 2.37887 2.77519 2.98518 4.58477 3.61503
0.25521 4.27841 1.55407 0.61958 0.77255 0.47112 0.97897
68 2.59000 4.45724 3.26866 2.70609 3.44956 3.51035 3.74339 2.82531 2.60565 2.32106 3.55230 2.36839 3.89512 2.99664 2.86089 2.74199 2.65182 2.56180 5.01988 3.75681 252 - -
2.68618 4.42225 2.77519 2.73123 3.46354 2.40513 3.72494 3.29354 2.67741 2.69355 4.24690 2.90347 2.73739 3.18146 2.89801 2.37887 2.77519 2.98518 4.58477 3.61503
0.11603 4.10626 2.37431 0.61958 0.77255 0.34958 1.22074
69 2.34607 4.12542 3.17207 2.89165 3.13723 2.63277 3.84397 2.79777 2.85096 2.59432 3.34532 3.30744 3.96707 3.15792 3.21677 2.50315 2.72421 2.22094 4.95958 3.71962 253 - -
2.68627 4.42239 2.77522 2.73104 3.46358 2.40520 3.72509 3.29343 2.67755 2.69358 4.24656 2.90355 2.73717 3.18142 2.89806 2.37892 2.77506 2.98509 4.58491 3.61517
0.43734 1.86642 1.61159 1.29160 0.32135 0.57939 0.82152
70 2.33721 4.39102 3.31041 2.74881 3.40072 2.83315 3.75613 2.92609 2.71164 2.38613 3.49986 3.18907 3.35502 3.03298 2.98654 2.44764 2.80358 2.68361 4.00760 3.71304 262 - -
2.68618 4.42225 2.77519 2.73123 3.46354 2.40513 3.72494 3.29354 2.67741 2.69355 4.24690 2.90347 2.73739 3.18146 2.89801 2.37887 2.77519 2.98518 4.58477 3.61503
0.07679 4.07899 2.86481 0.61958 0.77255 0.64544 0.74324
71 2.34809 4.33098 3.41217 2.84647 3.32778 2.94045 3.79869 2.75777 2.80242 2.47800 3.11630 3.26370 3.92583 3.11199 2.85344 2.53613 2.72145 2.31187 4.91536 3.67530 263 - -
2.68618 4.42225 2.77519 2.73123 3.46354 2.40513 3.72494 3.29354 2.67741 2.69355 4.24690 2.90347 2.73739 3.18146 2.89801 2.37887 2.77519 2.98518 4.58477 3.61503
0.13201 4.11692 2.23145 0.61958 0.77255 0.76104 0.62957
72 2.61525 4.97524 2.57545 2.32450 4.28854 3.20549 3.59042 3.73653 2.34343 3.27464 4.05726 2.74038 3.54080 2.61743 2.16986 2.45379 2.71111 3.35566 5.45475 4.08243 264 - -
2.68618 4.42225 2.77519 2.73123 3.46354 2.40513 3.72494 3.29354 2.67741 2.69355 4.24690 2.90347 2.73739 3.18146 2.89801 2.37887 2.77519 2.98518 4.58477 3.61503
0.02725 4.01216 4.73451 0.61958 0.77255 0.84544 0.56101
73 2.00093 4.27192 3.36554 2.99159 4.02390 1.53282 4.05350 3.31157 2.97881 3.06918 3.44061 3.26647 3.77352 3.31660 3.31458 2.52681 2.77178 2.93808 5.41364 4.16871 265 - -
2.68618 4.42225 2.77519 2.73123 3.46354 2.40513 3.72494 3.29354 2.67741 2.69355 4.24690 2.90347 2.73739 3.18146 2.89801 2.37887 2.77519 2.98518 4.58477 3.61503
0.02725 4.01216 4.73451 0.61958 0.77255 0.66472 0.72240
74 2.58726 5.03580 2.38300 2.26793 4.33382 2.43102 3.63991 3.78596 2.37811 3.15840 4.11771 2.86486 3.81204 2.64186 2.93039 2.21196 2.90779 3.40657 5.51949 4.13377 266 - -
2.68609 4.42290 2.77428 2.73131 3.46388 2.40548 3.72514 3.29359 2.67770 2.69381 4.24689 2.90354 2.73747 3.18129 2.89814 2.37832 2.77526 2.98469 4.58542 3.61568
0.77004 0.67767 3.53336 1.91800 0.15888 0.75656 0.63352
75 2.42904 4.73413 2.46139 2.46545 3.96481 3.25324 3.42401 3.37219 2.47004 2.46635 3.82098 2.97711 3.82385 2.81581 2.83788 2.45886 2.82969 2.87536 5.25713 3.93508 283 - -
2.68618 4.42225 2.77519 2.73123 3.46354 2.40513 3.72494 3.29354 2.67741 2.69355 4.24690 2.90347 2.73739 3.18146 2.89801 2.37887 2.77519 2.98518 4.58477 3.61503
0.10928 4.10040 2.44232 0.61958 0.77255 0.64532 0.74337

76 2.51127 4.59723 2.93431 2.48965 3.45669 3.37089 3.42631 3.17957 2.54453 2.30437 3.68407 3.04802 3.44469 2.80564 2.97914 2.58862 2.51047 2.90324 5.13566 3.84253 284 - -
2.68618 4.42225 2.77519 2.73123 3.46354 2.40513 3.72494 3.29354 2.67741 2.69355 4.24690 2.90347 2.73739 3.18146 2.89801 2.37887 2.77519 2.98518 4.58477 3.61503
0.03760 4.09602 3.89900 0.61958 0.77255 0.41032 1.08897
77 2.49353 4.45124 3.27443 2.80363 3.60455 3.40123 3.81140 2.73909 2.77105 2.39436 3.40823 3.24721 3.22759 2.85902 3.16047 2.20602 2.65317 2.58706 4.51430 3.57342 285 - -
2.68618 4.42225 2.77519 2.73123 3.46354 2.40513 3.72494 3.29354 2.67741 2.69355 4.24690 2.90347 2.73739 3.18146 2.89801 2.37887 2.77519 2.98518 4.58477 3.61503
0.02033 4.30154 5.02389 0.61958 0.77255 0.44024 1.03250
78 2.25626 5.10121 2.81393 2.26202 4.41818 3.27877 3.07623 3.88420 1.96717 3.39427 4.15058 2.85190 3.21416 2.74218 2.82276 2.58125 2.70347 3.33822 5.55125 4.15850 286 - -
2.68618 4.42225 2.77519 2.73123 3.46354 2.40513 3.72494 3.29354 2.67741 2.69355 4.24690 2.90347 2.73739 3.18146 2.89801 2.37887 2.77519 2.98518 4.58477 3.61503
0.01922 4.35734 5.07968 0.61958 0.77255 0.48576 0.95510
79 2.54696 5.12208 2.36050 2.25818 4.44636 2.97721 3.49409 3.91787 2.27921 3.29008 4.16900 2.91427 3.83411 2.67298 2.50693 2.25593 2.74443 3.38065 5.56710 4.16888 287 - -
2.68618 4.42225 2.77519 2.73123 3.46354 2.40513 3.72494 3.29354 2.67741 2.69355 4.24690 2.90347 2.73739 3.18146 2.89801 2.37887 2.77519 2.98518 4.58477 3.61503
0.01922 4.35734 5.07968 0.61958 0.77255 0.48576 0.95510
80 2.48992 5.06425 2.80325 2.36161 4.37213 3.01006 3.65236 3.82887 2.31779 3.35803 4.12599 2.84232 3.08360 2.58735 2.89703 1.94191 2.77616 3.29929 5.53178 4.14785 288 - -
2.68618 4.42225 2.77519 2.73123 3.46354 2.40513 3.72494 3.29354 2.67741 2.69355 4.24690 2.90347 2.73739 3.18146 2.89801 2.37887 2.77519 2.98518 4.58477 3.61503
0.03620 4.35734 3.78345 0.61958 0.77255 0.48576 0.95510
81 2.18727 4.90589 2.93412 2.29621 4.16059 3.33456 3.67048 3.58672 2.45541 2.93162 3.97267 2.91158 3.67663 2.80906 2.33447 2.52993 2.69721 3.07494 5.39773 3.51944 289 - -
2.68618 4.42225 2.77519 2.73123 3.46354 2.40513 3.72494 3.29354 2.67741 2.69355 4.24690 2.90347 2.73739 3.18146 2.89801 2.37887 2.77519 2.98518 4.58477 3.61503
0.01954 4.34068 5.06302 0.61958 0.77255 0.47092 0.97931
82 2.15366 4.92142 3.02046 2.41063 4.18297 3.47341 3.67505 3.14015 2.03561 3.19168 3.99064 2.81024 3.24005 2.81182 2.84279 2.42149 2.77710 3.15383 5.41326 4.06447 290 - -
2.68611 4.42229 2.77524 2.73120 3.46358 2.40517 3.72485 3.29358 2.67739 2.69348 4.24694 2.90351 2.73738 3.18133 2.89805 2.37891 2.77515 2.98523 4.58481 3.61507
0.25001 1.53719 5.07968 0.20226 1.69764 0.48576 0.95510
83 2.27577 5.02406 2.88182 2.41080 4.31976 2.92450 3.45179 3.76899 2.25093 3.31107 4.08535 2.78602 3.23887 2.76937 2.81186 2.18245 2.72785 3.15943 5.49609 4.12101 292 - -
2.68618 4.42225 2.77519 2.73123 3.46354 2.40513 3.72494 3.29354 2.67741 2.69355 4.24690 2.90347 2.73739 3.18146 2.89801 2.37887 2.77519 2.98518 4.58477 3.61503
0.01922 4.35734 5.07968 0.61958 0.77255 0.48576 0.95510
84 2.39852 5.10767 2.71268 2.37921 4.42783 3.29799 2.90682 3.89482 1.95104 3.40100 4.15567 2.76580 3.83739 2.66733 2.53612 2.58502 2.89388 3.38596 5.55421 3.59275 293 - -
2.68618 4.42225 2.77519 2.73123 3.46354 2.40513 3.72494 3.29354 2.67741 2.69355 4.24690 2.90347 2.73739 3.18146 2.89801 2.37887 2.77519 2.98518 4.58477 3.61503
0.01922 4.35734 5.07968 0.61958 0.77255 0.48576 0.95510
85 2.73297 5.22811 0.95041 2.45308 4.59298 3.42116 3.97351 3.94326 2.92552 3.61287 3.96415 2.99998 4.01650 3.15064 3.44479 2.92687 3.25585 3.59619 5.88886 4.45991 294 - -
2.68618 4.42225 2.77519 2.73123 3.46354 2.40513 3.72494 3.29354 2.67741 2.69355 4.24690 2.90347 2.73739 3.18146 2.89801 2.37887 2.77519 2.98518 4.58477 3.61503
0.01922 4.35734 5.07968 0.61958 0.77255 0.48576 0.95510
86 2.86957 5.22208 3.17733 2.38074 4.59815 3.59926 3.68081 4.01603 1.41331 3.48629 4.27768 3.08211 3.97695 2.49036 2.10745 2.75267 3.07555 3.25014 5.58895 4.28508 295 - -
2.68618 4.42225 2.77519 2.73123 3.46354 2.40513 3.72494 3.29354 2.67741 2.69355 4.24690 2.90347 2.73739 3.18146 2.89801 2.37887 2.77519 2.98518 4.58477 3.61503
0.01922 4.35734 5.07968 0.61958 0.77255 0.48576 0.95510
87 3.71349 4.99218 5.25448 4.76013 2.60947 4.90141 4.97790 2.45921 4.60133 0.57935 3.03250 4.94584 5.04919 4.61611 4.64845 4.31305 3.93520 2.67329 5.10862 3.81077 296 - -
2.68618 4.42225 2.77519 2.73123 3.46354 2.40513 3.72494 3.29354 2.67741 2.69355 4.24690 2.90347 2.73739 3.18146 2.89801 2.37887 2.77519 2.98518 4.58477 3.61503

0.01922 4.35734 5.07968 0.61958 0.77255 0.48576 0.95510
88 3.07438 4.48173 4.60449 4.02876 2.96085 3.67937 4.52767 2.25200 3.89212 0.90222 2.95729 4.24048 4.53161 4.06308 4.03848 3.54765 3.30379 2.40128 5.02855 3.86533 297 - -
2.68618 4.42225 2.77519 2.73123 3.46354 2.40513 3.72494 3.29354 2.67741 2.69355 4.24690 2.90347 2.73739 3.18146 2.89801 2.37887 2.77519 2.98518 4.58477 3.61503
0.01922 4.35734 5.07968 0.61958 0.77255 0.48576 0.95510
89 2.75852 4.70931 3.31021 3.25071 4.80927 0.62070 4.56326 4.42340 3.58494 4.06194 4.92683 3.03152 4.08404 3.85807 3.90278 2.90243 3.25699 3.81823 6.05522 4.83835 298 - -
2.68618 4.42225 2.77519 2.73123 3.46354 2.40513 3.72494 3.29354 2.67741 2.69355 4.24690 2.90347 2.73739 3.18146 2.89801 2.37887 2.77519 2.98518 4.58477 3.61503
0.01922 4.35734 5.07968 0.61958 0.77255 0.48576 0.95510
90 2.70453 4.69213 2.89447 2.92214 4.36494 0.92896 4.20320 3.27501 3.16104 3.48218 4.36957 3.32009 4.01075 3.44871 3.55404 2.82697 3.10850 3.36320 5.71718 4.41267 299 - -
2.68618 4.42225 2.77519 2.73123 3.46354 2.40513 3.72494 3.29354 2.67741 2.69355 4.24690 2.90347 2.73739 3.18146 2.89801 2.37887 2.77519 2.98518 4.58477 3.61503
0.01922 4.35734 5.07968 0.61958 0.77255 0.48576 0.95510
91 3.11233 4.69369 4.05596 3.71109 3.40644 3.97119 4.42603 2.75887 3.49637 0.75914 3.53411 3.42166 4.45348 3.90601 3.70054 3.42402 3.41497 2.70662 5.13577 3.79633 300 - -
2.68618 4.42225 2.77519 2.73123 3.46354 2.40513 3.72494 3.29354 2.67741 2.69355 4.24690 2.90347 2.73739 3.18146 2.89801 2.37887 2.77519 2.98518 4.58477 3.61503
0.01922 4.35734 5.07968 0.61958 0.77255 0.48576 0.95510
92 3.75196 5.08526 4.96986 4.63779 3.40060 4.59502 5.14744 2.65269 4.40477 0.44704 3.31647 4.86983 4.96323 4.65054 4.49056 4.26644 4.03238 2.81087 5.42621 4.25328 301 - -
2.68618 4.42225 2.77519 2.73123 3.46354 2.40513 3.72494 3.29354 2.67741 2.69355 4.24690 2.90347 2.73739 3.18146 2.89801 2.37887 2.77519 2.98518 4.58477 3.61503
0.01922 4.35734 5.07968 0.61958 0.77255 0.48576 0.95510
93 2.11165 4.58608 3.27689 2.72909 3.85859 3.49291 3.82202 3.23833 2.70571 2.80948 3.76496 3.18708 3.68278 2.77610 3.12320 2.26440 2.06773 2.59044 5.22070 3.94269 302 - -
2.68618 4.42225 2.77519 2.73123 3.46354 2.40513 3.72494 3.29354 2.67741 2.69355 4.24690 2.90347 2.73739 3.18146 2.89801 2.37887 2.77519 2.98518 4.58477 3.61503
0.01922 4.35734 5.07968 0.61958 0.77255 0.48576 0.95510
94 1.86267 5.11362 2.33387 2.18680 4.45454 3.42659 3.72898 3.91249 2.53620 3.44999 4.23255 2.93898 2.56253 2.85671 3.03304 2.57081 2.87936 3.51709 5.63215 4.24005 303 - -
2.68618 4.42225 2.77519 2.73123 3.46354 2.40513 3.72494 3.29354 2.67741 2.69355 4.24690 2.90347 2.73739 3.18146 2.89801 2.37887 2.77519 2.98518 4.58477 3.61503
0.01922 4.35734 5.07968 0.61958 0.77255 0.48576 0.95510
95 2.42365 5.28890 1.65467 2.29227 4.72944 1.76295 3.88340 4.21096 2.80592 3.73964 4.54799 2.92499 3.95621 3.03459 3.34669 2.66991 3.18461 3.77901 5.91828 4.48356 304 - -
2.68619 4.42182 2.77520 2.73124 3.46355 2.40508 3.72495 3.29355 2.67742 2.69356 4.24691 2.90348 2.73740 3.18147 2.89802 2.37888 2.77520 2.98519 4.58478 3.61504
0.04449 3.28895 5.07968 0.63887 0.75054 0.48576 0.95510
96 3.48813 4.80196 5.02082 4.50232 0.99609 4.64091 4.51603 2.38635 4.34621 1.66580 2.92366 4.59978 4.86075 4.40367 4.42061 3.98806 3.70980 2.60786 4.73288 3.26025 307 - -
2.68618 4.42225 2.77519 2.73123 3.46354 2.40513 3.72494 3.29354 2.67741 2.69355 4.24690 2.90347 2.73739 3.18146 2.89801 2.37887 2.77519 2.98518 4.58477 3.61503
0.01922 4.35734 5.07968 0.61958 0.77255 0.48576 0.95510
97 3.11320 5.70442 0.95768 2.18194 5.00929 3.12718 3.94005 4.53390 2.80805 4.01701 4.85746 2.71007 4.01965 3.10235 3.56083 2.66710 3.40215 4.09322 6.17881 4.66309 308 - -
2.68618 4.42225 2.77519 2.73123 3.46354 2.40513 3.72494 3.29354 2.67741 2.69355 4.24690 2.90347 2.73739 3.18146 2.89801 2.37887 2.77519 2.98518 4.58477 3.61503
0.01922 4.35734 5.07968 0.61958 0.77255 0.48576 0.95510
98 2.69961 5.14487 2.66003 1.49539 4.46492 3.44967 3.65351 3.93159 2.27418 3.43560 4.19586 2.92617 3.64072 2.69782 2.65407 2.67015 2.93142 3.52303 4.68815 4.19416 309 - -
2.68618 4.42225 2.77519 2.73123 3.46354 2.40513 3.72494 3.29354 2.67741 2.69355 4.24690 2.90347 2.73739 3.18146 2.89801 2.37887 2.77519 2.98518 4.58477 3.61503
0.01922 4.35734 5.07968 0.61958 0.77255 0.48576 0.95510
99 2.61065 5.13378 2.30038 2.22030 4.45803 2.62866 3.48454 3.93065 2.33569 3.42999 4.18134 2.91226 3.83753 2.47951 2.70692 2.45022 2.82054 3.17071 5.57839 4.17817 310 - -

2.68618 4.42225 2.77519 2.73123 3.46354 2.40513 3.72494 3.29354 2.67741 2.69355 4.24690 2.90347 2.73739 3.18146 2.89801 2.37887 2.77519 2.98518 4.58477 3.61503

0.01922 4.35734 5.07968 0.61958 0.77255 0.48576 0.95510

100 2.11239 4.34941 3.75335 3.51526 4.65539 3.11910 4.56660 4.03380 3.57206 3.76266 4.58770 3.58223 3.90428 3.85206 3.85011 0.75265 2.73343 3.42968 5.99873 4.80606 311 - -

2.68618 4.42225 2.77519 2.73123 3.46354 2.40513 3.72494 3.29354 2.67741 2.69355 4.24690 2.90347 2.73739 3.18146 2.89801 2.37887 2.77519 2.98518 4.58477 3.61503

0.01922 4.35734 5.07968 0.61958 0.77255 0.48576 0.95510

101 3.27115 0.33019 4.85208 4.71986 4.64227 3.76654 5.26603 3.90356 4.56589 3.77826 4.92336 4.58858 4.50527 4.89142 4.59363 3.54004 3.77905 3.60551 5.91572 4.91808 312 - -

2.68618 4.42225 2.77519 2.73123 3.46354 2.40513 3.72494 3.29354 2.67741 2.69355 4.24690 2.90347 2.73739 3.18146 2.89801 2.37887 2.77519 2.98518 4.58477 3.61503

0.01922 4.35734 5.07968 0.61958 0.77255 0.48576 0.95510

102 2.66379 4.62280 3.41344 2.82905 3.78015 3.63360 2.87109 3.16638 2.57259 1.73395 3.70974 3.27100 4.01256 2.92947 2.30079 2.88502 2.96935 2.81684 5.14124 3.88449 313 - -

2.68618 4.42225 2.77519 2.73123 3.46354 2.40513 3.72494 3.29354 2.67741 2.69355 4.24690 2.90347 2.73739 3.18146 2.89801 2.37887 2.77519 2.98518 4.58477 3.61503

0.01922 4.35734 5.07968 0.61958 0.77255 0.48576 0.95510

103 2.63170 4.54429 3.71963 3.58782 4.61583 3.26961 4.66648 4.22100 3.69781 3.91089 4.81067 3.70784 4.05332 4.00945 3.94606 0.54363 3.15271 3.64085 5.95078 4.71167 314 - -

2.68618 4.42225 2.77519 2.73123 3.46354 2.40513 3.72494 3.29354 2.67741 2.69355 4.24690 2.90347 2.73739 3.18146 2.89801 2.37887 2.77519 2.98518 4.58477 3.61503

0.01922 4.35734 5.07968 0.61958 0.77255 0.48576 0.95510

104 3.64949 5.39807 4.19751 3.63803 4.88320 4.00593 4.30461 4.48533 2.60097 3.91208 4.92157 3.94850 4.50517 3.52055 0.43896 3.72487 3.89510 4.19300 5.77944 4.73142 315 - -

2.68618 4.42225 2.77519 2.73123 3.46354 2.40513 3.72494 3.29354 2.67741 2.69355 4.24690 2.90347 2.73739 3.18146 2.89801 2.37887 2.77519 2.98518 4.58477 3.61503

0.03780 4.35734 3.71820 0.61958 0.77255 0.48576 0.95510

105 3.88699 5.23142 4.39066 4.14717 2.20751 4.46473 3.12722 3.89820 3.94478 3.19944 4.43971 4.11542 4.84382 4.15488 4.07198 3.90498 4.12478 3.76964 3.87186 0.59549 316 - -

2.68618 4.42225 2.77519 2.73123 3.46354 2.40513 3.72494 3.29354 2.67741 2.69355 4.24690 2.90347 2.73739 3.18146 2.89801 2.37887 2.77519 2.98518 4.58477 3.61503

0.01957 4.33912 5.06146 0.61958 0.77255 0.51219 0.91425

106 2.75896 5.19861 2.91859 1.79001 4.54011 3.48228 3.40569 3.99817 2.21801 3.48346 4.25091 2.95638 3.89051 1.93530 2.59905 2.64605 2.88818 3.58980 5.61348 4.24039 317 - -

2.68618 4.42225 2.77519 2.73123 3.46354 2.40513 3.72494 3.29354 2.67741 2.69355 4.24690 2.90347 2.73739 3.18146 2.89801 2.37887 2.77519 2.98518 4.58477 3.61503

0.01957 4.33912 5.06146 0.61958 0.77255 0.51219 0.91425

107 2.24114 4.57118 3.27354 2.76994 3.13364 3.47728 3.85993 3.25154 2.62909 2.92632 3.79020 3.21362 3.93878 3.01841 3.15432 1.52240 2.90721 2.96560 5.22984 3.94825 318 - -

2.68618 4.42225 2.77519 2.73123 3.46354 2.40513 3.72494 3.29354 2.67741 2.69355 4.24690 2.90347 2.73739 3.18146 2.89801 2.37887 2.77519 2.98518 4.58477 3.61503

0.01957 4.33912 5.06146 0.61958 0.77255 0.51219 0.91425

108 1.91571 4.51217 3.34907 2.81185 3.83570 3.27880 3.87611 3.21632 2.79044 2.89656 3.75671 3.23877 3.54778 3.11557 3.19368 1.82449 2.80816 2.64491 5.21487 3.35012 319 - -

2.68618 4.42226 2.77520 2.73124 3.46339 2.40513 3.72495 3.29355 2.67741 2.69352 4.24690 2.90347 2.73740 3.18147 2.89801 2.37887 2.77520 2.98519 4.58477 3.61504

0.05310 3.09263 5.06146 0.46638 0.98689 0.51219 0.91425

109 2.58975 4.29995 3.66031 3.08581 3.42171 3.46572 3.46916 2.78118 2.98303 1.65978 2.71618 3.45048 4.03606 3.29904 2.82544 2.83119 2.88363 2.56738 4.88492 3.46594 321 - -

2.68618 4.42225 2.77519 2.73123 3.46354 2.40513 3.72494 3.29354 2.67741 2.69355 4.24690 2.90347 2.73739 3.18146 2.89801 2.37887 2.77519 2.98518 4.58477 3.61503

0.01957 4.33912 5.06146 0.61958 0.77255 0.51219 0.91425

110 3.53627 5.05719 4.09121 3.73180 2.38812 4.22954 3.29058 3.68364 3.42241 3.10440 4.23289 3.88439 4.61690 3.81560 3.29871 3.61736 3.76803 3.51361 4.03352 0.74176 322 - -

2.68618 4.42225 2.77519 2.73123 3.46354 2.40513 3.72494 3.29354 2.67741 2.69355 4.24690 2.90347 2.73739 3.18146 2.89801 2.37887 2.77519 2.98518 4.58477 3.61503

0.01957 4.33912 5.06146 0.61958 0.77255 0.51219 0.91425

111 2.94745 4.94373 3.48207 2.86509 3.53700 3.73875 2.99524 3.58142 2.40141 3.13924 4.03336 3.29690 4.10420 3.00647 1.29310 3.00469 3.15323 3.30957 4.30140 2.98426 323 - -
2.68618 4.42225 2.77519 2.73123 3.46354 2.40513 3.72494 3.29354 2.67741 2.69355 4.24690 2.90347 2.73739 3.18146 2.89801 2.37887 2.77519 2.98518 4.58477 3.61503
0.01957 4.33912 5.06146 0.61958 0.77255 0.51219 0.91425
112 3.35473 5.44989 3.84707 3.07490 4.99390 3.92291 3.77289 4.30107 0.95489 3.67481 4.55393 3.43941 4.26543 2.90046 1.56421 3.33323 3.48838 3.98240 5.65570 4.54105 324 - -
2.68618 4.42225 2.77519 2.73123 3.46354 2.40513 3.72494 3.29354 2.67741 2.69355 4.24690 2.90347 2.73739 3.18146 2.89801 2.37887 2.77519 2.98518 4.58477 3.61503
0.01957 4.33912 5.06146 0.61958 0.77255 0.51219 0.91425
113 2.52740 5.10212 2.90930 2.32333 4.41302 3.45345 3.43882 3.86911 2.33648 3.38889 4.15890 2.19298 2.44114 2.69157 2.62793 2.57156 2.92767 3.47576 5.55301 4.17224 325 - -
2.68620 4.42227 2.77521 2.73125 3.46356 2.40514 3.72496 3.29356 2.67737 2.69357 4.24691 2.90320 2.73741 3.18148 2.89802 2.37889 2.77521 2.98520 4.58479 3.61505
0.11524 2.27777 5.06146 0.32570 1.28021 0.51219 0.91425
114 2.19629 4.45057 3.43277 2.88984 3.04914 3.48922 3.90394 3.09885 2.85779 2.79925 3.45273 3.29687 3.45844 3.17560 3.24142 1.61236 2.77915 2.83082 5.14258 3.88919 327 - -
2.68618 4.42225 2.77519 2.73123 3.46354 2.40513 3.72494 3.29354 2.67741 2.69355 4.24690 2.90347 2.73739 3.18146 2.89801 2.37887 2.77519 2.98518 4.58477 3.61503
0.01957 4.33912 5.06146 0.61958 0.77255 0.51219 0.91425
115 2.39589 4.48256 3.41686 2.99685 4.28573 2.29279 4.11660 3.68898 3.01563 3.12266 4.17752 3.32561 1.50222 3.33688 3.40014 2.52361 2.43516 3.25392 5.61072 4.35281 328 - -
2.68618 4.42225 2.77519 2.73123 3.46354 2.40513 3.72494 3.29354 2.67741 2.69355 4.24690 2.90347 2.73739 3.18146 2.89801 2.37887 2.77519 2.98518 4.58477 3.61503
0.01957 4.33912 5.06146 0.61958 0.77255 0.51219 0.91425
116 2.74656 4.67187 3.34444 2.77664 2.75798 3.62398 2.93170 3.24181 2.34446 2.88462 3.76493 3.23209 4.00363 3.04354 2.39679 2.85787 2.97293 2.98432 4.94786 2.21719 329 - -
2.68618 4.42225 2.77519 2.73123 3.46354 2.40513 3.72494 3.29354 2.67741 2.69355 4.24690 2.90347 2.73739 3.18146 2.89801 2.37887 2.77519 2.98518 4.58477 3.61503
0.01957 4.33912 5.06146 0.61958 0.77255 0.51219 0.91425
117 2.51860 4.97881 2.99042 2.19166 3.97863 3.46242 3.65639 3.29724 1.86092 3.25337 3.88648 2.96473 3.47851 2.78203 2.58987 2.44665 2.88833 3.20956 5.45431 4.09198 330 - -
2.68618 4.42225 2.77519 2.73123 3.46354 2.40513 3.72494 3.29354 2.67741 2.69355 4.24690 2.90347 2.73739 3.18146 2.89801 2.37887 2.77519 2.98518 4.58477 3.61503
0.01937 4.34956 5.07191 0.61958 0.77255 0.51219 0.91425
118 2.69721 4.59773 3.38899 2.92148 3.89461 3.51676 3.97285 3.20653 2.80841 2.64376 3.83193 3.33004 1.41341 2.99116 3.15559 2.84717 2.92004 2.71627 5.31089 4.04177 331 - -
2.68618 4.42225 2.77519 2.73123 3.46354 2.40513 3.72494 3.29354 2.67741 2.69355 4.24690 2.90347 2.73739 3.18146 2.89801 2.37887 2.77519 2.98518 4.58477 3.61503
0.01937 4.34956 5.07191 0.61958 0.77255 0.51219 0.91425
119 2.41228 3.67683 4.07136 3.77992 4.51090 3.13855 4.65445 3.93514 3.70250 3.69136 4.55072 3.71342 3.93061 3.99607 3.92645 0.65352 2.93745 3.37026 5.89661 4.69012 332 - -
2.68618 4.42225 2.77519 2.73123 3.46354 2.40513 3.72494 3.29354 2.67741 2.69355 4.24690 2.90347 2.73739 3.18146 2.89801 2.37887 2.77519 2.98518 4.58477 3.61503
0.01937 4.34956 5.07191 0.61958 0.77255 0.51219 0.91425
120 2.61807 4.99651 2.99573 2.24885 4.30659 3.46988 3.68266 3.74160 2.41937 3.17712 4.08679 2.98884 2.31983 2.81076 2.25887 2.32423 2.84037 3.37600 5.48973 4.13602 333 - -
2.68618 4.42225 2.77519 2.73123 3.46354 2.40513 3.72494 3.29354 2.67741 2.69355 4.24690 2.90347 2.73739 3.18146 2.89801 2.37887 2.77519 2.98518 4.58477 3.61503
0.01937 4.34956 5.07191 0.61958 0.77255 0.51219 0.91425
121 3.73290 5.19606 4.21661 3.88504 2.26586 4.37112 1.72410 3.85394 3.69173 3.19151 4.38038 3.97984 4.73990 3.96196 3.88625 3.75585 3.96002 3.70177 3.92379 0.94956 334 - -
2.68618 4.42225 2.77519 2.73123 3.46354 2.40513 3.72494 3.29354 2.67741 2.69355 4.24690 2.90347 2.73739 3.18146 2.89801 2.37887 2.77519 2.98518 4.58477 3.61503
0.01937 4.34956 5.07191 0.61958 0.77255 0.51219 0.91425
122 3.74146 5.07665 4.95725 4.62440 3.39499 4.58507 5.13620 2.64614 4.39124 0.45190 3.31204 4.85699 4.95414 4.63915 4.47861 4.25420 4.02202 2.80246 5.41870 4.24420 335 - -
2.68618 4.42225 2.77519 2.73123 3.46354 2.40513 3.72494 3.29354 2.67741 2.69355 4.24690 2.90347 2.73739 3.18146 2.89801 2.37887 2.77519 2.98518 4.58477 3.61503

0.01937 4.34956 5.07191 0.61958 0.77255 0.51219 0.91425
123 2.99922 4.41001 3.81341 3.91742 3.55604 4.20693 4.59740 1.99717 3.82175 1.81786 3.45061 4.17490 4.54778 4.07767 4.04407 3.52776 3.24525 1.07948 5.27232 4.06569 336 - -
2.68618 4.42225 2.77519 2.73123 3.46354 2.40513 3.72494 3.29354 2.67741 2.69355 4.24690 2.90347 2.73739 3.18146 2.89801 2.37887 2.77519 2.98518 4.58477 3.61503
0.01937 4.34956 5.07191 0.61958 0.77255 0.51219 0.91425
124 2.48881 5.07605 2.79263 2.13706 4.37312 3.44930 3.66287 3.82672 2.37270 3.36016 4.13449 2.93725 3.85743 2.59666 2.81586 1.74415 2.92130 3.44166 5.53796 3.79049 337 - -
2.68618 4.42225 2.77519 2.73123 3.46354 2.40513 3.72494 3.29354 2.67741 2.69355 4.24690 2.90347 2.73739 3.18146 2.89801 2.37887 2.77519 2.98518 4.58477 3.61503
0.01937 4.34956 5.07191 0.61958 0.77255 0.51219 0.91425
125 2.95502 5.33800 3.49772 2.75467 4.80322 3.77196 3.73204 4.15444 1.28216 3.58121 4.41805 3.27026 4.13346 2.85415 1.50813 2.93550 3.29147 3.80687 5.62594 4.42642 338 - -
2.68618 4.42225 2.77519 2.73123 3.46354 2.40513 3.72494 3.29354 2.67741 2.69355 4.24690 2.90347 2.73739 3.18146 2.89801 2.37887 2.77519 2.98518 4.58477 3.61503
0.01937 4.34956 5.07191 0.61958 0.77255 0.51219 0.91425
126 3.74146 5.07665 4.95725 4.62440 3.39499 4.58507 5.13620 2.64614 4.39124 0.45190 3.31204 4.85699 4.95414 4.63915 4.47861 4.25420 4.02202 2.80246 5.41870 4.24420 339 - -
2.68618 4.42225 2.77519 2.73123 3.46354 2.40513 3.72494 3.29354 2.67741 2.69355 4.24690 2.90347 2.73739 3.18146 2.89801 2.37887 2.77519 2.98518 4.58477 3.61503
0.01937 4.34956 5.07191 0.61958 0.77255 0.51219 0.91425
127 3.35493 5.35158 3.80123 3.15620 4.77849 3.89388 3.87173 4.22241 2.09626 3.63142 4.55733 3.50778 4.29713 2.76767 0.76233 3.36962 3.53244 3.92293 5.63399 4.48109 340 - -
2.68618 4.42225 2.77519 2.73123 3.46354 2.40513 3.72494 3.29354 2.67741 2.69355 4.24690 2.90347 2.73739 3.18146 2.89801 2.37887 2.77519 2.98518 4.58477 3.61503
0.01937 4.34956 5.07191 0.61958 0.77255 0.51219 0.91425
128 2.53793 5.09471 2.81508 2.29934 4.40935 3.45287 3.64182 3.86850 2.26870 3.38345 3.88821 2.81737 3.84852 2.67746 2.08634 2.06679 2.90681 3.46958 5.54323 4.16001 341 - -
2.68618 4.42225 2.77519 2.73123 3.46354 2.40513 3.72494 3.29354 2.67741 2.69355 4.24690 2.90347 2.73739 3.18146 2.89801 2.37887 2.77519 2.98518 4.58477 3.61503
0.01937 4.34956 5.07191 0.61958 0.77255 0.46959 0.98152
129 3.14658 4.99417 3.53345 3.08312 2.82895 3.89679 2.20360 3.61715 2.89793 3.12422 4.11507 3.46998 4.28894 2.58678 3.21372 3.20394 3.37012 3.38368 4.41920 1.33993 342 - -
2.68618 4.42225 2.77519 2.73123 3.46354 2.40513 3.72494 3.29354 2.67741 2.69355 4.24690 2.90347 2.73739 3.18146 2.89801 2.37887 2.77519 2.98518 4.58477 3.61503
0.01902 4.36759 5.08994 0.61958 0.77255 0.48576 0.95510
130 3.49436 5.61867 2.83110 0.48423 5.04562 3.68015 4.34993 4.61026 3.30511 4.15618 5.14062 3.35048 4.31736 3.57532 3.74473 3.44198 3.81256 4.25293 6.11616 4.88247 343 - -
2.68618 4.42225 2.77519 2.73123 3.46354 2.40513 3.72494 3.29354 2.67741 2.69355 4.24690 2.90347 2.73739 3.18146 2.89801 2.37887 2.77519 2.98518 4.58477 3.61503
0.01902 4.36759 5.08994 0.61958 0.77255 0.48576 0.95510
131 1.96134 4.88960 2.79780 2.13719 4.13534 3.48378 3.71029 3.55137 2.43185 3.15433 2.61976 3.01475 3.88936 2.86171 2.98036 2.56984 2.91638 3.22572 5.39704 4.05847 344 - -
2.68618 4.42225 2.77519 2.73123 3.46354 2.40513 3.72494 3.29354 2.67741 2.69355 4.24690 2.90347 2.73739 3.18146 2.89801 2.37887 2.77519 2.98518 4.58477 3.61503
0.01902 4.36759 5.08994 0.61958 0.77255 0.48576 0.95510
132 2.78179 4.38524 3.85200 3.27131 3.36958 3.78405 3.99858 2.67412 3.00265 1.25221 3.41813 3.40964 4.15452 3.41284 2.78852 3.06842 3.01072 2.60078 4.89160 3.41144 345 - -
2.68618 4.42225 2.77519 2.73123 3.46354 2.40513 3.72494 3.29354 2.67741 2.69355 4.24690 2.90347 2.73739 3.18146 2.89801 2.37887 2.77519 2.98518 4.58477 3.61503
0.01902 4.36759 5.08994 0.61958 0.77255 0.48576 0.95510
133 3.06553 5.25999 3.04731 2.69572 4.16428 3.64716 1.51889 4.04254 2.43916 3.52732 4.40819 3.18586 4.11153 1.75448 2.74685 3.05201 3.29677 3.71061 5.42981 3.99966 346 - -
2.68618 4.42225 2.77519 2.73123 3.46354 2.40513 3.72494 3.29354 2.67741 2.69355 4.24690 2.90347 2.73739 3.18146 2.89801 2.37887 2.77519 2.98518 4.58477 3.61503
0.01902 4.36759 5.08994 0.61958 0.77255 0.48576 0.95510
134 3.24321 5.37966 3.74619 2.99181 4.87111 3.87188 3.76126 4.19104 1.20341 3.60603 4.46963 3.38328 4.21632 2.88754 1.36900 3.22983 3.39561 3.50251 5.63209 4.47869 347 - -

2.68618 4.42225 2.77519 2.73123 3.46354 2.40513 3.72494 3.29354 2.67741 2.69355 4.24690 2.90347 2.73739 3.18146 2.89801 2.37887 2.77519 2.98518 4.58477 3.61503
0.01902 4.36759 5.08994 0.61958 0.77255 0.48576 0.95510
135 3.34167 5.40339 3.89663 3.10130 4.88678 3.93774 3.78386 4.20154 1.66388 3.32606 4.49912 3.45951 4.27528 2.91675 0.93460 3.33525 3.47786 3.89987 5.62739 4.50134 348 - -
2.68618 4.42225 2.77519 2.73123 3.46354 2.40513 3.72494 3.29354 2.67741 2.69355 4.24690 2.90347 2.73739 3.18146 2.89801 2.37887 2.77519 2.98518 4.58477 3.61503
0.01902 4.36759 5.08994 0.61958 0.77255 0.48576 0.95510
136 3.28827 0.32295 4.86911 4.73977 4.66193 3.78046 5.28405 3.92672 4.58673 3.80000 4.94570 4.60654 4.51962 4.91175 4.61223 3.55704 3.79696 3.62749 5.93137 4.93730 349 - -
2.68618 4.42225 2.77519 2.73123 3.46354 2.40513 3.72494 3.29354 2.67741 2.69355 4.24690 2.90347 2.73739 3.18146 2.89801 2.37887 2.77519 2.98518 4.58477 3.61503
0.01902 4.36759 5.08994 0.61958 0.77255 0.48576 0.95510
137 2.42268 4.55733 3.67682 3.60477 4.86504 0.57320 4.76562 4.32983 3.84017 4.04381 4.92048 3.72540 4.06492 4.09861 4.08622 2.82812 3.17912 3.70595 6.12966 5.00118 350 - -
2.68618 4.42225 2.77519 2.73123 3.46354 2.40513 3.72494 3.29354 2.67741 2.69355 4.24690 2.90347 2.73739 3.18146 2.89801 2.37887 2.77519 2.98518 4.58477 3.61503
0.01902 4.36759 5.08994 0.61958 0.77255 0.48576 0.95510
138 3.54539 5.18809 4.20814 4.13635 5.04512 3.84929 5.14538 4.75467 4.24336 4.30986 5.39830 4.38159 0.27068 4.61398 4.40637 3.73410 4.02880 4.33588 6.06192 5.18068 351 - -
2.68618 4.42225 2.77519 2.73123 3.46354 2.40513 3.72494 3.29354 2.67741 2.69355 4.24690 2.90347 2.73739 3.18146 2.89801 2.37887 2.77519 2.98518 4.58477 3.61503
0.01902 4.36759 5.08994 0.61958 0.77255 0.48576 0.95510
139 2.77770 4.89572 2.70969 2.57390 3.63681 1.29785 3.58230 3.67037 2.78279 3.28909 4.13790 2.93650 3.96824 3.08071 3.23969 2.82521 3.06358 3.33728 5.46372 4.08310 352 - -
2.68618 4.42225 2.77519 2.73123 3.46354 2.40513 3.72494 3.29354 2.67741 2.69355 4.24690 2.90347 2.73739 3.18146 2.89801 2.37887 2.77519 2.98518 4.58477 3.61503
0.01902 4.36759 5.08994 0.61958 0.77255 0.48576 0.95510
140 2.51475 4.41500 3.77715 3.56236 4.46397 3.22376 4.56806 3.72736 3.53855 3.56143 4.48008 3.65038 3.99091 3.88787 3.78213 2.56051 0.72186 3.27195 5.86861 4.64358 353 - -
2.68618 4.42225 2.77519 2.73123 3.46354 2.40513 3.72494 3.29354 2.67741 2.69355 4.24690 2.90347 2.73739 3.18146 2.89801 2.37887 2.77519 2.98518 4.58477 3.61503
0.01902 4.36759 5.08994 0.61958 0.77255 0.48576 0.95510
141 2.11760 5.11027 2.82474 1.88122 4.42633 3.44819 3.64698 3.89039 2.16598 3.25279 4.16240 2.92880 3.37601 2.75608 2.69928 2.52669 2.90961 3.48595 5.56117 4.17033 354 - -
2.68618 4.42225 2.77519 2.73123 3.46354 2.40513 3.72494 3.29354 2.67741 2.69355 4.24690 2.90347 2.73739 3.18146 2.89801 2.37887 2.77519 2.98518 4.58477 3.61503
0.01902 4.36759 5.08994 0.61958 0.77255 0.48576 0.95510
142 1.78798 4.66626 3.19501 2.63851 3.63172 3.30435 3.75909 3.26108 2.61883 2.73613 3.76408 3.12673 3.34114 2.87643 2.86616 2.32679 2.88884 2.98206 5.21488 3.76210 355 - -
2.68618 4.42225 2.77519 2.73123 3.46354 2.40513 3.72494 3.29354 2.67741 2.69355 4.24690 2.90347 2.73739 3.18146 2.89801 2.37887 2.77519 2.98518 4.58477 3.61503
0.01902 4.36759 5.08994 0.61958 0.77255 0.48576 0.95510
143 4.05778 5.26353 4.85140 4.63111 2.06578 4.66033 3.71147 3.77497 4.45721 3.05800 4.37791 4.34860 5.00531 4.44106 4.47677 4.09734 4.29964 3.70623 3.83925 0.53404 356 - -
2.68618 4.42225 2.77519 2.73123 3.46354 2.40513 3.72494 3.29354 2.67741 2.69355 4.24690 2.90347 2.73739 3.18146 2.89801 2.37887 2.77519 2.98518 4.58477 3.61503
0.01902 4.36759 5.08994 0.61958 0.77255 0.48576 0.95510
144 2.57885 5.12074 2.85225 2.24060 4.44444 3.45097 3.63731 3.91120 1.76006 3.41392 4.16869 2.65615 3.84486 2.67833 2.63152 2.31596 2.82999 3.50020 4.79201 4.17238 357 - -
2.68618 4.42225 2.77519 2.73123 3.46354 2.40513 3.72494 3.29354 2.67741 2.69355 4.24690 2.90347 2.73739 3.18146 2.89801 2.37887 2.77519 2.98518 4.58477 3.61503
0.01902 4.36759 5.08994 0.61958 0.77255 0.48576 0.95510
145 2.65218 5.18113 2.78870 2.25329 4.52509 3.46938 3.64474 3.99344 1.72406 3.27253 4.22740 2.54132 3.86588 2.56351 2.35054 2.67986 2.94142 3.57233 5.60300 4.21646 358 - -
2.68618 4.42225 2.77519 2.73123 3.46354 2.40513 3.72494 3.29354 2.67741 2.69355 4.24690 2.90347 2.73739 3.18146 2.89801 2.37887 2.77519 2.98518 4.58477 3.61503
0.01902 4.36759 5.08994 0.61958 0.77255 0.48576 0.95510

146 0.75143 4.31091 3.91268 3.64279 4.57834 3.12297 4.61064 3.91960 3.65759 3.67799 4.51083 3.64258 3.90966 3.92201 3.91232 2.12191 2.64998 3.35151 5.94671 4.77440 359 - -
2.68618 4.42225 2.77519 2.73123 3.46354 2.40513 3.72494 3.29354 2.67741 2.69355 4.24690 2.90347 2.73739 3.18146 2.89801 2.37887 2.77519 2.98518 4.58477 3.61503
0.01902 4.36759 5.08994 0.61958 0.77255 0.48576 0.95510
147 2.59891 4.29499 3.43215 3.04457 3.43409 3.69710 3.97878 2.48224 3.09199 2.18340 3.41046 3.51080 4.07592 3.37599 3.40095 2.85578 2.18193 1.80301 4.91498 3.69628 360 - -
2.68618 4.42225 2.77519 2.73123 3.46354 2.40513 3.72494 3.29354 2.67741 2.69355 4.24690 2.90347 2.73739 3.18146 2.89801 2.37887 2.77519 2.98518 4.58477 3.61503
0.01902 4.36759 5.08994 0.61958 0.77255 0.48576 0.95510
148 2.57544 5.15336 2.78291 1.51936 4.47733 3.45921 3.65781 3.94172 2.18222 3.44349 3.88020 2.93584 3.86512 2.69158 2.61775 2.68081 2.94121 3.53353 5.59190 4.20304 361 - -
2.68618 4.42225 2.77519 2.73123 3.46354 2.40513 3.72494 3.29354 2.67741 2.69355 4.24690 2.90347 2.73739 3.18146 2.89801 2.37887 2.77519 2.98518 4.58477 3.61503
0.01902 4.36759 5.08994 0.61958 0.77255 0.48576 0.95510
149 2.69940 4.85782 3.12932 2.55986 4.09672 3.52752 3.42634 2.59676 2.27824 2.77447 3.93161 3.06640 3.91308 2.24605 2.39934 2.45194 2.92583 3.19097 5.35087 4.03669 362 - -
2.68618 4.42225 2.77519 2.73123 3.46354 2.40513 3.72494 3.29354 2.67741 2.69355 4.24690 2.90347 2.73739 3.18146 2.89801 2.37887 2.77519 2.98518 4.58477 3.61503
0.01902 4.36759 5.08994 0.61958 0.77255 0.48576 0.95510
150 3.32270 4.82407 4.47978 4.10798 3.44250 4.20386 4.77675 2.69205 3.89582 0.60092 3.37027 4.34922 3.82995 4.22522 4.05998 3.70545 3.62608 2.75437 5.33571 4.14394 363 - -
2.68618 4.42225 2.77519 2.73123 3.46354 2.40513 3.72494 3.29354 2.67741 2.69355 4.24690 2.90347 2.73739 3.18146 2.89801 2.37887 2.77519 2.98518 4.58477 3.61503
0.01902 4.36759 5.08994 0.61958 0.77255 0.48576 0.95510
151 2.75736 5.12108 2.91921 2.48185 4.47077 2.43512 3.68617 3.91208 1.99282 3.42363 4.20456 3.00497 3.90963 2.80369 1.86635 2.58380 2.86869 3.52367 5.57271 4.22213 364 - -
2.68618 4.42225 2.77519 2.73123 3.46354 2.40513 3.72494 3.29354 2.67741 2.69355 4.24690 2.90347 2.73739 3.18146 2.89801 2.37887 2.77519 2.98518 4.58477 3.61503
0.01902 4.36759 5.08994 0.61958 0.77255 0.48576 0.95510
152 2.56530 5.02855 2.83296 2.45301 4.32537 3.46892 2.54066 3.76825 2.46981 3.32567 4.12007 2.98402 3.89177 2.68925 2.80245 1.69177 2.95739 3.40411 5.52059 4.15879 365 - -
2.68618 4.42225 2.77519 2.73123 3.46354 2.40513 3.72494 3.29354 2.67741 2.69355 4.24690 2.90347 2.73739 3.18146 2.89801 2.37887 2.77519 2.98518 4.58477 3.61503
0.01902 4.36759 5.08994 0.61958 0.77255 0.48576 0.95510
153 2.67706 5.22756 1.99370 2.30709 4.55556 1.94352 3.73740 4.02842 2.48223 3.54246 4.31965 2.91567 3.42313 2.86161 2.94026 2.52278 3.03269 3.61868 5.70776 4.29548 366 - -
2.68618 4.42225 2.77519 2.73123 3.46354 2.40513 3.72494 3.29354 2.67741 2.69355 4.24690 2.90347 2.73739 3.18146 2.89801 2.37887 2.77519 2.98518 4.58477 3.61503
0.01902 4.36759 5.08994 0.61958 0.77255 0.48576 0.95510
154 2.67666 5.16514 2.12020 2.30204 4.50162 3.30721 3.22269 3.98049 2.10760 3.46504 4.20946 2.91076 3.64067 2.40036 2.56484 2.57788 2.90804 3.55146 5.60057 3.89405 367 - -
2.68618 4.42225 2.77519 2.73123 3.46354 2.40513 3.72494 3.29354 2.67741 2.69355 4.24690 2.90347 2.73739 3.18146 2.89801 2.37887 2.77519 2.98518 4.58477 3.61503
0.25183 4.36759 1.56092 0.61958 0.77255 0.48576 0.95510
155 2.56821 5.02166 2.78582 1.88715 4.35137 3.06256 3.63415 3.80641 2.41169 3.34127 4.12040 2.63367 3.56994 2.75767 2.75572 2.17816 2.79859 3.41532 5.52005 4.13675 368 - -
2.68618 4.42225 2.77519 2.73123 3.46354 2.40513 3.72494 3.29354 2.67741 2.69355 4.24690 2.90347 2.73739 3.18146 2.89801 2.37887 2.77519 2.98518 4.58477 3.61503
0.04324 4.13971 3.63491 0.61958 0.77255 0.72766 0.65979
156 2.26786 4.74317 2.94366 2.48834 4.14442 2.57663 3.72095 3.57047 2.54511 3.17361 3.98711 2.18305 3.58805 2.88079 3.00245 2.35352 2.85628 3.21071 5.40874 3.66907 369 - -
2.68618 4.42225 2.77519 2.73123 3.46354 2.40513 3.72494 3.29354 2.67741 2.69355 4.24690 2.90347 2.73739 3.18146 2.89801 2.37887 2.77519 2.98518 4.58477 3.61503
0.02409 4.13386 4.85620 0.61958 0.77255 0.75752 0.63267
157 2.48497 4.86239 2.81255 2.35002 4.11741 2.74092 2.67721 3.36774 2.41181 3.12957 3.93176 2.70313 3.81078 2.76310 2.88126 2.62471 2.75874 3.13807 5.35504 3.71752 370 - -
2.68618 4.42225 2.77519 2.73123 3.46354 2.40513 3.72494 3.29354 2.67741 2.69355 4.24690 2.90347 2.73739 3.18146 2.89801 2.37887 2.77519 2.98518 4.58477 3.61503

0.04461 4.13386 3.58961 0.61958 0.77255 0.60270 0.79259

158 2.11644 4.51379 3.07200 2.71164 3.86376 2.67263 3.81581 2.62174 2.71066 2.91702 3.76982 3.15293 3.86774 3.03630 3.12746 2.09877 2.63481 2.93853 5.22457 3.94819 371 - -

2.68618 4.42225 2.77519 2.73123 3.46354 2.40513 3.72494 3.29354 2.67741 2.69355 4.24690 2.90347 2.73739 3.18146 2.89801 2.37887 2.77519 2.98518 4.58477 3.61503

0.02256 4.19874 4.92109 0.61958 0.77255 0.37414 1.16436

159 2.42223 4.93849 2.78182 2.33786 4.21685 2.80054 3.68144 3.50447 2.46445 3.22446 4.01909 2.81566 3.86501 2.81546 2.93795 2.25925 2.30906 2.92871 5.44052 4.08578 372 - -

2.68616 4.42233 2.77515 2.73127 3.46350 2.40517 3.72488 3.29362 2.67749 2.69363 4.24698 2.90355 2.73719 3.18154 2.89809 2.37861 2.77528 2.98519 4.58485 3.61511

0.29519 1.55971 3.09198 0.62982 0.76076 0.48576 0.95510

160 2.31131 5.08106 2.70116 2.31078 4.39309 3.16644 3.63340 3.85562 1.99161 3.37300 3.66421 2.71395 2.91144 2.74322 2.87147 2.51066 2.88905 3.45450 5.53644 4.14666 376 - -

2.68618 4.42225 2.77519 2.73123 3.46354 2.40513 3.72494 3.29354 2.67741 2.69355 4.24690 2.90347 2.73739 3.18146 2.89801 2.37887 2.77519 2.98518 4.58477 3.61503

0.12967 4.32755 2.22175 0.61958 0.77255 0.54291 0.87002

161 2.50870 4.97960 2.50379 2.25490 4.26952 2.91682 3.63502 3.71369 2.35788 3.26707 3.48139 2.91586 3.82171 2.76030 2.89349 2.04071 2.78390 3.34307 5.46283 4.09125 377 - -

2.68618 4.42225 2.77519 2.73123 3.46354 2.40513 3.72494 3.29354 2.67741 2.69355 4.24690 2.90347 2.73739 3.18146 2.89801 2.37887 2.77519 2.98518 4.58477 3.61503

0.04205 4.21995 3.63136 0.61958 0.77255 0.41006 1.08949

162 2.65889 4.96060 2.46913 2.38831 4.22945 3.28676 3.66411 3.66452 2.44233 3.10444 4.02479 2.76764 3.65194 2.50546 2.83915 2.60502 2.68929 2.28470 5.44346 4.08367 378 - -

2.68618 4.42225 2.77519 2.73123 3.46354 2.40513 3.72494 3.29354 2.67741 2.69355 4.24690 2.90347 2.73739 3.18146 2.89801 2.37887 2.77519 2.98518 4.58477 3.61503

0.01963 4.33656 5.05890 0.61958 0.77255 0.45875 0.99987

163 2.43125 4.92001 2.74942 2.32240 4.18249 2.42469 3.68181 3.60991 2.46859 3.19335 3.50550 2.99272 3.86831 2.75025 2.93966 2.47297 2.43089 3.00956 5.41727 4.06760 379 - -

2.68618 4.42225 2.77519 2.73123 3.46354 2.40513 3.72494 3.29354 2.67741 2.69355 4.24690 2.90347 2.73739 3.18146 2.89801 2.37887 2.77519 2.98518 4.58477 3.61503

0.01902 4.36759 5.08994 0.61958 0.77255 0.48576 0.95510

164 2.88266 5.59086 2.05333 1.12707 4.89617 2.68400 3.87176 4.40877 2.81127 3.88929 4.69820 2.86928 3.98418 2.86484 3.38078 2.91789 3.28664 3.97049 6.04172 4.55542 380 - -

2.68618 4.42225 2.77519 2.73123 3.46354 2.40513 3.72494 3.29354 2.67741 2.69355 4.24690 2.90347 2.73739 3.18146 2.89801 2.37887 2.77519 2.98518 4.58477 3.61503

0.01902 4.36759 5.08994 0.61958 0.77255 0.48576 0.95510

165 3.28827 0.32295 4.86911 4.73977 4.66193 3.78046 5.28405 3.92672 4.58673 3.80000 4.94570 4.60654 4.51962 4.91175 4.61223 3.55704 3.79696 3.62749 5.93137 4.93730 381 - -

2.68618 4.42225 2.77519 2.73123 3.46354 2.40513 3.72494 3.29354 2.67741 2.69355 4.24690 2.90347 2.73739 3.18146 2.89801 2.37887 2.77519 2.98518 4.58477 3.61503

0.01902 4.36759 5.08994 0.61958 0.77255 0.48576 0.95510

166 2.93590 5.25376 3.21299 2.63743 4.64830 3.40291 3.70908 4.05315 1.91321 3.51845 4.32297 2.45342 4.02084 2.72784 1.43527 2.91262 2.97673 3.68029 5.61221 4.33020 382 - -

2.68618 4.42225 2.77519 2.73123 3.46354 2.40513 3.72494 3.29354 2.67741 2.69355 4.24690 2.90347 2.73739 3.18146 2.89801 2.37887 2.77519 2.98518 4.58477 3.61503

0.01902 4.36759 5.08994 0.61958 0.77255 0.48576 0.95510

167 4.03870 5.32308 4.60931 4.45436 2.49387 4.43826 3.96720 3.99215 4.30240 3.31100 4.61656 4.38690 4.91067 4.47972 4.37220 4.11426 4.33336 3.88849 4.14795 0.42355 383 - -

2.68618 4.42225 2.77519 2.73123 3.46354 2.40513 3.72494 3.29354 2.67741 2.69355 4.24690 2.90347 2.73739 3.18146 2.89801 2.37887 2.77519 2.98518 4.58477 3.61503

0.01902 4.36759 5.08994 0.61958 0.77255 0.48576 0.95510

168 3.39363 4.64617 5.28659 4.75157 3.52188 4.82026 5.28430 1.46002 4.63858 1.21397 3.29793 4.92845 5.02212 4.76192 4.73656 4.19910 3.63264 1.44074 5.57830 4.47165 384 - -

2.68618 4.42225 2.77519 2.73123 3.46354 2.40513 3.72494 3.29354 2.67741 2.69355 4.24690 2.90347 2.73739 3.18146 2.89801 2.37887 2.77519 2.98518 4.58477 3.61503

0.01902 4.36759 5.08994 0.61958 0.77255 0.48576 0.95510

169 3.25301 4.55958 5.02253 4.51060 2.98093 4.58843 5.04148 1.87609 4.40096 2.17539 3.47048 4.67870 4.88698 4.60343 4.55480 3.96264 3.51038 0.79791 5.46517 4.19784 385 - -

2.68618 4.42225 2.77519 2.73123 3.46354 2.40513 3.72494 3.29354 2.67741 2.69355 4.24690 2.90347 2.73739 3.18146 2.89801 2.37887 2.77519 2.98518 4.58477 3.61503
0.01902 4.36759 5.08994 0.61958 0.77255 0.48576 0.95510
170 2.69762 4.37025 3.73540 3.20570 3.34165 3.32171 3.96805 2.92712 3.08455 2.27656 3.55385 3.52524 4.08324 3.41294 3.37390 2.17923 2.96595 2.70217 1.83684 3.50182 386 - -
2.68618 4.42225 2.77519 2.73123 3.46354 2.40513 3.72494 3.29354 2.67741 2.69355 4.24690 2.90347 2.73739 3.18146 2.89801 2.37887 2.77519 2.98518 4.58477 3.61503
0.01902 4.36759 5.08994 0.61958 0.77255 0.48576 0.95510
171 3.00626 4.36159 4.73358 4.16154 3.20332 4.25552 4.62807 1.33794 4.03146 1.79617 3.13636 4.31688 4.56871 4.21198 4.15730 3.57827 2.42597 1.80555 5.14943 3.98397 387 - -
2.68618 4.42225 2.77519 2.73123 3.46354 2.40513 3.72494 3.29354 2.67741 2.69355 4.24690 2.90347 2.73739 3.18146 2.89801 2.37887 2.77519 2.98518 4.58477 3.61503
0.01902 4.36759 5.08994 0.61958 0.77255 0.48576 0.95510
172 1.75446 4.52925 3.39114 2.89774 4.14967 3.33099 4.00696 3.54736 2.89403 3.20734 4.04012 3.03061 2.36438 3.21820 3.30051 1.88630 2.89301 2.65558 5.48268 4.21163 388 - -
2.68618 4.42225 2.77519 2.73123 3.46354 2.40513 3.72494 3.29354 2.67741 2.69355 4.24690 2.90347 2.73739 3.18146 2.89801 2.37887 2.77519 2.98518 4.58477 3.61503
0.01902 4.36759 5.08994 0.61958 0.77255 0.48576 0.95510
173 2.64718 3.58970 3.83166 3.43013 2.81115 3.58527 3.28301 2.54013 3.32451 2.29208 3.26549 3.67455 4.10339 3.55974 3.42535 2.86907 2.87921 1.95865 4.75260 2.27736 389 - -
2.68618 4.42225 2.77519 2.73123 3.46354 2.40513 3.72494 3.29354 2.67741 2.69355 4.24690 2.90347 2.73739 3.18146 2.89801 2.37887 2.77519 2.98518 4.58477 3.61503
0.01902 4.36759 5.08994 0.61958 0.77255 0.48576 0.95510
174 2.45312 5.05361 2.70551 2.35471 4.34822 3.29822 3.65686 3.80000 2.41632 3.33682 4.11007 2.52240 3.85411 2.70769 2.32686 2.49201 2.90577 3.41740 5.51727 2.70984 390 - -
2.68619 4.42226 2.77521 2.73124 3.46355 2.40508 3.72496 3.29355 2.67742 2.69356 4.24691 2.90348 2.73741 3.18147 2.89788 2.37888 2.77521 2.98519 4.58478 3.61504
0.07917 2.65988 5.08994 0.38815 1.13417 0.48576 0.95510
175 3.05928 5.01993 2.74371 3.10496 4.98027 0.54409 4.56414 4.56960 3.65427 4.18060 5.07904 3.53180 4.21558 3.84221 4.05911 3.15603 3.51438 4.03919 6.12101 4.96175 392 - -
2.68619 4.42226 2.77520 2.73124 3.46355 2.40513 3.72478 3.29355 2.67741 2.69356 4.24690 2.90347 2.73740 3.18147 2.89801 2.37883 2.77520 2.98519 4.58478 3.61504
0.03813 3.46552 5.08994 0.63533 0.75451 0.48576 0.95510
176 3.76585 5.09668 4.98648 4.65545 3.40807 4.60815 5.16228 2.66140 4.42265 0.44071 3.32239 4.88679 4.97522 4.66557 4.50633 4.28261 4.04609 2.82202 5.43615 4.26530 395 - -
2.68618 4.42225 2.77519 2.73123 3.46354 2.40513 3.72494 3.29354 2.67741 2.69355 4.24690 2.90347 2.73739 3.18146 2.89801 2.37887 2.77519 2.98518 4.58477 3.61503
0.01902 4.36759 5.08994 0.61958 0.77255 0.48576 0.95510
177 3.39714 5.09428 4.16865 4.14229 5.22056 0.25704 5.20174 4.97041 4.38850 4.54829 5.54795 4.33563 4.49699 4.67429 4.54243 3.58728 3.91504 4.40245 6.15571 5.34880 396 - -
2.68618 4.42225 2.77519 2.73123 3.46354 2.40513 3.72494 3.29354 2.67741 2.69355 4.24690 2.90347 2.73739 3.18146 2.89801 2.37887 2.77519 2.98518 4.58477 3.61503
0.01902 4.36759 5.08994 0.61958 0.77255 0.48576 0.95510
178 3.11572 5.37520 2.41505 2.53535 4.79789 3.44822 4.12990 4.45613 3.13733 4.01956 4.91177 0.77548 4.10965 3.34036 3.63980 3.08199 3.46819 4.01729 6.04281 4.60828 397 - -
2.68618 4.42225 2.77519 2.73123 3.46354 2.40513 3.72494 3.29354 2.67741 2.69355 4.24690 2.90347 2.73739 3.18146 2.89801 2.37887 2.77519 2.98518 4.58477 3.61503
0.01902 4.36759 5.08994 0.61958 0.77255 0.48576 0.95510
179 3.66548 5.41101 4.21482 3.65437 4.89915 4.01833 4.31768 4.50316 2.61231 3.92795 4.93800 3.96361 4.51784 3.53387 0.43109 3.74094 3.91086 4.21054 5.79156 4.74622 398 - -
2.68618 4.42225 2.77519 2.73123 3.46354 2.40513 3.72494 3.29354 2.67741 2.69355 4.24690 2.90347 2.73739 3.18146 2.89801 2.37887 2.77519 2.98518 4.58477 3.61503
0.01902 4.36759 5.08994 0.61958 0.77255 0.48576 0.95510
180 3.37808 4.66781 5.15920 4.59767 3.36044 4.70959 5.08447 1.33310 4.46179 1.66391 1.61445 4.78934 4.91261 4.56357 4.55089 4.06541 3.60739 2.05339 5.39371 4.31429 399 - -
2.68618 4.42225 2.77519 2.73123 3.46354 2.40513 3.72494 3.29354 2.67741 2.69355 4.24690 2.90347 2.73739 3.18146 2.89801 2.37887 2.77519 2.98518 4.58477 3.61503
0.01902 4.36759 5.08994 0.61958 0.77255 0.48576 0.95510

181 3.66694 4.93921 5.31727 4.79375 3.25008 4.95693 5.27858 1.99359 4.59956 0.61843 3.04029 5.03822 5.08899 4.67756 4.66875 4.36425 3.89345 2.42226 5.43092 4.35957 400 - -
2.68618 4.42225 2.77519 2.73123 3.46354 2.40513 3.72494 3.29354 2.67741 2.69355 4.24690 2.90347 2.73739 3.18146 2.89801 2.37887 2.77519 2.98518 4.58477 3.61503
0.01902 4.36759 5.08994 0.61958 0.77255 0.48576 0.95510
182 1.91771 4.69486 3.16370 2.46092 4.19600 3.38501 3.87614 3.60678 2.71790 3.23385 4.05455 3.14592 3.59085 3.05246 3.15685 2.05912 1.89670 3.23820 5.48617 4.17908 401 - -
2.68618 4.42225 2.77519 2.73123 3.46354 2.40513 3.72494 3.29354 2.67741 2.69355 4.24690 2.90347 2.73739 3.18146 2.89801 2.37887 2.77519 2.98518 4.58477 3.61503
0.01902 4.36759 5.08994 0.61958 0.77255 0.48576 0.95510
183 2.30500 4.44459 4.71111 4.13388 3.30580 4.27759 4.62094 1.77107 3.99932 1.25182 2.59101 4.31920 4.57368 4.16278 4.12814 3.59675 3.30057 2.21150 5.11664 3.98605 402 - -
2.68618 4.42225 2.77519 2.73123 3.46354 2.40513 3.72494 3.29354 2.67741 2.69355 4.24690 2.90347 2.73739 3.18146 2.89801 2.37887 2.77519 2.98518 4.58477 3.61503
0.01902 4.36759 5.08994 0.61958 0.77255 0.48576 0.95510
184 1.14931 4.35946 3.97958 3.48795 3.85994 3.52958 4.35661 2.57101 3.41410 2.78442 3.77998 3.70662 4.11655 3.70915 3.70759 2.90642 2.58388 2.08735 5.38999 4.17886 403 - -
2.68618 4.42225 2.77519 2.73123 3.46354 2.40513 3.72494 3.29354 2.67741 2.69355 4.24690 2.90347 2.73739 3.18146 2.89801 2.37887 2.77519 2.98518 4.58477 3.61503
0.01902 4.36759 5.08994 0.61958 0.77255 0.48576 0.95510
185 2.26602 4.39775 3.69807 3.52556 4.66758 3.14716 4.60855 4.10144 3.62390 3.82809 4.67625 3.60442 3.94111 3.91025 3.88888 0.66505 2.98205 3.49245 6.02300 4.80169 404 - -
2.68618 4.42225 2.77519 2.73123 3.46354 2.40513 3.72494 3.29354 2.67741 2.69355 4.24690 2.90347 2.73739 3.18146 2.89801 2.37887 2.77519 2.98518 4.58477 3.61503
0.01902 4.36759 5.08994 0.61958 0.77255 0.48576 0.95510
186 1.61091 4.30155 3.75291 3.21144 3.57477 3.30413 4.05544 2.84547 3.14663 2.07496 3.54607 3.51979 4.03240 3.43411 3.45410 2.70807 2.60552 2.24958 5.04299 3.82681 405 - -
2.68618 4.42225 2.77519 2.73123 3.46354 2.40513 3.72494 3.29354 2.67741 2.69355 4.24690 2.90347 2.73739 3.18146 2.89801 2.37887 2.77519 2.98518 4.58477 3.61503
0.01902 4.36759 5.08994 0.61958 0.77255 0.48576 0.95510
187 3.96680 5.22813 4.93070 4.76244 0.45489 4.48565 4.20311 3.46284 4.66611 2.72677 4.13082 4.62019 4.95043 4.70150 4.66152 4.19211 4.27685 3.48583 4.35051 2.67426 406 - -
2.68618 4.42225 2.77519 2.73123 3.46354 2.40513 3.72494 3.29354 2.67741 2.69355 4.24690 2.90347 2.73739 3.18146 2.89801 2.37887 2.77519 2.98518 4.58477 3.61503
0.01902 4.36759 5.08994 0.61958 0.77255 0.48576 0.95510
188 3.76585 5.09668 4.98648 4.65545 3.40807 4.60815 5.16228 2.66140 4.42265 0.44071 3.32239 4.88679 4.97522 4.66557 4.50633 4.28261 4.04609 2.82202 5.43615 4.26530 407 - -
2.68618 4.42225 2.77519 2.73123 3.46354 2.40513 3.72494 3.29354 2.67741 2.69355 4.24690 2.90347 2.73739 3.18146 2.89801 2.37887 2.77519 2.98518 4.58477 3.61503
0.01902 4.36759 5.08994 0.61958 0.77255 0.48576 0.95510
189 4.03870 5.32308 4.60931 4.45436 2.49387 4.43826 3.96720 3.99215 4.30240 3.31100 4.61656 4.38690 4.91067 4.47972 4.37220 4.11426 4.33336 3.88849 4.14795 0.42355 408 - -
2.68618 4.42225 2.77519 2.73123 3.46354 2.40513 3.72494 3.29354 2.67741 2.69355 4.24690 2.90347 2.73739 3.18146 2.89801 2.37887 2.77519 2.98518 4.58477 3.61503
0.01902 4.36759 5.08994 0.61958 0.77255 0.48576 0.95510
190 0.48610 4.52363 4.05151 3.90062 4.63123 3.32935 4.85045 3.83932 3.93244 3.69441 4.69080 3.89759 4.11714 4.22893 4.12566 2.88015 3.19325 3.39969 6.00219 4.87285 409 - -
2.68618 4.42225 2.77519 2.73123 3.46354 2.40513 3.72494 3.29354 2.67741 2.69355 4.24690 2.90347 2.73739 3.18146 2.89801 2.37887 2.77519 2.98518 4.58477 3.61503
0.01902 4.36759 5.08994 0.61958 0.77255 0.48576 0.95510
191 3.53483 4.81497 5.28314 4.70522 3.22397 4.84364 5.15379 2.16908 4.57145 0.83552 2.03396 4.92257 4.97684 4.58358 4.61977 4.19996 3.74886 2.15851 5.34997 4.34065 410 - -
2.68618 4.42225 2.77519 2.73123 3.46354 2.40513 3.72494 3.29354 2.67741 2.69355 4.24690 2.90347 2.73739 3.18146 2.89801 2.37887 2.77519 2.98518 4.58477 3.61503
0.01902 4.36759 5.08994 0.61958 0.77255 0.48576 0.95510
192 3.67264 4.98093 5.19760 4.71405 3.23063 4.86338 5.19389 2.26629 4.47037 0.55750 3.06149 4.95431 5.05217 4.61674 4.55579 4.29935 3.91326 2.53038 5.38491 4.25342 411 - -
2.68618 4.42225 2.77519 2.73123 3.46354 2.40513 3.72494 3.29354 2.67741 2.69355 4.24690 2.90347 2.73739 3.18146 2.89801 2.37887 2.77519 2.98518 4.58477 3.61503

0.01902 4.36759 5.08994 0.61958 0.77255 0.48576 0.95510

193 2.50920 4.41098 3.77438 3.55756 4.46729 3.21907 4.56445 3.73713 3.53598 3.56627 4.47961 3.64559 3.98636 3.88295 3.78099 2.53827 0.72695 3.27682 5.86935 4.64558 412 - -

2.68618 4.42225 2.77519 2.73123 3.46354 2.40513 3.72494 3.29354 2.67741 2.69355 4.24690 2.90347 2.73739 3.18146 2.89801 2.37887 2.77519 2.98518 4.58477 3.61503

0.01902 4.36759 5.08994 0.61958 0.77255 0.48576 0.95510

194 2.92664 5.48848 1.43285 2.01048 4.79625 2.67829 3.48731 4.30171 2.68629 3.77906 4.56481 2.36964 3.94648 2.93819 3.06526 2.84420 3.18379 3.86411 5.92366 4.45921 413 - -

2.68618 4.42225 2.77519 2.73123 3.46354 2.40513 3.72494 3.29354 2.67741 2.69355 4.24690 2.90347 2.73739 3.18146 2.89801 2.37887 2.77519 2.98518 4.58477 3.61503

0.01902 4.36759 5.08994 0.61958 0.77255 0.48576 0.95510

195 3.66548 5.41101 4.21482 3.65437 4.89915 4.01833 4.31768 4.50316 2.61231 3.92795 4.93800 3.96361 4.51784 3.53387 0.43109 3.74094 3.91086 4.21054 5.79156 4.74622 414 - -

2.68618 4.42225 2.77519 2.73123 3.46354 2.40513 3.72494 3.29354 2.67741 2.69355 4.24690 2.90347 2.73739 3.18146 2.89801 2.37887 2.77519 2.98518 4.58477 3.61503

0.01902 4.36759 5.08994 0.61958 0.77255 0.48576 0.95510

196 2.69202 4.45042 4.97842 4.44538 3.31810 4.50895 5.00736 1.35083 4.34006 2.33354 3.53281 4.60311 4.81520 4.55736 4.49114 3.86699 3.39927 1.08402 5.51226 4.31293 415 - -

2.68618 4.42225 2.77519 2.73123 3.46354 2.40513 3.72494 3.29354 2.67741 2.69355 4.24690 2.90347 2.73739 3.18146 2.89801 2.37887 2.77519 2.98518 4.58477 3.61503

0.01902 4.36759 5.08994 0.61958 0.77255 0.48576 0.95510

197 3.52114 4.80030 5.31683 4.72831 2.72328 4.81213 5.09534 2.15139 4.59188 0.84381 2.06621 4.91345 4.94396 4.56414 4.60534 4.16350 3.73056 2.38968 5.27296 4.25853 416 - -

2.68618 4.42225 2.77519 2.73123 3.46354 2.40513 3.72494 3.29354 2.67741 2.69355 4.24690 2.90347 2.73739 3.18146 2.89801 2.37887 2.77519 2.98518 4.58477 3.61503

0.01902 4.36759 5.08994 0.61958 0.77255 0.48576 0.95510

198 3.58661 4.94033 5.08711 4.63310 3.26575 4.71442 5.14891 2.39011 4.39778 0.57181 3.10382 4.85207 4.97991 4.57404 4.49630 4.16117 3.85088 2.39392 5.39533 4.25471 417 - -

2.68618 4.42225 2.77519 2.73123 3.46354 2.40513 3.72494 3.29354 2.67741 2.69355 4.24690 2.90347 2.73739 3.18146 2.89801 2.37887 2.77519 2.98518 4.58477 3.61503

0.01902 4.36759 5.08994 0.61958 0.77255 0.48576 0.95510

199 3.24377 4.55564 4.98621 4.43889 3.02575 4.56147 4.94511 2.04749 4.31836 1.79853 3.10107 4.62156 4.82594 4.48571 4.45136 3.91112 3.48402 0.91324 5.36140 4.17216 418 - -

2.68618 4.42225 2.77519 2.73123 3.46354 2.40513 3.72494 3.29354 2.67741 2.69355 4.24690 2.90347 2.73739 3.18146 2.89801 2.37887 2.77519 2.98518 4.58477 3.61503

0.01902 4.36759 5.08994 0.61958 0.77255 0.48576 0.95510

200 2.70228 5.52647 1.22140 2.13457 4.81063 3.42131 2.50926 4.31973 2.78122 3.81678 4.62830 2.88212 3.98068 3.00638 3.33381 2.90721 3.26066 3.89916 5.97318 4.50378 419 - -

2.68618 4.42225 2.77519 2.73123 3.46354 2.40513 3.72494 3.29354 2.67741 2.69355 4.24690 2.90347 2.73739 3.18146 2.89801 2.37887 2.77519 2.98518 4.58477 3.61503

0.01902 4.36759 5.08994 0.61958 0.77255 0.48576 0.95510

201 2.60186 4.95703 3.00611 2.40178 3.83942 3.34408 3.49905 3.65821 2.43929 2.96013 4.01967 2.81317 2.68877 2.26068 2.53132 2.60718 2.89236 3.06241 5.43868 4.08203 420 - -

2.68618 4.42225 2.77519 2.73123 3.46354 2.40513 3.72494 3.29354 2.67741 2.69355 4.24690 2.90347 2.73739 3.18146 2.89801 2.37887 2.77519 2.98518 4.58477 3.61503

0.01902 4.36759 5.08994 0.61958 0.77255 0.48576 0.95510

202 2.59343 4.94670 2.91625 2.45874 4.22624 2.88697 3.67885 3.65923 2.45406 3.23159 4.02509 2.98607 3.04712 2.81152 2.25284 2.20963 2.80342 3.30585 5.44412 3.23738 421 - -

2.68614 4.42226 2.77520 2.73124 3.46355 2.40506 3.72495 3.29355 2.67742 2.69356 4.24691 2.90348 2.73740 3.18147 2.89802 2.37888 2.77520 2.98519 4.58478 3.61504

0.05312 3.08844 5.08994 0.55697 0.85083 0.48576 0.95510

203 2.60440 5.14700 2.63361 2.23207 4.47613 3.43904 3.43897 3.95181 2.11893 3.44509 4.19295 2.33767 3.50542 2.56737 2.87020 2.63966 2.37484 3.33045 5.58832 4.18555 424 - -

2.68619 4.42226 2.77520 2.73124 3.46354 2.40505 3.72495 3.29355 2.67741 2.69355 4.24690 2.90347 2.73740 3.18147 2.89801 2.37887 2.77520 2.98519 4.58477 3.61504

0.05488 3.05251 5.08994 0.45524 1.00594 0.48576 0.95510

204 3.02571 5.61852 1.34329 1.58241 4.92037 3.00625 3.87508 4.43833 2.82298 3.91305 4.72170 2.86373 3.98581 2.87674 3.40006 2.78489 3.08843 3.99522 6.06290 4.56894 426 - -

2.68618 4.42225 2.77519 2.73123 3.46354 2.40513 3.72494 3.29354 2.67741 2.69355 4.24690 2.90347 2.73739 3.18146 2.89801 2.37887 2.77519 2.98518 4.58477 3.61503
0.01902 4.36759 5.08994 0.61958 0.77255 0.48576 0.95510
205 2.71683 4.13406 4.39068 3.79606 2.80052 3.86327 4.18341 1.81947 3.64245 2.08390 2.11331 3.92294 4.22003 3.57321 3.75378 3.00267 2.84357 2.10178 4.76074 3.58287 427 - -
2.68618 4.42225 2.77519 2.73123 3.46354 2.40513 3.72494 3.29354 2.67741 2.69355 4.24690 2.90347 2.73739 3.18146 2.89801 2.37887 2.77519 2.98518 4.58477 3.61503
0.01902 4.36759 5.08994 0.61958 0.77255 0.48576 0.95510
206 2.17194 5.11977 2.48080 2.28066 4.44233 2.56288 3.69243 3.90403 2.41237 3.42972 4.20202 2.93314 3.87011 2.81157 2.86631 2.02863 2.95487 3.50567 5.60176 4.20894 428 - -
2.68618 4.42225 2.77519 2.73123 3.46354 2.40513 3.72494 3.29354 2.67741 2.69355 4.24690 2.90347 2.73739 3.18146 2.89801 2.37887 2.77519 2.98518 4.58477 3.61503
0.01902 4.36759 5.08994 0.61958 0.77255 0.48576 0.95510
207 2.73573 5.66261 1.15959 1.80804 4.98005 2.67874 3.92073 4.50039 2.91422 3.98262 4.80889 2.86537 4.00817 3.07603 3.51103 2.96616 3.35984 4.05543 6.13983 4.63203 429 - -
2.68618 4.42225 2.77519 2.73123 3.46354 2.40513 3.72494 3.29354 2.67741 2.69355 4.24690 2.90347 2.73739 3.18146 2.89801 2.37887 2.77519 2.98518 4.58477 3.61503
0.01902 4.36759 5.08994 0.61958 0.77255 0.48576 0.95510
208 3.26273 4.78059 4.20627 3.79667 3.15575 4.15012 3.57706 2.83641 3.48561 0.73074 3.44032 4.06402 4.54648 3.90744 3.67195 3.57618 3.51795 2.85752 4.89862 3.47502 430 - -
2.68618 4.42225 2.77519 2.73123 3.46354 2.40513 3.72494 3.29354 2.67741 2.69355 4.24690 2.90347 2.73739 3.18146 2.89801 2.37887 2.77519 2.98518 4.58477 3.61503
0.01902 4.36759 5.08994 0.61958 0.77255 0.48576 0.95510
209 3.78247 5.05964 4.99708 4.67658 0.70144 4.64680 4.09694 2.97469 4.49800 2.02917 3.63520 4.53850 4.96547 4.51368 4.52443 4.08937 4.03627 3.04658 4.23752 2.56558 431 - -
2.68618 4.42225 2.77519 2.73123 3.46354 2.40513 3.72494 3.29354 2.67741 2.69355 4.24690 2.90347 2.73739 3.18146 2.89801 2.37887 2.77519 2.98518 4.58477 3.61503
0.01902 4.36759 5.08994 0.61958 0.77255 0.48576 0.95510
210 3.28827 0.32295 4.86911 4.73977 4.66193 3.78046 5.28405 3.92672 4.58673 3.80000 4.94570 4.60654 4.51962 4.91175 4.61223 3.55704 3.79696 3.62749 5.93137 4.93730 432 - -
2.68618 4.42225 2.77519 2.73123 3.46354 2.40513 3.72494 3.29354 2.67741 2.69355 4.24690 2.90347 2.73739 3.18146 2.89801 2.37887 2.77519 2.98518 4.58477 3.61503
0.01902 4.36759 5.08994 0.61958 0.77255 0.48576 0.95510
211 3.49436 5.61867 2.83110 0.48423 5.04562 3.68015 4.34993 4.61026 3.30511 4.15618 5.14062 3.35048 4.31736 3.57532 3.74473 3.44198 3.81256 4.25293 6.11616 4.88247 433 - -
2.68618 4.42225 2.77519 2.73123 3.46354 2.40513 3.72494 3.29354 2.67741 2.69355 4.24690 2.90347 2.73739 3.18146 2.89801 2.37887 2.77519 2.98518 4.58477 3.61503
0.01902 4.36759 5.08994 0.61958 0.77255 0.48576 0.95510
212 3.54539 5.18809 4.20814 4.13635 5.04512 3.84929 5.14538 4.75467 4.24336 4.30986 5.39830 4.38159 0.27068 4.61398 4.40637 3.73410 4.02880 4.33588 6.06192 5.18068 434 - -
2.68618 4.42225 2.77519 2.73123 3.46354 2.40513 3.72494 3.29354 2.67741 2.69355 4.24690 2.90347 2.73739 3.18146 2.89801 2.37887 2.77519 2.98518 4.58477 3.61503
0.01902 4.36759 5.08994 0.61958 0.77255 0.48576 0.95510
213 3.96680 5.22813 4.93070 4.76244 0.45489 4.48565 4.20311 3.46284 4.66611 2.72677 4.13082 4.62019 4.95043 4.70150 4.66152 4.19211 4.27685 3.48583 4.35051 2.67426 435 - -
2.68618 4.42225 2.77519 2.73123 3.46354 2.40513 3.72494 3.29354 2.67741 2.69355 4.24690 2.90347 2.73739 3.18146 2.89801 2.37887 2.77519 2.98518 4.58477 3.61503
0.01902 4.36759 5.08994 0.61958 0.77255 0.48576 0.95510
214 2.62746 4.68880 3.28311 2.95558 4.12515 3.45173 4.07849 3.51985 2.87658 2.84290 4.10929 3.34665 1.13602 3.09224 3.20148 2.86783 3.09014 3.21668 5.51189 4.21682 436 - -
2.68618 4.42225 2.77519 2.73123 3.46354 2.40513 3.72494 3.29354 2.67741 2.69355 4.24690 2.90347 2.73739 3.18146 2.89801 2.37887 2.77519 2.98518 4.58477 3.61503
0.01902 4.36759 5.08994 0.61958 0.77255 0.48576 0.95510
215 2.55531 5.05898 2.52635 2.41178 4.66943 1.10473 4.02306 4.12351 2.98615 3.71199 4.55084 2.80067 3.98835 3.20901 3.48553 2.86753 3.20105 3.69171 5.92456 4.54261 437 - -
2.68618 4.42225 2.77519 2.73123 3.46354 2.40513 3.72494 3.29354 2.67741 2.69355 4.24690 2.90347 2.73739 3.18146 2.89801 2.37887 2.77519 2.98518 4.58477 3.61503
0.01902 4.36759 5.08994 0.61958 0.77255 0.48576 0.95510

216 2.11399 4.39693 3.64093 3.17948 4.13702 2.98002 4.19277 3.49951 3.14744 3.20876 3.69869 3.44246 3.92342 3.45956 3.49609 2.34267 1.18127 3.10998 5.51416 4.28448 438 - -
2.68613 4.42228 2.77523 2.73110 3.46357 2.40510 3.72498 3.29357 2.67744 2.69358 4.24693 2.90350 2.73743 3.18149 2.89804 2.37890 2.77505 2.98521 4.58480 3.61506
0.04403 3.29921 5.08994 1.06979 0.42020 0.48576 0.95510
217 2.44353 4.38742 3.70844 3.40012 4.52107 3.17009 4.44973 3.90740 3.41497 3.62289 4.45828 3.54111 3.91899 3.72060 3.72054 1.16416 1.46623 3.37041 5.86823 4.65163 445 - -
2.68618 4.42225 2.77519 2.73123 3.46354 2.40513 3.72494 3.29354 2.67741 2.69355 4.24690 2.90347 2.73739 3.18146 2.89801 2.37887 2.77519 2.98518 4.58477 3.61503
0.01902 4.36759 5.08994 0.61958 0.77255 0.48576 0.95510
218 4.28294 5.45469 4.92255 4.76947 3.35081 4.35334 4.64627 4.36823 4.50690 3.66404 4.97164 4.83254 4.91539 4.86500 4.53722 4.48214 4.61453 4.26905 0.29072 3.33196 446 - -
2.68618 4.42225 2.77519 2.73123 3.46354 2.40513 3.72494 3.29354 2.67741 2.69355 4.24690 2.90347 2.73739 3.18146 2.89801 2.37887 2.77519 2.98518 4.58477 3.61503
0.01902 4.36759 5.08994 0.61958 0.77255 0.48576 0.95510
219 2.88411 4.32022 4.38350 3.50535 3.05195 4.02986 4.34233 2.31765 3.67919 1.14404 3.03629 4.01846 4.36244 3.88178 3.85003 3.32796 2.85595 2.08533 4.95190 3.78911 447 - -
2.68618 4.42225 2.77519 2.73123 3.46354 2.40513 3.72494 3.29354 2.67741 2.69355 4.24690 2.90347 2.73739 3.18146 2.89801 2.37887 2.77519 2.98518 4.58477 3.61503
0.01902 4.36759 5.08994 0.61958 0.77255 0.48576 0.95510
220 3.18650 4.63551 4.49922 3.94704 3.16816 4.27209 3.89290 2.56121 3.69243 0.80311 2.73538 4.20340 4.57010 3.98368 3.86476 3.59948 3.41461 2.62606 5.00997 3.76963 448 - -
2.68618 4.42225 2.77519 2.73123 3.46354 2.40513 3.72494 3.29354 2.67741 2.69355 4.24690 2.90347 2.73739 3.18146 2.89801 2.37887 2.77519 2.98518 4.58477 3.61503
0.01902 4.36759 5.08994 0.61958 0.77255 0.48576 0.95510
221 3.54539 5.18809 4.20814 4.13635 5.04512 3.84929 5.14538 4.75467 4.24336 4.30986 5.39830 4.38159 0.27068 4.61398 4.40637 3.73410 4.02880 4.33588 6.06192 5.18068 449 - -
2.68618 4.42225 2.77519 2.73123 3.46354 2.40513 3.72494 3.29354 2.67741 2.69355 4.24690 2.90347 2.73739 3.18146 2.89801 2.37887 2.77519 2.98518 4.58477 3.61503
0.01902 4.36759 5.08994 0.61958 0.77255 0.48576 0.95510
222 2.53433 4.82954 2.95265 2.40890 4.06301 3.07901 3.71770 3.47303 2.47649 2.24696 3.91208 3.04085 2.47306 2.87932 2.89569 2.53899 2.90589 3.16058 5.34631 4.02070 450 - -
2.68620 4.42227 2.77522 2.73126 3.46356 2.40499 3.72497 3.29356 2.67738 2.69357 4.24692 2.90349 2.73742 3.18149 2.89789 2.37889 2.77522 2.98521 4.58479 3.61505
0.09024 2.52409 5.08994 0.59340 0.80396 0.48576 0.95510
223 2.94539 5.48688 1.31285 2.05629 4.79477 2.52497 3.82163 4.29385 2.59835 3.77914 4.57353 2.88234 3.95832 2.95754 2.89807 2.86415 3.20451 3.86441 5.92935 4.47062 454 - -
2.68618 4.42225 2.77519 2.73123 3.46354 2.40513 3.72494 3.29354 2.67741 2.69355 4.24690 2.90347 2.73739 3.18146 2.89801 2.37887 2.77519 2.98518 4.58477 3.61503
0.01902 4.36759 5.08994 0.61958 0.77255 0.48576 0.95510
224 3.96680 5.22813 4.93070 4.76244 0.45489 4.48565 4.20311 3.46284 4.66611 2.72677 4.13082 4.62019 4.95043 4.70150 4.66152 4.19211 4.27685 3.48583 4.35051 2.67426 455 - -
2.68618 4.42225 2.77519 2.73123 3.46354 2.40513 3.72494 3.29354 2.67741 2.69355 4.24690 2.90347 2.73739 3.18146 2.89801 2.37887 2.77519 2.98518 4.58477 3.61503
0.01902 4.36759 5.08994 0.61958 0.77255 0.48576 0.95510
225 2.69516 4.58003 3.58740 3.27868 4.12198 3.42085 4.31492 3.40694 3.18481 2.72088 4.15196 3.55711 0.96803 3.60264 3.47330 2.87505 2.91883 3.11648 5.59089 4.30884 456 - -
2.68618 4.42225 2.77519 2.73123 3.46354 2.40513 3.72494 3.29354 2.67741 2.69355 4.24690 2.90347 2.73739 3.18146 2.89801 2.37887 2.77519 2.98518 4.58477 3.61503
0.03426 4.36759 3.86318 0.61958 0.77255 0.48576 0.95510
226 2.97189 4.36519 4.61588 4.02590 3.01917 4.14243 3.94665 2.14154 3.88131 1.08095 2.79067 4.18771 4.44818 4.02963 3.99108 3.45097 3.19856 2.11882 4.93824 3.79314 457 - -
2.68618 4.42225 2.77519 2.73123 3.46354 2.40513 3.72494 3.29354 2.67741 2.69355 4.24690 2.90347 2.73739 3.18146 2.89801 2.37887 2.77519 2.98518 4.58477 3.61503
0.01931 4.35264 5.07499 0.61958 0.77255 0.50776 0.92091
227 2.34183 4.82574 3.07539 2.45803 4.05710 3.49292 3.50013 3.46845 2.39387 2.86351 3.55119 2.62904 3.88247 2.85442 2.78030 2.63003 2.08933 3.06350 5.33353 4.00667 458 - -
2.68618 4.42225 2.77519 2.73123 3.46354 2.40513 3.72494 3.29354 2.67741 2.69355 4.24690 2.90347 2.73739 3.18146 2.89801 2.37887 2.77519 2.98518 4.58477 3.61503

0.16454 4.35264 1.97443 0.61958 0.77255 0.50776 0.92091
228 2.65547 5.09595 2.11721 2.32834 4.40680 3.06024 3.43654 3.87406 2.31520 3.38634 4.14923 2.51065 3.81225 2.65533 2.77670 2.41340 2.88983 3.46906 5.54650 3.52553 459 - -
2.68618 4.42225 2.77519 2.73123 3.46354 2.40513 3.72494 3.29354 2.67741 2.69355 4.24690 2.90347 2.73739 3.18146 2.89801 2.37887 2.77519 2.98518 4.58477 3.61503
0.14026 4.21040 2.15393 0.61958 0.77255 0.37969 1.15225
229 2.63136 4.76905 3.07099 2.51311 3.76908 3.35051 3.29760 3.39761 2.49940 2.69369 3.46726 2.54602 3.64838 2.18841 2.95272 2.61628 2.86257 3.09173 5.28034 3.40585 460 - -
2.68619 4.42226 2.77520 2.73124 3.46355 2.40514 3.72495 3.29355 2.67742 2.69356 4.24690 2.90347 2.73740 3.18147 2.89801 2.37888 2.77513 2.98519 4.58478 3.61486
0.04559 3.27618 4.99234 0.63727 0.75234 0.50031 0.93228
230 2.49285 4.17693 3.66183 3.16354 2.34593 3.69628 3.98125 2.55562 3.09601 1.86612 3.28844 3.59009 4.06896 3.15958 3.46029 2.96846 2.76146 2.43536 4.10419 3.15430 463 - -
2.68618 4.42225 2.77519 2.73123 3.46354 2.40513 3.72494 3.29354 2.67741 2.69355 4.24690 2.90347 2.73739 3.18146 2.89801 2.37887 2.77519 2.98518 4.58477 3.61503
0.14802 4.32413 2.08473 0.61958 0.77255 0.44898 1.01688
231 2.57029 5.03269 2.24280 2.36554 4.33463 2.92066 3.61476 3.79087 2.32069 3.31923 4.08618 2.79127 3.81426 2.60397 2.62064 2.55976 2.50652 3.40021 4.90105 3.94249 464 - -
2.68618 4.42225 2.77519 2.73123 3.46354 2.40513 3.72494 3.29354 2.67741 2.69355 4.24690 2.90347 2.73739 3.18146 2.89801 2.37887 2.77519 2.98518 4.58477 3.61503
0.02147 4.24780 4.97015 0.61958 0.77255 0.39915 1.11137
232 2.70443 5.16364 2.53638 2.17078 4.48532 2.53233 3.66499 3.95768 2.42878 3.46095 4.21924 2.74745 3.85684 2.68646 2.71230 1.96791 2.94008 3.39338 5.61386 4.21073 465 - -
2.68618 4.42225 2.77519 2.73123 3.46354 2.40513 3.72494 3.29354 2.67741 2.69355 4.24690 2.90347 2.73739 3.18146 2.89801 2.37887 2.77519 2.98518 4.58477 3.61503
0.04244 4.36759 3.54480 0.61958 0.77255 0.48576 0.95510
233 3.54790 4.83280 4.91880 4.48828 1.07813 4.53220 3.95648 2.95304 4.31852 1.55502 3.53733 4.36827 4.80737 4.32159 4.34871 3.87655 3.76654 2.97590 4.12794 2.20010 466 - -
2.68618 4.42225 2.77519 2.73123 3.46354 2.40513 3.72494 3.29354 2.67741 2.69355 4.24690 2.90347 2.73739 3.18146 2.89801 2.37887 2.77519 2.98518 4.58477 3.61503
0.01947 4.34462 5.06696 0.61958 0.77255 0.51923 0.90381
234 2.68551 5.06814 2.70455 2.39398 4.37481 2.70628 3.37837 3.83027 2.44682 3.36626 4.14146 2.14513 3.85630 2.79177 2.93390 2.20663 2.56852 3.44315 5.54640 3.91705 467 - -
2.68618 4.42225 2.77519 2.73123 3.46354 2.40513 3.72494 3.29354 2.67741 2.69355 4.24690 2.90347 2.73739 3.18146 2.89801 2.37887 2.77519 2.98518 4.58477 3.61503
0.01947 4.34462 5.06696 0.61958 0.77255 0.51923 0.90381
235 2.47755 4.89588 3.03151 2.41690 4.14704 3.09218 3.67496 3.27651 1.99215 3.06926 3.75710 2.90900 3.62105 2.52129 2.78403 2.67790 2.88577 2.65103 5.38858 4.04530 468 - -
2.68618 4.42225 2.77519 2.73123 3.46354 2.40513 3.72494 3.29354 2.67741 2.69355 4.24690 2.90347 2.73739 3.18146 2.89801 2.37887 2.77519 2.98518 4.58477 3.61503
0.01947 4.34462 5.06696 0.61958 0.77255 0.51923 0.90381
236 2.60458 5.13994 2.47166 1.87227 4.47006 2.94469 3.28582 3.94615 2.27011 3.43840 4.18539 2.83097 3.83046 2.72961 2.62613 2.47591 2.89502 3.52270 4.82105 4.17782 469 - -
2.68618 4.42225 2.77519 2.73123 3.46354 2.40513 3.72494 3.29354 2.67741 2.69355 4.24690 2.90347 2.73739 3.18146 2.89801 2.37887 2.77519 2.98518 4.58477 3.61503
0.01947 4.34462 5.06696 0.61958 0.77255 0.51923 0.90381
237 2.35191 4.55382 3.31035 2.79643 3.95044 3.41972 3.89442 3.34248 2.78379 3.01043 3.86077 3.22307 3.57579 3.11562 3.19299 1.61972 2.16822 3.02731 5.30760 3.27028 470 - -
2.68618 4.42225 2.77519 2.73123 3.46354 2.40513 3.72494 3.29354 2.67741 2.69355 4.24690 2.90347 2.73739 3.18146 2.89801 2.37887 2.77519 2.98518 4.58477 3.61503
0.01947 4.34462 5.06696 0.61958 0.77255 0.51923 0.90381
238 2.44405 5.00395 2.95828 2.30712 4.30391 3.45613 3.67269 3.74504 2.37522 3.29815 4.08349 2.75678 2.23799 2.79854 2.64984 2.50066 2.57800 3.37560 5.49206 4.12791 471 - -
2.68618 4.42225 2.77519 2.73123 3.46354 2.40513 3.72494 3.29354 2.67741 2.69355 4.24690 2.90347 2.73739 3.18146 2.89801 2.37887 2.77519 2.98518 4.58477 3.61503
0.01947 4.34462 5.06696 0.61958 0.77255 0.51923 0.90381
239 2.81429 5.21805 2.97985 1.63220 4.56732 3.52411 3.20076 4.01214 2.29744 3.49398 4.27458 3.00170 3.92962 2.55862 1.98062 2.78215 2.92569 3.61628 5.61487 4.26689 472 - -

2.68618 4.42225 2.77519 2.73123 3.46354 2.40513 3.72494 3.29354 2.67741 2.69355 4.24690 2.90347 2.73739 3.18146 2.89801 2.37887 2.77519 2.98518 4.58477 3.61503
0.01947 4.34462 5.06696 0.61958 0.77255 0.51923 0.90381
240 2.43723 1.89530 3.94228 3.52356 4.14812 3.21867 4.38735 3.53185 3.39952 3.26174 4.12969 3.60931 3.93041 3.72449 3.36411 1.18321 2.89550 3.11450 5.55773 4.35106 473 - -
2.68618 4.42225 2.77519 2.73123 3.46354 2.40513 3.72494 3.29354 2.67741 2.69355 4.24690 2.90347 2.73739 3.18146 2.89801 2.37887 2.77519 2.98518 4.58477 3.61503
0.01947 4.34462 5.06696 0.61958 0.77255 0.51923 0.90381
241 3.62235 5.00445 4.46800 4.08506 2.25926 4.39650 3.35505 3.52260 3.84651 2.30951 4.11057 4.07756 4.73812 4.05600 3.98949 3.75050 3.84729 3.39581 3.92179 0.77578 474 - -
2.68618 4.42225 2.77519 2.73123 3.46354 2.40513 3.72494 3.29354 2.67741 2.69355 4.24690 2.90347 2.73739 3.18146 2.89801 2.37887 2.77519 2.98518 4.58477 3.61503
0.01947 4.34462 5.06696 0.61958 0.77255 0.51923 0.90381
242 2.47488 4.56981 3.32951 2.90929 4.18591 1.25912 4.02199 3.55489 2.72788 3.22994 4.07970 3.28908 3.93762 3.24025 3.03340 2.72510 2.77353 2.85981 5.50800 4.24789 475 - -
2.68618 4.42225 2.77519 2.73123 3.46354 2.40513 3.72494 3.29354 2.67741 2.69355 4.24690 2.90347 2.73739 3.18146 2.89801 2.37887 2.77519 2.98518 4.58477 3.61503
0.01947 4.34462 5.06696 0.61958 0.77255 0.51923 0.90381
243 2.63420 4.96895 2.79533 2.46883 4.26664 3.45337 3.74492 3.69485 2.48947 3.28306 4.09455 1.81309 3.90110 2.89390 3.01680 2.73635 2.02701 3.34700 5.50497 3.87615 476 - -
2.68618 4.42225 2.77519 2.73123 3.46354 2.40513 3.72494 3.29354 2.67741 2.69355 4.24690 2.90347 2.73739 3.18146 2.89801 2.37887 2.77519 2.98518 4.58477 3.61503
0.01947 4.34462 5.06696 0.61958 0.77255 0.51923 0.90381
244 2.82543 4.57673 4.63793 4.07794 3.29672 4.30775 4.67517 2.34946 3.92322 1.49503 1.33456 4.32069 4.61196 4.12876 4.09179 3.63882 3.39768 2.27219 5.19635 4.07995 477 - -
2.68618 4.42225 2.77519 2.73123 3.46354 2.40513 3.72494 3.29354 2.67741 2.69355 4.24690 2.90347 2.73739 3.18146 2.89801 2.37887 2.77519 2.98518 4.58477 3.61503
0.01947 4.34462 5.06696 0.61958 0.77255 0.51923 0.90381
245 2.68087 4.35889 4.02790 3.47551 3.41758 3.81181 4.18026 2.59771 3.25352 1.15634 3.36802 3.75390 4.21884 3.61061 3.14401 3.02282 3.05003 2.25934 5.00750 3.80943 478 - -
2.68618 4.42225 2.77519 2.73123 3.46354 2.40513 3.72494 3.29354 2.67741 2.69355 4.24690 2.90347 2.73739 3.18146 2.89801 2.37887 2.77519 2.98518 4.58477 3.61503
0.01947 4.34462 5.06696 0.61958 0.77255 0.51923 0.90381
246 2.62306 5.15794 2.96433 2.27385 4.49468 3.16933 3.65624 3.94791 1.54482 3.44308 4.21153 2.79393 3.88451 2.44384 2.67188 2.71102 2.96388 3.37105 5.58096 4.21350 479 - -
2.68618 4.42225 2.77519 2.73123 3.46354 2.40513 3.72494 3.29354 2.67741 2.69355 4.24690 2.90347 2.73739 3.18146 2.89801 2.37887 2.77519 2.98518 4.58477 3.61503
0.01947 4.34462 5.06696 0.61958 0.77255 0.46539 0.98857
247 2.47654 5.13997 2.78793 2.40816 4.45853 3.46100 3.69140 3.91482 2.32273 3.43509 4.21113 1.69100 3.88577 2.69169 2.64655 2.48830 2.97420 3.52129 5.59978 4.21760 480 - -
2.68618 4.42225 2.77519 2.73123 3.46354 2.40513 3.72494 3.29354 2.67741 2.69355 4.24690 2.90347 2.73739 3.18146 2.89801 2.37887 2.77519 2.98518 4.58477 3.61503
0.01902 4.36759 5.08994 0.61958 0.77255 0.48576 0.95510
248 2.71455 5.16646 2.83645 2.39943 4.49978 2.98332 2.52422 3.96552 1.78913 3.45798 4.21618 2.67743 3.86726 2.65308 2.67135 2.58551 2.94367 3.55217 5.59719 4.21046 481 - -
2.68618 4.42225 2.77519 2.73123 3.46354 2.40513 3.72494 3.29354 2.67741 2.69355 4.24690 2.90347 2.73739 3.18146 2.89801 2.37887 2.77519 2.98518 4.58477 3.61503
0.03642 4.36759 3.76885 0.61958 0.77255 0.48576 0.95510
249 2.05232 4.59347 3.25656 2.55881 3.77819 3.38380 3.78114 3.15500 2.41726 2.65014 3.69124 3.17299 3.93885 3.00652 2.97664 2.67224 2.76287 2.13724 5.14896 3.87310 482 - -
2.68618 4.42225 2.77519 2.73123 3.46354 2.40513 3.72494 3.29354 2.67741 2.69355 4.24690 2.90347 2.73739 3.18146 2.89801 2.37887 2.77519 2.98518 4.58477 3.61503
0.01935 4.35053 5.07288 0.61958 0.77255 0.51080 0.91633
250 2.83676 4.27054 4.38100 3.81903 3.43042 3.43286 4.38372 1.26819 3.69218 2.12386 3.37662 4.01803 3.94579 3.92977 3.87362 3.30917 3.08446 1.78407 5.04187 3.84715 483 - -
2.68618 4.42225 2.77519 2.73123 3.46354 2.40513 3.72494 3.29354 2.67741 2.69355 4.24690 2.90347 2.73739 3.18146 2.89801 2.37887 2.77519 2.98518 4.58477 3.61503
0.01935 4.35053 5.07288 0.61958 0.77255 0.47042 0.98013

251 2.53933 5.03494 2.88189 2.41700 4.34577 2.92698 3.66196 3.79663 2.36134 3.33519 4.10856 2.20215 3.85329 2.77990 2.52969 2.46910 2.38128 3.27429 5.51640 4.14055 484 - -
2.68618 4.42225 2.77519 2.73123 3.46354 2.40513 3.72494 3.29354 2.67741 2.69355 4.24690 2.90347 2.73739 3.18146 2.89801 2.37887 2.77519 2.98518 4.58477 3.61503
0.01902 4.36759 5.08994 0.61958 0.77255 0.48576 0.95510
252 2.52486 5.04558 2.73440 2.36023 4.34740 3.13325 3.50383 3.79995 2.41968 3.33689 4.10887 2.68261 3.67850 2.77403 2.82068 2.01757 2.32606 3.26963 5.51750 4.13823 485 - -
2.68618 4.42225 2.77519 2.73123 3.46354 2.40513 3.72494 3.29354 2.67741 2.69355 4.24690 2.90347 2.73739 3.18146 2.89801 2.37887 2.77519 2.98518 4.58477 3.61503
0.08742 4.36759 2.64473 0.61958 0.77255 0.48576 0.95510
253 2.61696 5.05714 2.06778 2.33412 4.35903 3.42619 3.67310 3.80960 2.39753 3.35338 4.13483 2.69241 3.85227 2.79767 2.94754 2.50849 2.16653 3.11025 5.54076 4.15841 486 - -
2.68618 4.42225 2.77519 2.73123 3.46354 2.40513 3.72494 3.29354 2.67741 2.69355 4.24690 2.90347 2.73739 3.18146 2.89801 2.37887 2.77519 2.98518 4.58477 3.61503
0.04005 4.30053 3.66139 0.61958 0.77255 0.57851 0.82265
254 2.20613 4.76019 2.73562 2.54523 4.13959 2.77970 3.74185 3.55846 2.55654 3.09074 3.97648 3.03940 3.51362 2.89821 3.01449 2.43871 2.05690 3.04391 5.40660 4.07887 487 - -
2.68618 4.42225 2.77519 2.73123 3.46354 2.40513 3.72494 3.29354 2.67741 2.69355 4.24690 2.90347 2.73739 3.18146 2.89801 2.37887 2.77519 2.98518 4.58477 3.61503
0.16096 4.28123 2.00360 0.61958 0.77255 0.60262 0.79269
255 2.08424 4.76380 2.61132 2.47318 4.15276 3.21117 3.69878 3.57774 2.51298 3.17476 3.98113 2.83541 3.33426 2.85021 2.97728 2.53402 2.21364 3.08438 5.40587 4.06845 488 - -
2.68618 4.42225 2.77519 2.73123 3.46354 2.40513 3.72494 3.29354 2.67741 2.69355 4.24690 2.90347 2.73739 3.18146 2.89801 2.37887 2.77519 2.98518 4.58477 3.61503
0.02384 4.14411 4.86646 0.61958 0.77255 0.63702 0.75261
256 2.25358 5.05473 2.84158 1.90688 4.36015 2.92838 3.44018 3.81649 2.38216 3.34339 4.11584 2.78047 3.81817 2.54673 2.73173 2.56968 2.79973 3.42593 5.51524 4.13102 489 - -
2.68613 4.42255 2.77488 2.73145 3.46384 2.40500 3.72525 3.29384 2.67764 2.69371 4.24695 2.90355 2.73705 3.18176 2.89823 2.37862 2.77492 2.98495 4.58433 3.61533
0.80615 1.09474 1.51961 1.48809 0.25593 0.61449 0.77852
257 2.03426 4.59334 3.01167 2.53922 4.03012 2.94149 2.89109 3.44373 2.56077 3.06902 3.89473 3.00730 3.19413 2.90379 2.99579 2.31855 2.70365 3.09267 5.32415 4.01649 500 - -
2.68618 4.42225 2.77519 2.73123 3.46354 2.40513 3.72494 3.29354 2.67741 2.69355 4.24690 2.90347 2.73739 3.18146 2.89801 2.37887 2.77519 2.98518 4.58477 3.61503
0.02723 4.01268 4.73503 0.61958 0.77255 0.49069 0.94727
258 2.39469 4.95285 2.85205 2.34143 4.23692 3.00064 3.63529 3.67660 2.40618 3.23637 4.02237 2.93380 3.62027 2.42066 2.71139 2.14198 2.86546 2.92762 5.43757 4.07336 501 - -
2.68618 4.42225 2.77519 2.73123 3.46354 2.40513 3.72494 3.29354 2.67741 2.69355 4.24690 2.90347 2.73739 3.18146 2.89801 2.37887 2.77519 2.98518 4.58477 3.61503
0.02159 4.24217 4.96451 0.61958 0.77255 0.64840 0.73999
259 2.48598 4.57411 3.21481 2.66660 3.77939 3.50293 3.75776 2.43776 2.63369 2.71956 3.68862 3.13878 3.05646 2.47679 3.04771 2.21915 2.86147 2.88357 5.14597 3.86728 502 - -
2.68618 4.42225 2.77519 2.73123 3.46354 2.40513 3.72494 3.29354 2.67741 2.69355 4.24690 2.90347 2.73739 3.18146 2.89801 2.37887 2.77519 2.98518 4.58477 3.61503
0.04251 4.24217 3.60303 0.61958 0.77255 0.39604 1.11773
260 2.64365 4.31911 3.59845 2.93012 3.34640 3.45444 3.90304 2.15174 2.97308 2.05741 3.27898 3.09400 3.10747 3.26725 2.99005 2.81100 2.79993 2.48909 4.90729 3.68137 503 - -
2.68618 4.42225 2.77519 2.73123 3.46354 2.40513 3.72494 3.29354 2.67741 2.69355 4.24690 2.90347 2.73739 3.18146 2.89801 2.37887 2.77519 2.98518 4.58477 3.61503
0.01937 4.34956 5.07191 0.61958 0.77255 0.46959 0.98152
261 2.95448 4.75723 3.77010 3.46552 4.00677 3.63380 4.43331 3.40041 3.35097 2.34516 4.15084 3.76285 0.82650 3.77725 3.61893 3.14637 3.34481 3.19127 5.51577 4.22587 504 - -
2.68618 4.42225 2.77519 2.73123 3.46354 2.40513 3.72494 3.29354 2.67741 2.69355 4.24690 2.90347 2.73739 3.18146 2.89801 2.37887 2.77519 2.98518 4.58477 3.61503
0.01902 4.36759 5.08994 0.61958 0.77255 0.48576 0.95510
262 2.02201 4.62427 3.27635 2.75340 4.07433 3.40937 3.88066 3.47207 2.72646 3.11876 3.56845 3.19270 2.14683 3.07463 2.99954 1.94422 2.90003 3.13174 5.39429 4.10617 505 - -
2.68618 4.42225 2.77519 2.73123 3.46354 2.40513 3.72494 3.29354 2.67741 2.69355 4.24690 2.90347 2.73739 3.18146 2.89801 2.37887 2.77519 2.98518 4.58477 3.61503

0.01902 4.36759 5.08994 0.61958 0.77255 0.48576 0.95510
263 3.25116 4.65248 4.47006 3.98625 1.74076 4.20636 2.67788 3.16219 3.86265 2.74889 3.78734 4.01169 3.80674 3.98026 4.00324 3.51326 3.47625 2.75920 3.30956 1.29148 506 - -
2.68618 4.42225 2.77519 2.73123 3.46354 2.40513 3.72494 3.29354 2.67741 2.69355 4.24690 2.90347 2.73739 3.18146 2.89801 2.37887 2.77519 2.98518 4.58477 3.61503
0.01902 4.36759 5.08994 0.61958 0.77255 0.48576 0.95510
264 2.03660 4.36485 4.45959 3.89315 3.40704 4.10221 4.48041 2.24381 3.77546 1.52146 2.97380 4.11215 4.44994 3.99661 3.96107 3.41616 3.18056 1.60974 5.10789 3.93652 507 - -
2.68618 4.42225 2.77519 2.73123 3.46354 2.40513 3.72494 3.29354 2.67741 2.69355 4.24690 2.90347 2.73739 3.18146 2.89801 2.37887 2.77519 2.98518 4.58477 3.61503
0.01902 4.36759 5.08994 0.61958 0.77255 0.48576 0.95510
265 3.23797 2.42422 4.64347 4.17686 2.03826 4.21472 3.84003 3.04066 4.01744 2.63750 3.71731 4.11454 4.57193 4.10451 4.09745 3.54665 3.47706 2.88935 4.08646 1.11731 508 - -
2.68618 4.42225 2.77519 2.73123 3.46354 2.40513 3.72494 3.29354 2.67741 2.69355 4.24690 2.90347 2.73739 3.18146 2.89801 2.37887 2.77519 2.98518 4.58477 3.61503
0.01902 4.36759 5.08994 0.61958 0.77255 0.48576 0.95510
266 2.81703 4.34590 4.56744 3.98340 3.28809 4.13108 4.45991 1.95282 3.83889 1.21289 2.97936 4.16234 4.45051 4.02517 3.55190 3.44040 3.17988 1.92073 5.00775 3.85656 509 - -
2.68618 4.42225 2.77519 2.73123 3.46354 2.40513 3.72494 3.29354 2.67741 2.69355 4.24690 2.90347 2.73739 3.18146 2.89801 2.37887 2.77519 2.98518 4.58477 3.61503
0.01902 4.36759 5.08994 0.61958 0.77255 0.48576 0.95510
267 3.07077 5.01544 2.91357 2.89425 2.96255 3.77873 1.50214 3.65425 2.95009 3.17762 4.14353 3.33806 4.20936 3.27585 3.33957 3.10866 3.30904 3.39963 4.54367 1.86068 510 - -
2.68618 4.42225 2.77519 2.73123 3.46354 2.40513 3.72494 3.29354 2.67741 2.69355 4.24690 2.90347 2.73739 3.18146 2.89801 2.37887 2.77519 2.98518 4.58477 3.61503
0.01902 4.36759 5.08994 0.61958 0.77255 0.48576 0.95510
268 2.96564 4.43931 4.32450 3.75961 3.32638 4.07510 4.39268 2.37271 3.60398 1.00722 3.07547 3.43070 4.41674 3.86740 3.81150 3.38243 3.20436 2.17388 5.05378 3.86920 511 - -
2.68618 4.42225 2.77519 2.73123 3.46354 2.40513 3.72494 3.29354 2.67741 2.69355 4.24690 2.90347 2.73739 3.18146 2.89801 2.37887 2.77519 2.98518 4.58477 3.61503
0.01902 4.36759 5.08994 0.61958 0.77255 0.48576 0.95510
269 2.45407 4.79337 2.19691 2.37103 4.01497 3.50475 3.71790 2.52460 2.53869 2.82064 3.87147 3.05485 3.89644 2.88469 2.81649 2.63076 2.89439 2.89081 5.31221 3.99252 512 - -
2.68618 4.42225 2.77519 2.73123 3.46354 2.40513 3.72494 3.29354 2.67741 2.69355 4.24690 2.90347 2.73739 3.18146 2.89801 2.37887 2.77519 2.98518 4.58477 3.61503
0.01902 4.36759 5.08994 0.61958 0.77255 0.48576 0.95510
270 2.76433 4.93494 3.10944 2.58148 4.12015 2.89206 1.73641 3.60160 2.43339 3.03164 4.01714 3.08479 3.94871 2.63061 2.69770 2.79610 2.99037 3.28478 5.37002 3.79184 513 - -
2.68618 4.42225 2.77519 2.73123 3.46354 2.40513 3.72494 3.29354 2.67741 2.69355 4.24690 2.90347 2.73739 3.18146 2.89801 2.37887 2.77519 2.98518 4.58477 3.61503
0.03453 4.36759 3.85110 0.61958 0.77255 0.48576 0.95510
271 2.63797 5.07192 1.57969 2.40782 3.99419 2.86303 3.72436 3.61846 2.53301 3.39161 4.18188 2.62707 3.88406 2.85652 3.02515 2.71429 2.88260 3.45674 5.58574 4.20248 514 - -
2.68618 4.42225 2.77519 2.73123 3.46354 2.40513 3.72494 3.29354 2.67741 2.69355 4.24690 2.90347 2.73739 3.18146 2.89801 2.37887 2.77519 2.98518 4.58477 3.61503
0.01931 4.35238 5.07473 0.61958 0.77255 0.50813 0.92035
272 2.44337 4.05366 3.49985 2.93652 3.51913 3.61918 3.65856 2.91796 2.86685 2.52021 3.51480 3.34678 4.00505 3.08741 3.08912 2.49607 2.89337 2.68850 4.96113 1.88496 515 - -
2.68618 4.42225 2.77519 2.73123 3.46354 2.40513 3.72494 3.29354 2.67741 2.69355 4.24690 2.90347 2.73739 3.18146 2.89801 2.37887 2.77519 2.98518 4.58477 3.61503
0.01931 4.35238 5.07473 0.61958 0.77255 0.50813 0.92035
273 2.67227 5.12725 2.26034 2.24467 4.44847 3.19965 3.63912 3.91878 2.38853 3.17329 4.17641 2.75904 3.83924 2.58427 2.55163 2.49744 2.32951 3.50545 5.57399 4.17586 516 - -
2.68618 4.42225 2.77519 2.73123 3.46354 2.40513 3.72494 3.29354 2.67741 2.69355 4.24690 2.90347 2.73739 3.18146 2.89801 2.37887 2.77519 2.98518 4.58477 3.61503
0.01931 4.35238 5.07473 0.61958 0.77255 0.47203 0.97747
274 2.65847 4.89974 2.08347 2.42662 3.03817 3.27753 3.68166 3.57312 2.41554 3.16321 3.70161 3.00026 3.66263 2.73785 2.71072 2.62059 2.88982 3.14211 5.39391 3.44962 517 - -

2.68609 4.42227 2.77521 2.73125 3.46355 2.40510 3.72496 3.29356 2.67742 2.69357 4.24691 2.90342 2.73736 3.18148 2.89802 2.37888 2.77521 2.98520 4.58479 3.61505

0.05312 3.08844 5.08994 0.72317 0.66400 0.48576 0.95510

275 2.51758 4.85297 2.91711 2.45590 3.85584 3.34912 2.52346 3.50438 2.49923 2.92272 3.92474 2.88012 3.88129 2.70204 2.96158 2.44321 2.89167 3.18363 5.35564 2.72745 521 - -

2.68618 4.42225 2.77519 2.73123 3.46354 2.40513 3.72494 3.29354 2.67741 2.69355 4.24690 2.90347 2.73739 3.18146 2.89801 2.37887 2.77519 2.98518 4.58477 3.61503

0.01902 4.36759 5.08994 0.61958 0.77255 0.48576 0.95510

276 3.08957 5.62975 0.94131 2.29853 4.96164 3.40583 3.94461 4.48281 2.94209 3.97860 4.81806 2.72613 4.02154 2.72464 3.52515 2.81528 3.16133 4.04622 6.13912 4.63998 522 - -

2.68618 4.42225 2.77519 2.73123 3.46354 2.40513 3.72494 3.29354 2.67741 2.69355 4.24690 2.90347 2.73739 3.18146 2.89801 2.37887 2.77519 2.98518 4.58477 3.61503

0.01902 4.36759 5.08994 0.61958 0.77255 0.48576 0.95510

277 2.82283 5.25419 2.72378 2.35599 4.60565 3.51393 3.69447 4.05726 1.30042 3.53425 4.31048 2.88464 3.66194 2.70077 2.56589 2.78412 3.04692 3.65208 5.65471 4.29288 523 - -

2.68618 4.42225 2.77519 2.73123 3.46354 2.40513 3.72494 3.29354 2.67741 2.69355 4.24690 2.90347 2.73739 3.18146 2.89801 2.37887 2.77519 2.98518 4.58477 3.61503

0.01902 4.36759 5.08994 0.61958 0.77255 0.48576 0.95510

278 2.59356 4.17181 4.01774 3.43454 2.69704 3.74665 3.78123 2.61209 3.31198 1.72366 2.21628 3.47537 4.11608 3.56124 3.13792 3.02762 2.89651 2.42175 4.76285 3.55595 524 - -

2.68618 4.42225 2.77519 2.73123 3.46354 2.40513 3.72494 3.29354 2.67741 2.69355 4.24690 2.90347 2.73739 3.18146 2.89801 2.37887 2.77519 2.98518 4.58477 3.61503

0.01902 4.36759 5.08994 0.61958 0.77255 0.48576 0.95510

279 2.94869 4.55287 4.55171 4.04459 1.07983 4.18090 4.34490 2.41060 3.89849 2.00876 3.34233 4.19332 4.54878 4.09248 4.05427 3.52554 3.16765 2.45937 4.76257 3.31754 525 - -

2.68618 4.42225 2.77519 2.73123 3.46354 2.40513 3.72494 3.29354 2.67741 2.69355 4.24690 2.90347 2.73739 3.18146 2.89801 2.37887 2.77519 2.98518 4.58477 3.61503

0.01902 4.36759 5.08994 0.61958 0.77255 0.48576 0.95510

280 3.90805 5.10366 5.00108 4.71282 0.72590 4.67680 3.72167 3.49848 4.53531 2.80819 4.09930 4.37322 4.97335 4.44330 4.51304 4.04840 4.12953 3.21995 3.84398 1.68352 526 - -

2.68618 4.42225 2.77519 2.73123 3.46354 2.40513 3.72494 3.29354 2.67741 2.69355 4.24690 2.90347 2.73739 3.18146 2.89801 2.37887 2.77519 2.98518 4.58477 3.61503

0.01902 4.36759 5.08994 0.61958 0.77255 0.48576 0.95510

281 3.28827 0.32295 4.86911 4.73977 4.66193 3.78046 5.28405 3.92672 4.58673 3.80000 4.94570 4.60654 4.51962 4.91175 4.61223 3.55704 3.79696 3.62749 5.93137 4.93730 527 - -

2.68618 4.42225 2.77519 2.73123 3.46354 2.40513 3.72494 3.29354 2.67741 2.69355 4.24690 2.90347 2.73739 3.18146 2.89801 2.37887 2.77519 2.98518 4.58477 3.61503

0.01902 4.36759 5.08994 0.61958 0.77255 0.48576 0.95510

282 3.05690 5.68471 1.46519 1.43466 4.97866 3.13348 3.88723 4.50741 2.84874 3.96982 4.78160 2.65805 3.99486 2.59789 3.43513 2.94326 3.32987 4.05565 6.11302 4.60444 528 - -

2.68618 4.42225 2.77519 2.73123 3.46354 2.40513 3.72494 3.29354 2.67741 2.69355 4.24690 2.90347 2.73739 3.18146 2.89801 2.37887 2.77519 2.98518 4.58477 3.61503

0.01902 4.36759 5.08994 0.61958 0.77255 0.48576 0.95510

283 2.92228 5.47249 1.50937 1.90446 4.78503 3.24535 3.80715 4.28710 2.39652 3.76822 4.55498 2.87774 3.94635 2.93911 3.22604 2.13058 3.18001 3.85189 5.91507 4.45458 529 - -

2.68618 4.42225 2.77519 2.73123 3.46354 2.40513 3.72494 3.29354 2.67741 2.69355 4.24690 2.90347 2.73739 3.18146 2.89801 2.37887 2.77519 2.98518 4.58477 3.61503

0.01902 4.36759 5.08994 0.61958 0.77255 0.48576 0.95510

284 3.02761 5.59998 1.09736 2.21096 4.89113 3.00463 3.53003 4.40323 2.82165 3.88716 4.70045 2.73635 3.98907 2.71614 3.39197 2.73300 3.29768 3.97095 6.04315 4.55712 530 - -

2.68618 4.42225 2.77519 2.73123 3.46354 2.40513 3.72494 3.29354 2.67741 2.69355 4.24690 2.90347 2.73739 3.18146 2.89801 2.37887 2.77519 2.98518 4.58477 3.61503

0.01902 4.36759 5.08994 0.61958 0.77255 0.48576 0.95510

285 3.09387 5.28581 3.20535 2.75687 4.53468 3.69639 3.51219 4.07763 2.24501 3.54838 4.41023 3.22320 4.12448 1.08274 2.32413 3.08144 3.30107 3.74259 5.58634 4.29107 531 - -

2.68618 4.42225 2.77519 2.73123 3.46354 2.40513 3.72494 3.29354 2.67741 2.69355 4.24690 2.90347 2.73739 3.18146 2.89801 2.37887 2.77519 2.98518 4.58477 3.61503

0.01902 4.36759 5.08994 0.61958 0.77255 0.48576 0.95510

286 2.65963 4.85058 2.87996 2.50720 4.08692 3.49187 3.52809 2.67498 2.49400 2.87214 3.92237 2.94077 3.88166 2.47642 2.46752 2.28030 2.73540 3.03512 5.35424 4.02208 532 - -

2.68618 4.42225 2.77519 2.73123 3.46354 2.40513 3.72494 3.29354 2.67741 2.69355 4.24690 2.90347 2.73739 3.18146 2.89801 2.37887 2.77519 2.98518 4.58477 3.61503

0.01902 4.36759 5.08994 0.61958 0.77255 0.48576 0.95510

287 2.45707 4.23847 4.52398 3.93419 2.51565 4.00430 4.31849 2.31431 3.78432 1.42658 3.17245 4.06538 4.34191 3.95103 3.89095 3.31093 3.07024 1.82562 4.85258 3.44593 533 - -

2.68618 4.42225 2.77519 2.73123 3.46354 2.40513 3.72494 3.29354 2.67741 2.69355 4.24690 2.90347 2.73739 3.18146 2.89801 2.37887 2.77519 2.98518 4.58477 3.61503

0.01902 4.36759 5.08994 0.61958 0.77255 0.48576 0.95510

288 3.54794 4.80198 5.35531 4.81104 3.35094 4.92831 5.31417 1.48321 4.67217 0.87839 3.11737 5.02528 5.07119 4.73242 4.74152 4.31286 3.77636 2.08336 5.50149 4.45027 534 - -

2.68618 4.42225 2.77519 2.73123 3.46354 2.40513 3.72494 3.29354 2.67741 2.69355 4.24690 2.90347 2.73739 3.18146 2.89801 2.37887 2.77519 2.98518 4.58477 3.61503

0.01902 4.36759 5.08994 0.61958 0.77255 0.48576 0.95510

289 2.39767 5.15131 2.60089 2.30877 4.47878 3.32800 3.33161 3.94934 2.33416 3.44577 4.19964 2.83408 3.85101 2.57172 1.92028 2.54047 2.92163 3.53309 5.59066 4.19455 535 - -

2.68618 4.42225 2.77519 2.73123 3.46354 2.40513 3.72494 3.29354 2.67741 2.69355 4.24690 2.90347 2.73739 3.18146 2.89801 2.37887 2.77519 2.98518 4.58477 3.61503

0.01902 4.36759 5.08994 0.61958 0.77255 0.48576 0.95510

290 2.70351 5.19576 2.67602 2.29922 4.53709 3.16423 3.64580 4.01561 1.95478 3.49424 4.24134 2.36817 3.85380 2.25628 2.49729 2.60623 2.93466 3.58522 5.62420 4.22147 536 - -

2.68618 4.42225 2.77519 2.73123 3.46354 2.40513 3.72494 3.29354 2.67741 2.69355 4.24690 2.90347 2.73739 3.18146 2.89801 2.37887 2.77519 2.98518 4.58477 3.61503

0.01902 4.36759 5.08994 0.61958 0.77255 0.48576 0.95510

291 2.87356 4.36994 4.72393 4.17209 2.99489 4.27352 4.71196 1.82413 4.05613 2.32386 3.45910 4.34053 4.61211 4.27381 4.21474 3.60868 2.86230 1.00026 5.28291 4.08568 537 - -

2.68618 4.42225 2.77519 2.73123 3.46354 2.40513 3.72494 3.29354 2.67741 2.69355 4.24690 2.90347 2.73739 3.18146 2.89801 2.37887 2.77519 2.98518 4.58477 3.61503

0.01902 4.36759 5.08994 0.61958 0.77255 0.48576 0.95510

292 2.81621 5.07308 2.76561 2.50543 4.54615 3.02776 3.87988 4.00569 2.69876 3.56370 4.38033 2.29622 1.50182 2.65721 3.14898 2.82209 3.11346 3.60237 5.74857 4.38123 538 - -

2.68618 4.42225 2.77519 2.73123 3.46354 2.40513 3.72494 3.29354 2.67741 2.69355 4.24690 2.90347 2.73739 3.18146 2.89801 2.37887 2.77519 2.98518 4.58477 3.61503

0.01902 4.36759 5.08994 0.61958 0.77255 0.48576 0.95510

293 4.28294 5.45469 4.92255 4.76947 3.35081 4.35334 4.64627 4.36823 4.50690 3.66404 4.97164 4.83254 4.91539 4.86500 4.53722 4.48214 4.61453 4.26905 0.29072 3.33196 539 - -

2.68618 4.42225 2.77519 2.73123 3.46354 2.40513 3.72494 3.29354 2.67741 2.69355 4.24690 2.90347 2.73739 3.18146 2.89801 2.37887 2.77519 2.98518 4.58477 3.61503

0.01902 4.36759 5.08994 0.61958 0.77255 0.48576 0.95510

294 3.58837 4.87815 5.26198 4.69773 3.23231 4.87875 5.19645 2.31559 4.54039 0.67141 2.57184 4.94322 5.01268 4.58952 4.61269 4.24717 3.80752 2.17591 5.39027 4.37141 540 - -

2.68618 4.42225 2.77519 2.73123 3.46354 2.40513 3.72494 3.29354 2.67741 2.69355 4.24690 2.90347 2.73739 3.18146 2.89801 2.37887 2.77519 2.98518 4.58477 3.61503

0.01902 4.36759 5.08994 0.61958 0.77255 0.48576 0.95510

295 3.35526 4.59270 5.29701 4.78741 3.73198 4.84983 5.41005 1.31731 4.69366 1.81194 3.50396 4.96184 5.07737 4.89027 4.83363 4.24339 3.60501 1.04556 5.76528 4.59759 541 - -

2.68618 4.42225 2.77519 2.73123 3.46354 2.40513 3.72494 3.29354 2.67741 2.69355 4.24690 2.90347 2.73739 3.18146 2.89801 2.37887 2.77519 2.98518 4.58477 3.61503

0.01902 4.36759 5.08994 0.61958 0.77255 0.48576 0.95510

296 3.35015 4.65974 5.15013 4.55838 2.13199 4.60121 4.88865 2.24827 4.40925 1.19569 1.78956 4.70427 4.79778 4.42998 4.43555 3.93864 3.56583 2.25956 5.15676 4.08914 542 - -

2.68618 4.42225 2.77519 2.73123 3.46354 2.40513 3.72494 3.29354 2.67741 2.69355 4.24690 2.90347 2.73739 3.18146 2.89801 2.37887 2.77519 2.98518 4.58477 3.61503

0.01902 4.36759 5.08994 0.61958 0.77255 0.48576 0.95510

297 2.82264 5.17376 3.14381 2.55076 4.52339 3.57271 3.67612 3.95088 1.86277 3.12969 4.22890 2.35593 3.95167 2.55558 1.81479 2.80369 3.03430 3.57203 5.56324 3.83796 543 - -

2.68618 4.42225 2.77519 2.73123 3.46354 2.40513 3.72494 3.29354 2.67741 2.69355 4.24690 2.90347 2.73739 3.18146 2.89801 2.37887 2.77519 2.98518 4.58477 3.61503

0.01902 4.36759 5.08994 0.61958 0.77255 0.48576 0.95510

298 2.08513 4.36336 3.75332 3.38822 4.48470 3.16310 4.41754 3.86374 3.39102 3.57123 4.39433 3.52989 3.90121 3.68189 3.71110 1.30462 1.48669 3.33337 5.82925 4.62185 544 - -

2.68618 4.42225 2.77519 2.73123 3.46354 2.40513 3.72494 3.29354 2.67741 2.69355 4.24690 2.90347 2.73739 3.18146 2.89801 2.37887 2.77519 2.98518 4.58477 3.61503

0.01902 4.36759 5.08994 0.61958 0.77255 0.48576 0.95510

299 3.19827 5.77837 1.05539 2.29830 5.09547 3.40784 4.02208 4.68674 3.10342 4.16924 5.04066 1.65430 4.06530 3.20048 3.73347 3.07159 3.51103 4.22745 6.28588 4.74607 545 - -

2.68618 4.42225 2.77519 2.73123 3.46354 2.40513 3.72494 3.29354 2.67741 2.69355 4.24690 2.90347 2.73739 3.18146 2.89801 2.37887 2.77519 2.98518 4.58477 3.61503

0.01902 4.36759 5.08994 0.61958 0.77255 0.48576 0.95510

300 2.60135 4.41617 3.47345 2.91316 3.58116 3.42824 3.87669 2.93467 2.86954 1.98283 3.14913 2.79870 3.99427 3.18265 3.23717 2.08670 2.79848 2.49795 5.01325 3.77134 546 - -

2.68618 4.42225 2.77519 2.73123 3.46354 2.40513 3.72494 3.29354 2.67741 2.69355 4.24690 2.90347 2.73739 3.18146 2.89801 2.37887 2.77519 2.98518 4.58477 3.61503

0.01902 4.36759 5.08994 0.61958 0.77255 0.48576 0.95510

301 4.12793 5.27565 4.97509 4.75972 1.89950 4.74142 3.67838 3.79013 4.58429 3.04893 4.37364 4.38404 5.05242 4.48757 4.56221 4.15219 4.35316 3.72999 3.79055 0.56017 547 - -

2.68618 4.42225 2.77519 2.73123 3.46354 2.40513 3.72494 3.29354 2.67741 2.69355 4.24690 2.90347 2.73739 3.18146 2.89801 2.37887 2.77519 2.98518 4.58477 3.61503

0.01902 4.36759 5.08994 0.61958 0.77255 0.48576 0.95510

302 3.44008 4.73736 5.09374 4.57320 1.18273 4.64655 4.62264 1.79847 4.42190 1.68689 3.18903 4.65697 4.87136 4.47409 4.47893 4.00047 3.66605 2.42638 4.84492 3.41211 548 - -

2.68618 4.42225 2.77519 2.73123 3.46354 2.40513 3.72494 3.29354 2.67741 2.69355 4.24690 2.90347 2.73739 3.18146 2.89801 2.37887 2.77519 2.98518 4.58477 3.61503

0.01902 4.36759 5.08994 0.61958 0.77255 0.48576 0.95510

303 2.41688 4.30669 4.50364 3.94445 3.52604 4.10044 4.51810 1.59462 3.82997 2.28987 3.45034 4.13735 3.99878 4.06388 4.01194 3.42173 2.97774 1.29299 5.16307 3.96513 549 - -

2.68618 4.42225 2.77519 2.73123 3.46354 2.40513 3.72494 3.29354 2.67741 2.69355 4.24690 2.90347 2.73739 3.18146 2.89801 2.37887 2.77519 2.98518 4.58477 3.61503

0.01902 4.36759 5.08994 0.61958 0.77255 0.48576 0.95510

304 2.43550 4.47601 3.67211 3.45213 4.41220 3.26672 4.51067 3.68349 3.45404 3.44279 4.42244 3.61231 0.82916 3.81043 3.72246 2.75437 3.05306 3.02776 5.84131 4.59785 550 - -

2.68618 4.42225 2.77519 2.73123 3.46354 2.40513 3.72494 3.29354 2.67741 2.69355 4.24690 2.90347 2.73739 3.18146 2.89801 2.37887 2.77519 2.98518 4.58477 3.61503

0.01902 4.36759 5.08994 0.61958 0.77255 0.48576 0.95510

305 2.18236 4.44257 3.55571 3.14236 4.38959 1.84864 4.23386 3.80054 3.15325 3.46585 3.79056 3.40789 3.89899 3.46355 3.52142 1.27169 2.90340 3.31629 5.71515 4.47096 551 - -

2.68618 4.42225 2.77519 2.73123 3.46354 2.40513 3.72494 3.29354 2.67741 2.69355 4.24690 2.90347 2.73739 3.18146 2.89801 2.37887 2.77519 2.98518 4.58477 3.61503

0.01902 4.36759 5.08994 0.61958 0.77255 0.48576 0.95510

306 3.67258 4.98103 5.19709 4.71374 3.23062 4.86292 5.19356 2.26700 4.46990 0.55738 3.06166 4.95394 5.05199 4.61654 4.55537 4.29903 3.91328 2.53071 5.38475 4.25303 552 - -

2.68618 4.42225 2.77519 2.73123 3.46354 2.40513 3.72494 3.29354 2.67741 2.69355 4.24690 2.90347 2.73739 3.18146 2.89801 2.37887 2.77519 2.98518 4.58477 3.61503

0.01902 4.36759 5.08994 0.61958 0.77255 0.48576 0.95510

307 4.16498 5.23432 5.18863 4.99576 0.89172 4.81876 3.63777 3.81251 4.78703 3.07384 4.36420 4.43329 5.08999 4.56015 4.66739 4.19979 4.36848 3.73679 1.75296 1.76957 553 - -

2.68618 4.42225 2.77519 2.73123 3.46354 2.40513 3.72494 3.29354 2.67741 2.69355 4.24690 2.90347 2.73739 3.18146 2.89801 2.37887 2.77519 2.98518 4.58477 3.61503

0.01902 4.36759 5.08994 0.61958 0.77255 0.48576 0.95510

308 2.69818 4.35695 4.24573 3.68350 3.05577 3.94976 4.28607 2.51089 3.55738 1.06704 3.06751 3.92608 4.31646 3.79415 3.75996 2.86720 3.10952 2.41827 4.94872 3.75011 554 - -

2.68618 4.42225 2.77519 2.73123 3.46354 2.40513 3.72494 3.29354 2.67741 2.69355 4.24690 2.90347 2.73739 3.18146 2.89801 2.37887 2.77519 2.98518 4.58477 3.61503

0.01902 4.36759 5.08994 0.61958 0.77255 0.48576 0.95510

309 2.85514 4.30700 4.28566 3.72503 3.49864 4.01084 4.37697 1.51667 3.61705 2.40478 3.25968 2.93317 4.37447 3.87642 3.83835 3.31532 2.95412 1.46000 5.10000 3.89541 555 - -

2.68618 4.42225 2.77519 2.73123 3.46354 2.40513 3.72494 3.29354 2.67741 2.69355 4.24690 2.90347 2.73739 3.18146 2.89801 2.37887 2.77519 2.98518 4.58477 3.61503
0.01902 4.36759 5.08994 0.61958 0.77255 0.48576 0.95510
310 2.43058 4.59064 3.05546 2.88369 4.37574 3.28486 4.09601 3.78403 2.99717 3.43355 4.25829 3.26579 1.34784 3.30164 3.40833 2.42416 2.24714 3.34447 5.68223 4.40031 556 - -
2.68618 4.42225 2.77519 2.73123 3.46354 2.40513 3.72494 3.29354 2.67741 2.69355 4.24690 2.90347 2.73739 3.18146 2.89801 2.37887 2.77519 2.98518 4.58477 3.61503
0.01902 4.36759 5.08994 0.61958 0.77255 0.48576 0.95510
311 2.33090 4.92675 3.01629 2.32654 4.20888 2.81013 3.68718 3.63847 2.46651 3.21701 3.76841 2.99565 3.86942 2.73481 2.76572 1.93015 2.62861 3.28909 5.43528 4.08500 557 - -
2.68618 4.42225 2.77519 2.73123 3.46354 2.40513 3.72494 3.29354 2.67741 2.69355 4.24690 2.90347 2.73739 3.18146 2.89801 2.37887 2.77519 2.98518 4.58477 3.61503
0.01902 4.36759 5.08994 0.61958 0.77255 0.48576 0.95510
312 3.97153 5.13008 5.06990 4.79407 0.84860 4.72803 3.70471 3.54322 4.60625 2.47695 4.12455 4.39898 5.00727 4.47652 4.55704 4.09724 4.18319 3.49774 3.81778 1.44741 558 - -
2.68618 4.42225 2.77519 2.73123 3.46354 2.40513 3.72494 3.29354 2.67741 2.69355 4.24690 2.90347 2.73739 3.18146 2.89801 2.37887 2.77519 2.98518 4.58477 3.61503
0.01902 4.36759 5.08994 0.61958 0.77255 0.48576 0.95510
313 2.80808 5.26376 2.77570 2.08214 4.60786 3.49011 3.69580 4.06972 2.23258 3.54775 4.31882 2.82790 3.91688 1.58771 2.54459 2.76524 3.03671 3.65701 5.67280 4.29344 559 - -
2.68618 4.42225 2.77519 2.73123 3.46354 2.40513 3.72494 3.29354 2.67741 2.69355 4.24690 2.90347 2.73739 3.18146 2.89801 2.37887 2.77519 2.98518 4.58477 3.61503
0.01902 4.36759 5.08994 0.61958 0.77255 0.48576 0.95510
314 2.37140 5.14492 2.27894 2.22887 4.46929 3.03435 3.64667 3.94220 2.34583 3.44222 4.19518 2.91466 3.84480 2.65353 2.66527 2.20741 2.67358 3.52524 5.59155 4.19011 560 - -
2.68618 4.42225 2.77519 2.73123 3.46354 2.40513 3.72494 3.29354 2.67741 2.69355 4.24690 2.90347 2.73739 3.18146 2.89801 2.37887 2.77519 2.98518 4.58477 3.61503
0.01902 4.36759 5.08994 0.61958 0.77255 0.48576 0.95510
315 2.90358 5.48496 2.50728 0.84623 4.85968 3.45944 3.96509 4.28741 2.73840 3.85132 4.72564 2.97603 4.05959 3.13335 3.29951 3.02556 3.38470 3.90075 6.02644 4.60272 561 - -
2.68618 4.42225 2.77519 2.73123 3.46354 2.40513 3.72494 3.29354 2.67741 2.69355 4.24690 2.90347 2.73739 3.18146 2.89801 2.37887 2.77519 2.98518 4.58477 3.61503
0.01902 4.36759 5.08994 0.61958 0.77255 0.48576 0.95510
316 3.76585 5.09668 4.98648 4.65545 3.40807 4.60815 5.16228 2.66140 4.42265 0.44071 3.32239 4.88679 4.97522 4.66557 4.50633 4.28261 4.04609 2.82202 5.43615 4.26530 562 - -
2.68618 4.42225 2.77519 2.73123 3.46354 2.40513 3.72494 3.29354 2.67741 2.69355 4.24690 2.90347 2.73739 3.18146 2.89801 2.37887 2.77519 2.98518 4.58477 3.61503
0.01902 4.36759 5.08994 0.61958 0.77255 0.48576 0.95510
317 2.60907 5.09641 2.35830 2.23781 4.41095 3.32267 3.63884 3.76159 2.38772 3.38884 3.99456 2.85616 3.84008 2.65224 2.60271 2.03974 2.70322 3.29340 5.54864 4.15705 563 - -
2.68618 4.42225 2.77519 2.73123 3.46354 2.40513 3.72494 3.29354 2.67741 2.69355 4.24690 2.90347 2.73739 3.18146 2.89801 2.37887 2.77519 2.98518 4.58477 3.61503
0.01902 4.36759 5.08994 0.61958 0.77255 0.48576 0.95510
318 2.57849 4.90925 3.11601 2.54295 4.16412 3.52545 3.69732 3.57614 1.85554 3.16500 3.33424 3.05294 3.91057 2.76621 2.17173 2.39892 2.93439 3.25189 4.02385 4.06911 564 - -
2.68618 4.42225 2.77519 2.73123 3.46354 2.40513 3.72494 3.29354 2.67741 2.69355 4.24690 2.90347 2.73739 3.18146 2.89801 2.37887 2.77519 2.98518 4.58477 3.61503
0.01902 4.36759 5.08994 0.61958 0.77255 0.48576 0.95510
319 3.67774 4.96241 5.30317 4.74661 3.18282 4.93898 5.21448 2.39713 4.55120 0.71727 1.87577 5.00186 5.04783 4.59872 4.60873 4.32246 3.89113 2.62251 5.36532 4.34432 565 - -
2.68618 4.42225 2.77519 2.73123 3.46354 2.40513 3.72494 3.29354 2.67741 2.69355 4.24690 2.90347 2.73739 3.18146 2.89801 2.37887 2.77519 2.98518 4.58477 3.61503
0.01902 4.36759 5.08994 0.61958 0.77255 0.48576 0.95510
320 3.81315 5.09538 4.96334 4.68488 0.63943 4.60720 4.05942 3.08580 4.51383 2.22294 3.75369 4.51998 4.96174 4.53069 4.53490 4.08224 4.08163 3.14160 4.19856 2.51332 566 - -
2.68618 4.42225 2.77519 2.73123 3.46354 2.40513 3.72494 3.29354 2.67741 2.69355 4.24690 2.90347 2.73739 3.18146 2.89801 2.37887 2.77519 2.98518 4.58477 3.61503
0.01902 4.36759 5.08994 0.61958 0.77255 0.48576 0.95510

321 3.54539 5.18809 4.20814 4.13635 5.04512 3.84929 5.14538 4.75467 4.24336 4.30986 5.39830 4.38159 0.27068 4.61398 4.40637 3.73410 4.02880 4.33588 6.06192 5.18068 567 - -
2.68618 4.42225 2.77519 2.73123 3.46354 2.40513 3.72494 3.29354 2.67741 2.69355 4.24690 2.90347 2.73739 3.18146 2.89801 2.37887 2.77519 2.98518 4.58477 3.61503
0.01902 4.36759 5.08994 0.61958 0.77255 0.48576 0.95510
322 2.51547 5.22008 2.85400 1.69137 4.55402 3.47701 3.69552 4.01491 2.40319 3.50953 4.28225 2.84525 3.90488 1.95159 2.58716 2.74590 3.01444 3.60876 5.64981 4.26728 568 - -
2.68618 4.42225 2.77519 2.73123 3.46354 2.40513 3.72494 3.29354 2.67741 2.69355 4.24690 2.90347 2.73739 3.18146 2.89801 2.37887 2.77519 2.98518 4.58477 3.61503
0.01902 4.36759 5.08994 0.61958 0.77255 0.48576 0.95510
323 3.07574 5.27164 3.48876 2.83458 4.72330 3.74970 3.74905 4.07910 1.11380 3.53863 4.37732 3.27134 4.12229 2.87565 1.95219 3.06889 2.55288 3.73557 5.61042 4.39976 569 - -
2.68618 4.42225 2.77519 2.73123 3.46354 2.40513 3.72494 3.29354 2.67741 2.69355 4.24690 2.90347 2.73739 3.18146 2.89801 2.37887 2.77519 2.98518 4.58477 3.61503
0.01902 4.36759 5.08994 0.61958 0.77255 0.48576 0.95510
324 2.96454 5.76057 1.27354 1.40370 5.06066 2.99724 3.94322 4.59501 2.96559 4.06475 4.90126 2.85568 4.02406 3.10248 3.58085 3.00181 3.41557 4.14178 6.21653 4.68622 570 - -
2.68618 4.42225 2.77519 2.73123 3.46354 2.40513 3.72494 3.29354 2.67741 2.69355 4.24690 2.90347 2.73739 3.18146 2.89801 2.37887 2.77519 2.98518 4.58477 3.61503
0.01902 4.36759 5.08994 0.61958 0.77255 0.48576 0.95510
325 1.34272 4.34276 3.80878 3.42471 4.42565 3.16675 4.42169 3.79845 3.41423 3.51321 4.34180 3.54888 3.90211 3.69829 3.72752 1.93783 1.53955 3.28826 5.78442 4.58276 571 - -
2.68618 4.42225 2.77519 2.73123 3.46354 2.40513 3.72494 3.29354 2.67741 2.69355 4.24690 2.90347 2.73739 3.18146 2.89801 2.37887 2.77519 2.98518 4.58477 3.61503
0.01902 4.36759 5.08994 0.61958 0.77255 0.48576 0.95510
326 2.42328 4.46712 4.58560 4.22537 3.97460 3.93323 5.00017 2.14514 4.12891 2.63580 3.82900 4.29701 4.52824 4.42718 4.32476 3.38430 3.30577 0.75606 5.76628 4.54593 572 - -
2.68618 4.42225 2.77519 2.73123 3.46354 2.40513 3.72494 3.29354 2.67741 2.69355 4.24690 2.90347 2.73739 3.18146 2.89801 2.37887 2.77519 2.98518 4.58477 3.61503
0.01902 4.36759 5.08994 0.61958 0.77255 0.48576 0.95510
327 2.92220 3.76844 4.51300 4.05092 0.97909 3.87423 4.26281 2.73017 3.89663 2.39610 3.56030 4.07649 4.38459 4.08800 4.03192 3.26593 2.94654 2.59665 4.69335 3.19685 573 - -
2.68618 4.42225 2.77519 2.73123 3.46354 2.40513 3.72494 3.29354 2.67741 2.69355 4.24690 2.90347 2.73739 3.18146 2.89801 2.37887 2.77519 2.98518 4.58477 3.61503
0.01902 4.36759 5.08994 0.61958 0.77255 0.48576 0.95510
328 3.44050 5.15969 3.71966 3.38895 2.66407 4.05752 0.89456 3.84076 3.17201 3.25142 4.35355 3.69764 4.49420 3.61908 3.42922 3.48004 3.68580 3.64001 4.28694 2.27844 574 - -
2.68618 4.42225 2.77519 2.73123 3.46354 2.40513 3.72494 3.29354 2.67741 2.69355 4.24690 2.90347 2.73739 3.18146 2.89801 2.37887 2.77519 2.98518 4.58477 3.61503
0.01902 4.36759 5.08994 0.61958 0.77255 0.48576 0.95510
329 3.16100 4.83793 3.85516 3.37400 2.57885 4.00042 1.18980 3.25206 3.19480 2.22850 3.84413 3.68670 4.38440 3.55855 3.47995 3.31107 3.39844 3.11116 4.38862 2.75875 575 - -
2.68618 4.42225 2.77519 2.73123 3.46354 2.40513 3.72494 3.29354 2.67741 2.69355 4.24690 2.90347 2.73739 3.18146 2.89801 2.37887 2.77519 2.98518 4.58477 3.61503
0.01902 4.36759 5.08994 0.61958 0.77255 0.48576 0.95510
330 3.67363 4.97909 5.20729 4.72002 3.23088 4.87216 5.20033 2.25249 4.47947 0.55990 3.05836 4.96127 5.05567 4.62068 4.56392 4.30544 3.91288 2.52394 5.38793 4.26110 576 - -
2.68618 4.42225 2.77519 2.73123 3.46354 2.40513 3.72494 3.29354 2.67741 2.69355 4.24690 2.90347 2.73739 3.18146 2.89801 2.37887 2.77519 2.98518 4.58477 3.61503
0.01902 4.36759 5.08994 0.61958 0.77255 0.48576 0.95510
331 1.05908 4.33798 3.78621 3.50738 4.65019 1.75512 4.55105 4.08689 3.58063 3.76407 4.56386 3.57580 3.89220 3.83062 3.87838 2.05311 2.90431 3.45541 5.98300 4.79738 577 - -
2.68618 4.42225 2.77519 2.73123 3.46354 2.40513 3.72494 3.29354 2.67741 2.69355 4.24690 2.90347 2.73739 3.18146 2.89801 2.37887 2.77519 2.98518 4.58477 3.61503
0.01902 4.36759 5.08994 0.61958 0.77255 0.48576 0.95510
332 3.32890 5.38581 3.73664 3.06483 4.73955 3.90187 3.47218 4.20445 2.03468 3.61597 4.50754 3.43751 4.26899 2.73310 0.84054 3.32410 3.48136 3.89925 5.59437 4.41094 578 - -
2.68618 4.42225 2.77519 2.73123 3.46354 2.40513 3.72494 3.29354 2.67741 2.69355 4.24690 2.90347 2.73739 3.18146 2.89801 2.37887 2.77519 2.98518 4.58477 3.61503

0.01902 4.36759 5.08994 0.61958 0.77255 0.48576 0.95510
333 4.03870 5.32308 4.60931 4.45436 2.49387 4.43826 3.96720 3.99215 4.30240 3.31100 4.61656 4.38690 4.91067 4.47972 4.37220 4.11426 4.33336 3.88849 4.14795 0.42355 579 - -
2.68618 4.42225 2.77519 2.73123 3.46354 2.40513 3.72494 3.29354 2.67741 2.69355 4.24690 2.90347 2.73739 3.18146 2.89801 2.37887 2.77519 2.98518 4.58477 3.61503
0.01902 4.36759 5.08994 0.61958 0.77255 0.48576 0.95510
334 3.76585 5.09668 4.98648 4.65545 3.40807 4.60815 5.16228 2.66140 4.42265 0.44071 3.32239 4.88679 4.97522 4.66557 4.50633 4.28261 4.04609 2.82202 5.43615 4.26530 580 - -
2.68618 4.42225 2.77519 2.73123 3.46354 2.40513 3.72494 3.29354 2.67741 2.69355 4.24690 2.90347 2.73739 3.18146 2.89801 2.37887 2.77519 2.98518 4.58477 3.61503
0.01902 4.36759 5.08994 0.61958 0.77255 0.48576 0.95510
335 3.69035 4.96665 5.09419 4.67546 0.95405 4.72112 4.32790 2.71613 4.50496 1.46036 3.33044 4.64366 4.96288 4.51190 4.53618 4.11851 3.92015 2.84191 4.47590 2.88036 581 - -
2.68618 4.42225 2.77519 2.73123 3.46354 2.40513 3.72494 3.29354 2.67741 2.69355 4.24690 2.90347 2.73739 3.18146 2.89801 2.37887 2.77519 2.98518 4.58477 3.61503
0.01902 4.36759 5.08994 0.61958 0.77255 0.48576 0.95510
336 3.70582 5.37978 3.77262 3.60812 3.77250 3.98143 0.42872 4.49303 3.44540 3.90784 5.01226 3.98613 4.57250 3.98907 3.66642 3.78104 4.03786 4.21845 5.22021 3.71509 582 - -
2.68618 4.42225 2.77519 2.73123 3.46354 2.40513 3.72494 3.29354 2.67741 2.69355 4.24690 2.90347 2.73739 3.18146 2.89801 2.37887 2.77519 2.98518 4.58477 3.61503
0.01902 4.36759 5.08994 0.61958 0.77255 0.48576 0.95510
337 3.54539 5.18809 4.20814 4.13635 5.04512 3.84929 5.14538 4.75467 4.24336 4.30986 5.39830 4.38159 0.27068 4.61398 4.40637 3.73410 4.02880 4.33588 6.06192 5.18068 583 - -
2.68618 4.42225 2.77519 2.73123 3.46354 2.40513 3.72494 3.29354 2.67741 2.69355 4.24690 2.90347 2.73739 3.18146 2.89801 2.37887 2.77519 2.98518 4.58477 3.61503
0.01902 4.36759 5.08994 0.61958 0.77255 0.48576 0.95510
338 2.33004 4.67896 3.17492 2.72880 4.27072 3.18065 3.92513 3.69057 2.69548 3.31090 4.12715 2.61459 3.90022 3.10281 3.20149 2.10059 1.56398 3.29495 5.55284 4.24866 584 - -
2.68618 4.42225 2.77519 2.73123 3.46354 2.40513 3.72494 3.29354 2.67741 2.69355 4.24690 2.90347 2.73739 3.18146 2.89801 2.37887 2.77519 2.98518 4.58477 3.61503
0.01902 4.36759 5.08994 0.61958 0.77255 0.48576 0.95510
339 3.23805 5.19967 3.12672 3.07643 4.65411 3.60375 4.45882 4.50763 3.42586 4.10045 5.07485 0.51439 4.29067 3.75696 3.77714 3.30087 3.65033 4.07495 5.89823 4.58577 585 - -
2.68618 4.42225 2.77519 2.73123 3.46354 2.40513 3.72494 3.29354 2.67741 2.69355 4.24690 2.90347 2.73739 3.18146 2.89801 2.37887 2.77519 2.98518 4.58477 3.61503
0.01902 4.36759 5.08994 0.61958 0.77255 0.48576 0.95510
340 2.46645 5.09998 2.77755 2.32137 4.41510 3.44737 2.84165 3.87937 2.23888 3.39156 4.14957 2.92763 3.84213 2.13546 2.79699 2.40926 2.74271 3.17406 5.55016 4.15953 586 - -
2.68618 4.42225 2.77519 2.73123 3.46354 2.40513 3.72494 3.29354 2.67741 2.69355 4.24690 2.90347 2.73739 3.18146 2.89801 2.37887 2.77519 2.98518 4.58477 3.61503
0.01902 4.36759 5.08994 0.61958 0.77255 0.48576 0.95510
341 3.08388 4.55280 5.14488 4.66559 3.88341 4.69456 5.38087 1.49407 4.57123 2.39225 3.66198 4.83814 5.01044 4.83943 4.75658 4.10077 3.54547 0.79126 5.86411 4.64841 587 - -
2.68618 4.42225 2.77519 2.73123 3.46354 2.40513 3.72494 3.29354 2.67741 2.69355 4.24690 2.90347 2.73739 3.18146 2.89801 2.37887 2.77519 2.98518 4.58477 3.61503
0.01902 4.36759 5.08994 0.61958 0.77255 0.48576 0.95510
342 4.28294 5.45469 4.92255 4.76947 3.35081 4.35334 4.64627 4.36823 4.50690 3.66404 4.97164 4.83254 4.91539 4.86500 4.53722 4.48214 4.61453 4.26905 0.29072 3.33196 588 - -
2.68618 4.42225 2.77519 2.73123 3.46354 2.40513 3.72494 3.29354 2.67741 2.69355 4.24690 2.90347 2.73739 3.18146 2.89801 2.37887 2.77519 2.98518 4.58477 3.61503
0.01902 4.36759 5.08994 0.61958 0.77255 0.48576 0.95510
343 2.81342 5.06339 2.57803 2.36680 4.26559 1.48636 3.31473 3.75117 2.64477 3.33858 4.16609 2.98889 3.94589 2.95540 3.12247 2.81441 3.06157 3.41537 5.53814 3.42518 589 - -
2.68618 4.42225 2.77519 2.73123 3.46354 2.40513 3.72494 3.29354 2.67741 2.69355 4.24690 2.90347 2.73739 3.18146 2.89801 2.37887 2.77519 2.98518 4.58477 3.61503
0.01902 4.36759 5.08994 0.61958 0.77255 0.48576 0.95510
344 2.60030 4.25628 4.30409 3.72317 3.27110 3.92925 4.25467 2.20815 3.59018 1.60128 1.90409 3.92883 4.28332 3.80650 3.76109 2.87399 3.03940 2.31372 4.89126 3.71731 590 - -

2.68618 4.42225 2.77519 2.73123 3.46354 2.40513 3.72494 3.29354 2.67741 2.69355 4.24690 2.90347 2.73739 3.18146 2.89801 2.37887 2.77519 2.98518 4.58477 3.61503
0.01902 4.36759 5.08994 0.61958 0.77255 0.48576 0.95510
345 3.35315 4.59328 5.29838 4.82624 3.88320 4.85591 5.54551 1.19014 4.73998 2.33881 3.64584 4.99988 5.12313 4.99623 4.91385 4.27653 3.61880 0.92849 5.93933 4.72537 591 - -
2.68618 4.42225 2.77519 2.73123 3.46354 2.40513 3.72494 3.29354 2.67741 2.69355 4.24690 2.90347 2.73739 3.18146 2.89801 2.37887 2.77519 2.98518 4.58477 3.61503
0.01902 4.36759 5.08994 0.61958 0.77255 0.48576 0.95510
346 2.53952 4.68538 3.20411 2.59767 3.89990 3.54016 3.58201 3.28453 2.43214 2.94008 3.33339 3.14669 3.94092 2.97703 3.01025 2.69368 1.60148 3.00657 5.23591 3.95008 592 - -
2.68618 4.42225 2.77519 2.73123 3.46354 2.40513 3.72494 3.29354 2.67741 2.69355 4.24690 2.90347 2.73739 3.18146 2.89801 2.37887 2.77519 2.98518 4.58477 3.61503
0.01902 4.36759 5.08994 0.61958 0.77255 0.48576 0.95510
347 2.64294 5.09568 3.03125 2.64896 4.50493 3.07307 3.77142 3.90865 2.24227 3.43920 4.26014 3.13374 4.00749 2.91013 1.24351 2.88584 3.11264 3.54956 5.58882 4.30082 593 - -
2.68618 4.42225 2.77519 2.73123 3.46354 2.40513 3.72494 3.29354 2.67741 2.69355 4.24690 2.90347 2.73739 3.18146 2.89801 2.37887 2.77519 2.98518 4.58477 3.61503
0.01902 4.36759 5.08994 0.61958 0.77255 0.48576 0.95510
348 3.22247 4.73892 4.19779 3.74365 2.11085 4.10197 3.77332 3.28367 3.66029 2.84274 3.89778 3.88404 4.48937 3.84695 3.88087 2.10547 3.47129 3.10261 4.10973 1.13228 594 - -
2.68618 4.42225 2.77519 2.73123 3.46354 2.40513 3.72494 3.29354 2.67741 2.69355 4.24690 2.90347 2.73739 3.18146 2.89801 2.37887 2.77519 2.98518 4.58477 3.61503
0.01902 4.36759 5.08994 0.61958 0.77255 0.48576 0.95510
349 3.83867 5.23654 4.33714 4.03717 2.23521 4.45292 2.25470 3.89937 3.83496 3.21450 4.42904 4.06595 4.81713 4.07209 3.99842 3.85340 4.06571 3.75923 3.89710 0.72903 595 - -
2.68618 4.42225 2.77519 2.73123 3.46354 2.40513 3.72494 3.29354 2.67741 2.69355 4.24690 2.90347 2.73739 3.18146 2.89801 2.37887 2.77519 2.98518 4.58477 3.61503
0.01902 4.36759 5.08994 0.61958 0.77255 0.48576 0.95510
350 2.63215 5.19943 2.28367 2.21593 4.53158 3.29174 3.09466 4.01268 2.40884 3.49812 4.24714 2.16411 3.85243 2.69058 2.43322 2.66546 2.83719 3.58384 5.63605 4.22543 596 - -
2.68618 4.42225 2.77519 2.73123 3.46354 2.40513 3.72494 3.29354 2.67741 2.69355 4.24690 2.90347 2.73739 3.18146 2.89801 2.37887 2.77519 2.98518 4.58477 3.61503
0.01902 4.36759 5.08994 0.61958 0.77255 0.48576 0.95510
351 1.60951 4.71273 2.67059 2.72525 4.48960 2.12374 4.00541 3.93507 2.88155 3.53273 4.33132 3.16185 3.89831 3.17631 3.33601 1.88907 2.96389 3.46463 5.74254 4.41971 597 - -
2.68618 4.42225 2.77519 2.73123 3.46354 2.40513 3.72494 3.29354 2.67741 2.69355 4.24690 2.90347 2.73739 3.18146 2.89801 2.37887 2.77519 2.98518 4.58477 3.61503
0.01902 4.36759 5.08994 0.61958 0.77255 0.48576 0.95510
352 3.86505 5.24362 4.36618 4.07943 2.22628 4.47035 2.47579 3.90784 3.87452 3.21718 4.43934 4.08774 4.83508 4.10233 4.02732 3.87832 4.09302 3.77129 3.88915 0.67725 598 - -
2.68618 4.42225 2.77519 2.73123 3.46354 2.40513 3.72494 3.29354 2.67741 2.69355 4.24690 2.90347 2.73739 3.18146 2.89801 2.37887 2.77519 2.98518 4.58477 3.61503
0.01902 4.36759 5.08994 0.61958 0.77255 0.48576 0.95510
353 3.70514 4.99768 5.27356 4.74182 3.19823 4.93959 5.22604 2.39383 4.50997 0.56129 2.64198 5.00294 5.06794 4.60759 4.58296 4.34509 3.92728 2.60662 5.38527 4.32654 599 - -
2.68618 4.42225 2.77519 2.73123 3.46354 2.40513 3.72494 3.29354 2.67741 2.69355 4.24690 2.90347 2.73739 3.18146 2.89801 2.37887 2.77519 2.98518 4.58477 3.61503
0.01902 4.36759 5.08994 0.61958 0.77255 0.48576 0.95510
354 1.30529 4.58793 3.32654 2.90266 4.26915 3.33435 4.03675 3.67125 2.72960 3.31940 4.15310 3.28272 3.92871 3.24277 3.06132 1.78798 2.94875 3.27094 5.57305 4.30332 600 - -
2.68618 4.42225 2.77519 2.73123 3.46354 2.40513 3.72494 3.29354 2.67741 2.69355 4.24690 2.90347 2.73739 3.18146 2.89801 2.37887 2.77519 2.98518 4.58477 3.61503
0.01902 4.36759 5.08994 0.61958 0.77255 0.48576 0.95510
355 2.66527 4.50545 2.88397 2.40858 4.35228 3.09190 3.50747 3.80491 2.04105 3.33664 4.10615 2.81055 3.57137 2.67485 2.17828 2.65246 2.48370 3.41872 4.83535 4.13414 601 - -
2.68618 4.42225 2.77519 2.73123 3.46354 2.40513 3.72494 3.29354 2.67741 2.69355 4.24690 2.90347 2.73739 3.18146 2.89801 2.37887 2.77519 2.98518 4.58477 3.61503
0.01902 4.36759 5.08994 0.61958 0.77255 0.48576 0.95510

356 0.88937 4.35151 3.88673 3.46658 3.51887 3.33859 4.35865 3.17378 3.41711 3.03388 3.98459 3.62810 4.00715 3.70841 3.70594 2.61744 2.95160 2.70426 5.45846 4.20685 602 - -

2.68618 4.42225 2.77519 2.73123 3.46354 2.40513 3.72494 3.29354 2.67741 2.69355 4.24690 2.90347 2.73739 3.18146 2.89801 2.37887 2.77519 2.98518 4.58477 3.61503

0.01902 4.36759 5.08994 0.61958 0.77255 0.48576 0.95510

357 2.64845 5.28274 1.68088 2.21654 4.60641 3.10637 3.71015 4.09136 2.50299 3.57947 4.34218 2.57197 3.88734 2.74413 2.46092 2.62334 3.02162 3.66444 5.72332 4.29989 603 - -

2.68618 4.42225 2.77519 2.73123 3.46354 2.40513 3.72494 3.29354 2.67741 2.69355 4.24690 2.90347 2.73739 3.18146 2.89801 2.37887 2.77519 2.98518 4.58477 3.61503

0.01902 4.36759 5.08994 0.61958 0.77255 0.48576 0.95510

358 2.82776 5.26401 2.92239 1.48003 4.61667 3.51638 3.28080 4.07005 2.18215 3.54294 4.31858 2.98796 3.93335 2.24773 2.55491 2.78750 3.05110 3.66305 5.65924 4.29737 604 - -

2.68618 4.42225 2.77519 2.73123 3.46354 2.40513 3.72494 3.29354 2.67741 2.69355 4.24690 2.90347 2.73739 3.18146 2.89801 2.37887 2.77519 2.98518 4.58477 3.61503

0.01902 4.36759 5.08994 0.61958 0.77255 0.48576 0.95510

359 2.76635 4.83131 3.27467 2.68114 4.06746 3.59620 3.74398 3.35347 2.30044 2.67028 3.56373 3.01888 3.97533 2.91675 1.57069 2.83237 2.68239 3.15793 5.32034 4.04273 605 - -

2.68618 4.42225 2.77519 2.73123 3.46354 2.40513 3.72494 3.29354 2.67741 2.69355 4.24690 2.90347 2.73739 3.18146 2.89801 2.37887 2.77519 2.98518 4.58477 3.61503

0.01902 4.36759 5.08994 0.61958 0.77255 0.48576 0.95510

360 3.37144 4.62487 5.26739 4.73594 3.56058 4.80407 5.28273 1.54776 4.62537 1.30757 3.33990 4.91094 5.01697 4.76673 4.73323 4.18320 3.61283 1.26155 5.59994 4.47847 606 - -

2.68618 4.42225 2.77519 2.73123 3.46354 2.40513 3.72494 3.29354 2.67741 2.69355 4.24690 2.90347 2.73739 3.18146 2.89801 2.37887 2.77519 2.98518 4.58477 3.61503

0.01902 4.36759 5.08994 0.61958 0.77255 0.48576 0.95510

361 3.39714 5.09428 4.16865 4.14229 5.22056 0.25704 5.20174 4.97041 4.38850 4.54829 5.54795 4.33563 4.49699 4.67429 4.54243 3.58728 3.91504 4.40245 6.15571 5.34880 607 - -

2.68618 4.42225 2.77519 2.73123 3.46354 2.40513 3.72494 3.29354 2.67741 2.69355 4.24690 2.90347 2.73739 3.18146 2.89801 2.37887 2.77519 2.98518 4.58477 3.61503

0.01902 4.36759 5.08994 0.61958 0.77255 0.48576 0.95510

362 3.33620 4.58342 5.25519 4.73637 3.36710 4.80055 5.31339 0.99595 4.64142 2.01491 3.46275 4.90543 5.03196 4.81986 4.77230 4.18396 3.58304 1.34399 5.67107 4.49663 608 - -

2.68618 4.42225 2.77519 2.73123 3.46354 2.40513 3.72494 3.29354 2.67741 2.69355 4.24690 2.90347 2.73739 3.18146 2.89801 2.37887 2.77519 2.98518 4.58477 3.61503

0.01902 4.36759 5.08994 0.61958 0.77255 0.48576 0.95510

363 3.09161 5.16295 3.17324 2.82818 3.85947 3.69322 3.89443 3.84786 2.58460 3.32370 4.30698 3.29522 4.16986 0.97119 2.88590 3.11411 3.34045 3.58259 5.26072 3.33810 609 - -

2.68618 4.42225 2.77519 2.73123 3.46354 2.40513 3.72494 3.29354 2.67741 2.69355 4.24690 2.90347 2.73739 3.18146 2.89801 2.37887 2.77519 2.98518 4.58477 3.61503

0.01902 4.36759 5.08994 0.61958 0.77255 0.48576 0.95510

364 3.35445 4.59380 5.30162 4.82944 3.88277 4.85895 5.54839 1.15697 4.74349 2.33726 3.64491 5.00296 5.12500 4.99904 4.91688 4.27970 3.61991 0.95444 5.94021 4.72682 610 - -

2.68618 4.42225 2.77519 2.73123 3.46354 2.40513 3.72494 3.29354 2.67741 2.69355 4.24690 2.90347 2.73739 3.18146 2.89801 2.37887 2.77519 2.98518 4.58477 3.61503

0.01902 4.36759 5.08994 0.61958 0.77255 0.48576 0.95510

365 3.01017 5.02814 3.61408 3.07168 4.68190 3.23629 3.94159 4.05682 2.30345 3.58499 4.46056 3.42917 4.15497 3.10407 0.86781 3.08013 3.03836 3.68687 5.67393 4.49227 611 - -

2.68618 4.42225 2.77519 2.73123 3.46354 2.40513 3.72494 3.29354 2.67741 2.69355 4.24690 2.90347 2.73739 3.18146 2.89801 2.37887 2.77519 2.98518 4.58477 3.61503

0.01902 4.36759 5.08994 0.61958 0.77255 0.48576 0.95510

366 2.95129 4.33617 4.59764 4.03963 3.54592 4.17916 4.60121 1.82727 3.91704 2.36100 2.96720 4.22458 4.53015 4.15063 4.08991 3.28560 2.87596 1.04630 5.21842 4.02251 612 - -

2.68618 4.42225 2.77519 2.73123 3.46354 2.40513 3.72494 3.29354 2.67741 2.69355 4.24690 2.90347 2.73739 3.18146 2.89801 2.37887 2.77519 2.98518 4.58477 3.61503

0.01902 4.36759 5.08994 0.61958 0.77255 0.48576 0.95510

367 3.23512 4.66278 4.45370 4.00559 0.92422 4.17087 3.83948 3.09961 3.87558 2.65977 3.75975 4.03555 4.54695 4.01521 4.01498 2.96648 3.48291 2.79876 4.12094 2.25537 613 - -

2.68618 4.42225 2.77519 2.73123 3.46354 2.40513 3.72494 3.29354 2.67741 2.69355 4.24690 2.90347 2.73739 3.18146 2.89801 2.37887 2.77519 2.98518 4.58477 3.61503

0.01902 4.36759 5.08994 0.61958 0.77255 0.48576 0.95510

368 2.60597 5.14258 2.48313 2.18185 4.46376 2.76345 3.31945 3.93579 2.40330 3.43812 4.19239 2.81119 3.34851 2.75491 2.84016 2.03041 2.91593 3.52089 5.58960 3.91117 614 - -

2.68618 4.42225 2.77519 2.73123 3.46354 2.40513 3.72494 3.29354 2.67741 2.69355 4.24690 2.90347 2.73739 3.18146 2.89801 2.37887 2.77519 2.98518 4.58477 3.61503

0.01902 4.36759 5.08994 0.61958 0.77255 0.48576 0.95510

369 2.37037 5.03657 2.86014 2.14895 3.97292 3.08129 3.49034 3.78070 1.92622 3.31925 4.09145 2.94641 3.65843 2.70151 2.84371 2.56437 2.89170 3.39919 3.73207 4.12429 615 - -

2.68618 4.42225 2.77519 2.73123 3.46354 2.40513 3.72494 3.29354 2.67741 2.69355 4.24690 2.90347 2.73739 3.18146 2.89801 2.37887 2.77519 2.98518 4.58477 3.61503

0.01902 4.36759 5.08994 0.61958 0.77255 0.48576 0.95510

370 2.39458 5.12034 2.70710 2.09638 4.44327 3.26159 3.63442 3.91347 2.28605 3.41570 3.73567 2.91917 2.73046 2.60340 2.48059 2.56748 2.89628 3.49918 5.56603 4.16931 616 - -

2.68626 4.42233 2.77528 2.73126 3.46362 2.40511 3.72503 3.29337 2.67736 2.69350 4.24698 2.90355 2.73743 3.18087 2.89809 2.37895 2.77510 2.98527 4.58456 3.61512

0.27778 1.44232 5.08994 0.57888 0.82218 0.48576 0.95510

371 2.11639 5.10887 2.83061 2.10813 4.42348 3.34782 3.66019 3.88661 2.36054 3.40492 4.16891 2.61722 2.73764 2.77266 2.82253 2.40173 2.87211 3.48538 5.56974 4.17784 622 - -

2.68618 4.42225 2.77519 2.73123 3.46354 2.40513 3.72494 3.29354 2.67741 2.69355 4.24690 2.90347 2.73739 3.18146 2.89801 2.37887 2.77519 2.98518 4.58477 3.61503

0.01902 4.36759 5.08994 0.61958 0.77255 0.48576 0.95510

372 2.65239 4.52706 3.32391 2.66060 3.35645 2.21208 3.81276 2.52316 2.62690 2.75769 3.62872 3.22420 3.51455 3.06514 3.14283 2.39721 2.88679 2.66000 5.09403 3.83016 623 - -

2.68618 4.42225 2.77519 2.73123 3.46354 2.40513 3.72494 3.29354 2.67741 2.69355 4.24690 2.90347 2.73739 3.18146 2.89801 2.37887 2.77519 2.98518 4.58477 3.61503

0.16189 4.36759 1.98938 0.61958 0.77255 0.48576 0.95510

373 2.63228 4.65314 3.15427 2.59739 3.84984 3.49906 3.71273 3.24409 2.47738 2.89401 3.74202 3.08652 2.92286 2.78445 2.67535 2.43801 2.86350 2.69774 5.18425 2.57677 624 - -

2.68618 4.42225 2.77519 2.73123 3.46354 2.40513 3.72494 3.29354 2.67741 2.69355 4.24690 2.90347 2.73739 3.18146 2.89801 2.37887 2.77519 2.98518 4.58477 3.61503

0.02191 4.22762 4.94996 0.61958 0.77255 0.66449 0.72265

374 2.61338 4.20204 3.80464 3.24995 1.83892 3.63177 3.96234 2.67507 3.16759 2.41300 3.34055 3.54333 3.74107 3.43433 3.43319 2.52653 2.66477 2.28564 4.78214 3.39789 625 - -

2.68618 4.42225 2.77519 2.73123 3.46354 2.40513 3.72494 3.29354 2.67741 2.69355 4.24690 2.90347 2.73739 3.18146 2.89801 2.37887 2.77519 2.98518 4.58477 3.61503

0.02191 4.22762 4.94996 0.61958 0.77255 0.38831 1.13383

375 2.76890 5.26480 2.28409 2.22669 4.58948 3.43901 3.49448 4.07118 2.32830 3.55852 4.31980 2.90538 2.59061 1.87205 2.96710 2.72080 3.00749 3.64634 5.69992 4.28358 626 - -

2.68618 4.42225 2.77519 2.73123 3.46354 2.40513 3.72494 3.29354 2.67741 2.69355 4.24690 2.90347 2.73739 3.18146 2.89801 2.37887 2.77519 2.98518 4.58477 3.61503

0.01902 4.36759 5.08994 0.61958 0.77255 0.48576 0.95510

376 2.67379 5.07109 2.72564 2.21339 4.37221 3.45511 2.26699 3.82808 2.16445 3.35611 4.12462 2.78044 3.85170 2.76475 2.80603 2.65769 2.90498 2.97791 5.52803 3.57406 627 - -

2.68618 4.42225 2.77519 2.73123 3.46354 2.40513 3.72494 3.29354 2.67741 2.69355 4.24690 2.90347 2.73739 3.18146 2.89801 2.37887 2.77519 2.98518 4.58477 3.61503

0.01902 4.36759 5.08994 0.61958 0.77255 0.48576 0.95510

377 3.05018 4.39897 4.79660 4.22037 3.35082 4.29706 4.65181 1.99473 4.07945 1.73705 2.62650 4.36710 4.59532 4.23941 4.18665 3.62151 3.28346 1.17780 5.13017 3.59895 628 - -

2.68618 4.42225 2.77519 2.73123 3.46354 2.40513 3.72494 3.29354 2.67741 2.69355 4.24690 2.90347 2.73739 3.18146 2.89801 2.37887 2.77519 2.98518 4.58477 3.61503

0.01902 4.36759 5.08994 0.61958 0.77255 0.48576 0.95510

378 2.89324 4.29375 4.55669 3.96470 2.97640 4.04856 4.33425 2.31474 3.81337 1.40197 2.08955 4.10189 4.37194 3.96440 3.91479 3.35419 2.98826 2.41194 4.83661 2.54792 629 - -

2.68618 4.42225 2.77519 2.73123 3.46354 2.40513 3.72494 3.29354 2.67741 2.69355 4.24690 2.90347 2.73739 3.18146 2.89801 2.37887 2.77519 2.98518 4.58477 3.61503

0.01902 4.36759 5.08994 0.61958 0.77255 0.48576 0.95510

379 2.92703 5.49342 1.46016 1.76291 4.80525 3.04790 3.80355 4.31095 2.35476 3.78380 4.56793 2.72599 3.94662 2.93371 3.06881 2.84389 3.18315 3.87082 5.92427 4.46099 630 - -

2.68618 4.42225 2.77519 2.73123 3.46354 2.40513 3.72494 3.29354 2.67741 2.69355 4.24690 2.90347 2.73739 3.18146 2.89801 2.37887 2.77519 2.98518 4.58477 3.61503
0.01902 4.36759 5.08994 0.61958 0.77255 0.48576 0.95510
380 3.03643 5.12017 3.09343 2.84151 4.48834 3.57787 4.03182 4.00213 2.63754 3.52451 4.49551 3.29664 4.14398 0.87302 2.92542 3.07682 3.12847 3.67818 5.71471 4.39598 631 - -
2.68618 4.42225 2.77519 2.73123 3.46354 2.40513 3.72494 3.29354 2.67741 2.69355 4.24690 2.90347 2.73739 3.18146 2.89801 2.37887 2.77519 2.98518 4.58477 3.61503
0.01902 4.36759 5.08994 0.61958 0.77255 0.48576 0.95510
381 3.37874 4.61765 5.29890 4.80907 3.78826 4.87714 5.49486 0.85592 4.71046 2.13884 3.55491 4.99630 5.11761 4.94137 4.87266 4.28704 3.63669 1.39282 5.85786 4.66877 632 - -
2.68618 4.42225 2.77519 2.73123 3.46354 2.40513 3.72494 3.29354 2.67741 2.69355 4.24690 2.90347 2.73739 3.18146 2.89801 2.37887 2.77519 2.98518 4.58477 3.61503
0.01902 4.36759 5.08994 0.61958 0.77255 0.48576 0.95510
382 2.80764 4.24679 4.33294 3.75181 3.30711 3.95238 4.28132 2.16314 3.62708 1.46453 2.90828 3.95365 4.30377 3.84043 3.79774 3.11920 2.24790 2.00812 4.91733 3.73851 633 - -
2.68618 4.42225 2.77519 2.73123 3.46354 2.40513 3.72494 3.29354 2.67741 2.69355 4.24690 2.90347 2.73739 3.18146 2.89801 2.37887 2.77519 2.98518 4.58477 3.61503
0.01902 4.36759 5.08994 0.61958 0.77255 0.48576 0.95510
383 1.58730 4.71402 3.18244 2.59398 4.17797 3.41338 3.85519 3.58421 2.66410 3.20966 4.03412 3.14861 3.90961 3.03058 2.74075 1.94541 2.69996 3.22792 5.46063 4.15857 634 - -
2.68618 4.42225 2.77519 2.73123 3.46354 2.40513 3.72494 3.29354 2.67741 2.69355 4.24690 2.90347 2.73739 3.18146 2.89801 2.37887 2.77519 2.98518 4.58477 3.61503
0.01902 4.36759 5.08994 0.61958 0.77255 0.48576 0.95510
384 3.28827 0.32295 4.86911 4.73977 4.66193 3.78046 5.28405 3.92672 4.58673 3.80000 4.94570 4.60654 4.51962 4.91175 4.61223 3.55704 3.79696 3.62749 5.93137 4.93730 635 - -
2.68618 4.42225 2.77519 2.73123 3.46354 2.40513 3.72494 3.29354 2.67741 2.69355 4.24690 2.90347 2.73739 3.18146 2.89801 2.37887 2.77519 2.98518 4.58477 3.61503
0.01902 4.36759 5.08994 0.61958 0.77255 0.48576 0.95510
385 1.71845 4.39927 3.61908 3.11764 4.01391 3.05363 4.12020 3.14733 3.08326 3.08294 3.94193 3.41563 3.93004 3.39073 3.44365 2.32471 1.56280 2.87625 5.40420 4.16920 636 - -
2.68618 4.42225 2.77519 2.73123 3.46354 2.40513 3.72494 3.29354 2.67741 2.69355 4.24690 2.90347 2.73739 3.18146 2.89801 2.37887 2.77519 2.98518 4.58477 3.61503
0.01902 4.36759 5.08994 0.61958 0.77255 0.48576 0.95510
386 2.69630 5.02524 3.02422 2.40903 4.31448 3.48936 2.96415 3.75291 2.38131 3.17751 4.08449 2.98793 3.87841 1.90550 2.42854 2.40592 2.80426 3.38747 4.71685 4.12786 637 - -
2.68618 4.42225 2.77519 2.73123 3.46354 2.40513 3.72494 3.29354 2.67741 2.69355 4.24690 2.90347 2.73739 3.18146 2.89801 2.37887 2.77519 2.98518 4.58477 3.61503
0.01902 4.36759 5.08994 0.61958 0.77255 0.48576 0.95510
387 2.69286 5.12454 2.88477 2.30805 4.44978 3.19625 3.64385 3.91049 2.18513 3.41373 3.32234 2.66673 3.85894 2.33899 2.02971 2.66797 2.92105 3.50521 5.56194 4.18042 638 - -
2.68618 4.42225 2.77519 2.73123 3.46354 2.40513 3.72494 3.29354 2.67741 2.69355 4.24690 2.90347 2.73739 3.18146 2.89801 2.37887 2.77519 2.98518 4.58477 3.61503
0.01902 4.36759 5.08994 0.61958 0.77255 0.48576 0.95510
388 3.05934 5.51072 2.53867 0.95710 4.74183 3.47808 3.21322 4.29771 2.68584 3.78852 4.62739 2.95000 4.03041 2.86304 3.13741 2.98033 3.31784 3.90366 5.89613 4.46106 639 - -
2.68618 4.42225 2.77519 2.73123 3.46354 2.40513 3.72494 3.29354 2.67741 2.69355 4.24690 2.90347 2.73739 3.18146 2.89801 2.37887 2.77519 2.98518 4.58477 3.61503
0.01902 4.36759 5.08994 0.61958 0.77255 0.48576 0.95510
389 2.83308 5.22316 3.05645 2.51736 4.58665 3.10897 3.25255 4.02440 1.50896 3.50007 4.28208 2.23896 3.94878 2.79743 2.37843 2.80364 3.04932 3.63020 5.61235 4.27839 640 - -
2.68618 4.42225 2.77519 2.73123 3.46354 2.40513 3.72494 3.29354 2.67741 2.69355 4.24690 2.90347 2.73739 3.18146 2.89801 2.37887 2.77519 2.98518 4.58477 3.61503
0.01902 4.36759 5.08994 0.61958 0.77255 0.48576 0.95510
390 3.60488 4.86986 5.34149 4.79469 3.28965 4.94631 5.29845 1.88659 4.64153 0.69006 3.05720 5.03044 5.07491 4.69275 4.71034 4.33166 3.82997 2.19916 5.46449 4.42579 641 - -
2.68618 4.42225 2.77519 2.73123 3.46354 2.40513 3.72494 3.29354 2.67741 2.69355 4.24690 2.90347 2.73739 3.18146 2.89801 2.37887 2.77519 2.98518 4.58477 3.61503
0.01902 4.36759 5.08994 0.61958 0.77255 0.48576 0.95510

391 3.67321 4.97991 5.20308 4.71742 3.23075 4.86836 5.19751 2.25857 4.47548 0.55884 3.05970 4.95824 5.05415 4.61895 4.56036 4.30279 3.91306 2.52676 5.38660 4.25774 642 - -
2.68618 4.42225 2.77519 2.73123 3.46354 2.40513 3.72494 3.29354 2.67741 2.69355 4.24690 2.90347 2.73739 3.18146 2.89801 2.37887 2.77519 2.98518 4.58477 3.61503
0.01902 4.36759 5.08994 0.61958 0.77255 0.48576 0.95510
392 3.54539 5.18809 4.20814 4.13635 5.04512 3.84929 5.14538 4.75467 4.24336 4.30986 5.39830 4.38159 0.27068 4.61398 4.40637 3.73410 4.02880 4.33588 6.06192 5.18068 643 - -
2.68618 4.42225 2.77519 2.73123 3.46354 2.40513 3.72494 3.29354 2.67741 2.69355 4.24690 2.90347 2.73739 3.18146 2.89801 2.37887 2.77519 2.98518 4.58477 3.61503
0.01902 4.36759 5.08994 0.61958 0.77255 0.48576 0.95510
393 2.56590 5.17452 2.43546 1.57443 4.09377 3.28898 3.66192 3.97030 2.36985 3.46918 4.22558 2.91309 3.85662 2.69972 2.61246 2.67146 2.94100 3.55343 5.61822 4.21382 644 - -
2.68618 4.42225 2.77519 2.73123 3.46354 2.40513 3.72494 3.29354 2.67741 2.69355 4.24690 2.90347 2.73739 3.18146 2.89801 2.37887 2.77519 2.98518 4.58477 3.61503
0.01902 4.36759 5.08994 0.61958 0.77255 0.48576 0.95510
394 2.69487 4.21545 4.39656 3.81715 3.05402 3.96536 4.31136 2.01036 3.68934 2.09399 3.31366 3.99221 3.73093 3.89953 3.84508 3.26937 2.72443 1.32124 4.93382 3.74481 645 - -
2.68618 4.42225 2.77519 2.73123 3.46354 2.40513 3.72494 3.29354 2.67741 2.69355 4.24690 2.90347 2.73739 3.18146 2.89801 2.37887 2.77519 2.98518 4.58477 3.61503
0.03617 4.36759 3.77900 0.61958 0.77255 0.48576 0.95510
395 2.48540 4.49745 2.81967 2.73281 2.70114 3.27423 3.81229 2.60112 2.75351 2.58942 3.59377 3.11831 3.95926 3.07497 3.04108 2.55011 2.55691 2.63357 5.06149 3.80202 646 - -
2.68618 4.42225 2.77519 2.73123 3.46354 2.40513 3.72494 3.29354 2.67741 2.69355 4.24690 2.90347 2.73739 3.18146 2.89801 2.37887 2.77519 2.98518 4.58477 3.61503
0.02998 4.35077 4.09596 0.61958 0.77255 0.51046 0.91684
396 2.46228 4.80995 2.89975 2.40688 4.03793 3.28067 3.69691 3.44820 2.38259 3.06415 3.41310 3.03079 3.64636 2.70774 2.96711 2.57084 2.32433 2.43513 5.32132 3.99547 647 - -
2.68618 4.42225 2.77519 2.73123 3.46354 2.40513 3.72494 3.29354 2.67741 2.69355 4.24690 2.90347 2.73739 3.18146 2.89801 2.37887 2.77519 2.98518 4.58477 3.61503
0.01955 4.34034 5.06268 0.61958 0.77255 0.52526 0.89502
397 2.48024 4.96592 2.80135 2.43258 4.23901 3.03787 3.66096 3.67569 2.25830 2.50078 4.02995 2.86543 3.70947 2.45682 2.90925 2.53279 2.46976 3.31798 5.44759 4.08655 648 - -
2.68618 4.42225 2.77519 2.73123 3.46354 2.40513 3.72494 3.29354 2.67741 2.69355 4.24690 2.90347 2.73739 3.18146 2.89801 2.37887 2.77519 2.98518 4.58477 3.61503
0.17158 4.34034 1.93352 0.61958 0.77255 0.50072 0.93165
398 2.56113 5.05297 2.53980 2.06447 4.35990 2.65783 3.61471 3.82031 2.37872 3.34383 4.11061 2.77766 3.80807 2.65547 2.74942 2.49267 2.71174 3.06618 5.51338 4.12378 649 - -
2.68618 4.42225 2.77519 2.73123 3.46354 2.40513 3.72494 3.29354 2.67741 2.69355 4.24690 2.90347 2.73739 3.18146 2.89801 2.37887 2.77519 2.98518 4.58477 3.61503
0.04314 4.20357 3.60143 0.61958 0.77255 0.40656 1.09643
399 2.59720 5.15256 2.63598 1.53278 4.46683 3.42851 3.47405 3.93539 2.44726 3.44795 4.21373 2.78015 3.30026 2.78523 2.94305 2.60305 2.74275 3.37358 5.60943 4.20790 650 - -
2.68618 4.42225 2.77519 2.73123 3.46354 2.40513 3.72494 3.29354 2.67741 2.69355 4.24690 2.90347 2.73739 3.18146 2.89801 2.37887 2.77519 2.98518 4.58477 3.61503
0.04560 4.33333 3.45941 0.61958 0.77255 0.49517 0.94025
400 2.45428 4.90237 2.92158 2.45708 4.16394 3.08228 3.66802 3.43656 2.45344 3.02874 3.97475 2.79185 3.03168 2.67699 2.85159 2.37242 2.29837 3.14222 5.39948 4.05090 651 - -
2.68618 4.42225 2.77519 2.73123 3.46354 2.40513 3.72494 3.29354 2.67741 2.69355 4.24690 2.90347 2.73739 3.18146 2.89801 2.37887 2.77519 2.98518 4.58477 3.61503
0.07502 4.32549 2.82940 0.61958 0.77255 0.54570 0.86616
401 2.29526 5.03595 2.81635 2.21057 4.02565 3.28484 3.62047 3.79307 2.25460 3.32230 4.08934 2.91157 3.42645 2.24029 2.86024 2.39405 2.79507 3.40289 5.49607 3.88021 652 - -
2.68618 4.42225 2.77519 2.73123 3.46354 2.40513 3.72494 3.29354 2.67741 2.69355 4.24690 2.90347 2.73739 3.18146 2.89801 2.37887 2.77519 2.98518 4.58477 3.61503
0.03663 4.27143 3.81636 0.61958 0.77255 0.61447 0.77854
402 2.06083 4.52240 2.98170 2.71053 3.53080 3.20536 3.78270 3.09951 2.62526 2.78373 3.64914 3.17136 3.70597 3.02144 3.10757 2.63661 2.76495 2.08234 5.11141 3.84118 653 - -
2.68618 4.42225 2.77519 2.73123 3.46354 2.40513 3.72494 3.29354 2.67741 2.69355 4.24690 2.90347 2.73739 3.18146 2.89801 2.37887 2.77519 2.98518 4.58477 3.61503

0.09003 4.25609 2.63228 0.61958 0.77255 0.63253 0.75768

403 2.54087 4.75852 2.90445 2.48713 4.01471 3.29625 3.67142 3.42530 2.48569 3.04166 3.64512 2.79544 3.11101 2.75485 2.94228 2.55493 2.06794 2.90890 5.29777 3.97304 654 - -

2.68618 4.42225 2.77519 2.73123 3.46354 2.40513 3.72494 3.29354 2.67741 2.69355 4.24690 2.90347 2.73739 3.18146 2.89801 2.37887 2.77519 2.98518 4.58477 3.61503

0.04370 4.18885 3.59010 0.61958 0.77255 0.54486 0.86731

404 2.46161 4.96736 2.86438 2.34000 4.24933 3.43111 3.63439 3.69010 2.40144 3.10237 4.03113 2.17941 3.47494 2.65764 2.73466 2.63681 2.40298 3.20064 5.44484 4.07902 655 - -

2.68618 4.42225 2.77519 2.73123 3.46354 2.40513 3.72494 3.29354 2.67741 2.69355 4.24690 2.90347 2.73739 3.18146 2.89801 2.37887 2.77519 2.98518 4.58477 3.61503

0.04778 4.25191 3.42911 0.61958 0.77255 0.47024 0.98044

405 2.23098 4.67284 3.16277 2.60552 3.89380 3.35413 3.57106 2.99061 2.58347 2.93557 3.77837 3.09636 3.15655 2.92820 2.65033 2.58443 2.15905 2.91744 5.22621 3.92870 656 - -

2.68556 4.42250 2.77532 2.73148 3.46379 2.40521 3.72505 3.29369 2.67766 2.69374 4.24715 2.90339 2.73698 3.18128 2.89810 2.37883 2.77492 2.98530 4.58502 3.61512

0.39602 1.40267 2.51248 1.48639 0.25643 0.43752 1.03743

406 2.28051 4.86653 3.01044 2.41971 4.15855 3.29753 3.68423 3.58364 2.47537 3.17296 3.97659 2.79704 3.12130 2.74454 2.86166 1.95572 2.79347 2.98396 5.40129 4.05824 667 - -

2.68618 4.42225 2.77519 2.73123 3.46354 2.40513 3.72494 3.29354 2.67741 2.69355 4.24690 2.90347 2.73739 3.18146 2.89801 2.37887 2.77519 2.98518 4.58477 3.61503

0.04108 4.29474 3.62674 0.61958 0.77255 0.42790 1.05519

407 2.63022 5.07378 2.97882 2.32243 4.38523 3.01053 3.64980 3.83446 2.17826 3.17935 4.13061 2.78115 3.48599 2.76432 1.94530 2.67494 2.60955 3.44772 5.52388 4.15467 668 - -

2.68618 4.42225 2.77519 2.73123 3.46354 2.40513 3.72494 3.29354 2.67741 2.69355 4.24690 2.90347 2.73739 3.18146 2.89801 2.37887 2.77519 2.98518 4.58477 3.61503

0.01938 4.34884 5.07119 0.61958 0.77255 0.46897 0.98255

408 2.48399 4.99997 2.97953 2.38103 4.28760 3.03610 3.49856 3.44677 2.42538 3.28358 4.06452 2.60049 3.32468 2.78319 2.59188 2.04351 2.69075 3.36108 5.47824 4.11024 669 - -

2.68618 4.42225 2.77519 2.73123 3.46354 2.40513 3.72494 3.29354 2.67741 2.69355 4.24690 2.90347 2.73739 3.18146 2.89801 2.37887 2.77519 2.98518 4.58477 3.61503

0.01902 4.36759 5.08994 0.61958 0.77255 0.48576 0.95510

409 2.46269 4.83976 3.06796 2.52023 4.10940 2.91108 3.71220 3.52599 2.51416 2.61025 3.94447 2.72130 3.36568 2.81692 2.84799 2.03752 2.65926 3.19791 5.37553 4.04367 670 - -

2.68618 4.42225 2.77519 2.73123 3.46354 2.40513 3.72494 3.29354 2.67741 2.69355 4.24690 2.90347 2.73739 3.18146 2.89801 2.37887 2.77519 2.98518 4.58477 3.61503

0.01902 4.36759 5.08994 0.61958 0.77255 0.48576 0.95510

410 2.60939 5.21556 3.13644 2.56168 4.58979 3.58216 3.68590 4.01417 1.40768 3.48892 4.27712 2.81302 3.96754 2.56783 2.19068 2.63195 3.06507 3.62822 5.59745 4.28299 671 - -

2.68618 4.42225 2.77519 2.73123 3.46354 2.40513 3.72494 3.29354 2.67741 2.69355 4.24690 2.90347 2.73739 3.18146 2.89801 2.37887 2.77519 2.98518 4.58477 3.61503

0.01902 4.36759 5.08994 0.61958 0.77255 0.48576 0.95510

411 2.65544 4.65429 2.97632 2.64333 3.84360 3.53650 3.59533 3.23218 2.62477 2.11120 3.74173 3.13146 3.10531 2.96284 2.86738 2.32549 2.58227 2.79088 5.19483 3.64367 672 - -

2.68618 4.42225 2.77519 2.73123 3.46354 2.40513 3.72494 3.29354 2.67741 2.69355 4.24690 2.90347 2.73739 3.18146 2.89801 2.37887 2.77519 2.98518 4.58477 3.61503

0.01902 4.36759 5.08994 0.61958 0.77255 0.48576 0.95510

412 2.80073 5.01686 3.16413 2.60349 4.32997 3.56810 3.72172 3.22921 1.31803 3.29127 4.11051 3.09975 3.96394 2.66174 2.69132 2.62926 2.76535 3.39173 5.47966 4.17722 673 - -

2.68618 4.42225 2.77519 2.73123 3.46354 2.40513 3.72494 3.29354 2.67741 2.69355 4.24690 2.90347 2.73739 3.18146 2.89801 2.37887 2.77519 2.98518 4.58477 3.61503

0.01902 4.36759 5.08994 0.61958 0.77255 0.48576 0.95510

413 0.73047 4.31146 3.94543 3.66799 4.51500 3.13922 4.61123 3.81669 3.66102 3.61129 4.46825 3.66059 3.92214 3.93297 3.90838 2.16872 2.91810 3.11259 5.90606 4.72162 674 - -

2.68618 4.42225 2.77519 2.73123 3.46354 2.40513 3.72494 3.29354 2.67741 2.69355 4.24690 2.90347 2.73739 3.18146 2.89801 2.37887 2.77519 2.98518 4.58477 3.61503

0.01902 4.36759 5.08994 0.61958 0.77255 0.48576 0.95510

414 3.31179 4.59528 5.15955 4.69872 3.85475 4.71333 5.42217 1.64438 4.58814 2.33699 3.64413 4.87302 5.03706 4.86630 4.77261 4.13673 3.59047 0.70985 5.87735 4.64850 675 - -

2.68618 4.42225 2.77519 2.73123 3.46354 2.40513 3.72494 3.29354 2.67741 2.69355 4.24690 2.90347 2.73739 3.18146 2.89801 2.37887 2.77519 2.98518 4.58477 3.61503
0.01902 4.36759 5.08994 0.61958 0.77255 0.48576 0.95510
415 3.66438 4.95617 5.26174 4.73929 2.86679 4.90027 5.10086 2.39108 4.58221 0.58276 3.01451 4.95767 5.04463 4.61350 4.64560 4.28966 3.88965 2.47517 5.25110 4.05938 676 - -
2.68618 4.42225 2.77519 2.73123 3.46354 2.40513 3.72494 3.29354 2.67741 2.69355 4.24690 2.90347 2.73739 3.18146 2.89801 2.37887 2.77519 2.98518 4.58477 3.61503
0.01902 4.36759 5.08994 0.61958 0.77255 0.48576 0.95510
416 3.14482 4.46302 4.90737 4.37522 3.72771 4.49076 5.00112 1.59242 4.26823 2.36266 3.31939 4.56279 4.80635 4.51176 4.44711 3.84749 2.76840 0.93261 5.55371 4.35435 677 - -
2.68618 4.42225 2.77519 2.73123 3.46354 2.40513 3.72494 3.29354 2.67741 2.69355 4.24690 2.90347 2.73739 3.18146 2.89801 2.37887 2.77519 2.98518 4.58477 3.61503
0.01902 4.36759 5.08994 0.61958 0.77255 0.48576 0.95510
417 2.01321 4.33534 3.83446 3.39452 4.14151 3.26372 4.33387 3.38377 3.34730 3.19842 4.07568 3.56216 3.94585 3.64102 3.65512 2.37245 1.14926 2.56017 5.55561 4.34813 678 - -
2.68618 4.42225 2.77519 2.73123 3.46354 2.40513 3.72494 3.29354 2.67741 2.69355 4.24690 2.90347 2.73739 3.18146 2.89801 2.37887 2.77519 2.98518 4.58477 3.61503
0.01902 4.36759 5.08994 0.61958 0.77255 0.48576 0.95510
418 2.49507 4.44462 3.61633 3.46080 4.74485 2.70022 4.59970 4.26121 3.62364 3.93021 4.74848 3.58613 3.95557 3.89235 3.90851 0.65636 3.01716 3.60209 6.05503 4.84887 679 - -
2.68612 4.42225 2.77520 2.73123 3.46354 2.40513 3.72495 3.29354 2.67741 2.69355 4.24690 2.90347 2.73740 3.18147 2.89801 2.37887 2.77520 2.98519 4.58477 3.61503
0.03813 3.46552 5.08994 0.51862 0.90471 0.48576 0.95510
419 2.96316 4.58134 3.89609 3.49159 3.43257 3.86416 4.26673 2.79186 3.24856 0.90705 3.45722 3.79497 4.31983 3.35756 3.47162 2.98800 3.24432 2.71901 5.10637 3.83531 681 - -
2.68618 4.42225 2.77519 2.73123 3.46354 2.40513 3.72494 3.29354 2.67741 2.69355 4.24690 2.90347 2.73739 3.18146 2.89801 2.37887 2.77519 2.98518 4.58477 3.61503
0.01902 4.36759 5.08994 0.61958 0.77255 0.48576 0.95510
420 2.66147 4.88288 3.04468 2.48936 3.88856 3.48576 3.36701 3.54794 2.34179 3.14381 3.95220 2.59181 3.64713 2.72127 2.85749 2.35840 2.52681 3.21873 5.37964 2.57856 682 - -
2.68618 4.42225 2.77519 2.73123 3.46354 2.40513 3.72494 3.29354 2.67741 2.69355 4.24690 2.90347 2.73739 3.18146 2.89801 2.37887 2.77519 2.98518 4.58477 3.61503
0.01902 4.36759 5.08994 0.61958 0.77255 0.48576 0.95510
421 2.46353 4.58567 3.32704 2.84079 4.25197 2.96753 3.98331 3.66772 2.83517 3.30068 4.11868 3.23869 1.95716 3.17288 3.08562 1.65468 2.78257 3.00169 5.54972 4.26506 683 - -
2.68618 4.42225 2.77519 2.73123 3.46354 2.40513 3.72494 3.29354 2.67741 2.69355 4.24690 2.90347 2.73739 3.18146 2.89801 2.37887 2.77519 2.98518 4.58477 3.61503
0.01902 4.36759 5.08994 0.61958 0.77255 0.48576 0.95510
422 2.65692 5.01593 2.65883 1.73026 4.26928 2.80257 3.73827 3.71209 2.55691 3.29314 4.10324 2.98159 3.90710 2.88584 3.03481 2.74745 2.98425 3.36740 2.49883 4.14542 684 - -
2.68618 4.42225 2.77519 2.73123 3.46354 2.40513 3.72494 3.29354 2.67741 2.69355 4.24690 2.90347 2.73739 3.18146 2.89801 2.37887 2.77519 2.98518 4.58477 3.61503
0.01902 4.36759 5.08994 0.61958 0.77255 0.48576 0.95510
423 4.10297 5.27084 4.93304 4.71368 1.96572 4.71500 3.68836 3.78160 4.53948 3.04885 4.37201 4.37132 5.03648 4.47030 4.53297 4.13276 4.33341 3.71893 3.80563 0.54881 685 - -
2.68618 4.42225 2.77519 2.73123 3.46354 2.40513 3.72494 3.29354 2.67741 2.69355 4.24690 2.90347 2.73739 3.18146 2.89801 2.37887 2.77519 2.98518 4.58477 3.61503
0.01902 4.36759 5.08994 0.61958 0.77255 0.48576 0.95510
424 2.58248 4.49491 3.58123 3.04774 2.87840 3.69765 3.57704 3.02974 3.00235 2.70611 3.62332 3.43006 4.09325 3.30214 3.34964 1.87291 3.00578 2.80319 4.70735 1.94007 686 - -
2.68618 4.42225 2.77519 2.73123 3.46354 2.40513 3.72494 3.29354 2.67741 2.69355 4.24690 2.90347 2.73739 3.18146 2.89801 2.37887 2.77519 2.98518 4.58477 3.61503
0.01902 4.36759 5.08994 0.61958 0.77255 0.48576 0.95510
425 2.95660 5.51551 1.95664 1.19622 4.82163 3.26941 3.82713 4.32558 2.50505 3.80630 4.60056 2.80448 3.96070 2.96325 3.26361 2.87046 3.21702 3.68113 5.95378 4.48679 687 - -
2.68618 4.42225 2.77519 2.73123 3.46354 2.40513 3.72494 3.29354 2.67741 2.69355 4.24690 2.90347 2.73739 3.18146 2.89801 2.37887 2.77519 2.98518 4.58477 3.61503
0.01902 4.36759 5.08994 0.61958 0.77255 0.48576 0.95510

426 2.73801 5.02118 3.08876 2.51677 4.31293 3.52626 3.68154 2.63219 1.85266 3.28657 4.08520 2.69537 3.91218 2.60588 2.26097 2.56259 2.90453 3.38449 5.47204 4.14074 688 - -
2.68618 4.42225 2.77519 2.73123 3.46354 2.40513 3.72494 3.29354 2.67741 2.69355 4.24690 2.90347 2.73739 3.18146 2.89801 2.37887 2.77519 2.98518 4.58477 3.61503
0.01902 4.36759 5.08994 0.61958 0.77255 0.48576 0.95510
427 3.52839 4.78352 5.35649 4.79426 3.31552 4.90174 5.26234 1.41344 4.66554 0.95805 2.78971 4.99888 5.03684 4.69499 4.71758 4.27288 3.75006 2.14162 5.44979 4.42130 689 - -
2.68618 4.42225 2.77519 2.73123 3.46354 2.40513 3.72494 3.29354 2.67741 2.69355 4.24690 2.90347 2.73739 3.18146 2.89801 2.37887 2.77519 2.98518 4.58477 3.61503
0.01902 4.36759 5.08994 0.61958 0.77255 0.48576 0.95510
428 3.10486 5.36186 3.49153 2.73152 4.81939 3.77127 3.47254 4.17925 1.46920 3.59451 4.42156 3.25508 4.12395 2.54956 1.41201 3.07685 3.04082 3.82289 5.63360 4.42339 690 - -
2.68618 4.42225 2.77519 2.73123 3.46354 2.40513 3.72494 3.29354 2.67741 2.69355 4.24690 2.90347 2.73739 3.18146 2.89801 2.37887 2.77519 2.98518 4.58477 3.61503
0.01902 4.36759 5.08994 0.61958 0.77255 0.48576 0.95510
429 2.47928 5.18220 2.38040 2.35963 4.51102 2.51650 3.66513 3.98788 2.26084 3.48366 4.23833 2.69580 3.85626 2.25835 2.92422 2.43394 2.85954 3.56640 5.63048 4.22273 691 - -
2.68618 4.42225 2.77519 2.73123 3.46354 2.40513 3.72494 3.29354 2.67741 2.69355 4.24690 2.90347 2.73739 3.18146 2.89801 2.37887 2.77519 2.98518 4.58477 3.61503
0.01902 4.36759 5.08994 0.61958 0.77255 0.48576 0.95510
430 2.61657 4.48663 3.43996 2.69032 3.64302 3.62057 3.84882 2.99149 2.46123 2.61735 1.81757 3.30409 4.00260 3.12802 3.00244 2.86533 2.80033 2.57972 5.05413 3.80967 692 - -
2.68618 4.42225 2.77519 2.73123 3.46354 2.40513 3.72494 3.29354 2.67741 2.69355 4.24690 2.90347 2.73739 3.18146 2.89801 2.37887 2.77519 2.98518 4.58477 3.61503
0.01902 4.36759 5.08994 0.61958 0.77255 0.48576 0.95510
431 3.93659 5.14737 4.92372 4.61765 1.97601 4.67842 3.70970 3.54437 4.43513 2.49662 4.15147 4.33988 4.97363 4.39552 4.45539 4.04605 4.15471 3.49016 3.84083 0.65929 693 - -
2.68618 4.42225 2.77519 2.73123 3.46354 2.40513 3.72494 3.29354 2.67741 2.69355 4.24690 2.90347 2.73739 3.18146 2.89801 2.37887 2.77519 2.98518 4.58477 3.61503
0.05488 4.36759 3.20111 0.61958 0.77255 0.48576 0.95510
432 3.19544 4.68951 4.19799 3.70391 2.45239 4.10637 3.42657 3.22161 3.55269 2.70530 3.82677 3.85296 4.46062 3.78100 3.76574 2.65569 3.42302 3.03875 1.48237 1.82383 694 - -
2.68618 4.42225 2.77519 2.73123 3.46354 2.40513 3.72494 3.29354 2.67741 2.69355 4.24690 2.90347 2.73739 3.18146 2.89801 2.37887 2.77519 2.98518 4.58477 3.61503
0.01971 4.33242 5.05477 0.61958 0.77255 0.53624 0.87934
433 2.66587 5.22939 2.87838 1.48798 4.57194 3.49460 3.69489 4.02061 1.89712 3.51080 4.29320 2.97229 3.92153 2.71821 2.66795 2.77791 2.93032 3.62276 5.64329 4.27992 695 - -
2.68618 4.42225 2.77519 2.73123 3.46354 2.40513 3.72494 3.29354 2.67741 2.69355 4.24690 2.90347 2.73739 3.18146 2.89801 2.37887 2.77519 2.98518 4.58477 3.61503
0.01971 4.33242 5.05477 0.61958 0.77255 0.53624 0.87934
434 2.70149 5.03961 2.97836 2.44593 4.33396 2.60741 2.50307 3.78254 2.38523 3.32300 4.10862 2.61593 3.87473 2.78974 2.21628 2.55272 2.93182 3.41152 5.49829 3.89464 696 - -
2.68618 4.42225 2.77519 2.73123 3.46354 2.40513 3.72494 3.29354 2.67741 2.69355 4.24690 2.90347 2.73739 3.18146 2.89801 2.37887 2.77519 2.98518 4.58477 3.61503
0.01971 4.33242 5.05477 0.61958 0.77255 0.53624 0.87934
435 1.48192 4.39247 3.58272 3.29781 4.53324 2.72728 4.40212 3.94291 3.37940 3.62672 4.44985 3.48131 1.34245 3.66333 3.70677 2.61044 2.92193 3.39302 5.86101 4.64976 697 - -
2.68618 4.42225 2.77519 2.73123 3.46354 2.40513 3.72494 3.29354 2.67741 2.69355 4.24690 2.90347 2.73739 3.18146 2.89801 2.37887 2.77519 2.98518 4.58477 3.61503
0.01971 4.33242 5.05477 0.61958 0.77255 0.53624 0.87934
436 2.09290 4.38953 3.59044 3.20630 4.38044 3.18165 4.27785 3.77836 3.22201 3.46143 4.28564 3.24386 3.88326 3.52513 3.57046 1.67316 1.33303 3.28742 5.71832 4.48969 698 - -
2.68612 4.42227 2.77521 2.73125 3.46356 2.40509 3.72496 3.29356 2.67743 2.69357 4.24692 2.90349 2.73742 3.18148 2.89803 2.37883 2.77513 2.98520 4.58479 3.61505
0.04564 3.26404 5.05477 0.87736 0.53765 0.53624 0.87934
437 2.06497 4.68702 3.16073 2.49282 3.90808 3.28241 3.74236 3.30116 2.52087 2.94903 3.65402 3.09952 3.90285 2.93269 2.90455 2.11433 2.75260 2.53133 5.23868 3.93984 703 - -
2.68618 4.42225 2.77519 2.73123 3.46354 2.40513 3.72494 3.29354 2.67741 2.69355 4.24690 2.90347 2.73739 3.18146 2.89801 2.37887 2.77519 2.98518 4.58477 3.61503

0.01971 4.33242 5.05477 0.61958 0.77255 0.45544 1.00558
438 2.65074 4.79169 2.77384 2.58232 4.16076 2.62064 3.79570 3.57479 2.62672 3.19168 3.63703 2.95911 3.89880 2.96144 3.08160 2.56329 1.62175 3.23120 5.44158 4.11746 704 - -
2.68618 4.42225 2.77519 2.73123 3.46354 2.40513 3.72494 3.29354 2.67741 2.69355 4.24690 2.90347 2.73739 3.18146 2.89801 2.37887 2.77519 2.98518 4.58477 3.61503
0.01902 4.36759 5.08994 0.61958 0.77255 0.48576 0.95510
439 2.92744 4.86181 3.51019 3.25980 4.71999 0.71430 4.37707 4.24906 3.10110 3.84019 4.72094 3.59687 4.16833 3.62034 2.48894 3.05358 3.34432 3.78230 5.87075 4.70717 705 - -
2.68618 4.42225 2.77519 2.73123 3.46354 2.40513 3.72494 3.29354 2.67741 2.69355 4.24690 2.90347 2.73739 3.18146 2.89801 2.37887 2.77519 2.98518 4.58477 3.61503
0.03740 4.36759 3.72846 0.61958 0.77255 0.48576 0.95510
440 2.76244 5.18804 2.54397 1.34963 4.50400 3.26301 3.71517 3.96646 2.50858 3.48894 4.26774 2.91994 3.88808 2.83849 2.83933 2.72879 2.73475 3.33650 5.65793 4.25659 706 - -
2.68618 4.42225 2.77519 2.73123 3.46354 2.40513 3.72494 3.29354 2.67741 2.69355 4.24690 2.90347 2.73739 3.18146 2.89801 2.37887 2.77519 2.98518 4.58477 3.61503
0.01937 4.34956 5.07191 0.61958 0.77255 0.46959 0.98152
441 2.26988 4.25600 3.53976 3.16244 3.37646 3.31158 3.95038 1.94890 3.09317 2.45947 3.22645 3.50467 3.75611 3.36907 2.91833 2.84216 2.87918 2.25251 4.15779 3.46802 707 - -
2.68618 4.42225 2.77519 2.73123 3.46354 2.40513 3.72494 3.29354 2.67741 2.69355 4.24690 2.90347 2.73739 3.18146 2.89801 2.37887 2.77519 2.98518 4.58477 3.61503
0.01902 4.36759 5.08994 0.61958 0.77255 0.48576 0.95510
442 3.03372 4.40777 4.73697 4.22406 3.69591 3.48026 4.83327 1.37515 4.10738 2.43029 3.57241 4.38896 4.65733 4.35986 4.28374 3.63918 3.30493 1.08768 5.43978 4.23016 708 - -
2.68618 4.42225 2.77519 2.73123 3.46354 2.40513 3.72494 3.29354 2.67741 2.69355 4.24690 2.90347 2.73739 3.18146 2.89801 2.37887 2.77519 2.98518 4.58477 3.61503
0.01902 4.36759 5.08994 0.61958 0.77255 0.48576 0.95510
443 2.49849 5.08179 2.91806 2.16125 4.39376 2.46008 3.66575 3.84934 2.38402 3.37726 4.14801 2.94428 3.86010 2.27701 2.81499 2.20257 2.92252 3.33302 5.54934 4.16743 709 - -
2.68618 4.42225 2.77519 2.73123 3.46354 2.40513 3.72494 3.29354 2.67741 2.69355 4.24690 2.90347 2.73739 3.18146 2.89801 2.37887 2.77519 2.98518 4.58477 3.61503
0.01902 4.36759 5.08994 0.61958 0.77255 0.48576 0.95510
444 3.28968 4.55182 5.18395 4.66880 3.20290 4.72493 5.23920 1.45644 4.57183 2.21653 3.50805 4.83067 4.98505 4.76954 4.71298 4.10553 3.54089 0.89941 5.63794 4.43021 710 - -
2.68618 4.42225 2.77519 2.73123 3.46354 2.40513 3.72494 3.29354 2.67741 2.69355 4.24690 2.90347 2.73739 3.18146 2.89801 2.37887 2.77519 2.98518 4.58477 3.61503
0.01902 4.36759 5.08994 0.61958 0.77255 0.48576 0.95510
445 3.30207 4.85315 3.70853 3.62518 2.00901 4.15875 1.77438 3.41078 3.55383 2.74590 3.98578 3.82287 4.50819 3.75661 3.81955 3.44469 3.52671 3.22716 4.05800 1.41041 711 - -
2.68618 4.42225 2.77519 2.73123 3.46354 2.40513 3.72494 3.29354 2.67741 2.69355 4.24690 2.90347 2.73739 3.18146 2.89801 2.37887 2.77519 2.98518 4.58477 3.61503
0.01902 4.36759 5.08994 0.61958 0.77255 0.48576 0.95510
446 3.17135 5.33239 3.06172 2.77039 4.66125 3.66680 3.92418 4.16862 2.28000 3.64959 4.55768 3.24861 4.16974 0.91478 2.68916 3.15932 3.41496 3.83607 5.72714 4.44274 712 - -
2.68618 4.42225 2.77519 2.73123 3.46354 2.40513 3.72494 3.29354 2.67741 2.69355 4.24690 2.90347 2.73739 3.18146 2.89801 2.37887 2.77519 2.98518 4.58477 3.61503
0.01902 4.36759 5.08994 0.61958 0.77255 0.48576 0.95510
447 3.54539 5.18809 4.20814 4.13635 5.04512 3.84929 5.14538 4.75467 4.24336 4.30986 5.39830 4.38159 0.27068 4.61398 4.40637 3.73410 4.02880 4.33588 6.06192 5.18068 713 - -
2.68618 4.42225 2.77519 2.73123 3.46354 2.40513 3.72494 3.29354 2.67741 2.69355 4.24690 2.90347 2.73739 3.18146 2.89801 2.37887 2.77519 2.98518 4.58477 3.61503
0.01902 4.36759 5.08994 0.61958 0.77255 0.48576 0.95510
448 2.28129 4.36201 3.75257 3.48892 4.62985 3.13540 4.53765 4.00626 3.52786 3.73074 4.55597 3.57531 3.91087 3.81660 3.81472 0.77977 2.39930 3.41863 5.97121 4.77464 714 - -
2.68618 4.42225 2.77519 2.73123 3.46354 2.40513 3.72494 3.29354 2.67741 2.69355 4.24690 2.90347 2.73739 3.18146 2.89801 2.37887 2.77519 2.98518 4.58477 3.61503
0.01902 4.36759 5.08994 0.61958 0.77255 0.48576 0.95510
449 2.70834 5.00932 3.01919 2.52212 4.23061 3.05973 1.85937 3.71046 2.43089 3.27632 4.08979 3.03919 3.93095 2.21979 2.78912 2.77379 2.99236 3.37105 5.44722 3.70760 715 - -

2.68618 4.42225 2.77519 2.73123 3.46354 2.40513 3.72494 3.29354 2.67741 2.69355 4.24690 2.90347 2.73739 3.18146 2.89801 2.37887 2.77519 2.98518 4.58477 3.61503
0.01902 4.36759 5.08994 0.61958 0.77255 0.48576 0.95510
450 2.74514 5.27831 2.57175 1.14947 4.57419 3.45702 3.79066 4.01499 2.48210 3.16210 4.37057 2.94046 3.95097 2.93008 3.08574 2.83365 3.12528 3.63574 5.74513 4.34346 716 - -
2.68618 4.42225 2.77519 2.73123 3.46354 2.40513 3.72494 3.29354 2.67741 2.69355 4.24690 2.90347 2.73739 3.18146 2.89801 2.37887 2.77519 2.98518 4.58477 3.61503
0.01902 4.36759 5.08994 0.61958 0.77255 0.48576 0.95510
451 2.68107 4.99285 2.75769 1.82299 4.26592 2.73608 3.67801 2.73638 2.45156 3.14231 4.05761 2.97231 3.86979 2.80781 2.61362 2.68383 2.91372 3.34263 5.47255 4.11050 717 - -
2.68618 4.42225 2.77519 2.73123 3.46354 2.40513 3.72494 3.29354 2.67741 2.69355 4.24690 2.90347 2.73739 3.18146 2.89801 2.37887 2.77519 2.98518 4.58477 3.61503
0.01902 4.36759 5.08994 0.61958 0.77255 0.48576 0.95510
452 2.65868 4.68390 3.08335 2.56385 3.75384 3.37894 3.16088 3.27026 2.44120 2.83375 3.76817 3.11643 3.91910 2.78341 2.82861 2.74782 2.88971 2.82377 4.53388 2.15255 718 - -
2.68618 4.42225 2.77519 2.73123 3.46354 2.40513 3.72494 3.29354 2.67741 2.69355 4.24690 2.90347 2.73739 3.18146 2.89801 2.37887 2.77519 2.98518 4.58477 3.61503
0.01902 4.36759 5.08994 0.61958 0.77255 0.48576 0.95510
453 3.40513 5.33925 3.35795 3.15211 4.57541 3.78360 4.30295 4.31917 2.94384 3.76573 4.80952 3.60503 4.36268 0.55884 3.20449 3.44586 3.71597 4.02497 5.76482 4.51091 719 - -
2.68618 4.42225 2.77519 2.73123 3.46354 2.40513 3.72494 3.29354 2.67741 2.69355 4.24690 2.90347 2.73739 3.18146 2.89801 2.37887 2.77519 2.98518 4.58477 3.61503
0.03813 4.36759 3.69960 0.61958 0.77255 0.48576 0.95510
454 2.64959 5.12600 3.05224 2.38690 4.45815 3.51425 3.18421 3.89842 2.22235 3.25938 3.92048 2.90035 3.89933 1.98047 1.99664 2.72871 2.96766 3.51187 5.54424 4.19676 720 - -
2.68618 4.42225 2.77519 2.73123 3.46354 2.40513 3.72494 3.29354 2.67741 2.69355 4.24690 2.90347 2.73739 3.18146 2.89801 2.37887 2.77519 2.98518 4.58477 3.61503
0.01938 4.34884 5.07119 0.61958 0.77255 0.51321 0.91272
455 2.65371 4.25426 3.77043 3.20101 2.58812 3.68188 3.96198 2.71932 3.12607 2.44621 2.90891 3.52978 4.06415 3.39989 3.41285 2.42269 1.87754 2.51362 4.83130 3.27274 721 - -
2.68618 4.42225 2.77519 2.73123 3.46354 2.40513 3.72494 3.29354 2.67741 2.69355 4.24690 2.90347 2.73739 3.18146 2.89801 2.37887 2.77519 2.98518 4.58477 3.61503
0.01938 4.34884 5.07119 0.61958 0.77255 0.51321 0.91272
456 2.71776 5.18452 2.06870 2.12425 4.09927 2.31348 3.67120 3.97595 2.29800 3.47844 4.23909 2.90402 3.55899 2.68225 2.94451 2.68078 2.85395 3.56130 5.63184 4.22399 722 - -
2.68618 4.42225 2.77519 2.73123 3.46354 2.40513 3.72494 3.29354 2.67741 2.69355 4.24690 2.90347 2.73739 3.18146 2.89801 2.37887 2.77519 2.98518 4.58477 3.61503
0.01938 4.34884 5.07119 0.61958 0.77255 0.51321 0.91272
457 2.50563 5.14485 2.72495 2.22398 4.47378 3.43759 3.63640 3.94729 2.05838 3.44209 4.19289 2.24306 3.83924 2.74070 2.59577 2.29129 2.90814 3.39820 5.58621 4.18611 723 - -
2.68618 4.42225 2.77519 2.73123 3.46354 2.40513 3.72494 3.29354 2.67741 2.69355 4.24690 2.90347 2.73739 3.18146 2.89801 2.37887 2.77519 2.98518 4.58477 3.61503
0.01938 4.34884 5.07119 0.61958 0.77255 0.51321 0.91272
458 2.72418 5.09595 3.02404 2.46004 4.41885 2.94483 3.65670 3.86104 1.69049 3.09505 4.15430 2.76323 3.88619 2.69116 2.34974 2.63163 2.68291 3.47717 5.53201 4.17754 724 - -
2.68618 4.42225 2.77519 2.73123 3.46354 2.40513 3.72494 3.29354 2.67741 2.69355 4.24690 2.90347 2.73739 3.18146 2.89801 2.37887 2.77519 2.98518 4.58477 3.61503
0.01938 4.34884 5.07119 0.61958 0.77255 0.46897 0.98255
459 2.26359 4.38593 3.50526 2.84267 3.33104 3.45916 3.87563 2.88701 2.67735 2.05910 2.86815 3.35060 4.00349 3.19953 3.25269 2.35220 2.71436 2.61279 4.96729 3.73033 725 - -
2.68618 4.42225 2.77519 2.73123 3.46354 2.40513 3.72494 3.29354 2.67741 2.69355 4.24690 2.90347 2.73739 3.18146 2.89801 2.37887 2.77519 2.98518 4.58477 3.61503
0.01902 4.36759 5.08994 0.61958 0.77255 0.48576 0.95510
460 3.04885 5.18738 2.87719 2.49015 4.28429 3.57237 1.05798 3.98982 2.66622 3.54402 4.43014 3.16380 3.61184 3.14619 3.01059 3.05481 3.31893 3.65811 5.56460 4.16856 726 - -
2.68618 4.42225 2.77519 2.73123 3.46354 2.40513 3.72494 3.29354 2.67741 2.69355 4.24690 2.90347 2.73739 3.18146 2.89801 2.37887 2.77519 2.98518 4.58477 3.61503
0.01902 4.36759 5.08994 0.61958 0.77255 0.48576 0.95510

461 3.13613 5.75336 1.20560 2.19576 5.05885 3.40206 3.95508 4.59838 2.98598 4.07203 4.91463 1.67126 4.02970 3.11806 3.60097 2.77863 3.42858 4.14660 6.22439 4.69371 727 - -
2.68618 4.42225 2.77519 2.73123 3.46354 2.40513 3.72494 3.29354 2.67741 2.69355 4.24690 2.90347 2.73739 3.18146 2.89801 2.37887 2.77519 2.98518 4.58477 3.61503
0.01902 4.36759 5.08994 0.61958 0.77255 0.48576 0.95510
462 2.41778 4.91503 3.06278 2.45732 4.17188 3.13607 3.69596 3.58883 2.36874 3.17722 2.86471 3.02900 3.89649 1.86895 2.73532 2.71944 2.92330 3.25856 5.40358 4.07184 728 - -
2.68618 4.42225 2.77519 2.73123 3.46354 2.40513 3.72494 3.29354 2.67741 2.69355 4.24690 2.90347 2.73739 3.18146 2.89801 2.37887 2.77519 2.98518 4.58477 3.61503
0.01902 4.36759 5.08994 0.61958 0.77255 0.48576 0.95510
463 3.36770 5.45617 3.82605 3.08783 4.99246 3.92871 3.80116 4.31052 0.82582 3.69188 4.57422 3.45546 4.28167 2.93126 1.81815 3.34897 3.50784 3.99213 5.67535 4.55646 729 - -
2.68618 4.42225 2.77519 2.73123 3.46354 2.40513 3.72494 3.29354 2.67741 2.69355 4.24690 2.90347 2.73739 3.18146 2.89801 2.37887 2.77519 2.98518 4.58477 3.61503
0.01902 4.36759 5.08994 0.61958 0.77255 0.48576 0.95510
464 0.48610 4.52363 4.05151 3.90062 4.63123 3.32935 4.85045 3.83932 3.93244 3.69441 4.69080 3.89759 4.11714 4.22893 4.12566 2.88015 3.19325 3.39969 6.00219 4.87285 730 - -
2.68618 4.42225 2.77519 2.73123 3.46354 2.40513 3.72494 3.29354 2.67741 2.69355 4.24690 2.90347 2.73739 3.18146 2.89801 2.37887 2.77519 2.98518 4.58477 3.61503
0.01902 4.36759 5.08994 0.61958 0.77255 0.48576 0.95510
465 2.85524 3.13496 4.54205 3.95132 3.11626 3.99831 4.27408 2.48987 3.77276 1.15262 3.00653 4.06400 4.33827 3.94188 3.86805 3.30822 3.08719 2.40059 3.01455 3.54713 731 - -
2.68618 4.42225 2.77519 2.73123 3.46354 2.40513 3.72494 3.29354 2.67741 2.69355 4.24690 2.90347 2.73739 3.18146 2.89801 2.37887 2.77519 2.98518 4.58477 3.61503
0.01902 4.36759 5.08994 0.61958 0.77255 0.48576 0.95510
466 0.72259 4.31102 3.97326 3.68145 4.45302 3.15954 4.60428 3.68990 3.65566 3.52937 4.40825 3.67491 3.93523 3.93317 3.89954 2.33646 2.92392 2.93008 5.86344 4.67578 732 - -
2.68618 4.42225 2.77519 2.73123 3.46354 2.40513 3.72494 3.29354 2.67741 2.69355 4.24690 2.90347 2.73739 3.18146 2.89801 2.37887 2.77519 2.98518 4.58477 3.61503
0.01902 4.36759 5.08994 0.61958 0.77255 0.48576 0.95510
467 3.49436 5.61867 2.83110 0.48423 5.04562 3.68015 4.34993 4.61026 3.30511 4.15618 5.14062 3.35048 4.31736 3.57532 3.74473 3.44198 3.81256 4.25293 6.11616 4.88247 733 - -
2.68618 4.42225 2.77519 2.73123 3.46354 2.40513 3.72494 3.29354 2.67741 2.69355 4.24690 2.90347 2.73739 3.18146 2.89801 2.37887 2.77519 2.98518 4.58477 3.61503
0.01902 4.36759 5.08994 0.61958 0.77255 0.48576 0.95510
468 3.38333 4.68220 5.12381 4.58728 3.40897 4.69826 5.11211 1.10107 4.42532 1.81747 1.80996 4.78462 4.92810 4.57930 4.53443 4.07201 3.62324 2.08637 5.44533 4.33552 734 - -
2.68618 4.42225 2.77519 2.73123 3.46354 2.40513 3.72494 3.29354 2.67741 2.69355 4.24690 2.90347 2.73739 3.18146 2.89801 2.37887 2.77519 2.98518 4.58477 3.61503
0.01902 4.36759 5.08994 0.61958 0.77255 0.48576 0.95510
469 3.48761 4.84806 4.64519 4.19535 2.27239 4.41273 3.85155 3.10971 4.03411 2.36283 3.12104 4.18405 4.72538 4.14226 4.15624 3.74034 3.71175 3.05013 4.07417 0.84003 735 - -
2.68618 4.42225 2.77519 2.73123 3.46354 2.40513 3.72494 3.29354 2.67741 2.69355 4.24690 2.90347 2.73739 3.18146 2.89801 2.37887 2.77519 2.98518 4.58477 3.61503
0.01902 4.36759 5.08994 0.61958 0.77255 0.48576 0.95510
470 3.76585 5.09668 4.98648 4.65545 3.40807 4.60815 5.16228 2.66140 4.42265 0.44071 3.32239 4.88679 4.97522 4.66557 4.50633 4.28261 4.04609 2.82202 5.43615 4.26530 736 - -
2.68618 4.42225 2.77519 2.73123 3.46354 2.40513 3.72494 3.29354 2.67741 2.69355 4.24690 2.90347 2.73739 3.18146 2.89801 2.37887 2.77519 2.98518 4.58477 3.61503
0.01902 4.36759 5.08994 0.61958 0.77255 0.48576 0.95510
471 3.41109 4.89010 4.41396 4.02029 3.30194 4.34757 4.68215 2.64942 3.71183 0.63169 3.24001 4.31569 4.70786 3.58094 3.88258 3.79853 3.67037 2.77627 5.23902 4.00887 737 - -
2.68618 4.42225 2.77519 2.73123 3.46354 2.40513 3.72494 3.29354 2.67741 2.69355 4.24690 2.90347 2.73739 3.18146 2.89801 2.37887 2.77519 2.98518 4.58477 3.61503
0.01902 4.36759 5.08994 0.61958 0.77255 0.48576 0.95510
472 2.51248 4.45684 3.62961 3.48207 4.73533 2.85802 4.61381 4.26301 3.63730 3.93765 4.76395 3.60386 3.96902 3.91378 3.91411 0.62706 3.03557 3.61067 6.04729 4.83864 738 - -
2.68618 4.42225 2.77519 2.73123 3.46354 2.40513 3.72494 3.29354 2.67741 2.69355 4.24690 2.90347 2.73739 3.18146 2.89801 2.37887 2.77519 2.98518 4.58477 3.61503

0.01902 4.36759 5.08994 0.61958 0.77255 0.48576 0.95510

473 2.74068 3.71703 4.45073 3.85385 2.48695 3.88786 4.19589 2.47917 3.69211 1.38070 2.73869 3.96137 4.23938 3.85786 3.78548 3.19080 2.66768 2.34098 4.73913 3.17000 739 - -

2.68618 4.42225 2.77519 2.73123 3.46354 2.40513 3.72494 3.29354 2.67741 2.69355 4.24690 2.90347 2.73739 3.18146 2.89801 2.37887 2.77519 2.98518 4.58477 3.61503

0.01937 4.34956 5.07191 0.61958 0.77255 0.48576 0.95510

474 2.24725 2.71293 3.98163 3.53968 4.16611 3.19411 4.40697 3.51533 3.47298 3.25656 4.11337 3.60920 3.91043 3.74262 3.74948 1.64638 1.39759 3.09048 5.57687 4.38856 740 - -

2.68618 4.42225 2.77519 2.73123 3.46354 2.40513 3.72494 3.29354 2.67741 2.69355 4.24690 2.90347 2.73739 3.18146 2.89801 2.37887 2.77519 2.98518 4.58477 3.61503

0.01937 4.34956 5.07191 0.61958 0.77255 0.48576 0.95510

475 3.58782 5.70718 0.42178 2.76869 5.16839 3.65128 4.45780 4.88404 3.64643 4.41231 5.42630 3.34032 4.33782 3.71470 4.20863 3.51282 3.94432 4.47910 6.23121 4.99499 741 - -

2.68618 4.42225 2.77519 2.73123 3.46354 2.40513 3.72494 3.29354 2.67741 2.69355 4.24690 2.90347 2.73739 3.18146 2.89801 2.37887 2.77519 2.98518 4.58477 3.61503

0.01937 4.34956 5.07191 0.61958 0.77255 0.48576 0.95510

476 2.14103 4.60490 3.24632 2.60359 3.78453 3.55163 3.77582 3.16566 2.39406 2.83635 3.69695 2.87554 3.93927 2.99899 2.96787 2.77893 2.89182 2.24882 5.15203 2.77497 742 - -

2.68618 4.42225 2.77519 2.73123 3.46354 2.40513 3.72494 3.29354 2.67741 2.69355 4.24690 2.90347 2.73739 3.18146 2.89801 2.37887 2.77519 2.98518 4.58477 3.61503

0.01937 4.34956 5.07191 0.61958 0.77255 0.48576 0.95510

477 2.84906 4.35358 4.68596 4.11313 3.38560 4.21702 4.58734 1.51557 3.97553 1.38944 3.10336 4.27435 4.53582 4.16317 4.10584 3.28367 3.22378 1.87587 5.11895 3.95577 743 - -

2.68618 4.42225 2.77519 2.73123 3.46354 2.40513 3.72494 3.29354 2.67741 2.69355 4.24690 2.90347 2.73739 3.18146 2.89801 2.37887 2.77519 2.98518 4.58477 3.61503

0.01937 4.34956 5.07191 0.61958 0.77255 0.48576 0.95510

478 3.03721 4.53246 5.04152 4.52712 3.75074 4.63907 5.19392 1.71824 4.42048 2.02313 3.55038 4.72353 4.92997 4.66761 4.60104 4.01642 3.49978 0.83341 5.68749 4.49192 744 - -

2.68618 4.42225 2.77519 2.73123 3.46354 2.40513 3.72494 3.29354 2.67741 2.69355 4.24690 2.90347 2.73739 3.18146 2.89801 2.37887 2.77519 2.98518 4.58477 3.61503

0.01937 4.34956 5.07191 0.61958 0.77255 0.48576 0.95510

479 2.67090 4.41685 3.92466 3.39481 3.69603 3.71007 4.27309 2.45373 3.29674 2.54266 3.35026 3.70170 4.19559 3.61681 3.60248 3.04276 1.24802 2.16787 5.27352 4.05666 745 - -

2.68618 4.42225 2.77520 2.73124 3.46354 2.40513 3.72495 3.29354 2.67741 2.69355 4.24690 2.90347 2.73740 3.18147 2.89801 2.37887 2.77512 2.98519 4.58477 3.61503

0.04323 3.32307 5.07191 0.50053 0.93193 0.48576 0.95510

480 2.41442 4.36990 3.65663 3.40992 4.64867 2.81019 4.50043 4.08344 3.48950 3.76496 4.57373 3.53026 3.89562 3.77089 3.79102 0.79023 2.45261 3.46530 5.97267 4.76920 747 - -

2.68618 4.42225 2.77519 2.73123 3.46354 2.40513 3.72494 3.29354 2.67741 2.69355 4.24690 2.90347 2.73739 3.18146 2.89801 2.37887 2.77519 2.98518 4.58477 3.61503

0.01937 4.34956 5.07191 0.61958 0.77255 0.48576 0.95510

481 2.00529 4.91363 2.15769 2.52324 4.41102 1.86098 3.85104 3.84446 2.70493 3.16767 4.24184 3.03072 3.90406 3.00616 3.18538 2.65567 2.99735 3.44503 5.64984 4.29130 748 - -

2.68618 4.42225 2.77519 2.73123 3.46354 2.40513 3.72494 3.29354 2.67741 2.69355 4.24690 2.90347 2.73739 3.18146 2.89801 2.37887 2.77519 2.98518 4.58477 3.61503

0.01937 4.34956 5.07191 0.61958 0.77255 0.48576 0.95510

482 2.50207 4.22733 3.79401 3.21861 2.89924 3.68601 3.96344 2.69472 3.13688 2.39342 2.99174 3.53993 4.06138 3.40792 3.00744 2.33676 2.79853 2.43041 2.43109 3.60834 749 - -

2.68618 4.42225 2.77519 2.73123 3.46354 2.40513 3.72494 3.29354 2.67741 2.69355 4.24690 2.90347 2.73739 3.18146 2.89801 2.37887 2.77519 2.98518 4.58477 3.61503

0.01937 4.34956 5.07191 0.61958 0.77255 0.48576 0.95510

483 2.62527 4.53817 3.70974 3.57668 4.60603 3.26348 4.65617 4.20959 3.68601 3.89993 4.79999 3.69886 4.04645 3.99832 3.93523 0.55002 3.14559 3.63151 5.94165 4.70151 750 - -

2.68618 4.42225 2.77519 2.73123 3.46354 2.40513 3.72494 3.29354 2.67741 2.69355 4.24690 2.90347 2.73739 3.18146 2.89801 2.37887 2.77519 2.98518 4.58477 3.61503

0.01937 4.34956 5.07191 0.61958 0.77255 0.48576 0.95510

484 2.48583 4.39131 3.74891 3.52302 4.45506 3.19849 4.53474 3.73931 3.50667 3.55545 4.45690 3.61742 3.96352 3.84852 3.75779 2.46701 0.75874 3.27094 5.85108 4.62933 751 - -

2.68618 4.42225 2.77519 2.73123 3.46354 2.40513 3.72494 3.29354 2.67741 2.69355 4.24690 2.90347 2.73739 3.18146 2.89801 2.37887 2.77519 2.98518 4.58477 3.61503
0.01937 4.34956 5.07191 0.61958 0.77255 0.48576 0.95510
485 3.56087 4.89291 4.93225 4.52529 0.83679 4.55415 4.20744 2.72694 4.35548 1.94404 3.44777 4.48746 4.86579 4.41524 4.42216 3.94138 3.81564 2.59951 4.38808 2.76293 752 - -
2.68618 4.42225 2.77519 2.73123 3.46354 2.40513 3.72494 3.29354 2.67741 2.69355 4.24690 2.90347 2.73739 3.18146 2.89801 2.37887 2.77519 2.98518 4.58477 3.61503
0.01937 4.34956 5.07191 0.61958 0.77255 0.48576 0.95510
486 2.60505 4.50166 3.76794 3.61860 4.58305 0.64584 4.67309 3.98271 3.71211 3.75030 4.67992 3.71837 4.04347 4.01489 3.95476 2.79669 3.12107 2.99708 5.94088 4.73879 753 - -
2.68618 4.42225 2.77519 2.73123 3.46354 2.40513 3.72494 3.29354 2.67741 2.69355 4.24690 2.90347 2.73739 3.18146 2.89801 2.37887 2.77519 2.98518 4.58477 3.61503
0.01937 4.34956 5.07191 0.61958 0.77255 0.48576 0.95510
487 3.41583 5.03770 3.69552 3.04474 2.74236 4.02717 3.89767 3.61221 3.40814 3.12910 4.24146 3.76536 4.50355 3.77518 3.65105 3.52315 3.68247 3.43153 4.36683 0.72438 754 - -
2.68618 4.42225 2.77519 2.73123 3.46354 2.40513 3.72494 3.29354 2.67741 2.69355 4.24690 2.90347 2.73739 3.18146 2.89801 2.37887 2.77519 2.98518 4.58477 3.61503
0.01937 4.34956 5.07191 0.61958 0.77255 0.48576 0.95510
488 2.71215 4.47347 4.77985 4.26038 3.72620 4.37362 4.94346 1.88618 4.14454 2.34690 3.20006 4.46202 4.73655 4.41114 4.34657 3.73916 3.37757 0.83953 5.55508 4.35426 755 - -
2.68618 4.42225 2.77519 2.73123 3.46354 2.40513 3.72494 3.29354 2.67741 2.69355 4.24690 2.90347 2.73739 3.18146 2.89801 2.37887 2.77519 2.98518 4.58477 3.61503
0.01937 4.34956 5.07191 0.61958 0.77255 0.48576 0.95510
489 0.87560 4.31841 3.79241 3.52576 4.62418 2.22402 4.55721 4.05291 3.59795 3.74004 4.54517 3.57827 3.88336 3.84645 3.88459 2.10858 2.89429 3.42971 5.96492 4.78371 756 - -
2.68618 4.42225 2.77519 2.73123 3.46354 2.40513 3.72494 3.29354 2.67741 2.69355 4.24690 2.90347 2.73739 3.18146 2.89801 2.37887 2.77519 2.98518 4.58477 3.61503
0.01937 4.34956 5.07191 0.61958 0.77255 0.48576 0.95510
490 2.64839 4.97280 3.02098 2.51017 4.14196 3.50788 2.68665 3.66743 2.44682 3.23850 4.05253 3.02804 3.91315 1.71979 2.87277 2.53747 2.96308 3.33020 5.39193 3.36584 757 - -
2.68618 4.42225 2.77519 2.73123 3.46354 2.40513 3.72494 3.29354 2.67741 2.69355 4.24690 2.90347 2.73739 3.18146 2.89801 2.37887 2.77519 2.98518 4.58477 3.61503
0.01937 4.34956 5.07191 0.61958 0.77255 0.48576 0.95510
491 2.12432 4.35553 3.64262 3.46731 4.74572 0.90709 4.58895 4.19712 3.63838 3.87501 4.67456 3.55283 3.89341 3.87467 3.92729 2.01023 2.92898 3.52254 6.07036 4.88280 758 - -
2.68618 4.42225 2.77519 2.73123 3.46354 2.40513 3.72494 3.29354 2.67741 2.69355 4.24690 2.90347 2.73739 3.18146 2.89801 2.37887 2.77519 2.98518 4.58477 3.61503
0.01937 4.34956 5.07191 0.61958 0.77255 0.48576 0.95510
492 3.69934 4.99787 5.18047 4.71023 2.89431 4.85049 5.03277 2.44653 4.53260 0.55114 3.05417 4.92167 5.03463 4.60371 4.60178 4.29049 3.93301 2.64436 5.19098 3.92946 759 - -
2.68618 4.42225 2.77519 2.73123 3.46354 2.40513 3.72494 3.29354 2.67741 2.69355 4.24690 2.90347 2.73739 3.18146 2.89801 2.37887 2.77519 2.98518 4.58477 3.61503
0.01937 4.34956 5.07191 0.61958 0.77255 0.48576 0.95510
493 1.31908 4.33032 3.74985 3.48999 4.65775 1.34770 4.54749 4.09523 3.58037 3.77336 4.57285 3.56123 3.88162 3.82770 3.87697 2.13039 2.89770 3.45629 5.98890 4.80325 760 - -
2.68618 4.42225 2.77519 2.73123 3.46354 2.40513 3.72494 3.29354 2.67741 2.69355 4.24690 2.90347 2.73739 3.18146 2.89801 2.37887 2.77519 2.98518 4.58477 3.61503
0.01937 4.34956 5.07191 0.61958 0.77255 0.48576 0.95510
494 3.36705 5.06793 4.13526 4.10606 5.18829 0.26688 5.16884 4.93167 4.35009 4.51300 5.51120 4.30214 4.47049 4.63729 4.50790 3.55643 3.88379 4.36718 6.13016 5.31529 761 - -
2.68618 4.42225 2.77519 2.73123 3.46354 2.40513 3.72494 3.29354 2.67741 2.69355 4.24690 2.90347 2.73739 3.18146 2.89801 2.37887 2.77519 2.98518 4.58477 3.61503
0.01937 4.34956 5.07191 0.61958 0.77255 0.48576 0.95510
495 3.48048 4.81187 5.10061 4.59677 3.31341 4.70325 5.13811 2.24858 4.41390 0.71846 3.12077 4.81435 4.94685 4.56159 4.52215 4.10657 3.73266 1.90012 5.42015 4.31344 762 - -
2.68618 4.42225 2.77519 2.73123 3.46354 2.40513 3.72494 3.29354 2.67741 2.69355 4.24690 2.90347 2.73739 3.18146 2.89801 2.37887 2.77519 2.98518 4.58477 3.61503
0.01937 4.34956 5.07191 0.61958 0.77255 0.48576 0.95510

496 2.92523 5.16848 3.30972 2.69514 4.56814 3.64590 3.71722 3.95718 1.50790 3.45037 4.26787 3.16600 4.02875 2.84308 1.85478 2.80681 2.20883 3.60245 5.56280 4.30078 763 - -
2.68618 4.42225 2.77519 2.73123 3.46354 2.40513 3.72494 3.29354 2.67741 2.69355 4.24690 2.90347 2.73739 3.18146 2.89801 2.37887 2.77519 2.98518 4.58477 3.61503
0.01937 4.34956 5.07191 0.61958 0.77255 0.48576 0.95510
497 2.58510 4.66601 3.65383 3.52794 4.65368 3.37934 4.65625 4.08179 3.62785 3.77315 4.75202 3.73237 0.61437 3.98116 3.88169 2.95511 3.27615 3.61786 5.95700 4.79048 764 - -
2.68618 4.42225 2.77519 2.73123 3.46354 2.40513 3.72494 3.29354 2.67741 2.69355 4.24690 2.90347 2.73739 3.18146 2.89801 2.37887 2.77519 2.98518 4.58477 3.61503
0.01937 4.34956 5.07191 0.61958 0.77255 0.48576 0.95510
498 2.63376 4.48561 3.67595 3.10003 3.40215 3.70787 3.88181 3.00473 2.79205 2.68298 3.60581 3.46168 4.09903 3.26070 2.59412 2.99316 2.86065 2.78542 1.63240 3.31953 765 - -
2.68618 4.42225 2.77519 2.73123 3.46354 2.40513 3.72494 3.29354 2.67741 2.69355 4.24690 2.90347 2.73739 3.18146 2.89801 2.37887 2.77519 2.98518 4.58477 3.61503
0.01937 4.34956 5.07191 0.61958 0.77255 0.48576 0.95510
499 3.08274 4.42018 4.82898 4.26096 3.42309 4.35852 4.73859 1.34324 4.12409 1.80241 3.01682 4.42263 4.65184 3.88161 4.24650 3.68919 3.31886 1.44922 5.22560 4.07219 766 - -
2.68618 4.42225 2.77519 2.73123 3.46354 2.40513 3.72494 3.29354 2.67741 2.69355 4.24690 2.90347 2.73739 3.18146 2.89801 2.37887 2.77519 2.98518 4.58477 3.61503
0.01937 4.34956 5.07191 0.61958 0.77255 0.48576 0.95510
500 3.64883 4.93951 5.26378 4.70721 3.17491 4.90400 5.18094 2.38676 4.51213 0.75682 1.80319 4.96223 5.02188 4.56954 4.57573 4.28462 3.86321 2.60536 5.34793 4.32142 767 - -
2.68618 4.42225 2.77519 2.73123 3.46354 2.40513 3.72494 3.29354 2.67741 2.69355 4.24690 2.90347 2.73739 3.18146 2.89801 2.37887 2.77519 2.98518 4.58477 3.61503
0.01937 4.34956 5.07191 0.61958 0.77255 0.48576 0.95510
501 2.58693 4.16963 3.98746 3.40772 2.72529 3.73405 3.77235 2.54664 3.30471 2.11935 2.88624 3.39583 3.61300 3.54399 3.52472 3.01264 2.88877 2.42627 4.72657 2.01450 768 - -
2.68618 4.42225 2.77519 2.73123 3.46354 2.40513 3.72494 3.29354 2.67741 2.69355 4.24690 2.90347 2.73739 3.18146 2.89801 2.37887 2.77519 2.98518 4.58477 3.61503
0.01937 4.34956 5.07191 0.61958 0.77255 0.48576 0.95510
502 2.76285 5.10431 3.09070 2.50923 4.43461 3.53106 3.66654 3.86473 1.56447 3.27797 4.16531 3.02467 3.45128 2.62915 2.21648 2.66798 2.68714 3.32471 5.52774 4.19404 769 - -
2.68618 4.42225 2.77519 2.73123 3.46354 2.40513 3.72494 3.29354 2.67741 2.69355 4.24690 2.90347 2.73739 3.18146 2.89801 2.37887 2.77519 2.98518 4.58477 3.61503
0.01937 4.34956 5.07191 0.61958 0.77255 0.48576 0.95510
503 2.33405 4.72706 3.17785 2.69194 4.13280 3.44669 3.83961 3.53531 2.62219 3.03097 3.99936 3.15374 1.64383 2.90618 2.79403 2.50791 2.94744 3.20044 5.41890 4.12499 770 - -
2.68618 4.42225 2.77519 2.73123 3.46354 2.40513 3.72494 3.29354 2.67741 2.69355 4.24690 2.90347 2.73739 3.18146 2.89801 2.37887 2.77519 2.98518 4.58477 3.61503
0.01937 4.34956 5.07191 0.61958 0.77255 0.48576 0.95510
504 2.36591 5.04954 2.79103 2.00228 3.92635 3.44669 3.44476 3.80407 2.02963 3.21267 4.10280 2.87918 3.49284 2.75388 2.87893 2.64153 2.70665 3.10572 5.51043 4.12884 771 - -
2.68618 4.42225 2.77519 2.73123 3.46354 2.40513 3.72494 3.29354 2.67741 2.69355 4.24690 2.90347 2.73739 3.18146 2.89801 2.37887 2.77519 2.98518 4.58477 3.61503
0.01937 4.34956 5.07191 0.61958 0.77255 0.48576 0.95510
505 2.68386 4.91851 2.83032 2.47910 4.17272 3.21424 3.70080 3.60300 2.45620 3.19239 4.00113 1.67071 3.88382 2.84690 2.85591 2.63710 2.91944 3.15918 3.92932 4.07197 772 - -
2.68618 4.42225 2.77519 2.73123 3.46354 2.40513 3.72494 3.29354 2.67741 2.69355 4.24690 2.90347 2.73739 3.18146 2.89801 2.37887 2.77519 2.98518 4.58477 3.61503
0.03388 4.34956 3.89233 0.61958 0.77255 0.48576 0.95510
506 2.65053 5.00739 2.80879 2.20932 3.80454 3.16152 2.36995 3.56658 2.30725 3.17507 4.06402 2.88846 3.56333 2.69894 2.66608 2.64501 2.88165 3.36632 4.72214 4.10417 773 - -
2.68619 4.42226 2.77520 2.73118 3.46354 2.40513 3.72495 3.29355 2.67736 2.69355 4.24690 2.90347 2.73740 3.18147 2.89801 2.37887 2.77520 2.98519 4.58477 3.61504
0.03738 3.49556 5.05768 0.63405 0.75596 0.47311 0.97568
507 2.49656 4.87016 3.05920 2.44647 4.12688 3.48576 3.69436 3.38989 2.14516 3.14122 3.95302 2.94037 3.88138 2.84265 2.81527 2.42487 1.93317 3.10793 5.37800 4.04535 776 - -
2.68553 4.42232 2.77527 2.73125 3.46361 2.40520 3.72502 3.29361 2.67748 2.69362 4.24674 2.90354 2.73747 3.18154 2.89808 2.37878 2.77521 2.98526 4.58484 3.61510

0.22918 1.61675 5.07191 0.63114 0.75925 0.48576 0.95510
508 1.96073 4.42551 3.44902 2.88833 3.60609 3.29309 3.86587 2.96772 2.84435 2.67434 3.15788 3.18310 3.97838 3.16121 2.89110 2.68360 2.61745 2.08000 5.03035 3.78566 781 - -
2.68618 4.42225 2.77519 2.73123 3.46354 2.40513 3.72494 3.29354 2.67741 2.69355 4.24690 2.90347 2.73739 3.18146 2.89801 2.37887 2.77519 2.98518 4.58477 3.61503
0.01937 4.34956 5.07191 0.61958 0.77255 0.48576 0.95510
509 2.40242 4.79035 2.84994 2.63438 4.27874 3.38690 3.48972 3.70341 2.77107 3.32468 4.15686 3.11183 1.41466 3.09437 3.20909 2.63823 2.88159 3.33437 5.56768 4.24684 782 - -
2.68618 4.42225 2.77519 2.73123 3.46354 2.40513 3.72494 3.29354 2.67741 2.69355 4.24690 2.90347 2.73739 3.18146 2.89801 2.37887 2.77519 2.98518 4.58477 3.61503
0.01937 4.34956 5.07191 0.61958 0.77255 0.48576 0.95510
510 2.66090 5.46459 1.62963 1.68397 4.77715 3.41146 3.79148 4.28081 2.66217 3.75815 4.54139 2.29937 3.93410 2.92131 2.96669 2.82713 3.16315 3.84282 5.90156 4.44057 783 - -
2.68618 4.42225 2.77519 2.73123 3.46354 2.40513 3.72494 3.29354 2.67741 2.69355 4.24690 2.90347 2.73739 3.18146 2.89801 2.37887 2.77519 2.98518 4.58477 3.61503
0.01937 4.34956 5.07191 0.61958 0.77255 0.48576 0.95510
511 2.53612 4.43332 3.69655 3.38782 4.28385 3.27626 4.40979 3.47012 3.34026 3.27362 4.24148 3.57201 0.97571 3.69768 3.62658 2.72957 2.71934 2.72177 5.72161 4.48844 784 - -
2.68618 4.42225 2.77519 2.73123 3.46354 2.40513 3.72494 3.29354 2.67741 2.69355 4.24690 2.90347 2.73739 3.18146 2.89801 2.37887 2.77519 2.98518 4.58477 3.61503
0.01937 4.34956 5.07191 0.61958 0.77255 0.48576 0.95510
512 2.31465 4.55116 3.32441 2.97402 4.07238 3.35585 4.08671 3.52666 2.99475 3.21291 4.09132 3.33535 1.21420 3.34619 3.35220 2.64503 2.98436 3.17144 5.45941 3.57973 785 - -
2.68618 4.42225 2.77519 2.73123 3.46354 2.40513 3.72494 3.29354 2.67741 2.69355 4.24690 2.90347 2.73739 3.18146 2.89801 2.37887 2.77519 2.98518 4.58477 3.61503
0.01937 4.34956 5.07191 0.61958 0.77255 0.48576 0.95510
513 3.25823 0.33579 4.83918 4.70476 4.62742 3.75603 5.25237 3.88607 4.55011 3.76184 4.90648 4.57499 4.49442 4.87602 4.57954 3.52720 3.76552 3.58890 5.90388 4.90355 786 - -
2.68618 4.42225 2.77519 2.73123 3.46354 2.40513 3.72494 3.29354 2.67741 2.69355 4.24690 2.90347 2.73739 3.18146 2.89801 2.37887 2.77519 2.98518 4.58477 3.61503
0.01937 4.34956 5.07191 0.61958 0.77255 0.48576 0.95510
514 2.52069 4.46937 3.49660 2.93477 3.68462 3.06353 3.90250 2.77337 2.80811 2.72372 3.62252 3.34612 4.01241 3.18439 2.08890 2.86444 2.92471 1.82508 5.10320 3.86097 787 - -
2.68618 4.42225 2.77519 2.73123 3.46354 2.40513 3.72494 3.29354 2.67741 2.69355 4.24690 2.90347 2.73739 3.18146 2.89801 2.37887 2.77519 2.98518 4.58477 3.61503
0.01937 4.34956 5.07191 0.61958 0.77255 0.48576 0.95510
515 3.20906 5.30236 3.70281 2.97686 4.71722 3.85485 3.75794 4.06032 1.94039 3.06372 4.38234 3.37132 4.20099 2.55444 1.01016 3.21101 3.36460 3.75611 5.57344 4.40756 788 - -
2.68618 4.42225 2.77519 2.73123 3.46354 2.40513 3.72494 3.29354 2.67741 2.69355 4.24690 2.90347 2.73739 3.18146 2.89801 2.37887 2.77519 2.98518 4.58477 3.61503
0.01937 4.34956 5.07191 0.61958 0.77255 0.48576 0.95510
516 1.58698 4.76445 2.40262 2.60352 4.22625 3.39140 3.82197 3.63730 2.65318 3.25472 4.06726 3.08564 3.63664 2.98350 3.11015 2.33031 2.78878 2.91435 5.49561 4.16954 789 - -
2.68618 4.42225 2.77519 2.73123 3.46354 2.40513 3.72494 3.29354 2.67741 2.69355 4.24690 2.90347 2.73739 3.18146 2.89801 2.37887 2.77519 2.98518 4.58477 3.61503
0.01937 4.34956 5.07191 0.61958 0.77255 0.48576 0.95510
517 2.38853 4.25679 3.75237 3.06497 3.38172 3.68613 3.96129 2.65249 2.94841 2.39538 2.68363 3.51753 4.06184 3.38008 3.18504 2.94974 2.60041 1.71027 4.86137 3.64820 790 - -
2.68618 4.42225 2.77519 2.73123 3.46354 2.40513 3.72494 3.29354 2.67741 2.69355 4.24690 2.90347 2.73739 3.18146 2.89801 2.37887 2.77519 2.98518 4.58477 3.61503
0.01937 4.34956 5.07191 0.61958 0.77255 0.48576 0.95510
518 2.43336 4.37729 3.69414 3.44817 4.59176 3.14244 4.51116 3.99032 3.48820 3.71240 4.55153 3.55736 3.91428 3.79265 3.77367 0.78251 2.31115 3.41695 5.93881 4.72326 791 - -
2.68618 4.42225 2.77519 2.73123 3.46354 2.40513 3.72494 3.29354 2.67741 2.69355 4.24690 2.90347 2.73739 3.18146 2.89801 2.37887 2.77519 2.98518 4.58477 3.61503
0.01937 4.34956 5.07191 0.61958 0.77255 0.48576 0.95510
519 2.93482 4.37487 4.42542 3.85594 3.33282 4.07901 4.43480 2.01169 3.72595 2.03731 1.47574 4.08031 3.64709 3.94569 3.90720 3.39022 3.17560 2.00157 5.05165 3.89039 792 - -

2.68618 4.42225 2.77519 2.73123 3.46354 2.40513 3.72494 3.29354 2.67741 2.69355 4.24690 2.90347 2.73739 3.18146 2.89801 2.37887 2.77519 2.98518 4.58477 3.61503
0.01937 4.34956 5.07191 0.61958 0.77255 0.48576 0.95510
520 3.21636 5.71937 2.22285 0.74959 5.03944 3.44717 4.02975 4.57048 3.05115 4.08375 4.96541 2.93880 4.08522 3.20843 3.62392 3.10178 3.51589 4.15037 6.19651 4.72684 793 - -
2.68618 4.42225 2.77519 2.73123 3.46354 2.40513 3.72494 3.29354 2.67741 2.69355 4.24690 2.90347 2.73739 3.18146 2.89801 2.37887 2.77519 2.98518 4.58477 3.61503
0.01937 4.34956 5.07191 0.61958 0.77255 0.48576 0.95510
521 3.51242 5.16033 4.17329 4.09793 5.01100 3.82241 5.11050 4.71318 4.20288 4.27180 5.35836 4.34562 0.28188 4.57437 4.36978 3.70020 3.99437 4.29705 6.03519 5.14582 794 - -
2.68618 4.42225 2.77519 2.73123 3.46354 2.40513 3.72494 3.29354 2.67741 2.69355 4.24690 2.90347 2.73739 3.18146 2.89801 2.37887 2.77519 2.98518 4.58477 3.61503
0.01937 4.34956 5.07191 0.61958 0.77255 0.48576 0.95510
522 3.25823 0.33579 4.83918 4.70476 4.62742 3.75603 5.25237 3.88607 4.55011 3.76184 4.90648 4.57499 4.49442 4.87602 4.57954 3.52720 3.76552 3.58890 5.90388 4.90355 795 - -
2.68618 4.42225 2.77519 2.73123 3.46354 2.40513 3.72494 3.29354 2.67741 2.69355 4.24690 2.90347 2.73739 3.18146 2.89801 2.37887 2.77519 2.98518 4.58477 3.61503
0.01937 4.34956 5.07191 0.61958 0.77255 0.48576 0.95510
523 2.78370 4.36757 3.55123 3.28902 1.47316 3.78242 3.72098 2.85063 3.22116 2.26896 3.48305 3.33427 4.15854 3.48194 3.49838 2.88149 3.01607 2.64705 4.56570 2.93880 796 - -
2.68618 4.42225 2.77519 2.73123 3.46354 2.40513 3.72494 3.29354 2.67741 2.69355 4.24690 2.90347 2.73739 3.18146 2.89801 2.37887 2.77519 2.98518 4.58477 3.61503
0.01937 4.34956 5.07191 0.61958 0.77255 0.48576 0.95510
524 2.88762 4.72501 3.43736 2.94086 3.51653 3.71944 1.89473 3.22129 2.66800 1.73269 3.76821 3.36473 4.11803 2.84009 2.96789 3.02424 3.11473 3.01716 4.99030 3.59221 797 - -
2.68618 4.42225 2.77519 2.73123 3.46354 2.40513 3.72494 3.29354 2.67741 2.69355 4.24690 2.90347 2.73739 3.18146 2.89801 2.37887 2.77519 2.98518 4.58477 3.61503
0.01937 4.34956 5.07191 0.61958 0.77255 0.48576 0.95510
525 2.30729 4.96877 3.00229 2.44959 4.25846 3.19572 3.66794 3.69444 2.34788 3.25627 4.04494 2.88597 3.86102 2.60896 2.35017 2.38163 2.28636 3.22483 5.45805 4.10090 798 - -
2.68618 4.42225 2.77519 2.73123 3.46354 2.40513 3.72494 3.29354 2.67741 2.69355 4.24690 2.90347 2.73739 3.18146 2.89801 2.37887 2.77519 2.98518 4.58477 3.61503
0.01937 4.34956 5.07191 0.61958 0.77255 0.48576 0.95510
526 1.63909 4.42583 3.58798 3.18872 4.24617 3.26220 4.24152 3.54025 3.16770 3.07454 4.16252 3.44660 1.43722 3.49747 3.50775 2.67078 2.70265 3.14295 5.62208 4.38780 799 - -
2.68618 4.42225 2.77519 2.73123 3.46354 2.40513 3.72494 3.29354 2.67741 2.69355 4.24690 2.90347 2.73739 3.18146 2.89801 2.37887 2.77519 2.98518 4.58477 3.61503
0.01937 4.34956 5.07191 0.61958 0.77255 0.48576 0.95510
527 2.63983 4.56000 3.48243 3.22261 4.20672 3.33469 4.29774 3.66301 3.18533 3.00318 4.31758 3.49507 0.93550 3.58943 3.47868 2.63609 3.07756 3.28393 5.62190 4.30724 800 - -
2.68618 4.42225 2.77519 2.73123 3.46354 2.40513 3.72494 3.29354 2.67741 2.69355 4.24690 2.90347 2.73739 3.18146 2.89801 2.37887 2.77519 2.98518 4.58477 3.61503
0.01937 4.34956 5.07191 0.61958 0.77255 0.48576 0.95510
528 2.64348 4.22713 3.78850 3.21353 2.50347 3.68556 3.71161 2.61630 3.13580 1.98807 3.33727 3.36530 3.50361 3.40474 3.11237 2.68028 2.81451 2.45426 4.81853 2.71414 801 - -
2.68618 4.42225 2.77519 2.73123 3.46354 2.40513 3.72494 3.29354 2.67741 2.69355 4.24690 2.90347 2.73739 3.18146 2.89801 2.37887 2.77519 2.98518 4.58477 3.61503
0.01937 4.34956 5.07191 0.61958 0.77255 0.48576 0.95510
529 2.57927 4.50044 3.42583 2.86698 3.28522 3.62510 3.06501 3.02681 2.81664 2.56179 3.60092 2.80472 4.00673 3.14199 3.07780 2.86355 2.92401 2.68491 4.91054 1.92519 802 - -
2.68618 4.42225 2.77519 2.73123 3.46354 2.40513 3.72494 3.29354 2.67741 2.69355 4.24690 2.90347 2.73739 3.18146 2.89801 2.37887 2.77519 2.98518 4.58477 3.61503
0.01937 4.34956 5.07191 0.61958 0.77255 0.48576 0.95510
530 2.60342 5.14242 2.06693 2.25711 4.45868 2.22274 3.68790 3.92336 2.47388 3.44467 3.92254 2.55859 3.86654 2.80637 2.97061 2.61077 2.95954 3.36746 5.61352 4.21501 803 - -
2.68606 4.42227 2.77522 2.73112 3.46356 2.40515 3.72497 3.29356 2.67743 2.69357 4.24692 2.90349 2.73742 3.18149 2.89803 2.37889 2.77513 2.98521 4.58479 3.61505
0.06659 2.84474 5.07191 0.73631 0.65177 0.48576 0.95510

531 3.25823 0.33579 4.83918 4.70476 4.62742 3.75603 5.25237 3.88607 4.55011 3.76184 4.90648 4.57499 4.49442 4.87602 4.57954 3.52720 3.76552 3.58890 5.90388 4.90355 807 - -
2.68618 4.42225 2.77519 2.73123 3.46354 2.40513 3.72494 3.29354 2.67741 2.69355 4.24690 2.90347 2.73739 3.18146 2.89801 2.37887 2.77519 2.98518 4.58477 3.61503
0.01937 4.34956 5.07191 0.61958 0.77255 0.48576 0.95510
532 2.72065 5.21756 3.08220 2.31030 4.58766 3.37770 3.66511 4.02445 1.85268 3.49277 4.26953 3.02739 3.93587 2.07838 1.95838 2.78538 2.91143 3.62551 5.59947 4.26644 808 - -
2.68618 4.42225 2.77519 2.73123 3.46354 2.40513 3.72494 3.29354 2.67741 2.69355 4.24690 2.90347 2.73739 3.18146 2.89801 2.37887 2.77519 2.98518 4.58477 3.61503
0.03388 4.34956 3.89233 0.61958 0.77255 0.48576 0.95510
533 1.72864 4.67305 3.01456 2.53373 3.87666 3.29414 3.74416 3.26637 2.53370 2.84485 3.11238 3.10645 3.64327 2.94042 3.03715 2.73589 2.71862 2.89523 5.21667 3.92090 809 - -
2.68618 4.42225 2.77519 2.73123 3.46354 2.40513 3.72494 3.29354 2.67741 2.69355 4.24690 2.90347 2.73739 3.18146 2.89801 2.37887 2.77519 2.98518 4.58477 3.61503
0.34891 4.31159 1.26894 0.61958 0.77255 0.50634 0.92306
534 2.61735 4.92868 2.97362 2.49629 4.23954 3.46825 3.59403 3.63567 1.62976 3.18805 3.62345 2.98911 3.85661 2.73122 2.22284 2.72179 2.92496 3.30207 5.36454 4.06987 810 - -
2.68618 4.42225 2.77519 2.73123 3.46354 2.40513 3.72494 3.29354 2.67741 2.69355 4.24690 2.90347 2.73739 3.18146 2.89801 2.37887 2.77519 2.98518 4.58477 3.61503
0.06485 3.99054 3.11662 0.61958 0.77255 0.33922 1.24594
535 2.52325 5.14213 2.79951 2.35048 4.47839 3.48729 3.66852 3.91656 1.40675 3.42465 4.21072 2.97591 3.89935 2.78795 2.55986 2.74622 2.88142 3.53206 5.57012 4.21989 811 - -
2.68619 4.42226 2.77515 2.73118 3.46355 2.40514 3.72495 3.29345 2.67742 2.69356 4.24691 2.90348 2.73741 3.18147 2.89802 2.37888 2.77520 2.98519 4.58478 3.61504
0.34490 3.43795 1.34868 0.73166 0.65607 0.50805 0.92046
536 2.57989 4.76512 3.19420 2.59772 4.01275 3.50495 3.63254 3.40603 2.09658 3.00909 3.85513 2.92872 3.88531 2.80221 1.86544 2.75710 2.81268 3.11334 3.78907 3.96243 815 - -
2.68618 4.42225 2.77519 2.73123 3.46354 2.40513 3.72494 3.29354 2.67741 2.69355 4.24690 2.90347 2.73739 3.18146 2.89801 2.37887 2.77519 2.98518 4.58477 3.61503
0.02720 4.01404 4.73638 0.61958 0.77255 0.55042 0.85970
537 2.60683 4.80234 2.82016 2.39295 4.03913 2.53449 3.64275 3.30493 2.33527 2.91484 3.87392 2.74604 3.82823 2.79415 2.90619 2.38098 2.83826 3.00226 4.69264 3.73439 816 - -
2.68618 4.42225 2.77519 2.73123 3.46354 2.40513 3.72494 3.29354 2.67741 2.69355 4.24690 2.90347 2.73739 3.18146 2.89801 2.37887 2.77519 2.98518 4.58477 3.61503
0.02311 4.17501 4.89735 0.61958 0.77255 0.66945 0.71742
538 2.00125 4.55326 3.06078 2.57364 3.76021 3.03793 3.73322 2.87385 2.61837 2.81118 3.66849 3.11037 3.87814 2.95197 2.94315 2.63639 2.55785 2.59855 5.12576 3.84490 817 - -
2.68618 4.42225 2.77519 2.73123 3.46354 2.40513 3.72494 3.29354 2.67741 2.69355 4.24690 2.90347 2.73739 3.18146 2.89801 2.37887 2.77519 2.98518 4.58477 3.61503
0.02311 4.17501 4.89735 0.61958 0.77255 0.66945 0.71742
539 2.79437 5.24105 1.53136 2.22480 4.51703 3.35962 2.87289 4.00783 2.56389 3.52969 4.32741 2.56492 3.86147 2.84874 3.07911 2.74198 3.04655 3.26772 5.69428 4.26737 818 - -
2.68618 4.42225 2.77519 2.73123 3.46354 2.40513 3.72494 3.29354 2.67741 2.69355 4.24690 2.90347 2.73739 3.18146 2.89801 2.37887 2.77519 2.98518 4.58477 3.61503
0.02311 4.17501 4.89735 0.61958 0.77255 0.66945 0.71742
540 2.46124 4.51904 3.22766 2.67498 3.72659 3.48339 3.75004 2.88588 2.55146 2.49240 3.64316 3.01866 3.66851 2.98089 3.05349 2.19040 2.26747 2.83530 5.10251 3.82983 819 - -
2.68618 4.42225 2.77519 2.73123 3.46354 2.40513 3.72494 3.29354 2.67741 2.69355 4.24690 2.90347 2.73739 3.18146 2.89801 2.37887 2.77519 2.98518 4.58477 3.61503
0.02311 4.17501 4.89735 0.61958 0.77255 0.38597 1.13879
541 2.39323 4.57073 3.21746 2.52718 4.02208 1.61520 3.97508 3.31137 2.88651 3.06179 3.93470 3.24585 3.94893 3.20876 3.28482 2.76086 2.94870 2.19115 5.39428 4.12285 820 - -
2.68618 4.42225 2.77519 2.73123 3.46354 2.40513 3.72494 3.29354 2.67741 2.69355 4.24690 2.90347 2.73739 3.18146 2.89801 2.37887 2.77519 2.98518 4.58477 3.61503
0.01983 4.32616 5.04851 0.61958 0.77255 0.48576 0.95510
542 2.49982 5.13425 2.72515 2.40078 4.47634 3.44250 3.69211 3.93202 1.69351 3.45079 4.22748 2.11798 3.87868 2.81208 2.89739 2.71022 2.69441 3.53245 5.61162 4.22887 821 - -
2.68618 4.42225 2.77519 2.73123 3.46354 2.40513 3.72494 3.29354 2.67741 2.69355 4.24690 2.90347 2.73739 3.18146 2.89801 2.37887 2.77519 2.98518 4.58477 3.61503

0.01983 4.32616 5.04851 0.61958 0.77255 0.48576 0.95510
543 2.77850 4.17611 4.48559 3.89498 3.08395 3.95168 3.92646 1.69957 3.74221 1.95576 2.76094 4.01896 4.29793 3.91988 3.84935 3.25815 2.88715 1.63693 4.83922 3.66362 822 - -
2.68618 4.42225 2.77519 2.73123 3.46354 2.40513 3.72494 3.29354 2.67741 2.69355 4.24690 2.90347 2.73739 3.18146 2.89801 2.37887 2.77519 2.98518 4.58477 3.61503
0.01983 4.32616 5.04851 0.61958 0.77255 0.48576 0.95510
544 2.62434 4.30228 3.97755 3.42556 3.50016 3.79981 4.18476 2.41365 3.14101 2.16915 3.45036 3.72368 4.20442 3.61343 3.58146 3.10103 2.16837 1.44868 5.04366 3.83422 823 - -
2.68618 4.42225 2.77519 2.73123 3.46354 2.40513 3.72494 3.29354 2.67741 2.69355 4.24690 2.90347 2.73739 3.18146 2.89801 2.37887 2.77519 2.98518 4.58477 3.61503
0.01983 4.32616 5.04851 0.61958 0.77255 0.48576 0.95510
545 2.24886 4.70824 3.29714 2.84830 4.32316 3.40228 3.95176 3.71799 2.64791 3.33783 4.18440 3.25338 1.46311 3.13697 2.30112 2.76852 3.00657 3.33680 5.56427 4.29724 824 - -
2.68618 4.42225 2.77519 2.73123 3.46354 2.40513 3.72494 3.29354 2.67741 2.69355 4.24690 2.90347 2.73739 3.18146 2.89801 2.37887 2.77519 2.98518 4.58477 3.61503
0.01983 4.32616 5.04851 0.61958 0.77255 0.48576 0.95510
546 3.74497 5.07431 4.57874 4.20416 1.23859 4.48929 1.46775 3.66122 4.06948 3.02999 4.22065 4.12059 4.80717 4.14006 4.19768 3.82526 3.96188 3.52935 3.81157 1.83249 825 - -
2.68618 4.42225 2.77519 2.73123 3.46354 2.40513 3.72494 3.29354 2.67741 2.69355 4.24690 2.90347 2.73739 3.18146 2.89801 2.37887 2.77519 2.98518 4.58477 3.61503
0.01983 4.32616 5.04851 0.61958 0.77255 0.48576 0.95510
547 3.30165 4.57299 5.13851 4.64957 3.78283 4.73496 5.35347 1.63843 4.53762 2.18718 3.57223 4.84476 5.01934 4.80171 4.72166 4.13827 3.56673 0.76189 5.79883 4.58269 826 - -
2.68618 4.42225 2.77519 2.73123 3.46354 2.40513 3.72494 3.29354 2.67741 2.69355 4.24690 2.90347 2.73739 3.18146 2.89801 2.37887 2.77519 2.98518 4.58477 3.61503
0.01983 4.32616 5.04851 0.61958 0.77255 0.48576 0.95510
548 2.92897 5.01908 3.43697 2.78657 4.32478 3.69021 3.73444 3.68440 1.83300 3.24037 2.60563 3.23339 4.05582 2.88601 1.52220 2.96818 2.98304 3.38927 5.42936 4.19498 827 - -
2.68618 4.42225 2.77519 2.73123 3.46354 2.40513 3.72494 3.29354 2.67741 2.69355 4.24690 2.90347 2.73739 3.18146 2.89801 2.37887 2.77519 2.98518 4.58477 3.61503
0.01983 4.32616 5.04851 0.61958 0.77255 0.48576 0.95510
549 2.69164 4.75670 3.16566 2.61166 3.83535 3.53794 2.19499 3.36738 2.53977 2.89754 3.84160 2.69398 3.92665 2.92236 2.72880 2.67808 2.92100 2.94593 5.18360 2.67558 828 - -
2.68618 4.42225 2.77519 2.73123 3.46354 2.40513 3.72494 3.29354 2.67741 2.69355 4.24690 2.90347 2.73739 3.18146 2.89801 2.37887 2.77519 2.98518 4.58477 3.61503
0.01983 4.32616 5.04851 0.61958 0.77255 0.48576 0.95510
550 2.87520 0.76849 4.37475 4.02169 3.57454 3.63932 4.50880 3.10648 3.78994 2.91293 4.01606 4.04674 4.28650 4.13652 3.94336 3.15484 3.28870 2.88900 5.14954 3.31359 829 - -
2.68618 4.42225 2.77519 2.73123 3.46354 2.40513 3.72494 3.29354 2.67741 2.69355 4.24690 2.90347 2.73739 3.18146 2.89801 2.37887 2.77519 2.98518 4.58477 3.61503
0.01983 4.32616 5.04851 0.61958 0.77255 0.48576 0.95510
551 2.87018 5.33299 2.43217 1.23097 4.59782 3.42779 2.81814 4.10548 2.60051 3.38647 4.41269 2.89764 3.92800 2.90457 3.10196 2.81263 3.11984 3.70382 5.76156 4.33126 830 - -
2.68618 4.42225 2.77519 2.73123 3.46354 2.40513 3.72494 3.29354 2.67741 2.69355 4.24690 2.90347 2.73739 3.18146 2.89801 2.37887 2.77519 2.98518 4.58477 3.61503
0.01983 4.32616 5.04851 0.61958 0.77255 0.48576 0.95510
552 3.16775 5.56546 0.70949 2.37787 4.98864 3.38767 4.10309 4.58903 3.23106 4.14872 5.06459 2.95081 4.07696 3.30757 3.84537 2.86415 3.52278 4.13802 6.23413 4.73057 831 - -
2.68618 4.42225 2.77519 2.73123 3.46354 2.40513 3.72494 3.29354 2.67741 2.69355 4.24690 2.90347 2.73739 3.18146 2.89801 2.37887 2.77519 2.98518 4.58477 3.61503
0.01983 4.32616 5.04851 0.61958 0.77255 0.48576 0.95510
553 2.69415 4.17914 4.09934 3.51538 2.94901 3.79528 4.09161 2.35846 3.39948 2.04972 2.91971 3.75042 4.15693 3.63018 2.53300 3.07985 2.92567 1.70187 4.78918 3.40642 832 - -
2.68618 4.42225 2.77519 2.73123 3.46354 2.40513 3.72494 3.29354 2.67741 2.69355 4.24690 2.90347 2.73739 3.18146 2.89801 2.37887 2.77519 2.98518 4.58477 3.61503
0.01983 4.32616 5.04851 0.61958 0.77255 0.48576 0.95510
554 2.54940 4.77109 2.97003 2.47546 3.98964 3.13328 3.70223 2.57204 2.46532 3.02054 3.71666 3.04455 2.90728 2.73042 2.84281 2.28050 2.87620 3.09187 4.80229 3.97020 833 - -

2.68618 4.42225 2.77519 2.73123 3.46354 2.40513 3.72494 3.29354 2.67741 2.69355 4.24690 2.90347 2.73739 3.18146 2.89801 2.37887 2.77519 2.98518 4.58477 3.61503
0.03135 4.32616 4.03750 0.61958 0.77255 0.48576 0.95510
555 2.52613 4.64560 2.95505 2.62263 3.62148 3.29955 3.63198 3.22467 2.48485 2.88179 3.73164 2.95079 3.90730 2.94280 2.91692 2.73749 2.33126 2.95007 2.62875 3.61494 834 - -
2.68614 4.42226 2.77517 2.73124 3.46355 2.40509 3.72496 3.29355 2.67742 2.69356 4.24691 2.90348 2.73741 3.18147 2.89802 2.37888 2.77521 2.98519 4.58478 3.61497
0.08185 2.62973 5.03722 0.39211 1.12586 0.47588 0.97110
556 2.68625 4.56987 3.67761 3.49831 4.42764 0.66599 4.56300 3.35606 3.55888 3.60662 4.57190 3.68311 4.07721 3.90023 3.82113 2.87050 3.17257 3.41917 5.80095 4.56200 836 - -
2.68618 4.42225 2.77519 2.73123 3.46354 2.40513 3.72494 3.29354 2.67741 2.69355 4.24690 2.90347 2.73739 3.18146 2.89801 2.37887 2.77519 2.98518 4.58477 3.61503
0.01983 4.32616 5.04851 0.61958 0.77255 0.48576 0.95510
557 3.13489 4.51255 4.72464 4.14530 3.04522 4.33298 4.63823 2.07210 4.00357 0.94657 3.08378 4.35284 4.60440 4.15798 3.71307 3.65149 3.36145 2.08846 5.09815 3.97096 837 - -
2.68618 4.42225 2.77519 2.73123 3.46354 2.40513 3.72494 3.29354 2.67741 2.69355 4.24690 2.90347 2.73739 3.18146 2.89801 2.37887 2.77519 2.98518 4.58477 3.61503
0.01983 4.32616 5.04851 0.61958 0.77255 0.48576 0.95510
558 2.88354 5.30828 2.71039 2.44197 4.66112 3.50314 3.72509 4.11448 1.36201 3.58964 4.38161 2.26033 3.95331 2.53964 2.76307 2.83620 3.11250 3.71313 5.70604 4.34382 838 - -
2.68618 4.42225 2.77519 2.73123 3.46354 2.40513 3.72494 3.29354 2.67741 2.69355 4.24690 2.90347 2.73739 3.18146 2.89801 2.37887 2.77519 2.98518 4.58477 3.61503
0.01983 4.32616 5.04851 0.61958 0.77255 0.48576 0.95510
559 2.81809 4.46928 2.61170 3.19867 3.20613 3.82998 4.09205 2.62808 3.15311 1.36611 3.18754 3.59342 4.18973 3.43861 3.48691 3.09210 3.05083 2.32419 5.04203 3.81878 839 - -
2.68618 4.42225 2.77519 2.73123 3.46354 2.40513 3.72494 3.29354 2.67741 2.69355 4.24690 2.90347 2.73739 3.18146 2.89801 2.37887 2.77519 2.98518 4.58477 3.61503
0.01983 4.32616 5.04851 0.61958 0.77255 0.48576 0.95510
560 2.58089 4.15004 4.08496 3.50498 2.52233 3.75798 4.04937 2.54857 3.39029 2.17244 3.25694 3.72497 4.12982 3.61712 3.58393 2.37706 2.89857 1.71254 4.73194 3.11550 840 - -
2.68618 4.42225 2.77519 2.73123 3.46354 2.40513 3.72494 3.29354 2.67741 2.69355 4.24690 2.90347 2.73739 3.18146 2.89801 2.37887 2.77519 2.98518 4.58477 3.61503
0.02013 4.31164 5.03399 0.61958 0.77255 0.48576 0.95510
561 2.53548 5.25013 1.86172 1.94219 4.57391 3.41431 3.48002 4.05798 2.46654 3.54571 4.30629 2.69147 3.86276 2.79459 2.50784 2.62244 2.98906 3.63129 5.68847 4.26763 841 - -
2.68618 4.42225 2.77519 2.73123 3.46354 2.40513 3.72494 3.29354 2.67741 2.69355 4.24690 2.90347 2.73739 3.18146 2.89801 2.37887 2.77519 2.98518 4.58477 3.61503
0.04330 4.16883 3.61546 0.61958 0.77255 0.48576 0.95510
562 2.45978 5.06017 2.73986 2.23088 4.37578 3.24327 3.59755 3.83811 2.34397 3.35186 4.11576 2.79148 3.26979 2.23293 2.39804 2.50604 2.86641 3.43636 5.51178 4.12266 842 - -
2.68618 4.42225 2.77519 2.73123 3.46354 2.40513 3.72494 3.29354 2.67741 2.69355 4.24690 2.90347 2.73739 3.18146 2.89801 2.37887 2.77519 2.98518 4.58477 3.61503
0.02423 4.12788 4.85022 0.61958 0.77255 0.50930 0.91858
563 2.59421 4.64112 3.09973 2.48018 3.41178 3.33037 3.67559 3.10733 2.36408 2.77462 3.72451 2.89159 3.84887 2.33259 2.95561 2.60201 2.71055 2.59679 5.17183 3.87216 843 - -
2.68618 4.42225 2.77519 2.73123 3.46354 2.40513 3.72494 3.29354 2.67741 2.69355 4.24690 2.90347 2.73739 3.18146 2.89801 2.37887 2.77519 2.98518 4.58477 3.61503
0.02757 4.00074 4.72309 0.61958 0.77255 0.50930 0.91858
564 2.67966 4.91790 2.58614 1.94702 4.34198 3.06303 3.72577 3.77857 2.49965 3.35962 4.18773 2.86729 3.81329 2.89033 3.01477 1.71275 2.97006 3.39993 5.56678 4.20445 844 - -
2.68618 4.42225 2.77519 2.73123 3.46354 2.40513 3.72494 3.29354 2.67741 2.69355 4.24690 2.90347 2.73739 3.18146 2.89801 2.37887 2.77519 2.98518 4.58477 3.61503
0.14135 3.97602 2.17988 0.61958 0.77255 0.47132 0.97864
565 2.61081 4.83703 2.83572 1.93785 4.08947 3.36642 3.58941 3.51907 2.34030 3.10308 3.92065 2.82749 3.78484 2.42840 2.76734 2.54365 2.74086 3.18746 4.56385 3.97968 845 - -
2.68618 4.42225 2.77519 2.73123 3.46354 2.40513 3.72494 3.29354 2.67741 2.69355 4.24690 2.90347 2.73739 3.18146 2.89801 2.37887 2.77519 2.98518 4.58477 3.61503
0.03149 3.86960 4.59194 0.61958 0.77255 0.58585 0.81335

566 2.61165 4.73991 2.97925 2.41885 4.00431 3.39867 3.59994 3.39909 1.87970 3.00782 3.24214 2.83755 3.54521 2.76350 2.67989 2.56352 2.84284 3.09035 5.24443 3.94428 846 - -
2.68618 4.42225 2.77519 2.73123 3.46354 2.40513 3.72494 3.29354 2.67741 2.69355 4.24690 2.90347 2.73739 3.18146 2.89801 2.37887 2.77519 2.98518 4.58477 3.61503
0.03149 3.86960 4.59194 0.61958 0.77255 0.58585 0.81335
567 2.44387 4.41408 3.10853 2.68033 3.66417 2.88543 3.74914 3.02977 2.62157 2.01971 3.62304 2.94624 3.83453 2.85453 2.97416 2.68896 2.81482 2.76779 5.05862 3.78912 847 - -
2.68618 4.42225 2.77519 2.73123 3.46354 2.40513 3.72494 3.29354 2.67741 2.69355 4.24690 2.90347 2.73739 3.18146 2.89801 2.37887 2.77519 2.98518 4.58477 3.61503
0.03286 3.82770 4.55005 0.61958 0.77255 0.58585 0.81335
568 2.83793 4.32932 4.09831 3.54005 2.96563 3.91070 4.11841 2.30097 3.36703 1.40752 3.04370 3.81397 4.24482 3.64392 3.58579 3.21997 3.07530 1.97303 3.96488 3.39982 848 - -
2.68618 4.42225 2.77519 2.73123 3.46354 2.40513 3.72494 3.29354 2.67741 2.69355 4.24690 2.90347 2.73739 3.18146 2.89801 2.37887 2.77519 2.98518 4.58477 3.61503
0.03385 3.79865 4.52100 0.61958 0.77255 0.58585 0.81335
569 2.49057 4.28139 3.37787 2.89737 3.29365 2.17193 3.77363 2.93776 2.78542 2.61562 3.55494 3.24233 3.84063 3.15164 3.10319 2.68205 2.79647 2.55181 3.36135 3.40306 849 - -
2.68618 4.42225 2.77519 2.73123 3.46354 2.40513 3.72494 3.29354 2.67741 2.69355 4.24690 2.90347 2.73739 3.18146 2.89801 2.37887 2.77519 2.98518 4.58477 3.61503
0.03484 3.77017 4.49252 0.61958 0.77255 0.58585 0.81335
570 2.61893 4.69872 2.51706 2.00746 4.25466 1.78624 3.78210 3.68739 2.67049 3.32575 4.21028 2.87041 3.74101 2.99430 3.07910 2.66513 2.96555 3.30927 5.48718 4.19320 850 - -
2.68606 4.42227 2.77521 2.73111 3.46356 2.40514 3.72496 3.29356 2.67742 2.69357 4.24691 2.90348 2.73741 3.18148 2.89802 2.37888 2.77521 2.98520 4.58479 3.61505
0.11827 2.29906 4.49252 0.64784 0.74060 0.58585 0.81335
571 3.04313 4.53765 4.02310 3.66600 3.10228 3.84857 4.35390 2.30515 3.43459 1.01279 3.16360 3.95091 4.30396 3.82765 3.62603 3.41241 3.34344 2.31521 4.94095 3.63959 853 - -
2.68618 4.42225 2.77519 2.73123 3.46354 2.40513 3.72494 3.29354 2.67741 2.69355 4.24690 2.90347 2.73739 3.18146 2.89801 2.37887 2.77519 2.98518 4.58477 3.61503
0.03484 3.77017 4.49252 0.61958 0.77255 0.58585 0.81335
572 2.94488 4.43525 4.19611 3.71780 3.15850 4.00147 4.45024 2.04771 3.52453 1.28618 3.07404 4.00278 4.38539 3.85225 3.74899 3.38683 3.22545 1.79805 5.07585 3.82704 854 - -
2.68618 4.42225 2.77519 2.73123 3.46354 2.40513 3.72494 3.29354 2.67741 2.69355 4.24690 2.90347 2.73739 3.18146 2.89801 2.37887 2.77519 2.98518 4.58477 3.61503
0.03484 3.77017 4.49252 0.61958 0.77255 0.58585 0.81335
573 1.39786 4.08892 3.21129 2.98542 4.11490 2.28688 4.09177 3.41501 3.07220 3.19965 4.08344 3.18000 3.60549 3.38008 3.37157 2.34939 2.64869 2.95198 5.47259 4.26125 855 - -
2.68618 4.42225 2.77519 2.73123 3.46354 2.40513 3.72494 3.29354 2.67741 2.69355 4.24690 2.90347 2.73739 3.18146 2.89801 2.37887 2.77519 2.98518 4.58477 3.61503
0.03484 3.77017 4.49252 0.61958 0.77255 0.58585 0.81335
574 2.52840 4.49263 3.00903 2.64913 3.97135 3.23427 3.75668 3.22475 2.15413 2.96614 3.88548 3.05917 3.78696 2.97020 2.76150 2.64498 1.91351 2.92301 5.27969 4.01473 856 - -
2.68618 4.42225 2.77519 2.73123 3.46354 2.40513 3.72494 3.29354 2.67741 2.69355 4.24690 2.90347 2.73739 3.18146 2.89801 2.37887 2.77519 2.98518 4.58477 3.61503
0.03484 3.77017 4.49252 0.61958 0.77255 0.58585 0.81335
575 2.54697 4.32873 3.19933 3.08768 4.22468 0.90617 4.21120 3.80211 3.25454 3.46863 4.44617 3.36448 3.71657 3.58745 3.49887 2.71655 3.02040 3.35061 5.34389 4.32424 857 - -
2.68618 4.42225 2.77519 2.73123 3.46354 2.40513 3.72494 3.29354 2.67741 2.69355 4.24690 2.90347 2.73739 3.18146 2.89801 2.37887 2.77519 2.98518 4.58477 3.61503
0.03484 3.77017 4.49252 0.61958 0.77255 0.58585 0.81335
576 2.61551 4.29589 3.59344 3.20783 1.82531 3.45187 3.78054 2.77061 3.15528 2.41513 3.51010 3.44731 3.97824 3.44143 3.41951 2.39347 2.94397 2.58774 4.39309 2.84189 858 - -
2.68618 4.42225 2.77519 2.73123 3.46354 2.40513 3.72494 3.29354 2.67741 2.69355 4.24690 2.90347 2.73739 3.18146 2.89801 2.37887 2.77519 2.98518 4.58477 3.61503
0.03484 3.77017 4.49252 0.61958 0.77255 0.58585 0.81335
577 2.87006 4.95084 2.45507 1.18098 4.28523 3.21208 3.79453 3.71882 2.66249 3.35417 4.33630 2.88488 3.81288 3.01470 3.05788 2.86528 3.17475 3.41464 5.47656 4.20262 859 - -
2.68618 4.42225 2.77519 2.73123 3.46354 2.40513 3.72494 3.29354 2.67741 2.69355 4.24690 2.90347 2.73739 3.18146 2.89801 2.37887 2.77519 2.98518 4.58477 3.61503

0.03484 3.77017 4.49252 0.61958 0.77255 0.58585 0.81335
578 2.67468 4.66018 2.85210 1.84767 3.81878 3.38993 3.73864 2.54473 2.54506 2.74689 3.75781 3.01482 3.86646 2.94820 2.92536 2.76748 2.93594 2.73006 5.23756 3.91669 860 - -
2.68618 4.42225 2.77519 2.73123 3.46354 2.40513 3.72494 3.29354 2.67741 2.69355 4.24690 2.90347 2.73739 3.18146 2.89801 2.37887 2.77519 2.98518 4.58477 3.61503
0.03484 3.77017 4.49252 0.61958 0.77255 0.58585 0.81335
579 2.67381 4.74361 2.83581 2.51634 4.23445 2.60815 3.67977 3.64857 1.57469 3.24318 4.11754 2.97402 3.79793 2.86186 2.58578 2.72193 2.96680 3.29710 5.37699 4.12114 861 - -
2.68618 4.42225 2.77519 2.73123 3.46354 2.40513 3.72494 3.29354 2.67741 2.69355 4.24690 2.90347 2.73739 3.18146 2.89801 2.37887 2.77519 2.98518 4.58477 3.61503
0.03484 3.77017 4.49252 0.61958 0.77255 0.58585 0.81335
580 2.40733 4.27848 3.24788 2.88970 3.61481 3.16671 3.89563 2.98596 2.80237 2.32943 3.74702 3.20592 3.77747 3.20404 3.11071 1.74880 2.78705 2.71178 5.09714 3.75277 862 - -
2.68618 4.42225 2.77519 2.73123 3.46354 2.40513 3.72494 3.29354 2.67741 2.69355 4.24690 2.90347 2.73739 3.18146 2.89801 2.37887 2.77519 2.98518 4.58477 3.61503
0.03484 3.77017 4.49252 0.61958 0.77255 0.58585 0.81335
581 1.88101 1.94615 3.85878 3.49166 3.73865 2.99601 4.25561 2.73045 3.34865 2.71977 3.72810 3.48229 3.71582 3.65744 3.55279 2.45698 2.66453 2.43540 5.25417 4.05347 863 - -
2.68618 4.42225 2.77519 2.73123 3.46354 2.40513 3.72494 3.29354 2.67741 2.69355 4.24690 2.90347 2.73739 3.18146 2.89801 2.37887 2.77519 2.98518 4.58477 3.61503
0.03484 3.77017 4.49252 0.61958 0.77255 0.58585 0.81335
582 3.05924 4.48548 4.34440 3.83868 3.09148 4.18988 4.52521 1.79720 3.63721 1.21365 2.98786 4.13861 4.49431 3.93767 3.84715 3.55953 3.31132 1.99997 5.06037 3.82862 864 - -
2.68618 4.42225 2.77519 2.73123 3.46354 2.40513 3.72494 3.29354 2.67741 2.69355 4.24690 2.90347 2.73739 3.18146 2.89801 2.37887 2.77519 2.98518 4.58477 3.61503
0.03484 3.77017 4.49252 0.61958 0.77255 0.58585 0.81335
583 2.18813 4.12440 3.18845 2.92084 4.05717 2.90063 4.01934 3.38659 2.94257 3.14996 4.03454 3.14827 3.62004 3.29028 3.25068 1.55485 2.20892 2.94213 5.43018 4.17681 865 - -
2.68618 4.42225 2.77519 2.73123 3.46354 2.40513 3.72494 3.29354 2.67741 2.69355 4.24690 2.90347 2.73739 3.18146 2.89801 2.37887 2.77519 2.98518 4.58477 3.61503
0.03484 3.77017 4.49252 0.61958 0.77255 0.58585 0.81335
584 2.77205 4.65813 3.38880 2.82550 3.71543 3.50893 3.68504 3.12799 2.20753 2.32420 3.74927 3.21474 3.93735 2.91012 1.72078 2.88658 3.01230 2.93048 5.07310 3.78236 866 - -
2.68618 4.42225 2.77519 2.73123 3.46354 2.40513 3.72494 3.29354 2.67741 2.69355 4.24690 2.90347 2.73739 3.18146 2.89801 2.37887 2.77519 2.98518 4.58477 3.61503
0.03484 3.77017 4.49252 0.61958 0.77255 0.58585 0.81335
585 2.97815 4.52789 3.81217 3.37506 2.38097 3.83112 3.62437 2.76701 3.17754 1.97863 3.47826 3.60114 4.21834 3.50596 3.40805 3.18888 3.22585 2.66897 4.04501 1.63674 867 - -
2.68618 4.42225 2.77519 2.73123 3.46354 2.40513 3.72494 3.29354 2.67741 2.69355 4.24690 2.90347 2.73739 3.18146 2.89801 2.37887 2.77519 2.98518 4.58477 3.61503
0.11827 3.77017 2.42478 0.61958 0.77255 0.58585 0.81335
586 2.80535 4.87482 1.22337 2.25740 4.23128 3.08281 3.79410 3.78082 2.84781 3.42775 4.41212 2.79951 3.72433 3.04114 3.32941 2.79671 3.14408 3.44355 5.43550 4.14798 868 - -
2.68618 4.42225 2.77519 2.73123 3.46354 2.40513 3.72494 3.29354 2.67741 2.69355 4.24690 2.90347 2.73739 3.18146 2.89801 2.37887 2.77519 2.98518 4.58477 3.61503
0.03782 3.68971 4.41206 0.61958 0.77255 0.64191 0.74715
587 2.75145 4.71252 2.80109 2.56401 3.85261 3.25189 3.71665 3.46461 2.40147 2.98463 4.02911 3.01766 3.80997 1.60421 2.68491 2.81243 3.05413 3.20938 5.16412 3.84799 869 - -
2.68618 4.42225 2.77519 2.73123 3.46354 2.40513 3.72494 3.29354 2.67741 2.69355 4.24690 2.90347 2.73739 3.18146 2.89801 2.37887 2.77519 2.98518 4.58477 3.61503
0.03782 3.68971 4.41206 0.61958 0.77255 0.64191 0.74715
588 2.24189 4.15412 3.06133 2.87814 3.89444 2.88796 3.98828 3.39642 2.93811 3.13522 4.08514 3.12721 3.61351 3.30518 3.22334 1.40697 2.70989 2.97363 5.28456 3.97893 870 - -
2.68618 4.42225 2.77519 2.73123 3.46354 2.40513 3.72494 3.29354 2.67741 2.69355 4.24690 2.90347 2.73739 3.18146 2.89801 2.37887 2.77519 2.98518 4.58477 3.61503
0.03782 3.68971 4.41206 0.61958 0.77255 0.64191 0.74715
589 2.83528 4.41805 3.85371 3.44211 3.14741 3.69369 4.22020 2.30593 3.21644 1.77982 1.78422 3.73947 4.16154 3.61600 3.44155 3.16108 3.14135 2.28792 4.95809 3.69770 871 - -

2.68618 4.42225 2.77519 2.73123 3.46354 2.40513 3.72494 3.29354 2.67741 2.69355 4.24690 2.90347 2.73739 3.18146 2.89801 2.37887 2.77519 2.98518 4.58477 3.61503
0.03782 3.68971 4.41206 0.61958 0.77255 0.54335 0.86941
590 2.24013 4.20506 3.04296 2.78853 4.08218 2.92829 3.95139 3.52831 2.86553 3.20893 4.07314 3.08243 2.72187 3.20563 3.20232 1.56893 2.68629 3.06307 5.42198 4.15107 872 - -
2.68618 4.42225 2.77519 2.73123 3.46354 2.40513 3.72494 3.29354 2.67741 2.69355 4.24690 2.90347 2.73739 3.18146 2.89801 2.37887 2.77519 2.98518 4.58477 3.61503
0.03484 3.77017 4.49252 0.61958 0.77255 0.58585 0.81335
591 2.76849 4.72063 3.18485 2.69780 3.85695 3.47885 3.66453 3.17090 1.62253 2.42364 3.82557 3.12581 3.91317 2.86620 2.45693 2.85618 3.00423 2.95021 5.16536 3.88061 873 - -
2.68618 4.42225 2.77519 2.73123 3.46354 2.40513 3.72494 3.29354 2.67741 2.69355 4.24690 2.90347 2.73739 3.18146 2.89801 2.37887 2.77519 2.98518 4.58477 3.61503
0.03484 3.77017 4.49252 0.61958 0.77255 0.58585 0.81335
592 2.54807 4.38467 3.26273 2.89498 3.58303 3.28587 3.90783 2.91241 2.78758 2.17170 3.66608 3.25452 2.01224 3.20793 3.09401 2.72382 2.89363 2.70463 5.08541 3.77972 874 - -
2.68618 4.42225 2.77519 2.73123 3.46354 2.40513 3.72494 3.29354 2.67741 2.69355 4.24690 2.90347 2.73739 3.18146 2.89801 2.37887 2.77519 2.98518 4.58477 3.61503
0.03484 3.77017 4.49252 0.61958 0.77255 0.58585 0.81335
593 1.85759 4.09720 3.18974 2.94922 4.08930 2.86744 4.05680 3.42140 3.00998 3.19078 4.07275 3.15463 3.60385 3.33511 3.31523 1.52154 2.63750 2.95607 5.46531 4.21573 875 - -
2.68618 4.42225 2.77519 2.73123 3.46354 2.40513 3.72494 3.29354 2.67741 2.69355 4.24690 2.90347 2.73739 3.18146 2.89801 2.37887 2.77519 2.98518 4.58477 3.61503
0.03484 3.77017 4.49252 0.61958 0.77255 0.58585 0.81335
594 2.38515 4.24455 3.31312 2.95698 3.73374 3.14284 3.98423 2.90467 2.89841 2.69869 3.73742 3.25209 2.03009 3.27440 3.20884 2.57651 2.78121 2.22962 5.21836 3.94758 876 - -
2.68618 4.42225 2.77519 2.73123 3.46354 2.40513 3.72494 3.29354 2.67741 2.69355 4.24690 2.90347 2.73739 3.18146 2.89801 2.37887 2.77519 2.98518 4.58477 3.61503
0.03484 3.77017 4.49252 0.61958 0.77255 0.58585 0.81335
595 3.20921 4.62169 4.10726 3.71061 1.74106 4.02345 3.43717 2.98899 3.58755 2.40525 3.63002 3.75016 4.37423 3.73290 3.75505 3.37650 3.44619 2.89639 3.67254 1.35242 877 - -
2.68618 4.42225 2.77519 2.73123 3.46354 2.40513 3.72494 3.29354 2.67741 2.69355 4.24690 2.90347 2.73739 3.18146 2.89801 2.37887 2.77519 2.98518 4.58477 3.61503
0.03484 3.77017 4.49252 0.61958 0.77255 0.58585 0.81335
596 2.76849 4.72063 3.18485 2.69780 3.85695 3.47885 3.66453 3.17090 1.62253 2.42364 3.82557 3.12581 3.91317 2.86620 2.45693 2.85618 3.00423 2.95021 5.16536 3.88061 878 - -
2.68618 4.42225 2.77519 2.73123 3.46354 2.40513 3.72494 3.29354 2.67741 2.69355 4.24690 2.90347 2.73739 3.18146 2.89801 2.37887 2.77519 2.98518 4.58477 3.61503
0.03484 3.77017 4.49252 0.61958 0.77255 0.58585 0.81335
597 2.54807 4.38467 3.26273 2.89498 3.58303 3.28587 3.90783 2.91241 2.78758 2.17170 3.66608 3.25452 2.01224 3.20793 3.09401 2.72382 2.89363 2.70463 5.08541 3.77972 879 - -
2.68618 4.42225 2.77519 2.73123 3.46354 2.40513 3.72494 3.29354 2.67741 2.69355 4.24690 2.90347 2.73739 3.18146 2.89801 2.37887 2.77519 2.98518 4.58477 3.61503
0.03484 3.77017 4.49252 0.61958 0.77255 0.58585 0.81335
598 2.18710 4.14614 3.04699 2.84366 4.16386 2.30148 4.02217 3.62709 2.98014 3.31330 4.16049 3.09670 3.59654 3.28983 3.30621 1.49811 2.66132 3.10487 5.49469 4.24102 880 - -
2.68618 4.42225 2.77519 2.73123 3.46354 2.40513 3.72494 3.29354 2.67741 2.69355 4.24690 2.90347 2.73739 3.18146 2.89801 2.37887 2.77519 2.98518 4.58477 3.61503
0.03484 3.77017 4.49252 0.61958 0.77255 0.58585 0.81335
599 2.80190 4.90694 2.94134 2.20444 4.24660 3.40887 3.59604 3.64475 2.08549 3.19437 4.07903 2.99166 3.86067 2.75938 1.68280 2.82038 3.02806 3.33545 5.33387 4.08451 881 - -
2.68618 4.42225 2.77519 2.73123 3.46354 2.40513 3.72494 3.29354 2.67741 2.69355 4.24690 2.90347 2.73739 3.18146 2.89801 2.37887 2.77519 2.98518 4.58477 3.61503
0.03484 3.77017 4.49252 0.61958 0.77255 0.58585 0.81335
600 2.71861 4.80300 2.70043 2.44235 3.61000 3.30160 2.12548 3.60139 2.42289 3.16298 4.06469 2.46784 3.82503 2.88810 2.76763 2.74591 2.99590 3.28008 5.01131 3.52408 882 - -
2.68618 4.42225 2.77519 2.73123 3.46354 2.40513 3.72494 3.29354 2.67741 2.69355 4.24690 2.90347 2.73739 3.18146 2.89801 2.37887 2.77519 2.98518 4.58477 3.61503
0.03484 3.77017 4.49252 0.61958 0.77255 0.58585 0.81335

601 2.93466 4.35132 4.37857 3.89567 3.36008 4.11363 4.65811 1.36434 3.75203 1.89175 3.21454 4.15667 4.48774 4.05784 3.98031 3.49829 3.21542 1.53752 5.28440 4.05823 883 - -
2.68618 4.42225 2.77519 2.73123 3.46354 2.40513 3.72494 3.29354 2.67741 2.69355 4.24690 2.90347 2.73739 3.18146 2.89801 2.37887 2.77519 2.98518 4.58477 3.61503
0.03484 3.77017 4.49252 0.61958 0.77255 0.58585 0.81335
602 2.59681 4.34698 3.49698 3.01165 3.54523 3.44884 3.90808 2.59774 2.69482 2.45758 3.52923 3.35339 3.94197 3.20418 2.51706 2.82300 2.89767 1.73585 5.05917 3.79380 884 - -
2.68618 4.42225 2.77519 2.73123 3.46354 2.40513 3.72494 3.29354 2.67741 2.69355 4.24690 2.90347 2.73739 3.18146 2.89801 2.37887 2.77519 2.98518 4.58477 3.61503
0.03484 3.77017 4.49252 0.61958 0.77255 0.58585 0.81335
603 2.40733 4.27848 3.24788 2.88970 3.61481 3.16671 3.89563 2.98596 2.80237 2.32943 3.74702 3.20592 3.77747 3.20404 3.11071 1.74880 2.78705 2.71178 5.09714 3.75277 885 - -
2.68618 4.42225 2.77519 2.73123 3.46354 2.40513 3.72494 3.29354 2.67741 2.69355 4.24690 2.90347 2.73739 3.18146 2.89801 2.37887 2.77519 2.98518 4.58477 3.61503
0.03484 3.77017 4.49252 0.61958 0.77255 0.58585 0.81335
604 2.28496 4.67823 2.94519 2.57586 4.13743 3.29408 3.68267 3.42353 1.63789 3.10004 4.00904 3.01367 3.81461 2.86528 2.55284 2.71182 2.93301 3.11239 5.34356 4.07876 886 - -
2.68618 4.42225 2.77519 2.73123 3.46354 2.40513 3.72494 3.29354 2.67741 2.69355 4.24690 2.90347 2.73739 3.18146 2.89801 2.37887 2.77519 2.98518 4.58477 3.61503
0.03484 3.77017 4.49252 0.61958 0.77255 0.58585 0.81335
605 2.94488 4.43525 4.19611 3.71780 3.15850 4.00147 4.45024 2.04771 3.52453 1.28618 3.07404 4.00278 4.38539 3.85225 3.74899 3.38683 3.22545 1.79805 5.07585 3.82704 887 - -
2.68618 4.42225 2.77519 2.73123 3.46354 2.40513 3.72494 3.29354 2.67741 2.69355 4.24690 2.90347 2.73739 3.18146 2.89801 2.37887 2.77519 2.98518 4.58477 3.61503
0.03484 3.77017 4.49252 0.61958 0.77255 0.58585 0.81335
606 2.72578 4.79172 3.01049 2.58440 4.14023 3.35969 3.61710 3.63223 2.12069 3.19209 4.07087 2.53141 3.84124 2.79535 1.69282 2.77020 2.98497 3.30162 5.28470 4.00484 888 - -
2.68618 4.42225 2.77519 2.73123 3.46354 2.40513 3.72494 3.29354 2.67741 2.69355 4.24690 2.90347 2.73739 3.18146 2.89801 2.37887 2.77519 2.98518 4.58477 3.61503
0.03484 3.77017 4.49252 0.61958 0.77255 0.58585 0.81335
607 2.50778 4.63233 2.15839 2.34208 4.19863 3.07212 3.76418 3.66683 2.71137 3.31414 4.17454 2.82973 3.70124 2.96878 3.17192 1.73321 2.87056 3.26005 5.50150 4.13248 889 - -
2.68618 4.42225 2.77519 2.73123 3.46354 2.40513 3.72494 3.29354 2.67741 2.69355 4.24690 2.90347 2.73739 3.18146 2.89801 2.37887 2.77519 2.98518 4.58477 3.61503
0.03484 3.77017 4.49252 0.61958 0.77255 0.58585 0.81335
608 3.20921 4.62169 4.10726 3.71061 1.74106 4.02345 3.43717 2.98899 3.58755 2.40525 3.63002 3.75016 4.37423 3.73290 3.75505 3.37650 3.44619 2.89639 3.67254 1.35242 890 - -
2.68618 4.42225 2.77519 2.73123 3.46354 2.40513 3.72494 3.29354 2.67741 2.69355 4.24690 2.90347 2.73739 3.18146 2.89801 2.37887 2.77519 2.98518 4.58477 3.61503
0.03484 3.77017 4.49252 0.61958 0.77255 0.58585 0.81335
609 2.28108 4.80114 2.48488 1.64200 4.21560 3.18979 3.71883 3.50300 2.56547 3.21031 4.12153 2.82880 3.76839 2.90817 2.98577 2.67951 2.96341 3.18122 5.49514 4.14848 891 - -
2.68618 4.42225 2.77519 2.73123 3.46354 2.40513 3.72494 3.29354 2.67741 2.69355 4.24690 2.90347 2.73739 3.18146 2.89801 2.37887 2.77519 2.98518 4.58477 3.61503
0.03484 3.77017 4.49252 0.61958 0.77255 0.58585 0.81335
610 2.67609 4.44998 3.37568 2.87891 3.50229 3.52814 3.80778 2.73551 2.27136 2.36811 2.22873 3.27701 3.95392 3.08025 2.83316 2.86756 2.92776 2.57828 5.00781 3.75417 892 - -
2.68618 4.42225 2.77519 2.73123 3.46354 2.40513 3.72494 3.29354 2.67741 2.69355 4.24690 2.90347 2.73739 3.18146 2.89801 2.37887 2.77519 2.98518 4.58477 3.61503
0.03484 3.77017 4.49252 0.61958 0.77255 0.58585 0.81335
611 2.29553 4.33986 3.71888 3.31108 3.34827 3.57621 4.18263 2.31654 3.15074 1.48933 3.30771 3.60695 4.08802 3.53800 3.41036 2.98540 3.01009 2.21041 5.08438 3.82081 893 - -
2.68618 4.42225 2.77519 2.73123 3.46354 2.40513 3.72494 3.29354 2.67741 2.69355 4.24690 2.90347 2.73739 3.18146 2.89801 2.37887 2.77519 2.98518 4.58477 3.61503
0.03484 3.77017 4.49252 0.61958 0.77255 0.58585 0.81335
612 2.89284 4.72322 3.03509 2.78791 3.21989 3.36687 1.37832 3.57938 2.63417 3.08509 4.12260 3.20013 3.91550 3.15376 2.90445 2.96541 3.19407 3.31680 4.68586 3.17432 894 - -
2.68618 4.42225 2.77519 2.73123 3.46354 2.40513 3.72494 3.29354 2.67741 2.69355 4.24690 2.90347 2.73739 3.18146 2.89801 2.37887 2.77519 2.98518 4.58477 3.61503

0.03484 3.77017 4.49252 0.61958 0.77255 0.58585 0.81335

613 2.84032 4.97742 2.91393 2.51382 4.30593 3.42392 3.57657 3.72324 1.51973 3.23707 4.12469 2.97751 3.87333 2.36166 2.28645 2.84314 3.05778 3.41056 5.36095 4.10373 895 - -

2.68618 4.42225 2.77519 2.73123 3.46354 2.40513 3.72494 3.29354 2.67741 2.69355 4.24690 2.90347 2.73739 3.18146 2.89801 2.37887 2.77519 2.98518 4.58477 3.61503

0.03484 3.77017 4.49252 0.61958 0.77255 0.58585 0.81335

614 2.65669 4.65927 3.12533 2.66383 4.09124 3.31934 3.67517 3.52673 2.22382 3.10359 4.00176 3.07902 2.86862 2.86432 1.67893 2.73430 2.94094 3.20347 5.26496 4.02774 896 - -

2.68618 4.42225 2.77519 2.73123 3.46354 2.40513 3.72494 3.29354 2.67741 2.69355 4.24690 2.90347 2.73739 3.18146 2.89801 2.37887 2.77519 2.98518 4.58477 3.61503

0.03484 3.77017 4.49252 0.61958 0.77255 0.58585 0.81335

615 1.88101 1.94615 3.85878 3.49166 3.73865 2.99601 4.25561 2.73045 3.34865 2.71977 3.72810 3.48229 3.71582 3.65744 3.55279 2.45698 2.66453 2.43540 5.25417 4.05347 897 - -

2.68618 4.42225 2.77519 2.73123 3.46354 2.40513 3.72494 3.29354 2.67741 2.69355 4.24690 2.90347 2.73739 3.18146 2.89801 2.37887 2.77519 2.98518 4.58477 3.61503

0.03484 3.77017 4.49252 0.61958 0.77255 0.58585 0.81335

616 2.54697 4.32873 3.19933 3.08768 4.22468 0.90617 4.21120 3.80211 3.25454 3.46863 4.44617 3.36448 3.71657 3.58745 3.49887 2.71655 3.02040 3.35061 5.34389 4.32424 898 - -

2.68618 4.42225 2.77519 2.73123 3.46354 2.40513 3.72494 3.29354 2.67741 2.69355 4.24690 2.90347 2.73739 3.18146 2.89801 2.37887 2.77519 2.98518 4.58477 3.61503

0.03484 3.77017 4.49252 0.61958 0.77255 0.58585 0.81335

617 2.30207 4.23404 3.11776 2.83458 4.00284 3.00180 3.95766 3.29734 2.84027 3.03633 3.96904 3.13452 1.94793 3.21557 3.16040 2.46262 2.25766 2.92174 5.37252 4.12454 899 - -

2.68618 4.42225 2.77519 2.73123 3.46354 2.40513 3.72494 3.29354 2.67741 2.69355 4.24690 2.90347 2.73739 3.18146 2.89801 2.37887 2.77519 2.98518 4.58477 3.61503

0.03484 3.77017 4.49252 0.61958 0.77255 0.58585 0.81335

618 2.19700 4.15531 3.00814 2.83235 4.18215 1.66290 4.03261 3.65027 2.99972 3.33932 4.19139 3.09084 3.59852 3.30432 3.32391 1.97123 2.67507 3.12342 5.51032 4.25662 900 - -

2.68618 4.42225 2.77519 2.73123 3.46354 2.40513 3.72494 3.29354 2.67741 2.69355 4.24690 2.90347 2.73739 3.18146 2.89801 2.37887 2.77519 2.98518 4.58477 3.61503

0.03484 3.77017 4.49252 0.61958 0.77255 0.58585 0.81335

619 2.50315 4.55664 2.28232 2.44144 4.07239 3.13177 3.78111 3.36062 2.69693 3.11554 4.01425 2.91203 3.73170 2.99178 3.12764 2.58492 1.87813 3.02390 5.40858 4.08163 901 - -

2.68618 4.42225 2.77519 2.73123 3.46354 2.40513 3.72494 3.29354 2.67741 2.69355 4.24690 2.90347 2.73739 3.18146 2.89801 2.37887 2.77519 2.98518 4.58477 3.61503

0.03484 3.77017 4.49252 0.61958 0.77255 0.58585 0.81335

620 2.65924 4.69858 2.98551 2.58522 4.13471 3.32363 3.66416 3.42221 1.62965 3.08812 3.99472 3.02216 3.82585 2.84279 2.50096 2.72765 2.49743 3.11659 5.32811 4.06818 902 - -

2.68618 4.42225 2.77519 2.73123 3.46354 2.40513 3.72494 3.29354 2.67741 2.69355 4.24690 2.90347 2.73739 3.18146 2.89801 2.37887 2.77519 2.98518 4.58477 3.61503

0.11827 3.77017 2.42478 0.61958 0.77255 0.58585 0.81335

621 1.31618 4.11764 3.28684 3.08071 3.87759 2.94439 4.12282 2.97833 3.09199 2.86583 3.92805 3.27364 3.66709 3.45348 3.34929 2.47460 2.73956 2.66635 5.32423 4.10541 903 - -

2.68618 4.42225 2.77519 2.73123 3.46354 2.40513 3.72494 3.29354 2.67741 2.69355 4.24690 2.90347 2.73739 3.18146 2.89801 2.37887 2.77519 2.98518 4.58477 3.61503

0.03782 3.68971 4.41206 0.61958 0.77255 0.64191 0.74715

622 2.99964 4.56315 3.57279 3.27716 2.34326 3.61999 3.56249 3.03542 3.13970 2.53489 3.73362 3.50967 4.10335 3.49096 3.35748 3.15189 3.28846 2.89883 3.97671 1.33703 904 - -

2.68618 4.42225 2.77519 2.73123 3.46354 2.40513 3.72494 3.29354 2.67741 2.69355 4.24690 2.90347 2.73739 3.18146 2.89801 2.37887 2.77519 2.98518 4.58477 3.61503

0.03782 3.68971 4.41206 0.61958 0.77255 0.64191 0.74715

623 2.81419 4.75052 2.97802 2.64754 4.09431 3.31300 3.68190 3.51431 1.32517 3.12542 4.10705 3.07652 3.83488 2.89173 2.43430 2.87431 3.08699 3.23582 5.23321 4.02251 905 - -

2.68618 4.42225 2.77519 2.73123 3.46354 2.40513 3.72494 3.29354 2.67741 2.69355 4.24690 2.90347 2.73739 3.18146 2.89801 2.37887 2.77519 2.98518 4.58477 3.61503

0.03782 3.68971 4.41206 0.61958 0.77255 0.64191 0.74715

624 2.81190 4.64511 3.28512 2.84967 3.95281 3.32202 3.71995 3.48986 2.20826 3.04039 4.05567 3.21864 3.84267 2.95856 1.30366 2.91066 3.09663 3.22431 5.11666 3.92964 906 - -

2.68618 4.42225 2.77519 2.73123 3.46354 2.40513 3.72494 3.29354 2.67741 2.69355 4.24690 2.90347 2.73739 3.18146 2.89801 2.37887 2.77519 2.98518 4.58477 3.61503

0.03782 3.68971 4.41206 0.61958 0.77255 0.64191 0.74715

625 1.31618 4.11764 3.28684 3.08071 3.87759 2.94439 4.12282 2.97833 3.09199 2.86583 3.92805 3.27364 3.66709 3.45348 3.34929 2.47460 2.73956 2.66635 5.32423 4.10541 907 - -

2.68618 4.42225 2.77519 2.73123 3.46354 2.40513 3.72494 3.29354 2.67741 2.69355 4.24690 2.90347 2.73739 3.18146 2.89801 2.37887 2.77519 2.98518 4.58477 3.61503

0.03782 3.68971 4.41206 0.61958 0.77255 0.64191 0.74715

626 2.33123 4.17207 3.27908 3.03470 3.81909 3.01404 4.06407 2.92517 2.97384 2.79402 3.86593 3.26778 3.70693 3.38105 3.23550 2.53154 1.52801 2.64069 5.27454 4.03869 908 - -

2.68618 4.42225 2.77519 2.73123 3.46354 2.40513 3.72494 3.29354 2.67741 2.69355 4.24690 2.90347 2.73739 3.18146 2.89801 2.37887 2.77519 2.98518 4.58477 3.61503

0.03782 3.68971 4.41206 0.61958 0.77255 0.64191 0.74715

627 2.81419 4.75052 2.97802 2.64754 4.09431 3.31300 3.68190 3.51431 1.32517 3.12542 4.10705 3.07652 3.83488 2.89173 2.43430 2.87431 3.08699 3.23582 5.23321 4.02251 909 - -

2.68618 4.42225 2.77519 2.73123 3.46354 2.40513 3.72494 3.29354 2.67741 2.69355 4.24690 2.90347 2.73739 3.18146 2.89801 2.37887 2.77519 2.98518 4.58477 3.61503

0.03782 3.68971 4.41206 0.61958 0.77255 0.64191 0.74715

628 2.75145 4.71252 2.80109 2.56401 3.85261 3.25189 3.71665 3.46461 2.40147 2.98463 4.02911 3.01766 3.80997 1.60421 2.68491 2.81243 3.05413 3.20938 5.16412 3.84799 910 - -

2.68618 4.42225 2.77519 2.73123 3.46354 2.40513 3.72494 3.29354 2.67741 2.69355 4.24690 2.90347 2.73739 3.18146 2.89801 2.37887 2.77519 2.98518 4.58477 3.61503

0.03782 3.68971 4.41206 0.61958 0.77255 0.64191 0.74715

629 2.95570 4.47801 3.88699 3.53725 3.08153 3.73572 4.25048 2.29059 3.30641 1.14168 3.18203 3.82854 4.21045 3.71971 3.50815 3.30044 3.26294 2.27804 4.88351 3.56168 911 - -

2.68618 4.42225 2.77519 2.73123 3.46354 2.40513 3.72494 3.29354 2.67741 2.69355 4.24690 2.90347 2.73739 3.18146 2.89801 2.37887 2.77519 2.98518 4.58477 3.61503

0.03782 3.68971 4.41206 0.61958 0.77255 0.54335 0.86941

630 2.40141 4.41700 2.70383 2.55453 4.18929 1.73123 3.88128 3.70990 2.80231 3.35698 4.22097 2.36040 3.67116 3.12102 3.17730 2.50649 2.82191 3.24651 5.47830 4.17548 912 - -

2.68618 4.42225 2.77519 2.73123 3.46354 2.40513 3.72494 3.29354 2.67741 2.69355 4.24690 2.90347 2.73739 3.18146 2.89801 2.37887 2.77519 2.98518 4.58477 3.61503

0.03484 3.77017 4.49252 0.61958 0.77255 0.58585 0.81335

631 2.59761 4.57547 2.94079 2.59921 3.63453 3.28420 2.16944 3.33659 2.47030 2.96229 3.88740 3.03214 3.81153 2.95185 2.79261 2.68973 2.46530 3.03997 5.03504 3.62643 913 - -

2.68618 4.42225 2.77519 2.73123 3.46354 2.40513 3.72494 3.29354 2.67741 2.69355 4.24690 2.90347 2.73739 3.18146 2.89801 2.37887 2.77519 2.98518 4.58477 3.61503

0.03484 3.77017 4.49252 0.61958 0.77255 0.58585 0.81335

632 2.45585 4.52277 2.63325 2.46119 4.11932 3.04783 3.79387 3.66554 2.67113 3.31043 4.17642 1.83613 3.69709 3.01712 3.05732 2.16941 2.84336 3.23852 5.43658 4.06906 914 - -

2.68618 4.42225 2.77519 2.73123 3.46354 2.40513 3.72494 3.29354 2.67741 2.69355 4.24690 2.90347 2.73739 3.18146 2.89801 2.37887 2.77519 2.98518 4.58477 3.61503

0.03484 3.77017 4.49252 0.61958 0.77255 0.58585 0.81335

633 2.28108 4.80114 2.48488 1.64200 4.21560 3.18979 3.71883 3.50300 2.56547 3.21031 4.12153 2.82880 3.76839 2.90817 2.98577 2.67951 2.96341 3.18122 5.49514 4.14848 915 - -

2.68618 4.42225 2.77519 2.73123 3.46354 2.40513 3.72494 3.29354 2.67741 2.69355 4.24690 2.90347 2.73739 3.18146 2.89801 2.37887 2.77519 2.98518 4.58477 3.61503

0.03484 3.77017 4.49252 0.61958 0.77255 0.58585 0.81335

634 3.09512 4.52617 4.30255 3.81711 2.32913 4.14552 4.22716 2.22933 3.67457 1.18049 2.97018 4.06304 4.44959 3.88828 3.86651 3.51184 3.34022 2.28435 4.61275 3.17335 916 - -

2.68618 4.42225 2.77519 2.73123 3.46354 2.40513 3.72494 3.29354 2.67741 2.69355 4.24690 2.90347 2.73739 3.18146 2.89801 2.37887 2.77519 2.98518 4.58477 3.61503

0.03484 3.77017 4.49252 0.61958 0.77255 0.58585 0.81335

635 2.26094 4.14081 3.34850 3.00787 3.79979 3.02045 4.01433 3.01561 2.96343 2.86998 3.83268 3.23662 3.69872 3.31726 3.26478 1.64551 2.69307 2.26337 5.25923 3.97440 917 - -

2.68618 4.42225 2.77519 2.73123 3.46354 2.40513 3.72494 3.29354 2.67741 2.69355 4.24690 2.90347 2.73739 3.18146 2.89801 2.37887 2.77519 2.98518 4.58477 3.61503

0.03484 3.77017 4.49252 0.61958 0.77255 0.58585 0.81335

636 2.27034 4.18754 3.12269 2.93699 3.97554 2.91956 4.04809 3.48576 3.00358 3.21526 4.15245 3.17622 3.65060 3.36184 3.29057 1.25678 2.74339 3.04360 5.35708 4.05926 918 - -
2.68618 4.42225 2.77519 2.73123 3.46354 2.40513 3.72494 3.29354 2.67741 2.69355 4.24690 2.90347 2.73739 3.18146 2.89801 2.37887 2.77519 2.98518 4.58477 3.61503
0.03484 3.77017 4.49252 0.61958 0.77255 0.58585 0.81335
637 2.27034 4.18754 3.12269 2.93699 3.97554 2.91956 4.04809 3.48576 3.00358 3.21526 4.15245 3.17622 3.65060 3.36184 3.29057 1.25678 2.74339 3.04360 5.35708 4.05926 919 - -
2.68618 4.42225 2.77519 2.73123 3.46354 2.40513 3.72494 3.29354 2.67741 2.69355 4.24690 2.90347 2.73739 3.18146 2.89801 2.37887 2.77519 2.98518 4.58477 3.61503
0.03484 3.77017 4.49252 0.61958 0.77255 0.58585 0.81335
638 2.44981 4.30845 3.19471 2.88893 3.72001 1.77264 3.93696 3.08369 2.85361 2.28743 3.83474 3.21660 3.77326 3.24564 3.15917 2.62230 2.83694 2.79937 5.15259 3.85884 920 - -
2.68618 4.42225 2.77519 2.73123 3.46354 2.40513 3.72494 3.29354 2.67741 2.69355 4.24690 2.90347 2.73739 3.18146 2.89801 2.37887 2.77519 2.98518 4.58477 3.61503
0.03484 3.77017 4.49252 0.61958 0.77255 0.58585 0.81335
639 2.65111 4.90543 1.58774 2.17930 4.35577 3.09546 3.72423 3.84738 2.71083 3.46494 4.33348 2.71827 3.72385 2.91603 3.22856 2.25043 2.98781 3.44391 5.63130 4.20546 921 - -
2.68618 4.42225 2.77519 2.73123 3.46354 2.40513 3.72494 3.29354 2.67741 2.69355 4.24690 2.90347 2.73739 3.18146 2.89801 2.37887 2.77519 2.98518 4.58477 3.61503
0.03484 3.77017 4.49252 0.61958 0.77255 0.58585 0.81335
640 2.65345 4.79904 2.52573 1.67202 4.19122 3.21184 3.70296 3.48058 2.52159 3.18071 4.08793 2.84104 3.77398 2.88874 2.93045 2.68095 2.49512 3.16410 5.46619 4.12477 922 - -
2.68618 4.42225 2.77519 2.73123 3.46354 2.40513 3.72494 3.29354 2.67741 2.69355 4.24690 2.90347 2.73739 3.18146 2.89801 2.37887 2.77519 2.98518 4.58477 3.61503
0.03484 3.77017 4.49252 0.61958 0.77255 0.58585 0.81335
641 2.48008 1.17448 3.97088 3.68967 3.70759 3.12172 4.35965 2.83708 3.51678 2.75586 3.87522 3.70400 3.82057 3.86754 3.65826 2.75098 2.93872 2.58795 5.14219 3.99513 923 - -
2.68618 4.42225 2.77519 2.73123 3.46354 2.40513 3.72494 3.29354 2.67741 2.69355 4.24690 2.90347 2.73739 3.18146 2.89801 2.37887 2.77519 2.98518 4.58477 3.61503
0.03484 3.77017 4.49252 0.61958 0.77255 0.58585 0.81335
642 2.69483 4.86526 2.33489 2.38836 4.18026 3.29527 3.62958 3.64692 2.31691 3.22207 4.08551 2.89267 3.79069 2.80009 1.83525 2.70709 2.95487 3.30922 5.36335 4.04633 924 - -
2.68618 4.42225 2.77519 2.73123 3.46354 2.40513 3.72494 3.29354 2.67741 2.69355 4.24690 2.90347 2.73739 3.18146 2.89801 2.37887 2.77519 2.98518 4.58477 3.61503
0.03484 3.77017 4.49252 0.61958 0.77255 0.58585 0.81335
643 2.73450 4.65894 2.41909 2.57171 3.06625 3.40262 3.61895 3.23820 2.73611 2.85725 3.82656 3.03786 3.88997 3.03863 3.13514 2.80830 2.99859 2.99240 4.60574 1.96520 925 - -
2.68618 4.42225 2.77519 2.73123 3.46354 2.40513 3.72494 3.29354 2.67741 2.69355 4.24690 2.90347 2.73739 3.18146 2.89801 2.37887 2.77519 2.98518 4.58477 3.61503
0.03484 3.77017 4.49252 0.61958 0.77255 0.58585 0.81335
644 2.37693 4.15764 3.48566 3.14103 3.68408 2.54033 4.09410 2.63818 3.09058 2.65613 3.68217 3.37294 3.81276 3.43620 3.37064 2.61644 2.77892 1.60077 5.19704 3.94814 926 - -
2.68618 4.42225 2.77519 2.73123 3.46354 2.40513 3.72494 3.29354 2.67741 2.69355 4.24690 2.90347 2.73739 3.18146 2.89801 2.37887 2.77519 2.98518 4.58477 3.61503
0.03484 3.77017 4.49252 0.61958 0.77255 0.58585 0.81335
645 2.58196 4.42545 2.55590 2.72830 3.69970 3.35425 3.90552 2.63071 2.86049 2.65197 3.68165 3.16412 3.88758 3.17295 3.24709 2.76060 2.89494 1.73310 5.20312 3.89272 927 - -
2.68618 4.42225 2.77519 2.73123 3.46354 2.40513 3.72494 3.29354 2.67741 2.69355 4.24690 2.90347 2.73739 3.18146 2.89801 2.37887 2.77519 2.98518 4.58477 3.61503
0.03484 3.77017 4.49252 0.61958 0.77255 0.58585 0.81335
646 3.17564 4.58511 3.94100 3.62404 2.57579 3.62057 3.78512 3.19762 3.34942 2.60887 3.83593 3.79443 4.13066 3.74275 3.49488 3.38009 3.47161 3.08517 1.12004 2.58381 928 - -
2.68618 4.42225 2.77519 2.73123 3.46354 2.40513 3.72494 3.29354 2.67741 2.69355 4.24690 2.90347 2.73739 3.18146 2.89801 2.37887 2.77519 2.98518 4.58477 3.61503
0.03484 3.77017 4.49252 0.61958 0.77255 0.58585 0.81335
647 2.68132 4.42881 3.50068 2.96272 3.42197 3.54956 3.83103 2.72501 2.58680 2.26855 2.18581 3.33734 3.97164 3.12022 2.47347 2.88709 2.93635 2.59192 4.96239 3.70776 929 - -
2.68618 4.42225 2.77519 2.73123 3.46354 2.40513 3.72494 3.29354 2.67741 2.69355 4.24690 2.90347 2.73739 3.18146 2.89801 2.37887 2.77519 2.98518 4.58477 3.61503

0.03484 3.77017 4.49252 0.61958 0.77255 0.58585 0.81335

648 2.27660 4.23768 3.01058 2.77030 4.05860 2.95284 3.94086 3.50511 2.84434 3.19142 4.07186 3.07968 1.93049 3.19832 3.17646 2.05199 2.71634 3.06074 5.40205 4.12410 930 - -

2.68623 4.42230 2.77525 2.73129 3.46359 2.40518 3.72500 3.29359 2.67746 2.69360 4.24695 2.90352 2.73731 3.18152 2.89789 2.37892 2.77495 2.98505 4.58391 3.61508

0.11827 2.29906 4.49252 1.22767 0.34669 0.58585 0.81335

649 2.99296 4.48709 4.17002 3.65072 3.05848 4.04812 4.36666 2.17613 3.42907 1.43366 1.94421 3.96785 4.37573 3.75614 3.65166 3.40307 3.24424 2.21062 4.98380 3.76885 937 - -

2.68618 4.42225 2.77519 2.73123 3.46354 2.40513 3.72494 3.29354 2.67741 2.69355 4.24690 2.90347 2.73739 3.18146 2.89801 2.37887 2.77519 2.98518 4.58477 3.61503

0.03484 3.77017 4.49252 0.61958 0.77255 0.58585 0.81335

650 2.67341 4.30167 3.75394 3.33125 1.90252 3.56946 3.95163 2.49754 3.24609 2.13228 3.32882 3.57899 4.06248 3.53575 3.49942 2.95417 2.49647 2.37758 4.59028 3.11954 938 - -

2.68618 4.42225 2.77519 2.73123 3.46354 2.40513 3.72494 3.29354 2.67741 2.69355 4.24690 2.90347 2.73739 3.18146 2.89801 2.37887 2.77519 2.98518 4.58477 3.61503

0.03484 3.77017 4.49252 0.61958 0.77255 0.58585 0.81335

651 2.54697 4.32873 3.19933 3.08768 4.22468 0.90617 4.21120 3.80211 3.25454 3.46863 4.44617 3.36448 3.71657 3.58745 3.49887 2.71655 3.02040 3.35061 5.34389 4.32424 939 - -

2.68618 4.42225 2.77519 2.73123 3.46354 2.40513 3.72494 3.29354 2.67741 2.69355 4.24690 2.90347 2.73739 3.18146 2.89801 2.37887 2.77519 2.98518 4.58477 3.61503

0.03484 3.77017 4.49252 0.61958 0.77255 0.58585 0.81335

652 2.73870 4.52874 3.15575 2.40297 3.50657 3.51877 3.85558 2.69292 2.68568 1.70601 3.50664 3.23800 3.97497 3.13453 2.99702 2.91565 2.99792 2.54928 5.05739 3.76289 940 - -

2.68618 4.42225 2.77519 2.73123 3.46354 2.40513 3.72494 3.29354 2.67741 2.69355 4.24690 2.90347 2.73739 3.18146 2.89801 2.37887 2.77519 2.98518 4.58477 3.61503

0.03484 3.77017 4.49252 0.61958 0.77255 0.58585 0.81335

653 2.32147 4.25744 3.00334 2.80454 4.19042 1.56210 4.00746 3.65793 2.95622 3.31879 4.19290 3.11673 2.54157 3.27665 3.28820 2.46887 2.77256 3.17815 5.44597 4.25707 941 - -

2.68618 4.42225 2.77519 2.73123 3.46354 2.40513 3.72494 3.29354 2.67741 2.69355 4.24690 2.90347 2.73739 3.18146 2.89801 2.37887 2.77519 2.98518 4.58477 3.61503

0.03484 3.77017 4.49252 0.61958 0.77255 0.58585 0.81335

654 2.48238 4.43605 2.95134 2.65343 3.78805 3.18738 3.81301 3.11130 2.67556 2.92421 3.87902 1.96356 3.77564 3.06702 3.02877 2.61113 2.83540 2.39620 5.20912 3.85130 942 - -

2.68618 4.42225 2.77519 2.73123 3.46354 2.40513 3.72494 3.29354 2.67741 2.69355 4.24690 2.90347 2.73739 3.18146 2.89801 2.37887 2.77519 2.98518 4.58477 3.61503

0.03484 3.77017 4.49252 0.61958 0.77255 0.58585 0.81335

655 2.61894 4.59360 3.21368 2.72357 4.03744 3.32415 3.68719 3.37173 2.21195 3.00867 3.93254 3.11092 3.82987 2.87932 1.73387 2.71882 2.46432 3.07052 5.25752 4.01572 943 - -

2.68618 4.42225 2.77519 2.73123 3.46354 2.40513 3.72494 3.29354 2.67741 2.69355 4.24690 2.90347 2.73739 3.18146 2.89801 2.37887 2.77519 2.98518 4.58477 3.61503

0.03484 3.77017 4.49252 0.61958 0.77255 0.58585 0.81335

656 2.27529 4.29244 3.69503 3.24296 3.37272 3.52128 4.11825 2.39040 3.09056 2.07542 2.07709 3.54151 4.02675 3.45963 3.36161 2.90168 2.93604 2.27352 5.06515 3.84077 944 - -

2.68618 4.42225 2.77519 2.73123 3.46354 2.40513 3.72494 3.29354 2.67741 2.69355 4.24690 2.90347 2.73739 3.18146 2.89801 2.37887 2.77519 2.98518 4.58477 3.61503

0.03484 3.77017 4.49252 0.61958 0.77255 0.58585 0.81335

657 2.69488 4.34888 3.72239 3.29227 3.34176 3.59876 4.15608 2.32231 3.10054 1.50034 3.29612 3.59787 4.09302 3.50499 3.35981 2.99561 2.51905 2.21833 5.06997 3.80979 945 - -

2.68609 4.42230 2.77524 2.73128 3.46358 2.40517 3.72499 3.29358 2.67745 2.69359 4.24694 2.90334 2.73744 3.18151 2.89805 2.37891 2.77509 2.98486 4.58481 3.61508

0.11827 2.29906 4.49252 1.11113 0.39926 0.58585 0.81335

658 2.44623 4.43610 3.04047 2.67220 3.99781 3.14370 3.76161 3.45881 2.46715 3.09760 3.97471 3.05190 3.73366 2.97924 2.35580 1.75347 2.80434 3.08599 5.28553 3.99681 951 - -

2.68618 4.42225 2.77519 2.73123 3.46354 2.40513 3.72494 3.29354 2.67741 2.69355 4.24690 2.90347 2.73739 3.18146 2.89801 2.37887 2.77519 2.98518 4.58477 3.61503

0.03484 3.77017 4.49252 0.61958 0.77255 0.58585 0.81335

659 2.75857 4.49046 3.52993 3.01527 3.37635 3.58885 3.84821 2.71662 2.57262 1.63094 3.42600 3.38198 4.01551 3.15253 2.40468 2.96324 3.01349 2.60216 4.94067 3.64349 952 - -

2.68618 4.42225 2.77519 2.73123 3.46354 2.40513 3.72494 3.29354 2.67741 2.69355 4.24690 2.90347 2.73739 3.18146 2.89801 2.37887 2.77519 2.98518 4.58477 3.61503
0.03484 3.77017 4.49252 0.61958 0.77255 0.58585 0.81335
660 2.15946 4.18672 3.79967 3.41990 3.62641 3.39453 4.31942 2.18835 3.32698 2.37422 3.53513 3.61900 4.00306 3.66926 3.58849 2.82385 2.89020 1.44675 5.30391 4.06572 953 - -
2.68618 4.42225 2.77519 2.73123 3.46354 2.40513 3.72494 3.29354 2.67741 2.69355 4.24690 2.90347 2.73739 3.18146 2.89801 2.37887 2.77519 2.98518 4.58477 3.61503
0.03484 3.77017 4.49252 0.61958 0.77255 0.58585 0.81335
661 2.18813 4.12440 3.18845 2.92084 4.05717 2.90063 4.01934 3.38659 2.94257 3.14996 4.03454 3.14827 3.62004 3.29028 3.25068 1.55485 2.20892 2.94213 5.43018 4.17681 954 - -
2.68618 4.42225 2.77519 2.73123 3.46354 2.40513 3.72494 3.29354 2.67741 2.69355 4.24690 2.90347 2.73739 3.18146 2.89801 2.37887 2.77519 2.98518 4.58477 3.61503
0.03484 3.77017 4.49252 0.61958 0.77255 0.58585 0.81335
662 2.45671 4.21330 3.52022 3.13081 3.59680 3.28656 4.07635 2.53346 3.04803 2.48397 3.56876 3.40291 2.91411 3.40750 3.33700 2.70526 2.82775 1.61978 5.15763 3.90625 955 - -
2.68618 4.42225 2.77519 2.73123 3.46354 2.40513 3.72494 3.29354 2.67741 2.69355 4.24690 2.90347 2.73739 3.18146 2.89801 2.37887 2.77519 2.98518 4.58477 3.61503
0.03484 3.77017 4.49252 0.61958 0.77255 0.58585 0.81335
663 3.13253 4.56584 4.09680 3.79468 1.19577 3.84066 3.84004 2.67211 3.70489 2.06767 3.41979 3.90547 4.30395 3.90218 3.84989 3.40595 3.43486 2.65464 4.12500 2.50711 956 - -
2.68618 4.42225 2.77519 2.73123 3.46354 2.40513 3.72494 3.29354 2.67741 2.69355 4.24690 2.90347 2.73739 3.18146 2.89801 2.37887 2.77519 2.98518 4.58477 3.61503
0.03484 3.77017 4.49252 0.61958 0.77255 0.58585 0.81335
664 3.04313 4.53765 4.02310 3.66600 3.10228 3.84857 4.35390 2.30515 3.43459 1.01279 3.16360 3.95091 4.30396 3.82765 3.62603 3.41241 3.34344 2.31521 4.94095 3.63959 957 - -
2.68618 4.42225 2.77519 2.73123 3.46354 2.40513 3.72494 3.29354 2.67741 2.69355 4.24690 2.90347 2.73739 3.18146 2.89801 2.37887 2.77519 2.98518 4.58477 3.61503
0.03484 3.77017 4.49252 0.61958 0.77255 0.58585 0.81335
665 2.66322 4.43265 3.29057 2.93006 2.78407 3.43385 3.64113 3.06314 2.86050 2.69718 3.69911 3.26280 3.93884 3.22381 3.15986 2.39153 2.96565 2.82856 4.36693 1.85417 958 - -
2.68618 4.42225 2.77519 2.73123 3.46354 2.40513 3.72494 3.29354 2.67741 2.69355 4.24690 2.90347 2.73739 3.18146 2.89801 2.37887 2.77519 2.98518 4.58477 3.61503
0.03484 3.77017 4.49252 0.61958 0.77255 0.58585 0.81335
666 1.43345 4.07085 3.25810 3.00975 4.05952 2.86807 4.08492 3.33577 3.05268 3.14453 4.03703 3.18786 3.60730 3.37460 3.34633 1.98274 2.62978 2.89084 5.45085 4.21302 959 - -
2.68618 4.42225 2.77519 2.73123 3.46354 2.40513 3.72494 3.29354 2.67741 2.69355 4.24690 2.90347 2.73739 3.18146 2.89801 2.37887 2.77519 2.98518 4.58477 3.61503
0.03484 3.77017 4.49252 0.61958 0.77255 0.58585 0.81335
667 3.04313 4.53765 4.02310 3.66600 3.10228 3.84857 4.35390 2.30515 3.43459 1.01279 3.16360 3.95091 4.30396 3.82765 3.62603 3.41241 3.34344 2.31521 4.94095 3.63959 960 - -
2.68618 4.42225 2.77519 2.73123 3.46354 2.40513 3.72494 3.29354 2.67741 2.69355 4.24690 2.90347 2.73739 3.18146 2.89801 2.37887 2.77519 2.98518 4.58477 3.61503
0.03484 3.77017 4.49252 0.61958 0.77255 0.58585 0.81335
668 2.55900 4.30410 3.45920 3.06729 3.36248 3.38779 3.95816 2.62762 2.91384 1.67306 3.48439 3.37881 3.93011 3.32528 3.18945 2.37950 2.89365 2.45532 4.95388 3.61334 961 - -
2.68618 4.42225 2.77519 2.73123 3.46354 2.40513 3.72494 3.29354 2.67741 2.69355 4.24690 2.90347 2.73739 3.18146 2.89801 2.37887 2.77519 2.98518 4.58477 3.61503
0.11827 3.77017 2.42478 0.61958 0.77255 0.58585 0.81335
669 2.33123 4.17207 3.27908 3.03470 3.81909 3.01404 4.06407 2.92517 2.97384 2.79402 3.86593 3.26778 3.70693 3.38105 3.23550 2.53154 1.52801 2.64069 5.27454 4.03869 962 - -
2.68618 4.42225 2.77519 2.73123 3.46354 2.40513 3.72494 3.29354 2.67741 2.69355 4.24690 2.90347 2.73739 3.18146 2.89801 2.37887 2.77519 2.98518 4.58477 3.61503
0.03782 3.68971 4.41206 0.61958 0.77255 0.64191 0.74715
670 2.80535 4.87482 1.22337 2.25740 4.23128 3.08281 3.79410 3.78082 2.84781 3.42775 4.41212 2.79951 3.72433 3.04114 3.32941 2.79671 3.14408 3.44355 5.43550 4.14798 963 - -
2.68618 4.42225 2.77519 2.73123 3.46354 2.40513 3.72494 3.29354 2.67741 2.69355 4.24690 2.90347 2.73739 3.18146 2.89801 2.37887 2.77519 2.98518 4.58477 3.61503
0.03782 3.68971 4.41206 0.61958 0.77255 0.64191 0.74715

671 2.81190 4.64511 3.28512 2.84967 3.95281 3.32202 3.71995 3.48986 2.20826 3.04039 4.05567 3.21864 3.84267 2.95856 1.30366 2.91066 3.09663 3.22431 5.11666 3.92964 964 - -
2.68618 4.42225 2.77519 2.73123 3.46354 2.40513 3.72494 3.29354 2.67741 2.69355 4.24690 2.90347 2.73739 3.18146 2.89801 2.37887 2.77519 2.98518 4.58477 3.61503
0.03782 3.68971 4.41206 0.61958 0.77255 0.64191 0.74715
672 2.66384 4.26718 3.86051 3.52869 3.42541 3.52084 4.36452 2.09728 3.39277 2.15394 3.43086 3.74448 4.09895 3.77068 3.62046 3.02131 3.03475 1.29720 5.17454 3.88959 965 - -
2.68618 4.42225 2.77519 2.73123 3.46354 2.40513 3.72494 3.29354 2.67741 2.69355 4.24690 2.90347 2.73739 3.18146 2.89801 2.37887 2.77519 2.98518 4.58477 3.61503
0.03782 3.68971 4.41206 0.61958 0.77255 0.64191 0.74715
673 2.83528 4.41805 3.85371 3.44211 3.14741 3.69369 4.22020 2.30593 3.21644 1.77982 1.78422 3.73947 4.16154 3.61600 3.44155 3.16108 3.14135 2.28792 4.95809 3.69770 966 - -
2.68618 4.42225 2.77519 2.73123 3.46354 2.40513 3.72494 3.29354 2.67741 2.69355 4.24690 2.90347 2.73739 3.18146 2.89801 2.37887 2.77519 2.98518 4.58477 3.61503
0.03782 3.68971 4.41206 0.61958 0.77255 0.64191 0.74715
674 2.95570 4.47801 3.88699 3.53725 3.08153 3.73572 4.25048 2.29059 3.30641 1.14168 3.18203 3.82854 4.21045 3.71971 3.50815 3.30044 3.26294 2.27804 4.88351 3.56168 967 - -
2.68618 4.42225 2.77519 2.73123 3.46354 2.40513 3.72494 3.29354 2.67741 2.69355 4.24690 2.90347 2.73739 3.18146 2.89801 2.37887 2.77519 2.98518 4.58477 3.61503
0.03782 3.68971 4.41206 0.61958 0.77255 0.64191 0.74715
675 2.66384 4.26718 3.86051 3.52869 3.42541 3.52084 4.36452 2.09728 3.39277 2.15394 3.43086 3.74448 4.09895 3.77068 3.62046 3.02131 3.03475 1.29720 5.17454 3.88959 968 - -
2.68618 4.42225 2.77519 2.73123 3.46354 2.40513 3.72494 3.29354 2.67741 2.69355 4.24690 2.90347 2.73739 3.18146 2.89801 2.37887 2.77519 2.98518 4.58477 3.61503
0.03782 3.68971 4.41206 0.61958 0.77255 0.64191 0.74715
676 2.80535 4.87482 1.22337 2.25740 4.23128 3.08281 3.79410 3.78082 2.84781 3.42775 4.41212 2.79951 3.72433 3.04114 3.32941 2.79671 3.14408 3.44355 5.43550 4.14798 969 - -
2.68618 4.42225 2.77519 2.73123 3.46354 2.40513 3.72494 3.29354 2.67741 2.69355 4.24690 2.90347 2.73739 3.18146 2.89801 2.37887 2.77519 2.98518 4.58477 3.61503
0.03782 3.68971 4.41206 0.61958 0.77255 0.64191 0.74715
677 2.75145 4.71252 2.80109 2.56401 3.85261 3.25189 3.71665 3.46461 2.40147 2.98463 4.02911 3.01766 3.80997 1.60421 2.68491 2.81243 3.05413 3.20938 5.16412 3.84799 970 - -
2.68618 4.42225 2.77519 2.73123 3.46354 2.40513 3.72494 3.29354 2.67741 2.69355 4.24690 2.90347 2.73739 3.18146 2.89801 2.37887 2.77519 2.98518 4.58477 3.61503
0.03782 3.68971 4.41206 0.61958 0.77255 0.64191 0.74715
678 2.81190 4.64511 3.28512 2.84967 3.95281 3.32202 3.71995 3.48986 2.20826 3.04039 4.05567 3.21864 3.84267 2.95856 1.30366 2.91066 3.09663 3.22431 5.11666 3.92964 971 - -
2.68618 4.42225 2.77519 2.73123 3.46354 2.40513 3.72494 3.29354 2.67741 2.69355 4.24690 2.90347 2.73739 3.18146 2.89801 2.37887 2.77519 2.98518 4.58477 3.61503
0.03782 3.68971 4.41206 0.61958 0.77255 0.64191 0.74715
679 2.58408 4.55777 2.63767 2.50951 3.89313 3.06999 3.79869 3.57286 2.69204 3.24473 4.20914 1.53216 3.71772 3.07984 3.03279 2.66399 2.96516 3.21422 5.22244 3.86110 972 - -
2.68618 4.42225 2.77519 2.73123 3.46354 2.40513 3.72494 3.29354 2.67741 2.69355 4.24690 2.90347 2.73739 3.18146 2.89801 2.37887 2.77519 2.98518 4.58477 3.61503
0.03782 3.68971 4.41206 0.61958 0.77255 0.64191 0.74715
680 2.80535 4.87482 1.22337 2.25740 4.23128 3.08281 3.79410 3.78082 2.84781 3.42775 4.41212 2.79951 3.72433 3.04114 3.32941 2.79671 3.14408 3.44355 5.43550 4.14798 973 - -
2.68618 4.42225 2.77519 2.73123 3.46354 2.40513 3.72494 3.29354 2.67741 2.69355 4.24690 2.90347 2.73739 3.18146 2.89801 2.37887 2.77519 2.98518 4.58477 3.61503
0.03782 3.68971 4.41206 0.61958 0.77255 0.64191 0.74715
681 2.88652 4.37019 4.05193 3.66978 3.26042 3.84728 4.43781 1.34176 3.49290 1.89866 3.23881 3.94049 4.30608 3.86681 3.71057 3.33382 3.19357 1.87474 5.10472 3.81929 974 - -
2.68618 4.42225 2.77519 2.73123 3.46354 2.40513 3.72494 3.29354 2.67741 2.69355 4.24690 2.90347 2.73739 3.18146 2.89801 2.37887 2.77519 2.98518 4.58477 3.61503
0.03782 3.68971 4.41206 0.61958 0.77255 0.64191 0.74715
682 2.33123 4.17207 3.27908 3.03470 3.81909 3.01404 4.06407 2.92517 2.97384 2.79402 3.86593 3.26778 3.70693 3.38105 3.23550 2.53154 1.52801 2.64069 5.27454 4.03869 975 - -
2.68618 4.42225 2.77519 2.73123 3.46354 2.40513 3.72494 3.29354 2.67741 2.69355 4.24690 2.90347 2.73739 3.18146 2.89801 2.37887 2.77519 2.98518 4.58477 3.61503

0.03782 3.68971 4.41206 0.61958 0.77255 0.64191 0.74715

683 2.80535 4.87482 1.22337 2.25740 4.23128 3.08281 3.79410 3.78082 2.84781 3.42775 4.41212 2.79951 3.72433 3.04114 3.32941 2.79671 3.14408 3.44355 5.43550 4.14798 976 - -

2.68618 4.42225 2.77519 2.73123 3.46354 2.40513 3.72494 3.29354 2.67741 2.69355 4.24690 2.90347 2.73739 3.18146 2.89801 2.37887 2.77519 2.98518 4.58477 3.61503

0.03782 3.68971 4.41206 0.61958 0.77255 0.64191 0.74715

684 2.95570 4.47801 3.88699 3.53725 3.08153 3.73572 4.25048 2.29059 3.30641 1.14168 3.18203 3.82854 4.21045 3.71971 3.50815 3.30044 3.26294 2.27804 4.88351 3.56168 977 - -

2.68618 4.42225 2.77519 2.73123 3.46354 2.40513 3.72494 3.29354 2.67741 2.69355 4.24690 2.90347 2.73739 3.18146 2.89801 2.37887 2.77519 2.98518 4.58477 3.61503

0.03782 3.68971 4.41206 0.61958 0.77255 0.64191 0.74715

685 3.03627 4.49697 3.98008 3.66865 1.36975 3.75169 3.82000 2.59321 3.58195 2.01239 3.35868 3.81567 4.22222 3.80445 3.74700 3.31035 3.34138 2.56812 4.14110 2.53835 978 - -

2.68618 4.42225 2.77519 2.73123 3.46354 2.40513 3.72494 3.29354 2.67741 2.69355 4.24690 2.90347 2.73739 3.18146 2.89801 2.37887 2.77519 2.98518 4.58477 3.61503

0.03782 3.68971 4.41206 0.61958 0.77255 0.64191 0.74715

686 2.40719 1.40547 3.87078 3.57616 3.61829 3.05978 4.26483 2.73897 3.40375 2.65893 3.77889 3.61209 3.75249 3.75916 3.55770 2.67680 2.86014 2.49438 5.06450 3.90470 979 - -

2.68618 4.42225 2.77519 2.73123 3.46354 2.40513 3.72494 3.29354 2.67741 2.69355 4.24690 2.90347 2.73739 3.18146 2.89801 2.37887 2.77519 2.98518 4.58477 3.61503

0.03782 3.68971 4.41206 0.61958 0.77255 0.64191 0.74715

687 2.80936 4.85412 2.44285 1.32179 4.18024 3.16567 3.75082 3.59744 2.60890 3.24971 4.24291 2.85883 3.76479 2.97779 2.98976 2.81828 3.11612 3.30456 5.38821 4.12184 980 - -

2.68618 4.42225 2.77519 2.73123 3.46354 2.40513 3.72494 3.29354 2.67741 2.69355 4.24690 2.90347 2.73739 3.18146 2.89801 2.37887 2.77519 2.98518 4.58477 3.61503

0.03782 3.68971 4.41206 0.61958 0.77255 0.64191 0.74715

688 2.53919 4.31574 3.12837 2.94944 3.94108 3.01406 4.03464 3.44720 2.99214 3.09147 4.14117 3.26964 1.25262 3.38748 3.25050 2.70343 2.96896 3.11334 5.17040 4.06284 981 - -

2.68618 4.42225 2.77519 2.73123 3.46354 2.40513 3.72494 3.29354 2.67741 2.69355 4.24690 2.90347 2.73739 3.18146 2.89801 2.37887 2.77519 2.98518 4.58477 3.61503

0.03782 3.68971 4.41206 0.61958 0.77255 0.64191 0.74715

689 3.03627 4.49697 3.98008 3.66865 1.36975 3.75169 3.82000 2.59321 3.58195 2.01239 3.35868 3.81567 4.22222 3.80445 3.74700 3.31035 3.34138 2.56812 4.14110 2.53835 982 - -

2.68618 4.42225 2.77519 2.73123 3.46354 2.40513 3.72494 3.29354 2.67741 2.69355 4.24690 2.90347 2.73739 3.18146 2.89801 2.37887 2.77519 2.98518 4.58477 3.61503

0.03782 3.68971 4.41206 0.61958 0.77255 0.64191 0.74715

690 2.53919 4.31574 3.12837 2.94944 3.94108 3.01406 4.03464 3.44720 2.99214 3.09147 4.14117 3.26964 1.25262 3.38748 3.25050 2.70343 2.96896 3.11334 5.17040 4.06284 983 - -

2.68618 4.42225 2.77519 2.73123 3.46354 2.40513 3.72494 3.29354 2.67741 2.69355 4.24690 2.90347 2.73739 3.18146 2.89801 2.37887 2.77519 2.98518 4.58477 3.61503

0.03782 3.68971 4.41206 0.61958 0.77255 0.64191 0.74715

691 2.80936 4.85412 2.44285 1.32179 4.18024 3.16567 3.75082 3.59744 2.60890 3.24971 4.24291 2.85883 3.76479 2.97779 2.98976 2.81828 3.11612 3.30456 5.38821 4.12184 984 - -

2.68618 4.42225 2.77519 2.73123 3.46354 2.40513 3.72494 3.29354 2.67741 2.69355 4.24690 2.90347 2.73739 3.18146 2.89801 2.37887 2.77519 2.98518 4.58477 3.61503

0.03782 3.68971 4.41206 0.61958 0.77255 0.64191 0.74715

692 2.33123 4.17207 3.27908 3.03470 3.81909 3.01404 4.06407 2.92517 2.97384 2.79402 3.86593 3.26778 3.70693 3.38105 3.23550 2.53154 1.52801 2.64069 5.27454 4.03869 985 - -

2.68618 4.42225 2.77519 2.73123 3.46354 2.40513 3.72494 3.29354 2.67741 2.69355 4.24690 2.90347 2.73739 3.18146 2.89801 2.37887 2.77519 2.98518 4.58477 3.61503

0.03782 3.68971 4.41206 0.61958 0.77255 0.64191 0.74715

693 2.24189 4.15412 3.06133 2.87814 3.89444 2.88796 3.98828 3.39642 2.93811 3.13522 4.08514 3.12721 3.61351 3.30518 3.22334 1.40697 2.70989 2.97363 5.28456 3.97893 986 - -

2.68618 4.42225 2.77519 2.73123 3.46354 2.40513 3.72494 3.29354 2.67741 2.69355 4.24690 2.90347 2.73739 3.18146 2.89801 2.37887 2.77519 2.98518 4.58477 3.61503

0.03782 3.68971 4.41206 0.61958 0.77255 0.64191 0.74715

694 3.06047 4.49594 3.82876 3.49619 2.52194 3.53862 3.70765 3.08393 3.22453 2.50664 3.72585 3.68429 4.04410 3.62345 3.38267 3.26521 3.35391 2.96950 1.35528 2.53548 987 - -

2.68618 4.42225 2.77519 2.73123 3.46354 2.40513 3.72494 3.29354 2.67741 2.69355 4.24690 2.90347 2.73739 3.18146 2.89801 2.37887 2.77519 2.98518 4.58477 3.61503
0.03782 3.68971 4.41206 0.61958 0.77255 0.64191 0.74715
695 2.95570 4.47801 3.88699 3.53725 3.08153 3.73572 4.25048 2.29059 3.30641 1.14168 3.18203 3.82854 4.21045 3.71971 3.50815 3.30044 3.26294 2.27804 4.88351 3.56168 988 - -
2.68618 4.42225 2.77519 2.73123 3.46354 2.40513 3.72494 3.29354 2.67741 2.69355 4.24690 2.90347 2.73739 3.18146 2.89801 2.37887 2.77519 2.98518 4.58477 3.61503
0.03782 3.68971 4.41206 0.61958 0.77255 0.64191 0.74715
696 2.95570 4.47801 3.88699 3.53725 3.08153 3.73572 4.25048 2.29059 3.30641 1.14168 3.18203 3.82854 4.21045 3.71971 3.50815 3.30044 3.26294 2.27804 4.88351 3.56168 989 - -
2.68618 4.42225 2.77519 2.73123 3.46354 2.40513 3.72494 3.29354 2.67741 2.69355 4.24690 2.90347 2.73739 3.18146 2.89801 2.37887 2.77519 2.98518 4.58477 3.61503
0.03782 3.68971 4.41206 0.61958 0.77255 0.64191 0.74715
697 2.53919 4.31574 3.12837 2.94944 3.94108 3.01406 4.03464 3.44720 2.99214 3.09147 4.14117 3.26964 1.25262 3.38748 3.25050 2.70343 2.96896 3.11334 5.17040 4.06284 990 - -
2.68618 4.42225 2.77519 2.73123 3.46354 2.40513 3.72494 3.29354 2.67741 2.69355 4.24690 2.90347 2.73739 3.18146 2.89801 2.37887 2.77519 2.98518 4.58477 3.61503
0.03782 3.68971 4.41206 0.61958 0.77255 0.64191 0.74715
698 2.95570 4.47801 3.88699 3.53725 3.08153 3.73572 4.25048 2.29059 3.30641 1.14168 3.18203 3.82854 4.21045 3.71971 3.50815 3.30044 3.26294 2.27804 4.88351 3.56168 991 - -
2.68618 4.42225 2.77519 2.73123 3.46354 2.40513 3.72494 3.29354 2.67741 2.69355 4.24690 2.90347 2.73739 3.18146 2.89801 2.37887 2.77519 2.98518 4.58477 3.61503
0.03782 3.68971 4.41206 0.61958 0.77255 0.64191 0.74715
699 2.80535 4.87482 1.22337 2.25740 4.23128 3.08281 3.79410 3.78082 2.84781 3.42775 4.41212 2.79951 3.72433 3.04114 3.32941 2.79671 3.14408 3.44355 5.43550 4.14798 992 - -
2.68618 4.42225 2.77519 2.73123 3.46354 2.40513 3.72494 3.29354 2.67741 2.69355 4.24690 2.90347 2.73739 3.18146 2.89801 2.37887 2.77519 2.98518 4.58477 3.61503
0.03782 3.68971 4.41206 0.61958 0.77255 0.64191 0.74715
700 3.03627 4.49697 3.98008 3.66865 1.36975 3.75169 3.82000 2.59321 3.58195 2.01239 3.35868 3.81567 4.22222 3.80445 3.74700 3.31035 3.34138 2.56812 4.14110 2.53835 993 - -
2.68618 4.42225 2.77519 2.73123 3.46354 2.40513 3.72494 3.29354 2.67741 2.69355 4.24690 2.90347 2.73739 3.18146 2.89801 2.37887 2.77519 2.98518 4.58477 3.61503
0.03782 3.68971 4.41206 0.61958 0.77255 0.64191 0.74715
701 2.53919 4.31574 3.12837 2.94944 3.94108 3.01406 4.03464 3.44720 2.99214 3.09147 4.14117 3.26964 1.25262 3.38748 3.25050 2.70343 2.96896 3.11334 5.17040 4.06284 994 - -
2.68618 4.42225 2.77519 2.73123 3.46354 2.40513 3.72494 3.29354 2.67741 2.69355 4.24690 2.90347 2.73739 3.18146 2.89801 2.37887 2.77519 2.98518 4.58477 3.61503
0.03782 3.68971 4.41206 0.61958 0.77255 0.64191 0.74715
702 2.95570 4.47801 3.88699 3.53725 3.08153 3.73572 4.25048 2.29059 3.30641 1.14168 3.18203 3.82854 4.21045 3.71971 3.50815 3.30044 3.26294 2.27804 4.88351 3.56168 995 - -
2.68618 4.42225 2.77519 2.73123 3.46354 2.40513 3.72494 3.29354 2.67741 2.69355 4.24690 2.90347 2.73739 3.18146 2.89801 2.37887 2.77519 2.98518 4.58477 3.61503
0.03782 3.68971 4.41206 0.61958 0.77255 0.64191 0.74715
703 2.58408 4.55777 2.63767 2.50951 3.89313 3.06999 3.79869 3.57286 2.69204 3.24473 4.20914 1.53216 3.71772 3.07984 3.03279 2.66399 2.96516 3.21422 5.22244 3.86110 996 - -
2.68618 4.42225 2.77519 2.73123 3.46354 2.40513 3.72494 3.29354 2.67741 2.69355 4.24690 2.90347 2.73739 3.18146 2.89801 2.37887 2.77519 2.98518 4.58477 3.61503
0.03782 3.68971 4.41206 0.61958 0.77255 0.64191 0.74715
704 2.80535 4.87482 1.22337 2.25740 4.23128 3.08281 3.79410 3.78082 2.84781 3.42775 4.41212 2.79951 3.72433 3.04114 3.32941 2.79671 3.14408 3.44355 5.43550 4.14798 997 - -
2.68618 4.42225 2.77519 2.73123 3.46354 2.40513 3.72494 3.29354 2.67741 2.69355 4.24690 2.90347 2.73739 3.18146 2.89801 2.37887 2.77519 2.98518 4.58477 3.61503
0.03782 3.68971 4.41206 0.61958 0.77255 0.64191 0.74715
705 2.75145 4.71252 2.80109 2.56401 3.85261 3.25189 3.71665 3.46461 2.40147 2.98463 4.02911 3.01766 3.80997 1.60421 2.68491 2.81243 3.05413 3.20938 5.16412 3.84799 998 - -
2.68618 4.42225 2.77519 2.73123 3.46354 2.40513 3.72494 3.29354 2.67741 2.69355 4.24690 2.90347 2.73739 3.18146 2.89801 2.37887 2.77519 2.98518 4.58477 3.61503
0.03782 3.68971 4.41206 0.61958 0.77255 0.64191 0.74715

706 2.95570 4.47801 3.88699 3.53725 3.08153 3.73572 4.25048 2.29059 3.30641 1.14168 3.18203 3.82854 4.21045 3.71971 3.50815 3.30044 3.26294 2.27804 4.88351 3.56168 999 - -
2.68618 4.42225 2.77519 2.73123 3.46354 2.40513 3.72494 3.29354 2.67741 2.69355 4.24690 2.90347 2.73739 3.18146 2.89801 2.37887 2.77519 2.98518 4.58477 3.61503
0.03782 3.68971 4.41206 0.61958 0.77255 0.64191 0.74715
707 2.80535 4.87482 1.22337 2.25740 4.23128 3.08281 3.79410 3.78082 2.84781 3.42775 4.41212 2.79951 3.72433 3.04114 3.32941 2.79671 3.14408 3.44355 5.43550 4.14798 1000 - -
2.68618 4.42225 2.77519 2.73123 3.46354 2.40513 3.72494 3.29354 2.67741 2.69355 4.24690 2.90347 2.73739 3.18146 2.89801 2.37887 2.77519 2.98518 4.58477 3.61503
0.03782 3.68971 4.41206 0.61958 0.77255 0.64191 0.74715
708 2.24189 4.15412 3.06133 2.87814 3.89444 2.88796 3.98828 3.39642 2.93811 3.13522 4.08514 3.12721 3.61351 3.30518 3.22334 1.40697 2.70989 2.97363 5.28456 3.97893 1001 - -
2.68618 4.42225 2.77519 2.73123 3.46354 2.40513 3.72494 3.29354 2.67741 2.69355 4.24690 2.90347 2.73739 3.18146 2.89801 2.37887 2.77519 2.98518 4.58477 3.61503
0.03782 3.68971 4.41206 0.61958 0.77255 0.64191 0.74715
709 3.03627 4.49697 3.98008 3.66865 1.36975 3.75169 3.82000 2.59321 3.58195 2.01239 3.35868 3.81567 4.22222 3.80445 3.74700 3.31035 3.34138 2.56812 4.14110 2.53835 1002 - -
2.68618 4.42225 2.77519 2.73123 3.46354 2.40513 3.72494 3.29354 2.67741 2.69355 4.24690 2.90347 2.73739 3.18146 2.89801 2.37887 2.77519 2.98518 4.58477 3.61503
0.03782 3.68971 4.41206 0.61958 0.77255 0.64191 0.74715
710 2.58408 4.55777 2.63767 2.50951 3.89313 3.06999 3.79869 3.57286 2.69204 3.24473 4.20914 1.53216 3.71772 3.07984 3.03279 2.66399 2.96516 3.21422 5.22244 3.86110 1003 - -
2.68618 4.42225 2.77519 2.73123 3.46354 2.40513 3.72494 3.29354 2.67741 2.69355 4.24690 2.90347 2.73739 3.18146 2.89801 2.37887 2.77519 2.98518 4.58477 3.61503
0.03782 3.68971 4.41206 0.61958 0.77255 0.64191 0.74715
711 2.81190 4.64511 3.28512 2.84967 3.95281 3.32202 3.71995 3.48986 2.20826 3.04039 4.05567 3.21864 3.84267 2.95856 1.30366 2.91066 3.09663 3.22431 5.11666 3.92964 1004 - -
2.68618 4.42225 2.77519 2.73123 3.46354 2.40513 3.72494 3.29354 2.67741 2.69355 4.24690 2.90347 2.73739 3.18146 2.89801 2.37887 2.77519 2.98518 4.58477 3.61503
0.03782 3.68971 4.41206 0.61958 0.77255 0.64191 0.74715
712 2.80936 4.85412 2.44285 1.32179 4.18024 3.16567 3.75082 3.59744 2.60890 3.24971 4.24291 2.85883 3.76479 2.97779 2.98976 2.81828 3.11612 3.30456 5.38821 4.12184 1005 - -
2.68618 4.42225 2.77519 2.73123 3.46354 2.40513 3.72494 3.29354 2.67741 2.69355 4.24690 2.90347 2.73739 3.18146 2.89801 2.37887 2.77519 2.98518 4.58477 3.61503
0.03782 3.68971 4.41206 0.61958 0.77255 0.64191 0.74715
713 2.82202 4.65647 2.97238 2.72383 3.17446 3.30735 1.59825 3.49438 2.57333 3.00834 4.04492 3.13670 3.85542 3.09072 2.84526 2.89735 3.12340 3.23414 4.64084 3.13275 1006 - -
2.68618 4.42225 2.77519 2.73123 3.46354 2.40513 3.72494 3.29354 2.67741 2.69355 4.24690 2.90347 2.73739 3.18146 2.89801 2.37887 2.77519 2.98518 4.58477 3.61503
0.03782 3.68971 4.41206 0.61958 0.77255 0.64191 0.74715
714 2.24189 4.15412 3.06133 2.87814 3.89444 2.88796 3.98828 3.39642 2.93811 3.13522 4.08514 3.12721 3.61351 3.30518 3.22334 1.40697 2.70989 2.97363 5.28456 3.97893 1007 - -
2.68618 4.42225 2.77519 2.73123 3.46354 2.40513 3.72494 3.29354 2.67741 2.69355 4.24690 2.90347 2.73739 3.18146 2.89801 2.37887 2.77519 2.98518 4.58477 3.61503
0.03782 3.68971 4.41206 0.61958 0.77255 0.64191 0.74715
715 2.81190 4.64511 3.28512 2.84967 3.95281 3.32202 3.71995 3.48986 2.20826 3.04039 4.05567 3.21864 3.84267 2.95856 1.30366 2.91066 3.09663 3.22431 5.11666 3.92964 1008 - -
2.68618 4.42225 2.77519 2.73123 3.46354 2.40513 3.72494 3.29354 2.67741 2.69355 4.24690 2.90347 2.73739 3.18146 2.89801 2.37887 2.77519 2.98518 4.58477 3.61503
0.03782 3.68971 4.41206 0.61958 0.77255 0.64191 0.74715
716 2.40719 1.40547 3.87078 3.57616 3.61829 3.05978 4.26483 2.73897 3.40375 2.65893 3.77889 3.61209 3.75249 3.75916 3.55770 2.67680 2.86014 2.49438 5.06450 3.90470 1009 - -
2.68618 4.42225 2.77519 2.73123 3.46354 2.40513 3.72494 3.29354 2.67741 2.69355 4.24690 2.90347 2.73739 3.18146 2.89801 2.37887 2.77519 2.98518 4.58477 3.61503
0.03782 3.68971 4.41206 0.61958 0.77255 0.64191 0.74715
717 2.99964 4.56315 3.57279 3.27716 2.34326 3.61999 3.56249 3.03542 3.13970 2.53489 3.73362 3.50967 4.10335 3.49096 3.35748 3.15189 3.28846 2.89883 3.97671 1.33703 1010 - -
2.68618 4.42225 2.77519 2.73123 3.46354 2.40513 3.72494 3.29354 2.67741 2.69355 4.24690 2.90347 2.73739 3.18146 2.89801 2.37887 2.77519 2.98518 4.58477 3.61503

0.03782 3.68971 4.41206 0.61958 0.77255 0.64191 0.74715

718 2.46513 4.24664 3.09956 2.98169 4.10469 1.07816 4.10397 3.66750 3.13599 3.34383 4.32651 3.26662 3.63194 3.47710 3.38330 2.63370 2.93263 3.23311 5.23953 4.20371 1011 - -

2.68618 4.42225 2.77519 2.73123 3.46354 2.40513 3.72494 3.29354 2.67741 2.69355 4.24690 2.90347 2.73739 3.18146 2.89801 2.37887 2.77519 2.98518 4.58477 3.61503

0.03782 3.68971 4.41206 0.61958 0.77255 0.64191 0.74715

719 2.33123 4.17207 3.27908 3.03470 3.81909 3.01404 4.06407 2.92517 2.97384 2.79402 3.86593 3.26778 3.70693 3.38105 3.23550 2.53154 1.52801 2.64069 5.27454 4.03869 1012 - -

2.68618 4.42225 2.77519 2.73123 3.46354 2.40513 3.72494 3.29354 2.67741 2.69355 4.24690 2.90347 2.73739 3.18146 2.89801 2.37887 2.77519 2.98518 4.58477 3.61503

0.03782 3.68971 4.41206 0.61958 0.77255 0.64191 0.74715

720 2.83528 4.41805 3.85371 3.44211 3.14741 3.69369 4.22020 2.30593 3.21644 1.77982 1.78422 3.73947 4.16154 3.61600 3.44155 3.16108 3.14135 2.28792 4.95809 3.69770 1013 - -

2.68618 4.42225 2.77519 2.73123 3.46354 2.40513 3.72494 3.29354 2.67741 2.69355 4.24690 2.90347 2.73739 3.18146 2.89801 2.37887 2.77519 2.98518 4.58477 3.61503

0.03782 3.68971 4.41206 0.61958 0.77255 0.64191 0.74715

721 2.95570 4.47801 3.88699 3.53725 3.08153 3.73572 4.25048 2.29059 3.30641 1.14168 3.18203 3.82854 4.21045 3.71971 3.50815 3.30044 3.26294 2.27804 4.88351 3.56168 1014 - -

2.68618 4.42225 2.77519 2.73123 3.46354 2.40513 3.72494 3.29354 2.67741 2.69355 4.24690 2.90347 2.73739 3.18146 2.89801 2.37887 2.77519 2.98518 4.58477 3.61503

0.03782 3.68971 4.41206 0.61958 0.77255 0.64191 0.74715

722 2.81419 4.75052 2.97802 2.64754 4.09431 3.31300 3.68190 3.51431 1.32517 3.12542 4.10705 3.07652 3.83488 2.89173 2.43430 2.87431 3.08699 3.23582 5.23321 4.02251 1015 - -

2.68618 4.42225 2.77519 2.73123 3.46354 2.40513 3.72494 3.29354 2.67741 2.69355 4.24690 2.90347 2.73739 3.18146 2.89801 2.37887 2.77519 2.98518 4.58477 3.61503

0.03782 3.68971 4.41206 0.61958 0.77255 0.64191 0.74715

723 2.58408 4.55777 2.63767 2.50951 3.89313 3.06999 3.79869 3.57286 2.69204 3.24473 4.20914 1.53216 3.71772 3.07984 3.03279 2.66399 2.96516 3.21422 5.22244 3.86110 1016 - -

2.68618 4.42225 2.77519 2.73123 3.46354 2.40513 3.72494 3.29354 2.67741 2.69355 4.24690 2.90347 2.73739 3.18146 2.89801 2.37887 2.77519 2.98518 4.58477 3.61503

0.03782 3.68971 4.41206 0.61958 0.77255 0.64191 0.74715

724 2.82202 4.65647 2.97238 2.72383 3.17446 3.30735 1.59825 3.49438 2.57333 3.00834 4.04492 3.13670 3.85542 3.09072 2.84526 2.89735 3.12340 3.23414 4.64084 3.13275 1017 - -

2.68618 4.42225 2.77519 2.73123 3.46354 2.40513 3.72494 3.29354 2.67741 2.69355 4.24690 2.90347 2.73739 3.18146 2.89801 2.37887 2.77519 2.98518 4.58477 3.61503

0.03782 3.68971 4.41206 0.61958 0.77255 0.64191 0.74715

725 2.46513 4.24664 3.09956 2.98169 4.10469 1.07816 4.10397 3.66750 3.13599 3.34383 4.32651 3.26662 3.63194 3.47710 3.38330 2.63370 2.93263 3.23311 5.23953 4.20371 1018 - -

2.68618 4.42225 2.77519 2.73123 3.46354 2.40513 3.72494 3.29354 2.67741 2.69355 4.24690 2.90347 2.73739 3.18146 2.89801 2.37887 2.77519 2.98518 4.58477 3.61503

0.03782 3.68971 4.41206 0.61958 0.77255 0.64191 0.74715

726 2.88652 4.37019 4.05193 3.66978 3.26042 3.84728 4.43781 1.34176 3.49290 1.89866 3.23881 3.94049 4.30608 3.86681 3.71057 3.33382 3.19357 1.87474 5.10472 3.81929 1019 - -

2.68618 4.42225 2.77519 2.73123 3.46354 2.40513 3.72494 3.29354 2.67741 2.69355 4.24690 2.90347 2.73739 3.18146 2.89801 2.37887 2.77519 2.98518 4.58477 3.61503

0.03782 3.68971 4.41206 0.61958 0.77255 0.64191 0.74715

727 2.58408 4.55777 2.63767 2.50951 3.89313 3.06999 3.79869 3.57286 2.69204 3.24473 4.20914 1.53216 3.71772 3.07984 3.03279 2.66399 2.96516 3.21422 5.22244 3.86110 1020 - -

2.68618 4.42225 2.77519 2.73123 3.46354 2.40513 3.72494 3.29354 2.67741 2.69355 4.24690 2.90347 2.73739 3.18146 2.89801 2.37887 2.77519 2.98518 4.58477 3.61503

0.03782 3.68971 4.41206 0.61958 0.77255 0.64191 0.74715

728 2.24189 4.15412 3.06133 2.87814 3.89444 2.88796 3.98828 3.39642 2.93811 3.13522 4.08514 3.12721 3.61351 3.30518 3.22334 1.40697 2.70989 2.97363 5.28456 3.97893 1021 - -

2.68618 4.42225 2.77519 2.73123 3.46354 2.40513 3.72494 3.29354 2.67741 2.69355 4.24690 2.90347 2.73739 3.18146 2.89801 2.37887 2.77519 2.98518 4.58477 3.61503

0.03782 3.68971 4.41206 0.61958 0.77255 0.64191 0.74715

729 2.33123 4.17207 3.27908 3.03470 3.81909 3.01404 4.06407 2.92517 2.97384 2.79402 3.86593 3.26778 3.70693 3.38105 3.23550 2.53154 1.52801 2.64069 5.27454 4.03869 1022 - -

2.68618 4.42225 2.77519 2.73123 3.46354 2.40513 3.72494 3.29354 2.67741 2.69355 4.24690 2.90347 2.73739 3.18146 2.89801 2.37887 2.77519 2.98518 4.58477 3.61503

0.03782 3.68971 4.41206 0.61958 0.77255 0.64191 0.74715

730 2.24189 4.15412 3.06133 2.87814 3.89444 2.88796 3.98828 3.39642 2.93811 3.13522 4.08514 3.12721 3.61351 3.30518 3.22334 1.40697 2.70989 2.97363 5.28456 3.97893 1023 - -

2.68618 4.42225 2.77519 2.73123 3.46354 2.40513 3.72494 3.29354 2.67741 2.69355 4.24690 2.90347 2.73739 3.18146 2.89801 2.37887 2.77519 2.98518 4.58477 3.61503

0.03782 3.68971 4.41206 0.61958 0.77255 0.64191 0.74715

731 2.88652 4.37019 4.05193 3.66978 3.26042 3.84728 4.43781 1.34176 3.49290 1.89866 3.23881 3.94049 4.30608 3.86681 3.71057 3.33382 3.19357 1.87474 5.10472 3.81929 1024 - -

2.68618 4.42225 2.77519 2.73123 3.46354 2.40513 3.72494 3.29354 2.67741 2.69355 4.24690 2.90347 2.73739 3.18146 2.89801 2.37887 2.77519 2.98518 4.58477 3.61503

0.03782 3.68971 4.41206 0.61958 0.77255 0.64191 0.74715

732 2.88652 4.37019 4.05193 3.66978 3.26042 3.84728 4.43781 1.34176 3.49290 1.89866 3.23881 3.94049 4.30608 3.86681 3.71057 3.33382 3.19357 1.87474 5.10472 3.81929 1025 - -

2.68618 4.42225 2.77519 2.73123 3.46354 2.40513 3.72494 3.29354 2.67741 2.69355 4.24690 2.90347 2.73739 3.18146 2.89801 2.37887 2.77519 2.98518 4.58477 3.61503

0.03782 3.68971 4.41206 0.61958 0.77255 0.64191 0.74715

733 2.53919 4.31574 3.12837 2.94944 3.94108 3.01406 4.03464 3.44720 2.99214 3.09147 4.14117 3.26964 1.25262 3.38748 3.25050 2.70343 2.96896 3.11334 5.17040 4.06284 1026 - -

2.68618 4.42225 2.77519 2.73123 3.46354 2.40513 3.72494 3.29354 2.67741 2.69355 4.24690 2.90347 2.73739 3.18146 2.89801 2.37887 2.77519 2.98518 4.58477 3.61503

0.03782 3.68971 4.41206 0.61958 0.77255 0.64191 0.74715

734 2.24189 4.15412 3.06133 2.87814 3.89444 2.88796 3.98828 3.39642 2.93811 3.13522 4.08514 3.12721 3.61351 3.30518 3.22334 1.40697 2.70989 2.97363 5.28456 3.97893 1027 - -

2.68618 4.42225 2.77519 2.73123 3.46354 2.40513 3.72494 3.29354 2.67741 2.69355 4.24690 2.90347 2.73739 3.18146 2.89801 2.37887 2.77519 2.98518 4.58477 3.61503

0.03782 3.68971 4.41206 0.61958 0.77255 0.64191 0.74715

735 2.82202 4.65647 2.97238 2.72383 3.17446 3.30735 1.59825 3.49438 2.57333 3.00834 4.04492 3.13670 3.85542 3.09072 2.84526 2.89735 3.12340 3.23414 4.64084 3.13275 1028 - -

2.68618 4.42225 2.77519 2.73123 3.46354 2.40513 3.72494 3.29354 2.67741 2.69355 4.24690 2.90347 2.73739 3.18146 2.89801 2.37887 2.77519 2.98518 4.58477 3.61503

0.03782 3.68971 4.41206 0.61958 0.77255 0.64191 0.74715

736 2.95570 4.47801 3.88699 3.53725 3.08153 3.73572 4.25048 2.29059 3.30641 1.14168 3.18203 3.82854 4.21045 3.71971 3.50815 3.30044 3.26294 2.27804 4.88351 3.56168 1029 - -

2.68618 4.42225 2.77519 2.73123 3.46354 2.40513 3.72494 3.29354 2.67741 2.69355 4.24690 2.90347 2.73739 3.18146 2.89801 2.37887 2.77519 2.98518 4.58477 3.61503

0.03782 3.68971 4.41206 0.61958 0.77255 0.64191 0.74715

737 2.99964 4.56315 3.57279 3.27716 2.34326 3.61999 3.56249 3.03542 3.13970 2.53489 3.73362 3.50967 4.10335 3.49096 3.35748 3.15189 3.28846 2.89883 3.97671 1.33703 1030 - -

2.68618 4.42225 2.77519 2.73123 3.46354 2.40513 3.72494 3.29354 2.67741 2.69355 4.24690 2.90347 2.73739 3.18146 2.89801 2.37887 2.77519 2.98518 4.58477 3.61503

0.03782 3.68971 4.41206 0.61958 0.77255 0.64191 0.74715

738 2.95570 4.47801 3.88699 3.53725 3.08153 3.73572 4.25048 2.29059 3.30641 1.14168 3.18203 3.82854 4.21045 3.71971 3.50815 3.30044 3.26294 2.27804 4.88351 3.56168 1031 - -

2.68618 4.42225 2.77519 2.73123 3.46354 2.40513 3.72494 3.29354 2.67741 2.69355 4.24690 2.90347 2.73739 3.18146 2.89801 2.37887 2.77519 2.98518 4.58477 3.61503

0.03782 3.68971 4.41206 0.61958 0.77255 0.64191 0.74715

739 2.80535 4.87482 1.22337 2.25740 4.23128 3.08281 3.79410 3.78082 2.84781 3.42775 4.41212 2.79951 3.72433 3.04114 3.32941 2.79671 3.14408 3.44355 5.43550 4.14798 1032 - -

2.68618 4.42225 2.77519 2.73123 3.46354 2.40513 3.72494 3.29354 2.67741 2.69355 4.24690 2.90347 2.73739 3.18146 2.89801 2.37887 2.77519 2.98518 4.58477 3.61503

0.03782 3.68971 4.41206 0.61958 0.77255 0.64191 0.74715

740 2.88652 4.37019 4.05193 3.66978 3.26042 3.84728 4.43781 1.34176 3.49290 1.89866 3.23881 3.94049 4.30608 3.86681 3.71057 3.33382 3.19357 1.87474 5.10472 3.81929 1033 - -

2.68618 4.42225 2.77519 2.73123 3.46354 2.40513 3.72494 3.29354 2.67741 2.69355 4.24690 2.90347 2.73739 3.18146 2.89801 2.37887 2.77519 2.98518 4.58477 3.61503

0.03782 3.68971 4.41206 0.61958 0.77255 0.64191 0.74715

741 3.03627 4.49697 3.98008 3.66865 1.36975 3.75169 3.82000 2.59321 3.58195 2.01239 3.35868 3.81567 4.22222 3.80445 3.74700 3.31035 3.34138 2.56812 4.14110 2.53835 1034 - -
2.68618 4.42225 2.77519 2.73123 3.46354 2.40513 3.72494 3.29354 2.67741 2.69355 4.24690 2.90347 2.73739 3.18146 2.89801 2.37887 2.77519 2.98518 4.58477 3.61503
0.03782 3.68971 4.41206 0.61958 0.77255 0.64191 0.74715
742 2.82202 4.65647 2.97238 2.72383 3.17446 3.30735 1.59825 3.49438 2.57333 3.00834 4.04492 3.13670 3.85542 3.09072 2.84526 2.89735 3.12340 3.23414 4.64084 3.13275 1035 - -
2.68618 4.42225 2.77519 2.73123 3.46354 2.40513 3.72494 3.29354 2.67741 2.69355 4.24690 2.90347 2.73739 3.18146 2.89801 2.37887 2.77519 2.98518 4.58477 3.61503
0.03782 3.68971 4.41206 0.61958 0.77255 0.64191 0.74715
743 2.80535 4.87482 1.22337 2.25740 4.23128 3.08281 3.79410 3.78082 2.84781 3.42775 4.41212 2.79951 3.72433 3.04114 3.32941 2.79671 3.14408 3.44355 5.43550 4.14798 1036 - -
2.68618 4.42225 2.77519 2.73123 3.46354 2.40513 3.72494 3.29354 2.67741 2.69355 4.24690 2.90347 2.73739 3.18146 2.89801 2.37887 2.77519 2.98518 4.58477 3.61503
0.03782 3.68971 4.41206 0.61958 0.77255 0.64191 0.74715
744 2.24189 4.15412 3.06133 2.87814 3.89444 2.88796 3.98828 3.39642 2.93811 3.13522 4.08514 3.12721 3.61351 3.30518 3.22334 1.40697 2.70989 2.97363 5.28456 3.97893 1037 - -
2.68618 4.42225 2.77519 2.73123 3.46354 2.40513 3.72494 3.29354 2.67741 2.69355 4.24690 2.90347 2.73739 3.18146 2.89801 2.37887 2.77519 2.98518 4.58477 3.61503
0.03782 3.68971 4.41206 0.61958 0.77255 0.64191 0.74715
745 2.81190 4.64511 3.28512 2.84967 3.95281 3.32202 3.71995 3.48986 2.20826 3.04039 4.05567 3.21864 3.84267 2.95856 1.30366 2.91066 3.09663 3.22431 5.11666 3.92964 1038 - -
2.68618 4.42225 2.77519 2.73123 3.46354 2.40513 3.72494 3.29354 2.67741 2.69355 4.24690 2.90347 2.73739 3.18146 2.89801 2.37887 2.77519 2.98518 4.58477 3.61503
0.03782 3.68971 4.41206 0.61958 0.77255 0.64191 0.74715
746 2.80535 4.87482 1.22337 2.25740 4.23128 3.08281 3.79410 3.78082 2.84781 3.42775 4.41212 2.79951 3.72433 3.04114 3.32941 2.79671 3.14408 3.44355 5.43550 4.14798 1039 - -
2.68618 4.42225 2.77519 2.73123 3.46354 2.40513 3.72494 3.29354 2.67741 2.69355 4.24690 2.90347 2.73739 3.18146 2.89801 2.37887 2.77519 2.98518 4.58477 3.61503
0.03782 3.68971 4.41206 0.61958 0.77255 0.64191 0.74715
747 2.82202 4.65647 2.97238 2.72383 3.17446 3.30735 1.59825 3.49438 2.57333 3.00834 4.04492 3.13670 3.85542 3.09072 2.84526 2.89735 3.12340 3.23414 4.64084 3.13275 1040 - -
2.68618 4.42225 2.77519 2.73123 3.46354 2.40513 3.72494 3.29354 2.67741 2.69355 4.24690 2.90347 2.73739 3.18146 2.89801 2.37887 2.77519 2.98518 4.58477 3.61503
0.03782 3.68971 4.41206 0.61958 0.77255 0.64191 0.74715
748 2.80535 4.87482 1.22337 2.25740 4.23128 3.08281 3.79410 3.78082 2.84781 3.42775 4.41212 2.79951 3.72433 3.04114 3.32941 2.79671 3.14408 3.44355 5.43550 4.14798 1041 - -
2.68618 4.42225 2.77519 2.73123 3.46354 2.40513 3.72494 3.29354 2.67741 2.69355 4.24690 2.90347 2.73739 3.18146 2.89801 2.37887 2.77519 2.98518 4.58477 3.61503
0.03782 3.68971 4.41206 0.61958 0.77255 0.64191 0.74715
749 2.81419 4.75052 2.97802 2.64754 4.09431 3.31300 3.68190 3.51431 1.32517 3.12542 4.10705 3.07652 3.83488 2.89173 2.43430 2.87431 3.08699 3.23582 5.23321 4.02251 1042 - -
2.68618 4.42225 2.77519 2.73123 3.46354 2.40513 3.72494 3.29354 2.67741 2.69355 4.24690 2.90347 2.73739 3.18146 2.89801 2.37887 2.77519 2.98518 4.58477 3.61503
0.03782 3.68971 4.41206 0.61958 0.77255 0.64191 0.74715
750 2.81419 4.75052 2.97802 2.64754 4.09431 3.31300 3.68190 3.51431 1.32517 3.12542 4.10705 3.07652 3.83488 2.89173 2.43430 2.87431 3.08699 3.23582 5.23321 4.02251 1043 - -
2.68618 4.42225 2.77519 2.73123 3.46354 2.40513 3.72494 3.29354 2.67741 2.69355 4.24690 2.90347 2.73739 3.18146 2.89801 2.37887 2.77519 2.98518 4.58477 3.61503
0.03782 3.68971 4.41206 0.61958 0.77255 0.64191 0.74715
751 3.03627 4.49697 3.98008 3.66865 1.36975 3.75169 3.82000 2.59321 3.58195 2.01239 3.35868 3.81567 4.22222 3.80445 3.74700 3.31035 3.34138 2.56812 4.14110 2.53835 1044 - -
2.68618 4.42225 2.77519 2.73123 3.46354 2.40513 3.72494 3.29354 2.67741 2.69355 4.24690 2.90347 2.73739 3.18146 2.89801 2.37887 2.77519 2.98518 4.58477 3.61503
0.03782 3.68971 4.41206 0.61958 0.77255 0.64191 0.74715
752 3.03627 4.49697 3.98008 3.66865 1.36975 3.75169 3.82000 2.59321 3.58195 2.01239 3.35868 3.81567 4.22222 3.80445 3.74700 3.31035 3.34138 2.56812 4.14110 2.53835 1045 - -
2.68618 4.42225 2.77519 2.73123 3.46354 2.40513 3.72494 3.29354 2.67741 2.69355 4.24690 2.90347 2.73739 3.18146 2.89801 2.37887 2.77519 2.98518 4.58477 3.61503

0.03782 3.68971 4.41206 0.61958 0.77255 0.64191 0.74715

753 2.40719 1.40547 3.87078 3.57616 3.61829 3.05978 4.26483 2.73897 3.40375 2.65893 3.77889 3.61209 3.75249 3.75916 3.55770 2.67680 2.86014 2.49438 5.06450 3.90470 1046 - -

2.68618 4.42225 2.77519 2.73123 3.46354 2.40513 3.72494 3.29354 2.67741 2.69355 4.24690 2.90347 2.73739 3.18146 2.89801 2.37887 2.77519 2.98518 4.58477 3.61503

0.03782 3.68971 4.41206 0.61958 0.77255 0.64191 0.74715

754 2.80936 4.85412 2.44285 1.32179 4.18024 3.16567 3.75082 3.59744 2.60890 3.24971 4.24291 2.85883 3.76479 2.97779 2.98976 2.81828 3.11612 3.30456 5.38821 4.12184 1047 - -

2.68618 4.42225 2.77519 2.73123 3.46354 2.40513 3.72494 3.29354 2.67741 2.69355 4.24690 2.90347 2.73739 3.18146 2.89801 2.37887 2.77519 2.98518 4.58477 3.61503

0.03782 3.68971 4.41206 0.61958 0.77255 0.64191 0.74715

755 2.80936 4.85412 2.44285 1.32179 4.18024 3.16567 3.75082 3.59744 2.60890 3.24971 4.24291 2.85883 3.76479 2.97779 2.98976 2.81828 3.11612 3.30456 5.38821 4.12184 1048 - -

2.68618 4.42225 2.77519 2.73123 3.46354 2.40513 3.72494 3.29354 2.67741 2.69355 4.24690 2.90347 2.73739 3.18146 2.89801 2.37887 2.77519 2.98518 4.58477 3.61503

0.03782 3.68971 4.41206 0.61958 0.77255 0.64191 0.74715

756 2.80535 4.87482 1.22337 2.25740 4.23128 3.08281 3.79410 3.78082 2.84781 3.42775 4.41212 2.79951 3.72433 3.04114 3.32941 2.79671 3.14408 3.44355 5.43550 4.14798 1049 - -

2.68618 4.42225 2.77519 2.73123 3.46354 2.40513 3.72494 3.29354 2.67741 2.69355 4.24690 2.90347 2.73739 3.18146 2.89801 2.37887 2.77519 2.98518 4.58477 3.61503

0.03782 3.68971 4.41206 0.61958 0.77255 0.64191 0.74715

757 2.75145 4.71252 2.80109 2.56401 3.85261 3.25189 3.71665 3.46461 2.40147 2.98463 4.02911 3.01766 3.80997 1.60421 2.68491 2.81243 3.05413 3.20938 5.16412 3.84799 1050 - -

2.68618 4.42225 2.77519 2.73123 3.46354 2.40513 3.72494 3.29354 2.67741 2.69355 4.24690 2.90347 2.73739 3.18146 2.89801 2.37887 2.77519 2.98518 4.58477 3.61503

0.03782 3.68971 4.41206 0.61958 0.77255 0.64191 0.74715

758 1.31618 4.11764 3.28684 3.08071 3.87759 2.94439 4.12282 2.97833 3.09199 2.86583 3.92805 3.27364 3.66709 3.45348 3.34929 2.47460 2.73956 2.66635 5.32423 4.10541 1051 - -

2.68618 4.42225 2.77519 2.73123 3.46354 2.40513 3.72494 3.29354 2.67741 2.69355 4.24690 2.90347 2.73739 3.18146 2.89801 2.37887 2.77519 2.98518 4.58477 3.61503

0.03782 3.68971 4.41206 0.61958 0.77255 0.64191 0.74715

759 3.03627 4.49697 3.98008 3.66865 1.36975 3.75169 3.82000 2.59321 3.58195 2.01239 3.35868 3.81567 4.22222 3.80445 3.74700 3.31035 3.34138 2.56812 4.14110 2.53835 1052 - -

2.68618 4.42225 2.77519 2.73123 3.46354 2.40513 3.72494 3.29354 2.67741 2.69355 4.24690 2.90347 2.73739 3.18146 2.89801 2.37887 2.77519 2.98518 4.58477 3.61503

0.03782 3.68971 4.41206 0.61958 0.77255 0.64191 0.74715

760 2.95570 4.47801 3.88699 3.53725 3.08153 3.73572 4.25048 2.29059 3.30641 1.14168 3.18203 3.82854 4.21045 3.71971 3.50815 3.30044 3.26294 2.27804 4.88351 3.56168 1053 - -

2.68618 4.42225 2.77519 2.73123 3.46354 2.40513 3.72494 3.29354 2.67741 2.69355 4.24690 2.90347 2.73739 3.18146 2.89801 2.37887 2.77519 2.98518 4.58477 3.61503

0.03782 3.68971 4.41206 0.61958 0.77255 0.64191 0.74715

761 2.80535 4.87482 1.22337 2.25740 4.23128 3.08281 3.79410 3.78082 2.84781 3.42775 4.41212 2.79951 3.72433 3.04114 3.32941 2.79671 3.14408 3.44355 5.43550 4.14798 1054 - -

2.68618 4.42225 2.77519 2.73123 3.46354 2.40513 3.72494 3.29354 2.67741 2.69355 4.24690 2.90347 2.73739 3.18146 2.89801 2.37887 2.77519 2.98518 4.58477 3.61503

0.03782 3.68971 4.41206 0.61958 0.77255 0.64191 0.74715

762 2.81419 4.75052 2.97802 2.64754 4.09431 3.31300 3.68190 3.51431 1.32517 3.12542 4.10705 3.07652 3.83488 2.89173 2.43430 2.87431 3.08699 3.23582 5.23321 4.02251 1055 - -

2.68618 4.42225 2.77519 2.73123 3.46354 2.40513 3.72494 3.29354 2.67741 2.69355 4.24690 2.90347 2.73739 3.18146 2.89801 2.37887 2.77519 2.98518 4.58477 3.61503

0.03782 3.68971 4.41206 0.61958 0.77255 0.64191 0.74715

763 2.66384 4.26718 3.86051 3.52869 3.42541 3.52084 4.36452 2.09728 3.39277 2.15394 3.43086 3.74448 4.09895 3.77068 3.62046 3.02131 3.03475 1.29720 5.17454 3.88959 1056 - -

2.68618 4.42225 2.77519 2.73123 3.46354 2.40513 3.72494 3.29354 2.67741 2.69355 4.24690 2.90347 2.73739 3.18146 2.89801 2.37887 2.77519 2.98518 4.58477 3.61503

0.03782 3.68971 4.41206 0.61958 0.77255 0.64191 0.74715

764 2.33123 4.17207 3.27908 3.03470 3.81909 3.01404 4.06407 2.92517 2.97384 2.79402 3.86593 3.26778 3.70693 3.38105 3.23550 2.53154 1.52801 2.64069 5.27454 4.03869 1057 - -

2.68618 4.42225 2.77519 2.73123 3.46354 2.40513 3.72494 3.29354 2.67741 2.69355 4.24690 2.90347 2.73739 3.18146 2.89801 2.37887 2.77519 2.98518 4.58477 3.61503
0.03782 3.68971 4.41206 0.61958 0.77255 0.64191 0.74715
765 3.06047 4.49594 3.82876 3.49619 2.52194 3.53862 3.70765 3.08393 3.22453 2.50664 3.72585 3.68429 4.04410 3.62345 3.38267 3.26521 3.35391 2.96950 1.35528 2.53548 1058 - -
2.68618 4.42225 2.77519 2.73123 3.46354 2.40513 3.72494 3.29354 2.67741 2.69355 4.24690 2.90347 2.73739 3.18146 2.89801 2.37887 2.77519 2.98518 4.58477 3.61503
0.03782 3.68971 4.41206 0.61958 0.77255 0.64191 0.74715
766 2.95570 4.47801 3.88699 3.53725 3.08153 3.73572 4.25048 2.29059 3.30641 1.14168 3.18203 3.82854 4.21045 3.71971 3.50815 3.30044 3.26294 2.27804 4.88351 3.56168 1059 - -
2.68618 4.42225 2.77519 2.73123 3.46354 2.40513 3.72494 3.29354 2.67741 2.69355 4.24690 2.90347 2.73739 3.18146 2.89801 2.37887 2.77519 2.98518 4.58477 3.61503
0.03782 3.68971 4.41206 0.61958 0.77255 0.64191 0.74715
767 2.66384 4.26718 3.86051 3.52869 3.42541 3.52084 4.36452 2.09728 3.39277 2.15394 3.43086 3.74448 4.09895 3.77068 3.62046 3.02131 3.03475 1.29720 5.17454 3.88959 1060 - -
2.68618 4.42225 2.77519 2.73123 3.46354 2.40513 3.72494 3.29354 2.67741 2.69355 4.24690 2.90347 2.73739 3.18146 2.89801 2.37887 2.77519 2.98518 4.58477 3.61503
0.03782 3.68971 4.41206 0.61958 0.77255 0.64191 0.74715
768 2.66384 4.26718 3.86051 3.52869 3.42541 3.52084 4.36452 2.09728 3.39277 2.15394 3.43086 3.74448 4.09895 3.77068 3.62046 3.02131 3.03475 1.29720 5.17454 3.88959 1061 - -
2.68618 4.42225 2.77519 2.73123 3.46354 2.40513 3.72494 3.29354 2.67741 2.69355 4.24690 2.90347 2.73739 3.18146 2.89801 2.37887 2.77519 2.98518 4.58477 3.61503
0.03782 3.68971 4.41206 0.61958 0.77255 0.64191 0.74715
769 2.81419 4.75052 2.97802 2.64754 4.09431 3.31300 3.68190 3.51431 1.32517 3.12542 4.10705 3.07652 3.83488 2.89173 2.43430 2.87431 3.08699 3.23582 5.23321 4.02251 1062 - -
2.68618 4.42225 2.77519 2.73123 3.46354 2.40513 3.72494 3.29354 2.67741 2.69355 4.24690 2.90347 2.73739 3.18146 2.89801 2.37887 2.77519 2.98518 4.58477 3.61503
0.03782 3.68971 4.41206 0.61958 0.77255 0.64191 0.74715
770 2.24189 4.15412 3.06133 2.87814 3.89444 2.88796 3.98828 3.39642 2.93811 3.13522 4.08514 3.12721 3.61351 3.30518 3.22334 1.40697 2.70989 2.97363 5.28456 3.97893 1063 - -
2.68618 4.42225 2.77519 2.73123 3.46354 2.40513 3.72494 3.29354 2.67741 2.69355 4.24690 2.90347 2.73739 3.18146 2.89801 2.37887 2.77519 2.98518 4.58477 3.61503
0.03782 3.68971 4.41206 0.61958 0.77255 0.64191 0.74715
771 2.58408 4.55777 2.63767 2.50951 3.89313 3.06999 3.79869 3.57286 2.69204 3.24473 4.20914 1.53216 3.71772 3.07984 3.03279 2.66399 2.96516 3.21422 5.22244 3.86110 1064 - -
2.68618 4.42225 2.77519 2.73123 3.46354 2.40513 3.72494 3.29354 2.67741 2.69355 4.24690 2.90347 2.73739 3.18146 2.89801 2.37887 2.77519 2.98518 4.58477 3.61503
0.03782 3.68971 4.41206 0.61958 0.77255 0.64191 0.74715
772 2.95570 4.47801 3.88699 3.53725 3.08153 3.73572 4.25048 2.29059 3.30641 1.14168 3.18203 3.82854 4.21045 3.71971 3.50815 3.30044 3.26294 2.27804 4.88351 3.56168 1065 - -
2.68618 4.42225 2.77519 2.73123 3.46354 2.40513 3.72494 3.29354 2.67741 2.69355 4.24690 2.90347 2.73739 3.18146 2.89801 2.37887 2.77519 2.98518 4.58477 3.61503
0.03782 3.68971 4.41206 0.61958 0.77255 0.64191 0.74715
773 2.99964 4.56315 3.57279 3.27716 2.34326 3.61999 3.56249 3.03542 3.13970 2.53489 3.73362 3.50967 4.10335 3.49096 3.35748 3.15189 3.28846 2.89883 3.97671 1.33703 1066 - -
2.68618 4.42225 2.77519 2.73123 3.46354 2.40513 3.72494 3.29354 2.67741 2.69355 4.24690 2.90347 2.73739 3.18146 2.89801 2.37887 2.77519 2.98518 4.58477 3.61503
0.03782 3.68971 4.41206 0.61958 0.77255 0.64191 0.74715
774 3.03627 4.49697 3.98008 3.66865 1.36975 3.75169 3.82000 2.59321 3.58195 2.01239 3.35868 3.81567 4.22222 3.80445 3.74700 3.31035 3.34138 2.56812 4.14110 2.53835 1067 - -
2.68618 4.42225 2.77519 2.73123 3.46354 2.40513 3.72494 3.29354 2.67741 2.69355 4.24690 2.90347 2.73739 3.18146 2.89801 2.37887 2.77519 2.98518 4.58477 3.61503
0.03782 3.68971 4.41206 0.61958 0.77255 0.64191 0.74715
775 2.66384 4.26718 3.86051 3.52869 3.42541 3.52084 4.36452 2.09728 3.39277 2.15394 3.43086 3.74448 4.09895 3.77068 3.62046 3.02131 3.03475 1.29720 5.17454 3.88959 1068 - -
2.68618 4.42225 2.77519 2.73123 3.46354 2.40513 3.72494 3.29354 2.67741 2.69355 4.24690 2.90347 2.73739 3.18146 2.89801 2.37887 2.77519 2.98518 4.58477 3.61503
0.03782 3.68971 4.41206 0.61958 0.77255 0.64191 0.74715

776 2.53919 4.31574 3.12837 2.94944 3.94108 3.01406 4.03464 3.44720 2.99214 3.09147 4.14117 3.26964 1.25262 3.38748 3.25050 2.70343 2.96896 3.11334 5.17040 4.06284 1069 - -
2.68618 4.42225 2.77519 2.73123 3.46354 2.40513 3.72494 3.29354 2.67741 2.69355 4.24690 2.90347 2.73739 3.18146 2.89801 2.37887 2.77519 2.98518 4.58477 3.61503
0.03782 3.68971 4.41206 0.61958 0.77255 0.64191 0.74715
777 2.24189 4.15412 3.06133 2.87814 3.89444 2.88796 3.98828 3.39642 2.93811 3.13522 4.08514 3.12721 3.61351 3.30518 3.22334 1.40697 2.70989 2.97363 5.28456 3.97893 1070 - -
2.68618 4.42225 2.77519 2.73123 3.46354 2.40513 3.72494 3.29354 2.67741 2.69355 4.24690 2.90347 2.73739 3.18146 2.89801 2.37887 2.77519 2.98518 4.58477 3.61503
0.03782 3.68971 4.41206 0.61958 0.77255 0.64191 0.74715
778 2.95570 4.47801 3.88699 3.53725 3.08153 3.73572 4.25048 2.29059 3.30641 1.14168 3.18203 3.82854 4.21045 3.71971 3.50815 3.30044 3.26294 2.27804 4.88351 3.56168 1071 - -
2.68618 4.42225 2.77519 2.73123 3.46354 2.40513 3.72494 3.29354 2.67741 2.69355 4.24690 2.90347 2.73739 3.18146 2.89801 2.37887 2.77519 2.98518 4.58477 3.61503
0.03782 3.68971 4.41206 0.61958 0.77255 0.64191 0.74715
779 3.06047 4.49594 3.82876 3.49619 2.52194 3.53862 3.70765 3.08393 3.22453 2.50664 3.72585 3.68429 4.04410 3.62345 3.38267 3.26521 3.35391 2.96950 1.35528 2.53548 1072 - -
2.68618 4.42225 2.77519 2.73123 3.46354 2.40513 3.72494 3.29354 2.67741 2.69355 4.24690 2.90347 2.73739 3.18146 2.89801 2.37887 2.77519 2.98518 4.58477 3.61503
0.03782 3.68971 4.41206 0.61958 0.77255 0.64191 0.74715
780 2.83528 4.41805 3.85371 3.44211 3.14741 3.69369 4.22020 2.30593 3.21644 1.77982 1.78422 3.73947 4.16154 3.61600 3.44155 3.16108 3.14135 2.28792 4.95809 3.69770 1073 - -
2.68618 4.42225 2.77519 2.73123 3.46354 2.40513 3.72494 3.29354 2.67741 2.69355 4.24690 2.90347 2.73739 3.18146 2.89801 2.37887 2.77519 2.98518 4.58477 3.61503
0.03782 3.68971 4.41206 0.61958 0.77255 0.64191 0.74715
781 2.88652 4.37019 4.05193 3.66978 3.26042 3.84728 4.43781 1.34176 3.49290 1.89866 3.23881 3.94049 4.30608 3.86681 3.71057 3.33382 3.19357 1.87474 5.10472 3.81929 1074 - -
2.68618 4.42225 2.77519 2.73123 3.46354 2.40513 3.72494 3.29354 2.67741 2.69355 4.24690 2.90347 2.73739 3.18146 2.89801 2.37887 2.77519 2.98518 4.58477 3.61503
0.03782 3.68971 4.41206 0.61958 0.77255 0.64191 0.74715
782 2.53919 4.31574 3.12837 2.94944 3.94108 3.01406 4.03464 3.44720 2.99214 3.09147 4.14117 3.26964 1.25262 3.38748 3.25050 2.70343 2.96896 3.11334 5.17040 4.06284 1075 - -
2.68618 4.42225 2.77519 2.73123 3.46354 2.40513 3.72494 3.29354 2.67741 2.69355 4.24690 2.90347 2.73739 3.18146 2.89801 2.37887 2.77519 2.98518 4.58477 3.61503
0.03782 3.68971 4.41206 0.61958 0.77255 0.64191 0.74715
783 2.24189 4.15412 3.06133 2.87814 3.89444 2.88796 3.98828 3.39642 2.93811 3.13522 4.08514 3.12721 3.61351 3.30518 3.22334 1.40697 2.70989 2.97363 5.28456 3.97893 1076 - -
2.68618 4.42225 2.77519 2.73123 3.46354 2.40513 3.72494 3.29354 2.67741 2.69355 4.24690 2.90347 2.73739 3.18146 2.89801 2.37887 2.77519 2.98518 4.58477 3.61503
0.03782 3.68971 4.41206 0.61958 0.77255 0.64191 0.74715
784 3.03627 4.49697 3.98008 3.66865 1.36975 3.75169 3.82000 2.59321 3.58195 2.01239 3.35868 3.81567 4.22222 3.80445 3.74700 3.31035 3.34138 2.56812 4.14110 2.53835 1077 - -
2.68618 4.42225 2.77519 2.73123 3.46354 2.40513 3.72494 3.29354 2.67741 2.69355 4.24690 2.90347 2.73739 3.18146 2.89801 2.37887 2.77519 2.98518 4.58477 3.61503
0.03782 3.68971 4.41206 0.61958 0.77255 0.64191 0.74715
785 2.75145 4.71252 2.80109 2.56401 3.85261 3.25189 3.71665 3.46461 2.40147 2.98463 4.02911 3.01766 3.80997 1.60421 2.68491 2.81243 3.05413 3.20938 5.16412 3.84799 1078 - -
2.68618 4.42225 2.77519 2.73123 3.46354 2.40513 3.72494 3.29354 2.67741 2.69355 4.24690 2.90347 2.73739 3.18146 2.89801 2.37887 2.77519 2.98518 4.58477 3.61503
0.03782 3.68971 4.41206 0.61958 0.77255 0.64191 0.74715
786 2.33123 4.17207 3.27908 3.03470 3.81909 3.01404 4.06407 2.92517 2.97384 2.79402 3.86593 3.26778 3.70693 3.38105 3.23550 2.53154 1.52801 2.64069 5.27454 4.03869 1079 - -
2.68618 4.42225 2.77519 2.73123 3.46354 2.40513 3.72494 3.29354 2.67741 2.69355 4.24690 2.90347 2.73739 3.18146 2.89801 2.37887 2.77519 2.98518 4.58477 3.61503
0.03782 3.68971 4.41206 0.61958 0.77255 0.64191 0.74715
787 2.81419 4.75052 2.97802 2.64754 4.09431 3.31300 3.68190 3.51431 1.32517 3.12542 4.10705 3.07652 3.83488 2.89173 2.43430 2.87431 3.08699 3.23582 5.23321 4.02251 1080 - -
2.68618 4.42225 2.77519 2.73123 3.46354 2.40513 3.72494 3.29354 2.67741 2.69355 4.24690 2.90347 2.73739 3.18146 2.89801 2.37887 2.77519 2.98518 4.58477 3.61503

0.03782 3.68971 4.41206 0.61958 0.77255 0.64191 0.74715

788 2.95570 4.47801 3.88699 3.53725 3.08153 3.73572 4.25048 2.29059 3.30641 1.14168 3.18203 3.82854 4.21045 3.71971 3.50815 3.30044 3.26294 2.27804 4.88351 3.56168 1081 - -

2.68618 4.42225 2.77519 2.73123 3.46354 2.40513 3.72494 3.29354 2.67741 2.69355 4.24690 2.90347 2.73739 3.18146 2.89801 2.37887 2.77519 2.98518 4.58477 3.61503

0.03782 3.68971 4.41206 0.61958 0.77255 0.64191 0.74715

789 2.88652 4.37019 4.05193 3.66978 3.26042 3.84728 4.43781 1.34176 3.49290 1.89866 3.23881 3.94049 4.30608 3.86681 3.71057 3.33382 3.19357 1.87474 5.10472 3.81929 1082 - -

2.68618 4.42225 2.77519 2.73123 3.46354 2.40513 3.72494 3.29354 2.67741 2.69355 4.24690 2.90347 2.73739 3.18146 2.89801 2.37887 2.77519 2.98518 4.58477 3.61503

0.03782 3.68971 4.41206 0.61958 0.77255 0.64191 0.74715

790 2.81419 4.75052 2.97802 2.64754 4.09431 3.31300 3.68190 3.51431 1.32517 3.12542 4.10705 3.07652 3.83488 2.89173 2.43430 2.87431 3.08699 3.23582 5.23321 4.02251 1083 - -

2.68618 4.42225 2.77519 2.73123 3.46354 2.40513 3.72494 3.29354 2.67741 2.69355 4.24690 2.90347 2.73739 3.18146 2.89801 2.37887 2.77519 2.98518 4.58477 3.61503

0.03782 3.68971 4.41206 0.61958 0.77255 0.64191 0.74715

791 2.95570 4.47801 3.88699 3.53725 3.08153 3.73572 4.25048 2.29059 3.30641 1.14168 3.18203 3.82854 4.21045 3.71971 3.50815 3.30044 3.26294 2.27804 4.88351 3.56168 1084 - -

2.68618 4.42225 2.77519 2.73123 3.46354 2.40513 3.72494 3.29354 2.67741 2.69355 4.24690 2.90347 2.73739 3.18146 2.89801 2.37887 2.77519 2.98518 4.58477 3.61503

0.03782 3.68971 4.41206 0.61958 0.77255 0.64191 0.74715

792 3.03627 4.49697 3.98008 3.66865 1.36975 3.75169 3.82000 2.59321 3.58195 2.01239 3.35868 3.81567 4.22222 3.80445 3.74700 3.31035 3.34138 2.56812 4.14110 2.53835 1085 - -

2.68618 4.42225 2.77519 2.73123 3.46354 2.40513 3.72494 3.29354 2.67741 2.69355 4.24690 2.90347 2.73739 3.18146 2.89801 2.37887 2.77519 2.98518 4.58477 3.61503

0.03782 3.68971 4.41206 0.61958 0.77255 0.64191 0.74715

793 2.53919 4.31574 3.12837 2.94944 3.94108 3.01406 4.03464 3.44720 2.99214 3.09147 4.14117 3.26964 1.25262 3.38748 3.25050 2.70343 2.96896 3.11334 5.17040 4.06284 1086 - -

2.68618 4.42225 2.77519 2.73123 3.46354 2.40513 3.72494 3.29354 2.67741 2.69355 4.24690 2.90347 2.73739 3.18146 2.89801 2.37887 2.77519 2.98518 4.58477 3.61503

0.03782 3.68971 4.41206 0.61958 0.77255 0.64191 0.74715

794 2.75145 4.71252 2.80109 2.56401 3.85261 3.25189 3.71665 3.46461 2.40147 2.98463 4.02911 3.01766 3.80997 1.60421 2.68491 2.81243 3.05413 3.20938 5.16412 3.84799 1087 - -

2.68618 4.42225 2.77519 2.73123 3.46354 2.40513 3.72494 3.29354 2.67741 2.69355 4.24690 2.90347 2.73739 3.18146 2.89801 2.37887 2.77519 2.98518 4.58477 3.61503

0.03782 3.68971 4.41206 0.61958 0.77255 0.64191 0.74715

795 2.81419 4.75052 2.97802 2.64754 4.09431 3.31300 3.68190 3.51431 1.32517 3.12542 4.10705 3.07652 3.83488 2.89173 2.43430 2.87431 3.08699 3.23582 5.23321 4.02251 1088 - -

2.68618 4.42225 2.77519 2.73123 3.46354 2.40513 3.72494 3.29354 2.67741 2.69355 4.24690 2.90347 2.73739 3.18146 2.89801 2.37887 2.77519 2.98518 4.58477 3.61503

0.03782 3.68971 4.41206 0.61958 0.77255 0.64191 0.74715

796 2.80936 4.85412 2.44285 1.32179 4.18024 3.16567 3.75082 3.59744 2.60890 3.24971 4.24291 2.85883 3.76479 2.97779 2.98976 2.81828 3.11612 3.30456 5.38821 4.12184 1089 - -

2.68618 4.42225 2.77519 2.73123 3.46354 2.40513 3.72494 3.29354 2.67741 2.69355 4.24690 2.90347 2.73739 3.18146 2.89801 2.37887 2.77519 2.98518 4.58477 3.61503

0.03782 3.68971 4.41206 0.61958 0.77255 0.64191 0.74715

797 2.33123 4.17207 3.27908 3.03470 3.81909 3.01404 4.06407 2.92517 2.97384 2.79402 3.86593 3.26778 3.70693 3.38105 3.23550 2.53154 1.52801 2.64069 5.27454 4.03869 1090 - -

2.68618 4.42225 2.77519 2.73123 3.46354 2.40513 3.72494 3.29354 2.67741 2.69355 4.24690 2.90347 2.73739 3.18146 2.89801 2.37887 2.77519 2.98518 4.58477 3.61503

0.03782 3.68971 4.41206 0.61958 0.77255 0.64191 0.74715

798 2.66384 4.26718 3.86051 3.52869 3.42541 3.52084 4.36452 2.09728 3.39277 2.15394 3.43086 3.74448 4.09895 3.77068 3.62046 3.02131 3.03475 1.29720 5.17454 3.88959 1091 - -

2.68618 4.42225 2.77519 2.73123 3.46354 2.40513 3.72494 3.29354 2.67741 2.69355 4.24690 2.90347 2.73739 3.18146 2.89801 2.37887 2.77519 2.98518 4.58477 3.61503

0.03782 3.68971 4.41206 0.61958 0.77255 0.64191 0.74715

799 3.03627 4.49697 3.98008 3.66865 1.36975 3.75169 3.82000 2.59321 3.58195 2.01239 3.35868 3.81567 4.22222 3.80445 3.74700 3.31035 3.34138 2.56812 4.14110 2.53835 1092 - -

2.68618 4.42225 2.77519 2.73123 3.46354 2.40513 3.72494 3.29354 2.67741 2.69355 4.24690 2.90347 2.73739 3.18146 2.89801 2.37887 2.77519 2.98518 4.58477 3.61503
0.03782 3.68971 4.41206 0.61958 0.77255 0.64191 0.74715
800 2.82202 4.65647 2.97238 2.72383 3.17446 3.30735 1.59825 3.49438 2.57333 3.00834 4.04492 3.13670 3.85542 3.09072 2.84526 2.89735 3.12340 3.23414 4.64084 3.13275 1093 - -
2.68618 4.42225 2.77519 2.73123 3.46354 2.40513 3.72494 3.29354 2.67741 2.69355 4.24690 2.90347 2.73739 3.18146 2.89801 2.37887 2.77519 2.98518 4.58477 3.61503
0.03782 3.68971 4.41206 0.61958 0.77255 0.64191 0.74715
801 2.82202 4.65647 2.97238 2.72383 3.17446 3.30735 1.59825 3.49438 2.57333 3.00834 4.04492 3.13670 3.85542 3.09072 2.84526 2.89735 3.12340 3.23414 4.64084 3.13275 1094 - -
2.68618 4.42225 2.77519 2.73123 3.46354 2.40513 3.72494 3.29354 2.67741 2.69355 4.24690 2.90347 2.73739 3.18146 2.89801 2.37887 2.77519 2.98518 4.58477 3.61503
0.03782 3.68971 4.41206 0.61958 0.77255 0.64191 0.74715
802 2.95570 4.47801 3.88699 3.53725 3.08153 3.73572 4.25048 2.29059 3.30641 1.14168 3.18203 3.82854 4.21045 3.71971 3.50815 3.30044 3.26294 2.27804 4.88351 3.56168 1095 - -
2.68618 4.42225 2.77519 2.73123 3.46354 2.40513 3.72494 3.29354 2.67741 2.69355 4.24690 2.90347 2.73739 3.18146 2.89801 2.37887 2.77519 2.98518 4.58477 3.61503
0.03782 3.68971 4.41206 0.61958 0.77255 0.64191 0.74715
803 1.31618 4.11764 3.28684 3.08071 3.87759 2.94439 4.12282 2.97833 3.09199 2.86583 3.92805 3.27364 3.66709 3.45348 3.34929 2.47460 2.73956 2.66635 5.32423 4.10541 1096 - -
2.68618 4.42225 2.77519 2.73123 3.46354 2.40513 3.72494 3.29354 2.67741 2.69355 4.24690 2.90347 2.73739 3.18146 2.89801 2.37887 2.77519 2.98518 4.58477 3.61503
0.03782 3.68971 4.41206 0.61958 0.77255 0.64191 0.74715
804 2.81190 4.64511 3.28512 2.84967 3.95281 3.32202 3.71995 3.48986 2.20826 3.04039 4.05567 3.21864 3.84267 2.95856 1.30366 2.91066 3.09663 3.22431 5.11666 3.92964 1097 - -
2.68618 4.42225 2.77519 2.73123 3.46354 2.40513 3.72494 3.29354 2.67741 2.69355 4.24690 2.90347 2.73739 3.18146 2.89801 2.37887 2.77519 2.98518 4.58477 3.61503
0.03782 3.68971 4.41206 0.61958 0.77255 0.64191 0.74715
805 2.99964 4.56315 3.57279 3.27716 2.34326 3.61999 3.56249 3.03542 3.13970 2.53489 3.73362 3.50967 4.10335 3.49096 3.35748 3.15189 3.28846 2.89883 3.97671 1.33703 1098 - -
2.68618 4.42225 2.77519 2.73123 3.46354 2.40513 3.72494 3.29354 2.67741 2.69355 4.24690 2.90347 2.73739 3.18146 2.89801 2.37887 2.77519 2.98518 4.58477 3.61503
0.03782 3.68971 4.41206 0.61958 0.77255 0.64191 0.74715
806 2.95570 4.47801 3.88699 3.53725 3.08153 3.73572 4.25048 2.29059 3.30641 1.14168 3.18203 3.82854 4.21045 3.71971 3.50815 3.30044 3.26294 2.27804 4.88351 3.56168 1099 - -
2.68618 4.42225 2.77519 2.73123 3.46354 2.40513 3.72494 3.29354 2.67741 2.69355 4.24690 2.90347 2.73739 3.18146 2.89801 2.37887 2.77519 2.98518 4.58477 3.61503
0.03782 3.68971 4.41206 0.61958 0.77255 0.64191 0.74715
807 3.03627 4.49697 3.98008 3.66865 1.36975 3.75169 3.82000 2.59321 3.58195 2.01239 3.35868 3.81567 4.22222 3.80445 3.74700 3.31035 3.34138 2.56812 4.14110 2.53835 1100 - -
2.68618 4.42225 2.77519 2.73123 3.46354 2.40513 3.72494 3.29354 2.67741 2.69355 4.24690 2.90347 2.73739 3.18146 2.89801 2.37887 2.77519 2.98518 4.58477 3.61503
0.03782 3.68971 4.41206 0.61958 0.77255 0.64191 0.74715
808 2.82202 4.65647 2.97238 2.72383 3.17446 3.30735 1.59825 3.49438 2.57333 3.00834 4.04492 3.13670 3.85542 3.09072 2.84526 2.89735 3.12340 3.23414 4.64084 3.13275 1101 - -
2.68618 4.42225 2.77519 2.73123 3.46354 2.40513 3.72494 3.29354 2.67741 2.69355 4.24690 2.90347 2.73739 3.18146 2.89801 2.37887 2.77519 2.98518 4.58477 3.61503
0.03782 3.68971 4.41206 0.61958 0.77255 0.64191 0.74715
809 2.53919 4.31574 3.12837 2.94944 3.94108 3.01406 4.03464 3.44720 2.99214 3.09147 4.14117 3.26964 1.25262 3.38748 3.25050 2.70343 2.96896 3.11334 5.17040 4.06284 1102 - -
2.68618 4.42225 2.77519 2.73123 3.46354 2.40513 3.72494 3.29354 2.67741 2.69355 4.24690 2.90347 2.73739 3.18146 2.89801 2.37887 2.77519 2.98518 4.58477 3.61503
0.03782 3.68971 4.41206 0.61958 0.77255 0.64191 0.74715
810 2.33123 4.17207 3.27908 3.03470 3.81909 3.01404 4.06407 2.92517 2.97384 2.79402 3.86593 3.26778 3.70693 3.38105 3.23550 2.53154 1.52801 2.64069 5.27454 4.03869 1103 - -
2.68618 4.42225 2.77519 2.73123 3.46354 2.40513 3.72494 3.29354 2.67741 2.69355 4.24690 2.90347 2.73739 3.18146 2.89801 2.37887 2.77519 2.98518 4.58477 3.61503
0.03782 3.68971 4.41206 0.61958 0.77255 0.64191 0.74715

811 2.58408 4.55777 2.63767 2.50951 3.89313 3.06999 3.79869 3.57286 2.69204 3.24473 4.20914 1.53216 3.71772 3.07984 3.03279 2.66399 2.96516 3.21422 5.22244 3.86110 1104 - -
2.68618 4.42225 2.77519 2.73123 3.46354 2.40513 3.72494 3.29354 2.67741 2.69355 4.24690 2.90347 2.73739 3.18146 2.89801 2.37887 2.77519 2.98518 4.58477 3.61503
0.03782 3.68971 4.41206 0.61958 0.77255 0.64191 0.74715
812 2.75145 4.71252 2.80109 2.56401 3.85261 3.25189 3.71665 3.46461 2.40147 2.98463 4.02911 3.01766 3.80997 1.60421 2.68491 2.81243 3.05413 3.20938 5.16412 3.84799 1105 - -
2.68618 4.42225 2.77519 2.73123 3.46354 2.40513 3.72494 3.29354 2.67741 2.69355 4.24690 2.90347 2.73739 3.18146 2.89801 2.37887 2.77519 2.98518 4.58477 3.61503
0.03782 3.68971 4.41206 0.61958 0.77255 0.64191 0.74715
813 2.66384 4.26718 3.86051 3.52869 3.42541 3.52084 4.36452 2.09728 3.39277 2.15394 3.43086 3.74448 4.09895 3.77068 3.62046 3.02131 3.03475 1.29720 5.17454 3.88959 1106 - -
2.68618 4.42225 2.77519 2.73123 3.46354 2.40513 3.72494 3.29354 2.67741 2.69355 4.24690 2.90347 2.73739 3.18146 2.89801 2.37887 2.77519 2.98518 4.58477 3.61503
0.03782 3.68971 4.41206 0.61958 0.77255 0.64191 0.74715
814 3.06047 4.49594 3.82876 3.49619 2.52194 3.53862 3.70765 3.08393 3.22453 2.50664 3.72585 3.68429 4.04410 3.62345 3.38267 3.26521 3.35391 2.96950 1.35528 2.53548 1107 - -
2.68618 4.42225 2.77519 2.73123 3.46354 2.40513 3.72494 3.29354 2.67741 2.69355 4.24690 2.90347 2.73739 3.18146 2.89801 2.37887 2.77519 2.98518 4.58477 3.61503
0.03782 3.68971 4.41206 0.61958 0.77255 0.64191 0.74715
815 2.80936 4.85412 2.44285 1.32179 4.18024 3.16567 3.75082 3.59744 2.60890 3.24971 4.24291 2.85883 3.76479 2.97779 2.98976 2.81828 3.11612 3.30456 5.38821 4.12184 1108 - -
2.68618 4.42225 2.77519 2.73123 3.46354 2.40513 3.72494 3.29354 2.67741 2.69355 4.24690 2.90347 2.73739 3.18146 2.89801 2.37887 2.77519 2.98518 4.58477 3.61503
0.03782 3.68971 4.41206 0.61958 0.77255 0.64191 0.74715
816 2.99964 4.56315 3.57279 3.27716 2.34326 3.61999 3.56249 3.03542 3.13970 2.53489 3.73362 3.50967 4.10335 3.49096 3.35748 3.15189 3.28846 2.89883 3.97671 1.33703 1109 - -
2.68618 4.42225 2.77519 2.73123 3.46354 2.40513 3.72494 3.29354 2.67741 2.69355 4.24690 2.90347 2.73739 3.18146 2.89801 2.37887 2.77519 2.98518 4.58477 3.61503
0.03782 3.68971 4.41206 0.61958 0.77255 0.64191 0.74715
817 2.24189 4.15412 3.06133 2.87814 3.89444 2.88796 3.98828 3.39642 2.93811 3.13522 4.08514 3.12721 3.61351 3.30518 3.22334 1.40697 2.70989 2.97363 5.28456 3.97893 1110 - -
2.68618 4.42225 2.77519 2.73123 3.46354 2.40513 3.72494 3.29354 2.67741 2.69355 4.24690 2.90347 2.73739 3.18146 2.89801 2.37887 2.77519 2.98518 4.58477 3.61503
0.03782 3.68971 4.41206 0.61958 0.77255 0.64191 0.74715
818 2.80535 4.87482 1.22337 2.25740 4.23128 3.08281 3.79410 3.78082 2.84781 3.42775 4.41212 2.79951 3.72433 3.04114 3.32941 2.79671 3.14408 3.44355 5.43550 4.14798 1111 - -
2.68618 4.42225 2.77519 2.73123 3.46354 2.40513 3.72494 3.29354 2.67741 2.69355 4.24690 2.90347 2.73739 3.18146 2.89801 2.37887 2.77519 2.98518 4.58477 3.61503
0.03782 3.68971 4.41206 0.61958 0.77255 0.64191 0.74715
819 2.82202 4.65647 2.97238 2.72383 3.17446 3.30735 1.59825 3.49438 2.57333 3.00834 4.04492 3.13670 3.85542 3.09072 2.84526 2.89735 3.12340 3.23414 4.64084 3.13275 1112 - -
2.68618 4.42225 2.77519 2.73123 3.46354 2.40513 3.72494 3.29354 2.67741 2.69355 4.24690 2.90347 2.73739 3.18146 2.89801 2.37887 2.77519 2.98518 4.58477 3.61503
0.03782 3.68971 4.41206 0.61958 0.77255 0.64191 0.74715
820 2.95570 4.47801 3.88699 3.53725 3.08153 3.73572 4.25048 2.29059 3.30641 1.14168 3.18203 3.82854 4.21045 3.71971 3.50815 3.30044 3.26294 2.27804 4.88351 3.56168 1113 - -
2.68618 4.42225 2.77519 2.73123 3.46354 2.40513 3.72494 3.29354 2.67741 2.69355 4.24690 2.90347 2.73739 3.18146 2.89801 2.37887 2.77519 2.98518 4.58477 3.61503
0.03782 3.68971 4.41206 0.61958 0.77255 0.64191 0.74715
821 2.81419 4.75052 2.97802 2.64754 4.09431 3.31300 3.68190 3.51431 1.32517 3.12542 4.10705 3.07652 3.83488 2.89173 2.43430 2.87431 3.08699 3.23582 5.23321 4.02251 1114 - -
2.68618 4.42225 2.77519 2.73123 3.46354 2.40513 3.72494 3.29354 2.67741 2.69355 4.24690 2.90347 2.73739 3.18146 2.89801 2.37887 2.77519 2.98518 4.58477 3.61503
0.03782 3.68971 4.41206 0.61958 0.77255 0.64191 0.74715
822 2.58408 4.55777 2.63767 2.50951 3.89313 3.06999 3.79869 3.57286 2.69204 3.24473 4.20914 1.53216 3.71772 3.07984 3.03279 2.66399 2.96516 3.21422 5.22244 3.86110 1115 - -
2.68618 4.42225 2.77519 2.73123 3.46354 2.40513 3.72494 3.29354 2.67741 2.69355 4.24690 2.90347 2.73739 3.18146 2.89801 2.37887 2.77519 2.98518 4.58477 3.61503

0.03782 3.68971 4.41206 0.61958 0.77255 0.64191 0.74715

823 2.83528 4.41805 3.85371 3.44211 3.14741 3.69369 4.22020 2.30593 3.21644 1.77982 1.78422 3.73947 4.16154 3.61600 3.44155 3.16108 3.14135 2.28792 4.95809 3.69770 1116 - -

2.68618 4.42225 2.77519 2.73123 3.46354 2.40513 3.72494 3.29354 2.67741 2.69355 4.24690 2.90347 2.73739 3.18146 2.89801 2.37887 2.77519 2.98518 4.58477 3.61503

0.03782 3.68971 4.41206 0.61958 0.77255 0.64191 0.74715

824 3.03627 4.49697 3.98008 3.66865 1.36975 3.75169 3.82000 2.59321 3.58195 2.01239 3.35868 3.81567 4.22222 3.80445 3.74700 3.31035 3.34138 2.56812 4.14110 2.53835 1117 - -

2.68618 4.42225 2.77519 2.73123 3.46354 2.40513 3.72494 3.29354 2.67741 2.69355 4.24690 2.90347 2.73739 3.18146 2.89801 2.37887 2.77519 2.98518 4.58477 3.61503

0.03782 3.68971 4.41206 0.61958 0.77255 0.64191 0.74715

825 2.95570 4.47801 3.88699 3.53725 3.08153 3.73572 4.25048 2.29059 3.30641 1.14168 3.18203 3.82854 4.21045 3.71971 3.50815 3.30044 3.26294 2.27804 4.88351 3.56168 1118 - -

2.68618 4.42225 2.77519 2.73123 3.46354 2.40513 3.72494 3.29354 2.67741 2.69355 4.24690 2.90347 2.73739 3.18146 2.89801 2.37887 2.77519 2.98518 4.58477 3.61503

0.03782 3.68971 4.41206 0.61958 0.77255 0.64191 0.74715

826 2.80936 4.85412 2.44285 1.32179 4.18024 3.16567 3.75082 3.59744 2.60890 3.24971 4.24291 2.85883 3.76479 2.97779 2.98976 2.81828 3.11612 3.30456 5.38821 4.12184 1119 - -

2.68618 4.42225 2.77519 2.73123 3.46354 2.40513 3.72494 3.29354 2.67741 2.69355 4.24690 2.90347 2.73739 3.18146 2.89801 2.37887 2.77519 2.98518 4.58477 3.61503

0.03782 3.68971 4.41206 0.61958 0.77255 0.64191 0.74715

827 2.75145 4.71252 2.80109 2.56401 3.85261 3.25189 3.71665 3.46461 2.40147 2.98463 4.02911 3.01766 3.80997 1.60421 2.68491 2.81243 3.05413 3.20938 5.16412 3.84799 1120 - -

2.68618 4.42225 2.77519 2.73123 3.46354 2.40513 3.72494 3.29354 2.67741 2.69355 4.24690 2.90347 2.73739 3.18146 2.89801 2.37887 2.77519 2.98518 4.58477 3.61503

0.03782 3.68971 4.41206 0.61958 0.77255 0.64191 0.74715

828 1.31618 4.11764 3.28684 3.08071 3.87759 2.94439 4.12282 2.97833 3.09199 2.86583 3.92805 3.27364 3.66709 3.45348 3.34929 2.47460 2.73956 2.66635 5.32423 4.10541 1121 - -

2.68618 4.42225 2.77519 2.73123 3.46354 2.40513 3.72494 3.29354 2.67741 2.69355 4.24690 2.90347 2.73739 3.18146 2.89801 2.37887 2.77519 2.98518 4.58477 3.61503

0.03782 3.68971 4.41206 0.61958 0.77255 0.64191 0.74715

829 2.24189 4.15412 3.06133 2.87814 3.89444 2.88796 3.98828 3.39642 2.93811 3.13522 4.08514 3.12721 3.61351 3.30518 3.22334 1.40697 2.70989 2.97363 5.28456 3.97893 1122 - -

2.68618 4.42225 2.77519 2.73123 3.46354 2.40513 3.72494 3.29354 2.67741 2.69355 4.24690 2.90347 2.73739 3.18146 2.89801 2.37887 2.77519 2.98518 4.58477 3.61503

0.03782 3.68971 4.41206 0.61958 0.77255 0.64191 0.74715

830 2.24189 4.15412 3.06133 2.87814 3.89444 2.88796 3.98828 3.39642 2.93811 3.13522 4.08514 3.12721 3.61351 3.30518 3.22334 1.40697 2.70989 2.97363 5.28456 3.97893 1123 - -

2.68618 4.42225 2.77519 2.73123 3.46354 2.40513 3.72494 3.29354 2.67741 2.69355 4.24690 2.90347 2.73739 3.18146 2.89801 2.37887 2.77519 2.98518 4.58477 3.61503

0.03782 3.68971 4.41206 0.61958 0.77255 0.64191 0.74715

831 2.33123 4.17207 3.27908 3.03470 3.81909 3.01404 4.06407 2.92517 2.97384 2.79402 3.86593 3.26778 3.70693 3.38105 3.23550 2.53154 1.52801 2.64069 5.27454 4.03869 1124 - -

2.68618 4.42225 2.77519 2.73123 3.46354 2.40513 3.72494 3.29354 2.67741 2.69355 4.24690 2.90347 2.73739 3.18146 2.89801 2.37887 2.77519 2.98518 4.58477 3.61503

0.03782 3.68971 4.41206 0.61958 0.77255 0.64191 0.74715

832 2.46513 4.24664 3.09956 2.98169 4.10469 1.07816 4.10397 3.66750 3.13599 3.34383 4.32651 3.26662 3.63194 3.47710 3.38330 2.63370 2.93263 3.23311 5.23953 4.20371 1125 - -

2.68618 4.42225 2.77519 2.73123 3.46354 2.40513 3.72494 3.29354 2.67741 2.69355 4.24690 2.90347 2.73739 3.18146 2.89801 2.37887 2.77519 2.98518 4.58477 3.61503

0.03782 3.68971 4.41206 0.61958 0.77255 0.64191 0.74715

833 2.80936 4.85412 2.44285 1.32179 4.18024 3.16567 3.75082 3.59744 2.60890 3.24971 4.24291 2.85883 3.76479 2.97779 2.98976 2.81828 3.11612 3.30456 5.38821 4.12184 1126 - -

2.68618 4.42225 2.77519 2.73123 3.46354 2.40513 3.72494 3.29354 2.67741 2.69355 4.24690 2.90347 2.73739 3.18146 2.89801 2.37887 2.77519 2.98518 4.58477 3.61503

0.03782 3.68971 4.41206 0.61958 0.77255 0.64191 0.74715

834 2.33123 4.17207 3.27908 3.03470 3.81909 3.01404 4.06407 2.92517 2.97384 2.79402 3.86593 3.26778 3.70693 3.38105 3.23550 2.53154 1.52801 2.64069 5.27454 4.03869 1127 - -

2.68618 4.42225 2.77519 2.73123 3.46354 2.40513 3.72494 3.29354 2.67741 2.69355 4.24690 2.90347 2.73739 3.18146 2.89801 2.37887 2.77519 2.98518 4.58477 3.61503
0.03782 3.68971 4.41206 0.61958 0.77255 0.64191 0.74715
835 2.88652 4.37019 4.05193 3.66978 3.26042 3.84728 4.43781 1.34176 3.49290 1.89866 3.23881 3.94049 4.30608 3.86681 3.71057 3.33382 3.19357 1.87474 5.10472 3.81929 1128 - -
2.68618 4.42225 2.77519 2.73123 3.46354 2.40513 3.72494 3.29354 2.67741 2.69355 4.24690 2.90347 2.73739 3.18146 2.89801 2.37887 2.77519 2.98518 4.58477 3.61503
0.03782 3.68971 4.41206 0.61958 0.77255 0.64191 0.74715
836 2.80936 4.85412 2.44285 1.32179 4.18024 3.16567 3.75082 3.59744 2.60890 3.24971 4.24291 2.85883 3.76479 2.97779 2.98976 2.81828 3.11612 3.30456 5.38821 4.12184 1129 - -
2.68618 4.42225 2.77519 2.73123 3.46354 2.40513 3.72494 3.29354 2.67741 2.69355 4.24690 2.90347 2.73739 3.18146 2.89801 2.37887 2.77519 2.98518 4.58477 3.61503
0.03782 3.68971 4.41206 0.61958 0.77255 0.64191 0.74715
837 2.66384 4.26718 3.86051 3.52869 3.42541 3.52084 4.36452 2.09728 3.39277 2.15394 3.43086 3.74448 4.09895 3.77068 3.62046 3.02131 3.03475 1.29720 5.17454 3.88959 1130 - -
2.68618 4.42225 2.77519 2.73123 3.46354 2.40513 3.72494 3.29354 2.67741 2.69355 4.24690 2.90347 2.73739 3.18146 2.89801 2.37887 2.77519 2.98518 4.58477 3.61503
0.03782 3.68971 4.41206 0.61958 0.77255 0.64191 0.74715
838 2.99964 4.56315 3.57279 3.27716 2.34326 3.61999 3.56249 3.03542 3.13970 2.53489 3.73362 3.50967 4.10335 3.49096 3.35748 3.15189 3.28846 2.89883 3.97671 1.33703 1131 - -
2.68618 4.42225 2.77519 2.73123 3.46354 2.40513 3.72494 3.29354 2.67741 2.69355 4.24690 2.90347 2.73739 3.18146 2.89801 2.37887 2.77519 2.98518 4.58477 3.61503
0.03782 3.68971 4.41206 0.61958 0.77255 0.64191 0.74715
839 2.75145 4.71252 2.80109 2.56401 3.85261 3.25189 3.71665 3.46461 2.40147 2.98463 4.02911 3.01766 3.80997 1.60421 2.68491 2.81243 3.05413 3.20938 5.16412 3.84799 1132 - -
2.68618 4.42225 2.77519 2.73123 3.46354 2.40513 3.72494 3.29354 2.67741 2.69355 4.24690 2.90347 2.73739 3.18146 2.89801 2.37887 2.77519 2.98518 4.58477 3.61503
0.03782 3.68971 4.41206 0.61958 0.77255 0.64191 0.74715
840 2.53919 4.31574 3.12837 2.94944 3.94108 3.01406 4.03464 3.44720 2.99214 3.09147 4.14117 3.26964 1.25262 3.38748 3.25050 2.70343 2.96896 3.11334 5.17040 4.06284 1133 - -
2.68618 4.42225 2.77519 2.73123 3.46354 2.40513 3.72494 3.29354 2.67741 2.69355 4.24690 2.90347 2.73739 3.18146 2.89801 2.37887 2.77519 2.98518 4.58477 3.61503
0.03782 3.68971 4.41206 0.61958 0.77255 0.64191 0.74715
841 2.24189 4.15412 3.06133 2.87814 3.89444 2.88796 3.98828 3.39642 2.93811 3.13522 4.08514 3.12721 3.61351 3.30518 3.22334 1.40697 2.70989 2.97363 5.28456 3.97893 1134 - -
2.68618 4.42225 2.77519 2.73123 3.46354 2.40513 3.72494 3.29354 2.67741 2.69355 4.24690 2.90347 2.73739 3.18146 2.89801 2.37887 2.77519 2.98518 4.58477 3.61503
0.03782 3.68971 4.41206 0.61958 0.77255 0.64191 0.74715
842 2.46513 4.24664 3.09956 2.98169 4.10469 1.07816 4.10397 3.66750 3.13599 3.34383 4.32651 3.26662 3.63194 3.47710 3.38330 2.63370 2.93263 3.23311 5.23953 4.20371 1135 - -
2.68618 4.42225 2.77519 2.73123 3.46354 2.40513 3.72494 3.29354 2.67741 2.69355 4.24690 2.90347 2.73739 3.18146 2.89801 2.37887 2.77519 2.98518 4.58477 3.61503
0.03782 3.68971 4.41206 0.61958 0.77255 0.64191 0.74715
843 2.80936 4.85412 2.44285 1.32179 4.18024 3.16567 3.75082 3.59744 2.60890 3.24971 4.24291 2.85883 3.76479 2.97779 2.98976 2.81828 3.11612 3.30456 5.38821 4.12184 1136 - -
2.68618 4.42225 2.77519 2.73123 3.46354 2.40513 3.72494 3.29354 2.67741 2.69355 4.24690 2.90347 2.73739 3.18146 2.89801 2.37887 2.77519 2.98518 4.58477 3.61503
0.03782 3.68971 4.41206 0.61958 0.77255 0.64191 0.74715
844 2.81419 4.75052 2.97802 2.64754 4.09431 3.31300 3.68190 3.51431 1.32517 3.12542 4.10705 3.07652 3.83488 2.89173 2.43430 2.87431 3.08699 3.23582 5.23321 4.02251 1137 - -
2.68618 4.42225 2.77519 2.73123 3.46354 2.40513 3.72494 3.29354 2.67741 2.69355 4.24690 2.90347 2.73739 3.18146 2.89801 2.37887 2.77519 2.98518 4.58477 3.61503
0.03782 3.68971 4.41206 0.61958 0.77255 0.64191 0.74715
845 2.88652 4.37019 4.05193 3.66978 3.26042 3.84728 4.43781 1.34176 3.49290 1.89866 3.23881 3.94049 4.30608 3.86681 3.71057 3.33382 3.19357 1.87474 5.10472 3.81929 1138 - -
2.68618 4.42225 2.77519 2.73123 3.46354 2.40513 3.72494 3.29354 2.67741 2.69355 4.24690 2.90347 2.73739 3.18146 2.89801 2.37887 2.77519 2.98518 4.58477 3.61503
0.03782 3.68971 4.41206 0.61958 0.77255 0.64191 0.74715

846 2.75145 4.71252 2.80109 2.56401 3.85261 3.25189 3.71665 3.46461 2.40147 2.98463 4.02911 3.01766 3.80997 1.60421 2.68491 2.81243 3.05413 3.20938 5.16412 3.84799 1139 - -
2.68618 4.42225 2.77519 2.73123 3.46354 2.40513 3.72494 3.29354 2.67741 2.69355 4.24690 2.90347 2.73739 3.18146 2.89801 2.37887 2.77519 2.98518 4.58477 3.61503
0.03782 3.68971 4.41206 0.61958 0.77255 0.64191 0.74715
847 2.75145 4.71252 2.80109 2.56401 3.85261 3.25189 3.71665 3.46461 2.40147 2.98463 4.02911 3.01766 3.80997 1.60421 2.68491 2.81243 3.05413 3.20938 5.16412 3.84799 1140 - -
2.68618 4.42225 2.77519 2.73123 3.46354 2.40513 3.72494 3.29354 2.67741 2.69355 4.24690 2.90347 2.73739 3.18146 2.89801 2.37887 2.77519 2.98518 4.58477 3.61503
0.03782 3.68971 4.41206 0.61958 0.77255 0.64191 0.74715
848 2.33123 4.17207 3.27908 3.03470 3.81909 3.01404 4.06407 2.92517 2.97384 2.79402 3.86593 3.26778 3.70693 3.38105 3.23550 2.53154 1.52801 2.64069 5.27454 4.03869 1141 - -
2.68618 4.42225 2.77519 2.73123 3.46354 2.40513 3.72494 3.29354 2.67741 2.69355 4.24690 2.90347 2.73739 3.18146 2.89801 2.37887 2.77519 2.98518 4.58477 3.61503
0.03782 3.68971 4.41206 0.61958 0.77255 0.64191 0.74715
849 2.80535 4.87482 1.22337 2.25740 4.23128 3.08281 3.79410 3.78082 2.84781 3.42775 4.41212 2.79951 3.72433 3.04114 3.32941 2.79671 3.14408 3.44355 5.43550 4.14798 1142 - -
2.68618 4.42225 2.77519 2.73123 3.46354 2.40513 3.72494 3.29354 2.67741 2.69355 4.24690 2.90347 2.73739 3.18146 2.89801 2.37887 2.77519 2.98518 4.58477 3.61503
0.03782 3.68971 4.41206 0.61958 0.77255 0.64191 0.74715
850 2.81419 4.75052 2.97802 2.64754 4.09431 3.31300 3.68190 3.51431 1.32517 3.12542 4.10705 3.07652 3.83488 2.89173 2.43430 2.87431 3.08699 3.23582 5.23321 4.02251 1143 - -
2.68618 4.42225 2.77519 2.73123 3.46354 2.40513 3.72494 3.29354 2.67741 2.69355 4.24690 2.90347 2.73739 3.18146 2.89801 2.37887 2.77519 2.98518 4.58477 3.61503
0.03782 3.68971 4.41206 0.61958 0.77255 0.64191 0.74715
851 2.81419 4.75052 2.97802 2.64754 4.09431 3.31300 3.68190 3.51431 1.32517 3.12542 4.10705 3.07652 3.83488 2.89173 2.43430 2.87431 3.08699 3.23582 5.23321 4.02251 1144 - -
2.68618 4.42225 2.77519 2.73123 3.46354 2.40513 3.72494 3.29354 2.67741 2.69355 4.24690 2.90347 2.73739 3.18146 2.89801 2.37887 2.77519 2.98518 4.58477 3.61503
0.03782 3.68971 4.41206 0.61958 0.77255 0.64191 0.74715
852 2.95570 4.47801 3.88699 3.53725 3.08153 3.73572 4.25048 2.29059 3.30641 1.14168 3.18203 3.82854 4.21045 3.71971 3.50815 3.30044 3.26294 2.27804 4.88351 3.56168 1145 - -
2.68618 4.42225 2.77519 2.73123 3.46354 2.40513 3.72494 3.29354 2.67741 2.69355 4.24690 2.90347 2.73739 3.18146 2.89801 2.37887 2.77519 2.98518 4.58477 3.61503
0.03782 3.68971 4.41206 0.61958 0.77255 0.64191 0.74715
853 2.82202 4.65647 2.97238 2.72383 3.17446 3.30735 1.59825 3.49438 2.57333 3.00834 4.04492 3.13670 3.85542 3.09072 2.84526 2.89735 3.12340 3.23414 4.64084 3.13275 1146 - -
2.68618 4.42225 2.77519 2.73123 3.46354 2.40513 3.72494 3.29354 2.67741 2.69355 4.24690 2.90347 2.73739 3.18146 2.89801 2.37887 2.77519 2.98518 4.58477 3.61503
0.03782 3.68971 4.41206 0.61958 0.77255 0.64191 0.74715
854 2.80535 4.87482 1.22337 2.25740 4.23128 3.08281 3.79410 3.78082 2.84781 3.42775 4.41212 2.79951 3.72433 3.04114 3.32941 2.79671 3.14408 3.44355 5.43550 4.14798 1147 - -
2.68618 4.42225 2.77519 2.73123 3.46354 2.40513 3.72494 3.29354 2.67741 2.69355 4.24690 2.90347 2.73739 3.18146 2.89801 2.37887 2.77519 2.98518 4.58477 3.61503
0.03782 3.68971 4.41206 0.61958 0.77255 0.64191 0.74715
855 2.75145 4.71252 2.80109 2.56401 3.85261 3.25189 3.71665 3.46461 2.40147 2.98463 4.02911 3.01766 3.80997 1.60421 2.68491 2.81243 3.05413 3.20938 5.16412 3.84799 1148 - -
2.68618 4.42225 2.77519 2.73123 3.46354 2.40513 3.72494 3.29354 2.67741 2.69355 4.24690 2.90347 2.73739 3.18146 2.89801 2.37887 2.77519 2.98518 4.58477 3.61503
0.03782 3.68971 4.41206 0.61958 0.77255 0.64191 0.74715
856 2.81419 4.75052 2.97802 2.64754 4.09431 3.31300 3.68190 3.51431 1.32517 3.12542 4.10705 3.07652 3.83488 2.89173 2.43430 2.87431 3.08699 3.23582 5.23321 4.02251 1149 - -
2.68618 4.42225 2.77519 2.73123 3.46354 2.40513 3.72494 3.29354 2.67741 2.69355 4.24690 2.90347 2.73739 3.18146 2.89801 2.37887 2.77519 2.98518 4.58477 3.61503
0.03782 3.68971 4.41206 0.61958 0.77255 0.64191 0.74715
857 1.31618 4.11764 3.28684 3.08071 3.87759 2.94439 4.12282 2.97833 3.09199 2.86583 3.92805 3.27364 3.66709 3.45348 3.34929 2.47460 2.73956 2.66635 5.32423 4.10541 1150 - -
2.68618 4.42225 2.77519 2.73123 3.46354 2.40513 3.72494 3.29354 2.67741 2.69355 4.24690 2.90347 2.73739 3.18146 2.89801 2.37887 2.77519 2.98518 4.58477 3.61503

0.03782 3.68971 4.41206 0.61958 0.77255 0.64191 0.74715

858 2.95570 4.47801 3.88699 3.53725 3.08153 3.73572 4.25048 2.29059 3.30641 1.14168 3.18203 3.82854 4.21045 3.71971 3.50815 3.30044 3.26294 2.27804 4.88351 3.56168 1151 - -

2.68618 4.42225 2.77519 2.73123 3.46354 2.40513 3.72494 3.29354 2.67741 2.69355 4.24690 2.90347 2.73739 3.18146 2.89801 2.37887 2.77519 2.98518 4.58477 3.61503

0.03782 3.68971 4.41206 0.61958 0.77255 0.64191 0.74715

859 1.31618 4.11764 3.28684 3.08071 3.87759 2.94439 4.12282 2.97833 3.09199 2.86583 3.92805 3.27364 3.66709 3.45348 3.34929 2.47460 2.73956 2.66635 5.32423 4.10541 1152 - -

2.68618 4.42225 2.77519 2.73123 3.46354 2.40513 3.72494 3.29354 2.67741 2.69355 4.24690 2.90347 2.73739 3.18146 2.89801 2.37887 2.77519 2.98518 4.58477 3.61503

0.03782 3.68971 4.41206 0.61958 0.77255 0.64191 0.74715

860 2.80936 4.85412 2.44285 1.32179 4.18024 3.16567 3.75082 3.59744 2.60890 3.24971 4.24291 2.85883 3.76479 2.97779 2.98976 2.81828 3.11612 3.30456 5.38821 4.12184 1153 - -

2.68618 4.42225 2.77519 2.73123 3.46354 2.40513 3.72494 3.29354 2.67741 2.69355 4.24690 2.90347 2.73739 3.18146 2.89801 2.37887 2.77519 2.98518 4.58477 3.61503

0.03782 3.68971 4.41206 0.61958 0.77255 0.64191 0.74715

861 2.88652 4.37019 4.05193 3.66978 3.26042 3.84728 4.43781 1.34176 3.49290 1.89866 3.23881 3.94049 4.30608 3.86681 3.71057 3.33382 3.19357 1.87474 5.10472 3.81929 1154 - -

2.68618 4.42225 2.77519 2.73123 3.46354 2.40513 3.72494 3.29354 2.67741 2.69355 4.24690 2.90347 2.73739 3.18146 2.89801 2.37887 2.77519 2.98518 4.58477 3.61503

0.03782 3.68971 4.41206 0.61958 0.77255 0.64191 0.74715

862 2.99964 4.56315 3.57279 3.27716 2.34326 3.61999 3.56249 3.03542 3.13970 2.53489 3.73362 3.50967 4.10335 3.49096 3.35748 3.15189 3.28846 2.89883 3.97671 1.33703 1155 - -

2.68618 4.42225 2.77519 2.73123 3.46354 2.40513 3.72494 3.29354 2.67741 2.69355 4.24690 2.90347 2.73739 3.18146 2.89801 2.37887 2.77519 2.98518 4.58477 3.61503

0.03782 3.68971 4.41206 0.61958 0.77255 0.64191 0.74715

863 2.95570 4.47801 3.88699 3.53725 3.08153 3.73572 4.25048 2.29059 3.30641 1.14168 3.18203 3.82854 4.21045 3.71971 3.50815 3.30044 3.26294 2.27804 4.88351 3.56168 1156 - -

2.68618 4.42225 2.77519 2.73123 3.46354 2.40513 3.72494 3.29354 2.67741 2.69355 4.24690 2.90347 2.73739 3.18146 2.89801 2.37887 2.77519 2.98518 4.58477 3.61503

0.03782 3.68971 4.41206 0.61958 0.77255 0.64191 0.74715

864 2.95570 4.47801 3.88699 3.53725 3.08153 3.73572 4.25048 2.29059 3.30641 1.14168 3.18203 3.82854 4.21045 3.71971 3.50815 3.30044 3.26294 2.27804 4.88351 3.56168 1157 - -

2.68618 4.42225 2.77519 2.73123 3.46354 2.40513 3.72494 3.29354 2.67741 2.69355 4.24690 2.90347 2.73739 3.18146 2.89801 2.37887 2.77519 2.98518 4.58477 3.61503

0.03782 3.68971 4.41206 0.61958 0.77255 0.64191 0.74715

865 2.24189 4.15412 3.06133 2.87814 3.89444 2.88796 3.98828 3.39642 2.93811 3.13522 4.08514 3.12721 3.61351 3.30518 3.22334 1.40697 2.70989 2.97363 5.28456 3.97893 1158 - -

2.68618 4.42225 2.77519 2.73123 3.46354 2.40513 3.72494 3.29354 2.67741 2.69355 4.24690 2.90347 2.73739 3.18146 2.89801 2.37887 2.77519 2.98518 4.58477 3.61503

0.03782 3.68971 4.41206 0.61958 0.77255 0.64191 0.74715

866 2.95570 4.47801 3.88699 3.53725 3.08153 3.73572 4.25048 2.29059 3.30641 1.14168 3.18203 3.82854 4.21045 3.71971 3.50815 3.30044 3.26294 2.27804 4.88351 3.56168 1159 - -

2.68618 4.42225 2.77519 2.73123 3.46354 2.40513 3.72494 3.29354 2.67741 2.69355 4.24690 2.90347 2.73739 3.18146 2.89801 2.37887 2.77519 2.98518 4.58477 3.61503

0.03782 3.68971 4.41206 0.61958 0.77255 0.64191 0.74715

867 2.33123 4.17207 3.27908 3.03470 3.81909 3.01404 4.06407 2.92517 2.97384 2.79402 3.86593 3.26778 3.70693 3.38105 3.23550 2.53154 1.52801 2.64069 5.27454 4.03869 1160 - -

2.68618 4.42225 2.77519 2.73123 3.46354 2.40513 3.72494 3.29354 2.67741 2.69355 4.24690 2.90347 2.73739 3.18146 2.89801 2.37887 2.77519 2.98518 4.58477 3.61503

0.03782 3.68971 4.41206 0.61958 0.77255 0.64191 0.74715

868 2.80535 4.87482 1.22337 2.25740 4.23128 3.08281 3.79410 3.78082 2.84781 3.42775 4.41212 2.79951 3.72433 3.04114 3.32941 2.79671 3.14408 3.44355 5.43550 4.14798 1161 - -

2.68618 4.42225 2.77519 2.73123 3.46354 2.40513 3.72494 3.29354 2.67741 2.69355 4.24690 2.90347 2.73739 3.18146 2.89801 2.37887 2.77519 2.98518 4.58477 3.61503

0.03782 3.68971 4.41206 0.61958 0.77255 0.64191 0.74715

869 2.80936 4.85412 2.44285 1.32179 4.18024 3.16567 3.75082 3.59744 2.60890 3.24971 4.24291 2.85883 3.76479 2.97779 2.98976 2.81828 3.11612 3.30456 5.38821 4.12184 1162 - -

2.68618 4.42225 2.77519 2.73123 3.46354 2.40513 3.72494 3.29354 2.67741 2.69355 4.24690 2.90347 2.73739 3.18146 2.89801 2.37887 2.77519 2.98518 4.58477 3.61503
0.03782 3.68971 4.41206 0.61958 0.77255 0.64191 0.74715
870 2.95570 4.47801 3.88699 3.53725 3.08153 3.73572 4.25048 2.29059 3.30641 1.14168 3.18203 3.82854 4.21045 3.71971 3.50815 3.30044 3.26294 2.27804 4.88351 3.56168 1163 - -
2.68618 4.42225 2.77519 2.73123 3.46354 2.40513 3.72494 3.29354 2.67741 2.69355 4.24690 2.90347 2.73739 3.18146 2.89801 2.37887 2.77519 2.98518 4.58477 3.61503
0.03782 3.68971 4.41206 0.61958 0.77255 0.64191 0.74715
871 2.66384 4.26718 3.86051 3.52869 3.42541 3.52084 4.36452 2.09728 3.39277 2.15394 3.43086 3.74448 4.09895 3.77068 3.62046 3.02131 3.03475 1.29720 5.17454 3.88959 1164 - -
2.68618 4.42225 2.77519 2.73123 3.46354 2.40513 3.72494 3.29354 2.67741 2.69355 4.24690 2.90347 2.73739 3.18146 2.89801 2.37887 2.77519 2.98518 4.58477 3.61503
0.03782 3.68971 4.41206 0.61958 0.77255 0.64191 0.74715
872 2.33123 4.17207 3.27908 3.03470 3.81909 3.01404 4.06407 2.92517 2.97384 2.79402 3.86593 3.26778 3.70693 3.38105 3.23550 2.53154 1.52801 2.64069 5.27454 4.03869 1165 - -
2.68618 4.42225 2.77519 2.73123 3.46354 2.40513 3.72494 3.29354 2.67741 2.69355 4.24690 2.90347 2.73739 3.18146 2.89801 2.37887 2.77519 2.98518 4.58477 3.61503
0.03782 3.68971 4.41206 0.61958 0.77255 0.64191 0.74715
873 2.24189 4.15412 3.06133 2.87814 3.89444 2.88796 3.98828 3.39642 2.93811 3.13522 4.08514 3.12721 3.61351 3.30518 3.22334 1.40697 2.70989 2.97363 5.28456 3.97893 1166 - -
2.68618 4.42225 2.77519 2.73123 3.46354 2.40513 3.72494 3.29354 2.67741 2.69355 4.24690 2.90347 2.73739 3.18146 2.89801 2.37887 2.77519 2.98518 4.58477 3.61503
0.03782 3.68971 4.41206 0.61958 0.77255 0.64191 0.74715
874 2.33123 4.17207 3.27908 3.03470 3.81909 3.01404 4.06407 2.92517 2.97384 2.79402 3.86593 3.26778 3.70693 3.38105 3.23550 2.53154 1.52801 2.64069 5.27454 4.03869 1167 - -
2.68618 4.42225 2.77519 2.73123 3.46354 2.40513 3.72494 3.29354 2.67741 2.69355 4.24690 2.90347 2.73739 3.18146 2.89801 2.37887 2.77519 2.98518 4.58477 3.61503
0.03782 3.68971 4.41206 0.61958 0.77255 0.64191 0.74715
875 2.81190 4.64511 3.28512 2.84967 3.95281 3.32202 3.71995 3.48986 2.20826 3.04039 4.05567 3.21864 3.84267 2.95856 1.30366 2.91066 3.09663 3.22431 5.11666 3.92964 1168 - -
2.68618 4.42225 2.77519 2.73123 3.46354 2.40513 3.72494 3.29354 2.67741 2.69355 4.24690 2.90347 2.73739 3.18146 2.89801 2.37887 2.77519 2.98518 4.58477 3.61503
0.03782 3.68971 4.41206 0.61958 0.77255 0.64191 0.74715
876 2.24189 4.15412 3.06133 2.87814 3.89444 2.88796 3.98828 3.39642 2.93811 3.13522 4.08514 3.12721 3.61351 3.30518 3.22334 1.40697 2.70989 2.97363 5.28456 3.97893 1169 - -
2.68618 4.42225 2.77519 2.73123 3.46354 2.40513 3.72494 3.29354 2.67741 2.69355 4.24690 2.90347 2.73739 3.18146 2.89801 2.37887 2.77519 2.98518 4.58477 3.61503
0.03782 3.68971 4.41206 0.61958 0.77255 0.64191 0.74715
877 2.33123 4.17207 3.27908 3.03470 3.81909 3.01404 4.06407 2.92517 2.97384 2.79402 3.86593 3.26778 3.70693 3.38105 3.23550 2.53154 1.52801 2.64069 5.27454 4.03869 1170 - -
2.68618 4.42225 2.77519 2.73123 3.46354 2.40513 3.72494 3.29354 2.67741 2.69355 4.24690 2.90347 2.73739 3.18146 2.89801 2.37887 2.77519 2.98518 4.58477 3.61503
0.03782 3.68971 4.41206 0.61958 0.77255 0.64191 0.74715
878 3.03627 4.49697 3.98008 3.66865 1.36975 3.75169 3.82000 2.59321 3.58195 2.01239 3.35868 3.81567 4.22222 3.80445 3.74700 3.31035 3.34138 2.56812 4.14110 2.53835 1171 - -
2.68618 4.42225 2.77519 2.73123 3.46354 2.40513 3.72494 3.29354 2.67741 2.69355 4.24690 2.90347 2.73739 3.18146 2.89801 2.37887 2.77519 2.98518 4.58477 3.61503
0.03782 3.68971 4.41206 0.61958 0.77255 0.64191 0.74715
879 2.46513 4.24664 3.09956 2.98169 4.10469 1.07816 4.10397 3.66750 3.13599 3.34383 4.32651 3.26662 3.63194 3.47710 3.38330 2.63370 2.93263 3.23311 5.23953 4.20371 1172 - -
2.68618 4.42225 2.77519 2.73123 3.46354 2.40513 3.72494 3.29354 2.67741 2.69355 4.24690 2.90347 2.73739 3.18146 2.89801 2.37887 2.77519 2.98518 4.58477 3.61503
0.03782 3.68971 4.41206 0.61958 0.77255 0.64191 0.74715
880 2.99964 4.56315 3.57279 3.27716 2.34326 3.61999 3.56249 3.03542 3.13970 2.53489 3.73362 3.50967 4.10335 3.49096 3.35748 3.15189 3.28846 2.89883 3.97671 1.33703 1173 - -
2.68618 4.42225 2.77519 2.73123 3.46354 2.40513 3.72494 3.29354 2.67741 2.69355 4.24690 2.90347 2.73739 3.18146 2.89801 2.37887 2.77519 2.98518 4.58477 3.61503
0.03782 3.68971 4.41206 0.61958 0.77255 0.64191 0.74715

881 2.66384 4.26718 3.86051 3.52869 3.42541 3.52084 4.36452 2.09728 3.39277 2.15394 3.43086 3.74448 4.09895 3.77068 3.62046 3.02131 3.03475 1.29720 5.17454 3.88959 1174 - -
2.68618 4.42225 2.77519 2.73123 3.46354 2.40513 3.72494 3.29354 2.67741 2.69355 4.24690 2.90347 2.73739 3.18146 2.89801 2.37887 2.77519 2.98518 4.58477 3.61503
0.03782 3.68971 4.41206 0.61958 0.77255 0.64191 0.74715
882 1.31618 4.11764 3.28684 3.08071 3.87759 2.94439 4.12282 2.97833 3.09199 2.86583 3.92805 3.27364 3.66709 3.45348 3.34929 2.47460 2.73956 2.66635 5.32423 4.10541 1175 - -
2.68618 4.42225 2.77519 2.73123 3.46354 2.40513 3.72494 3.29354 2.67741 2.69355 4.24690 2.90347 2.73739 3.18146 2.89801 2.37887 2.77519 2.98518 4.58477 3.61503
0.03782 3.68971 4.41206 0.61958 0.77255 0.64191 0.74715
883 2.75145 4.71252 2.80109 2.56401 3.85261 3.25189 3.71665 3.46461 2.40147 2.98463 4.02911 3.01766 3.80997 1.60421 2.68491 2.81243 3.05413 3.20938 5.16412 3.84799 1176 - -
2.68618 4.42225 2.77519 2.73123 3.46354 2.40513 3.72494 3.29354 2.67741 2.69355 4.24690 2.90347 2.73739 3.18146 2.89801 2.37887 2.77519 2.98518 4.58477 3.61503
0.03782 3.68971 4.41206 0.61958 0.77255 0.64191 0.74715
884 2.46513 4.24664 3.09956 2.98169 4.10469 1.07816 4.10397 3.66750 3.13599 3.34383 4.32651 3.26662 3.63194 3.47710 3.38330 2.63370 2.93263 3.23311 5.23953 4.20371 1177 - -
2.68618 4.42225 2.77519 2.73123 3.46354 2.40513 3.72494 3.29354 2.67741 2.69355 4.24690 2.90347 2.73739 3.18146 2.89801 2.37887 2.77519 2.98518 4.58477 3.61503
0.03782 3.68971 4.41206 0.61958 0.77255 0.64191 0.74715
885 2.95570 4.47801 3.88699 3.53725 3.08153 3.73572 4.25048 2.29059 3.30641 1.14168 3.18203 3.82854 4.21045 3.71971 3.50815 3.30044 3.26294 2.27804 4.88351 3.56168 1178 - -
2.68618 4.42225 2.77519 2.73123 3.46354 2.40513 3.72494 3.29354 2.67741 2.69355 4.24690 2.90347 2.73739 3.18146 2.89801 2.37887 2.77519 2.98518 4.58477 3.61503
0.03782 3.68971 4.41206 0.61958 0.77255 0.64191 0.74715
886 2.46513 4.24664 3.09956 2.98169 4.10469 1.07816 4.10397 3.66750 3.13599 3.34383 4.32651 3.26662 3.63194 3.47710 3.38330 2.63370 2.93263 3.23311 5.23953 4.20371 1179 - -
2.68618 4.42225 2.77519 2.73123 3.46354 2.40513 3.72494 3.29354 2.67741 2.69355 4.24690 2.90347 2.73739 3.18146 2.89801 2.37887 2.77519 2.98518 4.58477 3.61503
0.03782 3.68971 4.41206 0.61958 0.77255 0.64191 0.74715
887 2.46513 4.24664 3.09956 2.98169 4.10469 1.07816 4.10397 3.66750 3.13599 3.34383 4.32651 3.26662 3.63194 3.47710 3.38330 2.63370 2.93263 3.23311 5.23953 4.20371 1180 - -
2.68618 4.42225 2.77519 2.73123 3.46354 2.40513 3.72494 3.29354 2.67741 2.69355 4.24690 2.90347 2.73739 3.18146 2.89801 2.37887 2.77519 2.98518 4.58477 3.61503
0.03782 3.68971 4.41206 0.61958 0.77255 0.64191 0.74715
888 2.95570 4.47801 3.88699 3.53725 3.08153 3.73572 4.25048 2.29059 3.30641 1.14168 3.18203 3.82854 4.21045 3.71971 3.50815 3.30044 3.26294 2.27804 4.88351 3.56168 1181 - -
2.68618 4.42225 2.77519 2.73123 3.46354 2.40513 3.72494 3.29354 2.67741 2.69355 4.24690 2.90347 2.73739 3.18146 2.89801 2.37887 2.77519 2.98518 4.58477 3.61503
0.03782 3.68971 4.41206 0.61958 0.77255 0.64191 0.74715
889 2.81419 4.75052 2.97802 2.64754 4.09431 3.31300 3.68190 3.51431 1.32517 3.12542 4.10705 3.07652 3.83488 2.89173 2.43430 2.87431 3.08699 3.23582 5.23321 4.02251 1182 - -
2.68618 4.42225 2.77519 2.73123 3.46354 2.40513 3.72494 3.29354 2.67741 2.69355 4.24690 2.90347 2.73739 3.18146 2.89801 2.37887 2.77519 2.98518 4.58477 3.61503
0.03782 3.68971 4.41206 0.61958 0.77255 0.64191 0.74715
890 2.53919 4.31574 3.12837 2.94944 3.94108 3.01406 4.03464 3.44720 2.99214 3.09147 4.14117 3.26964 1.25262 3.38748 3.25050 2.70343 2.96896 3.11334 5.17040 4.06284 1183 - -
2.68618 4.42225 2.77519 2.73123 3.46354 2.40513 3.72494 3.29354 2.67741 2.69355 4.24690 2.90347 2.73739 3.18146 2.89801 2.37887 2.77519 2.98518 4.58477 3.61503
0.03782 3.68971 4.41206 0.61958 0.77255 0.64191 0.74715
891 3.06047 4.49594 3.82876 3.49619 2.52194 3.53862 3.70765 3.08393 3.22453 2.50664 3.72585 3.68429 4.04410 3.62345 3.38267 3.26521 3.35391 2.96950 1.35528 2.53548 1184 - -
2.68618 4.42225 2.77519 2.73123 3.46354 2.40513 3.72494 3.29354 2.67741 2.69355 4.24690 2.90347 2.73739 3.18146 2.89801 2.37887 2.77519 2.98518 4.58477 3.61503
0.03782 3.68971 4.41206 0.61958 0.77255 0.64191 0.74715
892 2.88652 4.37019 4.05193 3.66978 3.26042 3.84728 4.43781 1.34176 3.49290 1.89866 3.23881 3.94049 4.30608 3.86681 3.71057 3.33382 3.19357 1.87474 5.10472 3.81929 1185 - -
2.68618 4.42225 2.77519 2.73123 3.46354 2.40513 3.72494 3.29354 2.67741 2.69355 4.24690 2.90347 2.73739 3.18146 2.89801 2.37887 2.77519 2.98518 4.58477 3.61503

0.03782 3.68971 4.41206 0.61958 0.77255 0.64191 0.74715

893 2.95570 4.47801 3.88699 3.53725 3.08153 3.73572 4.25048 2.29059 3.30641 1.14168 3.18203 3.82854 4.21045 3.71971 3.50815 3.30044 3.26294 2.27804 4.88351 3.56168 1186 - -

2.68618 4.42225 2.77519 2.73123 3.46354 2.40513 3.72494 3.29354 2.67741 2.69355 4.24690 2.90347 2.73739 3.18146 2.89801 2.37887 2.77519 2.98518 4.58477 3.61503

0.03782 3.68971 4.41206 0.61958 0.77255 0.64191 0.74715

894 2.99964 4.56315 3.57279 3.27716 2.34326 3.61999 3.56249 3.03542 3.13970 2.53489 3.73362 3.50967 4.10335 3.49096 3.35748 3.15189 3.28846 2.89883 3.97671 1.33703 1187 - -

2.68618 4.42225 2.77519 2.73123 3.46354 2.40513 3.72494 3.29354 2.67741 2.69355 4.24690 2.90347 2.73739 3.18146 2.89801 2.37887 2.77519 2.98518 4.58477 3.61503

0.03782 3.68971 4.41206 0.61958 0.77255 0.64191 0.74715

895 2.80936 4.85412 2.44285 1.32179 4.18024 3.16567 3.75082 3.59744 2.60890 3.24971 4.24291 2.85883 3.76479 2.97779 2.98976 2.81828 3.11612 3.30456 5.38821 4.12184 1188 - -

2.68618 4.42225 2.77519 2.73123 3.46354 2.40513 3.72494 3.29354 2.67741 2.69355 4.24690 2.90347 2.73739 3.18146 2.89801 2.37887 2.77519 2.98518 4.58477 3.61503

0.03782 3.68971 4.41206 0.61958 0.77255 0.64191 0.74715

896 2.53919 4.31574 3.12837 2.94944 3.94108 3.01406 4.03464 3.44720 2.99214 3.09147 4.14117 3.26964 1.25262 3.38748 3.25050 2.70343 2.96896 3.11334 5.17040 4.06284 1189 - -

2.68618 4.42225 2.77519 2.73123 3.46354 2.40513 3.72494 3.29354 2.67741 2.69355 4.24690 2.90347 2.73739 3.18146 2.89801 2.37887 2.77519 2.98518 4.58477 3.61503

0.03782 3.68971 4.41206 0.61958 0.77255 0.64191 0.74715

897 2.81190 4.64511 3.28512 2.84967 3.95281 3.32202 3.71995 3.48986 2.20826 3.04039 4.05567 3.21864 3.84267 2.95856 1.30366 2.91066 3.09663 3.22431 5.11666 3.92964 1190 - -

2.68618 4.42225 2.77519 2.73123 3.46354 2.40513 3.72494 3.29354 2.67741 2.69355 4.24690 2.90347 2.73739 3.18146 2.89801 2.37887 2.77519 2.98518 4.58477 3.61503

0.03782 3.68971 4.41206 0.61958 0.77255 0.64191 0.74715

898 2.80535 4.87482 1.22337 2.25740 4.23128 3.08281 3.79410 3.78082 2.84781 3.42775 4.41212 2.79951 3.72433 3.04114 3.32941 2.79671 3.14408 3.44355 5.43550 4.14798 1191 - -

2.68618 4.42225 2.77519 2.73123 3.46354 2.40513 3.72494 3.29354 2.67741 2.69355 4.24690 2.90347 2.73739 3.18146 2.89801 2.37887 2.77519 2.98518 4.58477 3.61503

0.03782 3.68971 4.41206 0.61958 0.77255 0.64191 0.74715

899 2.81419 4.75052 2.97802 2.64754 4.09431 3.31300 3.68190 3.51431 1.32517 3.12542 4.10705 3.07652 3.83488 2.89173 2.43430 2.87431 3.08699 3.23582 5.23321 4.02251 1192 - -

2.68618 4.42225 2.77519 2.73123 3.46354 2.40513 3.72494 3.29354 2.67741 2.69355 4.24690 2.90347 2.73739 3.18146 2.89801 2.37887 2.77519 2.98518 4.58477 3.61503

0.03782 3.68971 4.41206 0.61958 0.77255 0.64191 0.74715

900 2.81419 4.75052 2.97802 2.64754 4.09431 3.31300 3.68190 3.51431 1.32517 3.12542 4.10705 3.07652 3.83488 2.89173 2.43430 2.87431 3.08699 3.23582 5.23321 4.02251 1193 - -

2.68618 4.42225 2.77519 2.73123 3.46354 2.40513 3.72494 3.29354 2.67741 2.69355 4.24690 2.90347 2.73739 3.18146 2.89801 2.37887 2.77519 2.98518 4.58477 3.61503

0.03782 3.68971 4.41206 0.61958 0.77255 0.64191 0.74715

901 2.33123 4.17207 3.27908 3.03470 3.81909 3.01404 4.06407 2.92517 2.97384 2.79402 3.86593 3.26778 3.70693 3.38105 3.23550 2.53154 1.52801 2.64069 5.27454 4.03869 1194 - -

2.68618 4.42225 2.77519 2.73123 3.46354 2.40513 3.72494 3.29354 2.67741 2.69355 4.24690 2.90347 2.73739 3.18146 2.89801 2.37887 2.77519 2.98518 4.58477 3.61503

0.03782 3.68971 4.41206 0.61958 0.77255 0.64191 0.74715

902 2.53919 4.31574 3.12837 2.94944 3.94108 3.01406 4.03464 3.44720 2.99214 3.09147 4.14117 3.26964 1.25262 3.38748 3.25050 2.70343 2.96896 3.11334 5.17040 4.06284 1195 - -

2.68618 4.42225 2.77519 2.73123 3.46354 2.40513 3.72494 3.29354 2.67741 2.69355 4.24690 2.90347 2.73739 3.18146 2.89801 2.37887 2.77519 2.98518 4.58477 3.61503

0.03782 3.68971 4.41206 0.61958 0.77255 0.64191 0.74715

903 2.58408 4.55777 2.63767 2.50951 3.89313 3.06999 3.79869 3.57286 2.69204 3.24473 4.20914 1.53216 3.71772 3.07984 3.03279 2.66399 2.96516 3.21422 5.22244 3.86110 1196 - -

2.68618 4.42225 2.77519 2.73123 3.46354 2.40513 3.72494 3.29354 2.67741 2.69355 4.24690 2.90347 2.73739 3.18146 2.89801 2.37887 2.77519 2.98518 4.58477 3.61503

0.03782 3.68971 4.41206 0.61958 0.77255 0.64191 0.74715

904 2.53919 4.31574 3.12837 2.94944 3.94108 3.01406 4.03464 3.44720 2.99214 3.09147 4.14117 3.26964 1.25262 3.38748 3.25050 2.70343 2.96896 3.11334 5.17040 4.06284 1197 - -

2.68618 4.42225 2.77519 2.73123 3.46354 2.40513 3.72494 3.29354 2.67741 2.69355 4.24690 2.90347 2.73739 3.18146 2.89801 2.37887 2.77519 2.98518 4.58477 3.61503

0.03782 3.68971 4.41206 0.61958 0.77255 0.64191 0.74715

905 2.53919 4.31574 3.12837 2.94944 3.94108 3.01406 4.03464 3.44720 2.99214 3.09147 4.14117 3.26964 1.25262 3.38748 3.25050 2.70343 2.96896 3.11334 5.17040 4.06284 1198 - -

2.68618 4.42225 2.77519 2.73123 3.46354 2.40513 3.72494 3.29354 2.67741 2.69355 4.24690 2.90347 2.73739 3.18146 2.89801 2.37887 2.77519 2.98518 4.58477 3.61503

0.03782 3.68971 4.41206 0.61958 0.77255 0.64191 0.74715

906 2.40719 1.40547 3.87078 3.57616 3.61829 3.05978 4.26483 2.73897 3.40375 2.65893 3.77889 3.61209 3.75249 3.75916 3.55770 2.67680 2.86014 2.49438 5.06450 3.90470 1199 - -

2.68618 4.42225 2.77519 2.73123 3.46354 2.40513 3.72494 3.29354 2.67741 2.69355 4.24690 2.90347 2.73739 3.18146 2.89801 2.37887 2.77519 2.98518 4.58477 3.61503

0.03782 3.68971 4.41206 0.61958 0.77255 0.64191 0.74715

907 2.66384 4.26718 3.86051 3.52869 3.42541 3.52084 4.36452 2.09728 3.39277 2.15394 3.43086 3.74448 4.09895 3.77068 3.62046 3.02131 3.03475 1.29720 5.17454 3.88959 1200 - -

2.68618 4.42225 2.77519 2.73123 3.46354 2.40513 3.72494 3.29354 2.67741 2.69355 4.24690 2.90347 2.73739 3.18146 2.89801 2.37887 2.77519 2.98518 4.58477 3.61503

0.03782 3.68971 4.41206 0.61958 0.77255 0.64191 0.74715

908 2.81190 4.64511 3.28512 2.84967 3.95281 3.32202 3.71995 3.48986 2.20826 3.04039 4.05567 3.21864 3.84267 2.95856 1.30366 2.91066 3.09663 3.22431 5.11666 3.92964 1201 - -

2.68618 4.42225 2.77519 2.73123 3.46354 2.40513 3.72494 3.29354 2.67741 2.69355 4.24690 2.90347 2.73739 3.18146 2.89801 2.37887 2.77519 2.98518 4.58477 3.61503

0.03782 3.68971 4.41206 0.61958 0.77255 0.64191 0.74715

909 1.31618 4.11764 3.28684 3.08071 3.87759 2.94439 4.12282 2.97833 3.09199 2.86583 3.92805 3.27364 3.66709 3.45348 3.34929 2.47460 2.73956 2.66635 5.32423 4.10541 1202 - -

2.68618 4.42225 2.77519 2.73123 3.46354 2.40513 3.72494 3.29354 2.67741 2.69355 4.24690 2.90347 2.73739 3.18146 2.89801 2.37887 2.77519 2.98518 4.58477 3.61503

0.03782 3.68971 4.41206 0.61958 0.77255 0.64191 0.74715

910 2.83528 4.41805 3.85371 3.44211 3.14741 3.69369 4.22020 2.30593 3.21644 1.77982 1.78422 3.73947 4.16154 3.61600 3.44155 3.16108 3.14135 2.28792 4.95809 3.69770 1203 - -

2.68618 4.42225 2.77519 2.73123 3.46354 2.40513 3.72494 3.29354 2.67741 2.69355 4.24690 2.90347 2.73739 3.18146 2.89801 2.37887 2.77519 2.98518 4.58477 3.61503

0.03782 3.68971 4.41206 0.61958 0.77255 0.64191 0.74715

911 2.24189 4.15412 3.06133 2.87814 3.89444 2.88796 3.98828 3.39642 2.93811 3.13522 4.08514 3.12721 3.61351 3.30518 3.22334 1.40697 2.70989 2.97363 5.28456 3.97893 1204 - -

2.68618 4.42225 2.77519 2.73123 3.46354 2.40513 3.72494 3.29354 2.67741 2.69355 4.24690 2.90347 2.73739 3.18146 2.89801 2.37887 2.77519 2.98518 4.58477 3.61503

0.03782 3.68971 4.41206 0.61958 0.77255 0.64191 0.74715

912 2.83528 4.41805 3.85371 3.44211 3.14741 3.69369 4.22020 2.30593 3.21644 1.77982 1.78422 3.73947 4.16154 3.61600 3.44155 3.16108 3.14135 2.28792 4.95809 3.69770 1205 - -

2.68618 4.42225 2.77519 2.73123 3.46354 2.40513 3.72494 3.29354 2.67741 2.69355 4.24690 2.90347 2.73739 3.18146 2.89801 2.37887 2.77519 2.98518 4.58477 3.61503

0.03782 3.68971 4.41206 0.61958 0.77255 0.64191 0.74715

913 2.80936 4.85412 2.44285 1.32179 4.18024 3.16567 3.75082 3.59744 2.60890 3.24971 4.24291 2.85883 3.76479 2.97779 2.98976 2.81828 3.11612 3.30456 5.38821 4.12184 1206 - -

2.68618 4.42225 2.77519 2.73123 3.46354 2.40513 3.72494 3.29354 2.67741 2.69355 4.24690 2.90347 2.73739 3.18146 2.89801 2.37887 2.77519 2.98518 4.58477 3.61503

0.03782 3.68971 4.41206 0.61958 0.77255 0.64191 0.74715

914 2.53919 4.31574 3.12837 2.94944 3.94108 3.01406 4.03464 3.44720 2.99214 3.09147 4.14117 3.26964 1.25262 3.38748 3.25050 2.70343 2.96896 3.11334 5.17040 4.06284 1207 - -

2.68618 4.42225 2.77519 2.73123 3.46354 2.40513 3.72494 3.29354 2.67741 2.69355 4.24690 2.90347 2.73739 3.18146 2.89801 2.37887 2.77519 2.98518 4.58477 3.61503

0.03782 3.68971 4.41206 0.61958 0.77255 0.64191 0.74715

915 2.40719 1.40547 3.87078 3.57616 3.61829 3.05978 4.26483 2.73897 3.40375 2.65893 3.77889 3.61209 3.75249 3.75916 3.55770 2.67680 2.86014 2.49438 5.06450 3.90470 1208 - -

2.68618 4.42225 2.77519 2.73123 3.46354 2.40513 3.72494 3.29354 2.67741 2.69355 4.24690 2.90347 2.73739 3.18146 2.89801 2.37887 2.77519 2.98518 4.58477 3.61503

0.03782 3.68971 4.41206 0.61958 0.77255 0.64191 0.74715

916 3.03627 4.49697 3.98008 3.66865 1.36975 3.75169 3.82000 2.59321 3.58195 2.01239 3.35868 3.81567 4.22222 3.80445 3.74700 3.31035 3.34138 2.56812 4.14110 2.53835 1209 - -
2.68618 4.42225 2.77519 2.73123 3.46354 2.40513 3.72494 3.29354 2.67741 2.69355 4.24690 2.90347 2.73739 3.18146 2.89801 2.37887 2.77519 2.98518 4.58477 3.61503
0.03782 3.68971 4.41206 0.61958 0.77255 0.64191 0.74715

917 2.95570 4.47801 3.88699 3.53725 3.08153 3.73572 4.25048 2.29059 3.30641 1.14168 3.18203 3.82854 4.21045 3.71971 3.50815 3.30044 3.26294 2.27804 4.88351 3.56168 1210 - -
2.68618 4.42225 2.77519 2.73123 3.46354 2.40513 3.72494 3.29354 2.67741 2.69355 4.24690 2.90347 2.73739 3.18146 2.89801 2.37887 2.77519 2.98518 4.58477 3.61503
0.03782 3.68971 4.41206 0.61958 0.77255 0.64191 0.74715

918 2.81190 4.64511 3.28512 2.84967 3.95281 3.32202 3.71995 3.48986 2.20826 3.04039 4.05567 3.21864 3.84267 2.95856 1.30366 2.91066 3.09663 3.22431 5.11666 3.92964 1211 - -
2.68618 4.42225 2.77519 2.73123 3.46354 2.40513 3.72494 3.29354 2.67741 2.69355 4.24690 2.90347 2.73739 3.18146 2.89801 2.37887 2.77519 2.98518 4.58477 3.61503
0.03782 3.68971 4.41206 0.61958 0.77255 0.64191 0.74715

919 1.31618 4.11764 3.28684 3.08071 3.87759 2.94439 4.12282 2.97833 3.09199 2.86583 3.92805 3.27364 3.66709 3.45348 3.34929 2.47460 2.73956 2.66635 5.32423 4.10541 1212 - -
2.68618 4.42225 2.77519 2.73123 3.46354 2.40513 3.72494 3.29354 2.67741 2.69355 4.24690 2.90347 2.73739 3.18146 2.89801 2.37887 2.77519 2.98518 4.58477 3.61503
0.03782 3.68971 4.41206 0.61958 0.77255 0.64191 0.74715

920 2.53919 4.31574 3.12837 2.94944 3.94108 3.01406 4.03464 3.44720 2.99214 3.09147 4.14117 3.26964 1.25262 3.38748 3.25050 2.70343 2.96896 3.11334 5.17040 4.06284 1213 - -
2.68618 4.42225 2.77519 2.73123 3.46354 2.40513 3.72494 3.29354 2.67741 2.69355 4.24690 2.90347 2.73739 3.18146 2.89801 2.37887 2.77519 2.98518 4.58477 3.61503
0.03782 3.68971 4.41206 0.61958 0.77255 0.64191 0.74715

921 2.95570 4.47801 3.88699 3.53725 3.08153 3.73572 4.25048 2.29059 3.30641 1.14168 3.18203 3.82854 4.21045 3.71971 3.50815 3.30044 3.26294 2.27804 4.88351 3.56168 1214 - -
2.68618 4.42225 2.77519 2.73123 3.46354 2.40513 3.72494 3.29354 2.67741 2.69355 4.24690 2.90347 2.73739 3.18146 2.89801 2.37887 2.77519 2.98518 4.58477 3.61503
0.03782 3.68971 4.41206 0.61958 0.77255 0.64191 0.74715

922 2.82202 4.65647 2.97238 2.72383 3.17446 3.30735 1.59825 3.49438 2.57333 3.00834 4.04492 3.13670 3.85542 3.09072 2.84526 2.89735 3.12340 3.23414 4.64084 3.13275 1215 - -
2.68618 4.42225 2.77519 2.73123 3.46354 2.40513 3.72494 3.29354 2.67741 2.69355 4.24690 2.90347 2.73739 3.18146 2.89801 2.37887 2.77519 2.98518 4.58477 3.61503
0.03782 3.68971 4.41206 0.61958 0.77255 0.64191 0.74715

923 2.46513 4.24664 3.09956 2.98169 4.10469 1.07816 4.10397 3.66750 3.13599 3.34383 4.32651 3.26662 3.63194 3.47710 3.38330 2.63370 2.93263 3.23311 5.23953 4.20371 1216 - -
2.68618 4.42225 2.77519 2.73123 3.46354 2.40513 3.72494 3.29354 2.67741 2.69355 4.24690 2.90347 2.73739 3.18146 2.89801 2.37887 2.77519 2.98518 4.58477 3.61503
0.03782 3.68971 4.41206 0.61958 0.77255 0.64191 0.74715

924 2.40719 1.40547 3.87078 3.57616 3.61829 3.05978 4.26483 2.73897 3.40375 2.65893 3.77889 3.61209 3.75249 3.75916 3.55770 2.67680 2.86014 2.49438 5.06450 3.90470 1217 - -
2.68618 4.42225 2.77519 2.73123 3.46354 2.40513 3.72494 3.29354 2.67741 2.69355 4.24690 2.90347 2.73739 3.18146 2.89801 2.37887 2.77519 2.98518 4.58477 3.61503
0.03782 3.68971 4.41206 0.61958 0.77255 0.64191 0.74715

925 2.75145 4.71252 2.80109 2.56401 3.85261 3.25189 3.71665 3.46461 2.40147 2.98463 4.02911 3.01766 3.80997 1.60421 2.68491 2.81243 3.05413 3.20938 5.16412 3.84799 1218 - -
2.68618 4.42225 2.77519 2.73123 3.46354 2.40513 3.72494 3.29354 2.67741 2.69355 4.24690 2.90347 2.73739 3.18146 2.89801 2.37887 2.77519 2.98518 4.58477 3.61503
0.03782 3.68971 4.41206 0.61958 0.77255 0.64191 0.74715

926 1.31618 4.11764 3.28684 3.08071 3.87759 2.94439 4.12282 2.97833 3.09199 2.86583 3.92805 3.27364 3.66709 3.45348 3.34929 2.47460 2.73956 2.66635 5.32423 4.10541 1219 - -
2.68618 4.42225 2.77519 2.73123 3.46354 2.40513 3.72494 3.29354 2.67741 2.69355 4.24690 2.90347 2.73739 3.18146 2.89801 2.37887 2.77519 2.98518 4.58477 3.61503
0.03782 3.68971 4.41206 0.61958 0.77255 0.64191 0.74715

927 2.81419 4.75052 2.97802 2.64754 4.09431 3.31300 3.68190 3.51431 1.32517 3.12542 4.10705 3.07652 3.83488 2.89173 2.43430 2.87431 3.08699 3.23582 5.23321 4.02251 1220 - -
2.68618 4.42225 2.77519 2.73123 3.46354 2.40513 3.72494 3.29354 2.67741 2.69355 4.24690 2.90347 2.73739 3.18146 2.89801 2.37887 2.77519 2.98518 4.58477 3.61503

0.03782 3.68971 4.41206 0.61958 0.77255 0.64191 0.74715
928 2.33123 4.17207 3.27908 3.03470 3.81909 3.01404 4.06407 2.92517 2.97384 2.79402 3.86593 3.26778 3.70693 3.38105 3.23550 2.53154 1.52801 2.64069 5.27454 4.03869 1221 - -
2.68618 4.42225 2.77519 2.73123 3.46354 2.40513 3.72494 3.29354 2.67741 2.69355 4.24690 2.90347 2.73739 3.18146 2.89801 2.37887 2.77519 2.98518 4.58477 3.61503
0.03782 3.68971 4.41206 0.61958 0.77255 0.64191 0.74715
929 2.88652 4.37019 4.05193 3.66978 3.26042 3.84728 4.43781 1.34176 3.49290 1.89866 3.23881 3.94049 4.30608 3.86681 3.71057 3.33382 3.19357 1.87474 5.10472 3.81929 1222 - -
2.68618 4.42225 2.77519 2.73123 3.46354 2.40513 3.72494 3.29354 2.67741 2.69355 4.24690 2.90347 2.73739 3.18146 2.89801 2.37887 2.77519 2.98518 4.58477 3.61503
0.03782 3.68971 4.41206 0.61958 0.77255 0.64191 0.74715
930 2.81419 4.75052 2.97802 2.64754 4.09431 3.31300 3.68190 3.51431 1.32517 3.12542 4.10705 3.07652 3.83488 2.89173 2.43430 2.87431 3.08699 3.23582 5.23321 4.02251 1223 - -
2.68618 4.42225 2.77519 2.73123 3.46354 2.40513 3.72494 3.29354 2.67741 2.69355 4.24690 2.90347 2.73739 3.18146 2.89801 2.37887 2.77519 2.98518 4.58477 3.61503
0.03782 3.68971 4.41206 0.61958 0.77255 0.64191 0.74715
931 2.88652 4.37019 4.05193 3.66978 3.26042 3.84728 4.43781 1.34176 3.49290 1.89866 3.23881 3.94049 4.30608 3.86681 3.71057 3.33382 3.19357 1.87474 5.10472 3.81929 1224 - -
2.68618 4.42225 2.77519 2.73123 3.46354 2.40513 3.72494 3.29354 2.67741 2.69355 4.24690 2.90347 2.73739 3.18146 2.89801 2.37887 2.77519 2.98518 4.58477 3.61503
0.03782 3.68971 4.41206 0.61958 0.77255 0.64191 0.74715
932 2.53919 4.31574 3.12837 2.94944 3.94108 3.01406 4.03464 3.44720 2.99214 3.09147 4.14117 3.26964 1.25262 3.38748 3.25050 2.70343 2.96896 3.11334 5.17040 4.06284 1225 - -
2.68618 4.42225 2.77519 2.73123 3.46354 2.40513 3.72494 3.29354 2.67741 2.69355 4.24690 2.90347 2.73739 3.18146 2.89801 2.37887 2.77519 2.98518 4.58477 3.61503
0.03782 3.68971 4.41206 0.61958 0.77255 0.64191 0.74715
933 2.53919 4.31574 3.12837 2.94944 3.94108 3.01406 4.03464 3.44720 2.99214 3.09147 4.14117 3.26964 1.25262 3.38748 3.25050 2.70343 2.96896 3.11334 5.17040 4.06284 1226 - -
2.68618 4.42225 2.77519 2.73123 3.46354 2.40513 3.72494 3.29354 2.67741 2.69355 4.24690 2.90347 2.73739 3.18146 2.89801 2.37887 2.77519 2.98518 4.58477 3.61503
0.03782 3.68971 4.41206 0.61958 0.77255 0.64191 0.74715
934 3.03627 4.49697 3.98008 3.66865 1.36975 3.75169 3.82000 2.59321 3.58195 2.01239 3.35868 3.81567 4.22222 3.80445 3.74700 3.31035 3.34138 2.56812 4.14110 2.53835 1227 - -
2.68618 4.42225 2.77519 2.73123 3.46354 2.40513 3.72494 3.29354 2.67741 2.69355 4.24690 2.90347 2.73739 3.18146 2.89801 2.37887 2.77519 2.98518 4.58477 3.61503
0.03782 3.68971 4.41206 0.61958 0.77255 0.64191 0.74715
935 2.66384 4.26718 3.86051 3.52869 3.42541 3.52084 4.36452 2.09728 3.39277 2.15394 3.43086 3.74448 4.09895 3.77068 3.62046 3.02131 3.03475 1.29720 5.17454 3.88959 1228 - -
2.68618 4.42225 2.77519 2.73123 3.46354 2.40513 3.72494 3.29354 2.67741 2.69355 4.24690 2.90347 2.73739 3.18146 2.89801 2.37887 2.77519 2.98518 4.58477 3.61503
0.03782 3.68971 4.41206 0.61958 0.77255 0.64191 0.74715
936 2.81190 4.64511 3.28512 2.84967 3.95281 3.32202 3.71995 3.48986 2.20826 3.04039 4.05567 3.21864 3.84267 2.95856 1.30366 2.91066 3.09663 3.22431 5.11666 3.92964 1229 - -
2.68618 4.42225 2.77519 2.73123 3.46354 2.40513 3.72494 3.29354 2.67741 2.69355 4.24690 2.90347 2.73739 3.18146 2.89801 2.37887 2.77519 2.98518 4.58477 3.61503
0.03782 3.68971 4.41206 0.61958 0.77255 0.64191 0.74715
937 2.88652 4.37019 4.05193 3.66978 3.26042 3.84728 4.43781 1.34176 3.49290 1.89866 3.23881 3.94049 4.30608 3.86681 3.71057 3.33382 3.19357 1.87474 5.10472 3.81929 1230 - -
2.68618 4.42225 2.77519 2.73123 3.46354 2.40513 3.72494 3.29354 2.67741 2.69355 4.24690 2.90347 2.73739 3.18146 2.89801 2.37887 2.77519 2.98518 4.58477 3.61503
0.03782 3.68971 4.41206 0.61958 0.77255 0.64191 0.74715
938 2.40719 1.40547 3.87078 3.57616 3.61829 3.05978 4.26483 2.73897 3.40375 2.65893 3.77889 3.61209 3.75249 3.75916 3.55770 2.67680 2.86014 2.49438 5.06450 3.90470 1231 - -
2.68618 4.42225 2.77519 2.73123 3.46354 2.40513 3.72494 3.29354 2.67741 2.69355 4.24690 2.90347 2.73739 3.18146 2.89801 2.37887 2.77519 2.98518 4.58477 3.61503
0.03782 3.68971 4.41206 0.61958 0.77255 0.64191 0.74715
939 2.80936 4.85412 2.44285 1.32179 4.18024 3.16567 3.75082 3.59744 2.60890 3.24971 4.24291 2.85883 3.76479 2.97779 2.98976 2.81828 3.11612 3.30456 5.38821 4.12184 1232 - -

2.68618 4.42225 2.77519 2.73123 3.46354 2.40513 3.72494 3.29354 2.67741 2.69355 4.24690 2.90347 2.73739 3.18146 2.89801 2.37887 2.77519 2.98518 4.58477 3.61503
0.03782 3.68971 4.41206 0.61958 0.77255 0.64191 0.74715

940 2.80535 4.87482 1.22337 2.25740 4.23128 3.08281 3.79410 3.78082 2.84781 3.42775 4.41212 2.79951 3.72433 3.04114 3.32941 2.79671 3.14408 3.44355 5.43550 4.14798 1233 - -
2.68618 4.42225 2.77519 2.73123 3.46354 2.40513 3.72494 3.29354 2.67741 2.69355 4.24690 2.90347 2.73739 3.18146 2.89801 2.37887 2.77519 2.98518 4.58477 3.61503
0.03782 3.68971 4.41206 0.61958 0.77255 0.64191 0.74715

941 3.06047 4.49594 3.82876 3.49619 2.52194 3.53862 3.70765 3.08393 3.22453 2.50664 3.72585 3.68429 4.04410 3.62345 3.38267 3.26521 3.35391 2.96950 1.35528 2.53548 1234 - -
2.68618 4.42225 2.77519 2.73123 3.46354 2.40513 3.72494 3.29354 2.67741 2.69355 4.24690 2.90347 2.73739 3.18146 2.89801 2.37887 2.77519 2.98518 4.58477 3.61503
0.03782 3.68971 4.41206 0.61958 0.77255 0.64191 0.74715

942 2.81419 4.75052 2.97802 2.64754 4.09431 3.31300 3.68190 3.51431 1.32517 3.12542 4.10705 3.07652 3.83488 2.89173 2.43430 2.87431 3.08699 3.23582 5.23321 4.02251 1235 - -
2.68618 4.42225 2.77519 2.73123 3.46354 2.40513 3.72494 3.29354 2.67741 2.69355 4.24690 2.90347 2.73739 3.18146 2.89801 2.37887 2.77519 2.98518 4.58477 3.61503
0.03782 3.68971 4.41206 0.61958 0.77255 0.64191 0.74715

943 2.33123 4.17207 3.27908 3.03470 3.81909 3.01404 4.06407 2.92517 2.97384 2.79402 3.86593 3.26778 3.70693 3.38105 3.23550 2.53154 1.52801 2.64069 5.27454 4.03869 1236 - -
2.68618 4.42225 2.77519 2.73123 3.46354 2.40513 3.72494 3.29354 2.67741 2.69355 4.24690 2.90347 2.73739 3.18146 2.89801 2.37887 2.77519 2.98518 4.58477 3.61503
0.03782 3.68971 4.41206 0.61958 0.77255 0.64191 0.74715

944 2.46513 4.24664 3.09956 2.98169 4.10469 1.07816 4.10397 3.66750 3.13599 3.34383 4.32651 3.26662 3.63194 3.47710 3.38330 2.63370 2.93263 3.23311 5.23953 4.20371 1237 - -
2.68618 4.42225 2.77519 2.73123 3.46354 2.40513 3.72494 3.29354 2.67741 2.69355 4.24690 2.90347 2.73739 3.18146 2.89801 2.37887 2.77519 2.98518 4.58477 3.61503
0.03782 3.68971 4.41206 0.61958 0.77255 0.64191 0.74715

945 2.95570 4.47801 3.88699 3.53725 3.08153 3.73572 4.25048 2.29059 3.30641 1.14168 3.18203 3.82854 4.21045 3.71971 3.50815 3.30044 3.26294 2.27804 4.88351 3.56168 1238 - -
2.68618 4.42225 2.77519 2.73123 3.46354 2.40513 3.72494 3.29354 2.67741 2.69355 4.24690 2.90347 2.73739 3.18146 2.89801 2.37887 2.77519 2.98518 4.58477 3.61503
0.03782 3.68971 4.41206 0.61958 0.77255 0.64191 0.74715

946 2.81419 4.75052 2.97802 2.64754 4.09431 3.31300 3.68190 3.51431 1.32517 3.12542 4.10705 3.07652 3.83488 2.89173 2.43430 2.87431 3.08699 3.23582 5.23321 4.02251 1239 - -
2.68618 4.42225 2.77519 2.73123 3.46354 2.40513 3.72494 3.29354 2.67741 2.69355 4.24690 2.90347 2.73739 3.18146 2.89801 2.37887 2.77519 2.98518 4.58477 3.61503
0.03782 3.68971 4.41206 0.61958 0.77255 0.64191 0.74715

947 2.95570 4.47801 3.88699 3.53725 3.08153 3.73572 4.25048 2.29059 3.30641 1.14168 3.18203 3.82854 4.21045 3.71971 3.50815 3.30044 3.26294 2.27804 4.88351 3.56168 1240 - -
2.68618 4.42225 2.77519 2.73123 3.46354 2.40513 3.72494 3.29354 2.67741 2.69355 4.24690 2.90347 2.73739 3.18146 2.89801 2.37887 2.77519 2.98518 4.58477 3.61503
0.03782 3.68971 4.41206 0.61958 0.77255 0.64191 0.74715

948 2.66384 4.26718 3.86051 3.52869 3.42541 3.52084 4.36452 2.09728 3.39277 2.15394 3.43086 3.74448 4.09895 3.77068 3.62046 3.02131 3.03475 1.29720 5.17454 3.88959 1241 - -
2.68618 4.42225 2.77519 2.73123 3.46354 2.40513 3.72494 3.29354 2.67741 2.69355 4.24690 2.90347 2.73739 3.18146 2.89801 2.37887 2.77519 2.98518 4.58477 3.61503
0.03782 3.68971 4.41206 0.61958 0.77255 0.64191 0.74715

949 2.80535 4.87482 1.22337 2.25740 4.23128 3.08281 3.79410 3.78082 2.84781 3.42775 4.41212 2.79951 3.72433 3.04114 3.32941 2.79671 3.14408 3.44355 5.43550 4.14798 1242 - -
2.68618 4.42225 2.77519 2.73123 3.46354 2.40513 3.72494 3.29354 2.67741 2.69355 4.24690 2.90347 2.73739 3.18146 2.89801 2.37887 2.77519 2.98518 4.58477 3.61503
0.03782 3.68971 4.41206 0.61958 0.77255 0.64191 0.74715

950 2.66384 4.26718 3.86051 3.52869 3.42541 3.52084 4.36452 2.09728 3.39277 2.15394 3.43086 3.74448 4.09895 3.77068 3.62046 3.02131 3.03475 1.29720 5.17454 3.88959 1243 - -
2.68618 4.42225 2.77519 2.73123 3.46354 2.40513 3.72494 3.29354 2.67741 2.69355 4.24690 2.90347 2.73739 3.18146 2.89801 2.37887 2.77519 2.98518 4.58477 3.61503
0.03782 3.68971 4.41206 0.61958 0.77255 0.64191 0.74715

951 2.24189 4.15412 3.06133 2.87814 3.89444 2.88796 3.98828 3.39642 2.93811 3.13522 4.08514 3.12721 3.61351 3.30518 3.22334 1.40697 2.70989 2.97363 5.28456 3.97893 1244 - -
2.68618 4.42225 2.77519 2.73123 3.46354 2.40513 3.72494 3.29354 2.67741 2.69355 4.24690 2.90347 2.73739 3.18146 2.89801 2.37887 2.77519 2.98518 4.58477 3.61503
0.03782 3.68971 4.41206 0.61958 0.77255 0.64191 0.74715
952 2.80535 4.87482 1.22337 2.25740 4.23128 3.08281 3.79410 3.78082 2.84781 3.42775 4.41212 2.79951 3.72433 3.04114 3.32941 2.79671 3.14408 3.44355 5.43550 4.14798 1245 - -
2.68618 4.42225 2.77519 2.73123 3.46354 2.40513 3.72494 3.29354 2.67741 2.69355 4.24690 2.90347 2.73739 3.18146 2.89801 2.37887 2.77519 2.98518 4.58477 3.61503
0.03782 3.68971 4.41206 0.61958 0.77255 0.64191 0.74715
953 2.80936 4.85412 2.44285 1.32179 4.18024 3.16567 3.75082 3.59744 2.60890 3.24971 4.24291 2.85883 3.76479 2.97779 2.98976 2.81828 3.11612 3.30456 5.38821 4.12184 1246 - -
2.68618 4.42225 2.77519 2.73123 3.46354 2.40513 3.72494 3.29354 2.67741 2.69355 4.24690 2.90347 2.73739 3.18146 2.89801 2.37887 2.77519 2.98518 4.58477 3.61503
0.03782 3.68971 4.41206 0.61958 0.77255 0.64191 0.74715
954 2.95570 4.47801 3.88699 3.53725 3.08153 3.73572 4.25048 2.29059 3.30641 1.14168 3.18203 3.82854 4.21045 3.71971 3.50815 3.30044 3.26294 2.27804 4.88351 3.56168 1247 - -
2.68618 4.42225 2.77519 2.73123 3.46354 2.40513 3.72494 3.29354 2.67741 2.69355 4.24690 2.90347 2.73739 3.18146 2.89801 2.37887 2.77519 2.98518 4.58477 3.61503
0.02561 3.67751 * 0.61958 0.77255 0.00000 *
//

HMMER3/b [3.0 | March 2010]

NAME Xyloglucan galactosyltransferases

LENG 1381

ALPH amino

RF no

CS no

MAP yes

DATE Mon Aug 8 15:41:52 2016

NSEQ 39

EFFN 2.251831

CKSUM 1890529988

STATS LOCAL MSV -13.4866 0.69492

STATS LOCAL VITERBI -14.6177 0.69492

STATS LOCAL FORWARD -7.5045 0.69492

HMM A C D E F G H I K L M N P Q R S T V W Y

m->m m->i m->d i->m i->i d->m d->d

COMPO 2.56739 4.05183 2.97743 2.71268 3.19413 2.95152 3.66759 2.81321 2.71213 2.41548 3.59582 3.09096 3.35588 3.19634 2.93930 2.51522 2.84259 2.62924 4.60901 3.46550

2.68618 4.42225 2.77519 2.73123 3.46354 2.40513 3.72494 3.29354 2.67741 2.69355 4.24690 2.90347 2.73739 3.18146 2.89801 2.37887 2.77519 2.98518 4.58477 3.61503

0.03157 3.86699 4.58934 0.61958 0.77255 0.00000 *

1 2.96489 4.51452 4.04353 3.63658 3.20533 3.82998 4.37299 2.38038 3.40496 1.80083 1.38846 3.91475 4.29056 3.78719 3.61092 3.31343 3.27009 2.38586 5.04542 3.80671 1 - -

2.68618 4.42225 2.77519 2.73123 3.46354 2.40513 3.72494 3.29354 2.67741 2.69355 4.24690 2.90347 2.73739 3.18146 2.89801 2.37887 2.77519 2.98518 4.58477 3.61503

0.03157 3.86699 4.58934 0.61958 0.77255 0.48576 0.95510

2 2.31230 4.23263 3.20165 3.01716 4.07082 2.96261 4.12717 3.59269 3.09056 3.31334 4.23988 3.24282 3.70096 3.44030 3.37676 1.09934 2.79202 3.12899 5.44375 4.15432 2 - -

2.68618 4.42225 2.77519 2.73123 3.46354 2.40513 3.72494 3.29354 2.67741 2.69355 4.24690 2.90347 2.73739 3.18146 2.89801 2.37887 2.77519 2.98518 4.58477 3.61503

0.03157 3.86699 4.58934 0.61958 0.77255 0.48576 0.95510

3 2.40976 4.25482 3.42322 3.17496 3.96403 3.09429 4.19270 3.09049 3.11783 2.95418 4.00504 3.38522 3.79760 3.51292 3.37678 2.61097 1.18122 2.78606 5.40231 4.18065 3 - -

2.68618 4.42225 2.77519 2.73123 3.46354 2.40513 3.72494 3.29354 2.67741 2.69355 4.24690 2.90347 2.73739 3.18146 2.89801 2.37887 2.77519 2.98518 4.58477 3.61503

0.03157 3.86699 4.58934 0.61958 0.77255 0.48576 0.95510

4 2.80376 4.34788 4.11775 3.78070 3.54607 3.71822 4.58336 2.05053 3.64840 2.23405 3.51204 3.97141 4.27903 4.00859 3.86143 3.22141 3.16544 1.03182 5.32959 4.06087 4 - -

2.68618 4.42225 2.77519 2.73123 3.46354 2.40513 3.72494 3.29354 2.67741 2.69355 4.24690 2.90347 2.73739 3.18146 2.89801 2.37887 2.77519 2.98518 4.58477 3.61503

0.03157 3.86699 4.58934 0.61958 0.77255 0.48576 0.95510

5 2.71888 4.71528 2.70526 2.59018 4.07794 3.18029 3.91656 3.78713 2.81979 3.43461 4.38989 1.16778 3.83188 3.18930 3.17427 2.78628 3.10056 3.40989 5.38448 4.02666 5 - -

2.68618 4.42225 2.77519 2.73123 3.46354 2.40513 3.72494 3.29354 2.67741 2.69355 4.24690 2.90347 2.73739 3.18146 2.89801 2.37887 2.77519 2.98518 4.58477 3.61503

0.03157 3.86699 4.58934 0.61958 0.77255 0.48576 0.95510

6 2.98886 4.80727 3.12036 2.87975 3.28089 3.44287 1.15410 3.69093 2.72295 3.18558 4.22810 3.28930 3.99510 3.24591 2.98899 3.05959 3.29203 3.42616 4.74602 3.23190 6 - -

2.68618 4.42225 2.77519 2.73123 3.46354 2.40513 3.72494 3.29354 2.67741 2.69355 4.24690 2.90347 2.73739 3.18146 2.89801 2.37887 2.77519 2.98518 4.58477 3.61503

0.03157 3.86699 4.58934 0.61958 0.77255 0.48576 0.95510

7 2.98886 4.80727 3.12036 2.87975 3.28089 3.44287 1.15410 3.69093 2.72295 3.18558 4.22810 3.28930 3.99510 3.24591 2.98899 3.05959 3.29203 3.42616 4.74602 3.23190 7 - -

2.68618 4.42225 2.77519 2.73123 3.46354 2.40513 3.72494 3.29354 2.67741 2.69355 4.24690 2.90347 2.73739 3.18146 2.89801 2.37887 2.77519 2.98518 4.58477 3.61503

0.03157 3.86699 4.58934 0.61958 0.77255 0.48576 0.95510

8 2.58006 0.95161 4.09776 3.83614 3.82875 3.20419 4.48443 2.97175 3.66306 2.88747 4.00858 3.82459 3.91071 4.00937 3.78840 2.85170 3.04637 2.71670 5.24602 4.11647 8 - -

2.68618 4.42225 2.77519 2.73123 3.46354 2.40513 3.72494 3.29354 2.67741 2.69355 4.24690 2.90347 2.73739 3.18146 2.89801 2.37887 2.77519 2.98518 4.58477 3.61503

0.03157 3.86699 4.58934 0.61958 0.77255 0.48576 0.95510

9 3.14912 4.61302 4.18070 3.82053 3.13362 3.97519 4.47798 2.33736 3.58833 0.88196 3.15827 4.09653 4.41149 3.95797 3.76529 3.54541 3.44362 2.37328 5.01092 3.73297 9 - -

2.68618 4.42225 2.77519 2.73123 3.46354 2.40513 3.72494 3.29354 2.67741 2.69355 4.24690 2.90347 2.73739 3.18146 2.89801 2.37887 2.77519 2.98518 4.58477 3.61503

0.03157 3.86699 4.58934 0.61958 0.77255 0.48576 0.95510

10 3.14912 4.61302 4.18070 3.82053 3.13362 3.97519 4.47798 2.33736 3.58833 0.88196 3.15827 4.09653 4.41149 3.95797 3.76529 3.54541 3.44362 2.37328 5.01092 3.73297 10 - -

2.68618 4.42225 2.77519 2.73123 3.46354 2.40513 3.72494 3.29354 2.67741 2.69355 4.24690 2.90347 2.73739 3.18146 2.89801 2.37887 2.77519 2.98518 4.58477 3.61503

0.03157 3.86699 4.58934 0.61958 0.77255 0.48576 0.95510

11 2.71888 4.71528 2.70526 2.59018 4.07794 3.18029 3.91656 3.78713 2.81979 3.43461 4.38989 1.16778 3.83188 3.18930 3.17427 2.78628 3.10056 3.40989 5.38448 4.02666 11 - -

2.68618 4.42225 2.77519 2.73123 3.46354 2.40513 3.72494 3.29354 2.67741 2.69355 4.24690 2.90347 2.73739 3.18146 2.89801 2.37887 2.77519 2.98518 4.58477 3.61503

0.03157 3.86699 4.58934 0.61958 0.77255 0.48576 0.95510

12 3.25138 4.65475 4.23121 3.94466 1.02072 3.94245 3.87481 2.77574 3.85183 2.14705 3.50670 4.01301 4.40115 4.02131 3.97290 3.52160 3.55251 2.76604 4.12549 2.49318 12 - -

2.68618 4.42225 2.77519 2.73123 3.46354 2.40513 3.72494 3.29354 2.67741 2.69355 4.24690 2.90347 2.73739 3.18146 2.89801 2.37887 2.77519 2.98518 4.58477 3.61503

0.03157 3.86699 4.58934 0.61958 0.77255 0.48576 0.95510

13 2.74420 4.50247 3.35077 3.18900 4.18468 3.19029 4.26692 3.72867 3.24583 3.35611 4.40571 3.49627 0.83787 3.63478 3.49200 2.91151 3.18532 3.37467 5.37275 4.30526 13 - -

2.68618 4.42225 2.77519 2.73123 3.46354 2.40513 3.72494 3.29354 2.67741 2.69355 4.24690 2.90347 2.73739 3.18146 2.89801 2.37887 2.77519 2.98518 4.58477 3.61503

0.03157 3.86699 4.58934 0.61958 0.77255 0.48576 0.95510

14 2.98886 4.80727 3.12036 2.87975 3.28089 3.44287 1.15410 3.69093 2.72295 3.18558 4.22810 3.28930 3.99510 3.24591 2.98899 3.05959 3.29203 3.42616 4.74602 3.23190 14 - -

2.68618 4.42225 2.77519 2.73123 3.46354 2.40513 3.72494 3.29354 2.67741 2.69355 4.24690 2.90347 2.73739 3.18146 2.89801 2.37887 2.77519 2.98518 4.58477 3.61503

0.03157 3.86699 4.58934 0.61958 0.77255 0.48576 0.95510

15 3.02684 4.44709 4.32791 3.94350 3.37049 4.06063 4.67263 1.08471 3.77153 1.95764 3.30670 4.18960 4.49236 4.12038 3.96872 3.55539 3.32610 1.84575 5.26019 3.99802 15 - -

2.68618 4.42225 2.77519 2.73123 3.46354 2.40513 3.72494 3.29354 2.67741 2.69355 4.24690 2.90347 2.73739 3.18146 2.89801 2.37887 2.77519 2.98518 4.58477 3.61503

0.03157 3.86699 4.58934 0.61958 0.77255 0.48576 0.95510

16 2.74420 4.50247 3.35077 3.18900 4.18468 3.19029 4.26692 3.72867 3.24583 3.35611 4.40571 3.49627 0.83787 3.63478 3.49200 2.91151 3.18532 3.37467 5.37275 4.30526 16 - -

2.68618 4.42225 2.77519 2.73123 3.46354 2.40513 3.72494 3.29354 2.67741 2.69355 4.24690 2.90347 2.73739 3.18146 2.89801 2.37887 2.77519 2.98518 4.58477 3.61503

0.03157 3.86699 4.58934 0.61958 0.77255 0.48576 0.95510

17 2.74420 4.50247 3.35077 3.18900 4.18468 3.19029 4.26692 3.72867 3.24583 3.35611 4.40571 3.49627 0.83787 3.63478 3.49200 2.91151 3.18532 3.37467 5.37275 4.30526 17 - -

2.68618 4.42225 2.77519 2.73123 3.46354 2.40513 3.72494 3.29354 2.67741 2.69355 4.24690 2.90347 2.73739 3.18146 2.89801 2.37887 2.77519 2.98518 4.58477 3.61503

0.03157 3.86699 4.58934 0.61958 0.77255 0.48576 0.95510

18 2.31230 4.23263 3.20165 3.01716 4.07082 2.96261 4.12717 3.59269 3.09056 3.31334 4.23988 3.24282 3.70096 3.44030 3.37676 1.09934 2.79202 3.12899 5.44375 4.15432 18 - -

2.68618 4.42225 2.77519 2.73123 3.46354 2.40513 3.72494 3.29354 2.67741 2.69355 4.24690 2.90347 2.73739 3.18146 2.89801 2.37887 2.77519 2.98518 4.58477 3.61503

0.03157 3.86699 4.58934 0.61958 0.77255 0.48576 0.95510

19 3.02684 4.44709 4.32791 3.94350 3.37049 4.06063 4.67263 1.08471 3.77153 1.95764 3.30670 4.18960 4.49236 4.12038 3.96872 3.55539 3.32610 1.84575 5.26019 3.99802 19 - -

2.68618 4.42225 2.77519 2.73123 3.46354 2.40513 3.72494 3.29354 2.67741 2.69355 4.24690 2.90347 2.73739 3.18146 2.89801 2.37887 2.77519 2.98518 4.58477 3.61503

0.03157 3.86699 4.58934 0.61958 0.77255 0.48576 0.95510

20 2.74420 4.50247 3.35077 3.18900 4.18468 3.19029 4.26692 3.72867 3.24583 3.35611 4.40571 3.49627 0.83787 3.63478 3.49200 2.91151 3.18532 3.37467 5.37275 4.30526 20 - -

2.68618 4.42225 2.77519 2.73123 3.46354 2.40513 3.72494 3.29354 2.67741 2.69355 4.24690 2.90347 2.73739 3.18146 2.89801 2.37887 2.77519 2.98518 4.58477 3.61503

0.03157 3.86699 4.58934 0.61958 0.77255 0.48576 0.95510

21 2.74420 4.50247 3.35077 3.18900 4.18468 3.19029 4.26692 3.72867 3.24583 3.35611 4.40571 3.49627 0.83787 3.63478 3.49200 2.91151 3.18532 3.37467 5.37275 4.30526 21 - -

2.68618 4.42225 2.77519 2.73123 3.46354 2.40513 3.72494 3.29354 2.67741 2.69355 4.24690 2.90347 2.73739 3.18146 2.89801 2.37887 2.77519 2.98518 4.58477 3.61503

0.03157 3.86699 4.58934 0.61958 0.77255 0.48576 0.95510

22 2.71888 4.71528 2.70526 2.59018 4.07794 3.18029 3.91656 3.78713 2.81979 3.43461 4.38989 1.16778 3.83188 3.18930 3.17427 2.78628 3.10056 3.40989 5.38448 4.02666 22 - -

2.68618 4.42225 2.77519 2.73123 3.46354 2.40513 3.72494 3.29354 2.67741 2.69355 4.24690 2.90347 2.73739 3.18146 2.89801 2.37887 2.77519 2.98518 4.58477 3.61503

0.03157 3.86699 4.58934 0.61958 0.77255 0.48576 0.95510

23 2.98886 4.80727 3.12036 2.87975 3.28089 3.44287 1.15410 3.69093 2.72295 3.18558 4.22810 3.28930 3.99510 3.24591 2.98899 3.05959 3.29203 3.42616 4.74602 3.23190 23 - -

2.68618 4.42225 2.77519 2.73123 3.46354 2.40513 3.72494 3.29354 2.67741 2.69355 4.24690 2.90347 2.73739 3.18146 2.89801 2.37887 2.77519 2.98518 4.58477 3.61503

0.03157 3.86699 4.58934 0.61958 0.77255 0.48576 0.95510

24 2.99928 4.83880 3.48937 3.00157 4.19200 3.48833 3.82030 3.73332 2.23516 3.25025 4.25056 3.36173 3.99118 3.04089 0.98678 3.08371 3.26560 3.45751 5.27725 4.12054 24 - -

2.68618 4.42225 2.77519 2.73123 3.46354 2.40513 3.72494 3.29354 2.67741 2.69355 4.24690 2.90347 2.73739 3.18146 2.89801 2.37887 2.77519 2.98518 4.58477 3.61503

0.03157 3.86699 4.58934 0.61958 0.77255 0.48576 0.95510

25 2.74420 4.50247 3.35077 3.18900 4.18468 3.19029 4.26692 3.72867 3.24583 3.35611 4.40571 3.49627 0.83787 3.63478 3.49200 2.91151 3.18532 3.37467 5.37275 4.30526 25 - -

2.68618 4.42225 2.77519 2.73123 3.46354 2.40513 3.72494 3.29354 2.67741 2.69355 4.24690 2.90347 2.73739 3.18146 2.89801 2.37887 2.77519 2.98518 4.58477 3.61503

0.03157 3.86699 4.58934 0.61958 0.77255 0.48576 0.95510

26 2.96711 4.93068 3.14273 2.76522 4.31586 3.45716 3.75616 3.74479 1.04579 3.31106 4.27280 3.18993 3.95788 2.94794 2.43058 3.00945 3.22078 3.45288 5.37276 4.18474 26 - -

2.68618 4.42225 2.77519 2.73123 3.46354 2.40513 3.72494 3.29354 2.67741 2.69355 4.24690 2.90347 2.73739 3.18146 2.89801 2.37887 2.77519 2.98518 4.58477 3.61503

0.03157 3.86699 4.58934 0.61958 0.77255 0.48576 0.95510

27 3.14912 4.61302 4.18070 3.82053 3.13362 3.97519 4.47798 2.33736 3.58833 0.88196 3.15827 4.09653 4.41149 3.95797 3.76529 3.54541 3.44362 2.37328 5.01092 3.73297 27 - -

2.68618 4.42225 2.77519 2.73123 3.46354 2.40513 3.72494 3.29354 2.67741 2.69355 4.24690 2.90347 2.73739 3.18146 2.89801 2.37887 2.77519 2.98518 4.58477 3.61503

0.03157 3.86699 4.58934 0.61958 0.77255 0.48576 0.95510

28 3.14912 4.61302 4.18070 3.82053 3.13362 3.97519 4.47798 2.33736 3.58833 0.88196 3.15827 4.09653 4.41149 3.95797 3.76529 3.54541 3.44362 2.37328 5.01092 3.73297 28 - -

2.68618 4.42225 2.77519 2.73123 3.46354 2.40513 3.72494 3.29354 2.67741 2.69355 4.24690 2.90347 2.73739 3.18146 2.89801 2.37887 2.77519 2.98518 4.58477 3.61503

0.03157 3.86699 4.58934 0.61958 0.77255 0.48576 0.95510

29 2.31230 4.23263 3.20165 3.01716 4.07082 2.96261 4.12717 3.59269 3.09056 3.31334 4.23988 3.24282 3.70096 3.44030 3.37676 1.09934 2.79202 3.12899 5.44375 4.15432 29 - -

2.68618 4.42225 2.77519 2.73123 3.46354 2.40513 3.72494 3.29354 2.67741 2.69355 4.24690 2.90347 2.73739 3.18146 2.89801 2.37887 2.77519 2.98518 4.58477 3.61503
0.03157 3.86699 4.58934 0.61958 0.77255 0.48576 0.95510
30 2.31230 4.23263 3.20165 3.01716 4.07082 2.96261 4.12717 3.59269 3.09056 3.31334 4.23988 3.24282 3.70096 3.44030 3.37676 1.09934 2.79202 3.12899 5.44375 4.15432 30 - -
2.68618 4.42225 2.77519 2.73123 3.46354 2.40513 3.72494 3.29354 2.67741 2.69355 4.24690 2.90347 2.73739 3.18146 2.89801 2.37887 2.77519 2.98518 4.58477 3.61503
0.03157 3.86699 4.58934 0.61958 0.77255 0.48576 0.95510
31 3.14912 4.61302 4.18070 3.82053 3.13362 3.97519 4.47798 2.33736 3.58833 0.88196 3.15827 4.09653 4.41149 3.95797 3.76529 3.54541 3.44362 2.37328 5.01092 3.73297 31 - -
2.68618 4.42225 2.77519 2.73123 3.46354 2.40513 3.72494 3.29354 2.67741 2.69355 4.24690 2.90347 2.73739 3.18146 2.89801 2.37887 2.77519 2.98518 4.58477 3.61503
0.03157 3.86699 4.58934 0.61958 0.77255 0.48576 0.95510
32 2.31230 4.23263 3.20165 3.01716 4.07082 2.96261 4.12717 3.59269 3.09056 3.31334 4.23988 3.24282 3.70096 3.44030 3.37676 1.09934 2.79202 3.12899 5.44375 4.15432 32 - -
2.68618 4.42225 2.77519 2.73123 3.46354 2.40513 3.72494 3.29354 2.67741 2.69355 4.24690 2.90347 2.73739 3.18146 2.89801 2.37887 2.77519 2.98518 4.58477 3.61503
0.03157 3.86699 4.58934 0.61958 0.77255 0.48576 0.95510
33 3.14912 4.61302 4.18070 3.82053 3.13362 3.97519 4.47798 2.33736 3.58833 0.88196 3.15827 4.09653 4.41149 3.95797 3.76529 3.54541 3.44362 2.37328 5.01092 3.73297 33 - -
2.68618 4.42225 2.77519 2.73123 3.46354 2.40513 3.72494 3.29354 2.67741 2.69355 4.24690 2.90347 2.73739 3.18146 2.89801 2.37887 2.77519 2.98518 4.58477 3.61503
0.03157 3.86699 4.58934 0.61958 0.77255 0.48576 0.95510
34 3.23918 4.73039 3.83015 3.55850 2.29358 3.82376 3.61597 3.24916 3.41618 2.70450 3.92476 3.71671 4.29772 3.72354 3.60101 3.37642 3.52583 3.11945 3.93713 0.97494 34 - -
2.68618 4.42225 2.77519 2.73123 3.46354 2.40513 3.72494 3.29354 2.67741 2.69355 4.24690 2.90347 2.73739 3.18146 2.89801 2.37887 2.77519 2.98518 4.58477 3.61503
0.03157 3.86699 4.58934 0.61958 0.77255 0.48576 0.95510
35 2.99928 4.83880 3.48937 3.00157 4.19200 3.48833 3.82030 3.73332 2.23516 3.25025 4.25056 3.36173 3.99118 3.04089 0.98678 3.08371 3.26560 3.45751 5.27725 4.12054 35 - -
2.68618 4.42225 2.77519 2.73123 3.46354 2.40513 3.72494 3.29354 2.67741 2.69355 4.24690 2.90347 2.73739 3.18146 2.89801 2.37887 2.77519 2.98518 4.58477 3.61503
0.03157 3.86699 4.58934 0.61958 0.77255 0.48576 0.95510
36 2.96711 4.93068 3.14273 2.76522 4.31586 3.45716 3.75616 3.74479 1.04579 3.31106 4.27280 3.18993 3.95788 2.94794 2.43058 3.00945 3.22078 3.45288 5.37276 4.18474 36 - -
2.68618 4.42225 2.77519 2.73123 3.46354 2.40513 3.72494 3.29354 2.67741 2.69355 4.24690 2.90347 2.73739 3.18146 2.89801 2.37887 2.77519 2.98518 4.58477 3.61503
0.03157 3.86699 4.58934 0.61958 0.77255 0.48576 0.95510
37 2.74420 4.50247 3.35077 3.18900 4.18468 3.19029 4.26692 3.72867 3.24583 3.35611 4.40571 3.49627 0.83787 3.63478 3.49200 2.91151 3.18532 3.37467 5.37275 4.30526 37 - -
2.68618 4.42225 2.77519 2.73123 3.46354 2.40513 3.72494 3.29354 2.67741 2.69355 4.24690 2.90347 2.73739 3.18146 2.89801 2.37887 2.77519 2.98518 4.58477 3.61503
0.03157 3.86699 4.58934 0.61958 0.77255 0.48576 0.95510
38 2.94758 5.05751 2.48529 1.03318 4.40304 3.27053 3.85685 3.85586 2.73669 3.47385 4.44812 2.92979 3.87486 3.07262 3.14497 2.93053 3.25177 3.54026 5.57565 4.29788 38 - -
2.68618 4.42225 2.77519 2.73123 3.46354 2.40513 3.72494 3.29354 2.67741 2.69355 4.24690 2.90347 2.73739 3.18146 2.89801 2.37887 2.77519 2.98518 4.58477 3.61503
0.03157 3.86699 4.58934 0.61958 0.77255 0.48576 0.95510
39 2.99928 4.83880 3.48937 3.00157 4.19200 3.48833 3.82030 3.73332 2.23516 3.25025 4.25056 3.36173 3.99118 3.04089 0.98678 3.08371 3.26560 3.45751 5.27725 4.12054 39 - -
2.68618 4.42225 2.77519 2.73123 3.46354 2.40513 3.72494 3.29354 2.67741 2.69355 4.24690 2.90347 2.73739 3.18146 2.89801 2.37887 2.77519 2.98518 4.58477 3.61503
0.03157 3.86699 4.58934 0.61958 0.77255 0.48576 0.95510
40 3.14912 4.61302 4.18070 3.82053 3.13362 3.97519 4.47798 2.33736 3.58833 0.88196 3.15827 4.09653 4.41149 3.95797 3.76529 3.54541 3.44362 2.37328 5.01092 3.73297 40 - -
2.68618 4.42225 2.77519 2.73123 3.46354 2.40513 3.72494 3.29354 2.67741 2.69355 4.24690 2.90347 2.73739 3.18146 2.89801 2.37887 2.77519 2.98518 4.58477 3.61503
0.03157 3.86699 4.58934 0.61958 0.77255 0.48576 0.95510

41 3.25138 4.65475 4.23121 3.94466 1.02072 3.94245 3.87481 2.77574 3.85183 2.14705 3.50670 4.01301 4.40115 4.02131 3.97290 3.52160 3.55251 2.76604 4.12549 2.49318 41 - -
2.68618 4.42225 2.77519 2.73123 3.46354 2.40513 3.72494 3.29354 2.67741 2.69355 4.24690 2.90347 2.73739 3.18146 2.89801 2.37887 2.77519 2.98518 4.58477 3.61503
0.03157 3.86699 4.58934 0.61958 0.77255 0.48576 0.95510
42 1.02168 4.18931 3.45704 3.24808 4.04693 3.01400 4.27077 3.17162 3.26419 3.05349 4.08591 3.39907 3.75270 3.60586 3.51510 2.53965 2.81663 2.82697 5.47463 4.27427 42 - -
2.68618 4.42225 2.77519 2.73123 3.46354 2.40513 3.72494 3.29354 2.67741 2.69355 4.24690 2.90347 2.73739 3.18146 2.89801 2.37887 2.77519 2.98518 4.58477 3.61503
0.03157 3.86699 4.58934 0.61958 0.77255 0.48576 0.95510
43 3.14912 4.61302 4.18070 3.82053 3.13362 3.97519 4.47798 2.33736 3.58833 0.88196 3.15827 4.09653 4.41149 3.95797 3.76529 3.54541 3.44362 2.37328 5.01092 3.73297 43 - -
2.68618 4.42225 2.77519 2.73123 3.46354 2.40513 3.72494 3.29354 2.67741 2.69355 4.24690 2.90347 2.73739 3.18146 2.89801 2.37887 2.77519 2.98518 4.58477 3.61503
0.03157 3.86699 4.58934 0.61958 0.77255 0.48576 0.95510
44 2.31230 4.23263 3.20165 3.01716 4.07082 2.96261 4.12717 3.59269 3.09056 3.31334 4.23988 3.24282 3.70096 3.44030 3.37676 1.09934 2.79202 3.12899 5.44375 4.15432 44 - -
2.68618 4.42225 2.77519 2.73123 3.46354 2.40513 3.72494 3.29354 2.67741 2.69355 4.24690 2.90347 2.73739 3.18146 2.89801 2.37887 2.77519 2.98518 4.58477 3.61503
0.03157 3.86699 4.58934 0.61958 0.77255 0.48576 0.95510
45 1.02168 4.18931 3.45704 3.24808 4.04693 3.01400 4.27077 3.17162 3.26419 3.05349 4.08591 3.39907 3.75270 3.60586 3.51510 2.53965 2.81663 2.82697 5.47463 4.27427 45 - -
2.68618 4.42225 2.77519 2.73123 3.46354 2.40513 3.72494 3.29354 2.67741 2.69355 4.24690 2.90347 2.73739 3.18146 2.89801 2.37887 2.77519 2.98518 4.58477 3.61503
0.03157 3.86699 4.58934 0.61958 0.77255 0.48576 0.95510
46 2.31230 4.23263 3.20165 3.01716 4.07082 2.96261 4.12717 3.59269 3.09056 3.31334 4.23988 3.24282 3.70096 3.44030 3.37676 1.09934 2.79202 3.12899 5.44375 4.15432 46 - -
2.68618 4.42225 2.77519 2.73123 3.46354 2.40513 3.72494 3.29354 2.67741 2.69355 4.24690 2.90347 2.73739 3.18146 2.89801 2.37887 2.77519 2.98518 4.58477 3.61503
0.03157 3.86699 4.58934 0.61958 0.77255 0.48576 0.95510
47 3.14912 4.61302 4.18070 3.82053 3.13362 3.97519 4.47798 2.33736 3.58833 0.88196 3.15827 4.09653 4.41149 3.95797 3.76529 3.54541 3.44362 2.37328 5.01092 3.73297 47 - -
2.68618 4.42225 2.77519 2.73123 3.46354 2.40513 3.72494 3.29354 2.67741 2.69355 4.24690 2.90347 2.73739 3.18146 2.89801 2.37887 2.77519 2.98518 4.58477 3.61503
0.03157 3.86699 4.58934 0.61958 0.77255 0.48576 0.95510
48 2.31230 4.23263 3.20165 3.01716 4.07082 2.96261 4.12717 3.59269 3.09056 3.31334 4.23988 3.24282 3.70096 3.44030 3.37676 1.09934 2.79202 3.12899 5.44375 4.15432 48 - -
2.68618 4.42225 2.77519 2.73123 3.46354 2.40513 3.72494 3.29354 2.67741 2.69355 4.24690 2.90347 2.73739 3.18146 2.89801 2.37887 2.77519 2.98518 4.58477 3.61503
0.03157 3.86699 4.58934 0.61958 0.77255 0.48576 0.95510
49 3.14912 4.61302 4.18070 3.82053 3.13362 3.97519 4.47798 2.33736 3.58833 0.88196 3.15827 4.09653 4.41149 3.95797 3.76529 3.54541 3.44362 2.37328 5.01092 3.73297 49 - -
2.68618 4.42225 2.77519 2.73123 3.46354 2.40513 3.72494 3.29354 2.67741 2.69355 4.24690 2.90347 2.73739 3.18146 2.89801 2.37887 2.77519 2.98518 4.58477 3.61503
0.03157 3.86699 4.58934 0.61958 0.77255 0.48576 0.95510
50 2.31230 4.23263 3.20165 3.01716 4.07082 2.96261 4.12717 3.59269 3.09056 3.31334 4.23988 3.24282 3.70096 3.44030 3.37676 1.09934 2.79202 3.12899 5.44375 4.15432 50 - -
2.68618 4.42225 2.77519 2.73123 3.46354 2.40513 3.72494 3.29354 2.67741 2.69355 4.24690 2.90347 2.73739 3.18146 2.89801 2.37887 2.77519 2.98518 4.58477 3.61503
0.03157 3.86699 4.58934 0.61958 0.77255 0.48576 0.95510
51 2.74420 4.50247 3.35077 3.18900 4.18468 3.19029 4.26692 3.72867 3.24583 3.35611 4.40571 3.49627 0.83787 3.63478 3.49200 2.91151 3.18532 3.37467 5.37275 4.30526 51 - -
2.68618 4.42225 2.77519 2.73123 3.46354 2.40513 3.72494 3.29354 2.67741 2.69355 4.24690 2.90347 2.73739 3.18146 2.89801 2.37887 2.77519 2.98518 4.58477 3.61503
0.03157 3.86699 4.58934 0.61958 0.77255 0.48576 0.95510
52 1.02168 4.18931 3.45704 3.24808 4.04693 3.01400 4.27077 3.17162 3.26419 3.05349 4.08591 3.39907 3.75270 3.60586 3.51510 2.53965 2.81663 2.82697 5.47463 4.27427 52 - -
2.68618 4.42225 2.77519 2.73123 3.46354 2.40513 3.72494 3.29354 2.67741 2.69355 4.24690 2.90347 2.73739 3.18146 2.89801 2.37887 2.77519 2.98518 4.58477 3.61503

0.03157 3.86699 4.58934 0.61958 0.77255 0.48576 0.95510

53 2.40976 4.25482 3.42322 3.17496 3.96403 3.09429 4.19270 3.09049 3.11783 2.95418 4.00504 3.38522 3.79760 3.51292 3.37678 2.61097 1.18122 2.78606 5.40231 4.18065 53 - -

2.68618 4.42225 2.77519 2.73123 3.46354 2.40513 3.72494 3.29354 2.67741 2.69355 4.24690 2.90347 2.73739 3.18146 2.89801 2.37887 2.77519 2.98518 4.58477 3.61503

0.03157 3.86699 4.58934 0.61958 0.77255 0.48576 0.95510

54 3.02684 4.44709 4.32791 3.94350 3.37049 4.06063 4.67263 1.08471 3.77153 1.95764 3.30670 4.18960 4.49236 4.12038 3.96872 3.55539 3.32610 1.84575 5.26019 3.99802 54 - -

2.68618 4.42225 2.77519 2.73123 3.46354 2.40513 3.72494 3.29354 2.67741 2.69355 4.24690 2.90347 2.73739 3.18146 2.89801 2.37887 2.77519 2.98518 4.58477 3.61503

0.03157 3.86699 4.58934 0.61958 0.77255 0.48576 0.95510

55 2.98886 4.80727 3.12036 2.87975 3.28089 3.44287 1.15410 3.69093 2.72295 3.18558 4.22810 3.28930 3.99510 3.24591 2.98899 3.05959 3.29203 3.42616 4.74602 3.23190 55 - -

2.68618 4.42225 2.77519 2.73123 3.46354 2.40513 3.72494 3.29354 2.67741 2.69355 4.24690 2.90347 2.73739 3.18146 2.89801 2.37887 2.77519 2.98518 4.58477 3.61503

0.03157 3.86699 4.58934 0.61958 0.77255 0.48576 0.95510

56 2.94758 5.05751 2.48529 1.03318 4.40304 3.27053 3.85685 3.85586 2.73669 3.47385 4.44812 2.92979 3.87486 3.07262 3.14497 2.93053 3.25177 3.54026 5.57565 4.29788 56 - -

2.68618 4.42225 2.77519 2.73123 3.46354 2.40513 3.72494 3.29354 2.67741 2.69355 4.24690 2.90347 2.73739 3.18146 2.89801 2.37887 2.77519 2.98518 4.58477 3.61503

0.03157 3.86699 4.58934 0.61958 0.77255 0.48576 0.95510

57 2.58006 0.95161 4.09776 3.83614 3.82875 3.20419 4.48443 2.97175 3.66306 2.88747 4.00858 3.82459 3.91071 4.00937 3.78840 2.85170 3.04637 2.71670 5.24602 4.11647 57 - -

2.68618 4.42225 2.77519 2.73123 3.46354 2.40513 3.72494 3.29354 2.67741 2.69355 4.24690 2.90347 2.73739 3.18146 2.89801 2.37887 2.77519 2.98518 4.58477 3.61503

0.03157 3.86699 4.58934 0.61958 0.77255 0.48576 0.95510

58 2.31230 4.23263 3.20165 3.01716 4.07082 2.96261 4.12717 3.59269 3.09056 3.31334 4.23988 3.24282 3.70096 3.44030 3.37676 1.09934 2.79202 3.12899 5.44375 4.15432 58 - -

2.68618 4.42225 2.77519 2.73123 3.46354 2.40513 3.72494 3.29354 2.67741 2.69355 4.24690 2.90347 2.73739 3.18146 2.89801 2.37887 2.77519 2.98518 4.58477 3.61503

0.03157 3.86699 4.58934 0.61958 0.77255 0.48576 0.95510

59 2.31230 4.23263 3.20165 3.01716 4.07082 2.96261 4.12717 3.59269 3.09056 3.31334 4.23988 3.24282 3.70096 3.44030 3.37676 1.09934 2.79202 3.12899 5.44375 4.15432 59 - -

2.68618 4.42225 2.77519 2.73123 3.46354 2.40513 3.72494 3.29354 2.67741 2.69355 4.24690 2.90347 2.73739 3.18146 2.89801 2.37887 2.77519 2.98518 4.58477 3.61503

0.03157 3.86699 4.58934 0.61958 0.77255 0.48576 0.95510

60 2.31230 4.23263 3.20165 3.01716 4.07082 2.96261 4.12717 3.59269 3.09056 3.31334 4.23988 3.24282 3.70096 3.44030 3.37676 1.09934 2.79202 3.12899 5.44375 4.15432 60 - -

2.68618 4.42225 2.77519 2.73123 3.46354 2.40513 3.72494 3.29354 2.67741 2.69355 4.24690 2.90347 2.73739 3.18146 2.89801 2.37887 2.77519 2.98518 4.58477 3.61503

0.03157 3.86699 4.58934 0.61958 0.77255 0.48576 0.95510

61 2.31230 4.23263 3.20165 3.01716 4.07082 2.96261 4.12717 3.59269 3.09056 3.31334 4.23988 3.24282 3.70096 3.44030 3.37676 1.09934 2.79202 3.12899 5.44375 4.15432 61 - -

2.68618 4.42225 2.77519 2.73123 3.46354 2.40513 3.72494 3.29354 2.67741 2.69355 4.24690 2.90347 2.73739 3.18146 2.89801 2.37887 2.77519 2.98518 4.58477 3.61503

0.03157 3.86699 4.58934 0.61958 0.77255 0.48576 0.95510

62 2.31230 4.23263 3.20165 3.01716 4.07082 2.96261 4.12717 3.59269 3.09056 3.31334 4.23988 3.24282 3.70096 3.44030 3.37676 1.09934 2.79202 3.12899 5.44375 4.15432 62 - -

2.68618 4.42225 2.77519 2.73123 3.46354 2.40513 3.72494 3.29354 2.67741 2.69355 4.24690 2.90347 2.73739 3.18146 2.89801 2.37887 2.77519 2.98518 4.58477 3.61503

0.03157 3.86699 4.58934 0.61958 0.77255 0.48576 0.95510

63 2.31230 4.23263 3.20165 3.01716 4.07082 2.96261 4.12717 3.59269 3.09056 3.31334 4.23988 3.24282 3.70096 3.44030 3.37676 1.09934 2.79202 3.12899 5.44375 4.15432 63 - -

2.68618 4.42225 2.77519 2.73123 3.46354 2.40513 3.72494 3.29354 2.67741 2.69355 4.24690 2.90347 2.73739 3.18146 2.89801 2.37887 2.77519 2.98518 4.58477 3.61503

0.03157 3.86699 4.58934 0.61958 0.77255 0.48576 0.95510

64 2.31230 4.23263 3.20165 3.01716 4.07082 2.96261 4.12717 3.59269 3.09056 3.31334 4.23988 3.24282 3.70096 3.44030 3.37676 1.09934 2.79202 3.12899 5.44375 4.15432 64 - -

2.68618 4.42225 2.77519 2.73123 3.46354 2.40513 3.72494 3.29354 2.67741 2.69355 4.24690 2.90347 2.73739 3.18146 2.89801 2.37887 2.77519 2.98518 4.58477 3.61503
0.03157 3.86699 4.58934 0.61958 0.77255 0.48576 0.95510
65 2.31230 4.23263 3.20165 3.01716 4.07082 2.96261 4.12717 3.59269 3.09056 3.31334 4.23988 3.24282 3.70096 3.44030 3.37676 1.09934 2.79202 3.12899 5.44375 4.15432 65 - -
2.68618 4.42225 2.77519 2.73123 3.46354 2.40513 3.72494 3.29354 2.67741 2.69355 4.24690 2.90347 2.73739 3.18146 2.89801 2.37887 2.77519 2.98518 4.58477 3.61503
0.03157 3.86699 4.58934 0.61958 0.77255 0.48576 0.95510
66 2.31230 4.23263 3.20165 3.01716 4.07082 2.96261 4.12717 3.59269 3.09056 3.31334 4.23988 3.24282 3.70096 3.44030 3.37676 1.09934 2.79202 3.12899 5.44375 4.15432 66 - -
2.68618 4.42225 2.77519 2.73123 3.46354 2.40513 3.72494 3.29354 2.67741 2.69355 4.24690 2.90347 2.73739 3.18146 2.89801 2.37887 2.77519 2.98518 4.58477 3.61503
0.03157 3.86699 4.58934 0.61958 0.77255 0.48576 0.95510
67 2.31230 4.23263 3.20165 3.01716 4.07082 2.96261 4.12717 3.59269 3.09056 3.31334 4.23988 3.24282 3.70096 3.44030 3.37676 1.09934 2.79202 3.12899 5.44375 4.15432 67 - -
2.68618 4.42225 2.77519 2.73123 3.46354 2.40513 3.72494 3.29354 2.67741 2.69355 4.24690 2.90347 2.73739 3.18146 2.89801 2.37887 2.77519 2.98518 4.58477 3.61503
0.03157 3.86699 4.58934 0.61958 0.77255 0.48576 0.95510
68 3.25138 4.65475 4.23121 3.94466 1.02072 3.94245 3.87481 2.77574 3.85183 2.14705 3.50670 4.01301 4.40115 4.02131 3.97290 3.52160 3.55251 2.76604 4.12549 2.49318 68 - -
2.68618 4.42225 2.77519 2.73123 3.46354 2.40513 3.72494 3.29354 2.67741 2.69355 4.24690 2.90347 2.73739 3.18146 2.89801 2.37887 2.77519 2.98518 4.58477 3.61503
0.03157 3.86699 4.58934 0.61958 0.77255 0.48576 0.95510
69 2.98371 5.11116 0.92594 2.33150 4.48744 3.20651 3.92621 4.07436 3.00929 3.68366 4.66164 2.88760 3.85892 3.16534 3.52477 2.94668 3.32216 3.71413 5.65711 4.36124 69 - -
2.68618 4.42225 2.77519 2.73123 3.46354 2.40513 3.72494 3.29354 2.67741 2.69355 4.24690 2.90347 2.73739 3.18146 2.89801 2.37887 2.77519 2.98518 4.58477 3.61503
0.03157 3.86699 4.58934 0.61958 0.77255 0.48576 0.95510
70 2.96711 4.93068 3.14273 2.76522 4.31586 3.45716 3.75616 3.74479 1.04579 3.31106 4.27280 3.18993 3.95788 2.94794 2.43058 3.00945 3.22078 3.45288 5.37276 4.18474 70 - -
2.68618 4.42225 2.77519 2.73123 3.46354 2.40513 3.72494 3.29354 2.67741 2.69355 4.24690 2.90347 2.73739 3.18146 2.89801 2.37887 2.77519 2.98518 4.58477 3.61503
0.03157 3.86699 4.58934 0.61958 0.77255 0.48576 0.95510
71 2.94758 5.05751 2.48529 1.03318 4.40304 3.27053 3.85685 3.85586 2.73669 3.47385 4.44812 2.92979 3.87486 3.07262 3.14497 2.93053 3.25177 3.54026 5.57565 4.29788 71 - -
2.68618 4.42225 2.77519 2.73123 3.46354 2.40513 3.72494 3.29354 2.67741 2.69355 4.24690 2.90347 2.73739 3.18146 2.89801 2.37887 2.77519 2.98518 4.58477 3.61503
0.03157 3.86699 4.58934 0.61958 0.77255 0.48576 0.95510
72 2.94758 5.05751 2.48529 1.03318 4.40304 3.27053 3.85685 3.85586 2.73669 3.47385 4.44812 2.92979 3.87486 3.07262 3.14497 2.93053 3.25177 3.54026 5.57565 4.29788 72 - -
2.68618 4.42225 2.77519 2.73123 3.46354 2.40513 3.72494 3.29354 2.67741 2.69355 4.24690 2.90347 2.73739 3.18146 2.89801 2.37887 2.77519 2.98518 4.58477 3.61503
0.03157 3.86699 4.58934 0.61958 0.77255 0.48576 0.95510
73 2.40976 4.25482 3.42322 3.17496 3.96403 3.09429 4.19270 3.09049 3.11783 2.95418 4.00504 3.38522 3.79760 3.51292 3.37678 2.61097 1.18122 2.78606 5.40231 4.18065 73 - -
2.68618 4.42225 2.77519 2.73123 3.46354 2.40513 3.72494 3.29354 2.67741 2.69355 4.24690 2.90347 2.73739 3.18146 2.89801 2.37887 2.77519 2.98518 4.58477 3.61503
0.03157 3.86699 4.58934 0.61958 0.77255 0.48576 0.95510
74 2.94758 5.05751 2.48529 1.03318 4.40304 3.27053 3.85685 3.85586 2.73669 3.47385 4.44812 2.92979 3.87486 3.07262 3.14497 2.93053 3.25177 3.54026 5.57565 4.29788 74 - -
2.68618 4.42225 2.77519 2.73123 3.46354 2.40513 3.72494 3.29354 2.67741 2.69355 4.24690 2.90347 2.73739 3.18146 2.89801 2.37887 2.77519 2.98518 4.58477 3.61503
0.03157 3.86699 4.58934 0.61958 0.77255 0.48576 0.95510
75 2.98371 5.11116 0.92594 2.33150 4.48744 3.20651 3.92621 4.07436 3.00929 3.68366 4.66164 2.88760 3.85892 3.16534 3.52477 2.94668 3.32216 3.71413 5.65711 4.36124 75 - -
2.68618 4.42225 2.77519 2.73123 3.46354 2.40513 3.72494 3.29354 2.67741 2.69355 4.24690 2.90347 2.73739 3.18146 2.89801 2.37887 2.77519 2.98518 4.58477 3.61503
0.03157 3.86699 4.58934 0.61958 0.77255 0.48576 0.95510

76 3.02684 4.44709 4.32791 3.94350 3.37049 4.06063 4.67263 1.08471 3.77153 1.95764 3.30670 4.18960 4.49236 4.12038 3.96872 3.55539 3.32610 1.84575 5.26019 3.99802 76 - -

2.68618 4.42225 2.77519 2.73123 3.46354 2.40513 3.72494 3.29354 2.67741 2.69355 4.24690 2.90347 2.73739 3.18146 2.89801 2.37887 2.77519 2.98518 4.58477 3.61503

0.03157 3.86699 4.58934 0.61958 0.77255 0.48576 0.95510

77 2.94758 5.05751 2.48529 1.03318 4.40304 3.27053 3.85685 3.85586 2.73669 3.47385 4.44812 2.92979 3.87486 3.07262 3.14497 2.93053 3.25177 3.54026 5.57565 4.29788 77 - -

2.68618 4.42225 2.77519 2.73123 3.46354 2.40513 3.72494 3.29354 2.67741 2.69355 4.24690 2.90347 2.73739 3.18146 2.89801 2.37887 2.77519 2.98518 4.58477 3.61503

0.03157 3.86699 4.58934 0.61958 0.77255 0.48576 0.95510

78 2.31230 4.23263 3.20165 3.01716 4.07082 2.96261 4.12717 3.59269 3.09056 3.31334 4.23988 3.24282 3.70096 3.44030 3.37676 1.09934 2.79202 3.12899 5.44375 4.15432 78 - -

2.68618 4.42225 2.77519 2.73123 3.46354 2.40513 3.72494 3.29354 2.67741 2.69355 4.24690 2.90347 2.73739 3.18146 2.89801 2.37887 2.77519 2.98518 4.58477 3.61503

0.03157 3.86699 4.58934 0.61958 0.77255 0.48576 0.95510

79 2.80376 4.34788 4.11775 3.78070 3.54607 3.71822 4.58336 2.05053 3.64840 2.23405 3.51204 3.97141 4.27903 4.00859 3.86143 3.22141 3.16544 1.03182 5.32959 4.06087 79 - -

2.68618 4.42225 2.77519 2.73123 3.46354 2.40513 3.72494 3.29354 2.67741 2.69355 4.24690 2.90347 2.73739 3.18146 2.89801 2.37887 2.77519 2.98518 4.58477 3.61503

0.03157 3.86699 4.58934 0.61958 0.77255 0.48576 0.95510

80 3.02684 4.44709 4.32791 3.94350 3.37049 4.06063 4.67263 1.08471 3.77153 1.95764 3.30670 4.18960 4.49236 4.12038 3.96872 3.55539 3.32610 1.84575 5.26019 3.99802 80 - -

2.68618 4.42225 2.77519 2.73123 3.46354 2.40513 3.72494 3.29354 2.67741 2.69355 4.24690 2.90347 2.73739 3.18146 2.89801 2.37887 2.77519 2.98518 4.58477 3.61503

0.03157 3.86699 4.58934 0.61958 0.77255 0.48576 0.95510

81 2.98371 5.11116 0.92594 2.33150 4.48744 3.20651 3.92621 4.07436 3.00929 3.68366 4.66164 2.88760 3.85892 3.16534 3.52477 2.94668 3.32216 3.71413 5.65711 4.36124 81 - -

2.68618 4.42225 2.77519 2.73123 3.46354 2.40513 3.72494 3.29354 2.67741 2.69355 4.24690 2.90347 2.73739 3.18146 2.89801 2.37887 2.77519 2.98518 4.58477 3.61503

0.03157 3.86699 4.58934 0.61958 0.77255 0.48576 0.95510

82 2.65811 4.43408 3.33076 3.22947 4.37188 0.73761 4.35008 3.97016 3.41000 3.62498 4.60067 3.49506 3.82505 3.73459 3.64665 2.82986 3.13892 3.49954 5.46848 4.47359 82 - -

2.68618 4.42225 2.77519 2.73123 3.46354 2.40513 3.72494 3.29354 2.67741 2.69355 4.24690 2.90347 2.73739 3.18146 2.89801 2.37887 2.77519 2.98518 4.58477 3.61503

0.03157 3.86699 4.58934 0.61958 0.77255 0.48576 0.95510

83 3.25138 4.65475 4.23121 3.94466 1.02072 3.94245 3.87481 2.77574 3.85183 2.14705 3.50670 4.01301 4.40115 4.02131 3.97290 3.52160 3.55251 2.76604 4.12549 2.49318 83 - -

2.68618 4.42225 2.77519 2.73123 3.46354 2.40513 3.72494 3.29354 2.67741 2.69355 4.24690 2.90347 2.73739 3.18146 2.89801 2.37887 2.77519 2.98518 4.58477 3.61503

0.03157 3.86699 4.58934 0.61958 0.77255 0.48576 0.95510

84 3.25138 4.65475 4.23121 3.94466 1.02072 3.94245 3.87481 2.77574 3.85183 2.14705 3.50670 4.01301 4.40115 4.02131 3.97290 3.52160 3.55251 2.76604 4.12549 2.49318 84 - -

2.68618 4.42225 2.77519 2.73123 3.46354 2.40513 3.72494 3.29354 2.67741 2.69355 4.24690 2.90347 2.73739 3.18146 2.89801 2.37887 2.77519 2.98518 4.58477 3.61503

0.03157 3.86699 4.58934 0.61958 0.77255 0.48576 0.95510

85 3.23918 4.73039 3.83015 3.55850 2.29358 3.82376 3.61597 3.24916 3.41618 2.70450 3.92476 3.71671 4.29772 3.72354 3.60101 3.37642 3.52583 3.11945 3.93713 0.97494 85 - -

2.68618 4.42225 2.77519 2.73123 3.46354 2.40513 3.72494 3.29354 2.67741 2.69355 4.24690 2.90347 2.73739 3.18146 2.89801 2.37887 2.77519 2.98518 4.58477 3.61503

0.03157 3.86699 4.58934 0.61958 0.77255 0.48576 0.95510

86 3.14912 4.61302 4.18070 3.82053 3.13362 3.97519 4.47798 2.33736 3.58833 0.88196 3.15827 4.09653 4.41149 3.95797 3.76529 3.54541 3.44362 2.37328 5.01092 3.73297 86 - -

2.68618 4.42225 2.77519 2.73123 3.46354 2.40513 3.72494 3.29354 2.67741 2.69355 4.24690 2.90347 2.73739 3.18146 2.89801 2.37887 2.77519 2.98518 4.58477 3.61503

0.03157 3.86699 4.58934 0.61958 0.77255 0.48576 0.95510

87 3.14912 4.61302 4.18070 3.82053 3.13362 3.97519 4.47798 2.33736 3.58833 0.88196 3.15827 4.09653 4.41149 3.95797 3.76529 3.54541 3.44362 2.37328 5.01092 3.73297 87 - -

2.68618 4.42225 2.77519 2.73123 3.46354 2.40513 3.72494 3.29354 2.67741 2.69355 4.24690 2.90347 2.73739 3.18146 2.89801 2.37887 2.77519 2.98518 4.58477 3.61503

0.03157 3.86699 4.58934 0.61958 0.77255 0.48576 0.95510

88 2.99928 4.83880 3.48937 3.00157 4.19200 3.48833 3.82030 3.73332 2.23516 3.25025 4.25056 3.36173 3.99118 3.04089 0.98678 3.08371 3.26560 3.45751 5.27725 4.12054 88 - -

2.68618 4.42225 2.77519 2.73123 3.46354 2.40513 3.72494 3.29354 2.67741 2.69355 4.24690 2.90347 2.73739 3.18146 2.89801 2.37887 2.77519 2.98518 4.58477 3.61503

0.03157 3.86699 4.58934 0.61958 0.77255 0.48576 0.95510

89 3.14912 4.61302 4.18070 3.82053 3.13362 3.97519 4.47798 2.33736 3.58833 0.88196 3.15827 4.09653 4.41149 3.95797 3.76529 3.54541 3.44362 2.37328 5.01092 3.73297 89 - -

2.68618 4.42225 2.77519 2.73123 3.46354 2.40513 3.72494 3.29354 2.67741 2.69355 4.24690 2.90347 2.73739 3.18146 2.89801 2.37887 2.77519 2.98518 4.58477 3.61503

0.03157 3.86699 4.58934 0.61958 0.77255 0.48576 0.95510

90 2.31230 4.23263 3.20165 3.01716 4.07082 2.96261 4.12717 3.59269 3.09056 3.31334 4.23988 3.24282 3.70096 3.44030 3.37676 1.09934 2.79202 3.12899 5.44375 4.15432 90 - -

2.68618 4.42225 2.77519 2.73123 3.46354 2.40513 3.72494 3.29354 2.67741 2.69355 4.24690 2.90347 2.73739 3.18146 2.89801 2.37887 2.77519 2.98518 4.58477 3.61503

0.03157 3.86699 4.58934 0.61958 0.77255 0.48576 0.95510

91 1.02168 4.18931 3.45704 3.24808 4.04693 3.01400 4.27077 3.17162 3.26419 3.05349 4.08591 3.39907 3.75270 3.60586 3.51510 2.53965 2.81663 2.82697 5.47463 4.27427 91 - -

2.68618 4.42225 2.77519 2.73123 3.46354 2.40513 3.72494 3.29354 2.67741 2.69355 4.24690 2.90347 2.73739 3.18146 2.89801 2.37887 2.77519 2.98518 4.58477 3.61503

0.03157 3.86699 4.58934 0.61958 0.77255 0.48576 0.95510

92 2.87640 4.86196 2.89930 2.65395 4.02980 3.36241 3.81221 3.65971 2.47512 3.16261 4.19062 3.11364 3.91579 1.24381 2.75940 2.92438 3.17337 3.38973 5.30278 3.99617 92 - -

2.68618 4.42225 2.77519 2.73123 3.46354 2.40513 3.72494 3.29354 2.67741 2.69355 4.24690 2.90347 2.73739 3.18146 2.89801 2.37887 2.77519 2.98518 4.58477 3.61503

0.03157 3.86699 4.58934 0.61958 0.77255 0.48576 0.95510

93 3.23918 4.73039 3.83015 3.55850 2.29358 3.82376 3.61597 3.24916 3.41618 2.70450 3.92476 3.71671 4.29772 3.72354 3.60101 3.37642 3.52583 3.11945 3.93713 0.97494 93 - -

2.68618 4.42225 2.77519 2.73123 3.46354 2.40513 3.72494 3.29354 2.67741 2.69355 4.24690 2.90347 2.73739 3.18146 2.89801 2.37887 2.77519 2.98518 4.58477 3.61503

0.03157 3.86699 4.58934 0.61958 0.77255 0.48576 0.95510

94 2.98886 4.80727 3.12036 2.87975 3.28089 3.44287 1.15410 3.69093 2.72295 3.18558 4.22810 3.28930 3.99510 3.24591 2.98899 3.05959 3.29203 3.42616 4.74602 3.23190 94 - -

2.68618 4.42225 2.77519 2.73123 3.46354 2.40513 3.72494 3.29354 2.67741 2.69355 4.24690 2.90347 2.73739 3.18146 2.89801 2.37887 2.77519 2.98518 4.58477 3.61503

0.03157 3.86699 4.58934 0.61958 0.77255 0.48576 0.95510

95 2.98371 5.11116 0.92594 2.33150 4.48744 3.20651 3.92621 4.07436 3.00929 3.68366 4.66164 2.88760 3.85892 3.16534 3.52477 2.94668 3.32216 3.71413 5.65711 4.36124 95 - -

2.68618 4.42225 2.77519 2.73123 3.46354 2.40513 3.72494 3.29354 2.67741 2.69355 4.24690 2.90347 2.73739 3.18146 2.89801 2.37887 2.77519 2.98518 4.58477 3.61503

0.03157 3.86699 4.58934 0.61958 0.77255 0.48576 0.95510

96 2.80376 4.34788 4.11775 3.78070 3.54607 3.71822 4.58336 2.05053 3.64840 2.23405 3.51204 3.97141 4.27903 4.00859 3.86143 3.22141 3.16544 1.03182 5.32959 4.06087 96 - -

2.68618 4.42225 2.77519 2.73123 3.46354 2.40513 3.72494 3.29354 2.67741 2.69355 4.24690 2.90347 2.73739 3.18146 2.89801 2.37887 2.77519 2.98518 4.58477 3.61503

0.03157 3.86699 4.58934 0.61958 0.77255 0.48576 0.95510

97 2.94758 5.05751 2.48529 1.03318 4.40304 3.27053 3.85685 3.85586 2.73669 3.47385 4.44812 2.92979 3.87486 3.07262 3.14497 2.93053 3.25177 3.54026 5.57565 4.29788 97 - -

2.68618 4.42225 2.77519 2.73123 3.46354 2.40513 3.72494 3.29354 2.67741 2.69355 4.24690 2.90347 2.73739 3.18146 2.89801 2.37887 2.77519 2.98518 4.58477 3.61503

0.03157 3.86699 4.58934 0.61958 0.77255 0.48576 0.95510

98 2.80376 4.34788 4.11775 3.78070 3.54607 3.71822 4.58336 2.05053 3.64840 2.23405 3.51204 3.97141 4.27903 4.00859 3.86143 3.22141 3.16544 1.03182 5.32959 4.06087 98 - -

2.68618 4.42225 2.77519 2.73123 3.46354 2.40513 3.72494 3.29354 2.67741 2.69355 4.24690 2.90347 2.73739 3.18146 2.89801 2.37887 2.77519 2.98518 4.58477 3.61503

0.03157 3.86699 4.58934 0.61958 0.77255 0.48576 0.95510

99 2.40976 4.25482 3.42322 3.17496 3.96403 3.09429 4.19270 3.09049 3.11783 2.95418 4.00504 3.38522 3.79760 3.51292 3.37678 2.61097 1.18122 2.78606 5.40231 4.18065 99 - -

2.68618 4.42225 2.77519 2.73123 3.46354 2.40513 3.72494 3.29354 2.67741 2.69355 4.24690 2.90347 2.73739 3.18146 2.89801 2.37887 2.77519 2.98518 4.58477 3.61503
0.03157 3.86699 4.58934 0.61958 0.77255 0.48576 0.95510
100 2.96711 4.93068 3.14273 2.76522 4.31586 3.45716 3.75616 3.74479 1.04579 3.31106 4.27280 3.18993 3.95788 2.94794 2.43058 3.00945 3.22078 3.45288 5.37276 4.18474 100 - -
2.68618 4.42225 2.77519 2.73123 3.46354 2.40513 3.72494 3.29354 2.67741 2.69355 4.24690 2.90347 2.73739 3.18146 2.89801 2.37887 2.77519 2.98518 4.58477 3.61503
0.03157 3.86699 4.58934 0.61958 0.77255 0.48576 0.95510
101 1.02168 4.18931 3.45704 3.24808 4.04693 3.01400 4.27077 3.17162 3.26419 3.05349 4.08591 3.39907 3.75270 3.60586 3.51510 2.53965 2.81663 2.82697 5.47463 4.27427 101 - -
2.68618 4.42225 2.77519 2.73123 3.46354 2.40513 3.72494 3.29354 2.67741 2.69355 4.24690 2.90347 2.73739 3.18146 2.89801 2.37887 2.77519 2.98518 4.58477 3.61503
0.03157 3.86699 4.58934 0.61958 0.77255 0.48576 0.95510
102 2.80376 4.34788 4.11775 3.78070 3.54607 3.71822 4.58336 2.05053 3.64840 2.23405 3.51204 3.97141 4.27903 4.00859 3.86143 3.22141 3.16544 1.03182 5.32959 4.06087 102 - -
2.68618 4.42225 2.77519 2.73123 3.46354 2.40513 3.72494 3.29354 2.67741 2.69355 4.24690 2.90347 2.73739 3.18146 2.89801 2.37887 2.77519 2.98518 4.58477 3.61503
0.03157 3.86699 4.58934 0.61958 0.77255 0.48576 0.95510
103 2.98886 4.80727 3.12036 2.87975 3.28089 3.44287 1.15410 3.69093 2.72295 3.18558 4.22810 3.28930 3.99510 3.24591 2.98899 3.05959 3.29203 3.42616 4.74602 3.23190 103 - -
2.68618 4.42225 2.77519 2.73123 3.46354 2.40513 3.72494 3.29354 2.67741 2.69355 4.24690 2.90347 2.73739 3.18146 2.89801 2.37887 2.77519 2.98518 4.58477 3.61503
0.03157 3.86699 4.58934 0.61958 0.77255 0.48576 0.95510
104 1.02168 4.18931 3.45704 3.24808 4.04693 3.01400 4.27077 3.17162 3.26419 3.05349 4.08591 3.39907 3.75270 3.60586 3.51510 2.53965 2.81663 2.82697 5.47463 4.27427 104 - -
2.68618 4.42225 2.77519 2.73123 3.46354 2.40513 3.72494 3.29354 2.67741 2.69355 4.24690 2.90347 2.73739 3.18146 2.89801 2.37887 2.77519 2.98518 4.58477 3.61503
0.03157 3.86699 4.58934 0.61958 0.77255 0.48576 0.95510
105 2.31230 4.23263 3.20165 3.01716 4.07082 2.96261 4.12717 3.59269 3.09056 3.31334 4.23988 3.24282 3.70096 3.44030 3.37676 1.09934 2.79202 3.12899 5.44375 4.15432 105 - -
2.68618 4.42225 2.77519 2.73123 3.46354 2.40513 3.72494 3.29354 2.67741 2.69355 4.24690 2.90347 2.73739 3.18146 2.89801 2.37887 2.77519 2.98518 4.58477 3.61503
0.03157 3.86699 4.58934 0.61958 0.77255 0.48576 0.95510
106 3.25138 4.65475 4.23121 3.94466 1.02072 3.94245 3.87481 2.77574 3.85183 2.14705 3.50670 4.01301 4.40115 4.02131 3.97290 3.52160 3.55251 2.76604 4.12549 2.49318 106 - -
2.68618 4.42225 2.77519 2.73123 3.46354 2.40513 3.72494 3.29354 2.67741 2.69355 4.24690 2.90347 2.73739 3.18146 2.89801 2.37887 2.77519 2.98518 4.58477 3.61503
0.03157 3.86699 4.58934 0.61958 0.77255 0.48576 0.95510
107 3.14912 4.61302 4.18070 3.82053 3.13362 3.97519 4.47798 2.33736 3.58833 0.88196 3.15827 4.09653 4.41149 3.95797 3.76529 3.54541 3.44362 2.37328 5.01092 3.73297 107 - -
2.68618 4.42225 2.77519 2.73123 3.46354 2.40513 3.72494 3.29354 2.67741 2.69355 4.24690 2.90347 2.73739 3.18146 2.89801 2.37887 2.77519 2.98518 4.58477 3.61503
0.03157 3.86699 4.58934 0.61958 0.77255 0.48576 0.95510
108 2.96711 4.93068 3.14273 2.76522 4.31586 3.45716 3.75616 3.74479 1.04579 3.31106 4.27280 3.18993 3.95788 2.94794 2.43058 3.00945 3.22078 3.45288 5.37276 4.18474 108 - -
2.68618 4.42225 2.77519 2.73123 3.46354 2.40513 3.72494 3.29354 2.67741 2.69355 4.24690 2.90347 2.73739 3.18146 2.89801 2.37887 2.77519 2.98518 4.58477 3.61503
0.03157 3.86699 4.58934 0.61958 0.77255 0.48576 0.95510
109 3.14912 4.61302 4.18070 3.82053 3.13362 3.97519 4.47798 2.33736 3.58833 0.88196 3.15827 4.09653 4.41149 3.95797 3.76529 3.54541 3.44362 2.37328 5.01092 3.73297 109 - -
2.68618 4.42225 2.77519 2.73123 3.46354 2.40513 3.72494 3.29354 2.67741 2.69355 4.24690 2.90347 2.73739 3.18146 2.89801 2.37887 2.77519 2.98518 4.58477 3.61503
0.03157 3.86699 4.58934 0.61958 0.77255 0.48576 0.95510
110 2.99928 4.83880 3.48937 3.00157 4.19200 3.48833 3.82030 3.73332 2.23516 3.25025 4.25056 3.36173 3.99118 3.04089 0.98678 3.08371 3.26560 3.45751 5.27725 4.12054 110 - -
2.68618 4.42225 2.77519 2.73123 3.46354 2.40513 3.72494 3.29354 2.67741 2.69355 4.24690 2.90347 2.73739 3.18146 2.89801 2.37887 2.77519 2.98518 4.58477 3.61503
0.03157 3.86699 4.58934 0.61958 0.77255 0.48576 0.95510

111 2.94758 5.05751 2.48529 1.03318 4.40304 3.27053 3.85685 3.85586 2.73669 3.47385 4.44812 2.92979 3.87486 3.07262 3.14497 2.93053 3.25177 3.54026 5.57565 4.29788 111 - -
2.68618 4.42225 2.77519 2.73123 3.46354 2.40513 3.72494 3.29354 2.67741 2.69355 4.24690 2.90347 2.73739 3.18146 2.89801 2.37887 2.77519 2.98518 4.58477 3.61503
0.03157 3.86699 4.58934 0.61958 0.77255 0.48576 0.95510
112 2.94758 5.05751 2.48529 1.03318 4.40304 3.27053 3.85685 3.85586 2.73669 3.47385 4.44812 2.92979 3.87486 3.07262 3.14497 2.93053 3.25177 3.54026 5.57565 4.29788 112 - -
2.68618 4.42225 2.77519 2.73123 3.46354 2.40513 3.72494 3.29354 2.67741 2.69355 4.24690 2.90347 2.73739 3.18146 2.89801 2.37887 2.77519 2.98518 4.58477 3.61503
0.03157 3.86699 4.58934 0.61958 0.77255 0.48576 0.95510
113 2.96711 4.93068 3.14273 2.76522 4.31586 3.45716 3.75616 3.74479 1.04579 3.31106 4.27280 3.18993 3.95788 2.94794 2.43058 3.00945 3.22078 3.45288 5.37276 4.18474 113 - -
2.68618 4.42225 2.77519 2.73123 3.46354 2.40513 3.72494 3.29354 2.67741 2.69355 4.24690 2.90347 2.73739 3.18146 2.89801 2.37887 2.77519 2.98518 4.58477 3.61503
0.03157 3.86699 4.58934 0.61958 0.77255 0.48576 0.95510
114 2.40976 4.25482 3.42322 3.17496 3.96403 3.09429 4.19270 3.09049 3.11783 2.95418 4.00504 3.38522 3.79760 3.51292 3.37678 2.61097 1.18122 2.78606 5.40231 4.18065 114 - -
2.68618 4.42225 2.77519 2.73123 3.46354 2.40513 3.72494 3.29354 2.67741 2.69355 4.24690 2.90347 2.73739 3.18146 2.89801 2.37887 2.77519 2.98518 4.58477 3.61503
0.03157 3.86699 4.58934 0.61958 0.77255 0.48576 0.95510
115 2.99928 4.83880 3.48937 3.00157 4.19200 3.48833 3.82030 3.73332 2.23516 3.25025 4.25056 3.36173 3.99118 3.04089 0.98678 3.08371 3.26560 3.45751 5.27725 4.12054 115 - -
2.68618 4.42225 2.77519 2.73123 3.46354 2.40513 3.72494 3.29354 2.67741 2.69355 4.24690 2.90347 2.73739 3.18146 2.89801 2.37887 2.77519 2.98518 4.58477 3.61503
0.03157 3.86699 4.58934 0.61958 0.77255 0.48576 0.95510
116 3.14912 4.61302 4.18070 3.82053 3.13362 3.97519 4.47798 2.33736 3.58833 0.88196 3.15827 4.09653 4.41149 3.95797 3.76529 3.54541 3.44362 2.37328 5.01092 3.73297 116 - -
2.68618 4.42225 2.77519 2.73123 3.46354 2.40513 3.72494 3.29354 2.67741 2.69355 4.24690 2.90347 2.73739 3.18146 2.89801 2.37887 2.77519 2.98518 4.58477 3.61503
0.03157 3.86699 4.58934 0.61958 0.77255 0.48576 0.95510
117 2.65811 4.43408 3.33076 3.22947 4.37188 0.73761 4.35008 3.97016 3.41000 3.62498 4.60067 3.49506 3.82505 3.73459 3.64665 2.82986 3.13892 3.49954 5.46848 4.47359 117 - -
2.68618 4.42225 2.77519 2.73123 3.46354 2.40513 3.72494 3.29354 2.67741 2.69355 4.24690 2.90347 2.73739 3.18146 2.89801 2.37887 2.77519 2.98518 4.58477 3.61503
0.03157 3.86699 4.58934 0.61958 0.77255 0.48576 0.95510
118 2.71888 4.71528 2.70526 2.59018 4.07794 3.18029 3.91656 3.78713 2.81979 3.43461 4.38989 1.16778 3.83188 3.18930 3.17427 2.78628 3.10056 3.40989 5.38448 4.02666 118 - -
2.68618 4.42225 2.77519 2.73123 3.46354 2.40513 3.72494 3.29354 2.67741 2.69355 4.24690 2.90347 2.73739 3.18146 2.89801 2.37887 2.77519 2.98518 4.58477 3.61503
0.03157 3.86699 4.58934 0.61958 0.77255 0.48576 0.95510
119 1.02168 4.18931 3.45704 3.24808 4.04693 3.01400 4.27077 3.17162 3.26419 3.05349 4.08591 3.39907 3.75270 3.60586 3.51510 2.53965 2.81663 2.82697 5.47463 4.27427 119 - -
2.68618 4.42225 2.77519 2.73123 3.46354 2.40513 3.72494 3.29354 2.67741 2.69355 4.24690 2.90347 2.73739 3.18146 2.89801 2.37887 2.77519 2.98518 4.58477 3.61503
0.03157 3.86699 4.58934 0.61958 0.77255 0.48576 0.95510
120 3.14912 4.61302 4.18070 3.82053 3.13362 3.97519 4.47798 2.33736 3.58833 0.88196 3.15827 4.09653 4.41149 3.95797 3.76529 3.54541 3.44362 2.37328 5.01092 3.73297 120 - -
2.68618 4.42225 2.77519 2.73123 3.46354 2.40513 3.72494 3.29354 2.67741 2.69355 4.24690 2.90347 2.73739 3.18146 2.89801 2.37887 2.77519 2.98518 4.58477 3.61503
0.03157 3.86699 4.58934 0.61958 0.77255 0.48576 0.95510
121 3.02684 4.44709 4.32791 3.94350 3.37049 4.06063 4.67263 1.08471 3.77153 1.95764 3.30670 4.18960 4.49236 4.12038 3.96872 3.55539 3.32610 1.84575 5.26019 3.99802 121 - -
2.68618 4.42225 2.77519 2.73123 3.46354 2.40513 3.72494 3.29354 2.67741 2.69355 4.24690 2.90347 2.73739 3.18146 2.89801 2.37887 2.77519 2.98518 4.58477 3.61503
0.03157 3.86699 4.58934 0.61958 0.77255 0.48576 0.95510
122 2.31230 4.23263 3.20165 3.01716 4.07082 2.96261 4.12717 3.59269 3.09056 3.31334 4.23988 3.24282 3.70096 3.44030 3.37676 1.09934 2.79202 3.12899 5.44375 4.15432 122 - -
2.68618 4.42225 2.77519 2.73123 3.46354 2.40513 3.72494 3.29354 2.67741 2.69355 4.24690 2.90347 2.73739 3.18146 2.89801 2.37887 2.77519 2.98518 4.58477 3.61503

0.03157 3.86699 4.58934 0.61958 0.77255 0.48576 0.95510
123 2.40976 4.25482 3.42322 3.17496 3.96403 3.09429 4.19270 3.09049 3.11783 2.95418 4.00504 3.38522 3.79760 3.51292 3.37678 2.61097 1.18122 2.78606 5.40231 4.18065 123 - -
2.68618 4.42225 2.77519 2.73123 3.46354 2.40513 3.72494 3.29354 2.67741 2.69355 4.24690 2.90347 2.73739 3.18146 2.89801 2.37887 2.77519 2.98518 4.58477 3.61503
0.03157 3.86699 4.58934 0.61958 0.77255 0.48576 0.95510
124 3.23918 4.73039 3.83015 3.55850 2.29358 3.82376 3.61597 3.24916 3.41618 2.70450 3.92476 3.71671 4.29772 3.72354 3.60101 3.37642 3.52583 3.11945 3.93713 0.97494 124 - -
2.68618 4.42225 2.77519 2.73123 3.46354 2.40513 3.72494 3.29354 2.67741 2.69355 4.24690 2.90347 2.73739 3.18146 2.89801 2.37887 2.77519 2.98518 4.58477 3.61503
0.03157 3.86699 4.58934 0.61958 0.77255 0.48576 0.95510
125 3.14912 4.61302 4.18070 3.82053 3.13362 3.97519 4.47798 2.33736 3.58833 0.88196 3.15827 4.09653 4.41149 3.95797 3.76529 3.54541 3.44362 2.37328 5.01092 3.73297 125 - -
2.68618 4.42225 2.77519 2.73123 3.46354 2.40513 3.72494 3.29354 2.67741 2.69355 4.24690 2.90347 2.73739 3.18146 2.89801 2.37887 2.77519 2.98518 4.58477 3.61503
0.03157 3.86699 4.58934 0.61958 0.77255 0.48576 0.95510
126 2.96711 4.93068 3.14273 2.76522 4.31586 3.45716 3.75616 3.74479 1.04579 3.31106 4.27280 3.18993 3.95788 2.94794 2.43058 3.00945 3.22078 3.45288 5.37276 4.18474 126 - -
2.68618 4.42225 2.77519 2.73123 3.46354 2.40513 3.72494 3.29354 2.67741 2.69355 4.24690 2.90347 2.73739 3.18146 2.89801 2.37887 2.77519 2.98518 4.58477 3.61503
0.03157 3.86699 4.58934 0.61958 0.77255 0.48576 0.95510
127 3.14912 4.61302 4.18070 3.82053 3.13362 3.97519 4.47798 2.33736 3.58833 0.88196 3.15827 4.09653 4.41149 3.95797 3.76529 3.54541 3.44362 2.37328 5.01092 3.73297 127 - -
2.68618 4.42225 2.77519 2.73123 3.46354 2.40513 3.72494 3.29354 2.67741 2.69355 4.24690 2.90347 2.73739 3.18146 2.89801 2.37887 2.77519 2.98518 4.58477 3.61503
0.03157 3.86699 4.58934 0.61958 0.77255 0.48576 0.95510
128 2.65811 4.43408 3.33076 3.22947 4.37188 0.73761 4.35008 3.97016 3.41000 3.62498 4.60067 3.49506 3.82505 3.73459 3.64665 2.82986 3.13892 3.49954 5.46848 4.47359 128 - -
2.68618 4.42225 2.77519 2.73123 3.46354 2.40513 3.72494 3.29354 2.67741 2.69355 4.24690 2.90347 2.73739 3.18146 2.89801 2.37887 2.77519 2.98518 4.58477 3.61503
0.03157 3.86699 4.58934 0.61958 0.77255 0.48576 0.95510
129 3.25138 4.65475 4.23121 3.94466 1.02072 3.94245 3.87481 2.77574 3.85183 2.14705 3.50670 4.01301 4.40115 4.02131 3.97290 3.52160 3.55251 2.76604 4.12549 2.49318 129 - -
2.68618 4.42225 2.77519 2.73123 3.46354 2.40513 3.72494 3.29354 2.67741 2.69355 4.24690 2.90347 2.73739 3.18146 2.89801 2.37887 2.77519 2.98518 4.58477 3.61503
0.03157 3.86699 4.58934 0.61958 0.77255 0.48576 0.95510
130 2.74420 4.50247 3.35077 3.18900 4.18468 3.19029 4.26692 3.72867 3.24583 3.35611 4.40571 3.49627 0.83787 3.63478 3.49200 2.91151 3.18532 3.37467 5.37275 4.30526 130 - -
2.68618 4.42225 2.77519 2.73123 3.46354 2.40513 3.72494 3.29354 2.67741 2.69355 4.24690 2.90347 2.73739 3.18146 2.89801 2.37887 2.77519 2.98518 4.58477 3.61503
0.03157 3.86699 4.58934 0.61958 0.77255 0.48576 0.95510
131 2.99928 4.83880 3.48937 3.00157 4.19200 3.48833 3.82030 3.73332 2.23516 3.25025 4.25056 3.36173 3.99118 3.04089 0.98678 3.08371 3.26560 3.45751 5.27725 4.12054 131 - -
2.68618 4.42225 2.77519 2.73123 3.46354 2.40513 3.72494 3.29354 2.67741 2.69355 4.24690 2.90347 2.73739 3.18146 2.89801 2.37887 2.77519 2.98518 4.58477 3.61503
0.03157 3.86699 4.58934 0.61958 0.77255 0.48576 0.95510
132 2.94758 5.05751 2.48529 1.03318 4.40304 3.27053 3.85685 3.85586 2.73669 3.47385 4.44812 2.92979 3.87486 3.07262 3.14497 2.93053 3.25177 3.54026 5.57565 4.29788 132 - -
2.68618 4.42225 2.77519 2.73123 3.46354 2.40513 3.72494 3.29354 2.67741 2.69355 4.24690 2.90347 2.73739 3.18146 2.89801 2.37887 2.77519 2.98518 4.58477 3.61503
0.03157 3.86699 4.58934 0.61958 0.77255 0.48576 0.95510
133 1.02168 4.18931 3.45704 3.24808 4.04693 3.01400 4.27077 3.17162 3.26419 3.05349 4.08591 3.39907 3.75270 3.60586 3.51510 2.53965 2.81663 2.82697 5.47463 4.27427 133 - -
2.68618 4.42225 2.77519 2.73123 3.46354 2.40513 3.72494 3.29354 2.67741 2.69355 4.24690 2.90347 2.73739 3.18146 2.89801 2.37887 2.77519 2.98518 4.58477 3.61503
0.03157 3.86699 4.58934 0.61958 0.77255 0.48576 0.95510
134 3.02684 4.44709 4.32791 3.94350 3.37049 4.06063 4.67263 1.08471 3.77153 1.95764 3.30670 4.18960 4.49236 4.12038 3.96872 3.55539 3.32610 1.84575 5.26019 3.99802 134 - -

2.68618 4.42225 2.77519 2.73123 3.46354 2.40513 3.72494 3.29354 2.67741 2.69355 4.24690 2.90347 2.73739 3.18146 2.89801 2.37887 2.77519 2.98518 4.58477 3.61503
0.03157 3.86699 4.58934 0.61958 0.77255 0.48576 0.95510
135 3.14912 4.61302 4.18070 3.82053 3.13362 3.97519 4.47798 2.33736 3.58833 0.88196 3.15827 4.09653 4.41149 3.95797 3.76529 3.54541 3.44362 2.37328 5.01092 3.73297 135 - -
2.68618 4.42225 2.77519 2.73123 3.46354 2.40513 3.72494 3.29354 2.67741 2.69355 4.24690 2.90347 2.73739 3.18146 2.89801 2.37887 2.77519 2.98518 4.58477 3.61503
0.03157 3.86699 4.58934 0.61958 0.77255 0.48576 0.95510
136 2.80376 4.34788 4.11775 3.78070 3.54607 3.71822 4.58336 2.05053 3.64840 2.23405 3.51204 3.97141 4.27903 4.00859 3.86143 3.22141 3.16544 1.03182 5.32959 4.06087 136 - -
2.68618 4.42225 2.77519 2.73123 3.46354 2.40513 3.72494 3.29354 2.67741 2.69355 4.24690 2.90347 2.73739 3.18146 2.89801 2.37887 2.77519 2.98518 4.58477 3.61503
0.03157 3.86699 4.58934 0.61958 0.77255 0.48576 0.95510
137 3.25138 4.65475 4.23121 3.94466 1.02072 3.94245 3.87481 2.77574 3.85183 2.14705 3.50670 4.01301 4.40115 4.02131 3.97290 3.52160 3.55251 2.76604 4.12549 2.49318 137 - -
2.68618 4.42225 2.77519 2.73123 3.46354 2.40513 3.72494 3.29354 2.67741 2.69355 4.24690 2.90347 2.73739 3.18146 2.89801 2.37887 2.77519 2.98518 4.58477 3.61503
0.03157 3.86699 4.58934 0.61958 0.77255 0.48576 0.95510
138 2.80376 4.34788 4.11775 3.78070 3.54607 3.71822 4.58336 2.05053 3.64840 2.23405 3.51204 3.97141 4.27903 4.00859 3.86143 3.22141 3.16544 1.03182 5.32959 4.06087 138 - -
2.68618 4.42225 2.77519 2.73123 3.46354 2.40513 3.72494 3.29354 2.67741 2.69355 4.24690 2.90347 2.73739 3.18146 2.89801 2.37887 2.77519 2.98518 4.58477 3.61503
0.03157 3.86699 4.58934 0.61958 0.77255 0.48576 0.95510
139 2.31230 4.23263 3.20165 3.01716 4.07082 2.96261 4.12717 3.59269 3.09056 3.31334 4.23988 3.24282 3.70096 3.44030 3.37676 1.09934 2.79202 3.12899 5.44375 4.15432 139 - -
2.68618 4.42225 2.77519 2.73123 3.46354 2.40513 3.72494 3.29354 2.67741 2.69355 4.24690 2.90347 2.73739 3.18146 2.89801 2.37887 2.77519 2.98518 4.58477 3.61503
0.03157 3.86699 4.58934 0.61958 0.77255 0.48576 0.95510
140 3.14912 4.61302 4.18070 3.82053 3.13362 3.97519 4.47798 2.33736 3.58833 0.88196 3.15827 4.09653 4.41149 3.95797 3.76529 3.54541 3.44362 2.37328 5.01092 3.73297 140 - -
2.68618 4.42225 2.77519 2.73123 3.46354 2.40513 3.72494 3.29354 2.67741 2.69355 4.24690 2.90347 2.73739 3.18146 2.89801 2.37887 2.77519 2.98518 4.58477 3.61503
0.03157 3.86699 4.58934 0.61958 0.77255 0.48576 0.95510
141 2.31230 4.23263 3.20165 3.01716 4.07082 2.96261 4.12717 3.59269 3.09056 3.31334 4.23988 3.24282 3.70096 3.44030 3.37676 1.09934 2.79202 3.12899 5.44375 4.15432 141 - -
2.68618 4.42225 2.77519 2.73123 3.46354 2.40513 3.72494 3.29354 2.67741 2.69355 4.24690 2.90347 2.73739 3.18146 2.89801 2.37887 2.77519 2.98518 4.58477 3.61503
0.03157 3.86699 4.58934 0.61958 0.77255 0.48576 0.95510
142 2.31230 4.23263 3.20165 3.01716 4.07082 2.96261 4.12717 3.59269 3.09056 3.31334 4.23988 3.24282 3.70096 3.44030 3.37676 1.09934 2.79202 3.12899 5.44375 4.15432 142 - -
2.68618 4.42225 2.77519 2.73123 3.46354 2.40513 3.72494 3.29354 2.67741 2.69355 4.24690 2.90347 2.73739 3.18146 2.89801 2.37887 2.77519 2.98518 4.58477 3.61503
0.03157 3.86699 4.58934 0.61958 0.77255 0.48576 0.95510
143 2.74420 4.50247 3.35077 3.18900 4.18468 3.19029 4.26692 3.72867 3.24583 3.35611 4.40571 3.49627 0.83787 3.63478 3.49200 2.91151 3.18532 3.37467 5.37275 4.30526 143 - -
2.68618 4.42225 2.77519 2.73123 3.46354 2.40513 3.72494 3.29354 2.67741 2.69355 4.24690 2.90347 2.73739 3.18146 2.89801 2.37887 2.77519 2.98518 4.58477 3.61503
0.03157 3.86699 4.58934 0.61958 0.77255 0.48576 0.95510
144 2.40976 4.25482 3.42322 3.17496 3.96403 3.09429 4.19270 3.09049 3.11783 2.95418 4.00504 3.38522 3.79760 3.51292 3.37678 2.61097 1.18122 2.78606 5.40231 4.18065 144 - -
2.68618 4.42225 2.77519 2.73123 3.46354 2.40513 3.72494 3.29354 2.67741 2.69355 4.24690 2.90347 2.73739 3.18146 2.89801 2.37887 2.77519 2.98518 4.58477 3.61503
0.03157 3.86699 4.58934 0.61958 0.77255 0.48576 0.95510
145 2.80376 4.34788 4.11775 3.78070 3.54607 3.71822 4.58336 2.05053 3.64840 2.23405 3.51204 3.97141 4.27903 4.00859 3.86143 3.22141 3.16544 1.03182 5.32959 4.06087 145 - -
2.68618 4.42225 2.77519 2.73123 3.46354 2.40513 3.72494 3.29354 2.67741 2.69355 4.24690 2.90347 2.73739 3.18146 2.89801 2.37887 2.77519 2.98518 4.58477 3.61503
0.03157 3.86699 4.58934 0.61958 0.77255 0.48576 0.95510

146 2.80376 4.34788 4.11775 3.78070 3.54607 3.71822 4.58336 2.05053 3.64840 2.23405 3.51204 3.97141 4.27903 4.00859 3.86143 3.22141 3.16544 1.03182 5.32959 4.06087 146 - -
2.68618 4.42225 2.77519 2.73123 3.46354 2.40513 3.72494 3.29354 2.67741 2.69355 4.24690 2.90347 2.73739 3.18146 2.89801 2.37887 2.77519 2.98518 4.58477 3.61503
0.03157 3.86699 4.58934 0.61958 0.77255 0.48576 0.95510
147 2.31230 4.23263 3.20165 3.01716 4.07082 2.96261 4.12717 3.59269 3.09056 3.31334 4.23988 3.24282 3.70096 3.44030 3.37676 1.09934 2.79202 3.12899 5.44375 4.15432 147 - -
2.68618 4.42225 2.77519 2.73123 3.46354 2.40513 3.72494 3.29354 2.67741 2.69355 4.24690 2.90347 2.73739 3.18146 2.89801 2.37887 2.77519 2.98518 4.58477 3.61503
0.03157 3.86699 4.58934 0.61958 0.77255 0.48576 0.95510
148 3.23918 4.73039 3.83015 3.55850 2.29358 3.82376 3.61597 3.24916 3.41618 2.70450 3.92476 3.71671 4.29772 3.72354 3.60101 3.37642 3.52583 3.11945 3.93713 0.97494 148 - -
2.68618 4.42225 2.77519 2.73123 3.46354 2.40513 3.72494 3.29354 2.67741 2.69355 4.24690 2.90347 2.73739 3.18146 2.89801 2.37887 2.77519 2.98518 4.58477 3.61503
0.03157 3.86699 4.58934 0.61958 0.77255 0.48576 0.95510
149 2.40976 4.25482 3.42322 3.17496 3.96403 3.09429 4.19270 3.09049 3.11783 2.95418 4.00504 3.38522 3.79760 3.51292 3.37678 2.61097 1.18122 2.78606 5.40231 4.18065 149 - -
2.68618 4.42225 2.77519 2.73123 3.46354 2.40513 3.72494 3.29354 2.67741 2.69355 4.24690 2.90347 2.73739 3.18146 2.89801 2.37887 2.77519 2.98518 4.58477 3.61503
0.03157 3.86699 4.58934 0.61958 0.77255 0.48576 0.95510
150 1.02168 4.18931 3.45704 3.24808 4.04693 3.01400 4.27077 3.17162 3.26419 3.05349 4.08591 3.39907 3.75270 3.60586 3.51510 2.53965 2.81663 2.82697 5.47463 4.27427 150 - -
2.68618 4.42225 2.77519 2.73123 3.46354 2.40513 3.72494 3.29354 2.67741 2.69355 4.24690 2.90347 2.73739 3.18146 2.89801 2.37887 2.77519 2.98518 4.58477 3.61503
0.03157 3.86699 4.58934 0.61958 0.77255 0.48576 0.95510
151 3.14912 4.61302 4.18070 3.82053 3.13362 3.97519 4.47798 2.33736 3.58833 0.88196 3.15827 4.09653 4.41149 3.95797 3.76529 3.54541 3.44362 2.37328 5.01092 3.73297 151 - -
2.68618 4.42225 2.77519 2.73123 3.46354 2.40513 3.72494 3.29354 2.67741 2.69355 4.24690 2.90347 2.73739 3.18146 2.89801 2.37887 2.77519 2.98518 4.58477 3.61503
0.03157 3.86699 4.58934 0.61958 0.77255 0.48576 0.95510
152 3.02684 4.44709 4.32791 3.94350 3.37049 4.06063 4.67263 1.08471 3.77153 1.95764 3.30670 4.18960 4.49236 4.12038 3.96872 3.55539 3.32610 1.84575 5.26019 3.99802 152 - -
2.68618 4.42225 2.77519 2.73123 3.46354 2.40513 3.72494 3.29354 2.67741 2.69355 4.24690 2.90347 2.73739 3.18146 2.89801 2.37887 2.77519 2.98518 4.58477 3.61503
0.03157 3.86699 4.58934 0.61958 0.77255 0.48576 0.95510
153 2.31230 4.23263 3.20165 3.01716 4.07082 2.96261 4.12717 3.59269 3.09056 3.31334 4.23988 3.24282 3.70096 3.44030 3.37676 1.09934 2.79202 3.12899 5.44375 4.15432 153 - -
2.68618 4.42225 2.77519 2.73123 3.46354 2.40513 3.72494 3.29354 2.67741 2.69355 4.24690 2.90347 2.73739 3.18146 2.89801 2.37887 2.77519 2.98518 4.58477 3.61503
0.03157 3.86699 4.58934 0.61958 0.77255 0.48576 0.95510
154 2.65811 4.43408 3.33076 3.22947 4.37188 0.73761 4.35008 3.97016 3.41000 3.62498 4.60067 3.49506 3.82505 3.73459 3.64665 2.82986 3.13892 3.49954 5.46848 4.47359 154 - -
2.68618 4.42225 2.77519 2.73123 3.46354 2.40513 3.72494 3.29354 2.67741 2.69355 4.24690 2.90347 2.73739 3.18146 2.89801 2.37887 2.77519 2.98518 4.58477 3.61503
0.03157 3.86699 4.58934 0.61958 0.77255 0.48576 0.95510
155 3.25138 4.65475 4.23121 3.94466 1.02072 3.94245 3.87481 2.77574 3.85183 2.14705 3.50670 4.01301 4.40115 4.02131 3.97290 3.52160 3.55251 2.76604 4.12549 2.49318 155 - -
2.68618 4.42225 2.77519 2.73123 3.46354 2.40513 3.72494 3.29354 2.67741 2.69355 4.24690 2.90347 2.73739 3.18146 2.89801 2.37887 2.77519 2.98518 4.58477 3.61503
0.03157 3.86699 4.58934 0.61958 0.77255 0.48576 0.95510
156 2.31230 4.23263 3.20165 3.01716 4.07082 2.96261 4.12717 3.59269 3.09056 3.31334 4.23988 3.24282 3.70096 3.44030 3.37676 1.09934 2.79202 3.12899 5.44375 4.15432 156 - -
2.68618 4.42225 2.77519 2.73123 3.46354 2.40513 3.72494 3.29354 2.67741 2.69355 4.24690 2.90347 2.73739 3.18146 2.89801 2.37887 2.77519 2.98518 4.58477 3.61503
0.03157 3.86699 4.58934 0.61958 0.77255 0.48576 0.95510
157 2.99928 4.83880 3.48937 3.00157 4.19200 3.48833 3.82030 3.73332 2.23516 3.25025 4.25056 3.36173 3.99118 3.04089 0.98678 3.08371 3.26560 3.45751 5.27725 4.12054 157 - -
2.68618 4.42225 2.77519 2.73123 3.46354 2.40513 3.72494 3.29354 2.67741 2.69355 4.24690 2.90347 2.73739 3.18146 2.89801 2.37887 2.77519 2.98518 4.58477 3.61503

0.03157 3.86699 4.58934 0.61958 0.77255 0.48576 0.95510

158 3.14912 4.61302 4.18070 3.82053 3.13362 3.97519 4.47798 2.33736 3.58833 0.88196 3.15827 4.09653 4.41149 3.95797 3.76529 3.54541 3.44362 2.37328 5.01092 3.73297 158 - -

2.68618 4.42225 2.77519 2.73123 3.46354 2.40513 3.72494 3.29354 2.67741 2.69355 4.24690 2.90347 2.73739 3.18146 2.89801 2.37887 2.77519 2.98518 4.58477 3.61503

0.03157 3.86699 4.58934 0.61958 0.77255 0.48576 0.95510

159 2.71888 4.71528 2.70526 2.59018 4.07794 3.18029 3.91656 3.78713 2.81979 3.43461 4.38989 1.16778 3.83188 3.18930 3.17427 2.78628 3.10056 3.40989 5.38448 4.02666 159 - -

2.68618 4.42225 2.77519 2.73123 3.46354 2.40513 3.72494 3.29354 2.67741 2.69355 4.24690 2.90347 2.73739 3.18146 2.89801 2.37887 2.77519 2.98518 4.58477 3.61503

0.03157 3.86699 4.58934 0.61958 0.77255 0.48576 0.95510

160 3.14912 4.61302 4.18070 3.82053 3.13362 3.97519 4.47798 2.33736 3.58833 0.88196 3.15827 4.09653 4.41149 3.95797 3.76529 3.54541 3.44362 2.37328 5.01092 3.73297 160 - -

2.68618 4.42225 2.77519 2.73123 3.46354 2.40513 3.72494 3.29354 2.67741 2.69355 4.24690 2.90347 2.73739 3.18146 2.89801 2.37887 2.77519 2.98518 4.58477 3.61503

0.03157 3.86699 4.58934 0.61958 0.77255 0.48576 0.95510

161 2.94758 5.05751 2.48529 1.03318 4.40304 3.27053 3.85685 3.85586 2.73669 3.47385 4.44812 2.92979 3.87486 3.07262 3.14497 2.93053 3.25177 3.54026 5.57565 4.29788 161 - -

2.68618 4.42225 2.77519 2.73123 3.46354 2.40513 3.72494 3.29354 2.67741 2.69355 4.24690 2.90347 2.73739 3.18146 2.89801 2.37887 2.77519 2.98518 4.58477 3.61503

0.03157 3.86699 4.58934 0.61958 0.77255 0.48576 0.95510

162 3.02684 4.44709 4.32791 3.94350 3.37049 4.06063 4.67263 1.08471 3.77153 1.95764 3.30670 4.18960 4.49236 4.12038 3.96872 3.55539 3.32610 1.84575 5.26019 3.99802 162 - -

2.68618 4.42225 2.77519 2.73123 3.46354 2.40513 3.72494 3.29354 2.67741 2.69355 4.24690 2.90347 2.73739 3.18146 2.89801 2.37887 2.77519 2.98518 4.58477 3.61503

0.03157 3.86699 4.58934 0.61958 0.77255 0.48576 0.95510

163 2.94758 5.05751 2.48529 1.03318 4.40304 3.27053 3.85685 3.85586 2.73669 3.47385 4.44812 2.92979 3.87486 3.07262 3.14497 2.93053 3.25177 3.54026 5.57565 4.29788 163 - -

2.68618 4.42225 2.77519 2.73123 3.46354 2.40513 3.72494 3.29354 2.67741 2.69355 4.24690 2.90347 2.73739 3.18146 2.89801 2.37887 2.77519 2.98518 4.58477 3.61503

0.03157 3.86699 4.58934 0.61958 0.77255 0.48576 0.95510

164 1.02168 4.18931 3.45704 3.24808 4.04693 3.01400 4.27077 3.17162 3.26419 3.05349 4.08591 3.39907 3.75270 3.60586 3.51510 2.53965 2.81663 2.82697 5.47463 4.27427 164 - -

2.68618 4.42225 2.77519 2.73123 3.46354 2.40513 3.72494 3.29354 2.67741 2.69355 4.24690 2.90347 2.73739 3.18146 2.89801 2.37887 2.77519 2.98518 4.58477 3.61503

0.03157 3.86699 4.58934 0.61958 0.77255 0.48576 0.95510

165 3.14912 4.61302 4.18070 3.82053 3.13362 3.97519 4.47798 2.33736 3.58833 0.88196 3.15827 4.09653 4.41149 3.95797 3.76529 3.54541 3.44362 2.37328 5.01092 3.73297 165 - -

2.68618 4.42225 2.77519 2.73123 3.46354 2.40513 3.72494 3.29354 2.67741 2.69355 4.24690 2.90347 2.73739 3.18146 2.89801 2.37887 2.77519 2.98518 4.58477 3.61503

0.03157 3.86699 4.58934 0.61958 0.77255 0.48576 0.95510

166 2.96711 4.93068 3.14273 2.76522 4.31586 3.45716 3.75616 3.74479 1.04579 3.31106 4.27280 3.18993 3.95788 2.94794 2.43058 3.00945 3.22078 3.45288 5.37276 4.18474 166 - -

2.68618 4.42225 2.77519 2.73123 3.46354 2.40513 3.72494 3.29354 2.67741 2.69355 4.24690 2.90347 2.73739 3.18146 2.89801 2.37887 2.77519 2.98518 4.58477 3.61503

0.03157 3.86699 4.58934 0.61958 0.77255 0.48576 0.95510

167 2.80376 4.34788 4.11775 3.78070 3.54607 3.71822 4.58336 2.05053 3.64840 2.23405 3.51204 3.97141 4.27903 4.00859 3.86143 3.22141 3.16544 1.03182 5.32959 4.06087 167 - -

2.68618 4.42225 2.77519 2.73123 3.46354 2.40513 3.72494 3.29354 2.67741 2.69355 4.24690 2.90347 2.73739 3.18146 2.89801 2.37887 2.77519 2.98518 4.58477 3.61503

0.03157 3.86699 4.58934 0.61958 0.77255 0.48576 0.95510

168 3.25138 4.65475 4.23121 3.94466 1.02072 3.94245 3.87481 2.77574 3.85183 2.14705 3.50670 4.01301 4.40115 4.02131 3.97290 3.52160 3.55251 2.76604 4.12549 2.49318 168 - -

2.68618 4.42225 2.77519 2.73123 3.46354 2.40513 3.72494 3.29354 2.67741 2.69355 4.24690 2.90347 2.73739 3.18146 2.89801 2.37887 2.77519 2.98518 4.58477 3.61503

0.03157 3.86699 4.58934 0.61958 0.77255 0.48576 0.95510

169 3.25138 4.65475 4.23121 3.94466 1.02072 3.94245 3.87481 2.77574 3.85183 2.14705 3.50670 4.01301 4.40115 4.02131 3.97290 3.52160 3.55251 2.76604 4.12549 2.49318 169 - -

2.68618 4.42225 2.77519 2.73123 3.46354 2.40513 3.72494 3.29354 2.67741 2.69355 4.24690 2.90347 2.73739 3.18146 2.89801 2.37887 2.77519 2.98518 4.58477 3.61503

0.03157 3.86699 4.58934 0.61958 0.77255 0.48576 0.95510

170 2.99928 4.83880 3.48937 3.00157 4.19200 3.48833 3.82030 3.73332 2.23516 3.25025 4.25056 3.36173 3.99118 3.04089 0.98678 3.08371 3.26560 3.45751 5.27725 4.12054 170 - -

2.68618 4.42225 2.77519 2.73123 3.46354 2.40513 3.72494 3.29354 2.67741 2.69355 4.24690 2.90347 2.73739 3.18146 2.89801 2.37887 2.77519 2.98518 4.58477 3.61503

0.03157 3.86699 4.58934 0.61958 0.77255 0.48576 0.95510

171 2.96489 4.51452 4.04353 3.63658 3.20533 3.82998 4.37299 2.38038 3.40496 1.80083 1.38846 3.91475 4.29056 3.78719 3.61092 3.31343 3.27009 2.38586 5.04542 3.80671 171 - -

2.68618 4.42225 2.77519 2.73123 3.46354 2.40513 3.72494 3.29354 2.67741 2.69355 4.24690 2.90347 2.73739 3.18146 2.89801 2.37887 2.77519 2.98518 4.58477 3.61503

0.03157 3.86699 4.58934 0.61958 0.77255 0.48576 0.95510

172 2.99928 4.83880 3.48937 3.00157 4.19200 3.48833 3.82030 3.73332 2.23516 3.25025 4.25056 3.36173 3.99118 3.04089 0.98678 3.08371 3.26560 3.45751 5.27725 4.12054 172 - -

2.68618 4.42225 2.77519 2.73123 3.46354 2.40513 3.72494 3.29354 2.67741 2.69355 4.24690 2.90347 2.73739 3.18146 2.89801 2.37887 2.77519 2.98518 4.58477 3.61503

0.03157 3.86699 4.58934 0.61958 0.77255 0.48576 0.95510

173 2.96711 4.93068 3.14273 2.76522 4.31586 3.45716 3.75616 3.74479 1.04579 3.31106 4.27280 3.18993 3.95788 2.94794 2.43058 3.00945 3.22078 3.45288 5.37276 4.18474 173 - -

2.68618 4.42225 2.77519 2.73123 3.46354 2.40513 3.72494 3.29354 2.67741 2.69355 4.24690 2.90347 2.73739 3.18146 2.89801 2.37887 2.77519 2.98518 4.58477 3.61503

0.03157 3.86699 4.58934 0.61958 0.77255 0.48576 0.95510

174 1.02168 4.18931 3.45704 3.24808 4.04693 3.01400 4.27077 3.17162 3.26419 3.05349 4.08591 3.39907 3.75270 3.60586 3.51510 2.53965 2.81663 2.82697 5.47463 4.27427 174 - -

2.68618 4.42225 2.77519 2.73123 3.46354 2.40513 3.72494 3.29354 2.67741 2.69355 4.24690 2.90347 2.73739 3.18146 2.89801 2.37887 2.77519 2.98518 4.58477 3.61503

0.03157 3.86699 4.58934 0.61958 0.77255 0.48576 0.95510

175 2.65811 4.43408 3.33076 3.22947 4.37188 0.73761 4.35008 3.97016 3.41000 3.62498 4.60067 3.49506 3.82505 3.73459 3.64665 2.82986 3.13892 3.49954 5.46848 4.47359 175 - -

2.68618 4.42225 2.77519 2.73123 3.46354 2.40513 3.72494 3.29354 2.67741 2.69355 4.24690 2.90347 2.73739 3.18146 2.89801 2.37887 2.77519 2.98518 4.58477 3.61503

0.03157 3.86699 4.58934 0.61958 0.77255 0.48576 0.95510

176 3.14912 4.61302 4.18070 3.82053 3.13362 3.97519 4.47798 2.33736 3.58833 0.88196 3.15827 4.09653 4.41149 3.95797 3.76529 3.54541 3.44362 2.37328 5.01092 3.73297 176 - -

2.68618 4.42225 2.77519 2.73123 3.46354 2.40513 3.72494 3.29354 2.67741 2.69355 4.24690 2.90347 2.73739 3.18146 2.89801 2.37887 2.77519 2.98518 4.58477 3.61503

0.03157 3.86699 4.58934 0.61958 0.77255 0.48576 0.95510

177 2.80376 4.34788 4.11775 3.78070 3.54607 3.71822 4.58336 2.05053 3.64840 2.23405 3.51204 3.97141 4.27903 4.00859 3.86143 3.22141 3.16544 1.03182 5.32959 4.06087 177 - -

2.68618 4.42225 2.77519 2.73123 3.46354 2.40513 3.72494 3.29354 2.67741 2.69355 4.24690 2.90347 2.73739 3.18146 2.89801 2.37887 2.77519 2.98518 4.58477 3.61503

0.03157 3.86699 4.58934 0.61958 0.77255 0.48576 0.95510

178 2.87640 4.86196 2.89930 2.65395 4.02980 3.36241 3.81221 3.65971 2.47512 3.16261 4.19062 3.11364 3.91579 1.24381 2.75940 2.92438 3.17337 3.38973 5.30278 3.99617 178 - -

2.68618 4.42225 2.77519 2.73123 3.46354 2.40513 3.72494 3.29354 2.67741 2.69355 4.24690 2.90347 2.73739 3.18146 2.89801 2.37887 2.77519 2.98518 4.58477 3.61503

0.03157 3.86699 4.58934 0.61958 0.77255 0.48576 0.95510

179 2.74420 4.50247 3.35077 3.18900 4.18468 3.19029 4.26692 3.72867 3.24583 3.35611 4.40571 3.49627 0.83787 3.63478 3.49200 2.91151 3.18532 3.37467 5.37275 4.30526 179 - -

2.68618 4.42225 2.77519 2.73123 3.46354 2.40513 3.72494 3.29354 2.67741 2.69355 4.24690 2.90347 2.73739 3.18146 2.89801 2.37887 2.77519 2.98518 4.58477 3.61503

0.03157 3.86699 4.58934 0.61958 0.77255 0.48576 0.95510

180 2.71888 4.71528 2.70526 2.59018 4.07794 3.18029 3.91656 3.78713 2.81979 3.43461 4.38989 1.16778 3.83188 3.18930 3.17427 2.78628 3.10056 3.40989 5.38448 4.02666 180 - -

2.68618 4.42225 2.77519 2.73123 3.46354 2.40513 3.72494 3.29354 2.67741 2.69355 4.24690 2.90347 2.73739 3.18146 2.89801 2.37887 2.77519 2.98518 4.58477 3.61503

0.03157 3.86699 4.58934 0.61958 0.77255 0.48576 0.95510

181 2.94758 5.05751 2.48529 1.03318 4.40304 3.27053 3.85685 3.85586 2.73669 3.47385 4.44812 2.92979 3.87486 3.07262 3.14497 2.93053 3.25177 3.54026 5.57565 4.29788 181 - -

2.68618 4.42225 2.77519 2.73123 3.46354 2.40513 3.72494 3.29354 2.67741 2.69355 4.24690 2.90347 2.73739 3.18146 2.89801 2.37887 2.77519 2.98518 4.58477 3.61503

0.03157 3.86699 4.58934 0.61958 0.77255 0.48576 0.95510

182 3.23918 4.73039 3.83015 3.55850 2.29358 3.82376 3.61597 3.24916 3.41618 2.70450 3.92476 3.71671 4.29772 3.72354 3.60101 3.37642 3.52583 3.11945 3.93713 0.97494 182 - -

2.68618 4.42225 2.77519 2.73123 3.46354 2.40513 3.72494 3.29354 2.67741 2.69355 4.24690 2.90347 2.73739 3.18146 2.89801 2.37887 2.77519 2.98518 4.58477 3.61503

0.03157 3.86699 4.58934 0.61958 0.77255 0.48576 0.95510

183 2.40976 4.25482 3.42322 3.17496 3.96403 3.09429 4.19270 3.09049 3.11783 2.95418 4.00504 3.38522 3.79760 3.51292 3.37678 2.61097 1.18122 2.78606 5.40231 4.18065 183 - -

2.68618 4.42225 2.77519 2.73123 3.46354 2.40513 3.72494 3.29354 2.67741 2.69355 4.24690 2.90347 2.73739 3.18146 2.89801 2.37887 2.77519 2.98518 4.58477 3.61503

0.03157 3.86699 4.58934 0.61958 0.77255 0.48576 0.95510

184 3.25138 4.65475 4.23121 3.94466 1.02072 3.94245 3.87481 2.77574 3.85183 2.14705 3.50670 4.01301 4.40115 4.02131 3.97290 3.52160 3.55251 2.76604 4.12549 2.49318 184 - -

2.68618 4.42225 2.77519 2.73123 3.46354 2.40513 3.72494 3.29354 2.67741 2.69355 4.24690 2.90347 2.73739 3.18146 2.89801 2.37887 2.77519 2.98518 4.58477 3.61503

0.03157 3.86699 4.58934 0.61958 0.77255 0.48576 0.95510

185 2.80376 4.34788 4.11775 3.78070 3.54607 3.71822 4.58336 2.05053 3.64840 2.23405 3.51204 3.97141 4.27903 4.00859 3.86143 3.22141 3.16544 1.03182 5.32959 4.06087 185 - -

2.68618 4.42225 2.77519 2.73123 3.46354 2.40513 3.72494 3.29354 2.67741 2.69355 4.24690 2.90347 2.73739 3.18146 2.89801 2.37887 2.77519 2.98518 4.58477 3.61503

0.03157 3.86699 4.58934 0.61958 0.77255 0.48576 0.95510

186 1.02168 4.18931 3.45704 3.24808 4.04693 3.01400 4.27077 3.17162 3.26419 3.05349 4.08591 3.39907 3.75270 3.60586 3.51510 2.53965 2.81663 2.82697 5.47463 4.27427 186 - -

2.68618 4.42225 2.77519 2.73123 3.46354 2.40513 3.72494 3.29354 2.67741 2.69355 4.24690 2.90347 2.73739 3.18146 2.89801 2.37887 2.77519 2.98518 4.58477 3.61503

0.03157 3.86699 4.58934 0.61958 0.77255 0.48576 0.95510

187 3.02684 4.44709 4.32791 3.94350 3.37049 4.06063 4.67263 1.08471 3.77153 1.95764 3.30670 4.18960 4.49236 4.12038 3.96872 3.55539 3.32610 1.84575 5.26019 3.99802 187 - -

2.68618 4.42225 2.77519 2.73123 3.46354 2.40513 3.72494 3.29354 2.67741 2.69355 4.24690 2.90347 2.73739 3.18146 2.89801 2.37887 2.77519 2.98518 4.58477 3.61503

0.03157 3.86699 4.58934 0.61958 0.77255 0.48576 0.95510

188 3.14912 4.61302 4.18070 3.82053 3.13362 3.97519 4.47798 2.33736 3.58833 0.88196 3.15827 4.09653 4.41149 3.95797 3.76529 3.54541 3.44362 2.37328 5.01092 3.73297 188 - -

2.68618 4.42225 2.77519 2.73123 3.46354 2.40513 3.72494 3.29354 2.67741 2.69355 4.24690 2.90347 2.73739 3.18146 2.89801 2.37887 2.77519 2.98518 4.58477 3.61503

0.03157 3.86699 4.58934 0.61958 0.77255 0.48576 0.95510

189 2.40976 4.25482 3.42322 3.17496 3.96403 3.09429 4.19270 3.09049 3.11783 2.95418 4.00504 3.38522 3.79760 3.51292 3.37678 2.61097 1.18122 2.78606 5.40231 4.18065 189 - -

2.68618 4.42225 2.77519 2.73123 3.46354 2.40513 3.72494 3.29354 2.67741 2.69355 4.24690 2.90347 2.73739 3.18146 2.89801 2.37887 2.77519 2.98518 4.58477 3.61503

0.03157 3.86699 4.58934 0.61958 0.77255 0.48576 0.95510

190 1.02168 4.18931 3.45704 3.24808 4.04693 3.01400 4.27077 3.17162 3.26419 3.05349 4.08591 3.39907 3.75270 3.60586 3.51510 2.53965 2.81663 2.82697 5.47463 4.27427 190 - -

2.68618 4.42225 2.77519 2.73123 3.46354 2.40513 3.72494 3.29354 2.67741 2.69355 4.24690 2.90347 2.73739 3.18146 2.89801 2.37887 2.77519 2.98518 4.58477 3.61503

0.03157 3.86699 4.58934 0.61958 0.77255 0.48576 0.95510

191 2.58006 0.95161 4.09776 3.83614 3.82875 3.20419 4.48443 2.97175 3.66306 2.88747 4.00858 3.82459 3.91071 4.00937 3.78840 2.85170 3.04637 2.71670 5.24602 4.11647 191 - -

2.68618 4.42225 2.77519 2.73123 3.46354 2.40513 3.72494 3.29354 2.67741 2.69355 4.24690 2.90347 2.73739 3.18146 2.89801 2.37887 2.77519 2.98518 4.58477 3.61503

0.03157 3.86699 4.58934 0.61958 0.77255 0.48576 0.95510

192 2.80376 4.34788 4.11775 3.78070 3.54607 3.71822 4.58336 2.05053 3.64840 2.23405 3.51204 3.97141 4.27903 4.00859 3.86143 3.22141 3.16544 1.03182 5.32959 4.06087 192 - -

2.68618 4.42225 2.77519 2.73123 3.46354 2.40513 3.72494 3.29354 2.67741 2.69355 4.24690 2.90347 2.73739 3.18146 2.89801 2.37887 2.77519 2.98518 4.58477 3.61503

0.03157 3.86699 4.58934 0.61958 0.77255 0.48576 0.95510

193 2.99928 4.83880 3.48937 3.00157 4.19200 3.48833 3.82030 3.73332 2.23516 3.25025 4.25056 3.36173 3.99118 3.04089 0.98678 3.08371 3.26560 3.45751 5.27725 4.12054 193 - -

2.68618 4.42225 2.77519 2.73123 3.46354 2.40513 3.72494 3.29354 2.67741 2.69355 4.24690 2.90347 2.73739 3.18146 2.89801 2.37887 2.77519 2.98518 4.58477 3.61503

0.03157 3.86699 4.58934 0.61958 0.77255 0.48576 0.95510

194 2.80376 4.34788 4.11775 3.78070 3.54607 3.71822 4.58336 2.05053 3.64840 2.23405 3.51204 3.97141 4.27903 4.00859 3.86143 3.22141 3.16544 1.03182 5.32959 4.06087 194 - -

2.68618 4.42225 2.77519 2.73123 3.46354 2.40513 3.72494 3.29354 2.67741 2.69355 4.24690 2.90347 2.73739 3.18146 2.89801 2.37887 2.77519 2.98518 4.58477 3.61503

0.03157 3.86699 4.58934 0.61958 0.77255 0.48576 0.95510

195 2.31230 4.23263 3.20165 3.01716 4.07082 2.96261 4.12717 3.59269 3.09056 3.31334 4.23988 3.24282 3.70096 3.44030 3.37676 1.09934 2.79202 3.12899 5.44375 4.15432 195 - -

2.68618 4.42225 2.77519 2.73123 3.46354 2.40513 3.72494 3.29354 2.67741 2.69355 4.24690 2.90347 2.73739 3.18146 2.89801 2.37887 2.77519 2.98518 4.58477 3.61503

0.03157 3.86699 4.58934 0.61958 0.77255 0.48576 0.95510

196 2.99928 4.83880 3.48937 3.00157 4.19200 3.48833 3.82030 3.73332 2.23516 3.25025 4.25056 3.36173 3.99118 3.04089 0.98678 3.08371 3.26560 3.45751 5.27725 4.12054 196 - -

2.68618 4.42225 2.77519 2.73123 3.46354 2.40513 3.72494 3.29354 2.67741 2.69355 4.24690 2.90347 2.73739 3.18146 2.89801 2.37887 2.77519 2.98518 4.58477 3.61503

0.03157 3.86699 4.58934 0.61958 0.77255 0.48576 0.95510

197 3.25138 4.65475 4.23121 3.94466 1.02072 3.94245 3.87481 2.77574 3.85183 2.14705 3.50670 4.01301 4.40115 4.02131 3.97290 3.52160 3.55251 2.76604 4.12549 2.49318 197 - -

2.68618 4.42225 2.77519 2.73123 3.46354 2.40513 3.72494 3.29354 2.67741 2.69355 4.24690 2.90347 2.73739 3.18146 2.89801 2.37887 2.77519 2.98518 4.58477 3.61503

0.03157 3.86699 4.58934 0.61958 0.77255 0.48576 0.95510

198 2.31230 4.23263 3.20165 3.01716 4.07082 2.96261 4.12717 3.59269 3.09056 3.31334 4.23988 3.24282 3.70096 3.44030 3.37676 1.09934 2.79202 3.12899 5.44375 4.15432 198 - -

2.68618 4.42225 2.77519 2.73123 3.46354 2.40513 3.72494 3.29354 2.67741 2.69355 4.24690 2.90347 2.73739 3.18146 2.89801 2.37887 2.77519 2.98518 4.58477 3.61503

0.03157 3.86699 4.58934 0.61958 0.77255 0.48576 0.95510

199 3.14912 4.61302 4.18070 3.82053 3.13362 3.97519 4.47798 2.33736 3.58833 0.88196 3.15827 4.09653 4.41149 3.95797 3.76529 3.54541 3.44362 2.37328 5.01092 3.73297 199 - -

2.68618 4.42225 2.77519 2.73123 3.46354 2.40513 3.72494 3.29354 2.67741 2.69355 4.24690 2.90347 2.73739 3.18146 2.89801 2.37887 2.77519 2.98518 4.58477 3.61503

0.03157 3.86699 4.58934 0.61958 0.77255 0.48576 0.95510

200 2.65811 4.43408 3.33076 3.22947 4.37188 0.73761 4.35008 3.97016 3.41000 3.62498 4.60067 3.49506 3.82505 3.73459 3.64665 2.82986 3.13892 3.49954 5.46848 4.47359 200 - -

2.68618 4.42225 2.77519 2.73123 3.46354 2.40513 3.72494 3.29354 2.67741 2.69355 4.24690 2.90347 2.73739 3.18146 2.89801 2.37887 2.77519 2.98518 4.58477 3.61503

0.03157 3.86699 4.58934 0.61958 0.77255 0.48576 0.95510

201 3.02684 4.44709 4.32791 3.94350 3.37049 4.06063 4.67263 1.08471 3.77153 1.95764 3.30670 4.18960 4.49236 4.12038 3.96872 3.55539 3.32610 1.84575 5.26019 3.99802 201 - -

2.68618 4.42225 2.77519 2.73123 3.46354 2.40513 3.72494 3.29354 2.67741 2.69355 4.24690 2.90347 2.73739 3.18146 2.89801 2.37887 2.77519 2.98518 4.58477 3.61503

0.03157 3.86699 4.58934 0.61958 0.77255 0.48576 0.95510

202 2.87640 4.86196 2.89930 2.65395 4.02980 3.36241 3.81221 3.65971 2.47512 3.16261 4.19062 3.11364 3.91579 1.24381 2.75940 2.92438 3.17337 3.38973 5.30278 3.99617 202 - -

2.68618 4.42225 2.77519 2.73123 3.46354 2.40513 3.72494 3.29354 2.67741 2.69355 4.24690 2.90347 2.73739 3.18146 2.89801 2.37887 2.77519 2.98518 4.58477 3.61503

0.03157 3.86699 4.58934 0.61958 0.77255 0.48576 0.95510

203 3.02684 4.44709 4.32791 3.94350 3.37049 4.06063 4.67263 1.08471 3.77153 1.95764 3.30670 4.18960 4.49236 4.12038 3.96872 3.55539 3.32610 1.84575 5.26019 3.99802 203 - -

2.68618 4.42225 2.77519 2.73123 3.46354 2.40513 3.72494 3.29354 2.67741 2.69355 4.24690 2.90347 2.73739 3.18146 2.89801 2.37887 2.77519 2.98518 4.58477 3.61503

0.03157 3.86699 4.58934 0.61958 0.77255 0.48576 0.95510

204 2.98886 4.80727 3.12036 2.87975 3.28089 3.44287 1.15410 3.69093 2.72295 3.18558 4.22810 3.28930 3.99510 3.24591 2.98899 3.05959 3.29203 3.42616 4.74602 3.23190 204 - -

2.68618 4.42225 2.77519 2.73123 3.46354 2.40513 3.72494 3.29354 2.67741 2.69355 4.24690 2.90347 2.73739 3.18146 2.89801 2.37887 2.77519 2.98518 4.58477 3.61503
0.03157 3.86699 4.58934 0.61958 0.77255 0.48576 0.95510
205 2.65811 4.43408 3.33076 3.22947 4.37188 0.73761 4.35008 3.97016 3.41000 3.62498 4.60067 3.49506 3.82505 3.73459 3.64665 2.82986 3.13892 3.49954 5.46848 4.47359 205 - -
2.68618 4.42225 2.77519 2.73123 3.46354 2.40513 3.72494 3.29354 2.67741 2.69355 4.24690 2.90347 2.73739 3.18146 2.89801 2.37887 2.77519 2.98518 4.58477 3.61503
0.03157 3.86699 4.58934 0.61958 0.77255 0.48576 0.95510
206 3.14912 4.61302 4.18070 3.82053 3.13362 3.97519 4.47798 2.33736 3.58833 0.88196 3.15827 4.09653 4.41149 3.95797 3.76529 3.54541 3.44362 2.37328 5.01092 3.73297 206 - -
2.68618 4.42225 2.77519 2.73123 3.46354 2.40513 3.72494 3.29354 2.67741 2.69355 4.24690 2.90347 2.73739 3.18146 2.89801 2.37887 2.77519 2.98518 4.58477 3.61503
0.03157 3.86699 4.58934 0.61958 0.77255 0.48576 0.95510
207 3.02684 4.44709 4.32791 3.94350 3.37049 4.06063 4.67263 1.08471 3.77153 1.95764 3.30670 4.18960 4.49236 4.12038 3.96872 3.55539 3.32610 1.84575 5.26019 3.99802 207 - -
2.68618 4.42225 2.77519 2.73123 3.46354 2.40513 3.72494 3.29354 2.67741 2.69355 4.24690 2.90347 2.73739 3.18146 2.89801 2.37887 2.77519 2.98518 4.58477 3.61503
0.03157 3.86699 4.58934 0.61958 0.77255 0.48576 0.95510
208 2.80376 4.34788 4.11775 3.78070 3.54607 3.71822 4.58336 2.05053 3.64840 2.23405 3.51204 3.97141 4.27903 4.00859 3.86143 3.22141 3.16544 1.03182 5.32959 4.06087 208 - -
2.68618 4.42225 2.77519 2.73123 3.46354 2.40513 3.72494 3.29354 2.67741 2.69355 4.24690 2.90347 2.73739 3.18146 2.89801 2.37887 2.77519 2.98518 4.58477 3.61503
0.03157 3.86699 4.58934 0.61958 0.77255 0.48576 0.95510
209 2.96711 4.93068 3.14273 2.76522 4.31586 3.45716 3.75616 3.74479 1.04579 3.31106 4.27280 3.18993 3.95788 2.94794 2.43058 3.00945 3.22078 3.45288 5.37276 4.18474 209 - -
2.68618 4.42225 2.77519 2.73123 3.46354 2.40513 3.72494 3.29354 2.67741 2.69355 4.24690 2.90347 2.73739 3.18146 2.89801 2.37887 2.77519 2.98518 4.58477 3.61503
0.03157 3.86699 4.58934 0.61958 0.77255 0.48576 0.95510
210 2.31230 4.23263 3.20165 3.01716 4.07082 2.96261 4.12717 3.59269 3.09056 3.31334 4.23988 3.24282 3.70096 3.44030 3.37676 1.09934 2.79202 3.12899 5.44375 4.15432 210 - -
2.68618 4.42225 2.77519 2.73123 3.46354 2.40513 3.72494 3.29354 2.67741 2.69355 4.24690 2.90347 2.73739 3.18146 2.89801 2.37887 2.77519 2.98518 4.58477 3.61503
0.03157 3.86699 4.58934 0.61958 0.77255 0.48576 0.95510
211 2.65811 4.43408 3.33076 3.22947 4.37188 0.73761 4.35008 3.97016 3.41000 3.62498 4.60067 3.49506 3.82505 3.73459 3.64665 2.82986 3.13892 3.49954 5.46848 4.47359 211 - -
2.68618 4.42225 2.77519 2.73123 3.46354 2.40513 3.72494 3.29354 2.67741 2.69355 4.24690 2.90347 2.73739 3.18146 2.89801 2.37887 2.77519 2.98518 4.58477 3.61503
0.03157 3.86699 4.58934 0.61958 0.77255 0.48576 0.95510
212 3.25138 4.65475 4.23121 3.94466 1.02072 3.94245 3.87481 2.77574 3.85183 2.14705 3.50670 4.01301 4.40115 4.02131 3.97290 3.52160 3.55251 2.76604 4.12549 2.49318 212 - -
2.68618 4.42225 2.77519 2.73123 3.46354 2.40513 3.72494 3.29354 2.67741 2.69355 4.24690 2.90347 2.73739 3.18146 2.89801 2.37887 2.77519 2.98518 4.58477 3.61503
0.03157 3.86699 4.58934 0.61958 0.77255 0.48576 0.95510
213 3.14912 4.61302 4.18070 3.82053 3.13362 3.97519 4.47798 2.33736 3.58833 0.88196 3.15827 4.09653 4.41149 3.95797 3.76529 3.54541 3.44362 2.37328 5.01092 3.73297 213 - -
2.68618 4.42225 2.77519 2.73123 3.46354 2.40513 3.72494 3.29354 2.67741 2.69355 4.24690 2.90347 2.73739 3.18146 2.89801 2.37887 2.77519 2.98518 4.58477 3.61503
0.03157 3.86699 4.58934 0.61958 0.77255 0.48576 0.95510
214 2.71888 4.71528 2.70526 2.59018 4.07794 3.18029 3.91656 3.78713 2.81979 3.43461 4.38989 1.16778 3.83188 3.18930 3.17427 2.78628 3.10056 3.40989 5.38448 4.02666 214 - -
2.68618 4.42225 2.77519 2.73123 3.46354 2.40513 3.72494 3.29354 2.67741 2.69355 4.24690 2.90347 2.73739 3.18146 2.89801 2.37887 2.77519 2.98518 4.58477 3.61503
0.03157 3.86699 4.58934 0.61958 0.77255 0.48576 0.95510
215 2.31230 4.23263 3.20165 3.01716 4.07082 2.96261 4.12717 3.59269 3.09056 3.31334 4.23988 3.24282 3.70096 3.44030 3.37676 1.09934 2.79202 3.12899 5.44375 4.15432 215 - -
2.68618 4.42225 2.77519 2.73123 3.46354 2.40513 3.72494 3.29354 2.67741 2.69355 4.24690 2.90347 2.73739 3.18146 2.89801 2.37887 2.77519 2.98518 4.58477 3.61503
0.03157 3.86699 4.58934 0.61958 0.77255 0.48576 0.95510

216 2.80376 4.34788 4.11775 3.78070 3.54607 3.71822 4.58336 2.05053 3.64840 2.23405 3.51204 3.97141 4.27903 4.00859 3.86143 3.22141 3.16544 1.03182 5.32959 4.06087 216 - -
2.68618 4.42225 2.77519 2.73123 3.46354 2.40513 3.72494 3.29354 2.67741 2.69355 4.24690 2.90347 2.73739 3.18146 2.89801 2.37887 2.77519 2.98518 4.58477 3.61503
0.03157 3.86699 4.58934 0.61958 0.77255 0.48576 0.95510
217 3.25138 4.65475 4.23121 3.94466 1.02072 3.94245 3.87481 2.77574 3.85183 2.14705 3.50670 4.01301 4.40115 4.02131 3.97290 3.52160 3.55251 2.76604 4.12549 2.49318 217 - -
2.68618 4.42225 2.77519 2.73123 3.46354 2.40513 3.72494 3.29354 2.67741 2.69355 4.24690 2.90347 2.73739 3.18146 2.89801 2.37887 2.77519 2.98518 4.58477 3.61503
0.03157 3.86699 4.58934 0.61958 0.77255 0.48576 0.95510
218 2.80376 4.34788 4.11775 3.78070 3.54607 3.71822 4.58336 2.05053 3.64840 2.23405 3.51204 3.97141 4.27903 4.00859 3.86143 3.22141 3.16544 1.03182 5.32959 4.06087 218 - -
2.68618 4.42225 2.77519 2.73123 3.46354 2.40513 3.72494 3.29354 2.67741 2.69355 4.24690 2.90347 2.73739 3.18146 2.89801 2.37887 2.77519 2.98518 4.58477 3.61503
0.03157 3.86699 4.58934 0.61958 0.77255 0.48576 0.95510
219 2.31230 4.23263 3.20165 3.01716 4.07082 2.96261 4.12717 3.59269 3.09056 3.31334 4.23988 3.24282 3.70096 3.44030 3.37676 1.09934 2.79202 3.12899 5.44375 4.15432 219 - -
2.68618 4.42225 2.77519 2.73123 3.46354 2.40513 3.72494 3.29354 2.67741 2.69355 4.24690 2.90347 2.73739 3.18146 2.89801 2.37887 2.77519 2.98518 4.58477 3.61503
0.03157 3.86699 4.58934 0.61958 0.77255 0.48576 0.95510
220 2.71888 4.71528 2.70526 2.59018 4.07794 3.18029 3.91656 3.78713 2.81979 3.43461 4.38989 1.16778 3.83188 3.18930 3.17427 2.78628 3.10056 3.40989 5.38448 4.02666 220 - -
2.68618 4.42225 2.77519 2.73123 3.46354 2.40513 3.72494 3.29354 2.67741 2.69355 4.24690 2.90347 2.73739 3.18146 2.89801 2.37887 2.77519 2.98518 4.58477 3.61503
0.03157 3.86699 4.58934 0.61958 0.77255 0.48576 0.95510
221 2.31230 4.23263 3.20165 3.01716 4.07082 2.96261 4.12717 3.59269 3.09056 3.31334 4.23988 3.24282 3.70096 3.44030 3.37676 1.09934 2.79202 3.12899 5.44375 4.15432 221 - -
2.68618 4.42225 2.77519 2.73123 3.46354 2.40513 3.72494 3.29354 2.67741 2.69355 4.24690 2.90347 2.73739 3.18146 2.89801 2.37887 2.77519 2.98518 4.58477 3.61503
0.03157 3.86699 4.58934 0.61958 0.77255 0.48576 0.95510
222 3.14912 4.61302 4.18070 3.82053 3.13362 3.97519 4.47798 2.33736 3.58833 0.88196 3.15827 4.09653 4.41149 3.95797 3.76529 3.54541 3.44362 2.37328 5.01092 3.73297 222 - -
2.68618 4.42225 2.77519 2.73123 3.46354 2.40513 3.72494 3.29354 2.67741 2.69355 4.24690 2.90347 2.73739 3.18146 2.89801 2.37887 2.77519 2.98518 4.58477 3.61503
0.03157 3.86699 4.58934 0.61958 0.77255 0.48576 0.95510
223 2.96489 4.51452 4.04353 3.63658 3.20533 3.82998 4.37299 2.38038 3.40496 1.80083 1.38846 3.91475 4.29056 3.78719 3.61092 3.31343 3.27009 2.38586 5.04542 3.80671 223 - -
2.68618 4.42225 2.77519 2.73123 3.46354 2.40513 3.72494 3.29354 2.67741 2.69355 4.24690 2.90347 2.73739 3.18146 2.89801 2.37887 2.77519 2.98518 4.58477 3.61503
0.03157 3.86699 4.58934 0.61958 0.77255 0.48576 0.95510
224 2.31230 4.23263 3.20165 3.01716 4.07082 2.96261 4.12717 3.59269 3.09056 3.31334 4.23988 3.24282 3.70096 3.44030 3.37676 1.09934 2.79202 3.12899 5.44375 4.15432 224 - -
2.68618 4.42225 2.77519 2.73123 3.46354 2.40513 3.72494 3.29354 2.67741 2.69355 4.24690 2.90347 2.73739 3.18146 2.89801 2.37887 2.77519 2.98518 4.58477 3.61503
0.03157 3.86699 4.58934 0.61958 0.77255 0.48576 0.95510
225 3.14912 4.61302 4.18070 3.82053 3.13362 3.97519 4.47798 2.33736 3.58833 0.88196 3.15827 4.09653 4.41149 3.95797 3.76529 3.54541 3.44362 2.37328 5.01092 3.73297 225 - -
2.68618 4.42225 2.77519 2.73123 3.46354 2.40513 3.72494 3.29354 2.67741 2.69355 4.24690 2.90347 2.73739 3.18146 2.89801 2.37887 2.77519 2.98518 4.58477 3.61503
0.03157 3.86699 4.58934 0.61958 0.77255 0.48576 0.95510
226 3.23918 4.73039 3.83015 3.55850 2.29358 3.82376 3.61597 3.24916 3.41618 2.70450 3.92476 3.71671 4.29772 3.72354 3.60101 3.37642 3.52583 3.11945 3.93713 0.97494 226 - -
2.68618 4.42225 2.77519 2.73123 3.46354 2.40513 3.72494 3.29354 2.67741 2.69355 4.24690 2.90347 2.73739 3.18146 2.89801 2.37887 2.77519 2.98518 4.58477 3.61503
0.03157 3.86699 4.58934 0.61958 0.77255 0.48576 0.95510
227 2.98371 5.11116 0.92594 2.33150 4.48744 3.20651 3.92621 4.07436 3.00929 3.68366 4.66164 2.88760 3.85892 3.16534 3.52477 2.94668 3.32216 3.71413 5.65711 4.36124 227 - -
2.68618 4.42225 2.77519 2.73123 3.46354 2.40513 3.72494 3.29354 2.67741 2.69355 4.24690 2.90347 2.73739 3.18146 2.89801 2.37887 2.77519 2.98518 4.58477 3.61503

0.03157 3.86699 4.58934 0.61958 0.77255 0.48576 0.95510

228 2.96711 4.93068 3.14273 2.76522 4.31586 3.45716 3.75616 3.74479 1.04579 3.31106 4.27280 3.18993 3.95788 2.94794 2.43058 3.00945 3.22078 3.45288 5.37276 4.18474 228 - -

2.68618 4.42225 2.77519 2.73123 3.46354 2.40513 3.72494 3.29354 2.67741 2.69355 4.24690 2.90347 2.73739 3.18146 2.89801 2.37887 2.77519 2.98518 4.58477 3.61503

0.03157 3.86699 4.58934 0.61958 0.77255 0.48576 0.95510

229 2.98371 5.11116 0.92594 2.33150 4.48744 3.20651 3.92621 4.07436 3.00929 3.68366 4.66164 2.88760 3.85892 3.16534 3.52477 2.94668 3.32216 3.71413 5.65711 4.36124 229 - -

2.68618 4.42225 2.77519 2.73123 3.46354 2.40513 3.72494 3.29354 2.67741 2.69355 4.24690 2.90347 2.73739 3.18146 2.89801 2.37887 2.77519 2.98518 4.58477 3.61503

0.03157 3.86699 4.58934 0.61958 0.77255 0.48576 0.95510

230 2.31230 4.23263 3.20165 3.01716 4.07082 2.96261 4.12717 3.59269 3.09056 3.31334 4.23988 3.24282 3.70096 3.44030 3.37676 1.09934 2.79202 3.12899 5.44375 4.15432 230 - -

2.68618 4.42225 2.77519 2.73123 3.46354 2.40513 3.72494 3.29354 2.67741 2.69355 4.24690 2.90347 2.73739 3.18146 2.89801 2.37887 2.77519 2.98518 4.58477 3.61503

0.03157 3.86699 4.58934 0.61958 0.77255 0.48576 0.95510

231 2.65811 4.43408 3.33076 3.22947 4.37188 0.73761 4.35008 3.97016 3.41000 3.62498 4.60067 3.49506 3.82505 3.73459 3.64665 2.82986 3.13892 3.49954 5.46848 4.47359 231 - -

2.68618 4.42225 2.77519 2.73123 3.46354 2.40513 3.72494 3.29354 2.67741 2.69355 4.24690 2.90347 2.73739 3.18146 2.89801 2.37887 2.77519 2.98518 4.58477 3.61503

0.03157 3.86699 4.58934 0.61958 0.77255 0.48576 0.95510

232 2.31230 4.23263 3.20165 3.01716 4.07082 2.96261 4.12717 3.59269 3.09056 3.31334 4.23988 3.24282 3.70096 3.44030 3.37676 1.09934 2.79202 3.12899 5.44375 4.15432 232 - -

2.68618 4.42225 2.77519 2.73123 3.46354 2.40513 3.72494 3.29354 2.67741 2.69355 4.24690 2.90347 2.73739 3.18146 2.89801 2.37887 2.77519 2.98518 4.58477 3.61503

0.03157 3.86699 4.58934 0.61958 0.77255 0.48576 0.95510

233 2.31230 4.23263 3.20165 3.01716 4.07082 2.96261 4.12717 3.59269 3.09056 3.31334 4.23988 3.24282 3.70096 3.44030 3.37676 1.09934 2.79202 3.12899 5.44375 4.15432 233 - -

2.68618 4.42225 2.77519 2.73123 3.46354 2.40513 3.72494 3.29354 2.67741 2.69355 4.24690 2.90347 2.73739 3.18146 2.89801 2.37887 2.77519 2.98518 4.58477 3.61503

0.03157 3.86699 4.58934 0.61958 0.77255 0.48576 0.95510

234 2.58006 0.95161 4.09776 3.83614 3.82875 3.20419 4.48443 2.97175 3.66306 2.88747 4.00858 3.82459 3.91071 4.00937 3.78840 2.85170 3.04637 2.71670 5.24602 4.11647 234 - -

2.68618 4.42225 2.77519 2.73123 3.46354 2.40513 3.72494 3.29354 2.67741 2.69355 4.24690 2.90347 2.73739 3.18146 2.89801 2.37887 2.77519 2.98518 4.58477 3.61503

0.03157 3.86699 4.58934 0.61958 0.77255 0.48576 0.95510

235 2.98371 5.11116 0.92594 2.33150 4.48744 3.20651 3.92621 4.07436 3.00929 3.68366 4.66164 2.88760 3.85892 3.16534 3.52477 2.94668 3.32216 3.71413 5.65711 4.36124 235 - -

2.68618 4.42225 2.77519 2.73123 3.46354 2.40513 3.72494 3.29354 2.67741 2.69355 4.24690 2.90347 2.73739 3.18146 2.89801 2.37887 2.77519 2.98518 4.58477 3.61503

0.03157 3.86699 4.58934 0.61958 0.77255 0.48576 0.95510

236 2.98371 5.11116 0.92594 2.33150 4.48744 3.20651 3.92621 4.07436 3.00929 3.68366 4.66164 2.88760 3.85892 3.16534 3.52477 2.94668 3.32216 3.71413 5.65711 4.36124 236 - -

2.68618 4.42225 2.77519 2.73123 3.46354 2.40513 3.72494 3.29354 2.67741 2.69355 4.24690 2.90347 2.73739 3.18146 2.89801 2.37887 2.77519 2.98518 4.58477 3.61503

0.03157 3.86699 4.58934 0.61958 0.77255 0.48576 0.95510

237 2.80376 4.34788 4.11775 3.78070 3.54607 3.71822 4.58336 2.05053 3.64840 2.23405 3.51204 3.97141 4.27903 4.00859 3.86143 3.22141 3.16544 1.03182 5.32959 4.06087 237 - -

2.68618 4.42225 2.77519 2.73123 3.46354 2.40513 3.72494 3.29354 2.67741 2.69355 4.24690 2.90347 2.73739 3.18146 2.89801 2.37887 2.77519 2.98518 4.58477 3.61503

0.03157 3.86699 4.58934 0.61958 0.77255 0.48576 0.95510

238 3.14912 4.61302 4.18070 3.82053 3.13362 3.97519 4.47798 2.33736 3.58833 0.88196 3.15827 4.09653 4.41149 3.95797 3.76529 3.54541 3.44362 2.37328 5.01092 3.73297 238 - -

2.68618 4.42225 2.77519 2.73123 3.46354 2.40513 3.72494 3.29354 2.67741 2.69355 4.24690 2.90347 2.73739 3.18146 2.89801 2.37887 2.77519 2.98518 4.58477 3.61503

0.03157 3.86699 4.58934 0.61958 0.77255 0.48576 0.95510

239 2.96711 4.93068 3.14273 2.76522 4.31586 3.45716 3.75616 3.74479 1.04579 3.31106 4.27280 3.18993 3.95788 2.94794 2.43058 3.00945 3.22078 3.45288 5.37276 4.18474 239 - -

2.68618 4.42225 2.77519 2.73123 3.46354 2.40513 3.72494 3.29354 2.67741 2.69355 4.24690 2.90347 2.73739 3.18146 2.89801 2.37887 2.77519 2.98518 4.58477 3.61503

0.03157 3.86699 4.58934 0.61958 0.77255 0.48576 0.95510

240 3.14912 4.61302 4.18070 3.82053 3.13362 3.97519 4.47798 2.33736 3.58833 0.88196 3.15827 4.09653 4.41149 3.95797 3.76529 3.54541 3.44362 2.37328 5.01092 3.73297 240 - -

2.68618 4.42225 2.77519 2.73123 3.46354 2.40513 3.72494 3.29354 2.67741 2.69355 4.24690 2.90347 2.73739 3.18146 2.89801 2.37887 2.77519 2.98518 4.58477 3.61503

0.03157 3.86699 4.58934 0.61958 0.77255 0.48576 0.95510

241 3.25138 4.65475 4.23121 3.94466 1.02072 3.94245 3.87481 2.77574 3.85183 2.14705 3.50670 4.01301 4.40115 4.02131 3.97290 3.52160 3.55251 2.76604 4.12549 2.49318 241 - -

2.68618 4.42225 2.77519 2.73123 3.46354 2.40513 3.72494 3.29354 2.67741 2.69355 4.24690 2.90347 2.73739 3.18146 2.89801 2.37887 2.77519 2.98518 4.58477 3.61503

0.03157 3.86699 4.58934 0.61958 0.77255 0.48576 0.95510

242 2.98371 5.11116 0.92594 2.33150 4.48744 3.20651 3.92621 4.07436 3.00929 3.68366 4.66164 2.88760 3.85892 3.16534 3.52477 2.94668 3.32216 3.71413 5.65711 4.36124 242 - -

2.68618 4.42225 2.77519 2.73123 3.46354 2.40513 3.72494 3.29354 2.67741 2.69355 4.24690 2.90347 2.73739 3.18146 2.89801 2.37887 2.77519 2.98518 4.58477 3.61503

0.03157 3.86699 4.58934 0.61958 0.77255 0.48576 0.95510

243 2.94758 5.05751 2.48529 1.03318 4.40304 3.27053 3.85685 3.85586 2.73669 3.47385 4.44812 2.92979 3.87486 3.07262 3.14497 2.93053 3.25177 3.54026 5.57565 4.29788 243 - -

2.68618 4.42225 2.77519 2.73123 3.46354 2.40513 3.72494 3.29354 2.67741 2.69355 4.24690 2.90347 2.73739 3.18146 2.89801 2.37887 2.77519 2.98518 4.58477 3.61503

0.03157 3.86699 4.58934 0.61958 0.77255 0.48576 0.95510

244 3.02684 4.44709 4.32791 3.94350 3.37049 4.06063 4.67263 1.08471 3.77153 1.95764 3.30670 4.18960 4.49236 4.12038 3.96872 3.55539 3.32610 1.84575 5.26019 3.99802 244 - -

2.68618 4.42225 2.77519 2.73123 3.46354 2.40513 3.72494 3.29354 2.67741 2.69355 4.24690 2.90347 2.73739 3.18146 2.89801 2.37887 2.77519 2.98518 4.58477 3.61503

0.03157 3.86699 4.58934 0.61958 0.77255 0.48576 0.95510

245 2.74420 4.50247 3.35077 3.18900 4.18468 3.19029 4.26692 3.72867 3.24583 3.35611 4.40571 3.49627 0.83787 3.63478 3.49200 2.91151 3.18532 3.37467 5.37275 4.30526 245 - -

2.68618 4.42225 2.77519 2.73123 3.46354 2.40513 3.72494 3.29354 2.67741 2.69355 4.24690 2.90347 2.73739 3.18146 2.89801 2.37887 2.77519 2.98518 4.58477 3.61503

0.03157 3.86699 4.58934 0.61958 0.77255 0.48576 0.95510

246 2.87640 4.86196 2.89930 2.65395 4.02980 3.36241 3.81221 3.65971 2.47512 3.16261 4.19062 3.11364 3.91579 1.24381 2.75940 2.92438 3.17337 3.38973 5.30278 3.99617 246 - -

2.68618 4.42225 2.77519 2.73123 3.46354 2.40513 3.72494 3.29354 2.67741 2.69355 4.24690 2.90347 2.73739 3.18146 2.89801 2.37887 2.77519 2.98518 4.58477 3.61503

0.03157 3.86699 4.58934 0.61958 0.77255 0.48576 0.95510

247 2.99928 4.83880 3.48937 3.00157 4.19200 3.48833 3.82030 3.73332 2.23516 3.25025 4.25056 3.36173 3.99118 3.04089 0.98678 3.08371 3.26560 3.45751 5.27725 4.12054 247 - -

2.68618 4.42225 2.77519 2.73123 3.46354 2.40513 3.72494 3.29354 2.67741 2.69355 4.24690 2.90347 2.73739 3.18146 2.89801 2.37887 2.77519 2.98518 4.58477 3.61503

0.03157 3.86699 4.58934 0.61958 0.77255 0.48576 0.95510

248 2.98371 5.11116 0.92594 2.33150 4.48744 3.20651 3.92621 4.07436 3.00929 3.68366 4.66164 2.88760 3.85892 3.16534 3.52477 2.94668 3.32216 3.71413 5.65711 4.36124 248 - -

2.68618 4.42225 2.77519 2.73123 3.46354 2.40513 3.72494 3.29354 2.67741 2.69355 4.24690 2.90347 2.73739 3.18146 2.89801 2.37887 2.77519 2.98518 4.58477 3.61503

0.03157 3.86699 4.58934 0.61958 0.77255 0.48576 0.95510

249 2.80376 4.34788 4.11775 3.78070 3.54607 3.71822 4.58336 2.05053 3.64840 2.23405 3.51204 3.97141 4.27903 4.00859 3.86143 3.22141 3.16544 1.03182 5.32959 4.06087 249 - -

2.68618 4.42225 2.77519 2.73123 3.46354 2.40513 3.72494 3.29354 2.67741 2.69355 4.24690 2.90347 2.73739 3.18146 2.89801 2.37887 2.77519 2.98518 4.58477 3.61503

0.03157 3.86699 4.58934 0.61958 0.77255 0.48576 0.95510

250 1.02168 4.18931 3.45704 3.24808 4.04693 3.01400 4.27077 3.17162 3.26419 3.05349 4.08591 3.39907 3.75270 3.60586 3.51510 2.53965 2.81663 2.82697 5.47463 4.27427 250 - -

2.68618 4.42225 2.77519 2.73123 3.46354 2.40513 3.72494 3.29354 2.67741 2.69355 4.24690 2.90347 2.73739 3.18146 2.89801 2.37887 2.77519 2.98518 4.58477 3.61503

0.03157 3.86699 4.58934 0.61958 0.77255 0.48576 0.95510

251 2.31230 4.23263 3.20165 3.01716 4.07082 2.96261 4.12717 3.59269 3.09056 3.31334 4.23988 3.24282 3.70096 3.44030 3.37676 1.09934 2.79202 3.12899 5.44375 4.15432 251 - -
2.68618 4.42225 2.77519 2.73123 3.46354 2.40513 3.72494 3.29354 2.67741 2.69355 4.24690 2.90347 2.73739 3.18146 2.89801 2.37887 2.77519 2.98518 4.58477 3.61503
0.03157 3.86699 4.58934 0.61958 0.77255 0.48576 0.95510
252 3.32650 4.70314 4.08279 3.78752 2.66069 3.72402 3.89311 3.34986 3.50985 2.74569 3.98391 3.93769 4.24129 3.89775 3.63917 3.53025 3.62662 3.24004 0.89641 2.66307 252 - -
2.68618 4.42225 2.77519 2.73123 3.46354 2.40513 3.72494 3.29354 2.67741 2.69355 4.24690 2.90347 2.73739 3.18146 2.89801 2.37887 2.77519 2.98518 4.58477 3.61503
0.03157 3.86699 4.58934 0.61958 0.77255 0.48576 0.95510
253 2.71888 4.71528 2.70526 2.59018 4.07794 3.18029 3.91656 3.78713 2.81979 3.43461 4.38989 1.16778 3.83188 3.18930 3.17427 2.78628 3.10056 3.40989 5.38448 4.02666 253 - -
2.68618 4.42225 2.77519 2.73123 3.46354 2.40513 3.72494 3.29354 2.67741 2.69355 4.24690 2.90347 2.73739 3.18146 2.89801 2.37887 2.77519 2.98518 4.58477 3.61503
0.03157 3.86699 4.58934 0.61958 0.77255 0.48576 0.95510
254 2.40976 4.25482 3.42322 3.17496 3.96403 3.09429 4.19270 3.09049 3.11783 2.95418 4.00504 3.38522 3.79760 3.51292 3.37678 2.61097 1.18122 2.78606 5.40231 4.18065 254 - -
2.68618 4.42225 2.77519 2.73123 3.46354 2.40513 3.72494 3.29354 2.67741 2.69355 4.24690 2.90347 2.73739 3.18146 2.89801 2.37887 2.77519 2.98518 4.58477 3.61503
0.03157 3.86699 4.58934 0.61958 0.77255 0.48576 0.95510
255 2.80376 4.34788 4.11775 3.78070 3.54607 3.71822 4.58336 2.05053 3.64840 2.23405 3.51204 3.97141 4.27903 4.00859 3.86143 3.22141 3.16544 1.03182 5.32959 4.06087 255 - -
2.68618 4.42225 2.77519 2.73123 3.46354 2.40513 3.72494 3.29354 2.67741 2.69355 4.24690 2.90347 2.73739 3.18146 2.89801 2.37887 2.77519 2.98518 4.58477 3.61503
0.03157 3.86699 4.58934 0.61958 0.77255 0.48576 0.95510
256 2.80376 4.34788 4.11775 3.78070 3.54607 3.71822 4.58336 2.05053 3.64840 2.23405 3.51204 3.97141 4.27903 4.00859 3.86143 3.22141 3.16544 1.03182 5.32959 4.06087 256 - -
2.68618 4.42225 2.77519 2.73123 3.46354 2.40513 3.72494 3.29354 2.67741 2.69355 4.24690 2.90347 2.73739 3.18146 2.89801 2.37887 2.77519 2.98518 4.58477 3.61503
0.03157 3.86699 4.58934 0.61958 0.77255 0.48576 0.95510
257 2.31230 4.23263 3.20165 3.01716 4.07082 2.96261 4.12717 3.59269 3.09056 3.31334 4.23988 3.24282 3.70096 3.44030 3.37676 1.09934 2.79202 3.12899 5.44375 4.15432 257 - -
2.68618 4.42225 2.77519 2.73123 3.46354 2.40513 3.72494 3.29354 2.67741 2.69355 4.24690 2.90347 2.73739 3.18146 2.89801 2.37887 2.77519 2.98518 4.58477 3.61503
0.03157 3.86699 4.58934 0.61958 0.77255 0.48576 0.95510
258 2.31230 4.23263 3.20165 3.01716 4.07082 2.96261 4.12717 3.59269 3.09056 3.31334 4.23988 3.24282 3.70096 3.44030 3.37676 1.09934 2.79202 3.12899 5.44375 4.15432 258 - -
2.68618 4.42225 2.77519 2.73123 3.46354 2.40513 3.72494 3.29354 2.67741 2.69355 4.24690 2.90347 2.73739 3.18146 2.89801 2.37887 2.77519 2.98518 4.58477 3.61503
0.03157 3.86699 4.58934 0.61958 0.77255 0.48576 0.95510
259 3.14912 4.61302 4.18070 3.82053 3.13362 3.97519 4.47798 2.33736 3.58833 0.88196 3.15827 4.09653 4.41149 3.95797 3.76529 3.54541 3.44362 2.37328 5.01092 3.73297 259 - -
2.68618 4.42225 2.77519 2.73123 3.46354 2.40513 3.72494 3.29354 2.67741 2.69355 4.24690 2.90347 2.73739 3.18146 2.89801 2.37887 2.77519 2.98518 4.58477 3.61503
0.03157 3.86699 4.58934 0.61958 0.77255 0.48576 0.95510
260 2.80376 4.34788 4.11775 3.78070 3.54607 3.71822 4.58336 2.05053 3.64840 2.23405 3.51204 3.97141 4.27903 4.00859 3.86143 3.22141 3.16544 1.03182 5.32959 4.06087 260 - -
2.68618 4.42225 2.77519 2.73123 3.46354 2.40513 3.72494 3.29354 2.67741 2.69355 4.24690 2.90347 2.73739 3.18146 2.89801 2.37887 2.77519 2.98518 4.58477 3.61503
0.03157 3.86699 4.58934 0.61958 0.77255 0.48576 0.95510
261 2.96711 4.93068 3.14273 2.76522 4.31586 3.45716 3.75616 3.74479 1.04579 3.31106 4.27280 3.18993 3.95788 2.94794 2.43058 3.00945 3.22078 3.45288 5.37276 4.18474 261 - -
2.68618 4.42225 2.77519 2.73123 3.46354 2.40513 3.72494 3.29354 2.67741 2.69355 4.24690 2.90347 2.73739 3.18146 2.89801 2.37887 2.77519 2.98518 4.58477 3.61503
0.03157 3.86699 4.58934 0.61958 0.77255 0.48576 0.95510
262 2.94758 5.05751 2.48529 1.03318 4.40304 3.27053 3.85685 3.85586 2.73669 3.47385 4.44812 2.92979 3.87486 3.07262 3.14497 2.93053 3.25177 3.54026 5.57565 4.29788 262 - -
2.68618 4.42225 2.77519 2.73123 3.46354 2.40513 3.72494 3.29354 2.67741 2.69355 4.24690 2.90347 2.73739 3.18146 2.89801 2.37887 2.77519 2.98518 4.58477 3.61503

0.03157 3.86699 4.58934 0.61958 0.77255 0.48576 0.95510

263 2.65811 4.43408 3.33076 3.22947 4.37188 0.73761 4.35008 3.97016 3.41000 3.62498 4.60067 3.49506 3.82505 3.73459 3.64665 2.82986 3.13892 3.49954 5.46848 4.47359 263 - -

2.68618 4.42225 2.77519 2.73123 3.46354 2.40513 3.72494 3.29354 2.67741 2.69355 4.24690 2.90347 2.73739 3.18146 2.89801 2.37887 2.77519 2.98518 4.58477 3.61503

0.03157 3.86699 4.58934 0.61958 0.77255 0.48576 0.95510

264 2.96711 4.93068 3.14273 2.76522 4.31586 3.45716 3.75616 3.74479 1.04579 3.31106 4.27280 3.18993 3.95788 2.94794 2.43058 3.00945 3.22078 3.45288 5.37276 4.18474 264 - -

2.68618 4.42225 2.77519 2.73123 3.46354 2.40513 3.72494 3.29354 2.67741 2.69355 4.24690 2.90347 2.73739 3.18146 2.89801 2.37887 2.77519 2.98518 4.58477 3.61503

0.03157 3.86699 4.58934 0.61958 0.77255 0.48576 0.95510

265 2.31230 4.23263 3.20165 3.01716 4.07082 2.96261 4.12717 3.59269 3.09056 3.31334 4.23988 3.24282 3.70096 3.44030 3.37676 1.09934 2.79202 3.12899 5.44375 4.15432 265 - -

2.68618 4.42225 2.77519 2.73123 3.46354 2.40513 3.72494 3.29354 2.67741 2.69355 4.24690 2.90347 2.73739 3.18146 2.89801 2.37887 2.77519 2.98518 4.58477 3.61503

0.03157 3.86699 4.58934 0.61958 0.77255 0.48576 0.95510

266 2.98886 4.80727 3.12036 2.87975 3.28089 3.44287 1.15410 3.69093 2.72295 3.18558 4.22810 3.28930 3.99510 3.24591 2.98899 3.05959 3.29203 3.42616 4.74602 3.23190 266 - -

2.68618 4.42225 2.77519 2.73123 3.46354 2.40513 3.72494 3.29354 2.67741 2.69355 4.24690 2.90347 2.73739 3.18146 2.89801 2.37887 2.77519 2.98518 4.58477 3.61503

0.03157 3.86699 4.58934 0.61958 0.77255 0.48576 0.95510

267 2.96711 4.93068 3.14273 2.76522 4.31586 3.45716 3.75616 3.74479 1.04579 3.31106 4.27280 3.18993 3.95788 2.94794 2.43058 3.00945 3.22078 3.45288 5.37276 4.18474 267 - -

2.68618 4.42225 2.77519 2.73123 3.46354 2.40513 3.72494 3.29354 2.67741 2.69355 4.24690 2.90347 2.73739 3.18146 2.89801 2.37887 2.77519 2.98518 4.58477 3.61503

0.03157 3.86699 4.58934 0.61958 0.77255 0.48576 0.95510

268 1.02168 4.18931 3.45704 3.24808 4.04693 3.01400 4.27077 3.17162 3.26419 3.05349 4.08591 3.39907 3.75270 3.60586 3.51510 2.53965 2.81663 2.82697 5.47463 4.27427 268 - -

2.68618 4.42225 2.77519 2.73123 3.46354 2.40513 3.72494 3.29354 2.67741 2.69355 4.24690 2.90347 2.73739 3.18146 2.89801 2.37887 2.77519 2.98518 4.58477 3.61503

0.03157 3.86699 4.58934 0.61958 0.77255 0.48576 0.95510

269 3.25138 4.65475 4.23121 3.94466 1.02072 3.94245 3.87481 2.77574 3.85183 2.14705 3.50670 4.01301 4.40115 4.02131 3.97290 3.52160 3.55251 2.76604 4.12549 2.49318 269 - -

2.68618 4.42225 2.77519 2.73123 3.46354 2.40513 3.72494 3.29354 2.67741 2.69355 4.24690 2.90347 2.73739 3.18146 2.89801 2.37887 2.77519 2.98518 4.58477 3.61503

0.03157 3.86699 4.58934 0.61958 0.77255 0.48576 0.95510

270 2.98371 5.11116 0.92594 2.33150 4.48744 3.20651 3.92621 4.07436 3.00929 3.68366 4.66164 2.88760 3.85892 3.16534 3.52477 2.94668 3.32216 3.71413 5.65711 4.36124 270 - -

2.68618 4.42225 2.77519 2.73123 3.46354 2.40513 3.72494 3.29354 2.67741 2.69355 4.24690 2.90347 2.73739 3.18146 2.89801 2.37887 2.77519 2.98518 4.58477 3.61503

0.03157 3.86699 4.58934 0.61958 0.77255 0.48576 0.95510

271 3.14912 4.61302 4.18070 3.82053 3.13362 3.97519 4.47798 2.33736 3.58833 0.88196 3.15827 4.09653 4.41149 3.95797 3.76529 3.54541 3.44362 2.37328 5.01092 3.73297 271 - -

2.68618 4.42225 2.77519 2.73123 3.46354 2.40513 3.72494 3.29354 2.67741 2.69355 4.24690 2.90347 2.73739 3.18146 2.89801 2.37887 2.77519 2.98518 4.58477 3.61503

0.03157 3.86699 4.58934 0.61958 0.77255 0.48576 0.95510

272 3.25138 4.65475 4.23121 3.94466 1.02072 3.94245 3.87481 2.77574 3.85183 2.14705 3.50670 4.01301 4.40115 4.02131 3.97290 3.52160 3.55251 2.76604 4.12549 2.49318 272 - -

2.68618 4.42225 2.77519 2.73123 3.46354 2.40513 3.72494 3.29354 2.67741 2.69355 4.24690 2.90347 2.73739 3.18146 2.89801 2.37887 2.77519 2.98518 4.58477 3.61503

0.03157 3.86699 4.58934 0.61958 0.77255 0.48576 0.95510

273 3.23918 4.73039 3.83015 3.55850 2.29358 3.82376 3.61597 3.24916 3.41618 2.70450 3.92476 3.71671 4.29772 3.72354 3.60101 3.37642 3.52583 3.11945 3.93713 0.97494 273 - -

2.68618 4.42225 2.77519 2.73123 3.46354 2.40513 3.72494 3.29354 2.67741 2.69355 4.24690 2.90347 2.73739 3.18146 2.89801 2.37887 2.77519 2.98518 4.58477 3.61503

0.03157 3.86699 4.58934 0.61958 0.77255 0.48576 0.95510

274 2.94758 5.05751 2.48529 1.03318 4.40304 3.27053 3.85685 3.85586 2.73669 3.47385 4.44812 2.92979 3.87486 3.07262 3.14497 2.93053 3.25177 3.54026 5.57565 4.29788 274 - -

2.68618 4.42225 2.77519 2.73123 3.46354 2.40513 3.72494 3.29354 2.67741 2.69355 4.24690 2.90347 2.73739 3.18146 2.89801 2.37887 2.77519 2.98518 4.58477 3.61503

0.03157 3.86699 4.58934 0.61958 0.77255 0.48576 0.95510

275 2.96489 4.51452 4.04353 3.63658 3.20533 3.82998 4.37299 2.38038 3.40496 1.80083 1.38846 3.91475 4.29056 3.78719 3.61092 3.31343 3.27009 2.38586 5.04542 3.80671 275 - -

2.68618 4.42225 2.77519 2.73123 3.46354 2.40513 3.72494 3.29354 2.67741 2.69355 4.24690 2.90347 2.73739 3.18146 2.89801 2.37887 2.77519 2.98518 4.58477 3.61503

0.03157 3.86699 4.58934 0.61958 0.77255 0.48576 0.95510

276 2.71888 4.71528 2.70526 2.59018 4.07794 3.18029 3.91656 3.78713 2.81979 3.43461 4.38989 1.16778 3.83188 3.18930 3.17427 2.78628 3.10056 3.40989 5.38448 4.02666 276 - -

2.68618 4.42225 2.77519 2.73123 3.46354 2.40513 3.72494 3.29354 2.67741 2.69355 4.24690 2.90347 2.73739 3.18146 2.89801 2.37887 2.77519 2.98518 4.58477 3.61503

0.03157 3.86699 4.58934 0.61958 0.77255 0.48576 0.95510

277 2.99928 4.83880 3.48937 3.00157 4.19200 3.48833 3.82030 3.73332 2.23516 3.25025 4.25056 3.36173 3.99118 3.04089 0.98678 3.08371 3.26560 3.45751 5.27725 4.12054 277 - -

2.68618 4.42225 2.77519 2.73123 3.46354 2.40513 3.72494 3.29354 2.67741 2.69355 4.24690 2.90347 2.73739 3.18146 2.89801 2.37887 2.77519 2.98518 4.58477 3.61503

0.03157 3.86699 4.58934 0.61958 0.77255 0.48576 0.95510

278 2.80376 4.34788 4.11775 3.78070 3.54607 3.71822 4.58336 2.05053 3.64840 2.23405 3.51204 3.97141 4.27903 4.00859 3.86143 3.22141 3.16544 1.03182 5.32959 4.06087 278 - -

2.68618 4.42225 2.77519 2.73123 3.46354 2.40513 3.72494 3.29354 2.67741 2.69355 4.24690 2.90347 2.73739 3.18146 2.89801 2.37887 2.77519 2.98518 4.58477 3.61503

0.03157 3.86699 4.58934 0.61958 0.77255 0.48576 0.95510

279 2.94758 5.05751 2.48529 1.03318 4.40304 3.27053 3.85685 3.85586 2.73669 3.47385 4.44812 2.92979 3.87486 3.07262 3.14497 2.93053 3.25177 3.54026 5.57565 4.29788 279 - -

2.68618 4.42225 2.77519 2.73123 3.46354 2.40513 3.72494 3.29354 2.67741 2.69355 4.24690 2.90347 2.73739 3.18146 2.89801 2.37887 2.77519 2.98518 4.58477 3.61503

0.03157 3.86699 4.58934 0.61958 0.77255 0.48576 0.95510

280 2.65811 4.43408 3.33076 3.22947 4.37188 0.73761 4.35008 3.97016 3.41000 3.62498 4.60067 3.49506 3.82505 3.73459 3.64665 2.82986 3.13892 3.49954 5.46848 4.47359 280 - -

2.68618 4.42225 2.77519 2.73123 3.46354 2.40513 3.72494 3.29354 2.67741 2.69355 4.24690 2.90347 2.73739 3.18146 2.89801 2.37887 2.77519 2.98518 4.58477 3.61503

0.03157 3.86699 4.58934 0.61958 0.77255 0.48576 0.95510

281 3.25138 4.65475 4.23121 3.94466 1.02072 3.94245 3.87481 2.77574 3.85183 2.14705 3.50670 4.01301 4.40115 4.02131 3.97290 3.52160 3.55251 2.76604 4.12549 2.49318 281 - -

2.68618 4.42225 2.77519 2.73123 3.46354 2.40513 3.72494 3.29354 2.67741 2.69355 4.24690 2.90347 2.73739 3.18146 2.89801 2.37887 2.77519 2.98518 4.58477 3.61503

0.03157 3.86699 4.58934 0.61958 0.77255 0.48576 0.95510

282 2.65811 4.43408 3.33076 3.22947 4.37188 0.73761 4.35008 3.97016 3.41000 3.62498 4.60067 3.49506 3.82505 3.73459 3.64665 2.82986 3.13892 3.49954 5.46848 4.47359 282 - -

2.68618 4.42225 2.77519 2.73123 3.46354 2.40513 3.72494 3.29354 2.67741 2.69355 4.24690 2.90347 2.73739 3.18146 2.89801 2.37887 2.77519 2.98518 4.58477 3.61503

0.03157 3.86699 4.58934 0.61958 0.77255 0.48576 0.95510

283 2.80376 4.34788 4.11775 3.78070 3.54607 3.71822 4.58336 2.05053 3.64840 2.23405 3.51204 3.97141 4.27903 4.00859 3.86143 3.22141 3.16544 1.03182 5.32959 4.06087 283 - -

2.68618 4.42225 2.77519 2.73123 3.46354 2.40513 3.72494 3.29354 2.67741 2.69355 4.24690 2.90347 2.73739 3.18146 2.89801 2.37887 2.77519 2.98518 4.58477 3.61503

0.03157 3.86699 4.58934 0.61958 0.77255 0.48576 0.95510

284 2.98371 5.11116 0.92594 2.33150 4.48744 3.20651 3.92621 4.07436 3.00929 3.68366 4.66164 2.88760 3.85892 3.16534 3.52477 2.94668 3.32216 3.71413 5.65711 4.36124 284 - -

2.68618 4.42225 2.77519 2.73123 3.46354 2.40513 3.72494 3.29354 2.67741 2.69355 4.24690 2.90347 2.73739 3.18146 2.89801 2.37887 2.77519 2.98518 4.58477 3.61503

0.03157 3.86699 4.58934 0.61958 0.77255 0.48576 0.95510

285 2.31230 4.23263 3.20165 3.01716 4.07082 2.96261 4.12717 3.59269 3.09056 3.31334 4.23988 3.24282 3.70096 3.44030 3.37676 1.09934 2.79202 3.12899 5.44375 4.15432 285 - -

2.68618 4.42225 2.77519 2.73123 3.46354 2.40513 3.72494 3.29354 2.67741 2.69355 4.24690 2.90347 2.73739 3.18146 2.89801 2.37887 2.77519 2.98518 4.58477 3.61503

0.03157 3.86699 4.58934 0.61958 0.77255 0.48576 0.95510

286 3.25138 4.65475 4.23121 3.94466 1.02072 3.94245 3.87481 2.77574 3.85183 2.14705 3.50670 4.01301 4.40115 4.02131 3.97290 3.52160 3.55251 2.76604 4.12549 2.49318 286 - -

2.68618 4.42225 2.77519 2.73123 3.46354 2.40513 3.72494 3.29354 2.67741 2.69355 4.24690 2.90347 2.73739 3.18146 2.89801 2.37887 2.77519 2.98518 4.58477 3.61503

0.03157 3.86699 4.58934 0.61958 0.77255 0.48576 0.95510

287 2.40976 4.25482 3.42322 3.17496 3.96403 3.09429 4.19270 3.09049 3.11783 2.95418 4.00504 3.38522 3.79760 3.51292 3.37678 2.61097 1.18122 2.78606 5.40231 4.18065 287 - -

2.68618 4.42225 2.77519 2.73123 3.46354 2.40513 3.72494 3.29354 2.67741 2.69355 4.24690 2.90347 2.73739 3.18146 2.89801 2.37887 2.77519 2.98518 4.58477 3.61503

0.03157 3.86699 4.58934 0.61958 0.77255 0.48576 0.95510

288 3.14912 4.61302 4.18070 3.82053 3.13362 3.97519 4.47798 2.33736 3.58833 0.88196 3.15827 4.09653 4.41149 3.95797 3.76529 3.54541 3.44362 2.37328 5.01092 3.73297 288 - -

2.68618 4.42225 2.77519 2.73123 3.46354 2.40513 3.72494 3.29354 2.67741 2.69355 4.24690 2.90347 2.73739 3.18146 2.89801 2.37887 2.77519 2.98518 4.58477 3.61503

0.03157 3.86699 4.58934 0.61958 0.77255 0.48576 0.95510

289 2.31230 4.23263 3.20165 3.01716 4.07082 2.96261 4.12717 3.59269 3.09056 3.31334 4.23988 3.24282 3.70096 3.44030 3.37676 1.09934 2.79202 3.12899 5.44375 4.15432 289 - -

2.68618 4.42225 2.77519 2.73123 3.46354 2.40513 3.72494 3.29354 2.67741 2.69355 4.24690 2.90347 2.73739 3.18146 2.89801 2.37887 2.77519 2.98518 4.58477 3.61503

0.03157 3.86699 4.58934 0.61958 0.77255 0.48576 0.95510

290 2.40976 4.25482 3.42322 3.17496 3.96403 3.09429 4.19270 3.09049 3.11783 2.95418 4.00504 3.38522 3.79760 3.51292 3.37678 2.61097 1.18122 2.78606 5.40231 4.18065 290 - -

2.68618 4.42225 2.77519 2.73123 3.46354 2.40513 3.72494 3.29354 2.67741 2.69355 4.24690 2.90347 2.73739 3.18146 2.89801 2.37887 2.77519 2.98518 4.58477 3.61503

0.03157 3.86699 4.58934 0.61958 0.77255 0.48576 0.95510

291 3.14912 4.61302 4.18070 3.82053 3.13362 3.97519 4.47798 2.33736 3.58833 0.88196 3.15827 4.09653 4.41149 3.95797 3.76529 3.54541 3.44362 2.37328 5.01092 3.73297 291 - -

2.68618 4.42225 2.77519 2.73123 3.46354 2.40513 3.72494 3.29354 2.67741 2.69355 4.24690 2.90347 2.73739 3.18146 2.89801 2.37887 2.77519 2.98518 4.58477 3.61503

0.03157 3.86699 4.58934 0.61958 0.77255 0.48576 0.95510

292 3.14912 4.61302 4.18070 3.82053 3.13362 3.97519 4.47798 2.33736 3.58833 0.88196 3.15827 4.09653 4.41149 3.95797 3.76529 3.54541 3.44362 2.37328 5.01092 3.73297 292 - -

2.68618 4.42225 2.77519 2.73123 3.46354 2.40513 3.72494 3.29354 2.67741 2.69355 4.24690 2.90347 2.73739 3.18146 2.89801 2.37887 2.77519 2.98518 4.58477 3.61503

0.03157 3.86699 4.58934 0.61958 0.77255 0.48576 0.95510

293 2.31230 4.23263 3.20165 3.01716 4.07082 2.96261 4.12717 3.59269 3.09056 3.31334 4.23988 3.24282 3.70096 3.44030 3.37676 1.09934 2.79202 3.12899 5.44375 4.15432 293 - -

2.68618 4.42225 2.77519 2.73123 3.46354 2.40513 3.72494 3.29354 2.67741 2.69355 4.24690 2.90347 2.73739 3.18146 2.89801 2.37887 2.77519 2.98518 4.58477 3.61503

0.03157 3.86699 4.58934 0.61958 0.77255 0.48576 0.95510

294 2.31230 4.23263 3.20165 3.01716 4.07082 2.96261 4.12717 3.59269 3.09056 3.31334 4.23988 3.24282 3.70096 3.44030 3.37676 1.09934 2.79202 3.12899 5.44375 4.15432 294 - -

2.68618 4.42225 2.77519 2.73123 3.46354 2.40513 3.72494 3.29354 2.67741 2.69355 4.24690 2.90347 2.73739 3.18146 2.89801 2.37887 2.77519 2.98518 4.58477 3.61503

0.03157 3.86699 4.58934 0.61958 0.77255 0.48576 0.95510

295 2.58006 0.95161 4.09776 3.83614 3.82875 3.20419 4.48443 2.97175 3.66306 2.88747 4.00858 3.82459 3.91071 4.00937 3.78840 2.85170 3.04637 2.71670 5.24602 4.11647 295 - -

2.68618 4.42225 2.77519 2.73123 3.46354 2.40513 3.72494 3.29354 2.67741 2.69355 4.24690 2.90347 2.73739 3.18146 2.89801 2.37887 2.77519 2.98518 4.58477 3.61503

0.03157 3.86699 4.58934 0.61958 0.77255 0.48576 0.95510

296 2.40976 4.25482 3.42322 3.17496 3.96403 3.09429 4.19270 3.09049 3.11783 2.95418 4.00504 3.38522 3.79760 3.51292 3.37678 2.61097 1.18122 2.78606 5.40231 4.18065 296 - -

2.68618 4.42225 2.77519 2.73123 3.46354 2.40513 3.72494 3.29354 2.67741 2.69355 4.24690 2.90347 2.73739 3.18146 2.89801 2.37887 2.77519 2.98518 4.58477 3.61503

0.03157 3.86699 4.58934 0.61958 0.77255 0.48576 0.95510

297 2.98371 5.11116 0.92594 2.33150 4.48744 3.20651 3.92621 4.07436 3.00929 3.68366 4.66164 2.88760 3.85892 3.16534 3.52477 2.94668 3.32216 3.71413 5.65711 4.36124 297 - -

2.68618 4.42225 2.77519 2.73123 3.46354 2.40513 3.72494 3.29354 2.67741 2.69355 4.24690 2.90347 2.73739 3.18146 2.89801 2.37887 2.77519 2.98518 4.58477 3.61503

0.03157 3.86699 4.58934 0.61958 0.77255 0.48576 0.95510

298 2.31230 4.23263 3.20165 3.01716 4.07082 2.96261 4.12717 3.59269 3.09056 3.31334 4.23988 3.24282 3.70096 3.44030 3.37676 1.09934 2.79202 3.12899 5.44375 4.15432 298 - -

2.68618 4.42225 2.77519 2.73123 3.46354 2.40513 3.72494 3.29354 2.67741 2.69355 4.24690 2.90347 2.73739 3.18146 2.89801 2.37887 2.77519 2.98518 4.58477 3.61503

0.03157 3.86699 4.58934 0.61958 0.77255 0.48576 0.95510

299 2.31230 4.23263 3.20165 3.01716 4.07082 2.96261 4.12717 3.59269 3.09056 3.31334 4.23988 3.24282 3.70096 3.44030 3.37676 1.09934 2.79202 3.12899 5.44375 4.15432 299 - -

2.68618 4.42225 2.77519 2.73123 3.46354 2.40513 3.72494 3.29354 2.67741 2.69355 4.24690 2.90347 2.73739 3.18146 2.89801 2.37887 2.77519 2.98518 4.58477 3.61503

0.03157 3.86699 4.58934 0.61958 0.77255 0.48576 0.95510

300 2.80376 4.34788 4.11775 3.78070 3.54607 3.71822 4.58336 2.05053 3.64840 2.23405 3.51204 3.97141 4.27903 4.00859 3.86143 3.22141 3.16544 1.03182 5.32959 4.06087 300 - -

2.68618 4.42225 2.77519 2.73123 3.46354 2.40513 3.72494 3.29354 2.67741 2.69355 4.24690 2.90347 2.73739 3.18146 2.89801 2.37887 2.77519 2.98518 4.58477 3.61503

0.03157 3.86699 4.58934 0.61958 0.77255 0.48576 0.95510

301 3.14912 4.61302 4.18070 3.82053 3.13362 3.97519 4.47798 2.33736 3.58833 0.88196 3.15827 4.09653 4.41149 3.95797 3.76529 3.54541 3.44362 2.37328 5.01092 3.73297 301 - -

2.68618 4.42225 2.77519 2.73123 3.46354 2.40513 3.72494 3.29354 2.67741 2.69355 4.24690 2.90347 2.73739 3.18146 2.89801 2.37887 2.77519 2.98518 4.58477 3.61503

0.03157 3.86699 4.58934 0.61958 0.77255 0.48576 0.95510

302 3.14912 4.61302 4.18070 3.82053 3.13362 3.97519 4.47798 2.33736 3.58833 0.88196 3.15827 4.09653 4.41149 3.95797 3.76529 3.54541 3.44362 2.37328 5.01092 3.73297 302 - -

2.68618 4.42225 2.77519 2.73123 3.46354 2.40513 3.72494 3.29354 2.67741 2.69355 4.24690 2.90347 2.73739 3.18146 2.89801 2.37887 2.77519 2.98518 4.58477 3.61503

0.03157 3.86699 4.58934 0.61958 0.77255 0.48576 0.95510

303 2.99928 4.83880 3.48937 3.00157 4.19200 3.48833 3.82030 3.73332 2.23516 3.25025 4.25056 3.36173 3.99118 3.04089 0.98678 3.08371 3.26560 3.45751 5.27725 4.12054 303 - -

2.68618 4.42225 2.77519 2.73123 3.46354 2.40513 3.72494 3.29354 2.67741 2.69355 4.24690 2.90347 2.73739 3.18146 2.89801 2.37887 2.77519 2.98518 4.58477 3.61503

0.03157 3.86699 4.58934 0.61958 0.77255 0.48576 0.95510

304 2.65811 4.43408 3.33076 3.22947 4.37188 0.73761 4.35008 3.97016 3.41000 3.62498 4.60067 3.49506 3.82505 3.73459 3.64665 2.82986 3.13892 3.49954 5.46848 4.47359 304 - -

2.68618 4.42225 2.77519 2.73123 3.46354 2.40513 3.72494 3.29354 2.67741 2.69355 4.24690 2.90347 2.73739 3.18146 2.89801 2.37887 2.77519 2.98518 4.58477 3.61503

0.03157 3.86699 4.58934 0.61958 0.77255 0.48576 0.95510

305 2.99928 4.83880 3.48937 3.00157 4.19200 3.48833 3.82030 3.73332 2.23516 3.25025 4.25056 3.36173 3.99118 3.04089 0.98678 3.08371 3.26560 3.45751 5.27725 4.12054 305 - -

2.68618 4.42225 2.77519 2.73123 3.46354 2.40513 3.72494 3.29354 2.67741 2.69355 4.24690 2.90347 2.73739 3.18146 2.89801 2.37887 2.77519 2.98518 4.58477 3.61503

0.03157 3.86699 4.58934 0.61958 0.77255 0.48576 0.95510

306 2.94758 5.05751 2.48529 1.03318 4.40304 3.27053 3.85685 3.85586 2.73669 3.47385 4.44812 2.92979 3.87486 3.07262 3.14497 2.93053 3.25177 3.54026 5.57565 4.29788 306 - -

2.68618 4.42225 2.77519 2.73123 3.46354 2.40513 3.72494 3.29354 2.67741 2.69355 4.24690 2.90347 2.73739 3.18146 2.89801 2.37887 2.77519 2.98518 4.58477 3.61503

0.03157 3.86699 4.58934 0.61958 0.77255 0.48576 0.95510

307 3.14912 4.61302 4.18070 3.82053 3.13362 3.97519 4.47798 2.33736 3.58833 0.88196 3.15827 4.09653 4.41149 3.95797 3.76529 3.54541 3.44362 2.37328 5.01092 3.73297 307 - -

2.68618 4.42225 2.77519 2.73123 3.46354 2.40513 3.72494 3.29354 2.67741 2.69355 4.24690 2.90347 2.73739 3.18146 2.89801 2.37887 2.77519 2.98518 4.58477 3.61503

0.03157 3.86699 4.58934 0.61958 0.77255 0.48576 0.95510

308 2.98886 4.80727 3.12036 2.87975 3.28089 3.44287 1.15410 3.69093 2.72295 3.18558 4.22810 3.28930 3.99510 3.24591 2.98899 3.05959 3.29203 3.42616 4.74602 3.23190 308 - -

2.68618 4.42225 2.77519 2.73123 3.46354 2.40513 3.72494 3.29354 2.67741 2.69355 4.24690 2.90347 2.73739 3.18146 2.89801 2.37887 2.77519 2.98518 4.58477 3.61503

0.03157 3.86699 4.58934 0.61958 0.77255 0.48576 0.95510

309 2.65811 4.43408 3.33076 3.22947 4.37188 0.73761 4.35008 3.97016 3.41000 3.62498 4.60067 3.49506 3.82505 3.73459 3.64665 2.82986 3.13892 3.49954 5.46848 4.47359 309 - -

2.68618 4.42225 2.77519 2.73123 3.46354 2.40513 3.72494 3.29354 2.67741 2.69355 4.24690 2.90347 2.73739 3.18146 2.89801 2.37887 2.77519 2.98518 4.58477 3.61503

0.03157 3.86699 4.58934 0.61958 0.77255 0.48576 0.95510

310 2.99928 4.83880 3.48937 3.00157 4.19200 3.48833 3.82030 3.73332 2.23516 3.25025 4.25056 3.36173 3.99118 3.04089 0.98678 3.08371 3.26560 3.45751 5.27725 4.12054 310 - -

2.68618 4.42225 2.77519 2.73123 3.46354 2.40513 3.72494 3.29354 2.67741 2.69355 4.24690 2.90347 2.73739 3.18146 2.89801 2.37887 2.77519 2.98518 4.58477 3.61503

0.03157 3.86699 4.58934 0.61958 0.77255 0.48576 0.95510

311 1.02168 4.18931 3.45704 3.24808 4.04693 3.01400 4.27077 3.17162 3.26419 3.05349 4.08591 3.39907 3.75270 3.60586 3.51510 2.53965 2.81663 2.82697 5.47463 4.27427 311 - -

2.68618 4.42225 2.77519 2.73123 3.46354 2.40513 3.72494 3.29354 2.67741 2.69355 4.24690 2.90347 2.73739 3.18146 2.89801 2.37887 2.77519 2.98518 4.58477 3.61503

0.03157 3.86699 4.58934 0.61958 0.77255 0.48576 0.95510

312 3.02684 4.44709 4.32791 3.94350 3.37049 4.06063 4.67263 1.08471 3.77153 1.95764 3.30670 4.18960 4.49236 4.12038 3.96872 3.55539 3.32610 1.84575 5.26019 3.99802 312 - -

2.68618 4.42225 2.77519 2.73123 3.46354 2.40513 3.72494 3.29354 2.67741 2.69355 4.24690 2.90347 2.73739 3.18146 2.89801 2.37887 2.77519 2.98518 4.58477 3.61503

0.03157 3.86699 4.58934 0.61958 0.77255 0.48576 0.95510

313 2.99928 4.83880 3.48937 3.00157 4.19200 3.48833 3.82030 3.73332 2.23516 3.25025 4.25056 3.36173 3.99118 3.04089 0.98678 3.08371 3.26560 3.45751 5.27725 4.12054 313 - -

2.68618 4.42225 2.77519 2.73123 3.46354 2.40513 3.72494 3.29354 2.67741 2.69355 4.24690 2.90347 2.73739 3.18146 2.89801 2.37887 2.77519 2.98518 4.58477 3.61503

0.03157 3.86699 4.58934 0.61958 0.77255 0.48576 0.95510

314 3.02684 4.44709 4.32791 3.94350 3.37049 4.06063 4.67263 1.08471 3.77153 1.95764 3.30670 4.18960 4.49236 4.12038 3.96872 3.55539 3.32610 1.84575 5.26019 3.99802 314 - -

2.68618 4.42225 2.77519 2.73123 3.46354 2.40513 3.72494 3.29354 2.67741 2.69355 4.24690 2.90347 2.73739 3.18146 2.89801 2.37887 2.77519 2.98518 4.58477 3.61503

0.03157 3.86699 4.58934 0.61958 0.77255 0.48576 0.95510

315 2.65811 4.43408 3.33076 3.22947 4.37188 0.73761 4.35008 3.97016 3.41000 3.62498 4.60067 3.49506 3.82505 3.73459 3.64665 2.82986 3.13892 3.49954 5.46848 4.47359 315 - -

2.68618 4.42225 2.77519 2.73123 3.46354 2.40513 3.72494 3.29354 2.67741 2.69355 4.24690 2.90347 2.73739 3.18146 2.89801 2.37887 2.77519 2.98518 4.58477 3.61503

0.03157 3.86699 4.58934 0.61958 0.77255 0.48576 0.95510

316 3.14912 4.61302 4.18070 3.82053 3.13362 3.97519 4.47798 2.33736 3.58833 0.88196 3.15827 4.09653 4.41149 3.95797 3.76529 3.54541 3.44362 2.37328 5.01092 3.73297 316 - -

2.68618 4.42225 2.77519 2.73123 3.46354 2.40513 3.72494 3.29354 2.67741 2.69355 4.24690 2.90347 2.73739 3.18146 2.89801 2.37887 2.77519 2.98518 4.58477 3.61503

0.03157 3.86699 4.58934 0.61958 0.77255 0.48576 0.95510

317 2.96489 4.51452 4.04353 3.63658 3.20533 3.82998 4.37299 2.38038 3.40496 1.80083 1.38846 3.91475 4.29056 3.78719 3.61092 3.31343 3.27009 2.38586 5.04542 3.80671 317 - -

2.68618 4.42225 2.77519 2.73123 3.46354 2.40513 3.72494 3.29354 2.67741 2.69355 4.24690 2.90347 2.73739 3.18146 2.89801 2.37887 2.77519 2.98518 4.58477 3.61503

0.03157 3.86699 4.58934 0.61958 0.77255 0.48576 0.95510

318 2.87640 4.86196 2.89930 2.65395 4.02980 3.36241 3.81221 3.65971 2.47512 3.16261 4.19062 3.11364 3.91579 1.24381 2.75940 2.92438 3.17337 3.38973 5.30278 3.99617 318 - -

2.68618 4.42225 2.77519 2.73123 3.46354 2.40513 3.72494 3.29354 2.67741 2.69355 4.24690 2.90347 2.73739 3.18146 2.89801 2.37887 2.77519 2.98518 4.58477 3.61503

0.03157 3.86699 4.58934 0.61958 0.77255 0.48576 0.95510

319 2.94758 5.05751 2.48529 1.03318 4.40304 3.27053 3.85685 3.85586 2.73669 3.47385 4.44812 2.92979 3.87486 3.07262 3.14497 2.93053 3.25177 3.54026 5.57565 4.29788 319 - -

2.68618 4.42225 2.77519 2.73123 3.46354 2.40513 3.72494 3.29354 2.67741 2.69355 4.24690 2.90347 2.73739 3.18146 2.89801 2.37887 2.77519 2.98518 4.58477 3.61503

0.03157 3.86699 4.58934 0.61958 0.77255 0.48576 0.95510

320 3.14912 4.61302 4.18070 3.82053 3.13362 3.97519 4.47798 2.33736 3.58833 0.88196 3.15827 4.09653 4.41149 3.95797 3.76529 3.54541 3.44362 2.37328 5.01092 3.73297 320 - -

2.68618 4.42225 2.77519 2.73123 3.46354 2.40513 3.72494 3.29354 2.67741 2.69355 4.24690 2.90347 2.73739 3.18146 2.89801 2.37887 2.77519 2.98518 4.58477 3.61503

0.03157 3.86699 4.58934 0.61958 0.77255 0.48576 0.95510

321 2.31230 4.23263 3.20165 3.01716 4.07082 2.96261 4.12717 3.59269 3.09056 3.31334 4.23988 3.24282 3.70096 3.44030 3.37676 1.09934 2.79202 3.12899 5.44375 4.15432 321 - -
2.68618 4.42225 2.77519 2.73123 3.46354 2.40513 3.72494 3.29354 2.67741 2.69355 4.24690 2.90347 2.73739 3.18146 2.89801 2.37887 2.77519 2.98518 4.58477 3.61503
0.03157 3.86699 4.58934 0.61958 0.77255 0.48576 0.95510
322 2.80376 4.34788 4.11775 3.78070 3.54607 3.71822 4.58336 2.05053 3.64840 2.23405 3.51204 3.97141 4.27903 4.00859 3.86143 3.22141 3.16544 1.03182 5.32959 4.06087 322 - -
2.68618 4.42225 2.77519 2.73123 3.46354 2.40513 3.72494 3.29354 2.67741 2.69355 4.24690 2.90347 2.73739 3.18146 2.89801 2.37887 2.77519 2.98518 4.58477 3.61503
0.03157 3.86699 4.58934 0.61958 0.77255 0.48576 0.95510
323 2.71888 4.71528 2.70526 2.59018 4.07794 3.18029 3.91656 3.78713 2.81979 3.43461 4.38989 1.16778 3.83188 3.18930 3.17427 2.78628 3.10056 3.40989 5.38448 4.02666 323 - -
2.68618 4.42225 2.77519 2.73123 3.46354 2.40513 3.72494 3.29354 2.67741 2.69355 4.24690 2.90347 2.73739 3.18146 2.89801 2.37887 2.77519 2.98518 4.58477 3.61503
0.03157 3.86699 4.58934 0.61958 0.77255 0.48576 0.95510
324 2.71888 4.71528 2.70526 2.59018 4.07794 3.18029 3.91656 3.78713 2.81979 3.43461 4.38989 1.16778 3.83188 3.18930 3.17427 2.78628 3.10056 3.40989 5.38448 4.02666 324 - -
2.68618 4.42225 2.77519 2.73123 3.46354 2.40513 3.72494 3.29354 2.67741 2.69355 4.24690 2.90347 2.73739 3.18146 2.89801 2.37887 2.77519 2.98518 4.58477 3.61503
0.03157 3.86699 4.58934 0.61958 0.77255 0.48576 0.95510
325 1.02168 4.18931 3.45704 3.24808 4.04693 3.01400 4.27077 3.17162 3.26419 3.05349 4.08591 3.39907 3.75270 3.60586 3.51510 2.53965 2.81663 2.82697 5.47463 4.27427 325 - -
2.68618 4.42225 2.77519 2.73123 3.46354 2.40513 3.72494 3.29354 2.67741 2.69355 4.24690 2.90347 2.73739 3.18146 2.89801 2.37887 2.77519 2.98518 4.58477 3.61503
0.03157 3.86699 4.58934 0.61958 0.77255 0.48576 0.95510
326 3.14912 4.61302 4.18070 3.82053 3.13362 3.97519 4.47798 2.33736 3.58833 0.88196 3.15827 4.09653 4.41149 3.95797 3.76529 3.54541 3.44362 2.37328 5.01092 3.73297 326 - -
2.68618 4.42225 2.77519 2.73123 3.46354 2.40513 3.72494 3.29354 2.67741 2.69355 4.24690 2.90347 2.73739 3.18146 2.89801 2.37887 2.77519 2.98518 4.58477 3.61503
0.03157 3.86699 4.58934 0.61958 0.77255 0.48576 0.95510
327 3.02684 4.44709 4.32791 3.94350 3.37049 4.06063 4.67263 1.08471 3.77153 1.95764 3.30670 4.18960 4.49236 4.12038 3.96872 3.55539 3.32610 1.84575 5.26019 3.99802 327 - -
2.68618 4.42225 2.77519 2.73123 3.46354 2.40513 3.72494 3.29354 2.67741 2.69355 4.24690 2.90347 2.73739 3.18146 2.89801 2.37887 2.77519 2.98518 4.58477 3.61503
0.03157 3.86699 4.58934 0.61958 0.77255 0.48576 0.95510
328 2.65811 4.43408 3.33076 3.22947 4.37188 0.73761 4.35008 3.97016 3.41000 3.62498 4.60067 3.49506 3.82505 3.73459 3.64665 2.82986 3.13892 3.49954 5.46848 4.47359 328 - -
2.68618 4.42225 2.77519 2.73123 3.46354 2.40513 3.72494 3.29354 2.67741 2.69355 4.24690 2.90347 2.73739 3.18146 2.89801 2.37887 2.77519 2.98518 4.58477 3.61503
0.03157 3.86699 4.58934 0.61958 0.77255 0.48576 0.95510
329 3.25138 4.65475 4.23121 3.94466 1.02072 3.94245 3.87481 2.77574 3.85183 2.14705 3.50670 4.01301 4.40115 4.02131 3.97290 3.52160 3.55251 2.76604 4.12549 2.49318 329 - -
2.68618 4.42225 2.77519 2.73123 3.46354 2.40513 3.72494 3.29354 2.67741 2.69355 4.24690 2.90347 2.73739 3.18146 2.89801 2.37887 2.77519 2.98518 4.58477 3.61503
0.03157 3.86699 4.58934 0.61958 0.77255 0.48576 0.95510
330 3.23918 4.73039 3.83015 3.55850 2.29358 3.82376 3.61597 3.24916 3.41618 2.70450 3.92476 3.71671 4.29772 3.72354 3.60101 3.37642 3.52583 3.11945 3.93713 0.97494 330 - -
2.68618 4.42225 2.77519 2.73123 3.46354 2.40513 3.72494 3.29354 2.67741 2.69355 4.24690 2.90347 2.73739 3.18146 2.89801 2.37887 2.77519 2.98518 4.58477 3.61503
0.03157 3.86699 4.58934 0.61958 0.77255 0.48576 0.95510
331 2.31230 4.23263 3.20165 3.01716 4.07082 2.96261 4.12717 3.59269 3.09056 3.31334 4.23988 3.24282 3.70096 3.44030 3.37676 1.09934 2.79202 3.12899 5.44375 4.15432 331 - -
2.68618 4.42225 2.77519 2.73123 3.46354 2.40513 3.72494 3.29354 2.67741 2.69355 4.24690 2.90347 2.73739 3.18146 2.89801 2.37887 2.77519 2.98518 4.58477 3.61503
0.03157 3.86699 4.58934 0.61958 0.77255 0.48576 0.95510
332 2.96711 4.93068 3.14273 2.76522 4.31586 3.45716 3.75616 3.74479 1.04579 3.31106 4.27280 3.18993 3.95788 2.94794 2.43058 3.00945 3.22078 3.45288 5.37276 4.18474 332 - -
2.68618 4.42225 2.77519 2.73123 3.46354 2.40513 3.72494 3.29354 2.67741 2.69355 4.24690 2.90347 2.73739 3.18146 2.89801 2.37887 2.77519 2.98518 4.58477 3.61503

0.03157 3.86699 4.58934 0.61958 0.77255 0.48576 0.95510

333 3.25138 4.65475 4.23121 3.94466 1.02072 3.94245 3.87481 2.77574 3.85183 2.14705 3.50670 4.01301 4.40115 4.02131 3.97290 3.52160 3.55251 2.76604 4.12549 2.49318 333 - -

2.68618 4.42225 2.77519 2.73123 3.46354 2.40513 3.72494 3.29354 2.67741 2.69355 4.24690 2.90347 2.73739 3.18146 2.89801 2.37887 2.77519 2.98518 4.58477 3.61503

0.03157 3.86699 4.58934 0.61958 0.77255 0.48576 0.95510

334 3.32650 4.70314 4.08279 3.78752 2.66069 3.72402 3.89311 3.34986 3.50985 2.74569 3.98391 3.93769 4.24129 3.89775 3.63917 3.53025 3.62662 3.24004 0.89641 2.66307 334 - -

2.68618 4.42225 2.77519 2.73123 3.46354 2.40513 3.72494 3.29354 2.67741 2.69355 4.24690 2.90347 2.73739 3.18146 2.89801 2.37887 2.77519 2.98518 4.58477 3.61503

0.03157 3.86699 4.58934 0.61958 0.77255 0.48576 0.95510

335 2.98371 5.11116 0.92594 2.33150 4.48744 3.20651 3.92621 4.07436 3.00929 3.68366 4.66164 2.88760 3.85892 3.16534 3.52477 2.94668 3.32216 3.71413 5.65711 4.36124 335 - -

2.68618 4.42225 2.77519 2.73123 3.46354 2.40513 3.72494 3.29354 2.67741 2.69355 4.24690 2.90347 2.73739 3.18146 2.89801 2.37887 2.77519 2.98518 4.58477 3.61503

0.03157 3.86699 4.58934 0.61958 0.77255 0.48576 0.95510

336 2.96489 4.51452 4.04353 3.63658 3.20533 3.82998 4.37299 2.38038 3.40496 1.80083 1.38846 3.91475 4.29056 3.78719 3.61092 3.31343 3.27009 2.38586 5.04542 3.80671 336 - -

2.68618 4.42225 2.77519 2.73123 3.46354 2.40513 3.72494 3.29354 2.67741 2.69355 4.24690 2.90347 2.73739 3.18146 2.89801 2.37887 2.77519 2.98518 4.58477 3.61503

0.03157 3.86699 4.58934 0.61958 0.77255 0.48576 0.95510

337 2.96711 4.93068 3.14273 2.76522 4.31586 3.45716 3.75616 3.74479 1.04579 3.31106 4.27280 3.18993 3.95788 2.94794 2.43058 3.00945 3.22078 3.45288 5.37276 4.18474 337 - -

2.68618 4.42225 2.77519 2.73123 3.46354 2.40513 3.72494 3.29354 2.67741 2.69355 4.24690 2.90347 2.73739 3.18146 2.89801 2.37887 2.77519 2.98518 4.58477 3.61503

0.03157 3.86699 4.58934 0.61958 0.77255 0.48576 0.95510

338 2.96711 4.93068 3.14273 2.76522 4.31586 3.45716 3.75616 3.74479 1.04579 3.31106 4.27280 3.18993 3.95788 2.94794 2.43058 3.00945 3.22078 3.45288 5.37276 4.18474 338 - -

2.68618 4.42225 2.77519 2.73123 3.46354 2.40513 3.72494 3.29354 2.67741 2.69355 4.24690 2.90347 2.73739 3.18146 2.89801 2.37887 2.77519 2.98518 4.58477 3.61503

0.03157 3.86699 4.58934 0.61958 0.77255 0.48576 0.95510

339 2.80376 4.34788 4.11775 3.78070 3.54607 3.71822 4.58336 2.05053 3.64840 2.23405 3.51204 3.97141 4.27903 4.00859 3.86143 3.22141 3.16544 1.03182 5.32959 4.06087 339 - -

2.68618 4.42225 2.77519 2.73123 3.46354 2.40513 3.72494 3.29354 2.67741 2.69355 4.24690 2.90347 2.73739 3.18146 2.89801 2.37887 2.77519 2.98518 4.58477 3.61503

0.03157 3.86699 4.58934 0.61958 0.77255 0.48576 0.95510

340 2.94758 5.05751 2.48529 1.03318 4.40304 3.27053 3.85685 3.85586 2.73669 3.47385 4.44812 2.92979 3.87486 3.07262 3.14497 2.93053 3.25177 3.54026 5.57565 4.29788 340 - -

2.68618 4.42225 2.77519 2.73123 3.46354 2.40513 3.72494 3.29354 2.67741 2.69355 4.24690 2.90347 2.73739 3.18146 2.89801 2.37887 2.77519 2.98518 4.58477 3.61503

0.03157 3.86699 4.58934 0.61958 0.77255 0.48576 0.95510

341 2.31230 4.23263 3.20165 3.01716 4.07082 2.96261 4.12717 3.59269 3.09056 3.31334 4.23988 3.24282 3.70096 3.44030 3.37676 1.09934 2.79202 3.12899 5.44375 4.15432 341 - -

2.68618 4.42225 2.77519 2.73123 3.46354 2.40513 3.72494 3.29354 2.67741 2.69355 4.24690 2.90347 2.73739 3.18146 2.89801 2.37887 2.77519 2.98518 4.58477 3.61503

0.03157 3.86699 4.58934 0.61958 0.77255 0.48576 0.95510

342 3.14912 4.61302 4.18070 3.82053 3.13362 3.97519 4.47798 2.33736 3.58833 0.88196 3.15827 4.09653 4.41149 3.95797 3.76529 3.54541 3.44362 2.37328 5.01092 3.73297 342 - -

2.68618 4.42225 2.77519 2.73123 3.46354 2.40513 3.72494 3.29354 2.67741 2.69355 4.24690 2.90347 2.73739 3.18146 2.89801 2.37887 2.77519 2.98518 4.58477 3.61503

0.03157 3.86699 4.58934 0.61958 0.77255 0.48576 0.95510

343 3.23918 4.73039 3.83015 3.55850 2.29358 3.82376 3.61597 3.24916 3.41618 2.70450 3.92476 3.71671 4.29772 3.72354 3.60101 3.37642 3.52583 3.11945 3.93713 0.97494 343 - -

2.68618 4.42225 2.77519 2.73123 3.46354 2.40513 3.72494 3.29354 2.67741 2.69355 4.24690 2.90347 2.73739 3.18146 2.89801 2.37887 2.77519 2.98518 4.58477 3.61503

0.03157 3.86699 4.58934 0.61958 0.77255 0.48576 0.95510

344 2.94758 5.05751 2.48529 1.03318 4.40304 3.27053 3.85685 3.85586 2.73669 3.47385 4.44812 2.92979 3.87486 3.07262 3.14497 2.93053 3.25177 3.54026 5.57565 4.29788 344 - -

2.68618 4.42225 2.77519 2.73123 3.46354 2.40513 3.72494 3.29354 2.67741 2.69355 4.24690 2.90347 2.73739 3.18146 2.89801 2.37887 2.77519 2.98518 4.58477 3.61503

0.03157 3.86699 4.58934 0.61958 0.77255 0.48576 0.95510

345 2.96489 4.51452 4.04353 3.63658 3.20533 3.82998 4.37299 2.38038 3.40496 1.80083 1.38846 3.91475 4.29056 3.78719 3.61092 3.31343 3.27009 2.38586 5.04542 3.80671 345 - -

2.68618 4.42225 2.77519 2.73123 3.46354 2.40513 3.72494 3.29354 2.67741 2.69355 4.24690 2.90347 2.73739 3.18146 2.89801 2.37887 2.77519 2.98518 4.58477 3.61503

0.03157 3.86699 4.58934 0.61958 0.77255 0.48576 0.95510

346 2.96489 4.51452 4.04353 3.63658 3.20533 3.82998 4.37299 2.38038 3.40496 1.80083 1.38846 3.91475 4.29056 3.78719 3.61092 3.31343 3.27009 2.38586 5.04542 3.80671 346 - -

2.68618 4.42225 2.77519 2.73123 3.46354 2.40513 3.72494 3.29354 2.67741 2.69355 4.24690 2.90347 2.73739 3.18146 2.89801 2.37887 2.77519 2.98518 4.58477 3.61503

0.03157 3.86699 4.58934 0.61958 0.77255 0.48576 0.95510

347 2.96489 4.51452 4.04353 3.63658 3.20533 3.82998 4.37299 2.38038 3.40496 1.80083 1.38846 3.91475 4.29056 3.78719 3.61092 3.31343 3.27009 2.38586 5.04542 3.80671 347 - -

2.68618 4.42225 2.77519 2.73123 3.46354 2.40513 3.72494 3.29354 2.67741 2.69355 4.24690 2.90347 2.73739 3.18146 2.89801 2.37887 2.77519 2.98518 4.58477 3.61503

0.03157 3.86699 4.58934 0.61958 0.77255 0.48576 0.95510

348 1.02168 4.18931 3.45704 3.24808 4.04693 3.01400 4.27077 3.17162 3.26419 3.05349 4.08591 3.39907 3.75270 3.60586 3.51510 2.53965 2.81663 2.82697 5.47463 4.27427 348 - -

2.68618 4.42225 2.77519 2.73123 3.46354 2.40513 3.72494 3.29354 2.67741 2.69355 4.24690 2.90347 2.73739 3.18146 2.89801 2.37887 2.77519 2.98518 4.58477 3.61503

0.03157 3.86699 4.58934 0.61958 0.77255 0.48576 0.95510

349 2.87640 4.86196 2.89930 2.65395 4.02980 3.36241 3.81221 3.65971 2.47512 3.16261 4.19062 3.11364 3.91579 1.24381 2.75940 2.92438 3.17337 3.38973 5.30278 3.99617 349 - -

2.68618 4.42225 2.77519 2.73123 3.46354 2.40513 3.72494 3.29354 2.67741 2.69355 4.24690 2.90347 2.73739 3.18146 2.89801 2.37887 2.77519 2.98518 4.58477 3.61503

0.03157 3.86699 4.58934 0.61958 0.77255 0.48576 0.95510

350 2.98371 5.11116 0.92594 2.33150 4.48744 3.20651 3.92621 4.07436 3.00929 3.68366 4.66164 2.88760 3.85892 3.16534 3.52477 2.94668 3.32216 3.71413 5.65711 4.36124 350 - -

2.68618 4.42225 2.77519 2.73123 3.46354 2.40513 3.72494 3.29354 2.67741 2.69355 4.24690 2.90347 2.73739 3.18146 2.89801 2.37887 2.77519 2.98518 4.58477 3.61503

0.03157 3.86699 4.58934 0.61958 0.77255 0.48576 0.95510

351 1.02168 4.18931 3.45704 3.24808 4.04693 3.01400 4.27077 3.17162 3.26419 3.05349 4.08591 3.39907 3.75270 3.60586 3.51510 2.53965 2.81663 2.82697 5.47463 4.27427 351 - -

2.68618 4.42225 2.77519 2.73123 3.46354 2.40513 3.72494 3.29354 2.67741 2.69355 4.24690 2.90347 2.73739 3.18146 2.89801 2.37887 2.77519 2.98518 4.58477 3.61503

0.03157 3.86699 4.58934 0.61958 0.77255 0.48576 0.95510

352 2.80376 4.34788 4.11775 3.78070 3.54607 3.71822 4.58336 2.05053 3.64840 2.23405 3.51204 3.97141 4.27903 4.00859 3.86143 3.22141 3.16544 1.03182 5.32959 4.06087 352 - -

2.68618 4.42225 2.77519 2.73123 3.46354 2.40513 3.72494 3.29354 2.67741 2.69355 4.24690 2.90347 2.73739 3.18146 2.89801 2.37887 2.77519 2.98518 4.58477 3.61503

0.03157 3.86699 4.58934 0.61958 0.77255 0.48576 0.95510

353 2.40976 4.25482 3.42322 3.17496 3.96403 3.09429 4.19270 3.09049 3.11783 2.95418 4.00504 3.38522 3.79760 3.51292 3.37678 2.61097 1.18122 2.78606 5.40231 4.18065 353 - -

2.68618 4.42225 2.77519 2.73123 3.46354 2.40513 3.72494 3.29354 2.67741 2.69355 4.24690 2.90347 2.73739 3.18146 2.89801 2.37887 2.77519 2.98518 4.58477 3.61503

0.03157 3.86699 4.58934 0.61958 0.77255 0.48576 0.95510

354 3.25138 4.65475 4.23121 3.94466 1.02072 3.94245 3.87481 2.77574 3.85183 2.14705 3.50670 4.01301 4.40115 4.02131 3.97290 3.52160 3.55251 2.76604 4.12549 2.49318 354 - -

2.68618 4.42225 2.77519 2.73123 3.46354 2.40513 3.72494 3.29354 2.67741 2.69355 4.24690 2.90347 2.73739 3.18146 2.89801 2.37887 2.77519 2.98518 4.58477 3.61503

0.03157 3.86699 4.58934 0.61958 0.77255 0.48576 0.95510

355 2.40976 4.25482 3.42322 3.17496 3.96403 3.09429 4.19270 3.09049 3.11783 2.95418 4.00504 3.38522 3.79760 3.51292 3.37678 2.61097 1.18122 2.78606 5.40231 4.18065 355 - -

2.68618 4.42225 2.77519 2.73123 3.46354 2.40513 3.72494 3.29354 2.67741 2.69355 4.24690 2.90347 2.73739 3.18146 2.89801 2.37887 2.77519 2.98518 4.58477 3.61503

0.03157 3.86699 4.58934 0.61958 0.77255 0.48576 0.95510

356 2.94758 5.05751 2.48529 1.03318 4.40304 3.27053 3.85685 3.85586 2.73669 3.47385 4.44812 2.92979 3.87486 3.07262 3.14497 2.93053 3.25177 3.54026 5.57565 4.29788 356 - -
2.68618 4.42225 2.77519 2.73123 3.46354 2.40513 3.72494 3.29354 2.67741 2.69355 4.24690 2.90347 2.73739 3.18146 2.89801 2.37887 2.77519 2.98518 4.58477 3.61503
0.03157 3.86699 4.58934 0.61958 0.77255 0.48576 0.95510
357 2.96489 4.51452 4.04353 3.63658 3.20533 3.82998 4.37299 2.38038 3.40496 1.80083 1.38846 3.91475 4.29056 3.78719 3.61092 3.31343 3.27009 2.38586 5.04542 3.80671 357 - -
2.68618 4.42225 2.77519 2.73123 3.46354 2.40513 3.72494 3.29354 2.67741 2.69355 4.24690 2.90347 2.73739 3.18146 2.89801 2.37887 2.77519 2.98518 4.58477 3.61503
0.03157 3.86699 4.58934 0.61958 0.77255 0.48576 0.95510
358 3.02684 4.44709 4.32791 3.94350 3.37049 4.06063 4.67263 1.08471 3.77153 1.95764 3.30670 4.18960 4.49236 4.12038 3.96872 3.55539 3.32610 1.84575 5.26019 3.99802 358 - -
2.68618 4.42225 2.77519 2.73123 3.46354 2.40513 3.72494 3.29354 2.67741 2.69355 4.24690 2.90347 2.73739 3.18146 2.89801 2.37887 2.77519 2.98518 4.58477 3.61503
0.03157 3.86699 4.58934 0.61958 0.77255 0.48576 0.95510
359 2.40976 4.25482 3.42322 3.17496 3.96403 3.09429 4.19270 3.09049 3.11783 2.95418 4.00504 3.38522 3.79760 3.51292 3.37678 2.61097 1.18122 2.78606 5.40231 4.18065 359 - -
2.68618 4.42225 2.77519 2.73123 3.46354 2.40513 3.72494 3.29354 2.67741 2.69355 4.24690 2.90347 2.73739 3.18146 2.89801 2.37887 2.77519 2.98518 4.58477 3.61503
0.03157 3.86699 4.58934 0.61958 0.77255 0.48576 0.95510
360 1.02168 4.18931 3.45704 3.24808 4.04693 3.01400 4.27077 3.17162 3.26419 3.05349 4.08591 3.39907 3.75270 3.60586 3.51510 2.53965 2.81663 2.82697 5.47463 4.27427 360 - -
2.68618 4.42225 2.77519 2.73123 3.46354 2.40513 3.72494 3.29354 2.67741 2.69355 4.24690 2.90347 2.73739 3.18146 2.89801 2.37887 2.77519 2.98518 4.58477 3.61503
0.03157 3.86699 4.58934 0.61958 0.77255 0.48576 0.95510
361 3.23918 4.73039 3.83015 3.55850 2.29358 3.82376 3.61597 3.24916 3.41618 2.70450 3.92476 3.71671 4.29772 3.72354 3.60101 3.37642 3.52583 3.11945 3.93713 0.97494 361 - -
2.68618 4.42225 2.77519 2.73123 3.46354 2.40513 3.72494 3.29354 2.67741 2.69355 4.24690 2.90347 2.73739 3.18146 2.89801 2.37887 2.77519 2.98518 4.58477 3.61503
0.03157 3.86699 4.58934 0.61958 0.77255 0.48576 0.95510
362 2.96489 4.51452 4.04353 3.63658 3.20533 3.82998 4.37299 2.38038 3.40496 1.80083 1.38846 3.91475 4.29056 3.78719 3.61092 3.31343 3.27009 2.38586 5.04542 3.80671 362 - -
2.68618 4.42225 2.77519 2.73123 3.46354 2.40513 3.72494 3.29354 2.67741 2.69355 4.24690 2.90347 2.73739 3.18146 2.89801 2.37887 2.77519 2.98518 4.58477 3.61503
0.03157 3.86699 4.58934 0.61958 0.77255 0.48576 0.95510
363 2.31230 4.23263 3.20165 3.01716 4.07082 2.96261 4.12717 3.59269 3.09056 3.31334 4.23988 3.24282 3.70096 3.44030 3.37676 1.09934 2.79202 3.12899 5.44375 4.15432 363 - -
2.68618 4.42225 2.77519 2.73123 3.46354 2.40513 3.72494 3.29354 2.67741 2.69355 4.24690 2.90347 2.73739 3.18146 2.89801 2.37887 2.77519 2.98518 4.58477 3.61503
0.03157 3.86699 4.58934 0.61958 0.77255 0.48576 0.95510
364 3.25138 4.65475 4.23121 3.94466 1.02072 3.94245 3.87481 2.77574 3.85183 2.14705 3.50670 4.01301 4.40115 4.02131 3.97290 3.52160 3.55251 2.76604 4.12549 2.49318 364 - -
2.68618 4.42225 2.77519 2.73123 3.46354 2.40513 3.72494 3.29354 2.67741 2.69355 4.24690 2.90347 2.73739 3.18146 2.89801 2.37887 2.77519 2.98518 4.58477 3.61503
0.03157 3.86699 4.58934 0.61958 0.77255 0.48576 0.95510
365 2.65811 4.43408 3.33076 3.22947 4.37188 0.73761 4.35008 3.97016 3.41000 3.62498 4.60067 3.49506 3.82505 3.73459 3.64665 2.82986 3.13892 3.49954 5.46848 4.47359 365 - -
2.68618 4.42225 2.77519 2.73123 3.46354 2.40513 3.72494 3.29354 2.67741 2.69355 4.24690 2.90347 2.73739 3.18146 2.89801 2.37887 2.77519 2.98518 4.58477 3.61503
0.03157 3.86699 4.58934 0.61958 0.77255 0.48576 0.95510
366 2.96489 4.51452 4.04353 3.63658 3.20533 3.82998 4.37299 2.38038 3.40496 1.80083 1.38846 3.91475 4.29056 3.78719 3.61092 3.31343 3.27009 2.38586 5.04542 3.80671 366 - -
2.68618 4.42225 2.77519 2.73123 3.46354 2.40513 3.72494 3.29354 2.67741 2.69355 4.24690 2.90347 2.73739 3.18146 2.89801 2.37887 2.77519 2.98518 4.58477 3.61503
0.03157 3.86699 4.58934 0.61958 0.77255 0.48576 0.95510
367 2.80376 4.34788 4.11775 3.78070 3.54607 3.71822 4.58336 2.05053 3.64840 2.23405 3.51204 3.97141 4.27903 4.00859 3.86143 3.22141 3.16544 1.03182 5.32959 4.06087 367 - -
2.68618 4.42225 2.77519 2.73123 3.46354 2.40513 3.72494 3.29354 2.67741 2.69355 4.24690 2.90347 2.73739 3.18146 2.89801 2.37887 2.77519 2.98518 4.58477 3.61503

0.03157 3.86699 4.58934 0.61958 0.77255 0.48576 0.95510

368 2.98371 5.11116 0.92594 2.33150 4.48744 3.20651 3.92621 4.07436 3.00929 3.68366 4.66164 2.88760 3.85892 3.16534 3.52477 2.94668 3.32216 3.71413 5.65711 4.36124 368 - -

2.68618 4.42225 2.77519 2.73123 3.46354 2.40513 3.72494 3.29354 2.67741 2.69355 4.24690 2.90347 2.73739 3.18146 2.89801 2.37887 2.77519 2.98518 4.58477 3.61503

0.03157 3.86699 4.58934 0.61958 0.77255 0.48576 0.95510

369 2.31230 4.23263 3.20165 3.01716 4.07082 2.96261 4.12717 3.59269 3.09056 3.31334 4.23988 3.24282 3.70096 3.44030 3.37676 1.09934 2.79202 3.12899 5.44375 4.15432 369 - -

2.68618 4.42225 2.77519 2.73123 3.46354 2.40513 3.72494 3.29354 2.67741 2.69355 4.24690 2.90347 2.73739 3.18146 2.89801 2.37887 2.77519 2.98518 4.58477 3.61503

0.03157 3.86699 4.58934 0.61958 0.77255 0.48576 0.95510

370 1.02168 4.18931 3.45704 3.24808 4.04693 3.01400 4.27077 3.17162 3.26419 3.05349 4.08591 3.39907 3.75270 3.60586 3.51510 2.53965 2.81663 2.82697 5.47463 4.27427 370 - -

2.68618 4.42225 2.77519 2.73123 3.46354 2.40513 3.72494 3.29354 2.67741 2.69355 4.24690 2.90347 2.73739 3.18146 2.89801 2.37887 2.77519 2.98518 4.58477 3.61503

0.03157 3.86699 4.58934 0.61958 0.77255 0.48576 0.95510

371 2.80376 4.34788 4.11775 3.78070 3.54607 3.71822 4.58336 2.05053 3.64840 2.23405 3.51204 3.97141 4.27903 4.00859 3.86143 3.22141 3.16544 1.03182 5.32959 4.06087 371 - -

2.68618 4.42225 2.77519 2.73123 3.46354 2.40513 3.72494 3.29354 2.67741 2.69355 4.24690 2.90347 2.73739 3.18146 2.89801 2.37887 2.77519 2.98518 4.58477 3.61503

0.03157 3.86699 4.58934 0.61958 0.77255 0.48576 0.95510

372 2.94758 5.05751 2.48529 1.03318 4.40304 3.27053 3.85685 3.85586 2.73669 3.47385 4.44812 2.92979 3.87486 3.07262 3.14497 2.93053 3.25177 3.54026 5.57565 4.29788 372 - -

2.68618 4.42225 2.77519 2.73123 3.46354 2.40513 3.72494 3.29354 2.67741 2.69355 4.24690 2.90347 2.73739 3.18146 2.89801 2.37887 2.77519 2.98518 4.58477 3.61503

0.03157 3.86699 4.58934 0.61958 0.77255 0.48576 0.95510

373 3.02684 4.44709 4.32791 3.94350 3.37049 4.06063 4.67263 1.08471 3.77153 1.95764 3.30670 4.18960 4.49236 4.12038 3.96872 3.55539 3.32610 1.84575 5.26019 3.99802 373 - -

2.68618 4.42225 2.77519 2.73123 3.46354 2.40513 3.72494 3.29354 2.67741 2.69355 4.24690 2.90347 2.73739 3.18146 2.89801 2.37887 2.77519 2.98518 4.58477 3.61503

0.03157 3.86699 4.58934 0.61958 0.77255 0.48576 0.95510

374 3.25138 4.65475 4.23121 3.94466 1.02072 3.94245 3.87481 2.77574 3.85183 2.14705 3.50670 4.01301 4.40115 4.02131 3.97290 3.52160 3.55251 2.76604 4.12549 2.49318 374 - -

2.68618 4.42225 2.77519 2.73123 3.46354 2.40513 3.72494 3.29354 2.67741 2.69355 4.24690 2.90347 2.73739 3.18146 2.89801 2.37887 2.77519 2.98518 4.58477 3.61503

0.03157 3.86699 4.58934 0.61958 0.77255 0.48576 0.95510

375 1.02168 4.18931 3.45704 3.24808 4.04693 3.01400 4.27077 3.17162 3.26419 3.05349 4.08591 3.39907 3.75270 3.60586 3.51510 2.53965 2.81663 2.82697 5.47463 4.27427 375 - -

2.68618 4.42225 2.77519 2.73123 3.46354 2.40513 3.72494 3.29354 2.67741 2.69355 4.24690 2.90347 2.73739 3.18146 2.89801 2.37887 2.77519 2.98518 4.58477 3.61503

0.03157 3.86699 4.58934 0.61958 0.77255 0.48576 0.95510

376 2.71888 4.71528 2.70526 2.59018 4.07794 3.18029 3.91656 3.78713 2.81979 3.43461 4.38989 1.16778 3.83188 3.18930 3.17427 2.78628 3.10056 3.40989 5.38448 4.02666 376 - -

2.68618 4.42225 2.77519 2.73123 3.46354 2.40513 3.72494 3.29354 2.67741 2.69355 4.24690 2.90347 2.73739 3.18146 2.89801 2.37887 2.77519 2.98518 4.58477 3.61503

0.03157 3.86699 4.58934 0.61958 0.77255 0.48576 0.95510

377 2.80376 4.34788 4.11775 3.78070 3.54607 3.71822 4.58336 2.05053 3.64840 2.23405 3.51204 3.97141 4.27903 4.00859 3.86143 3.22141 3.16544 1.03182 5.32959 4.06087 377 - -

2.68618 4.42225 2.77519 2.73123 3.46354 2.40513 3.72494 3.29354 2.67741 2.69355 4.24690 2.90347 2.73739 3.18146 2.89801 2.37887 2.77519 2.98518 4.58477 3.61503

0.03157 3.86699 4.58934 0.61958 0.77255 0.48576 0.95510

378 2.40976 4.25482 3.42322 3.17496 3.96403 3.09429 4.19270 3.09049 3.11783 2.95418 4.00504 3.38522 3.79760 3.51292 3.37678 2.61097 1.18122 2.78606 5.40231 4.18065 378 - -

2.68618 4.42225 2.77519 2.73123 3.46354 2.40513 3.72494 3.29354 2.67741 2.69355 4.24690 2.90347 2.73739 3.18146 2.89801 2.37887 2.77519 2.98518 4.58477 3.61503

0.03157 3.86699 4.58934 0.61958 0.77255 0.48576 0.95510

379 2.94758 5.05751 2.48529 1.03318 4.40304 3.27053 3.85685 3.85586 2.73669 3.47385 4.44812 2.92979 3.87486 3.07262 3.14497 2.93053 3.25177 3.54026 5.57565 4.29788 379 - -

2.68618 4.42225 2.77519 2.73123 3.46354 2.40513 3.72494 3.29354 2.67741 2.69355 4.24690 2.90347 2.73739 3.18146 2.89801 2.37887 2.77519 2.98518 4.58477 3.61503
0.03157 3.86699 4.58934 0.61958 0.77255 0.48576 0.95510
380 2.96711 4.93068 3.14273 2.76522 4.31586 3.45716 3.75616 3.74479 1.04579 3.31106 4.27280 3.18993 3.95788 2.94794 2.43058 3.00945 3.22078 3.45288 5.37276 4.18474 380 - -
2.68618 4.42225 2.77519 2.73123 3.46354 2.40513 3.72494 3.29354 2.67741 2.69355 4.24690 2.90347 2.73739 3.18146 2.89801 2.37887 2.77519 2.98518 4.58477 3.61503
0.03157 3.86699 4.58934 0.61958 0.77255 0.48576 0.95510
381 2.71888 4.71528 2.70526 2.59018 4.07794 3.18029 3.91656 3.78713 2.81979 3.43461 4.38989 1.16778 3.83188 3.18930 3.17427 2.78628 3.10056 3.40989 5.38448 4.02666 381 - -
2.68618 4.42225 2.77519 2.73123 3.46354 2.40513 3.72494 3.29354 2.67741 2.69355 4.24690 2.90347 2.73739 3.18146 2.89801 2.37887 2.77519 2.98518 4.58477 3.61503
0.03157 3.86699 4.58934 0.61958 0.77255 0.48576 0.95510
382 2.40976 4.25482 3.42322 3.17496 3.96403 3.09429 4.19270 3.09049 3.11783 2.95418 4.00504 3.38522 3.79760 3.51292 3.37678 2.61097 1.18122 2.78606 5.40231 4.18065 382 - -
2.68618 4.42225 2.77519 2.73123 3.46354 2.40513 3.72494 3.29354 2.67741 2.69355 4.24690 2.90347 2.73739 3.18146 2.89801 2.37887 2.77519 2.98518 4.58477 3.61503
0.03157 3.86699 4.58934 0.61958 0.77255 0.48576 0.95510
383 3.02684 4.44709 4.32791 3.94350 3.37049 4.06063 4.67263 1.08471 3.77153 1.95764 3.30670 4.18960 4.49236 4.12038 3.96872 3.55539 3.32610 1.84575 5.26019 3.99802 383 - -
2.68618 4.42225 2.77519 2.73123 3.46354 2.40513 3.72494 3.29354 2.67741 2.69355 4.24690 2.90347 2.73739 3.18146 2.89801 2.37887 2.77519 2.98518 4.58477 3.61503
0.03157 3.86699 4.58934 0.61958 0.77255 0.48576 0.95510
384 2.40976 4.25482 3.42322 3.17496 3.96403 3.09429 4.19270 3.09049 3.11783 2.95418 4.00504 3.38522 3.79760 3.51292 3.37678 2.61097 1.18122 2.78606 5.40231 4.18065 384 - -
2.68618 4.42225 2.77519 2.73123 3.46354 2.40513 3.72494 3.29354 2.67741 2.69355 4.24690 2.90347 2.73739 3.18146 2.89801 2.37887 2.77519 2.98518 4.58477 3.61503
0.03157 3.86699 4.58934 0.61958 0.77255 0.48576 0.95510
385 3.23918 4.73039 3.83015 3.55850 2.29358 3.82376 3.61597 3.24916 3.41618 2.70450 3.92476 3.71671 4.29772 3.72354 3.60101 3.37642 3.52583 3.11945 3.93713 0.97494 385 - -
2.68618 4.42225 2.77519 2.73123 3.46354 2.40513 3.72494 3.29354 2.67741 2.69355 4.24690 2.90347 2.73739 3.18146 2.89801 2.37887 2.77519 2.98518 4.58477 3.61503
0.03157 3.86699 4.58934 0.61958 0.77255 0.48576 0.95510
386 2.71888 4.71528 2.70526 2.59018 4.07794 3.18029 3.91656 3.78713 2.81979 3.43461 4.38989 1.16778 3.83188 3.18930 3.17427 2.78628 3.10056 3.40989 5.38448 4.02666 386 - -
2.68618 4.42225 2.77519 2.73123 3.46354 2.40513 3.72494 3.29354 2.67741 2.69355 4.24690 2.90347 2.73739 3.18146 2.89801 2.37887 2.77519 2.98518 4.58477 3.61503
0.03157 3.86699 4.58934 0.61958 0.77255 0.48576 0.95510
387 1.02168 4.18931 3.45704 3.24808 4.04693 3.01400 4.27077 3.17162 3.26419 3.05349 4.08591 3.39907 3.75270 3.60586 3.51510 2.53965 2.81663 2.82697 5.47463 4.27427 387 - -
2.68618 4.42225 2.77519 2.73123 3.46354 2.40513 3.72494 3.29354 2.67741 2.69355 4.24690 2.90347 2.73739 3.18146 2.89801 2.37887 2.77519 2.98518 4.58477 3.61503
0.03157 3.86699 4.58934 0.61958 0.77255 0.48576 0.95510
388 3.14912 4.61302 4.18070 3.82053 3.13362 3.97519 4.47798 2.33736 3.58833 0.88196 3.15827 4.09653 4.41149 3.95797 3.76529 3.54541 3.44362 2.37328 5.01092 3.73297 388 - -
2.68618 4.42225 2.77519 2.73123 3.46354 2.40513 3.72494 3.29354 2.67741 2.69355 4.24690 2.90347 2.73739 3.18146 2.89801 2.37887 2.77519 2.98518 4.58477 3.61503
0.03157 3.86699 4.58934 0.61958 0.77255 0.48576 0.95510
389 2.96489 4.51452 4.04353 3.63658 3.20533 3.82998 4.37299 2.38038 3.40496 1.80083 1.38846 3.91475 4.29056 3.78719 3.61092 3.31343 3.27009 2.38586 5.04542 3.80671 389 - -
2.68618 4.42225 2.77519 2.73123 3.46354 2.40513 3.72494 3.29354 2.67741 2.69355 4.24690 2.90347 2.73739 3.18146 2.89801 2.37887 2.77519 2.98518 4.58477 3.61503
0.03157 3.86699 4.58934 0.61958 0.77255 0.48576 0.95510
390 1.02168 4.18931 3.45704 3.24808 4.04693 3.01400 4.27077 3.17162 3.26419 3.05349 4.08591 3.39907 3.75270 3.60586 3.51510 2.53965 2.81663 2.82697 5.47463 4.27427 390 - -
2.68618 4.42225 2.77519 2.73123 3.46354 2.40513 3.72494 3.29354 2.67741 2.69355 4.24690 2.90347 2.73739 3.18146 2.89801 2.37887 2.77519 2.98518 4.58477 3.61503
0.03157 3.86699 4.58934 0.61958 0.77255 0.48576 0.95510

391 2.65811 4.43408 3.33076 3.22947 4.37188 0.73761 4.35008 3.97016 3.41000 3.62498 4.60067 3.49506 3.82505 3.73459 3.64665 2.82986 3.13892 3.49954 5.46848 4.47359 391 - -
2.68618 4.42225 2.77519 2.73123 3.46354 2.40513 3.72494 3.29354 2.67741 2.69355 4.24690 2.90347 2.73739 3.18146 2.89801 2.37887 2.77519 2.98518 4.58477 3.61503
0.03157 3.86699 4.58934 0.61958 0.77255 0.48576 0.95510
392 3.25138 4.65475 4.23121 3.94466 1.02072 3.94245 3.87481 2.77574 3.85183 2.14705 3.50670 4.01301 4.40115 4.02131 3.97290 3.52160 3.55251 2.76604 4.12549 2.49318 392 - -
2.68618 4.42225 2.77519 2.73123 3.46354 2.40513 3.72494 3.29354 2.67741 2.69355 4.24690 2.90347 2.73739 3.18146 2.89801 2.37887 2.77519 2.98518 4.58477 3.61503
0.03157 3.86699 4.58934 0.61958 0.77255 0.48576 0.95510
393 2.58006 0.95161 4.09776 3.83614 3.82875 3.20419 4.48443 2.97175 3.66306 2.88747 4.00858 3.82459 3.91071 4.00937 3.78840 2.85170 3.04637 2.71670 5.24602 4.11647 393 - -
2.68618 4.42225 2.77519 2.73123 3.46354 2.40513 3.72494 3.29354 2.67741 2.69355 4.24690 2.90347 2.73739 3.18146 2.89801 2.37887 2.77519 2.98518 4.58477 3.61503
0.03157 3.86699 4.58934 0.61958 0.77255 0.48576 0.95510
394 2.99928 4.83880 3.48937 3.00157 4.19200 3.48833 3.82030 3.73332 2.23516 3.25025 4.25056 3.36173 3.99118 3.04089 0.98678 3.08371 3.26560 3.45751 5.27725 4.12054 394 - -
2.68618 4.42225 2.77519 2.73123 3.46354 2.40513 3.72494 3.29354 2.67741 2.69355 4.24690 2.90347 2.73739 3.18146 2.89801 2.37887 2.77519 2.98518 4.58477 3.61503
0.03157 3.86699 4.58934 0.61958 0.77255 0.48576 0.95510
395 2.71888 4.71528 2.70526 2.59018 4.07794 3.18029 3.91656 3.78713 2.81979 3.43461 4.38989 1.16778 3.83188 3.18930 3.17427 2.78628 3.10056 3.40989 5.38448 4.02666 395 - -
2.68618 4.42225 2.77519 2.73123 3.46354 2.40513 3.72494 3.29354 2.67741 2.69355 4.24690 2.90347 2.73739 3.18146 2.89801 2.37887 2.77519 2.98518 4.58477 3.61503
0.03157 3.86699 4.58934 0.61958 0.77255 0.48576 0.95510
396 2.65811 4.43408 3.33076 3.22947 4.37188 0.73761 4.35008 3.97016 3.41000 3.62498 4.60067 3.49506 3.82505 3.73459 3.64665 2.82986 3.13892 3.49954 5.46848 4.47359 396 - -
2.68618 4.42225 2.77519 2.73123 3.46354 2.40513 3.72494 3.29354 2.67741 2.69355 4.24690 2.90347 2.73739 3.18146 2.89801 2.37887 2.77519 2.98518 4.58477 3.61503
0.03157 3.86699 4.58934 0.61958 0.77255 0.48576 0.95510
397 2.98886 4.80727 3.12036 2.87975 3.28089 3.44287 1.15410 3.69093 2.72295 3.18558 4.22810 3.28930 3.99510 3.24591 2.98899 3.05959 3.29203 3.42616 4.74602 3.23190 397 - -
2.68618 4.42225 2.77519 2.73123 3.46354 2.40513 3.72494 3.29354 2.67741 2.69355 4.24690 2.90347 2.73739 3.18146 2.89801 2.37887 2.77519 2.98518 4.58477 3.61503
0.03157 3.86699 4.58934 0.61958 0.77255 0.48576 0.95510
398 2.65811 4.43408 3.33076 3.22947 4.37188 0.73761 4.35008 3.97016 3.41000 3.62498 4.60067 3.49506 3.82505 3.73459 3.64665 2.82986 3.13892 3.49954 5.46848 4.47359 398 - -
2.68618 4.42225 2.77519 2.73123 3.46354 2.40513 3.72494 3.29354 2.67741 2.69355 4.24690 2.90347 2.73739 3.18146 2.89801 2.37887 2.77519 2.98518 4.58477 3.61503
0.03157 3.86699 4.58934 0.61958 0.77255 0.48576 0.95510
399 3.14912 4.61302 4.18070 3.82053 3.13362 3.97519 4.47798 2.33736 3.58833 0.88196 3.15827 4.09653 4.41149 3.95797 3.76529 3.54541 3.44362 2.37328 5.01092 3.73297 399 - -
2.68618 4.42225 2.77519 2.73123 3.46354 2.40513 3.72494 3.29354 2.67741 2.69355 4.24690 2.90347 2.73739 3.18146 2.89801 2.37887 2.77519 2.98518 4.58477 3.61503
0.03157 3.86699 4.58934 0.61958 0.77255 0.48576 0.95510
400 2.96711 4.93068 3.14273 2.76522 4.31586 3.45716 3.75616 3.74479 1.04579 3.31106 4.27280 3.18993 3.95788 2.94794 2.43058 3.00945 3.22078 3.45288 5.37276 4.18474 400 - -
2.68618 4.42225 2.77519 2.73123 3.46354 2.40513 3.72494 3.29354 2.67741 2.69355 4.24690 2.90347 2.73739 3.18146 2.89801 2.37887 2.77519 2.98518 4.58477 3.61503
0.03157 3.86699 4.58934 0.61958 0.77255 0.48576 0.95510
401 1.02168 4.18931 3.45704 3.24808 4.04693 3.01400 4.27077 3.17162 3.26419 3.05349 4.08591 3.39907 3.75270 3.60586 3.51510 2.53965 2.81663 2.82697 5.47463 4.27427 401 - -
2.68618 4.42225 2.77519 2.73123 3.46354 2.40513 3.72494 3.29354 2.67741 2.69355 4.24690 2.90347 2.73739 3.18146 2.89801 2.37887 2.77519 2.98518 4.58477 3.61503
0.03157 3.86699 4.58934 0.61958 0.77255 0.48576 0.95510
402 3.14912 4.61302 4.18070 3.82053 3.13362 3.97519 4.47798 2.33736 3.58833 0.88196 3.15827 4.09653 4.41149 3.95797 3.76529 3.54541 3.44362 2.37328 5.01092 3.73297 402 - -
2.68618 4.42225 2.77519 2.73123 3.46354 2.40513 3.72494 3.29354 2.67741 2.69355 4.24690 2.90347 2.73739 3.18146 2.89801 2.37887 2.77519 2.98518 4.58477 3.61503

0.03157 3.86699 4.58934 0.61958 0.77255 0.48576 0.95510

403 2.96711 4.93068 3.14273 2.76522 4.31586 3.45716 3.75616 3.74479 1.04579 3.31106 4.27280 3.18993 3.95788 2.94794 2.43058 3.00945 3.22078 3.45288 5.37276 4.18474 403 - -

2.68618 4.42225 2.77519 2.73123 3.46354 2.40513 3.72494 3.29354 2.67741 2.69355 4.24690 2.90347 2.73739 3.18146 2.89801 2.37887 2.77519 2.98518 4.58477 3.61503

0.03157 3.86699 4.58934 0.61958 0.77255 0.48576 0.95510

404 3.14912 4.61302 4.18070 3.82053 3.13362 3.97519 4.47798 2.33736 3.58833 0.88196 3.15827 4.09653 4.41149 3.95797 3.76529 3.54541 3.44362 2.37328 5.01092 3.73297 404 - -

2.68618 4.42225 2.77519 2.73123 3.46354 2.40513 3.72494 3.29354 2.67741 2.69355 4.24690 2.90347 2.73739 3.18146 2.89801 2.37887 2.77519 2.98518 4.58477 3.61503

0.03157 3.86699 4.58934 0.61958 0.77255 0.48576 0.95510

405 3.25138 4.65475 4.23121 3.94466 1.02072 3.94245 3.87481 2.77574 3.85183 2.14705 3.50670 4.01301 4.40115 4.02131 3.97290 3.52160 3.55251 2.76604 4.12549 2.49318 405 - -

2.68618 4.42225 2.77519 2.73123 3.46354 2.40513 3.72494 3.29354 2.67741 2.69355 4.24690 2.90347 2.73739 3.18146 2.89801 2.37887 2.77519 2.98518 4.58477 3.61503

0.03157 3.86699 4.58934 0.61958 0.77255 0.48576 0.95510

406 2.40976 4.25482 3.42322 3.17496 3.96403 3.09429 4.19270 3.09049 3.11783 2.95418 4.00504 3.38522 3.79760 3.51292 3.37678 2.61097 1.18122 2.78606 5.40231 4.18065 406 - -

2.68618 4.42225 2.77519 2.73123 3.46354 2.40513 3.72494 3.29354 2.67741 2.69355 4.24690 2.90347 2.73739 3.18146 2.89801 2.37887 2.77519 2.98518 4.58477 3.61503

0.03157 3.86699 4.58934 0.61958 0.77255 0.48576 0.95510

407 2.98371 5.11116 0.92594 2.33150 4.48744 3.20651 3.92621 4.07436 3.00929 3.68366 4.66164 2.88760 3.85892 3.16534 3.52477 2.94668 3.32216 3.71413 5.65711 4.36124 407 - -

2.68618 4.42225 2.77519 2.73123 3.46354 2.40513 3.72494 3.29354 2.67741 2.69355 4.24690 2.90347 2.73739 3.18146 2.89801 2.37887 2.77519 2.98518 4.58477 3.61503

0.03157 3.86699 4.58934 0.61958 0.77255 0.48576 0.95510

408 2.96489 4.51452 4.04353 3.63658 3.20533 3.82998 4.37299 2.38038 3.40496 1.80083 1.38846 3.91475 4.29056 3.78719 3.61092 3.31343 3.27009 2.38586 5.04542 3.80671 408 - -

2.68618 4.42225 2.77519 2.73123 3.46354 2.40513 3.72494 3.29354 2.67741 2.69355 4.24690 2.90347 2.73739 3.18146 2.89801 2.37887 2.77519 2.98518 4.58477 3.61503

0.03157 3.86699 4.58934 0.61958 0.77255 0.48576 0.95510

409 3.14912 4.61302 4.18070 3.82053 3.13362 3.97519 4.47798 2.33736 3.58833 0.88196 3.15827 4.09653 4.41149 3.95797 3.76529 3.54541 3.44362 2.37328 5.01092 3.73297 409 - -

2.68618 4.42225 2.77519 2.73123 3.46354 2.40513 3.72494 3.29354 2.67741 2.69355 4.24690 2.90347 2.73739 3.18146 2.89801 2.37887 2.77519 2.98518 4.58477 3.61503

0.03157 3.86699 4.58934 0.61958 0.77255 0.48576 0.95510

410 2.87640 4.86196 2.89930 2.65395 4.02980 3.36241 3.81221 3.65971 2.47512 3.16261 4.19062 3.11364 3.91579 1.24381 2.75940 2.92438 3.17337 3.38973 5.30278 3.99617 410 - -

2.68618 4.42225 2.77519 2.73123 3.46354 2.40513 3.72494 3.29354 2.67741 2.69355 4.24690 2.90347 2.73739 3.18146 2.89801 2.37887 2.77519 2.98518 4.58477 3.61503

0.03157 3.86699 4.58934 0.61958 0.77255 0.48576 0.95510

411 2.99928 4.83880 3.48937 3.00157 4.19200 3.48833 3.82030 3.73332 2.23516 3.25025 4.25056 3.36173 3.99118 3.04089 0.98678 3.08371 3.26560 3.45751 5.27725 4.12054 411 - -

2.68618 4.42225 2.77519 2.73123 3.46354 2.40513 3.72494 3.29354 2.67741 2.69355 4.24690 2.90347 2.73739 3.18146 2.89801 2.37887 2.77519 2.98518 4.58477 3.61503

0.03157 3.86699 4.58934 0.61958 0.77255 0.48576 0.95510

412 2.65811 4.43408 3.33076 3.22947 4.37188 0.73761 4.35008 3.97016 3.41000 3.62498 4.60067 3.49506 3.82505 3.73459 3.64665 2.82986 3.13892 3.49954 5.46848 4.47359 412 - -

2.68618 4.42225 2.77519 2.73123 3.46354 2.40513 3.72494 3.29354 2.67741 2.69355 4.24690 2.90347 2.73739 3.18146 2.89801 2.37887 2.77519 2.98518 4.58477 3.61503

0.03157 3.86699 4.58934 0.61958 0.77255 0.48576 0.95510

413 2.80376 4.34788 4.11775 3.78070 3.54607 3.71822 4.58336 2.05053 3.64840 2.23405 3.51204 3.97141 4.27903 4.00859 3.86143 3.22141 3.16544 1.03182 5.32959 4.06087 413 - -

2.68618 4.42225 2.77519 2.73123 3.46354 2.40513 3.72494 3.29354 2.67741 2.69355 4.24690 2.90347 2.73739 3.18146 2.89801 2.37887 2.77519 2.98518 4.58477 3.61503

0.03157 3.86699 4.58934 0.61958 0.77255 0.48576 0.95510

414 2.94758 5.05751 2.48529 1.03318 4.40304 3.27053 3.85685 3.85586 2.73669 3.47385 4.44812 2.92979 3.87486 3.07262 3.14497 2.93053 3.25177 3.54026 5.57565 4.29788 414 - -

2.68618 4.42225 2.77519 2.73123 3.46354 2.40513 3.72494 3.29354 2.67741 2.69355 4.24690 2.90347 2.73739 3.18146 2.89801 2.37887 2.77519 2.98518 4.58477 3.61503

0.03157 3.86699 4.58934 0.61958 0.77255 0.48576 0.95510

415 3.14912 4.61302 4.18070 3.82053 3.13362 3.97519 4.47798 2.33736 3.58833 0.88196 3.15827 4.09653 4.41149 3.95797 3.76529 3.54541 3.44362 2.37328 5.01092 3.73297 415 - -

2.68618 4.42225 2.77519 2.73123 3.46354 2.40513 3.72494 3.29354 2.67741 2.69355 4.24690 2.90347 2.73739 3.18146 2.89801 2.37887 2.77519 2.98518 4.58477 3.61503

0.03157 3.86699 4.58934 0.61958 0.77255 0.48576 0.95510

416 2.40976 4.25482 3.42322 3.17496 3.96403 3.09429 4.19270 3.09049 3.11783 2.95418 4.00504 3.38522 3.79760 3.51292 3.37678 2.61097 1.18122 2.78606 5.40231 4.18065 416 - -

2.68618 4.42225 2.77519 2.73123 3.46354 2.40513 3.72494 3.29354 2.67741 2.69355 4.24690 2.90347 2.73739 3.18146 2.89801 2.37887 2.77519 2.98518 4.58477 3.61503

0.03157 3.86699 4.58934 0.61958 0.77255 0.48576 0.95510

417 2.98371 5.11116 0.92594 2.33150 4.48744 3.20651 3.92621 4.07436 3.00929 3.68366 4.66164 2.88760 3.85892 3.16534 3.52477 2.94668 3.32216 3.71413 5.65711 4.36124 417 - -

2.68618 4.42225 2.77519 2.73123 3.46354 2.40513 3.72494 3.29354 2.67741 2.69355 4.24690 2.90347 2.73739 3.18146 2.89801 2.37887 2.77519 2.98518 4.58477 3.61503

0.03157 3.86699 4.58934 0.61958 0.77255 0.48576 0.95510

418 3.25138 4.65475 4.23121 3.94466 1.02072 3.94245 3.87481 2.77574 3.85183 2.14705 3.50670 4.01301 4.40115 4.02131 3.97290 3.52160 3.55251 2.76604 4.12549 2.49318 418 - -

2.68618 4.42225 2.77519 2.73123 3.46354 2.40513 3.72494 3.29354 2.67741 2.69355 4.24690 2.90347 2.73739 3.18146 2.89801 2.37887 2.77519 2.98518 4.58477 3.61503

0.03157 3.86699 4.58934 0.61958 0.77255 0.48576 0.95510

419 2.31230 4.23263 3.20165 3.01716 4.07082 2.96261 4.12717 3.59269 3.09056 3.31334 4.23988 3.24282 3.70096 3.44030 3.37676 1.09934 2.79202 3.12899 5.44375 4.15432 419 - -

2.68618 4.42225 2.77519 2.73123 3.46354 2.40513 3.72494 3.29354 2.67741 2.69355 4.24690 2.90347 2.73739 3.18146 2.89801 2.37887 2.77519 2.98518 4.58477 3.61503

0.03157 3.86699 4.58934 0.61958 0.77255 0.48576 0.95510

420 3.14912 4.61302 4.18070 3.82053 3.13362 3.97519 4.47798 2.33736 3.58833 0.88196 3.15827 4.09653 4.41149 3.95797 3.76529 3.54541 3.44362 2.37328 5.01092 3.73297 420 - -

2.68618 4.42225 2.77519 2.73123 3.46354 2.40513 3.72494 3.29354 2.67741 2.69355 4.24690 2.90347 2.73739 3.18146 2.89801 2.37887 2.77519 2.98518 4.58477 3.61503

0.03157 3.86699 4.58934 0.61958 0.77255 0.48576 0.95510

421 2.40976 4.25482 3.42322 3.17496 3.96403 3.09429 4.19270 3.09049 3.11783 2.95418 4.00504 3.38522 3.79760 3.51292 3.37678 2.61097 1.18122 2.78606 5.40231 4.18065 421 - -

2.68618 4.42225 2.77519 2.73123 3.46354 2.40513 3.72494 3.29354 2.67741 2.69355 4.24690 2.90347 2.73739 3.18146 2.89801 2.37887 2.77519 2.98518 4.58477 3.61503

0.03157 3.86699 4.58934 0.61958 0.77255 0.48576 0.95510

422 2.31230 4.23263 3.20165 3.01716 4.07082 2.96261 4.12717 3.59269 3.09056 3.31334 4.23988 3.24282 3.70096 3.44030 3.37676 1.09934 2.79202 3.12899 5.44375 4.15432 422 - -

2.68618 4.42225 2.77519 2.73123 3.46354 2.40513 3.72494 3.29354 2.67741 2.69355 4.24690 2.90347 2.73739 3.18146 2.89801 2.37887 2.77519 2.98518 4.58477 3.61503

0.03157 3.86699 4.58934 0.61958 0.77255 0.48576 0.95510

423 1.02168 4.18931 3.45704 3.24808 4.04693 3.01400 4.27077 3.17162 3.26419 3.05349 4.08591 3.39907 3.75270 3.60586 3.51510 2.53965 2.81663 2.82697 5.47463 4.27427 423 - -

2.68618 4.42225 2.77519 2.73123 3.46354 2.40513 3.72494 3.29354 2.67741 2.69355 4.24690 2.90347 2.73739 3.18146 2.89801 2.37887 2.77519 2.98518 4.58477 3.61503

0.03157 3.86699 4.58934 0.61958 0.77255 0.48576 0.95510

424 2.80376 4.34788 4.11775 3.78070 3.54607 3.71822 4.58336 2.05053 3.64840 2.23405 3.51204 3.97141 4.27903 4.00859 3.86143 3.22141 3.16544 1.03182 5.32959 4.06087 424 - -

2.68618 4.42225 2.77519 2.73123 3.46354 2.40513 3.72494 3.29354 2.67741 2.69355 4.24690 2.90347 2.73739 3.18146 2.89801 2.37887 2.77519 2.98518 4.58477 3.61503

0.03157 3.86699 4.58934 0.61958 0.77255 0.48576 0.95510

425 2.98371 5.11116 0.92594 2.33150 4.48744 3.20651 3.92621 4.07436 3.00929 3.68366 4.66164 2.88760 3.85892 3.16534 3.52477 2.94668 3.32216 3.71413 5.65711 4.36124 425 - -

2.68618 4.42225 2.77519 2.73123 3.46354 2.40513 3.72494 3.29354 2.67741 2.69355 4.24690 2.90347 2.73739 3.18146 2.89801 2.37887 2.77519 2.98518 4.58477 3.61503

0.03157 3.86699 4.58934 0.61958 0.77255 0.48576 0.95510

426 1.02168 4.18931 3.45704 3.24808 4.04693 3.01400 4.27077 3.17162 3.26419 3.05349 4.08591 3.39907 3.75270 3.60586 3.51510 2.53965 2.81663 2.82697 5.47463 4.27427 426 - -
2.68618 4.42225 2.77519 2.73123 3.46354 2.40513 3.72494 3.29354 2.67741 2.69355 4.24690 2.90347 2.73739 3.18146 2.89801 2.37887 2.77519 2.98518 4.58477 3.61503
0.03157 3.86699 4.58934 0.61958 0.77255 0.48576 0.95510
427 2.58006 0.95161 4.09776 3.83614 3.82875 3.20419 4.48443 2.97175 3.66306 2.88747 4.00858 3.82459 3.91071 4.00937 3.78840 2.85170 3.04637 2.71670 5.24602 4.11647 427 - -
2.68618 4.42225 2.77519 2.73123 3.46354 2.40513 3.72494 3.29354 2.67741 2.69355 4.24690 2.90347 2.73739 3.18146 2.89801 2.37887 2.77519 2.98518 4.58477 3.61503
0.03157 3.86699 4.58934 0.61958 0.77255 0.48576 0.95510
428 2.65811 4.43408 3.33076 3.22947 4.37188 0.73761 4.35008 3.97016 3.41000 3.62498 4.60067 3.49506 3.82505 3.73459 3.64665 2.82986 3.13892 3.49954 5.46848 4.47359 428 - -
2.68618 4.42225 2.77519 2.73123 3.46354 2.40513 3.72494 3.29354 2.67741 2.69355 4.24690 2.90347 2.73739 3.18146 2.89801 2.37887 2.77519 2.98518 4.58477 3.61503
0.03157 3.86699 4.58934 0.61958 0.77255 0.48576 0.95510
429 3.14912 4.61302 4.18070 3.82053 3.13362 3.97519 4.47798 2.33736 3.58833 0.88196 3.15827 4.09653 4.41149 3.95797 3.76529 3.54541 3.44362 2.37328 5.01092 3.73297 429 - -
2.68618 4.42225 2.77519 2.73123 3.46354 2.40513 3.72494 3.29354 2.67741 2.69355 4.24690 2.90347 2.73739 3.18146 2.89801 2.37887 2.77519 2.98518 4.58477 3.61503
0.03157 3.86699 4.58934 0.61958 0.77255 0.48576 0.95510
430 2.80376 4.34788 4.11775 3.78070 3.54607 3.71822 4.58336 2.05053 3.64840 2.23405 3.51204 3.97141 4.27903 4.00859 3.86143 3.22141 3.16544 1.03182 5.32959 4.06087 430 - -
2.68618 4.42225 2.77519 2.73123 3.46354 2.40513 3.72494 3.29354 2.67741 2.69355 4.24690 2.90347 2.73739 3.18146 2.89801 2.37887 2.77519 2.98518 4.58477 3.61503
0.03157 3.86699 4.58934 0.61958 0.77255 0.48576 0.95510
431 2.31230 4.23263 3.20165 3.01716 4.07082 2.96261 4.12717 3.59269 3.09056 3.31334 4.23988 3.24282 3.70096 3.44030 3.37676 1.09934 2.79202 3.12899 5.44375 4.15432 431 - -
2.68618 4.42225 2.77519 2.73123 3.46354 2.40513 3.72494 3.29354 2.67741 2.69355 4.24690 2.90347 2.73739 3.18146 2.89801 2.37887 2.77519 2.98518 4.58477 3.61503
0.03157 3.86699 4.58934 0.61958 0.77255 0.48576 0.95510
432 2.94758 5.05751 2.48529 1.03318 4.40304 3.27053 3.85685 3.85586 2.73669 3.47385 4.44812 2.92979 3.87486 3.07262 3.14497 2.93053 3.25177 3.54026 5.57565 4.29788 432 - -
2.68618 4.42225 2.77519 2.73123 3.46354 2.40513 3.72494 3.29354 2.67741 2.69355 4.24690 2.90347 2.73739 3.18146 2.89801 2.37887 2.77519 2.98518 4.58477 3.61503
0.03157 3.86699 4.58934 0.61958 0.77255 0.48576 0.95510
433 2.96711 4.93068 3.14273 2.76522 4.31586 3.45716 3.75616 3.74479 1.04579 3.31106 4.27280 3.18993 3.95788 2.94794 2.43058 3.00945 3.22078 3.45288 5.37276 4.18474 433 - -
2.68618 4.42225 2.77519 2.73123 3.46354 2.40513 3.72494 3.29354 2.67741 2.69355 4.24690 2.90347 2.73739 3.18146 2.89801 2.37887 2.77519 2.98518 4.58477 3.61503
0.03157 3.86699 4.58934 0.61958 0.77255 0.48576 0.95510
434 2.96711 4.93068 3.14273 2.76522 4.31586 3.45716 3.75616 3.74479 1.04579 3.31106 4.27280 3.18993 3.95788 2.94794 2.43058 3.00945 3.22078 3.45288 5.37276 4.18474 434 - -
2.68618 4.42225 2.77519 2.73123 3.46354 2.40513 3.72494 3.29354 2.67741 2.69355 4.24690 2.90347 2.73739 3.18146 2.89801 2.37887 2.77519 2.98518 4.58477 3.61503
0.03157 3.86699 4.58934 0.61958 0.77255 0.48576 0.95510
435 2.80376 4.34788 4.11775 3.78070 3.54607 3.71822 4.58336 2.05053 3.64840 2.23405 3.51204 3.97141 4.27903 4.00859 3.86143 3.22141 3.16544 1.03182 5.32959 4.06087 435 - -
2.68618 4.42225 2.77519 2.73123 3.46354 2.40513 3.72494 3.29354 2.67741 2.69355 4.24690 2.90347 2.73739 3.18146 2.89801 2.37887 2.77519 2.98518 4.58477 3.61503
0.03157 3.86699 4.58934 0.61958 0.77255 0.48576 0.95510
436 2.31230 4.23263 3.20165 3.01716 4.07082 2.96261 4.12717 3.59269 3.09056 3.31334 4.23988 3.24282 3.70096 3.44030 3.37676 1.09934 2.79202 3.12899 5.44375 4.15432 436 - -
2.68618 4.42225 2.77519 2.73123 3.46354 2.40513 3.72494 3.29354 2.67741 2.69355 4.24690 2.90347 2.73739 3.18146 2.89801 2.37887 2.77519 2.98518 4.58477 3.61503
0.03157 3.86699 4.58934 0.61958 0.77255 0.48576 0.95510
437 2.94758 5.05751 2.48529 1.03318 4.40304 3.27053 3.85685 3.85586 2.73669 3.47385 4.44812 2.92979 3.87486 3.07262 3.14497 2.93053 3.25177 3.54026 5.57565 4.29788 437 - -
2.68618 4.42225 2.77519 2.73123 3.46354 2.40513 3.72494 3.29354 2.67741 2.69355 4.24690 2.90347 2.73739 3.18146 2.89801 2.37887 2.77519 2.98518 4.58477 3.61503

0.03157 3.86699 4.58934 0.61958 0.77255 0.48576 0.95510

438 2.87640 4.86196 2.89930 2.65395 4.02980 3.36241 3.81221 3.65971 2.47512 3.16261 4.19062 3.11364 3.91579 1.24381 2.75940 2.92438 3.17337 3.38973 5.30278 3.99617 438 - -

2.68618 4.42225 2.77519 2.73123 3.46354 2.40513 3.72494 3.29354 2.67741 2.69355 4.24690 2.90347 2.73739 3.18146 2.89801 2.37887 2.77519 2.98518 4.58477 3.61503

0.03157 3.86699 4.58934 0.61958 0.77255 0.48576 0.95510

439 3.02684 4.44709 4.32791 3.94350 3.37049 4.06063 4.67263 1.08471 3.77153 1.95764 3.30670 4.18960 4.49236 4.12038 3.96872 3.55539 3.32610 1.84575 5.26019 3.99802 439 - -

2.68618 4.42225 2.77519 2.73123 3.46354 2.40513 3.72494 3.29354 2.67741 2.69355 4.24690 2.90347 2.73739 3.18146 2.89801 2.37887 2.77519 2.98518 4.58477 3.61503

0.03157 3.86699 4.58934 0.61958 0.77255 0.48576 0.95510

440 2.98886 4.80727 3.12036 2.87975 3.28089 3.44287 1.15410 3.69093 2.72295 3.18558 4.22810 3.28930 3.99510 3.24591 2.98899 3.05959 3.29203 3.42616 4.74602 3.23190 440 - -

2.68618 4.42225 2.77519 2.73123 3.46354 2.40513 3.72494 3.29354 2.67741 2.69355 4.24690 2.90347 2.73739 3.18146 2.89801 2.37887 2.77519 2.98518 4.58477 3.61503

0.03157 3.86699 4.58934 0.61958 0.77255 0.48576 0.95510

441 2.65811 4.43408 3.33076 3.22947 4.37188 0.73761 4.35008 3.97016 3.41000 3.62498 4.60067 3.49506 3.82505 3.73459 3.64665 2.82986 3.13892 3.49954 5.46848 4.47359 441 - -

2.68618 4.42225 2.77519 2.73123 3.46354 2.40513 3.72494 3.29354 2.67741 2.69355 4.24690 2.90347 2.73739 3.18146 2.89801 2.37887 2.77519 2.98518 4.58477 3.61503

0.03157 3.86699 4.58934 0.61958 0.77255 0.48576 0.95510

442 3.25138 4.65475 4.23121 3.94466 1.02072 3.94245 3.87481 2.77574 3.85183 2.14705 3.50670 4.01301 4.40115 4.02131 3.97290 3.52160 3.55251 2.76604 4.12549 2.49318 442 - -

2.68618 4.42225 2.77519 2.73123 3.46354 2.40513 3.72494 3.29354 2.67741 2.69355 4.24690 2.90347 2.73739 3.18146 2.89801 2.37887 2.77519 2.98518 4.58477 3.61503

0.03157 3.86699 4.58934 0.61958 0.77255 0.48576 0.95510

443 2.58006 0.95161 4.09776 3.83614 3.82875 3.20419 4.48443 2.97175 3.66306 2.88747 4.00858 3.82459 3.91071 4.00937 3.78840 2.85170 3.04637 2.71670 5.24602 4.11647 443 - -

2.68618 4.42225 2.77519 2.73123 3.46354 2.40513 3.72494 3.29354 2.67741 2.69355 4.24690 2.90347 2.73739 3.18146 2.89801 2.37887 2.77519 2.98518 4.58477 3.61503

0.03157 3.86699 4.58934 0.61958 0.77255 0.48576 0.95510

444 3.02684 4.44709 4.32791 3.94350 3.37049 4.06063 4.67263 1.08471 3.77153 1.95764 3.30670 4.18960 4.49236 4.12038 3.96872 3.55539 3.32610 1.84575 5.26019 3.99802 444 - -

2.68618 4.42225 2.77519 2.73123 3.46354 2.40513 3.72494 3.29354 2.67741 2.69355 4.24690 2.90347 2.73739 3.18146 2.89801 2.37887 2.77519 2.98518 4.58477 3.61503

0.03157 3.86699 4.58934 0.61958 0.77255 0.48576 0.95510

445 2.96711 4.93068 3.14273 2.76522 4.31586 3.45716 3.75616 3.74479 1.04579 3.31106 4.27280 3.18993 3.95788 2.94794 2.43058 3.00945 3.22078 3.45288 5.37276 4.18474 445 - -

2.68618 4.42225 2.77519 2.73123 3.46354 2.40513 3.72494 3.29354 2.67741 2.69355 4.24690 2.90347 2.73739 3.18146 2.89801 2.37887 2.77519 2.98518 4.58477 3.61503

0.03157 3.86699 4.58934 0.61958 0.77255 0.48576 0.95510

446 3.25138 4.65475 4.23121 3.94466 1.02072 3.94245 3.87481 2.77574 3.85183 2.14705 3.50670 4.01301 4.40115 4.02131 3.97290 3.52160 3.55251 2.76604 4.12549 2.49318 446 - -

2.68618 4.42225 2.77519 2.73123 3.46354 2.40513 3.72494 3.29354 2.67741 2.69355 4.24690 2.90347 2.73739 3.18146 2.89801 2.37887 2.77519 2.98518 4.58477 3.61503

0.03157 3.86699 4.58934 0.61958 0.77255 0.48576 0.95510

447 2.65811 4.43408 3.33076 3.22947 4.37188 0.73761 4.35008 3.97016 3.41000 3.62498 4.60067 3.49506 3.82505 3.73459 3.64665 2.82986 3.13892 3.49954 5.46848 4.47359 447 - -

2.68618 4.42225 2.77519 2.73123 3.46354 2.40513 3.72494 3.29354 2.67741 2.69355 4.24690 2.90347 2.73739 3.18146 2.89801 2.37887 2.77519 2.98518 4.58477 3.61503

0.03157 3.86699 4.58934 0.61958 0.77255 0.48576 0.95510

448 2.40976 4.25482 3.42322 3.17496 3.96403 3.09429 4.19270 3.09049 3.11783 2.95418 4.00504 3.38522 3.79760 3.51292 3.37678 2.61097 1.18122 2.78606 5.40231 4.18065 448 - -

2.68618 4.42225 2.77519 2.73123 3.46354 2.40513 3.72494 3.29354 2.67741 2.69355 4.24690 2.90347 2.73739 3.18146 2.89801 2.37887 2.77519 2.98518 4.58477 3.61503

0.03157 3.86699 4.58934 0.61958 0.77255 0.48576 0.95510

449 1.02168 4.18931 3.45704 3.24808 4.04693 3.01400 4.27077 3.17162 3.26419 3.05349 4.08591 3.39907 3.75270 3.60586 3.51510 2.53965 2.81663 2.82697 5.47463 4.27427 449 - -

2.68618 4.42225 2.77519 2.73123 3.46354 2.40513 3.72494 3.29354 2.67741 2.69355 4.24690 2.90347 2.73739 3.18146 2.89801 2.37887 2.77519 2.98518 4.58477 3.61503

0.03157 3.86699 4.58934 0.61958 0.77255 0.48576 0.95510

450 3.25138 4.65475 4.23121 3.94466 1.02072 3.94245 3.87481 2.77574 3.85183 2.14705 3.50670 4.01301 4.40115 4.02131 3.97290 3.52160 3.55251 2.76604 4.12549 2.49318 450 - -

2.68618 4.42225 2.77519 2.73123 3.46354 2.40513 3.72494 3.29354 2.67741 2.69355 4.24690 2.90347 2.73739 3.18146 2.89801 2.37887 2.77519 2.98518 4.58477 3.61503

0.03157 3.86699 4.58934 0.61958 0.77255 0.48576 0.95510

451 2.71888 4.71528 2.70526 2.59018 4.07794 3.18029 3.91656 3.78713 2.81979 3.43461 4.38989 1.16778 3.83188 3.18930 3.17427 2.78628 3.10056 3.40989 5.38448 4.02666 451 - -

2.68618 4.42225 2.77519 2.73123 3.46354 2.40513 3.72494 3.29354 2.67741 2.69355 4.24690 2.90347 2.73739 3.18146 2.89801 2.37887 2.77519 2.98518 4.58477 3.61503

0.03157 3.86699 4.58934 0.61958 0.77255 0.48576 0.95510

452 2.74420 4.50247 3.35077 3.18900 4.18468 3.19029 4.26692 3.72867 3.24583 3.35611 4.40571 3.49627 0.83787 3.63478 3.49200 2.91151 3.18532 3.37467 5.37275 4.30526 452 - -

2.68618 4.42225 2.77519 2.73123 3.46354 2.40513 3.72494 3.29354 2.67741 2.69355 4.24690 2.90347 2.73739 3.18146 2.89801 2.37887 2.77519 2.98518 4.58477 3.61503

0.03157 3.86699 4.58934 0.61958 0.77255 0.48576 0.95510

453 2.58006 0.95161 4.09776 3.83614 3.82875 3.20419 4.48443 2.97175 3.66306 2.88747 4.00858 3.82459 3.91071 4.00937 3.78840 2.85170 3.04637 2.71670 5.24602 4.11647 453 - -

2.68618 4.42225 2.77519 2.73123 3.46354 2.40513 3.72494 3.29354 2.67741 2.69355 4.24690 2.90347 2.73739 3.18146 2.89801 2.37887 2.77519 2.98518 4.58477 3.61503

0.03157 3.86699 4.58934 0.61958 0.77255 0.48576 0.95510

454 3.02684 4.44709 4.32791 3.94350 3.37049 4.06063 4.67263 1.08471 3.77153 1.95764 3.30670 4.18960 4.49236 4.12038 3.96872 3.55539 3.32610 1.84575 5.26019 3.99802 454 - -

2.68618 4.42225 2.77519 2.73123 3.46354 2.40513 3.72494 3.29354 2.67741 2.69355 4.24690 2.90347 2.73739 3.18146 2.89801 2.37887 2.77519 2.98518 4.58477 3.61503

0.03157 3.86699 4.58934 0.61958 0.77255 0.48576 0.95510

455 2.87640 4.86196 2.89930 2.65395 4.02980 3.36241 3.81221 3.65971 2.47512 3.16261 4.19062 3.11364 3.91579 1.24381 2.75940 2.92438 3.17337 3.38973 5.30278 3.99617 455 - -

2.68618 4.42225 2.77519 2.73123 3.46354 2.40513 3.72494 3.29354 2.67741 2.69355 4.24690 2.90347 2.73739 3.18146 2.89801 2.37887 2.77519 2.98518 4.58477 3.61503

0.03157 3.86699 4.58934 0.61958 0.77255 0.48576 0.95510

456 2.40976 4.25482 3.42322 3.17496 3.96403 3.09429 4.19270 3.09049 3.11783 2.95418 4.00504 3.38522 3.79760 3.51292 3.37678 2.61097 1.18122 2.78606 5.40231 4.18065 456 - -

2.68618 4.42225 2.77519 2.73123 3.46354 2.40513 3.72494 3.29354 2.67741 2.69355 4.24690 2.90347 2.73739 3.18146 2.89801 2.37887 2.77519 2.98518 4.58477 3.61503

0.03157 3.86699 4.58934 0.61958 0.77255 0.48576 0.95510

457 1.02168 4.18931 3.45704 3.24808 4.04693 3.01400 4.27077 3.17162 3.26419 3.05349 4.08591 3.39907 3.75270 3.60586 3.51510 2.53965 2.81663 2.82697 5.47463 4.27427 457 - -

2.68618 4.42225 2.77519 2.73123 3.46354 2.40513 3.72494 3.29354 2.67741 2.69355 4.24690 2.90347 2.73739 3.18146 2.89801 2.37887 2.77519 2.98518 4.58477 3.61503

0.03157 3.86699 4.58934 0.61958 0.77255 0.48576 0.95510

458 3.14912 4.61302 4.18070 3.82053 3.13362 3.97519 4.47798 2.33736 3.58833 0.88196 3.15827 4.09653 4.41149 3.95797 3.76529 3.54541 3.44362 2.37328 5.01092 3.73297 458 - -

2.68618 4.42225 2.77519 2.73123 3.46354 2.40513 3.72494 3.29354 2.67741 2.69355 4.24690 2.90347 2.73739 3.18146 2.89801 2.37887 2.77519 2.98518 4.58477 3.61503

0.03157 3.86699 4.58934 0.61958 0.77255 0.48576 0.95510

459 3.14912 4.61302 4.18070 3.82053 3.13362 3.97519 4.47798 2.33736 3.58833 0.88196 3.15827 4.09653 4.41149 3.95797 3.76529 3.54541 3.44362 2.37328 5.01092 3.73297 459 - -

2.68618 4.42225 2.77519 2.73123 3.46354 2.40513 3.72494 3.29354 2.67741 2.69355 4.24690 2.90347 2.73739 3.18146 2.89801 2.37887 2.77519 2.98518 4.58477 3.61503

0.03157 3.86699 4.58934 0.61958 0.77255 0.48576 0.95510

460 2.98371 5.11116 0.92594 2.33150 4.48744 3.20651 3.92621 4.07436 3.00929 3.68366 4.66164 2.88760 3.85892 3.16534 3.52477 2.94668 3.32216 3.71413 5.65711 4.36124 460 - -

2.68618 4.42225 2.77519 2.73123 3.46354 2.40513 3.72494 3.29354 2.67741 2.69355 4.24690 2.90347 2.73739 3.18146 2.89801 2.37887 2.77519 2.98518 4.58477 3.61503

0.03157 3.86699 4.58934 0.61958 0.77255 0.48576 0.95510

461 2.96489 4.51452 4.04353 3.63658 3.20533 3.82998 4.37299 2.38038 3.40496 1.80083 1.38846 3.91475 4.29056 3.78719 3.61092 3.31343 3.27009 2.38586 5.04542 3.80671 461 - -
2.68618 4.42225 2.77519 2.73123 3.46354 2.40513 3.72494 3.29354 2.67741 2.69355 4.24690 2.90347 2.73739 3.18146 2.89801 2.37887 2.77519 2.98518 4.58477 3.61503
0.03157 3.86699 4.58934 0.61958 0.77255 0.48576 0.95510

462 2.58006 0.95161 4.09776 3.83614 3.82875 3.20419 4.48443 2.97175 3.66306 2.88747 4.00858 3.82459 3.91071 4.00937 3.78840 2.85170 3.04637 2.71670 5.24602 4.11647 462 - -
2.68618 4.42225 2.77519 2.73123 3.46354 2.40513 3.72494 3.29354 2.67741 2.69355 4.24690 2.90347 2.73739 3.18146 2.89801 2.37887 2.77519 2.98518 4.58477 3.61503
0.03157 3.86699 4.58934 0.61958 0.77255 0.48576 0.95510

463 2.40976 4.25482 3.42322 3.17496 3.96403 3.09429 4.19270 3.09049 3.11783 2.95418 4.00504 3.38522 3.79760 3.51292 3.37678 2.61097 1.18122 2.78606 5.40231 4.18065 463 - -
2.68618 4.42225 2.77519 2.73123 3.46354 2.40513 3.72494 3.29354 2.67741 2.69355 4.24690 2.90347 2.73739 3.18146 2.89801 2.37887 2.77519 2.98518 4.58477 3.61503
0.03157 3.86699 4.58934 0.61958 0.77255 0.48576 0.95510

464 2.99928 4.83880 3.48937 3.00157 4.19200 3.48833 3.82030 3.73332 2.23516 3.25025 4.25056 3.36173 3.99118 3.04089 0.98678 3.08371 3.26560 3.45751 5.27725 4.12054 464 - -
2.68618 4.42225 2.77519 2.73123 3.46354 2.40513 3.72494 3.29354 2.67741 2.69355 4.24690 2.90347 2.73739 3.18146 2.89801 2.37887 2.77519 2.98518 4.58477 3.61503
0.03157 3.86699 4.58934 0.61958 0.77255 0.48576 0.95510

465 2.58006 0.95161 4.09776 3.83614 3.82875 3.20419 4.48443 2.97175 3.66306 2.88747 4.00858 3.82459 3.91071 4.00937 3.78840 2.85170 3.04637 2.71670 5.24602 4.11647 465 - -
2.68618 4.42225 2.77519 2.73123 3.46354 2.40513 3.72494 3.29354 2.67741 2.69355 4.24690 2.90347 2.73739 3.18146 2.89801 2.37887 2.77519 2.98518 4.58477 3.61503
0.03157 3.86699 4.58934 0.61958 0.77255 0.48576 0.95510

466 2.94758 5.05751 2.48529 1.03318 4.40304 3.27053 3.85685 3.85586 2.73669 3.47385 4.44812 2.92979 3.87486 3.07262 3.14497 2.93053 3.25177 3.54026 5.57565 4.29788 466 - -
2.68618 4.42225 2.77519 2.73123 3.46354 2.40513 3.72494 3.29354 2.67741 2.69355 4.24690 2.90347 2.73739 3.18146 2.89801 2.37887 2.77519 2.98518 4.58477 3.61503
0.03157 3.86699 4.58934 0.61958 0.77255 0.48576 0.95510

467 2.99928 4.83880 3.48937 3.00157 4.19200 3.48833 3.82030 3.73332 2.23516 3.25025 4.25056 3.36173 3.99118 3.04089 0.98678 3.08371 3.26560 3.45751 5.27725 4.12054 467 - -
2.68618 4.42225 2.77519 2.73123 3.46354 2.40513 3.72494 3.29354 2.67741 2.69355 4.24690 2.90347 2.73739 3.18146 2.89801 2.37887 2.77519 2.98518 4.58477 3.61503
0.03157 3.86699 4.58934 0.61958 0.77255 0.48576 0.95510

468 2.96489 4.51452 4.04353 3.63658 3.20533 3.82998 4.37299 2.38038 3.40496 1.80083 1.38846 3.91475 4.29056 3.78719 3.61092 3.31343 3.27009 2.38586 5.04542 3.80671 468 - -
2.68618 4.42225 2.77519 2.73123 3.46354 2.40513 3.72494 3.29354 2.67741 2.69355 4.24690 2.90347 2.73739 3.18146 2.89801 2.37887 2.77519 2.98518 4.58477 3.61503
0.03157 3.86699 4.58934 0.61958 0.77255 0.48576 0.95510

469 1.02168 4.18931 3.45704 3.24808 4.04693 3.01400 4.27077 3.17162 3.26419 3.05349 4.08591 3.39907 3.75270 3.60586 3.51510 2.53965 2.81663 2.82697 5.47463 4.27427 469 - -
2.68618 4.42225 2.77519 2.73123 3.46354 2.40513 3.72494 3.29354 2.67741 2.69355 4.24690 2.90347 2.73739 3.18146 2.89801 2.37887 2.77519 2.98518 4.58477 3.61503
0.03157 3.86699 4.58934 0.61958 0.77255 0.48576 0.95510

470 2.98371 5.11116 0.92594 2.33150 4.48744 3.20651 3.92621 4.07436 3.00929 3.68366 4.66164 2.88760 3.85892 3.16534 3.52477 2.94668 3.32216 3.71413 5.65711 4.36124 470 - -
2.68618 4.42225 2.77519 2.73123 3.46354 2.40513 3.72494 3.29354 2.67741 2.69355 4.24690 2.90347 2.73739 3.18146 2.89801 2.37887 2.77519 2.98518 4.58477 3.61503
0.03157 3.86699 4.58934 0.61958 0.77255 0.48576 0.95510

471 1.02168 4.18931 3.45704 3.24808 4.04693 3.01400 4.27077 3.17162 3.26419 3.05349 4.08591 3.39907 3.75270 3.60586 3.51510 2.53965 2.81663 2.82697 5.47463 4.27427 471 - -
2.68618 4.42225 2.77519 2.73123 3.46354 2.40513 3.72494 3.29354 2.67741 2.69355 4.24690 2.90347 2.73739 3.18146 2.89801 2.37887 2.77519 2.98518 4.58477 3.61503
0.03157 3.86699 4.58934 0.61958 0.77255 0.48576 0.95510

472 2.94758 5.05751 2.48529 1.03318 4.40304 3.27053 3.85685 3.85586 2.73669 3.47385 4.44812 2.92979 3.87486 3.07262 3.14497 2.93053 3.25177 3.54026 5.57565 4.29788 472 - -
2.68618 4.42225 2.77519 2.73123 3.46354 2.40513 3.72494 3.29354 2.67741 2.69355 4.24690 2.90347 2.73739 3.18146 2.89801 2.37887 2.77519 2.98518 4.58477 3.61503

0.03157 3.86699 4.58934 0.61958 0.77255 0.48576 0.95510

473 2.94758 5.05751 2.48529 1.03318 4.40304 3.27053 3.85685 3.85586 2.73669 3.47385 4.44812 2.92979 3.87486 3.07262 3.14497 2.93053 3.25177 3.54026 5.57565 4.29788 473 - -

2.68618 4.42225 2.77519 2.73123 3.46354 2.40513 3.72494 3.29354 2.67741 2.69355 4.24690 2.90347 2.73739 3.18146 2.89801 2.37887 2.77519 2.98518 4.58477 3.61503

0.03157 3.86699 4.58934 0.61958 0.77255 0.48576 0.95510

474 2.96489 4.51452 4.04353 3.63658 3.20533 3.82998 4.37299 2.38038 3.40496 1.80083 1.38846 3.91475 4.29056 3.78719 3.61092 3.31343 3.27009 2.38586 5.04542 3.80671 474 - -

2.68618 4.42225 2.77519 2.73123 3.46354 2.40513 3.72494 3.29354 2.67741 2.69355 4.24690 2.90347 2.73739 3.18146 2.89801 2.37887 2.77519 2.98518 4.58477 3.61503

0.03157 3.86699 4.58934 0.61958 0.77255 0.48576 0.95510

475 3.25138 4.65475 4.23121 3.94466 1.02072 3.94245 3.87481 2.77574 3.85183 2.14705 3.50670 4.01301 4.40115 4.02131 3.97290 3.52160 3.55251 2.76604 4.12549 2.49318 475 - -

2.68618 4.42225 2.77519 2.73123 3.46354 2.40513 3.72494 3.29354 2.67741 2.69355 4.24690 2.90347 2.73739 3.18146 2.89801 2.37887 2.77519 2.98518 4.58477 3.61503

0.03157 3.86699 4.58934 0.61958 0.77255 0.48576 0.95510

476 2.98371 5.11116 0.92594 2.33150 4.48744 3.20651 3.92621 4.07436 3.00929 3.68366 4.66164 2.88760 3.85892 3.16534 3.52477 2.94668 3.32216 3.71413 5.65711 4.36124 476 - -

2.68618 4.42225 2.77519 2.73123 3.46354 2.40513 3.72494 3.29354 2.67741 2.69355 4.24690 2.90347 2.73739 3.18146 2.89801 2.37887 2.77519 2.98518 4.58477 3.61503

0.03157 3.86699 4.58934 0.61958 0.77255 0.48576 0.95510

477 2.87640 4.86196 2.89930 2.65395 4.02980 3.36241 3.81221 3.65971 2.47512 3.16261 4.19062 3.11364 3.91579 1.24381 2.75940 2.92438 3.17337 3.38973 5.30278 3.99617 477 - -

2.68618 4.42225 2.77519 2.73123 3.46354 2.40513 3.72494 3.29354 2.67741 2.69355 4.24690 2.90347 2.73739 3.18146 2.89801 2.37887 2.77519 2.98518 4.58477 3.61503

0.03157 3.86699 4.58934 0.61958 0.77255 0.48576 0.95510

478 3.32650 4.70314 4.08279 3.78752 2.66069 3.72402 3.89311 3.34986 3.50985 2.74569 3.98391 3.93769 4.24129 3.89775 3.63917 3.53025 3.62662 3.24004 0.89641 2.66307 478 - -

2.68618 4.42225 2.77519 2.73123 3.46354 2.40513 3.72494 3.29354 2.67741 2.69355 4.24690 2.90347 2.73739 3.18146 2.89801 2.37887 2.77519 2.98518 4.58477 3.61503

0.03157 3.86699 4.58934 0.61958 0.77255 0.48576 0.95510

479 2.74420 4.50247 3.35077 3.18900 4.18468 3.19029 4.26692 3.72867 3.24583 3.35611 4.40571 3.49627 0.83787 3.63478 3.49200 2.91151 3.18532 3.37467 5.37275 4.30526 479 - -

2.68618 4.42225 2.77519 2.73123 3.46354 2.40513 3.72494 3.29354 2.67741 2.69355 4.24690 2.90347 2.73739 3.18146 2.89801 2.37887 2.77519 2.98518 4.58477 3.61503

0.03157 3.86699 4.58934 0.61958 0.77255 0.48576 0.95510

480 2.31230 4.23263 3.20165 3.01716 4.07082 2.96261 4.12717 3.59269 3.09056 3.31334 4.23988 3.24282 3.70096 3.44030 3.37676 1.09934 2.79202 3.12899 5.44375 4.15432 480 - -

2.68618 4.42225 2.77519 2.73123 3.46354 2.40513 3.72494 3.29354 2.67741 2.69355 4.24690 2.90347 2.73739 3.18146 2.89801 2.37887 2.77519 2.98518 4.58477 3.61503

0.03157 3.86699 4.58934 0.61958 0.77255 0.48576 0.95510

481 2.71888 4.71528 2.70526 2.59018 4.07794 3.18029 3.91656 3.78713 2.81979 3.43461 4.38989 1.16778 3.83188 3.18930 3.17427 2.78628 3.10056 3.40989 5.38448 4.02666 481 - -

2.68618 4.42225 2.77519 2.73123 3.46354 2.40513 3.72494 3.29354 2.67741 2.69355 4.24690 2.90347 2.73739 3.18146 2.89801 2.37887 2.77519 2.98518 4.58477 3.61503

0.03157 3.86699 4.58934 0.61958 0.77255 0.48576 0.95510

482 3.14912 4.61302 4.18070 3.82053 3.13362 3.97519 4.47798 2.33736 3.58833 0.88196 3.15827 4.09653 4.41149 3.95797 3.76529 3.54541 3.44362 2.37328 5.01092 3.73297 482 - -

2.68618 4.42225 2.77519 2.73123 3.46354 2.40513 3.72494 3.29354 2.67741 2.69355 4.24690 2.90347 2.73739 3.18146 2.89801 2.37887 2.77519 2.98518 4.58477 3.61503

0.03157 3.86699 4.58934 0.61958 0.77255 0.48576 0.95510

483 2.98371 5.11116 0.92594 2.33150 4.48744 3.20651 3.92621 4.07436 3.00929 3.68366 4.66164 2.88760 3.85892 3.16534 3.52477 2.94668 3.32216 3.71413 5.65711 4.36124 483 - -

2.68618 4.42225 2.77519 2.73123 3.46354 2.40513 3.72494 3.29354 2.67741 2.69355 4.24690 2.90347 2.73739 3.18146 2.89801 2.37887 2.77519 2.98518 4.58477 3.61503

0.03157 3.86699 4.58934 0.61958 0.77255 0.48576 0.95510

484 2.31230 4.23263 3.20165 3.01716 4.07082 2.96261 4.12717 3.59269 3.09056 3.31334 4.23988 3.24282 3.70096 3.44030 3.37676 1.09934 2.79202 3.12899 5.44375 4.15432 484 - -

2.68618 4.42225 2.77519 2.73123 3.46354 2.40513 3.72494 3.29354 2.67741 2.69355 4.24690 2.90347 2.73739 3.18146 2.89801 2.37887 2.77519 2.98518 4.58477 3.61503
0.03157 3.86699 4.58934 0.61958 0.77255 0.48576 0.95510
485 2.31230 4.23263 3.20165 3.01716 4.07082 2.96261 4.12717 3.59269 3.09056 3.31334 4.23988 3.24282 3.70096 3.44030 3.37676 1.09934 2.79202 3.12899 5.44375 4.15432 485 - -
2.68618 4.42225 2.77519 2.73123 3.46354 2.40513 3.72494 3.29354 2.67741 2.69355 4.24690 2.90347 2.73739 3.18146 2.89801 2.37887 2.77519 2.98518 4.58477 3.61503
0.03157 3.86699 4.58934 0.61958 0.77255 0.48576 0.95510
486 2.96711 4.93068 3.14273 2.76522 4.31586 3.45716 3.75616 3.74479 1.04579 3.31106 4.27280 3.18993 3.95788 2.94794 2.43058 3.00945 3.22078 3.45288 5.37276 4.18474 486 - -
2.68618 4.42225 2.77519 2.73123 3.46354 2.40513 3.72494 3.29354 2.67741 2.69355 4.24690 2.90347 2.73739 3.18146 2.89801 2.37887 2.77519 2.98518 4.58477 3.61503
0.03157 3.86699 4.58934 0.61958 0.77255 0.48576 0.95510
487 1.02168 4.18931 3.45704 3.24808 4.04693 3.01400 4.27077 3.17162 3.26419 3.05349 4.08591 3.39907 3.75270 3.60586 3.51510 2.53965 2.81663 2.82697 5.47463 4.27427 487 - -
2.68618 4.42225 2.77519 2.73123 3.46354 2.40513 3.72494 3.29354 2.67741 2.69355 4.24690 2.90347 2.73739 3.18146 2.89801 2.37887 2.77519 2.98518 4.58477 3.61503
0.03157 3.86699 4.58934 0.61958 0.77255 0.48576 0.95510
488 2.40976 4.25482 3.42322 3.17496 3.96403 3.09429 4.19270 3.09049 3.11783 2.95418 4.00504 3.38522 3.79760 3.51292 3.37678 2.61097 1.18122 2.78606 5.40231 4.18065 488 - -
2.68618 4.42225 2.77519 2.73123 3.46354 2.40513 3.72494 3.29354 2.67741 2.69355 4.24690 2.90347 2.73739 3.18146 2.89801 2.37887 2.77519 2.98518 4.58477 3.61503
0.03157 3.86699 4.58934 0.61958 0.77255 0.48576 0.95510
489 2.40976 4.25482 3.42322 3.17496 3.96403 3.09429 4.19270 3.09049 3.11783 2.95418 4.00504 3.38522 3.79760 3.51292 3.37678 2.61097 1.18122 2.78606 5.40231 4.18065 489 - -
2.68618 4.42225 2.77519 2.73123 3.46354 2.40513 3.72494 3.29354 2.67741 2.69355 4.24690 2.90347 2.73739 3.18146 2.89801 2.37887 2.77519 2.98518 4.58477 3.61503
0.03157 3.86699 4.58934 0.61958 0.77255 0.48576 0.95510
490 2.31230 4.23263 3.20165 3.01716 4.07082 2.96261 4.12717 3.59269 3.09056 3.31334 4.23988 3.24282 3.70096 3.44030 3.37676 1.09934 2.79202 3.12899 5.44375 4.15432 490 - -
2.68618 4.42225 2.77519 2.73123 3.46354 2.40513 3.72494 3.29354 2.67741 2.69355 4.24690 2.90347 2.73739 3.18146 2.89801 2.37887 2.77519 2.98518 4.58477 3.61503
0.03157 3.86699 4.58934 0.61958 0.77255 0.48576 0.95510
491 3.02684 4.44709 4.32791 3.94350 3.37049 4.06063 4.67263 1.08471 3.77153 1.95764 3.30670 4.18960 4.49236 4.12038 3.96872 3.55539 3.32610 1.84575 5.26019 3.99802 491 - -
2.68618 4.42225 2.77519 2.73123 3.46354 2.40513 3.72494 3.29354 2.67741 2.69355 4.24690 2.90347 2.73739 3.18146 2.89801 2.37887 2.77519 2.98518 4.58477 3.61503
0.03157 3.86699 4.58934 0.61958 0.77255 0.48576 0.95510
492 3.02684 4.44709 4.32791 3.94350 3.37049 4.06063 4.67263 1.08471 3.77153 1.95764 3.30670 4.18960 4.49236 4.12038 3.96872 3.55539 3.32610 1.84575 5.26019 3.99802 492 - -
2.68618 4.42225 2.77519 2.73123 3.46354 2.40513 3.72494 3.29354 2.67741 2.69355 4.24690 2.90347 2.73739 3.18146 2.89801 2.37887 2.77519 2.98518 4.58477 3.61503
0.03157 3.86699 4.58934 0.61958 0.77255 0.48576 0.95510
493 2.65811 4.43408 3.33076 3.22947 4.37188 0.73761 4.35008 3.97016 3.41000 3.62498 4.60067 3.49506 3.82505 3.73459 3.64665 2.82986 3.13892 3.49954 5.46848 4.47359 493 - -
2.68618 4.42225 2.77519 2.73123 3.46354 2.40513 3.72494 3.29354 2.67741 2.69355 4.24690 2.90347 2.73739 3.18146 2.89801 2.37887 2.77519 2.98518 4.58477 3.61503
0.03157 3.86699 4.58934 0.61958 0.77255 0.48576 0.95510
494 2.65811 4.43408 3.33076 3.22947 4.37188 0.73761 4.35008 3.97016 3.41000 3.62498 4.60067 3.49506 3.82505 3.73459 3.64665 2.82986 3.13892 3.49954 5.46848 4.47359 494 - -
2.68618 4.42225 2.77519 2.73123 3.46354 2.40513 3.72494 3.29354 2.67741 2.69355 4.24690 2.90347 2.73739 3.18146 2.89801 2.37887 2.77519 2.98518 4.58477 3.61503
0.03157 3.86699 4.58934 0.61958 0.77255 0.48576 0.95510
495 3.23918 4.73039 3.83015 3.55850 2.29358 3.82376 3.61597 3.24916 3.41618 2.70450 3.92476 3.71671 4.29772 3.72354 3.60101 3.37642 3.52583 3.11945 3.93713 0.97494 495 - -
2.68618 4.42225 2.77519 2.73123 3.46354 2.40513 3.72494 3.29354 2.67741 2.69355 4.24690 2.90347 2.73739 3.18146 2.89801 2.37887 2.77519 2.98518 4.58477 3.61503
0.03157 3.86699 4.58934 0.61958 0.77255 0.48576 0.95510

496 1.02168 4.18931 3.45704 3.24808 4.04693 3.01400 4.27077 3.17162 3.26419 3.05349 4.08591 3.39907 3.75270 3.60586 3.51510 2.53965 2.81663 2.82697 5.47463 4.27427 496 - -
2.68618 4.42225 2.77519 2.73123 3.46354 2.40513 3.72494 3.29354 2.67741 2.69355 4.24690 2.90347 2.73739 3.18146 2.89801 2.37887 2.77519 2.98518 4.58477 3.61503
0.03157 3.86699 4.58934 0.61958 0.77255 0.48576 0.95510

497 2.99928 4.83880 3.48937 3.00157 4.19200 3.48833 3.82030 3.73332 2.23516 3.25025 4.25056 3.36173 3.99118 3.04089 0.98678 3.08371 3.26560 3.45751 5.27725 4.12054 497 - -
2.68618 4.42225 2.77519 2.73123 3.46354 2.40513 3.72494 3.29354 2.67741 2.69355 4.24690 2.90347 2.73739 3.18146 2.89801 2.37887 2.77519 2.98518 4.58477 3.61503
0.03157 3.86699 4.58934 0.61958 0.77255 0.48576 0.95510

498 2.71888 4.71528 2.70526 2.59018 4.07794 3.18029 3.91656 3.78713 2.81979 3.43461 4.38989 1.16778 3.83188 3.18930 3.17427 2.78628 3.10056 3.40989 5.38448 4.02666 498 - -
2.68618 4.42225 2.77519 2.73123 3.46354 2.40513 3.72494 3.29354 2.67741 2.69355 4.24690 2.90347 2.73739 3.18146 2.89801 2.37887 2.77519 2.98518 4.58477 3.61503
0.03157 3.86699 4.58934 0.61958 0.77255 0.48576 0.95510

499 2.65811 4.43408 3.33076 3.22947 4.37188 0.73761 4.35008 3.97016 3.41000 3.62498 4.60067 3.49506 3.82505 3.73459 3.64665 2.82986 3.13892 3.49954 5.46848 4.47359 499 - -
2.68618 4.42225 2.77519 2.73123 3.46354 2.40513 3.72494 3.29354 2.67741 2.69355 4.24690 2.90347 2.73739 3.18146 2.89801 2.37887 2.77519 2.98518 4.58477 3.61503
0.03157 3.86699 4.58934 0.61958 0.77255 0.48576 0.95510

500 3.14912 4.61302 4.18070 3.82053 3.13362 3.97519 4.47798 2.33736 3.58833 0.88196 3.15827 4.09653 4.41149 3.95797 3.76529 3.54541 3.44362 2.37328 5.01092 3.73297 500 - -
2.68618 4.42225 2.77519 2.73123 3.46354 2.40513 3.72494 3.29354 2.67741 2.69355 4.24690 2.90347 2.73739 3.18146 2.89801 2.37887 2.77519 2.98518 4.58477 3.61503
0.03157 3.86699 4.58934 0.61958 0.77255 0.48576 0.95510

501 2.74420 4.50247 3.35077 3.18900 4.18468 3.19029 4.26692 3.72867 3.24583 3.35611 4.40571 3.49627 0.83787 3.63478 3.49200 2.91151 3.18532 3.37467 5.37275 4.30526 501 - -
2.68618 4.42225 2.77519 2.73123 3.46354 2.40513 3.72494 3.29354 2.67741 2.69355 4.24690 2.90347 2.73739 3.18146 2.89801 2.37887 2.77519 2.98518 4.58477 3.61503
0.03157 3.86699 4.58934 0.61958 0.77255 0.48576 0.95510

502 2.98371 5.11116 0.92594 2.33150 4.48744 3.20651 3.92621 4.07436 3.00929 3.68366 4.66164 2.88760 3.85892 3.16534 3.52477 2.94668 3.32216 3.71413 5.65711 4.36124 502 - -
2.68618 4.42225 2.77519 2.73123 3.46354 2.40513 3.72494 3.29354 2.67741 2.69355 4.24690 2.90347 2.73739 3.18146 2.89801 2.37887 2.77519 2.98518 4.58477 3.61503
0.03157 3.86699 4.58934 0.61958 0.77255 0.48576 0.95510

503 2.96711 4.93068 3.14273 2.76522 4.31586 3.45716 3.75616 3.74479 1.04579 3.31106 4.27280 3.18993 3.95788 2.94794 2.43058 3.00945 3.22078 3.45288 5.37276 4.18474 503 - -
2.68618 4.42225 2.77519 2.73123 3.46354 2.40513 3.72494 3.29354 2.67741 2.69355 4.24690 2.90347 2.73739 3.18146 2.89801 2.37887 2.77519 2.98518 4.58477 3.61503
0.03157 3.86699 4.58934 0.61958 0.77255 0.48576 0.95510

504 1.02168 4.18931 3.45704 3.24808 4.04693 3.01400 4.27077 3.17162 3.26419 3.05349 4.08591 3.39907 3.75270 3.60586 3.51510 2.53965 2.81663 2.82697 5.47463 4.27427 504 - -
2.68618 4.42225 2.77519 2.73123 3.46354 2.40513 3.72494 3.29354 2.67741 2.69355 4.24690 2.90347 2.73739 3.18146 2.89801 2.37887 2.77519 2.98518 4.58477 3.61503
0.03157 3.86699 4.58934 0.61958 0.77255 0.48576 0.95510

505 2.80376 4.34788 4.11775 3.78070 3.54607 3.71822 4.58336 2.05053 3.64840 2.23405 3.51204 3.97141 4.27903 4.00859 3.86143 3.22141 3.16544 1.03182 5.32959 4.06087 505 - -
2.68618 4.42225 2.77519 2.73123 3.46354 2.40513 3.72494 3.29354 2.67741 2.69355 4.24690 2.90347 2.73739 3.18146 2.89801 2.37887 2.77519 2.98518 4.58477 3.61503
0.03157 3.86699 4.58934 0.61958 0.77255 0.48576 0.95510

506 2.31230 4.23263 3.20165 3.01716 4.07082 2.96261 4.12717 3.59269 3.09056 3.31334 4.23988 3.24282 3.70096 3.44030 3.37676 1.09934 2.79202 3.12899 5.44375 4.15432 506 - -
2.68618 4.42225 2.77519 2.73123 3.46354 2.40513 3.72494 3.29354 2.67741 2.69355 4.24690 2.90347 2.73739 3.18146 2.89801 2.37887 2.77519 2.98518 4.58477 3.61503
0.03157 3.86699 4.58934 0.61958 0.77255 0.48576 0.95510

507 3.14912 4.61302 4.18070 3.82053 3.13362 3.97519 4.47798 2.33736 3.58833 0.88196 3.15827 4.09653 4.41149 3.95797 3.76529 3.54541 3.44362 2.37328 5.01092 3.73297 507 - -
2.68618 4.42225 2.77519 2.73123 3.46354 2.40513 3.72494 3.29354 2.67741 2.69355 4.24690 2.90347 2.73739 3.18146 2.89801 2.37887 2.77519 2.98518 4.58477 3.61503

0.03157 3.86699 4.58934 0.61958 0.77255 0.48576 0.95510

508 3.25138 4.65475 4.23121 3.94466 1.02072 3.94245 3.87481 2.77574 3.85183 2.14705 3.50670 4.01301 4.40115 4.02131 3.97290 3.52160 3.55251 2.76604 4.12549 2.49318 508 - -

2.68618 4.42225 2.77519 2.73123 3.46354 2.40513 3.72494 3.29354 2.67741 2.69355 4.24690 2.90347 2.73739 3.18146 2.89801 2.37887 2.77519 2.98518 4.58477 3.61503

0.03157 3.86699 4.58934 0.61958 0.77255 0.48576 0.95510

509 2.98886 4.80727 3.12036 2.87975 3.28089 3.44287 1.15410 3.69093 2.72295 3.18558 4.22810 3.28930 3.99510 3.24591 2.98899 3.05959 3.29203 3.42616 4.74602 3.23190 509 - -

2.68618 4.42225 2.77519 2.73123 3.46354 2.40513 3.72494 3.29354 2.67741 2.69355 4.24690 2.90347 2.73739 3.18146 2.89801 2.37887 2.77519 2.98518 4.58477 3.61503

0.03157 3.86699 4.58934 0.61958 0.77255 0.48576 0.95510

510 2.99928 4.83880 3.48937 3.00157 4.19200 3.48833 3.82030 3.73332 2.23516 3.25025 4.25056 3.36173 3.99118 3.04089 0.98678 3.08371 3.26560 3.45751 5.27725 4.12054 510 - -

2.68618 4.42225 2.77519 2.73123 3.46354 2.40513 3.72494 3.29354 2.67741 2.69355 4.24690 2.90347 2.73739 3.18146 2.89801 2.37887 2.77519 2.98518 4.58477 3.61503

0.03157 3.86699 4.58934 0.61958 0.77255 0.48576 0.95510

511 2.40976 4.25482 3.42322 3.17496 3.96403 3.09429 4.19270 3.09049 3.11783 2.95418 4.00504 3.38522 3.79760 3.51292 3.37678 2.61097 1.18122 2.78606 5.40231 4.18065 511 - -

2.68618 4.42225 2.77519 2.73123 3.46354 2.40513 3.72494 3.29354 2.67741 2.69355 4.24690 2.90347 2.73739 3.18146 2.89801 2.37887 2.77519 2.98518 4.58477 3.61503

0.03157 3.86699 4.58934 0.61958 0.77255 0.48576 0.95510

512 3.14912 4.61302 4.18070 3.82053 3.13362 3.97519 4.47798 2.33736 3.58833 0.88196 3.15827 4.09653 4.41149 3.95797 3.76529 3.54541 3.44362 2.37328 5.01092 3.73297 512 - -

2.68618 4.42225 2.77519 2.73123 3.46354 2.40513 3.72494 3.29354 2.67741 2.69355 4.24690 2.90347 2.73739 3.18146 2.89801 2.37887 2.77519 2.98518 4.58477 3.61503

0.03157 3.86699 4.58934 0.61958 0.77255 0.48576 0.95510

513 2.58006 0.95161 4.09776 3.83614 3.82875 3.20419 4.48443 2.97175 3.66306 2.88747 4.00858 3.82459 3.91071 4.00937 3.78840 2.85170 3.04637 2.71670 5.24602 4.11647 513 - -

2.68618 4.42225 2.77519 2.73123 3.46354 2.40513 3.72494 3.29354 2.67741 2.69355 4.24690 2.90347 2.73739 3.18146 2.89801 2.37887 2.77519 2.98518 4.58477 3.61503

0.03157 3.86699 4.58934 0.61958 0.77255 0.48576 0.95510

514 2.94758 5.05751 2.48529 1.03318 4.40304 3.27053 3.85685 3.85586 2.73669 3.47385 4.44812 2.92979 3.87486 3.07262 3.14497 2.93053 3.25177 3.54026 5.57565 4.29788 514 - -

2.68618 4.42225 2.77519 2.73123 3.46354 2.40513 3.72494 3.29354 2.67741 2.69355 4.24690 2.90347 2.73739 3.18146 2.89801 2.37887 2.77519 2.98518 4.58477 3.61503

0.03157 3.86699 4.58934 0.61958 0.77255 0.48576 0.95510

515 2.87640 4.86196 2.89930 2.65395 4.02980 3.36241 3.81221 3.65971 2.47512 3.16261 4.19062 3.11364 3.91579 1.24381 2.75940 2.92438 3.17337 3.38973 5.30278 3.99617 515 - -

2.68618 4.42225 2.77519 2.73123 3.46354 2.40513 3.72494 3.29354 2.67741 2.69355 4.24690 2.90347 2.73739 3.18146 2.89801 2.37887 2.77519 2.98518 4.58477 3.61503

0.03157 3.86699 4.58934 0.61958 0.77255 0.48576 0.95510

516 2.96711 4.93068 3.14273 2.76522 4.31586 3.45716 3.75616 3.74479 1.04579 3.31106 4.27280 3.18993 3.95788 2.94794 2.43058 3.00945 3.22078 3.45288 5.37276 4.18474 516 - -

2.68618 4.42225 2.77519 2.73123 3.46354 2.40513 3.72494 3.29354 2.67741 2.69355 4.24690 2.90347 2.73739 3.18146 2.89801 2.37887 2.77519 2.98518 4.58477 3.61503

0.03157 3.86699 4.58934 0.61958 0.77255 0.48576 0.95510

517 3.14912 4.61302 4.18070 3.82053 3.13362 3.97519 4.47798 2.33736 3.58833 0.88196 3.15827 4.09653 4.41149 3.95797 3.76529 3.54541 3.44362 2.37328 5.01092 3.73297 517 - -

2.68618 4.42225 2.77519 2.73123 3.46354 2.40513 3.72494 3.29354 2.67741 2.69355 4.24690 2.90347 2.73739 3.18146 2.89801 2.37887 2.77519 2.98518 4.58477 3.61503

0.03157 3.86699 4.58934 0.61958 0.77255 0.48576 0.95510

518 3.25138 4.65475 4.23121 3.94466 1.02072 3.94245 3.87481 2.77574 3.85183 2.14705 3.50670 4.01301 4.40115 4.02131 3.97290 3.52160 3.55251 2.76604 4.12549 2.49318 518 - -

2.68618 4.42225 2.77519 2.73123 3.46354 2.40513 3.72494 3.29354 2.67741 2.69355 4.24690 2.90347 2.73739 3.18146 2.89801 2.37887 2.77519 2.98518 4.58477 3.61503

0.03157 3.86699 4.58934 0.61958 0.77255 0.48576 0.95510

519 3.14912 4.61302 4.18070 3.82053 3.13362 3.97519 4.47798 2.33736 3.58833 0.88196 3.15827 4.09653 4.41149 3.95797 3.76529 3.54541 3.44362 2.37328 5.01092 3.73297 519 - -

2.68618 4.42225 2.77519 2.73123 3.46354 2.40513 3.72494 3.29354 2.67741 2.69355 4.24690 2.90347 2.73739 3.18146 2.89801 2.37887 2.77519 2.98518 4.58477 3.61503
0.03157 3.86699 4.58934 0.61958 0.77255 0.48576 0.95510
520 2.98371 5.11116 0.92594 2.33150 4.48744 3.20651 3.92621 4.07436 3.00929 3.68366 4.66164 2.88760 3.85892 3.16534 3.52477 2.94668 3.32216 3.71413 5.65711 4.36124 520 - -
2.68618 4.42225 2.77519 2.73123 3.46354 2.40513 3.72494 3.29354 2.67741 2.69355 4.24690 2.90347 2.73739 3.18146 2.89801 2.37887 2.77519 2.98518 4.58477 3.61503
0.03157 3.86699 4.58934 0.61958 0.77255 0.48576 0.95510
521 2.94758 5.05751 2.48529 1.03318 4.40304 3.27053 3.85685 3.85586 2.73669 3.47385 4.44812 2.92979 3.87486 3.07262 3.14497 2.93053 3.25177 3.54026 5.57565 4.29788 521 - -
2.68618 4.42225 2.77519 2.73123 3.46354 2.40513 3.72494 3.29354 2.67741 2.69355 4.24690 2.90347 2.73739 3.18146 2.89801 2.37887 2.77519 2.98518 4.58477 3.61503
0.03157 3.86699 4.58934 0.61958 0.77255 0.48576 0.95510
522 2.80376 4.34788 4.11775 3.78070 3.54607 3.71822 4.58336 2.05053 3.64840 2.23405 3.51204 3.97141 4.27903 4.00859 3.86143 3.22141 3.16544 1.03182 5.32959 4.06087 522 - -
2.68618 4.42225 2.77519 2.73123 3.46354 2.40513 3.72494 3.29354 2.67741 2.69355 4.24690 2.90347 2.73739 3.18146 2.89801 2.37887 2.77519 2.98518 4.58477 3.61503
0.03157 3.86699 4.58934 0.61958 0.77255 0.48576 0.95510
523 2.31230 4.23263 3.20165 3.01716 4.07082 2.96261 4.12717 3.59269 3.09056 3.31334 4.23988 3.24282 3.70096 3.44030 3.37676 1.09934 2.79202 3.12899 5.44375 4.15432 523 - -
2.68618 4.42225 2.77519 2.73123 3.46354 2.40513 3.72494 3.29354 2.67741 2.69355 4.24690 2.90347 2.73739 3.18146 2.89801 2.37887 2.77519 2.98518 4.58477 3.61503
0.03157 3.86699 4.58934 0.61958 0.77255 0.48576 0.95510
524 3.14912 4.61302 4.18070 3.82053 3.13362 3.97519 4.47798 2.33736 3.58833 0.88196 3.15827 4.09653 4.41149 3.95797 3.76529 3.54541 3.44362 2.37328 5.01092 3.73297 524 - -
2.68618 4.42225 2.77519 2.73123 3.46354 2.40513 3.72494 3.29354 2.67741 2.69355 4.24690 2.90347 2.73739 3.18146 2.89801 2.37887 2.77519 2.98518 4.58477 3.61503
0.03157 3.86699 4.58934 0.61958 0.77255 0.48576 0.95510
525 2.40976 4.25482 3.42322 3.17496 3.96403 3.09429 4.19270 3.09049 3.11783 2.95418 4.00504 3.38522 3.79760 3.51292 3.37678 2.61097 1.18122 2.78606 5.40231 4.18065 525 - -
2.68618 4.42225 2.77519 2.73123 3.46354 2.40513 3.72494 3.29354 2.67741 2.69355 4.24690 2.90347 2.73739 3.18146 2.89801 2.37887 2.77519 2.98518 4.58477 3.61503
0.03157 3.86699 4.58934 0.61958 0.77255 0.48576 0.95510
526 3.14912 4.61302 4.18070 3.82053 3.13362 3.97519 4.47798 2.33736 3.58833 0.88196 3.15827 4.09653 4.41149 3.95797 3.76529 3.54541 3.44362 2.37328 5.01092 3.73297 526 - -
2.68618 4.42225 2.77519 2.73123 3.46354 2.40513 3.72494 3.29354 2.67741 2.69355 4.24690 2.90347 2.73739 3.18146 2.89801 2.37887 2.77519 2.98518 4.58477 3.61503
0.03157 3.86699 4.58934 0.61958 0.77255 0.48576 0.95510
527 3.02684 4.44709 4.32791 3.94350 3.37049 4.06063 4.67263 1.08471 3.77153 1.95764 3.30670 4.18960 4.49236 4.12038 3.96872 3.55539 3.32610 1.84575 5.26019 3.99802 527 - -
2.68618 4.42225 2.77519 2.73123 3.46354 2.40513 3.72494 3.29354 2.67741 2.69355 4.24690 2.90347 2.73739 3.18146 2.89801 2.37887 2.77519 2.98518 4.58477 3.61503
0.03157 3.86699 4.58934 0.61958 0.77255 0.48576 0.95510
528 3.14912 4.61302 4.18070 3.82053 3.13362 3.97519 4.47798 2.33736 3.58833 0.88196 3.15827 4.09653 4.41149 3.95797 3.76529 3.54541 3.44362 2.37328 5.01092 3.73297 528 - -
2.68618 4.42225 2.77519 2.73123 3.46354 2.40513 3.72494 3.29354 2.67741 2.69355 4.24690 2.90347 2.73739 3.18146 2.89801 2.37887 2.77519 2.98518 4.58477 3.61503
0.03157 3.86699 4.58934 0.61958 0.77255 0.48576 0.95510
529 1.02168 4.18931 3.45704 3.24808 4.04693 3.01400 4.27077 3.17162 3.26419 3.05349 4.08591 3.39907 3.75270 3.60586 3.51510 2.53965 2.81663 2.82697 5.47463 4.27427 529 - -
2.68618 4.42225 2.77519 2.73123 3.46354 2.40513 3.72494 3.29354 2.67741 2.69355 4.24690 2.90347 2.73739 3.18146 2.89801 2.37887 2.77519 2.98518 4.58477 3.61503
0.03157 3.86699 4.58934 0.61958 0.77255 0.48576 0.95510
530 2.80376 4.34788 4.11775 3.78070 3.54607 3.71822 4.58336 2.05053 3.64840 2.23405 3.51204 3.97141 4.27903 4.00859 3.86143 3.22141 3.16544 1.03182 5.32959 4.06087 530 - -
2.68618 4.42225 2.77519 2.73123 3.46354 2.40513 3.72494 3.29354 2.67741 2.69355 4.24690 2.90347 2.73739 3.18146 2.89801 2.37887 2.77519 2.98518 4.58477 3.61503
0.03157 3.86699 4.58934 0.61958 0.77255 0.48576 0.95510

531 2.58006 0.95161 4.09776 3.83614 3.82875 3.20419 4.48443 2.97175 3.66306 2.88747 4.00858 3.82459 3.91071 4.00937 3.78840 2.85170 3.04637 2.71670 5.24602 4.11647 531 - -
2.68618 4.42225 2.77519 2.73123 3.46354 2.40513 3.72494 3.29354 2.67741 2.69355 4.24690 2.90347 2.73739 3.18146 2.89801 2.37887 2.77519 2.98518 4.58477 3.61503
0.03157 3.86699 4.58934 0.61958 0.77255 0.48576 0.95510
532 2.65811 4.43408 3.33076 3.22947 4.37188 0.73761 4.35008 3.97016 3.41000 3.62498 4.60067 3.49506 3.82505 3.73459 3.64665 2.82986 3.13892 3.49954 5.46848 4.47359 532 - -
2.68618 4.42225 2.77519 2.73123 3.46354 2.40513 3.72494 3.29354 2.67741 2.69355 4.24690 2.90347 2.73739 3.18146 2.89801 2.37887 2.77519 2.98518 4.58477 3.61503
0.03157 3.86699 4.58934 0.61958 0.77255 0.48576 0.95510
533 2.40976 4.25482 3.42322 3.17496 3.96403 3.09429 4.19270 3.09049 3.11783 2.95418 4.00504 3.38522 3.79760 3.51292 3.37678 2.61097 1.18122 2.78606 5.40231 4.18065 533 - -
2.68618 4.42225 2.77519 2.73123 3.46354 2.40513 3.72494 3.29354 2.67741 2.69355 4.24690 2.90347 2.73739 3.18146 2.89801 2.37887 2.77519 2.98518 4.58477 3.61503
0.03157 3.86699 4.58934 0.61958 0.77255 0.48576 0.95510
534 3.14912 4.61302 4.18070 3.82053 3.13362 3.97519 4.47798 2.33736 3.58833 0.88196 3.15827 4.09653 4.41149 3.95797 3.76529 3.54541 3.44362 2.37328 5.01092 3.73297 534 - -
2.68618 4.42225 2.77519 2.73123 3.46354 2.40513 3.72494 3.29354 2.67741 2.69355 4.24690 2.90347 2.73739 3.18146 2.89801 2.37887 2.77519 2.98518 4.58477 3.61503
0.03157 3.86699 4.58934 0.61958 0.77255 0.48576 0.95510
535 2.65811 4.43408 3.33076 3.22947 4.37188 0.73761 4.35008 3.97016 3.41000 3.62498 4.60067 3.49506 3.82505 3.73459 3.64665 2.82986 3.13892 3.49954 5.46848 4.47359 535 - -
2.68618 4.42225 2.77519 2.73123 3.46354 2.40513 3.72494 3.29354 2.67741 2.69355 4.24690 2.90347 2.73739 3.18146 2.89801 2.37887 2.77519 2.98518 4.58477 3.61503
0.03157 3.86699 4.58934 0.61958 0.77255 0.48576 0.95510
536 3.25138 4.65475 4.23121 3.94466 1.02072 3.94245 3.87481 2.77574 3.85183 2.14705 3.50670 4.01301 4.40115 4.02131 3.97290 3.52160 3.55251 2.76604 4.12549 2.49318 536 - -
2.68618 4.42225 2.77519 2.73123 3.46354 2.40513 3.72494 3.29354 2.67741 2.69355 4.24690 2.90347 2.73739 3.18146 2.89801 2.37887 2.77519 2.98518 4.58477 3.61503
0.03157 3.86699 4.58934 0.61958 0.77255 0.48576 0.95510
537 2.99928 4.83880 3.48937 3.00157 4.19200 3.48833 3.82030 3.73332 2.23516 3.25025 4.25056 3.36173 3.99118 3.04089 0.98678 3.08371 3.26560 3.45751 5.27725 4.12054 537 - -
2.68618 4.42225 2.77519 2.73123 3.46354 2.40513 3.72494 3.29354 2.67741 2.69355 4.24690 2.90347 2.73739 3.18146 2.89801 2.37887 2.77519 2.98518 4.58477 3.61503
0.03157 3.86699 4.58934 0.61958 0.77255 0.48576 0.95510
538 2.94758 5.05751 2.48529 1.03318 4.40304 3.27053 3.85685 3.85586 2.73669 3.47385 4.44812 2.92979 3.87486 3.07262 3.14497 2.93053 3.25177 3.54026 5.57565 4.29788 538 - -
2.68618 4.42225 2.77519 2.73123 3.46354 2.40513 3.72494 3.29354 2.67741 2.69355 4.24690 2.90347 2.73739 3.18146 2.89801 2.37887 2.77519 2.98518 4.58477 3.61503
0.03157 3.86699 4.58934 0.61958 0.77255 0.48576 0.95510
539 2.96489 4.51452 4.04353 3.63658 3.20533 3.82998 4.37299 2.38038 3.40496 1.80083 1.38846 3.91475 4.29056 3.78719 3.61092 3.31343 3.27009 2.38586 5.04542 3.80671 539 - -
2.68618 4.42225 2.77519 2.73123 3.46354 2.40513 3.72494 3.29354 2.67741 2.69355 4.24690 2.90347 2.73739 3.18146 2.89801 2.37887 2.77519 2.98518 4.58477 3.61503
0.03157 3.86699 4.58934 0.61958 0.77255 0.48576 0.95510
540 2.65811 4.43408 3.33076 3.22947 4.37188 0.73761 4.35008 3.97016 3.41000 3.62498 4.60067 3.49506 3.82505 3.73459 3.64665 2.82986 3.13892 3.49954 5.46848 4.47359 540 - -
2.68618 4.42225 2.77519 2.73123 3.46354 2.40513 3.72494 3.29354 2.67741 2.69355 4.24690 2.90347 2.73739 3.18146 2.89801 2.37887 2.77519 2.98518 4.58477 3.61503
0.03157 3.86699 4.58934 0.61958 0.77255 0.48576 0.95510
541 3.23918 4.73039 3.83015 3.55850 2.29358 3.82376 3.61597 3.24916 3.41618 2.70450 3.92476 3.71671 4.29772 3.72354 3.60101 3.37642 3.52583 3.11945 3.93713 0.97494 541 - -
2.68618 4.42225 2.77519 2.73123 3.46354 2.40513 3.72494 3.29354 2.67741 2.69355 4.24690 2.90347 2.73739 3.18146 2.89801 2.37887 2.77519 2.98518 4.58477 3.61503
0.03157 3.86699 4.58934 0.61958 0.77255 0.48576 0.95510
542 2.87640 4.86196 2.89930 2.65395 4.02980 3.36241 3.81221 3.65971 2.47512 3.16261 4.19062 3.11364 3.91579 1.24381 2.75940 2.92438 3.17337 3.38973 5.30278 3.99617 542 - -
2.68618 4.42225 2.77519 2.73123 3.46354 2.40513 3.72494 3.29354 2.67741 2.69355 4.24690 2.90347 2.73739 3.18146 2.89801 2.37887 2.77519 2.98518 4.58477 3.61503

0.03157 3.86699 4.58934 0.61958 0.77255 0.48576 0.95510

543 3.02684 4.44709 4.32791 3.94350 3.37049 4.06063 4.67263 1.08471 3.77153 1.95764 3.30670 4.18960 4.49236 4.12038 3.96872 3.55539 3.32610 1.84575 5.26019 3.99802 543 - -

2.68618 4.42225 2.77519 2.73123 3.46354 2.40513 3.72494 3.29354 2.67741 2.69355 4.24690 2.90347 2.73739 3.18146 2.89801 2.37887 2.77519 2.98518 4.58477 3.61503

0.03157 3.86699 4.58934 0.61958 0.77255 0.48576 0.95510

544 2.98886 4.80727 3.12036 2.87975 3.28089 3.44287 1.15410 3.69093 2.72295 3.18558 4.22810 3.28930 3.99510 3.24591 2.98899 3.05959 3.29203 3.42616 4.74602 3.23190 544 - -

2.68618 4.42225 2.77519 2.73123 3.46354 2.40513 3.72494 3.29354 2.67741 2.69355 4.24690 2.90347 2.73739 3.18146 2.89801 2.37887 2.77519 2.98518 4.58477 3.61503

0.03157 3.86699 4.58934 0.61958 0.77255 0.48576 0.95510

545 2.58006 0.95161 4.09776 3.83614 3.82875 3.20419 4.48443 2.97175 3.66306 2.88747 4.00858 3.82459 3.91071 4.00937 3.78840 2.85170 3.04637 2.71670 5.24602 4.11647 545 - -

2.68618 4.42225 2.77519 2.73123 3.46354 2.40513 3.72494 3.29354 2.67741 2.69355 4.24690 2.90347 2.73739 3.18146 2.89801 2.37887 2.77519 2.98518 4.58477 3.61503

0.03157 3.86699 4.58934 0.61958 0.77255 0.48576 0.95510

546 3.23918 4.73039 3.83015 3.55850 2.29358 3.82376 3.61597 3.24916 3.41618 2.70450 3.92476 3.71671 4.29772 3.72354 3.60101 3.37642 3.52583 3.11945 3.93713 0.97494 546 - -

2.68618 4.42225 2.77519 2.73123 3.46354 2.40513 3.72494 3.29354 2.67741 2.69355 4.24690 2.90347 2.73739 3.18146 2.89801 2.37887 2.77519 2.98518 4.58477 3.61503

0.03157 3.86699 4.58934 0.61958 0.77255 0.48576 0.95510

547 1.02168 4.18931 3.45704 3.24808 4.04693 3.01400 4.27077 3.17162 3.26419 3.05349 4.08591 3.39907 3.75270 3.60586 3.51510 2.53965 2.81663 2.82697 5.47463 4.27427 547 - -

2.68618 4.42225 2.77519 2.73123 3.46354 2.40513 3.72494 3.29354 2.67741 2.69355 4.24690 2.90347 2.73739 3.18146 2.89801 2.37887 2.77519 2.98518 4.58477 3.61503

0.03157 3.86699 4.58934 0.61958 0.77255 0.48576 0.95510

548 3.14912 4.61302 4.18070 3.82053 3.13362 3.97519 4.47798 2.33736 3.58833 0.88196 3.15827 4.09653 4.41149 3.95797 3.76529 3.54541 3.44362 2.37328 5.01092 3.73297 548 - -

2.68618 4.42225 2.77519 2.73123 3.46354 2.40513 3.72494 3.29354 2.67741 2.69355 4.24690 2.90347 2.73739 3.18146 2.89801 2.37887 2.77519 2.98518 4.58477 3.61503

0.03157 3.86699 4.58934 0.61958 0.77255 0.48576 0.95510

549 2.96711 4.93068 3.14273 2.76522 4.31586 3.45716 3.75616 3.74479 1.04579 3.31106 4.27280 3.18993 3.95788 2.94794 2.43058 3.00945 3.22078 3.45288 5.37276 4.18474 549 - -

2.68618 4.42225 2.77519 2.73123 3.46354 2.40513 3.72494 3.29354 2.67741 2.69355 4.24690 2.90347 2.73739 3.18146 2.89801 2.37887 2.77519 2.98518 4.58477 3.61503

0.03157 3.86699 4.58934 0.61958 0.77255 0.48576 0.95510

550 1.02168 4.18931 3.45704 3.24808 4.04693 3.01400 4.27077 3.17162 3.26419 3.05349 4.08591 3.39907 3.75270 3.60586 3.51510 2.53965 2.81663 2.82697 5.47463 4.27427 550 - -

2.68618 4.42225 2.77519 2.73123 3.46354 2.40513 3.72494 3.29354 2.67741 2.69355 4.24690 2.90347 2.73739 3.18146 2.89801 2.37887 2.77519 2.98518 4.58477 3.61503

0.03157 3.86699 4.58934 0.61958 0.77255 0.48576 0.95510

551 2.65811 4.43408 3.33076 3.22947 4.37188 0.73761 4.35008 3.97016 3.41000 3.62498 4.60067 3.49506 3.82505 3.73459 3.64665 2.82986 3.13892 3.49954 5.46848 4.47359 551 - -

2.68618 4.42225 2.77519 2.73123 3.46354 2.40513 3.72494 3.29354 2.67741 2.69355 4.24690 2.90347 2.73739 3.18146 2.89801 2.37887 2.77519 2.98518 4.58477 3.61503

0.03157 3.86699 4.58934 0.61958 0.77255 0.48576 0.95510

552 3.23918 4.73039 3.83015 3.55850 2.29358 3.82376 3.61597 3.24916 3.41618 2.70450 3.92476 3.71671 4.29772 3.72354 3.60101 3.37642 3.52583 3.11945 3.93713 0.97494 552 - -

2.68618 4.42225 2.77519 2.73123 3.46354 2.40513 3.72494 3.29354 2.67741 2.69355 4.24690 2.90347 2.73739 3.18146 2.89801 2.37887 2.77519 2.98518 4.58477 3.61503

0.03157 3.86699 4.58934 0.61958 0.77255 0.48576 0.95510

553 3.25138 4.65475 4.23121 3.94466 1.02072 3.94245 3.87481 2.77574 3.85183 2.14705 3.50670 4.01301 4.40115 4.02131 3.97290 3.52160 3.55251 2.76604 4.12549 2.49318 553 - -

2.68618 4.42225 2.77519 2.73123 3.46354 2.40513 3.72494 3.29354 2.67741 2.69355 4.24690 2.90347 2.73739 3.18146 2.89801 2.37887 2.77519 2.98518 4.58477 3.61503

0.03157 3.86699 4.58934 0.61958 0.77255 0.48576 0.95510

554 2.31230 4.23263 3.20165 3.01716 4.07082 2.96261 4.12717 3.59269 3.09056 3.31334 4.23988 3.24282 3.70096 3.44030 3.37676 1.09934 2.79202 3.12899 5.44375 4.15432 554 - -

2.68618 4.42225 2.77519 2.73123 3.46354 2.40513 3.72494 3.29354 2.67741 2.69355 4.24690 2.90347 2.73739 3.18146 2.89801 2.37887 2.77519 2.98518 4.58477 3.61503
0.03157 3.86699 4.58934 0.61958 0.77255 0.48576 0.95510
555 2.98371 5.11116 0.92594 2.33150 4.48744 3.20651 3.92621 4.07436 3.00929 3.68366 4.66164 2.88760 3.85892 3.16534 3.52477 2.94668 3.32216 3.71413 5.65711 4.36124 555 - -
2.68618 4.42225 2.77519 2.73123 3.46354 2.40513 3.72494 3.29354 2.67741 2.69355 4.24690 2.90347 2.73739 3.18146 2.89801 2.37887 2.77519 2.98518 4.58477 3.61503
0.03157 3.86699 4.58934 0.61958 0.77255 0.48576 0.95510
556 3.02684 4.44709 4.32791 3.94350 3.37049 4.06063 4.67263 1.08471 3.77153 1.95764 3.30670 4.18960 4.49236 4.12038 3.96872 3.55539 3.32610 1.84575 5.26019 3.99802 556 - -
2.68618 4.42225 2.77519 2.73123 3.46354 2.40513 3.72494 3.29354 2.67741 2.69355 4.24690 2.90347 2.73739 3.18146 2.89801 2.37887 2.77519 2.98518 4.58477 3.61503
0.03157 3.86699 4.58934 0.61958 0.77255 0.48576 0.95510
557 2.31230 4.23263 3.20165 3.01716 4.07082 2.96261 4.12717 3.59269 3.09056 3.31334 4.23988 3.24282 3.70096 3.44030 3.37676 1.09934 2.79202 3.12899 5.44375 4.15432 557 - -
2.68618 4.42225 2.77519 2.73123 3.46354 2.40513 3.72494 3.29354 2.67741 2.69355 4.24690 2.90347 2.73739 3.18146 2.89801 2.37887 2.77519 2.98518 4.58477 3.61503
0.03157 3.86699 4.58934 0.61958 0.77255 0.48576 0.95510
558 3.14912 4.61302 4.18070 3.82053 3.13362 3.97519 4.47798 2.33736 3.58833 0.88196 3.15827 4.09653 4.41149 3.95797 3.76529 3.54541 3.44362 2.37328 5.01092 3.73297 558 - -
2.68618 4.42225 2.77519 2.73123 3.46354 2.40513 3.72494 3.29354 2.67741 2.69355 4.24690 2.90347 2.73739 3.18146 2.89801 2.37887 2.77519 2.98518 4.58477 3.61503
0.03157 3.86699 4.58934 0.61958 0.77255 0.48576 0.95510
559 2.65811 4.43408 3.33076 3.22947 4.37188 0.73761 4.35008 3.97016 3.41000 3.62498 4.60067 3.49506 3.82505 3.73459 3.64665 2.82986 3.13892 3.49954 5.46848 4.47359 559 - -
2.68618 4.42225 2.77519 2.73123 3.46354 2.40513 3.72494 3.29354 2.67741 2.69355 4.24690 2.90347 2.73739 3.18146 2.89801 2.37887 2.77519 2.98518 4.58477 3.61503
0.03157 3.86699 4.58934 0.61958 0.77255 0.48576 0.95510
560 2.71888 4.71528 2.70526 2.59018 4.07794 3.18029 3.91656 3.78713 2.81979 3.43461 4.38989 1.16778 3.83188 3.18930 3.17427 2.78628 3.10056 3.40989 5.38448 4.02666 560 - -
2.68618 4.42225 2.77519 2.73123 3.46354 2.40513 3.72494 3.29354 2.67741 2.69355 4.24690 2.90347 2.73739 3.18146 2.89801 2.37887 2.77519 2.98518 4.58477 3.61503
0.03157 3.86699 4.58934 0.61958 0.77255 0.48576 0.95510
561 2.31230 4.23263 3.20165 3.01716 4.07082 2.96261 4.12717 3.59269 3.09056 3.31334 4.23988 3.24282 3.70096 3.44030 3.37676 1.09934 2.79202 3.12899 5.44375 4.15432 561 - -
2.68618 4.42225 2.77519 2.73123 3.46354 2.40513 3.72494 3.29354 2.67741 2.69355 4.24690 2.90347 2.73739 3.18146 2.89801 2.37887 2.77519 2.98518 4.58477 3.61503
0.03157 3.86699 4.58934 0.61958 0.77255 0.48576 0.95510
562 3.14912 4.61302 4.18070 3.82053 3.13362 3.97519 4.47798 2.33736 3.58833 0.88196 3.15827 4.09653 4.41149 3.95797 3.76529 3.54541 3.44362 2.37328 5.01092 3.73297 562 - -
2.68618 4.42225 2.77519 2.73123 3.46354 2.40513 3.72494 3.29354 2.67741 2.69355 4.24690 2.90347 2.73739 3.18146 2.89801 2.37887 2.77519 2.98518 4.58477 3.61503
0.03157 3.86699 4.58934 0.61958 0.77255 0.48576 0.95510
563 3.02684 4.44709 4.32791 3.94350 3.37049 4.06063 4.67263 1.08471 3.77153 1.95764 3.30670 4.18960 4.49236 4.12038 3.96872 3.55539 3.32610 1.84575 5.26019 3.99802 563 - -
2.68618 4.42225 2.77519 2.73123 3.46354 2.40513 3.72494 3.29354 2.67741 2.69355 4.24690 2.90347 2.73739 3.18146 2.89801 2.37887 2.77519 2.98518 4.58477 3.61503
0.03157 3.86699 4.58934 0.61958 0.77255 0.48576 0.95510
564 2.31230 4.23263 3.20165 3.01716 4.07082 2.96261 4.12717 3.59269 3.09056 3.31334 4.23988 3.24282 3.70096 3.44030 3.37676 1.09934 2.79202 3.12899 5.44375 4.15432 564 - -
2.68618 4.42225 2.77519 2.73123 3.46354 2.40513 3.72494 3.29354 2.67741 2.69355 4.24690 2.90347 2.73739 3.18146 2.89801 2.37887 2.77519 2.98518 4.58477 3.61503
0.03157 3.86699 4.58934 0.61958 0.77255 0.48576 0.95510
565 2.96489 4.51452 4.04353 3.63658 3.20533 3.82998 4.37299 2.38038 3.40496 1.80083 1.38846 3.91475 4.29056 3.78719 3.61092 3.31343 3.27009 2.38586 5.04542 3.80671 565 - -
2.68618 4.42225 2.77519 2.73123 3.46354 2.40513 3.72494 3.29354 2.67741 2.69355 4.24690 2.90347 2.73739 3.18146 2.89801 2.37887 2.77519 2.98518 4.58477 3.61503
0.03157 3.86699 4.58934 0.61958 0.77255 0.48576 0.95510

566 3.23918 4.73039 3.83015 3.55850 2.29358 3.82376 3.61597 3.24916 3.41618 2.70450 3.92476 3.71671 4.29772 3.72354 3.60101 3.37642 3.52583 3.11945 3.93713 0.97494 566 - -
2.68618 4.42225 2.77519 2.73123 3.46354 2.40513 3.72494 3.29354 2.67741 2.69355 4.24690 2.90347 2.73739 3.18146 2.89801 2.37887 2.77519 2.98518 4.58477 3.61503
0.03157 3.86699 4.58934 0.61958 0.77255 0.48576 0.95510
567 1.02168 4.18931 3.45704 3.24808 4.04693 3.01400 4.27077 3.17162 3.26419 3.05349 4.08591 3.39907 3.75270 3.60586 3.51510 2.53965 2.81663 2.82697 5.47463 4.27427 567 - -
2.68618 4.42225 2.77519 2.73123 3.46354 2.40513 3.72494 3.29354 2.67741 2.69355 4.24690 2.90347 2.73739 3.18146 2.89801 2.37887 2.77519 2.98518 4.58477 3.61503
0.03157 3.86699 4.58934 0.61958 0.77255 0.48576 0.95510
568 2.96711 4.93068 3.14273 2.76522 4.31586 3.45716 3.75616 3.74479 1.04579 3.31106 4.27280 3.18993 3.95788 2.94794 2.43058 3.00945 3.22078 3.45288 5.37276 4.18474 568 - -
2.68618 4.42225 2.77519 2.73123 3.46354 2.40513 3.72494 3.29354 2.67741 2.69355 4.24690 2.90347 2.73739 3.18146 2.89801 2.37887 2.77519 2.98518 4.58477 3.61503
0.03157 3.86699 4.58934 0.61958 0.77255 0.48576 0.95510
569 2.58006 0.95161 4.09776 3.83614 3.82875 3.20419 4.48443 2.97175 3.66306 2.88747 4.00858 3.82459 3.91071 4.00937 3.78840 2.85170 3.04637 2.71670 5.24602 4.11647 569 - -
2.68618 4.42225 2.77519 2.73123 3.46354 2.40513 3.72494 3.29354 2.67741 2.69355 4.24690 2.90347 2.73739 3.18146 2.89801 2.37887 2.77519 2.98518 4.58477 3.61503
0.03157 3.86699 4.58934 0.61958 0.77255 0.48576 0.95510
570 2.58006 0.95161 4.09776 3.83614 3.82875 3.20419 4.48443 2.97175 3.66306 2.88747 4.00858 3.82459 3.91071 4.00937 3.78840 2.85170 3.04637 2.71670 5.24602 4.11647 570 - -
2.68618 4.42225 2.77519 2.73123 3.46354 2.40513 3.72494 3.29354 2.67741 2.69355 4.24690 2.90347 2.73739 3.18146 2.89801 2.37887 2.77519 2.98518 4.58477 3.61503
0.03157 3.86699 4.58934 0.61958 0.77255 0.48576 0.95510
571 2.98371 5.11116 0.92594 2.33150 4.48744 3.20651 3.92621 4.07436 3.00929 3.68366 4.66164 2.88760 3.85892 3.16534 3.52477 2.94668 3.32216 3.71413 5.65711 4.36124 571 - -
2.68618 4.42225 2.77519 2.73123 3.46354 2.40513 3.72494 3.29354 2.67741 2.69355 4.24690 2.90347 2.73739 3.18146 2.89801 2.37887 2.77519 2.98518 4.58477 3.61503
0.03157 3.86699 4.58934 0.61958 0.77255 0.48576 0.95510
572 2.31230 4.23263 3.20165 3.01716 4.07082 2.96261 4.12717 3.59269 3.09056 3.31334 4.23988 3.24282 3.70096 3.44030 3.37676 1.09934 2.79202 3.12899 5.44375 4.15432 572 - -
2.68618 4.42225 2.77519 2.73123 3.46354 2.40513 3.72494 3.29354 2.67741 2.69355 4.24690 2.90347 2.73739 3.18146 2.89801 2.37887 2.77519 2.98518 4.58477 3.61503
0.03157 3.86699 4.58934 0.61958 0.77255 0.48576 0.95510
573 2.98371 5.11116 0.92594 2.33150 4.48744 3.20651 3.92621 4.07436 3.00929 3.68366 4.66164 2.88760 3.85892 3.16534 3.52477 2.94668 3.32216 3.71413 5.65711 4.36124 573 - -
2.68618 4.42225 2.77519 2.73123 3.46354 2.40513 3.72494 3.29354 2.67741 2.69355 4.24690 2.90347 2.73739 3.18146 2.89801 2.37887 2.77519 2.98518 4.58477 3.61503
0.03157 3.86699 4.58934 0.61958 0.77255 0.48576 0.95510
574 2.98371 5.11116 0.92594 2.33150 4.48744 3.20651 3.92621 4.07436 3.00929 3.68366 4.66164 2.88760 3.85892 3.16534 3.52477 2.94668 3.32216 3.71413 5.65711 4.36124 574 - -
2.68618 4.42225 2.77519 2.73123 3.46354 2.40513 3.72494 3.29354 2.67741 2.69355 4.24690 2.90347 2.73739 3.18146 2.89801 2.37887 2.77519 2.98518 4.58477 3.61503
0.03157 3.86699 4.58934 0.61958 0.77255 0.48576 0.95510
575 1.02168 4.18931 3.45704 3.24808 4.04693 3.01400 4.27077 3.17162 3.26419 3.05349 4.08591 3.39907 3.75270 3.60586 3.51510 2.53965 2.81663 2.82697 5.47463 4.27427 575 - -
2.68618 4.42225 2.77519 2.73123 3.46354 2.40513 3.72494 3.29354 2.67741 2.69355 4.24690 2.90347 2.73739 3.18146 2.89801 2.37887 2.77519 2.98518 4.58477 3.61503
0.03157 3.86699 4.58934 0.61958 0.77255 0.48576 0.95510
576 3.02684 4.44709 4.32791 3.94350 3.37049 4.06063 4.67263 1.08471 3.77153 1.95764 3.30670 4.18960 4.49236 4.12038 3.96872 3.55539 3.32610 1.84575 5.26019 3.99802 576 - -
2.68618 4.42225 2.77519 2.73123 3.46354 2.40513 3.72494 3.29354 2.67741 2.69355 4.24690 2.90347 2.73739 3.18146 2.89801 2.37887 2.77519 2.98518 4.58477 3.61503
0.03157 3.86699 4.58934 0.61958 0.77255 0.48576 0.95510
577 2.96711 4.93068 3.14273 2.76522 4.31586 3.45716 3.75616 3.74479 1.04579 3.31106 4.27280 3.18993 3.95788 2.94794 2.43058 3.00945 3.22078 3.45288 5.37276 4.18474 577 - -
2.68618 4.42225 2.77519 2.73123 3.46354 2.40513 3.72494 3.29354 2.67741 2.69355 4.24690 2.90347 2.73739 3.18146 2.89801 2.37887 2.77519 2.98518 4.58477 3.61503

0.03157 3.86699 4.58934 0.61958 0.77255 0.48576 0.95510

578 3.02684 4.44709 4.32791 3.94350 3.37049 4.06063 4.67263 1.08471 3.77153 1.95764 3.30670 4.18960 4.49236 4.12038 3.96872 3.55539 3.32610 1.84575 5.26019 3.99802 578 - -

2.68618 4.42225 2.77519 2.73123 3.46354 2.40513 3.72494 3.29354 2.67741 2.69355 4.24690 2.90347 2.73739 3.18146 2.89801 2.37887 2.77519 2.98518 4.58477 3.61503

0.03157 3.86699 4.58934 0.61958 0.77255 0.48576 0.95510

579 3.25138 4.65475 4.23121 3.94466 1.02072 3.94245 3.87481 2.77574 3.85183 2.14705 3.50670 4.01301 4.40115 4.02131 3.97290 3.52160 3.55251 2.76604 4.12549 2.49318 579 - -

2.68618 4.42225 2.77519 2.73123 3.46354 2.40513 3.72494 3.29354 2.67741 2.69355 4.24690 2.90347 2.73739 3.18146 2.89801 2.37887 2.77519 2.98518 4.58477 3.61503

0.03157 3.86699 4.58934 0.61958 0.77255 0.48576 0.95510

580 2.71888 4.71528 2.70526 2.59018 4.07794 3.18029 3.91656 3.78713 2.81979 3.43461 4.38989 1.16778 3.83188 3.18930 3.17427 2.78628 3.10056 3.40989 5.38448 4.02666 580 - -

2.68618 4.42225 2.77519 2.73123 3.46354 2.40513 3.72494 3.29354 2.67741 2.69355 4.24690 2.90347 2.73739 3.18146 2.89801 2.37887 2.77519 2.98518 4.58477 3.61503

0.03157 3.86699 4.58934 0.61958 0.77255 0.48576 0.95510

581 2.40976 4.25482 3.42322 3.17496 3.96403 3.09429 4.19270 3.09049 3.11783 2.95418 4.00504 3.38522 3.79760 3.51292 3.37678 2.61097 1.18122 2.78606 5.40231 4.18065 581 - -

2.68618 4.42225 2.77519 2.73123 3.46354 2.40513 3.72494 3.29354 2.67741 2.69355 4.24690 2.90347 2.73739 3.18146 2.89801 2.37887 2.77519 2.98518 4.58477 3.61503

0.03157 3.86699 4.58934 0.61958 0.77255 0.48576 0.95510

582 2.96489 4.51452 4.04353 3.63658 3.20533 3.82998 4.37299 2.38038 3.40496 1.80083 1.38846 3.91475 4.29056 3.78719 3.61092 3.31343 3.27009 2.38586 5.04542 3.80671 582 - -

2.68618 4.42225 2.77519 2.73123 3.46354 2.40513 3.72494 3.29354 2.67741 2.69355 4.24690 2.90347 2.73739 3.18146 2.89801 2.37887 2.77519 2.98518 4.58477 3.61503

0.03157 3.86699 4.58934 0.61958 0.77255 0.48576 0.95510

583 2.99928 4.83880 3.48937 3.00157 4.19200 3.48833 3.82030 3.73332 2.23516 3.25025 4.25056 3.36173 3.99118 3.04089 0.98678 3.08371 3.26560 3.45751 5.27725 4.12054 583 - -

2.68618 4.42225 2.77519 2.73123 3.46354 2.40513 3.72494 3.29354 2.67741 2.69355 4.24690 2.90347 2.73739 3.18146 2.89801 2.37887 2.77519 2.98518 4.58477 3.61503

0.03157 3.86699 4.58934 0.61958 0.77255 0.48576 0.95510

584 2.94758 5.05751 2.48529 1.03318 4.40304 3.27053 3.85685 3.85586 2.73669 3.47385 4.44812 2.92979 3.87486 3.07262 3.14497 2.93053 3.25177 3.54026 5.57565 4.29788 584 - -

2.68618 4.42225 2.77519 2.73123 3.46354 2.40513 3.72494 3.29354 2.67741 2.69355 4.24690 2.90347 2.73739 3.18146 2.89801 2.37887 2.77519 2.98518 4.58477 3.61503

0.03157 3.86699 4.58934 0.61958 0.77255 0.48576 0.95510

585 2.98886 4.80727 3.12036 2.87975 3.28089 3.44287 1.15410 3.69093 2.72295 3.18558 4.22810 3.28930 3.99510 3.24591 2.98899 3.05959 3.29203 3.42616 4.74602 3.23190 585 - -

2.68618 4.42225 2.77519 2.73123 3.46354 2.40513 3.72494 3.29354 2.67741 2.69355 4.24690 2.90347 2.73739 3.18146 2.89801 2.37887 2.77519 2.98518 4.58477 3.61503

0.03157 3.86699 4.58934 0.61958 0.77255 0.48576 0.95510

586 2.98371 5.11116 0.92594 2.33150 4.48744 3.20651 3.92621 4.07436 3.00929 3.68366 4.66164 2.88760 3.85892 3.16534 3.52477 2.94668 3.32216 3.71413 5.65711 4.36124 586 - -

2.68618 4.42225 2.77519 2.73123 3.46354 2.40513 3.72494 3.29354 2.67741 2.69355 4.24690 2.90347 2.73739 3.18146 2.89801 2.37887 2.77519 2.98518 4.58477 3.61503

0.03157 3.86699 4.58934 0.61958 0.77255 0.48576 0.95510

587 2.80376 4.34788 4.11775 3.78070 3.54607 3.71822 4.58336 2.05053 3.64840 2.23405 3.51204 3.97141 4.27903 4.00859 3.86143 3.22141 3.16544 1.03182 5.32959 4.06087 587 - -

2.68618 4.42225 2.77519 2.73123 3.46354 2.40513 3.72494 3.29354 2.67741 2.69355 4.24690 2.90347 2.73739 3.18146 2.89801 2.37887 2.77519 2.98518 4.58477 3.61503

0.03157 3.86699 4.58934 0.61958 0.77255 0.48576 0.95510

588 3.02684 4.44709 4.32791 3.94350 3.37049 4.06063 4.67263 1.08471 3.77153 1.95764 3.30670 4.18960 4.49236 4.12038 3.96872 3.55539 3.32610 1.84575 5.26019 3.99802 588 - -

2.68618 4.42225 2.77519 2.73123 3.46354 2.40513 3.72494 3.29354 2.67741 2.69355 4.24690 2.90347 2.73739 3.18146 2.89801 2.37887 2.77519 2.98518 4.58477 3.61503

0.03157 3.86699 4.58934 0.61958 0.77255 0.48576 0.95510

589 2.31230 4.23263 3.20165 3.01716 4.07082 2.96261 4.12717 3.59269 3.09056 3.31334 4.23988 3.24282 3.70096 3.44030 3.37676 1.09934 2.79202 3.12899 5.44375 4.15432 589 - -

2.68618 4.42225 2.77519 2.73123 3.46354 2.40513 3.72494 3.29354 2.67741 2.69355 4.24690 2.90347 2.73739 3.18146 2.89801 2.37887 2.77519 2.98518 4.58477 3.61503
0.03157 3.86699 4.58934 0.61958 0.77255 0.48576 0.95510
590 3.32650 4.70314 4.08279 3.78752 2.66069 3.72402 3.89311 3.34986 3.50985 2.74569 3.98391 3.93769 4.24129 3.89775 3.63917 3.53025 3.62662 3.24004 0.89641 2.66307 590 - -
2.68618 4.42225 2.77519 2.73123 3.46354 2.40513 3.72494 3.29354 2.67741 2.69355 4.24690 2.90347 2.73739 3.18146 2.89801 2.37887 2.77519 2.98518 4.58477 3.61503
0.03157 3.86699 4.58934 0.61958 0.77255 0.48576 0.95510
591 2.71888 4.71528 2.70526 2.59018 4.07794 3.18029 3.91656 3.78713 2.81979 3.43461 4.38989 1.16778 3.83188 3.18930 3.17427 2.78628 3.10056 3.40989 5.38448 4.02666 591 - -
2.68618 4.42225 2.77519 2.73123 3.46354 2.40513 3.72494 3.29354 2.67741 2.69355 4.24690 2.90347 2.73739 3.18146 2.89801 2.37887 2.77519 2.98518 4.58477 3.61503
0.03157 3.86699 4.58934 0.61958 0.77255 0.48576 0.95510
592 2.31230 4.23263 3.20165 3.01716 4.07082 2.96261 4.12717 3.59269 3.09056 3.31334 4.23988 3.24282 3.70096 3.44030 3.37676 1.09934 2.79202 3.12899 5.44375 4.15432 592 - -
2.68618 4.42225 2.77519 2.73123 3.46354 2.40513 3.72494 3.29354 2.67741 2.69355 4.24690 2.90347 2.73739 3.18146 2.89801 2.37887 2.77519 2.98518 4.58477 3.61503
0.03157 3.86699 4.58934 0.61958 0.77255 0.48576 0.95510
593 3.14912 4.61302 4.18070 3.82053 3.13362 3.97519 4.47798 2.33736 3.58833 0.88196 3.15827 4.09653 4.41149 3.95797 3.76529 3.54541 3.44362 2.37328 5.01092 3.73297 593 - -
2.68618 4.42225 2.77519 2.73123 3.46354 2.40513 3.72494 3.29354 2.67741 2.69355 4.24690 2.90347 2.73739 3.18146 2.89801 2.37887 2.77519 2.98518 4.58477 3.61503
0.03157 3.86699 4.58934 0.61958 0.77255 0.48576 0.95510
594 3.02684 4.44709 4.32791 3.94350 3.37049 4.06063 4.67263 1.08471 3.77153 1.95764 3.30670 4.18960 4.49236 4.12038 3.96872 3.55539 3.32610 1.84575 5.26019 3.99802 594 - -
2.68618 4.42225 2.77519 2.73123 3.46354 2.40513 3.72494 3.29354 2.67741 2.69355 4.24690 2.90347 2.73739 3.18146 2.89801 2.37887 2.77519 2.98518 4.58477 3.61503
0.03157 3.86699 4.58934 0.61958 0.77255 0.48576 0.95510
595 2.31230 4.23263 3.20165 3.01716 4.07082 2.96261 4.12717 3.59269 3.09056 3.31334 4.23988 3.24282 3.70096 3.44030 3.37676 1.09934 2.79202 3.12899 5.44375 4.15432 595 - -
2.68618 4.42225 2.77519 2.73123 3.46354 2.40513 3.72494 3.29354 2.67741 2.69355 4.24690 2.90347 2.73739 3.18146 2.89801 2.37887 2.77519 2.98518 4.58477 3.61503
0.03157 3.86699 4.58934 0.61958 0.77255 0.48576 0.95510
596 2.58006 0.95161 4.09776 3.83614 3.82875 3.20419 4.48443 2.97175 3.66306 2.88747 4.00858 3.82459 3.91071 4.00937 3.78840 2.85170 3.04637 2.71670 5.24602 4.11647 596 - -
2.68618 4.42225 2.77519 2.73123 3.46354 2.40513 3.72494 3.29354 2.67741 2.69355 4.24690 2.90347 2.73739 3.18146 2.89801 2.37887 2.77519 2.98518 4.58477 3.61503
0.03157 3.86699 4.58934 0.61958 0.77255 0.48576 0.95510
597 3.23918 4.73039 3.83015 3.55850 2.29358 3.82376 3.61597 3.24916 3.41618 2.70450 3.92476 3.71671 4.29772 3.72354 3.60101 3.37642 3.52583 3.11945 3.93713 0.97494 597 - -
2.68618 4.42225 2.77519 2.73123 3.46354 2.40513 3.72494 3.29354 2.67741 2.69355 4.24690 2.90347 2.73739 3.18146 2.89801 2.37887 2.77519 2.98518 4.58477 3.61503
0.03157 3.86699 4.58934 0.61958 0.77255 0.48576 0.95510
598 3.02684 4.44709 4.32791 3.94350 3.37049 4.06063 4.67263 1.08471 3.77153 1.95764 3.30670 4.18960 4.49236 4.12038 3.96872 3.55539 3.32610 1.84575 5.26019 3.99802 598 - -
2.68618 4.42225 2.77519 2.73123 3.46354 2.40513 3.72494 3.29354 2.67741 2.69355 4.24690 2.90347 2.73739 3.18146 2.89801 2.37887 2.77519 2.98518 4.58477 3.61503
0.03157 3.86699 4.58934 0.61958 0.77255 0.48576 0.95510
599 3.14912 4.61302 4.18070 3.82053 3.13362 3.97519 4.47798 2.33736 3.58833 0.88196 3.15827 4.09653 4.41149 3.95797 3.76529 3.54541 3.44362 2.37328 5.01092 3.73297 599 - -
2.68618 4.42225 2.77519 2.73123 3.46354 2.40513 3.72494 3.29354 2.67741 2.69355 4.24690 2.90347 2.73739 3.18146 2.89801 2.37887 2.77519 2.98518 4.58477 3.61503
0.03157 3.86699 4.58934 0.61958 0.77255 0.48576 0.95510
600 2.87640 4.86196 2.89930 2.65395 4.02980 3.36241 3.81221 3.65971 2.47512 3.16261 4.19062 3.11364 3.91579 1.24381 2.75940 2.92438 3.17337 3.38973 5.30278 3.99617 600 - -
2.68618 4.42225 2.77519 2.73123 3.46354 2.40513 3.72494 3.29354 2.67741 2.69355 4.24690 2.90347 2.73739 3.18146 2.89801 2.37887 2.77519 2.98518 4.58477 3.61503
0.03157 3.86699 4.58934 0.61958 0.77255 0.48576 0.95510

601 2.99928 4.83880 3.48937 3.00157 4.19200 3.48833 3.82030 3.73332 2.23516 3.25025 4.25056 3.36173 3.99118 3.04089 0.98678 3.08371 3.26560 3.45751 5.27725 4.12054 601 - -
2.68618 4.42225 2.77519 2.73123 3.46354 2.40513 3.72494 3.29354 2.67741 2.69355 4.24690 2.90347 2.73739 3.18146 2.89801 2.37887 2.77519 2.98518 4.58477 3.61503
0.03157 3.86699 4.58934 0.61958 0.77255 0.48576 0.95510
602 2.71888 4.71528 2.70526 2.59018 4.07794 3.18029 3.91656 3.78713 2.81979 3.43461 4.38989 1.16778 3.83188 3.18930 3.17427 2.78628 3.10056 3.40989 5.38448 4.02666 602 - -
2.68618 4.42225 2.77519 2.73123 3.46354 2.40513 3.72494 3.29354 2.67741 2.69355 4.24690 2.90347 2.73739 3.18146 2.89801 2.37887 2.77519 2.98518 4.58477 3.61503
0.03157 3.86699 4.58934 0.61958 0.77255 0.48576 0.95510
603 2.65811 4.43408 3.33076 3.22947 4.37188 0.73761 4.35008 3.97016 3.41000 3.62498 4.60067 3.49506 3.82505 3.73459 3.64665 2.82986 3.13892 3.49954 5.46848 4.47359 603 - -
2.68618 4.42225 2.77519 2.73123 3.46354 2.40513 3.72494 3.29354 2.67741 2.69355 4.24690 2.90347 2.73739 3.18146 2.89801 2.37887 2.77519 2.98518 4.58477 3.61503
0.03157 3.86699 4.58934 0.61958 0.77255 0.48576 0.95510
604 2.98371 5.11116 0.92594 2.33150 4.48744 3.20651 3.92621 4.07436 3.00929 3.68366 4.66164 2.88760 3.85892 3.16534 3.52477 2.94668 3.32216 3.71413 5.65711 4.36124 604 - -
2.68618 4.42225 2.77519 2.73123 3.46354 2.40513 3.72494 3.29354 2.67741 2.69355 4.24690 2.90347 2.73739 3.18146 2.89801 2.37887 2.77519 2.98518 4.58477 3.61503
0.03157 3.86699 4.58934 0.61958 0.77255 0.48576 0.95510
605 2.94758 5.05751 2.48529 1.03318 4.40304 3.27053 3.85685 3.85586 2.73669 3.47385 4.44812 2.92979 3.87486 3.07262 3.14497 2.93053 3.25177 3.54026 5.57565 4.29788 605 - -
2.68618 4.42225 2.77519 2.73123 3.46354 2.40513 3.72494 3.29354 2.67741 2.69355 4.24690 2.90347 2.73739 3.18146 2.89801 2.37887 2.77519 2.98518 4.58477 3.61503
0.03157 3.86699 4.58934 0.61958 0.77255 0.48576 0.95510
606 1.02168 4.18931 3.45704 3.24808 4.04693 3.01400 4.27077 3.17162 3.26419 3.05349 4.08591 3.39907 3.75270 3.60586 3.51510 2.53965 2.81663 2.82697 5.47463 4.27427 606 - -
2.68618 4.42225 2.77519 2.73123 3.46354 2.40513 3.72494 3.29354 2.67741 2.69355 4.24690 2.90347 2.73739 3.18146 2.89801 2.37887 2.77519 2.98518 4.58477 3.61503
0.03157 3.86699 4.58934 0.61958 0.77255 0.48576 0.95510
607 3.14912 4.61302 4.18070 3.82053 3.13362 3.97519 4.47798 2.33736 3.58833 0.88196 3.15827 4.09653 4.41149 3.95797 3.76529 3.54541 3.44362 2.37328 5.01092 3.73297 607 - -
2.68618 4.42225 2.77519 2.73123 3.46354 2.40513 3.72494 3.29354 2.67741 2.69355 4.24690 2.90347 2.73739 3.18146 2.89801 2.37887 2.77519 2.98518 4.58477 3.61503
0.03157 3.86699 4.58934 0.61958 0.77255 0.48576 0.95510
608 1.02168 4.18931 3.45704 3.24808 4.04693 3.01400 4.27077 3.17162 3.26419 3.05349 4.08591 3.39907 3.75270 3.60586 3.51510 2.53965 2.81663 2.82697 5.47463 4.27427 608 - -
2.68618 4.42225 2.77519 2.73123 3.46354 2.40513 3.72494 3.29354 2.67741 2.69355 4.24690 2.90347 2.73739 3.18146 2.89801 2.37887 2.77519 2.98518 4.58477 3.61503
0.03157 3.86699 4.58934 0.61958 0.77255 0.48576 0.95510
609 3.14912 4.61302 4.18070 3.82053 3.13362 3.97519 4.47798 2.33736 3.58833 0.88196 3.15827 4.09653 4.41149 3.95797 3.76529 3.54541 3.44362 2.37328 5.01092 3.73297 609 - -
2.68618 4.42225 2.77519 2.73123 3.46354 2.40513 3.72494 3.29354 2.67741 2.69355 4.24690 2.90347 2.73739 3.18146 2.89801 2.37887 2.77519 2.98518 4.58477 3.61503
0.03157 3.86699 4.58934 0.61958 0.77255 0.48576 0.95510
610 3.32650 4.70314 4.08279 3.78752 2.66069 3.72402 3.89311 3.34986 3.50985 2.74569 3.98391 3.93769 4.24129 3.89775 3.63917 3.53025 3.62662 3.24004 0.89641 2.66307 610 - -
2.68618 4.42225 2.77519 2.73123 3.46354 2.40513 3.72494 3.29354 2.67741 2.69355 4.24690 2.90347 2.73739 3.18146 2.89801 2.37887 2.77519 2.98518 4.58477 3.61503
0.03157 3.86699 4.58934 0.61958 0.77255 0.48576 0.95510
611 2.31230 4.23263 3.20165 3.01716 4.07082 2.96261 4.12717 3.59269 3.09056 3.31334 4.23988 3.24282 3.70096 3.44030 3.37676 1.09934 2.79202 3.12899 5.44375 4.15432 611 - -
2.68618 4.42225 2.77519 2.73123 3.46354 2.40513 3.72494 3.29354 2.67741 2.69355 4.24690 2.90347 2.73739 3.18146 2.89801 2.37887 2.77519 2.98518 4.58477 3.61503
0.03157 3.86699 4.58934 0.61958 0.77255 0.48576 0.95510
612 2.99928 4.83880 3.48937 3.00157 4.19200 3.48833 3.82030 3.73332 2.23516 3.25025 4.25056 3.36173 3.99118 3.04089 0.98678 3.08371 3.26560 3.45751 5.27725 4.12054 612 - -
2.68618 4.42225 2.77519 2.73123 3.46354 2.40513 3.72494 3.29354 2.67741 2.69355 4.24690 2.90347 2.73739 3.18146 2.89801 2.37887 2.77519 2.98518 4.58477 3.61503

0.03157 3.86699 4.58934 0.61958 0.77255 0.48576 0.95510
613 2.96489 4.51452 4.04353 3.63658 3.20533 3.82998 4.37299 2.38038 3.40496 1.80083 1.38846 3.91475 4.29056 3.78719 3.61092 3.31343 3.27009 2.38586 5.04542 3.80671 613 - -
2.68618 4.42225 2.77519 2.73123 3.46354 2.40513 3.72494 3.29354 2.67741 2.69355 4.24690 2.90347 2.73739 3.18146 2.89801 2.37887 2.77519 2.98518 4.58477 3.61503
0.03157 3.86699 4.58934 0.61958 0.77255 0.48576 0.95510
614 2.71888 4.71528 2.70526 2.59018 4.07794 3.18029 3.91656 3.78713 2.81979 3.43461 4.38989 1.16778 3.83188 3.18930 3.17427 2.78628 3.10056 3.40989 5.38448 4.02666 614 - -
2.68618 4.42225 2.77519 2.73123 3.46354 2.40513 3.72494 3.29354 2.67741 2.69355 4.24690 2.90347 2.73739 3.18146 2.89801 2.37887 2.77519 2.98518 4.58477 3.61503
0.03157 3.86699 4.58934 0.61958 0.77255 0.48576 0.95510
615 2.94758 5.05751 2.48529 1.03318 4.40304 3.27053 3.85685 3.85586 2.73669 3.47385 4.44812 2.92979 3.87486 3.07262 3.14497 2.93053 3.25177 3.54026 5.57565 4.29788 615 - -
2.68618 4.42225 2.77519 2.73123 3.46354 2.40513 3.72494 3.29354 2.67741 2.69355 4.24690 2.90347 2.73739 3.18146 2.89801 2.37887 2.77519 2.98518 4.58477 3.61503
0.03157 3.86699 4.58934 0.61958 0.77255 0.48576 0.95510
616 2.96711 4.93068 3.14273 2.76522 4.31586 3.45716 3.75616 3.74479 1.04579 3.31106 4.27280 3.18993 3.95788 2.94794 2.43058 3.00945 3.22078 3.45288 5.37276 4.18474 616 - -
2.68618 4.42225 2.77519 2.73123 3.46354 2.40513 3.72494 3.29354 2.67741 2.69355 4.24690 2.90347 2.73739 3.18146 2.89801 2.37887 2.77519 2.98518 4.58477 3.61503
0.03157 3.86699 4.58934 0.61958 0.77255 0.48576 0.95510
617 2.94758 5.05751 2.48529 1.03318 4.40304 3.27053 3.85685 3.85586 2.73669 3.47385 4.44812 2.92979 3.87486 3.07262 3.14497 2.93053 3.25177 3.54026 5.57565 4.29788 617 - -
2.68618 4.42225 2.77519 2.73123 3.46354 2.40513 3.72494 3.29354 2.67741 2.69355 4.24690 2.90347 2.73739 3.18146 2.89801 2.37887 2.77519 2.98518 4.58477 3.61503
0.03157 3.86699 4.58934 0.61958 0.77255 0.48576 0.95510
618 3.02684 4.44709 4.32791 3.94350 3.37049 4.06063 4.67263 1.08471 3.77153 1.95764 3.30670 4.18960 4.49236 4.12038 3.96872 3.55539 3.32610 1.84575 5.26019 3.99802 618 - -
2.68618 4.42225 2.77519 2.73123 3.46354 2.40513 3.72494 3.29354 2.67741 2.69355 4.24690 2.90347 2.73739 3.18146 2.89801 2.37887 2.77519 2.98518 4.58477 3.61503
0.03157 3.86699 4.58934 0.61958 0.77255 0.48576 0.95510
619 2.96711 4.93068 3.14273 2.76522 4.31586 3.45716 3.75616 3.74479 1.04579 3.31106 4.27280 3.18993 3.95788 2.94794 2.43058 3.00945 3.22078 3.45288 5.37276 4.18474 619 - -
2.68618 4.42225 2.77519 2.73123 3.46354 2.40513 3.72494 3.29354 2.67741 2.69355 4.24690 2.90347 2.73739 3.18146 2.89801 2.37887 2.77519 2.98518 4.58477 3.61503
0.03157 3.86699 4.58934 0.61958 0.77255 0.48576 0.95510
620 2.74420 4.50247 3.35077 3.18900 4.18468 3.19029 4.26692 3.72867 3.24583 3.35611 4.40571 3.49627 0.83787 3.63478 3.49200 2.91151 3.18532 3.37467 5.37275 4.30526 620 - -
2.68618 4.42225 2.77519 2.73123 3.46354 2.40513 3.72494 3.29354 2.67741 2.69355 4.24690 2.90347 2.73739 3.18146 2.89801 2.37887 2.77519 2.98518 4.58477 3.61503
0.03157 3.86699 4.58934 0.61958 0.77255 0.48576 0.95510
621 2.98371 5.11116 0.92594 2.33150 4.48744 3.20651 3.92621 4.07436 3.00929 3.68366 4.66164 2.88760 3.85892 3.16534 3.52477 2.94668 3.32216 3.71413 5.65711 4.36124 621 - -
2.68618 4.42225 2.77519 2.73123 3.46354 2.40513 3.72494 3.29354 2.67741 2.69355 4.24690 2.90347 2.73739 3.18146 2.89801 2.37887 2.77519 2.98518 4.58477 3.61503
0.03157 3.86699 4.58934 0.61958 0.77255 0.48576 0.95510
622 3.02684 4.44709 4.32791 3.94350 3.37049 4.06063 4.67263 1.08471 3.77153 1.95764 3.30670 4.18960 4.49236 4.12038 3.96872 3.55539 3.32610 1.84575 5.26019 3.99802 622 - -
2.68618 4.42225 2.77519 2.73123 3.46354 2.40513 3.72494 3.29354 2.67741 2.69355 4.24690 2.90347 2.73739 3.18146 2.89801 2.37887 2.77519 2.98518 4.58477 3.61503
0.03157 3.86699 4.58934 0.61958 0.77255 0.48576 0.95510
623 3.02684 4.44709 4.32791 3.94350 3.37049 4.06063 4.67263 1.08471 3.77153 1.95764 3.30670 4.18960 4.49236 4.12038 3.96872 3.55539 3.32610 1.84575 5.26019 3.99802 623 - -
2.68618 4.42225 2.77519 2.73123 3.46354 2.40513 3.72494 3.29354 2.67741 2.69355 4.24690 2.90347 2.73739 3.18146 2.89801 2.37887 2.77519 2.98518 4.58477 3.61503
0.03157 3.86699 4.58934 0.61958 0.77255 0.48576 0.95510
624 2.40976 4.25482 3.42322 3.17496 3.96403 3.09429 4.19270 3.09049 3.11783 2.95418 4.00504 3.38522 3.79760 3.51292 3.37678 2.61097 1.18122 2.78606 5.40231 4.18065 624 - -

2.68618 4.42225 2.77519 2.73123 3.46354 2.40513 3.72494 3.29354 2.67741 2.69355 4.24690 2.90347 2.73739 3.18146 2.89801 2.37887 2.77519 2.98518 4.58477 3.61503

0.03157 3.86699 4.58934 0.61958 0.77255 0.48576 0.95510

625 3.14912 4.61302 4.18070 3.82053 3.13362 3.97519 4.47798 2.33736 3.58833 0.88196 3.15827 4.09653 4.41149 3.95797 3.76529 3.54541 3.44362 2.37328 5.01092 3.73297 625 - -

2.68618 4.42225 2.77519 2.73123 3.46354 2.40513 3.72494 3.29354 2.67741 2.69355 4.24690 2.90347 2.73739 3.18146 2.89801 2.37887 2.77519 2.98518 4.58477 3.61503

0.03157 3.86699 4.58934 0.61958 0.77255 0.48576 0.95510

626 2.40976 4.25482 3.42322 3.17496 3.96403 3.09429 4.19270 3.09049 3.11783 2.95418 4.00504 3.38522 3.79760 3.51292 3.37678 2.61097 1.18122 2.78606 5.40231 4.18065 626 - -

2.68618 4.42225 2.77519 2.73123 3.46354 2.40513 3.72494 3.29354 2.67741 2.69355 4.24690 2.90347 2.73739 3.18146 2.89801 2.37887 2.77519 2.98518 4.58477 3.61503

0.03157 3.86699 4.58934 0.61958 0.77255 0.48576 0.95510

627 3.14912 4.61302 4.18070 3.82053 3.13362 3.97519 4.47798 2.33736 3.58833 0.88196 3.15827 4.09653 4.41149 3.95797 3.76529 3.54541 3.44362 2.37328 5.01092 3.73297 627 - -

2.68618 4.42225 2.77519 2.73123 3.46354 2.40513 3.72494 3.29354 2.67741 2.69355 4.24690 2.90347 2.73739 3.18146 2.89801 2.37887 2.77519 2.98518 4.58477 3.61503

0.03157 3.86699 4.58934 0.61958 0.77255 0.48576 0.95510

628 2.80376 4.34788 4.11775 3.78070 3.54607 3.71822 4.58336 2.05053 3.64840 2.23405 3.51204 3.97141 4.27903 4.00859 3.86143 3.22141 3.16544 1.03182 5.32959 4.06087 628 - -

2.68618 4.42225 2.77519 2.73123 3.46354 2.40513 3.72494 3.29354 2.67741 2.69355 4.24690 2.90347 2.73739 3.18146 2.89801 2.37887 2.77519 2.98518 4.58477 3.61503

0.03157 3.86699 4.58934 0.61958 0.77255 0.48576 0.95510

629 3.02684 4.44709 4.32791 3.94350 3.37049 4.06063 4.67263 1.08471 3.77153 1.95764 3.30670 4.18960 4.49236 4.12038 3.96872 3.55539 3.32610 1.84575 5.26019 3.99802 629 - -

2.68618 4.42225 2.77519 2.73123 3.46354 2.40513 3.72494 3.29354 2.67741 2.69355 4.24690 2.90347 2.73739 3.18146 2.89801 2.37887 2.77519 2.98518 4.58477 3.61503

0.03157 3.86699 4.58934 0.61958 0.77255 0.48576 0.95510

630 2.31230 4.23263 3.20165 3.01716 4.07082 2.96261 4.12717 3.59269 3.09056 3.31334 4.23988 3.24282 3.70096 3.44030 3.37676 1.09934 2.79202 3.12899 5.44375 4.15432 630 - -

2.68618 4.42225 2.77519 2.73123 3.46354 2.40513 3.72494 3.29354 2.67741 2.69355 4.24690 2.90347 2.73739 3.18146 2.89801 2.37887 2.77519 2.98518 4.58477 3.61503

0.03157 3.86699 4.58934 0.61958 0.77255 0.48576 0.95510

631 1.02168 4.18931 3.45704 3.24808 4.04693 3.01400 4.27077 3.17162 3.26419 3.05349 4.08591 3.39907 3.75270 3.60586 3.51510 2.53965 2.81663 2.82697 5.47463 4.27427 631 - -

2.68618 4.42225 2.77519 2.73123 3.46354 2.40513 3.72494 3.29354 2.67741 2.69355 4.24690 2.90347 2.73739 3.18146 2.89801 2.37887 2.77519 2.98518 4.58477 3.61503

0.03157 3.86699 4.58934 0.61958 0.77255 0.48576 0.95510

632 3.25138 4.65475 4.23121 3.94466 1.02072 3.94245 3.87481 2.77574 3.85183 2.14705 3.50670 4.01301 4.40115 4.02131 3.97290 3.52160 3.55251 2.76604 4.12549 2.49318 632 - -

2.68618 4.42225 2.77519 2.73123 3.46354 2.40513 3.72494 3.29354 2.67741 2.69355 4.24690 2.90347 2.73739 3.18146 2.89801 2.37887 2.77519 2.98518 4.58477 3.61503

0.03157 3.86699 4.58934 0.61958 0.77255 0.48576 0.95510

633 2.99928 4.83880 3.48937 3.00157 4.19200 3.48833 3.82030 3.73332 2.23516 3.25025 4.25056 3.36173 3.99118 3.04089 0.98678 3.08371 3.26560 3.45751 5.27725 4.12054 633 - -

2.68618 4.42225 2.77519 2.73123 3.46354 2.40513 3.72494 3.29354 2.67741 2.69355 4.24690 2.90347 2.73739 3.18146 2.89801 2.37887 2.77519 2.98518 4.58477 3.61503

0.03157 3.86699 4.58934 0.61958 0.77255 0.48576 0.95510

634 3.23918 4.73039 3.83015 3.55850 2.29358 3.82376 3.61597 3.24916 3.41618 2.70450 3.92476 3.71671 4.29772 3.72354 3.60101 3.37642 3.52583 3.11945 3.93713 0.97494 634 - -

2.68618 4.42225 2.77519 2.73123 3.46354 2.40513 3.72494 3.29354 2.67741 2.69355 4.24690 2.90347 2.73739 3.18146 2.89801 2.37887 2.77519 2.98518 4.58477 3.61503

0.03157 3.86699 4.58934 0.61958 0.77255 0.48576 0.95510

635 2.40976 4.25482 3.42322 3.17496 3.96403 3.09429 4.19270 3.09049 3.11783 2.95418 4.00504 3.38522 3.79760 3.51292 3.37678 2.61097 1.18122 2.78606 5.40231 4.18065 635 - -

2.68618 4.42225 2.77519 2.73123 3.46354 2.40513 3.72494 3.29354 2.67741 2.69355 4.24690 2.90347 2.73739 3.18146 2.89801 2.37887 2.77519 2.98518 4.58477 3.61503

0.03157 3.86699 4.58934 0.61958 0.77255 0.48576 0.95510

636 2.94758 5.05751 2.48529 1.03318 4.40304 3.27053 3.85685 3.85586 2.73669 3.47385 4.44812 2.92979 3.87486 3.07262 3.14497 2.93053 3.25177 3.54026 5.57565 4.29788 636 - -
2.68618 4.42225 2.77519 2.73123 3.46354 2.40513 3.72494 3.29354 2.67741 2.69355 4.24690 2.90347 2.73739 3.18146 2.89801 2.37887 2.77519 2.98518 4.58477 3.61503
0.03157 3.86699 4.58934 0.61958 0.77255 0.48576 0.95510
637 2.31230 4.23263 3.20165 3.01716 4.07082 2.96261 4.12717 3.59269 3.09056 3.31334 4.23988 3.24282 3.70096 3.44030 3.37676 1.09934 2.79202 3.12899 5.44375 4.15432 637 - -
2.68618 4.42225 2.77519 2.73123 3.46354 2.40513 3.72494 3.29354 2.67741 2.69355 4.24690 2.90347 2.73739 3.18146 2.89801 2.37887 2.77519 2.98518 4.58477 3.61503
0.03157 3.86699 4.58934 0.61958 0.77255 0.48576 0.95510
638 2.71888 4.71528 2.70526 2.59018 4.07794 3.18029 3.91656 3.78713 2.81979 3.43461 4.38989 1.16778 3.83188 3.18930 3.17427 2.78628 3.10056 3.40989 5.38448 4.02666 638 - -
2.68618 4.42225 2.77519 2.73123 3.46354 2.40513 3.72494 3.29354 2.67741 2.69355 4.24690 2.90347 2.73739 3.18146 2.89801 2.37887 2.77519 2.98518 4.58477 3.61503
0.03157 3.86699 4.58934 0.61958 0.77255 0.48576 0.95510
639 2.96711 4.93068 3.14273 2.76522 4.31586 3.45716 3.75616 3.74479 1.04579 3.31106 4.27280 3.18993 3.95788 2.94794 2.43058 3.00945 3.22078 3.45288 5.37276 4.18474 639 - -
2.68618 4.42225 2.77519 2.73123 3.46354 2.40513 3.72494 3.29354 2.67741 2.69355 4.24690 2.90347 2.73739 3.18146 2.89801 2.37887 2.77519 2.98518 4.58477 3.61503
0.03157 3.86699 4.58934 0.61958 0.77255 0.48576 0.95510
640 3.14912 4.61302 4.18070 3.82053 3.13362 3.97519 4.47798 2.33736 3.58833 0.88196 3.15827 4.09653 4.41149 3.95797 3.76529 3.54541 3.44362 2.37328 5.01092 3.73297 640 - -
2.68618 4.42225 2.77519 2.73123 3.46354 2.40513 3.72494 3.29354 2.67741 2.69355 4.24690 2.90347 2.73739 3.18146 2.89801 2.37887 2.77519 2.98518 4.58477 3.61503
0.03157 3.86699 4.58934 0.61958 0.77255 0.48576 0.95510
641 2.31230 4.23263 3.20165 3.01716 4.07082 2.96261 4.12717 3.59269 3.09056 3.31334 4.23988 3.24282 3.70096 3.44030 3.37676 1.09934 2.79202 3.12899 5.44375 4.15432 641 - -
2.68618 4.42225 2.77519 2.73123 3.46354 2.40513 3.72494 3.29354 2.67741 2.69355 4.24690 2.90347 2.73739 3.18146 2.89801 2.37887 2.77519 2.98518 4.58477 3.61503
0.03157 3.86699 4.58934 0.61958 0.77255 0.48576 0.95510
642 2.31230 4.23263 3.20165 3.01716 4.07082 2.96261 4.12717 3.59269 3.09056 3.31334 4.23988 3.24282 3.70096 3.44030 3.37676 1.09934 2.79202 3.12899 5.44375 4.15432 642 - -
2.68618 4.42225 2.77519 2.73123 3.46354 2.40513 3.72494 3.29354 2.67741 2.69355 4.24690 2.90347 2.73739 3.18146 2.89801 2.37887 2.77519 2.98518 4.58477 3.61503
0.03157 3.86699 4.58934 0.61958 0.77255 0.48576 0.95510
643 2.58006 0.95161 4.09776 3.83614 3.82875 3.20419 4.48443 2.97175 3.66306 2.88747 4.00858 3.82459 3.91071 4.00937 3.78840 2.85170 3.04637 2.71670 5.24602 4.11647 643 - -
2.68618 4.42225 2.77519 2.73123 3.46354 2.40513 3.72494 3.29354 2.67741 2.69355 4.24690 2.90347 2.73739 3.18146 2.89801 2.37887 2.77519 2.98518 4.58477 3.61503
0.03157 3.86699 4.58934 0.61958 0.77255 0.48576 0.95510
644 2.99928 4.83880 3.48937 3.00157 4.19200 3.48833 3.82030 3.73332 2.23516 3.25025 4.25056 3.36173 3.99118 3.04089 0.98678 3.08371 3.26560 3.45751 5.27725 4.12054 644 - -
2.68618 4.42225 2.77519 2.73123 3.46354 2.40513 3.72494 3.29354 2.67741 2.69355 4.24690 2.90347 2.73739 3.18146 2.89801 2.37887 2.77519 2.98518 4.58477 3.61503
0.03157 3.86699 4.58934 0.61958 0.77255 0.48576 0.95510
645 2.98371 5.11116 0.92594 2.33150 4.48744 3.20651 3.92621 4.07436 3.00929 3.68366 4.66164 2.88760 3.85892 3.16534 3.52477 2.94668 3.32216 3.71413 5.65711 4.36124 645 - -
2.68618 4.42225 2.77519 2.73123 3.46354 2.40513 3.72494 3.29354 2.67741 2.69355 4.24690 2.90347 2.73739 3.18146 2.89801 2.37887 2.77519 2.98518 4.58477 3.61503
0.03157 3.86699 4.58934 0.61958 0.77255 0.48576 0.95510
646 3.14912 4.61302 4.18070 3.82053 3.13362 3.97519 4.47798 2.33736 3.58833 0.88196 3.15827 4.09653 4.41149 3.95797 3.76529 3.54541 3.44362 2.37328 5.01092 3.73297 646 - -
2.68618 4.42225 2.77519 2.73123 3.46354 2.40513 3.72494 3.29354 2.67741 2.69355 4.24690 2.90347 2.73739 3.18146 2.89801 2.37887 2.77519 2.98518 4.58477 3.61503
0.03157 3.86699 4.58934 0.61958 0.77255 0.48576 0.95510
647 3.25138 4.65475 4.23121 3.94466 1.02072 3.94245 3.87481 2.77574 3.85183 2.14705 3.50670 4.01301 4.40115 4.02131 3.97290 3.52160 3.55251 2.76604 4.12549 2.49318 647 - -
2.68618 4.42225 2.77519 2.73123 3.46354 2.40513 3.72494 3.29354 2.67741 2.69355 4.24690 2.90347 2.73739 3.18146 2.89801 2.37887 2.77519 2.98518 4.58477 3.61503

0.03157 3.86699 4.58934 0.61958 0.77255 0.48576 0.95510

648 3.14912 4.61302 4.18070 3.82053 3.13362 3.97519 4.47798 2.33736 3.58833 0.88196 3.15827 4.09653 4.41149 3.95797 3.76529 3.54541 3.44362 2.37328 5.01092 3.73297 648 - -

2.68618 4.42225 2.77519 2.73123 3.46354 2.40513 3.72494 3.29354 2.67741 2.69355 4.24690 2.90347 2.73739 3.18146 2.89801 2.37887 2.77519 2.98518 4.58477 3.61503

0.03157 3.86699 4.58934 0.61958 0.77255 0.48576 0.95510

649 2.31230 4.23263 3.20165 3.01716 4.07082 2.96261 4.12717 3.59269 3.09056 3.31334 4.23988 3.24282 3.70096 3.44030 3.37676 1.09934 2.79202 3.12899 5.44375 4.15432 649 - -

2.68618 4.42225 2.77519 2.73123 3.46354 2.40513 3.72494 3.29354 2.67741 2.69355 4.24690 2.90347 2.73739 3.18146 2.89801 2.37887 2.77519 2.98518 4.58477 3.61503

0.03157 3.86699 4.58934 0.61958 0.77255 0.48576 0.95510

650 2.96489 4.51452 4.04353 3.63658 3.20533 3.82998 4.37299 2.38038 3.40496 1.80083 1.38846 3.91475 4.29056 3.78719 3.61092 3.31343 3.27009 2.38586 5.04542 3.80671 650 - -

2.68618 4.42225 2.77519 2.73123 3.46354 2.40513 3.72494 3.29354 2.67741 2.69355 4.24690 2.90347 2.73739 3.18146 2.89801 2.37887 2.77519 2.98518 4.58477 3.61503

0.03157 3.86699 4.58934 0.61958 0.77255 0.48576 0.95510

651 2.96711 4.93068 3.14273 2.76522 4.31586 3.45716 3.75616 3.74479 1.04579 3.31106 4.27280 3.18993 3.95788 2.94794 2.43058 3.00945 3.22078 3.45288 5.37276 4.18474 651 - -

2.68618 4.42225 2.77519 2.73123 3.46354 2.40513 3.72494 3.29354 2.67741 2.69355 4.24690 2.90347 2.73739 3.18146 2.89801 2.37887 2.77519 2.98518 4.58477 3.61503

0.03157 3.86699 4.58934 0.61958 0.77255 0.48576 0.95510

652 2.40976 4.25482 3.42322 3.17496 3.96403 3.09429 4.19270 3.09049 3.11783 2.95418 4.00504 3.38522 3.79760 3.51292 3.37678 2.61097 1.18122 2.78606 5.40231 4.18065 652 - -

2.68618 4.42225 2.77519 2.73123 3.46354 2.40513 3.72494 3.29354 2.67741 2.69355 4.24690 2.90347 2.73739 3.18146 2.89801 2.37887 2.77519 2.98518 4.58477 3.61503

0.03157 3.86699 4.58934 0.61958 0.77255 0.48576 0.95510

653 3.02684 4.44709 4.32791 3.94350 3.37049 4.06063 4.67263 1.08471 3.77153 1.95764 3.30670 4.18960 4.49236 4.12038 3.96872 3.55539 3.32610 1.84575 5.26019 3.99802 653 - -

2.68618 4.42225 2.77519 2.73123 3.46354 2.40513 3.72494 3.29354 2.67741 2.69355 4.24690 2.90347 2.73739 3.18146 2.89801 2.37887 2.77519 2.98518 4.58477 3.61503

0.03157 3.86699 4.58934 0.61958 0.77255 0.48576 0.95510

654 3.23918 4.73039 3.83015 3.55850 2.29358 3.82376 3.61597 3.24916 3.41618 2.70450 3.92476 3.71671 4.29772 3.72354 3.60101 3.37642 3.52583 3.11945 3.93713 0.97494 654 - -

2.68618 4.42225 2.77519 2.73123 3.46354 2.40513 3.72494 3.29354 2.67741 2.69355 4.24690 2.90347 2.73739 3.18146 2.89801 2.37887 2.77519 2.98518 4.58477 3.61503

0.03157 3.86699 4.58934 0.61958 0.77255 0.48576 0.95510

655 2.98371 5.11116 0.92594 2.33150 4.48744 3.20651 3.92621 4.07436 3.00929 3.68366 4.66164 2.88760 3.85892 3.16534 3.52477 2.94668 3.32216 3.71413 5.65711 4.36124 655 - -

2.68618 4.42225 2.77519 2.73123 3.46354 2.40513 3.72494 3.29354 2.67741 2.69355 4.24690 2.90347 2.73739 3.18146 2.89801 2.37887 2.77519 2.98518 4.58477 3.61503

0.03157 3.86699 4.58934 0.61958 0.77255 0.48576 0.95510

656 3.02684 4.44709 4.32791 3.94350 3.37049 4.06063 4.67263 1.08471 3.77153 1.95764 3.30670 4.18960 4.49236 4.12038 3.96872 3.55539 3.32610 1.84575 5.26019 3.99802 656 - -

2.68618 4.42225 2.77519 2.73123 3.46354 2.40513 3.72494 3.29354 2.67741 2.69355 4.24690 2.90347 2.73739 3.18146 2.89801 2.37887 2.77519 2.98518 4.58477 3.61503

0.03157 3.86699 4.58934 0.61958 0.77255 0.48576 0.95510

657 2.94758 5.05751 2.48529 1.03318 4.40304 3.27053 3.85685 3.85586 2.73669 3.47385 4.44812 2.92979 3.87486 3.07262 3.14497 2.93053 3.25177 3.54026 5.57565 4.29788 657 - -

2.68618 4.42225 2.77519 2.73123 3.46354 2.40513 3.72494 3.29354 2.67741 2.69355 4.24690 2.90347 2.73739 3.18146 2.89801 2.37887 2.77519 2.98518 4.58477 3.61503

0.03157 3.86699 4.58934 0.61958 0.77255 0.48576 0.95510

658 2.74420 4.50247 3.35077 3.18900 4.18468 3.19029 4.26692 3.72867 3.24583 3.35611 4.40571 3.49627 0.83787 3.63478 3.49200 2.91151 3.18532 3.37467 5.37275 4.30526 658 - -

2.68618 4.42225 2.77519 2.73123 3.46354 2.40513 3.72494 3.29354 2.67741 2.69355 4.24690 2.90347 2.73739 3.18146 2.89801 2.37887 2.77519 2.98518 4.58477 3.61503

0.03157 3.86699 4.58934 0.61958 0.77255 0.48576 0.95510

659 2.40976 4.25482 3.42322 3.17496 3.96403 3.09429 4.19270 3.09049 3.11783 2.95418 4.00504 3.38522 3.79760 3.51292 3.37678 2.61097 1.18122 2.78606 5.40231 4.18065 659 - -

2.68618 4.42225 2.77519 2.73123 3.46354 2.40513 3.72494 3.29354 2.67741 2.69355 4.24690 2.90347 2.73739 3.18146 2.89801 2.37887 2.77519 2.98518 4.58477 3.61503

0.03157 3.86699 4.58934 0.61958 0.77255 0.48576 0.95510

660 2.40976 4.25482 3.42322 3.17496 3.96403 3.09429 4.19270 3.09049 3.11783 2.95418 4.00504 3.38522 3.79760 3.51292 3.37678 2.61097 1.18122 2.78606 5.40231 4.18065 660 - -

2.68618 4.42225 2.77519 2.73123 3.46354 2.40513 3.72494 3.29354 2.67741 2.69355 4.24690 2.90347 2.73739 3.18146 2.89801 2.37887 2.77519 2.98518 4.58477 3.61503

0.03157 3.86699 4.58934 0.61958 0.77255 0.48576 0.95510

661 2.94758 5.05751 2.48529 1.03318 4.40304 3.27053 3.85685 3.85586 2.73669 3.47385 4.44812 2.92979 3.87486 3.07262 3.14497 2.93053 3.25177 3.54026 5.57565 4.29788 661 - -

2.68618 4.42225 2.77519 2.73123 3.46354 2.40513 3.72494 3.29354 2.67741 2.69355 4.24690 2.90347 2.73739 3.18146 2.89801 2.37887 2.77519 2.98518 4.58477 3.61503

0.03157 3.86699 4.58934 0.61958 0.77255 0.48576 0.95510

662 2.98886 4.80727 3.12036 2.87975 3.28089 3.44287 1.15410 3.69093 2.72295 3.18558 4.22810 3.28930 3.99510 3.24591 2.98899 3.05959 3.29203 3.42616 4.74602 3.23190 662 - -

2.68618 4.42225 2.77519 2.73123 3.46354 2.40513 3.72494 3.29354 2.67741 2.69355 4.24690 2.90347 2.73739 3.18146 2.89801 2.37887 2.77519 2.98518 4.58477 3.61503

0.03157 3.86699 4.58934 0.61958 0.77255 0.48576 0.95510

663 3.23918 4.73039 3.83015 3.55850 2.29358 3.82376 3.61597 3.24916 3.41618 2.70450 3.92476 3.71671 4.29772 3.72354 3.60101 3.37642 3.52583 3.11945 3.93713 0.97494 663 - -

2.68618 4.42225 2.77519 2.73123 3.46354 2.40513 3.72494 3.29354 2.67741 2.69355 4.24690 2.90347 2.73739 3.18146 2.89801 2.37887 2.77519 2.98518 4.58477 3.61503

0.03157 3.86699 4.58934 0.61958 0.77255 0.48576 0.95510

664 2.40976 4.25482 3.42322 3.17496 3.96403 3.09429 4.19270 3.09049 3.11783 2.95418 4.00504 3.38522 3.79760 3.51292 3.37678 2.61097 1.18122 2.78606 5.40231 4.18065 664 - -

2.68618 4.42225 2.77519 2.73123 3.46354 2.40513 3.72494 3.29354 2.67741 2.69355 4.24690 2.90347 2.73739 3.18146 2.89801 2.37887 2.77519 2.98518 4.58477 3.61503

0.03157 3.86699 4.58934 0.61958 0.77255 0.48576 0.95510

665 1.02168 4.18931 3.45704 3.24808 4.04693 3.01400 4.27077 3.17162 3.26419 3.05349 4.08591 3.39907 3.75270 3.60586 3.51510 2.53965 2.81663 2.82697 5.47463 4.27427 665 - -

2.68618 4.42225 2.77519 2.73123 3.46354 2.40513 3.72494 3.29354 2.67741 2.69355 4.24690 2.90347 2.73739 3.18146 2.89801 2.37887 2.77519 2.98518 4.58477 3.61503

0.03157 3.86699 4.58934 0.61958 0.77255 0.48576 0.95510

666 3.25138 4.65475 4.23121 3.94466 1.02072 3.94245 3.87481 2.77574 3.85183 2.14705 3.50670 4.01301 4.40115 4.02131 3.97290 3.52160 3.55251 2.76604 4.12549 2.49318 666 - -

2.68618 4.42225 2.77519 2.73123 3.46354 2.40513 3.72494 3.29354 2.67741 2.69355 4.24690 2.90347 2.73739 3.18146 2.89801 2.37887 2.77519 2.98518 4.58477 3.61503

0.03157 3.86699 4.58934 0.61958 0.77255 0.48576 0.95510

667 2.80376 4.34788 4.11775 3.78070 3.54607 3.71822 4.58336 2.05053 3.64840 2.23405 3.51204 3.97141 4.27903 4.00859 3.86143 3.22141 3.16544 1.03182 5.32959 4.06087 667 - -

2.68618 4.42225 2.77519 2.73123 3.46354 2.40513 3.72494 3.29354 2.67741 2.69355 4.24690 2.90347 2.73739 3.18146 2.89801 2.37887 2.77519 2.98518 4.58477 3.61503

0.03157 3.86699 4.58934 0.61958 0.77255 0.48576 0.95510

668 2.99928 4.83880 3.48937 3.00157 4.19200 3.48833 3.82030 3.73332 2.23516 3.25025 4.25056 3.36173 3.99118 3.04089 0.98678 3.08371 3.26560 3.45751 5.27725 4.12054 668 - -

2.68618 4.42225 2.77519 2.73123 3.46354 2.40513 3.72494 3.29354 2.67741 2.69355 4.24690 2.90347 2.73739 3.18146 2.89801 2.37887 2.77519 2.98518 4.58477 3.61503

0.03157 3.86699 4.58934 0.61958 0.77255 0.48576 0.95510

669 2.80376 4.34788 4.11775 3.78070 3.54607 3.71822 4.58336 2.05053 3.64840 2.23405 3.51204 3.97141 4.27903 4.00859 3.86143 3.22141 3.16544 1.03182 5.32959 4.06087 669 - -

2.68618 4.42225 2.77519 2.73123 3.46354 2.40513 3.72494 3.29354 2.67741 2.69355 4.24690 2.90347 2.73739 3.18146 2.89801 2.37887 2.77519 2.98518 4.58477 3.61503

0.03157 3.86699 4.58934 0.61958 0.77255 0.48576 0.95510

670 3.14912 4.61302 4.18070 3.82053 3.13362 3.97519 4.47798 2.33736 3.58833 0.88196 3.15827 4.09653 4.41149 3.95797 3.76529 3.54541 3.44362 2.37328 5.01092 3.73297 670 - -

2.68618 4.42225 2.77519 2.73123 3.46354 2.40513 3.72494 3.29354 2.67741 2.69355 4.24690 2.90347 2.73739 3.18146 2.89801 2.37887 2.77519 2.98518 4.58477 3.61503

0.03157 3.86699 4.58934 0.61958 0.77255 0.48576 0.95510

671 2.65811 4.43408 3.33076 3.22947 4.37188 0.73761 4.35008 3.97016 3.41000 3.62498 4.60067 3.49506 3.82505 3.73459 3.64665 2.82986 3.13892 3.49954 5.46848 4.47359 671 - -
2.68618 4.42225 2.77519 2.73123 3.46354 2.40513 3.72494 3.29354 2.67741 2.69355 4.24690 2.90347 2.73739 3.18146 2.89801 2.37887 2.77519 2.98518 4.58477 3.61503
0.03157 3.86699 4.58934 0.61958 0.77255 0.48576 0.95510
672 2.98886 4.80727 3.12036 2.87975 3.28089 3.44287 1.15410 3.69093 2.72295 3.18558 4.22810 3.28930 3.99510 3.24591 2.98899 3.05959 3.29203 3.42616 4.74602 3.23190 672 - -
2.68618 4.42225 2.77519 2.73123 3.46354 2.40513 3.72494 3.29354 2.67741 2.69355 4.24690 2.90347 2.73739 3.18146 2.89801 2.37887 2.77519 2.98518 4.58477 3.61503
0.03157 3.86699 4.58934 0.61958 0.77255 0.48576 0.95510
673 3.32650 4.70314 4.08279 3.78752 2.66069 3.72402 3.89311 3.34986 3.50985 2.74569 3.98391 3.93769 4.24129 3.89775 3.63917 3.53025 3.62662 3.24004 0.89641 2.66307 673 - -
2.68618 4.42225 2.77519 2.73123 3.46354 2.40513 3.72494 3.29354 2.67741 2.69355 4.24690 2.90347 2.73739 3.18146 2.89801 2.37887 2.77519 2.98518 4.58477 3.61503
0.03157 3.86699 4.58934 0.61958 0.77255 0.48576 0.95510
674 2.65811 4.43408 3.33076 3.22947 4.37188 0.73761 4.35008 3.97016 3.41000 3.62498 4.60067 3.49506 3.82505 3.73459 3.64665 2.82986 3.13892 3.49954 5.46848 4.47359 674 - -
2.68618 4.42225 2.77519 2.73123 3.46354 2.40513 3.72494 3.29354 2.67741 2.69355 4.24690 2.90347 2.73739 3.18146 2.89801 2.37887 2.77519 2.98518 4.58477 3.61503
0.03157 3.86699 4.58934 0.61958 0.77255 0.48576 0.95510
675 3.14912 4.61302 4.18070 3.82053 3.13362 3.97519 4.47798 2.33736 3.58833 0.88196 3.15827 4.09653 4.41149 3.95797 3.76529 3.54541 3.44362 2.37328 5.01092 3.73297 675 - -
2.68618 4.42225 2.77519 2.73123 3.46354 2.40513 3.72494 3.29354 2.67741 2.69355 4.24690 2.90347 2.73739 3.18146 2.89801 2.37887 2.77519 2.98518 4.58477 3.61503
0.03157 3.86699 4.58934 0.61958 0.77255 0.48576 0.95510
676 3.14912 4.61302 4.18070 3.82053 3.13362 3.97519 4.47798 2.33736 3.58833 0.88196 3.15827 4.09653 4.41149 3.95797 3.76529 3.54541 3.44362 2.37328 5.01092 3.73297 676 - -
2.68618 4.42225 2.77519 2.73123 3.46354 2.40513 3.72494 3.29354 2.67741 2.69355 4.24690 2.90347 2.73739 3.18146 2.89801 2.37887 2.77519 2.98518 4.58477 3.61503
0.03157 3.86699 4.58934 0.61958 0.77255 0.48576 0.95510
677 2.94758 5.05751 2.48529 1.03318 4.40304 3.27053 3.85685 3.85586 2.73669 3.47385 4.44812 2.92979 3.87486 3.07262 3.14497 2.93053 3.25177 3.54026 5.57565 4.29788 677 - -
2.68618 4.42225 2.77519 2.73123 3.46354 2.40513 3.72494 3.29354 2.67741 2.69355 4.24690 2.90347 2.73739 3.18146 2.89801 2.37887 2.77519 2.98518 4.58477 3.61503
0.03157 3.86699 4.58934 0.61958 0.77255 0.48576 0.95510
678 2.94758 5.05751 2.48529 1.03318 4.40304 3.27053 3.85685 3.85586 2.73669 3.47385 4.44812 2.92979 3.87486 3.07262 3.14497 2.93053 3.25177 3.54026 5.57565 4.29788 678 - -
2.68618 4.42225 2.77519 2.73123 3.46354 2.40513 3.72494 3.29354 2.67741 2.69355 4.24690 2.90347 2.73739 3.18146 2.89801 2.37887 2.77519 2.98518 4.58477 3.61503
0.03157 3.86699 4.58934 0.61958 0.77255 0.48576 0.95510
679 1.02168 4.18931 3.45704 3.24808 4.04693 3.01400 4.27077 3.17162 3.26419 3.05349 4.08591 3.39907 3.75270 3.60586 3.51510 2.53965 2.81663 2.82697 5.47463 4.27427 679 - -
2.68618 4.42225 2.77519 2.73123 3.46354 2.40513 3.72494 3.29354 2.67741 2.69355 4.24690 2.90347 2.73739 3.18146 2.89801 2.37887 2.77519 2.98518 4.58477 3.61503
0.03157 3.86699 4.58934 0.61958 0.77255 0.48576 0.95510
680 2.94758 5.05751 2.48529 1.03318 4.40304 3.27053 3.85685 3.85586 2.73669 3.47385 4.44812 2.92979 3.87486 3.07262 3.14497 2.93053 3.25177 3.54026 5.57565 4.29788 680 - -
2.68618 4.42225 2.77519 2.73123 3.46354 2.40513 3.72494 3.29354 2.67741 2.69355 4.24690 2.90347 2.73739 3.18146 2.89801 2.37887 2.77519 2.98518 4.58477 3.61503
0.03157 3.86699 4.58934 0.61958 0.77255 0.48576 0.95510
681 2.98371 5.11116 0.92594 2.33150 4.48744 3.20651 3.92621 4.07436 3.00929 3.68366 4.66164 2.88760 3.85892 3.16534 3.52477 2.94668 3.32216 3.71413 5.65711 4.36124 681 - -
2.68618 4.42225 2.77519 2.73123 3.46354 2.40513 3.72494 3.29354 2.67741 2.69355 4.24690 2.90347 2.73739 3.18146 2.89801 2.37887 2.77519 2.98518 4.58477 3.61503
0.03157 3.86699 4.58934 0.61958 0.77255 0.48576 0.95510
682 2.40976 4.25482 3.42322 3.17496 3.96403 3.09429 4.19270 3.09049 3.11783 2.95418 4.00504 3.38522 3.79760 3.51292 3.37678 2.61097 1.18122 2.78606 5.40231 4.18065 682 - -
2.68618 4.42225 2.77519 2.73123 3.46354 2.40513 3.72494 3.29354 2.67741 2.69355 4.24690 2.90347 2.73739 3.18146 2.89801 2.37887 2.77519 2.98518 4.58477 3.61503

0.03157 3.86699 4.58934 0.61958 0.77255 0.48576 0.95510

683 3.02684 4.44709 4.32791 3.94350 3.37049 4.06063 4.67263 1.08471 3.77153 1.95764 3.30670 4.18960 4.49236 4.12038 3.96872 3.55539 3.32610 1.84575 5.26019 3.99802 683 - -

2.68618 4.42225 2.77519 2.73123 3.46354 2.40513 3.72494 3.29354 2.67741 2.69355 4.24690 2.90347 2.73739 3.18146 2.89801 2.37887 2.77519 2.98518 4.58477 3.61503

0.03157 3.86699 4.58934 0.61958 0.77255 0.48576 0.95510

684 2.71888 4.71528 2.70526 2.59018 4.07794 3.18029 3.91656 3.78713 2.81979 3.43461 4.38989 1.16778 3.83188 3.18930 3.17427 2.78628 3.10056 3.40989 5.38448 4.02666 684 - -

2.68618 4.42225 2.77519 2.73123 3.46354 2.40513 3.72494 3.29354 2.67741 2.69355 4.24690 2.90347 2.73739 3.18146 2.89801 2.37887 2.77519 2.98518 4.58477 3.61503

0.03157 3.86699 4.58934 0.61958 0.77255 0.48576 0.95510

685 2.31230 4.23263 3.20165 3.01716 4.07082 2.96261 4.12717 3.59269 3.09056 3.31334 4.23988 3.24282 3.70096 3.44030 3.37676 1.09934 2.79202 3.12899 5.44375 4.15432 685 - -

2.68618 4.42225 2.77519 2.73123 3.46354 2.40513 3.72494 3.29354 2.67741 2.69355 4.24690 2.90347 2.73739 3.18146 2.89801 2.37887 2.77519 2.98518 4.58477 3.61503

0.03157 3.86699 4.58934 0.61958 0.77255 0.48576 0.95510

686 2.96489 4.51452 4.04353 3.63658 3.20533 3.82998 4.37299 2.38038 3.40496 1.80083 1.38846 3.91475 4.29056 3.78719 3.61092 3.31343 3.27009 2.38586 5.04542 3.80671 686 - -

2.68618 4.42225 2.77519 2.73123 3.46354 2.40513 3.72494 3.29354 2.67741 2.69355 4.24690 2.90347 2.73739 3.18146 2.89801 2.37887 2.77519 2.98518 4.58477 3.61503

0.03157 3.86699 4.58934 0.61958 0.77255 0.48576 0.95510

687 2.74420 4.50247 3.35077 3.18900 4.18468 3.19029 4.26692 3.72867 3.24583 3.35611 4.40571 3.49627 0.83787 3.63478 3.49200 2.91151 3.18532 3.37467 5.37275 4.30526 687 - -

2.68618 4.42225 2.77519 2.73123 3.46354 2.40513 3.72494 3.29354 2.67741 2.69355 4.24690 2.90347 2.73739 3.18146 2.89801 2.37887 2.77519 2.98518 4.58477 3.61503

0.03157 3.86699 4.58934 0.61958 0.77255 0.48576 0.95510

688 2.80376 4.34788 4.11775 3.78070 3.54607 3.71822 4.58336 2.05053 3.64840 2.23405 3.51204 3.97141 4.27903 4.00859 3.86143 3.22141 3.16544 1.03182 5.32959 4.06087 688 - -

2.68618 4.42225 2.77519 2.73123 3.46354 2.40513 3.72494 3.29354 2.67741 2.69355 4.24690 2.90347 2.73739 3.18146 2.89801 2.37887 2.77519 2.98518 4.58477 3.61503

0.03157 3.86699 4.58934 0.61958 0.77255 0.48576 0.95510

689 2.87640 4.86196 2.89930 2.65395 4.02980 3.36241 3.81221 3.65971 2.47512 3.16261 4.19062 3.11364 3.91579 1.24381 2.75940 2.92438 3.17337 3.38973 5.30278 3.99617 689 - -

2.68618 4.42225 2.77519 2.73123 3.46354 2.40513 3.72494 3.29354 2.67741 2.69355 4.24690 2.90347 2.73739 3.18146 2.89801 2.37887 2.77519 2.98518 4.58477 3.61503

0.03157 3.86699 4.58934 0.61958 0.77255 0.48576 0.95510

690 2.74420 4.50247 3.35077 3.18900 4.18468 3.19029 4.26692 3.72867 3.24583 3.35611 4.40571 3.49627 0.83787 3.63478 3.49200 2.91151 3.18532 3.37467 5.37275 4.30526 690 - -

2.68618 4.42225 2.77519 2.73123 3.46354 2.40513 3.72494 3.29354 2.67741 2.69355 4.24690 2.90347 2.73739 3.18146 2.89801 2.37887 2.77519 2.98518 4.58477 3.61503

0.03157 3.86699 4.58934 0.61958 0.77255 0.48576 0.95510

691 2.94758 5.05751 2.48529 1.03318 4.40304 3.27053 3.85685 3.85586 2.73669 3.47385 4.44812 2.92979 3.87486 3.07262 3.14497 2.93053 3.25177 3.54026 5.57565 4.29788 691 - -

2.68618 4.42225 2.77519 2.73123 3.46354 2.40513 3.72494 3.29354 2.67741 2.69355 4.24690 2.90347 2.73739 3.18146 2.89801 2.37887 2.77519 2.98518 4.58477 3.61503

0.03157 3.86699 4.58934 0.61958 0.77255 0.48576 0.95510

692 2.80376 4.34788 4.11775 3.78070 3.54607 3.71822 4.58336 2.05053 3.64840 2.23405 3.51204 3.97141 4.27903 4.00859 3.86143 3.22141 3.16544 1.03182 5.32959 4.06087 692 - -

2.68618 4.42225 2.77519 2.73123 3.46354 2.40513 3.72494 3.29354 2.67741 2.69355 4.24690 2.90347 2.73739 3.18146 2.89801 2.37887 2.77519 2.98518 4.58477 3.61503

0.03157 3.86699 4.58934 0.61958 0.77255 0.48576 0.95510

693 2.31230 4.23263 3.20165 3.01716 4.07082 2.96261 4.12717 3.59269 3.09056 3.31334 4.23988 3.24282 3.70096 3.44030 3.37676 1.09934 2.79202 3.12899 5.44375 4.15432 693 - -

2.68618 4.42225 2.77519 2.73123 3.46354 2.40513 3.72494 3.29354 2.67741 2.69355 4.24690 2.90347 2.73739 3.18146 2.89801 2.37887 2.77519 2.98518 4.58477 3.61503

0.03157 3.86699 4.58934 0.61958 0.77255 0.48576 0.95510

694 2.80376 4.34788 4.11775 3.78070 3.54607 3.71822 4.58336 2.05053 3.64840 2.23405 3.51204 3.97141 4.27903 4.00859 3.86143 3.22141 3.16544 1.03182 5.32959 4.06087 694 - -

2.68618 4.42225 2.77519 2.73123 3.46354 2.40513 3.72494 3.29354 2.67741 2.69355 4.24690 2.90347 2.73739 3.18146 2.89801 2.37887 2.77519 2.98518 4.58477 3.61503
0.03157 3.86699 4.58934 0.61958 0.77255 0.48576 0.95510
695 3.14912 4.61302 4.18070 3.82053 3.13362 3.97519 4.47798 2.33736 3.58833 0.88196 3.15827 4.09653 4.41149 3.95797 3.76529 3.54541 3.44362 2.37328 5.01092 3.73297 695 - -
2.68618 4.42225 2.77519 2.73123 3.46354 2.40513 3.72494 3.29354 2.67741 2.69355 4.24690 2.90347 2.73739 3.18146 2.89801 2.37887 2.77519 2.98518 4.58477 3.61503
0.03157 3.86699 4.58934 0.61958 0.77255 0.48576 0.95510
696 2.99928 4.83880 3.48937 3.00157 4.19200 3.48833 3.82030 3.73332 2.23516 3.25025 4.25056 3.36173 3.99118 3.04089 0.98678 3.08371 3.26560 3.45751 5.27725 4.12054 696 - -
2.68618 4.42225 2.77519 2.73123 3.46354 2.40513 3.72494 3.29354 2.67741 2.69355 4.24690 2.90347 2.73739 3.18146 2.89801 2.37887 2.77519 2.98518 4.58477 3.61503
0.03157 3.86699 4.58934 0.61958 0.77255 0.48576 0.95510
697 1.02168 4.18931 3.45704 3.24808 4.04693 3.01400 4.27077 3.17162 3.26419 3.05349 4.08591 3.39907 3.75270 3.60586 3.51510 2.53965 2.81663 2.82697 5.47463 4.27427 697 - -
2.68618 4.42225 2.77519 2.73123 3.46354 2.40513 3.72494 3.29354 2.67741 2.69355 4.24690 2.90347 2.73739 3.18146 2.89801 2.37887 2.77519 2.98518 4.58477 3.61503
0.03157 3.86699 4.58934 0.61958 0.77255 0.48576 0.95510
698 3.14912 4.61302 4.18070 3.82053 3.13362 3.97519 4.47798 2.33736 3.58833 0.88196 3.15827 4.09653 4.41149 3.95797 3.76529 3.54541 3.44362 2.37328 5.01092 3.73297 698 - -
2.68618 4.42225 2.77519 2.73123 3.46354 2.40513 3.72494 3.29354 2.67741 2.69355 4.24690 2.90347 2.73739 3.18146 2.89801 2.37887 2.77519 2.98518 4.58477 3.61503
0.03157 3.86699 4.58934 0.61958 0.77255 0.48576 0.95510
699 3.14912 4.61302 4.18070 3.82053 3.13362 3.97519 4.47798 2.33736 3.58833 0.88196 3.15827 4.09653 4.41149 3.95797 3.76529 3.54541 3.44362 2.37328 5.01092 3.73297 699 - -
2.68618 4.42225 2.77519 2.73123 3.46354 2.40513 3.72494 3.29354 2.67741 2.69355 4.24690 2.90347 2.73739 3.18146 2.89801 2.37887 2.77519 2.98518 4.58477 3.61503
0.03157 3.86699 4.58934 0.61958 0.77255 0.48576 0.95510
700 2.98371 5.11116 0.92594 2.33150 4.48744 3.20651 3.92621 4.07436 3.00929 3.68366 4.66164 2.88760 3.85892 3.16534 3.52477 2.94668 3.32216 3.71413 5.65711 4.36124 700 - -
2.68618 4.42225 2.77519 2.73123 3.46354 2.40513 3.72494 3.29354 2.67741 2.69355 4.24690 2.90347 2.73739 3.18146 2.89801 2.37887 2.77519 2.98518 4.58477 3.61503
0.03157 3.86699 4.58934 0.61958 0.77255 0.48576 0.95510
701 2.31230 4.23263 3.20165 3.01716 4.07082 2.96261 4.12717 3.59269 3.09056 3.31334 4.23988 3.24282 3.70096 3.44030 3.37676 1.09934 2.79202 3.12899 5.44375 4.15432 701 - -
2.68618 4.42225 2.77519 2.73123 3.46354 2.40513 3.72494 3.29354 2.67741 2.69355 4.24690 2.90347 2.73739 3.18146 2.89801 2.37887 2.77519 2.98518 4.58477 3.61503
0.03157 3.86699 4.58934 0.61958 0.77255 0.48576 0.95510
702 2.58006 0.95161 4.09776 3.83614 3.82875 3.20419 4.48443 2.97175 3.66306 2.88747 4.00858 3.82459 3.91071 4.00937 3.78840 2.85170 3.04637 2.71670 5.24602 4.11647 702 - -
2.68618 4.42225 2.77519 2.73123 3.46354 2.40513 3.72494 3.29354 2.67741 2.69355 4.24690 2.90347 2.73739 3.18146 2.89801 2.37887 2.77519 2.98518 4.58477 3.61503
0.03157 3.86699 4.58934 0.61958 0.77255 0.48576 0.95510
703 2.99928 4.83880 3.48937 3.00157 4.19200 3.48833 3.82030 3.73332 2.23516 3.25025 4.25056 3.36173 3.99118 3.04089 0.98678 3.08371 3.26560 3.45751 5.27725 4.12054 703 - -
2.68618 4.42225 2.77519 2.73123 3.46354 2.40513 3.72494 3.29354 2.67741 2.69355 4.24690 2.90347 2.73739 3.18146 2.89801 2.37887 2.77519 2.98518 4.58477 3.61503
0.03157 3.86699 4.58934 0.61958 0.77255 0.48576 0.95510
704 3.02684 4.44709 4.32791 3.94350 3.37049 4.06063 4.67263 1.08471 3.77153 1.95764 3.30670 4.18960 4.49236 4.12038 3.96872 3.55539 3.32610 1.84575 5.26019 3.99802 704 - -
2.68618 4.42225 2.77519 2.73123 3.46354 2.40513 3.72494 3.29354 2.67741 2.69355 4.24690 2.90347 2.73739 3.18146 2.89801 2.37887 2.77519 2.98518 4.58477 3.61503
0.03157 3.86699 4.58934 0.61958 0.77255 0.48576 0.95510
705 2.98886 4.80727 3.12036 2.87975 3.28089 3.44287 1.15410 3.69093 2.72295 3.18558 4.22810 3.28930 3.99510 3.24591 2.98899 3.05959 3.29203 3.42616 4.74602 3.23190 705 - -
2.68618 4.42225 2.77519 2.73123 3.46354 2.40513 3.72494 3.29354 2.67741 2.69355 4.24690 2.90347 2.73739 3.18146 2.89801 2.37887 2.77519 2.98518 4.58477 3.61503
0.03157 3.86699 4.58934 0.61958 0.77255 0.48576 0.95510

706 2.31230 4.23263 3.20165 3.01716 4.07082 2.96261 4.12717 3.59269 3.09056 3.31334 4.23988 3.24282 3.70096 3.44030 3.37676 1.09934 2.79202 3.12899 5.44375 4.15432 706 - -
2.68618 4.42225 2.77519 2.73123 3.46354 2.40513 3.72494 3.29354 2.67741 2.69355 4.24690 2.90347 2.73739 3.18146 2.89801 2.37887 2.77519 2.98518 4.58477 3.61503
0.03157 3.86699 4.58934 0.61958 0.77255 0.48576 0.95510
707 2.71888 4.71528 2.70526 2.59018 4.07794 3.18029 3.91656 3.78713 2.81979 3.43461 4.38989 1.16778 3.83188 3.18930 3.17427 2.78628 3.10056 3.40989 5.38448 4.02666 707 - -
2.68618 4.42225 2.77519 2.73123 3.46354 2.40513 3.72494 3.29354 2.67741 2.69355 4.24690 2.90347 2.73739 3.18146 2.89801 2.37887 2.77519 2.98518 4.58477 3.61503
0.03157 3.86699 4.58934 0.61958 0.77255 0.48576 0.95510
708 2.40976 4.25482 3.42322 3.17496 3.96403 3.09429 4.19270 3.09049 3.11783 2.95418 4.00504 3.38522 3.79760 3.51292 3.37678 2.61097 1.18122 2.78606 5.40231 4.18065 708 - -
2.68618 4.42225 2.77519 2.73123 3.46354 2.40513 3.72494 3.29354 2.67741 2.69355 4.24690 2.90347 2.73739 3.18146 2.89801 2.37887 2.77519 2.98518 4.58477 3.61503
0.03157 3.86699 4.58934 0.61958 0.77255 0.48576 0.95510
709 2.31230 4.23263 3.20165 3.01716 4.07082 2.96261 4.12717 3.59269 3.09056 3.31334 4.23988 3.24282 3.70096 3.44030 3.37676 1.09934 2.79202 3.12899 5.44375 4.15432 709 - -
2.68618 4.42225 2.77519 2.73123 3.46354 2.40513 3.72494 3.29354 2.67741 2.69355 4.24690 2.90347 2.73739 3.18146 2.89801 2.37887 2.77519 2.98518 4.58477 3.61503
0.03157 3.86699 4.58934 0.61958 0.77255 0.48576 0.95510
710 2.80376 4.34788 4.11775 3.78070 3.54607 3.71822 4.58336 2.05053 3.64840 2.23405 3.51204 3.97141 4.27903 4.00859 3.86143 3.22141 3.16544 1.03182 5.32959 4.06087 710 - -
2.68618 4.42225 2.77519 2.73123 3.46354 2.40513 3.72494 3.29354 2.67741 2.69355 4.24690 2.90347 2.73739 3.18146 2.89801 2.37887 2.77519 2.98518 4.58477 3.61503
0.03157 3.86699 4.58934 0.61958 0.77255 0.48576 0.95510
711 1.02168 4.18931 3.45704 3.24808 4.04693 3.01400 4.27077 3.17162 3.26419 3.05349 4.08591 3.39907 3.75270 3.60586 3.51510 2.53965 2.81663 2.82697 5.47463 4.27427 711 - -
2.68618 4.42225 2.77519 2.73123 3.46354 2.40513 3.72494 3.29354 2.67741 2.69355 4.24690 2.90347 2.73739 3.18146 2.89801 2.37887 2.77519 2.98518 4.58477 3.61503
0.03157 3.86699 4.58934 0.61958 0.77255 0.48576 0.95510
712 2.96711 4.93068 3.14273 2.76522 4.31586 3.45716 3.75616 3.74479 1.04579 3.31106 4.27280 3.18993 3.95788 2.94794 2.43058 3.00945 3.22078 3.45288 5.37276 4.18474 712 - -
2.68618 4.42225 2.77519 2.73123 3.46354 2.40513 3.72494 3.29354 2.67741 2.69355 4.24690 2.90347 2.73739 3.18146 2.89801 2.37887 2.77519 2.98518 4.58477 3.61503
0.03157 3.86699 4.58934 0.61958 0.77255 0.48576 0.95510
713 2.99928 4.83880 3.48937 3.00157 4.19200 3.48833 3.82030 3.73332 2.23516 3.25025 4.25056 3.36173 3.99118 3.04089 0.98678 3.08371 3.26560 3.45751 5.27725 4.12054 713 - -
2.68618 4.42225 2.77519 2.73123 3.46354 2.40513 3.72494 3.29354 2.67741 2.69355 4.24690 2.90347 2.73739 3.18146 2.89801 2.37887 2.77519 2.98518 4.58477 3.61503
0.03157 3.86699 4.58934 0.61958 0.77255 0.48576 0.95510
714 2.80376 4.34788 4.11775 3.78070 3.54607 3.71822 4.58336 2.05053 3.64840 2.23405 3.51204 3.97141 4.27903 4.00859 3.86143 3.22141 3.16544 1.03182 5.32959 4.06087 714 - -
2.68618 4.42225 2.77519 2.73123 3.46354 2.40513 3.72494 3.29354 2.67741 2.69355 4.24690 2.90347 2.73739 3.18146 2.89801 2.37887 2.77519 2.98518 4.58477 3.61503
0.03157 3.86699 4.58934 0.61958 0.77255 0.48576 0.95510
715 1.02168 4.18931 3.45704 3.24808 4.04693 3.01400 4.27077 3.17162 3.26419 3.05349 4.08591 3.39907 3.75270 3.60586 3.51510 2.53965 2.81663 2.82697 5.47463 4.27427 715 - -
2.68618 4.42225 2.77519 2.73123 3.46354 2.40513 3.72494 3.29354 2.67741 2.69355 4.24690 2.90347 2.73739 3.18146 2.89801 2.37887 2.77519 2.98518 4.58477 3.61503
0.03157 3.86699 4.58934 0.61958 0.77255 0.48576 0.95510
716 2.96711 4.93068 3.14273 2.76522 4.31586 3.45716 3.75616 3.74479 1.04579 3.31106 4.27280 3.18993 3.95788 2.94794 2.43058 3.00945 3.22078 3.45288 5.37276 4.18474 716 - -
2.68618 4.42225 2.77519 2.73123 3.46354 2.40513 3.72494 3.29354 2.67741 2.69355 4.24690 2.90347 2.73739 3.18146 2.89801 2.37887 2.77519 2.98518 4.58477 3.61503
0.03157 3.86699 4.58934 0.61958 0.77255 0.48576 0.95510
717 3.14912 4.61302 4.18070 3.82053 3.13362 3.97519 4.47798 2.33736 3.58833 0.88196 3.15827 4.09653 4.41149 3.95797 3.76529 3.54541 3.44362 2.37328 5.01092 3.73297 717 - -
2.68618 4.42225 2.77519 2.73123 3.46354 2.40513 3.72494 3.29354 2.67741 2.69355 4.24690 2.90347 2.73739 3.18146 2.89801 2.37887 2.77519 2.98518 4.58477 3.61503

0.03157 3.86699 4.58934 0.61958 0.77255 0.48576 0.95510
718 3.02684 4.44709 4.32791 3.94350 3.37049 4.06063 4.67263 1.08471 3.77153 1.95764 3.30670 4.18960 4.49236 4.12038 3.96872 3.55539 3.32610 1.84575 5.26019 3.99802 718 - -
2.68618 4.42225 2.77519 2.73123 3.46354 2.40513 3.72494 3.29354 2.67741 2.69355 4.24690 2.90347 2.73739 3.18146 2.89801 2.37887 2.77519 2.98518 4.58477 3.61503
0.03157 3.86699 4.58934 0.61958 0.77255 0.48576 0.95510
719 3.14912 4.61302 4.18070 3.82053 3.13362 3.97519 4.47798 2.33736 3.58833 0.88196 3.15827 4.09653 4.41149 3.95797 3.76529 3.54541 3.44362 2.37328 5.01092 3.73297 719 - -
2.68618 4.42225 2.77519 2.73123 3.46354 2.40513 3.72494 3.29354 2.67741 2.69355 4.24690 2.90347 2.73739 3.18146 2.89801 2.37887 2.77519 2.98518 4.58477 3.61503
0.03157 3.86699 4.58934 0.61958 0.77255 0.48576 0.95510
720 2.31230 4.23263 3.20165 3.01716 4.07082 2.96261 4.12717 3.59269 3.09056 3.31334 4.23988 3.24282 3.70096 3.44030 3.37676 1.09934 2.79202 3.12899 5.44375 4.15432 720 - -
2.68618 4.42225 2.77519 2.73123 3.46354 2.40513 3.72494 3.29354 2.67741 2.69355 4.24690 2.90347 2.73739 3.18146 2.89801 2.37887 2.77519 2.98518 4.58477 3.61503
0.03157 3.86699 4.58934 0.61958 0.77255 0.48576 0.95510
721 2.40976 4.25482 3.42322 3.17496 3.96403 3.09429 4.19270 3.09049 3.11783 2.95418 4.00504 3.38522 3.79760 3.51292 3.37678 2.61097 1.18122 2.78606 5.40231 4.18065 721 - -
2.68618 4.42225 2.77519 2.73123 3.46354 2.40513 3.72494 3.29354 2.67741 2.69355 4.24690 2.90347 2.73739 3.18146 2.89801 2.37887 2.77519 2.98518 4.58477 3.61503
0.03157 3.86699 4.58934 0.61958 0.77255 0.48576 0.95510
722 2.96711 4.93068 3.14273 2.76522 4.31586 3.45716 3.75616 3.74479 1.04579 3.31106 4.27280 3.18993 3.95788 2.94794 2.43058 3.00945 3.22078 3.45288 5.37276 4.18474 722 - -
2.68618 4.42225 2.77519 2.73123 3.46354 2.40513 3.72494 3.29354 2.67741 2.69355 4.24690 2.90347 2.73739 3.18146 2.89801 2.37887 2.77519 2.98518 4.58477 3.61503
0.03157 3.86699 4.58934 0.61958 0.77255 0.48576 0.95510
723 2.74420 4.50247 3.35077 3.18900 4.18468 3.19029 4.26692 3.72867 3.24583 3.35611 4.40571 3.49627 0.83787 3.63478 3.49200 2.91151 3.18532 3.37467 5.37275 4.30526 723 - -
2.68618 4.42225 2.77519 2.73123 3.46354 2.40513 3.72494 3.29354 2.67741 2.69355 4.24690 2.90347 2.73739 3.18146 2.89801 2.37887 2.77519 2.98518 4.58477 3.61503
0.03157 3.86699 4.58934 0.61958 0.77255 0.48576 0.95510
724 2.94758 5.05751 2.48529 1.03318 4.40304 3.27053 3.85685 3.85586 2.73669 3.47385 4.44812 2.92979 3.87486 3.07262 3.14497 2.93053 3.25177 3.54026 5.57565 4.29788 724 - -
2.68618 4.42225 2.77519 2.73123 3.46354 2.40513 3.72494 3.29354 2.67741 2.69355 4.24690 2.90347 2.73739 3.18146 2.89801 2.37887 2.77519 2.98518 4.58477 3.61503
0.03157 3.86699 4.58934 0.61958 0.77255 0.48576 0.95510
725 2.40976 4.25482 3.42322 3.17496 3.96403 3.09429 4.19270 3.09049 3.11783 2.95418 4.00504 3.38522 3.79760 3.51292 3.37678 2.61097 1.18122 2.78606 5.40231 4.18065 725 - -
2.68618 4.42225 2.77519 2.73123 3.46354 2.40513 3.72494 3.29354 2.67741 2.69355 4.24690 2.90347 2.73739 3.18146 2.89801 2.37887 2.77519 2.98518 4.58477 3.61503
0.03157 3.86699 4.58934 0.61958 0.77255 0.48576 0.95510
726 2.74420 4.50247 3.35077 3.18900 4.18468 3.19029 4.26692 3.72867 3.24583 3.35611 4.40571 3.49627 0.83787 3.63478 3.49200 2.91151 3.18532 3.37467 5.37275 4.30526 726 - -
2.68618 4.42225 2.77519 2.73123 3.46354 2.40513 3.72494 3.29354 2.67741 2.69355 4.24690 2.90347 2.73739 3.18146 2.89801 2.37887 2.77519 2.98518 4.58477 3.61503
0.03157 3.86699 4.58934 0.61958 0.77255 0.48576 0.95510
727 2.31230 4.23263 3.20165 3.01716 4.07082 2.96261 4.12717 3.59269 3.09056 3.31334 4.23988 3.24282 3.70096 3.44030 3.37676 1.09934 2.79202 3.12899 5.44375 4.15432 727 - -
2.68618 4.42225 2.77519 2.73123 3.46354 2.40513 3.72494 3.29354 2.67741 2.69355 4.24690 2.90347 2.73739 3.18146 2.89801 2.37887 2.77519 2.98518 4.58477 3.61503
0.03157 3.86699 4.58934 0.61958 0.77255 0.48576 0.95510
728 2.94758 5.05751 2.48529 1.03318 4.40304 3.27053 3.85685 3.85586 2.73669 3.47385 4.44812 2.92979 3.87486 3.07262 3.14497 2.93053 3.25177 3.54026 5.57565 4.29788 728 - -
2.68618 4.42225 2.77519 2.73123 3.46354 2.40513 3.72494 3.29354 2.67741 2.69355 4.24690 2.90347 2.73739 3.18146 2.89801 2.37887 2.77519 2.98518 4.58477 3.61503
0.03157 3.86699 4.58934 0.61958 0.77255 0.48576 0.95510
729 3.23918 4.73039 3.83015 3.55850 2.29358 3.82376 3.61597 3.24916 3.41618 2.70450 3.92476 3.71671 4.29772 3.72354 3.60101 3.37642 3.52583 3.11945 3.93713 0.97494 729 - -

2.68618 4.42225 2.77519 2.73123 3.46354 2.40513 3.72494 3.29354 2.67741 2.69355 4.24690 2.90347 2.73739 3.18146 2.89801 2.37887 2.77519 2.98518 4.58477 3.61503
0.03157 3.86699 4.58934 0.61958 0.77255 0.48576 0.95510
730 3.02684 4.44709 4.32791 3.94350 3.37049 4.06063 4.67263 1.08471 3.77153 1.95764 3.30670 4.18960 4.49236 4.12038 3.96872 3.55539 3.32610 1.84575 5.26019 3.99802 730 - -
2.68618 4.42225 2.77519 2.73123 3.46354 2.40513 3.72494 3.29354 2.67741 2.69355 4.24690 2.90347 2.73739 3.18146 2.89801 2.37887 2.77519 2.98518 4.58477 3.61503
0.03157 3.86699 4.58934 0.61958 0.77255 0.48576 0.95510
731 3.14912 4.61302 4.18070 3.82053 3.13362 3.97519 4.47798 2.33736 3.58833 0.88196 3.15827 4.09653 4.41149 3.95797 3.76529 3.54541 3.44362 2.37328 5.01092 3.73297 731 - -
2.68618 4.42225 2.77519 2.73123 3.46354 2.40513 3.72494 3.29354 2.67741 2.69355 4.24690 2.90347 2.73739 3.18146 2.89801 2.37887 2.77519 2.98518 4.58477 3.61503
0.03157 3.86699 4.58934 0.61958 0.77255 0.48576 0.95510
732 2.96711 4.93068 3.14273 2.76522 4.31586 3.45716 3.75616 3.74479 1.04579 3.31106 4.27280 3.18993 3.95788 2.94794 2.43058 3.00945 3.22078 3.45288 5.37276 4.18474 732 - -
2.68618 4.42225 2.77519 2.73123 3.46354 2.40513 3.72494 3.29354 2.67741 2.69355 4.24690 2.90347 2.73739 3.18146 2.89801 2.37887 2.77519 2.98518 4.58477 3.61503
0.03157 3.86699 4.58934 0.61958 0.77255 0.48576 0.95510
733 2.31230 4.23263 3.20165 3.01716 4.07082 2.96261 4.12717 3.59269 3.09056 3.31334 4.23988 3.24282 3.70096 3.44030 3.37676 1.09934 2.79202 3.12899 5.44375 4.15432 733 - -
2.68618 4.42225 2.77519 2.73123 3.46354 2.40513 3.72494 3.29354 2.67741 2.69355 4.24690 2.90347 2.73739 3.18146 2.89801 2.37887 2.77519 2.98518 4.58477 3.61503
0.03157 3.86699 4.58934 0.61958 0.77255 0.48576 0.95510
734 2.71888 4.71528 2.70526 2.59018 4.07794 3.18029 3.91656 3.78713 2.81979 3.43461 4.38989 1.16778 3.83188 3.18930 3.17427 2.78628 3.10056 3.40989 5.38448 4.02666 734 - -
2.68618 4.42225 2.77519 2.73123 3.46354 2.40513 3.72494 3.29354 2.67741 2.69355 4.24690 2.90347 2.73739 3.18146 2.89801 2.37887 2.77519 2.98518 4.58477 3.61503
0.03157 3.86699 4.58934 0.61958 0.77255 0.48576 0.95510
735 3.02684 4.44709 4.32791 3.94350 3.37049 4.06063 4.67263 1.08471 3.77153 1.95764 3.30670 4.18960 4.49236 4.12038 3.96872 3.55539 3.32610 1.84575 5.26019 3.99802 735 - -
2.68618 4.42225 2.77519 2.73123 3.46354 2.40513 3.72494 3.29354 2.67741 2.69355 4.24690 2.90347 2.73739 3.18146 2.89801 2.37887 2.77519 2.98518 4.58477 3.61503
0.03157 3.86699 4.58934 0.61958 0.77255 0.48576 0.95510
736 3.23918 4.73039 3.83015 3.55850 2.29358 3.82376 3.61597 3.24916 3.41618 2.70450 3.92476 3.71671 4.29772 3.72354 3.60101 3.37642 3.52583 3.11945 3.93713 0.97494 736 - -
2.68618 4.42225 2.77519 2.73123 3.46354 2.40513 3.72494 3.29354 2.67741 2.69355 4.24690 2.90347 2.73739 3.18146 2.89801 2.37887 2.77519 2.98518 4.58477 3.61503
0.03157 3.86699 4.58934 0.61958 0.77255 0.48576 0.95510
737 2.31230 4.23263 3.20165 3.01716 4.07082 2.96261 4.12717 3.59269 3.09056 3.31334 4.23988 3.24282 3.70096 3.44030 3.37676 1.09934 2.79202 3.12899 5.44375 4.15432 737 - -
2.68618 4.42225 2.77519 2.73123 3.46354 2.40513 3.72494 3.29354 2.67741 2.69355 4.24690 2.90347 2.73739 3.18146 2.89801 2.37887 2.77519 2.98518 4.58477 3.61503
0.03157 3.86699 4.58934 0.61958 0.77255 0.48576 0.95510
738 1.38927 4.25358 3.56982 3.15592 3.70709 3.26519 4.11091 2.72322 3.05155 2.57584 3.15188 3.41227 3.88083 3.41678 3.34759 2.67320 2.83538 2.49867 5.25758 4.01122 738 - -
2.68618 4.42225 2.77519 2.73123 3.46354 2.40513 3.72494 3.29354 2.67741 2.69355 4.24690 2.90347 2.73739 3.18146 2.89801 2.37887 2.77519 2.98518 4.58477 3.61503
0.03038 3.90497 4.62731 0.61958 0.77255 0.48576 0.95510
739 2.39258 4.30290 3.26912 2.94937 3.81423 3.11961 3.99127 3.24633 2.89945 2.67211 3.95292 3.23780 3.77826 3.28827 3.20963 1.35478 2.80864 2.90867 5.26085 3.91947 739 - -
2.68618 4.42225 2.77519 2.73123 3.46354 2.40513 3.72494 3.29354 2.67741 2.69355 4.24690 2.90347 2.73739 3.18146 2.89801 2.37887 2.77519 2.98518 4.58477 3.61503
0.03038 3.90497 4.62731 0.61958 0.77255 0.48576 0.95510
740 2.43374 4.41489 2.87153 2.74212 4.29507 1.26704 4.04308 3.83635 2.99168 3.49194 4.35931 2.66999 3.73519 3.30389 3.34247 2.55909 2.88440 3.33628 5.58018 4.30594 740 - -
2.68618 4.42225 2.77519 2.73123 3.46354 2.40513 3.72494 3.29354 2.67741 2.69355 4.24690 2.90347 2.73739 3.18146 2.89801 2.37887 2.77519 2.98518 4.58477 3.61503
0.03038 3.90497 4.62731 0.61958 0.77255 0.48576 0.95510

741 3.15763 4.55167 4.49256 3.97591 1.49723 4.24421 4.18909 2.36063 3.83100 1.57537 2.70242 4.15440 4.52108 3.98537 3.98507 3.57880 3.38903 2.42023 4.51009 3.02838 741 - -
2.68618 4.42225 2.77519 2.73123 3.46354 2.40513 3.72494 3.29354 2.67741 2.69355 4.24690 2.90347 2.73739 3.18146 2.89801 2.37887 2.77519 2.98518 4.58477 3.61503
0.02911 3.94717 4.66952 0.61958 0.77255 0.48576 0.95510
742 2.92343 4.59672 3.48741 2.66019 3.04786 3.62779 3.80889 3.15026 2.86732 2.73447 3.77058 3.45513 4.08881 3.33841 3.12042 3.09512 3.18054 2.95746 1.43089 3.08064 742 - -
2.68618 4.42225 2.77519 2.73123 3.46354 2.40513 3.72494 3.29354 2.67741 2.69355 4.24690 2.90347 2.73739 3.18146 2.89801 2.37887 2.77519 2.98518 4.58477 3.61503
0.02911 3.94717 4.66952 0.61958 0.77255 0.48576 0.95510
743 2.68176 4.48989 3.39962 2.84942 3.34172 3.58371 2.07851 2.88489 2.68146 2.37848 3.15825 3.26958 3.97436 3.08867 3.01811 2.85056 2.91884 2.53060 4.85395 3.48008 743 - -
2.68618 4.42225 2.77519 2.73123 3.46354 2.40513 3.72494 3.29354 2.67741 2.69355 4.24690 2.90347 2.73739 3.18146 2.89801 2.37887 2.77519 2.98518 4.58477 3.61503
0.02790 3.98901 4.71136 0.61958 0.77255 0.48576 0.95510
744 3.14012 4.96892 3.64297 3.13119 4.35316 3.60431 3.91369 3.90388 2.29130 3.39869 4.39682 3.48152 4.10211 3.12909 0.81427 3.21926 3.39871 3.62304 5.38952 4.25625 744 - -
2.68618 4.42225 2.77519 2.73123 3.46354 2.40513 3.72494 3.29354 2.67741 2.69355 4.24690 2.90347 2.73739 3.18146 2.89801 2.37887 2.77519 2.98518 4.58477 3.61503
0.02790 3.98901 4.71136 0.61958 0.77255 0.48576 0.95510
745 2.62491 4.72986 2.85876 2.36685 4.18607 3.29899 3.74356 3.60772 2.48035 3.21688 4.06306 2.99227 3.82758 2.92375 2.60614 1.55505 2.92521 3.25086 5.42764 4.11920 745 - -
2.68618 4.42225 2.77519 2.73123 3.46354 2.40513 3.72494 3.29354 2.67741 2.69355 4.24690 2.90347 2.73739 3.18146 2.89801 2.37887 2.77519 2.98518 4.58477 3.61503
0.02790 3.98901 4.71136 0.61958 0.77255 0.48576 0.95510
746 2.88859 5.21658 2.43265 1.22764 4.38863 3.31975 3.22091 3.98956 2.55368 3.51912 4.38940 2.83260 3.87581 2.91036 2.98478 2.83570 3.15472 3.62878 5.60685 4.19014 746 - -
2.68618 4.42225 2.77519 2.73123 3.46354 2.40513 3.72494 3.29354 2.67741 2.69355 4.24690 2.90347 2.73739 3.18146 2.89801 2.37887 2.77519 2.98518 4.58477 3.61503
0.11442 3.98901 2.41237 0.61958 0.77255 0.48576 0.95510
747 3.05067 4.54264 4.25091 3.74931 3.10549 4.08557 4.43871 2.24719 3.50815 1.54208 1.56943 4.04646 4.43171 3.84241 3.71630 3.46133 3.31117 2.28809 5.03306 3.81292 747 - -
2.68618 4.42225 2.77519 2.73123 3.46354 2.40513 3.72494 3.29354 2.67741 2.69355 4.24690 2.90347 2.73739 3.18146 2.89801 2.37887 2.77519 2.98518 4.58477 3.61503
0.03038 3.90497 4.62731 0.61958 0.77255 0.56523 0.83986
748 3.02937 4.41366 4.54896 4.08158 3.41533 4.23942 4.81044 1.14431 3.92800 1.92800 3.26573 4.31842 4.60504 4.22822 4.13508 3.64082 3.31124 1.62024 5.37258 4.14595 748 - -
2.68618 4.42225 2.77519 2.73123 3.46354 2.40513 3.72494 3.29354 2.67741 2.69355 4.24690 2.90347 2.73739 3.18146 2.89801 2.37887 2.77519 2.98518 4.58477 3.61503
0.03038 3.90497 4.62731 0.61958 0.77255 0.56523 0.83986
749 2.86372 4.82228 3.35758 2.79290 3.73336 3.56202 3.64628 3.47142 2.15866 3.01172 3.96247 3.19768 3.97447 2.87830 1.41494 2.93103 3.08383 3.21708 5.04426 3.18894 749 - -
2.68618 4.42225 2.77519 2.73123 3.46354 2.40513 3.72494 3.29354 2.67741 2.69355 4.24690 2.90347 2.73739 3.18146 2.89801 2.37887 2.77519 2.98518 4.58477 3.61503
0.03038 3.90497 4.62731 0.61958 0.77255 0.56523 0.83986
750 2.73451 4.87369 2.62976 1.42762 4.15078 3.31312 3.75821 3.40373 2.57497 3.12771 4.08339 2.92265 3.85308 2.94463 2.98700 2.76359 3.02111 2.82154 5.47260 4.11592 750 - -
2.68618 4.42225 2.77519 2.73123 3.46354 2.40513 3.72494 3.29354 2.67741 2.69355 4.24690 2.90347 2.73739 3.18146 2.89801 2.37887 2.77519 2.98518 4.58477 3.61503
0.03038 3.90497 4.62731 0.61958 0.77255 0.56523 0.83986
751 2.59160 3.89656 3.06077 1.73744 3.85288 3.40855 3.71333 3.10127 2.52332 2.83543 3.42168 3.05010 3.84590 2.90716 2.92771 2.68610 2.84895 2.84117 5.21453 3.91152 751 - -
2.68618 4.42225 2.77519 2.73123 3.46354 2.40513 3.72494 3.29354 2.67741 2.69355 4.24690 2.90347 2.73739 3.18146 2.89801 2.37887 2.77519 2.98518 4.58477 3.61503
0.02940 3.93713 4.65948 0.61958 0.77255 0.56523 0.83986
752 2.68410 4.47784 3.02778 2.82244 3.48129 3.53841 3.42035 2.95894 2.77625 2.60928 1.71912 3.25046 3.97191 3.14457 3.11792 2.85886 2.93352 2.74743 4.95674 3.63473 752 - -
2.68618 4.42225 2.77519 2.73123 3.46354 2.40513 3.72494 3.29354 2.67741 2.69355 4.24690 2.90347 2.73739 3.18146 2.89801 2.37887 2.77519 2.98518 4.58477 3.61503

0.02541 4.08111 4.80345 0.61958 0.77255 0.56523 0.83986
753 2.70239 4.85652 3.11229 2.54024 3.50433 3.49200 3.63372 3.52557 2.19637 3.10507 3.93771 3.03014 3.45669 2.53930 1.77585 2.73718 2.92163 3.21255 5.31120 4.01419 753 - -
2.68618 4.42225 2.77519 2.73123 3.46354 2.40513 3.72494 3.29354 2.67741 2.69355 4.24690 2.90347 2.73739 3.18146 2.89801 2.37887 2.77519 2.98518 4.58477 3.61503
0.02541 4.08111 4.80345 0.61958 0.77255 0.56523 0.83986
754 2.61944 4.90871 2.89017 1.78792 4.18390 3.39966 3.61964 3.61533 2.39318 3.18689 3.65374 2.80288 3.21260 2.63849 2.85812 2.53619 2.85417 3.04813 5.39790 4.04183 754 - -
2.68618 4.42225 2.77519 2.73123 3.46354 2.40513 3.72494 3.29354 2.67741 2.69355 4.24690 2.90347 2.73739 3.18146 2.89801 2.37887 2.77519 2.98518 4.58477 3.61503
0.02468 4.10983 4.83217 0.61958 0.77255 0.43910 1.03457
755 2.80429 4.99522 3.17281 2.60412 4.33139 3.54514 3.66744 3.73258 2.13135 3.00541 4.10127 2.74756 3.94071 2.80819 1.55578 2.48274 3.02115 3.39858 5.43329 4.15496 755 - -
2.68623 4.42237 2.77532 2.73135 3.46366 2.40525 3.72507 3.29340 2.67744 2.69353 4.24616 2.90351 2.73742 3.18159 2.89794 2.37875 2.77524 2.98475 4.58489 3.61499
0.34341 1.34558 3.49781 0.85526 0.55368 0.48576 0.95510
756 2.63113 4.67601 3.14070 2.57507 3.59288 2.26432 3.68903 3.27460 2.24193 2.76153 3.75971 3.06639 3.87661 2.77125 2.61283 2.71104 2.85981 2.99092 4.21886 3.89346 759 - -
2.68618 4.42225 2.77519 2.73123 3.46354 2.40513 3.72494 3.29354 2.67741 2.69355 4.24690 2.90347 2.73739 3.18146 2.89801 2.37887 2.77519 2.98518 4.58477 3.61503
0.02295 4.18183 4.90418 0.61958 0.77255 0.51369 0.91202
757 2.64370 4.23592 3.75813 3.18361 2.91543 3.67676 3.92329 2.58905 3.09308 2.28831 2.75264 3.51162 4.04864 3.12686 2.75122 2.94159 2.87475 2.49324 4.76933 2.21256 760 - -
2.68618 4.42225 2.77519 2.73123 3.46354 2.40513 3.72494 3.29354 2.67741 2.69355 4.24690 2.90347 2.73739 3.18146 2.89801 2.37887 2.77519 2.98518 4.58477 3.61503
0.04393 4.25065 3.54993 0.61958 0.77255 0.51369 0.91202
758 2.65807 4.63959 3.22243 2.65149 3.16292 3.31968 3.45710 3.21959 2.44373 2.87161 3.72838 3.12755 3.91417 2.93289 1.84477 2.62483 2.88543 2.94940 4.18357 3.84286 761 - -
2.68618 4.42225 2.77519 2.73123 3.46354 2.40513 3.72494 3.29354 2.67741 2.69355 4.24690 2.90347 2.73739 3.18146 2.89801 2.37887 2.77519 2.98518 4.58477 3.61503
0.02189 4.22860 4.95095 0.61958 0.77255 0.49540 0.93989
759 2.73006 5.11184 2.97753 2.36324 4.44441 3.47811 3.38196 3.88621 1.65980 3.39009 4.16915 2.96303 3.49384 2.64434 2.55676 2.16325 2.95258 3.49885 5.53274 4.18195 762 - -
2.68618 4.42225 2.77519 2.73123 3.46354 2.40513 3.72494 3.29354 2.67741 2.69355 4.24690 2.90347 2.73739 3.18146 2.89801 2.37887 2.77519 2.98518 4.58477 3.61503
0.11847 4.25065 2.32822 0.61958 0.77255 0.51369 0.91202
760 2.49187 4.79909 3.04960 2.50428 4.04495 3.46239 2.27207 3.45270 2.33358 3.05918 3.88494 3.00927 3.85818 2.69198 2.66948 2.46976 2.87789 2.81374 5.30059 3.98715 763 - -
2.68618 4.42225 2.77519 2.73123 3.46354 2.40513 3.72494 3.29354 2.67741 2.69355 4.24690 2.90347 2.73739 3.18146 2.89801 2.37887 2.77519 2.98518 4.58477 3.61503
0.02356 4.15574 4.87808 0.61958 0.77255 0.62465 0.76668
761 2.72553 5.10564 2.51939 2.00942 4.41364 3.08122 3.68324 3.86867 2.38945 3.40989 4.20561 2.86276 2.08648 2.81887 2.99634 2.69742 2.77062 3.48537 5.59754 4.20347 764 - -
2.68618 4.42225 2.77519 2.73123 3.46354 2.40513 3.72494 3.29354 2.67741 2.69355 4.24690 2.90347 2.73739 3.18146 2.89801 2.37887 2.77519 2.98518 4.58477 3.61503
0.02356 4.15574 4.87808 0.61958 0.77255 0.49006 0.94828
762 1.87860 4.49512 3.29324 2.73197 3.66773 3.53473 3.17223 2.79353 2.57392 2.72651 3.40916 3.18934 3.92350 3.02704 3.09616 2.51628 2.74413 2.62015 5.06237 3.79859 765 - -
2.68618 4.42225 2.77519 2.73123 3.46354 2.40513 3.72494 3.29354 2.67741 2.69355 4.24690 2.90347 2.73739 3.18146 2.89801 2.37887 2.77519 2.98518 4.58477 3.61503
0.02150 4.24648 4.96882 0.61958 0.77255 0.54437 0.86799
763 2.65898 4.33817 2.87820 2.41703 4.30087 3.44976 3.62650 3.74094 1.74881 3.28105 3.71164 2.94606 3.83957 2.49645 2.46123 2.44041 2.88667 3.36940 5.46073 4.10212 766 - -
2.68618 4.42225 2.77519 2.73123 3.46354 2.40513 3.72494 3.29354 2.67741 2.69355 4.24690 2.90347 2.73739 3.18146 2.89801 2.37887 2.77519 2.98518 4.58477 3.61503
0.02150 4.24648 4.96882 0.61958 0.77255 0.54437 0.86799
764 2.69739 4.97118 3.04947 2.48922 4.28608 3.47835 3.65709 3.46730 1.89150 3.26171 4.06125 3.00138 3.41830 2.78786 2.41346 1.93353 2.92696 3.35390 5.44682 4.11631 767 - -

2.68618 4.42225 2.77519 2.73123 3.46354 2.40513 3.72494 3.29354 2.67741 2.69355 4.24690 2.90347 2.73739 3.18146 2.89801 2.37887 2.77519 2.98518 4.58477 3.61503

0.02150 4.24648 4.96882 0.61958 0.77255 0.54437 0.86799

765 2.75870 4.25788 4.09320 3.52366 2.92476 3.82845 4.04327 2.57488 3.39593 1.95847 3.26111 3.75873 4.19349 3.63717 3.60493 3.11966 2.46796 2.33700 1.95547 3.34454 768 - -

2.68618 4.42225 2.77519 2.73123 3.46354 2.40513 3.72494 3.29354 2.67741 2.69355 4.24690 2.90347 2.73739 3.18146 2.89801 2.37887 2.77519 2.98518 4.58477 3.61503

0.10805 4.24648 2.42928 0.61958 0.77255 0.54437 0.86799

766 2.52065 4.30295 3.63319 2.86714 3.44523 3.66737 3.93204 1.83377 2.60122 2.17407 3.39981 3.44502 4.04249 3.29163 3.27104 2.93122 2.88922 2.30877 4.93225 3.71001 769 - -

2.68618 4.42225 2.77519 2.73123 3.46354 2.40513 3.72494 3.29354 2.67741 2.69355 4.24690 2.90347 2.73739 3.18146 2.89801 2.37887 2.77519 2.98518 4.58477 3.61503

0.02342 4.16185 4.88419 0.61958 0.77255 0.64111 0.74804

767 2.64664 4.09296 4.19384 3.60495 2.68942 3.76684 4.05490 1.68594 3.46051 2.01217 3.14277 3.77725 4.12632 3.66778 3.31726 3.06091 2.70221 2.28807 3.89201 3.45345 770 - -

2.68618 4.42225 2.77519 2.73123 3.46354 2.40513 3.72494 3.29354 2.67741 2.69355 4.24690 2.90347 2.73739 3.18146 2.89801 2.37887 2.77519 2.98518 4.58477 3.61503

0.02342 4.16185 4.88419 0.61958 0.77255 0.64111 0.74804

768 2.62033 4.52985 3.23367 2.69874 3.33639 3.03847 2.25582 3.10548 2.65982 2.41225 3.64987 3.15693 3.90849 3.00431 3.05884 2.46293 2.86174 2.84640 5.01867 3.69124 771 - -

2.68618 4.42225 2.77519 2.73123 3.46354 2.40513 3.72494 3.29354 2.67741 2.69355 4.24690 2.90347 2.73739 3.18146 2.89801 2.37887 2.77519 2.98518 4.58477 3.61503

0.02342 4.16185 4.88419 0.61958 0.77255 0.64111 0.74804

769 2.50471 4.52339 3.22098 1.94729 3.69105 3.50609 3.73197 2.92440 2.62824 2.61287 3.39272 2.94563 3.89111 2.96445 3.04083 2.73084 2.83353 2.67387 3.67416 3.79758 772 - -

2.68618 4.42225 2.77519 2.73123 3.46354 2.40513 3.72494 3.29354 2.67741 2.69355 4.24690 2.90347 2.73739 3.18146 2.89801 2.37887 2.77519 2.98518 4.58477 3.61503

0.18867 4.16185 1.85560 0.61958 0.77255 0.64111 0.74804

770 2.53837 4.66772 2.72871 2.49423 4.33866 3.16277 3.83950 3.81427 2.70489 3.41406 4.24091 1.61937 3.19956 3.02505 3.13413 2.18402 2.90249 3.37111 5.59762 4.25325 773 - -

2.68618 4.42225 2.77519 2.73123 3.46354 2.40513 3.72494 3.29354 2.67741 2.69355 4.24690 2.90347 2.73739 3.18146 2.89801 2.37887 2.77519 2.98518 4.58477 3.61503

0.02757 4.00074 4.72309 0.61958 0.77255 0.78552 0.60859

771 2.90769 5.18108 2.92356 2.33083 4.56085 3.50142 3.61952 3.95597 1.38758 3.42910 4.26703 2.99358 3.92982 2.33620 2.36999 2.87659 3.11142 3.60483 5.52500 4.25558 774 - -

2.68618 4.42225 2.77519 2.73123 3.46354 2.40513 3.72494 3.29354 2.67741 2.69355 4.24690 2.90347 2.73739 3.18146 2.89801 2.37887 2.77519 2.98518 4.58477 3.61503

0.02757 4.00074 4.72309 0.61958 0.77255 0.73475 0.65321

772 2.74296 4.39105 3.65816 3.12692 3.51749 3.68572 3.94904 1.51836 2.55841 2.44883 3.47310 3.48012 4.08651 3.27786 2.80696 3.01278 2.98401 2.39747 5.01631 3.78804 775 - -

2.68618 4.42225 2.77519 2.73123 3.46354 2.40513 3.72494 3.29354 2.67741 2.69355 4.24690 2.90347 2.73739 3.18146 2.89801 2.37887 2.77519 2.98518 4.58477 3.61503

0.02673 4.03115 4.75349 0.61958 0.77255 0.76149 0.62918

773 2.22344 4.57318 3.06094 2.65752 3.93731 3.31800 1.94633 3.38519 2.59770 3.04409 3.91441 3.09358 3.84318 3.01086 2.96386 2.67218 2.49711 3.06151 5.27685 3.96009 776 - -

2.68618 4.42225 2.77519 2.73123 3.46354 2.40513 3.72494 3.29354 2.67741 2.69355 4.24690 2.90347 2.73739 3.18146 2.89801 2.37887 2.77519 2.98518 4.58477 3.61503

0.02673 4.03115 4.75349 0.61958 0.77255 0.60016 0.79567

774 2.60367 4.64263 3.10818 2.42859 3.88168 3.45155 3.71186 3.26545 2.55054 2.75879 3.38846 2.94021 3.86313 2.90399 2.97861 1.88021 2.46576 2.97618 5.21116 3.91400 777 - -

2.68618 4.42225 2.77519 2.73123 3.46354 2.40513 3.72494 3.29354 2.67741 2.69355 4.24690 2.90347 2.73739 3.18146 2.89801 2.37887 2.77519 2.98518 4.58477 3.61503

0.10033 4.16565 2.52645 0.61958 0.77255 0.67620 0.71038

775 2.42714 4.38189 3.31660 2.81023 2.13722 3.25567 3.80588 2.94792 2.49624 2.64995 3.53967 3.22795 3.19583 3.10232 3.12776 2.76027 2.83342 2.70298 4.98482 3.72221 778 - -

2.68618 4.42225 2.77519 2.73123 3.46354 2.40513 3.72494 3.29354 2.67741 2.69355 4.24690 2.90347 2.73739 3.18146 2.89801 2.37887 2.77519 2.98518 4.58477 3.61503

0.02517 4.09049 4.81283 0.61958 0.77255 0.74691 0.64213

776 2.62924 4.76965 2.81304 2.47532 3.99067 3.43759 2.25214 3.40560 2.29372 3.02140 3.11577 2.98443 3.84027 2.81735 2.85877 2.67560 2.73755 3.09910 5.27001 3.94870 779 - -
2.68618 4.42225 2.77519 2.73123 3.46354 2.40513 3.72494 3.29354 2.67741 2.69355 4.24690 2.90347 2.73739 3.18146 2.89801 2.37887 2.77519 2.98518 4.58477 3.61503
0.02517 4.09049 4.81283 0.61958 0.77255 0.74691 0.64213
777 1.88143 4.61332 3.08708 2.30134 3.55950 3.44716 3.69273 3.03342 2.46901 2.86563 3.71812 3.04743 3.48166 2.89069 2.97267 2.68277 2.83055 2.92013 5.16819 3.87315 780 - -
2.68618 4.42225 2.77519 2.73123 3.46354 2.40513 3.72494 3.29354 2.67741 2.69355 4.24690 2.90347 2.73739 3.18146 2.89801 2.37887 2.77519 2.98518 4.58477 3.61503
0.02517 4.09049 4.81283 0.61958 0.77255 0.70192 0.68445
778 2.97389 5.21384 3.21204 2.39616 4.63125 3.61661 3.63754 4.00341 1.79053 3.45560 4.28969 2.91049 4.00079 2.76419 1.40724 2.95133 3.15874 3.65609 5.52648 4.29618 781 - -
2.68618 4.42225 2.77519 2.73123 3.46354 2.40513 3.72494 3.29354 2.67741 2.69355 4.24690 2.90347 2.73739 3.18146 2.89801 2.37887 2.77519 2.98518 4.58477 3.61503
0.02455 4.11511 4.83746 0.61958 0.77255 0.72486 0.66241
779 2.73448 5.10659 1.59782 2.28750 4.50035 2.91853 3.72985 3.96558 2.49032 3.50640 4.30886 2.83805 3.83463 2.87482 3.11675 2.38987 2.62374 3.55741 5.69195 4.28196 782 - -
2.68626 4.42055 2.77528 2.73131 3.46343 2.40521 3.72483 3.29350 2.67749 2.69347 4.24698 2.90311 2.73748 3.18155 2.89809 2.37885 2.77522 2.98527 4.58485 3.61511
0.22835 1.62848 4.83746 0.89671 0.52410 0.72486 0.66241
780 2.49453 4.44930 3.27819 2.76135 3.88308 2.96601 3.84445 3.26265 2.73614 2.94002 3.47331 2.98301 3.83964 3.07069 3.13650 2.15106 1.82417 2.80269 5.24269 3.97466 789 - -
2.68618 4.42225 2.77519 2.73123 3.46354 2.40513 3.72494 3.29354 2.67741 2.69355 4.24690 2.90347 2.73739 3.18146 2.89801 2.37887 2.77519 2.98518 4.58477 3.61503
0.02396 4.13906 4.86141 0.61958 0.77255 0.72486 0.66241
781 2.59890 4.56678 3.18717 2.63369 3.78072 3.47488 3.72518 2.94253 2.36363 2.82428 3.45440 2.96714 3.87718 2.93865 2.98615 1.86473 2.83961 2.65190 5.13751 3.85618 790 - -
2.68618 4.42225 2.77519 2.73123 3.46354 2.40513 3.72494 3.29354 2.67741 2.69355 4.24690 2.90347 2.73739 3.18146 2.89801 2.37887 2.77519 2.98518 4.58477 3.61503
0.02396 4.13906 4.86141 0.61958 0.77255 0.72486 0.66241
782 2.66148 4.81455 3.00514 2.49378 3.29966 3.45894 2.23548 3.46701 2.18859 3.06919 3.90083 2.67000 3.86269 2.83127 2.85215 2.60435 2.89289 3.15552 5.24713 3.88837 791 - -
2.68618 4.42225 2.77519 2.73123 3.46354 2.40513 3.72494 3.29354 2.67741 2.69355 4.24690 2.90347 2.73739 3.18146 2.89801 2.37887 2.77519 2.98518 4.58477 3.61503
0.02396 4.13906 4.86141 0.61958 0.77255 0.59027 0.80784
783 2.61143 4.51991 3.24474 2.68444 3.44635 3.51893 3.74857 2.94757 2.65323 2.74810 3.61422 3.01673 2.32268 2.78788 2.94908 2.61701 2.84355 2.54029 5.07232 3.43397 792 - -
2.68618 4.42225 2.77519 2.73123 3.46354 2.40513 3.72494 3.29354 2.67741 2.69355 4.24690 2.90347 2.73739 3.18146 2.89801 2.37887 2.77519 2.98518 4.58477 3.61503
0.04820 4.21078 3.43507 0.61958 0.77255 0.65252 0.73549
784 2.25324 4.91042 2.98275 2.37383 4.18553 3.44532 3.42090 3.60885 2.37406 3.18212 3.98568 2.96382 3.84315 1.98905 2.65039 2.66739 2.69566 3.11441 5.39251 4.05360 793 - -
2.68618 4.42225 2.77519 2.73123 3.46354 2.40513 3.72494 3.29354 2.67741 2.69355 4.24690 2.90347 2.73739 3.18146 2.89801 2.37887 2.77519 2.98518 4.58477 3.61503
0.02286 4.18545 4.90779 0.61958 0.77255 0.62851 0.76226
785 2.65715 5.04855 2.86251 1.78627 4.36003 3.41139 3.62701 3.81414 2.37617 3.19064 4.11522 2.54167 3.57523 2.74450 2.60412 2.40009 2.72445 3.42444 5.51280 4.13279 794 - -
2.68618 4.42225 2.77519 2.73123 3.46354 2.40513 3.72494 3.29354 2.67741 2.69355 4.24690 2.90347 2.73739 3.18146 2.89801 2.37887 2.77519 2.98518 4.58477 3.61503
0.02229 4.21078 4.93313 0.61958 0.77255 0.65252 0.73549
786 2.65962 4.91684 3.02214 2.45838 3.81222 3.46276 3.43020 3.60959 1.72125 3.18062 3.98342 2.86124 3.85115 2.62620 2.61737 2.55328 2.88609 2.86251 5.38730 4.05118 795 - -
2.68618 4.42225 2.77519 2.73123 3.46354 2.40513 3.72494 3.29354 2.67741 2.69355 4.24690 2.90347 2.73739 3.18146 2.89801 2.37887 2.77519 2.98518 4.58477 3.61503
0.02229 4.21078 4.93313 0.61958 0.77255 0.39404 1.12186
787 2.65184 4.48770 2.25075 2.80357 3.11244 2.94841 3.82737 2.44419 2.77645 2.52873 3.58473 3.25276 3.69660 3.09589 3.17152 2.81887 2.88479 2.49884 5.05908 3.80065 796 - -
2.68618 4.42225 2.77519 2.73123 3.46354 2.40513 3.72494 3.29354 2.67741 2.69355 4.24690 2.90347 2.73739 3.18146 2.89801 2.37887 2.77519 2.98518 4.58477 3.61503

0.01949 4.34329 5.06564 0.61958 0.77255 0.48576 0.95510

788 2.69729 3.51835 4.52051 3.91806 3.14205 3.86097 4.18277 1.70094 3.73515 1.84894 3.15832 3.97378 4.21780 3.88946 3.79384 3.16877 2.92942 2.00037 3.18006 3.28157 797 - -

2.68618 4.42225 2.77519 2.73123 3.46354 2.40513 3.72494 3.29354 2.67741 2.69355 4.24690 2.90347 2.73739 3.18146 2.89801 2.37887 2.77519 2.98518 4.58477 3.61503

0.01949 4.34329 5.06564 0.61958 0.77255 0.48576 0.95510

789 2.67794 4.16580 4.08057 3.49832 3.06427 3.77350 4.05881 2.45590 3.20251 2.01755 3.25772 3.72823 4.14029 3.61417 3.58118 3.05796 2.65001 2.07423 4.75177 1.98675 798 - -

2.68618 4.42225 2.77519 2.73123 3.46354 2.40513 3.72494 3.29354 2.67741 2.69355 4.24690 2.90347 2.73739 3.18146 2.89801 2.37887 2.77519 2.98518 4.58477 3.61503

0.01949 4.34329 5.06564 0.61958 0.77255 0.48576 0.95510

790 2.45773 4.48184 3.38998 2.73299 3.15629 3.59293 3.82654 2.99482 2.66682 2.54669 3.57989 3.26564 3.97529 3.09868 2.09112 2.82889 2.77402 2.20042 5.04938 3.79697 799 - -

2.68618 4.42225 2.77519 2.73123 3.46354 2.40513 3.72494 3.29354 2.67741 2.69355 4.24690 2.90347 2.73739 3.18146 2.89801 2.37887 2.77519 2.98518 4.58477 3.61503

0.27616 4.34329 1.47703 0.61958 0.77255 0.48576 0.95510

791 2.05747 4.28812 3.39618 3.00364 4.11101 1.56849 4.07743 3.06566 3.02017 3.18470 4.03227 3.27617 3.77707 3.33099 3.37480 2.12026 2.77270 3.05156 5.47274 4.23225 800 - -

2.68618 4.42225 2.77519 2.73123 3.46354 2.40513 3.72494 3.29354 2.67741 2.69355 4.24690 2.90347 2.73739 3.18146 2.89801 2.37887 2.77519 2.98518 4.58477 3.61503

0.02512 4.09226 4.81460 0.61958 0.77255 0.34316 1.23623

792 2.64193 4.25534 4.43747 3.85219 2.71549 3.99626 4.31607 2.21801 3.43721 1.29090 3.18659 4.02461 4.33474 3.90332 3.85312 3.29934 3.07081 1.97356 4.89278 3.72191 801 - -

2.68618 4.42225 2.77519 2.73123 3.46354 2.40513 3.72494 3.29354 2.67741 2.69355 4.24690 2.90347 2.73739 3.18146 2.89801 2.37887 2.77519 2.98518 4.58477 3.61503

0.01949 4.34329 5.06564 0.61958 0.77255 0.48576 0.95510

793 2.51066 4.65408 3.19260 1.96797 3.85069 3.52814 3.75387 2.89214 2.61795 2.89330 3.41187 3.12396 3.07363 2.95615 3.05120 2.58822 2.74597 2.48704 5.20061 3.90908 802 - -

2.68618 4.42225 2.77519 2.73123 3.46354 2.40513 3.72494 3.29354 2.67741 2.69355 4.24690 2.90347 2.73739 3.18146 2.89801 2.37887 2.77519 2.98518 4.58477 3.61503

0.01949 4.34329 5.06564 0.61958 0.77255 0.48576 0.95510

794 2.42458 4.23702 3.76837 3.19543 3.35989 3.67871 3.04399 1.95085 3.11879 2.16901 3.34991 3.52508 3.76491 3.39290 3.40373 2.72280 2.65390 2.30615 4.84114 3.62963 803 - -

2.68618 4.42225 2.77519 2.73123 3.46354 2.40513 3.72494 3.29354 2.67741 2.69355 4.24690 2.90347 2.73739 3.18146 2.89801 2.37887 2.77519 2.98518 4.58477 3.61503

0.20042 4.34329 1.78012 0.61958 0.77255 0.48576 0.95510

795 2.50371 4.24239 3.73108 3.18832 3.18085 3.59245 3.99288 2.64952 3.10811 1.59964 3.37817 3.50596 4.02448 3.39802 3.39855 2.48978 2.48390 2.45141 4.90902 3.68845 804 - -

2.68618 4.42225 2.77519 2.73123 3.46354 2.40513 3.72494 3.29354 2.67741 2.69355 4.24690 2.90347 2.73739 3.18146 2.89801 2.37887 2.77519 2.98518 4.58477 3.61503

0.02331 4.16619 4.88854 0.61958 0.77255 0.48225 0.96074

796 2.67119 4.12951 4.18585 3.60333 2.60718 3.49623 4.10951 1.65388 3.47493 2.01236 3.22082 3.79390 3.80525 3.69091 3.64216 3.08347 2.60529 2.26650 4.76312 3.56982 805 - -

2.68618 4.42225 2.77519 2.73123 3.46354 2.40513 3.72494 3.29354 2.67741 2.69355 4.24690 2.90347 2.73739 3.18146 2.89801 2.37887 2.77519 2.98518 4.58477 3.61503

0.02076 4.28076 5.00310 0.61958 0.77255 0.57069 0.83272

797 3.30349 4.62192 5.04987 4.46174 2.39615 4.54915 4.83618 2.22888 4.32151 1.19340 1.73850 4.63078 4.74907 4.36065 4.37045 3.88388 3.51987 2.21929 5.12503 4.05439 806 - -

2.68618 4.42225 2.77519 2.73123 3.46354 2.40513 3.72494 3.29354 2.67741 2.69355 4.24690 2.90347 2.73739 3.18146 2.89801 2.37887 2.77519 2.98518 4.58477 3.61503

0.07684 4.28076 2.81126 0.61958 0.77255 0.57069 0.83272

798 2.66910 4.75044 3.07590 1.87189 3.10500 3.50025 3.71231 3.31874 2.53749 2.44613 3.80267 3.06090 3.89754 2.44257 2.96886 2.73477 2.90080 3.04703 5.22929 3.90023 807 - -

2.68618 4.42225 2.77519 2.73123 3.46354 2.40513 3.72494 3.29354 2.67741 2.69355 4.24690 2.90347 2.73739 3.18146 2.89801 2.37887 2.77519 2.98518 4.58477 3.61503

0.02195 4.22587 4.94821 0.61958 0.77255 0.52325 0.89793

799 2.75076 1.99012 4.38621 3.81485 2.33301 3.85848 3.97217 2.60171 3.65335 2.20888 3.27461 3.88444 4.21950 3.81245 3.74963 2.99883 2.98231 2.41943 3.22060 2.65926 808 - -

2.68618 4.42225 2.77519 2.73123 3.46354 2.40513 3.72494 3.29354 2.67741 2.69355 4.24690 2.90347 2.73739 3.18146 2.89801 2.37887 2.77519 2.98518 4.58477 3.61503
0.02076 4.28076 5.00310 0.61958 0.77255 0.43610 1.04003
800 2.83372 4.53629 4.87629 4.29171 2.49474 4.40350 4.71091 2.20307 4.15486 0.94958 2.87155 4.46437 4.65570 4.25492 4.24264 3.72753 3.40847 2.25770 5.10169 3.99727 809 - -
2.68618 4.42225 2.77519 2.73123 3.46354 2.40513 3.72494 3.29354 2.67741 2.69355 4.24690 2.90347 2.73739 3.18146 2.89801 2.37887 2.77519 2.98518 4.58477 3.61503
0.01949 4.34329 5.06564 0.61958 0.77255 0.48576 0.95510
801 2.65428 4.70478 3.15417 2.41869 2.94649 3.31896 3.73331 3.30027 1.93969 2.60490 3.78841 2.95025 3.90724 2.92186 3.00995 2.54184 2.88562 2.74726 5.23437 3.93378 810 - -
2.68618 4.42225 2.77519 2.73123 3.46354 2.40513 3.72494 3.29354 2.67741 2.69355 4.24690 2.90347 2.73739 3.18146 2.89801 2.37887 2.77519 2.98518 4.58477 3.61503
0.01949 4.34329 5.06564 0.61958 0.77255 0.48576 0.95510
802 3.04594 4.40161 4.77663 4.19683 3.05296 4.26852 4.58379 2.06294 4.05281 1.30677 3.16046 4.33644 4.56235 4.19274 4.14833 3.58996 3.27607 1.48009 5.03400 3.38698 811 - -
2.68618 4.42225 2.77519 2.73123 3.46354 2.40513 3.72494 3.29354 2.67741 2.69355 4.24690 2.90347 2.73739 3.18146 2.89801 2.37887 2.77519 2.98518 4.58477 3.61503
0.01949 4.34329 5.06564 0.61958 0.77255 0.48576 0.95510
803 2.64267 4.49324 3.38008 2.83624 3.70667 1.98758 3.85619 2.88446 2.77751 2.12754 3.64544 3.26849 3.31629 3.12139 2.98092 2.69478 2.89605 2.81894 5.10910 3.85378 812 - -
2.68618 4.42225 2.77519 2.73123 3.46354 2.40513 3.72494 3.29354 2.67741 2.69355 4.24690 2.90347 2.73739 3.18146 2.89801 2.37887 2.77519 2.98518 4.58477 3.61503
0.05839 4.34329 3.12990 0.61958 0.77255 0.48576 0.95510
804 2.63823 3.61040 3.15675 2.90162 3.54377 3.55372 3.86957 2.99903 2.87640 2.70053 3.58916 3.31222 3.03856 3.18894 3.24445 2.71669 2.89460 2.75471 4.98942 1.78246 813 - -
2.68618 4.42225 2.77519 2.73123 3.46354 2.40513 3.72494 3.29354 2.67741 2.69355 4.24690 2.90347 2.73739 3.18146 2.89801 2.37887 2.77519 2.98518 4.58477 3.61503
0.02026 4.30516 5.02751 0.61958 0.77255 0.53902 0.87543
805 2.41861 4.42645 3.41424 2.04392 3.09157 3.59069 3.83356 2.81818 2.81129 2.32770 3.52098 3.28306 3.97173 3.12648 3.03370 2.82761 2.87008 2.37452 4.99851 3.47430 814 - -
2.68618 4.42225 2.77519 2.73123 3.46354 2.40513 3.72494 3.29354 2.67741 2.69355 4.24690 2.90347 2.73739 3.18146 2.89801 2.37887 2.77519 2.98518 4.58477 3.61503
0.02026 4.30516 5.02751 0.61958 0.77255 0.53902 0.87543
806 2.62954 3.90792 3.34839 2.97576 3.18525 3.37026 3.44337 2.67973 2.92644 2.36005 3.43249 3.18446 2.39819 3.22381 3.27054 2.76435 2.86137 2.37116 4.12050 3.68204 815 - -
2.68618 4.42225 2.77519 2.73123 3.46354 2.40513 3.72494 3.29354 2.67741 2.69355 4.24690 2.90347 2.73739 3.18146 2.89801 2.37887 2.77519 2.98518 4.58477 3.61503
0.02026 4.30516 5.02751 0.61958 0.77255 0.53902 0.87543
807 2.64081 4.75529 3.09340 2.53940 3.53088 3.28133 3.70300 3.37911 2.52721 2.69329 3.83762 2.16267 3.26010 2.87450 2.82905 2.30574 2.87352 3.07817 5.27548 3.69279 816 - -
2.68618 4.42225 2.77519 2.73123 3.46354 2.40513 3.72494 3.29354 2.67741 2.69355 4.24690 2.90347 2.73739 3.18146 2.89801 2.37887 2.77519 2.98518 4.58477 3.61503
0.02026 4.30516 5.02751 0.61958 0.77255 0.53902 0.87543
808 2.63369 4.51536 3.11794 2.74657 3.44020 3.54684 3.79623 3.05894 2.72146 2.66028 3.62234 3.20451 3.93960 3.04699 3.12665 2.12700 1.98648 2.63858 5.08278 3.48285 817 - -
2.68618 4.42225 2.77519 2.73123 3.46354 2.40513 3.72494 3.29354 2.67741 2.69355 4.24690 2.90347 2.73739 3.18146 2.89801 2.37887 2.77519 2.98518 4.58477 3.61503
0.02026 4.30516 5.02751 0.61958 0.77255 0.53902 0.87543
809 2.55265 3.89098 3.57306 2.09304 2.84879 3.63291 3.89486 2.45782 2.95351 2.35175 3.42792 3.39496 4.01171 3.24949 3.29277 2.58845 2.87042 2.42553 4.91664 3.68779 818 - -
2.68618 4.42225 2.77519 2.73123 3.46354 2.40513 3.72494 3.29354 2.67741 2.69355 4.24690 2.90347 2.73739 3.18146 2.89801 2.37887 2.77519 2.98518 4.58477 3.61503
0.02026 4.30516 5.02751 0.61958 0.77255 0.53902 0.87543
810 2.50686 4.28618 3.08337 3.08348 2.96782 3.65378 3.91321 2.74986 3.02480 2.08007 3.38880 3.44825 4.03123 3.30950 3.34158 2.91104 2.62004 2.45556 4.85471 2.27926 819 - -
2.68618 4.42225 2.77519 2.73123 3.46354 2.40513 3.72494 3.29354 2.67741 2.69355 4.24690 2.90347 2.73739 3.18146 2.89801 2.37887 2.77519 2.98518 4.58477 3.61503
0.02026 4.30516 5.02751 0.61958 0.77255 0.53902 0.87543

811 2.62775 4.50115 3.31948 2.76260 3.46431 3.21264 3.80476 3.06098 2.58746 2.74864 3.61944 3.21443 3.56706 3.05837 2.99898 2.19275 2.62238 2.01625 5.08418 3.82205 820 - -
2.68618 4.42225 2.77519 2.73123 3.46354 2.40513 3.72494 3.29354 2.67741 2.69355 4.24690 2.90347 2.73739 3.18146 2.89801 2.37887 2.77519 2.98518 4.58477 3.61503
0.14019 4.30516 2.14294 0.61958 0.77255 0.53902 0.87543
812 2.60022 4.30604 3.23044 2.94756 3.45324 3.34202 3.85816 2.49617 2.90065 1.79054 3.42138 3.34182 3.39133 3.20173 3.24497 2.68110 2.83824 2.44455 4.90709 3.67559 821 - -
2.68618 4.42225 2.77519 2.73123 3.46354 2.40513 3.72494 3.29354 2.67741 2.69355 4.24690 2.90347 2.73739 3.18146 2.89801 2.37887 2.77519 2.98518 4.58477 3.61503
0.02281 4.18778 4.91013 0.61958 0.77255 0.45103 1.01328
813 2.64427 4.67191 3.17134 2.62599 3.90336 3.08515 3.75198 2.80401 2.58918 2.83142 3.79004 3.11169 3.90695 2.07251 3.01306 2.62306 2.18975 2.99861 5.23663 3.94276 822 - -
2.68618 4.42225 2.77519 2.73123 3.46354 2.40513 3.72494 3.29354 2.67741 2.69355 4.24690 2.90347 2.73739 3.18146 2.89801 2.37887 2.77519 2.98518 4.58477 3.61503
0.08668 4.30516 2.66597 0.61958 0.77255 0.53902 0.87543
814 2.67089 5.09996 2.73096 1.78069 4.41343 3.41233 3.63066 3.87652 2.22292 3.39067 4.15678 2.77915 3.82670 2.74405 2.72210 2.18974 2.90488 3.19453 5.55149 4.15976 823 - -
2.68618 4.42225 2.77519 2.73123 3.46354 2.40513 3.72494 3.29354 2.67741 2.69355 4.24690 2.90347 2.73739 3.18146 2.89801 2.37887 2.77519 2.98518 4.58477 3.61503
0.07976 4.24012 2.77653 0.61958 0.77255 0.61972 0.77239
815 2.61973 4.56411 3.19984 2.64776 3.74398 3.24236 3.37537 3.12761 2.59368 2.79606 3.65701 2.89108 3.89536 2.71519 2.83386 2.73752 2.74608 1.97821 5.10850 3.55036 824 - -
2.68618 4.42225 2.77519 2.73123 3.46354 2.40513 3.72494 3.29354 2.67741 2.69355 4.24690 2.90347 2.73739 3.18146 2.89801 2.37887 2.77519 2.98518 4.58477 3.61503
0.05020 4.18327 3.38990 0.61958 0.77255 0.68151 0.70492
816 2.70806 5.00017 1.69866 2.36600 4.24262 3.39149 3.69517 3.21882 2.52650 3.01878 4.10208 2.43825 3.85361 2.84480 3.01067 2.70476 2.95666 3.31764 5.49347 4.10744 825 - -
2.68618 4.42225 2.77519 2.73123 3.46354 2.40513 3.72494 3.29354 2.67741 2.69355 4.24690 2.90347 2.73739 3.18146 2.89801 2.37887 2.77519 2.98518 4.58477 3.61503
0.02354 4.15661 4.87896 0.61958 0.77255 0.70811 0.67841
817 2.69547 5.14908 2.52818 1.65410 4.46058 3.37912 3.27099 3.93092 2.15239 3.43702 4.20774 2.72303 3.81951 2.74991 2.89087 2.65649 2.93305 3.22506 5.59248 4.18910 826 - -
2.68618 4.42225 2.77519 2.73123 3.46354 2.40513 3.72494 3.29354 2.67741 2.69355 4.24690 2.90347 2.73739 3.18146 2.89801 2.37887 2.77519 2.98518 4.58477 3.61503
0.02354 4.15661 4.87896 0.61958 0.77255 0.64200 0.74705
818 2.62740 4.20512 3.77173 2.95736 2.00656 3.67236 3.94782 2.39269 2.85932 2.25400 3.29487 3.52313 4.04481 3.38788 3.38242 2.94162 2.74420 2.29985 4.80445 3.58892 827 - -
2.68618 4.42225 2.77519 2.73123 3.46354 2.40513 3.72494 3.29354 2.67741 2.69355 4.24690 2.90347 2.73739 3.18146 2.89801 2.37887 2.77519 2.98518 4.58477 3.61503
0.07063 4.19068 2.93635 0.61958 0.77255 0.45275 1.01027
819 2.63336 4.54825 3.25965 2.69801 3.72281 3.53582 3.24823 3.10196 2.64603 2.77712 2.32433 3.16553 3.10461 2.79499 2.86010 2.56985 2.86480 2.56441 5.10018 3.82958 828 - -
2.68586 4.42192 2.77563 2.73164 3.46385 2.40680 3.72613 3.29228 2.67903 2.69320 4.24752 2.90272 2.73667 3.18081 2.89846 2.37738 2.77582 2.98303 4.58476 3.61617
0.42812 1.07460 4.98589 3.34808 0.03578 0.59200 0.80569
820 2.57196 5.17255 2.83091 2.07946 4.50993 3.44897 3.68237 3.96920 1.66251 3.47377 4.24834 2.68229 3.88147 2.79976 2.85750 2.22373 2.98691 3.56575 5.62305 4.24005 891 - -
2.68618 4.42225 2.77519 2.73123 3.46354 2.40513 3.72494 3.29354 2.67741 2.69355 4.24690 2.90347 2.73739 3.18146 2.89801 2.37887 2.77519 2.98518 4.58477 3.61503
0.19547 4.31287 1.80692 0.61958 0.77255 0.59200 0.80569
821 2.83025 5.16804 2.80210 2.06678 4.53254 3.45038 3.67829 3.96004 1.45901 3.46577 4.27803 2.95176 3.90651 2.81101 2.66193 2.41886 3.06277 3.58314 5.59958 4.26549 892 - -
2.68618 4.42225 2.77519 2.73123 3.46354 2.40513 3.72494 3.29354 2.67741 2.69355 4.24690 2.90347 2.73739 3.18146 2.89801 2.37887 2.77519 2.98518 4.58477 3.61503
0.05219 4.14131 3.35397 0.61958 0.77255 0.77522 0.61731
822 2.58773 3.42979 2.73073 2.36457 4.05947 3.36283 3.71169 3.47137 2.54934 3.09302 3.91874 2.78813 3.82427 2.88198 3.00304 1.77220 2.85642 3.13784 5.34766 4.02376 893 - -
2.68620 4.42227 2.77501 2.73125 3.46356 2.40515 3.72496 3.29356 2.67733 2.69357 4.24692 2.90348 2.73741 3.18148 2.89803 2.37889 2.77521 2.98520 4.58479 3.61505

0.15133 2.02118 4.83605 0.32023 1.29455 0.75037 0.63902

823 2.26549 4.49700 3.23363 2.48035 2.23549 3.51648 3.75794 3.03678 2.50065 2.72176 3.60058 3.16804 3.91452 3.01210 2.86182 2.77226 2.86025 2.78879 5.04852 3.78059 895 - -

2.68618 4.42225 2.77519 2.73123 3.46354 2.40513 3.72494 3.29354 2.67741 2.69355 4.24690 2.90347 2.73739 3.18146 2.89801 2.37887 2.77519 2.98518 4.58477 3.61503

0.09239 4.13989 2.62656 0.61958 0.77255 0.77648 0.61623

824 2.89634 4.37591 4.28442 3.75580 3.37569 4.00612 4.47358 2.12783 3.61795 1.33617 3.24239 4.01715 4.39074 3.89755 3.84376 3.34807 2.66656 1.70144 5.16747 3.98927 896 - -

2.68618 4.42225 2.77519 2.73123 3.46354 2.40513 3.72494 3.29354 2.67741 2.69355 4.24690 2.90347 2.73739 3.18146 2.89801 2.37887 2.77519 2.98518 4.58477 3.61503

0.02562 4.07312 4.79546 0.61958 0.77255 0.83251 0.57085

825 2.16185 4.28926 4.05829 3.56210 1.70340 3.71093 4.15361 2.45848 3.46947 2.18961 3.33389 3.77076 4.17638 3.71416 3.69638 3.06215 3.01920 2.15844 4.80160 3.44047 897 - -

2.68618 4.42225 2.77519 2.73123 3.46354 2.40513 3.72494 3.29354 2.67741 2.69355 4.24690 2.90347 2.73739 3.18146 2.89801 2.37887 2.77519 2.98518 4.58477 3.61503

0.06917 4.07312 2.99961 0.61958 0.77255 0.83251 0.57085

826 2.72612 4.86234 3.02826 2.53259 4.05659 3.47079 2.06211 3.54166 2.05212 2.93109 3.96691 3.02049 3.40626 2.81117 2.62478 2.75983 2.95219 3.23124 5.28540 3.96204 898 - -

2.68618 4.42225 2.77519 2.73123 3.46354 2.40513 3.72494 3.29354 2.67741 2.69355 4.24690 2.90347 2.73739 3.18146 2.89801 2.37887 2.77519 2.98518 4.58477 3.61503

0.02674 4.03069 4.75304 0.61958 0.77255 0.86476 0.54672

827 2.57054 4.90569 2.75159 2.13540 4.17714 3.37654 2.02412 3.61477 2.44197 3.20808 4.04639 2.92579 3.84834 2.84599 2.85164 2.72303 2.72942 3.28405 5.41654 4.07373 899 - -

2.68618 4.42225 2.77519 2.73123 3.46354 2.40513 3.72494 3.29354 2.67741 2.69355 4.24690 2.90347 2.73739 3.18146 2.89801 2.37887 2.77519 2.98518 4.58477 3.61503

0.02674 4.03069 4.75304 0.61958 0.77255 0.79211 0.60310

828 2.15452 4.22839 3.55295 3.12281 4.02391 3.11998 4.12323 3.25564 3.09003 3.06917 3.94787 3.34696 3.78854 3.40113 3.42063 1.40444 2.47493 2.34537 5.42338 4.19953 900 - -

2.68618 4.42225 2.77519 2.73123 3.46354 2.40513 3.72494 3.29354 2.67741 2.69355 4.24690 2.90347 2.73739 3.18146 2.89801 2.37887 2.77519 2.98518 4.58477 3.61503

0.02562 4.07312 4.79546 0.61958 0.77255 0.76851 0.62307

829 1.72440 5.08023 1.97149 2.29747 4.44411 3.31035 3.73860 3.90022 2.62043 3.46122 4.27650 2.62755 3.84267 2.71982 3.12864 2.72060 3.02085 3.51194 5.66022 4.25884 901 - -

2.68632 4.42239 2.77533 2.73137 3.46368 2.40526 3.72508 3.29298 2.67683 2.69286 4.24703 2.90360 2.73753 3.18160 2.89814 2.37900 2.77533 2.98519 4.58491 3.61517

0.56697 0.85618 4.83125 0.68006 0.70640 0.74778 0.64134

830 1.57890 4.79765 2.76532 1.86800 4.31024 3.29503 3.83041 3.69643 2.68930 3.33648 4.17416 2.98736 3.85462 3.00723 3.13557 2.50126 2.96706 3.32078 5.57744 4.23881 904 - -

2.68618 4.42225 2.77519 2.73123 3.46354 2.40513 3.72494 3.29354 2.67741 2.69355 4.24690 2.90347 2.73739 3.18146 2.89801 2.37887 2.77519 2.98518 4.58477 3.61503

0.02397 4.13884 4.86118 0.61958 0.77255 0.77742 0.61543

831 2.40118 4.56785 3.13493 2.39093 3.79046 3.47227 3.75390 3.12046 2.48787 2.82942 3.69824 3.11370 3.15992 2.97609 3.02687 2.73716 2.86508 1.95638 5.15628 3.87868 905 - -

2.68618 4.42225 2.77519 2.73123 3.46354 2.40513 3.72494 3.29354 2.67741 2.69355 4.24690 2.90347 2.73739 3.18146 2.89801 2.37887 2.77519 2.98518 4.58477 3.61503

0.02397 4.13884 4.86118 0.61958 0.77255 0.72551 0.66179

832 2.46877 4.26502 3.69779 3.14187 3.47295 3.60994 3.99368 2.29909 3.07335 2.34601 3.43032 3.48495 4.03208 3.36841 3.38757 2.61618 1.91189 2.17183 4.96781 3.74703 906 - -

2.68618 4.42225 2.77519 2.73123 3.46354 2.40513 3.72494 3.29354 2.67741 2.69355 4.24690 2.90347 2.73739 3.18146 2.89801 2.37887 2.77519 2.98518 4.58477 3.61503

0.02332 4.16597 4.88831 0.61958 0.77255 0.75263 0.63700

833 2.61191 4.59430 3.09868 2.46718 3.81451 2.93759 3.74276 3.20910 2.62197 2.87125 3.72739 3.08865 3.47942 2.95812 3.04400 2.57792 2.51170 2.93098 5.17185 2.28212 907 - -

2.68618 4.42225 2.77519 2.73123 3.46354 2.40513 3.72494 3.29354 2.67741 2.69355 4.24690 2.90347 2.73739 3.18146 2.89801 2.37887 2.77519 2.98518 4.58477 3.61503

0.02332 4.16597 4.88831 0.61958 0.77255 0.70111 0.68524

834 2.73190 4.86187 2.80173 2.56290 4.41763 1.36399 3.87637 3.85380 2.42362 3.44314 4.28604 3.04370 3.91251 2.82170 3.02638 2.59574 3.05741 3.46178 5.64104 4.32251 908 - -

2.68618 4.42225 2.77519 2.73123 3.46354 2.40513 3.72494 3.29354 2.67741 2.69355 4.24690 2.90347 2.73739 3.18146 2.89801 2.37887 2.77519 2.98518 4.58477 3.61503
0.02271 4.19218 4.91453 0.61958 0.77255 0.72741 0.66002
835 2.69715 4.29691 3.76879 2.93598 3.40576 3.72953 3.99330 1.68296 3.03248 2.10589 3.35973 3.54251 4.09799 3.38106 2.82168 3.00259 2.92876 2.27450 4.92462 3.71282 909 - -
2.68618 4.42225 2.77519 2.73123 3.46354 2.40513 3.72494 3.29354 2.67741 2.69355 4.24690 2.90347 2.73739 3.18146 2.89801 2.37887 2.77519 2.98518 4.58477 3.61503
0.04953 4.19218 3.40484 0.61958 0.77255 0.72741 0.66002
836 2.71248 4.76788 3.18428 2.61687 3.99379 3.53108 3.34195 3.38787 2.01243 2.00444 3.85328 2.88453 3.91604 2.86288 2.54336 2.65420 2.93234 3.10052 5.25149 3.97233 910 - -
2.68618 4.42225 2.77519 2.73123 3.46354 2.40513 3.72494 3.29354 2.67741 2.69355 4.24690 2.90347 2.73739 3.18146 2.89801 2.37887 2.77519 2.98518 4.58477 3.61503
0.02332 4.16597 4.88831 0.61958 0.77255 0.75263 0.63700
837 2.52320 3.28429 3.40487 2.71017 3.69163 3.40812 3.87351 3.04290 2.83210 2.75641 3.63234 3.25999 3.19805 3.15130 3.20627 1.82364 2.81784 2.34098 5.10103 3.85452 911 - -
2.68618 4.42225 2.77519 2.73123 3.46354 2.40513 3.72494 3.29354 2.67741 2.69355 4.24690 2.90347 2.73739 3.18146 2.89801 2.37887 2.77519 2.98518 4.58477 3.61503
0.05314 4.16597 3.31771 0.61958 0.77255 0.75263 0.63700
838 2.60858 4.47550 3.26936 2.57095 3.62444 3.51690 3.76015 2.99959 2.67202 2.55631 2.87641 3.17669 3.91020 3.01714 3.06110 1.88578 2.84418 2.75600 5.03051 3.49037 912 - -
2.68618 4.42225 2.77519 2.73123 3.46354 2.40513 3.72494 3.29354 2.67741 2.69355 4.24690 2.90347 2.73739 3.18146 2.89801 2.37887 2.77519 2.98518 4.58477 3.61503
0.06476 4.13685 3.06334 0.61958 0.77255 0.67483 0.71180
839 2.72076 5.10040 2.53921 2.21163 4.39286 3.37762 3.67026 3.84677 2.37536 3.09720 4.18280 1.79836 3.83960 2.80410 2.96055 2.38944 2.96459 3.46801 5.57383 4.18364 913 - -
2.68626 4.42233 2.77497 2.73124 3.46362 2.40514 3.72456 3.29362 2.67731 2.69356 4.24698 2.90344 2.73748 3.18155 2.89801 2.37895 2.77521 2.98515 4.58485 3.61489
0.28261 1.43310 4.87695 0.72791 0.65955 0.76317 0.62771
840 2.45582 3.24861 3.83691 3.27085 3.34054 3.62951 4.00140 2.43310 3.18447 2.38669 3.32364 3.56051 4.03923 3.45178 3.44962 2.92030 1.86307 2.14528 4.84507 3.35267 918 - -
2.68618 4.42225 2.77519 2.73123 3.46354 2.40513 3.72494 3.29354 2.67741 2.69355 4.24690 2.90347 2.73739 3.18146 2.89801 2.37887 2.77519 2.98518 4.58477 3.61503
0.07888 4.15461 2.81078 0.61958 0.77255 0.76317 0.62771
841 2.64333 4.69102 3.22055 2.66339 4.05320 3.43939 3.72326 3.14302 2.38662 3.05595 3.90846 3.11278 3.88371 2.90057 1.73973 2.26357 2.70007 3.10827 5.30480 4.03028 919 - -
2.68618 4.42225 2.77519 2.73123 3.46354 2.40513 3.72494 3.29354 2.67741 2.69355 4.24690 2.90347 2.73739 3.18146 2.89801 2.37887 2.77519 2.98518 4.58477 3.61503
0.02491 4.10064 4.82299 0.61958 0.77255 0.81024 0.58834
842 2.12947 4.32258 3.36971 3.23916 4.51592 0.91757 4.40715 3.91901 3.42753 3.65113 4.52572 3.41388 3.81645 3.70859 3.71072 2.57017 2.90472 3.36069 5.82196 4.64009 920 - -
2.68618 4.42225 2.77519 2.73123 3.46354 2.40513 3.72494 3.29354 2.67741 2.69355 4.24690 2.90347 2.73739 3.18146 2.89801 2.37887 2.77519 2.98518 4.58477 3.61503
0.02491 4.10064 4.82299 0.61958 0.77255 0.81024 0.58834
843 2.67292 4.82440 3.04730 2.51122 3.10423 3.46770 3.64816 3.47713 1.71716 3.06885 3.90671 3.01130 3.45720 2.49537 2.74204 2.71197 2.90014 3.16821 5.28863 3.96738 921 - -
2.68618 4.42225 2.77519 2.73123 3.46354 2.40513 3.72494 3.29354 2.67741 2.69355 4.24690 2.90347 2.73739 3.18146 2.89801 2.37887 2.77519 2.98518 4.58477 3.61503
0.02491 4.10064 4.82299 0.61958 0.77255 0.81024 0.58834
844 2.60201 4.80243 2.63003 2.44495 4.27957 2.54694 3.74813 3.71773 2.58061 3.30356 4.11458 2.94786 2.04359 2.90654 2.84461 2.46586 2.90084 3.32830 5.51910 4.17251 922 - -
2.68618 4.42225 2.77519 2.73123 3.46354 2.40513 3.72494 3.29354 2.67741 2.69355 4.24690 2.90347 2.73739 3.18146 2.89801 2.37887 2.77519 2.98518 4.58477 3.61503
0.02491 4.10064 4.82299 0.61958 0.77255 0.81024 0.58834
845 2.63191 4.34620 3.52104 2.96804 3.51740 3.58128 3.86352 2.75507 2.63721 2.41345 3.47245 3.35253 3.50545 3.18448 2.90941 2.86239 2.87491 1.63691 4.96494 3.73555 923 - -
2.68618 4.42225 2.77519 2.73123 3.46354 2.40513 3.72494 3.29354 2.67741 2.69355 4.24690 2.90347 2.73739 3.18146 2.89801 2.37887 2.77519 2.98518 4.58477 3.61503
0.05683 4.10064 3.25239 0.61958 0.77255 0.81024 0.58834

846 2.65183 3.29555 3.30544 2.72627 3.97016 3.46030 3.72409 3.35379 2.36403 2.98659 3.84688 3.15421 3.89786 2.74811 1.68316 2.56792 2.90933 3.05647 5.23523 3.98008 924 - -
2.68618 4.42225 2.77519 2.73123 3.46354 2.40513 3.72494 3.29354 2.67741 2.69355 4.24690 2.90347 2.73739 3.18146 2.89801 2.37887 2.77519 2.98518 4.58477 3.61503
0.02571 4.06952 4.79187 0.61958 0.77255 0.83534 0.56868
847 2.41572 4.42294 3.29447 2.82060 3.72566 3.42750 3.85353 2.96583 2.73718 2.76366 3.66487 2.52868 3.90573 3.11846 2.88742 2.74535 2.85954 1.76408 5.13238 3.87628 925 - -
2.68630 4.42237 2.77532 2.73135 3.46366 2.40525 3.72453 3.29366 2.67681 2.69367 4.24357 2.90348 2.73742 3.18159 2.89813 2.37899 2.77532 2.98531 4.58489 3.61515
0.64534 0.82561 3.28218 0.59850 0.79770 0.77978 0.61343
848 2.82277 5.21172 2.42861 1.87060 4.56166 3.30302 3.74255 4.03690 2.60540 3.57175 4.39775 1.59904 3.29228 2.89787 3.09341 2.77285 3.09821 3.63960 5.74081 4.32495 928 - -
2.68618 4.42225 2.77519 2.73123 3.46354 2.40513 3.72494 3.29354 2.67741 2.69355 4.24690 2.90347 2.73739 3.18146 2.89801 2.37887 2.77519 2.98518 4.58477 3.61503
0.06379 4.07188 3.10652 0.61958 0.77255 0.78458 0.60938
849 2.46742 4.28327 3.23029 2.83046 2.88773 3.58807 3.89119 2.61526 2.95186 2.46766 3.40201 3.36698 3.98572 3.24706 3.28934 2.86339 2.84914 1.72239 4.91156 3.67280 929 - -
2.68618 4.42225 2.77519 2.73123 3.46354 2.40513 3.72494 3.29354 2.67741 2.69355 4.24690 2.90347 2.73739 3.18146 2.89801 2.37887 2.77519 2.98518 4.58477 3.61503
0.02588 4.06302 4.78537 0.61958 0.77255 0.84040 0.56482
850 2.35890 4.23259 3.61490 3.09533 3.52290 2.80379 3.97253 2.74670 3.01718 2.53514 1.97427 3.40905 3.93184 3.32819 3.32676 2.76165 2.64454 2.40157 5.00287 3.78517 930 - -
2.68618 4.42225 2.77519 2.73123 3.46354 2.40513 3.72494 3.29354 2.67741 2.69355 4.24690 2.90347 2.73739 3.18146 2.89801 2.37887 2.77519 2.98518 4.58477 3.61503
0.02588 4.06302 4.78537 0.61958 0.77255 0.78888 0.60578
851 3.31803 4.66688 4.83430 4.30046 3.17398 4.54674 4.90206 2.00683 4.12433 0.85184 2.98664 4.56614 4.77332 4.30661 4.27418 3.91566 3.55811 2.04458 5.25624 4.13553 931 - -
2.68618 4.42225 2.77519 2.73123 3.46354 2.40513 3.72494 3.29354 2.67741 2.69355 4.24690 2.90347 2.73739 3.18146 2.89801 2.37887 2.77519 2.98518 4.58477 3.61503
0.02513 4.09204 4.81438 0.61958 0.77255 0.75333 0.63639
852 2.56904 1.97274 3.34116 2.83391 3.74678 3.42219 3.83816 3.11686 2.44630 2.80504 3.68083 3.23274 3.41677 2.89064 3.03024 2.56187 2.85466 2.84128 5.12636 3.88159 932 - -
2.68618 4.42225 2.77519 2.73123 3.46354 2.40513 3.72494 3.29354 2.67741 2.69355 4.24690 2.90347 2.73739 3.18146 2.89801 2.37887 2.77519 2.98518 4.58477 3.61503
0.02425 4.12716 4.84951 0.61958 0.77255 0.78770 0.60677
853 2.64512 4.72841 3.06933 2.42830 3.97559 1.99354 3.68830 3.38771 2.44648 3.01094 3.85088 3.04192 3.86838 2.86687 2.51642 2.59929 2.88486 3.08589 4.31273 3.95789 933 - -
2.68618 4.42225 2.77519 2.73123 3.46354 2.40513 3.72494 3.29354 2.67741 2.69355 4.24690 2.90347 2.73739 3.18146 2.89801 2.37887 2.77519 2.98518 4.58477 3.61503
0.02425 4.12716 4.84951 0.61958 0.77255 0.78770 0.60677
854 2.61076 3.56758 1.99464 2.45406 4.02452 3.42007 3.38836 3.44705 2.46124 2.87531 3.87916 2.96913 3.82847 2.81341 2.78935 2.55739 2.84869 3.12753 5.30144 3.96750 934 - -
2.68618 4.42225 2.77519 2.73123 3.46354 2.40513 3.72494 3.29354 2.67741 2.69355 4.24690 2.90347 2.73739 3.18146 2.89801 2.37887 2.77519 2.98518 4.58477 3.61503
0.02425 4.12716 4.84951 0.61958 0.77255 0.78770 0.60677
855 2.59672 2.38294 2.84255 2.60310 3.03954 3.49128 3.76763 3.00724 2.71287 2.70415 3.58101 2.81086 3.90012 3.03101 3.11291 2.74908 2.83872 2.75726 5.03229 3.75928 935 - -
2.68618 4.42225 2.77519 2.73123 3.46354 2.40513 3.72494 3.29354 2.67741 2.69355 4.24690 2.90347 2.73739 3.18146 2.89801 2.37887 2.77519 2.98518 4.58477 3.61503
0.05530 4.12716 3.27891 0.61958 0.77255 0.78770 0.60677
856 2.58793 4.91383 2.77553 2.30740 4.17924 3.39589 1.75174 3.64633 2.48416 3.23764 4.08230 2.96386 3.37059 2.89613 2.88300 2.76191 2.99794 3.31569 5.43103 4.08602 936 - -
2.68618 4.42225 2.77519 2.73123 3.46354 2.40513 3.72494 3.29354 2.67741 2.69355 4.24690 2.90347 2.73739 3.18146 2.89801 2.37887 2.77519 2.98518 4.58477 3.61503
0.02501 4.09687 4.81922 0.61958 0.77255 0.64297 0.74597
857 2.65551 4.99956 2.82945 1.76823 3.86106 2.76853 3.64463 3.74009 2.35168 3.29202 4.07921 2.72830 3.82493 2.77292 2.90888 2.42593 2.76797 3.36802 5.48643 4.11213 937 - -
2.68618 4.42225 2.77519 2.73123 3.46354 2.40513 3.72494 3.29354 2.67741 2.69355 4.24690 2.90347 2.73739 3.18146 2.89801 2.37887 2.77519 2.98518 4.58477 3.61503

0.02271 4.19218 4.91453 0.61958 0.77255 0.66240 0.72487
858 2.68536 4.38301 3.48779 2.76979 1.76556 3.63155 3.85527 2.85893 2.71288 2.34397 3.48558 3.15241 4.02361 3.23412 3.20573 2.91114 2.91863 2.64393 4.84728 3.53474 938 - -
2.68665 4.42272 2.77556 2.73170 3.46224 2.40560 3.72542 3.29135 2.67696 2.69402 4.24737 2.90383 2.73787 3.18194 2.89836 2.37873 2.77567 2.98306 4.58524 3.61367
0.42935 1.07306 4.94687 1.86605 0.16810 0.69442 0.69188
859 2.92999 4.33732 4.47497 3.89930 3.27949 4.11414 4.45203 2.08523 3.77782 1.68155 1.90200 4.11611 4.43099 3.97533 3.94504 3.42309 2.65391 1.85069 5.02768 3.87605 947 - -
2.68618 4.42225 2.77519 2.73123 3.46354 2.40513 3.72494 3.29354 2.67741 2.69355 4.24690 2.90347 2.73739 3.18146 2.89801 2.37887 2.77519 2.98518 4.58477 3.61503
0.05004 4.22452 3.37627 0.61958 0.77255 0.69442 0.69188
860 2.65337 4.74908 2.82330 2.62786 4.47877 1.25796 3.97285 3.92924 2.55546 3.53141 4.36859 2.83214 3.89005 3.16834 3.20515 2.53511 3.02949 3.48457 5.72666 4.40949 948 - -
2.68618 4.42225 2.77519 2.73123 3.46354 2.40513 3.72494 3.29354 2.67741 2.69355 4.24690 2.90347 2.73739 3.18146 2.89801 2.37887 2.77519 2.98518 4.58477 3.61503
0.02260 4.19708 4.91943 0.61958 0.77255 0.72255 0.66459
861 2.82023 5.34844 1.58546 1.92891 4.65999 3.35988 3.71156 4.15299 2.40284 3.63785 4.42019 2.63024 3.86594 2.83943 2.80687 2.60504 3.07142 3.72527 5.78414 4.34032 949 - -
2.68618 4.42225 2.77519 2.73123 3.46354 2.40513 3.72494 3.29354 2.67741 2.69355 4.24690 2.90347 2.73739 3.18146 2.89801 2.37887 2.77519 2.98518 4.58477 3.61503
0.04928 4.19708 3.40974 0.61958 0.77255 0.72255 0.66459
862 2.77636 5.25695 2.05330 1.58479 4.56869 3.35010 3.69784 4.04966 2.54538 3.55775 4.34305 2.63148 3.84795 2.82784 3.06862 2.42067 3.02893 3.25796 5.72014 4.28844 950 - -
2.68618 4.42225 2.77519 2.73123 3.46354 2.40513 3.72494 3.29354 2.67741 2.69355 4.24690 2.90347 2.73739 3.18146 2.89801 2.37887 2.77519 2.98518 4.58477 3.61503
0.02320 4.17100 4.89334 0.61958 0.77255 0.74789 0.64124
863 2.47662 3.48067 1.83104 2.45352 4.16487 3.10273 3.42810 3.59201 2.53181 3.18994 4.00156 2.96614 3.83044 2.86122 3.00046 2.49326 2.87922 3.23909 5.42240 4.07910 951 - -
2.68618 4.42225 2.77519 2.73123 3.46354 2.40513 3.72494 3.29354 2.67741 2.69355 4.24690 2.90347 2.73739 3.18146 2.89801 2.37887 2.77519 2.98518 4.58477 3.61503
0.05084 4.17100 3.37763 0.61958 0.77255 0.74789 0.64124
864 2.59550 4.45320 3.04624 2.72886 3.32895 3.50860 3.77152 2.97297 2.46883 2.68907 3.56728 3.18051 3.90672 3.03106 3.10066 2.47527 2.68186 1.94391 5.03622 3.77450 952 - -
2.68618 4.42225 2.77519 2.73123 3.46354 2.40513 3.72494 3.29354 2.67741 2.69355 4.24690 2.90347 2.73739 3.18146 2.89801 2.37887 2.77519 2.98518 4.58477 3.61503
0.02384 4.14400 4.86635 0.61958 0.77255 0.77280 0.61937
865 2.40612 5.10671 1.88707 2.20719 4.40567 3.37504 3.63784 3.55184 2.31612 3.39152 4.17121 2.85497 3.81809 2.46021 2.91821 2.65619 2.92685 3.47298 5.56462 4.16742 953 - -
2.68646 4.41852 2.77557 2.73142 3.46372 2.40488 3.72531 3.29391 2.67615 2.69373 4.24382 2.90283 2.73777 3.18183 2.89814 2.37924 2.77557 2.98555 4.58514 3.61540
0.53380 0.90158 4.86635 1.56242 0.23525 0.77280 0.61937
866 2.60889 4.64475 3.12703 2.48894 3.86407 3.46806 3.69993 3.25292 2.52999 2.59730 3.50520 3.06385 3.49924 2.89184 2.77467 1.89075 2.69984 2.96794 5.19246 3.89763 960 - -
2.68618 4.42225 2.77519 2.73123 3.46354 2.40513 3.72494 3.29354 2.67741 2.69355 4.24690 2.90347 2.73739 3.18146 2.89801 2.37887 2.77519 2.98518 4.58477 3.61503
0.06269 4.16818 3.09468 0.61958 0.77255 0.77280 0.61937
867 1.57484 4.55067 2.63549 2.68491 3.04608 3.40712 3.82862 3.15498 2.76253 2.87854 3.77727 3.13953 3.89332 3.07087 3.18200 2.72519 2.88772 2.88213 5.19259 3.87183 961 - -
2.68618 4.42225 2.77519 2.73123 3.46354 2.40513 3.72494 3.29354 2.67741 2.69355 4.24690 2.90347 2.73739 3.18146 2.89801 2.37887 2.77519 2.98518 4.58477 3.61503
0.05130 4.12968 3.38373 0.61958 0.77255 0.68259 0.70382
868 2.49849 2.07318 3.24192 2.33781 3.80202 3.44921 3.80100 3.16777 2.65467 2.85055 3.71828 3.17415 3.45506 3.03545 2.79971 2.73474 2.86764 2.89102 5.16688 3.90383 962 - -
2.68618 4.42225 2.77519 2.73123 3.46354 2.40513 3.72494 3.29354 2.67741 2.69355 4.24690 2.90347 2.73739 3.18146 2.89801 2.37887 2.77519 2.98518 4.58477 3.61503
0.02318 4.17169 4.89404 0.61958 0.77255 0.76956 0.62216
869 2.68378 5.00681 2.36012 2.36147 4.48059 2.74455 3.75507 3.94697 2.60991 3.49062 4.28396 2.48498 3.47917 2.89769 3.11239 1.66808 2.97550 3.52353 5.67462 4.28140 963 - -

2.68618 4.42225 2.77519 2.73123 3.46354 2.40513 3.72494 3.29354 2.67741 2.69355 4.24690 2.90347 2.73739 3.18146 2.89801 2.37887 2.77519 2.98518 4.58477 3.61503
0.02318 4.17169 4.89404 0.61958 0.77255 0.71804 0.66886
870 2.60817 4.47265 3.29112 2.73184 3.62956 3.25873 3.40213 2.87902 2.69161 2.70147 3.57452 3.18573 3.91529 3.02419 2.95580 1.98690 2.84198 2.42219 5.03039 3.15526 964 - -
2.68618 4.42225 2.77519 2.73123 3.46354 2.40513 3.72494 3.29354 2.67741 2.69355 4.24690 2.90347 2.73739 3.18146 2.89801 2.37887 2.77519 2.98518 4.58477 3.61503
0.02258 4.19776 4.92011 0.61958 0.77255 0.74476 0.64407
871 2.66142 4.89011 3.02809 2.26765 4.15493 3.46618 3.65150 3.57062 2.06385 3.15393 3.44710 2.98939 3.85819 2.79348 2.65278 2.59454 2.04829 3.23751 5.37318 4.04305 965 - -
2.68618 4.42225 2.77519 2.73123 3.46354 2.40513 3.72494 3.29354 2.67741 2.69355 4.24690 2.90347 2.73739 3.18146 2.89801 2.37887 2.77519 2.98518 4.58477 3.61503
0.02211 4.21852 4.94086 0.61958 0.77255 0.68829 0.69803
872 2.68450 5.07532 2.46545 2.26169 4.37950 3.42039 3.64770 3.83296 2.20722 3.36293 4.14047 2.90868 3.84026 2.76809 2.67875 2.53743 1.93208 3.44623 5.53709 4.15529 966 - -
2.68618 4.42225 2.77519 2.73123 3.46354 2.40513 3.72494 3.29354 2.67741 2.69355 4.24690 2.90347 2.73739 3.18146 2.89801 2.37887 2.77519 2.98518 4.58477 3.61503
0.02151 4.24583 4.96818 0.61958 0.77255 0.63542 0.75441
873 2.64916 4.90492 2.84780 2.46662 4.25070 3.42004 3.70377 3.68328 2.39327 3.25855 4.05675 2.98466 2.78392 2.84306 2.82650 1.79577 2.49170 3.31890 5.47075 4.12076 967 - -
2.68618 4.42225 2.77519 2.73123 3.46354 2.40513 3.72494 3.29354 2.67741 2.69355 4.24690 2.90347 2.73739 3.18146 2.89801 2.37887 2.77519 2.98518 4.58477 3.61503
0.02069 4.28440 5.00674 0.61958 0.77255 0.67451 0.71214
874 2.62945 4.10234 2.90206 2.58237 3.73072 3.53259 3.76053 2.81968 2.66000 2.78631 3.64886 3.15606 3.26649 2.99099 2.88890 2.59970 2.86127 2.71228 5.10891 2.33721 968 - -
2.68618 4.42225 2.77519 2.73123 3.46354 2.40513 3.72494 3.29354 2.67741 2.69355 4.24690 2.90347 2.73739 3.18146 2.89801 2.37887 2.77519 2.98518 4.58477 3.61503
0.02069 4.28440 5.00674 0.61958 0.77255 0.67451 0.71214
875 2.53037 4.83214 3.04078 2.29535 4.08608 3.46731 3.68510 3.11576 2.17975 3.10333 3.92012 3.01115 3.86626 2.83798 2.92637 1.88701 2.75925 3.02125 5.34939 4.01949 969 - -
2.68618 4.42225 2.77519 2.73123 3.46354 2.40513 3.72494 3.29354 2.67741 2.69355 4.24690 2.90347 2.73739 3.18146 2.89801 2.37887 2.77519 2.98518 4.58477 3.61503
0.06007 4.28440 3.11175 0.61958 0.77255 0.67451 0.71214
876 2.48272 4.25067 3.68500 3.11566 3.38788 3.63937 3.93374 2.54854 3.04749 2.19331 2.89563 3.46627 3.72723 3.33196 3.35394 2.74201 1.93484 2.38394 4.86820 3.65144 970 - -
2.68618 4.42225 2.77519 2.73123 3.46354 2.40513 3.72494 3.29354 2.67741 2.69355 4.24690 2.90347 2.73739 3.18146 2.89801 2.37887 2.77519 2.98518 4.58477 3.61503
0.04688 4.24583 3.45849 0.61958 0.77255 0.71679 0.67005
877 2.67826 5.04695 2.78202 2.40171 4.37320 3.43396 3.63452 3.81924 1.67508 3.34450 4.12318 2.93266 2.97676 2.75322 2.62758 2.57354 2.63346 3.43362 5.50971 4.14536 971 - -
2.68618 4.42225 2.77519 2.73123 3.46354 2.40513 3.72494 3.29354 2.67741 2.69355 4.24690 2.90347 2.73739 3.18146 2.89801 2.37887 2.77519 2.98518 4.58477 3.61503
0.08912 4.22101 2.65096 0.61958 0.77255 0.69076 0.69554
878 2.71937 4.24374 3.96282 3.10692 3.21259 3.79182 4.05405 2.37279 3.27490 1.45506 3.22460 3.68108 4.14938 3.54197 3.51455 3.07343 2.95062 2.24984 4.79897 2.81819 972 - -
2.68618 4.42225 2.77519 2.73123 3.46354 2.40513 3.72494 3.29354 2.67741 2.69355 4.24690 2.90347 2.73739 3.18146 2.89801 2.37887 2.77519 2.98518 4.58477 3.61503
0.02294 4.18194 4.90428 0.61958 0.77255 0.77992 0.61331
879 2.72821 4.24859 4.03753 3.48766 1.92655 2.78414 4.05556 2.54070 3.38271 1.83026 3.26435 3.72978 4.16674 3.62232 3.60328 3.08166 2.97653 2.27052 4.71245 3.39626 973 - -
2.68619 4.42226 2.77521 2.73115 3.46355 2.40514 3.72496 3.29355 2.67742 2.69356 4.24691 2.90341 2.73741 3.18148 2.89802 2.37888 2.77514 2.98520 4.58478 3.61504
0.25054 2.37139 2.05364 0.37001 1.17353 0.77992 0.61331
880 2.56935 4.52869 3.26035 2.77650 3.71045 3.52200 3.78050 3.02611 2.25334 1.73531 3.65370 2.93723 3.94586 3.03270 2.86128 2.83212 2.92322 2.79125 5.09513 3.83823 975 - -
2.68618 4.42225 2.77519 2.73123 3.46354 2.40513 3.72494 3.29354 2.67741 2.69355 4.24690 2.90347 2.73739 3.18146 2.89801 2.37887 2.77519 2.98518 4.58477 3.61503
0.02614 4.05313 4.77548 0.61958 0.77255 0.88613 0.53145

881 3.41737 4.70647 4.66113 4.25423 1.11232 4.32175 3.63318 3.00413 4.07439 2.12556 3.59132 4.10241 4.62131 4.09511 4.12112 3.66711 3.63843 2.96679 2.66967 2.11488 976 - -

2.68647 4.42049 2.77560 2.73163 3.46382 2.40553 3.72481 3.29348 2.67781 2.69304 4.24385 2.90387 2.73704 3.18187 2.89817 2.37894 2.77547 2.98312 4.58517 3.61543

0.62782 0.78130 4.77548 1.61579 0.22156 0.62797 0.76287

882 2.49750 4.54343 2.82726 2.65486 3.51298 3.50621 3.48434 2.97182 2.63676 2.77658 3.63821 2.94373 3.58262 2.96706 3.05613 2.63607 2.70143 2.04034 5.09794 3.82116 986 - -

2.68618 4.42225 2.77519 2.73123 3.46354 2.40513 3.72494 3.29354 2.67741 2.69355 4.24690 2.90347 2.73739 3.18146 2.89801 2.37887 2.77519 2.98518 4.58477 3.61503

0.02238 4.20642 4.92876 0.61958 0.77255 0.75667 0.63342

883 2.49802 4.25949 3.66373 3.12262 3.47737 2.86362 3.97266 1.78202 3.05587 2.43316 3.44672 3.45729 3.52071 3.35133 3.36547 2.85638 2.40945 2.48639 4.95297 3.73423 987 - -

2.68618 4.42225 2.77519 2.73123 3.46354 2.40513 3.72494 3.29354 2.67741 2.69355 4.24690 2.90347 2.73739 3.18146 2.89801 2.37887 2.77519 2.98518 4.58477 3.61503

0.02238 4.20642 4.92876 0.61958 0.77255 0.75667 0.63342

884 2.58343 4.64864 3.12340 2.59519 3.97229 3.41165 3.74525 3.18131 2.57988 3.01099 3.84606 2.87602 3.53753 2.79734 3.01283 1.76001 2.43007 2.92633 5.28580 3.98431 988 - -

2.68618 4.42225 2.77519 2.73123 3.46354 2.40513 3.72494 3.29354 2.67741 2.69355 4.24690 2.90347 2.73739 3.18146 2.89801 2.37887 2.77519 2.98518 4.58477 3.61503

0.02238 4.20642 4.92876 0.61958 0.77255 0.70590 0.68055

885 2.68842 4.97726 2.96021 2.36807 4.29951 2.13529 3.66505 3.72950 2.19788 3.28266 4.07993 2.97276 3.86327 2.79704 2.41689 2.47359 2.64019 3.36708 5.47172 4.12854 989 - -

2.68618 4.42225 2.77519 2.73123 3.46354 2.40513 3.72494 3.29354 2.67741 2.69355 4.24690 2.90347 2.73739 3.18146 2.89801 2.37887 2.77519 2.98518 4.58477 3.61503

0.11007 4.23123 2.41131 0.61958 0.77255 0.73193 0.65581

886 2.33276 4.39987 3.05544 2.82873 2.01031 3.51008 3.82686 2.92807 2.83976 2.64929 3.54800 2.84717 3.93840 3.14338 3.21434 2.79343 2.86118 2.58236 4.95891 3.66413 990 - -

2.68618 4.42225 2.77519 2.73123 3.46354 2.40513 3.72494 3.29354 2.67741 2.69355 4.24690 2.90347 2.73739 3.18146 2.89801 2.37887 2.77519 2.98518 4.58477 3.61503

0.02382 4.14498 4.86732 0.61958 0.77255 0.81301 0.58613

887 2.51225 4.31835 3.46606 2.75209 3.48714 3.55663 3.85541 2.78048 2.86997 2.54720 2.92786 3.07850 3.95863 3.18638 3.21459 2.59563 2.83949 1.78242 4.93599 3.70220 991 - -

2.68622 4.42121 2.77524 2.73127 3.46358 2.40517 3.72499 3.29348 2.67745 2.69344 4.24694 2.90351 2.73744 3.18150 2.89805 2.37891 2.77506 2.98513 4.58481 3.61507

0.30956 1.35273 4.86732 0.20629 1.67983 0.68774 0.69858

888 2.95215 4.55333 4.07081 3.73395 1.01584 3.77213 3.93697 3.08079 3.67071 2.65555 3.78766 3.83507 4.30760 3.88670 3.86111 2.53096 3.29353 2.91096 4.33451 2.71023 993 - -

2.68618 4.42225 2.77519 2.73123 3.46354 2.40513 3.72494 3.29354 2.67741 2.69355 4.24690 2.90347 2.73739 3.18146 2.89801 2.37887 2.77519 2.98518 4.58477 3.61503

0.02228 4.21120 4.93355 0.61958 0.77255 0.75200 0.63757

889 2.47566 3.22056 3.98036 3.41678 3.32801 3.62266 4.06114 2.34934 3.31717 2.39117 3.32016 3.64465 3.56461 3.56198 3.54242 2.59582 2.67535 1.63061 4.83551 3.63760 994 - -

2.68618 4.42225 2.77519 2.73123 3.46354 2.40513 3.72494 3.29354 2.67741 2.69355 4.24690 2.90347 2.73739 3.18146 2.89801 2.37887 2.77519 2.98518 4.58477 3.61503

0.05122 4.21120 3.34963 0.61958 0.77255 0.70009 0.68625

890 2.61778 5.09662 2.32294 2.34624 4.57701 2.24297 3.79624 4.05207 2.68016 3.58815 4.38714 1.77981 3.86678 2.94313 3.19678 2.42168 3.04511 3.61935 5.76733 4.35717 995 - -

2.68618 4.42225 2.77519 2.73123 3.46354 2.40513 3.72494 3.29354 2.67741 2.69355 4.24690 2.90347 2.73739 3.18146 2.89801 2.37887 2.77519 2.98518 4.58477 3.61503

0.02233 4.20888 4.93123 0.61958 0.77255 0.75427 0.63555

891 2.68531 4.69065 3.13427 1.98503 3.89891 3.52046 3.73610 2.11280 2.44487 2.91736 3.78243 3.10686 3.92056 2.93392 2.72164 2.77546 2.79276 2.96371 5.22472 3.94111 996 - -

2.68618 4.42225 2.77519 2.73123 3.46354 2.40513 3.72494 3.29354 2.67741 2.69355 4.24690 2.90347 2.73739 3.18146 2.89801 2.37887 2.77519 2.98518 4.58477 3.61503

0.02233 4.20888 4.93123 0.61958 0.77255 0.75427 0.63555

892 2.64230 4.81221 2.86184 2.31388 4.05797 2.06491 3.68210 3.25799 2.41605 2.87769 3.90370 2.98975 3.85517 2.84225 2.94614 2.68219 2.59806 3.14682 5.33392 4.00486 997 - -

2.68618 4.42225 2.77519 2.73123 3.46354 2.40513 3.72494 3.29354 2.67741 2.69355 4.24690 2.90347 2.73739 3.18146 2.89801 2.37887 2.77519 2.98518 4.58477 3.61503

0.02233 4.20888 4.93123 0.61958 0.77255 0.75427 0.63555

893 2.50662 4.73374 3.02061 2.31450 4.07494 3.40171 3.73881 3.47511 2.55943 2.93353 3.93061 3.03385 2.17155 2.90888 3.00163 2.45056 2.41969 3.14519 5.36160 4.04458 998 - -

2.68618 4.42225 2.77519 2.73123 3.46354 2.40513 3.72494 3.29354 2.67741 2.69355 4.24690 2.90347 2.73739 3.18146 2.89801 2.37887 2.77519 2.98518 4.58477 3.61503

0.02233 4.20888 4.93123 0.61958 0.77255 0.75427 0.63555

894 2.43933 4.94897 2.95056 2.35581 4.26530 3.00811 3.65734 3.69265 1.65536 3.25509 4.05238 2.84839 3.84839 2.79040 2.82325 2.67289 2.77422 3.09735 5.45220 4.10594 999 - -

2.68637 4.42244 2.77524 2.73099 3.46373 2.40532 3.72514 3.29373 2.67718 2.69367 4.24668 2.90334 2.73739 3.18151 2.89765 2.37883 2.77539 2.98505 4.58496 3.61474

0.31194 1.34417 4.93123 1.29597 0.31970 0.59250 0.80507

895 2.64853 4.98963 2.06556 2.40210 4.27596 3.43680 3.64520 3.42745 2.34624 3.27251 4.05474 2.83312 3.23958 2.76899 2.78889 2.32502 2.88201 2.94361 5.46766 4.09793 1006 - -

2.68618 4.42225 2.77519 2.73123 3.46354 2.40513 3.72494 3.29354 2.67741 2.69355 4.24690 2.90347 2.73739 3.18146 2.89801 2.37887 2.77519 2.98518 4.58477 3.61503

0.18712 4.29001 1.85181 0.61958 0.77255 0.66807 0.71887

896 2.63926 4.74958 3.05618 2.60242 4.21352 3.37490 3.75490 3.61744 2.21384 3.21796 4.04950 3.06532 3.86694 2.92190 2.65772 1.53258 2.70873 3.26065 5.43320 4.14026 1007 - -

2.68618 4.42225 2.77519 2.73123 3.46354 2.40513 3.72494 3.29354 2.67741 2.69355 4.24690 2.90347 2.73739 3.18146 2.89801 2.37887 2.77519 2.98518 4.58477 3.61503

0.02425 4.12714 4.84949 0.61958 0.77255 0.60287 0.79238

897 2.64483 4.95938 2.69083 2.24615 4.24256 3.42895 3.64732 3.68063 2.42321 3.04491 4.03281 2.93760 3.58663 2.77661 2.73721 2.25190 2.07070 3.31916 5.44732 4.08382 1008 - -

2.68618 4.42225 2.77519 2.73123 3.46354 2.40513 3.72494 3.29354 2.67741 2.69355 4.24690 2.90347 2.73739 3.18146 2.89801 2.37887 2.77519 2.98518 4.58477 3.61503

0.04625 4.25111 3.47540 0.61958 0.77255 0.71120 0.67541

898 2.65443 4.88522 2.98164 2.46536 3.78990 3.44245 3.45847 3.59611 2.43777 3.17884 3.98810 2.29207 3.85507 2.81764 2.59516 1.88106 2.89362 3.25508 5.37969 4.02857 1009 - -

2.68618 4.42225 2.77519 2.73123 3.46354 2.40513 3.72494 3.29354 2.67741 2.69355 4.24690 2.90347 2.73739 3.18146 2.89801 2.37887 2.77519 2.98518 4.58477 3.61503

0.05040 4.22678 3.36521 0.61958 0.77255 0.73646 0.65164

899 2.53059 4.81161 2.79019 2.30097 4.03676 3.44543 3.69359 3.44461 2.52252 1.97953 3.89555 2.64935 3.55646 2.70683 2.97742 2.70364 2.89772 3.13687 5.32656 3.99993 1010 - -

2.68618 4.42225 2.77519 2.73123 3.46354 2.40513 3.72494 3.29354 2.67741 2.69355 4.24690 2.90347 2.73739 3.18146 2.89801 2.37887 2.77519 2.98518 4.58477 3.61503

0.02255 4.19894 4.92128 0.61958 0.77255 0.76389 0.62708

900 2.60705 4.44318 2.78045 2.76011 3.60338 3.26954 3.78352 2.83175 2.73249 1.94061 3.54450 3.20811 3.92448 3.05306 3.12583 2.65710 2.59895 2.61551 5.01500 3.53504 1011 - -

2.68617 4.42243 2.77526 2.73141 3.46359 2.40525 3.72512 3.29372 2.67750 2.69366 4.24707 2.90337 2.73757 3.18121 2.89811 2.37894 2.77442 2.98501 4.58495 3.61465

0.61629 0.79238 4.92128 0.76179 0.62892 0.76389 0.62708

901 2.42661 4.79479 3.00160 2.36352 4.04655 3.43906 3.67189 3.45949 2.47658 3.07030 3.89020 2.99047 3.49520 2.60112 2.93028 1.86089 2.86165 2.98778 5.32026 3.64629 1022 - -

2.68618 4.42225 2.77519 2.73123 3.46354 2.40513 3.72494 3.29354 2.67741 2.69355 4.24690 2.90347 2.73739 3.18146 2.89801 2.37887 2.77519 2.98518 4.58477 3.61503

0.14335 4.19894 2.13250 0.61958 0.77255 0.76389 0.62708

902 2.63812 4.24223 3.71629 3.15299 3.10308 3.66149 3.93624 2.44217 2.83543 1.60978 3.29185 3.49326 4.03886 3.34914 3.32665 2.62215 2.87419 2.40289 4.82465 3.59238 1023 - -

2.68618 4.42225 2.77519 2.73123 3.46354 2.40513 3.72494 3.29354 2.67741 2.69355 4.24690 2.90347 2.73739 3.18146 2.89801 2.37887 2.77519 2.98518 4.58477 3.61503

0.02541 4.08099 4.80334 0.61958 0.77255 0.81443 0.58500

903 2.66552 4.84315 2.80784 2.47403 4.16203 3.33621 2.83205 3.71691 2.55707 3.29863 4.12835 2.52373 3.85221 2.92715 2.98098 1.61668 2.95572 3.34671 5.43921 4.06136 1024 - -

2.68618 4.42225 2.77519 2.73123 3.46354 2.40513 3.72494 3.29354 2.67741 2.69355 4.24690 2.90347 2.73739 3.18146 2.89801 2.37887 2.77519 2.98518 4.58477 3.61503

0.02470 4.10908 4.83143 0.61958 0.77255 0.84303 0.56283

904 2.45200 4.44731 3.28665 2.59644 3.64396 3.50303 3.77721 2.81028 2.69563 2.49812 3.57217 3.01611 3.90564 3.03308 3.08672 2.75034 1.96900 2.55582 5.04970 3.78942 1025 - -

2.68618 4.42225 2.77519 2.73123 3.46354 2.40513 3.72494 3.29354 2.67741 2.69355 4.24690 2.90347 2.73739 3.18146 2.89801 2.37887 2.77519 2.98518 4.58477 3.61503
0.02470 4.10908 4.83143 0.61958 0.77255 0.78656 0.60772
905 2.63962 4.79832 3.02574 2.36812 4.04384 3.45164 3.65409 3.45231 2.08690 3.05904 3.88339 2.85743 3.84947 2.81272 2.82972 2.58793 2.01101 3.13828 4.50517 3.98421 1026 - -
2.68618 4.42225 2.77519 2.73123 3.46354 2.40513 3.72494 3.29354 2.67741 2.69355 4.24690 2.90347 2.73739 3.18146 2.89801 2.37887 2.77519 2.98518 4.58477 3.61503
0.02395 4.13951 4.86186 0.61958 0.77255 0.61162 0.78192
906 2.62611 4.66304 3.11606 2.49391 3.90314 3.47058 3.74021 2.72311 2.59152 2.94455 3.78965 2.61020 3.50765 2.93427 3.02510 2.49614 1.97832 3.00089 5.23606 3.93857 1027 - -
2.68620 4.42246 2.77530 2.73120 3.46358 2.40534 3.72446 3.29321 2.67741 2.69353 4.24711 2.90346 2.73754 3.18141 2.89822 2.37840 2.77541 2.98508 4.58498 3.61524
0.27719 1.44746 4.97345 1.42267 0.27585 0.57660 0.82509
907 2.50509 4.53971 3.27861 2.62707 3.76465 3.24462 3.81630 3.13271 2.72485 2.44504 3.68700 3.04804 3.93436 3.05572 3.13121 1.83399 2.88111 2.38055 5.14804 3.87915 1036 - -
2.68618 4.42225 2.77519 2.73123 3.46354 2.40513 3.72494 3.29354 2.67741 2.69355 4.24690 2.90347 2.73739 3.18146 2.89801 2.37887 2.77519 2.98518 4.58477 3.61503
0.09775 4.31547 2.52874 0.61958 0.77255 0.63783 0.75171
908 2.68573 5.12415 2.51724 2.19042 4.43366 2.98538 2.27961 3.90044 2.31702 3.41485 4.18279 2.88114 3.17702 2.75903 2.91112 2.57274 2.80649 3.49729 5.57789 4.17840 1037 - -
2.68618 4.42225 2.77519 2.73123 3.46354 2.40513 3.72494 3.29354 2.67741 2.69355 4.24690 2.90347 2.73739 3.18146 2.89801 2.37887 2.77519 2.98518 4.58477 3.61503
0.02165 4.23937 4.96172 0.61958 0.77255 0.72354 0.66365
909 2.34114 4.49235 3.28698 2.47409 3.45931 3.53802 3.77211 3.02838 2.55806 1.98102 3.36309 3.01749 3.92313 3.02430 3.09547 2.63912 2.74855 2.77950 5.05331 3.78983 1038 - -
2.68618 4.42225 2.77519 2.73123 3.46354 2.40513 3.72494 3.29354 2.67741 2.69355 4.24690 2.90347 2.73739 3.18146 2.89801 2.37887 2.77519 2.98518 4.58477 3.61503
0.02165 4.23937 4.96172 0.61958 0.77255 0.72354 0.66365
910 2.70210 5.10452 2.64729 2.24021 4.41148 3.40834 3.66166 3.86967 2.35413 3.39718 4.17629 2.65926 2.19448 2.78429 2.77582 2.24647 2.94111 3.47885 5.57092 4.18110 1039 - -
2.68618 4.42225 2.77519 2.73123 3.46354 2.40513 3.72494 3.29354 2.67741 2.69355 4.24690 2.90347 2.73739 3.18146 2.89801 2.37887 2.77519 2.98518 4.58477 3.61503
0.02165 4.23937 4.96172 0.61958 0.77255 0.72354 0.66365
911 2.57558 3.24107 3.79050 3.23800 3.46493 3.55362 4.02859 2.42498 3.16110 2.52046 3.44147 3.52846 2.02995 3.43923 3.44815 2.70306 2.72721 2.17073 4.94989 3.73890 1040 - -
2.68618 4.42225 2.77519 2.73123 3.46354 2.40513 3.72494 3.29354 2.67741 2.69355 4.24690 2.90347 2.73739 3.18146 2.89801 2.37887 2.77519 2.98518 4.58477 3.61503
0.04719 4.23937 3.45203 0.61958 0.77255 0.72354 0.66365
912 1.96364 4.51596 3.17870 2.79123 4.34728 2.59304 3.99526 3.78259 2.87442 3.40221 4.20865 2.82238 1.89639 3.18471 3.29525 2.17571 2.85422 3.31245 5.62822 4.33918 1041 - -
2.68618 4.42225 2.77519 2.73123 3.46354 2.40513 3.72494 3.29354 2.67741 2.69355 4.24690 2.90347 2.73739 3.18146 2.89801 2.37887 2.77519 2.98518 4.58477 3.61503
0.02220 4.21438 4.93673 0.61958 0.77255 0.69996 0.68638
913 3.14062 4.55953 4.38041 3.90126 1.11984 4.11496 3.80561 2.92394 3.76183 2.16437 3.58210 3.96501 4.46525 3.91512 3.90836 3.02380 3.37499 2.79916 4.10662 2.31876 1042 - -
2.68618 4.42225 2.77519 2.73123 3.46354 2.40513 3.72494 3.29354 2.67741 2.69355 4.24690 2.90347 2.73739 3.18146 2.89801 2.37887 2.77519 2.98518 4.58477 3.61503
0.17354 4.23809 1.93184 0.61958 0.77255 0.72488 0.66239
914 2.85575 4.36597 4.08148 3.50989 2.85865 3.94363 4.20351 2.21475 3.40231 1.32046 3.08191 3.82133 4.26720 2.90470 3.64439 3.22842 3.08366 2.28534 4.89781 3.70747 1043 - -
2.68618 4.42225 2.77519 2.73123 3.46354 2.40513 3.72494 3.29354 2.67741 2.69355 4.24690 2.90347 2.73739 3.18146 2.89801 2.37887 2.77519 2.98518 4.58477 3.61503
0.02519 4.08973 4.81208 0.61958 0.77255 0.85842 0.55135
915 2.43499 4.50775 3.16131 2.50470 3.83181 3.39212 3.79415 2.67929 2.67312 2.86829 3.73952 3.12852 3.86280 3.01836 3.07508 1.75617 2.71809 2.85645 5.20054 3.92517 1044 - -
2.68618 4.42225 2.77519 2.73123 3.46354 2.40513 3.72494 3.29354 2.67741 2.69355 4.24690 2.90347 2.73739 3.18146 2.89801 2.37887 2.77519 2.98518 4.58477 3.61503
0.02519 4.08973 4.81208 0.61958 0.77255 0.85842 0.55135

916 2.03418 4.38233 3.29069 2.85534 4.02370 3.21138 3.95466 3.33121 2.85113 3.07149 3.93044 2.59999 3.80986 3.18660 3.22904 2.57875 1.66205 2.79164 5.38167 4.12217 1045 - -

2.68618 4.42225 2.77519 2.73123 3.46354 2.40513 3.72494 3.29354 2.67741 2.69355 4.24690 2.90347 2.73739 3.18146 2.89801 2.37887 2.77519 2.98518 4.58477 3.61503

0.02519 4.08973 4.81208 0.61958 0.77255 0.85842 0.55135

917 1.50094 4.44927 3.16572 2.79183 4.16130 3.18900 3.94441 3.54165 2.52527 3.21397 4.05641 3.16358 3.80413 3.15902 3.16376 1.90530 2.83399 3.13979 5.47471 4.20629 1046 - -

2.68618 4.42225 2.77519 2.73123 3.46354 2.40513 3.72494 3.29354 2.67741 2.69355 4.24690 2.90347 2.73739 3.18146 2.89801 2.37887 2.77519 2.98518 4.58477 3.61503

0.04944 4.08973 3.45781 0.61958 0.77255 0.63942 0.74993

918 2.73034 4.85212 3.05481 2.44961 4.12506 3.49544 3.70630 3.25637 2.26495 3.12247 3.96410 3.05822 1.80694 2.87187 2.61711 2.77537 2.96335 3.20778 5.35839 4.06230 1047 - -

2.68618 4.42225 2.77519 2.73123 3.46354 2.40513 3.72494 3.29354 2.67741 2.69355 4.24690 2.90347 2.73739 3.18146 2.89801 2.37887 2.77519 2.98518 4.58477 3.61503

0.02268 4.19354 4.91588 0.61958 0.77255 0.65638 0.73132

919 1.70280 4.85163 2.55432 2.48113 4.29817 3.35851 3.75351 3.73583 2.57362 3.31555 4.11382 2.68435 3.53353 2.89957 3.04735 2.39450 2.54705 3.34724 5.52875 4.17669 1048 - -

2.68618 4.42225 2.77519 2.73123 3.46354 2.40513 3.72494 3.29354 2.67741 2.69355 4.24690 2.90347 2.73739 3.18146 2.89801 2.37887 2.77519 2.98518 4.58477 3.61503

0.02141 4.25018 4.97253 0.61958 0.77255 0.63082 0.75962

920 2.63600 4.56161 3.25529 2.69644 3.74003 3.53662 3.49075 2.70127 2.66699 2.70058 3.51462 2.80327 2.29343 3.00232 3.08052 2.44375 2.86990 2.70371 5.11827 3.84333 1049 - -

2.68618 4.42225 2.77519 2.73123 3.46354 2.40513 3.72494 3.29354 2.67741 2.69355 4.24690 2.90347 2.73739 3.18146 2.89801 2.37887 2.77519 2.98518 4.58477 3.61503

0.26289 4.30241 1.52491 0.61958 0.77255 0.66971 0.71714

921 1.55252 4.49217 3.09570 2.68839 4.14480 2.68713 3.86547 3.55983 2.71456 3.19402 4.02098 2.79898 3.78412 3.06207 2.85778 2.35282 2.81323 3.15851 5.44226 4.15774 1050 - -

2.68618 4.42225 2.77519 2.73123 3.46354 2.40513 3.72494 3.29354 2.67741 2.69355 4.24690 2.90347 2.73739 3.18146 2.89801 2.37887 2.77519 2.98518 4.58477 3.61503

0.02582 4.06533 4.78768 0.61958 0.77255 0.89004 0.52872

922 2.66181 4.80819 2.76003 2.48474 4.30314 3.29199 3.78659 3.74238 2.40815 3.33758 4.17504 2.53853 1.73499 2.96365 2.96504 2.60056 2.97302 3.36199 5.53771 4.20855 1051 - -

2.68618 4.42225 2.77519 2.73123 3.46354 2.40513 3.72494 3.29354 2.67741 2.69355 4.24690 2.90347 2.73739 3.18146 2.89801 2.37887 2.77519 2.98518 4.58477 3.61503

0.02582 4.06533 4.78768 0.61958 0.77255 0.89004 0.52872

923 2.61090 4.39183 3.48610 2.94741 3.54485 3.52869 3.90872 2.60797 2.85564 2.34277 3.20835 3.34035 3.97159 3.20546 3.20435 1.67872 2.88432 2.55204 5.04632 3.77950 1052 - -

2.68633 4.42240 2.77535 2.73130 3.46369 2.40523 3.72509 3.29355 2.67756 2.69281 4.24705 2.90362 2.73669 3.18151 2.89816 2.37890 2.77535 2.98521 4.58492 3.61518

0.67851 0.72506 4.78768 0.68101 0.70543 0.89004 0.52872

924 2.72160 4.33127 3.79586 3.23214 3.31234 3.71887 4.00041 2.58685 2.99363 1.39257 2.97900 3.56387 4.10182 3.38613 3.01725 2.84572 2.96344 2.46026 4.90044 3.67830 1055 - -

2.68618 4.42225 2.77519 2.73123 3.46354 2.40513 3.72494 3.29354 2.67741 2.69355 4.24690 2.90347 2.73739 3.18146 2.89801 2.37887 2.77519 2.98518 4.58477 3.61503

0.02582 4.06533 4.78768 0.61958 0.77255 0.37515 1.16214

925 2.66221 4.14000 3.15911 2.33808 3.67622 3.52649 3.51479 3.31832 2.48060 2.86687 3.80322 2.86099 2.17433 2.92850 3.02533 2.74034 2.89387 2.59882 5.24979 3.94717 1056 - -

2.68684 4.42301 2.77472 2.73134 3.46403 2.40588 3.72515 3.29334 2.67665 2.69324 4.24587 2.90356 2.73663 3.18166 2.89852 2.37884 2.77488 2.98506 4.58374 3.61579

0.49400 1.02993 3.41781 2.06246 0.13598 0.56632 0.83843

926 2.72422 5.21343 2.15086 1.71155 4.54034 3.44378 3.52577 3.49687 2.31810 3.51008 4.26302 2.80941 3.86599 2.51205 2.93186 2.68377 2.84785 3.59508 5.65227 4.24080 1068 - -

2.68618 4.42225 2.77519 2.73123 3.46354 2.40513 3.72494 3.29354 2.67741 2.69355 4.24690 2.90347 2.73739 3.18146 2.89801 2.37887 2.77519 2.98518 4.58477 3.61503

0.09715 4.39119 2.52325 0.61958 0.77255 0.60315 0.79205

927 2.66029 4.19253 3.93522 3.35540 3.30212 3.72971 4.02062 1.78126 3.23285 1.90298 3.29315 3.63474 4.10164 3.22628 3.19489 2.86365 2.70647 2.25401 4.80928 3.60919 1069 - -

2.68618 4.42225 2.77519 2.73123 3.46354 2.40513 3.72494 3.29354 2.67741 2.69355 4.24690 2.90347 2.73739 3.18146 2.89801 2.37887 2.77519 2.98518 4.58477 3.61503

0.02008 4.31411 5.03646 0.61958 0.77255 0.69954 0.68679

928 2.65546 4.91116 2.49514 2.35143 4.16529 3.46349 3.66927 3.21266 2.39867 2.21978 3.97812 2.58854 3.85915 2.70719 2.81359 2.67336 2.79129 3.25184 5.40126 4.05334 1070 - -

2.68618 4.42225 2.77519 2.73123 3.46354 2.40513 3.72494 3.29354 2.67741 2.69355 4.24690 2.90347 2.73739 3.18146 2.89801 2.37887 2.77519 2.98518 4.58477 3.61503

0.09402 4.31411 2.57230 0.61958 0.77255 0.64398 0.74485

929 2.57135 4.41493 3.41348 2.85212 3.29015 3.57627 3.83225 2.60615 2.60814 2.44103 3.52130 3.28078 2.14062 3.12562 3.17557 2.70760 2.75660 2.68067 4.99832 3.75152 1071 - -

2.68618 4.42225 2.77519 2.73123 3.46354 2.40513 3.72494 3.29354 2.67741 2.69355 4.24690 2.90347 2.73739 3.18146 2.89801 2.37887 2.77519 2.98518 4.58477 3.61503

0.09832 4.26795 2.53036 0.61958 0.77255 0.74998 0.63937

930 2.60255 4.31891 3.50906 2.94877 3.22704 3.57515 3.86284 2.55113 2.89552 2.18615 3.43942 3.14656 3.97022 3.20133 3.24213 1.97744 2.59329 2.56924 4.92144 3.68309 1072 - -

2.68618 4.42225 2.77519 2.73123 3.46354 2.40513 3.72494 3.29354 2.67741 2.69355 4.24690 2.90347 2.73739 3.18146 2.89801 2.37887 2.77519 2.98518 4.58477 3.61503

0.06215 4.19233 3.09777 0.61958 0.77255 0.82307 0.57818

931 2.56564 4.27640 3.80565 3.24401 3.38474 3.72515 4.03152 2.27308 2.87819 1.56143 3.33112 3.57703 4.10572 3.43264 3.38527 3.00907 2.55347 2.32009 4.92861 3.72282 1073 - -

2.68618 4.42225 2.77519 2.73123 3.46354 2.40513 3.72494 3.29354 2.67741 2.69355 4.24690 2.90347 2.73739 3.18146 2.89801 2.37887 2.77519 2.98518 4.58477 3.61503

0.02361 4.15379 4.87614 0.61958 0.77255 0.74722 0.64185

932 2.55012 4.87874 2.77985 2.35512 4.15090 3.42529 3.66145 3.39905 2.35413 3.16364 3.96950 2.95640 2.35298 2.80506 2.92229 2.55989 2.36781 3.23387 5.39185 4.04502 1074 - -

2.68618 4.42225 2.77519 2.73123 3.46354 2.40513 3.72494 3.29354 2.67741 2.69355 4.24690 2.90347 2.73739 3.18146 2.89801 2.37887 2.77519 2.98518 4.58477 3.61503

0.08264 4.21082 2.74132 0.61958 0.77255 0.80619 0.59160

933 1.86139 4.84491 2.90682 2.36212 4.13248 3.41283 3.68335 3.54466 2.34094 2.95718 3.96822 2.40255 3.84784 2.83987 2.91088 2.58077 2.89853 3.21368 5.38387 4.05154 1075 - -

2.68618 4.42225 2.77519 2.73123 3.46354 2.40513 3.72494 3.29354 2.67741 2.69355 4.24690 2.90347 2.73739 3.18146 2.89801 2.37887 2.77519 2.98518 4.58477 3.61503

0.02365 4.15184 4.87418 0.61958 0.77255 0.85807 0.55161

934 2.48382 4.65495 3.13914 2.62372 4.00888 3.40099 3.49346 3.41825 2.52039 3.04937 3.88916 3.08698 3.86165 2.93267 2.56142 1.63713 2.87262 2.86687 5.30202 4.00624 1076 - -

2.68618 4.42225 2.77519 2.73123 3.46354 2.40513 3.72494 3.29354 2.67741 2.69355 4.24690 2.90347 2.73739 3.18146 2.89801 2.37887 2.77519 2.98518 4.58477 3.61503

0.04478 4.15184 3.57363 0.61958 0.77255 0.85807 0.55161

935 2.34016 4.30342 3.35094 3.15088 4.49347 2.31513 4.31476 3.97793 3.30160 3.64384 4.46548 3.34617 3.78159 3.58323 3.62121 0.97471 2.83842 3.37687 5.80754 4.57818 1077 - -

2.68618 4.42225 2.77519 2.73123 3.46354 2.40513 3.72494 3.29354 2.67741 2.69355 4.24690 2.90347 2.73739 3.18146 2.89801 2.37887 2.77519 2.98518 4.58477 3.61503

0.04988 4.13121 3.42374 0.61958 0.77255 0.75458 0.63527

936 2.62429 4.62731 2.93708 2.49937 3.81928 3.48924 3.71013 2.90556 2.23046 2.02608 3.71353 3.07950 3.43798 2.91282 2.97429 2.71955 2.85562 2.92288 5.16431 3.87514 1078 - -

2.68618 4.42225 2.77519 2.73123 3.46354 2.40513 3.72494 3.29354 2.67741 2.69355 4.24690 2.90347 2.73739 3.18146 2.89801 2.37887 2.77519 2.98518 4.58477 3.61503

0.02317 4.17209 4.89443 0.61958 0.77255 0.74911 0.64015

937 2.42956 4.75133 3.05447 2.39464 3.72016 3.46213 3.67638 2.98370 2.41907 3.00496 3.83238 3.01491 3.55661 2.57075 2.94367 1.97805 2.85378 3.07572 5.27041 3.95198 1079 - -

2.68622 4.42229 2.77523 2.73127 3.46341 2.40516 3.72498 3.29358 2.67745 2.69337 4.24693 2.90350 2.73713 3.18150 2.89804 2.37891 2.77523 2.98522 4.58481 3.61507

0.15988 2.13129 3.53795 0.66136 0.72598 0.60579 0.78887

938 2.63207 4.54176 3.26577 2.70447 3.71169 3.05183 3.49315 3.09158 2.67874 2.59363 3.63520 2.84372 3.49081 3.00850 3.09143 2.63673 2.06648 2.72741 3.92567 3.56873 1082 - -

2.68608 4.42130 2.77501 2.73136 3.46367 2.40526 3.72507 3.29367 2.67754 2.69368 4.24703 2.90323 2.73741 3.18159 2.89813 2.37878 2.77532 2.98490 4.58363 3.61516

0.28366 1.91115 2.31198 1.30455 0.31648 0.62099 0.77092

939 2.65304 4.95142 2.99238 2.43700 4.24133 3.44951 3.64210 3.67331 1.81236 3.07438 4.02506 2.77073 3.02259 2.77084 2.70417 2.40472 2.78983 3.07924 5.43129 4.08109 1088 - -

2.68618 4.42226 2.77520 2.73124 3.46354 2.40513 3.72495 3.29355 2.67741 2.69355 4.24690 2.90347 2.73740 3.18147 2.89801 2.37887 2.77520 2.98505 4.58477 3.61504
0.13036 3.01258 2.61650 0.46815 0.98392 0.62878 0.76194
940 2.64092 4.48236 3.31798 2.30451 3.68323 3.54737 3.84178 2.77383 2.77546 2.72412 3.61583 3.24278 3.69089 3.10577 3.16378 2.46846 2.88742 1.77430 5.09769 3.83899 1090 - -
2.68618 4.42225 2.77519 2.73123 3.46354 2.40513 3.72494 3.29354 2.67741 2.69355 4.24690 2.90347 2.73739 3.18146 2.89801 2.37887 2.77519 2.98518 4.58477 3.61503
0.02137 4.25227 4.97462 0.61958 0.77255 0.76605 0.62520
941 2.56919 5.07868 2.40518 1.78627 4.39161 3.41439 3.43623 3.85600 2.28441 3.36952 3.78849 2.89426 3.81548 2.72541 2.74658 2.54381 2.87775 3.21720 5.52974 4.13755 1091 - -
2.68618 4.42225 2.77519 2.73123 3.46354 2.40513 3.72494 3.29354 2.67741 2.69355 4.24690 2.90347 2.73739 3.18146 2.89801 2.37887 2.77519 2.98518 4.58477 3.61503
0.04319 4.25227 3.57403 0.61958 0.77255 0.76605 0.62520
942 2.41074 4.96611 2.76822 2.40163 4.25778 3.42667 3.63551 3.45526 2.19389 3.25292 4.03867 2.80502 3.82851 2.76138 2.63999 1.91453 2.87397 3.33107 5.44871 4.08530 1092 - -
2.68618 4.42225 2.77519 2.73123 3.46354 2.40513 3.72494 3.29354 2.67741 2.69355 4.24690 2.90347 2.73739 3.18146 2.89801 2.37887 2.77519 2.98518 4.58477 3.61503
0.02184 4.23092 4.95326 0.61958 0.77255 0.78714 0.60724
943 2.62197 4.14276 3.92788 3.34820 2.75481 3.69627 3.97238 1.85782 3.24370 2.23959 3.24868 3.12700 4.06671 3.49193 3.06684 2.97368 2.85386 2.28358 4.71887 2.96753 1093 - -
2.68618 4.42225 2.77519 2.73123 3.46354 2.40513 3.72494 3.29354 2.67741 2.69355 4.24690 2.90347 2.73739 3.18146 2.89801 2.37887 2.77519 2.98518 4.58477 3.61503
0.04781 4.23092 3.43755 0.61958 0.77255 0.78714 0.60724
944 2.40363 4.98689 2.65339 2.38327 4.29177 3.41194 3.63190 3.73664 2.39135 3.28353 4.06538 2.91748 3.82140 1.99476 2.66152 2.36091 2.87658 3.12958 5.47138 4.10147 1094 - -
2.68618 4.42225 2.77519 2.73123 3.46354 2.40513 3.72494 3.29354 2.67741 2.69355 4.24690 2.90347 2.73739 3.18146 2.89801 2.37887 2.77519 2.98518 4.58477 3.61503
0.02240 4.20551 4.92786 0.61958 0.77255 0.81110 0.58765
945 2.64312 4.96072 2.63015 2.32674 4.24094 2.24979 3.63989 3.67964 2.20706 3.24181 3.30229 2.92162 3.47902 2.77022 2.89499 2.54236 2.87844 3.31850 5.44567 4.08062 1095 - -
2.68618 4.42225 2.77519 2.73123 3.46354 2.40513 3.72494 3.29354 2.67741 2.69355 4.24690 2.90347 2.73739 3.18146 2.89801 2.37887 2.77519 2.98518 4.58477 3.61503
0.06669 4.20551 3.00372 0.61958 0.77255 0.65829 0.72926
946 2.85393 5.36533 1.50948 2.18908 4.68337 3.39305 3.73018 4.16901 1.99247 3.65172 4.43893 2.71525 3.89560 2.85874 2.84226 2.78483 3.10217 3.74672 5.79224 4.36237 1096 - -
2.68618 4.42225 2.77519 2.73123 3.46354 2.40513 3.72494 3.29354 2.67741 2.69355 4.24690 2.90347 2.73739 3.18146 2.89801 2.37887 2.77519 2.98518 4.58477 3.61503
0.06792 4.24220 2.97027 0.61958 0.77255 0.67772 0.70882
947 2.64193 4.30949 3.59991 3.03484 3.39534 3.63452 3.88414 2.77110 2.65850 2.30615 3.41254 3.40988 3.35410 3.25983 3.25606 2.89430 2.87470 2.08807 4.86654 2.24555 1097 - -
2.68618 4.42225 2.77519 2.73123 3.46354 2.40513 3.72494 3.29354 2.67741 2.69355 4.24690 2.90347 2.73739 3.18146 2.89801 2.37887 2.77519 2.98518 4.58477 3.61503
0.02151 4.24602 4.96836 0.61958 0.77255 0.77232 0.61979
948 2.82283 5.25750 2.78581 2.15823 4.59874 3.46243 3.38860 4.05444 1.76520 3.53393 4.31976 1.91915 3.90520 2.67947 2.75168 2.77689 3.05127 3.65208 5.65737 4.28805 1098 - -
2.68618 4.42225 2.77519 2.73123 3.46354 2.40513 3.72494 3.29354 2.67741 2.69355 4.24690 2.90347 2.73739 3.18146 2.89801 2.37887 2.77519 2.98518 4.58477 3.61503
0.02151 4.24602 4.96836 0.61958 0.77255 0.59317 0.80424
949 2.57438 4.92889 2.89604 2.31861 4.19087 3.47254 3.42494 3.00380 2.29946 3.19546 3.63612 2.98580 3.86271 2.80086 2.01262 2.67477 2.62186 3.27411 5.41243 4.06459 1099 - -
2.68673 4.41939 2.77522 2.73120 3.46373 2.40528 3.72525 3.29373 2.67615 2.69380 4.24641 2.90346 2.73748 3.18203 2.89783 2.37908 2.77550 2.98517 4.58437 3.61449
0.49768 1.01891 3.47152 2.27022 0.10902 0.67732 0.70923
950 2.63762 4.36200 3.50939 2.84746 3.01507 3.61531 3.41753 2.64851 2.79228 2.48663 3.21387 3.34937 3.56648 3.19901 3.25022 2.74734 1.92561 2.53613 4.94340 3.70836 1119 - -
2.68618 4.42225 2.77519 2.73123 3.46354 2.40513 3.72494 3.29354 2.67741 2.69355 4.24690 2.90347 2.73739 3.18146 2.89801 2.37887 2.77519 2.98518 4.58477 3.61503
0.22450 4.32759 1.67192 0.61958 0.77255 0.70640 0.68007

951 2.58915 4.32711 3.52126 2.98637 3.55677 3.51737 3.89976 1.85712 2.85531 2.59655 3.51439 3.35566 3.96128 3.22264 2.85161 2.21603 2.67516 2.57557 5.00428 3.77297 1120 - -
2.68618 4.42225 2.77519 2.73123 3.46354 2.40513 3.72494 3.29354 2.67741 2.69355 4.24690 2.90347 2.73739 3.18146 2.89801 2.37887 2.77519 2.98518 4.58477 3.61503
0.02425 4.12734 4.84968 0.61958 0.77255 0.89661 0.52416
952 2.66612 4.70037 3.11932 2.59507 3.92274 3.48617 3.69920 2.49039 2.44514 2.95279 3.80762 2.93110 3.89144 1.95340 2.70605 2.62333 2.89921 3.02632 5.22300 3.93396 1121 - -
2.68618 4.42225 2.77519 2.73123 3.46354 2.40513 3.72494 3.29354 2.67741 2.69355 4.24690 2.90347 2.73739 3.18146 2.89801 2.37887 2.77519 2.98518 4.58477 3.61503
0.02425 4.12734 4.84968 0.61958 0.77255 0.89661 0.52416
953 2.59824 4.39880 3.11884 2.79566 3.55458 3.54002 3.79044 2.78677 2.64757 1.89794 3.31622 3.23129 3.92680 3.07582 3.12973 2.45029 2.61530 2.66224 4.97782 3.72849 1122 - -
2.68618 4.42225 2.77519 2.73123 3.46354 2.40513 3.72494 3.29354 2.67741 2.69355 4.24690 2.90347 2.73739 3.18146 2.89801 2.37887 2.77519 2.98518 4.58477 3.61503
0.07251 4.14999 2.91553 0.61958 0.77255 0.89661 0.52416
954 2.68124 5.26322 2.47032 1.85330 4.57643 3.33825 3.71288 4.04684 2.53676 3.56280 4.37519 1.73316 3.86243 2.53153 3.01611 2.76973 3.08499 3.65064 5.72645 4.31177 1123 - -
2.68618 4.42225 2.77519 2.73123 3.46354 2.40513 3.72494 3.29354 2.67741 2.69355 4.24690 2.90347 2.73739 3.18146 2.89801 2.37887 2.77519 2.98518 4.58477 3.61503
0.05432 4.10236 3.31501 0.61958 0.77255 0.93311 0.49977
955 3.29968 4.79844 4.46564 4.10249 3.42624 4.12150 4.75380 2.64127 3.84719 1.97839 0.81972 4.33037 4.59711 4.21179 4.00651 3.67942 3.61911 2.68701 5.30583 4.09381 1124 - -
2.68618 4.42225 2.77519 2.73123 3.46354 2.40513 3.72494 3.29354 2.67741 2.69355 4.24690 2.90347 2.73739 3.18146 2.89801 2.37887 2.77519 2.98518 4.58477 3.61503
0.02174 4.23523 4.95758 0.61958 0.77255 0.81909 0.58130
956 2.52779 3.33925 3.87091 3.29573 3.31199 3.44105 3.99133 1.90693 3.20869 2.39485 2.98209 2.86380 4.06646 3.46552 3.46262 2.55898 2.86749 2.27784 4.80381 3.59972 1125 - -
2.68618 4.42225 2.77519 2.73123 3.46354 2.40513 3.72494 3.29354 2.67741 2.69355 4.24690 2.90347 2.73739 3.18146 2.89801 2.37887 2.77519 2.98518 4.58477 3.61503
0.01997 4.31948 5.04182 0.61958 0.77255 0.71876 0.66817
957 2.71735 5.18673 2.86672 1.94756 4.51627 3.20296 3.66996 3.99020 2.23303 3.48480 4.23985 1.99526 3.39326 2.77731 2.76159 2.50082 2.85205 3.57074 5.62932 4.22724 1126 - -
2.68637 4.42244 2.77539 2.73143 3.46373 2.40532 3.72514 3.29300 2.67760 2.69320 4.24678 2.90366 2.73739 3.18151 2.89820 2.37906 2.77452 2.98432 4.58496 3.61522
0.78492 0.81211 2.30336 0.68339 0.70300 0.72699 0.66042
958 1.92347 4.75865 3.13800 2.57517 3.97580 3.51501 3.28853 3.19719 2.13073 2.88707 3.84013 3.07664 3.90294 2.89312 2.71697 2.62954 2.89450 2.74961 5.27726 3.97047 1129 - -
2.68618 4.42225 2.77519 2.73123 3.46354 2.40513 3.72494 3.29354 2.67741 2.69355 4.24690 2.90347 2.73739 3.18146 2.89801 2.37887 2.77519 2.98518 4.58477 3.61503
0.04135 4.34038 3.59442 0.61958 0.77255 0.50158 0.93033
959 2.70464 5.08194 2.75831 2.44770 4.37713 3.49445 3.69082 3.22943 2.39339 3.36563 4.13816 2.78729 3.67594 2.13231 2.79786 2.56234 2.01773 3.44589 5.54655 4.16944 1130 - -
2.68625 4.42124 2.77527 2.73114 3.46361 2.40520 3.72502 3.29361 2.67741 2.69362 4.24697 2.90354 2.73731 3.18137 2.89808 2.37886 2.77506 2.98510 4.58484 3.61510
0.41034 1.44613 2.29168 0.36221 1.19117 0.55982 0.84702
960 2.68687 3.77333 3.01219 2.25390 3.97112 3.31238 3.42382 3.73517 2.33796 3.29224 4.07611 2.67912 3.88166 2.80974 2.61227 1.82726 2.91810 3.37076 5.49088 4.12609 1133 - -
2.68618 4.42225 2.77519 2.73123 3.46354 2.40513 3.72494 3.29354 2.67741 2.69355 4.24690 2.90347 2.73739 3.18146 2.89801 2.37887 2.77519 2.98518 4.58477 3.61503
0.06434 4.45219 2.98269 0.61958 0.77255 0.70613 0.68034
961 2.60936 4.91926 3.06879 2.51001 4.17212 3.50762 3.70355 3.41218 2.41357 2.94678 3.98902 1.92584 3.89793 2.67634 2.50067 2.65874 2.74851 2.89275 5.41161 4.07308 1134 - -
2.68618 4.42225 2.77519 2.73123 3.46354 2.40513 3.72494 3.29354 2.67741 2.69355 4.24690 2.90347 2.73739 3.18146 2.89801 2.37887 2.77519 2.98518 4.58477 3.61503
0.04440 4.42464 3.45929 0.61958 0.77255 0.58550 0.81380
962 2.59377 4.68174 3.25453 2.69534 2.59710 3.41393 3.56855 3.06585 2.67505 2.91673 3.58157 1.96864 3.96802 2.85427 3.00619 2.64837 2.73420 2.98434 5.22383 3.93653 1135 - -
2.68618 4.42225 2.77519 2.73123 3.46354 2.40513 3.72494 3.29354 2.67741 2.69355 4.24690 2.90347 2.73739 3.18146 2.89801 2.37887 2.77519 2.98518 4.58477 3.61503

0.01668 4.49795 5.22030 0.61958 0.77255 0.66919 0.71770
963 2.63378 4.75480 3.20340 2.64536 3.71181 3.36420 3.78405 3.35224 2.38610 2.73222 3.83883 2.86064 3.65492 2.69008 3.07040 2.12324 2.93335 2.13075 5.28643 3.98486 1136 - -
2.68618 4.42225 2.77519 2.73123 3.46354 2.40513 3.72494 3.29354 2.67741 2.69355 4.24690 2.90347 2.73739 3.18146 2.89801 2.37887 2.77519 2.98518 4.58477 3.61503
0.04561 4.51365 3.39232 0.61958 0.77255 0.64821 0.74020
964 2.53131 3.37467 3.28552 2.89817 3.13133 3.64351 3.11312 2.79907 2.86285 2.51487 3.34736 3.19633 3.56995 3.17685 3.10225 1.93264 2.92516 2.76864 5.05987 3.81146 1137 - -
2.68618 4.42225 2.77519 2.73123 3.46354 2.40513 3.72494 3.29354 2.67741 2.69355 4.24690 2.90347 2.73739 3.18146 2.89801 2.37887 2.77519 2.98518 4.58477 3.61503
0.03630 4.50053 3.70716 0.61958 0.77255 0.70861 0.67792
965 2.52112 4.76380 3.17845 2.62104 3.52601 3.54771 3.76913 3.37605 2.60786 3.01556 3.85239 2.79793 2.84286 2.95075 2.88943 1.84198 2.75597 2.94730 5.29741 3.48435 1138 - -
2.68618 4.42225 2.77519 2.73123 3.46354 2.40513 3.72494 3.29354 2.67741 2.69355 4.24690 2.90347 2.73739 3.18146 2.89801 2.37887 2.77519 2.98518 4.58477 3.61503
0.08466 4.48119 2.66127 0.61958 0.77255 0.68951 0.69680
966 2.58157 4.81084 3.00080 2.55975 3.76867 2.87483 3.73038 3.29076 2.38383 2.77181 3.88736 3.07070 3.91061 2.89589 2.73597 2.33859 2.13243 3.13312 5.32650 3.76289 1139 - -
2.68618 4.42225 2.77519 2.73123 3.46354 2.40513 3.72494 3.29354 2.67741 2.69355 4.24690 2.90347 2.73739 3.18146 2.89801 2.37887 2.77519 2.98518 4.58477 3.61503
0.11488 4.43254 2.33677 0.61958 0.77255 0.79481 0.60088
967 1.98207 4.81006 3.07085 2.51945 4.05093 3.04939 3.01581 3.46221 2.51135 2.81172 3.89660 2.76421 3.63034 2.76859 2.96828 2.61907 2.62748 3.02422 5.33157 4.00557 1140 - -
2.68618 4.42225 2.77519 2.73123 3.46354 2.40513 3.72494 3.29354 2.67741 2.69355 4.24690 2.90347 2.73739 3.18146 2.89801 2.37887 2.77519 2.98518 4.58477 3.61503
0.01961 4.33727 5.05962 0.61958 0.77255 0.78349 0.61030
968 2.58904 4.65070 3.23186 2.67248 3.84225 2.86868 3.77908 3.22929 2.65176 2.80714 3.46704 2.96127 3.94043 2.98891 2.92854 1.94279 2.67358 2.84897 5.19874 2.93220 1141 - -
2.68618 4.42225 2.77519 2.73123 3.46354 2.40513 3.72494 3.29354 2.67741 2.69355 4.24690 2.90347 2.73739 3.18146 2.89801 2.37887 2.77519 2.98518 4.58477 3.61503
0.11317 4.41123 2.35535 0.61958 0.77255 0.84625 0.56040
969 2.36637 4.43202 3.41314 2.85522 3.58937 3.30492 3.83864 2.95500 2.53049 1.87025 3.54066 3.28525 3.97251 3.13059 3.17846 2.50698 2.71198 2.71829 5.01133 3.19366 1142 - -
2.68620 4.42119 2.77522 2.73126 3.46356 2.40515 3.72497 3.29356 2.67743 2.69357 4.24692 2.90349 2.73742 3.18149 2.89780 2.37889 2.77522 2.98521 4.58479 3.61505
0.31059 2.58368 1.65292 0.65376 0.73415 0.73490 0.65306
970 2.63273 4.95960 2.92604 1.82953 4.24968 2.97788 3.63412 3.46836 2.28105 3.24735 4.03261 2.92813 3.39257 2.76015 2.87757 2.44873 2.66506 3.18002 5.44571 4.08006 1145 - -
2.68621 4.42234 2.77528 2.73132 3.46363 2.40507 3.72503 3.29351 2.67743 2.69364 4.24698 2.90355 2.73662 3.18155 2.89798 2.37895 2.77528 2.98527 4.58486 3.61512
0.66306 0.73890 4.94898 0.12079 2.17347 0.75497 0.63493
971 2.66047 4.54255 3.32431 2.76066 3.15947 3.57870 3.80872 2.88889 1.93132 2.57759 3.35551 3.22192 3.22854 3.05712 2.76982 2.69475 2.78768 2.83234 4.05037 3.83467 1147 - -
2.68618 4.42225 2.77519 2.73123 3.46354 2.40513 3.72494 3.29354 2.67741 2.69355 4.24690 2.90347 2.73739 3.18146 2.89801 2.37887 2.77519 2.98518 4.58477 3.61503
0.11858 4.38618 2.30891 0.61958 0.77255 0.84929 0.55812
972 2.66493 3.01648 3.19000 2.62388 3.91988 3.52400 3.73430 3.30777 1.81031 2.67900 3.79822 2.78057 3.91478 2.92329 2.82662 2.66632 2.82520 2.90243 5.23665 3.94540 1148 - -
2.68618 4.42225 2.77519 2.73123 3.46354 2.40513 3.72494 3.29354 2.67741 2.69355 4.24690 2.90347 2.73739 3.18146 2.89801 2.37887 2.77519 2.98518 4.58477 3.61503
0.04207 4.31162 3.58317 0.61958 0.77255 0.97108 0.47590
973 2.54593 4.90433 3.03827 2.47532 4.16615 3.47496 3.66242 3.39180 2.33399 2.94179 3.97372 2.90137 3.86434 2.54142 2.06788 2.30619 2.77363 3.15383 5.38902 4.05172 1149 - -
2.68618 4.42225 2.77519 2.73123 3.46354 2.40513 3.72494 3.29354 2.67741 2.69355 4.24690 2.90347 2.73739 3.18146 2.89801 2.37887 2.77519 2.98518 4.58477 3.61503
0.02057 4.29012 5.01246 0.61958 0.77255 0.57980 0.82101
974 2.70183 3.43127 3.34599 2.78045 3.77962 3.60985 3.83616 2.99301 2.48359 2.35405 3.70106 3.24509 3.69380 3.07408 2.02870 2.34614 2.93336 2.78135 5.16100 3.89300 1150 - -

2.68626 4.42244 2.77539 2.73142 3.46290 2.40532 3.72514 3.29317 2.67729 2.69312 4.24682 2.90366 2.73750 3.18165 2.89813 2.37862 2.77523 2.98515 4.58496 3.61522
0.15391 1.98619 5.21513 1.47140 0.26085 0.72940 0.65816
975 2.62240 4.77707 2.77359 2.63353 3.66110 3.56679 3.78009 2.94718 2.06333 3.02027 3.85796 3.02130 3.95454 2.96243 2.77701 2.70687 2.74301 2.18361 5.30399 3.57732 1157 - -
2.68618 4.42225 2.77519 2.73123 3.46354 2.40513 3.72494 3.29354 2.67741 2.69355 4.24690 2.90347 2.73739 3.18146 2.89801 2.37887 2.77519 2.98518 4.58477 3.61503
0.08475 4.52667 2.65302 0.61958 0.77255 0.57161 0.83152
976 2.45183 4.72000 3.24317 2.68266 3.91172 3.19725 3.55286 3.05079 2.36195 2.16113 3.80526 3.17364 3.25330 3.00308 2.80770 2.32295 2.76712 3.02543 4.58738 3.96424 1158 - -
2.68618 4.42225 2.77519 2.73123 3.46354 2.40513 3.72494 3.29354 2.67741 2.69355 4.24690 2.90347 2.73739 3.18146 2.89801 2.37887 2.77519 2.98518 4.58477 3.61503
0.01610 4.53256 5.25491 0.61958 0.77255 0.69199 0.69431
977 2.46575 4.75047 3.23167 2.67180 3.94927 3.11839 3.80237 2.90593 2.33662 2.55892 3.83569 3.16662 3.97276 2.87669 2.96506 1.92548 2.69768 2.88213 5.28502 3.98721 1159 - -
2.68618 4.42225 2.77519 2.73123 3.46354 2.40513 3.72494 3.29354 2.67741 2.69355 4.24690 2.90347 2.73739 3.18146 2.89801 2.37887 2.77519 2.98518 4.58477 3.61503
0.04383 4.55259 3.43126 0.61958 0.77255 0.41007 1.08947
978 2.74538 4.06324 3.67581 2.94369 3.56644 3.52604 3.79741 2.38348 2.95241 2.26904 3.53940 2.18033 4.11657 3.35101 3.09241 2.90947 2.79924 2.44612 3.00970 3.79204 1160 - -
2.68618 4.42225 2.77519 2.73123 3.46354 2.40513 3.72494 3.29354 2.67741 2.69355 4.24690 2.90347 2.73739 3.18146 2.89801 2.37887 2.77519 2.98518 4.58477 3.61503
0.01438 4.64522 5.36756 0.61958 0.77255 0.43554 1.04104
979 2.76227 4.09849 3.54247 2.89790 2.56910 3.34069 4.02744 2.82777 2.98067 1.86828 3.53494 3.54204 4.14283 3.25014 3.02308 2.57715 2.60497 2.28671 5.01787 3.79347 1161 - -
2.68618 4.42225 2.77519 2.73123 3.46354 2.40513 3.72494 3.29354 2.67741 2.69355 4.24690 2.90347 2.73739 3.18146 2.89801 2.37887 2.77519 2.98518 4.58477 3.61503
0.40501 4.68960 1.12750 0.61958 0.77255 0.48576 0.95510
980 2.55002 3.35961 3.61599 2.83199 3.26359 3.08643 3.89970 2.59298 2.98885 2.49394 3.39370 3.41945 4.01257 3.12247 3.31322 2.63203 2.29625 2.27657 4.87734 3.17424 1162 - -
2.68618 4.42225 2.77519 2.73123 3.46354 2.40513 3.72494 3.29354 2.67741 2.69355 4.24690 2.90347 2.73739 3.18146 2.89801 2.37887 2.77519 2.98518 4.58477 3.61503
0.03907 4.30485 3.69654 0.61958 0.77255 0.99274 0.46293
981 2.66820 4.19615 3.95224 3.37733 3.32404 3.74501 4.05348 2.27128 3.10362 1.81129 3.30816 3.65720 4.11985 3.53661 3.51698 2.79385 2.32056 1.94712 4.84088 3.63891 1163 - -
2.68618 4.42225 2.77519 2.73123 3.46354 2.40513 3.72494 3.29354 2.67741 2.69355 4.24690 2.90347 2.73739 3.18146 2.89801 2.37887 2.77519 2.98518 4.58477 3.61503
0.04179 4.28643 3.60540 0.61958 0.77255 0.91577 0.51117
982 2.48659 4.81015 2.74362 2.50119 3.60760 3.19212 3.68491 3.17890 2.49439 2.98047 3.88441 2.81937 3.86757 2.71236 2.95442 2.12053 2.50700 3.13785 5.31976 3.69953 1164 - -
2.68618 4.42225 2.77519 2.73123 3.46354 2.40513 3.72494 3.29354 2.67741 2.69355 4.24690 2.90347 2.73739 3.18146 2.89801 2.37887 2.77519 2.98518 4.58477 3.61503
0.02018 4.30875 5.03110 0.61958 0.77255 0.94442 0.49250
983 2.49772 3.15688 4.07934 3.49251 3.21863 3.73300 3.72561 2.18736 2.76292 1.81638 3.22883 3.70702 4.10268 3.59711 3.55731 3.01800 2.75608 2.22635 4.15426 3.53342 1165 - -
2.68639 4.42246 2.77541 2.73144 3.46125 2.40534 3.72516 3.29272 2.67762 2.69321 4.24711 2.90368 2.73761 3.18167 2.89822 2.37894 2.77534 2.98488 4.58498 3.61465
0.51557 0.97273 3.69728 0.95844 0.48368 0.97216 0.47524
984 2.55237 4.04789 3.51617 2.94841 2.67918 3.61272 3.86677 2.67135 2.71630 2.38005 3.45196 3.35224 3.28578 2.87645 3.02455 2.75511 2.42921 2.46005 4.93096 3.69744 1169 - -
2.68618 4.42225 2.77519 2.73123 3.46354 2.40513 3.72494 3.29354 2.67741 2.69355 4.24690 2.90347 2.73739 3.18146 2.89801 2.37887 2.77519 2.98518 4.58477 3.61503
0.02016 4.31004 5.03238 0.61958 0.77255 0.98832 0.46554
985 2.64223 4.73871 3.11231 2.55301 3.61784 3.49715 3.70708 3.35136 2.46533 2.66260 3.81810 2.80652 3.88533 2.71283 2.64843 2.02584 2.67029 2.95894 5.26007 3.57463 1170 - -
2.68618 4.42225 2.77519 2.73123 3.46354 2.40513 3.72494 3.29354 2.67741 2.69355 4.24690 2.90347 2.73739 3.18146 2.89801 2.37887 2.77519 2.98518 4.58477 3.61503
0.02016 4.31004 5.03238 0.61958 0.77255 0.98832 0.46554

986 2.56540 4.76797 3.08604 2.34650 3.33200 3.48804 3.49545 3.39208 2.51931 2.91540 3.84498 2.66101 3.87732 2.71684 2.97278 2.40720 2.21883 2.98232 5.28468 3.70858 1171 - -
2.68618 4.42225 2.77519 2.73123 3.46354 2.40513 3.72494 3.29354 2.67741 2.69355 4.24690 2.90347 2.73739 3.18146 2.89801 2.37887 2.77519 2.98518 4.58477 3.61503
0.02016 4.31004 5.03238 0.61958 0.77255 0.98832 0.46554
987 2.40440 4.13367 3.99511 3.41298 2.22247 3.71092 4.01012 2.58654 3.30686 2.09222 3.24836 3.65671 3.59701 3.54275 3.51830 2.67515 2.49859 2.30392 3.67056 3.54096 1172 - -
2.68618 4.42225 2.77519 2.73123 3.46354 2.40513 3.72494 3.29354 2.67741 2.69355 4.24690 2.90347 2.73739 3.18146 2.89801 2.37887 2.77519 2.98518 4.58477 3.61503
0.02016 4.31004 5.03238 0.61958 0.77255 0.98832 0.46554
988 2.55307 4.32031 3.56403 2.99587 2.94812 3.62311 3.88503 2.68908 2.94432 2.26598 3.42713 3.06864 3.74691 3.24011 3.28364 2.01853 2.66698 2.44620 4.90817 3.47299 1173 - -
2.68640 4.42276 2.77509 2.73133 3.46299 2.40528 3.72478 3.29339 2.67776 2.69363 4.24714 2.90367 2.73750 3.18125 2.89783 2.37852 2.77523 2.98496 4.58401 3.61527
0.95812 0.61514 2.57939 1.46614 0.26242 0.28610 1.39106
989 2.73775 4.95499 2.83388 1.97303 3.90568 3.41332 3.76842 3.07402 2.48101 3.08254 4.02431 2.95518 3.95561 2.91489 3.03037 2.08502 2.65089 2.99636 3.65925 4.11537 1182 - -
2.68618 4.42225 2.77519 2.73123 3.46354 2.40513 3.72494 3.29354 2.67741 2.69355 4.24690 2.90347 2.73739 3.18146 2.89801 2.37887 2.77519 2.98518 4.58477 3.61503
0.05748 4.62748 3.07733 0.61958 0.77255 0.45528 1.00586
990 2.65683 5.04012 2.99180 2.52846 3.30097 3.55176 3.40417 3.74209 1.80785 2.88059 3.70700 2.80125 3.51884 2.87919 2.75304 2.31466 2.86008 3.20969 5.52224 4.16392 1183 - -
2.68618 4.42225 2.77519 2.73123 3.46354 2.40513 3.72494 3.29354 2.67741 2.69355 4.24690 2.90347 2.73739 3.18146 2.89801 2.37887 2.77519 2.98518 4.58477 3.61503
0.01450 4.63655 5.35890 0.61958 0.77255 0.48753 0.95229
991 2.54161 4.49491 3.60288 3.03582 3.63494 3.72632 3.97641 2.07464 2.99047 2.58444 3.34718 3.44980 2.47973 3.00584 2.52710 2.85175 2.73462 2.53607 5.07428 3.64554 1184 - -
2.68618 4.42225 2.77519 2.73123 3.46354 2.40513 3.72494 3.29354 2.67741 2.69355 4.24690 2.90347 2.73739 3.18146 2.89801 2.37887 2.77519 2.98518 4.58477 3.61503
0.38767 4.66893 1.16481 0.61958 0.77255 0.52719 0.89224
992 2.63122 5.29709 2.20419 1.52212 4.61673 3.40166 3.71740 4.10335 2.43582 3.59630 4.36803 2.86894 3.11916 2.83875 3.05796 2.44497 3.04111 3.67846 5.74646 4.31495 1185 - -
2.68618 4.42225 2.77519 2.73123 3.46354 2.40513 3.72494 3.29354 2.67741 2.69355 4.24690 2.90347 2.73739 3.18146 2.89801 2.37887 2.77519 2.98518 4.58477 3.61503
0.02033 4.30159 5.02393 0.61958 0.77255 0.64164 0.74746
993 2.36255 4.69182 3.23015 2.61841 2.46225 3.56778 3.52684 3.27062 2.56682 2.78772 3.48468 2.84450 3.71780 2.87896 2.98095 2.15285 2.91880 2.99610 5.23045 3.93936 1186 - -
2.68618 4.42225 2.77519 2.73123 3.46354 2.40513 3.72494 3.29354 2.67741 2.69355 4.24690 2.90347 2.73739 3.18146 2.89801 2.37887 2.77519 2.98518 4.58477 3.61503
0.01711 4.47237 5.19472 0.61958 0.77255 0.82483 0.57680
994 2.60871 5.15742 2.78466 1.98910 4.19443 3.47015 3.17971 3.72264 1.85956 3.45480 4.20312 2.83595 3.21054 2.76276 2.89136 2.53974 2.83390 3.38598 5.60096 4.20128 1187 - -
2.68618 4.42225 2.77519 2.73123 3.46354 2.40513 3.72494 3.29354 2.67741 2.69355 4.24690 2.90347 2.73739 3.18146 2.89801 2.37887 2.77519 2.98518 4.58477 3.61503
0.05704 4.47237 3.12305 0.61958 0.77255 0.82483 0.57680
995 2.74246 4.30805 3.93117 3.35446 3.37649 3.78887 4.07631 2.46688 3.22419 1.37768 3.15072 3.66301 4.16191 3.51955 3.16112 2.33933 2.97729 2.50422 4.90973 3.70744 1188 - -
2.68618 4.42225 2.77519 2.73123 3.46354 2.40513 3.72494 3.29354 2.67741 2.69355 4.24690 2.90347 2.73739 3.18146 2.89801 2.37887 2.77519 2.98518 4.58477 3.61503
0.08556 4.43314 2.65752 0.61958 0.77255 0.86943 0.54333
996 2.51951 4.97191 2.76179 2.26325 3.94016 3.33025 3.66373 3.68066 2.37139 2.69762 4.03301 2.97038 3.85814 2.07986 2.84639 2.66463 2.69803 3.04751 5.45106 4.08960 1189 - -
2.68618 4.42225 2.77519 2.73123 3.46354 2.40513 3.72494 3.29354 2.67741 2.69355 4.24690 2.90347 2.73739 3.18146 2.89801 2.37887 2.77519 2.98518 4.58477 3.61503
0.03759 4.36662 3.72129 0.61958 0.77255 0.93722 0.49711
997 2.66157 4.91738 2.91364 2.46964 4.17460 3.47747 3.67334 3.60003 1.82377 3.07233 3.98372 2.99420 3.86791 2.68429 2.76115 2.53109 2.51332 2.80138 5.40510 3.49448 1190 - -
2.68618 4.42225 2.77519 2.73123 3.46354 2.40513 3.72494 3.29354 2.67741 2.69355 4.24690 2.90347 2.73739 3.18146 2.89801 2.37887 2.77519 2.98518 4.58477 3.61503

0.01939 4.34841 5.07076 0.61958 0.77255 0.91337 0.51278

998 1.26326 4.40505 3.64947 3.14970 3.94868 3.38815 4.13602 2.99490 3.10439 2.97611 3.87598 3.45332 2.50910 3.41833 3.45501 2.74294 2.76435 2.58738 5.37231 4.13939 1191 - -

2.68618 4.42225 2.77519 2.73123 3.46354 2.40513 3.72494 3.29354 2.67741 2.69355 4.24690 2.90347 2.73739 3.18146 2.89801 2.37887 2.77519 2.98518 4.58477 3.61503

0.08137 4.36574 2.72655 0.61958 0.77255 0.93806 0.49657

999 2.89875 5.13810 3.24380 2.49135 4.49818 3.62229 3.69663 3.88916 2.01310 3.39645 4.21670 3.13130 3.37770 2.82581 1.35244 2.89668 3.10249 3.26162 5.52540 4.25588 1192 - -

2.68618 4.42225 2.77519 2.73123 3.46354 2.40513 3.72494 3.29354 2.67741 2.69355 4.24690 2.90347 2.73739 3.18146 2.89801 2.37887 2.77519 2.98518 4.58477 3.61503

0.02027 4.30463 5.02698 0.61958 0.77255 0.95152 0.48801

1000 1.90177 4.60456 3.07681 2.66157 3.78579 3.53409 3.75742 2.91387 2.46681 2.64104 3.69365 3.14240 3.92001 2.88022 2.96396 2.65506 2.62356 2.81027 5.15050 3.36225 1193 - -

2.68618 4.42225 2.77519 2.73123 3.46354 2.40513 3.72494 3.29354 2.67741 2.69355 4.24690 2.90347 2.73739 3.18146 2.89801 2.37887 2.77519 2.98518 4.58477 3.61503

0.01990 4.32295 5.04529 0.61958 0.77255 0.93419 0.49907

1001 2.52958 4.97226 2.73126 2.22213 3.95037 3.45727 3.47613 3.47986 2.36212 2.92740 4.03265 2.87545 3.84958 2.67913 2.90289 1.94791 2.88377 3.19122 5.44994 4.08644 1194 - -

2.68618 4.42225 2.77519 2.73123 3.46354 2.40513 3.72494 3.29354 2.67741 2.69355 4.24690 2.90347 2.73739 3.18146 2.89801 2.37887 2.77519 2.98518 4.58477 3.61503

0.01953 4.34162 5.06396 0.61958 0.77255 0.81217 0.58680

1002 2.73672 4.25093 3.93684 3.80370 3.03494 3.98050 4.29712 1.24982 3.67742 2.00808 3.29047 3.98969 4.33010 3.88474 3.83710 3.28092 3.05919 2.13247 4.91015 3.46523 1195 - -

2.68512 4.42331 2.77375 2.73169 3.46429 2.40628 3.72531 3.29411 2.67694 2.69372 4.24475 2.90351 2.73674 3.18224 2.89844 2.37867 2.77557 2.98443 4.58607 3.61562

0.97984 0.50853 3.76150 2.18311 0.11956 0.76274 0.62808

1003 2.60682 5.11477 1.99364 2.29186 4.42770 3.23931 3.45207 3.63741 2.23773 3.27299 4.16431 2.94879 3.86188 2.76833 2.81479 2.30304 2.82951 3.37962 5.56698 3.87834 1221 - -

2.68618 4.42225 2.77519 2.73123 3.46354 2.40513 3.72494 3.29354 2.67741 2.69355 4.24690 2.90347 2.73739 3.18146 2.89801 2.37887 2.77519 2.98518 4.58477 3.61503

0.12626 4.44562 2.23598 0.61958 0.77255 0.85564 0.55340

1004 2.65957 5.08173 2.82483 2.05496 4.39353 3.44285 3.63307 3.69554 2.23774 3.16874 3.85785 2.71058 2.45889 2.68211 2.62963 2.63887 2.89035 3.45454 5.53332 4.14635 1222 - -

2.68618 4.42225 2.77519 2.73123 3.46354 2.40513 3.72494 3.29354 2.67741 2.69355 4.24690 2.90347 2.73739 3.18146 2.89801 2.37887 2.77519 2.98518 4.58477 3.61503

0.01958 4.33894 5.06128 0.61958 0.77255 0.96291 0.48091

1005 2.54031 5.14038 1.78496 2.22064 3.97836 3.43267 3.66901 3.91510 2.24649 3.43192 4.19906 2.83221 3.85638 2.78421 2.93913 2.49131 2.75929 3.51292 5.59676 4.19819 1223 - -

2.68628 4.42235 2.77523 2.73127 3.46364 2.40523 3.72505 3.29364 2.67751 2.69358 4.24700 2.90333 2.73732 3.18157 2.89800 2.37897 2.77522 2.98529 4.58487 3.61305

0.82042 0.68829 2.85913 0.10539 2.30235 0.96291 0.48091

1006 2.62887 4.30777 3.34732 2.74513 3.18749 3.38746 3.88735 2.71216 2.95281 2.35443 3.24361 3.39238 4.00446 3.13483 3.28817 2.87720 2.74938 1.70931 4.89878 3.67167 1225 - -

2.68618 4.42225 2.77519 2.73123 3.46354 2.40513 3.72494 3.29354 2.67741 2.69355 4.24690 2.90347 2.73739 3.18146 2.89801 2.37887 2.77519 2.98518 4.58477 3.61503

0.02064 4.28661 5.00896 0.61958 0.77255 1.00793 0.45409

1007 2.68616 4.38322 3.61174 3.05181 3.26896 3.66324 3.94581 2.66876 2.96796 1.97105 3.43317 3.43930 1.97578 3.28883 3.16754 2.92761 2.81133 2.58873 4.97100 3.73487 1226 - -

2.68618 4.42225 2.77519 2.73123 3.46354 2.40513 3.72494 3.29354 2.67741 2.69355 4.24690 2.90347 2.73739 3.18146 2.89801 2.37887 2.77519 2.98518 4.58477 3.61503

0.02064 4.28661 5.00896 0.61958 0.77255 0.77116 0.62078

1008 2.66387 4.63795 3.03294 2.45290 3.56365 3.07679 3.35033 3.20636 2.65491 2.09461 3.72589 2.88775 3.93920 2.98922 3.08280 2.77207 2.69702 2.74797 5.18148 3.03754 1227 - -

2.68618 4.42225 2.77519 2.73123 3.46354 2.40513 3.72494 3.29354 2.67741 2.69355 4.24690 2.90347 2.73739 3.18146 2.89801 2.37887 2.77519 2.98518 4.58477 3.61503

0.01843 4.39901 5.12136 0.61958 0.77255 0.86445 0.54694

1009 2.42697 5.11793 2.75170 2.25367 4.44247 2.16236 3.68175 3.90607 2.44730 3.42546 4.18988 2.65469 3.33605 2.79508 2.84015 2.39560 2.73099 3.50296 5.59111 4.19894 1228 - -

2.68618 4.42225 2.77519 2.73123 3.46354 2.40513 3.72494 3.29354 2.67741 2.69355 4.24690 2.90347 2.73739 3.18146 2.89801 2.37887 2.77519 2.98518 4.58477 3.61503
0.05459 4.41549 3.19328 0.61958 0.77255 0.88832 0.52992
1010 2.68085 4.90999 2.61731 2.49114 4.16063 3.48976 3.69899 3.36498 2.41954 2.71834 3.98273 3.01396 2.04308 2.78021 2.79464 2.62166 2.91309 3.24961 5.40639 4.06558 1229 - -
2.68618 4.42225 2.77519 2.73123 3.46354 2.40513 3.72494 3.29354 2.67741 2.69355 4.24690 2.90347 2.73739 3.18146 2.89801 2.37887 2.77519 2.98518 4.58477 3.61503
0.01879 4.37969 5.10203 0.61958 0.77255 0.92461 0.50532
1011 3.35303 4.63648 5.19901 4.63191 3.35380 4.68937 5.06461 1.45426 4.49995 1.47150 1.83088 4.79264 4.89543 4.57677 4.56378 4.04269 3.58080 1.86585 5.36341 4.28749 1230 - -
2.68618 4.42225 2.77519 2.73123 3.46354 2.40513 3.72494 3.29354 2.67741 2.69355 4.24690 2.90347 2.73739 3.18146 2.89801 2.37887 2.77519 2.98518 4.58477 3.61503
0.04762 4.37969 3.38216 0.61958 0.77255 0.92461 0.50532
1012 2.73913 4.63746 3.34768 2.79342 3.04917 3.62444 3.52317 3.19689 2.51591 2.85089 3.73333 2.81771 4.00743 3.07040 2.73874 2.86294 2.96732 2.94425 4.94649 1.80124 1231 - -
2.68618 4.42225 2.77519 2.73123 3.46354 2.40513 3.72494 3.29354 2.67741 2.69355 4.24690 2.90347 2.73739 3.18146 2.89801 2.37887 2.77519 2.98518 4.58477 3.61503
0.08260 4.35140 2.71221 0.61958 0.77255 0.95151 0.48802
1013 2.72126 4.93623 2.95815 2.44595 4.19459 3.51587 3.26287 3.61509 2.20048 3.19101 4.00584 2.92231 3.90394 2.82106 2.23240 2.74315 2.94391 3.28639 2.34127 4.07564 1232 - -
2.68559 4.42267 2.77561 2.73159 3.46387 2.40540 3.72520 3.29359 2.67698 2.69367 4.24653 2.90213 2.73755 3.18188 2.89835 2.37888 2.77561 2.98429 4.58519 3.61531
0.93746 0.59536 2.86446 1.28480 0.32394 0.93917 0.49586
1014 2.72028 4.38537 3.58645 2.95648 1.37189 3.61802 3.98602 2.88230 3.16598 2.60495 3.57132 3.51109 4.09285 3.46762 3.45095 2.73801 2.82641 2.67114 4.82191 3.43773 1237 - -
2.68618 4.42225 2.77519 2.73123 3.46354 2.40513 3.72494 3.29354 2.67741 2.69355 4.24690 2.90347 2.73739 3.18146 2.89801 2.37887 2.77519 2.98518 4.58477 3.61503
0.04676 4.26936 3.45154 0.61958 0.77255 0.32429 1.28388
1015 2.73863 5.13578 2.94972 2.42742 4.08561 3.01419 2.35602 3.70422 2.07921 2.90504 4.18842 3.01806 3.48927 2.76269 2.67118 2.43469 2.88106 3.36214 5.59574 4.21357 1238 - -
2.68647 4.42103 2.77489 2.73078 3.46340 2.40548 3.72538 3.29367 2.67756 2.69366 4.24608 2.90323 2.73765 3.18122 2.89768 2.37867 2.77531 2.98543 4.58474 3.61564
0.48207 0.97352 5.35088 1.66948 0.20868 0.41976 1.07062
1016 2.66310 5.18029 2.90070 2.33878 4.18301 3.40419 3.73825 3.66576 2.40798 2.98772 4.23051 2.70242 3.93985 2.64284 1.82525 2.42312 2.82525 3.33128 5.63469 4.24652 1257 - -
2.68618 4.42225 2.77519 2.73123 3.46354 2.40513 3.72494 3.29354 2.67741 2.69355 4.24690 2.90347 2.73739 3.18146 2.89801 2.37887 2.77519 2.98518 4.58477 3.61503
0.01375 4.68960 5.41194 0.61958 0.77255 0.48576 0.95510
1017 2.76206 5.11984 3.07525 2.40710 4.40794 3.14948 3.59368 3.50445 2.19733 3.20585 4.17698 2.96361 3.32435 2.87375 2.89560 1.58496 2.90201 3.28915 5.58865 4.21659 1258 - -
2.68618 4.42225 2.77519 2.73123 3.46354 2.40513 3.72494 3.29354 2.67741 2.69355 4.24690 2.90347 2.73739 3.18146 2.89801 2.37887 2.77519 2.98518 4.58477 3.61503
0.01375 4.68960 5.41194 0.61958 0.77255 0.48576 0.95510
1018 2.54519 4.17388 2.71130 2.47542 4.02018 3.33884 3.27916 3.70809 2.50127 3.29026 4.08881 2.93159 3.33463 2.91346 2.94743 2.51849 2.82025 3.22703 5.51327 2.08431 1259 - -
2.68618 4.42225 2.77519 2.73123 3.46354 2.40513 3.72494 3.29354 2.67741 2.69355 4.24690 2.90347 2.73739 3.18146 2.89801 2.37887 2.77519 2.98518 4.58477 3.61503
0.03047 4.68960 3.87178 0.61958 0.77255 0.48576 0.95510
1019 2.62400 5.07432 2.36714 2.14866 4.34865 3.33872 3.75638 3.48208 2.52726 2.33237 4.13440 2.93267 3.75785 2.88232 2.82648 2.41776 2.80877 3.04502 5.55187 3.78632 1260 - -
2.68618 4.42225 2.77519 2.73123 3.46354 2.40513 3.72494 3.29354 2.67741 2.69355 4.24690 2.90347 2.73739 3.18146 2.89801 2.37887 2.77519 2.98518 4.58477 3.61503
0.01398 4.67310 5.39545 0.61958 0.77255 0.46551 0.98836
1020 2.76072 5.13654 2.83681 1.84859 4.43023 3.14364 3.75039 3.88107 2.51006 2.61308 4.19225 3.04682 2.94389 2.86698 2.80441 2.45863 2.59340 3.00108 5.60246 4.22518 1261 - -
2.68618 4.42225 2.77519 2.73123 3.46354 2.40513 3.72494 3.29354 2.67741 2.69355 4.24690 2.90347 2.73739 3.18146 2.89801 2.37887 2.77519 2.98518 4.58477 3.61503
0.01375 4.68960 5.41194 0.61958 0.77255 0.48576 0.95510

1021 2.77220 2.43545 3.78104 3.44877 3.42669 3.83661 4.12482 2.62406 2.95653 2.36656 1.78752 3.55576 3.77017 3.61102 3.60405 2.77960 3.00453 2.40420 4.91936 3.71678 1262 - -
2.68618 4.42225 2.77519 2.73123 3.46354 2.40513 3.72494 3.29354 2.67741 2.69355 4.24690 2.90347 2.73739 3.18146 2.89801 2.37887 2.77519 2.98518 4.58477 3.61503
0.01347 4.70993 5.43228 0.61958 0.77255 0.48576 0.95510
1022 2.57119 5.21804 2.84834 1.56066 3.73245 3.17754 3.52724 4.00898 2.24598 3.36725 3.81284 3.02572 3.71310 2.67835 2.89169 2.66771 2.87774 3.36886 5.66515 4.26897 1263 - -
2.68618 4.42225 2.77519 2.73123 3.46354 2.40513 3.72494 3.29354 2.67741 2.69355 4.24690 2.90347 2.73739 3.18146 2.89801 2.37887 2.77519 2.98518 4.58477 3.61503
0.01347 4.70993 5.43228 0.61958 0.77255 0.48576 0.95510
1023 2.77065 5.22760 2.95331 2.48348 4.02393 2.43610 3.58000 4.02264 1.65487 3.20525 4.27419 2.65015 3.74969 2.75861 2.61879 2.65475 2.91103 3.60735 5.67186 4.27569 1264 - -
2.68618 4.42225 2.77519 2.73123 3.46354 2.40513 3.72494 3.29354 2.67741 2.69355 4.24690 2.90347 2.73739 3.18146 2.89801 2.37887 2.77519 2.98518 4.58477 3.61503
0.20214 4.70993 1.74863 0.61958 0.77255 0.48576 0.95510
1024 2.70971 5.09432 3.01212 2.45461 4.39260 3.17108 3.69322 3.17033 2.21090 3.37831 3.88583 2.82103 3.14443 2.11502 2.37441 2.61168 2.78383 3.32340 5.55523 4.17758 1265 - -
2.68618 4.42225 2.77519 2.73123 3.46354 2.40513 3.72494 3.29354 2.67741 2.69355 4.24690 2.90347 2.73739 3.18146 2.89801 2.37887 2.77519 2.98518 4.58477 3.61503
0.01624 4.52403 5.24638 0.61958 0.77255 0.33318 1.26104
1025 2.77352 4.35296 3.93004 3.35384 2.05197 3.54602 4.09580 2.58449 2.64522 1.83295 3.30243 3.67363 3.43727 3.54149 2.85223 3.08416 2.91586 2.54994 4.95224 3.74339 1266 - -
2.68618 4.42225 2.77519 2.73123 3.46354 2.40513 3.72494 3.29354 2.67741 2.69355 4.24690 2.90347 2.73739 3.18146 2.89801 2.37887 2.77519 2.98518 4.58477 3.61503
0.01347 4.70993 5.43228 0.61958 0.77255 0.48576 0.95510
1026 2.81781 5.00551 3.23033 2.66048 4.24601 3.22817 3.81760 3.66414 1.36278 2.90863 4.07704 3.01801 4.02548 2.96832 2.70633 2.85195 2.95413 3.34382 4.50109 2.92045 1267 - -
2.68618 4.42225 2.77519 2.73123 3.46354 2.40513 3.72494 3.29354 2.67741 2.69355 4.24690 2.90347 2.73739 3.18146 2.89801 2.37887 2.77519 2.98518 4.58477 3.61503
0.01347 4.70993 5.43228 0.61958 0.77255 0.48576 0.95510
1027 3.62409 4.81853 5.71136 5.26810 4.21040 5.25388 6.07401 0.97520 5.21793 2.61219 3.93174 5.43112 5.48015 5.48957 5.39705 4.70786 3.89076 0.90468 6.37194 5.14030 1268 - -
2.68618 4.42225 2.77519 2.73123 3.46354 2.40513 3.72494 3.29354 2.67741 2.69355 4.24690 2.90347 2.73739 3.18146 2.89801 2.37887 2.77519 2.98518 4.58477 3.61503
0.01347 4.70993 5.43228 0.61958 0.77255 0.48576 0.95510
1028 4.77746 5.69930 5.58339 5.50989 1.91967 5.24710 3.32460 4.39515 5.28588 3.54948 4.90934 4.76670 5.49747 4.94038 5.09210 4.64820 4.96561 4.33189 3.00274 0.45609 1269 - -
2.68618 4.42225 2.77519 2.73123 3.46354 2.40513 3.72494 3.29354 2.67741 2.69355 4.24690 2.90347 2.73739 3.18146 2.89801 2.37887 2.77519 2.98518 4.58477 3.61503
0.01347 4.70993 5.43228 0.61958 0.77255 0.48576 0.95510
1029 2.89744 4.45165 4.95130 4.38489 3.65981 4.37574 4.79467 1.80079 4.23862 2.50789 2.38024 4.48599 4.71494 4.43159 4.34259 3.52884 3.08440 0.98789 5.32340 4.13374 1270 - -
2.68618 4.42225 2.77519 2.73123 3.46354 2.40513 3.72494 3.29354 2.67741 2.69355 4.24690 2.90347 2.73739 3.18146 2.89801 2.37887 2.77519 2.98518 4.58477 3.61503
0.01347 4.70993 5.43228 0.61958 0.77255 0.48576 0.95510
1030 3.11655 5.09960 4.01012 3.56191 2.89914 4.20138 2.59394 3.70161 3.26707 3.23438 4.25223 3.85668 4.59463 3.71976 3.18758 3.53639 3.65053 3.49266 4.50760 0.80165 1271 - -
2.68618 4.42225 2.77519 2.73123 3.46354 2.40513 3.72494 3.29354 2.67741 2.69355 4.24690 2.90347 2.73739 3.18146 2.89801 2.37887 2.77519 2.98518 4.58477 3.61503
0.01347 4.70993 5.43228 0.61958 0.77255 0.48576 0.95510
1031 2.80264 5.31619 2.11510 2.02632 4.65824 3.54695 3.75424 4.14509 1.80134 3.61956 4.35990 2.50079 3.52112 2.85413 2.79428 2.67872 2.81513 3.70565 5.74819 4.33377 1272 - -
2.68618 4.42225 2.77519 2.73123 3.46354 2.40513 3.72494 3.29354 2.67741 2.69355 4.24690 2.90347 2.73739 3.18146 2.89801 2.37887 2.77519 2.98518 4.58477 3.61503
0.01347 4.70993 5.43228 0.61958 0.77255 0.48576 0.95510
1032 2.81720 4.78265 3.07113 1.68073 3.93421 3.69657 3.92856 3.26578 2.80647 1.75832 3.04616 3.29916 4.08687 3.14129 3.23159 2.92863 3.04975 2.86147 5.32996 4.04862 1273 - -
2.68618 4.42225 2.77519 2.73123 3.46354 2.40513 3.72494 3.29354 2.67741 2.69355 4.24690 2.90347 2.73739 3.18146 2.89801 2.37887 2.77519 2.98518 4.58477 3.61503

0.01347 4.70993 5.43228 0.61958 0.77255 0.48576 0.95510

1033 2.91527 5.05116 2.87457 2.89487 4.95412 1.37421 4.34483 4.43197 3.31141 4.01472 4.82780 3.08354 1.36149 3.53486 3.78806 2.80766 3.32421 3.90485 6.20443 4.85394 1274 - -

2.68618 4.42225 2.77519 2.73123 3.46354 2.40513 3.72494 3.29354 2.67741 2.69355 4.24690 2.90347 2.73739 3.18146 2.89801 2.37887 2.77519 2.98518 4.58477 3.61503

0.01347 4.70993 5.43228 0.61958 0.77255 0.48576 0.95510

1034 2.59028 5.27388 2.67800 1.81961 4.61499 3.36910 3.73217 4.09868 2.18987 3.57745 4.31598 2.76403 3.12976 2.83019 2.77557 2.10491 2.92497 3.66247 5.70867 3.99494 1275 - -

2.68618 4.42225 2.77519 2.73123 3.46354 2.40513 3.72494 3.29354 2.67741 2.69355 4.24690 2.90347 2.73739 3.18146 2.89801 2.37887 2.77519 2.98518 4.58477 3.61503

0.01347 4.70993 5.43228 0.61958 0.77255 0.48576 0.95510

1035 2.73180 5.24110 2.76505 2.28293 4.57150 3.54638 3.73449 3.54088 1.92414 3.30524 4.28584 2.94134 2.44792 2.78303 2.36786 2.58053 2.86727 3.45684 5.68228 4.28126 1276 - -

2.68618 4.42225 2.77519 2.73123 3.46354 2.40513 3.72494 3.29354 2.67741 2.69355 4.24690 2.90347 2.73739 3.18146 2.89801 2.37887 2.77519 2.98518 4.58477 3.61503

0.01347 4.70993 5.43228 0.61958 0.77255 0.48576 0.95510

1036 2.92270 4.57701 3.52313 3.29783 1.63102 3.88787 4.02190 3.07524 3.25720 2.63747 3.69221 3.44313 1.80724 3.53458 3.57568 3.15891 3.15777 2.86274 4.80077 2.98342 1277 - -

2.68618 4.42225 2.77519 2.73123 3.46354 2.40513 3.72494 3.29354 2.67741 2.69355 4.24690 2.90347 2.73739 3.18146 2.89801 2.37887 2.77519 2.98518 4.58477 3.61503

0.01347 4.70993 5.43228 0.61958 0.77255 0.48576 0.95510

1037 2.77644 4.52280 3.61124 3.04863 3.66533 3.50509 3.38380 2.44015 3.00070 1.93039 3.62707 1.89703 4.12487 3.31265 3.36173 2.98677 2.63408 2.69235 5.10344 3.86378 1278 - -

2.68618 4.42225 2.77519 2.73123 3.46354 2.40513 3.72494 3.29354 2.67741 2.69355 4.24690 2.90347 2.73739 3.18146 2.89801 2.37887 2.77519 2.98518 4.58477 3.61503

0.10760 4.70993 2.37508 0.61958 0.77255 0.48576 0.95510

1038 2.73517 4.33830 3.60545 2.65747 1.77465 3.54577 4.04168 2.65614 3.01041 2.24808 3.21511 3.60030 4.14334 3.46571 3.48419 2.90657 2.96703 2.48080 4.93442 3.18322 1279 - -

2.68618 4.42225 2.77519 2.73123 3.46354 2.40513 3.72494 3.29354 2.67741 2.69355 4.24690 2.90347 2.73739 3.18146 2.89801 2.37887 2.77519 2.98518 4.58477 3.61503

0.01479 4.61712 5.33947 0.61958 0.77255 0.65719 0.73044

1039 2.71453 5.23521 2.21352 2.30197 4.54551 2.70913 1.88823 4.01362 2.44493 3.52574 4.28947 3.00431 3.94550 2.86693 3.01517 2.76249 3.02770 3.38330 5.68608 3.97093 1280 - -

2.68618 4.42225 2.77519 2.73123 3.46354 2.40513 3.72494 3.29354 2.67741 2.69355 4.24690 2.90347 2.73739 3.18146 2.89801 2.37887 2.77519 2.98518 4.58477 3.61503

0.01479 4.61712 5.33947 0.61958 0.77255 0.52259 0.89890

1040 2.75038 4.84885 2.20101 2.50543 4.06001 3.60599 3.81745 2.95241 2.53358 2.15161 3.32513 3.16593 3.99425 2.80825 3.09862 2.48951 2.75412 3.06917 5.37112 3.81938 1281 - -

2.68661 4.41803 2.77516 2.73120 3.46377 2.40541 3.72477 3.29295 2.67736 2.69244 4.24707 2.90357 2.73777 3.18139 2.89819 2.37920 2.77554 2.98553 4.58338 3.61507

0.48519 0.96803 5.38453 1.31727 0.31179 0.43127 1.04892

1041 2.76660 5.21022 2.84058 2.43444 4.15946 2.09717 3.47234 3.99556 2.14649 3.50383 4.25817 2.81343 2.81597 2.84655 2.72608 2.52908 2.91275 3.58631 4.04727 4.26541 1286 - -

2.68618 4.42225 2.77519 2.73123 3.46354 2.40513 3.72494 3.29354 2.67741 2.69355 4.24690 2.90347 2.73739 3.18146 2.89801 2.37887 2.77519 2.98518 4.58477 3.61503

0.37953 4.70993 1.18153 0.61958 0.77255 0.48576 0.95510

1042 2.59110 5.14355 2.46427 2.24446 4.47763 3.30264 3.46458 3.73814 2.06342 3.44382 4.18759 2.66420 3.44239 2.72381 2.50918 2.41279 2.74602 3.52837 5.58237 4.17812 1287 - -

2.68618 4.42225 2.77519 2.73123 3.46354 2.40513 3.72494 3.29354 2.67741 2.69355 4.24690 2.90347 2.73739 3.18146 2.89801 2.37887 2.77519 2.98518 4.58477 3.61503

0.07845 4.34977 2.77191 0.61958 0.77255 0.97928 0.47093

1043 2.46972 4.28819 3.41302 2.92820 3.41615 3.38433 3.22629 2.44725 2.97516 2.05735 3.15692 3.40841 4.00655 3.26516 3.15561 2.74379 2.70210 2.55645 3.92106 3.21936 1288 - -

2.68627 4.42234 2.77528 2.73106 3.46280 2.40518 3.72503 3.29363 2.67721 2.69364 4.24699 2.90356 2.73748 3.18155 2.89803 2.37890 2.77520 2.98520 4.58486 3.61488

0.64007 1.75264 1.20587 0.91432 0.51214 0.31706 1.30300

1044 2.67450 3.46620 3.62805 2.88105 3.48137 3.48760 3.63811 2.50102 2.89775 2.32940 3.26200 3.44236 2.12620 3.29735 3.33650 2.80123 2.90643 2.45424 4.93900 3.71292 1293 - -

2.68618 4.42225 2.77519 2.73123 3.46354 2.40513 3.72494 3.29354 2.67741 2.69355 4.24690 2.90347 2.73739 3.18146 2.89801 2.37887 2.77519 2.98518 4.58477 3.61503
0.01757 4.44596 5.16831 0.61958 0.77255 0.88418 0.53282
1045 2.59173 2.32919 3.64930 3.44863 3.29282 3.76000 4.05681 2.48219 3.34512 2.05324 3.06363 3.69843 3.76476 3.29748 3.56089 3.03962 2.65528 2.03072 4.79513 3.59800 1294 - -
2.68618 4.42225 2.77519 2.73123 3.46354 2.40513 3.72494 3.29354 2.67741 2.69355 4.24690 2.90347 2.73739 3.18146 2.89801 2.37887 2.77519 2.98518 4.58477 3.61503
0.03518 4.44596 3.77893 0.61958 0.77255 0.29885 1.35352
1046 2.69158 5.20813 3.03900 2.37340 4.52759 3.40852 3.73634 3.59202 1.71187 3.33859 4.25615 2.39043 3.65646 2.84197 2.67228 2.35620 2.68199 3.36294 5.65625 4.26284 1295 - -
2.68620 4.42227 2.77522 2.73125 3.46356 2.40506 3.72497 3.29356 2.67732 2.69357 4.24692 2.90349 2.73736 3.18148 2.89803 2.37885 2.77522 2.98520 4.58479 3.61505
0.05236 3.06657 5.41901 0.65143 0.73668 0.46899 0.98252
1047 2.76635 5.22735 2.63041 2.42740 4.55229 2.67914 3.73705 3.56578 2.42589 3.26737 4.27364 2.59770 3.28380 2.84036 2.41398 1.99653 2.89134 3.46540 5.67247 4.27411 1298 - -
2.68682 4.41898 2.77508 2.73200 3.46276 2.40504 3.72512 3.29312 2.67776 2.69335 4.24573 2.90289 2.73664 3.18234 2.89885 2.37931 2.77517 2.98559 4.58167 3.61394
0.36883 1.19046 5.43228 2.58261 0.07858 0.48576 0.95510
1048 2.77082 4.82338 3.03866 2.49172 4.02386 3.46357 3.43378 1.85732 2.49261 2.75785 3.90595 3.21111 2.64486 3.04073 3.14055 2.85068 2.71124 3.07647 5.35378 4.05219 1320 - -
2.68672 4.42304 2.77538 2.73106 3.46409 2.40480 3.72485 3.29366 2.67759 2.69339 4.24679 2.90303 2.73796 3.18118 2.89783 2.37877 2.77503 2.98480 4.58556 3.61503
0.43150 1.06104 5.43228 1.93022 0.15679 0.48576 0.95510
1049 2.82577 4.56244 4.01309 3.44980 3.14632 3.96141 2.73703 3.05303 3.31816 2.74081 3.67658 3.75310 4.32982 3.61841 2.98832 3.23098 3.19696 2.54417 2.32386 1.56184 1338 - -
2.68618 4.42225 2.77519 2.73123 3.46354 2.40513 3.72494 3.29354 2.67741 2.69355 4.24690 2.90347 2.73739 3.18146 2.89801 2.37887 2.77519 2.98518 4.58477 3.61503
0.01347 4.70993 5.43228 0.61958 0.77255 0.48576 0.95510
1050 2.15721 3.20572 4.27382 3.68537 2.69323 3.49867 4.18609 2.68811 3.55957 2.43582 3.11101 3.87736 4.25071 3.77323 3.72765 1.86757 3.00591 2.49426 3.67481 2.66977 1339 - -
2.68618 4.42225 2.77519 2.73123 3.46354 2.40513 3.72494 3.29354 2.67741 2.69355 4.24690 2.90347 2.73739 3.18146 2.89801 2.37887 2.77519 2.98518 4.58477 3.61503
0.01347 4.70993 5.43228 0.61958 0.77255 0.48576 0.95510
1051 2.24098 4.29168 4.09006 3.50880 3.40030 3.84882 3.67065 2.37762 3.41053 2.32518 2.26336 3.77184 4.22046 3.65311 3.17499 2.29242 2.55224 2.33347 4.23960 3.50998 1340 - -
2.68618 4.42225 2.77519 2.73123 3.46354 2.40513 3.72494 3.29354 2.67741 2.69355 4.24690 2.90347 2.73739 3.18146 2.89801 2.37887 2.77519 2.98518 4.58477 3.61503
0.01347 4.70993 5.43228 0.61958 0.77255 0.48576 0.95510
1052 3.00024 5.52734 2.35989 1.23432 4.84699 3.57671 3.91038 4.34067 2.62981 3.82508 4.59786 2.82630 4.06819 3.03306 3.26998 2.81914 2.02247 3.90863 5.97004 4.52811 1341 - -
2.68618 4.42225 2.77519 2.73123 3.46354 2.40513 3.72494 3.29354 2.67741 2.69355 4.24690 2.90347 2.73739 3.18146 2.89801 2.37887 2.77519 2.98518 4.58477 3.61503
0.01347 4.70993 5.43228 0.61958 0.77255 0.48576 0.95510
1053 2.80207 5.03865 2.86710 2.60400 4.16549 1.84288 2.85457 3.71400 2.61467 3.29822 4.10959 2.50447 4.00556 2.96331 3.08184 2.82248 3.03445 3.38061 4.33449 2.69644 1342 - -
2.68618 4.42225 2.77519 2.73123 3.46354 2.40513 3.72494 3.29354 2.67741 2.69355 4.24690 2.90347 2.73739 3.18146 2.89801 2.37887 2.77519 2.98518 4.58477 3.61503
0.01347 4.70993 5.43228 0.61958 0.77255 0.48576 0.95510
1054 2.77451 4.56917 3.54355 2.97798 3.70972 3.72330 3.96244 3.08588 2.93435 2.37227 3.23857 3.41230 3.74937 2.15014 3.24164 2.17174 3.00593 2.85017 2.51973 3.36406 1343 - -
2.68618 4.42225 2.77519 2.73123 3.46354 2.40513 3.72494 3.29354 2.67741 2.69355 4.24690 2.90347 2.73739 3.18146 2.89801 2.37887 2.77519 2.98518 4.58477 3.61503
0.01347 4.70993 5.43228 0.61958 0.77255 0.48576 0.95510
1055 3.79130 5.03304 5.26931 4.88464 0.81100 4.74097 4.03969 3.07766 4.68588 1.94354 3.99923 4.57978 5.03747 4.60930 4.63279 4.08760 4.00941 3.33636 3.62240 1.98779 1344 - -
2.68618 4.42225 2.77519 2.73123 3.46354 2.40513 3.72494 3.29354 2.67741 2.69355 4.24690 2.90347 2.73739 3.18146 2.89801 2.37887 2.77519 2.98518 4.58477 3.61503
0.01347 4.70993 5.43228 0.61958 0.77255 0.48576 0.95510

1056 2.37980 4.18523 4.51787 3.91652 3.03807 3.11447 4.23827 1.88482 3.74720 2.19389 2.11503 4.00411 4.28282 3.91897 3.83241 2.90570 2.58545 2.35532 4.07331 3.45593 1345 - -
2.68618 4.42225 2.77519 2.73123 3.46354 2.40513 3.72494 3.29354 2.67741 2.69355 4.24690 2.90347 2.73739 3.18146 2.89801 2.37887 2.77519 2.98518 4.58477 3.61503
0.01347 4.70993 5.43228 0.61958 0.77255 0.48576 0.95510
1057 2.34299 4.92522 2.91577 2.63691 4.15166 3.39217 3.81583 3.56160 2.28499 2.27908 3.99970 2.90760 3.99794 2.88968 3.08672 2.41392 2.83887 3.04216 5.43651 2.59768 1346 - -
2.68618 4.42225 2.77519 2.73123 3.46354 2.40513 3.72494 3.29354 2.67741 2.69355 4.24690 2.90347 2.73739 3.18146 2.89801 2.37887 2.77519 2.98518 4.58477 3.61503
0.01347 4.70993 5.43228 0.61958 0.77255 0.48576 0.95510
1058 2.78245 5.23635 2.35928 1.49190 4.19789 3.55165 3.75128 3.68828 2.50153 2.94465 4.28473 2.93811 3.95155 2.47333 2.99151 2.61872 3.01466 3.61348 5.68335 4.28526 1347 - -
2.68618 4.42225 2.77519 2.73123 3.46354 2.40513 3.72494 3.29354 2.67741 2.69355 4.24690 2.90347 2.73739 3.18146 2.89801 2.37887 2.77519 2.98518 4.58477 3.61503
0.01347 4.70993 5.43228 0.61958 0.77255 0.48576 0.95510
1059 3.13171 4.47453 5.00689 4.41773 3.44949 4.36734 4.72401 1.51099 4.24880 1.62359 2.16169 4.49149 3.58795 4.37967 4.30180 3.69203 3.36422 1.72778 5.17562 4.02768 1348 - -
2.68618 4.42225 2.77519 2.73123 3.46354 2.40513 3.72494 3.29354 2.67741 2.69355 4.24690 2.90347 2.73739 3.18146 2.89801 2.37887 2.77519 2.98518 4.58477 3.61503
0.01347 4.70993 5.43228 0.61958 0.77255 0.48576 0.95510
1060 2.42337 4.23418 3.39742 1.77041 3.69090 3.67722 3.90539 2.15571 2.62427 2.60522 3.77586 3.19057 4.06181 3.14064 3.11184 2.84001 2.91753 2.97655 5.23770 3.78289 1349 - -
2.68618 4.42225 2.77519 2.73123 3.46354 2.40513 3.72494 3.29354 2.67741 2.69355 4.24690 2.90347 2.73739 3.18146 2.89801 2.37887 2.77519 2.98518 4.58477 3.61503
0.01347 4.70993 5.43228 0.61958 0.77255 0.48576 0.95510
1061 2.62409 4.46240 3.69496 3.01851 2.16115 3.47875 4.01954 2.75297 3.01856 2.52164 3.32026 3.16912 4.14011 3.24446 3.28857 2.40864 2.09970 2.65225 5.04803 3.66700 1350 - -
2.68618 4.42225 2.77519 2.73123 3.46354 2.40513 3.72494 3.29354 2.67741 2.69355 4.24690 2.90347 2.73739 3.18146 2.89801 2.37887 2.77519 2.98518 4.58477 3.61503
0.20571 4.70993 1.73201 0.61958 0.77255 0.48576 0.95510
1062 2.70822 5.13547 2.80111 2.43074 4.44720 3.21713 2.36366 3.91044 2.43450 3.42645 4.18577 2.55685 3.29293 2.73363 2.71410 2.20071 2.93967 3.39966 5.58854 3.47927 1351 - -
2.68618 4.42225 2.77519 2.73123 3.46354 2.40513 3.72494 3.29354 2.67741 2.69355 4.24690 2.90347 2.73739 3.18146 2.89801 2.37887 2.77519 2.98518 4.58477 3.61503
0.12179 4.52052 2.26548 0.61958 0.77255 0.35905 1.19844
1063 2.67489 5.23938 2.21511 1.91058 4.57958 3.23204 3.69973 4.06270 2.32350 3.54254 3.93420 2.42264 3.62314 2.79812 2.59559 2.52939 2.97003 3.46895 5.67464 4.26562 1352 - -
2.68618 4.42225 2.77519 2.73123 3.46354 2.40513 3.72494 3.29354 2.67741 2.69355 4.24690 2.90347 2.73739 3.18146 2.89801 2.37887 2.77519 2.98518 4.58477 3.61503
0.28686 4.61104 1.42946 0.61958 0.77255 0.66697 0.72003
1064 2.51151 4.92928 3.00881 2.31908 3.76377 3.35597 3.66521 3.62036 2.27438 2.93665 3.99345 2.69257 3.62326 2.79935 2.19669 2.55241 2.79984 3.05328 5.41552 3.80144 1353 - -
2.68618 4.42225 2.77519 2.73123 3.46354 2.40513 3.72494 3.29354 2.67741 2.69355 4.24690 2.90347 2.73739 3.18146 2.89801 2.37887 2.77519 2.98518 4.58477 3.61503
0.01948 4.34367 5.06601 0.61958 0.77255 0.98474 0.46767
1065 2.64343 4.39925 3.17208 2.66076 3.35833 3.46946 3.85660 2.32671 2.57106 2.35118 2.59892 3.32144 3.49904 3.16829 3.22747 2.84874 2.87513 2.51454 4.97676 3.73580 1354 - -
2.68618 4.42225 2.77519 2.73123 3.46354 2.40513 3.72494 3.29354 2.67741 2.69355 4.24690 2.90347 2.73739 3.18146 2.89801 2.37887 2.77519 2.98518 4.58477 3.61503
0.03778 4.34367 3.72621 0.61958 0.77255 0.98474 0.46767
1066 2.69688 4.86767 3.11114 2.34816 4.11335 3.51709 3.69438 3.33078 2.10923 2.10170 3.94105 2.93767 3.71912 2.84777 2.38480 2.64341 2.92266 3.20434 5.35553 4.04080 1355 - -
2.68618 4.42225 2.77519 2.73123 3.46354 2.40513 3.72494 3.29354 2.67741 2.69355 4.24690 2.90347 2.73739 3.18146 2.89801 2.37887 2.77519 2.98518 4.58477 3.61503
0.01984 4.32573 5.04808 0.61958 0.77255 0.95900 0.48333
1067 2.38761 5.09896 2.82697 2.19402 4.41737 3.43796 3.30296 3.42805 2.23566 3.39235 4.14703 2.64764 3.83146 2.48493 2.64453 2.26411 2.88664 3.33955 5.54742 4.15393 1356 - -
2.68629 4.42205 2.77530 2.73128 3.46365 2.40510 3.72505 3.29365 2.67737 2.69350 4.24701 2.90351 2.73739 3.18157 2.89782 2.37857 2.77530 2.98521 4.58488 3.61514

0.22611 1.62933 5.06601 0.93663 0.49749 0.26607 1.45407

1068 2.56548 5.03927 3.12514 2.51222 3.91107 3.33083 2.98343 3.72790 2.35429 3.19154 4.10377 2.94983 3.60784 2.91687 2.91870 2.29024 2.08419 3.38288 5.52712 2.85676 1363 - -

2.68618 4.42225 2.77519 2.73123 3.46354 2.40513 3.72494 3.29354 2.67741 2.69355 4.24690 2.90347 2.73739 3.18146 2.89801 2.37887 2.77519 2.98518 4.58477 3.61503

0.01347 4.70993 5.43228 0.61958 0.77255 0.48576 0.95510

1069 2.28498 5.23714 2.85785 2.32672 4.56585 3.54918 3.73662 3.65864 2.07570 3.53455 4.28259 3.02364 2.96826 2.65011 1.97670 2.74044 2.88981 3.37758 5.67896 4.28019 1364 - -

2.68618 4.42225 2.77519 2.73123 3.46354 2.40513 3.72494 3.29354 2.67741 2.69355 4.24690 2.90347 2.73739 3.18146 2.89801 2.37887 2.77519 2.98518 4.58477 3.61503

0.01347 4.70993 5.43228 0.61958 0.77255 0.48576 0.95510

1070 2.78332 2.38312 4.49699 3.89851 1.76205 3.92191 3.42806 2.42801 3.29532 2.38834 3.31648 3.99678 4.28783 3.90981 3.82747 3.22209 3.01543 2.07785 4.79756 3.19541 1365 - -

2.68618 4.42225 2.77519 2.73123 3.46354 2.40513 3.72494 3.29354 2.67741 2.69355 4.24690 2.90347 2.73739 3.18146 2.89801 2.37887 2.77519 2.98518 4.58477 3.61503

0.01347 4.70993 5.43228 0.61958 0.77255 0.48576 0.95510

1071 2.89431 4.76182 3.73821 3.12593 3.94952 3.84805 4.02780 3.21016 2.67881 2.15287 3.85501 3.53266 4.23013 3.27571 1.25981 3.12348 2.98132 2.49836 5.32298 4.09848 1366 - -

2.68618 4.42225 2.77519 2.73123 3.46354 2.40513 3.72494 3.29354 2.67741 2.69355 4.24690 2.90347 2.73739 3.18146 2.89801 2.37887 2.77519 2.98518 4.58477 3.61503

0.01347 4.70993 5.43228 0.61958 0.77255 0.48576 0.95510

1072 2.47632 4.53182 4.45568 4.03990 4.19428 3.68985 4.81208 2.88439 3.94915 3.09480 4.11123 4.08979 4.35698 4.21923 4.17244 3.11696 0.96829 1.64127 5.75934 4.56957 1367 - -

2.68618 4.42225 2.77519 2.73123 3.46354 2.40513 3.72494 3.29354 2.67741 2.69355 4.24690 2.90347 2.73739 3.18146 2.89801 2.37887 2.77519 2.98518 4.58477 3.61503

0.01347 4.70993 5.43228 0.61958 0.77255 0.48576 0.95510

1073 2.72994 5.10956 2.98571 2.47435 3.60665 3.56830 3.50235 3.60219 2.01965 2.94610 3.49530 2.05389 3.67053 2.76108 2.77888 2.65264 2.99728 3.46578 5.58199 3.46530 1368 - -

2.68618 4.42225 2.77519 2.73123 3.46354 2.40513 3.72494 3.29354 2.67741 2.69355 4.24690 2.90347 2.73739 3.18146 2.89801 2.37887 2.77519 2.98518 4.58477 3.61503

0.01347 4.70993 5.43228 0.61958 0.77255 0.48576 0.95510

1074 3.06662 5.62288 1.52864 2.40293 4.94143 3.58805 3.95019 4.44361 2.52430 3.91604 4.69526 1.48257 4.10127 3.07693 3.03846 2.76269 3.31967 4.00408 6.05374 4.59918 1369 - -

2.68618 4.42225 2.77519 2.73123 3.46354 2.40513 3.72494 3.29354 2.67741 2.69355 4.24690 2.90347 2.73739 3.18146 2.89801 2.37887 2.77519 2.98518 4.58477 3.61503

0.01347 4.70993 5.43228 0.61958 0.77255 0.48576 0.95510

1075 2.92663 5.30558 2.90875 2.27668 4.64857 3.05146 3.89752 4.10651 2.58756 3.63833 4.42724 3.09558 1.46437 2.84506 3.17308 2.12243 3.17883 3.71211 5.81541 4.42594 1370 - -

2.68618 4.42225 2.77519 2.73123 3.46354 2.40513 3.72494 3.29354 2.67741 2.69355 4.24690 2.90347 2.73739 3.18146 2.89801 2.37887 2.77519 2.98518 4.58477 3.61503

0.01347 4.70993 5.43228 0.61958 0.77255 0.48576 0.95510

1076 2.55894 5.27977 1.97360 2.05381 4.61733 3.54418 3.74192 4.09967 2.48489 3.58186 4.32352 2.77352 3.94449 2.72114 2.66931 2.15307 2.70647 3.66659 5.71623 4.00684 1371 - -

2.68618 4.42225 2.77519 2.73123 3.46354 2.40513 3.72494 3.29354 2.67741 2.69355 4.24690 2.90347 2.73739 3.18146 2.89801 2.37887 2.77519 2.98518 4.58477 3.61503

0.01347 4.70993 5.43228 0.61958 0.77255 0.48576 0.95510

1077 2.78712 5.27246 2.69800 1.87986 4.60848 3.55215 3.74419 4.08627 1.73146 2.58987 4.31699 2.92504 3.95028 2.61546 2.86066 2.66108 2.93568 3.65853 5.70798 4.30485 1372 - -

2.68618 4.42225 2.77519 2.73123 3.46354 2.40513 3.72494 3.29354 2.67741 2.69355 4.24690 2.90347 2.73739 3.18146 2.89801 2.37887 2.77519 2.98518 4.58477 3.61503

0.01347 4.70993 5.43228 0.61958 0.77255 0.48576 0.95510

1078 0.38865 4.59246 4.34471 4.23550 5.03154 3.38696 5.14663 4.37083 4.28668 4.18385 5.04286 4.05809 4.22279 4.52050 4.44602 2.71885 3.24383 3.73441 6.41562 5.26467 1373 - -

2.68618 4.42225 2.77519 2.73123 3.46354 2.40513 3.72494 3.29354 2.67741 2.69355 4.24690 2.90347 2.73739 3.18146 2.89801 2.37887 2.77519 2.98518 4.58477 3.61503

0.01347 4.70993 5.43228 0.61958 0.77255 0.48576 0.95510

1079 2.67930 5.27872 1.91494 2.49232 4.59508 3.56240 1.95233 4.06496 2.39690 3.57410 4.33687 3.04067 3.98326 2.90538 3.05171 2.32882 2.88700 3.65639 5.73151 4.14333 1374 - -

2.68618 4.42225 2.77519 2.73123 3.46354 2.40513 3.72494 3.29354 2.67741 2.69355 4.24690 2.90347 2.73739 3.18146 2.89801 2.37887 2.77519 2.98518 4.58477 3.61503
0.01347 4.70993 5.43228 0.61958 0.77255 0.48576 0.95510
1080 2.11613 4.20314 4.67183 4.06542 2.63765 3.97751 4.30127 2.45525 3.87495 1.70908 3.28674 4.10299 4.33700 4.02240 3.92046 3.28546 3.04745 1.57116 4.08080 3.43319 1375 - -
2.68618 4.42225 2.77519 2.73123 3.46354 2.40513 3.72494 3.29354 2.67741 2.69355 4.24690 2.90347 2.73739 3.18146 2.89801 2.37887 2.77519 2.98518 4.58477 3.61503
0.01347 4.70993 5.43228 0.61958 0.77255 0.48576 0.95510
1081 3.24879 4.58963 5.00331 4.45515 1.06406 4.40472 4.42221 2.14163 4.27947 2.29052 3.47209 4.45338 4.72031 4.37069 4.31789 3.73007 3.47844 2.04100 4.74060 2.81624 1376 - -
2.68618 4.42225 2.77519 2.73123 3.46354 2.40513 3.72494 3.29354 2.67741 2.69355 4.24690 2.90347 2.73739 3.18146 2.89801 2.37887 2.77519 2.98518 4.58477 3.61503
0.01347 4.70993 5.43228 0.61958 0.77255 0.48576 0.95510
1082 4.74046 5.68184 5.58087 5.50571 1.13587 5.23790 3.89367 4.28102 5.28280 3.27427 4.80978 4.77932 5.49114 4.94632 5.09090 4.64531 4.93511 4.24758 3.96878 0.70821 1377 - -
2.68618 4.42225 2.77519 2.73123 3.46354 2.40513 3.72494 3.29354 2.67741 2.69355 4.24690 2.90347 2.73739 3.18146 2.89801 2.37887 2.77519 2.98518 4.58477 3.61503
0.01347 4.70993 5.43228 0.61958 0.77255 0.48576 0.95510
1083 3.46041 4.72520 5.42524 4.87496 3.69045 4.85341 5.29906 1.81910 4.74499 1.33947 2.97661 4.98989 5.09160 4.86370 4.81058 4.21726 3.48242 1.08685 5.63662 4.51282 1378 - -
2.68618 4.42225 2.77519 2.73123 3.46354 2.40513 3.72494 3.29354 2.67741 2.69355 4.24690 2.90347 2.73739 3.18146 2.89801 2.37887 2.77519 2.98518 4.58477 3.61503
0.01347 4.70993 5.43228 0.61958 0.77255 0.48576 0.95510
1084 3.06789 4.96226 4.01580 3.96553 5.10117 3.65380 5.09563 4.74507 4.12864 4.39910 5.31969 4.10111 0.37386 4.45693 4.34769 3.00282 3.60020 4.13195 6.37547 5.19770 1379 - -
2.68618 4.42225 2.77519 2.73123 3.46354 2.40513 3.72494 3.29354 2.67741 2.69355 4.24690 2.90347 2.73739 3.18146 2.89801 2.37887 2.77519 2.98518 4.58477 3.61503
0.01347 4.70993 5.43228 0.61958 0.77255 0.48576 0.95510
1085 2.74637 4.37352 4.73288 4.15664 1.28320 4.13374 4.30777 2.40686 3.98421 2.37949 3.41572 4.20286 4.48380 4.12617 4.05206 3.44546 3.22221 2.00544 4.74344 2.48722 1380 - -
2.68618 4.42225 2.77519 2.73123 3.46354 2.40513 3.72494 3.29354 2.67741 2.69355 4.24690 2.90347 2.73739 3.18146 2.89801 2.37887 2.77519 2.98518 4.58477 3.61503
0.01347 4.70993 5.43228 0.61958 0.77255 0.48576 0.95510
1086 3.07856 4.59734 4.25628 3.72212 2.16468 3.88829 4.04714 3.08364 3.62813 2.44686 3.72344 3.93264 4.45316 3.84602 3.85505 1.55803 3.31471 2.89131 4.53881 1.69898 1381 - -
2.68618 4.42225 2.77519 2.73123 3.46354 2.40513 3.72494 3.29354 2.67741 2.69355 4.24690 2.90347 2.73739 3.18146 2.89801 2.37887 2.77519 2.98518 4.58477 3.61503
0.01347 4.70993 5.43228 0.61958 0.77255 0.48576 0.95510
1087 2.05413 4.20296 3.05329 2.93401 3.81132 3.12743 3.96270 2.98927 2.90528 2.87462 3.74758 3.37671 3.51889 3.22548 3.30193 2.30860 2.78285 1.83091 5.21502 3.95689 1382 - -
2.68618 4.42225 2.77519 2.73123 3.46354 2.40513 3.72494 3.29354 2.67741 2.69355 4.24690 2.90347 2.73739 3.18146 2.89801 2.37887 2.77519 2.98518 4.58477 3.61503
0.01347 4.70993 5.43228 0.61958 0.77255 0.48576 0.95510
1088 2.61055 4.95857 3.18457 2.62827 4.20581 2.36310 3.62244 3.62051 2.33403 3.22650 4.04135 3.14386 3.99631 2.96724 2.71277 2.17981 2.60668 2.27351 5.47250 4.14178 1383 - -
2.68618 4.42225 2.77519 2.73123 3.46354 2.40513 3.72494 3.29354 2.67741 2.69355 4.24690 2.90347 2.73739 3.18146 2.89801 2.37887 2.77519 2.98518 4.58477 3.61503
0.01347 4.70993 5.43228 0.61958 0.77255 0.48576 0.95510
1089 2.67412 2.84525 4.29700 3.70697 2.86927 3.88488 4.19181 2.39687 2.24746 1.83619 2.63366 3.51446 4.25352 3.53735 3.73680 3.17424 3.00474 2.48562 4.53401 3.43749 1384 - -
2.68618 4.42225 2.77519 2.73123 3.46354 2.40513 3.72494 3.29354 2.67741 2.69355 4.24690 2.90347 2.73739 3.18146 2.89801 2.37887 2.77519 2.98518 4.58477 3.61503
0.01347 4.70993 5.43228 0.61958 0.77255 0.48576 0.95510
1090 2.67067 4.53320 2.35376 3.01877 3.67678 3.73320 3.98089 2.55237 2.97976 2.24127 2.36888 3.16202 4.11401 3.28880 3.35008 2.34922 3.00262 2.63072 5.11016 3.67100 1385 - -
2.68618 4.42225 2.77519 2.73123 3.46354 2.40513 3.72494 3.29354 2.67741 2.69355 4.24690 2.90347 2.73739 3.18146 2.89801 2.37887 2.77519 2.98518 4.58477 3.61503
0.01347 4.70993 5.43228 0.61958 0.77255 0.48576 0.95510

1091 2.59261 4.25153 4.22657 3.00726 2.72923 3.87342 4.17550 2.26928 3.52110 2.12960 3.21300 3.85095 4.24304 3.32015 3.14752 2.99587 3.00449 1.66263 4.86260 3.17608 1386 - -
2.68618 4.42225 2.77519 2.73123 3.46354 2.40513 3.72494 3.29354 2.67741 2.69355 4.24690 2.90347 2.73739 3.18146 2.89801 2.37887 2.77519 2.98518 4.58477 3.61503
0.01347 4.70993 5.43228 0.61958 0.77255 0.48576 0.95510
1092 2.63519 5.04720 3.12052 2.41456 3.86837 3.25964 3.27297 3.44064 2.49068 2.89804 3.50888 2.54147 3.97155 2.82129 2.12683 2.39774 2.86376 3.20067 5.53332 3.80878 1387 - -
2.68629 4.42236 2.77530 2.73134 3.46365 2.40523 3.72505 3.29365 2.67751 2.69356 4.24661 2.90357 2.73750 3.18144 2.89687 2.37894 2.77511 2.98515 4.58488 3.61514
0.45332 1.02134 5.43228 0.10204 2.33298 0.48576 0.95510
1093 2.53970 4.57913 3.38000 2.82894 2.98128 3.71343 3.24249 2.99292 2.67226 2.58787 3.67730 3.26689 3.71465 3.23651 3.18288 2.66567 2.64155 2.78547 3.95334 2.12965 1389 - -
2.68618 4.42225 2.77519 2.73123 3.46354 2.40513 3.72494 3.29354 2.67741 2.69355 4.24690 2.90347 2.73739 3.18146 2.89801 2.37887 2.77519 2.98518 4.58477 3.61503
0.02639 4.70993 4.07251 0.61958 0.77255 0.48576 0.95510
1094 2.69419 4.55444 3.09321 2.82758 3.37895 3.34269 3.42420 2.94816 2.93993 1.82816 3.65389 3.05093 4.09793 3.25306 3.31976 2.63261 2.92152 2.02203 5.12689 3.87942 1390 - -
2.68618 4.42225 2.77519 2.73123 3.46354 2.40513 3.72494 3.29354 2.67741 2.69355 4.24690 2.90347 2.73739 3.18146 2.89801 2.37887 2.77519 2.98518 4.58477 3.61503
0.01364 4.69719 5.41953 0.61958 0.77255 0.51213 0.91434
1095 2.78041 4.69779 3.00207 2.84000 3.19997 2.63875 3.89830 3.25067 2.42855 2.92318 3.78998 3.30888 4.06847 3.14432 3.00624 2.90870 3.01173 2.99376 3.08820 1.97091 1391 - -
2.68618 4.42225 2.77519 2.73123 3.46354 2.40513 3.72494 3.29354 2.67741 2.69355 4.24690 2.90347 2.73739 3.18146 2.89801 2.37887 2.77519 2.98518 4.58477 3.61503
0.01364 4.69719 5.41953 0.61958 0.77255 0.51213 0.91434
1096 2.55076 5.14865 2.97149 1.93349 3.85754 2.72597 3.74879 3.89887 2.37019 2.90364 4.20235 2.84182 3.94811 2.69292 2.61232 2.66011 2.99301 2.66327 5.61123 4.23144 1392 - -
2.68618 4.42225 2.77519 2.73123 3.46354 2.40513 3.72494 3.29354 2.67741 2.69355 4.24690 2.90347 2.73739 3.18146 2.89801 2.37887 2.77519 2.98518 4.58477 3.61503
0.04659 4.69719 3.31320 0.61958 0.77255 0.51213 0.91434
1097 2.75777 5.10786 3.07529 2.51814 4.05351 3.28140 3.54536 3.83873 2.50442 3.38639 3.91223 2.57955 2.26583 2.57968 2.07822 2.75338 2.98839 3.46676 5.57534 3.40294 1393 - -
2.68618 4.42225 2.77519 2.73123 3.46354 2.40513 3.72494 3.29354 2.67741 2.69355 4.24690 2.90347 2.73739 3.18146 2.89801 2.37887 2.77519 2.98518 4.58477 3.61503
0.12123 4.66469 2.25624 0.61958 0.77255 0.52307 0.89821
1098 2.64899 4.97731 2.64342 2.53147 3.78256 3.19394 3.23245 3.36924 2.44612 3.01475 4.04330 1.89580 3.93125 2.87947 2.99750 2.66574 2.95262 2.91966 5.46860 3.68751 1394 - -
2.68612 4.42226 2.77521 2.73125 3.46355 2.40514 3.72496 3.29355 2.67742 2.69356 4.24691 2.90348 2.73741 3.18148 2.89802 2.37877 2.77521 2.98520 4.58478 3.61504
0.07725 2.66859 5.29940 0.35268 1.21335 0.48704 0.95306
1099 2.65032 4.50513 3.57731 2.89573 3.43552 3.18519 3.96588 2.44820 2.97094 2.48839 3.60761 3.43117 3.25938 3.13827 3.33807 1.83115 2.69842 2.40879 5.08368 3.84305 1396 - -
2.68621 4.42238 2.77522 2.73124 3.46349 2.40521 3.72491 3.29367 2.67739 2.69368 4.24612 2.90305 2.73748 3.18128 2.89803 2.37884 2.77529 2.98523 4.58490 3.61516
0.55976 1.73004 1.38081 0.89555 0.52490 0.54447 0.86786
1100 2.68482 5.03199 3.00254 2.38844 3.79002 3.21347 2.55960 3.76434 2.32673 3.30914 4.08944 2.97586 3.65632 2.53936 2.06565 2.67999 2.82384 3.39305 5.49476 3.84315 1401 - -
2.68618 4.42225 2.77519 2.73123 3.46354 2.40513 3.72494 3.29354 2.67741 2.69355 4.24690 2.90347 2.73739 3.18146 2.89801 2.37887 2.77519 2.98518 4.58477 3.61503
0.01854 4.39279 5.11513 0.61958 0.77255 0.86327 0.54780
1101 2.67306 5.13719 1.97828 2.40016 4.10323 3.45837 3.68963 3.91922 2.33197 3.43859 4.20455 2.54768 3.87672 2.80398 2.95090 2.14648 2.49924 3.51716 5.60446 4.21016 1402 - -
2.68618 4.42225 2.77519 2.73123 3.46354 2.40513 3.72494 3.29354 2.67741 2.69355 4.24690 2.90347 2.73739 3.18146 2.89801 2.37887 2.77519 2.98518 4.58477 3.61503
0.03994 4.42380 3.60599 0.61958 0.77255 0.90775 0.51656
1102 2.62662 4.74013 3.00511 2.59402 2.47017 2.75909 3.44641 3.17965 2.44456 2.98357 3.82105 3.09564 3.63958 2.92305 2.56142 2.66523 2.72731 3.05350 5.26658 3.72051 1403 - -
2.68622 4.42229 2.77523 2.73127 3.46358 2.40516 3.72498 3.29358 2.67744 2.69339 4.24693 2.90350 2.73743 3.18150 2.89781 2.37876 2.77523 2.98522 4.58481 3.61507

0.07834 2.66785 5.12457 0.86633 0.54558 0.35213 1.21465
1103 2.44048 5.17997 2.76813 2.35395 3.64465 3.39066 3.73184 3.95415 2.23079 3.47078 4.22984 2.70719 3.93332 2.84035 2.83894 1.80650 2.64259 3.55238 5.63346 3.78908 1407 - -
2.68618 4.42225 2.77519 2.73123 3.46354 2.40513 3.72494 3.29354 2.67741 2.69355 4.24690 2.90347 2.73739 3.18146 2.89801 2.37887 2.77519 2.98518 4.58477 3.61503
0.01399 4.67231 5.39465 0.61958 0.77255 0.56078 0.84575
1104 2.68054 5.14931 2.85176 2.41837 4.13108 3.54529 3.40335 3.90531 2.36460 2.96374 3.78183 2.55413 2.47329 2.72940 2.59237 2.46612 2.98394 2.87848 5.60967 4.22747 1408 - -
2.68618 4.42225 2.77519 2.73123 3.46354 2.40513 3.72494 3.29354 2.67741 2.69355 4.24690 2.90347 2.73739 3.18146 2.89801 2.37887 2.77519 2.98518 4.58477 3.61503
0.01399 4.67231 5.39465 0.61958 0.77255 0.44156 1.03012
1105 2.62104 4.08782 2.64944 2.80154 3.89554 3.66588 3.89054 2.32585 2.58921 2.19468 3.61344 3.15955 4.05121 3.11274 2.42079 2.65795 2.72493 3.01478 5.26585 3.98636 1409 - -
2.68618 4.42225 2.77519 2.73123 3.46354 2.40513 3.72494 3.29354 2.67741 2.69355 4.24690 2.90347 2.73739 3.18146 2.89801 2.37887 2.77519 2.98518 4.58477 3.61503
0.01347 4.70993 5.43228 0.61958 0.77255 0.48576 0.95510
1106 2.59505 5.22475 2.25401 2.34048 4.54878 3.54789 3.37599 3.48301 2.22111 3.11842 4.27105 3.02395 3.63351 2.80226 2.02728 2.65870 2.91245 3.43236 5.67009 3.90056 1410 - -
2.68618 4.42225 2.77519 2.73123 3.46354 2.40513 3.72494 3.29354 2.67741 2.69355 4.24690 2.90347 2.73739 3.18146 2.89801 2.37887 2.77519 2.98518 4.58477 3.61503
0.01347 4.70993 5.43228 0.61958 0.77255 0.48576 0.95510
1107 2.39623 5.26022 2.73441 2.06210 4.59847 3.54194 3.72982 3.80439 2.27028 3.30005 4.30275 2.23461 3.93543 2.55293 2.49600 2.53649 2.95027 3.28955 5.69690 4.29023 1411 - -
2.68618 4.42225 2.77519 2.73123 3.46354 2.40513 3.72494 3.29354 2.67741 2.69355 4.24690 2.90347 2.73739 3.18146 2.89801 2.37887 2.77519 2.98518 4.58477 3.61503
0.01347 4.70993 5.43228 0.61958 0.77255 0.48576 0.95510
1108 2.43441 4.21607 4.36879 3.77537 2.54731 3.89583 3.32278 2.42329 3.63335 1.78686 2.92163 3.92828 4.26357 3.83116 3.48657 3.05826 2.15012 2.37648 4.83075 3.20392 1412 - -
2.68618 4.42225 2.77519 2.73123 3.46354 2.40513 3.72494 3.29354 2.67741 2.69355 4.24690 2.90347 2.73739 3.18146 2.89801 2.37887 2.77519 2.98518 4.58477 3.61503
0.01347 4.70993 5.43228 0.61958 0.77255 0.48576 0.95510
1109 2.50225 3.87317 4.12188 3.53941 3.21266 3.08857 4.14907 2.51143 3.43683 2.03179 3.17509 3.53442 3.78666 2.75562 3.65357 2.82336 3.00374 1.86047 4.88915 2.97970 1413 - -
2.68618 4.42225 2.77519 2.73123 3.46354 2.40513 3.72494 3.29354 2.67741 2.69355 4.24690 2.90347 2.73739 3.18146 2.89801 2.37887 2.77519 2.98518 4.58477 3.61503
0.01347 4.70993 5.43228 0.61958 0.77255 0.48576 0.95510
1110 2.70311 5.20823 3.05043 2.21213 4.52563 3.26449 3.55691 3.33972 1.64672 3.01630 4.25676 2.88391 3.94803 2.68900 2.55482 2.55646 3.00209 3.31816 5.65661 4.26605 1414 - -
2.68618 4.42225 2.77519 2.73123 3.46354 2.40513 3.72494 3.29354 2.67741 2.69355 4.24690 2.90347 2.73739 3.18146 2.89801 2.37887 2.77519 2.98518 4.58477 3.61503
0.01347 4.70993 5.43228 0.61958 0.77255 0.48576 0.95510
1111 2.78522 5.29097 1.80671 1.95985 4.63160 3.40661 3.74250 4.11615 2.37715 3.59407 3.95230 2.65721 3.94544 2.73626 2.59550 2.52215 2.90606 3.67945 5.72499 4.31377 1415 - -
2.68618 4.42225 2.77519 2.73123 3.46354 2.40513 3.72494 3.29354 2.67741 2.69355 4.24690 2.90347 2.73739 3.18146 2.89801 2.37887 2.77519 2.98518 4.58477 3.61503
0.01347 4.70993 5.43228 0.61958 0.77255 0.48576 0.95510
1112 2.27210 4.25471 4.42894 3.83726 3.32746 3.94881 4.26035 2.51395 3.69156 1.74052 3.15338 3.98607 4.31286 3.88751 3.82384 3.13924 3.05171 2.23379 4.86740 1.73605 1416 - -
2.68618 4.42225 2.77519 2.73123 3.46354 2.40513 3.72494 3.29354 2.67741 2.69355 4.24690 2.90347 2.73739 3.18146 2.89801 2.37887 2.77519 2.98518 4.58477 3.61503
0.01347 4.70993 5.43228 0.61958 0.77255 0.48576 0.95510
1113 2.70468 4.27484 4.77556 4.17775 3.38307 4.09958 4.44688 1.60453 3.99618 2.00383 2.86777 4.22535 4.45119 4.15157 4.05013 3.41352 2.80911 1.51342 4.96074 3.16097 1417 - -
2.68618 4.42225 2.77519 2.73123 3.46354 2.40513 3.72494 3.29354 2.67741 2.69355 4.24690 2.90347 2.73739 3.18146 2.89801 2.37887 2.77519 2.98518 4.58477 3.61503
0.01347 4.70993 5.43228 0.61958 0.77255 0.48576 0.95510
1114 2.84878 5.37402 2.07090 2.02843 4.71350 3.55378 3.78856 4.20419 2.02642 3.67605 4.42092 2.09030 3.98094 2.45801 2.99442 2.68871 2.97907 3.76258 5.80437 4.38222 1418 - -

2.68618 4.42225 2.77519 2.73123 3.46354 2.40513 3.72494 3.29354 2.67741 2.69355 4.24690 2.90347 2.73739 3.18146 2.89801 2.37887 2.77519 2.98518 4.58477 3.61503

0.01347 4.70993 5.43228 0.61958 0.77255 0.48576 0.95510

1115 2.79558 4.19333 4.60083 3.99877 2.45661 3.69522 3.19655 2.55359 3.81600 1.63007 2.98490 4.05069 4.30875 3.97233 3.87673 3.24982 3.02751 2.23654 2.37183 3.22057 1419 - -

2.68618 4.42225 2.77519 2.73123 3.46354 2.40513 3.72494 3.29354 2.67741 2.69355 4.24690 2.90347 2.73739 3.18146 2.89801 2.37887 2.77519 2.98518 4.58477 3.61503

0.39228 4.70993 1.15367 0.61958 0.77255 0.48576 0.95510

1116 3.33800 4.57518 5.27334 4.76871 3.75168 4.83945 5.41716 1.23097 4.67733 1.95252 3.52204 4.94860 5.07189 4.88978 4.82886 4.23603 3.58992 1.05562 5.78653 4.60733 1420 - -

2.68618 4.42225 2.77519 2.73123 3.46354 2.40513 3.72494 3.29354 2.67741 2.69355 4.24690 2.90347 2.73739 3.18146 2.89801 2.37887 2.77519 2.98518 4.58477 3.61503

0.01961 4.33726 5.05961 0.61958 0.77255 0.26436 1.45972

1117 2.37800 4.35784 3.99814 3.43090 3.51178 2.39000 4.15781 2.65564 3.34989 1.74461 3.50319 3.72387 4.20980 3.61230 3.61529 2.26737 3.02432 2.10192 5.00173 3.79570 1421 - -

2.68618 4.42225 2.77519 2.73123 3.46354 2.40513 3.72494 3.29354 2.67741 2.69355 4.24690 2.90347 2.73739 3.18146 2.89801 2.37887 2.77519 2.98518 4.58477 3.61503

0.01347 4.70993 5.43228 0.61958 0.77255 0.48576 0.95510

1118 2.60012 5.25421 2.21738 2.19093 4.59011 3.19071 3.52446 3.79694 2.20661 3.55556 3.80798 2.87054 3.93589 2.83003 2.68162 2.31024 2.46846 3.47248 5.69240 4.28704 1422 - -

2.68618 4.42225 2.77519 2.73123 3.46354 2.40513 3.72494 3.29354 2.67741 2.69355 4.24690 2.90347 2.73739 3.18146 2.89801 2.37887 2.77519 2.98518 4.58477 3.61503

0.01347 4.70993 5.43228 0.61958 0.77255 0.48576 0.95510

1119 2.70816 5.27801 2.87222 2.28509 4.61926 3.29211 3.55451 4.10138 1.51378 3.58039 4.32063 2.51873 3.94281 2.67254 2.80229 2.40551 3.00987 3.49179 5.71140 4.30420 1423 - -

2.68618 4.42225 2.77519 2.73123 3.46354 2.40513 3.72494 3.29354 2.67741 2.69355 4.24690 2.90347 2.73739 3.18146 2.89801 2.37887 2.77519 2.98518 4.58477 3.61503

0.01347 4.70993 5.43228 0.61958 0.77255 0.48576 0.95510

1120 2.77353 5.00560 3.01224 2.59646 4.25259 3.59640 3.40137 3.67594 2.42474 3.26753 3.78015 3.11887 3.98620 2.26931 2.71530 2.61315 2.88061 3.34350 4.47855 1.90378 1424 - -

2.68619 4.42226 2.77520 2.73124 3.46355 2.40514 3.72495 3.29355 2.67742 2.69356 4.24691 2.90348 2.73732 3.18134 2.89802 2.37888 2.77520 2.98519 4.58478 3.61504

0.03199 3.60777 5.43228 0.63961 0.74972 0.48576 0.95510

1121 2.93535 5.38721 2.83616 2.20227 4.71441 3.60535 3.84695 4.18006 2.14111 3.67468 4.44855 3.07811 1.41750 2.79029 2.93337 2.76766 3.17033 3.77154 5.81786 4.42520 1427 - -

2.68618 4.42225 2.77519 2.73123 3.46354 2.40513 3.72494 3.29354 2.67741 2.69355 4.24690 2.90347 2.73739 3.18146 2.89801 2.37887 2.77519 2.98518 4.58477 3.61503

0.01347 4.70993 5.43228 0.61958 0.77255 0.48576 0.95510

1122 2.55775 4.42418 3.80613 2.40328 2.48820 3.79932 4.04963 2.77883 3.17479 2.62737 3.53193 3.59659 4.17560 3.29806 3.48778 2.70054 3.01812 2.69190 3.85622 1.86880 1428 - -

2.68618 4.42225 2.77519 2.73123 3.46354 2.40513 3.72494 3.29354 2.67741 2.69355 4.24690 2.90347 2.73739 3.18146 2.89801 2.37887 2.77519 2.98518 4.58477 3.61503

0.01347 4.70993 5.43228 0.61958 0.77255 0.48576 0.95510

1123 4.48079 5.59676 5.42838 5.25035 2.20262 5.00902 4.10679 4.06390 4.99888 3.24726 4.61801 4.81999 5.36219 4.91923 4.92853 4.55204 4.72593 4.05987 0.47606 2.47918 1429 - -

2.68618 4.42225 2.77519 2.73123 3.46354 2.40513 3.72494 3.29354 2.67741 2.69355 4.24690 2.90347 2.73739 3.18146 2.89801 2.37887 2.77519 2.98518 4.58477 3.61503

0.01347 4.70993 5.43228 0.61958 0.77255 0.48576 0.95510

1124 2.88223 5.35528 2.79720 2.51840 4.70070 3.24750 3.80529 4.17267 2.02073 3.65370 4.41296 1.45688 4.01271 2.91375 2.44244 2.72036 3.11495 3.74988 5.78417 4.38962 1430 - -

2.68618 4.42225 2.77519 2.73123 3.46354 2.40513 3.72494 3.29354 2.67741 2.69355 4.24690 2.90347 2.73739 3.18146 2.89801 2.37887 2.77519 2.98518 4.58477 3.61503

0.01347 4.70993 5.43228 0.61958 0.77255 0.48576 0.95510

1125 3.07667 5.32724 3.25519 2.82973 4.68814 3.80577 3.86537 3.54130 2.06860 3.57994 4.39721 3.30846 4.17981 2.90668 1.01986 3.07171 2.97915 3.73233 5.70263 4.43356 1431 - -

2.68618 4.42225 2.77519 2.73123 3.46354 2.40513 3.72494 3.29354 2.67741 2.69355 4.24690 2.90347 2.73739 3.18146 2.89801 2.37887 2.77519 2.98518 4.58477 3.61503

0.01347 4.70993 5.43228 0.61958 0.77255 0.48576 0.95510

1126 2.76877 4.75984 3.34359 2.78921 3.75085 3.64349 3.89818 3.36471 2.76524 3.02537 2.81910 2.80170 4.04640 3.10597 3.03271 1.57223 2.21237 3.08680 5.32910 4.04171 1432 - -
2.68618 4.42225 2.77519 2.73123 3.46354 2.40513 3.72494 3.29354 2.67741 2.69355 4.24690 2.90347 2.73739 3.18146 2.89801 2.37887 2.77519 2.98518 4.58477 3.61503
0.01347 4.70993 5.43228 0.61958 0.77255 0.48576 0.95510
1127 2.43859 4.49779 2.67167 2.35984 4.40539 2.82699 3.55068 2.55517 2.52449 3.21352 4.17688 2.61788 3.95798 2.72239 2.80366 2.32484 2.99716 3.47814 5.59014 3.73500 1433 - -
2.68618 4.42225 2.77519 2.73123 3.46354 2.40513 3.72494 3.29354 2.67741 2.69355 4.24690 2.90347 2.73739 3.18146 2.89801 2.37887 2.77519 2.98518 4.58477 3.61503
0.01347 4.70993 5.43228 0.61958 0.77255 0.48576 0.95510
1128 3.99227 5.60978 4.81709 4.83944 5.82962 0.12816 5.82258 5.70860 5.11939 5.21140 6.24447 4.98512 5.00978 5.37976 5.19299 4.19709 4.52751 5.08067 6.63378 5.98272 1434 - -
2.68618 4.42225 2.77519 2.73123 3.46354 2.40513 3.72494 3.29354 2.67741 2.69355 4.24690 2.90347 2.73739 3.18146 2.89801 2.37887 2.77519 2.98518 4.58477 3.61503
0.01347 4.70993 5.43228 0.61958 0.77255 0.48576 0.95510
1129 1.81662 5.06793 3.13447 2.57427 4.03670 3.42274 3.52850 3.76776 2.12549 3.33796 4.13215 3.10116 3.98147 2.91447 2.08095 2.43119 3.01189 3.41761 4.59910 4.19617 1435 - -
2.68618 4.42225 2.77519 2.73123 3.46354 2.40513 3.72494 3.29354 2.67741 2.69355 4.24690 2.90347 2.73739 3.18146 2.89801 2.37887 2.77519 2.98518 4.58477 3.61503
0.01347 4.70993 5.43228 0.61958 0.77255 0.48576 0.95510
1130 3.63821 6.15031 0.42779 2.63733 5.51774 3.70385 4.45378 5.23686 3.67766 4.71609 5.67027 2.98771 4.42112 3.66810 4.36799 3.49704 3.99703 4.74695 6.72007 5.18059 1436 - -
2.68618 4.42225 2.77519 2.73123 3.46354 2.40513 3.72494 3.29354 2.67741 2.69355 4.24690 2.90347 2.73739 3.18146 2.89801 2.37887 2.77519 2.98518 4.58477 3.61503
0.01347 4.70993 5.43228 0.61958 0.77255 0.48576 0.95510
1131 4.36379 5.88137 4.39485 4.29673 4.28663 4.46827 0.20475 5.22986 4.14837 4.56902 5.73350 4.64516 5.08685 4.69582 4.32440 4.45346 4.72447 4.94360 5.70020 4.22926 1437 - -
2.68618 4.42225 2.77519 2.73123 3.46354 2.40513 3.72494 3.29354 2.67741 2.69355 4.24690 2.90347 2.73739 3.18146 2.89801 2.37887 2.77519 2.98518 4.58477 3.61503
0.01347 4.70993 5.43228 0.61958 0.77255 0.48576 0.95510
1132 3.47219 4.75789 5.35088 4.80203 1.10193 4.77925 5.04235 1.93162 4.65592 2.03862 3.42812 4.88600 5.02618 4.73975 4.70388 4.13386 3.70607 1.66746 5.33602 4.06334 1438 - -
2.68618 4.42225 2.77519 2.73123 3.46354 2.40513 3.72494 3.29354 2.67741 2.69355 4.24690 2.90347 2.73739 3.18146 2.89801 2.37887 2.77519 2.98518 4.58477 3.61503
0.01347 4.70993 5.43228 0.61958 0.77255 0.48576 0.95510
1133 3.04376 4.39916 4.97190 4.36352 1.98021 4.23028 4.53357 1.63375 4.16326 1.53798 2.62154 4.37961 4.54936 4.25510 4.16863 3.54824 3.27101 2.46784 3.56150 3.64480 1439 - -
2.68618 4.42225 2.77519 2.73123 3.46354 2.40513 3.72494 3.29354 2.67741 2.69355 4.24690 2.90347 2.73739 3.18146 2.89801 2.37887 2.77519 2.98518 4.58477 3.61503
0.01347 4.70993 5.43228 0.61958 0.77255 0.48576 0.95510
1134 2.62182 4.25251 4.52734 3.93363 2.92162 3.98455 4.31380 2.56039 3.77885 1.70899 3.18118 4.05234 3.41283 3.96204 3.88770 3.28674 2.71412 1.39106 4.89489 3.71320 1440 - -
2.68618 4.42225 2.77519 2.73123 3.46354 2.40513 3.72494 3.29354 2.67741 2.69355 4.24690 2.90347 2.73739 3.18146 2.89801 2.37887 2.77519 2.98518 4.58477 3.61503
0.01347 4.70993 5.43228 0.61958 0.77255 0.48576 0.95510
1135 1.82975 4.29979 4.09695 3.51660 2.86294 3.85536 4.15223 2.54934 3.41914 2.25627 2.94462 3.78018 4.22850 3.66218 3.64710 1.99647 2.60885 2.44107 4.91131 3.71234 1441 - -
2.68618 4.42225 2.77519 2.73123 3.46354 2.40513 3.72494 3.29354 2.67741 2.69355 4.24690 2.90347 2.73739 3.18146 2.89801 2.37887 2.77519 2.98518 4.58477 3.61503
0.01347 4.70993 5.43228 0.61958 0.77255 0.48576 0.95510
1136 2.58383 1.72434 3.62098 3.09538 4.08236 2.08925 3.06421 3.47012 3.06920 3.14889 4.00502 3.47342 3.34703 3.38770 3.45767 2.46677 2.66041 3.15131 5.46222 4.20846 1442 - -
2.68618 4.42225 2.77519 2.73123 3.46354 2.40513 3.72494 3.29354 2.67741 2.69355 4.24690 2.90347 2.73739 3.18146 2.89801 2.37887 2.77519 2.98518 4.58477 3.61503
0.01347 4.70993 5.43228 0.61958 0.77255 0.48576 0.95510
1137 2.85179 5.18473 2.83283 2.60097 4.46967 3.62919 1.60994 3.92183 2.32710 3.45653 4.24437 3.12490 4.01972 2.91671 2.18024 2.74188 3.07641 3.55423 3.90067 4.26687 1443 - -
2.68667 4.42294 2.77418 2.73115 3.46231 2.40568 3.72546 3.29325 2.67786 2.69404 4.24686 2.90388 2.73763 3.18182 2.89684 2.37883 2.77482 2.98549 4.58077 3.61549

0.21654 1.65901 5.43228 2.30733 0.10483 0.48576 0.95510

1138 2.83443 5.31130 1.08047 2.58289 4.75539 3.30108 4.00316 3.71819 2.86997 3.76373 4.56052 3.12475 2.46803 3.14624 3.38181 2.76646 3.24983 3.79963 5.94813 4.54427 1458 - -

2.68618 4.42225 2.77519 2.73123 3.46354 2.40513 3.72494 3.29354 2.67741 2.69355 4.24690 2.90347 2.73739 3.18146 2.89801 2.37887 2.77519 2.98518 4.58477 3.61503

0.01347 4.70993 5.43228 0.61958 0.77255 0.48576 0.95510

1139 2.71122 4.49094 3.74577 2.83573 2.90108 3.80253 4.03756 2.97946 3.13628 2.69278 3.60091 2.81647 4.18683 3.43686 3.46087 3.06470 3.05334 2.57542 1.50412 3.68551 1459 - -

2.68618 4.42225 2.77519 2.73123 3.46354 2.40513 3.72494 3.29354 2.67741 2.69355 4.24690 2.90347 2.73739 3.18146 2.89801 2.37887 2.77519 2.98518 4.58477 3.61503

0.01347 4.70993 5.43228 0.61958 0.77255 0.48576 0.95510

1140 1.58592 4.56031 4.22467 4.04924 5.12407 0.78744 5.04299 4.59243 4.16540 4.27112 5.04652 3.94988 4.15790 4.36166 4.39268 2.21455 3.17432 3.82900 6.45321 5.31903 1460 - -

2.68618 4.42225 2.77519 2.73123 3.46354 2.40513 3.72494 3.29354 2.67741 2.69355 4.24690 2.90347 2.73739 3.18146 2.89801 2.37887 2.77519 2.98518 4.58477 3.61503

0.20214 4.70993 1.74863 0.61958 0.77255 0.48576 0.95510

1141 2.20086 4.43523 3.56375 2.99803 3.16906 3.67405 3.69163 2.93858 2.95225 2.45781 3.03568 3.22649 1.96982 3.25766 3.30978 2.82109 2.93530 2.63415 5.01789 3.78044 1461 - -

2.68618 4.42225 2.77519 2.73123 3.46354 2.40513 3.72494 3.29354 2.67741 2.69355 4.24690 2.90347 2.73739 3.18146 2.89801 2.37887 2.77519 2.98518 4.58477 3.61503

0.01624 4.52403 5.24638 0.61958 0.77255 0.33318 1.26104

1142 2.76571 3.30282 2.81954 1.98107 4.47178 3.55710 3.53495 3.92897 2.31623 3.15271 4.22099 2.62328 3.94947 2.86111 2.75513 2.21239 2.71619 3.53670 4.61361 3.48441 1462 - -

2.68618 4.42225 2.77519 2.73123 3.46354 2.40513 3.72494 3.29354 2.67741 2.69355 4.24690 2.90347 2.73739 3.18146 2.89801 2.37887 2.77519 2.98518 4.58477 3.61503

0.01347 4.70993 5.43228 0.61958 0.77255 0.48576 0.95510

1143 2.04878 5.04169 3.02483 2.28326 3.36510 3.58178 3.53274 3.73163 2.30152 3.04128 3.88053 2.88014 3.97259 2.91534 2.66447 2.49024 2.76520 3.06239 5.52900 3.36182 1463 - -

2.68618 4.42225 2.77519 2.73123 3.46354 2.40513 3.72494 3.29354 2.67741 2.69355 4.24690 2.90347 2.73739 3.18146 2.89801 2.37887 2.77519 2.98518 4.58477 3.61503

0.01347 4.70993 5.43228 0.61958 0.77255 0.48576 0.95510

1144 2.34236 3.86478 4.15903 3.57522 2.72902 3.86108 3.35605 2.57281 3.46722 1.97705 3.38509 3.81244 4.00124 3.69920 3.67235 2.14109 2.40475 2.23342 4.88021 3.68475 1464 - -

2.68597 4.42238 2.77519 2.73093 3.46367 2.40522 3.72507 3.29328 2.67744 2.69368 4.24616 2.90359 2.73732 3.18144 2.89806 2.37899 2.77526 2.98531 4.58490 3.61493

0.24305 2.32648 2.13599 1.29755 0.31910 0.48576 0.95510

1145 2.68607 4.63013 3.01738 2.64436 2.45847 3.54071 3.86516 3.02835 2.53971 2.42902 2.93712 3.12023 4.02033 3.04521 2.42161 2.86039 2.84473 2.92236 4.55022 3.91471 1473 - -

2.68618 4.42225 2.77519 2.73123 3.46354 2.40513 3.72494 3.29354 2.67741 2.69355 4.24690 2.90347 2.73739 3.18146 2.89801 2.37887 2.77519 2.98518 4.58477 3.61503

0.31074 4.58917 1.35893 0.61958 0.77255 0.70087 0.68548

1146 2.62388 4.97342 2.95152 2.35402 4.24926 2.66427 3.37888 3.68532 2.26413 3.24848 4.04028 2.95813 3.85365 2.67870 2.88899 1.88228 2.89826 3.25207 5.45221 3.81121 1474 - -

2.68618 4.42225 2.77519 2.73123 3.46354 2.40513 3.72494 3.29354 2.67741 2.69355 4.24690 2.90347 2.73739 3.18146 2.89801 2.37887 2.77519 2.98518 4.58477 3.61503

0.02039 4.29881 5.02116 0.61958 0.77255 0.53941 0.87489

1147 2.70798 4.66414 3.22774 2.45388 3.30399 3.60335 2.23409 2.78504 2.66088 2.71910 3.75318 2.97361 3.71585 2.95374 3.02943 2.74182 2.82899 2.78156 4.43700 3.41101 1475 - -

2.68618 4.42225 2.77519 2.73123 3.46354 2.40513 3.72494 3.29354 2.67741 2.69355 4.24690 2.90347 2.73739 3.18146 2.89801 2.37887 2.77519 2.98518 4.58477 3.61503

0.12526 4.54060 2.23435 0.61958 0.77255 0.44055 1.03194

1148 2.74395 4.57444 3.47087 2.90024 3.73218 3.67366 3.90031 2.95162 2.72427 2.09520 3.13885 3.34254 2.10296 3.16777 2.40914 2.79487 2.97412 2.85752 5.13643 3.88500 1476 - -

2.68618 4.42225 2.77519 2.73123 3.46354 2.40513 3.72494 3.29354 2.67741 2.69355 4.24690 2.90347 2.73739 3.18146 2.89801 2.37887 2.77519 2.98518 4.58477 3.61503

0.01546 4.57293 5.29528 0.61958 0.77255 0.72486 0.66241

1149 2.72444 5.20916 2.78047 2.21673 4.54396 3.49954 2.42020 3.78336 2.20075 3.36517 4.25287 2.80663 2.66054 2.54089 2.79907 2.62126 2.84853 3.59434 5.64736 4.24348 1477 - -

2.68618 4.42225 2.77519 2.73123 3.46354 2.40513 3.72494 3.29354 2.67741 2.69355 4.24690 2.90347 2.73739 3.18146 2.89801 2.37887 2.77519 2.98518 4.58477 3.61503
0.01546 4.57293 5.29528 0.61958 0.77255 0.59027 0.80784
1150 2.60696 4.75296 3.27956 1.97296 2.94692 3.40122 3.83956 3.33255 2.54780 1.89824 3.83871 3.21298 4.01011 2.94573 2.85016 2.84283 2.97820 3.05798 5.28898 3.99870 1478 - -
2.68618 4.42225 2.77519 2.73123 3.46354 2.40513 3.72494 3.29354 2.67741 2.69355 4.24690 2.90347 2.73739 3.18146 2.89801 2.37887 2.77519 2.98518 4.58477 3.61503
0.01475 4.61999 5.34234 0.61958 0.77255 0.39404 1.12186
1151 2.61947 4.19042 4.49513 3.89515 2.60530 2.43732 4.23470 2.20929 3.49044 2.09531 2.33289 3.99343 4.28082 3.90636 3.82373 2.96348 3.00610 2.07641 4.80835 3.33681 1479 - -
2.68618 4.42225 2.77519 2.73123 3.46354 2.40513 3.72494 3.29354 2.67741 2.69355 4.24690 2.90347 2.73739 3.18146 2.89801 2.37887 2.77519 2.98518 4.58477 3.61503
0.01347 4.70993 5.43228 0.61958 0.77255 0.48576 0.95510
1152 2.49936 5.13019 3.07898 2.33902 3.42887 3.42372 2.52909 3.64675 2.00765 3.27695 3.94626 2.82364 3.95687 2.67758 2.54472 2.68782 2.99780 3.49032 3.98836 4.22398 1480 - -
2.68618 4.42225 2.77519 2.73123 3.46354 2.40513 3.72494 3.29354 2.67741 2.69355 4.24690 2.90347 2.73739 3.18146 2.89801 2.37887 2.77519 2.98518 4.58477 3.61503
0.01347 4.70993 5.43228 0.61958 0.77255 0.48576 0.95510
1153 2.75963 5.23004 3.09999 2.55833 4.55082 3.60375 3.41625 4.00058 2.26769 3.30361 4.28813 1.38322 3.54548 2.89580 2.61223 2.74325 3.00999 3.61017 5.67164 4.30480 1481 - -
2.68618 4.42225 2.77519 2.73123 3.46354 2.40513 3.72494 3.29354 2.67741 2.69355 4.24690 2.90347 2.73739 3.18146 2.89801 2.37887 2.77519 2.98518 4.58477 3.61503
0.01347 4.70993 5.43228 0.61958 0.77255 0.48576 0.95510
1154 2.19258 3.36358 4.10964 3.52842 3.08739 3.85094 4.14975 2.56881 3.42809 2.25890 2.76369 3.78434 3.54050 3.66814 3.64982 1.71476 2.90544 2.54313 4.89899 3.70095 1482 - -
2.68618 4.42225 2.77519 2.73123 3.46354 2.40513 3.72494 3.29354 2.67741 2.69355 4.24690 2.90347 2.73739 3.18146 2.89801 2.37887 2.77519 2.98518 4.58477 3.61503
0.01347 4.70993 5.43228 0.61958 0.77255 0.48576 0.95510
1155 2.89514 4.32346 4.52111 3.94057 3.46029 4.04336 4.40496 1.30502 3.80191 2.45321 2.59806 4.09491 4.41423 4.00883 3.94543 2.83733 2.16880 2.13088 5.02404 3.83654 1483 - -
2.68618 4.42225 2.77519 2.73123 3.46354 2.40513 3.72494 3.29354 2.67741 2.69355 4.24690 2.90347 2.73739 3.18146 2.89801 2.37887 2.77519 2.98518 4.58477 3.61503
0.01347 4.70993 5.43228 0.61958 0.77255 0.48576 0.95510
1156 2.66556 4.34428 3.96651 3.38864 2.93550 3.82918 4.10834 2.55197 2.72856 2.21188 2.47459 3.69761 4.20211 3.56631 1.85951 3.09762 3.00955 2.54004 4.15665 3.73745 1484 - -
2.68618 4.42225 2.77519 2.73123 3.46354 2.40513 3.72494 3.29354 2.67741 2.69355 4.24690 2.90347 2.73739 3.18146 2.89801 2.37887 2.77519 2.98518 4.58477 3.61503
0.01347 4.70993 5.43228 0.61958 0.77255 0.48576 0.95510
1157 1.74666 4.30423 4.29451 3.72127 3.44551 3.48224 4.27501 2.50701 3.60230 1.46475 3.44148 3.91959 4.29547 3.82912 3.78826 3.04093 3.06156 2.09075 4.97095 3.77911 1485 - -
2.68618 4.42225 2.77519 2.73123 3.46354 2.40513 3.72494 3.29354 2.67741 2.69355 4.24690 2.90347 2.73739 3.18146 2.89801 2.37887 2.77519 2.98518 4.58477 3.61503
0.01347 4.70993 5.43228 0.61958 0.77255 0.48576 0.95510
1158 2.78423 4.32141 4.06704 3.49185 3.43934 3.61264 4.15322 2.76423 3.38593 1.33987 3.35085 3.76533 4.22727 3.08456 3.62245 2.80393 2.68738 1.97362 4.93790 3.73753 1486 - -
2.68618 4.42225 2.77519 2.73123 3.46354 2.40513 3.72494 3.29354 2.67741 2.69355 4.24690 2.90347 2.73739 3.18146 2.89801 2.37887 2.77519 2.98518 4.58477 3.61503
0.01347 4.70993 5.43228 0.61958 0.77255 0.48576 0.95510
1159 2.68142 1.88283 4.22702 3.64174 3.36506 3.86939 3.41565 2.11996 3.52300 2.37076 3.37530 3.85222 3.51312 3.74598 3.70768 2.84792 2.60761 2.21287 4.87246 3.68005 1487 - -
2.68618 4.42225 2.77519 2.73123 3.46354 2.40513 3.72494 3.29354 2.67741 2.69355 4.24690 2.90347 2.73739 3.18146 2.89801 2.37887 2.77519 2.98518 4.58477 3.61503
0.01347 4.70993 5.43228 0.61958 0.77255 0.48576 0.95510
1160 2.80380 5.22849 2.38042 1.85552 3.56339 3.56083 3.77404 3.99808 2.53941 3.51699 4.28219 1.79185 3.96927 2.88786 3.02971 2.70599 3.03749 3.59899 3.95384 4.28032 1488 - -
2.68618 4.42225 2.77519 2.73123 3.46354 2.40513 3.72494 3.29354 2.67741 2.69355 4.24690 2.90347 2.73739 3.18146 2.89801 2.37887 2.77519 2.98518 4.58477 3.61503
0.01347 4.70993 5.43228 0.61958 0.77255 0.48576 0.95510

1161 1.76431 4.71397 3.37763 2.81829 2.88632 2.81362 3.90606 3.29601 2.79608 2.96601 3.51029 3.29095 3.80561 3.12867 2.66372 2.24175 2.81992 3.02853 5.28231 4.00378 1489 - -
2.68618 4.42225 2.77519 2.73123 3.46354 2.40513 3.72494 3.29354 2.67741 2.69355 4.24690 2.90347 2.73739 3.18146 2.89801 2.37887 2.77519 2.98518 4.58477 3.61503
0.01347 4.70993 5.43228 0.61958 0.77255 0.48576 0.95510
1162 2.80432 5.28494 2.28826 2.47486 4.61867 2.82547 3.76572 4.09711 2.52014 3.58860 4.33795 1.77471 3.96127 2.59991 2.88503 2.35177 2.93952 3.34074 5.73115 4.32377 1490 - -
2.68618 4.42225 2.77519 2.73123 3.46354 2.40513 3.72494 3.29354 2.67741 2.69355 4.24690 2.90347 2.73739 3.18146 2.89801 2.37887 2.77519 2.98518 4.58477 3.61503
0.01347 4.70993 5.43228 0.61958 0.77255 0.48576 0.95510
1163 2.69204 4.75791 3.32651 2.76580 3.94503 3.42281 3.87494 3.20885 2.67102 2.79876 3.84555 3.25203 2.94967 3.08299 2.48727 2.45806 1.95810 2.53893 5.29998 3.52076 1491 - -
2.68618 4.42225 2.77519 2.73123 3.46354 2.40513 3.72494 3.29354 2.67741 2.69355 4.24690 2.90347 2.73739 3.18146 2.89801 2.37887 2.77519 2.98518 4.58477 3.61503
0.03400 4.70993 3.71241 0.61958 0.77255 0.48576 0.95510
1164 2.67170 4.63853 3.43110 2.86845 3.59751 3.48118 3.91474 3.17763 2.71737 2.71756 3.73302 2.86800 4.06611 3.16599 3.12870 1.90048 2.56638 2.92790 2.63083 3.11187 1492 - -
2.68618 4.42225 2.77519 2.73123 3.46354 2.40513 3.72494 3.29354 2.67741 2.69355 4.24690 2.90347 2.73739 3.18146 2.89801 2.37887 2.77519 2.98518 4.58477 3.61503
0.01375 4.68968 5.41203 0.61958 0.77255 0.46070 0.99652
1165 2.60041 5.26143 2.72264 1.71713 4.11546 3.31780 3.73004 3.81815 2.36199 3.56387 4.30392 2.51251 3.49713 2.66242 2.81445 2.28034 2.99757 3.49223 5.69806 4.29098 1493 - -
2.68644 4.42275 2.77448 2.73145 3.46362 2.40528 3.72360 3.29354 2.67734 2.69396 4.24740 2.90327 2.73752 3.18169 2.89783 2.37880 2.77511 2.98568 4.58336 3.61481
0.45649 1.71035 1.68365 2.04223 0.13896 0.48576 0.95510
1166 2.64206 5.13027 2.65817 2.43270 4.44233 2.37516 3.02920 3.90388 2.43469 3.42191 4.18330 2.97420 3.30846 2.79601 2.16132 2.68926 2.64934 3.50388 5.58552 3.92020 1520 - -
2.68618 4.42124 2.77527 2.73130 3.46361 2.40520 3.72502 3.29338 2.67748 2.69362 4.24670 2.90354 2.73734 3.18107 2.89808 2.37894 2.77527 2.98497 4.58484 3.61510
0.35949 1.21529 5.23221 0.14131 2.02665 0.80969 0.58878
1167 2.34120 3.20746 4.35542 3.79926 1.40293 3.30660 4.25078 2.65611 3.66063 2.45603 3.39879 3.89773 4.19433 3.86061 3.80006 3.06979 2.97177 2.23273 4.89290 3.68736 1522 - -
2.68618 4.42225 2.77519 2.73123 3.46354 2.40513 3.72494 3.29354 2.67741 2.69355 4.24690 2.90347 2.73739 3.18146 2.89801 2.37887 2.77519 2.98518 4.58477 3.61503
0.01648 4.50987 5.23221 0.61958 0.77255 0.80969 0.58878
1168 2.70310 5.13093 2.71362 2.42896 4.44397 3.48928 3.67888 3.69468 1.85932 3.21446 4.02883 2.53417 3.88185 2.63694 2.60810 2.38841 2.88885 2.89910 5.58272 4.19355 1523 - -
2.68618 4.42225 2.77519 2.73123 3.46354 2.40513 3.72494 3.29354 2.67741 2.69355 4.24690 2.90347 2.73739 3.18146 2.89801 2.37887 2.77519 2.98518 4.58477 3.61503
0.01648 4.50987 5.23221 0.61958 0.77255 0.41384 1.08206
1169 2.60891 4.41775 3.57593 2.93590 3.02877 3.57485 3.50103 2.67507 3.01906 2.35010 3.52391 3.53832 2.05963 3.39522 3.32489 2.88788 2.98619 2.24195 4.26932 3.45432 1524 - -
2.68618 4.42225 2.77519 2.73123 3.46354 2.40513 3.72494 3.29354 2.67741 2.69355 4.24690 2.90347 2.73739 3.18146 2.89801 2.37887 2.77519 2.98518 4.58477 3.61503
0.16162 4.66942 1.96710 0.61958 0.77255 0.56621 0.83858
1170 2.70539 5.08050 2.83094 2.16036 4.37354 2.68536 3.51971 3.55451 2.45257 3.36310 3.99254 2.83384 3.89345 2.77027 2.04853 2.62951 2.88884 2.98255 4.61352 4.16878 1525 - -
2.68618 4.42225 2.77519 2.73123 3.46354 2.40513 3.72494 3.29354 2.67741 2.69355 4.24690 2.90347 2.73739 3.18146 2.89801 2.37887 2.77519 2.98518 4.58477 3.61503
0.01624 4.52403 5.24638 0.61958 0.77255 0.33318 1.26104
1171 2.78239 5.23385 3.05313 2.38676 4.10405 3.33630 3.45745 3.86009 1.43455 3.52776 4.28084 2.38235 3.95322 2.74243 2.74611 2.75521 2.90525 3.44572 5.67437 4.28295 1526 - -
2.68618 4.42225 2.77519 2.73123 3.46354 2.40513 3.72494 3.29354 2.67741 2.69355 4.24690 2.90347 2.73739 3.18146 2.89801 2.37887 2.77519 2.98518 4.58477 3.61503
0.01347 4.70993 5.43228 0.61958 0.77255 0.48576 0.95510
1172 3.36111 6.02693 0.76384 2.09903 5.30575 3.18753 3.44450 4.85171 3.19374 4.31037 5.15078 2.88899 4.23813 3.31797 3.81997 2.94231 3.64882 4.39451 6.45950 4.91600 1527 - -
2.68618 4.42225 2.77519 2.73123 3.46354 2.40513 3.72494 3.29354 2.67741 2.69355 4.24690 2.90347 2.73739 3.18146 2.89801 2.37887 2.77519 2.98518 4.58477 3.61503

0.01347 4.70993 5.43228 0.61958 0.77255 0.48576 0.95510
1173 2.73745 4.21684 4.65590 4.05308 2.47791 3.99297 4.32379 2.03940 3.87070 2.09449 3.31466 4.10802 4.35249 3.65686 3.93003 3.30060 2.77141 1.36980 4.85395 3.45967 1528 - -
2.68618 4.42225 2.77519 2.73123 3.46354 2.40513 3.72494 3.29354 2.67741 2.69355 4.24690 2.90347 2.73739 3.18146 2.89801 2.37887 2.77519 2.98518 4.58477 3.61503
0.01347 4.70993 5.43228 0.61958 0.77255 0.48576 0.95510
1174 1.94417 4.48278 3.76407 3.21182 3.73102 3.00664 4.11560 2.43354 3.16328 2.80603 3.69727 3.56683 3.83265 3.45775 3.50267 1.75952 2.79815 2.22862 5.17755 3.95050 1529 - -
2.68618 4.42225 2.77519 2.73123 3.46354 2.40513 3.72494 3.29354 2.67741 2.69355 4.24690 2.90347 2.73739 3.18146 2.89801 2.37887 2.77519 2.98518 4.58477 3.61503
0.01347 4.70993 5.43228 0.61958 0.77255 0.48576 0.95510
1175 2.75246 4.47928 5.03910 4.46974 3.40484 4.43686 4.84354 1.09266 4.31908 1.78076 3.54134 4.55655 4.75901 4.49160 4.40289 3.51904 3.39287 1.70596 5.33233 4.15228 1530 - -
2.68618 4.42225 2.77519 2.73123 3.46354 2.40513 3.72494 3.29354 2.67741 2.69355 4.24690 2.90347 2.73739 3.18146 2.89801 2.37887 2.77519 2.98518 4.58477 3.61503
0.01347 4.70993 5.43228 0.61958 0.77255 0.48576 0.95510
1176 4.19546 5.72905 4.88467 4.87584 5.69810 4.37493 5.80577 5.56089 5.02238 5.03903 6.16559 5.07870 0.12805 5.37280 5.10382 4.40233 4.70166 5.09038 6.56663 5.84907 1531 - -
2.68618 4.42225 2.77519 2.73123 3.46354 2.40513 3.72494 3.29354 2.67741 2.69355 4.24690 2.90347 2.73739 3.18146 2.89801 2.37887 2.77519 2.98518 4.58477 3.61503
0.01347 4.70993 5.43228 0.61958 0.77255 0.48576 0.95510
1177 2.56271 5.08623 3.08808 1.66699 4.35103 3.38956 3.34007 3.78886 2.56830 3.35804 4.15109 3.08948 3.32776 2.83884 3.04153 2.79395 3.01977 3.27155 5.56685 2.19810 1532 - -
2.68618 4.42225 2.77519 2.73123 3.46354 2.40513 3.72494 3.29354 2.67741 2.69355 4.24690 2.90347 2.73739 3.18146 2.89801 2.37887 2.77519 2.98518 4.58477 3.61503
0.01347 4.70993 5.43228 0.61958 0.77255 0.48576 0.95510
1178 2.77186 4.29656 4.07602 3.49533 3.28914 3.84624 4.13701 1.88302 3.11180 2.49462 3.40939 3.51408 2.41819 3.64371 3.62986 2.92610 2.31691 2.28655 3.70266 3.22691 1533 - -
2.68618 4.42225 2.77519 2.73123 3.46354 2.40513 3.72494 3.29354 2.67741 2.69355 4.24690 2.90347 2.73739 3.18146 2.89801 2.37887 2.77519 2.98518 4.58477 3.61503
0.01347 4.70993 5.43228 0.61958 0.77255 0.48576 0.95510
1179 2.65791 4.95224 3.17711 2.61905 3.79847 3.60183 2.88673 3.29922 2.33933 3.10145 4.02426 2.17346 3.64888 2.96030 3.07297 2.65560 2.14423 3.13829 5.45791 3.38928 1534 - -
2.68618 4.42225 2.77519 2.73123 3.46354 2.40513 3.72494 3.29354 2.67741 2.69355 4.24690 2.90347 2.73739 3.18146 2.89801 2.37887 2.77519 2.98518 4.58477 3.61503
0.01347 4.70993 5.43228 0.61958 0.77255 0.48576 0.95510
1180 2.77164 3.95034 3.74618 3.49176 3.40687 3.56416 4.13597 2.38273 3.39602 1.74359 3.41023 3.44772 3.55978 3.46066 3.32154 2.86587 3.00362 2.02285 4.90334 2.53902 1535 - -
2.68618 4.42225 2.77519 2.73123 3.46354 2.40513 3.72494 3.29354 2.67741 2.69355 4.24690 2.90347 2.73739 3.18146 2.89801 2.37887 2.77519 2.98518 4.58477 3.61503
0.01347 4.70993 5.43228 0.61958 0.77255 0.48576 0.95510
1181 2.77238 4.47699 3.45320 3.10791 2.31652 3.75823 4.01330 2.79875 3.05682 2.44379 3.58058 3.50603 2.65590 3.35745 2.08715 2.89507 3.00414 2.38260 5.05949 3.70905 1536 - -
2.68618 4.42225 2.77519 2.73123 3.46354 2.40513 3.72494 3.29354 2.67741 2.69355 4.24690 2.90347 2.73739 3.18146 2.89801 2.37887 2.77519 2.98518 4.58477 3.61503
0.01347 4.70993 5.43228 0.61958 0.77255 0.48576 0.95510
1182 2.58808 5.24671 2.50745 2.32170 4.22112 3.39244 2.41447 4.05585 2.29280 3.54645 4.29092 2.83929 3.48190 2.72198 2.96472 2.58492 2.20716 3.43222 5.68718 4.28394 1537 - -
2.68618 4.42225 2.77519 2.73123 3.46354 2.40513 3.72494 3.29354 2.67741 2.69355 4.24690 2.90347 2.73739 3.18146 2.89801 2.37887 2.77519 2.98518 4.58477 3.61503
0.01347 4.70993 5.43228 0.61958 0.77255 0.48576 0.95510
1183 2.41655 4.89169 3.22395 2.66604 4.11922 2.29515 3.83193 3.29175 2.55250 2.94479 3.70304 3.17369 2.17026 3.00011 2.90235 2.44088 2.91513 3.21966 5.41733 4.10024 1538 - -
2.68618 4.42225 2.77519 2.73123 3.46354 2.40513 3.72494 3.29354 2.67741 2.69355 4.24690 2.90347 2.73739 3.18146 2.89801 2.37887 2.77519 2.98518 4.58477 3.61503
0.42021 4.70993 1.09636 0.61958 0.77255 0.48576 0.95510
1184 2.48615 3.78140 3.56245 2.99343 3.29469 3.62412 3.54663 2.72242 2.61109 2.30721 3.17803 3.38381 4.00240 3.23738 2.83975 2.71045 2.65239 2.51202 4.90554 2.70084 1539 - -

2.68618 4.42225 2.77519 2.73123 3.46354 2.40513 3.72494 3.29354 2.67741 2.69355 4.24690 2.90347 2.73739 3.18146 2.89801 2.37887 2.77519 2.98518 4.58477 3.61503
0.02016 4.30988 5.03223 0.61958 0.77255 0.46026 0.99729
1185 2.74163 5.21848 2.64689 2.30773 4.54827 3.50696 3.44055 4.02446 2.20476 3.51690 4.26454 2.81544 3.90692 2.58563 2.93960 1.71511 2.56692 3.42196 5.65948 4.25613 1540 - -
2.68618 4.42225 2.77519 2.73123 3.46354 2.40513 3.72494 3.29354 2.67741 2.69355 4.24690 2.90347 2.73739 3.18146 2.89801 2.37887 2.77519 2.98518 4.58477 3.61503
0.01522 4.58879 5.31114 0.61958 0.77255 0.70144 0.68492
1186 2.66791 4.80181 2.66403 2.58564 4.00781 3.30700 3.79905 3.27059 2.50467 1.95406 3.30492 2.75010 3.97376 2.98016 2.98428 2.66869 2.73076 2.95099 5.32798 3.63603 1541 - -
2.68618 4.42225 2.77519 2.73123 3.46354 2.40513 3.72494 3.29354 2.67741 2.69355 4.24690 2.90347 2.73739 3.18146 2.89801 2.37887 2.77519 2.98518 4.58477 3.61503
0.15340 4.58879 2.02457 0.61958 0.77255 0.40295 1.10366
1187 2.55325 5.21154 2.74291 2.23174 4.54576 2.96148 3.69085 4.02432 2.24702 3.51216 4.25572 2.80767 3.44412 2.70730 2.58498 2.48906 2.06007 3.59648 5.65068 4.24589 1542 - -
2.68618 4.42225 2.77519 2.73123 3.46354 2.40513 3.72494 3.29354 2.67741 2.69355 4.24690 2.90347 2.73739 3.18146 2.89801 2.37887 2.77519 2.98518 4.58477 3.61503
0.04279 4.57608 3.45475 0.61958 0.77255 0.59251 0.80505
1188 2.64773 5.22525 2.52990 2.01471 3.94242 2.37790 3.59768 4.04417 2.20409 3.38168 4.26809 2.68803 3.47141 2.70199 2.72767 2.69595 2.96231 3.61200 5.66202 4.25492 1543 - -
2.68618 4.42225 2.77519 2.73123 3.46354 2.40513 3.72494 3.29354 2.67741 2.69355 4.24690 2.90347 2.73739 3.18146 2.89801 2.37887 2.77519 2.98518 4.58477 3.61503
0.01513 4.59475 5.31710 0.61958 0.77255 0.54409 0.86838
1189 2.66448 4.44214 3.48531 3.09438 3.57531 3.73501 3.62849 2.38071 2.98768 1.91364 3.39504 3.04672 2.58604 3.34183 3.38792 2.79385 2.70008 2.39812 5.02738 3.79750 1544 - -
2.68624 4.42248 2.77525 2.73112 3.46329 2.40530 3.72509 3.29342 2.67751 2.69325 4.24712 2.90332 2.73762 3.18143 2.89764 2.37897 2.77529 2.98515 4.58459 3.61508
0.58358 0.98294 2.68980 0.75366 0.63609 0.53982 0.87432
1190 2.64859 4.17801 3.96990 3.71290 3.27790 3.84994 4.16041 2.38924 3.29248 2.08098 2.99990 3.87452 4.21793 3.55187 3.71708 3.07473 2.81242 1.80241 2.43693 3.08945 1548 - -
2.68618 4.42225 2.77519 2.73123 3.46354 2.40513 3.72494 3.29354 2.67741 2.69355 4.24690 2.90347 2.73739 3.18146 2.89801 2.37887 2.77519 2.98518 4.58477 3.61503
0.01499 4.60392 5.32627 0.61958 0.77255 0.61172 0.78180
1191 2.74110 4.07054 3.31355 2.67411 3.72179 1.87233 3.85152 3.11112 2.61353 2.87533 3.80218 3.23478 4.01625 2.15341 3.15008 2.80608 2.97253 3.01450 3.84017 3.97242 1549 - -
2.68626 4.42233 2.77501 2.73118 3.46362 2.40521 3.72502 3.29349 2.67736 2.69353 4.24623 2.90327 2.73747 3.18154 2.89791 2.37895 2.77527 2.98526 4.58485 3.61511
0.34750 1.24204 5.34831 0.13151 2.09373 0.64269 0.74628
1192 2.66841 5.10325 3.05354 2.30179 3.98687 1.97858 3.73162 3.39223 2.16396 3.24539 4.15956 3.03298 3.66971 2.72185 2.55568 2.62358 2.92437 3.46379 5.57014 4.19761 1551 - -
2.68618 4.42225 2.77519 2.73123 3.46354 2.40513 3.72494 3.29354 2.67741 2.69355 4.24690 2.90347 2.73739 3.18146 2.89801 2.37887 2.77519 2.98518 4.58477 3.61503
0.01466 4.62597 5.34831 0.61958 0.77255 0.39882 1.11203
1193 2.69921 4.91268 3.09889 2.54335 4.13583 3.61143 3.81907 2.98158 2.32635 2.82518 3.10663 2.88138 2.34635 2.98212 2.80183 2.42006 2.99856 2.78911 5.42608 4.10371 1552 - -
2.68618 4.42225 2.77519 2.73123 3.46354 2.40513 3.72494 3.29354 2.67741 2.69355 4.24690 2.90347 2.73739 3.18146 2.89801 2.37887 2.77519 2.98518 4.58477 3.61503
0.04391 4.70993 3.38281 0.61958 0.77255 0.48576 0.95510
1194 2.75635 5.17881 2.86937 2.44308 4.48923 3.06322 3.73492 3.95106 2.38282 3.26796 3.91469 3.02648 2.80151 2.73982 2.29681 1.93070 2.80732 3.26707 5.63292 4.24447 1553 - -
2.68618 4.42225 2.77519 2.73123 3.46354 2.40513 3.72494 3.29354 2.67741 2.69355 4.24690 2.90347 2.73739 3.18146 2.89801 2.37887 2.77519 2.98518 4.58477 3.61503
0.01388 4.67990 5.40225 0.61958 0.77255 0.44969 1.01564
1195 2.52347 4.91904 3.11360 2.64042 3.36454 3.30783 3.81707 3.55328 2.58200 2.94461 3.77297 2.61108 2.26363 2.97849 2.58566 2.51467 2.90679 2.75346 5.43151 4.10756 1554 - -
2.68618 4.42225 2.77519 2.73123 3.46354 2.40513 3.72494 3.29354 2.67741 2.69355 4.24690 2.90347 2.73739 3.18146 2.89801 2.37887 2.77519 2.98518 4.58477 3.61503
0.01347 4.70993 5.43228 0.61958 0.77255 0.48576 0.95510

1196 2.76559 5.22101 2.85688 2.13183 4.54360 3.39764 3.64437 3.39350 2.26028 3.26183 3.99578 2.89102 3.62258 2.70463 2.70522 1.81324 2.84505 3.32224 5.66734 4.27045 1555 - -
2.68618 4.42225 2.77519 2.73123 3.46354 2.40513 3.72494 3.29354 2.67741 2.69355 4.24690 2.90347 2.73739 3.18146 2.89801 2.37887 2.77519 2.98518 4.58477 3.61503
0.01347 4.70993 5.43228 0.61958 0.77255 0.48576 0.95510
1197 2.70018 5.26449 2.89526 2.22785 4.60464 3.38327 3.72880 4.08697 2.08827 3.39622 3.87584 2.63958 3.33223 2.38289 2.58910 2.04392 2.99712 3.48178 5.70000 4.29229 1556 - -
2.68618 4.42225 2.77519 2.73123 3.46354 2.40513 3.72494 3.29354 2.67741 2.69355 4.24690 2.90347 2.73739 3.18146 2.89801 2.37887 2.77519 2.98518 4.58477 3.61503
0.01347 4.70993 5.43228 0.61958 0.77255 0.48576 0.95510
1198 4.24089 5.86697 4.83025 4.24934 5.45989 4.46113 4.80851 5.14010 3.06292 4.49832 5.53053 4.51787 4.97412 4.03920 0.23083 4.32408 4.48276 4.83671 6.22816 5.28120 1557 - -
2.68618 4.42225 2.77519 2.73123 3.46354 2.40513 3.72494 3.29354 2.67741 2.69355 4.24690 2.90347 2.73739 3.18146 2.89801 2.37887 2.77519 2.98518 4.58477 3.61503
0.01347 4.70993 5.43228 0.61958 0.77255 0.48576 0.95510
1199 2.77761 5.26328 2.84648 2.35281 4.59991 3.54926 3.49999 4.07794 2.06802 3.56338 4.30715 2.93786 2.19458 2.74167 2.48392 2.57237 2.36753 3.64959 5.69937 4.29634 1558 - -
2.68618 4.42225 2.77519 2.73123 3.46354 2.40513 3.72494 3.29354 2.67741 2.69355 4.24690 2.90347 2.73739 3.18146 2.89801 2.37887 2.77519 2.98518 4.58477 3.61503
0.01347 4.70993 5.43228 0.61958 0.77255 0.48576 0.95510
1200 2.77469 4.25154 4.23724 3.65049 3.35162 3.87640 4.17857 1.55493 3.52907 2.41549 3.36684 3.29516 4.24639 3.75020 3.70937 2.72551 2.19567 2.38067 4.01512 2.79983 1559 - -
2.68618 4.42225 2.77519 2.73123 3.46354 2.40513 3.72494 3.29354 2.67741 2.69355 4.24690 2.90347 2.73739 3.18146 2.89801 2.37887 2.77519 2.98518 4.58477 3.61503
0.01347 4.70993 5.43228 0.61958 0.77255 0.48576 0.95510
1201 3.88492 5.14224 5.52500 5.00981 2.79761 5.05006 4.84420 2.81414 4.81711 0.47343 3.29083 5.04539 5.20219 4.77646 4.80673 4.41936 4.09254 3.04276 3.68941 3.51989 1560 - -
2.68618 4.42225 2.77519 2.73123 3.46354 2.40513 3.72494 3.29354 2.67741 2.69355 4.24690 2.90347 2.73739 3.18146 2.89801 2.37887 2.77519 2.98518 4.58477 3.61503
0.01347 4.70993 5.43228 0.61958 0.77255 0.48576 0.95510
1202 1.54427 4.25244 4.45132 3.85901 2.20574 3.58513 4.27519 2.50072 3.71325 1.98322 3.13798 4.00096 4.31877 3.90444 3.83989 3.25244 3.05405 2.21906 4.87783 3.69350 1561 - -
2.68618 4.42225 2.77519 2.73123 3.46354 2.40513 3.72494 3.29354 2.67741 2.69355 4.24690 2.90347 2.73739 3.18146 2.89801 2.37887 2.77519 2.98518 4.58477 3.61503
0.01347 4.70993 5.43228 0.61958 0.77255 0.48576 0.95510
1203 2.83356 3.86720 4.25280 3.67813 1.52036 3.91385 4.15281 2.77778 3.56075 2.41767 3.44145 3.58104 4.28954 3.77996 3.74779 2.30187 2.94436 2.58245 4.78066 2.40694 1562 - -
2.68618 4.42225 2.77519 2.73123 3.46354 2.40513 3.72494 3.29354 2.67741 2.69355 4.24690 2.90347 2.73739 3.18146 2.89801 2.37887 2.77519 2.98518 4.58477 3.61503
0.01347 4.70993 5.43228 0.61958 0.77255 0.48576 0.95510
1204 4.68268 5.64575 5.57595 5.47880 0.40589 5.21476 3.89631 4.24366 5.24894 3.40589 4.76570 4.77063 5.46855 4.92935 5.06471 4.61700 4.87726 4.20585 3.52997 1.98154 1563 - -
2.68618 4.42225 2.77519 2.73123 3.46354 2.40513 3.72494 3.29354 2.67741 2.69355 4.24690 2.90347 2.73739 3.18146 2.89801 2.37887 2.77519 2.98518 4.58477 3.61503
0.01347 4.70993 5.43228 0.61958 0.77255 0.48576 0.95510
1205 1.46665 4.89011 3.24782 2.68627 4.10912 3.63251 3.83708 3.50948 2.55239 3.00876 3.59098 2.86693 4.02080 2.74835 2.57890 2.84498 3.01415 2.77091 5.40768 4.09742 1564 - -
2.68618 4.42225 2.77519 2.73123 3.46354 2.40513 3.72494 3.29354 2.67741 2.69355 4.24690 2.90347 2.73739 3.18146 2.89801 2.37887 2.77519 2.98518 4.58477 3.61503
0.01347 4.70993 5.43228 0.61958 0.77255 0.48576 0.95510
1206 3.99227 5.60978 4.81709 4.83944 5.82962 0.12816 5.82258 5.70860 5.11939 5.21140 6.24447 4.98512 5.00978 5.37976 5.19299 4.19709 4.52751 5.08067 6.63378 5.98272 1565 - -
2.68618 4.42225 2.77519 2.73123 3.46354 2.40513 3.72494 3.29354 2.67741 2.69355 4.24690 2.90347 2.73739 3.18146 2.89801 2.37887 2.77519 2.98518 4.58477 3.61503
0.01347 4.70993 5.43228 0.61958 0.77255 0.48576 0.95510
1207 2.06220 5.17917 3.06733 2.51768 4.49000 2.11540 3.58077 3.94554 2.27148 3.26124 4.23809 2.51624 3.96192 2.87350 2.62307 2.51598 3.01368 3.55324 5.64034 4.26027 1566 - -
2.68610 4.42232 2.77527 2.73131 3.46361 2.40514 3.72502 3.29361 2.67748 2.69343 4.24697 2.90347 2.73689 3.18144 2.89808 2.37894 2.77527 2.98526 4.58484 3.61510

0.29658 1.37726 5.43228 0.13706 2.05507 0.48576 0.95510

1208 2.62385 4.49388 3.38154 3.07512 3.37147 3.05510 4.00096 2.72498 2.89350 2.44415 2.84757 3.48237 4.12756 3.33233 2.19014 2.72225 2.90897 2.05422 5.07542 3.84049 1568 - -

2.68618 4.42225 2.77519 2.73123 3.46354 2.40513 3.72494 3.29354 2.67741 2.69355 4.24690 2.90347 2.73739 3.18146 2.89801 2.37887 2.77519 2.98518 4.58477 3.61503

0.01347 4.70993 5.43228 0.61958 0.77255 0.48576 0.95510

1209 2.68810 5.08394 3.10927 2.44324 4.35380 3.58546 1.93070 3.78841 2.40667 3.35506 4.14668 2.99477 2.48704 2.81054 3.01480 2.79006 2.88793 3.30358 5.56214 3.09996 1569 - -

2.68639 4.42282 2.77479 2.73107 3.46410 2.40470 3.72511 3.29374 2.67710 2.69384 4.24719 2.90291 2.73725 3.18193 2.89738 2.37912 2.77576 2.98544 4.58533 3.61496

0.50598 0.93467 5.43228 1.49160 0.25491 0.48576 0.95510

1210 2.72100 5.16187 2.79717 2.38127 3.84372 1.59757 3.77497 3.90367 2.43412 3.44473 4.22033 2.83349 3.66673 2.89432 3.02788 2.49724 3.02154 3.52384 5.62962 3.83416 1579 - -

2.68618 4.42225 2.77519 2.73123 3.46354 2.40513 3.72494 3.29354 2.67741 2.69355 4.24690 2.90347 2.73739 3.18146 2.89801 2.37887 2.77519 2.98518 4.58477 3.61503

0.01347 4.70993 5.43228 0.61958 0.77255 0.48576 0.95510

1211 2.70270 5.07631 2.93417 2.48710 3.79138 3.57503 3.77222 3.78374 2.17886 3.18183 4.13744 2.85099 2.54860 2.90021 2.91455 2.30100 2.91476 3.28953 5.55620 2.49233 1580 - -

2.68618 4.42225 2.77519 2.73123 3.46354 2.40513 3.72494 3.29354 2.67741 2.69355 4.24690 2.90347 2.73739 3.18146 2.89801 2.37887 2.77519 2.98518 4.58477 3.61503

0.01347 4.70993 5.43228 0.61958 0.77255 0.48576 0.95510

1212 2.68338 4.35122 4.79586 4.21264 3.26214 4.20050 4.57052 1.49606 4.05496 2.11067 3.47229 4.30260 4.54910 4.23396 4.14531 2.75928 3.22183 1.36636 5.10938 3.92347 1581 - -

2.68618 4.42225 2.77519 2.73123 3.46354 2.40513 3.72494 3.29354 2.67741 2.69355 4.24690 2.90347 2.73739 3.18146 2.89801 2.37887 2.77519 2.98518 4.58477 3.61503

0.01347 4.70993 5.43228 0.61958 0.77255 0.48576 0.95510

1213 3.32763 5.37882 3.89101 3.17220 4.78720 3.59467 3.96640 4.12408 2.12926 3.62219 3.33509 3.56436 4.37022 3.11121 0.77897 3.35540 3.50356 3.82473 5.72640 4.55372 1582 - -

2.68618 4.42225 2.77519 2.73123 3.46354 2.40513 3.72494 3.29354 2.67741 2.69355 4.24690 2.90347 2.73739 3.18146 2.89801 2.37887 2.77519 2.98518 4.58477 3.61503

0.01347 4.70993 5.43228 0.61958 0.77255 0.48576 0.95510

1214 2.65867 5.23316 2.80365 2.37044 4.55946 2.47937 3.73842 4.03212 2.39873 3.39276 4.27950 3.02292 2.19698 2.54003 2.65575 2.56727 2.77781 3.46128 5.67747 4.27835 1583 - -

2.68618 4.42225 2.77519 2.73123 3.46354 2.40513 3.72494 3.29354 2.67741 2.69355 4.24690 2.90347 2.73739 3.18146 2.89801 2.37887 2.77519 2.98518 4.58477 3.61503

0.01347 4.70993 5.43228 0.61958 0.77255 0.48576 0.95510

1215 2.65593 4.57298 3.53458 2.35583 3.72354 3.72030 3.96094 2.16772 2.52695 2.41167 3.67188 3.40629 4.10190 3.02746 3.18562 2.82959 2.89235 1.97734 5.14388 3.89483 1584 - -

2.68618 4.42225 2.77519 2.73123 3.46354 2.40513 3.72494 3.29354 2.67741 2.69355 4.24690 2.90347 2.73739 3.18146 2.89801 2.37887 2.77519 2.98518 4.58477 3.61503

0.01347 4.70993 5.43228 0.61958 0.77255 0.48576 0.95510

1216 2.97278 4.46022 4.34916 3.74498 3.46476 4.06109 4.32788 2.13812 3.23825 1.07327 3.40956 3.99098 4.40958 3.81216 2.67758 3.35722 3.20052 2.48827 5.06123 3.88588 1585 - -

2.68618 4.42225 2.77519 2.73123 3.46354 2.40513 3.72494 3.29354 2.67741 2.69355 4.24690 2.90347 2.73739 3.18146 2.89801 2.37887 2.77519 2.98518 4.58477 3.61503

0.01347 4.70993 5.43228 0.61958 0.77255 0.48576 0.95510

1217 2.57379 4.19529 4.46493 3.64513 2.73663 3.54587 4.22755 2.08691 3.70706 1.51325 3.08606 3.97765 4.27603 3.26596 3.51464 3.20796 2.80143 2.37243 4.15341 3.27870 1586 - -

2.68618 4.42225 2.77519 2.73123 3.46354 2.40513 3.72494 3.29354 2.67741 2.69355 4.24690 2.90347 2.73739 3.18146 2.89801 2.37887 2.77519 2.98518 4.58477 3.61503

0.01347 4.70993 5.43228 0.61958 0.77255 0.48576 0.95510

1218 2.50414 5.26448 2.48920 2.40423 4.60423 3.54218 3.56118 3.79940 2.00679 3.36571 4.30669 2.62468 3.93581 1.96002 2.77625 2.58758 2.99842 3.65229 5.70003 4.01069 1587 - -

2.68618 4.42225 2.77519 2.73123 3.46354 2.40513 3.72494 3.29354 2.67741 2.69355 4.24690 2.90347 2.73739 3.18146 2.89801 2.37887 2.77519 2.98518 4.58477 3.61503

0.01347 4.70993 5.43228 0.61958 0.77255 0.48576 0.95510

1219 2.76976 4.97685 3.03490 2.37694 4.21609 3.59840 2.12881 3.32414 2.59829 2.41417 4.04704 2.99861 3.98865 2.24885 3.06132 2.80206 3.00097 2.94439 5.47705 3.76694 1588 - -

2.68618 4.42225 2.77519 2.73123 3.46354 2.40513 3.72494 3.29354 2.67741 2.69355 4.24690 2.90347 2.73739 3.18146 2.89801 2.37887 2.77519 2.98518 4.58477 3.61503

0.01347 4.70993 5.43228 0.61958 0.77255 0.48576 0.95510

1220 2.56090 2.29437 4.57201 3.99094 3.30636 3.96911 4.32182 2.68006 3.80399 1.83068 3.39197 4.07820 4.36406 3.99962 3.90640 3.28937 3.11519 2.51353 1.70162 3.63443 1589 - -

2.68618 4.42225 2.77519 2.73123 3.46354 2.40513 3.72494 3.29354 2.67741 2.69355 4.24690 2.90347 2.73739 3.18146 2.89801 2.37887 2.77519 2.98518 4.58477 3.61503

0.01347 4.70993 5.43228 0.61958 0.77255 0.48576 0.95510

1221 2.64411 5.22303 3.04166 1.68599 4.54622 3.18161 3.73983 3.75631 1.99090 3.13264 4.27007 3.02894 3.94478 2.76983 2.56108 2.49588 2.88086 3.39331 5.66827 4.27330 1590 - -

2.68618 4.42225 2.77519 2.73123 3.46354 2.40513 3.72494 3.29354 2.67741 2.69355 4.24690 2.90347 2.73739 3.18146 2.89801 2.37887 2.77519 2.98518 4.58477 3.61503

0.01347 4.70993 5.43228 0.61958 0.77255 0.48576 0.95510

1222 2.56335 5.36864 2.58498 1.97906 4.70400 2.94969 3.79389 4.19297 2.32028 3.67032 4.41792 1.80811 3.98312 2.85008 3.06716 2.31859 3.08815 3.75524 5.80355 4.38166 1591 - -

2.68643 4.42259 2.77530 2.73137 3.46330 2.40517 3.72511 3.29373 2.67712 2.69381 4.24723 2.90282 2.73736 3.18145 2.89804 2.37824 2.77544 2.98552 4.58511 3.61537

0.56275 1.16400 2.13599 1.21435 0.35226 0.48576 0.95510

1223 2.72704 5.04493 3.06203 2.43439 3.66096 3.10628 3.56335 3.50451 1.61017 3.31892 3.83336 3.03671 3.68246 2.58335 2.73987 2.73069 2.78734 3.15498 5.52218 3.88772 1603 - -

2.68618 4.42225 2.77519 2.73123 3.46354 2.40513 3.72494 3.29354 2.67741 2.69355 4.24690 2.90347 2.73739 3.18146 2.89801 2.37887 2.77519 2.98518 4.58477 3.61503

0.01521 4.58917 5.31151 0.61958 0.77255 0.37150 1.17021

1224 2.79751 2.66604 1.85766 2.05408 4.53311 3.04649 3.77010 3.64385 2.27620 3.51437 4.27782 2.94933 3.96538 2.88201 3.01932 2.77399 3.03111 3.59551 5.67920 4.28699 1604 - -

2.68618 4.42225 2.77519 2.73123 3.46354 2.40513 3.72494 3.29354 2.67741 2.69355 4.24690 2.90347 2.73739 3.18146 2.89801 2.37887 2.77519 2.98518 4.58477 3.61503

0.01347 4.70993 5.43228 0.61958 0.77255 0.48576 0.95510

1225 2.76910 5.26407 2.76714 2.29537 4.60285 3.26476 3.73147 4.08406 1.99255 3.00031 4.30664 2.13695 2.87648 2.74719 2.59480 2.66993 3.00025 3.65138 5.70005 3.99159 1605 - -

2.68618 4.42225 2.77519 2.73123 3.46354 2.40513 3.72494 3.29354 2.67741 2.69355 4.24690 2.90347 2.73739 3.18146 2.89801 2.37887 2.77519 2.98518 4.58477 3.61503

0.01347 4.70993 5.43228 0.61958 0.77255 0.48576 0.95510

1226 2.79786 5.05879 1.56002 2.47753 3.02989 2.98918 3.81052 3.74047 2.60776 2.76031 4.12850 2.94835 3.99458 2.95151 2.97676 2.81294 2.90679 3.40010 5.55062 4.19820 1606 - -

2.68618 4.42225 2.77519 2.73123 3.46354 2.40513 3.72494 3.29354 2.67741 2.69355 4.24690 2.90347 2.73739 3.18146 2.89801 2.37887 2.77519 2.98518 4.58477 3.61503

0.02639 4.70993 4.07251 0.61958 0.77255 0.48576 0.95510

1227 2.79292 4.22036 4.44753 3.85124 3.31320 3.92844 4.24497 1.85398 3.14632 1.76470 2.60171 3.98143 3.80270 3.88591 3.39337 3.22616 3.02502 1.84072 4.84285 3.65975 1607 - -

2.68618 4.42225 2.77519 2.73123 3.46354 2.40513 3.72494 3.29354 2.67741 2.69355 4.24690 2.90347 2.73739 3.18146 2.89801 2.37887 2.77519 2.98518 4.58477 3.61503

0.01364 4.69719 5.41953 0.61958 0.77255 0.51213 0.91434

1228 2.76299 5.25955 2.58888 2.28598 4.59944 3.53832 3.16677 3.72166 2.03576 3.16848 4.30175 2.71474 3.81548 2.47132 2.14113 2.72785 2.91457 3.31459 5.69489 4.28812 1608 - -

2.68618 4.42225 2.77519 2.73123 3.46354 2.40513 3.72494 3.29354 2.67741 2.69355 4.24690 2.90347 2.73739 3.18146 2.89801 2.37887 2.77519 2.98518 4.58477 3.61503

0.01364 4.69719 5.41953 0.61958 0.77255 0.46962 0.98146

1229 2.81104 2.35918 4.69145 4.08472 2.43479 3.97890 3.44707 1.91607 3.89033 2.34949 3.10716 4.11236 4.34001 4.03782 3.93030 3.28835 3.04370 1.67372 4.82367 3.64233 1609 - -

2.68618 4.42225 2.77519 2.73123 3.46354 2.40513 3.72494 3.29354 2.67741 2.69355 4.24690 2.90347 2.73739 3.18146 2.89801 2.37887 2.77519 2.98518 4.58477 3.61503

0.01347 4.70993 5.43228 0.61958 0.77255 0.48576 0.95510

1230 2.49843 5.03837 2.67081 2.38956 3.01421 3.58265 2.49826 3.44443 2.46213 3.30598 4.10294 2.99288 3.97361 2.91753 3.03668 2.34603 2.68314 3.38171 5.52645 3.03902 1610 - -

2.68618 4.42225 2.77519 2.73123 3.46354 2.40513 3.72494 3.29354 2.67741 2.69355 4.24690 2.90347 2.73739 3.18146 2.89801 2.37887 2.77519 2.98518 4.58477 3.61503

0.01347 4.70993 5.43228 0.61958 0.77255 0.48576 0.95510

1231 2.76555 5.20019 2.77294 2.09815 3.40214 2.83366 3.58120 3.66139 1.94421 3.22601 4.24901 2.61080 3.94425 2.84921 2.90153 2.55267 2.91253 3.13920 5.65160 3.96661 1611 - -
2.68618 4.42225 2.77519 2.73123 3.46354 2.40513 3.72494 3.29354 2.67741 2.69355 4.24690 2.90347 2.73739 3.18146 2.89801 2.37887 2.77519 2.98518 4.58477 3.61503
0.04372 4.70993 3.38808 0.61958 0.77255 0.48576 0.95510
1232 2.76532 5.22912 2.74306 2.34137 4.55402 2.19881 3.60471 4.02665 2.39648 3.52554 4.27573 2.65716 3.48541 2.75943 2.68006 2.51384 2.99701 3.60900 5.67335 2.57478 1612 - -
2.68618 4.42225 2.77519 2.73123 3.46354 2.40513 3.72494 3.29354 2.67741 2.69355 4.24690 2.90347 2.73739 3.18146 2.89801 2.37887 2.77519 2.98518 4.58477 3.61503
0.01388 4.68009 5.40243 0.61958 0.77255 0.54594 0.86582
1233 2.67664 4.39112 3.79688 3.00071 3.29567 3.33766 4.04603 2.56784 3.01232 1.73691 3.11656 3.40058 4.15406 3.44433 3.05965 2.53644 2.51154 2.38423 4.98440 3.76615 1613 - -
2.68618 4.42225 2.77519 2.73123 3.46354 2.40513 3.72494 3.29354 2.67741 2.69355 4.24690 2.90347 2.73739 3.18146 2.89801 2.37887 2.77519 2.98518 4.58477 3.61503
0.36244 4.68009 1.22165 0.61958 0.77255 0.54594 0.86582
1234 2.64648 4.83370 2.79620 2.46555 4.06215 3.48955 3.50229 2.87731 2.55442 3.09084 3.92096 3.03715 1.88394 2.79517 3.00613 2.73679 2.85136 3.16169 5.35290 4.02891 1614 - -
2.68618 4.42225 2.77519 2.73123 3.46354 2.40513 3.72494 3.29354 2.67741 2.69355 4.24690 2.90347 2.73739 3.18146 2.89801 2.37887 2.77519 2.98518 4.58477 3.61503
0.11463 4.33726 2.35141 0.61958 0.77255 0.32378 1.28521
1235 2.56027 3.95257 2.96786 2.50485 3.90414 3.53702 3.52074 3.77618 2.26914 3.33480 4.11872 2.77430 3.46163 2.60374 2.09961 2.54653 2.85527 2.69098 5.53520 4.17006 1615 - -
2.68618 4.42225 2.77519 2.73123 3.46354 2.40513 3.72494 3.29354 2.67741 2.69355 4.24690 2.90347 2.73739 3.18146 2.89801 2.37887 2.77519 2.98518 4.58477 3.61503
0.29212 4.60899 1.41322 0.61958 0.77255 0.67021 0.71662
1236 2.66978 5.14468 2.61665 2.21878 4.47446 3.01893 3.63150 3.95010 2.18871 3.44317 4.19213 2.60103 3.83296 2.73506 2.48825 2.29816 2.46047 3.52765 5.58627 4.18335 1616 - -
2.68618 4.42225 2.77519 2.73123 3.46354 2.40513 3.72494 3.29354 2.67741 2.69355 4.24690 2.90347 2.73739 3.18146 2.89801 2.37887 2.77519 2.98518 4.58477 3.61503
0.07295 4.33650 2.86001 0.61958 0.77255 0.87840 0.53691
1237 2.48093 2.65250 3.12719 2.46088 3.95448 3.34584 3.72097 3.35412 2.29876 2.98942 3.82407 2.85155 3.10950 2.89859 2.81332 2.71737 2.79348 2.78753 5.26665 3.95825 1617 - -
2.68618 4.42225 2.77519 2.73123 3.46354 2.40513 3.72494 3.29354 2.67741 2.69355 4.24690 2.90347 2.73739 3.18146 2.89801 2.37887 2.77519 2.98518 4.58477 3.61503
0.07539 4.33610 2.82129 0.61958 0.77255 0.87791 0.53726
1238 2.47178 5.01662 2.57859 2.30538 3.91936 3.09583 2.92719 3.75496 2.33698 2.92517 4.07242 2.74192 3.83932 2.68517 2.88439 2.57451 2.59983 3.37712 5.48392 3.68266 1618 - -
2.68618 4.42225 2.77519 2.73123 3.46354 2.40513 3.72494 3.29354 2.67741 2.69355 4.24690 2.90347 2.73739 3.18146 2.89801 2.37887 2.77519 2.98518 4.58477 3.61503
0.01968 4.33386 5.05621 0.61958 0.77255 0.74234 0.64626
1239 2.56893 5.18115 2.63000 2.39183 4.51152 2.34631 3.39346 3.98663 2.24059 3.47975 4.22976 2.41971 3.86909 2.77180 2.42082 2.48990 2.94038 3.56473 5.62276 4.22056 1619 - -
2.68618 4.42225 2.77519 2.73123 3.46354 2.40513 3.72494 3.29354 2.67741 2.69355 4.24690 2.90347 2.73739 3.18146 2.89801 2.37887 2.77519 2.98518 4.58477 3.61503
0.01753 4.44826 5.17060 0.61958 0.77255 0.29972 1.35102
1240 2.76692 5.09298 3.09748 2.54056 3.81296 2.32866 3.34616 3.32044 2.20485 3.36842 3.92341 2.88805 2.55808 2.89304 2.72722 2.40133 2.87317 3.44626 5.56916 4.20445 1620 - -
2.68618 4.42225 2.77519 2.73123 3.46354 2.40513 3.72494 3.29354 2.67741 2.69355 4.24690 2.90347 2.73739 3.18146 2.89801 2.37887 2.77519 2.98518 4.58477 3.61503
0.01347 4.70993 5.43228 0.61958 0.77255 0.48576 0.95510
1241 2.62675 5.09862 2.68836 2.38114 4.37682 3.57036 3.76612 3.40746 2.08532 2.93766 3.37049 2.97730 3.96190 2.83341 2.85748 2.49267 2.12636 3.08549 5.57352 4.20704 1621 - -
2.68618 4.42225 2.77519 2.73123 3.46354 2.40513 3.72494 3.29354 2.67741 2.69355 4.24690 2.90347 2.73739 3.18146 2.89801 2.37887 2.77519 2.98518 4.58477 3.61503
0.01347 4.70993 5.43228 0.61958 0.77255 0.48576 0.95510
1242 2.47520 5.24341 2.81050 2.47341 4.57450 3.26583 3.73634 4.05021 2.27766 3.54316 4.28919 2.35013 3.94014 2.49779 2.85123 1.88444 2.63239 3.34232 5.68579 4.28382 1622 - -
2.68618 4.42225 2.77519 2.73123 3.46354 2.40513 3.72494 3.29354 2.67741 2.69355 4.24690 2.90347 2.73739 3.18146 2.89801 2.37887 2.77519 2.98518 4.58477 3.61503

0.01347 4.70993 5.43228 0.61958 0.77255 0.48576 0.95510
1243 2.44541 4.23691 4.39555 3.80467 3.13151 3.92217 4.23500 2.25401 3.66260 2.35498 3.35207 3.95644 4.29012 3.86116 3.79919 2.93638 2.92708 2.09581 4.85212 1.55061 1623 - -
2.68618 4.42225 2.77519 2.73123 3.46354 2.40513 3.72494 3.29354 2.67741 2.69355 4.24690 2.90347 2.73739 3.18146 2.89801 2.37887 2.77519 2.98518 4.58477 3.61503
0.01347 4.70993 5.43228 0.61958 0.77255 0.48576 0.95510
1244 2.77006 4.45975 3.69956 3.13073 3.31017 3.39152 3.26715 2.51937 2.98637 2.35924 2.57101 3.52173 4.14117 2.94425 3.12065 2.14250 2.62752 2.57390 5.04570 3.42262 1624 - -
2.68618 4.42225 2.77519 2.73123 3.46354 2.40513 3.72494 3.29354 2.67741 2.69355 4.24690 2.90347 2.73739 3.18146 2.89801 2.37887 2.77519 2.98518 4.58477 3.61503
0.01347 4.70993 5.43228 0.61958 0.77255 0.48576 0.95510
1245 2.81448 5.32823 2.12066 1.86785 4.67230 3.41007 3.76002 4.15848 1.92669 3.63070 4.37221 3.01874 3.96269 2.41886 2.55887 2.62320 3.04730 3.71895 5.75702 4.34425 1625 - -
2.68618 4.42225 2.77519 2.73123 3.46354 2.40513 3.72494 3.29354 2.67741 2.69355 4.24690 2.90347 2.73739 3.18146 2.89801 2.37887 2.77519 2.98518 4.58477 3.61503
0.01347 4.70993 5.43228 0.61958 0.77255 0.48576 0.95510
1246 2.76839 4.66966 2.87387 2.68878 3.21880 2.68501 3.44464 3.21549 2.82448 2.53248 2.29974 3.31562 3.72944 2.99437 3.12133 2.90574 2.78947 2.64252 5.22541 3.30094 1626 - -
2.68618 4.42225 2.77519 2.73123 3.46354 2.40513 3.72494 3.29354 2.67741 2.69355 4.24690 2.90347 2.73739 3.18146 2.89801 2.37887 2.77519 2.98518 4.58477 3.61503
0.01347 4.70993 5.43228 0.61958 0.77255 0.48576 0.95510
1247 3.66887 4.94907 5.50631 4.92431 1.97135 4.94316 5.17198 2.08007 4.76858 1.69531 1.08363 5.05434 5.10356 4.74737 4.76674 4.29462 3.88205 2.72370 5.36117 4.23597 1627 - -
2.68618 4.42225 2.77519 2.73123 3.46354 2.40513 3.72494 3.29354 2.67741 2.69355 4.24690 2.90347 2.73739 3.18146 2.89801 2.37887 2.77519 2.98518 4.58477 3.61503
0.01347 4.70993 5.43228 0.61958 0.77255 0.48576 0.95510
1248 2.37939 5.22020 3.00631 2.38698 4.54204 3.30691 3.31145 4.00779 2.11972 2.99465 4.26805 3.03784 3.95148 2.48161 1.85386 2.75278 3.00776 3.43824 5.66451 3.99452 1628 - -
2.68618 4.42225 2.77519 2.73123 3.46354 2.40513 3.72494 3.29354 2.67741 2.69355 4.24690 2.90347 2.73739 3.18146 2.89801 2.37887 2.77519 2.98518 4.58477 3.61503
0.01347 4.70993 5.43228 0.61958 0.77255 0.48576 0.95510
1249 2.77227 5.25099 2.72845 2.47330 4.58346 3.35868 3.61046 4.06003 2.39896 3.55050 3.98908 1.92459 3.94174 2.73484 2.41859 2.07677 2.70067 3.63502 5.69098 4.28843 1629 - -
2.68618 4.42225 2.77519 2.73123 3.46354 2.40513 3.72494 3.29354 2.67741 2.69355 4.24690 2.90347 2.73739 3.18146 2.89801 2.37887 2.77519 2.98518 4.58477 3.61503
0.01347 4.70993 5.43228 0.61958 0.77255 0.48576 0.95510
1250 2.18595 4.56684 4.23745 4.05336 5.08632 3.34208 5.02872 4.50133 4.11247 4.22211 5.01969 3.95591 4.16671 4.34751 4.33214 0.49986 2.95885 3.78619 6.42578 5.28137 1630 - -
2.68618 4.42225 2.77519 2.73123 3.46354 2.40513 3.72494 3.29354 2.67741 2.69355 4.24690 2.90347 2.73739 3.18146 2.89801 2.37887 2.77519 2.98518 4.58477 3.61503
0.01347 4.70993 5.43228 0.61958 0.77255 0.48576 0.95510
1251 2.77364 5.08364 2.98424 2.33630 4.35676 3.58037 3.77409 3.10548 1.55075 2.97161 4.14472 3.08486 3.97125 2.90171 2.64091 2.67944 2.77633 2.88227 5.56093 4.20169 1631 - -
2.68618 4.42225 2.77519 2.73123 3.46354 2.40513 3.72494 3.29354 2.67741 2.69355 4.24690 2.90347 2.73739 3.18146 2.89801 2.37887 2.77519 2.98518 4.58477 3.61503
0.01347 4.70993 5.43228 0.61958 0.77255 0.48576 0.95510
1252 4.68209 5.65961 5.53955 5.44965 0.45390 5.20026 3.91827 4.21491 5.23773 3.37351 4.75116 4.77787 5.46807 4.93632 5.06658 4.62129 4.88660 4.19135 3.99674 1.72322 1632 - -
2.68618 4.42225 2.77519 2.73123 3.46354 2.40513 3.72494 3.29354 2.67741 2.69355 4.24690 2.90347 2.73739 3.18146 2.89801 2.37887 2.77519 2.98518 4.58477 3.61503
0.01347 4.70993 5.43228 0.61958 0.77255 0.48576 0.95510
1253 3.89452 0.15590 5.43563 5.39492 5.34084 4.27186 5.88775 4.75000 5.28623 4.55813 5.72318 5.22145 5.01851 5.59262 5.23761 4.15987 4.42548 4.40485 6.45799 5.59917 1633 - -
2.68618 4.42225 2.77519 2.73123 3.46354 2.40513 3.72494 3.29354 2.67741 2.69355 4.24690 2.90347 2.73739 3.18146 2.89801 2.37887 2.77519 2.98518 4.58477 3.61503
0.01347 4.70993 5.43228 0.61958 0.77255 0.48576 0.95510
1254 3.18389 4.54304 4.95705 4.38019 3.35655 3.34966 4.76484 1.47048 4.22467 1.07964 3.36734 4.49993 4.71589 4.37450 4.31236 3.72995 3.41990 2.24177 5.23337 4.08131 1634 - -

2.68618 4.42225 2.77519 2.73123 3.46354 2.40513 3.72494 3.29354 2.67741 2.69355 4.24690 2.90347 2.73739 3.18146 2.89801 2.37887 2.77519 2.98518 4.58477 3.61503
0.01347 4.70993 5.43228 0.61958 0.77255 0.48576 0.95510
1255 2.47116 1.73924 3.28826 2.76788 4.13804 3.63867 3.27721 3.54344 2.71212 3.17880 4.01946 2.80948 4.06170 2.10366 3.12497 2.89137 3.06322 3.24233 5.44629 4.14537 1635 - -
2.68618 4.42225 2.77519 2.73123 3.46354 2.40513 3.72494 3.29354 2.67741 2.69355 4.24690 2.90347 2.73739 3.18146 2.89801 2.37887 2.77519 2.98518 4.58477 3.61503
0.01347 4.70993 5.43228 0.61958 0.77255 0.48576 0.95510
1256 2.44071 4.51519 4.00114 3.46181 3.04529 3.85537 4.26808 2.55997 3.38738 2.52681 3.62096 3.77845 1.21034 3.67355 3.68233 3.16028 3.14564 2.66886 5.16804 3.92332 1636 - -
2.68618 4.42225 2.77519 2.73123 3.46354 2.40513 3.72494 3.29354 2.67741 2.69355 4.24690 2.90347 2.73739 3.18146 2.89801 2.37887 2.77519 2.98518 4.58477 3.61503
0.01347 4.70993 5.43228 0.61958 0.77255 0.48576 0.95510
1257 2.27078 5.10021 3.10138 2.54415 4.38085 3.42511 3.76983 3.82099 2.25656 2.97980 3.85849 3.07666 2.53673 2.74596 2.70448 1.93527 3.00301 3.45633 5.57560 4.21148 1637 - -
2.68618 4.42225 2.77519 2.73123 3.46354 2.40513 3.72494 3.29354 2.67741 2.69355 4.24690 2.90347 2.73739 3.18146 2.89801 2.37887 2.77519 2.98518 4.58477 3.61503
0.01347 4.70993 5.43228 0.61958 0.77255 0.48576 0.95510
1258 3.99227 5.60978 4.81709 4.83944 5.82962 0.12816 5.82258 5.70860 5.11939 5.21140 6.24447 4.98512 5.00978 5.37976 5.19299 4.19709 4.52751 5.08067 6.63378 5.98272 1638 - -
2.68618 4.42225 2.77519 2.73123 3.46354 2.40513 3.72494 3.29354 2.67741 2.69355 4.24690 2.90347 2.73739 3.18146 2.89801 2.37887 2.77519 2.98518 4.58477 3.61503
0.05699 4.70993 3.07074 0.61958 0.77255 0.48576 0.95510
1259 2.85720 4.88085 1.81240 2.68787 3.32720 3.70702 3.37813 3.48190 2.79151 3.09982 3.96954 3.27805 4.09566 3.12197 3.22674 2.83352 3.08850 3.20476 3.34298 2.00440 1639 - -
2.68618 4.42225 2.77519 2.73123 3.46354 2.40513 3.72494 3.29354 2.67741 2.69355 4.24690 2.90347 2.73739 3.18146 2.89801 2.37887 2.77519 2.98518 4.58477 3.61503
0.01406 4.66701 5.38935 0.61958 0.77255 0.43610 1.04003
1260 2.56590 5.31810 2.64509 1.54795 4.65037 3.55779 3.80952 4.12694 2.58668 3.62687 4.38622 3.03651 3.99151 2.82970 3.08681 2.05562 2.25951 3.70671 5.77781 4.36797 1640 - -
2.68618 4.42225 2.77519 2.73123 3.46354 2.40513 3.72494 3.29354 2.67741 2.69355 4.24690 2.90347 2.73739 3.18146 2.89801 2.37887 2.77519 2.98518 4.58477 3.61503
0.01347 4.70993 5.43228 0.61958 0.77255 0.48576 0.95510
1261 2.67737 4.31616 4.03848 3.46265 3.11766 3.24366 4.13401 2.77677 3.37231 2.45345 3.43448 3.74319 2.32945 3.62382 3.61630 2.88383 3.00876 1.54716 4.92401 2.90297 1641 - -
2.68618 4.42225 2.77519 2.73123 3.46354 2.40513 3.72494 3.29354 2.67741 2.69355 4.24690 2.90347 2.73739 3.18146 2.89801 2.37887 2.77519 2.98518 4.58477 3.61503
0.01347 4.70993 5.43228 0.61958 0.77255 0.48576 0.95510
1262 1.92003 4.79410 3.30839 2.75786 4.01952 3.63183 3.62779 3.41537 2.74170 3.06644 3.91151 2.55998 4.03660 3.08248 3.17590 2.35917 2.06545 3.12891 3.09230 4.06274 1642 - -
2.68618 4.42225 2.77519 2.73123 3.46354 2.40513 3.72494 3.29354 2.67741 2.69355 4.24690 2.90347 2.73739 3.18146 2.89801 2.37887 2.77519 2.98518 4.58477 3.61503
0.01347 4.70993 5.43228 0.61958 0.77255 0.48576 0.95510
1263 2.59329 4.87622 3.53294 3.03679 4.63228 3.53803 4.15075 4.05023 2.85507 3.64331 4.45190 3.43221 4.10955 3.31661 1.92964 1.07547 2.79422 3.60607 5.83722 4.55987 1643 - -
2.68618 4.42225 2.77519 2.73123 3.46354 2.40513 3.72494 3.29354 2.67741 2.69355 4.24690 2.90347 2.73739 3.18146 2.89801 2.37887 2.77519 2.98518 4.58477 3.61503
0.01347 4.70993 5.43228 0.61958 0.77255 0.48576 0.95510
1264 2.59884 3.63104 3.15955 2.60150 4.31177 3.14366 3.80082 3.73727 2.34317 3.16495 4.11817 2.83564 1.87037 2.93852 2.32859 2.80303 3.01715 3.39330 5.53661 4.19331 1644 - -
2.68618 4.42225 2.77519 2.73123 3.46354 2.40513 3.72494 3.29354 2.67741 2.69355 4.24690 2.90347 2.73739 3.18146 2.89801 2.37887 2.77519 2.98518 4.58477 3.61503
0.01347 4.70993 5.43228 0.61958 0.77255 0.48576 0.95510
1265 2.79720 5.06467 3.71370 3.19628 4.84742 3.69396 4.16389 4.25273 2.63261 3.79180 4.62866 3.55669 4.24765 3.32421 0.90741 1.90366 3.34183 3.81824 5.90762 4.68684 1645 - -
2.68618 4.42225 2.77519 2.73123 3.46354 2.40513 3.72494 3.29354 2.67741 2.69355 4.24690 2.90347 2.73739 3.18146 2.89801 2.37887 2.77519 2.98518 4.58477 3.61503
0.01347 4.70993 5.43228 0.61958 0.77255 0.48576 0.95510

1266 2.68061 4.24590 4.67054 3.59534 3.16104 4.03289 4.36943 1.64410 3.89722 1.79728 2.80694 4.14011 4.38987 4.06064 3.96696 3.34140 2.59729 1.81937 4.90609 3.72687 1646 - -
2.68618 4.42225 2.77519 2.73123 3.46354 2.40513 3.72494 3.29354 2.67741 2.69355 4.24690 2.90347 2.73739 3.18146 2.89801 2.37887 2.77519 2.98518 4.58477 3.61503
0.01347 4.70993 5.43228 0.61958 0.77255 0.48576 0.95510
1267 2.80532 4.26745 4.30378 3.16401 1.81928 3.91562 4.21868 2.61856 3.59450 2.43743 3.37640 3.91295 3.95348 3.81065 3.76104 3.20752 2.93083 1.45872 4.87261 3.42853 1647 - -
2.68618 4.42225 2.77519 2.73123 3.46354 2.40513 3.72494 3.29354 2.67741 2.69355 4.24690 2.90347 2.73739 3.18146 2.89801 2.37887 2.77519 2.98518 4.58477 3.61503
0.01347 4.70993 5.43228 0.61958 0.77255 0.48576 0.95510
1268 3.35989 6.23947 1.10245 1.05991 5.50962 3.62600 4.25305 5.08456 3.38442 4.53082 5.41635 3.06920 4.30037 3.42994 4.06851 3.33703 3.81381 4.60926 6.67586 5.07833 1648 - -
2.68618 4.42225 2.77519 2.73123 3.46354 2.40513 3.72494 3.29354 2.67741 2.69355 4.24690 2.90347 2.73739 3.18146 2.89801 2.37887 2.77519 2.98518 4.58477 3.61503
0.01347 4.70993 5.43228 0.61958 0.77255 0.48576 0.95510
1269 0.91320 3.70883 4.47570 4.13558 4.88773 2.99587 4.96963 4.31816 4.07617 4.01589 4.81474 3.97802 4.14179 4.30212 4.29786 1.24880 3.12655 3.66776 6.24945 5.11037 1649 - -
2.68618 4.42225 2.77519 2.73123 3.46354 2.40513 3.72494 3.29354 2.67741 2.69355 4.24690 2.90347 2.73739 3.18146 2.89801 2.37887 2.77519 2.98518 4.58477 3.61503
0.01347 4.70993 5.43228 0.61958 0.77255 0.48576 0.95510
1270 3.25635 4.76998 5.42888 4.85408 3.22997 4.82040 5.19364 1.09810 4.70592 1.30141 2.68394 4.96150 5.03770 4.75890 4.73028 4.17279 3.71779 2.14045 5.48050 4.39913 1650 - -
2.68618 4.42225 2.77519 2.73123 3.46354 2.40513 3.72494 3.29354 2.67741 2.69355 4.24690 2.90347 2.73739 3.18146 2.89801 2.37887 2.77519 2.98518 4.58477 3.61503
0.01347 4.70993 5.43228 0.61958 0.77255 0.48576 0.95510
1271 2.47599 4.18904 4.52491 3.92392 2.68341 3.92176 3.92238 2.24611 3.75356 1.76369 3.30480 3.86435 4.28735 3.59736 3.83745 3.22327 3.01020 2.10647 4.79938 2.10211 1651 - -
2.68618 4.42225 2.77519 2.73123 3.46354 2.40513 3.72494 3.29354 2.67741 2.69355 4.24690 2.90347 2.73739 3.18146 2.89801 2.37887 2.77519 2.98518 4.58477 3.61503
0.01347 4.70993 5.43228 0.61958 0.77255 0.48576 0.95510
1272 2.08435 4.61735 3.47608 2.91399 3.48252 3.70051 3.93916 3.14988 2.88328 2.54185 3.71611 3.20505 4.08534 2.86945 3.27958 1.72572 3.00364 2.79827 5.17945 2.85012 1652 - -
2.68618 4.42225 2.77519 2.73123 3.46354 2.40513 3.72494 3.29354 2.67741 2.69355 4.24690 2.90347 2.73739 3.18146 2.89801 2.37887 2.77519 2.98518 4.58477 3.61503
0.01347 4.70993 5.43228 0.61958 0.77255 0.48576 0.95510
1273 2.96401 5.33558 2.70339 2.10568 4.61242 1.28105 3.11185 4.07483 2.73830 3.07115 4.42273 3.08922 4.07303 3.05146 3.23130 2.93557 3.21076 3.70059 5.80945 4.41440 1653 - -
2.68618 4.42225 2.77519 2.73123 3.46354 2.40513 3.72494 3.29354 2.67741 2.69355 4.24690 2.90347 2.73739 3.18146 2.89801 2.37887 2.77519 2.98518 4.58477 3.61503
0.01347 4.70993 5.43228 0.61958 0.77255 0.48576 0.95510
1274 2.82741 0.41975 4.89048 4.76142 4.80208 3.53198 5.30840 4.14431 4.55606 4.03920 4.99098 4.33638 4.36064 4.82225 4.60569 2.77915 3.38416 3.64583 6.23630 5.04838 1654 - -
2.68618 4.42225 2.77519 2.73123 3.46354 2.40513 3.72494 3.29354 2.67741 2.69355 4.24690 2.90347 2.73739 3.18146 2.89801 2.37887 2.77519 2.98518 4.58477 3.61503
0.01347 4.70993 5.43228 0.61958 0.77255 0.48576 0.95510
1275 3.62149 4.81699 5.70680 5.26358 4.21251 5.24861 6.06914 1.11156 5.21280 2.61675 3.93462 5.42614 5.47716 5.48592 5.39258 4.70224 3.88849 0.79231 6.37117 5.13800 1655 - -
2.68618 4.42225 2.77519 2.73123 3.46354 2.40513 3.72494 3.29354 2.67741 2.69355 4.24690 2.90347 2.73739 3.18146 2.89801 2.37887 2.77519 2.98518 4.58477 3.61503
0.01347 4.70993 5.43228 0.61958 0.77255 0.48576 0.95510
1276 4.19546 5.72905 4.88467 4.87584 5.69810 4.37493 5.80577 5.56089 5.02238 5.03903 6.16559 5.07870 0.12805 5.37280 5.10382 4.40233 4.70166 5.09038 6.56663 5.84907 1656 - -
2.68618 4.42225 2.77519 2.73123 3.46354 2.40513 3.72494 3.29354 2.67741 2.69355 4.24690 2.90347 2.73739 3.18146 2.89801 2.37887 2.77519 2.98518 4.58477 3.61503
0.01347 4.70993 5.43228 0.61958 0.77255 0.48576 0.95510
1277 3.51704 4.85189 5.34003 4.97992 4.11706 4.75955 5.67691 2.12742 4.82778 2.53385 3.96717 5.07501 5.20356 5.15233 4.98661 4.25493 3.83953 0.46680 6.12906 4.84484 1657 - -
2.68618 4.42225 2.77519 2.73123 3.46354 2.40513 3.72494 3.29354 2.67741 2.69355 4.24690 2.90347 2.73739 3.18146 2.89801 2.37887 2.77519 2.98518 4.58477 3.61503

0.01347 4.70993 5.43228 0.61958 0.77255 0.48576 0.95510

1278 3.61163 4.87027 5.56431 4.99439 2.10431 4.96182 5.30008 1.15852 4.84897 1.31343 3.28579 5.10706 5.13848 4.85674 4.85195 4.32501 3.83504 2.16950 5.50880 4.41723 1658 - -

2.68618 4.42225 2.77519 2.73123 3.46354 2.40513 3.72494 3.29354 2.67741 2.69355 4.24690 2.90347 2.73739 3.18146 2.89801 2.37887 2.77519 2.98518 4.58477 3.61503

0.01347 4.70993 5.43228 0.61958 0.77255 0.48576 0.95510

1279 3.51702 4.77737 5.47590 4.92677 1.98178 4.89577 5.27201 0.96517 4.79221 1.93417 3.44433 5.03019 5.11692 4.87456 4.83731 4.26167 3.75157 1.88186 5.55348 4.38361 1659 - -

2.68618 4.42225 2.77519 2.73123 3.46354 2.40513 3.72494 3.29354 2.67741 2.69355 4.24690 2.90347 2.73739 3.18146 2.89801 2.37887 2.77519 2.98518 4.58477 3.61503

0.01347 4.70993 5.43228 0.61958 0.77255 0.48576 0.95510

1280 2.38276 5.20662 2.90210 2.41615 4.52077 3.55335 3.10123 3.98564 2.49731 3.49874 4.25655 2.21143 3.94890 2.62558 2.98428 1.77115 2.90133 3.58079 4.65743 4.26653 1660 - -

2.68618 4.42225 2.77519 2.73123 3.46354 2.40513 3.72494 3.29354 2.67741 2.69355 4.24690 2.90347 2.73739 3.18146 2.89801 2.37887 2.77519 2.98518 4.58477 3.61503

0.01347 4.70993 5.43228 0.61958 0.77255 0.48576 0.95510

1281 2.86042 5.31968 1.54197 2.42868 4.64436 3.56261 3.81692 4.11828 2.59681 3.62290 4.38640 3.04101 2.68769 2.83208 2.95163 2.07609 3.10038 3.42111 5.77801 4.37033 1661 - -

2.68618 4.42225 2.77519 2.73123 3.46354 2.40513 3.72494 3.29354 2.67741 2.69355 4.24690 2.90347 2.73739 3.18146 2.89801 2.37887 2.77519 2.98518 4.58477 3.61503

0.01347 4.70993 5.43228 0.61958 0.77255 0.48576 0.95510

1282 2.78543 5.26200 2.64189 2.38816 4.59064 1.91840 3.32534 4.06686 2.44270 3.55999 4.30813 2.29570 3.94990 2.67843 2.87231 2.68765 2.89291 3.64401 5.70333 3.40946 1662 - -

2.68618 4.42225 2.77519 2.73123 3.46354 2.40513 3.72494 3.29354 2.67741 2.69355 4.24690 2.90347 2.73739 3.18146 2.89801 2.37887 2.77519 2.98518 4.58477 3.61503

0.01347 4.70993 5.43228 0.61958 0.77255 0.48576 0.95510

1283 2.68044 4.22646 4.38551 3.79369 2.57658 3.90812 4.21496 1.91547 3.65037 2.31837 3.34137 3.94256 4.27619 3.84701 3.78451 2.37840 2.60555 2.46683 4.82845 2.06229 1663 - -

2.68618 4.42225 2.77519 2.73123 3.46354 2.40513 3.72494 3.29354 2.67741 2.69355 4.24690 2.90347 2.73739 3.18146 2.89801 2.37887 2.77519 2.98518 4.58477 3.61503

0.01347 4.70993 5.43228 0.61958 0.77255 0.48576 0.95510

1284 2.12902 4.74959 3.20700 2.19798 3.93403 3.47939 3.87910 3.32006 2.75535 2.98344 3.45184 3.25781 4.04312 2.98591 3.07178 2.73327 3.00115 1.82472 5.29287 3.38405 1664 - -

2.68618 4.42225 2.77519 2.73123 3.46354 2.40513 3.72494 3.29354 2.67741 2.69355 4.24690 2.90347 2.73739 3.18146 2.89801 2.37887 2.77519 2.98518 4.58477 3.61503

0.01347 4.70993 5.43228 0.61958 0.77255 0.48576 0.95510

1285 2.86908 4.40857 4.08670 3.52071 3.07260 3.91728 4.12760 2.84450 3.18984 1.57613 3.48997 3.79743 2.08036 3.67369 3.65320 3.19591 3.10106 2.65939 4.84112 2.26969 1665 - -

2.68607 4.42230 2.77525 2.73129 3.46359 2.40518 3.72500 3.29359 2.67740 2.69360 4.24620 2.90337 2.73745 3.18152 2.89806 2.37892 2.77507 2.98524 4.58482 3.61469

0.21654 1.65901 5.43228 0.17075 1.85171 0.48576 0.95510

1286 3.27226 5.34849 3.31759 3.11273 4.91336 3.78477 4.38448 4.48232 3.08849 3.99933 4.90372 3.57285 0.83708 1.79060 3.41022 3.31879 3.61767 4.07753 6.08162 4.79993 1667 - -

2.68622 4.42236 2.77531 2.73134 3.46365 2.40524 3.72506 3.29336 2.67732 2.69360 4.24676 2.90358 2.73751 3.18145 2.89812 2.37898 2.77509 2.98529 4.58488 3.61306

0.21654 1.65901 5.43228 0.67764 0.70890 0.48576 0.95510

1287 4.74291 5.67133 5.61129 5.54627 0.66746 5.24139 3.88108 4.32470 5.31509 3.48713 4.84491 4.77899 5.49140 4.95319 5.10209 4.64465 4.93326 4.27160 1.76963 1.84581 1670 - -

2.68618 4.42225 2.77519 2.73123 3.46354 2.40513 3.72494 3.29354 2.67741 2.69355 4.24690 2.90347 2.73739 3.18146 2.89801 2.37887 2.77519 2.98518 4.58477 3.61503

0.01347 4.70993 5.43228 0.61958 0.77255 0.48576 0.95510

1288 2.66014 5.06108 3.11215 2.01660 3.10667 3.57877 2.68649 3.75991 2.55471 3.19541 4.12388 2.92991 3.74793 2.80907 3.02873 1.94015 2.95108 3.40824 5.54408 3.55376 1671 - -

2.68618 4.42225 2.77519 2.73123 3.46354 2.40513 3.72494 3.29354 2.67741 2.69355 4.24690 2.90347 2.73739 3.18146 2.89801 2.37887 2.77519 2.98518 4.58477 3.61503

0.01347 4.70993 5.43228 0.61958 0.77255 0.48576 0.95510

1289 2.78764 5.16037 1.76048 2.14479 3.85416 3.15752 3.77403 3.68244 2.54282 2.47615 4.21783 2.68397 3.96790 2.89348 3.02726 2.70182 3.02042 3.52094 5.62723 4.24865 1672 - -

2.68618 4.42225 2.77519 2.73123 3.46354 2.40513 3.72494 3.29354 2.67741 2.69355 4.24690 2.90347 2.73739 3.18146 2.89801 2.37887 2.77519 2.98518 4.58477 3.61503

0.01347 4.70993 5.43228 0.61958 0.77255 0.48576 0.95510

1290 2.69269 4.32076 4.22955 3.46247 3.45125 3.93411 4.25149 2.27592 3.54376 2.49760 2.98824 3.89067 2.37685 3.78182 3.75297 3.22126 2.75682 1.38375 4.97472 3.77774 1673 - -

2.68618 4.42225 2.77519 2.73123 3.46354 2.40513 3.72494 3.29354 2.67741 2.69355 4.24690 2.90347 2.73739 3.18146 2.89801 2.37887 2.77519 2.98518 4.58477 3.61503

0.01347 4.70993 5.43228 0.61958 0.77255 0.48576 0.95510

1291 2.79290 4.59107 3.20833 2.63597 3.73999 3.73627 3.97785 2.44194 2.37851 1.48001 3.68371 3.41987 4.11893 3.26245 3.31247 2.97689 3.02434 2.67471 5.16574 3.91722 1674 - -

2.68618 4.42225 2.77519 2.73123 3.46354 2.40513 3.72494 3.29354 2.67741 2.69355 4.24690 2.90347 2.73739 3.18146 2.89801 2.37887 2.77519 2.98518 4.58477 3.61503

0.01347 4.70993 5.43228 0.61958 0.77255 0.48576 0.95510

1292 3.01475 5.55158 1.64524 2.47329 4.87368 3.58074 3.91636 4.37009 2.62385 3.84831 4.62114 1.44310 3.61517 2.90918 3.03618 2.78902 3.26426 3.93446 5.98831 4.54442 1675 - -

2.68618 4.42225 2.77520 2.73115 3.46354 2.40513 3.72495 3.29354 2.67741 2.69355 4.24690 2.90347 2.73740 3.18147 2.89801 2.37887 2.77520 2.98519 4.58477 3.61503

0.03199 3.60777 5.43228 0.48908 0.94982 0.48576 0.95510

1293 2.86297 4.42132 4.03297 3.46355 3.06916 3.89963 3.25030 2.72942 3.22502 2.61104 3.53462 3.75278 3.83575 3.62906 3.43310 3.03682 3.09390 2.69080 1.73045 2.06734 1677 - -

2.68618 4.42225 2.77519 2.73123 3.46354 2.40513 3.72494 3.29354 2.67741 2.69355 4.24690 2.90347 2.73739 3.18146 2.89801 2.37887 2.77519 2.98518 4.58477 3.61503

0.01347 4.70993 5.43228 0.61958 0.77255 0.48576 0.95510

1294 2.49862 5.26523 2.81367 1.98720 4.60247 3.54492 3.73663 4.08256 2.36595 3.56703 4.30928 2.91764 3.54709 2.83641 1.98699 2.21461 2.66310 3.65194 5.70272 4.29673 1678 - -

2.68618 4.42225 2.77519 2.73123 3.46354 2.40513 3.72494 3.29354 2.67741 2.69355 4.24690 2.90347 2.73739 3.18146 2.89801 2.37887 2.77519 2.98518 4.58477 3.61503

0.01347 4.70993 5.43228 0.61958 0.77255 0.48576 0.95510

1295 2.60034 5.27225 2.85004 2.07473 4.60811 3.40659 3.74419 4.08734 1.95526 3.57316 4.31751 3.01905 3.94775 2.75070 2.84451 1.74184 2.76918 3.65838 5.71007 4.30451 1679 - -

2.68618 4.42225 2.77519 2.73123 3.46354 2.40513 3.72494 3.29354 2.67741 2.69355 4.24690 2.90347 2.73739 3.18146 2.89801 2.37887 2.77519 2.98518 4.58477 3.61503

0.01347 4.70993 5.43228 0.61958 0.77255 0.48576 0.95510

1296 3.58146 4.87692 5.12840 4.67672 0.92627 4.60807 4.13763 2.58414 4.49509 2.62135 3.81418 4.51396 4.91633 4.50054 4.49775 3.94470 3.80588 2.62126 4.33096 1.72607 1680 - -

2.68618 4.42225 2.77519 2.73123 3.46354 2.40513 3.72494 3.29354 2.67741 2.69355 4.24690 2.90347 2.73739 3.18146 2.89801 2.37887 2.77519 2.98518 4.58477 3.61503

0.01347 4.70993 5.43228 0.61958 0.77255 0.48576 0.95510

1297 1.67025 3.80218 4.44981 4.10842 4.92397 2.96326 4.96361 4.35933 4.05386 4.05131 4.84359 3.96653 4.13912 4.28473 4.28466 0.70171 3.01276 3.69106 6.27572 5.13548 1681 - -

2.68618 4.42225 2.77519 2.73123 3.46354 2.40513 3.72494 3.29354 2.67741 2.69355 4.24690 2.90347 2.73739 3.18146 2.89801 2.37887 2.77519 2.98518 4.58477 3.61503

0.01347 4.70993 5.43228 0.61958 0.77255 0.48576 0.95510

1298 3.61134 4.81578 5.65404 5.17687 4.07001 5.20072 5.88192 1.65551 5.10416 2.14719 3.81343 5.34518 5.41129 5.32988 5.25717 4.62729 3.86821 0.62445 6.18311 4.98850 1682 - -

2.68618 4.42225 2.77519 2.73123 3.46354 2.40513 3.72494 3.29354 2.67741 2.69355 4.24690 2.90347 2.73739 3.18146 2.89801 2.37887 2.77519 2.98518 4.58477 3.61503

0.01347 4.70993 5.43228 0.61958 0.77255 0.48576 0.95510

1299 2.77270 4.25004 4.23318 3.51028 1.96041 3.87450 3.87097 1.95429 3.32101 2.32026 3.36483 3.85443 4.24403 3.52761 3.16954 3.04420 2.68679 2.37049 4.86069 2.78061 1683 - -

2.68618 4.42225 2.77519 2.73123 3.46354 2.40513 3.72494 3.29354 2.67741 2.69355 4.24690 2.90347 2.73739 3.18146 2.89801 2.37887 2.77519 2.98518 4.58477 3.61503

0.01347 4.70993 5.43228 0.61958 0.77255 0.48576 0.95510

1300 2.87228 4.39318 4.95536 4.36657 3.28603 4.28537 4.64896 1.60129 4.19155 2.13885 3.24827 4.41815 4.61601 4.33822 4.24234 3.60898 3.28402 1.10871 4.26244 3.95357 1684 - -

2.68618 4.42225 2.77519 2.73123 3.46354 2.40513 3.72494 3.29354 2.67741 2.69355 4.24690 2.90347 2.73739 3.18146 2.89801 2.37887 2.77519 2.98518 4.58477 3.61503

0.01347 4.70993 5.43228 0.61958 0.77255 0.48576 0.95510

1301 2.44575 5.23680 2.73754 2.36894 4.56306 3.54806 3.57504 4.03587 2.48628 3.35059 4.28405 2.65347 2.43040 2.84533 2.67236 1.90536 2.91319 3.61764 5.68188 4.28225 1685 - -
2.68618 4.42225 2.77519 2.73123 3.46354 2.40513 3.72494 3.29354 2.67741 2.69355 4.24690 2.90347 2.73739 3.18146 2.89801 2.37887 2.77519 2.98518 4.58477 3.61503
0.01347 4.70993 5.43228 0.61958 0.77255 0.48576 0.95510
1302 2.64552 4.97904 3.16967 1.69210 4.22207 3.60414 3.80920 3.40038 2.42965 3.23937 4.05279 3.13600 3.99628 2.95909 2.85774 2.50881 2.91885 2.05923 5.48242 4.14855 1686 - -
2.68618 4.42225 2.77519 2.73123 3.46354 2.40513 3.72494 3.29354 2.67741 2.69355 4.24690 2.90347 2.73739 3.18146 2.89801 2.37887 2.77519 2.98518 4.58477 3.61503
0.01347 4.70993 5.43228 0.61958 0.77255 0.48576 0.95510
1303 2.80917 5.32346 2.47193 1.80839 4.66528 3.54860 3.75880 4.15229 1.79073 3.62646 4.36757 2.73539 3.95889 2.69197 2.90499 2.27883 2.92407 3.71286 5.75497 4.34010 1687 - -
2.68618 4.42225 2.77519 2.73123 3.46354 2.40513 3.72494 3.29354 2.67741 2.69355 4.24690 2.90347 2.73739 3.18146 2.89801 2.37887 2.77519 2.98518 4.58477 3.61503
0.01347 4.70993 5.43228 0.61958 0.77255 0.48576 0.95510
1304 2.83560 5.63433 1.13082 1.92164 4.95080 3.37217 3.95129 4.45682 2.63360 3.92719 4.70471 2.72644 4.09921 3.07771 2.98108 2.98686 3.32073 4.01368 6.06470 4.60393 1688 - -
2.68618 4.42225 2.77519 2.73123 3.46354 2.40513 3.72494 3.29354 2.67741 2.69355 4.24690 2.90347 2.73739 3.18146 2.89801 2.37887 2.77519 2.98518 4.58477 3.61503
0.01347 4.70993 5.43228 0.61958 0.77255 0.48576 0.95510
1305 2.29227 4.34898 4.68647 4.10670 3.51964 4.15637 4.52579 1.29113 3.96026 2.41396 3.21400 3.90202 4.51325 4.15813 4.08025 3.29450 3.19991 1.54634 5.10511 3.91514 1689 - -
2.68635 4.42242 2.77537 2.73111 3.46353 2.40530 3.72512 3.29354 2.67681 2.69326 4.24707 2.90347 2.73757 3.18139 2.89789 2.37904 2.77513 2.98517 4.58494 3.61520
0.35207 1.22964 5.43228 0.68241 0.70400 0.48576 0.95510
1306 2.80635 5.08316 3.15647 2.53465 4.35551 2.69509 3.79355 3.78323 2.22594 3.35142 4.14900 3.12047 1.69339 2.92646 2.62539 2.81744 2.94722 3.43554 4.70556 3.93397 1692 - -
2.68633 4.42240 2.77534 2.73132 3.46369 2.40492 3.72509 3.29337 2.67726 2.69370 4.24704 2.90325 2.73754 3.18161 2.89796 2.37896 2.77511 2.98471 4.58492 3.61518
0.37513 1.17627 5.43228 0.49886 0.93452 0.48576 0.95510
1307 2.76673 5.17780 2.83958 2.27444 3.40390 3.55620 3.58750 3.94263 2.34049 3.46604 4.22908 1.84761 3.94885 2.85874 2.63769 2.34945 2.99791 3.54718 4.06097 3.81044 1696 - -
2.68618 4.42225 2.77519 2.73123 3.46354 2.40513 3.72494 3.29354 2.67741 2.69355 4.24690 2.90347 2.73739 3.18146 2.89801 2.37887 2.77519 2.98518 4.58477 3.61503
0.01347 4.70993 5.43228 0.61958 0.77255 0.48576 0.95510
1308 3.81944 5.04082 5.76535 5.20331 3.19953 5.23457 5.58895 1.56349 5.08034 0.73185 3.24893 5.37989 5.32260 5.02429 5.07101 4.62200 4.03661 2.16337 5.67146 4.66252 1697 - -
2.68618 4.42225 2.77519 2.73123 3.46354 2.40513 3.72494 3.29354 2.67741 2.69355 4.24690 2.90347 2.73739 3.18146 2.89801 2.37887 2.77519 2.98518 4.58477 3.61503
0.01347 4.70993 5.43228 0.61958 0.77255 0.48576 0.95510
1309 2.67208 5.12196 2.94164 2.21065 4.40797 3.41547 3.76198 3.14549 1.59901 3.13756 3.89329 3.06444 3.96034 2.77367 2.72949 2.76434 2.99998 2.80211 5.59115 4.22048 1698 - -
2.68618 4.42225 2.77519 2.73123 3.46354 2.40513 3.72494 3.29354 2.67741 2.69355 4.24690 2.90347 2.73739 3.18146 2.89801 2.37887 2.77519 2.98518 4.58477 3.61503
0.01347 4.70993 5.43228 0.61958 0.77255 0.48576 0.95510
1310 2.80600 5.31551 2.57166 2.02403 4.65513 3.37814 3.75789 4.14084 2.16885 3.61790 4.35999 2.13405 3.95756 2.64736 2.91066 2.76471 2.09838 3.70368 5.74879 4.33493 1699 - -
2.68618 4.42225 2.77519 2.73123 3.46354 2.40513 3.72494 3.29354 2.67741 2.69355 4.24690 2.90347 2.73739 3.18146 2.89801 2.37887 2.77519 2.98518 4.58477 3.61503
0.01347 4.70993 5.43228 0.61958 0.77255 0.48576 0.95510
1311 2.77356 4.35213 3.93230 3.22829 3.46861 3.81844 3.42868 1.47462 2.98428 2.36552 3.31988 3.49464 4.19244 3.54196 3.11199 3.08470 2.70949 2.28383 4.95151 3.50030 1700 - -
2.68618 4.42225 2.77519 2.73123 3.46354 2.40513 3.72494 3.29354 2.67741 2.69355 4.24690 2.90347 2.73739 3.18146 2.89801 2.37887 2.77519 2.98518 4.58477 3.61503
0.01347 4.70993 5.43228 0.61958 0.77255 0.48576 0.95510
1312 4.03872 5.27121 5.81500 5.28908 3.42877 5.37074 5.70837 2.43931 5.08815 0.40792 3.19064 5.52467 5.42844 5.05228 5.08899 4.80406 4.26205 2.61064 5.69811 4.69000 1701 - -
2.68618 4.42225 2.77519 2.73123 3.46354 2.40513 3.72494 3.29354 2.67741 2.69355 4.24690 2.90347 2.73739 3.18146 2.89801 2.37887 2.77519 2.98518 4.58477 3.61503

0.01347 4.70993 5.43228 0.61958 0.77255 0.48576 0.95510
1313 2.61447 4.89405 3.21791 2.42716 4.11237 3.26190 3.82552 3.21813 2.48296 2.59373 3.05802 3.16845 4.00541 2.83845 2.30036 2.69772 2.15890 3.07375 5.41088 4.09312 1702 - -
2.68618 4.42225 2.77519 2.73123 3.46354 2.40513 3.72494 3.29354 2.67741 2.69355 4.24690 2.90347 2.73739 3.18146 2.89801 2.37887 2.77519 2.98518 4.58477 3.61503
0.01347 4.70993 5.43228 0.61958 0.77255 0.48576 0.95510
1314 2.65654 5.24642 2.93851 2.32650 4.57715 2.86339 3.40630 4.05251 2.38354 3.54501 3.99605 2.85360 3.94211 2.63790 2.58086 1.61447 3.00303 3.62945 5.68715 4.28579 1703 - -
2.68618 4.42225 2.77519 2.73123 3.46354 2.40513 3.72494 3.29354 2.67741 2.69355 4.24690 2.90347 2.73739 3.18146 2.89801 2.37887 2.77519 2.98518 4.58477 3.61503
0.01347 4.70993 5.43228 0.61958 0.77255 0.48576 0.95510
1315 2.92724 4.31599 4.68696 4.09439 2.67556 4.10284 4.42386 1.11033 3.31898 2.23891 3.36695 4.18833 4.45187 4.09768 4.01291 3.41190 3.16030 2.05654 4.95700 3.46446 1704 - -
2.68618 4.42225 2.77519 2.73123 3.46354 2.40513 3.72494 3.29354 2.67741 2.69355 4.24690 2.90347 2.73739 3.18146 2.89801 2.37887 2.77519 2.98518 4.58477 3.61503
0.01347 4.70993 5.43228 0.61958 0.77255 0.48576 0.95510
1316 2.45014 4.84621 3.37816 2.88823 4.49804 3.27630 4.07660 3.93029 2.69966 3.53195 4.33165 3.32574 1.93142 3.23948 3.33834 1.51619 2.29421 3.50512 5.75329 4.44124 1705 - -
2.68618 4.42225 2.77519 2.73123 3.46354 2.40513 3.72494 3.29354 2.67741 2.69355 4.24690 2.90347 2.73739 3.18146 2.89801 2.37887 2.77519 2.98518 4.58477 3.61503
0.01347 4.70993 5.43228 0.61958 0.77255 0.48576 0.95510
1317 2.51539 5.20918 2.93111 1.87490 4.52714 3.55043 3.46224 3.99425 2.21704 3.05510 3.93485 2.89217 2.40748 2.84619 2.75204 2.74170 2.85496 3.41620 5.14662 4.26461 1706 - -
2.68618 4.42225 2.77519 2.73123 3.46354 2.40513 3.72494 3.29354 2.67741 2.69355 4.24690 2.90347 2.73739 3.18146 2.89801 2.37887 2.77519 2.98518 4.58477 3.61503
0.01347 4.70993 5.43228 0.61958 0.77255 0.48576 0.95510
1318 2.55796 5.25117 2.68473 1.89024 4.04572 3.41493 3.73330 4.06302 2.27834 3.55155 4.29508 2.93616 3.93818 2.83356 1.97306 2.57368 2.70636 3.46692 5.69051 4.28657 1707 - -
2.68618 4.42225 2.77519 2.73123 3.46354 2.40513 3.72494 3.29354 2.67741 2.69355 4.24690 2.90347 2.73739 3.18146 2.89801 2.37887 2.77519 2.98518 4.58477 3.61503
0.01347 4.70993 5.43228 0.61958 0.77255 0.48576 0.95510
1319 2.80003 5.29987 2.80237 1.92393 4.64734 3.56006 3.74465 4.12783 2.08805 3.60083 4.34256 2.84504 3.95712 1.98175 2.20611 2.76168 3.02981 3.43444 5.72552 4.32295 1708 - -
2.68618 4.42225 2.77519 2.73123 3.46354 2.40513 3.72494 3.29354 2.67741 2.69355 4.24690 2.90347 2.73739 3.18146 2.89801 2.37887 2.77519 2.98518 4.58477 3.61503
0.01347 4.70993 5.43228 0.61958 0.77255 0.48576 0.95510
1320 2.78502 4.20180 4.49748 3.89880 3.13924 3.92363 4.23144 2.07167 3.26738 2.27041 3.31654 3.99774 4.28920 3.91009 3.50240 3.22374 3.01701 1.91859 3.72347 1.77963 1709 - -
2.68618 4.42225 2.77519 2.73123 3.46354 2.40513 3.72494 3.29354 2.67741 2.69355 4.24690 2.90347 2.73739 3.18146 2.89801 2.37887 2.77519 2.98518 4.58477 3.61503
0.01347 4.70993 5.43228 0.61958 0.77255 0.48576 0.95510
1321 2.76881 4.76161 3.32307 2.45682 3.59471 3.65307 3.87297 3.18549 2.39024 1.88373 3.64958 3.12078 4.03919 2.78675 2.74413 2.64119 2.79209 2.54441 5.30221 4.01364 1710 - -
2.68618 4.42225 2.77519 2.73123 3.46354 2.40513 3.72494 3.29354 2.67741 2.69355 4.24690 2.90347 2.73739 3.18146 2.89801 2.37887 2.77519 2.98518 4.58477 3.61503
0.01347 4.70993 5.43228 0.61958 0.77255 0.48576 0.95510
1322 2.58628 5.19257 2.82060 2.28208 3.65882 2.96257 3.74390 3.59077 2.12979 3.48341 4.24221 2.98830 3.94574 2.78379 1.92344 2.58163 2.87813 3.34564 5.12443 4.25610 1711 - -
2.68618 4.42225 2.77519 2.73123 3.46354 2.40513 3.72494 3.29354 2.67741 2.69355 4.24690 2.90347 2.73739 3.18146 2.89801 2.37887 2.77519 2.98518 4.58477 3.61503
0.01347 4.70993 5.43228 0.61958 0.77255 0.48576 0.95510
1323 2.83376 4.35834 4.13041 3.53871 3.43046 3.90203 4.17265 2.60277 3.01893 2.07467 1.36607 3.80828 4.26712 3.66063 3.07515 3.18271 3.06320 2.59401 4.94942 3.39487 1712 - -
2.68618 4.42225 2.77519 2.73123 3.46354 2.40513 3.72494 3.29354 2.67741 2.69355 4.24690 2.90347 2.73739 3.18146 2.89801 2.37887 2.77519 2.98518 4.58477 3.61503
0.01347 4.70993 5.43228 0.61958 0.77255 0.48576 0.95510
1324 2.81445 4.84268 3.34157 2.77034 4.00640 3.67779 3.65368 3.25819 2.54989 2.77271 3.92641 3.25686 4.06104 2.06255 2.13296 2.89886 3.04178 3.15637 3.68915 2.36170 1713 - -

2.68619 4.42226 2.77520 2.73124 3.46355 2.40514 3.72495 3.29355 2.67742 2.69356 4.24691 2.90348 2.73740 3.18147 2.89802 2.37876 2.77520 2.98519 4.58478 3.61504
0.04935 3.12859 5.43228 0.41022 1.08917 0.48576 0.95510
1325 2.60650 5.21091 3.04529 2.04227 4.52933 3.04369 3.74173 3.99578 2.24045 3.34985 3.38900 2.67505 3.94563 2.84775 1.99633 2.54579 3.00006 3.58722 5.65920 3.64071 1715 - -
2.68618 4.42225 2.77519 2.73123 3.46354 2.40513 3.72494 3.29354 2.67741 2.69355 4.24690 2.90347 2.73739 3.18146 2.89801 2.37887 2.77519 2.98518 4.58477 3.61503
0.01347 4.70993 5.43228 0.61958 0.77255 0.48576 0.95510
1326 2.68495 5.15534 3.10188 2.32894 4.45196 3.43094 3.76871 3.28742 2.50121 3.22941 4.21221 2.21585 3.97623 2.75199 1.66515 2.78596 3.02345 3.38765 5.61426 4.24741 1716 - -
2.68618 4.42225 2.77519 2.73123 3.46354 2.40513 3.72494 3.29354 2.67741 2.69355 4.24690 2.90347 2.73739 3.18146 2.89801 2.37887 2.77519 2.98518 4.58477 3.61503
0.01347 4.70993 5.43228 0.61958 0.77255 0.48576 0.95510
1327 3.21376 4.54580 5.03223 4.46937 3.66108 3.91445 4.91219 2.11931 4.32467 1.77460 3.17924 4.59523 4.81374 4.51316 4.43545 3.83536 3.45598 0.86620 5.41053 4.23542 1717 - -
2.68618 4.42225 2.77519 2.73123 3.46354 2.40513 3.72494 3.29354 2.67741 2.69355 4.24690 2.90347 2.73739 3.18146 2.89801 2.37887 2.77519 2.98518 4.58477 3.61503
0.01347 4.70993 5.43228 0.61958 0.77255 0.48576 0.95510
1328 2.47019 4.73247 3.36488 2.65268 3.91309 3.66961 3.41292 2.49303 1.98293 2.05636 3.67486 3.27963 4.05437 2.98666 2.85402 2.89085 3.00700 2.84562 5.27705 3.99757 1718 - -
2.68621 4.42228 2.77516 2.73120 3.46357 2.40515 3.72497 3.29357 2.67743 2.69358 4.24692 2.90338 2.73720 3.18149 2.89803 2.37889 2.77522 2.98521 4.58480 3.61506
0.06187 2.88912 5.43228 0.65567 0.73209 0.48576 0.95510
1329 2.68610 5.26493 2.83466 1.98389 4.60474 3.54356 3.73062 4.08610 1.64759 3.56733 3.70240 2.77967 3.93715 2.60381 2.66980 2.51710 2.99997 3.48692 5.70013 4.13107 1721 - -
2.68618 4.42225 2.77519 2.73123 3.46354 2.40513 3.72494 3.29354 2.67741 2.69355 4.24690 2.90347 2.73739 3.18146 2.89801 2.37887 2.77519 2.98518 4.58477 3.61503
0.01347 4.70993 5.43228 0.61958 0.77255 0.48576 0.95510
1330 2.85867 4.39949 4.92183 4.32760 3.22174 4.26120 4.60871 2.20146 4.15212 1.61068 2.90839 4.38741 4.58814 4.28806 4.20151 3.58087 3.27771 1.16561 5.08021 3.49268 1722 - -
2.68618 4.42225 2.77519 2.73123 3.46354 2.40513 3.72494 3.29354 2.67741 2.69355 4.24690 2.90347 2.73739 3.18146 2.89801 2.37887 2.77519 2.98518 4.58477 3.61503
0.01347 4.70993 5.43228 0.61958 0.77255 0.48576 0.95510
1331 2.70924 4.91930 3.22026 2.52513 4.14396 3.62176 3.82328 2.57548 2.35780 2.90308 3.68512 3.16835 4.00958 2.28604 2.01595 2.64139 3.00924 3.24362 3.91753 4.11104 1723 - -
2.68618 4.42225 2.77519 2.73123 3.46354 2.40513 3.72494 3.29354 2.67741 2.69355 4.24690 2.90347 2.73739 3.18146 2.89801 2.37887 2.77519 2.98518 4.58477 3.61503
0.01347 4.70993 5.43228 0.61958 0.77255 0.48576 0.95510
1332 2.84946 4.46713 3.14654 2.57419 4.58942 3.45652 3.58286 4.03848 2.11824 3.53806 4.30671 3.10242 2.41996 2.43273 1.69055 2.71519 3.07283 3.64155 5.67855 4.31797 1724 - -
2.68618 4.42225 2.77519 2.73123 3.46354 2.40513 3.72494 3.29354 2.67741 2.69355 4.24690 2.90347 2.73739 3.18146 2.89801 2.37887 2.77519 2.98518 4.58477 3.61503
0.01347 4.70993 5.43228 0.61958 0.77255 0.48576 0.95510
1333 2.53546 4.49492 3.16378 2.60400 4.24751 3.21967 1.86767 3.66782 2.35203 3.11981 4.06929 2.99364 3.98989 2.94244 2.39569 2.80367 3.00688 3.16828 5.49481 3.38190 1725 - -
2.68618 4.42225 2.77519 2.73123 3.46354 2.40513 3.72494 3.29354 2.67741 2.69355 4.24690 2.90347 2.73739 3.18146 2.89801 2.37887 2.77519 2.98518 4.58477 3.61503
0.01347 4.70993 5.43228 0.61958 0.77255 0.48576 0.95510
1334 3.37375 4.69824 5.22532 4.63344 1.15704 4.59330 4.84058 2.25450 4.46185 1.45688 2.95488 4.70960 4.84313 4.50667 4.47529 3.92337 3.59449 2.41830 5.15409 3.64324 1726 - -
2.68618 4.42225 2.77519 2.73123 3.46354 2.40513 3.72494 3.29354 2.67741 2.69355 4.24690 2.90347 2.73739 3.18146 2.89801 2.37887 2.77519 2.98518 4.58477 3.61503
0.01347 4.70993 5.43228 0.61958 0.77255 0.48576 0.95510
1335 2.77870 4.52354 3.17717 2.05811 3.66302 3.74853 3.99873 2.66499 3.00812 1.94709 2.92863 3.46705 4.12824 3.18649 3.37248 2.99047 2.92129 2.11292 5.10564 3.86675 1727 - -
2.68618 4.42225 2.77519 2.73123 3.46354 2.40513 3.72494 3.29354 2.67741 2.69355 4.24690 2.90347 2.73739 3.18146 2.89801 2.37887 2.77519 2.98518 4.58477 3.61503
0.01347 4.70993 5.43228 0.61958 0.77255 0.48576 0.95510

1336 3.16686 4.51313 4.91399 4.38085 2.13469 4.26402 4.13718 2.79345 4.18492 2.33914 3.59601 4.29212 4.60757 4.25881 4.19596 3.58371 3.39542 1.78859 2.55669 1.51619 1728 - -
2.68618 4.42225 2.77519 2.73123 3.46354 2.40513 3.72494 3.29354 2.67741 2.69355 4.24690 2.90347 2.73739 3.18146 2.89801 2.37887 2.77519 2.98518 4.58477 3.61503
0.01347 4.70993 5.43228 0.61958 0.77255 0.48576 0.95510

1337 2.39695 5.22881 2.83564 2.47834 4.00573 3.06681 2.84102 4.02602 2.48126 3.52574 4.27534 1.94862 3.63305 2.76999 2.50633 2.55981 2.84324 3.60885 5.67402 4.27546 1729 - -
2.68618 4.42225 2.77519 2.73123 3.46354 2.40513 3.72494 3.29354 2.67741 2.69355 4.24690 2.90347 2.73739 3.18146 2.89801 2.37887 2.77519 2.98518 4.58477 3.61503
0.01347 4.70993 5.43228 0.61958 0.77255 0.48576 0.95510

1338 2.56939 5.23533 2.29938 2.47459 3.72578 3.54571 3.22949 4.03774 2.21574 3.53337 4.28049 2.82680 3.49266 2.72422 2.65298 1.98056 2.99688 3.32091 5.67821 4.00819 1730 - -
2.68593 4.42257 2.77531 2.73075 3.46344 2.40525 3.72473 3.29351 2.67747 2.69348 4.24722 2.90379 2.73771 3.18116 2.89809 2.37891 2.77531 2.98525 4.58382 3.61487
0.08942 2.51121 5.43228 2.22425 0.11446 0.48576 0.95510

1339 2.84563 5.24617 2.87595 2.45489 4.55481 3.35037 3.52331 4.01112 2.09753 3.38957 4.31046 3.06788 1.54448 2.92460 3.03288 2.52380 3.08047 3.62013 5.70497 4.32137 1755 - -
2.68618 4.42225 2.77519 2.73123 3.46354 2.40513 3.72494 3.29354 2.67741 2.69355 4.24690 2.90347 2.73739 3.18146 2.89801 2.37887 2.77519 2.98518 4.58477 3.61503
0.01347 4.70993 5.43228 0.61958 0.77255 0.48576 0.95510

1340 1.94651 5.05667 3.11657 2.55996 4.32304 3.13211 3.53047 3.75584 2.49398 2.97898 3.86284 2.58741 2.56403 2.91008 2.86104 2.36364 2.99870 3.26191 5.54225 4.18622 1756 - -
2.68618 4.42225 2.77519 2.73123 3.46354 2.40513 3.72494 3.29354 2.67741 2.69355 4.24690 2.90347 2.73739 3.18146 2.89801 2.37887 2.77519 2.98518 4.58477 3.61503
0.05131 4.70993 3.19398 0.61958 0.77255 0.48576 0.95510

1341 2.36240 5.23321 3.02773 2.21636 4.56498 3.54159 3.72608 4.03770 1.66530 3.53031 4.27855 2.89855 3.93447 2.44940 2.68552 2.39187 2.99510 3.30611 5.67164 3.97055 1757 - -
2.68618 4.42225 2.77519 2.73123 3.46354 2.40513 3.72494 3.29354 2.67741 2.69355 4.24690 2.90347 2.73739 3.18146 2.89801 2.37887 2.77519 2.98518 4.58477 3.61503
0.01398 4.67261 5.39495 0.61958 0.77255 0.51538 0.90950

1342 2.75861 5.24569 2.80736 2.26891 4.20405 3.22069 3.24682 4.06024 2.04892 3.54669 4.28899 2.91568 2.94916 2.82297 2.03621 2.55967 2.91136 3.22756 5.68389 4.27918 1758 - -
2.68618 4.42225 2.77519 2.73123 3.46354 2.40513 3.72494 3.29354 2.67741 2.69355 4.24690 2.90347 2.73739 3.18146 2.89801 2.37887 2.77519 2.98518 4.58477 3.61503
0.01402 4.67036 5.39270 0.61958 0.77255 0.53368 0.88296

1343 2.75469 4.03959 2.91036 2.94688 2.08279 2.73605 3.98004 2.98960 2.89964 2.38588 3.35916 3.31907 4.10849 3.30550 3.36067 2.72696 2.98644 2.76295 5.06805 2.77589 1759 - -
2.68651 4.42258 2.77515 2.73040 3.46314 2.40511 3.72527 3.29324 2.67720 2.69368 4.24683 2.90359 2.73731 3.18161 2.89791 2.37915 2.77521 2.98551 4.58510 3.61522
0.26940 1.46268 5.39270 1.47723 0.25912 0.45692 1.00302

1344 3.26333 5.72959 0.72268 2.52850 5.08839 2.68038 4.17731 4.65403 3.20923 4.16884 5.01619 3.10377 4.22359 3.35168 3.80182 2.95378 3.57523 4.21242 6.31211 4.04279 1767 - -
2.68618 4.42225 2.77519 2.73123 3.46354 2.40513 3.72494 3.29354 2.67741 2.69355 4.24690 2.90347 2.73739 3.18146 2.89801 2.37887 2.77519 2.98518 4.58477 3.61503
0.01368 4.69427 5.41662 0.61958 0.77255 0.48576 0.95510

1345 1.60897 4.28408 4.25545 3.67383 3.40764 3.90694 4.23148 2.26028 3.55896 2.37420 3.04434 3.88966 3.77482 3.78578 3.74985 2.86400 3.04070 1.71641 4.92778 3.73406 1768 - -
2.68618 4.42225 2.77519 2.73123 3.46354 2.40513 3.72494 3.29354 2.67741 2.69355 4.24690 2.90347 2.73739 3.18146 2.89801 2.37887 2.77519 2.98518 4.58477 3.61503
0.01368 4.69427 5.41662 0.61958 0.77255 0.48576 0.95510

1346 2.90221 4.32329 4.52545 3.53061 1.32679 4.05068 4.36793 2.48172 3.79326 2.27429 3.19237 4.09022 4.40524 3.98819 3.92502 3.35257 2.88329 1.84641 4.95614 3.76217 1769 - -
2.68618 4.42225 2.77519 2.73123 3.46354 2.40513 3.72494 3.29354 2.67741 2.69355 4.24690 2.90347 2.73739 3.18146 2.89801 2.37887 2.77519 2.98518 4.58477 3.61503
0.01368 4.69427 5.41662 0.61958 0.77255 0.48576 0.95510

1347 2.56084 5.25424 2.17439 2.36410 4.58226 3.54527 1.97910 4.05739 2.37293 3.55150 4.30062 2.61635 3.94585 2.69041 2.93065 2.74891 3.01323 3.63601 4.34097 4.29315 1770 - -
2.68618 4.42225 2.77519 2.73123 3.46354 2.40513 3.72494 3.29354 2.67741 2.69355 4.24690 2.90347 2.73739 3.18146 2.89801 2.37887 2.77519 2.98518 4.58477 3.61503

0.01368 4.69427 5.41662 0.61958 0.77255 0.48576 0.95510

1348 2.76725 4.30298 4.03840 3.19275 3.41348 3.56828 3.53533 2.29638 3.36364 2.31035 1.71008 3.73885 4.20759 3.20417 3.60437 3.10923 2.70079 2.21492 4.90787 3.70638 1771 - -

2.68618 4.42225 2.77519 2.73123 3.46354 2.40513 3.72494 3.29354 2.67741 2.69355 4.24690 2.90347 2.73739 3.18146 2.89801 2.37887 2.77519 2.98518 4.58477 3.61503

0.01368 4.69427 5.41662 0.61958 0.77255 0.48576 0.95510

1349 2.18183 4.33125 4.49395 3.91579 3.48905 4.05176 4.41358 1.23317 3.78721 2.38229 3.27475 4.08747 4.42153 4.00165 3.94580 3.06721 2.61854 2.07681 5.05122 3.85954 1772 - -

2.68618 4.42225 2.77519 2.73123 3.46354 2.40513 3.72494 3.29354 2.67741 2.69355 4.24690 2.90347 2.73739 3.18146 2.89801 2.37887 2.77519 2.98518 4.58477 3.61503

0.01368 4.69427 5.41662 0.61958 0.77255 0.48576 0.95510

1350 3.16342 4.50704 5.07430 4.46841 3.09644 4.36923 4.68556 2.07603 4.27962 1.08976 2.78140 4.51403 4.66166 4.35585 4.28860 3.69172 3.38850 2.02830 2.69014 3.94936 1773 - -

2.68618 4.42225 2.77519 2.73123 3.46354 2.40513 3.72494 3.29354 2.67741 2.69355 4.24690 2.90347 2.73739 3.18146 2.89801 2.37887 2.77519 2.98518 4.58477 3.61503

0.01368 4.69427 5.41662 0.61958 0.77255 0.48576 0.95510

1351 2.69715 5.25709 2.54799 2.24078 4.59540 3.53934 2.03897 4.07563 2.24197 3.55872 4.29991 2.87592 3.93318 2.43902 2.62823 2.66680 2.99592 3.64419 4.70947 4.00670 1774 - -

2.68618 4.42225 2.77519 2.73123 3.46354 2.40513 3.72494 3.29354 2.67741 2.69355 4.24690 2.90347 2.73739 3.18146 2.89801 2.37887 2.77519 2.98518 4.58477 3.61503

0.40455 4.69427 1.12832 0.61958 0.77255 0.48576 0.95510

1352 2.22593 4.25309 3.04725 2.41307 4.07685 2.60180 3.67949 3.49257 2.39297 3.09729 3.55223 3.00789 3.86412 2.60806 2.75403 2.49685 2.60575 3.17027 5.34254 4.00953 1775 - -

2.68618 4.42225 2.77519 2.73123 3.46354 2.40513 3.72494 3.29354 2.67741 2.69355 4.24690 2.90347 2.73739 3.18146 2.89801 2.37887 2.77519 2.98518 4.58477 3.61503

0.02016 4.30988 5.03223 0.61958 0.77255 0.99429 0.46201

1353 3.29145 4.57857 5.09974 4.54344 3.40249 4.64382 5.04626 1.85666 4.42277 1.29817 2.79150 4.72540 4.86648 4.54488 4.52420 3.99839 3.52482 1.27342 5.39529 4.29374 1776 - -

2.68618 4.42225 2.77519 2.73123 3.46354 2.40513 3.72494 3.29354 2.67741 2.69355 4.24690 2.90347 2.73739 3.18146 2.89801 2.37887 2.77519 2.98518 4.58477 3.61503

0.02016 4.30988 5.03223 0.61958 0.77255 0.99429 0.46201

1354 2.22271 4.30796 3.58400 3.01440 3.01967 3.62959 3.22860 2.62658 2.85584 2.03851 3.41339 3.39840 4.00747 2.91551 3.29177 2.61629 2.77582 2.57739 4.29797 3.66783 1777 - -

2.68618 4.42225 2.77519 2.73123 3.46354 2.40513 3.72494 3.29354 2.67741 2.69355 4.24690 2.90347 2.73739 3.18146 2.89801 2.37887 2.77519 2.98518 4.58477 3.61503

0.02016 4.30988 5.03223 0.61958 0.77255 0.99429 0.46201

1355 2.65561 5.11468 2.63083 2.08120 3.54702 3.19734 3.18191 3.91107 2.09185 3.40993 4.16153 2.90480 3.82510 2.61861 2.34048 2.62680 2.88653 3.49513 5.55696 4.16062 1778 - -

2.68618 4.42225 2.77519 2.73123 3.46354 2.40513 3.72494 3.29354 2.67741 2.69355 4.24690 2.90347 2.73739 3.18146 2.89801 2.37887 2.77519 2.98518 4.58477 3.61503

0.02016 4.30988 5.03223 0.61958 0.77255 0.99429 0.46201

1356 3.29637 5.42988 3.75925 3.00077 4.95928 3.88441 3.73443 4.26653 1.02382 3.64101 4.50866 3.38096 4.22208 2.71191 1.58931 3.26968 3.43115 3.94189 5.63259 4.50299 1779 - -

2.68618 4.42225 2.77519 2.73123 3.46354 2.40513 3.72494 3.29354 2.67741 2.69355 4.24690 2.90347 2.73739 3.18146 2.89801 2.37887 2.77519 2.98518 4.58477 3.61503

0.02016 4.30988 5.03223 0.61958 0.77255 0.99429 0.46201

1357 2.75199 4.40633 4.88117 4.31752 3.11774 4.37997 4.77691 1.59166 4.18935 1.50697 3.28544 4.46329 4.66984 4.35336 4.30027 3.71643 3.32069 1.46871 5.24048 4.08464 1780 - -

2.68618 4.42225 2.77519 2.73123 3.46354 2.40513 3.72494 3.29354 2.67741 2.69355 4.24690 2.90347 2.73739 3.18146 2.89801 2.37887 2.77519 2.98518 4.58477 3.61503

0.02016 4.30988 5.03223 0.61958 0.77255 0.99429 0.46201

1358 2.47488 5.04062 2.84135 2.19073 4.34103 3.43584 3.13918 3.51722 2.31277 3.32652 4.09372 2.73211 2.78135 2.74234 2.68892 2.50205 2.71474 3.18255 5.50098 4.11908 1781 - -

2.68618 4.42225 2.77519 2.73123 3.46354 2.40513 3.72494 3.29354 2.67741 2.69355 4.24690 2.90347 2.73739 3.18146 2.89801 2.37887 2.77519 2.98518 4.58477 3.61503

0.02016 4.30988 5.03223 0.61958 0.77255 0.99429 0.46201

1359 2.64459 5.03480 2.76800 2.26142 4.33323 2.96831 3.19615 3.78722 2.30319 2.87951 3.53031 2.54157 3.82967 2.74389 2.55023 2.47626 2.87572 3.40017 5.49609 3.85223 1782 - -

2.68618 4.42225 2.77519 2.73123 3.46354 2.40513 3.72494 3.29354 2.67741 2.69355 4.24690 2.90347 2.73739 3.18146 2.89801 2.37887 2.77519 2.98518 4.58477 3.61503
0.02016 4.30988 5.03223 0.61958 0.77255 0.99429 0.46201
1360 2.63502 4.49735 2.83276 2.69039 3.26365 3.56045 3.79476 2.61217 2.72880 2.39077 3.59247 3.09425 3.94388 2.90686 2.85683 2.60577 2.72670 2.31593 5.05917 3.79743 1783 - -
2.68618 4.42225 2.77519 2.73123 3.46354 2.40513 3.72494 3.29354 2.67741 2.69355 4.24690 2.90347 2.73739 3.18146 2.89801 2.37887 2.77519 2.98518 4.58477 3.61503
0.02016 4.30988 5.03223 0.61958 0.77255 0.99429 0.46201
1361 2.71312 5.04825 3.03279 2.37932 4.35531 3.49284 3.65149 3.78907 1.83147 3.32133 4.10766 2.98848 3.22656 2.58614 2.27594 2.61929 2.93677 2.68859 5.49056 4.14505 1784 - -
2.68618 4.42225 2.77519 2.73123 3.46354 2.40513 3.72494 3.29354 2.67741 2.69355 4.24690 2.90347 2.73739 3.18146 2.89801 2.37887 2.77519 2.98518 4.58477 3.61503
0.05428 4.25201 3.25448 0.61958 0.77255 0.99429 0.46201
1362 2.61104 4.42849 3.35125 2.57547 3.39976 3.55170 3.78895 2.95091 2.37233 2.33771 3.04864 3.22786 3.93350 2.92177 2.78159 2.78541 2.84231 2.54292 3.65564 3.51404 1785 - -
2.68625 4.42246 2.77534 2.73093 3.46361 2.40533 3.72488 3.29325 2.67755 2.69370 4.24490 2.90346 2.73760 3.18167 2.89718 2.37894 2.77526 2.98532 4.58498 3.61524
0.84278 0.62772 3.33312 0.79618 0.59975 0.25574 1.48874
1363 2.73674 5.01774 3.07530 2.23120 4.28397 3.54377 3.74702 3.39431 2.46153 3.28974 4.08533 2.95556 3.93852 2.56061 2.99222 1.63179 2.72767 2.88085 5.50545 4.15194 1789 - -
2.68618 4.42225 2.77519 2.73123 3.46354 2.40513 3.72494 3.29354 2.67741 2.69355 4.24690 2.90347 2.73739 3.18146 2.89801 2.37887 2.77519 2.98518 4.58477 3.61503
0.15406 4.59385 2.01991 0.61958 0.77255 0.52088 0.90139
1364 2.43428 4.20650 4.02221 3.44405 3.13864 2.85354 4.07537 1.59024 3.34302 2.34202 3.32822 3.70124 4.14222 3.58738 3.36774 2.92444 2.81372 2.22923 4.83151 3.63259 1790 - -
2.68618 4.42225 2.77519 2.73123 3.46354 2.40513 3.72494 3.29354 2.67741 2.69355 4.24690 2.90347 2.73739 3.18146 2.89801 2.37887 2.77519 2.98518 4.58477 3.61503
0.01738 4.45716 5.17951 0.61958 0.77255 0.72929 0.65827
1365 2.70250 4.41912 3.38538 3.01172 3.31756 3.67693 3.90856 2.91747 2.58987 2.62582 3.52233 3.41180 4.05544 3.25642 3.02109 2.92512 2.93332 2.38248 1.86864 3.37828 1791 - -
2.68618 4.42225 2.77519 2.73123 3.46354 2.40513 3.72494 3.29354 2.67741 2.69355 4.24690 2.90347 2.73739 3.18146 2.89801 2.37887 2.77519 2.98518 4.58477 3.61503
0.01768 4.43973 5.16208 0.61958 0.77255 0.39992 1.10980
1366 2.72080 4.53129 3.16744 2.75459 3.46131 3.66492 3.19482 2.90850 2.87128 2.14637 3.62944 2.78670 4.04690 3.18769 3.05934 2.89879 2.77738 2.08249 5.10047 3.00010 1792 - -
2.68618 4.42225 2.77519 2.73123 3.46354 2.40513 3.72494 3.29354 2.67741 2.69355 4.24690 2.90347 2.73739 3.18146 2.89801 2.37887 2.77519 2.98518 4.58477 3.61503
0.01561 4.56352 5.28587 0.61958 0.77255 0.52088 0.90139
1367 2.83489 5.24507 3.07329 2.30326 4.58784 3.38455 3.47856 4.03607 2.27832 3.52682 4.29859 2.47180 3.97516 2.84660 1.47796 2.71513 3.05731 3.43687 5.65855 4.30104 1793 - -
2.68618 4.42225 2.77519 2.73123 3.46354 2.40513 3.72494 3.29354 2.67741 2.69355 4.24690 2.90347 2.73739 3.18146 2.89801 2.37887 2.77519 2.98518 4.58477 3.61503
0.01561 4.56352 5.28587 0.61958 0.77255 0.46441 0.99022
1368 2.72343 5.04532 2.96717 2.37459 3.98529 3.52966 3.54795 3.45976 2.44221 3.17338 4.10524 2.83497 3.74901 2.69305 1.85065 2.48843 2.64006 3.39764 4.57859 3.69763 1794 - -
2.68656 4.42288 2.77491 2.73130 3.46270 2.40551 3.72557 3.29385 2.67704 2.69358 4.24699 2.90356 2.73783 3.18146 2.89728 2.37911 2.77552 2.98502 4.58287 3.61348
0.43278 1.06037 5.30529 1.89130 0.16355 0.48576 0.95510
1369 2.60923 4.61594 3.35267 2.55384 3.78483 3.20231 3.45129 3.16260 2.57360 1.85764 3.70850 3.25747 3.44909 3.09219 2.94432 2.84932 2.94429 2.43160 5.17071 3.29343 1811 - -
2.68618 4.42225 2.77519 2.73123 3.46354 2.40513 3.72494 3.29354 2.67741 2.69355 4.24690 2.90347 2.73739 3.18146 2.89801 2.37887 2.77519 2.98518 4.58477 3.61503
0.22177 4.54789 1.66966 0.61958 0.77255 0.48576 0.95510
1370 2.67438 4.61046 3.24979 2.47813 2.87593 3.56339 3.77979 3.15443 2.68596 2.71200 3.69977 1.86243 3.95286 3.02098 3.10166 2.79423 2.90685 2.66127 5.10066 3.54217 1812 - -
2.68618 4.42231 2.77525 2.73104 3.46360 2.40510 3.72500 3.29360 2.67741 2.69361 4.24696 2.90352 2.73739 3.18124 2.89793 2.37893 2.77519 2.98524 4.58483 3.61509
0.09863 2.43464 5.06791 1.12774 0.39121 0.76992 0.62185

1371 2.42414 4.38640 3.73669 3.18039 3.53501 3.75174 4.05601 2.51152 3.08127 2.31995 3.47565 3.55313 4.13896 2.53477 3.39783 3.02607 2.98430 1.50094 5.05451 3.83052 1817 - -
2.68618 4.42225 2.77519 2.73123 3.46354 2.40513 3.72494 3.29354 2.67741 2.69355 4.24690 2.90347 2.73739 3.18146 2.89801 2.37887 2.77519 2.98518 4.58477 3.61503
0.01983 4.32613 5.04847 0.61958 0.77255 0.76992 0.62185
1372 2.55358 5.06711 2.79963 2.29697 4.37527 3.17178 3.63279 3.31167 1.76708 3.20623 4.11897 2.81845 3.83734 2.64803 2.62310 2.64125 2.78849 3.43801 5.52033 4.13814 1818 - -
2.68618 4.42225 2.77519 2.73123 3.46354 2.40513 3.72494 3.29354 2.67741 2.69355 4.24690 2.90347 2.73739 3.18146 2.89801 2.37887 2.77519 2.98518 4.58477 3.61503
0.02034 4.30137 5.02372 0.61958 0.77255 0.76992 0.62185
1373 2.54220 4.21718 3.75972 3.18472 2.99595 3.29318 3.94244 1.92310 2.92076 2.10802 3.32685 3.51235 4.04163 3.18632 3.12020 2.77016 2.85956 2.47847 4.81508 3.40698 1819 - -
2.68618 4.42225 2.77519 2.73123 3.46354 2.40513 3.72494 3.29354 2.67741 2.69355 4.24690 2.90347 2.73739 3.18146 2.89801 2.37887 2.77519 2.98518 4.58477 3.61503
0.02169 4.23761 4.95996 0.61958 0.77255 0.76992 0.62185
1374 2.66165 4.91126 3.03268 2.46854 4.18263 3.28518 3.05775 3.60406 2.28169 3.05155 3.98234 2.98618 3.46858 2.78562 1.95819 2.57941 2.72382 3.05365 5.38978 4.05506 1820 - -
2.68618 4.42225 2.77519 2.73123 3.46354 2.40513 3.72494 3.29354 2.67741 2.69355 4.24690 2.90347 2.73739 3.18146 2.89801 2.37887 2.77519 2.98518 4.58477 3.61503
0.02344 4.16085 4.88319 0.61958 0.77255 0.44055 1.03194
1375 2.66133 5.02628 2.94976 1.80086 4.33235 3.03645 3.65472 3.78158 2.41259 3.24949 4.09703 2.94667 3.20194 2.77347 2.61385 2.32685 2.79687 3.13186 5.50490 4.13046 1821 - -
2.68618 4.42225 2.77519 2.73123 3.46354 2.40513 3.72494 3.29354 2.67741 2.69355 4.24690 2.90347 2.73739 3.18146 2.89801 2.37887 2.77519 2.98518 4.58477 3.61503
0.02083 4.27750 4.99985 0.61958 0.77255 0.57069 0.83272
1376 2.51254 4.26694 3.27953 2.94892 3.04400 3.63278 3.90149 2.74701 3.00580 2.47380 3.37592 3.27157 4.01180 3.29135 3.32219 2.68251 2.85512 1.83078 3.06791 3.63322 1822 - -
2.68635 4.42242 2.77487 2.73134 3.46288 2.40526 3.72511 3.29371 2.67746 2.69366 4.24680 2.90363 2.73726 3.18158 2.89784 2.37874 2.77530 2.98524 4.58419 3.61495
0.87122 1.15745 1.31948 1.02548 0.44414 0.43610 1.04003
1377 2.57887 4.76964 2.73033 2.34418 4.01242 2.92213 3.37290 3.16019 2.42166 2.91941 3.85191 2.92453 2.79640 2.76891 2.88257 2.61368 2.71713 3.10616 5.28133 3.94704 1829 - -
2.68618 4.42225 2.77519 2.73123 3.46354 2.40513 3.72494 3.29354 2.67741 2.69355 4.24690 2.90347 2.73739 3.18146 2.89801 2.37887 2.77519 2.98518 4.58477 3.61503
0.02979 3.92439 4.64674 0.61958 0.77255 0.77645 0.61625
1378 2.53627 3.69645 3.41566 2.84620 3.40192 3.31253 3.76539 2.63599 2.58938 2.16057 3.15272 3.24983 3.43294 3.09458 2.98806 2.75886 2.76924 2.52821 4.84497 3.61145 1830 - -
2.68618 4.42225 2.77519 2.73123 3.46354 2.40513 3.72494 3.29354 2.67741 2.69355 4.24690 2.90347 2.73739 3.18146 2.89801 2.37887 2.77519 2.98518 4.58477 3.61503
0.02979 3.92439 4.64674 0.61958 0.77255 0.77645 0.61625
1379 2.68977 4.91131 2.89729 2.43296 4.23370 3.40160 3.60148 3.64826 2.15661 3.20219 4.02490 2.67646 2.45351 2.63610 2.29642 2.70002 2.92055 3.30725 5.38198 4.07350 1831 - -
2.68618 4.42225 2.77519 2.73123 3.46354 2.40513 3.72494 3.29354 2.67741 2.69355 4.24690 2.90347 2.73739 3.18146 2.89801 2.37887 2.77519 2.98518 4.58477 3.61503
0.02979 3.92439 4.64674 0.61958 0.77255 0.77645 0.61625
1380 2.55380 3.15199 2.86755 2.43217 3.79847 2.94146 3.65496 3.18671 2.50598 2.70774 3.69146 3.00085 3.80822 2.53131 2.93269 2.64290 2.79535 2.90326 5.13616 3.84121 1832 - -
2.68618 4.42225 2.77519 2.73123 3.46354 2.40513 3.72494 3.29354 2.67741 2.69355 4.24690 2.90347 2.73739 3.18146 2.89801 2.37887 2.77519 2.98518 4.58477 3.61503
0.02979 3.92439 4.64674 0.61958 0.77255 0.77645 0.61625
1381 2.69406 5.10428 2.31036 1.85223 4.40621 3.27525 3.62783 3.87052 2.47498 3.40418 4.20531 2.76180 3.37855 2.58555 2.98149 2.16432 2.94788 3.48146 5.58775 4.17776 1833 - -
2.68632 4.41954 2.77527 2.73168 3.46398 2.40524 3.72483 3.29326 2.67745 2.69342 4.24746 2.90339 2.73790 3.18149 2.89817 2.37815 2.77485 2.98568 4.58318 3.61560
0.49064 0.94736 * 3.18104 0.04243 0.00000 *
//

HMMER3/b [3.0 | March 2010]

NAME Homogalacturonan α-1,4-galacturonosyltransferases

LENG 761

ALPH amino

RF no

CS no

MAP yes

DATE Mon Aug 8 15:42:13 2016

NSEQ 41

EFFN 4.501892

CKSUM 266190659

STATS LOCAL MSV -12.0982 0.69635

STATS LOCAL VITERBI -13.7066 0.69635

STATS LOCAL FORWARD -6.2090 0.69635

HMM A C D E F G H I K L M N P Q R S T V W Y

m->m m->i m->d i->m i->i d->m d->d

COMPO 2.59529 4.18896 2.98552 2.76704 3.20696 2.95016 3.65712 2.88393 2.68400 2.38064 3.67193 3.05569 3.23570 3.11768 2.96558 2.56612 2.84603 2.64015 4.23131 3.40799

2.68537 4.42243 2.77537 2.73141 3.46372 2.40531 3.72512 3.29372 2.67759 2.69373 4.24551 2.90323 2.73688 3.18164 2.89819 2.37880 2.77537 2.98536 4.58495 3.61521

0.51173 1.86333 1.40491 1.70314 0.20103 0.00000 *

1 1.91281 4.25964 3.60946 3.14945 3.47462 3.42022 4.06036 2.49289 3.02818 2.25832 2.42519 3.45039 3.95110 3.38655 3.32038 2.79364 2.86725 2.33164 5.09212 3.86246 9 - -

2.68618 4.42225 2.77519 2.73123 3.46354 2.40513 3.72494 3.29354 2.67741 2.69355 4.24690 2.90347 2.73739 3.18146 2.89801 2.37887 2.77519 2.98518 4.58477 3.61503

0.03384 3.79868 4.52103 0.61958 0.77255 0.71972 0.66726

2 2.56863 4.31483 3.46483 3.03289 3.50561 3.42418 3.99153 2.00009 2.93571 2.38034 3.48531 3.37842 2.39921 3.30917 3.24445 2.80551 2.88786 2.37664 5.07481 3.80910 10 - -

2.68618 4.42225 2.77519 2.73123 3.46354 2.40513 3.72494 3.29354 2.67741 2.69355 4.24690 2.90347 2.73739 3.18146 2.89801 2.37887 2.77519 2.98518 4.58477 3.61503

0.03384 3.79868 4.52103 0.61958 0.77255 0.71972 0.66726

3 2.29736 4.22848 3.17257 2.85320 3.99501 3.01895 3.95748 3.28433 2.84711 3.02901 3.94261 3.14976 2.34281 3.21275 3.17469 2.46062 1.87160 2.90797 5.36476 4.12023 11 - -

2.68618 4.42225 2.77519 2.73123 3.46354 2.40513 3.72494 3.29354 2.67741 2.69355 4.24690 2.90347 2.73739 3.18146 2.89801 2.37887 2.77519 2.98518 4.58477 3.61503

0.03384 3.79868 4.52103 0.61958 0.77255 0.71972 0.66726

4 2.20372 4.13315 3.23997 2.94476 4.04195 2.92910 4.02423 3.35288 2.94916 3.12284 4.00467 3.17191 3.63918 3.29526 3.25968 1.79829 1.83026 2.92234 5.41719 4.17178 12 - -

2.68618 4.42225 2.77519 2.73123 3.46354 2.40513 3.72494 3.29354 2.67741 2.69355 4.24690 2.90347 2.73739 3.18146 2.89801 2.37887 2.77519 2.98518 4.58477 3.61503

0.03384 3.79868 4.52103 0.61958 0.77255 0.71972 0.66726

5 2.20372 4.13315 3.23997 2.94476 4.04195 2.92910 4.02423 3.35288 2.94916 3.12284 4.00467 3.17191 3.63918 3.29526 3.25968 1.79829 1.83026 2.92234 5.41719 4.17178 13 - -

2.68618 4.42225 2.77519 2.73123 3.46354 2.40513 3.72494 3.29354 2.67741 2.69355 4.24690 2.90347 2.73739 3.18146 2.89801 2.37887 2.77519 2.98518 4.58477 3.61503

0.03384 3.79868 4.52103 0.61958 0.77255 0.71972 0.66726

6 2.20372 4.13315 3.23997 2.94476 4.04195 2.92910 4.02423 3.35288 2.94916 3.12284 4.00467 3.17191 3.63918 3.29526 3.25968 1.79829 1.83026 2.92234 5.41719 4.17178 14 - -
2.68618 4.42225 2.77519 2.73123 3.46354 2.40513 3.72494 3.29354 2.67741 2.69355 4.24690 2.90347 2.73739 3.18146 2.89801 2.37887 2.77519 2.98518 4.58477 3.61503
0.03384 3.79868 4.52103 0.61958 0.77255 0.71972 0.66726
7 2.33953 4.28517 3.01614 2.80009 4.20235 1.76681 4.00374 3.66632 2.94310 3.32275 4.19071 3.12163 2.10388 3.26443 3.28397 2.48340 2.78391 3.19044 5.46043 4.26239 15 - -
2.68618 4.42225 2.77519 2.73123 3.46354 2.40513 3.72494 3.29354 2.67741 2.69355 4.24690 2.90347 2.73739 3.18146 2.89801 2.37887 2.77519 2.98518 4.58477 3.61503
0.03384 3.79868 4.52103 0.61958 0.77255 0.71972 0.66726
8 2.45964 4.27903 3.33044 2.91672 3.60108 2.18327 3.90550 2.88185 2.83452 2.59458 2.43907 3.25404 3.82191 3.20971 3.15008 2.65517 2.80320 2.64363 5.07722 3.81530 16 - -
2.68618 4.42225 2.77519 2.73123 3.46354 2.40513 3.72494 3.29354 2.67741 2.69355 4.24690 2.90347 2.73739 3.18146 2.89801 2.37887 2.77519 2.98518 4.58477 3.61503
0.03384 3.79868 4.52103 0.61958 0.77255 0.71972 0.66726
9 2.84222 5.32440 1.59815 1.63966 4.61065 3.14726 3.68453 4.10005 2.68280 3.64256 4.50730 2.62906 3.76137 2.85678 3.25029 2.74482 3.13054 3.70214 5.80596 4.33624 17 - -
2.68618 4.42225 2.77519 2.73123 3.46354 2.40513 3.72494 3.29354 2.67741 2.69355 4.24690 2.90347 2.73739 3.18146 2.89801 2.37887 2.77519 2.98518 4.58477 3.61503
0.03384 3.79868 4.52103 0.61958 0.77255 0.71972 0.66726
10 2.28190 4.20029 3.14531 2.95952 4.00362 2.93168 4.07052 3.51708 3.02819 3.24376 4.17741 3.19498 3.66479 3.38377 3.31525 1.20810 2.75684 3.06847 5.38248 4.08719 18 - -
2.68618 4.42225 2.77519 2.73123 3.46354 2.40513 3.72494 3.29354 2.67741 2.69355 4.24690 2.90347 2.73739 3.18146 2.89801 2.37887 2.77519 2.98518 4.58477 3.61503
0.03384 3.79868 4.52103 0.61958 0.77255 0.71972 0.66726
11 2.92534 4.76609 3.40879 2.93783 4.10199 3.42511 3.77641 3.64010 2.21590 3.16964 4.17354 3.30232 3.93290 3.00198 1.09761 3.01412 3.19740 3.36769 5.21606 4.04717 19 - -
2.68618 4.42225 2.77519 2.73123 3.46354 2.40513 3.72494 3.29354 2.67741 2.69355 4.24690 2.90347 2.73739 3.18146 2.89801 2.37887 2.77519 2.98518 4.58477 3.61503
0.03384 3.79868 4.52103 0.61958 0.77255 0.71972 0.66726
12 2.81038 4.86217 3.14703 2.63148 3.90730 3.47755 2.48586 3.60156 2.07074 3.12802 4.01732 3.06719 3.89609 2.77304 1.79388 2.84511 3.02656 3.30342 5.12586 3.78294 20 - -
2.68618 4.42225 2.77519 2.73123 3.46354 2.40513 3.72494 3.29354 2.67741 2.69355 4.24690 2.90347 2.73739 3.18146 2.89801 2.37887 2.77519 2.98518 4.58477 3.61503
0.03384 3.79868 4.52103 0.61958 0.77255 0.71972 0.66726
13 2.92534 4.76609 3.40879 2.93783 4.10199 3.42511 3.77641 3.64010 2.21590 3.16964 4.17354 3.30232 3.93290 3.00198 1.09761 3.01412 3.19740 3.36769 5.21606 4.04717 21 - -
2.68618 4.42225 2.77519 2.73123 3.46354 2.40513 3.72494 3.29354 2.67741 2.69355 4.24690 2.90347 2.73739 3.18146 2.89801 2.37887 2.77519 2.98518 4.58477 3.61503
0.03384 3.79868 4.52103 0.61958 0.77255 0.71972 0.66726
14 3.07411 4.55933 4.06997 3.71143 3.11078 3.88657 4.39036 2.31319 3.47982 0.97190 3.16033 3.99381 4.33596 3.86591 3.66719 3.45158 3.37249 2.33098 4.96135 3.66702 22 - -
2.68618 4.42225 2.77519 2.73123 3.46354 2.40513 3.72494 3.29354 2.67741 2.69355 4.24690 2.90347 2.73739 3.18146 2.89801 2.37887 2.77519 2.98518 4.58477 3.61503
0.03384 3.79868 4.52103 0.61958 0.77255 0.71972 0.66726
15 2.28190 4.20029 3.14531 2.95952 4.00362 2.93168 4.07052 3.51708 3.02819 3.24376 4.17741 3.19498 3.66479 3.38377 3.31525 1.20810 2.75684 3.06847 5.38248 4.08719 23 - -
2.68618 4.42225 2.77519 2.73123 3.46354 2.40513 3.72494 3.29354 2.67741 2.69355 4.24690 2.90347 2.73739 3.18146 2.89801 2.37887 2.77519 2.98518 4.58477 3.61503
0.03384 3.79868 4.52103 0.61958 0.77255 0.71972 0.66726
16 1.88811 4.24871 3.65003 3.22543 2.27938 3.43242 3.98793 2.55929 3.17332 2.28111 3.41397 3.48700 3.96829 3.46556 3.45370 2.81848 2.89330 2.39536 4.78044 3.37765 24 - -
2.68618 4.42225 2.77519 2.73123 3.46354 2.40513 3.72494 3.29354 2.67741 2.69355 4.24690 2.90347 2.73739 3.18146 2.89801 2.37887 2.77519 2.98518 4.58477 3.61503
0.03384 3.79868 4.52103 0.61958 0.77255 0.71972 0.66726
17 2.28190 4.20029 3.14531 2.95952 4.00362 2.93168 4.07052 3.51708 3.02819 3.24376 4.17741 3.19498 3.66479 3.38377 3.31525 1.20810 2.75684 3.06847 5.38248 4.08719 25 - -
2.68618 4.42225 2.77519 2.73123 3.46354 2.40513 3.72494 3.29354 2.67741 2.69355 4.24690 2.90347 2.73739 3.18146 2.89801 2.37887 2.77519 2.98518 4.58477 3.61503

0.03384 3.79868 4.52103 0.61958 0.77255 0.71972 0.66726
18 2.59872 4.39541 3.22522 2.83730 3.52757 3.42454 3.86438 2.00946 2.77147 2.57384 3.59668 2.39927 3.91992 3.16764 3.10092 2.79425 2.89275 2.44954 5.03649 3.70452 26 - -
2.68618 4.42225 2.77519 2.73123 3.46354 2.40513 3.72494 3.29354 2.67741 2.69355 4.24690 2.90347 2.73739 3.18146 2.89801 2.37887 2.77519 2.98518 4.58477 3.61503
0.03384 3.79868 4.52103 0.61958 0.77255 0.60318 0.79202
19 2.72034 4.70957 3.00203 1.79782 3.83251 3.48370 3.78242 3.10404 2.59346 2.73438 2.37586 3.09677 3.92851 2.98916 2.97931 2.81279 2.96873 2.89417 5.25153 3.95466 27 - -
2.68618 4.42225 2.77519 2.73123 3.46354 2.40513 3.72494 3.29354 2.67741 2.69355 4.24690 2.90347 2.73739 3.18146 2.89801 2.37887 2.77519 2.98518 4.58477 3.61503
0.02707 4.01884 4.74119 0.61958 0.77255 0.65765 0.72995
20 1.48160 4.59385 3.08731 2.71516 4.14637 3.29580 3.84667 3.44291 2.04364 3.14926 4.03554 3.12914 3.85660 3.04711 2.88135 2.69564 2.92380 3.10692 5.42368 4.15624 28 - -
2.68622 4.42229 2.77523 2.73127 3.46358 2.40517 3.72498 3.29297 2.67745 2.69359 4.24694 2.90351 2.73743 3.18150 2.89805 2.37891 2.77494 2.98522 4.58481 3.61507
0.34123 1.27162 4.74119 0.21291 1.65145 0.65765 0.72995
21 2.49457 2.81491 3.56780 3.02196 3.45437 3.43802 3.89170 2.76125 2.94951 2.28128 3.43150 2.90712 3.90245 3.25287 3.26838 2.29336 2.77875 2.30826 4.91395 3.68719 30 - -
2.68618 4.42225 2.77519 2.73123 3.46354 2.40513 3.72494 3.29354 2.67741 2.69355 4.24690 2.90347 2.73739 3.18146 2.89801 2.37887 2.77519 2.98518 4.58477 3.61503
0.02707 4.01884 4.74119 0.61958 0.77255 0.65765 0.72995
22 2.51482 5.03465 3.15796 2.56556 4.38533 3.52954 3.02316 3.78283 1.61754 3.28967 4.11408 3.04023 3.91002 2.72815 2.01596 2.80982 3.01173 3.44256 5.41389 4.14803 31 - -
2.68618 4.42225 2.77519 2.73123 3.46354 2.40513 3.72494 3.29354 2.67741 2.69355 4.24690 2.90347 2.73739 3.18146 2.89801 2.37887 2.77519 2.98518 4.58477 3.61503
0.17513 4.01884 1.94718 0.61958 0.77255 0.65765 0.72995
23 2.88947 4.93823 3.25790 2.70511 4.05045 3.53799 2.85234 3.70026 2.02288 3.20494 4.09583 3.12539 3.94736 2.78353 1.46996 2.91847 3.09527 3.39999 5.20169 3.89401 32 - -
2.68618 4.42225 2.77519 2.73123 3.46354 2.40513 3.72494 3.29354 2.67741 2.69355 4.24690 2.90347 2.73739 3.18146 2.89801 2.37887 2.77519 2.98518 4.58477 3.61503
0.03132 3.87503 4.59738 0.61958 0.77255 0.77029 0.62153
24 3.00818 4.84732 3.49908 3.00949 4.20254 3.49581 3.82586 3.74435 2.23804 3.25981 4.25983 3.36907 3.99819 3.04598 0.97448 3.09218 3.27391 3.46817 5.28449 4.12925 33 - -
2.68618 4.42225 2.77519 2.73123 3.46354 2.40513 3.72494 3.29354 2.67741 2.69355 4.24690 2.90347 2.73739 3.18146 2.89801 2.37887 2.77519 2.98518 4.58477 3.61503
0.03132 3.87503 4.59738 0.61958 0.77255 0.77029 0.62153
25 3.19970 4.59463 4.40117 3.96041 1.44893 4.18141 3.98521 2.44632 3.81331 1.58515 3.15953 4.08065 4.50356 3.97253 3.96104 3.56150 3.44762 2.48420 4.25450 2.66756 34 - -
2.68618 4.42225 2.77519 2.73123 3.46354 2.40513 3.72494 3.29354 2.67741 2.69355 4.24690 2.90347 2.73739 3.18146 2.89801 2.37887 2.77519 2.98518 4.58477 3.61503
0.03132 3.87503 4.59738 0.61958 0.77255 0.52502 0.89538
26 2.61627 4.45747 3.32258 2.75070 3.10968 3.53851 3.74967 2.94152 2.63633 2.36964 2.88376 3.19614 3.91611 2.53267 2.44003 2.77680 2.84466 2.72527 4.98652 3.72335 35 - -
2.68630 4.42237 2.77532 2.73107 3.46366 2.40525 3.72506 3.29317 2.67753 2.69367 4.24702 2.90318 2.73752 3.18158 2.89739 2.37875 2.77532 2.98530 4.58489 3.61515
0.33028 1.29908 4.76864 0.98742 0.46607 0.51602 0.90855
27 3.15330 5.26436 3.59295 2.88723 4.75262 3.75119 3.65932 4.08623 1.17978 3.50090 4.37379 3.27603 4.10940 2.79065 1.56608 3.14122 3.30748 3.76644 5.51768 4.36994 39 - -
2.68618 4.42225 2.77519 2.73123 3.46354 2.40513 3.72494 3.29354 2.67741 2.69355 4.24690 2.90347 2.73739 3.18146 2.89801 2.37887 2.77519 2.98518 4.58477 3.61503
0.02458 4.11395 4.83630 0.61958 0.77255 0.56464 0.84064
28 2.99381 4.73829 3.71648 3.64992 4.77453 0.43783 4.74884 4.43942 3.86366 4.06089 5.04340 3.88172 4.13665 4.16915 4.06687 3.17371 3.49379 3.92102 5.79916 4.88689 40 - -
2.68618 4.42225 2.77519 2.73123 3.46354 2.40513 3.72494 3.29354 2.67741 2.69355 4.24690 2.90347 2.73739 3.18146 2.89801 2.37887 2.77519 2.98518 4.58477 3.61503
0.02458 4.11395 4.83630 0.61958 0.77255 0.56464 0.84064
29 2.55773 4.33248 3.60401 3.14613 3.68374 1.71444 4.08803 2.77214 3.07419 2.31366 3.65908 3.45261 3.97549 3.41181 3.39055 2.78904 2.90111 2.07781 5.19388 3.93864 41 - -

2.68618 4.42225 2.77519 2.73123 3.46354 2.40513 3.72494 3.29354 2.67741 2.69355 4.24690 2.90347 2.73739 3.18146 2.89801 2.37887 2.77519 2.98518 4.58477 3.61503
0.14023 4.11395 2.16722 0.61958 0.77255 0.56464 0.84064

30 2.45855 4.48872 3.05647 2.71209 4.22912 1.67500 3.88445 3.66633 2.66642 3.28872 4.12283 3.09862 3.77597 3.08560 2.55125 2.20406 2.83918 3.23500 5.49434 4.21713 42 - -
2.68618 4.42225 2.77519 2.73123 3.46354 2.40513 3.72494 3.29354 2.67741 2.69355 4.24690 2.90347 2.73739 3.18146 2.89801 2.37887 2.77519 2.98518 4.58477 3.61503
0.02755 4.00127 4.72362 0.61958 0.77255 0.67303 0.71367

31 2.52806 4.59761 3.05903 2.64709 4.22705 1.98487 3.79310 3.65509 2.49959 3.25613 4.08680 3.06784 3.80111 2.97265 2.12696 2.30313 2.86617 3.25321 5.45180 4.16957 43 - -
2.68618 4.42225 2.77519 2.73123 3.46354 2.40513 3.72494 3.29354 2.67741 2.69355 4.24690 2.90347 2.73739 3.18146 2.89801 2.37887 2.77519 2.98518 4.58477 3.61503
0.02755 4.00127 4.72362 0.61958 0.77255 0.67303 0.71367

32 2.52288 4.38877 3.34107 2.80452 3.68819 2.52898 3.82939 3.05174 2.77158 2.75022 2.75942 3.21382 3.24096 3.09442 3.15624 2.20984 2.80720 2.49104 5.08986 3.83657 44 - -
2.68618 4.42225 2.77519 2.73123 3.46354 2.40513 3.72494 3.29354 2.67741 2.69355 4.24690 2.90347 2.73739 3.18146 2.89801 2.37887 2.77519 2.98518 4.58477 3.61503
0.02435 4.12306 4.84541 0.61958 0.77255 0.67303 0.71367

33 2.43995 5.02053 2.29469 2.32621 4.33380 2.81131 3.63301 3.78629 2.26822 3.32811 4.11076 2.37605 3.80501 2.75902 2.91433 2.63146 2.89119 3.01054 5.51346 4.12814 45 - -
2.68618 4.42225 2.77519 2.73123 3.46354 2.40513 3.72494 3.29354 2.67741 2.69355 4.24690 2.90347 2.73739 3.18146 2.89801 2.37887 2.77519 2.98518 4.58477 3.61503
0.14767 4.12306 2.11120 0.61958 0.77255 0.67303 0.71367

34 2.62383 4.67376 3.10219 2.55492 3.90574 2.82926 3.65387 3.29765 2.38808 2.92336 2.77007 3.03695 3.84981 2.37052 2.49080 2.69837 2.85583 3.01069 5.19569 3.90864 46 - -
2.68618 4.42225 2.77519 2.73123 3.46354 2.40513 3.72494 3.29354 2.67741 2.69355 4.24690 2.90347 2.73739 3.18146 2.89801 2.37887 2.77519 2.98518 4.58477 3.61503
0.13756 4.00289 2.20499 0.61958 0.77255 0.58203 0.81817

35 2.73985 4.24209 4.13200 3.61526 3.48031 3.09898 4.34741 1.61494 3.51258 2.30936 3.39873 3.86251 4.26126 3.79661 3.75393 3.17432 3.01941 1.46896 5.12564 3.91040 47 - -
2.68618 4.42225 2.77519 2.73123 3.46354 2.40513 3.72494 3.29354 2.67741 2.69355 4.24690 2.90347 2.73739 3.18146 2.89801 2.37887 2.77519 2.98518 4.58477 3.61503
0.02679 4.02885 4.75120 0.61958 0.77255 0.75688 0.63324

36 2.73381 4.86173 3.18337 2.58647 4.15680 3.51035 3.62487 3.02320 1.85057 3.11651 3.95612 3.05851 3.89422 2.77724 1.94156 2.50625 2.94828 3.22729 5.31401 4.04411 48 - -
2.68641 4.42277 2.77535 2.73175 3.46262 2.40548 3.72483 3.29231 2.67709 2.69407 4.24079 2.90317 2.73757 3.18144 2.89853 2.37914 2.77455 2.98570 4.58529 3.61555
0.18657 1.82288 4.75120 2.01621 0.14290 0.59865 0.79752

37 2.70057 4.99499 2.92096 2.48086 4.42547 2.16242 3.74226 3.87390 2.48652 3.41670 4.20657 2.14276 2.87728 2.87511 2.42166 2.70763 2.96775 3.47453 5.59194 4.23510 61 - -
2.68618 4.42225 2.77519 2.73123 3.46354 2.40513 3.72494 3.29354 2.67741 2.69355 4.24690 2.90347 2.73739 3.18146 2.89801 2.37887 2.77519 2.98518 4.58477 3.61503
0.12230 4.30960 2.28597 0.61958 0.77255 0.67303 0.71367

38 2.56523 4.65525 2.97726 2.68263 4.40009 2.24090 3.95924 3.85431 2.80907 3.45841 4.27817 3.11273 3.85663 2.68104 3.22121 1.37335 2.94170 3.40199 5.66136 4.34859 62 - -
2.68618 4.42225 2.77519 2.73123 3.46354 2.40513 3.72494 3.29354 2.67741 2.69355 4.24690 2.90347 2.73739 3.18146 2.89801 2.37887 2.77519 2.98518 4.58477 3.61503
0.02231 4.20961 4.93196 0.61958 0.77255 0.77878 0.61427

39 2.71311 4.99077 3.02247 2.49955 4.37284 2.53812 3.67176 3.79953 2.00689 3.33425 4.13266 3.00442 3.88019 2.80149 2.18761 2.09352 2.95382 3.42565 5.49859 4.17315 63 - -
2.68618 4.42225 2.77519 2.73123 3.46354 2.40513 3.72494 3.29354 2.67741 2.69355 4.24690 2.90347 2.73739 3.18146 2.89801 2.37887 2.77519 2.98518 4.58477 3.61503
0.02231 4.20961 4.93196 0.61958 0.77255 0.77878 0.61427

40 2.45316 2.70986 3.69291 3.17613 3.70841 2.01727 4.06252 2.65575 3.11784 2.78709 3.67102 3.43492 3.89897 3.40658 3.43398 2.16209 2.49531 2.75493 5.14735 3.93174 64 - -
2.68618 4.42225 2.77519 2.73123 3.46354 2.40513 3.72494 3.29354 2.67741 2.69355 4.24690 2.90347 2.73739 3.18146 2.89801 2.37887 2.77519 2.98518 4.58477 3.61503
0.02231 4.20961 4.93196 0.61958 0.77255 0.58104 0.81943

41 2.29593 3.06647 3.61967 3.05046 3.45109 2.52971 3.91512 2.80788 2.61980 2.52954 3.04253 3.42662 4.02474 3.28428 3.31711 2.89653 2.64827 2.58890 3.31110 3.68601   65 - -
2.68618 4.42225 2.77519 2.73123 3.46354 2.40513 3.72494 3.29354 2.67741 2.69355 4.24690 2.90347 2.73739 3.18146 2.89801 2.37887 2.77519 2.98518 4.58477 3.61503
0.01920 4.35828 5.08063 0.61958 0.77255 0.54781 0.86326
42 2.66592 3.04960 3.81905 3.24260 3.36276 3.70953 3.98669 2.41900 2.72867 1.93162 2.69145 3.56345 4.08365 2.79088 3.43146 2.69721 2.89774 2.50384 4.84656 3.63773   66 - -
2.68620 4.42227 2.77522 2.73125 3.46284 2.40515 3.72497 3.29356 2.67743 2.69357 4.24692 2.90349 2.73742 3.18148 2.89803 2.37889 2.77522 2.98520 4.58479 3.61505
0.17314 2.20613 3.01898 0.30051 1.34878 0.60255 0.79278
43 2.68699 4.34845 3.66787 3.09675 2.90630 3.69045 3.95761 2.35441 3.01248 2.50022 3.43741 3.47277 4.06899 3.32310 2.10523 2.94837 2.23434 2.35967 4.94868 3.72291   68 - -
2.68618 4.42225 2.77519 2.73123 3.46354 2.40513 3.72494 3.29354 2.67741 2.69355 4.24690 2.90347 2.73739 3.18146 2.89801 2.37887 2.77519 2.98518 4.58477 3.61503
0.01901 4.36819 5.09054 0.61958 0.77255 0.47725 0.96887
44 2.71364 4.83942 3.16786 2.59971 4.06914 3.55264 3.74310 2.84772 1.89391 3.08800 3.20363 3.10306 3.93913 2.90798 2.28726 2.39649 2.67193 3.16479 5.34575 4.03627   69 - -
2.68618 4.42225 2.77519 2.73123 3.46354 2.40513 3.72494 3.29354 2.67741 2.69355 4.24690 2.90347 2.73739 3.18146 2.89801 2.37887 2.77519 2.98518 4.58477 3.61503
0.01754 4.44759 5.16994 0.61958 0.77255 0.55089 0.85905
45 3.02620 4.38354 4.82306 4.24025 1.54083 4.19261 4.36369 2.20503 4.06129 1.60308 3.16707 4.28013 4.50271 4.15626 4.09735 3.51067 3.25252 2.24961 3.22237 3.42349   70 - -
2.68618 4.42225 2.77519 2.73123 3.46354 2.40513 3.72494 3.29354 2.67741 2.69355 4.24690 2.90347 2.73739 3.18146 2.89801 2.37887 2.77519 2.98518 4.58477 3.61503
0.01754 4.44759 5.16994 0.61958 0.77255 0.55089 0.85905
46 2.68189 4.78935 3.14447 2.58503 3.48289 3.53463 3.74366 2.83478 2.33595 2.77327 3.86751 2.35502 3.92276 2.48549 2.77469 2.52679 2.91300 3.10754 5.30840 3.26567   71 - -
2.68618 4.42225 2.77519 2.73123 3.46354 2.40513 3.72494 3.29354 2.67741 2.69355 4.24690 2.90347 2.73739 3.18146 2.89801 2.37887 2.77519 2.98518 4.58477 3.61503
0.01754 4.44759 5.16994 0.61958 0.77255 0.55089 0.85905
47 2.70032 4.21757 4.02857 3.45191 3.34124 3.78026 4.09135 2.20806 3.35063 1.74437 3.33647 3.21207 4.15678 3.59836 3.57815 2.78868 2.30874 2.02132 4.85089 3.65125   72 - -
2.68618 4.42225 2.77519 2.73123 3.46354 2.40513 3.72494 3.29354 2.67741 2.69355 4.24690 2.90347 2.73739 3.18146 2.89801 2.37887 2.77519 2.98518 4.58477 3.61503
0.01754 4.44759 5.16994 0.61958 0.77255 0.55089 0.85905
48 2.50825 3.34867 4.42659 3.82899 3.21247 3.84635 4.17367 2.22486 3.66508 1.67331 3.22568 3.92770 4.21142 3.84240 3.75951 2.82170 2.93173 1.78605 4.74471 3.13860   73 - -
2.68627 4.42234 2.77529 2.73132 3.46363 2.40505 3.72504 3.29321 2.67750 2.69341 4.24699 2.90356 2.73749 3.18155 2.89810 2.37896 2.77529 2.98436 4.58486 3.61512
0.08597 2.56792 5.16994 1.36709 0.29417 0.44709 1.02023
49 2.50006 4.58824 3.44398 2.86978 3.76045 3.65209 3.87109 3.10617 2.08660 1.72327 3.68582 3.31318 4.03609 3.12451 2.82003 2.89449 2.72347 2.63194 5.14965 3.89849   80 - -
2.68618 4.42225 2.77519 2.73123 3.46354 2.40513 3.72494 3.29354 2.67741 2.69355 4.24690 2.90347 2.73739 3.18146 2.89801 2.37887 2.77519 2.98518 4.58477 3.61503
0.01684 4.48822 5.21056 0.61958 0.77255 0.48576 0.95510
50 2.43827 4.50403 4.92858 4.36869 3.52087 4.44586 4.85937 1.82218 4.23995 1.18365 3.37443 4.52691 4.74625 4.41526 4.36375 3.78422 3.40888 1.62904 5.34107 4.18958   81 - -
2.68618 4.42225 2.77519 2.73123 3.46354 2.40513 3.72494 3.29354 2.67741 2.69355 4.24690 2.90347 2.73739 3.18146 2.89801 2.37887 2.77519 2.98518 4.58477 3.61503
0.01684 4.48822 5.21056 0.61958 0.77255 0.48576 0.95510
51 2.70459 4.16166 4.24446 3.65663 2.13194 2.95278 4.13362 2.30322 3.52489 1.91633 3.27072 3.83326 4.18926 3.73297 3.68130 2.51030 2.93878 2.12186 4.77852 3.58576   82 - -
2.68618 4.42225 2.77519 2.73123 3.46354 2.40513 3.72494 3.29354 2.67741 2.69355 4.24690 2.90347 2.73739 3.18146 2.89801 2.37887 2.77519 2.98518 4.58477 3.61503
0.01684 4.48822 5.21056 0.61958 0.77255 0.48576 0.95510
52 2.83167 4.61161 5.04253 4.46110 2.05413 4.50366 4.79435 2.04015 4.30927 0.99021 3.11931 4.59371 4.75398 4.38832 4.36636 3.83550 3.50154 2.44546 5.14155 3.99943   83 - -
2.68618 4.42225 2.77519 2.73123 3.46354 2.40513 3.72494 3.29354 2.67741 2.69355 4.24690 2.90347 2.73739 3.18146 2.89801 2.37887 2.77519 2.98518 4.58477 3.61503

0.01684 4.48822 5.21056 0.61958 0.77255 0.48576 0.95510
53 2.71567 3.44016 4.14427 3.56398 2.63933 3.80507 4.12901 2.28135 3.45089 2.36577 2.95105 3.78485 4.18471 3.67872 3.64702 1.59692 2.95468 2.41486 4.82931 3.62843 84 - -
2.68618 4.42225 2.77519 2.73123 3.46354 2.40513 3.72494 3.29354 2.67741 2.69355 4.24690 2.90347 2.73739 3.18146 2.89801 2.37887 2.77519 2.98518 4.58477 3.61503
0.01684 4.48822 5.21056 0.61958 0.77255 0.48576 0.95510
54 2.53365 3.10166 4.63197 4.02680 2.63281 3.92319 4.26010 2.08435 3.83493 1.80052 2.74186 4.05730 4.28076 3.98193 3.87575 3.23407 2.71124 1.86837 4.77140 3.59535 85 - -
2.68618 4.42225 2.77519 2.73123 3.46354 2.40513 3.72494 3.29354 2.67741 2.69355 4.24690 2.90347 2.73739 3.18146 2.89801 2.37887 2.77519 2.98518 4.58477 3.61503
0.01684 4.48822 5.21056 0.61958 0.77255 0.48576 0.95510
55 3.55435 4.81978 5.42466 4.84713 2.46985 4.87823 5.18165 1.82314 4.70743 0.83419 3.07937 5.00014 5.02607 4.69131 4.71481 4.23782 3.77070 2.15952 5.36306 4.30461 86 - -
2.68618 4.42225 2.77519 2.73123 3.46354 2.40513 3.72494 3.29354 2.67741 2.69355 4.24690 2.90347 2.73739 3.18146 2.89801 2.37887 2.77519 2.98518 4.58477 3.61503
0.17321 4.48822 1.91193 0.61958 0.77255 0.48576 0.95510
56 2.00087 4.18015 4.22209 3.63879 2.90478 3.84816 4.16808 2.42847 3.51856 1.92529 2.65577 3.84237 4.20940 3.73333 3.69379 3.14143 2.66519 1.89109 4.82542 3.63997 87 - -
2.68618 4.42225 2.77519 2.73123 3.46354 2.40513 3.72494 3.29354 2.67741 2.69355 4.24690 2.90347 2.73739 3.18146 2.89801 2.37887 2.77519 2.98518 4.58477 3.61503
0.01966 4.33467 5.05702 0.61958 0.77255 0.70134 0.68502
57 2.62369 4.39003 3.56763 3.03314 3.63784 3.54423 3.97611 2.92360 2.97741 2.12965 3.59584 3.40496 2.29090 3.29368 3.32424 2.15458 2.90545 2.22385 5.09063 3.85299 88 - -
2.68618 4.42225 2.77519 2.73123 3.46354 2.40513 3.72494 3.29354 2.67741 2.69355 4.24690 2.90347 2.73739 3.18146 2.89801 2.37887 2.77519 2.98518 4.58477 3.61503
0.01966 4.33467 5.05702 0.61958 0.77255 0.70134 0.68502
58 2.24953 4.15464 4.15289 3.57008 2.64420 2.94712 4.10839 2.29984 3.45157 1.70396 3.24005 3.77888 4.16021 3.67141 3.63345 3.08208 2.91803 2.15607 4.78364 3.59210 89 - -
2.68618 4.42225 2.77519 2.73123 3.46354 2.40513 3.72494 3.29354 2.67741 2.69355 4.24690 2.90347 2.73739 3.18146 2.89801 2.37887 2.77519 2.98518 4.58477 3.61503
0.07465 4.33468 2.83323 0.61958 0.77255 0.70134 0.68502
59 1.96658 4.30401 3.60770 3.04062 3.44218 3.63163 3.25258 2.55135 2.97784 2.18875 3.41446 3.41781 4.01632 3.27571 3.30596 2.88924 2.39080 2.34633 4.90916 3.68373 90 - -
2.68618 4.42225 2.77519 2.73123 3.46354 2.40513 3.72494 3.29354 2.67741 2.69355 4.24690 2.90347 2.73739 3.18146 2.89801 2.37887 2.77519 2.98518 4.58477 3.61503
0.42056 4.28079 1.11022 0.61958 0.77255 0.34565 1.23018
60 2.24478 3.22366 3.91521 3.38764 3.45519 2.75008 4.10286 2.68985 3.28371 1.53694 3.43899 3.59743 3.99997 3.55398 3.52710 2.83178 2.84585 2.46572 4.96009 3.75893 91 - -
2.68618 4.42225 2.77519 2.73123 3.46354 2.40513 3.72494 3.29354 2.67741 2.69355 4.24690 2.90347 2.73739 3.18146 2.89801 2.37887 2.77519 2.98518 4.58477 3.61503
0.02290 4.18399 4.90634 0.61958 0.77255 0.37899 1.15376
61 3.36000 4.68823 4.84775 4.36763 1.06590 4.39727 4.01852 2.90369 4.20339 2.00655 3.55694 4.30164 4.70473 4.25440 4.25297 3.72585 3.58441 2.42675 4.24393 2.09932 92 - -
2.68618 4.42225 2.77519 2.73123 3.46354 2.40513 3.72494 3.29354 2.67741 2.69355 4.24690 2.90347 2.73739 3.18146 2.89801 2.37887 2.77519 2.98518 4.58477 3.61503
0.08519 4.44559 2.66027 0.61958 0.77255 0.55392 0.85494
62 2.68494 4.25994 3.84782 3.27593 2.93481 3.72862 3.29674 2.67690 3.18977 2.04513 3.34992 3.59100 4.10434 3.46031 3.46158 2.99959 1.80712 2.16971 4.83959 3.61052 93 - -
2.68618 4.42225 2.77519 2.73123 3.46354 2.40513 3.72494 3.29354 2.67741 2.69355 4.24690 2.90347 2.73739 3.18146 2.89801 2.37887 2.77519 2.98518 4.58477 3.61503
0.11359 4.37920 2.35557 0.61958 0.77255 0.49043 0.94769
63 2.20169 4.41542 4.79844 4.24808 3.57904 4.36768 4.80874 1.34932 4.13428 1.83221 3.44112 4.42793 4.68495 4.34924 4.29409 3.70650 3.32139 1.56072 5.35616 4.17384 94 - -
2.68618 4.42225 2.77519 2.73123 3.46354 2.40513 3.72494 3.29354 2.67741 2.69355 4.24690 2.90347 2.73739 3.18146 2.89801 2.37887 2.77519 2.98518 4.58477 3.61503
0.33456 4.35889 1.30360 0.61958 0.77255 0.56375 0.84181
64 2.59803 4.60186 3.09155 2.59135 3.79607 2.32188 3.17289 3.24080 2.58493 2.89300 3.74631 3.07009 3.86166 2.93257 3.00466 2.47246 2.59992 2.95492 5.15373 2.94818 95 - -

2.68625 4.42232 2.77526 2.73130 3.46298 2.40506 3.72501 3.29333 2.67747 2.69339 4.24696 2.90353 2.73746 3.18153 2.89787 2.37893 2.77526 2.98525 4.58484 3.61510
0.55562 0.87146 4.83553 0.14735 1.98770 0.58006 0.82067
65 2.41263 4.36642 3.48622 2.91921 3.11315 3.13458 3.12857 2.45277 2.87033 2.17813 3.46940 3.33075 3.98382 3.17861 2.71556 2.63032 2.86300 2.64130 4.94570 3.70823 97 - -
2.68621 4.42228 2.77523 2.73126 3.46299 2.40516 3.72498 3.29357 2.67744 2.69358 4.24693 2.90322 2.73743 3.18150 2.89804 2.37890 2.77523 2.98522 4.58480 3.61506
0.58248 1.73097 1.33038 0.24000 1.54471 0.51613 0.90839
66 2.59077 4.33908 3.43354 2.87181 2.77081 2.93229 3.81326 2.36515 2.81059 2.55408 3.44936 3.28309 3.13070 3.13132 2.64501 2.80056 2.82728 2.61602 4.92013 3.67621 99 - -
2.68618 4.42225 2.77519 2.73123 3.46354 2.40513 3.72494 3.29354 2.67741 2.69355 4.24690 2.90347 2.73739 3.18146 2.89801 2.37887 2.77519 2.98518 4.58477 3.61503
0.02392 4.14085 4.86320 0.61958 0.77255 0.88856 0.52975
67 3.82991 5.00895 4.81079 4.54087 1.08478 4.53933 3.52690 3.48468 4.37395 2.78197 4.06893 4.19896 4.83722 4.28071 4.36020 3.91804 4.04777 3.41810 3.64733 1.18172 100 - -
2.68618 4.42225 2.77519 2.73123 3.46354 2.40513 3.72494 3.29354 2.67741 2.69355 4.24690 2.90347 2.73739 3.18146 2.89801 2.37887 2.77519 2.98518 4.58477 3.61503
0.02392 4.14085 4.86320 0.61958 0.77255 0.88856 0.52975
68 2.67633 4.93898 2.91530 2.43741 4.24463 3.42102 2.43072 3.68507 2.12157 3.24645 4.05269 2.95509 2.93521 2.79453 2.79127 2.22961 2.91616 3.33033 5.43545 4.09261 101 - -
2.68618 4.42225 2.77519 2.73123 3.46354 2.40513 3.72494 3.29354 2.67741 2.69355 4.24690 2.90347 2.73739 3.18146 2.89801 2.37887 2.77519 2.98518 4.58477 3.61503
0.02392 4.14085 4.86320 0.61958 0.77255 0.88856 0.52975
69 2.42897 4.39049 3.27623 3.06174 4.37830 3.10035 4.23191 3.85431 3.16471 3.52496 4.38424 3.32221 1.19600 3.49563 3.48736 2.02084 2.89887 3.34069 5.70602 4.44794 102 - -
2.68618 4.42225 2.77519 2.73123 3.46354 2.40513 3.72494 3.29354 2.67741 2.69355 4.24690 2.90347 2.73739 3.18146 2.89801 2.37887 2.77519 2.98518 4.58477 3.61503
0.02392 4.14085 4.86320 0.61958 0.77255 0.88856 0.52975
70 2.63140 4.70847 2.99184 2.13335 3.95400 3.43138 3.71264 3.34328 2.54453 2.22956 3.83453 3.02527 3.04805 2.89786 2.97631 2.28848 2.87874 3.04721 5.27030 3.96115 103 - -
2.68592 4.42232 2.77527 2.73130 3.46312 2.40503 3.72502 3.29361 2.67748 2.69362 4.24697 2.90354 2.73747 3.18153 2.89808 2.37872 2.77527 2.98526 4.58484 3.61510
0.58329 0.83422 4.86320 0.13995 2.03562 0.88856 0.52975
71 2.62333 4.56088 3.21454 2.65736 2.97940 3.51117 3.71036 3.12469 2.59661 2.78599 3.65376 3.12689 3.89750 2.51966 2.60779 2.34518 2.85412 2.86416 5.02212 2.81177 105 - -
2.68618 4.42225 2.77519 2.73123 3.46354 2.40513 3.72494 3.29354 2.67741 2.69355 4.24690 2.90347 2.73739 3.18146 2.89801 2.37887 2.77519 2.98518 4.58477 3.61503
0.02392 4.14085 4.86320 0.61958 0.77255 0.42636 1.05809
72 2.78257 4.20715 4.38004 3.79460 2.87626 3.92807 2.93181 1.78763 3.65329 2.07823 3.25539 3.95420 4.28158 3.85145 3.79301 3.22779 3.01539 1.60347 4.81858 3.60492 106 - -
2.68618 4.42225 2.77519 2.73123 3.46354 2.40513 3.72494 3.29354 2.67741 2.69355 4.24690 2.90347 2.73739 3.18146 2.89801 2.37887 2.77519 2.98518 4.58477 3.61503
0.07354 4.40529 2.83557 0.61958 0.77255 0.61197 0.78150
73 2.46768 4.52156 3.32824 2.77204 3.68257 3.56591 2.91060 3.06927 2.73859 2.75547 3.62785 3.22671 3.09659 3.06813 3.13770 2.80123 2.30359 2.61071 5.08269 2.50454 107 - -
2.68618 4.42225 2.77519 2.73123 3.46354 2.40513 3.72494 3.29354 2.67741 2.69355 4.24690 2.90347 2.73739 3.18146 2.89801 2.37887 2.77519 2.98518 4.58477 3.61503
0.09404 4.35109 2.56577 0.61958 0.77255 0.38019 1.15115
74 2.67441 4.32072 3.68244 2.64846 2.80006 3.68690 3.95519 2.54689 3.05311 2.18977 3.42609 3.48051 4.06429 3.33989 3.37202 2.05499 2.66491 2.31005 4.91704 3.69564 108 - -
2.68618 4.42225 2.77519 2.73123 3.46354 2.40513 3.72494 3.29354 2.67741 2.69355 4.24690 2.90347 2.73739 3.18146 2.89801 2.37887 2.77519 2.98518 4.58477 3.61503
0.01795 4.42469 5.14704 0.61958 0.77255 0.58474 0.81475
75 2.68966 5.07216 2.37588 2.25819 4.36586 3.46993 3.67380 3.09104 2.43750 2.74883 4.12804 2.41035 3.86965 2.50899 2.92133 2.35732 2.92199 3.43496 5.53619 4.15604 109 - -
2.68618 4.42225 2.77519 2.73123 3.46354 2.40513 3.72494 3.29354 2.67741 2.69355 4.24690 2.90347 2.73739 3.18146 2.89801 2.37887 2.77519 2.98518 4.58477 3.61503
0.10825 4.42469 2.40106 0.61958 0.77255 0.42850 1.05408

76 2.53319 4.50822 3.53731 3.28983 4.65010 3.22197 4.44311 4.13534 3.39184 3.78543 4.60836 3.50941 1.86774 3.69496 3.72433 0.97479 3.01845 3.55181 5.95719 4.71677 110 - -
2.68618 4.42225 2.77519 2.73123 3.46354 2.40513 3.72494 3.29354 2.67741 2.69355 4.24690 2.90347 2.73739 3.18146 2.89801 2.37887 2.77519 2.98518 4.58477 3.61503
0.06336 4.40529 3.01217 0.61958 0.77255 0.61197 0.78150
77 2.24709 4.94989 2.99475 2.45767 4.25991 2.82615 2.88178 3.69713 2.46256 3.26366 4.05304 2.98362 3.16962 2.57247 2.93489 2.32287 2.52444 3.33269 5.46995 4.11156 111 - -
2.68618 4.42225 2.77519 2.73123 3.46354 2.40513 3.72494 3.29354 2.67741 2.69355 4.24690 2.90347 2.73739 3.18146 2.89801 2.37887 2.77519 2.98518 4.58477 3.61503
0.06507 4.36107 2.99106 0.61958 0.77255 0.66959 0.71727
78 1.58672 4.47555 3.35171 3.02869 4.47688 3.18477 4.20750 3.91028 3.12540 3.55889 4.37101 2.69842 3.87705 3.42653 3.50672 1.28943 2.91310 3.39162 5.77967 4.51750 112 - -
2.68618 4.42225 2.77519 2.73123 3.46354 2.40513 3.72494 3.29354 2.67741 2.69355 4.24690 2.90347 2.73739 3.18146 2.89801 2.37887 2.77519 2.98518 4.58477 3.61503
0.02004 4.31603 5.03838 0.61958 0.77255 0.72270 0.66445
79 2.65032 4.73428 2.71831 2.59701 3.01731 3.44291 3.79151 3.45483 2.65568 3.09064 3.93007 3.08682 3.20369 2.98089 3.10407 1.57258 2.91954 3.13764 5.33900 4.00913 113 - -
2.68618 4.42225 2.77519 2.73123 3.46354 2.40513 3.72494 3.29354 2.67741 2.69355 4.24690 2.90347 2.73739 3.18146 2.89801 2.37887 2.77519 2.98518 4.58477 3.61503
0.02004 4.31603 5.03838 0.61958 0.77255 0.72270 0.66445
80 2.64678 4.40184 3.46475 2.90866 2.30704 2.90811 3.23368 2.91758 2.86883 2.62135 3.51179 3.32405 3.99040 3.17722 3.23333 2.38923 2.53845 2.68678 4.93649 3.15786 114 - -
2.68618 4.42225 2.77519 2.73123 3.46354 2.40513 3.72494 3.29354 2.67741 2.69355 4.24690 2.90347 2.73739 3.18146 2.89801 2.37887 2.77519 2.98518 4.58477 3.61503
0.07611 4.31603 2.81459 0.61958 0.77255 0.72270 0.66445
81 2.70229 4.74450 3.20903 2.34318 3.96140 3.54589 3.71757 3.33419 2.27239 2.26736 3.82540 3.11723 3.92853 2.90013 2.17054 2.77885 2.92562 2.54354 5.25069 3.96869 115 - -
2.68618 4.42225 2.77519 2.73123 3.46354 2.40513 3.72494 3.29354 2.67741 2.69355 4.24690 2.90347 2.73739 3.18146 2.89801 2.37887 2.77519 2.98518 4.58477 3.61503
0.25735 4.26111 1.54742 0.61958 0.77255 0.48662 0.95374
82 2.65567 4.95281 2.93401 2.23279 4.30088 2.72748 3.66429 3.73749 2.40178 3.29065 4.08218 2.95503 3.84181 2.79485 2.16318 2.18018 2.63516 3.36362 5.48029 4.12789 116 - -
2.68622 4.42062 2.77523 2.73127 3.46358 2.40517 3.72498 3.29358 2.67745 2.69359 4.24694 2.90350 2.73743 3.18150 2.89805 2.37869 2.77523 2.98522 4.58481 3.61507
0.13779 2.10772 4.93258 0.66166 0.72566 0.82950 0.57317
83 2.87739 4.29553 4.42597 3.84838 2.12286 4.02359 4.30057 1.89914 3.71561 1.52617 3.09781 4.03394 3.35440 3.89320 3.85922 3.32918 3.10803 2.30494 4.83224 3.62026 119 - -
2.68618 4.42225 2.77519 2.73123 3.46354 2.40513 3.72494 3.29354 2.67741 2.69355 4.24690 2.90347 2.73739 3.18146 2.89801 2.37887 2.77519 2.98518 4.58477 3.61503
0.08029 4.21023 2.77560 0.61958 0.77255 0.82950 0.57317
84 2.64128 4.98185 2.57606 2.39027 4.28208 3.42038 3.61124 3.71836 1.93791 3.26369 4.04819 2.92007 3.81842 2.73361 2.38941 2.63580 2.53058 2.96154 5.44649 4.08678 120 - -
2.68618 4.42225 2.77519 2.73123 3.46354 2.40513 3.72494 3.29354 2.67741 2.69355 4.24690 2.90347 2.73739 3.18146 2.89801 2.37887 2.77519 2.98518 4.58477 3.61503
0.02361 4.15355 4.87589 0.61958 0.77255 0.50616 0.92333
85 2.45387 4.95969 2.56469 2.45191 4.24822 3.46200 3.67654 3.68197 2.44742 2.82545 4.04102 2.98012 3.86314 2.80677 2.25231 2.20104 2.53155 3.32339 5.45777 4.10001 121 - -
2.68618 4.42225 2.77519 2.73123 3.46354 2.40513 3.72494 3.29354 2.67741 2.69355 4.24690 2.90347 2.73739 3.18146 2.89801 2.37887 2.77519 2.98518 4.58477 3.61503
0.07271 4.36002 2.85858 0.61958 0.77255 0.67089 0.71591
86 2.63119 3.12055 3.65943 3.08893 3.39787 3.64449 3.91716 2.14464 3.02116 2.47686 3.38101 3.44931 3.19756 2.65299 3.33291 2.90314 2.47261 2.53390 4.86658 3.11896 122 - -
2.68618 4.42225 2.77519 2.73123 3.46354 2.40513 3.72494 3.29354 2.67741 2.69355 4.24690 2.90347 2.73739 3.18146 2.89801 2.37887 2.77519 2.98518 4.58477 3.61503
0.02021 4.30752 5.02987 0.61958 0.77255 0.73217 0.65559
87 2.70211 5.11233 2.08889 2.37060 4.44750 3.41728 3.68042 3.91126 2.45926 3.43303 4.20487 2.54668 3.85502 2.79956 2.64235 2.38036 2.16629 3.50799 5.60059 4.20640 123 - -
2.68618 4.42225 2.77519 2.73123 3.46354 2.40513 3.72494 3.29354 2.67741 2.69355 4.24690 2.90347 2.73739 3.18146 2.89801 2.37887 2.77519 2.98518 4.58477 3.61503

0.02021 4.30752 5.02987 0.61958 0.77255 0.61563 0.77719
88 2.06525 4.38634 3.66682 3.26257 4.25825 3.23532 4.28798 3.58874 3.25109 3.32643 4.18195 3.48118 1.26352 3.55767 3.58517 2.30574 2.90639 2.62580 5.63416 4.40976 124 - -
2.68618 4.42225 2.77519 2.73123 3.46354 2.40513 3.72494 3.29354 2.67741 2.69355 4.24690 2.90347 2.73739 3.18146 2.89801 2.37887 2.77519 2.98518 4.58477 3.61503
0.01917 4.36002 5.08237 0.61958 0.77255 0.56235 0.84366
89 2.68962 4.79618 3.15535 2.58991 3.44026 3.53695 3.73389 3.41400 2.10155 2.74705 3.87376 3.09270 3.92330 2.58457 2.23233 2.75012 2.59076 2.64448 5.30839 4.00016 125 - -
2.68618 4.42225 2.77519 2.73123 3.46354 2.40513 3.72494 3.29354 2.67741 2.69355 4.24690 2.90347 2.73739 3.18146 2.89801 2.37887 2.77519 2.98518 4.58477 3.61503
0.28944 4.40637 1.43081 0.61958 0.77255 0.61049 0.78326
90 2.69116 4.38131 3.56151 3.02052 3.32352 3.64186 3.84876 2.78980 2.43416 2.54045 3.48506 3.39622 4.03223 3.23477 3.14596 2.92265 2.92703 1.94537 4.81957 2.22180 126 - -
2.68618 4.42225 2.77519 2.73123 3.46354 2.40513 3.72494 3.29354 2.67741 2.69355 4.24690 2.90347 2.73739 3.18146 2.89801 2.37887 2.77519 2.98518 4.58477 3.61503
0.02392 4.14085 4.86320 0.61958 0.77255 0.88856 0.52975
91 2.64597 4.33564 3.52965 2.98991 2.52952 3.61115 3.00379 2.81894 2.92867 2.52980 3.44807 2.78586 3.99972 3.23423 3.25922 2.87613 2.88254 1.95021 4.73391 3.37439 127 - -
2.68618 4.42225 2.77519 2.73123 3.46354 2.40513 3.72494 3.29354 2.67741 2.69355 4.24690 2.90347 2.73739 3.18146 2.89801 2.37887 2.77519 2.98518 4.58477 3.61503
0.29558 4.14085 1.42716 0.61958 0.77255 0.42464 1.06133
92 2.64593 5.08344 2.64142 2.34812 4.40661 3.08361 3.60528 3.87088 2.13641 3.37665 4.13611 2.88990 3.81089 2.31291 2.42554 2.43728 2.54983 3.46357 5.52876 4.13997 128 - -
2.68618 4.42225 2.77519 2.73123 3.46354 2.40513 3.72494 3.29354 2.67741 2.69355 4.24690 2.90347 2.73739 3.18146 2.89801 2.37887 2.77519 2.98518 4.58477 3.61503
0.10907 4.21023 2.42491 0.61958 0.77255 0.82950 0.57317
93 2.66547 5.11850 2.51095 2.31454 4.44559 2.71875 3.60191 3.91301 2.17643 3.40995 4.17346 2.58836 3.80481 2.39556 2.38239 2.63046 2.89832 3.50152 5.55493 4.16173 129 - -
2.68618 4.42225 2.77519 2.73123 3.46354 2.40513 3.72494 3.29354 2.67741 2.69355 4.24690 2.90347 2.73739 3.18146 2.89801 2.37887 2.77519 2.98518 4.58477 3.61503
0.02429 4.12546 4.84780 0.61958 0.77255 0.90069 0.52137
94 2.62264 4.63583 3.16295 2.60787 3.86554 3.02545 3.70266 3.24312 2.48899 2.61260 3.75289 2.72567 3.87581 2.89848 2.17621 2.71467 2.86048 2.46956 5.18639 3.90086 130 - -
2.68618 4.42225 2.77519 2.73123 3.46354 2.40513 3.72494 3.29354 2.67741 2.69355 4.24690 2.90347 2.73739 3.18146 2.89801 2.37887 2.77519 2.98518 4.58477 3.61503
0.02429 4.12546 4.84780 0.61958 0.77255 0.90069 0.52137
95 2.85469 5.41497 1.71441 1.84639 4.72110 3.31853 3.71882 4.22471 2.10513 3.70181 4.49605 2.39749 3.85698 2.85427 3.13433 2.76786 3.11342 3.79026 5.84574 4.38173 131 - -
2.68618 4.42225 2.77519 2.73123 3.46354 2.40513 3.72494 3.29354 2.67741 2.69355 4.24690 2.90347 2.73739 3.18146 2.89801 2.37887 2.77519 2.98518 4.58477 3.61503
0.02429 4.12546 4.84780 0.61958 0.77255 0.90069 0.52137
96 2.80266 4.19843 4.47563 3.88816 2.15869 3.95118 4.18763 2.40655 3.73228 1.71517 2.39740 4.00320 4.27710 3.87582 3.82454 3.25815 3.03042 2.04380 3.39911 3.40443 132 - -
2.68618 4.42225 2.77519 2.73123 3.46354 2.40513 3.72494 3.29354 2.67741 2.69355 4.24690 2.90347 2.73739 3.18146 2.89801 2.37887 2.77519 2.98518 4.58477 3.61503
0.02429 4.12546 4.84780 0.61958 0.77255 0.90069 0.52137
97 2.63128 4.61300 3.19010 2.62555 3.80530 3.49966 3.70294 2.61788 2.50019 2.58932 3.70192 3.10139 3.88738 2.24776 2.60945 2.41171 2.86068 2.91431 5.14408 3.86443 133 - -
2.68618 4.42225 2.77519 2.73123 3.46354 2.40513 3.72494 3.29354 2.67741 2.69355 4.24690 2.90347 2.73739 3.18146 2.89801 2.37887 2.77519 2.98518 4.58477 3.61503
0.02429 4.12546 4.84780 0.61958 0.77255 0.90069 0.52137
98 3.19513 5.33523 2.62642 0.71231 4.71707 3.45561 4.07468 4.22302 2.98929 3.80257 4.77371 3.10874 4.07471 3.29026 3.41860 3.15631 3.50397 3.88367 5.84035 4.57294 134 - -
2.68633 4.42241 2.77535 2.73108 3.46369 2.40528 3.72510 3.29369 2.67756 2.69295 4.24705 2.90362 2.73755 3.18162 2.89768 2.37902 2.77535 2.98440 4.58492 3.61474
0.77354 0.75333 2.69082 0.68134 0.70509 0.52853 0.89030
99 1.95861 5.14071 2.32350 2.02407 4.49794 3.39047 3.74568 3.95892 2.58250 3.49716 4.28782 2.90746 3.88088 2.88039 3.08894 1.93528 3.01901 3.55827 5.68013 4.27798 137 - -

2.68618 4.42225 2.77519 2.73123 3.46354 2.40513 3.72494 3.29354 2.67741 2.69355 4.24690 2.90347 2.73739 3.18146 2.89801 2.37887 2.77519 2.98518 4.58477 3.61503
0.02062 4.28748 5.00983 0.61958 0.77255 0.75381 0.63595
100 2.66745 5.09124 2.26489 1.93634 4.39959 3.42239 3.63788 3.86151 2.26952 3.38128 3.45948 2.90412 3.83203 2.75097 2.88905 2.43064 2.52071 3.46241 5.54732 4.15514 138 - -
2.68618 4.42225 2.77519 2.73123 3.46354 2.40513 3.72494 3.29354 2.67741 2.69355 4.24690 2.90347 2.73739 3.18146 2.89801 2.37887 2.77519 2.98518 4.58477 3.61503
0.21562 4.28748 1.71356 0.61958 0.77255 0.64527 0.74343
101 2.30480 4.89719 2.63227 2.39801 4.16602 3.40671 3.64596 3.59502 2.44285 2.57395 3.98118 2.47398 3.82324 2.54210 2.91471 2.41423 2.87047 3.25016 5.40004 4.04621 139 - -
2.68589 4.42232 2.77526 2.73130 3.46360 2.40519 3.72501 3.29361 2.67747 2.69361 4.24696 2.90353 2.73746 3.18153 2.89807 2.37881 2.77509 2.98482 4.58483 3.61510
0.23788 1.58914 4.87910 0.67043 0.71640 0.87569 0.53884
102 2.23467 5.18352 2.16513 1.75335 4.49834 3.36352 3.66356 3.96862 2.46871 3.48084 4.26047 2.84032 3.83192 2.78793 2.62855 2.68509 2.97471 3.56169 5.64302 4.23041 142 - -
2.68618 4.42225 2.77519 2.73123 3.46354 2.40513 3.72494 3.29354 2.67741 2.69355 4.24690 2.90347 2.73739 3.18146 2.89801 2.37887 2.77519 2.98518 4.58477 3.61503
0.02354 4.15675 4.87910 0.61958 0.77255 0.67794 0.70859
103 2.93374 4.33984 4.49851 3.95090 3.49780 4.13495 4.56343 1.18611 3.82446 2.06444 3.40173 4.16307 4.49303 4.07647 4.01756 2.88706 3.18718 1.73308 5.20319 4.00142 143 - -
2.68618 4.42225 2.77519 2.73123 3.46354 2.40513 3.72494 3.29354 2.67741 2.69355 4.24690 2.90347 2.73739 3.18146 2.89801 2.37887 2.77519 2.98518 4.58477 3.61503
0.02116 4.26189 4.98424 0.61958 0.77255 0.78018 0.61308
104 2.68285 4.89079 3.03370 2.49969 4.14329 3.47895 3.13198 3.57258 2.41656 2.82315 3.97623 1.95330 3.87968 2.82730 2.62580 2.70984 2.44979 3.24353 5.37663 4.04465 144 - -
2.68618 4.42225 2.77519 2.73123 3.46354 2.40513 3.72494 3.29354 2.67741 2.69355 4.24690 2.90347 2.73739 3.18146 2.89801 2.37887 2.77519 2.98518 4.58477 3.61503
0.02116 4.26189 4.98424 0.61958 0.77255 0.78018 0.61308
105 2.73338 4.45027 3.04840 3.00873 2.31349 3.68376 3.79210 2.96194 2.43309 2.63806 3.55610 3.39406 4.05987 3.25391 3.30163 2.93401 2.96390 2.74080 2.49972 2.76916 145 - -
2.68618 4.42225 2.77519 2.73123 3.46354 2.40513 3.72494 3.29354 2.67741 2.69355 4.24690 2.90347 2.73739 3.18146 2.89801 2.37887 2.77519 2.98518 4.58477 3.61503
0.02116 4.26189 4.98424 0.61958 0.77255 0.78018 0.61308
106 2.74159 5.21806 2.05992 2.31682 4.54831 3.39726 3.68002 4.02715 1.98530 3.52423 4.29197 2.24864 3.85440 2.79746 2.96927 2.44620 2.71674 3.60667 5.67382 4.25808 146 - -
2.68618 4.42225 2.77519 2.73123 3.46354 2.40513 3.72494 3.29354 2.67741 2.69355 4.24690 2.90347 2.73739 3.18146 2.89801 2.37887 2.77519 2.98518 4.58477 3.61503
0.02116 4.26189 4.98424 0.61958 0.77255 0.78018 0.61308
107 2.67637 5.10747 2.09789 2.34663 4.42171 3.41128 2.93588 3.88721 2.24078 3.40285 4.16893 2.89331 3.23174 2.75609 2.89907 2.45297 2.48270 3.48422 5.56584 4.16994 147 - -
2.68618 4.42225 2.77519 2.73123 3.46354 2.40513 3.72494 3.29354 2.67741 2.69355 4.24690 2.90347 2.73739 3.18146 2.89801 2.37887 2.77519 2.98518 4.58477 3.61503
0.12867 4.26189 2.23826 0.61958 0.77255 0.62394 0.76750
108 2.62823 4.69683 2.56565 2.36696 3.90144 3.48414 3.70353 3.28322 2.54574 2.50890 3.02599 2.67702 3.87578 2.88726 2.99050 2.70238 2.86038 2.50433 5.22690 3.92073 148 - -
2.68611 4.42277 2.77572 2.73145 3.46342 2.40565 3.72547 3.29406 2.67709 2.69265 4.24602 2.90329 2.73737 3.18150 2.89853 2.37876 2.77436 2.98535 4.58380 3.61555
0.63925 0.89751 2.73757 1.79895 0.18089 0.60489 0.78995
109 2.76909 5.10925 2.89226 1.48353 4.40966 3.47701 3.68690 3.85041 2.37997 2.90937 4.18324 2.70754 3.90163 2.81604 2.43893 2.75280 2.99944 3.48236 5.55059 4.19499 164 - -
2.68618 4.42225 2.77519 2.73123 3.46354 2.40513 3.72494 3.29354 2.67741 2.69355 4.24690 2.90347 2.73739 3.18146 2.89801 2.37887 2.77519 2.98518 4.58477 3.61503
0.06764 4.28244 2.96435 0.61958 0.77255 0.75911 0.63127
110 2.70517 5.15721 2.52364 2.04682 4.47205 3.40696 3.64652 3.94061 1.97925 2.87856 4.21371 2.22093 3.83822 2.76149 2.89134 2.66870 2.94110 3.53284 5.60010 4.19974 165 - -
2.68618 4.42225 2.77519 2.73123 3.46354 2.40513 3.72494 3.29354 2.67741 2.69355 4.24690 2.90347 2.73739 3.18146 2.89801 2.37887 2.77519 2.98518 4.58477 3.61503
0.02171 4.23652 4.95886 0.61958 0.77255 0.47183 0.97780

111 2.67943 4.73417 2.64174 2.43108 3.93512 3.53699 3.03645 2.04539 2.59696 2.97426 3.81718 3.10975 3.92837 2.94318 2.77950 2.75755 2.91105 2.67759 5.26310 3.96082 166 - -
2.68618 4.42225 2.77519 2.73123 3.46354 2.40513 3.72494 3.29354 2.67741 2.69355 4.24690 2.90347 2.73739 3.18146 2.89801 2.37887 2.77519 2.98518 4.58477 3.61503
0.30646 4.40637 1.37932 0.61958 0.77255 0.61049 0.78326
112 2.83116 4.43514 3.89885 3.32438 3.36458 3.83028 4.09976 2.53840 3.02239 1.35355 3.28958 3.66302 4.20083 3.45674 2.62996 3.13528 3.07119 2.17412 5.01105 3.80142 167 - -
2.68618 4.42225 2.77519 2.73123 3.46354 2.40513 3.72494 3.29354 2.67741 2.69355 4.24690 2.90347 2.73739 3.18146 2.89801 2.37887 2.77519 2.98518 4.58477 3.61503
0.02432 4.12424 4.84658 0.61958 0.77255 0.52948 0.88895
113 2.67292 5.08837 2.65835 2.09665 4.39689 3.43995 3.64630 3.85648 2.40169 3.37824 4.14369 2.25758 3.84362 2.75899 2.49510 2.65189 2.50385 2.88250 5.54524 4.15720 168 - -
2.68618 4.42225 2.77519 2.73123 3.46354 2.40513 3.72494 3.29354 2.67741 2.69355 4.24690 2.90347 2.73739 3.18146 2.89801 2.37887 2.77519 2.98518 4.58477 3.61503
0.24744 4.33624 1.57932 0.61958 0.77255 0.69951 0.68682
114 2.84264 4.24161 4.45317 3.87444 2.39603 3.99306 4.20940 2.10644 3.73321 1.51969 3.06961 4.01658 4.31433 3.88754 3.84918 3.29980 3.07101 2.06632 4.68185 2.69721 169 - -
2.68618 4.42225 2.77519 2.73123 3.46354 2.40513 3.72494 3.29354 2.67741 2.69355 4.24690 2.90347 2.73739 3.18146 2.89801 2.37887 2.77519 2.98518 4.58477 3.61503
0.02459 4.11340 4.83574 0.61958 0.77255 0.70951 0.67704
115 2.61060 4.37283 3.43770 2.87215 3.52801 2.88441 3.82877 2.61315 2.81583 2.17397 3.48371 3.29026 3.95585 3.13603 2.62921 2.60672 2.59944 2.41578 4.96057 3.71977 170 - -
2.68618 4.42225 2.77519 2.73123 3.46354 2.40513 3.72494 3.29354 2.67741 2.69355 4.24690 2.90347 2.73739 3.18146 2.89801 2.37887 2.77519 2.98518 4.58477 3.61503
0.02198 4.22451 4.94685 0.61958 0.77255 0.81637 0.58346
116 1.79332 4.65106 3.14875 2.66049 4.20698 2.56034 3.82880 3.62401 2.62373 3.23639 4.05220 3.10662 2.60216 2.99879 2.46077 2.63423 2.87187 3.23736 5.46969 4.16748 171 - -
2.68618 4.42225 2.77519 2.73123 3.46354 2.40513 3.72494 3.29354 2.67741 2.69355 4.24690 2.90347 2.73739 3.18146 2.89801 2.37887 2.77519 2.98518 4.58477 3.61503
0.02198 4.22451 4.94685 0.61958 0.77255 0.81637 0.58346
117 2.62232 4.53550 3.24789 2.40381 3.71219 3.52510 3.75464 2.58979 2.64405 2.38035 3.63095 3.15668 3.91233 2.98892 2.77075 2.40400 2.38966 2.82267 5.09224 3.82167 172 - -
2.68618 4.42225 2.77519 2.73123 3.46354 2.40513 3.72494 3.29354 2.67741 2.69355 4.24690 2.90347 2.73739 3.18146 2.89801 2.37887 2.77519 2.98518 4.58477 3.61503
0.02198 4.22451 4.94685 0.61958 0.77255 0.59363 0.80368
118 2.72254 5.10538 2.84582 1.61498 4.40284 2.77586 3.68475 3.85769 2.21199 3.39159 4.17316 2.93805 3.87417 2.80820 2.92490 2.47683 2.95871 3.47463 5.56829 3.39509 173 - -
2.68618 4.42225 2.77519 2.73123 3.46354 2.40513 3.72494 3.29354 2.67741 2.69355 4.24690 2.90347 2.73739 3.18146 2.89801 2.37887 2.77519 2.98518 4.58477 3.61503
0.11888 4.33624 2.31261 0.61958 0.77255 0.69951 0.68682
119 2.64580 4.86940 3.02233 2.47020 4.13175 2.75192 3.65860 3.55188 2.13840 3.14049 3.94908 2.98767 3.85289 2.80262 2.53620 2.29021 2.62739 3.21868 5.36662 3.12787 174 - -
2.68618 4.42225 2.77519 2.73123 3.46354 2.40513 3.72494 3.29354 2.67741 2.69355 4.24690 2.90347 2.73739 3.18146 2.89801 2.37887 2.77519 2.98518 4.58477 3.61503
0.02166 4.23902 4.96136 0.61958 0.77255 0.80264 0.59447
120 2.43916 4.92432 2.69097 2.41559 4.19505 3.43548 3.63617 3.05215 2.40761 3.19804 3.99027 2.94511 3.33129 2.43639 2.44428 2.63836 2.47294 3.27475 5.40867 4.05214 175 - -
2.68618 4.42225 2.77519 2.73123 3.46354 2.40513 3.72494 3.29354 2.67741 2.69355 4.24690 2.90347 2.73739 3.18146 2.89801 2.37887 2.77519 2.98518 4.58477 3.61503
0.02166 4.23902 4.96136 0.61958 0.77255 0.80264 0.59447
121 2.46331 5.07318 2.62476 2.35157 4.38522 2.84065 3.13153 3.84730 2.10899 3.36425 4.12805 2.89474 3.81730 2.44743 2.85445 2.62650 2.43613 3.44708 5.52733 4.13767 176 - -
2.68618 4.42225 2.77519 2.73123 3.46354 2.40513 3.72494 3.29354 2.67741 2.69355 4.24690 2.90347 2.73739 3.18146 2.89801 2.37887 2.77519 2.98518 4.58477 3.61503
0.07792 4.23902 2.80438 0.61958 0.77255 0.80264 0.59447
122 2.94222 4.31952 4.56003 3.99266 3.26262 4.15704 4.47344 1.35297 3.85671 1.84549 3.21667 4.17679 4.47165 4.05638 4.00650 3.47533 3.17769 1.76963 5.00175 2.99577 177 - -
2.68618 4.42225 2.77519 2.73123 3.46354 2.40513 3.72494 3.29354 2.67741 2.69355 4.24690 2.90347 2.73739 3.18146 2.89801 2.37887 2.77519 2.98518 4.58477 3.61503

0.02290 4.18399 4.90634 0.61958 0.77255 0.85274 0.55555

123 3.10035 4.83297 3.83690 3.78134 4.89539 0.37771 4.87100 4.58227 4.00432 4.19278 5.17920 4.00266 4.23298 4.30442 4.19520 3.28296 3.60557 4.05016 5.89658 5.01176 178 - -

2.68618 4.42225 2.77519 2.73123 3.46354 2.40513 3.72494 3.29354 2.67741 2.69355 4.24690 2.90347 2.73739 3.18146 2.89801 2.37887 2.77519 2.98518 4.58477 3.61503

0.02290 4.18399 4.90634 0.61958 0.77255 0.85274 0.55555

124 2.61318 4.15750 3.85412 3.27750 2.75178 3.67693 3.96391 2.57118 3.18760 2.04175 2.63442 3.03796 3.33127 3.44299 3.43803 2.95063 2.84595 2.04488 4.75968 3.55335 179 - -

2.68618 4.42225 2.77519 2.73123 3.46354 2.40513 3.72494 3.29354 2.67741 2.69355 4.24690 2.90347 2.73739 3.18146 2.89801 2.37887 2.77519 2.98518 4.58477 3.61503

0.02290 4.18399 4.90634 0.61958 0.77255 0.85274 0.55555

125 3.28396 4.56495 5.05931 4.51747 3.38308 4.65634 5.08356 1.49454 4.39925 1.21230 3.16855 4.72645 4.87083 4.54208 4.52310 4.02288 3.52170 1.58116 5.42420 4.31556 180 - -

2.68618 4.42225 2.77519 2.73123 3.46354 2.40513 3.72494 3.29354 2.67741 2.69355 4.24690 2.90347 2.73739 3.18146 2.89801 2.37887 2.77519 2.98518 4.58477 3.61503

0.02290 4.18399 4.90634 0.61958 0.77255 0.85274 0.55555

126 2.73627 4.94061 2.86678 2.52669 4.40656 3.38059 3.78133 3.87608 2.48289 3.43208 4.25560 1.48608 3.89981 2.93913 2.46025 2.41461 3.02625 3.48411 5.58398 4.24677 181 - -

2.68618 4.42225 2.77519 2.73123 3.46354 2.40513 3.72494 3.29354 2.67741 2.69355 4.24690 2.90347 2.73739 3.18146 2.89801 2.37887 2.77519 2.98518 4.58477 3.61503

0.02290 4.18399 4.90634 0.61958 0.77255 0.85274 0.55555

127 3.30953 4.61874 5.04939 4.46394 2.23999 4.56367 4.83292 1.94873 4.32570 1.08094 2.21607 4.64061 4.74565 4.35187 4.37124 3.90229 3.52399 2.41806 5.09275 4.01414 182 - -

2.68618 4.42225 2.77519 2.73123 3.46354 2.40513 3.72494 3.29354 2.67741 2.69355 4.24690 2.90347 2.73739 3.18146 2.89801 2.37887 2.77519 2.98518 4.58477 3.61503

0.02290 4.18399 4.90634 0.61958 0.77255 0.85274 0.55555

128 2.97809 5.58674 1.45431 1.79401 4.88313 2.68374 3.81178 4.40857 2.79248 3.88172 4.70240 2.19728 3.90988 2.96348 3.38564 2.86535 3.25602 3.96301 6.03337 4.52464 183 - -

2.68618 4.42225 2.77519 2.73123 3.46354 2.40513 3.72494 3.29354 2.67741 2.69355 4.24690 2.90347 2.73739 3.18146 2.89801 2.37887 2.77519 2.98518 4.58477 3.61503

0.02290 4.18399 4.90634 0.61958 0.77255 0.85274 0.55555

129 2.73899 5.20749 2.24546 1.79202 4.52696 3.37103 3.66958 4.00372 2.47147 3.50707 4.28280 2.84546 3.83923 2.79193 2.61153 2.04082 2.98411 3.59011 5.66248 4.24625 184 - -

2.68618 4.42225 2.77519 2.73123 3.46354 2.40513 3.72494 3.29354 2.67741 2.69355 4.24690 2.90347 2.73739 3.18146 2.89801 2.37887 2.77519 2.98518 4.58477 3.61503

0.02290 4.18399 4.90634 0.61958 0.77255 0.74420 0.64457

130 2.61753 3.13845 3.44338 2.88198 3.06915 3.57308 3.83850 2.87637 2.49415 2.60217 3.49405 2.30734 3.96364 3.14776 3.19220 2.82134 2.85503 2.24989 4.96625 3.72033 185 - -

2.68631 4.42238 2.77513 2.73136 3.46367 2.40490 3.72508 3.29367 2.67704 2.69353 4.24619 2.90319 2.73753 3.18160 2.89795 2.37900 2.77533 2.98532 4.58490 3.61516

0.21690 1.67140 4.96136 1.20044 0.35819 0.80264 0.59447

131 2.68536 4.92635 2.48387 1.77234 4.16054 3.46278 3.69534 3.55333 2.49728 2.37593 3.42864 2.98032 3.87563 2.84139 2.96616 2.70375 2.92025 3.23360 5.42153 4.07185 190 - -

2.68618 4.42225 2.77519 2.73123 3.46354 2.40513 3.72494 3.29354 2.67741 2.69355 4.24690 2.90347 2.73739 3.18146 2.89801 2.37887 2.77519 2.98518 4.58477 3.61503

0.02066 4.28584 5.00819 0.61958 0.77255 0.80264 0.59447

132 2.64458 4.25524 3.73397 3.16129 3.38631 3.67227 3.95613 2.00919 3.07202 2.13225 3.36564 3.50360 4.05193 3.36512 2.83632 2.41246 2.64819 2.28252 4.87034 3.65607 191 - -

2.68618 4.42225 2.77519 2.73123 3.46354 2.40513 3.72494 3.29354 2.67741 2.69355 4.24690 2.90347 2.73739 3.18146 2.89801 2.37887 2.77519 2.98518 4.58477 3.61503

0.07422 4.28584 2.85121 0.61958 0.77255 0.80264 0.59447

133 2.45951 5.14293 2.24131 2.10367 4.46024 3.39550 3.64242 3.93197 2.42063 3.43918 4.20326 2.87180 3.20584 2.31674 2.91844 2.65141 2.46668 3.52080 5.59644 4.19062 192 - -

2.68618 4.42225 2.77519 2.73123 3.46354 2.40513 3.72494 3.29354 2.67741 2.69355 4.24690 2.90347 2.73739 3.18146 2.89801 2.37887 2.77519 2.98518 4.58477 3.61503

0.07231 4.23340 2.89587 0.61958 0.77255 0.85274 0.55555

134 2.70835 4.92769 3.00885 2.54451 4.37338 3.41976 3.72093 3.81390 2.36843 3.36001 4.17066 2.41321 3.88730 2.86374 1.90459 1.93464 2.97490 3.43157 5.51853 4.19978 193 - -

2.68618 4.42225 2.77519 2.73123 3.46354 2.40513 3.72494 3.29354 2.67741 2.69355 4.24690 2.90347 2.73739 3.18146 2.89801 2.37887 2.77519 2.98518 4.58477 3.61503

0.02290 4.18399 4.90634 0.61958 0.77255 0.89556 0.52489

135 3.84619 5.00816 4.89923 4.61857 1.57230 4.57419 3.52853 3.55948 4.43215 2.88045 4.12710 4.22630 4.86184 4.31339 4.39540 3.93776 4.05640 3.46533 1.29738 1.58256 194 - -

2.68618 4.42225 2.77519 2.73123 3.46354 2.40513 3.72494 3.29354 2.67741 2.69355 4.24690 2.90347 2.73739 3.18146 2.89801 2.37887 2.77519 2.98518 4.58477 3.61503

0.02290 4.18399 4.90634 0.61958 0.77255 0.89556 0.52489

136 2.81156 5.09439 3.13912 2.24023 4.44231 3.54761 3.63700 3.84346 1.67912 3.35141 4.16036 3.04571 3.92826 2.76119 1.92237 2.80319 3.01883 2.93617 5.48436 4.19280 195 - -

2.68618 4.42225 2.77519 2.73123 3.46354 2.40513 3.72494 3.29354 2.67741 2.69355 4.24690 2.90347 2.73739 3.18146 2.89801 2.37887 2.77519 2.98518 4.58477 3.61503

0.02290 4.18399 4.90634 0.61958 0.77255 0.89556 0.52489

137 2.36438 4.92233 2.94451 1.89110 4.20768 3.00470 3.64647 3.63097 2.39354 2.82749 4.00718 2.95309 3.84010 2.78318 2.41243 2.66447 2.88757 3.28284 5.41470 4.06931 196 - -

2.68618 4.42225 2.77519 2.73123 3.46354 2.40513 3.72494 3.29354 2.67741 2.69355 4.24690 2.90347 2.73739 3.18146 2.89801 2.37887 2.77519 2.98518 4.58477 3.61503

0.02290 4.18399 4.90634 0.61958 0.77255 0.89556 0.52489

138 2.62220 4.46865 3.31175 2.49833 3.62817 3.54127 3.77285 2.98932 2.67796 2.68832 3.56808 3.20293 3.92761 3.03472 2.64967 2.78176 2.59218 2.06330 5.02880 2.91880 197 - -

2.68618 4.42225 2.77519 2.73123 3.46354 2.40513 3.72494 3.29354 2.67741 2.69355 4.24690 2.90347 2.73739 3.18146 2.89801 2.37887 2.77519 2.98518 4.58477 3.61503

0.02290 4.18399 4.90634 0.61958 0.77255 0.78702 0.60733

139 2.47265 4.52386 3.16540 2.82636 4.42008 1.63260 4.05207 3.86026 2.94235 3.48053 4.28705 2.61059 3.83529 3.24701 3.35637 1.99390 2.25169 3.36729 5.69889 4.41214 198 - -

2.68618 4.42225 2.77519 2.73123 3.46354 2.40513 3.72494 3.29354 2.67741 2.69355 4.24690 2.90347 2.73739 3.18146 2.89801 2.37887 2.77519 2.98518 4.58477 3.61503

0.13361 4.23902 2.20140 0.61958 0.77255 0.84761 0.55938

140 2.38863 4.73588 3.08199 2.56221 4.08706 3.41347 3.71073 3.44861 1.82703 3.08988 3.92749 3.04454 2.64181 2.87555 2.84662 2.68193 2.88140 2.74351 5.34679 4.04321 199 - -

2.68618 4.42225 2.77519 2.73123 3.46354 2.40513 3.72494 3.29354 2.67741 2.69355 4.24690 2.90347 2.73739 3.18146 2.89801 2.37887 2.77519 2.98518 4.58477 3.61503

0.02419 4.12960 4.85194 0.61958 0.77255 0.84073 0.56457

141 2.19865 4.58496 2.79041 2.63271 3.86536 3.43382 3.76328 3.20987 2.63567 2.52267 3.76138 3.10421 3.87188 2.96854 3.06473 1.99685 2.84797 2.64436 5.21795 3.92759 200 - -

2.68618 4.42225 2.77519 2.73123 3.46354 2.40513 3.72494 3.29354 2.67741 2.69355 4.24690 2.90347 2.73739 3.18146 2.89801 2.37887 2.77519 2.98518 4.58477 3.61503

0.02295 4.18170 4.90405 0.61958 0.77255 0.70938 0.67717

142 2.63434 3.89962 2.62112 2.47021 4.08603 3.46255 3.27778 3.50478 2.15814 3.10457 3.91503 2.99079 3.32055 2.81291 2.70857 2.66570 2.86552 2.78006 5.34471 3.03454 201 - -

2.68651 4.42181 2.77464 2.73122 3.46352 2.40511 3.72442 3.29387 2.67728 2.69375 4.24723 2.90303 2.73773 3.18151 2.89792 2.37861 2.77553 2.98528 4.58510 3.61536

0.49940 0.95095 5.00147 1.39502 0.28479 0.47353 0.97498

143 2.68959 3.48181 3.06578 1.90807 4.17171 3.50952 3.70892 3.59271 2.48904 2.51210 3.99053 2.79787 3.90061 2.47787 2.72100 2.71453 2.66708 3.26016 5.41523 4.07494 207 - -

2.68618 4.42225 2.77519 2.73123 3.46354 2.40513 3.72494 3.29354 2.67741 2.69355 4.24690 2.90347 2.73739 3.18146 2.89801 2.37887 2.77519 2.98518 4.58477 3.61503

0.10931 4.44383 2.38815 0.61958 0.77255 0.41332 1.08309

144 2.74521 5.25030 2.08626 2.11777 4.59134 3.46742 3.68229 4.07417 1.84109 3.54937 4.29702 2.93357 3.38076 2.48485 2.66867 2.70164 2.97846 3.63979 5.67790 4.26813 208 - -

2.68618 4.42225 2.77519 2.73123 3.46354 2.40513 3.72494 3.29354 2.67741 2.69355 4.24690 2.90347 2.73739 3.18146 2.89801 2.37887 2.77519 2.98518 4.58477 3.61503

0.06313 4.44218 3.00755 0.61958 0.77255 0.61609 0.77664

145 2.67656 5.02716 2.97421 2.28454 4.31405 2.83979 2.78946 3.75914 2.22580 3.30760 4.08701 2.64146 3.86484 2.78990 2.90759 2.50216 2.51989 2.76559 5.49840 4.12781 209 - -

2.68618 4.42225 2.77519 2.73123 3.46354 2.40513 3.72494 3.29354 2.67741 2.69355 4.24690 2.90347 2.73739 3.18146 2.89801 2.37887 2.77519 2.98518 4.58477 3.61503

0.01845 4.39750 5.11985 0.61958 0.77255 0.56763 0.83672

146 2.68100 4.41242 3.53502 2.53793 3.55378 3.65391 3.90760 2.31691 2.92398 2.43908 3.51602 3.38288 3.45434 2.93715 3.28060 2.63075 2.91325 1.86559 4.99684 3.76041 210 - -
2.68618 4.42225 2.77519 2.73123 3.46354 2.40513 3.72494 3.29354 2.67741 2.69355 4.24690 2.90347 2.73739 3.18146 2.89801 2.37887 2.77519 2.98518 4.58477 3.61503
0.41304 4.44218 1.11904 0.61958 0.77255 0.41212 1.08543
147 2.87370 5.40394 1.68721 2.22015 4.72215 2.49134 3.75802 4.22575 2.66538 3.71688 4.51819 2.08403 3.87927 2.48053 3.21527 2.79233 3.14072 3.79526 5.87173 4.40697 211 - -
2.68618 4.42225 2.77519 2.73123 3.46354 2.40513 3.72494 3.29354 2.67741 2.69355 4.24690 2.90347 2.73739 3.18146 2.89801 2.37887 2.77519 2.98518 4.58477 3.61503
0.02311 4.17477 4.89712 0.61958 0.77255 0.90313 0.51970
148 2.67440 4.72990 3.12347 2.58275 3.93614 3.50317 3.20547 3.33827 2.28223 2.12671 3.81464 3.07177 2.75462 2.61537 2.82530 2.74558 2.90095 3.05282 5.23103 3.93734 212 - -
2.68618 4.42225 2.77519 2.73123 3.46354 2.40513 3.72494 3.29354 2.67741 2.69355 4.24690 2.90347 2.73739 3.18146 2.89801 2.37887 2.77519 2.98518 4.58477 3.61503
0.02311 4.17477 4.89712 0.61958 0.77255 0.90313 0.51970
149 2.40387 4.30470 3.57938 3.01046 3.44551 3.62838 3.89622 2.67126 2.45874 2.14264 3.02844 3.39675 4.00751 3.24652 3.26268 2.88428 2.62028 2.03999 4.91937 3.69335 213 - -
2.68618 4.42225 2.77519 2.73123 3.46354 2.40513 3.72494 3.29354 2.67741 2.69355 4.24690 2.90347 2.73739 3.18146 2.89801 2.37887 2.77519 2.98518 4.58477 3.61503
0.02311 4.17477 4.89712 0.61958 0.77255 0.90313 0.51970
150 2.59850 4.32555 3.46765 2.64075 3.47457 3.16358 3.83781 2.53261 2.85774 2.01436 3.43683 3.31269 3.95711 3.16574 3.21145 2.58099 2.57472 2.40352 4.92048 3.68521 214 - -
2.68618 4.42225 2.77519 2.73123 3.46354 2.40513 3.72494 3.29354 2.67741 2.69355 4.24690 2.90347 2.73739 3.18146 2.89801 2.37887 2.77519 2.98518 4.58477 3.61503
0.02311 4.17477 4.89712 0.61958 0.77255 0.90313 0.51970
151 2.08772 4.40691 3.43248 3.22781 4.40337 3.15271 4.35555 3.73944 3.30300 3.47172 4.39486 3.44566 1.01803 3.63948 3.59692 2.65618 2.96241 3.28322 5.75131 4.53983 215 - -
2.68618 4.42225 2.77519 2.73123 3.46354 2.40513 3.72494 3.29354 2.67741 2.69355 4.24690 2.90347 2.73739 3.18146 2.89801 2.37887 2.77519 2.98518 4.58477 3.61503
0.02311 4.17477 4.89712 0.61958 0.77255 0.90313 0.51970
152 2.69181 5.09602 2.59135 2.37569 4.43276 3.02197 3.62191 3.88567 1.95712 3.39126 4.16531 2.91004 3.83590 2.73704 2.03841 2.46343 2.92275 3.48799 5.53890 4.16884 216 - -
2.68672 4.42308 2.77414 2.73159 3.46307 2.40523 3.72578 3.29342 2.67788 2.69380 4.24555 2.90258 2.73823 3.18152 2.89764 2.37849 2.77551 2.98460 4.58560 3.61514
0.59448 0.81943 4.89712 2.23134 0.11360 0.90313 0.51970
153 2.01827 4.27554 3.12045 3.04394 2.74458 3.62490 3.90785 2.66254 2.99074 2.44050 2.98731 3.41778 4.00822 3.28016 3.31716 2.88527 2.85605 2.10866 4.88668 3.65959 229 - -
2.68618 4.42225 2.77519 2.73123 3.46354 2.40513 3.72494 3.29354 2.67741 2.69355 4.24690 2.90347 2.73739 3.18146 2.89801 2.37887 2.77519 2.98518 4.58477 3.61503
0.07563 4.17477 2.85667 0.61958 0.77255 0.90313 0.51970
154 2.58467 4.51696 3.20912 2.68328 3.76884 3.03840 3.75936 3.13851 2.38548 2.18814 3.68243 3.13658 3.87851 2.98805 3.01258 2.43885 2.27105 2.86533 5.13399 3.86452 230 - -
2.68712 4.42333 2.77480 2.73160 3.46423 2.40554 3.72340 3.29321 2.67712 2.69423 4.24797 2.90269 2.73781 3.18093 2.89860 2.37791 2.77455 2.98469 4.58494 3.61611
0.55257 0.87546 4.84583 2.57427 0.07927 0.94301 0.49340
155 2.19328 4.94590 2.19398 2.36007 3.52295 3.38728 3.63597 3.65882 2.24898 3.22927 4.02678 2.62737 3.81270 2.77214 2.91430 2.63887 2.87775 3.30178 5.43720 4.06913 250 - -
2.68618 4.42225 2.77519 2.73123 3.46354 2.40513 3.72494 3.29354 2.67741 2.69355 4.24690 2.90347 2.73739 3.18146 2.89801 2.37887 2.77519 2.98518 4.58477 3.61503
0.02434 4.12349 4.84583 0.61958 0.77255 0.94301 0.49340
156 2.40093 4.21327 3.72569 3.15733 2.58601 3.65661 3.93875 2.36126 3.08507 2.19375 3.30513 2.62159 4.03268 3.36191 3.37736 2.92434 2.85358 2.18041 4.82002 3.59427 251 - -
2.68618 4.42225 2.77519 2.73123 3.46354 2.40513 3.72494 3.29354 2.67741 2.69355 4.24690 2.90347 2.73739 3.18146 2.89801 2.37887 2.77519 2.98518 4.58477 3.61503
0.02434 4.12349 4.84583 0.61958 0.77255 0.94301 0.49340
157 2.24909 4.99000 2.63785 2.18566 4.27969 3.39073 3.60382 3.72788 2.22531 2.92460 4.05006 2.88277 3.09680 2.52990 2.84819 2.61205 2.85658 3.35112 5.45677 4.08072 252 - -
2.68618 4.42225 2.77519 2.73123 3.46354 2.40513 3.72494 3.29354 2.67741 2.69355 4.24690 2.90347 2.73739 3.18146 2.89801 2.37887 2.77519 2.98518 4.58477 3.61503

0.02434 4.12349 4.84583 0.61958 0.77255 0.94301 0.49340

158 2.31945 4.26560 3.47296 3.17703 4.33178 3.04084 4.25350 3.70214 3.19966 3.42924 4.27836 3.35881 3.77756 3.51966 3.51171 1.13296 2.01868 3.19576 5.68314 4.45168 253 - -

2.68618 4.42225 2.77519 2.73123 3.46354 2.40513 3.72494 3.29354 2.67741 2.69355 4.24690 2.90347 2.73739 3.18146 2.89801 2.37887 2.77519 2.98518 4.58477 3.61503

0.02434 4.12349 4.84583 0.61958 0.77255 0.84735 0.55958

159 2.90826 5.41732 1.77216 1.84457 4.73024 3.32719 3.78733 4.22630 2.72093 3.73314 4.55018 2.79983 1.95623 2.93726 3.27661 2.82527 3.18104 3.80671 5.89714 4.43349 254 - -

2.68592 4.42239 2.77534 2.73123 3.46368 2.40527 3.72466 3.29368 2.67721 2.69369 4.24575 2.90333 2.73724 3.18161 2.89815 2.37890 2.77516 2.98533 4.58491 3.61517

0.60137 0.81087 4.89712 0.52985 0.88842 0.90313 0.51970

160 2.63688 4.23422 3.74892 3.17220 3.35062 3.67177 3.94298 2.28534 2.65981 1.88318 2.90321 3.50675 4.04543 3.36631 3.01867 2.70224 2.86833 2.29728 4.83360 3.62260 257 - -

2.68618 4.42225 2.77519 2.73123 3.46354 2.40513 3.72494 3.29354 2.67741 2.69355 4.24690 2.90347 2.73739 3.18146 2.89801 2.37887 2.77519 2.98518 4.58477 3.61503

0.12517 4.28780 2.26417 0.61958 0.77255 0.90313 0.51970

161 2.75835 5.09205 3.06114 2.48795 4.43368 3.50517 2.77935 3.85898 1.80104 3.36106 4.15360 2.68988 3.89052 2.74236 2.16479 2.74350 2.55135 3.48654 5.49484 4.17488 258 - -

2.68618 4.42225 2.77519 2.73123 3.46354 2.40513 3.72494 3.29354 2.67741 2.69355 4.24690 2.90347 2.73739 3.18146 2.89801 2.37887 2.77519 2.98518 4.58477 3.61503

0.02286 4.18549 4.90784 0.61958 0.77255 0.26507 1.45738

162 2.45018 5.19031 2.61861 2.32888 4.51388 3.51498 2.71681 3.98411 2.25527 3.48582 4.23711 2.99144 3.45239 2.80841 2.13561 2.70731 2.76733 2.90719 5.63555 4.23916 259 - -

2.68618 4.42225 2.77519 2.73123 3.46354 2.40513 3.72494 3.29354 2.67741 2.69355 4.24690 2.90347 2.73739 3.18146 2.89801 2.37887 2.77519 2.98518 4.58477 3.61503

0.08852 4.60585 2.59399 0.61958 0.77255 0.48576 0.95510

163 2.70704 4.59653 3.36252 2.59859 3.76273 2.98413 3.85301 2.66610 2.56807 2.18265 3.68989 3.26289 4.00687 2.79901 2.68143 2.54939 2.93842 2.58100 3.81146 3.88667 260 - -

2.68618 4.42225 2.77519 2.73123 3.46354 2.40513 3.72494 3.29354 2.67741 2.69355 4.24690 2.90347 2.73739 3.18146 2.89801 2.37887 2.77519 2.98518 4.58477 3.61503

0.01609 4.53342 5.25576 0.61958 0.77255 0.61049 0.78326

164 2.72049 5.14998 2.97159 2.23736 4.46248 2.71835 2.63153 3.36494 2.44559 3.44127 4.20120 2.39194 2.90821 2.59909 2.93170 2.54110 2.95247 3.52341 5.60275 4.21170 261 - -

2.68618 4.42225 2.77519 2.73123 3.46354 2.40513 3.72494 3.29354 2.67741 2.69355 4.24690 2.90347 2.73739 3.18146 2.89801 2.37887 2.77519 2.98518 4.58477 3.61503

0.01609 4.53342 5.25576 0.61958 0.77255 0.61049 0.78326

165 2.42772 4.28953 3.38694 3.29999 2.78486 3.76001 4.04063 2.73683 3.22327 1.56256 3.03101 2.90438 4.13408 3.48856 3.49782 3.02716 2.94568 2.33351 4.89075 3.68153 262 - -

2.68657 4.42277 2.77552 2.73095 3.46377 2.40511 3.72451 3.29406 2.67719 2.69393 4.24742 2.90363 2.73775 3.18110 2.89576 2.37912 2.77533 2.98540 4.58529 3.61464

0.51130 1.03514 3.09878 1.66474 0.20978 0.61049 0.78326

166 2.73997 5.21932 2.05107 1.86446 4.55045 3.48361 3.69048 4.02649 2.43149 3.51577 3.54786 2.95465 3.89254 2.56360 2.26953 2.70377 2.97254 3.60318 5.65561 4.25245 269 - -

2.68618 4.42225 2.77519 2.73123 3.46354 2.40513 3.72494 3.29354 2.67741 2.69355 4.24690 2.90347 2.73739 3.18146 2.89801 2.37887 2.77519 2.98518 4.58477 3.61503

0.01676 4.49271 5.21506 0.61958 0.77255 0.47540 0.97191

167 2.91519 5.24440 3.24477 2.44180 4.58638 3.66375 3.06618 4.01192 2.09861 3.50692 4.30115 3.15261 4.04150 2.65804 1.54558 2.89973 3.12496 3.64111 5.63037 2.58385 270 - -

2.68618 4.42225 2.77519 2.73123 3.46354 2.40513 3.72494 3.29354 2.67741 2.69355 4.24690 2.90347 2.73739 3.18146 2.89801 2.37887 2.77519 2.98518 4.58477 3.61503

0.01554 4.56804 5.29039 0.61958 0.77255 0.44538 1.02326

168 1.93106 4.50165 3.53878 2.97218 3.64658 3.69244 3.40991 2.41393 2.72334 2.39016 3.60225 3.39732 4.07308 2.93582 2.93020 2.93159 2.69800 2.30102 5.07651 3.83356 271 - -

2.68618 4.42225 2.77519 2.73123 3.46354 2.40513 3.72494 3.29354 2.67741 2.69355 4.24690 2.90347 2.73739 3.18146 2.89801 2.37887 2.77519 2.98518 4.58477 3.61503

0.01496 4.60585 5.32819 0.61958 0.77255 0.48576 0.95510

169 2.29094 4.75083 2.54461 2.70292 3.94611 3.60680 3.82807 2.64781 2.68861 2.51443 3.83791 2.71092 3.28417 3.02634 3.12543 2.47167 2.96361 2.53396 5.28994 3.99532 272 - -

2.68618 4.42225 2.77519 2.73123 3.46354 2.40513 3.72494 3.29354 2.67741 2.69355 4.24690 2.90347 2.73739 3.18146 2.89801 2.37887 2.77519 2.98518 4.58477 3.61503
0.01496 4.60585 5.32819 0.61958 0.77255 0.48576 0.95510
170 2.75921 5.16807 3.03601 2.09382 4.48145 3.53853 3.72316 3.93724 1.71676 3.13732 4.22062 3.02258 3.93225 2.48172 2.91475 2.20526 2.98867 2.93370 5.61639 4.23725 273 - -
2.68618 4.42225 2.77519 2.73123 3.46354 2.40513 3.72494 3.29354 2.67741 2.69355 4.24690 2.90347 2.73739 3.18146 2.89801 2.37887 2.77519 2.98518 4.58477 3.61503
0.01496 4.60585 5.32819 0.61958 0.77255 0.48576 0.95510
171 2.73727 4.58682 3.44297 2.90370 3.77731 3.04856 3.93588 2.65626 2.87713 1.92924 3.71805 2.07739 4.05690 3.20194 3.26826 2.30706 2.98314 2.89047 5.18424 3.92535 274 - -
2.68618 4.42225 2.77519 2.73123 3.46354 2.40513 3.72494 3.29354 2.67741 2.69355 4.24690 2.90347 2.73739 3.18146 2.89801 2.37887 2.77519 2.98518 4.58477 3.61503
0.01496 4.60585 5.32819 0.61958 0.77255 0.48576 0.95510
172 2.73039 4.31570 3.17378 3.29749 2.82109 3.77245 3.49001 2.27448 3.22253 2.51741 3.09828 3.62225 4.14692 2.85915 3.13416 3.03709 2.72157 1.86421 3.71638 3.70437 275 - -
2.68618 4.42225 2.77519 2.73123 3.46354 2.40513 3.72494 3.29354 2.67741 2.69355 4.24690 2.90347 2.73739 3.18146 2.89801 2.37887 2.77519 2.98518 4.58477 3.61503
0.04926 4.60585 3.26832 0.61958 0.77255 0.48576 0.95510
173 2.74800 5.11157 3.05991 2.28796 4.41025 3.53875 3.71886 3.30434 1.82960 3.39082 4.16744 3.03103 3.92891 2.46033 2.65974 2.73890 2.11499 3.16194 5.56768 4.20159 276 - -
2.68618 4.42225 2.77519 2.73123 3.46354 2.40513 3.72494 3.29354 2.67741 2.69355 4.24690 2.90347 2.73739 3.18146 2.89801 2.37887 2.77519 2.98518 4.58477 3.61503
0.05523 4.57206 3.13743 0.61958 0.77255 0.44929 1.01634
174 2.74700 4.69161 3.33824 2.77686 3.84429 3.63559 3.31729 3.25581 2.70510 2.92100 3.35696 2.97283 4.02115 2.42597 3.10523 2.86381 2.45536 2.99334 1.99684 3.92429 277 - -
2.68613 4.42249 2.77464 2.73129 3.46338 2.40523 3.72518 3.29352 2.67747 2.69328 4.24714 2.90350 2.73763 3.18170 2.89825 2.37901 2.77512 2.98485 4.58411 3.61527
1.03848 1.02712 1.24490 0.90496 0.51845 0.44538 1.02326
175 2.65482 5.05193 2.49034 2.21204 4.34978 3.10314 2.94045 3.80669 2.39524 3.33706 4.10686 2.91497 3.83028 2.55102 2.87984 2.16113 2.88735 3.41791 3.68795 4.12758 282 - -
2.68618 4.42225 2.77519 2.73123 3.46354 2.40513 3.72494 3.29354 2.67741 2.69355 4.24690 2.90347 2.73739 3.18146 2.89801 2.37887 2.77519 2.98518 4.58477 3.61503
0.10165 4.28780 2.48989 0.61958 0.77255 0.90313 0.51970
176 2.60768 4.32796 3.49564 2.92941 2.98470 3.58325 3.84763 2.60167 2.87227 2.36307 3.43527 3.33079 3.96838 3.18153 2.89732 2.26649 2.20614 2.59199 4.91570 3.67935 283 - -
2.68618 4.42225 2.77519 2.73123 3.46354 2.40513 3.72494 3.29354 2.67741 2.69355 4.24690 2.90347 2.73739 3.18146 2.89801 2.37887 2.77519 2.98518 4.58477 3.61503
0.02234 4.20848 4.93083 0.61958 0.77255 0.97031 0.47637
177 2.45048 4.79786 2.52808 2.47081 4.05149 3.43776 3.66867 3.04744 2.47801 2.82100 3.89120 2.72131 3.84118 2.82385 2.93965 2.14430 2.63959 3.13760 5.32438 3.99250 284 - -
2.68618 4.42225 2.77519 2.73123 3.46354 2.40513 3.72494 3.29354 2.67741 2.69355 4.24690 2.90347 2.73739 3.18146 2.89801 2.37887 2.77519 2.98518 4.58477 3.61503
0.02234 4.20848 4.93083 0.61958 0.77255 0.97031 0.47637
178 2.43687 4.61308 3.16880 2.11931 3.79459 3.51656 3.75163 3.12987 2.60877 2.10154 3.69414 3.12437 3.91316 2.71846 3.01996 2.75756 2.88153 2.57932 5.16546 3.88411 285 - -
2.68457 4.42305 2.77599 2.73136 3.46335 2.40579 3.72574 3.29313 2.67716 2.69270 4.24293 2.90389 2.73819 3.18109 2.89792 2.37911 2.77585 2.98530 4.58557 3.61438
0.53598 0.89726 4.93083 1.89913 0.16216 0.81207 0.58687
179 2.56334 5.19275 2.99563 2.28650 4.53604 3.50175 3.16163 3.99999 1.81433 3.48409 4.23959 2.97582 3.89358 2.35640 2.49959 2.20322 2.96463 3.58371 5.61420 4.23412 297 - -
2.68618 4.42225 2.77519 2.73123 3.46354 2.40513 3.72494 3.29354 2.67741 2.69355 4.24690 2.90347 2.73739 3.18146 2.89801 2.37887 2.77519 2.98518 4.58477 3.61503
0.01754 4.44760 5.16995 0.61958 0.77255 0.90313 0.51970
180 2.68825 4.13754 4.25315 3.66331 2.58437 2.99397 4.11566 2.55409 3.52631 1.79810 3.24547 3.82867 4.17522 3.72939 3.67296 3.10048 2.55748 1.85121 4.74564 3.04068 298 - -
2.68618 4.42225 2.77519 2.73123 3.46354 2.40513 3.72494 3.29354 2.67741 2.69355 4.24690 2.90347 2.73739 3.18146 2.89801 2.37887 2.77519 2.98518 4.58477 3.61503
0.01754 4.44760 5.16995 0.61958 0.77255 0.90313 0.51970

181 2.69309 3.66670 2.27004 2.14233 4.42304 3.47152 2.48733 3.88519 2.27859 3.40381 4.16576 2.95367 3.86867 2.60950 2.90462 2.67212 2.92475 3.48593 5.56800 3.52196 299 - -
2.68618 4.42225 2.77519 2.73123 3.46354 2.40513 3.72494 3.29354 2.67741 2.69355 4.24690 2.90347 2.73739 3.18146 2.89801 2.37887 2.77519 2.98518 4.58477 3.61503
0.01754 4.44760 5.16995 0.61958 0.77255 0.90313 0.51970
182 2.52499 4.83306 3.08525 2.19006 4.05574 3.52843 3.20782 2.12981 2.57836 3.08650 3.91622 2.57891 3.92989 2.92433 3.02971 2.39297 2.93784 3.15668 5.35262 4.03291 300 - -
2.68618 4.42225 2.77519 2.73123 3.46354 2.40513 3.72494 3.29354 2.67741 2.69355 4.24690 2.90347 2.73739 3.18146 2.89801 2.37887 2.77519 2.98518 4.58477 3.61503
0.05765 4.44760 3.11641 0.61958 0.77255 0.90313 0.51970
183 2.28894 4.66202 3.25002 2.71064 3.93985 3.51142 3.82652 3.30885 2.67935 2.73737 3.83344 3.17981 3.94389 2.48423 3.09911 1.70465 2.91898 2.62324 5.28489 3.99687 301 - -
2.68618 4.42225 2.77519 2.73123 3.46354 2.40513 3.72494 3.29354 2.67741 2.69355 4.24690 2.90347 2.73739 3.18146 2.89801 2.37887 2.77519 2.98518 4.58477 3.61503
0.10895 4.40821 2.39635 0.61958 0.77255 0.94360 0.49302
184 2.27865 4.77940 3.10723 2.57321 4.03994 3.49100 3.73243 3.43375 2.26787 3.06280 3.89569 3.07208 2.30968 2.60813 2.94820 2.72734 2.90863 2.54144 5.32713 4.01756 302 - -
2.68618 4.42225 2.77519 2.73123 3.46354 2.40513 3.72494 3.29354 2.67741 2.69355 4.24690 2.90347 2.73739 3.18146 2.89801 2.37887 2.77519 2.98518 4.58477 3.61503
0.14378 4.31923 2.11515 0.61958 0.77255 0.35965 1.19707
185 2.72231 5.15551 2.39469 2.03001 4.46972 3.50773 3.69829 3.38668 2.25603 2.88210 4.20453 2.98822 3.90067 2.47469 2.71879 2.69999 2.57404 3.52948 5.60685 3.40860 303 - -
2.68618 4.42225 2.77519 2.73123 3.46354 2.40513 3.72494 3.29354 2.67741 2.69355 4.24690 2.90347 2.73739 3.18146 2.89801 2.37887 2.77519 2.98518 4.58477 3.61503
0.01542 4.57591 5.29825 0.61958 0.77255 0.74488 0.64396
186 2.30954 5.01281 2.38150 2.56890 4.47190 2.13194 3.82668 3.92382 2.61411 3.47485 4.25208 3.08223 2.70472 2.95494 3.09233 2.16819 2.99533 3.14065 5.66138 4.29516 304 - -
2.68618 4.42225 2.77519 2.73123 3.46354 2.40513 3.72494 3.29354 2.67741 2.69355 4.24690 2.90347 2.73739 3.18146 2.89801 2.37887 2.77519 2.98518 4.58477 3.61503
0.01542 4.57591 5.29825 0.61958 0.77255 0.74488 0.64396
187 2.74334 4.69768 3.32946 2.76637 3.88183 3.63109 3.84580 2.70060 2.32356 1.87459 3.78826 2.56647 4.01662 3.06347 2.86227 2.36237 2.97342 2.99769 5.23996 3.96316 305 - -
2.68618 4.42225 2.77519 2.73123 3.46354 2.40513 3.72494 3.29354 2.67741 2.69355 4.24690 2.90347 2.73739 3.18146 2.89801 2.37887 2.77519 2.98518 4.58477 3.61503
0.01542 4.57591 5.29825 0.61958 0.77255 0.74488 0.64396
188 2.55136 4.62812 3.39526 2.84167 2.97515 3.65728 3.87503 3.17143 2.57258 2.84974 3.72395 3.29969 3.50870 2.44314 3.18956 2.89146 2.97986 2.92159 5.13547 1.82275 306 - -
2.68618 4.42225 2.77519 2.73123 3.46354 2.40513 3.72494 3.29354 2.67741 2.69355 4.24690 2.90347 2.73739 3.18146 2.89801 2.37887 2.77519 2.98518 4.58477 3.61503
0.01542 4.57591 5.29825 0.61958 0.77255 0.74488 0.64396
189 2.71776 3.40708 3.64026 3.07363 3.16049 3.70028 3.97435 2.92347 2.74700 2.16060 3.53621 3.46542 2.11608 3.32100 3.36408 2.33907 2.73713 2.69974 5.01635 3.78609 307 - -
2.68618 4.42225 2.77519 2.73123 3.46354 2.40513 3.72494 3.29354 2.67741 2.69355 4.24690 2.90347 2.73739 3.18146 2.89801 2.37887 2.77519 2.98518 4.58477 3.61503
0.01542 4.57591 5.29825 0.61958 0.77255 0.74488 0.64396
190 2.56336 4.65566 3.33368 1.82720 3.83415 3.21920 3.86255 2.34837 2.76083 2.38247 3.74999 2.94688 4.01780 3.09236 3.17780 2.85689 2.96222 2.68725 5.21429 3.93885 308 - -
2.68618 4.42225 2.77519 2.73123 3.46354 2.40513 3.72494 3.29354 2.67741 2.69355 4.24690 2.90347 2.73739 3.18146 2.89801 2.37887 2.77519 2.98518 4.58477 3.61503
0.01542 4.57591 5.29825 0.61958 0.77255 0.74488 0.64396
191 2.53840 4.50304 3.54371 2.98603 3.63374 3.68731 3.37551 3.02283 2.91775 2.49232 3.61395 3.08492 3.51775 3.24928 3.27577 2.93687 2.70757 2.79011 1.73006 3.81258 309 - -
2.68618 4.42225 2.77519 2.73123 3.46354 2.40513 3.72494 3.29354 2.67741 2.69355 4.24690 2.90347 2.73739 3.18146 2.89801 2.37887 2.77519 2.98518 4.58477 3.61503
0.01542 4.57591 5.29825 0.61958 0.77255 0.74488 0.64396
192 2.97487 5.07224 3.44235 2.53101 4.35436 3.75293 3.83265 1.78612 1.74911 3.30150 4.14952 3.29292 4.12434 2.98835 2.01262 3.01772 3.17913 3.41595 5.50520 4.25121 310 - -
2.68618 4.42225 2.77519 2.73123 3.46354 2.40513 3.72494 3.29354 2.67741 2.69355 4.24690 2.90347 2.73739 3.18146 2.89801 2.37887 2.77519 2.98518 4.58477 3.61503

0.01542 4.57591 5.29825 0.61958 0.77255 0.50992 0.91765

193 2.77998 5.28343 2.07787 2.25208 4.62269 3.53003 3.73435 4.10649 2.33913 3.58566 4.32799 2.53112 3.93577 2.83495 2.24846 2.23419 2.61686 3.67142 5.71779 4.30668 311 - -

2.68618 4.42225 2.77519 2.73123 3.46354 2.40513 3.72494 3.29354 2.67741 2.69355 4.24690 2.90347 2.73739 3.18146 2.89801 2.37887 2.77519 2.98518 4.58477 3.61503

0.01416 4.66053 5.38288 0.61958 0.77255 0.61049 0.78326

194 2.78615 5.24883 2.25090 1.44549 4.57047 3.53763 3.74738 4.04269 2.50031 3.18528 3.48967 2.76414 3.94552 2.85436 2.78772 2.75429 3.01939 3.62718 5.69323 4.29187 312 - -

2.68618 4.42225 2.77519 2.73123 3.46354 2.40513 3.72494 3.29354 2.67741 2.69355 4.24690 2.90347 2.73739 3.18146 2.89801 2.37887 2.77519 2.98518 4.58477 3.61503

0.17361 4.66053 1.89772 0.61958 0.77255 0.61049 0.78326

195 2.69901 4.92094 3.08476 1.88407 3.21479 3.52482 3.72733 3.58558 2.52079 3.18202 3.99008 2.70814 3.38165 2.87284 2.52174 2.72733 2.67408 2.79334 5.41887 3.56106 313 - -

2.68658 4.41657 2.77582 2.72941 3.46358 2.40575 3.72556 3.29416 2.67803 2.69417 4.24752 2.90409 2.73591 3.18154 2.89863 2.37932 2.77428 2.98416 4.58539 3.61565

0.38307 1.16200 5.22586 1.96287 0.15135 0.83969 0.56536

196 2.73003 5.21508 2.95425 2.00843 4.55580 3.10513 3.26557 4.03229 2.10331 3.51377 4.25992 2.52029 3.88885 2.77935 2.32571 2.22321 2.96016 3.60404 5.64549 4.24595 323 - -

2.68618 4.42225 2.77519 2.73123 3.46354 2.40513 3.72494 3.29354 2.67741 2.69355 4.24690 2.90347 2.73739 3.18146 2.89801 2.37887 2.77519 2.98518 4.58477 3.61503

0.01658 4.50351 5.22586 0.61958 0.77255 0.83969 0.56536

197 2.70565 4.35940 3.69437 2.72290 3.49230 3.71411 3.98242 1.82184 3.06822 2.27480 3.46829 3.14252 3.11053 3.35954 3.39109 2.97008 2.65083 2.25135 4.95955 3.73672 324 - -

2.68618 4.42225 2.77519 2.73123 3.46354 2.40513 3.72494 3.29354 2.67741 2.69355 4.24690 2.90347 2.73739 3.18146 2.89801 2.37887 2.77519 2.98518 4.58477 3.61503

0.01658 4.50351 5.22586 0.61958 0.77255 0.83969 0.56536

198 2.85312 5.19804 2.53582 2.23592 4.56611 2.97886 3.85930 4.02164 2.70777 3.57205 4.37188 3.01336 1.53111 3.00153 3.20994 2.83561 2.44668 3.62871 5.76627 4.37303 325 - -

2.68629 4.42236 2.77530 2.73134 3.46364 2.40523 3.72505 3.29365 2.67751 2.69303 4.24474 2.90316 2.73750 3.18157 2.89811 2.37886 2.77530 2.98529 4.58488 3.61514

1.04224 0.81907 1.57749 0.10240 2.32962 0.83969 0.56536

199 2.63092 4.40276 3.45402 2.90197 3.58419 3.57386 3.86830 2.12155 2.61895 2.64077 3.53562 3.31603 2.59436 3.17006 3.20339 2.40608 2.87624 2.41187 5.01685 3.77500 327 - -

2.68618 4.42225 2.77519 2.73123 3.46354 2.40513 3.72494 3.29354 2.67741 2.69355 4.24690 2.90347 2.73739 3.18146 2.89801 2.37887 2.77519 2.98518 4.58477 3.61503

0.02080 4.27897 5.00131 0.61958 0.77255 0.72761 0.65983

200 2.68154 4.94805 2.61340 2.48441 3.62755 3.49692 3.69688 2.84382 1.98416 3.21468 4.01338 3.01152 3.88836 2.29019 2.94801 2.48660 2.91282 2.99338 5.43676 4.08631 328 - -

2.68618 4.42225 2.77519 2.73123 3.46354 2.40513 3.72494 3.29354 2.67741 2.69355 4.24690 2.90347 2.73739 3.18146 2.89801 2.37887 2.77519 2.98518 4.58477 3.61503

0.01768 4.44009 5.16243 0.61958 0.77255 0.91111 0.51430

201 2.68784 4.21166 4.00099 3.42111 2.73004 2.94023 4.05971 2.33964 3.32414 2.40938 2.95833 3.68591 2.87483 3.56810 3.55350 2.48330 2.92145 1.92213 4.82226 3.62266 329 - -

2.68618 4.42225 2.77519 2.73123 3.46354 2.40513 3.72494 3.29354 2.67741 2.69355 4.24690 2.90347 2.73739 3.18146 2.89801 2.37887 2.77519 2.98518 4.58477 3.61503

0.01689 4.48547 5.20782 0.61958 0.77255 0.91111 0.51430

202 2.79033 5.20305 2.85789 1.60005 4.53867 3.48857 3.75231 4.00040 2.32657 3.51850 4.29361 2.98298 2.29609 2.87268 2.99823 2.47586 2.75523 3.59792 5.68222 4.29026 330 - -

2.68618 4.42225 2.77519 2.73123 3.46354 2.40513 3.72494 3.29354 2.67741 2.69355 4.24690 2.90347 2.73739 3.18146 2.89801 2.37887 2.77519 2.98518 4.58477 3.61503

0.01689 4.48547 5.20782 0.61958 0.77255 0.91111 0.51430

203 2.72841 5.00624 3.02993 2.53033 4.35737 1.96144 3.18041 3.79443 2.21448 3.35741 4.15102 3.04955 3.92711 2.89778 2.97604 2.17006 2.47786 3.42316 5.55673 4.20449 331 - -

2.68618 4.42225 2.77519 2.73123 3.46354 2.40513 3.72494 3.29354 2.67741 2.69355 4.24690 2.90347 2.73739 3.18146 2.89801 2.37887 2.77519 2.98518 4.58477 3.61503

0.01689 4.48547 5.20782 0.61958 0.77255 0.91111 0.51430

204 2.72215 4.98126 3.08829 2.52565 4.24673 3.53187 3.71712 3.67087 2.10092 2.50854 3.52428 2.39402 3.92107 2.85332 2.47377 2.17449 2.95068 3.32917 5.46078 4.11980 332 - -

2.68618 4.42225 2.77519 2.73123 3.46354 2.40513 3.72494 3.29354 2.67741 2.69355 4.24690 2.90347 2.73739 3.18146 2.89801 2.37887 2.77519 2.98518 4.58477 3.61503
0.01689 4.48547 5.20782 0.61958 0.77255 0.91111 0.51430
205 2.72456 5.21954 2.58854 1.89905 4.55471 2.83248 3.68131 4.03649 2.43173 3.52116 4.26667 2.51339 3.11790 2.78382 2.49117 2.68646 2.55710 3.60564 5.65814 4.24781 333 - -
2.68596 4.42242 2.77518 2.73140 3.46287 2.40517 3.72511 3.29370 2.67728 2.69356 4.24642 2.90335 2.73756 3.18163 2.89749 2.37903 2.77536 2.98535 4.58493 3.61520
0.88443 0.54202 5.20782 0.22537 1.60058 0.52292 0.89842
206 2.74851 3.47366 4.39223 3.79651 3.27978 3.87894 4.19444 2.13917 3.64554 1.87966 3.29761 3.34580 4.24602 3.83589 3.28883 3.17509 2.34889 1.87966 4.79820 2.86990 336 - -
2.68618 4.42225 2.77519 2.73123 3.46354 2.40513 3.72494 3.29354 2.67741 2.69355 4.24690 2.90347 2.73739 3.18146 2.89801 2.37887 2.77519 2.98518 4.58477 3.61503
0.01438 4.64514 5.36748 0.61958 0.77255 0.69951 0.68682
207 2.64443 5.45973 1.52601 1.76062 4.78657 3.10344 3.83970 4.28207 2.64816 3.75740 4.51840 2.99968 4.01010 2.49682 2.87534 2.86152 3.16735 3.84308 5.89311 4.45527 337 - -
2.68618 4.42225 2.77519 2.73123 3.46354 2.40513 3.72494 3.29354 2.67741 2.69355 4.24690 2.90347 2.73739 3.18146 2.89801 2.37887 2.77519 2.98518 4.58477 3.61503
0.01438 4.64514 5.36748 0.61958 0.77255 0.69951 0.68682
208 2.54550 4.61351 3.42336 2.47015 3.33215 3.66856 3.90281 3.14981 2.83337 1.79488 3.70871 2.75935 3.54668 3.15775 2.97559 2.65378 2.97729 2.42971 5.17475 3.91194 338 - -
2.68618 4.42225 2.77519 2.73123 3.46354 2.40513 3.72494 3.29354 2.67741 2.69355 4.24690 2.90347 2.73739 3.18146 2.89801 2.37887 2.77519 2.98518 4.58477 3.61503
0.01438 4.64514 5.36748 0.61958 0.77255 0.69951 0.68682
209 2.54116 4.45004 2.94905 2.82080 3.58588 3.32418 3.99423 2.15324 3.04178 2.65941 2.83557 3.48600 3.43868 3.33969 3.38776 2.98393 2.98089 1.82689 5.03762 3.80657 339 - -
2.68618 4.42225 2.77519 2.73123 3.46354 2.40513 3.72494 3.29354 2.67741 2.69355 4.24690 2.90347 2.73739 3.18146 2.89801 2.37887 2.77519 2.98518 4.58477 3.61503
0.01438 4.64514 5.36748 0.61958 0.77255 0.54128 0.87228
210 2.56259 4.52109 3.59430 3.02743 3.66383 3.73302 3.98045 2.63544 2.37765 2.41103 3.07189 2.79279 4.11321 3.29286 2.80990 2.97372 2.72302 1.90785 5.09889 3.85853 340 - -
2.68595 4.42231 2.77525 2.73129 3.46360 2.40519 3.72500 3.29360 2.67747 2.69361 4.24623 2.90353 2.73745 3.18152 2.89807 2.37859 2.77525 2.98524 4.58483 3.61509
0.23990 1.56608 5.41945 0.16063 1.90790 0.61049 0.78326
211 1.87591 4.41366 3.78098 3.21082 3.19458 3.77694 4.05018 2.61137 2.76457 2.62300 3.52494 3.57609 3.19039 3.43675 3.46862 2.76962 2.58325 1.88499 5.01058 3.78994 342 - -
2.68618 4.42225 2.77519 2.73123 3.46354 2.40513 3.72494 3.29354 2.67741 2.69355 4.24690 2.90347 2.73739 3.18146 2.89801 2.37887 2.77519 2.98518 4.58477 3.61503
0.01364 4.69710 5.41945 0.61958 0.77255 0.61049 0.78326
212 2.59983 5.29660 2.01074 2.34719 4.63574 3.55084 3.75343 4.11600 1.61572 3.59741 4.34309 3.02028 3.95696 2.85538 2.66622 2.54934 2.82661 3.68491 5.73058 4.32415 343 - -
2.68618 4.42225 2.77519 2.73123 3.46354 2.40513 3.72494 3.29354 2.67741 2.69355 4.24690 2.90347 2.73739 3.18146 2.89801 2.37887 2.77519 2.98518 4.58477 3.61503
0.01364 4.69710 5.41945 0.61958 0.77255 0.41499 1.07983
213 2.23738 4.74668 3.09037 2.62249 3.92747 3.68386 3.91171 3.30735 2.47371 1.98614 3.83771 3.29603 4.07173 3.13206 3.22189 2.36415 3.02412 2.13093 5.29575 4.01500 344 - -
2.68618 4.42225 2.77519 2.73123 3.46354 2.40513 3.72494 3.29354 2.67741 2.69355 4.24690 2.90347 2.73739 3.18146 2.89801 2.37887 2.77519 2.98518 4.58477 3.61503
0.01282 4.75893 5.48127 0.61958 0.77255 0.48576 0.95510
214 2.40068 4.90720 3.23807 2.67921 3.48599 3.26762 3.84483 3.14036 2.32533 2.91845 3.52938 2.91604 2.13306 2.62839 2.87449 2.84307 3.01679 2.98947 5.42509 4.10808 345 - -
2.68618 4.42225 2.77519 2.73123 3.46354 2.40513 3.72494 3.29354 2.67741 2.69355 4.24690 2.90347 2.73739 3.18146 2.89801 2.37887 2.77519 2.98518 4.58477 3.61503
0.01282 4.75893 5.48127 0.61958 0.77255 0.48576 0.95510
215 2.23306 1.99774 4.51010 3.92031 3.37891 3.95339 4.31081 2.42145 3.76775 2.04533 3.38909 4.03549 4.33417 3.95540 3.88183 2.65937 2.82480 1.87574 4.90774 3.72315 346 - -
2.68618 4.42225 2.77519 2.73123 3.46354 2.40513 3.72494 3.29354 2.67741 2.69355 4.24690 2.90347 2.73739 3.18146 2.89801 2.37887 2.77519 2.98518 4.58477 3.61503
0.01282 4.75893 5.48127 0.61958 0.77255 0.48576 0.95510

216 2.87241 5.33652 2.32755 2.56374 4.68331 3.62937 3.79904 4.15020 1.88753 3.62958 3.49874 2.67539 4.02380 2.90206 1.62525 2.83860 3.09883 3.72958 5.75589 4.37267 347 - -
2.68618 4.42225 2.77519 2.73123 3.46354 2.40513 3.72494 3.29354 2.67741 2.69355 4.24690 2.90347 2.73739 3.18146 2.89801 2.37887 2.77519 2.98518 4.58477 3.61503
0.01226 4.80366 5.52600 0.61958 0.77255 0.48576 0.95510
217 2.80682 5.20063 3.10513 2.30135 4.50226 2.87524 2.89964 3.50481 2.08497 2.43107 4.25350 3.08576 3.99004 2.34232 2.61932 2.79262 3.03736 3.56785 5.65878 3.32366 348 - -
2.68618 4.42225 2.77519 2.73123 3.46354 2.40513 3.72494 3.29354 2.67741 2.69355 4.24690 2.90347 2.73739 3.18146 2.89801 2.37887 2.77519 2.98518 4.58477 3.61503
0.01226 4.80366 5.52600 0.61958 0.77255 0.48576 0.95510
218 2.85294 4.26705 4.58621 3.05846 2.30254 3.99510 4.31562 2.21476 3.81832 2.16685 2.02542 4.07824 3.26997 3.99203 3.90740 2.81437 3.08505 2.38168 3.08930 3.70249 349 - -
2.68620 4.42249 2.77544 2.73069 3.46378 2.40470 3.72519 3.29378 2.67701 2.69379 4.24714 2.90302 2.73764 3.18171 2.89805 2.37883 2.77544 2.98543 4.58501 3.61527
0.28286 1.41586 5.61013 0.98796 0.46575 0.48576 0.95510
219 2.41483 5.17848 3.15560 2.46898 4.46111 2.91072 3.83018 3.32271 2.27929 3.45642 3.85229 2.92966 4.02736 2.77810 2.32106 2.21517 3.06514 3.53513 2.68102 4.28118 354 - -
2.68618 4.42225 2.77519 2.73123 3.46354 2.40513 3.72494 3.29354 2.67741 2.69355 4.24690 2.90347 2.73739 3.18146 2.89801 2.37887 2.77519 2.98518 4.58477 3.61503
0.01126 4.88778 5.61013 0.61958 0.77255 0.48576 0.95510
220 2.83509 5.19539 1.84926 2.42620 4.48292 3.63245 3.82772 3.93030 2.58998 2.73450 3.92659 3.12679 4.02516 2.94692 2.69043 1.96070 2.86410 3.55482 5.66395 3.23677 355 - -
2.68618 4.42225 2.77519 2.73123 3.46354 2.40513 3.72494 3.29354 2.67741 2.69355 4.24690 2.90347 2.73739 3.18146 2.89801 2.37887 2.77519 2.98518 4.58477 3.61503
0.43035 4.88778 1.07241 0.61958 0.77255 0.48576 0.95510
221 3.26705 5.36502 3.62947 3.03939 4.87552 3.32401 3.88563 4.24899 2.17280 3.68812 4.55827 3.43790 4.26869 2.62538 0.83637 3.27209 3.46095 3.91225 5.72038 4.54950 356 - -
2.68618 4.42225 2.77519 2.73123 3.46354 2.40513 3.72494 3.29354 2.67741 2.69355 4.24690 2.90347 2.73739 3.18146 2.89801 2.37887 2.77519 2.98518 4.58477 3.61503
0.04374 4.47450 3.46093 0.61958 0.77255 1.10270 0.40343
222 2.69472 4.91941 1.99614 2.22733 2.99803 3.50605 3.72101 3.58296 2.52173 3.18139 3.44904 3.03111 3.90462 2.86779 2.99007 2.60359 2.44910 3.25356 5.41927 4.07640 357 - -
2.68618 4.42225 2.77519 2.73123 3.46354 2.40513 3.72494 3.29354 2.67741 2.69355 4.24690 2.90347 2.73739 3.18146 2.89801 2.37887 2.77519 2.98518 4.58477 3.61503
0.01753 4.44830 5.17064 0.61958 0.77255 1.12570 0.39219
223 2.60323 4.27384 4.08845 3.54520 3.47639 3.75572 4.21734 2.56637 3.44043 1.97635 3.45438 3.77968 3.69378 3.70208 3.66997 2.59366 2.99398 1.32175 5.00547 3.80484 358 - -
2.68618 4.42225 2.77519 2.73123 3.46354 2.40513 3.72494 3.29354 2.67741 2.69355 4.24690 2.90347 2.73739 3.18146 2.89801 2.37887 2.77519 2.98518 4.58477 3.61503
0.04251 4.44830 3.50912 0.61958 0.77255 1.12570 0.39219
224 2.25336 4.21663 3.94133 3.36870 2.18332 2.60997 4.04005 2.67242 3.27686 1.98378 3.12327 3.64508 4.11090 3.52935 3.52019 2.82910 2.90943 2.47202 4.82919 3.62136 359 - -
2.68646 4.42253 2.77547 2.73151 3.46382 2.40541 3.72465 3.29382 2.67761 2.69221 4.24673 2.90374 2.73767 3.18174 2.89671 2.37891 2.77515 2.98546 4.58505 3.61470
1.07183 0.53130 2.66239 0.68604 0.70030 0.30442 1.33769
225 2.75822 5.04301 3.28316 2.71652 4.28251 3.69021 2.65796 3.18107 2.32358 2.44583 4.11405 3.22444 4.07660 1.52957 3.01629 2.90664 3.09525 3.37807 5.52840 4.21054 362 - -
2.68618 4.42225 2.77519 2.73123 3.46354 2.40513 3.72494 3.29354 2.67741 2.69355 4.24690 2.90347 2.73739 3.18146 2.89801 2.37887 2.77519 2.98518 4.58477 3.61503
0.07133 4.81328 2.80142 0.61958 0.77255 0.53589 0.87983
226 3.08991 4.44715 4.98377 4.38108 3.16849 4.28915 3.92036 1.86005 4.19410 1.20592 2.88511 4.42577 4.61113 4.30891 4.22387 3.60720 3.31938 1.70164 5.06914 3.92110 363 - -
2.68618 4.42225 2.77519 2.73123 3.46354 2.40513 3.72494 3.29354 2.67741 2.69355 4.24690 2.90347 2.73739 3.18146 2.89801 2.37887 2.77519 2.98518 4.58477 3.61503
0.01230 4.79973 5.52208 0.61958 0.77255 0.58595 0.81323
227 2.65005 4.43307 3.91538 3.34282 2.54257 3.85592 4.12958 1.90260 3.27307 2.15882 3.54192 2.49740 4.23169 2.53775 3.56796 2.95479 2.77357 2.69364 5.02991 3.81576 364 - -
2.68618 4.42225 2.77519 2.73123 3.46354 2.40513 3.72494 3.29354 2.67741 2.69355 4.24690 2.90347 2.73739 3.18146 2.89801 2.37887 2.77519 2.98518 4.58477 3.61503

0.01187 4.83543 5.55778 0.61958 0.77255 0.58262 0.81742

228 2.72360 4.37221 4.09900 3.51943 3.13839 3.57541 4.18799 2.83061 3.42503 1.35361 2.64318 3.80170 4.27309 2.99878 2.92347 2.80761 3.06596 2.39286 4.97653 3.77471 365 - -

2.68618 4.42225 2.77519 2.73123 3.46354 2.40513 3.72494 3.29354 2.67741 2.69355 4.24690 2.90347 2.73739 3.18146 2.89801 2.37887 2.77519 2.98518 4.58477 3.61503

0.03001 4.85176 3.82820 0.61958 0.77255 0.41499 1.07983

229 1.01813 4.47610 4.15503 3.60095 3.42211 3.86390 4.33381 2.93301 3.51909 2.40704 3.67815 3.86470 4.31836 3.78126 3.78192 2.88814 2.72631 2.36186 5.19060 3.98515 366 - -

2.68252 4.42346 2.77591 2.73228 3.46256 2.40538 3.72541 3.29445 2.67716 2.69359 4.24811 2.90336 2.73861 3.18194 2.89766 2.37916 2.77548 2.98434 4.58207 3.61624

1.09013 1.08525 1.12083 2.20310 0.11705 0.52849 0.89037

230 2.11058 4.82325 3.16779 2.60666 4.04535 3.55571 3.76118 3.44559 2.27408 2.45987 3.90386 2.68628 3.94465 2.93086 2.44630 2.76775 2.70090 2.73016 5.34083 4.02857 382 - -

2.68618 4.42225 2.77519 2.73123 3.46354 2.40513 3.72494 3.29354 2.67741 2.69355 4.24690 2.90347 2.73739 3.18146 2.89801 2.37887 2.77519 2.98518 4.58477 3.61503

0.01666 4.49866 5.22101 0.61958 0.77255 1.10425 0.40266

231 2.71608 4.24253 4.03110 3.45508 3.36471 2.87093 4.10567 2.51754 3.35663 1.79382 3.17642 3.72048 4.16903 3.60551 3.58913 2.34761 2.95446 1.80971 4.87450 3.67459 383 - -

2.68618 4.42225 2.77519 2.73123 3.46354 2.40513 3.72494 3.29354 2.67741 2.69355 4.24690 2.90347 2.73739 3.18146 2.89801 2.37887 2.77519 2.98518 4.58477 3.61503

0.01666 4.49866 5.22101 0.61958 0.77255 1.10425 0.40266

232 2.52255 3.69012 3.28856 2.72688 3.29115 3.59216 2.58706 3.20804 2.37256 2.87744 3.73618 3.20505 2.96678 3.03730 2.72550 2.81279 2.92710 2.82486 5.19328 2.91340 384 - -

2.68618 4.42225 2.77519 2.73123 3.46354 2.40513 3.72494 3.29354 2.67741 2.69355 4.24690 2.90347 2.73739 3.18146 2.89801 2.37887 2.77519 2.98518 4.58477 3.61503

0.01666 4.49866 5.22101 0.61958 0.77255 1.03570 0.43847

233 3.26991 4.57345 5.16575 4.58888 2.23683 4.56405 4.92142 2.04118 4.43702 1.57354 2.99946 4.68914 4.81918 4.52686 4.48128 3.90522 3.49974 1.19793 5.27516 4.14901 385 - -

2.68618 4.42225 2.77519 2.73123 3.46354 2.40513 3.72494 3.29354 2.67741 2.69355 4.24690 2.90347 2.73739 3.18146 2.89801 2.37887 2.77519 2.98518 4.58477 3.61503

0.01625 4.52390 5.24624 0.61958 0.77255 1.08045 0.41467

234 3.34080 4.61658 5.28876 4.72785 2.54257 4.69580 5.09349 1.43351 4.58801 1.51986 3.31808 4.83150 4.93431 4.68216 4.63603 4.05184 3.57457 1.40414 5.41874 4.28916 386 - -

2.68618 4.42225 2.77519 2.73123 3.46354 2.40513 3.72494 3.29354 2.67741 2.69355 4.24690 2.90347 2.73739 3.18146 2.89801 2.37887 2.77519 2.98518 4.58477 3.61503

0.01625 4.52390 5.24624 0.61958 0.77255 1.08045 0.41467

235 2.96583 4.56713 5.00123 4.42216 1.33743 4.45443 4.74795 2.13335 4.27082 1.49237 3.19180 4.54304 4.72614 4.37139 4.33689 3.78393 3.45572 2.17589 5.12691 3.95068 387 - -

2.68618 4.42225 2.77519 2.73123 3.46354 2.40513 3.72494 3.29354 2.67741 2.69355 4.24690 2.90347 2.73739 3.18146 2.89801 2.37887 2.77519 2.98518 4.58477 3.61503

0.01625 4.52390 5.24624 0.61958 0.77255 1.08045 0.41467

236 3.02857 4.37462 4.89527 4.30590 3.32480 4.25515 4.59304 1.62931 4.13113 1.61907 3.25940 4.37549 4.56962 4.26413 4.18357 3.57881 3.26075 1.48784 3.12546 3.87094 388 - -

2.68618 4.42225 2.77519 2.73123 3.46354 2.40513 3.72494 3.29354 2.67741 2.69355 4.24690 2.90347 2.73739 3.18146 2.89801 2.37887 2.77519 2.98518 4.58477 3.61503

0.01625 4.52390 5.24624 0.61958 0.77255 1.08045 0.41467

237 2.69959 4.56084 3.40513 2.84544 3.42985 2.77116 3.87873 3.11322 2.81151 2.13671 3.67532 3.29472 3.66646 3.13783 2.88650 1.82911 2.93874 2.72411 5.14075 3.88009 389 - -

2.68618 4.42225 2.77519 2.73123 3.46354 2.40513 3.72494 3.29354 2.67741 2.69355 4.24690 2.90347 2.73739 3.18146 2.89801 2.37887 2.77519 2.98518 4.58477 3.61503

0.07686 4.52390 2.76254 0.61958 0.77255 0.23863 1.54979

238 2.82401 5.25644 2.74467 2.54839 3.79916 3.60997 3.80003 3.57676 1.78383 3.36204 4.30560 3.09067 3.47904 2.90746 2.11014 2.17866 2.68070 3.63050 5.70787 4.31711 390 - -

2.68618 4.42225 2.77519 2.73123 3.46354 2.40513 3.72494 3.29354 2.67741 2.69355 4.24690 2.90347 2.73739 3.18146 2.89801 2.37887 2.77519 2.98518 4.58477 3.61503

0.09900 4.86462 2.44717 0.61958 0.77255 0.58231 0.81782

239 1.59423 1.94025 4.16129 3.60759 3.62801 3.79239 4.29851 2.72839 3.51738 2.69950 3.61835 2.97724 4.25993 3.76981 3.76088 3.11285 2.79345 2.15418 5.12308 3.92312 391 - -

2.68618 4.42225 2.77519 2.73123 3.46354 2.40513 3.72494 3.29354 2.67741 2.69355 4.24690 2.90347 2.73739 3.18146 2.89801 2.37887 2.77519 2.98518 4.58477 3.61503
0.01257 4.77820 5.50055 0.61958 0.77255 0.63435 0.75562
240 2.88278 4.36813 4.28032 3.70441 1.90100 2.90278 4.19408 2.68184 3.58876 2.54873 3.48049 3.91688 4.33534 3.00481 3.78110 3.25211 3.11490 2.25166 4.83045 1.79068 392 - -
2.68621 4.42228 2.77523 2.73126 3.46357 2.40516 3.72498 3.29357 2.67744 2.69358 4.24693 2.90350 2.73743 3.18149 2.89804 2.37877 2.77523 2.98510 4.58480 3.61449
0.45006 2.25679 1.35587 0.24246 1.53569 0.37578 1.16076
241 2.52528 4.49097 3.19677 3.02431 3.63877 3.71152 3.97135 2.84659 2.98312 2.04727 3.59914 3.43883 2.87608 3.28915 3.34664 2.39305 2.52302 2.08495 5.07744 3.83858 394 - -
2.68618 4.42225 2.77519 2.73123 3.46354 2.40513 3.72494 3.29354 2.67741 2.69355 4.24690 2.90347 2.73739 3.18146 2.89801 2.37887 2.77519 2.98518 4.58477 3.61503
0.05884 4.64206 3.04699 0.61958 0.77255 0.95123 0.48819
242 2.73000 4.98751 2.66589 2.54373 3.52187 2.68673 3.75904 3.68744 2.54675 3.27095 4.07025 2.45681 3.94099 2.89715 3.01783 2.10382 2.42236 2.80349 5.49442 4.14498 395 - -
2.68618 4.42225 2.77519 2.73123 3.46354 2.40513 3.72494 3.29354 2.67741 2.69355 4.24690 2.90347 2.73739 3.18146 2.89801 2.37887 2.77519 2.98518 4.58477 3.61503
0.01507 4.59830 5.32064 0.61958 0.77255 1.00287 0.45701
243 3.43056 4.72632 5.29368 4.71809 2.72127 4.72438 5.05998 1.32658 4.56784 1.06683 3.21627 4.84331 4.94438 4.62551 4.60489 4.07160 3.24921 2.36223 5.35423 4.25181 396 - -
2.68618 4.42225 2.77519 2.73123 3.46354 2.40513 3.72494 3.29354 2.67741 2.69355 4.24690 2.90347 2.73739 3.18146 2.89801 2.37887 2.77519 2.98518 4.58477 3.61503
0.06226 4.59830 2.98982 0.61958 0.77255 1.00287 0.45701
244 1.74291 5.13882 2.76776 2.14434 4.44609 3.50527 3.70284 3.43496 2.45530 3.42775 4.19169 2.98940 3.41518 2.40853 2.70696 2.70631 2.95730 3.50932 5.59524 4.20863 397 - -
2.68618 4.42225 2.77519 2.73123 3.46354 2.40513 3.72494 3.29354 2.67741 2.69355 4.24690 2.90347 2.73739 3.18146 2.89801 2.37887 2.77519 2.98518 4.58477 3.61503
0.01579 4.55183 5.27417 0.61958 0.77255 1.05270 0.42924
245 2.72376 5.05264 3.04905 2.49603 4.34252 2.82600 3.72176 3.78306 1.76978 2.88292 3.76898 2.80164 3.46884 2.84587 2.94772 2.09661 2.95648 3.41585 5.53269 4.16913 398 - -
2.68618 4.42225 2.77519 2.73123 3.46354 2.40513 3.72494 3.29354 2.67741 2.69355 4.24690 2.90347 2.73739 3.18146 2.89801 2.37887 2.77519 2.98518 4.58477 3.61503
0.01579 4.55183 5.27417 0.61958 0.77255 1.05270 0.42924
246 2.36566 4.70537 2.97455 2.35963 2.97430 3.59616 3.81662 2.95696 2.37694 2.70252 3.16470 3.19226 3.12966 3.02336 3.11930 2.58579 2.94335 2.70447 5.24620 3.95727 399 - -
2.68618 4.42225 2.77519 2.73123 3.46354 2.40513 3.72494 3.29354 2.67741 2.69355 4.24690 2.90347 2.73739 3.18146 2.89801 2.37887 2.77519 2.98518 4.58477 3.61503
0.07018 4.55183 2.86068 0.61958 0.77255 0.86190 0.54880
247 2.72084 4.95851 3.09911 2.54192 3.79060 2.93749 3.24749 3.63210 2.11760 3.04384 3.67153 3.06555 2.30047 2.88716 2.99988 2.34863 2.75639 3.29766 5.45424 4.11079 400 - -
2.68618 4.42225 2.77519 2.73123 3.46354 2.40513 3.72494 3.29354 2.67741 2.69355 4.24690 2.90347 2.73739 3.18146 2.89801 2.37887 2.77519 2.98518 4.58477 3.61503
0.01549 4.57133 5.29368 0.61958 0.77255 0.89038 0.52848
248 2.38408 5.04112 3.08035 2.52817 4.33759 3.04719 3.75384 3.77594 2.39582 3.14087 4.12603 2.02904 3.28134 2.88154 3.00207 2.23823 2.64587 3.06437 5.54360 4.18247 401 - -
2.68618 4.42225 2.77519 2.73123 3.46354 2.40513 3.72494 3.29354 2.67741 2.69355 4.24690 2.90347 2.73739 3.18146 2.89801 2.37887 2.77519 2.98518 4.58477 3.61503
0.05601 4.62117 3.10948 0.61958 0.77255 0.30179 1.34514
249 2.57848 3.93028 3.27530 2.71658 4.16818 3.67371 3.88291 3.57305 2.35690 2.83613 4.02700 2.22786 2.07590 2.74022 3.15681 2.88156 3.05633 2.72686 5.46715 3.62027 402 - -
2.68631 4.41763 2.77489 2.73151 3.46338 2.40502 3.72522 3.29363 2.67769 2.69326 4.24639 2.90375 2.73750 3.18174 2.89829 2.37851 2.77547 2.98546 4.58505 3.61531
0.59079 2.24671 1.07774 1.81103 0.17851 0.59703 0.79950
250 2.51554 4.91014 3.09619 2.53754 4.16360 3.52233 3.72035 3.57975 2.21330 2.32481 3.98656 2.78100 3.91412 2.86738 2.46838 2.19122 2.67032 3.25231 5.40874 4.07725 411 - -
2.68618 4.42225 2.77519 2.73123 3.46354 2.40513 3.72494 3.29354 2.67741 2.69355 4.24690 2.90347 2.73739 3.18146 2.89801 2.37887 2.77519 2.98518 4.58477 3.61503
0.01754 4.44760 5.16995 0.61958 0.77255 1.14904 0.38118

251 2.39210 5.18230 2.70374 2.39394 4.51960 3.46798 2.90557 3.99632 2.19116 3.48144 4.22667 2.68642 3.86373 2.30153 2.28799 2.50440 2.92983 3.56912 5.61671 4.21577 412 - -
2.68618 4.42225 2.77519 2.73123 3.46354 2.40513 3.72494 3.29354 2.67741 2.69355 4.24690 2.90347 2.73739 3.18146 2.89801 2.37887 2.77519 2.98518 4.58477 3.61503
0.01754 4.44760 5.16995 0.61958 0.77255 1.14904 0.38118
252 2.79223 4.53782 4.97561 4.39249 2.53406 4.43839 4.76784 1.99865 4.24622 0.98702 3.13532 4.52944 4.70217 4.34597 4.31557 3.76764 3.42730 2.13750 5.15576 4.03916 413 - -
2.68640 4.42279 2.77534 2.73080 3.46333 2.40526 3.72510 3.29383 2.67736 2.69246 4.24636 2.90400 2.73756 3.18151 2.89762 2.37940 2.77548 2.98489 4.58319 3.61512
1.09568 0.54791 2.43575 1.32935 0.30741 0.74357 0.64514
253 2.55337 5.16183 2.75250 2.18514 4.48178 3.49870 3.42076 3.94941 2.23921 2.81115 3.53207 2.97681 3.89152 2.59594 2.19737 2.36700 2.94662 3.53896 5.60918 4.21542 418 - -
2.68618 4.42225 2.77519 2.73123 3.46354 2.40513 3.72494 3.29354 2.67741 2.69355 4.24690 2.90347 2.73739 3.18146 2.89801 2.37887 2.77519 2.98518 4.58477 3.61503
0.04080 4.54988 3.52632 0.61958 0.77255 1.05468 0.42818
254 2.17461 4.65754 3.29817 2.73885 3.85137 2.64449 3.83175 3.05594 2.71760 2.57999 3.76052 3.21524 3.98448 2.48179 3.14068 2.46923 2.52426 2.60546 5.21832 3.93772 419 - -
2.68631 4.42238 2.77533 2.73137 3.46367 2.40526 3.72508 3.29367 2.67736 2.69368 4.24703 2.90332 2.73753 3.18086 2.89696 2.37888 2.77533 2.98532 4.58490 3.61516
1.17338 0.62561 1.85955 0.08500 2.50730 0.30005 1.35008
255 2.81306 3.46090 4.68600 4.07777 3.29000 3.97589 4.30604 1.59052 3.88332 1.90161 2.30663 4.10753 4.33846 4.03165 3.39437 3.28393 2.77860 2.19245 4.82530 3.23162 421 - -
2.68618 4.42225 2.77519 2.73123 3.46354 2.40513 3.72494 3.29354 2.67741 2.69355 4.24690 2.90347 2.73739 3.18146 2.89801 2.37887 2.77519 2.98518 4.58477 3.61503
0.01282 4.75893 5.48127 0.61958 0.77255 0.38233 1.14654
256 2.88276 5.29225 2.72323 2.61734 4.59737 3.12780 3.04926 2.83286 2.07234 3.58031 2.62932 3.15797 2.84486 2.70914 2.70606 2.70856 2.78359 3.32676 5.74959 4.36419 422 - -
2.68618 4.42225 2.77519 2.73123 3.46354 2.40513 3.72494 3.29354 2.67741 2.69355 4.24690 2.90347 2.73739 3.18146 2.89801 2.37887 2.77519 2.98518 4.58477 3.61503
0.15476 5.01329 1.98974 0.61958 0.77255 0.52849 0.89037
257 3.25811 5.87484 1.49687 1.38115 5.18339 3.68393 4.11302 4.70727 2.75132 4.16555 4.95617 2.06507 4.23862 3.24826 3.59647 2.52807 3.52219 4.25146 6.30067 4.80868 423 - -
2.68618 4.42225 2.77519 2.73123 3.46354 2.40513 3.72494 3.29354 2.67741 2.69355 4.24690 2.90347 2.73739 3.18146 2.89801 2.37887 2.77519 2.98518 4.58477 3.61503
0.01147 4.87000 5.59234 0.61958 0.77255 0.72246 0.66467
258 2.85908 3.54265 3.71469 3.82422 2.95298 3.97645 4.28672 1.99911 3.68940 1.47037 3.42757 3.09345 4.34490 3.89406 3.83917 2.37404 3.09137 2.40418 4.92694 3.73871 424 - -
2.68618 4.42225 2.77519 2.73123 3.46354 2.40513 3.72494 3.29354 2.67741 2.69355 4.24690 2.90347 2.73739 3.18146 2.89801 2.37887 2.77519 2.98518 4.58477 3.61503
0.09193 4.90053 2.52087 0.61958 0.77255 0.78467 0.60931
259 2.87897 5.21484 3.08795 2.00965 2.14404 2.94916 3.85397 3.93809 2.43318 3.48966 4.27926 3.13478 4.04899 1.81065 3.08162 2.87238 3.11188 3.57276 5.67981 4.31115 425 - -
2.68618 4.42225 2.77519 2.73123 3.46354 2.40513 3.72494 3.29354 2.67741 2.69355 4.24690 2.90347 2.73739 3.18146 2.89801 2.37887 2.77519 2.98518 4.58477 3.61503
0.03257 4.82065 3.73030 0.61958 0.77255 0.38317 1.14475
260 2.77582 5.21991 3.19138 2.48366 4.50507 3.08722 3.86578 3.67034 2.62595 2.94274 4.27791 2.94601 3.41102 2.85542 1.40653 2.76814 2.75265 3.36967 5.69039 4.32113 426 - -
2.68618 4.42225 2.77519 2.73123 3.46354 2.40513 3.72494 3.29354 2.67741 2.69355 4.24690 2.90347 2.73739 3.18146 2.89801 2.37887 2.77519 2.98518 4.58477 3.61503
0.50083 4.97387 0.94918 0.61958 0.77255 0.55502 0.85346
261 2.69076 2.20616 3.40394 2.60212 3.80957 2.40844 3.89665 3.18188 2.80731 2.86816 3.73622 3.29449 4.00785 3.14467 2.80264 2.83917 2.42490 2.67744 5.19800 3.93604 427 - -
2.68618 4.42225 2.77519 2.73123 3.46354 2.40513 3.72494 3.29354 2.67741 2.69355 4.24690 2.90347 2.73739 3.18146 2.89801 2.37887 2.77519 2.98518 4.58477 3.61503
0.01635 4.51741 5.23976 0.61958 0.77255 0.41444 1.08090
262 2.27624 4.89759 2.44856 2.78398 3.12585 3.70889 3.92382 3.04766 2.43600 2.10175 3.97967 3.03352 3.58170 2.73916 3.21346 2.75065 3.07336 2.71734 5.42764 4.12526 428 - -
2.68505 4.42365 2.77431 2.73018 3.46419 2.40605 3.72588 3.29373 2.67753 2.69313 4.24672 2.90461 2.73864 3.18116 2.89821 2.37829 2.77451 2.98538 4.58526 3.61554

0.55030 0.86843 5.62287 2.13055 0.12644 0.38233 1.14654
263 2.88275 5.30081 1.80800 1.95158 4.60904 3.26987 3.86289 3.75655 2.61383 3.38257 3.27595 3.15475 4.06405 2.64807 2.91276 2.55052 3.11395 2.78582 5.75618 3.75446 443 - -
2.68618 4.42225 2.77519 2.73123 3.46354 2.40513 3.72494 3.29354 2.67741 2.69355 4.24690 2.90347 2.73739 3.18146 2.89801 2.37887 2.77519 2.98518 4.58477 3.61503
0.00993 5.01329 5.73564 0.61958 0.77255 0.45994 0.99783
264 2.98077 3.97428 4.65599 4.05842 2.27795 3.78551 4.43209 2.61394 3.60205 1.40291 2.41715 4.17706 4.48184 3.19900 3.05637 2.72446 3.21287 2.49379 5.02331 3.83950 444 - -
2.68578 4.42449 2.77530 2.73081 3.46427 2.40614 3.72555 3.29322 2.67594 2.69278 4.24106 2.90502 2.73681 3.18066 2.89791 2.37919 2.77596 2.98460 4.58701 3.61727
0.57438 0.89014 3.63641 2.38704 0.09640 0.48576 0.95510
265 2.96746 4.44213 3.97170 3.13574 3.54595 4.07126 4.37479 2.15876 3.47577 1.69264 3.22548 4.05470 4.04596 3.94772 3.90703 2.69874 3.04911 1.47178 5.05474 3.86307 462 - -
2.68618 4.42225 2.77519 2.73123 3.46354 2.40513 3.72494 3.29354 2.67741 2.69355 4.24690 2.90347 2.73739 3.18146 2.89801 2.37887 2.77519 2.98518 4.58477 3.61503
0.09856 5.12430 2.43150 0.61958 0.77255 0.55987 0.84695
266 2.01501 3.38972 3.47237 2.91113 4.06641 3.79222 4.01401 3.45230 2.29941 3.11580 3.35232 3.39491 4.17837 3.22588 2.37004 1.98658 3.13549 2.96395 5.42536 2.81846 463 - -
2.68633 4.42240 2.77513 2.73076 3.46369 2.40528 3.72510 3.29369 2.67656 2.69352 4.24705 2.90362 2.73755 3.18139 2.89799 2.37902 2.77535 2.98534 4.58492 3.61518
0.46173 1.00335 5.75780 0.07561 2.61973 0.78467 0.60931
267 1.86223 4.45008 4.20613 3.62569 3.56038 3.49501 4.27926 2.90829 3.52814 1.82655 2.86460 3.90001 4.36212 3.77815 2.09797 3.01157 3.15124 2.40235 3.36176 3.85490 465 - -
2.68618 4.42225 2.77519 2.73123 3.46354 2.40513 3.72494 3.29354 2.67741 2.69355 4.24690 2.90347 2.73739 3.18146 2.89801 2.37887 2.77519 2.98518 4.58477 3.61503
0.00971 5.03545 5.75780 0.61958 0.77255 0.78467 0.60931
268 2.92683 5.38096 3.19845 2.38413 4.71410 3.70206 3.49858 4.18103 1.48706 3.67441 4.42742 3.17755 4.09308 2.38843 2.65241 2.58433 2.91454 2.29180 5.81461 4.42674 466 - -
2.68618 4.42225 2.77519 2.73123 3.46354 2.40513 3.72494 3.29354 2.67741 2.69355 4.24690 2.90347 2.73739 3.18146 2.89801 2.37887 2.77519 2.98518 4.58477 3.61503
0.05671 5.03545 3.02359 0.61958 0.77255 0.78467 0.60931
269 2.56562 5.33200 2.68672 2.30010 4.65636 2.54831 3.84302 4.12798 2.58630 2.43009 3.73620 2.85273 4.04662 2.62667 2.57155 2.60574 2.30575 3.71147 5.77728 4.37916 467 - -
2.68618 4.42225 2.77519 2.73123 3.46354 2.40513 3.72494 3.29354 2.67741 2.69355 4.24690 2.90347 2.73739 3.18146 2.89801 2.37887 2.77519 2.98518 4.58477 3.61503
0.01017 4.98892 5.71126 0.61958 0.77255 0.68313 0.70327
270 2.54498 5.36442 2.19912 2.24777 3.70917 3.67075 3.85973 3.66010 2.46066 3.35880 4.40922 2.59326 4.06402 2.63100 3.09098 1.77218 3.12256 3.42397 5.80652 4.40509 468 - -
2.68618 4.42225 2.77519 2.73123 3.46354 2.40513 3.72494 3.29354 2.67741 2.69355 4.24690 2.90347 2.73739 3.18146 2.89801 2.37887 2.77519 2.98518 4.58477 3.61503
0.00971 5.03545 5.75780 0.61958 0.77255 0.78467 0.60931
271 2.92115 4.37874 4.45756 3.86702 2.49595 4.03521 2.91588 2.51984 3.33445 1.58231 3.49439 4.04488 3.50014 3.46092 3.39648 2.83727 3.15321 2.32133 3.50503 2.94198 469 - -
2.68618 4.42225 2.77519 2.73123 3.46354 2.40513 3.72494 3.29354 2.67741 2.69355 4.24690 2.90347 2.73739 3.18146 2.89801 2.37887 2.77519 2.98518 4.58477 3.61503
0.00971 5.03545 5.75780 0.61958 0.77255 0.78467 0.60931
272 2.92132 4.43606 3.64614 3.67293 3.20722 4.00089 3.65127 2.89153 3.57241 2.29996 2.86207 3.50451 4.37221 3.81239 3.33983 3.00930 3.15328 2.45946 1.78025 2.20053 470 - -
2.68618 4.42225 2.77519 2.73123 3.46354 2.40513 3.72494 3.29354 2.67741 2.69355 4.24690 2.90347 2.73739 3.18146 2.89801 2.37887 2.77519 2.98518 4.58477 3.61503
0.00971 5.03545 5.75780 0.61958 0.77255 0.78467 0.60931
273 2.94415 4.72097 1.79651 2.93391 3.87587 3.89394 4.14837 2.31803 3.13348 2.10456 3.82300 2.99831 4.27939 3.44311 3.51316 3.14056 3.17710 1.93707 5.31199 4.06390 471 - -
2.68618 4.42225 2.77519 2.73123 3.46354 2.40513 3.72494 3.29354 2.67741 2.69355 4.24690 2.90347 2.73739 3.18146 2.89801 2.37887 2.77519 2.98518 4.58477 3.61503
0.00971 5.03545 5.75780 0.61958 0.77255 0.78467 0.60931
274 2.50352 2.25268 4.71777 4.11425 3.42564 4.07458 4.40123 1.90088 3.93541 1.93702 3.44839 3.61435 4.44111 4.09922 4.00451 3.10261 2.92208 2.57441 4.95007 2.07508 472 - -

2.68618 4.42225 2.77519 2.73123 3.46354 2.40513 3.72494 3.29354 2.67741 2.69355 4.24690 2.90347 2.73739 3.18146 2.89801 2.37887 2.77519 2.98518 4.58477 3.61503
0.00971 5.03545 5.75780 0.61958 0.77255 0.78467 0.60931
275 2.56790 5.39304 3.15526 2.31171 4.73376 3.67435 3.40748 4.21379 1.66092 2.53963 4.43553 2.83780 3.30120 2.63904 2.31607 2.69826 3.13027 3.78149 5.82665 4.42247 473 - -
2.68618 4.42225 2.77519 2.73123 3.46354 2.40513 3.72494 3.29354 2.67741 2.69355 4.24690 2.90347 2.73739 3.18146 2.89801 2.37887 2.77519 2.98518 4.58477 3.61503
0.14604 5.03545 2.04505 0.61958 0.77255 0.78467 0.60931
276 2.37657 5.01648 3.26023 2.39908 4.26834 3.68056 3.91601 3.67788 2.72921 2.49286 4.11499 2.86635 2.15325 3.07615 3.18618 2.90262 1.87064 3.36054 5.54941 4.22314 474 - -
2.68618 4.42225 2.77519 2.73123 3.46354 2.40513 3.72494 3.29354 2.67741 2.69355 4.24690 2.90347 2.73739 3.18146 2.89801 2.37887 2.77519 2.98518 4.58477 3.61503
0.01112 4.90053 5.62287 0.61958 0.77255 1.02641 0.44362
277 2.84651 4.66836 3.20761 2.63158 2.77649 3.78657 4.02577 2.75272 2.61820 2.66594 3.39005 2.54954 4.16884 3.30226 3.37466 3.01913 2.14433 2.25048 5.23612 3.31974 475 - -
2.68618 4.42225 2.77519 2.73123 3.46354 2.40513 3.72494 3.29354 2.67741 2.69355 4.24690 2.90347 2.73739 3.18146 2.89801 2.37887 2.77519 2.98518 4.58477 3.61503
0.01112 4.90053 5.62287 0.61958 0.77255 1.02641 0.44362
278 1.59631 4.37421 3.41470 3.73545 3.48625 3.99649 4.30255 2.28926 3.62412 1.81496 3.06998 3.96156 4.36455 3.85195 3.82016 3.28177 2.66278 2.14450 5.00462 3.81060 476 - -
2.68618 4.42225 2.77519 2.73123 3.46354 2.40513 3.72494 3.29354 2.67741 2.69355 4.24690 2.90347 2.73739 3.18146 2.89801 2.37887 2.77519 2.98518 4.58477 3.61503
0.01112 4.90053 5.62287 0.61958 0.77255 1.02641 0.44362
279 2.83662 5.24172 2.69562 2.57315 4.54519 3.26106 3.19151 4.00283 2.15201 3.12675 3.30937 2.91464 3.37977 2.47007 3.05775 2.63860 2.53929 3.03255 5.70014 2.72527 477 - -
2.68618 4.42225 2.77519 2.73123 3.46354 2.40513 3.72494 3.29354 2.67741 2.69355 4.24690 2.90347 2.73739 3.18146 2.89801 2.37887 2.77519 2.98518 4.58477 3.61503
0.01112 4.90053 5.62287 0.61958 0.77255 1.02641 0.44362
280 2.95579 5.37518 3.24867 2.66998 4.72604 3.71757 3.85632 4.17532 1.55342 3.65873 4.42499 2.65305 4.10238 2.61260 1.88971 2.92859 2.48279 2.93967 5.78334 4.42713 478 - -
2.68618 4.42225 2.77519 2.73123 3.46354 2.40513 3.72494 3.29354 2.67741 2.69355 4.24690 2.90347 2.73739 3.18146 2.89801 2.37887 2.77519 2.98518 4.58477 3.61503
0.01112 4.90053 5.62287 0.61958 0.77255 1.02641 0.44362
281 3.05740 4.56482 4.34732 2.38880 2.87898 4.14263 4.42778 2.81708 3.67544 0.98225 3.01132 4.05525 4.48971 3.91047 3.90120 3.42584 2.93687 2.72011 5.14114 3.96351 479 - -
2.68618 4.42225 2.77519 2.73123 3.46354 2.40513 3.72494 3.29354 2.67741 2.69355 4.24690 2.90347 2.73739 3.18146 2.89801 2.37887 2.77519 2.98518 4.58477 3.61503
0.01112 4.90053 5.62287 0.61958 0.77255 1.02641 0.44362
282 3.86499 5.93183 4.51004 3.55580 5.58516 4.38845 4.11029 4.81382 1.13957 4.11057 4.99917 3.87213 4.68860 2.33644 1.00810 3.82322 3.94942 4.50329 6.03782 4.98293 480 - -
2.68618 4.42225 2.77519 2.73123 3.46354 2.40513 3.72494 3.29354 2.67741 2.69355 4.24690 2.90347 2.73739 3.18146 2.89801 2.37887 2.77519 2.98518 4.58477 3.61503
0.01112 4.90053 5.62287 0.61958 0.77255 1.02641 0.44362
283 1.85120 5.45196 2.28117 2.40668 4.78316 3.64276 3.89006 4.26774 2.48157 3.75187 4.50545 3.10999 4.07738 1.56943 3.16230 2.65716 3.18672 3.83694 5.89050 4.47232 481 - -
2.68618 4.42225 2.77519 2.73123 3.46354 2.40513 3.72494 3.29354 2.67741 2.69355 4.24690 2.90347 2.73739 3.18146 2.89801 2.37887 2.77519 2.98518 4.58477 3.61503
0.01112 4.90053 5.62287 0.61958 0.77255 1.02641 0.44362
284 2.85782 4.63044 3.65888 3.08989 3.77679 3.81392 3.44304 2.58499 2.06500 2.15426 2.08584 3.51536 4.19402 3.35399 2.86280 2.84826 2.68292 2.90934 5.20290 3.96020 482 - -
2.68618 4.42225 2.77519 2.73123 3.46354 2.40513 3.72494 3.29354 2.67741 2.69355 4.24690 2.90347 2.73739 3.18146 2.89801 2.37887 2.77519 2.98518 4.58477 3.61503
0.01112 4.90053 5.62287 0.61958 0.77255 1.02641 0.44362
285 3.00140 4.37739 4.86942 4.26508 2.69847 4.17935 4.51114 2.05105 4.07733 1.05882 3.13295 3.65257 4.52541 4.21550 4.11968 3.49120 2.91630 2.24590 5.00596 3.84023 483 - -
2.68618 4.42225 2.77519 2.73123 3.46354 2.40513 3.72494 3.29354 2.67741 2.69355 4.24690 2.90347 2.73739 3.18146 2.89801 2.37887 2.77519 2.98518 4.58477 3.61503
0.01112 4.90053 5.62287 0.61958 0.77255 0.66092 0.72645

286 2.88020 5.16370 2.80359 2.48895 3.44280 3.69403 3.47326 3.85762 2.67216 2.34151 4.22718 3.20477 3.57693 1.76424 3.14545 2.41820 2.90145 3.32241 5.64927 3.23905 484 - -
2.68618 4.42225 2.77519 2.73123 3.46354 2.40513 3.72494 3.29354 2.67741 2.69355 4.24690 2.90347 2.73739 3.18146 2.89801 2.37887 2.77519 2.98518 4.58477 3.61503
0.08483 5.00158 2.59556 0.61958 0.77255 0.30946 1.32367
287 2.22851 4.94468 3.35751 2.97638 4.12829 3.85729 4.07969 3.51412 2.57543 1.58508 2.45574 3.20941 3.24918 3.03030 3.38548 2.68408 2.79766 3.24545 5.48852 4.20332 485 - -
2.68618 4.42225 2.77519 2.73123 3.46354 2.40513 3.72494 3.29354 2.67741 2.69355 4.24690 2.90347 2.73739 3.18146 2.89801 2.37887 2.77519 2.98518 4.58477 3.61503
0.47988 5.17497 0.97953 0.61958 0.77255 0.42521 1.06026
288 2.34253 4.36965 3.68770 3.41436 2.87880 3.55714 4.13595 2.83462 3.33390 1.99898 2.84785 3.72367 4.22828 2.83280 3.60062 2.95690 2.09806 2.21998 4.97084 3.51709 486 - -
2.68583 4.42401 2.77552 2.72891 3.46369 2.40625 3.72471 3.29503 2.67560 2.69413 4.24646 2.90373 2.73867 3.17751 2.89759 2.37955 2.77567 2.98535 4.58653 3.61645
1.16386 0.49488 2.55003 2.11837 0.12809 0.20120 1.70237
289 1.83205 5.40874 2.89247 2.28115 4.73051 2.93034 3.71107 3.51702 2.59931 3.70449 4.45669 3.21511 2.11372 3.03413 3.16376 2.44209 2.58268 3.78703 5.85671 4.46031 497 - -
2.68618 4.42225 2.77519 2.73123 3.46354 2.40513 3.72494 3.29354 2.67741 2.69355 4.24690 2.90347 2.73739 3.18146 2.89801 2.37887 2.77519 2.98518 4.58477 3.61503
0.00843 5.17542 5.89777 0.61958 0.77255 0.66804 0.71890
290 2.38089 4.45425 4.51521 3.92537 3.55585 4.10489 4.41193 2.78146 3.79593 1.70631 3.23573 4.10911 2.40564 3.73286 3.70328 2.98923 2.42737 1.79890 5.06725 3.29153 498 - -
2.68618 4.42225 2.77519 2.73123 3.46354 2.40513 3.72494 3.29354 2.67741 2.69355 4.24690 2.90347 2.73739 3.18146 2.89801 2.37887 2.77519 2.98518 4.58477 3.61503
0.00843 5.17542 5.89777 0.61958 0.77255 0.66804 0.71890
291 2.98898 4.62426 4.02092 2.84872 2.30720 2.07026 4.27428 2.84985 3.13894 2.66560 3.00147 3.80949 3.68233 2.85169 3.69961 2.99220 3.07052 2.01502 5.21714 3.24906 499 - -
2.68618 4.42225 2.77519 2.73123 3.46354 2.40513 3.72494 3.29354 2.67741 2.69355 4.24690 2.90347 2.73739 3.18146 2.89801 2.37887 2.77519 2.98518 4.58477 3.61503
0.00834 5.18707 5.90942 0.61958 0.77255 0.61829 0.77406
292 2.71578 4.87792 3.41708 2.56133 3.51144 3.89529 4.12561 3.03082 2.91709 2.05728 3.67846 3.14981 3.93424 3.36828 3.45228 1.54095 2.93014 3.03216 5.43437 3.08427 500 - -
2.68618 4.42225 2.77519 2.73123 3.46354 2.40513 3.72494 3.29354 2.67741 2.69355 4.24690 2.90347 2.73739 3.18146 2.89801 2.37887 2.77519 2.98518 4.58477 3.61503
0.00826 5.19626 5.91860 0.61958 0.77255 0.64154 0.74756
293 2.78930 2.24944 4.69558 4.09731 2.55659 3.23887 3.93009 2.05650 3.37299 2.53230 2.67832 3.58713 4.50934 4.12020 3.55970 2.82680 2.37296 2.67955 3.98758 3.27951 501 - -
2.68618 4.42225 2.77519 2.73123 3.46354 2.40513 3.72494 3.29354 2.67741 2.69355 4.24690 2.90347 2.73739 3.18146 2.89801 2.37887 2.77519 2.98518 4.58477 3.61503
0.12111 5.19626 2.22072 0.61958 0.77255 0.39939 1.11088
294 2.57185 5.41265 3.20157 2.01607 4.74277 3.71853 2.92630 4.21752 2.64547 3.14627 4.45761 2.71649 4.11195 1.48542 2.94009 2.90975 2.94695 3.34861 5.85387 4.45327 502 - -
2.68618 4.42225 2.77519 2.73123 3.46354 2.40513 3.72494 3.29354 2.67741 2.69355 4.24690 2.90347 2.73739 3.18146 2.89801 2.37887 2.77519 2.98518 4.58477 3.61503
0.01977 5.13787 4.29008 0.61958 0.77255 0.41840 1.07324
295 3.01626 4.43577 4.07874 4.12296 3.12486 4.15490 4.47393 1.90834 3.47994 1.19162 3.18009 3.87534 4.52123 4.13970 3.33031 3.45417 2.63781 2.33441 5.05337 3.87057 503 - -
2.68618 4.42225 2.77519 2.73123 3.46354 2.40513 3.72494 3.29354 2.67741 2.69355 4.24690 2.90347 2.73739 3.18146 2.89801 2.37887 2.77519 2.98518 4.58477 3.61503
0.07042 5.21134 2.77183 0.61958 0.77255 0.54037 0.87355
296 1.49397 5.45978 2.72575 2.00763 4.79492 3.73327 3.93640 4.27444 2.61354 3.76256 4.50782 2.37475 4.13635 3.03835 3.17730 2.28511 2.98504 3.39668 5.90187 4.49475 504 - -
2.68618 4.42225 2.77519 2.73123 3.46354 2.40513 3.72494 3.29354 2.67741 2.69355 4.24690 2.90347 2.73739 3.18146 2.89801 2.37887 2.77519 2.98518 4.58477 3.61503
0.12776 5.16024 2.16984 0.61958 0.77255 0.74036 0.64806
297 1.53883 4.90740 3.28029 2.90536 4.11463 3.78155 4.02192 3.50339 2.88988 2.43235 4.01124 2.81144 3.03038 3.22585 3.32275 2.43199 2.66504 2.58099 5.46431 4.17400 505 - -
2.68618 4.42225 2.77519 2.73123 3.46354 2.40513 3.72494 3.29354 2.67741 2.69355 4.24690 2.90347 2.73739 3.18146 2.89801 2.37887 2.77519 2.98518 4.58477 3.61503

0.02345 5.04212 4.09149 0.61958 0.77255 0.99179 0.46349

298 2.21275 5.38070 3.15365 2.34150 4.71737 3.67177 3.42941 4.19515 1.98420 3.31889 4.42422 3.14318 2.08264 2.69611 2.71847 2.33976 3.12657 3.76696 5.81711 4.41461 506 - -

2.68585 4.42253 2.77547 2.73111 3.46284 2.40474 3.72522 3.29382 2.67768 2.69293 4.24717 2.90374 2.73767 3.18031 2.89828 2.37914 2.77547 2.98546 4.58505 3.61531

0.40081 1.55653 2.12562 1.09780 0.40587 1.01558 0.44972

299 2.07931 4.66715 3.58722 2.62902 3.82045 3.33759 4.02979 2.70305 2.99051 2.88650 3.53790 3.46613 4.17207 3.12496 3.37952 2.31822 2.08090 2.75717 2.84041 3.98273 515 - -

2.68618 4.42225 2.77519 2.73123 3.46354 2.40513 3.72494 3.29354 2.67741 2.69355 4.24690 2.90347 2.73739 3.18146 2.89801 2.37887 2.77519 2.98518 4.58477 3.61503

0.12691 4.90495 2.19127 0.61958 0.77255 1.19659 0.35986

300 2.44620 4.20731 4.65064 4.04386 2.32970 3.96872 4.29569 1.74485 3.85604 2.11672 2.87175 4.08869 4.33314 4.01072 3.21086 3.27456 2.60653 2.19499 4.82788 3.00799 516 - -

2.68618 4.42225 2.77519 2.73123 3.46354 2.40513 3.72494 3.29354 2.67741 2.69355 4.24690 2.90347 2.73739 3.18146 2.89801 2.37887 2.77519 2.98518 4.58477 3.61503

0.01242 4.79047 5.51281 0.61958 0.77255 1.32433 0.30922

301 3.04614 5.27349 3.33178 2.80503 4.57030 3.77677 3.91994 4.00796 2.50161 3.54607 4.36707 3.13026 1.21867 2.62518 2.22137 3.05349 3.26568 3.66739 5.69604 3.82262 517 - -

2.68618 4.42225 2.77519 2.73123 3.46354 2.40513 3.72494 3.29354 2.67741 2.69355 4.24690 2.90347 2.73739 3.18146 2.89801 2.37887 2.77519 2.98518 4.58477 3.61503

0.01242 4.79047 5.51281 0.61958 0.77255 1.32433 0.30922

302 3.04009 5.55448 2.46377 2.55683 4.89937 3.65046 3.91945 4.38265 1.23073 3.84589 4.61530 1.98711 4.11439 2.55297 3.11587 2.97908 3.27820 3.94984 5.96460 4.55290 518 - -

2.68618 4.42225 2.77519 2.73123 3.46354 2.40513 3.72494 3.29354 2.67741 2.69355 4.24690 2.90347 2.73739 3.18146 2.89801 2.37887 2.77519 2.98518 4.58477 3.61503

0.01242 4.79047 5.51281 0.61958 0.77255 0.88746 0.53052

303 2.55328 5.33205 3.09830 2.56622 4.66444 1.94702 3.27068 4.13815 2.43711 3.63534 4.38551 2.32614 3.15578 2.75301 3.07118 2.05709 3.10104 3.71790 5.78158 4.38108 519 - -

2.68618 4.42225 2.77519 2.73123 3.46354 2.40513 3.72494 3.29354 2.67741 2.69355 4.24690 2.90347 2.73739 3.18146 2.89801 2.37887 2.77519 2.98518 4.58477 3.61503

0.01082 4.92796 5.65031 0.61958 0.77255 1.10395 0.40281

304 2.02646 4.73313 3.19220 2.60906 3.89509 3.78870 4.02252 2.57219 2.73629 1.64141 3.82796 3.11485 4.17244 3.27757 3.18024 3.01726 3.09713 2.64196 5.29415 4.03138 520 - -

2.68618 4.42225 2.77519 2.73123 3.46354 2.40513 3.72494 3.29354 2.67741 2.69355 4.24690 2.90347 2.73739 3.18146 2.89801 2.37887 2.77519 2.98518 4.58477 3.61503

0.01065 4.94307 5.66541 0.61958 0.77255 1.14642 0.38239

305 2.85863 5.23569 2.32158 2.33541 4.52899 3.65241 1.75255 3.98026 2.60431 3.51839 3.08519 3.14329 4.04536 2.75513 2.86118 2.84831 3.08984 2.93284 5.69981 3.77679 521 - -

2.68618 4.42225 2.77519 2.73123 3.46354 2.40513 3.72494 3.29354 2.67741 2.69355 4.24690 2.90347 2.73739 3.18146 2.89801 2.37887 2.77519 2.98518 4.58477 3.61503

0.01065 4.94307 5.66541 0.61958 0.77255 1.14642 0.38239

306 2.87499 1.24926 3.82983 3.28814 3.92931 3.78700 4.22510 3.28128 2.69618 2.99963 3.88549 2.79288 4.25386 3.53931 3.54854 2.92423 2.57948 2.50280 5.35713 4.12547 522 - -

2.68618 4.42225 2.77519 2.73123 3.46354 2.40513 3.72494 3.29354 2.67741 2.69355 4.24690 2.90347 2.73739 3.18146 2.89801 2.37887 2.77519 2.98518 4.58477 3.61503

0.01065 4.94307 5.66541 0.61958 0.77255 0.97088 0.47602

307 2.41009 4.56033 3.86182 2.86549 3.69467 3.58892 4.16103 3.05120 3.23930 1.29057 3.67176 3.67228 3.84854 3.53108 3.37736 2.86075 2.53773 2.35587 5.15449 3.93018 523 - -

2.68618 4.42225 2.77519 2.73123 3.46354 2.40513 3.72494 3.29354 2.67741 2.69355 4.24690 2.90347 2.73739 3.18146 2.89801 2.37887 2.77519 2.98518 4.58477 3.61503

0.01020 4.98643 5.70878 0.61958 0.77255 0.95940 0.48309

308 2.22450 4.95324 3.38813 2.82876 4.15681 3.40956 3.97125 3.55325 2.55309 3.04397 4.03587 3.15437 3.62136 3.15658 3.10128 1.82436 2.39946 2.27294 5.48305 3.31366 524 - -

2.68644 4.42300 2.77566 2.73199 3.46429 2.40582 3.72570 3.29363 2.67774 2.69101 4.24174 2.90373 2.73815 3.18180 2.89660 2.37931 2.77410 2.98545 4.58494 3.61578

0.76717 0.75504 2.72288 1.32011 0.31075 0.90039 0.52157

309 2.67468 5.35141 2.55164 2.21309 4.68468 3.33015 3.83570 3.44106 2.35885 3.34324 3.10191 2.91076 3.52411 2.54988 2.56926 2.40320 2.50669 3.23115 5.79122 4.38749 529 - -

2.68618 4.42225 2.77519 2.73123 3.46354 2.40513 3.72494 3.29354 2.67741 2.69355 4.24690 2.90347 2.73739 3.18146 2.89801 2.37887 2.77519 2.98518 4.58477 3.61503

0.08075 4.98393 2.64888 0.61958 0.77255 0.83215 0.57113

310 2.87676 5.20810 2.16721 1.50948 4.48614 3.67792 3.87665 3.09503 2.64623 2.74980 3.91131 3.17810 4.07131 3.00089 3.12467 2.33906 3.10838 3.35088 5.68475 4.31794 530 - -

2.68618 4.42225 2.77519 2.73123 3.46354 2.40513 3.72494 3.29354 2.67741 2.69355 4.24690 2.90347 2.73739 3.18146 2.89801 2.37887 2.77519 2.98518 4.58477 3.61503

0.04572 4.98097 3.27494 0.61958 0.77255 1.09180 0.40889

311 2.85864 5.29655 2.87701 2.30629 4.61319 3.64296 3.03156 4.07891 2.32831 3.58900 4.34502 2.88676 4.03566 2.93860 2.13346 2.83519 2.55051 3.67203 3.66120 2.20982 531 - -

2.68592 4.42346 2.77560 2.73072 3.46257 2.40621 3.72359 3.29363 2.67753 2.69276 4.24590 2.90329 2.73752 3.18199 2.89722 2.37854 2.77588 2.98593 4.58598 3.61473

0.57281 0.83793 5.66822 1.92383 0.15788 0.77039 0.62145

312 2.43724 5.39918 2.34702 2.37277 3.92014 2.60108 3.85666 4.22576 1.96588 3.49575 4.44056 2.94678 4.06273 2.05977 2.86758 2.85819 2.93830 3.57567 5.83340 4.42403 540 - -

2.68618 4.42225 2.77519 2.73123 3.46354 2.40513 3.72494 3.29354 2.67741 2.69355 4.24690 2.90347 2.73739 3.18146 2.89801 2.37887 2.77519 2.98518 4.58477 3.61503

0.06099 5.04467 2.94267 0.61958 0.77255 0.98726 0.46617

313 2.52180 4.44829 2.48291 3.21334 2.74666 3.95752 4.24239 2.55363 3.46307 1.57670 3.24506 3.84387 3.63859 3.71861 3.71735 3.07202 2.87370 2.25316 5.05156 3.84737 541 - -

2.68618 4.42225 2.77519 2.73123 3.46354 2.40513 3.72494 3.29354 2.67741 2.69355 4.24690 2.90347 2.73739 3.18146 2.89801 2.37887 2.77519 2.98518 4.58477 3.61503

0.19352 4.99380 1.77689 0.61958 0.77255 0.55174 0.85789

314 2.66655 5.34535 3.12932 2.27817 4.67713 2.39499 3.83560 4.15330 1.99634 3.64497 4.39013 2.65492 2.76200 2.93663 2.66517 2.74044 2.63443 2.82276 5.78645 4.38431 542 - -

2.68618 4.42225 2.77519 2.73123 3.46354 2.40513 3.72494 3.29354 2.67741 2.69355 4.24690 2.90347 2.73739 3.18146 2.89801 2.37887 2.77519 2.98518 4.58477 3.61503

0.04915 4.97767 3.19235 0.61958 0.77255 1.09676 0.40639

315 2.37695 5.25722 2.88125 2.35177 3.52840 3.64341 3.60265 4.01812 2.26468 3.07086 4.30920 3.12922 2.76799 2.94744 3.07387 2.04641 2.21904 3.62526 5.71580 4.33152 543 - -

2.68618 4.42225 2.77519 2.73123 3.46354 2.40513 3.72494 3.29354 2.67741 2.69355 4.24690 2.90347 2.73739 3.18146 2.89801 2.37887 2.77519 2.98518 4.58477 3.61503

0.13261 4.93921 2.14528 0.61958 0.77255 0.99545 0.46133

316 2.36817 5.01553 3.23077 2.13621 2.60625 3.65806 3.86274 3.66613 2.66579 3.27268 4.08714 3.19152 2.96680 2.57348 2.92031 2.22262 3.05592 2.91323 5.52016 3.34681 544 - -

2.68618 4.42225 2.77519 2.73123 3.46354 2.40513 3.72494 3.29354 2.67741 2.69355 4.24690 2.90347 2.73739 3.18146 2.89801 2.37887 2.77519 2.98518 4.58477 3.61503

0.01156 4.86196 5.58430 0.61958 0.77255 0.95138 0.48810

317 2.76135 5.32205 2.87141 2.11185 4.64947 2.49901 3.58286 3.78849 2.56572 2.72207 4.36750 2.85560 4.02776 2.92611 2.32194 2.09582 2.71277 3.70319 5.76546 4.36542 545 - -

2.68618 4.42225 2.77519 2.73123 3.46354 2.40513 3.72494 3.29354 2.67741 2.69355 4.24690 2.90347 2.73739 3.18146 2.89801 2.37887 2.77519 2.98518 4.58477 3.61503

0.10580 4.94499 2.37223 0.61958 0.77255 0.38161 1.14809

318 2.63990 5.36953 2.81805 2.63969 3.92343 3.07262 3.47327 4.15755 2.32409 3.32638 4.41696 3.18338 2.40074 2.89445 2.18615 2.13490 3.03685 3.05695 5.81774 4.00375 546 - -

2.68618 4.42225 2.77519 2.73123 3.46354 2.40513 3.72494 3.29354 2.67741 2.69355 4.24690 2.90347 2.73739 3.18146 2.89801 2.37887 2.77519 2.98518 4.58477 3.61503

0.03277 5.10510 3.64299 0.61958 0.77255 0.54030 0.87365

319 2.64310 5.41773 3.09175 2.06544 4.18686 2.94852 3.91270 3.53369 2.65362 3.21241 4.46246 2.32429 2.63412 2.90789 2.25625 2.44464 2.95492 3.47342 5.85973 4.45825 547 - -

2.68618 4.42225 2.77519 2.73123 3.46354 2.40513 3.72494 3.29354 2.67741 2.69355 4.24690 2.90347 2.73739 3.18146 2.89801 2.37887 2.77519 2.98518 4.58477 3.61503

0.04062 5.15703 3.38006 0.61958 0.77255 0.48458 0.95700

320 2.53738 5.45699 2.66302 2.10068 4.79820 3.37007 3.91803 4.28147 2.57326 3.17250 4.49870 3.19934 2.56269 2.42971 2.64991 1.92602 3.18665 3.18911 5.89198 4.48334 548 - -

2.68618 4.42225 2.77519 2.73123 3.46354 2.40513 3.72494 3.29354 2.67741 2.69355 4.24690 2.90347 2.73739 3.18146 2.89801 2.37887 2.77519 2.98518 4.58477 3.61503

0.00838 5.18181 5.90415 0.61958 0.77255 0.63306 0.75708

321 2.96146 5.46196 3.21379 2.13809 4.80334 3.38521 2.72579 4.28615 1.89503 3.14374 4.50374 3.20533 2.79697 2.56202 2.95575 1.94623 3.19248 3.45756 5.89652 4.48861 549 - -
2.68618 4.42225 2.77519 2.73123 3.46354 2.40513 3.72494 3.29354 2.67741 2.69355 4.24690 2.90347 2.73739 3.18146 2.89801 2.37887 2.77519 2.98518 4.58477 3.61503
0.00830 5.19104 5.91339 0.61958 0.77255 0.55547 0.85286
322 2.81139 5.46019 3.09976 2.40304 4.79721 3.29066 3.93399 3.92444 1.40472 3.53900 3.89391 2.70174 4.13978 3.03308 2.02115 2.68601 3.20098 3.38209 5.89748 4.49207 550 - -
2.68618 4.42225 2.77519 2.73123 3.46354 2.40513 3.72494 3.29354 2.67741 2.69355 4.24690 2.90347 2.73739 3.18146 2.89801 2.37887 2.77519 2.98518 4.58477 3.61503
0.00815 5.21006 5.93241 0.61958 0.77255 0.59987 0.79603
323 2.71759 4.76462 3.79733 2.29939 3.17071 3.95384 4.19980 3.27461 3.19280 1.82920 3.86508 2.58602 4.33470 3.50351 2.56706 3.06054 3.22614 1.94432 5.33958 3.31288 551 - -
2.68618 4.42225 2.77519 2.73123 3.46354 2.40513 3.72494 3.29354 2.67741 2.69355 4.24690 2.90347 2.73739 3.18146 2.89801 2.37887 2.77519 2.98518 4.58477 3.61503
0.00815 5.21006 5.93241 0.61958 0.77255 0.48005 0.96431
324 1.27609 3.26064 3.52538 2.19616 3.75362 3.86896 3.79709 3.60846 2.95107 3.26136 4.10757 3.05221 4.25841 3.28929 3.38836 2.60693 2.82574 2.85541 5.55870 4.26344 552 - -
2.68618 4.42225 2.77519 2.73123 3.46354 2.40513 3.72494 3.29354 2.67741 2.69355 4.24690 2.90347 2.73739 3.18146 2.89801 2.37887 2.77519 2.98518 4.58477 3.61503
0.00797 5.23232 5.95467 0.61958 0.77255 0.46056 0.99678
325 3.72770 6.30218 1.30151 2.71766 5.42825 3.99948 4.53321 5.08519 3.58219 4.57737 5.43361 2.39843 4.62180 3.71037 4.19525 3.31591 4.01427 4.66556 6.67440 1.05386 553 - -
2.68618 4.42225 2.77519 2.73123 3.46354 2.40513 3.72494 3.29354 2.67741 2.69355 4.24690 2.90347 2.73739 3.18146 2.89801 2.37887 2.77519 2.98518 4.58477 3.61503
0.00787 5.24422 5.96656 0.61958 0.77255 0.48576 0.95510
326 1.75092 4.90686 3.84495 3.31447 4.22683 3.90713 4.34752 3.59049 3.28567 3.28960 4.15880 2.58128 1.89252 3.60853 3.42169 3.19482 3.04023 1.58703 5.62321 4.36949 554 - -
2.68618 4.42225 2.77519 2.73123 3.46354 2.40513 3.72494 3.29354 2.67741 2.69355 4.24690 2.90347 2.73739 3.18146 2.89801 2.37887 2.77519 2.98518 4.58477 3.61503
0.00787 5.24422 5.96656 0.61958 0.77255 0.48576 0.95510
327 2.78592 5.48910 2.89236 2.71143 4.82058 3.78127 3.98827 4.29499 2.61085 3.79168 4.54254 2.37529 4.18639 2.89846 3.08612 2.06492 1.29945 3.87437 5.93785 4.53585 555 - -
2.68618 4.42225 2.77519 2.73123 3.46354 2.40513 3.72494 3.29354 2.67741 2.69355 4.24690 2.90347 2.73739 3.18146 2.89801 2.37887 2.77519 2.98518 4.58477 3.61503
0.00787 5.24422 5.96656 0.61958 0.77255 0.48576 0.95510
328 2.84127 4.48579 5.07381 4.45948 2.25382 4.28991 4.62115 2.21809 4.24758 1.19745 2.47944 4.45076 4.64328 3.93628 4.25478 3.60277 3.34673 2.24750 5.09859 3.08105 556 - -
2.68618 4.42225 2.77519 2.73123 3.46354 2.40513 3.72494 3.29354 2.67741 2.69355 4.24690 2.90347 2.73739 3.18146 2.89801 2.37887 2.77519 2.98518 4.58477 3.61503
0.00787 5.24422 5.96656 0.61958 0.77255 0.48576 0.95510
329 3.05083 4.43618 4.93855 4.32925 3.20138 4.21177 3.28616 2.65722 4.13036 1.23093 2.50694 3.79057 4.57488 4.27386 4.16424 3.52014 3.28284 2.20791 5.04398 2.10406 557 - -
2.68618 4.42225 2.77519 2.73123 3.46354 2.40513 3.72494 3.29354 2.67741 2.69355 4.24690 2.90347 2.73739 3.18146 2.89801 2.37887 2.77519 2.98518 4.58477 3.61503
0.00787 5.24422 5.96656 0.61958 0.77255 0.48576 0.95510
330 2.36909 5.58798 4.52176 4.19197 3.17550 4.65659 0.77603 4.30206 4.06670 3.77652 4.85364 4.40925 5.11886 4.39316 4.31028 4.04722 4.22501 4.08857 4.81253 1.59504 558 - -
2.68618 4.42225 2.77519 2.73123 3.46354 2.40513 3.72494 3.29354 2.67741 2.69355 4.24690 2.90347 2.73739 3.18146 2.89801 2.37887 2.77519 2.98518 4.58477 3.61503
0.00787 5.24422 5.96656 0.61958 0.77255 0.48576 0.95510
331 3.03758 4.44467 4.80743 4.20503 2.36972 2.09350 4.50553 2.36786 4.03072 2.63405 2.51947 4.28044 4.54990 4.19763 4.10696 2.10168 3.26986 2.34363 5.05986 2.23309 559 - -
2.68618 4.42225 2.77519 2.73123 3.46354 2.40513 3.72494 3.29354 2.67741 2.69355 4.24690 2.90347 2.73739 3.18146 2.89801 2.37887 2.77519 2.98518 4.58477 3.61503
0.00787 5.24422 5.96656 0.61958 0.77255 0.48576 0.95510
332 1.62692 3.63384 2.48246 2.88280 4.40149 2.67859 4.06154 3.55089 2.87488 3.42848 4.24856 2.68405 4.23232 3.22153 3.33269 2.39041 2.98108 2.40070 5.68496 4.36016 560 - -
2.68618 4.42225 2.77519 2.73123 3.46354 2.40513 3.72494 3.29354 2.67741 2.69355 4.24690 2.90347 2.73739 3.18146 2.89801 2.37887 2.77519 2.98518 4.58477 3.61503

0.45662 5.24422 1.01805 0.61958 0.77255 0.48576 0.95510

333 3.20206 4.53755 5.09954 4.51328 3.54131 4.45145 4.81823 1.55610 4.34519 1.56095 2.28533 4.58137 4.76304 4.47816 4.39652 3.77898 2.18850 1.90795 5.26747 4.11587 561 - -

2.68618 4.42225 2.77519 2.73123 3.46354 2.40513 3.72494 3.29354 2.67741 2.69355 4.24690 2.90347 2.73739 3.18146 2.89801 2.37887 2.77519 2.98518 4.58477 3.61503

0.01205 4.82038 5.54273 0.61958 0.77255 1.31491 0.31265

334 2.50865 4.34015 4.17788 3.59906 1.79614 3.89838 4.21363 2.50772 3.49656 2.37062 3.46091 3.84714 2.55223 3.73559 3.71449 2.98615 2.10265 2.59148 4.95787 3.75945 562 - -

2.68618 4.42225 2.77519 2.73123 3.46354 2.40513 3.72494 3.29354 2.67741 2.69355 4.24690 2.90347 2.73739 3.18146 2.89801 2.37887 2.77519 2.98518 4.58477 3.61503

0.01205 4.82038 5.54273 0.61958 0.77255 1.31491 0.31265

335 2.86212 4.83457 3.50258 2.94486 4.11321 3.71425 4.01145 3.49010 2.80845 2.10518 4.00900 3.39741 4.14419 3.22049 2.22109 1.48484 2.54945 3.20615 5.44912 4.17674 563 - -

2.68618 4.42225 2.77519 2.73123 3.46354 2.40513 3.72494 3.29354 2.67741 2.69355 4.24690 2.90347 2.73739 3.18146 2.89801 2.37887 2.77519 2.98518 4.58477 3.61503

0.01205 4.82038 5.54273 0.61958 0.77255 1.31491 0.31265

336 4.33245 6.31764 0.18486 3.42311 5.90131 4.24270 5.15124 5.77682 4.44865 5.21399 6.29234 3.99964 4.94960 4.44579 5.01856 4.24497 4.70959 5.33521 6.82067 5.73040 564 - -

2.68618 4.42225 2.77519 2.73123 3.46354 2.40513 3.72494 3.29354 2.67741 2.69355 4.24690 2.90347 2.73739 3.18146 2.89801 2.37887 2.77519 2.98518 4.58477 3.61503

0.01205 4.82038 5.54273 0.61958 0.77255 0.18383 1.78425

337 2.79154 5.47806 3.24882 1.84802 4.36616 2.97756 3.23665 4.00154 2.55811 3.77914 4.52132 1.72931 3.85154 2.67457 3.18626 2.78006 2.96161 3.68414 5.91667 2.89570 565 - -

2.68618 4.42225 2.77519 2.73123 3.46354 2.40513 3.72494 3.29354 2.67741 2.69355 4.24690 2.90347 2.73739 3.18146 2.89801 2.37887 2.77519 2.98518 4.58477 3.61503

0.11522 5.25740 2.26700 0.61958 0.77255 0.48576 0.95510

338 2.44093 4.89843 2.49534 2.11426 3.34282 3.86045 4.08844 2.44421 2.98496 3.12785 3.98944 3.29308 4.24669 3.31510 3.40559 2.71807 3.19295 1.70528 5.44991 4.17251 566 - -

2.68618 4.42225 2.77519 2.73123 3.46354 2.40513 3.72494 3.29354 2.67741 2.69355 4.24690 2.90347 2.73739 3.18146 2.89801 2.37887 2.77519 2.98518 4.58477 3.61503

0.39974 5.15082 1.12790 0.61958 0.77255 0.33005 1.26899

339 5.08006 5.94176 5.82089 5.81544 1.12837 5.47788 4.06283 4.60713 5.58558 3.70561 5.11343 4.99010 5.72655 5.18137 5.34575 4.90378 5.26882 4.58139 4.13053 0.62250 567 - -

2.68618 4.42225 2.77519 2.73123 3.46354 2.40513 3.72494 3.29354 2.67741 2.69355 4.24690 2.90347 2.73739 3.18146 2.89801 2.37887 2.77519 2.98518 4.58477 3.61503

0.01093 4.91708 5.63942 0.61958 0.77255 1.20466 0.35638

340 3.04351 4.41291 4.92211 4.32467 3.29379 4.20927 4.46045 2.59091 4.12582 1.41812 3.41970 4.33316 4.55505 4.24915 4.15198 3.52292 3.27407 1.42103 3.44890 2.34436 568 - -

2.68618 4.42225 2.77519 2.73123 3.46354 2.40513 3.72494 3.29354 2.67741 2.69355 4.24690 2.90347 2.73739 3.18146 2.89801 2.37887 2.77519 2.98518 4.58477 3.61503

0.01093 4.91708 5.63942 0.61958 0.77255 0.20763 1.67400

341 3.06017 2.27094 4.84479 4.24060 3.55505 4.21098 4.53467 2.30311 2.99431 1.39367 3.07173 4.31142 4.57482 4.22673 2.55906 3.51490 3.29224 2.11996 5.08523 3.90566 569 - -

2.68618 4.42225 2.77519 2.73123 3.46354 2.40513 3.72494 3.29354 2.67741 2.69355 4.24690 2.90347 2.73739 3.18146 2.89801 2.37887 2.77519 2.98518 4.58477 3.61503

0.00777 5.25740 5.97975 0.61958 0.77255 0.48576 0.95510

342 0.93706 5.03146 4.97461 4.86116 5.62699 0.83458 5.70598 5.05535 4.90744 4.00908 5.59405 4.56857 4.67571 5.08737 5.03864 3.32965 3.68733 4.30364 6.98378 5.88432 570 - -

2.68618 4.42225 2.77519 2.73123 3.46354 2.40513 3.72494 3.29354 2.67741 2.69355 4.24690 2.90347 2.73739 3.18146 2.89801 2.37887 2.77519 2.98518 4.58477 3.61503

0.00777 5.25740 5.97975 0.61958 0.77255 0.48576 0.95510

343 1.29754 4.86102 4.77645 4.33865 4.65511 3.87332 5.09348 3.92955 4.26274 3.73814 4.62045 4.33761 4.58364 4.49786 4.49148 1.85337 1.67505 1.63642 6.10705 4.93093 571 - -

2.68618 4.42225 2.77519 2.73123 3.46354 2.40513 3.72494 3.29354 2.67741 2.69355 4.24690 2.90347 2.73739 3.18146 2.89801 2.37887 2.77519 2.98518 4.58477 3.61503

0.00777 5.25740 5.97975 0.61958 0.77255 0.48576 0.95510

344 3.09138 4.62247 4.37639 3.79588 3.74871 4.16324 4.46385 1.76601 3.67112 2.80119 3.27414 4.07366 4.53811 3.94677 2.04204 1.63227 3.32672 2.01529 5.25594 4.05458 572 - -

2.68618 4.42225 2.77519 2.73123 3.46354 2.40513 3.72494 3.29354 2.67741 2.69355 4.24690 2.90347 2.73739 3.18146 2.89801 2.37887 2.77519 2.98518 4.58477 3.61503
0.00777 5.25740 5.97975 0.61958 0.77255 0.48576 0.95510
345 1.47680 4.84676 5.01058 4.63655 4.69506 2.35629 5.25751 3.94176 4.54373 3.78238 4.67212 4.47065 4.62034 4.73377 4.69198 3.08679 2.81920 0.86082 6.16634 5.01081 573 - -
2.68618 4.42225 2.77519 2.73123 3.46354 2.40513 3.72494 3.29354 2.67741 2.69355 4.24690 2.90347 2.73739 3.18146 2.89801 2.37887 2.77519 2.98518 4.58477 3.61503
0.00777 5.25740 5.97975 0.61958 0.77255 0.48576 0.95510
346 1.93821 4.72056 5.16757 4.62486 3.90963 3.94279 4.98730 2.75333 4.45994 1.17923 3.86231 4.65480 4.86173 4.63599 4.54011 3.78460 3.57639 1.28568 5.53361 4.35707 574 - -
2.68618 4.42225 2.77519 2.73123 3.46354 2.40513 3.72494 3.29354 2.67741 2.69355 4.24690 2.90347 2.73739 3.18146 2.89801 2.37887 2.77519 2.98518 4.58477 3.61503
0.00777 5.25740 5.97975 0.61958 0.77255 0.48576 0.95510
347 1.50805 4.75907 5.15599 4.62836 4.06229 2.51837 5.08021 1.74897 4.49347 2.97918 4.00570 4.67930 4.90276 4.69037 4.61075 3.80762 3.61188 1.21521 5.67795 4.48829 575 - -
2.68618 4.42225 2.77519 2.73123 3.46354 2.40513 3.72494 3.29354 2.67741 2.69355 4.24690 2.90347 2.73739 3.18146 2.89801 2.37887 2.77519 2.98518 4.58477 3.61503
0.00777 5.25740 5.97975 0.61958 0.77255 0.48576 0.95510
348 3.01762 5.50949 3.27490 2.71498 4.32253 3.78945 3.22561 4.33314 1.76265 3.21525 4.55191 1.91266 4.18122 2.06960 2.33593 2.60906 3.24673 3.90073 5.93741 4.53760 576 - -
2.68618 4.42225 2.77519 2.73123 3.46354 2.40513 3.72494 3.29354 2.67741 2.69355 4.24690 2.90347 2.73739 3.18146 2.89801 2.37887 2.77519 2.98518 4.58477 3.61503
0.00777 5.25740 5.97975 0.61958 0.77255 0.48576 0.95510
349 3.20054 5.12725 4.82853 4.82217 5.89151 2.24710 5.81591 5.46941 5.07258 5.12990 5.88721 4.59916 4.73653 5.19682 5.20240 0.30048 3.78429 4.54387 7.17756 6.11552 577 - -
2.68618 4.42225 2.77519 2.73123 3.46354 2.40513 3.72494 3.29354 2.67741 2.69355 4.24690 2.90347 2.73739 3.18146 2.89801 2.37887 2.77519 2.98518 4.58477 3.61503
0.00777 5.25740 5.97975 0.61958 0.77255 0.48576 0.95510
350 2.76674 4.70579 5.30162 4.71646 3.83099 4.61791 4.99928 1.40471 4.54151 1.47523 3.52044 4.75982 4.95182 4.69356 4.59097 3.94504 1.82941 2.04814 5.47945 4.30443 578 - -
2.68618 4.42225 2.77519 2.73123 3.46354 2.40513 3.72494 3.29354 2.67741 2.69355 4.24690 2.90347 2.73739 3.18146 2.89801 2.37887 2.77519 2.98518 4.58477 3.61503
0.00777 5.25740 5.97975 0.61958 0.77255 0.48576 0.95510
351 2.91819 4.64505 4.41097 3.81339 3.76434 4.18858 4.45790 2.36499 2.91712 1.93918 3.74985 4.08658 4.55489 3.93067 1.47043 3.47135 3.34881 1.76275 5.26517 4.06814 579 - -
2.68618 4.42225 2.77519 2.73123 3.46354 2.40513 3.72494 3.29354 2.67741 2.69355 4.24690 2.90347 2.73739 3.18146 2.89801 2.37887 2.77519 2.98518 4.58477 3.61503
0.00777 5.25740 5.97975 0.61958 0.77255 0.48576 0.95510
352 2.99290 5.45519 2.44229 2.70482 4.78079 3.77404 3.96290 4.25291 2.04391 3.15697 2.91114 3.24948 4.16702 1.76137 2.97236 2.07976 3.01403 3.33832 5.89953 4.50112 580 - -
2.68618 4.42225 2.77519 2.73123 3.46354 2.40513 3.72494 3.29354 2.67741 2.69355 4.24690 2.90347 2.73739 3.18146 2.89801 2.37887 2.77519 2.98518 4.58477 3.61503
0.00777 5.25740 5.97975 0.61958 0.77255 0.48576 0.95510
353 2.90958 5.06333 3.51615 2.95971 4.27439 3.87312 2.44738 3.66958 2.94549 2.48506 4.15593 2.02724 4.26608 3.28710 3.38699 1.95930 2.15760 2.67889 5.60328 4.30148 581 - -
2.68618 4.42225 2.77519 2.73123 3.46354 2.40513 3.72494 3.29354 2.67741 2.69355 4.24690 2.90347 2.73739 3.18146 2.89801 2.37887 2.77519 2.98518 4.58477 3.61503
0.00777 5.25740 5.97975 0.61958 0.77255 0.48576 0.95510
354 1.77448 5.45425 3.27575 2.73637 4.80354 1.99669 2.74020 4.27377 2.21068 3.77726 4.53018 2.87452 4.19041 3.10377 3.22749 2.10008 3.10906 3.85507 5.92760 4.53292 582 - -
2.68618 4.42225 2.77519 2.73123 3.46354 2.40513 3.72494 3.29354 2.67741 2.69355 4.24690 2.90347 2.73739 3.18146 2.89801 2.37887 2.77519 2.98518 4.58477 3.61503
0.00777 5.25740 5.97975 0.61958 0.77255 0.48576 0.95510
355 2.88760 5.40978 3.01964 2.60147 4.71745 3.78319 3.97431 4.17754 2.03362 3.38436 4.12237 3.26693 4.17569 2.91653 2.91240 1.39150 2.75745 2.50399 5.86540 4.47869 583 - -
2.68618 4.42225 2.77519 2.73123 3.46354 2.40513 3.72494 3.29354 2.67741 2.69355 4.24690 2.90347 2.73739 3.18146 2.89801 2.37887 2.77519 2.98518 4.58477 3.61503
0.00777 5.25740 5.97975 0.61958 0.77255 0.48576 0.95510

356 2.39586 2.87865 2.37112 1.99473 4.83128 3.76707 2.93178 4.31375 2.21891 3.79433 4.53332 3.00787 4.16089 3.05330 2.98562 2.95688 2.00545 3.87919 5.92685 4.51888 584 - -
2.68618 4.42225 2.77519 2.73123 3.46354 2.40513 3.72494 3.29354 2.67741 2.69355 4.24690 2.90347 2.73739 3.18146 2.89801 2.37887 2.77519 2.98518 4.58477 3.61503
0.00777 5.25740 5.97975 0.61958 0.77255 0.48576 0.95510
357 2.36142 5.41982 3.39058 2.82585 4.73067 3.85446 4.02833 4.17199 1.94331 3.70417 4.48467 3.35248 1.58454 3.14717 2.29046 2.78467 3.29785 3.79574 5.87475 2.85156 585 - -
2.68618 4.42225 2.77519 2.73123 3.46354 2.40513 3.72494 3.29354 2.67741 2.69355 4.24690 2.90347 2.73739 3.18146 2.89801 2.37887 2.77519 2.98518 4.58477 3.61503
0.00777 5.25740 5.97975 0.61958 0.77255 0.48576 0.95510
358 2.99795 5.50333 1.51134 1.90762 4.84559 3.76837 3.56750 4.32999 2.37824 3.45666 4.54502 3.23653 2.82742 2.67533 3.18805 2.65661 3.22938 3.89252 3.90190 4.52732 586 - -
2.68618 4.42225 2.77519 2.73123 3.46354 2.40513 3.72494 3.29354 2.67741 2.69355 4.24690 2.90347 2.73739 3.18146 2.89801 2.37887 2.77519 2.98518 4.58477 3.61503
0.00777 5.25740 5.97975 0.61958 0.77255 0.48576 0.95510
359 3.02552 5.06489 3.54188 2.97780 4.26533 3.89308 4.10066 3.00797 1.93050 1.80508 2.38105 2.44056 4.27889 3.01335 2.75068 2.83303 3.25542 3.37540 5.59285 4.29721 587 - -
2.68618 4.42225 2.77519 2.73123 3.46354 2.40513 3.72494 3.29354 2.67741 2.69355 4.24690 2.90347 2.73739 3.18146 2.89801 2.37887 2.77519 2.98518 4.58477 3.61503
0.00777 5.25740 5.97975 0.61958 0.77255 0.48576 0.95510
360 3.29167 4.64320 5.21190 4.61890 2.87884 4.50656 2.51466 1.59476 4.43033 2.38147 3.70217 4.64481 4.84783 4.57262 4.46771 3.82713 3.52616 1.06150 5.31181 4.10655 588 - -
2.68618 4.42225 2.77519 2.73123 3.46354 2.40513 3.72494 3.29354 2.67741 2.69355 4.24690 2.90347 2.73739 3.18146 2.89801 2.37887 2.77519 2.98518 4.58477 3.61503
0.00777 5.25740 5.97975 0.61958 0.77255 0.48576 0.95510
361 2.29683 4.63331 5.24865 4.65740 2.79837 4.52475 4.89875 2.11294 4.47055 2.12228 3.74709 4.67740 4.86917 4.61979 4.50657 3.55413 3.52680 0.90471 5.38368 4.20277 589 - -
2.68618 4.42225 2.77519 2.73123 3.46354 2.40513 3.72494 3.29354 2.67741 2.69355 4.24690 2.90347 2.73739 3.18146 2.89801 2.37887 2.77519 2.98518 4.58477 3.61503
0.00777 5.25740 5.97975 0.61958 0.77255 0.48576 0.95510
362 2.43182 5.04938 5.66536 5.07326 1.22885 4.97916 5.28205 2.10870 4.89388 1.18169 3.57439 5.13064 5.22882 4.92987 4.88914 4.31742 3.96100 2.92055 5.58483 4.45268 590 - -
2.68618 4.42225 2.77519 2.73123 3.46354 2.40513 3.72494 3.29354 2.67741 2.69355 4.24690 2.90347 2.73739 3.18146 2.89801 2.37887 2.77519 2.98518 4.58477 3.61503
0.00777 5.25740 5.97975 0.61958 0.77255 0.48576 0.95510
363 2.31694 4.59798 4.44937 3.87288 3.72157 4.16460 1.24504 2.86201 3.75676 2.77882 2.96754 4.11922 4.54719 4.00741 3.97330 3.45343 3.16722 1.73394 5.23611 4.03751 591 - -
2.68618 4.42225 2.77519 2.73123 3.46354 2.40513 3.72494 3.29354 2.67741 2.69355 4.24690 2.90347 2.73739 3.18146 2.89801 2.37887 2.77519 2.98518 4.58477 3.61503
0.00777 5.25740 5.97975 0.61958 0.77255 0.48576 0.95510
364 3.06899 4.52493 2.28814 4.00959 2.50176 4.18699 4.49731 2.11360 3.88152 1.86172 3.63257 4.19303 4.55377 4.09087 4.04187 1.96849 3.30156 2.03852 5.14751 3.95762 592 - -
2.68618 4.42225 2.77519 2.73123 3.46354 2.40513 3.72494 3.29354 2.67741 2.69355 4.24690 2.90347 2.73739 3.18146 2.89801 2.37887 2.77519 2.98518 4.58477 3.61503
0.00777 5.25740 5.97975 0.61958 0.77255 0.48576 0.95510
365 3.02461 4.79524 2.05800 2.87603 3.94036 3.98154 3.52175 2.42906 3.22306 2.04545 3.89565 3.68430 2.80915 3.53275 3.59666 3.22220 3.25651 1.75639 5.37160 4.12748 593 - -
2.68618 4.42225 2.77519 2.73123 3.46354 2.40513 3.72494 3.29354 2.67741 2.69355 4.24690 2.90347 2.73739 3.18146 2.89801 2.37887 2.77519 2.98518 4.58477 3.61503
0.00777 5.25740 5.97975 0.61958 0.77255 0.48576 0.95510
366 2.49104 5.41966 2.00409 2.80742 4.83842 2.56465 4.07791 4.30561 2.84408 3.82402 4.58383 2.84444 4.23115 3.19172 3.33088 2.45482 1.45651 3.47471 5.98475 4.59728 594 - -
2.68618 4.42225 2.77519 2.73123 3.46354 2.40513 3.72494 3.29354 2.67741 2.69355 4.24690 2.90347 2.73739 3.18146 2.89801 2.37887 2.77519 2.98518 4.58477 3.61503
0.00777 5.25740 5.97975 0.61958 0.77255 0.48576 0.95510
367 2.82967 4.80969 1.86882 3.27981 3.96724 3.99763 4.25802 2.07925 3.25128 2.90532 3.91886 3.07385 4.38445 3.55999 3.62568 2.67308 3.12965 1.58767 5.40286 4.15804 595 - -
2.68618 4.42225 2.77519 2.73123 3.46354 2.40513 3.72494 3.29354 2.67741 2.69355 4.24690 2.90347 2.73739 3.18146 2.89801 2.37887 2.77519 2.98518 4.58477 3.61503

0.00777 5.25740 5.97975 0.61958 0.77255 0.48576 0.95510

368 2.89066 5.49849 3.25030 2.25808 4.84076 3.55133 3.71036 4.32245 2.00246 3.80152 4.54112 3.24290 2.21189 3.05792 2.52394 1.70430 2.76153 3.88789 5.93229 4.52614 596 - -

2.68618 4.42225 2.77519 2.73123 3.46354 2.40513 3.72494 3.29354 2.67741 2.69355 4.24690 2.90347 2.73739 3.18146 2.89801 2.37887 2.77519 2.98518 4.58477 3.61503

0.00777 5.25740 5.97975 0.61958 0.77255 0.48576 0.95510

369 2.88263 5.41076 2.44903 2.09599 3.95844 3.14477 3.71141 2.75010 2.27369 2.26966 3.53677 2.78321 4.17414 2.71663 3.08437 2.86680 2.99361 3.38011 5.86615 4.47857 597 - -

2.68618 4.42225 2.77519 2.73123 3.46354 2.40513 3.72494 3.29354 2.67741 2.69355 4.24690 2.90347 2.73739 3.18146 2.89801 2.37887 2.77519 2.98518 4.58477 3.61503

0.03617 5.25740 3.49604 0.61958 0.77255 0.48576 0.95510

370 2.86990 5.45092 2.81000 2.28957 4.26382 3.75778 3.94664 3.72295 2.50211 3.74949 4.49574 1.83900 4.15097 3.04823 2.66519 2.57801 2.40178 3.83311 5.89307 2.32926 598 - -

2.68618 4.42225 2.77519 2.73123 3.46354 2.40513 3.72494 3.29354 2.67741 2.69355 4.24690 2.90347 2.73739 3.18146 2.89801 2.37887 2.77519 2.98518 4.58477 3.61503

0.07192 5.22922 2.74825 0.61958 0.77255 0.58231 0.81782

371 3.05214 4.67880 4.07912 3.51192 2.75756 4.06183 2.18036 3.16203 2.91653 2.55108 3.78827 3.85823 4.01964 3.72370 3.75209 2.02691 3.28346 2.95081 2.79731 1.98965 599 - -

2.68618 4.42225 2.77519 2.73123 3.46354 2.40513 3.72494 3.29354 2.67741 2.69355 4.24690 2.90347 2.73739 3.18146 2.89801 2.37887 2.77519 2.98518 4.58477 3.61503

0.00852 5.16581 5.88816 0.61958 0.77255 0.34484 1.23215

372 2.31095 5.36207 3.05440 2.60514 3.41953 2.79881 3.69199 3.24565 1.95978 2.68750 4.41761 3.28486 3.40800 2.98349 2.26490 2.80420 2.99703 3.06731 5.82910 4.45395 600 - -

2.68356 4.42384 2.77721 2.73264 3.46111 2.40687 3.72472 3.29031 2.67819 2.69398 4.23414 2.90351 2.73739 3.18109 2.89929 2.37960 2.77677 2.98342 4.57361 3.61754

0.49710 0.96858 4.41585 2.60276 0.07696 0.48576 0.95510

373 2.98683 5.48763 2.99220 2.29137 4.82883 3.76196 3.18192 3.29688 1.76654 2.84103 4.52937 3.12011 4.15529 2.19631 2.42608 2.29937 3.21792 3.47778 5.92248 4.51425 621 - -

2.68618 4.42225 2.77519 2.73123 3.46354 2.40513 3.72494 3.29354 2.67741 2.69355 4.24690 2.90347 2.73739 3.18146 2.89801 2.37887 2.77519 2.98518 4.58477 3.61503

0.11409 5.24780 2.27728 0.61958 0.77255 0.52002 0.90265

374 2.15579 5.13410 3.35393 2.79611 4.36927 2.24468 3.98480 2.84142 2.65194 2.44772 4.20632 2.26814 4.16957 2.89986 3.04040 2.53998 2.72051 3.46435 5.63959 4.30738 622 - -

2.68618 4.42225 2.77519 2.73123 3.46354 2.40513 3.72494 3.29354 2.67741 2.69355 4.24690 2.90347 2.73739 3.18146 2.89801 2.37887 2.77519 2.98518 4.58477 3.61503

0.00872 5.14243 5.86478 0.61958 0.77255 0.81948 0.58100

375 4.14286 5.35155 6.15620 5.59045 2.96750 5.57277 5.92720 1.96033 5.46212 0.63470 3.53424 5.74540 5.64432 5.36792 5.41853 4.96568 4.35776 1.79433 5.97175 4.97698 623 - -

2.68623 4.42230 2.77524 2.73052 3.46359 2.40517 3.72499 3.29359 2.67746 2.69360 4.24694 2.90351 2.73744 3.18151 2.89805 2.37892 2.77524 2.98523 4.58482 3.61508

0.11917 2.21177 5.86478 0.18674 1.76995 0.81948 0.58100

376 2.63289 5.44144 2.65321 1.55192 4.78327 3.71269 2.92898 4.26695 2.09785 3.35940 4.48302 2.93930 4.10641 2.99791 2.87639 2.52961 3.16963 2.79847 5.87597 4.46697 625 - -

2.68618 4.42225 2.77519 2.73123 3.46354 2.40513 3.72494 3.29354 2.67741 2.69355 4.24690 2.90347 2.73739 3.18146 2.89801 2.37887 2.77519 2.98518 4.58477 3.61503

0.00872 5.14243 5.86478 0.61958 0.77255 0.81948 0.58100

377 1.66278 5.47005 2.09835 1.81363 4.81019 3.72203 3.51717 4.29485 2.46870 3.39105 4.51318 3.18976 4.12405 2.61534 3.15912 2.70923 3.19667 3.85827 5.90444 4.49282 626 - -

2.68618 4.42225 2.77519 2.73123 3.46354 2.40513 3.72494 3.29354 2.67741 2.69355 4.24690 2.90347 2.73739 3.18146 2.89801 2.37887 2.77519 2.98518 4.58477 3.61503

0.00872 5.14243 5.86478 0.61958 0.77255 0.32240 1.28882

378 2.00724 4.75524 2.09795 3.32009 2.14317 4.00038 4.25081 3.25329 3.27658 2.08423 3.20286 3.73002 4.37997 2.58175 3.63426 3.24403 3.25706 3.02860 5.33036 3.28116 627 - -

2.68618 4.42225 2.77519 2.73123 3.46354 2.40513 3.72494 3.29354 2.67741 2.69355 4.24690 2.90347 2.73739 3.18146 2.89801 2.37887 2.77519 2.98518 4.58477 3.61503

0.00777 5.25740 5.97975 0.61958 0.77255 0.48576 0.95510

379 2.88979 5.50183 2.53618 2.35051 4.30437 1.43346 3.95913 4.32731 2.33301 3.80559 4.54381 3.01786 4.16419 2.93091 2.94971 2.50731 2.71569 3.89064 5.93640 4.52673 628 - -

2.68618 4.42225 2.77519 2.73123 3.46354 2.40513 3.72494 3.29354 2.67741 2.69355 4.24690 2.90347 2.73739 3.18146 2.89801 2.37887 2.77519 2.98518 4.58477 3.61503
0.00777 5.25740 5.97975 0.61958 0.77255 0.48576 0.95510
380 2.24825 2.79692 4.54123 3.95217 3.05163 4.14604 4.45187 2.94981 3.56342 2.48517 3.61857 3.76787 4.51514 3.53153 3.70371 3.04517 3.11289 2.47817 1.36912 3.92547 629 - -
2.68618 4.42225 2.77519 2.73123 3.46354 2.40513 3.72494 3.29354 2.67741 2.69355 4.24690 2.90347 2.73739 3.18146 2.89801 2.37887 2.77519 2.98518 4.58477 3.61503
0.00777 5.25740 5.97975 0.61958 0.77255 0.48576 0.95510
381 3.00162 5.16852 3.05917 2.86525 4.39734 3.84068 4.04675 2.87995 1.61498 1.74288 4.24200 3.03322 3.68970 2.78992 3.31538 2.93093 2.97145 3.06326 5.67773 3.94298 630 - -
2.68618 4.42225 2.77519 2.73123 3.46354 2.40513 3.72494 3.29354 2.67741 2.69355 4.24690 2.90347 2.73739 3.18146 2.89801 2.37887 2.77519 2.98518 4.58477 3.61503
0.00777 5.25740 5.97975 0.61958 0.77255 0.48576 0.95510
382 3.02681 4.67846 3.40131 3.45475 3.80538 4.03615 4.30185 1.99728 3.39755 2.22226 3.78562 2.24770 3.44802 3.31445 3.71873 3.05041 2.95309 1.59231 5.27016 4.04838 631 - -
2.68618 4.42225 2.77519 2.73123 3.46354 2.40513 3.72494 3.29354 2.67741 2.69355 4.24690 2.90347 2.73739 3.18146 2.89801 2.37887 2.77519 2.98518 4.58477 3.61503
0.00777 5.25740 5.97975 0.61958 0.77255 0.48576 0.95510
383 2.67059 5.46265 3.27584 2.07638 4.78977 3.78531 3.97001 4.25945 2.37716 3.41028 4.50915 3.26128 4.17769 2.76830 1.70536 1.97627 3.23597 2.62113 5.90421 4.50996 632 - -
2.68618 4.42225 2.77519 2.73123 3.46354 2.40513 3.72494 3.29354 2.67741 2.69355 4.24690 2.90347 2.73739 3.18146 2.89801 2.37887 2.77519 2.98518 4.58477 3.61503
0.00777 5.25740 5.97975 0.61958 0.77255 0.48576 0.95510
384 2.87317 5.48167 2.71799 2.13493 4.81848 3.43648 3.95559 4.29864 2.15357 3.31299 3.60793 3.23836 4.16096 3.05462 2.17683 1.75502 2.81286 3.47432 5.91931 3.88658 633 - -
2.68618 4.42225 2.77519 2.73123 3.46354 2.40513 3.72494 3.29354 2.67741 2.69355 4.24690 2.90347 2.73739 3.18146 2.89801 2.37887 2.77519 2.98518 4.58477 3.61503
0.00777 5.25740 5.97975 0.61958 0.77255 0.48576 0.95510
385 3.04090 4.45508 4.77426 4.17353 3.04430 4.18299 3.80799 1.50292 3.54867 2.64681 3.11879 3.21588 4.54910 4.18012 4.09536 3.10835 2.61607 1.95706 5.07282 2.14220 634 - -
2.68618 4.42225 2.77519 2.73123 3.46354 2.40513 3.72494 3.29354 2.67741 2.69355 4.24690 2.90347 2.73739 3.18146 2.89801 2.37887 2.77519 2.98518 4.58477 3.61503
0.00777 5.25740 5.97975 0.61958 0.77255 0.48576 0.95510
386 2.68118 3.63238 2.86679 1.86989 2.59682 3.81656 4.01671 3.27524 2.59208 3.52316 4.32282 3.33288 4.20719 2.45498 3.27266 2.05629 3.22900 2.70722 5.74790 4.39893 635 - -
2.68618 4.42225 2.77519 2.73123 3.46354 2.40513 3.72494 3.29354 2.67741 2.69355 4.24690 2.90347 2.73739 3.18146 2.89801 2.37887 2.77519 2.98518 4.58477 3.61503
0.00777 5.25740 5.97975 0.61958 0.77255 0.48576 0.95510
387 3.06634 5.47366 3.34293 2.63399 4.79512 3.83912 4.01139 4.24832 2.55923 2.40771 4.52561 2.48303 1.56763 2.94222 2.07108 2.82822 3.29316 3.85265 5.91083 4.53943 636 - -
2.68618 4.42225 2.77519 2.73123 3.46354 2.40513 3.72494 3.29354 2.67741 2.69355 4.24690 2.90347 2.73739 3.18146 2.89801 2.37887 2.77519 2.98518 4.58477 3.61503
0.00777 5.25740 5.97975 0.61958 0.77255 0.48576 0.95510
388 3.04826 4.44234 4.88762 3.95296 3.53739 4.20464 4.53191 1.90891 3.81050 1.74032 3.56044 4.32496 4.56924 4.24721 3.87752 2.73952 3.28048 1.30777 5.06446 3.04809 637 - -
2.68618 4.42225 2.77519 2.73123 3.46354 2.40513 3.72494 3.29354 2.67741 2.69355 4.24690 2.90347 2.73739 3.18146 2.89801 2.37887 2.77519 2.98518 4.58477 3.61503
0.00777 5.25740 5.97975 0.61958 0.77255 0.48576 0.95510
389 2.30236 5.11575 2.91219 2.55490 4.32953 3.85862 3.72447 3.73250 2.89463 1.55567 4.19385 3.41512 4.24725 3.24117 2.06287 3.06807 3.23796 3.12507 5.63531 2.96632 638 - -
2.68618 4.42225 2.77519 2.73123 3.46354 2.40513 3.72494 3.29354 2.67741 2.69355 4.24690 2.90347 2.73739 3.18146 2.89801 2.37887 2.77519 2.98518 4.58477 3.61503
0.11285 5.25740 2.28763 0.61958 0.77255 0.48576 0.95510
390 2.95091 5.44353 3.20286 2.26256 4.78307 3.72483 3.91136 4.26324 2.19978 3.74534 3.50760 1.62613 2.77828 3.01025 2.59306 2.27825 3.07046 3.83137 5.87862 4.47368 639 - -
2.68618 4.42225 2.77519 2.73123 3.46354 2.40513 3.72494 3.29354 2.67741 2.69355 4.24690 2.90347 2.73739 3.18146 2.89801 2.37887 2.77519 2.98518 4.58477 3.61503
0.05274 5.15317 3.08811 0.61958 0.77255 0.79400 0.60154

391 2.60128 5.31376 3.37764 2.84274 4.63075 3.82398 4.02599 4.05253 2.42891 3.61132 3.69922 3.35576 1.26246 1.98939 3.12695 3.06262 3.28536 3.69944 5.80679 4.47454 640 - -
2.68618 4.42225 2.77519 2.73123 3.46354 2.40513 3.72494 3.29354 2.67741 2.69355 4.24690 2.90347 2.73739 3.18146 2.89801 2.37887 2.77519 2.98518 4.58477 3.61503
0.00901 5.10945 5.83180 0.61958 0.77255 0.89231 0.52714
392 2.93049 5.10042 2.90589 2.41123 3.23864 3.76894 3.97499 3.74179 2.47531 1.90137 3.67071 2.22249 4.15829 2.76120 3.24412 2.97363 3.16184 3.07600 5.60911 2.76589 641 - -
2.68618 4.42225 2.77519 2.73123 3.46354 2.40513 3.72494 3.29354 2.67741 2.69355 4.24690 2.90347 2.73739 3.18146 2.89801 2.37887 2.77519 2.98518 4.58477 3.61503
0.00901 5.10945 5.83180 0.61958 0.77255 0.89231 0.52714
393 2.18977 5.43523 2.94101 1.92517 4.78003 3.69748 3.88535 4.26579 2.33790 3.33159 4.47611 2.29867 4.09163 2.19421 2.68513 2.32223 3.15636 3.82576 5.86785 4.45705 642 - -
2.68618 4.42225 2.77519 2.73123 3.46354 2.40513 3.72494 3.29354 2.67741 2.69355 4.24690 2.90347 2.73739 3.18146 2.89801 2.37887 2.77519 2.98518 4.58477 3.61503
0.05312 5.10945 3.08582 0.61958 0.77255 0.89231 0.52714
394 2.90520 5.39365 2.75541 2.15890 4.72973 3.68186 3.86976 3.65428 2.08922 3.69490 4.43679 3.15289 4.07525 2.27490 2.66871 2.03218 3.01400 2.36910 5.83154 4.42646 643 - -
2.68618 4.42225 2.77519 2.73123 3.46354 2.40513 3.72494 3.29354 2.67741 2.69355 4.24690 2.90347 2.73739 3.18146 2.89801 2.37887 2.77519 2.98518 4.58477 3.61503
0.39848 5.06575 1.13212 0.61958 0.77255 0.81988 0.58069
395 1.92723 5.20658 2.70004 2.50114 4.52086 3.56138 2.96156 3.98450 2.50260 3.49924 4.25773 3.04330 3.95573 2.17973 2.77924 2.60423 2.34209 3.37034 5.66017 4.26990 644 - -
2.68618 4.42225 2.77519 2.73123 3.46354 2.40513 3.72494 3.29354 2.67741 2.69355 4.24690 2.90347 2.73739 3.18146 2.89801 2.37887 2.77519 2.98518 4.58477 3.61503
0.01318 4.73131 5.45366 0.61958 0.77255 0.90184 0.52058
396 2.43242 5.34825 2.58533 2.22823 4.69271 3.09634 3.79868 4.17846 2.23546 3.65323 4.38925 2.35754 4.00462 2.54030 2.61880 2.54765 2.25793 3.73860 5.78113 4.37017 645 - -
2.68618 4.42225 2.77519 2.73123 3.46354 2.40513 3.72494 3.29354 2.67741 2.69355 4.24690 2.90347 2.73739 3.18146 2.89801 2.37887 2.77519 2.98518 4.58477 3.61503
0.01112 4.90004 5.62239 0.61958 0.77255 1.22580 0.34746
397 2.61937 4.37357 4.17515 3.59388 2.04338 3.57251 4.22386 2.82966 3.49507 1.95719 2.68539 2.50638 4.30352 3.73742 3.30790 3.02105 2.86641 2.63031 4.97982 2.80582 646 - -
2.68618 4.42225 2.77519 2.73123 3.46354 2.40513 3.72494 3.29354 2.67741 2.69355 4.24690 2.90347 2.73739 3.18146 2.89801 2.37887 2.77519 2.98518 4.58477 3.61503
0.01112 4.90004 5.62239 0.61958 0.77255 0.20282 1.69514
398 2.47884 5.37045 3.30086 2.74422 2.91527 3.79071 2.61743 3.16825 1.73254 3.65322 4.42519 3.17676 3.45123 2.64509 2.50675 2.54541 3.08948 3.23452 5.83546 4.45843 647 - -
2.68691 4.42651 2.77647 2.73262 3.45900 2.40773 3.72430 3.29235 2.67798 2.69429 4.25047 2.90150 2.73789 3.18116 2.89673 2.37708 2.77571 2.98508 4.58792 3.60840
0.51887 0.91062 5.97975 2.99140 0.05152 0.48576 0.95510
399 2.26929 5.48416 2.16922 2.67590 4.82637 3.75501 3.39882 4.31015 1.86367 3.78809 3.03983 2.54272 2.73685 3.03981 2.56872 2.76722 3.21184 3.87328 5.91843 4.50943 679 - -
2.68618 4.42225 2.77519 2.73123 3.46354 2.40513 3.72494 3.29354 2.67741 2.69355 4.24690 2.90347 2.73739 3.18146 2.89801 2.37887 2.77519 2.98518 4.58477 3.61503
0.00795 5.23378 5.95613 0.61958 0.77255 0.48576 0.95510
400 1.95665 3.78764 4.42892 3.84432 3.31936 4.11483 4.41442 2.77168 3.73342 2.06483 3.63011 3.15294 4.48566 3.96231 3.93228 2.23226 2.35545 2.77045 5.12535 2.00296 680 - -
2.68618 4.42225 2.77519 2.73123 3.46354 2.40513 3.72494 3.29354 2.67741 2.69355 4.24690 2.90347 2.73739 3.18146 2.89801 2.37887 2.77519 2.98518 4.58477 3.61503
0.00795 5.23378 5.95613 0.61958 0.77255 0.48576 0.95510
401 2.99838 4.99918 2.82148 2.58178 4.18723 3.87981 4.09881 2.81199 2.96429 2.08235 3.66124 3.20058 4.26622 3.30310 2.01944 3.09660 3.22972 3.30269 5.53776 1.93785 681 - -
2.68618 4.42225 2.77519 2.73123 3.46354 2.40513 3.72494 3.29354 2.67741 2.69355 4.24690 2.90347 2.73739 3.18146 2.89801 2.37887 2.77519 2.98518 4.58477 3.61503
0.00795 5.23378 5.95613 0.61958 0.77255 0.48576 0.95510
402 2.80721 4.28712 3.66431 3.10497 2.12488 3.92262 4.15543 3.44965 3.07973 3.13045 3.99967 2.07980 4.31013 2.81602 3.48782 1.69017 3.24364 3.19657 5.44252 2.76094 682 - -
2.68618 4.42225 2.77519 2.73123 3.46354 2.40513 3.72494 3.29354 2.67741 2.69355 4.24690 2.90347 2.73739 3.18146 2.89801 2.37887 2.77519 2.98518 4.58477 3.61503

0.00795 5.23378 5.95613 0.61958 0.77255 0.48576 0.95510

403 2.81463 4.66525 4.01049 2.12172 3.79165 4.02224 4.28682 2.64579 2.47821 2.22817 2.59357 3.81050 2.64600 3.66872 3.70303 3.27617 3.03387 2.35885 2.81665 4.03417 683 - -

2.68618 4.42225 2.77519 2.73123 3.46354 2.40513 3.72494 3.29354 2.67741 2.69355 4.24690 2.90347 2.73739 3.18146 2.89801 2.37887 2.77519 2.98518 4.58477 3.61503

0.00795 5.23378 5.95613 0.61958 0.77255 0.48576 0.95510

404 3.08637 4.46350 5.00498 4.39542 3.04821 2.60963 4.56178 2.33894 4.18983 1.09258 3.21731 4.39674 4.60995 4.32131 4.20985 3.56222 3.31788 2.70078 2.52794 3.13849 684 - -

2.68618 4.42225 2.77519 2.73123 3.46354 2.40513 3.72494 3.29354 2.67741 2.69355 4.24690 2.90347 2.73739 3.18146 2.89801 2.37887 2.77519 2.98518 4.58477 3.61503

0.00795 5.23378 5.95613 0.61958 0.77255 0.48576 0.95510

405 3.08887 5.06488 4.00284 3.63230 4.91447 3.83449 4.76768 4.27177 3.69227 3.98122 4.81707 0.72946 4.49571 3.99814 4.07138 2.32938 2.72271 2.30396 6.25199 4.99589 685 - -

2.68618 4.42225 2.77519 2.73123 3.46354 2.40513 3.72494 3.29354 2.67741 2.69355 4.24690 2.90347 2.73739 3.18146 2.89801 2.37887 2.77519 2.98518 4.58477 3.61503

0.00795 5.23378 5.95613 0.61958 0.77255 0.48576 0.95510

406 4.15619 5.60757 4.95914 4.51687 2.96637 4.94970 1.56777 4.17348 4.21045 2.27199 4.73910 4.60355 5.28794 3.35975 4.39106 4.28125 4.37109 4.01491 4.61625 0.82794 686 - -

2.68618 4.42225 2.77519 2.73123 3.46354 2.40513 3.72494 3.29354 2.67741 2.69355 4.24690 2.90347 2.73739 3.18146 2.89801 2.37887 2.77519 2.98518 4.58477 3.61503

0.00795 5.23378 5.95613 0.61958 0.77255 0.48576 0.95510

407 2.04214 4.85852 3.76201 3.20614 4.06670 3.92396 4.22930 3.15303 3.17609 1.94220 4.00450 2.56056 4.33814 3.49589 3.56875 1.62160 2.36175 3.18040 5.47282 4.21693 687 - -

2.68618 4.42225 2.77519 2.73123 3.46354 2.40513 3.72494 3.29354 2.67741 2.69355 4.24690 2.90347 2.73739 3.18146 2.89801 2.37887 2.77519 2.98518 4.58477 3.61503

0.00795 5.23378 5.95613 0.61958 0.77255 0.48576 0.95510

408 4.28552 6.25947 5.10534 3.97831 5.96982 4.74968 3.33530 5.15695 0.80878 4.41924 5.33099 4.23604 5.02896 3.51480 1.04436 4.24114 4.33087 4.86920 6.31334 5.30559 688 - -

2.68618 4.42225 2.77519 2.73123 3.46354 2.40513 3.72494 3.29354 2.67741 2.69355 4.24690 2.90347 2.73739 3.18146 2.89801 2.37887 2.77519 2.98518 4.58477 3.61503

0.00795 5.23378 5.95613 0.61958 0.77255 0.48576 0.95510

409 3.08327 4.47478 4.90906 4.30879 1.62178 4.23426 4.51936 2.23310 4.12053 1.31952 3.57948 3.11735 4.59657 4.26993 4.17146 3.03701 3.31520 2.71652 5.02153 3.22708 689 - -

2.68618 4.42225 2.77519 2.73123 3.46354 2.40513 3.72494 3.29354 2.67741 2.69355 4.24690 2.90347 2.73739 3.18146 2.89801 2.37887 2.77519 2.98518 4.58477 3.61503

0.00795 5.23378 5.95613 0.61958 0.77255 0.48576 0.95510

410 3.67517 5.72551 4.24646 3.49975 4.77956 4.37208 3.59348 4.44170 1.92685 3.21332 4.80220 3.89449 4.70921 3.43632 1.43087 3.69491 3.83744 4.15026 5.88285 1.18112 690 - -

2.68618 4.42225 2.77519 2.73123 3.46354 2.40513 3.72494 3.29354 2.67741 2.69355 4.24690 2.90347 2.73739 3.18146 2.89801 2.37887 2.77519 2.98518 4.58477 3.61503

0.00795 5.23378 5.95613 0.61958 0.77255 0.48576 0.95510

411 2.40787 5.06805 5.81784 5.27304 4.05021 5.20314 5.67576 1.29999 5.13887 0.95224 3.85674 5.36593 5.44845 5.25159 5.19133 4.57404 4.04804 2.07015 5.99936 4.87444 691 - -

2.68618 4.42225 2.77519 2.73123 3.46354 2.40513 3.72494 3.29354 2.67741 2.69355 4.24690 2.90347 2.73739 3.18146 2.89801 2.37887 2.77519 2.98518 4.58477 3.61503

0.00795 5.23378 5.95613 0.61958 0.77255 0.48576 0.95510

412 2.36338 4.63498 4.41502 3.87039 2.44815 3.49532 4.49772 3.18612 3.77686 2.92005 3.83531 4.09215 1.73795 4.02098 4.00402 3.18390 3.33413 2.96805 1.51385 4.00225 692 - -

2.68618 4.42225 2.77519 2.73123 3.46354 2.40513 3.72494 3.29354 2.67741 2.69355 4.24690 2.90347 2.73739 3.18146 2.89801 2.37887 2.77519 2.98518 4.58477 3.61503

0.00795 5.23378 5.95613 0.61958 0.77255 0.48576 0.95510

413 2.62356 5.52050 2.23627 1.73903 4.86548 3.76218 3.95982 4.35332 2.26843 3.82561 4.56217 2.28082 4.16321 2.05761 3.00442 2.59553 3.23620 3.91128 5.95208 4.53794 693 - -

2.68618 4.42225 2.77519 2.73123 3.46354 2.40513 3.72494 3.29354 2.67741 2.69355 4.24690 2.90347 2.73739 3.18146 2.89801 2.37887 2.77519 2.98518 4.58477 3.61503

0.00795 5.23378 5.95613 0.61958 0.77255 0.48576 0.95510

414 4.07851 5.32809 6.07131 5.47551 2.37844 5.40638 5.71793 1.86060 5.31230 0.93255 2.00538 5.58297 5.53210 5.22992 5.25388 4.77016 4.28921 2.03047 5.84191 4.83998 694 - -

2.68618 4.42225 2.77519 2.73123 3.46354 2.40513 3.72494 3.29354 2.67741 2.69355 4.24690 2.90347 2.73739 3.18146 2.89801 2.37887 2.77519 2.98518 4.58477 3.61503

0.00795 5.23378 5.95613 0.61958 0.77255 0.48576 0.95510

415 3.02827 4.45860 4.68214 3.47997 2.01689 4.15965 4.47495 2.78476 3.93361 2.07704 3.57496 4.21379 4.52667 4.12139 4.05001 2.48124 1.67679 2.26646 5.07383 2.72651 695 - -

2.68644 4.42251 2.77502 2.73126 3.46379 2.40538 3.72520 3.29363 2.67766 2.69380 4.24715 2.90372 2.73490 3.18172 2.89816 2.37892 2.77534 2.98544 4.58502 3.61529

0.49525 0.94677 5.95613 0.28635 1.39031 0.48576 0.95510

416 2.75764 3.17899 1.54811 2.03520 4.85962 3.17884 3.96918 4.34481 1.97185 3.82272 4.56286 2.95105 4.17037 3.06869 3.20912 2.63184 3.24459 3.90754 5.95390 4.54149 698 - -

2.68618 4.42225 2.77519 2.73123 3.46354 2.40513 3.72494 3.29354 2.67741 2.69355 4.24690 2.90347 2.73739 3.18146 2.89801 2.37887 2.77519 2.98518 4.58477 3.61503

0.00795 5.23378 5.95613 0.61958 0.77255 0.48576 0.95510

417 3.92282 5.21959 5.65281 5.14690 3.36581 5.07287 4.93287 3.09676 4.94199 1.21592 3.83130 5.10092 5.34046 4.98076 4.94562 4.42437 4.15046 2.13512 5.14585 0.99328 699 - -

2.68618 4.42225 2.77519 2.73123 3.46354 2.40513 3.72494 3.29354 2.67741 2.69355 4.24690 2.90347 2.73739 3.18146 2.89801 2.37887 2.77519 2.98518 4.58477 3.61503

0.00795 5.23378 5.95613 0.61958 0.77255 0.48576 0.95510

418 3.03198 5.55178 1.72264 2.07682 4.89557 3.77360 3.57281 4.38440 2.03452 3.85602 4.59494 2.16121 4.18273 3.08182 2.87369 2.22872 3.26540 3.94215 5.98257 4.56602 700 - -

2.68618 4.42225 2.77519 2.73123 3.46354 2.40513 3.72494 3.29354 2.67741 2.69355 4.24690 2.90347 2.73739 3.18146 2.89801 2.37887 2.77519 2.98518 4.58477 3.61503

0.00795 5.23378 5.95613 0.61958 0.77255 0.48576 0.95510

419 4.37629 6.29629 5.25076 4.07843 6.05057 4.80099 4.44063 5.22149 0.54628 4.47266 5.39729 4.31037 5.08544 3.56595 1.37061 4.33550 4.41307 4.94107 6.35182 5.37005 701 - -

2.68618 4.42225 2.77519 2.73123 3.46354 2.40513 3.72494 3.29354 2.67741 2.69355 4.24690 2.90347 2.73739 3.18146 2.89801 2.37887 2.77519 2.98518 4.58477 3.61503

0.00795 5.23378 5.95613 0.61958 0.77255 0.48576 0.95510

420 3.98701 5.18124 6.10044 5.60864 4.31670 5.57813 6.20573 1.32686 5.53429 2.18051 2.41656 5.74578 5.75861 5.68437 5.62918 4.99836 4.23544 0.82532 6.43231 5.29054 702 - -

2.68618 4.42225 2.77519 2.73123 3.46354 2.40513 3.72494 3.29354 2.67741 2.69355 4.24690 2.90347 2.73739 3.18146 2.89801 2.37887 2.77519 2.98518 4.58477 3.61503

0.00795 5.23378 5.95613 0.61958 0.77255 0.48576 0.95510

421 4.05819 5.22073 6.21995 5.77482 4.53191 5.77985 6.60273 1.13112 5.75200 1.92691 4.22912 5.95074 5.93445 5.97691 5.91258 5.24518 4.31585 0.82783 6.77539 5.58672 703 - -

2.68618 4.42225 2.77519 2.73123 3.46354 2.40513 3.72494 3.29354 2.67741 2.69355 4.24690 2.90347 2.73739 3.18146 2.89801 2.37887 2.77519 2.98518 4.58477 3.61503

0.00795 5.23378 5.95613 0.61958 0.77255 0.48576 0.95510

422 3.82845 5.12579 5.62130 5.10006 1.21161 4.95692 4.76694 2.79026 4.90108 2.08119 2.38049 4.99058 5.23285 4.89070 4.86460 4.29274 4.04880 3.25361 4.97866 1.35011 704 - -

2.68618 4.42225 2.77519 2.73123 3.46354 2.40513 3.72494 3.29354 2.67741 2.69355 4.24690 2.90347 2.73739 3.18146 2.89801 2.37887 2.77519 2.98518 4.58477 3.61503

0.00795 5.23378 5.95613 0.61958 0.77255 0.48576 0.95510

423 4.52337 5.67860 6.52185 5.94836 3.04697 6.00447 6.27302 1.68816 5.83656 0.47443 3.51662 6.18819 5.91158 5.58011 5.72117 5.43383 4.71681 2.86156 6.10813 5.19440 705 - -

2.68618 4.42225 2.77519 2.73123 3.46354 2.40513 3.72494 3.29354 2.67741 2.69355 4.24690 2.90347 2.73739 3.18146 2.89801 2.37887 2.77519 2.98518 4.58477 3.61503

0.00795 5.23378 5.95613 0.61958 0.77255 0.48576 0.95510

424 5.04227 6.84107 0.08872 4.11140 6.54517 4.83192 5.82430 6.55903 5.19496 5.90800 7.04210 4.68670 5.54030 5.16145 5.72655 4.96346 5.43001 6.09709 7.32158 6.40767 706 - -

2.68618 4.42225 2.77519 2.73123 3.46354 2.40513 3.72494 3.29354 2.67741 2.69355 4.24690 2.90347 2.73739 3.18146 2.89801 2.37887 2.77519 2.98518 4.58477 3.61503

0.00795 5.23378 5.95613 0.61958 0.77255 0.48576 0.95510

425 1.48040 5.39446 1.83472 2.78394 4.75178 2.85261 3.29270 4.20808 2.82486 3.74396 4.51579 3.31758 4.21847 3.17080 3.31023 2.23666 3.27844 3.05955 5.92194 4.54139 707 - -

2.68618 4.42225 2.77519 2.73123 3.46354 2.40513 3.72494 3.29354 2.67741 2.69355 4.24690 2.90347 2.73739 3.18146 2.89801 2.37887 2.77519 2.98518 4.58477 3.61503

0.00795 5.23378 5.95613 0.61958 0.77255 0.48576 0.95510

426 5.04227 6.84107 0.08872 4.11140 6.54517 4.83192 5.82430 6.55903 5.19496 5.90800 7.04210 4.68670 5.54030 5.16145 5.72655 4.96346 5.43001 6.09709 7.32158 6.40767 708 - -

2.68618 4.42225 2.77519 2.73123 3.46354 2.40513 3.72494 3.29354 2.67741 2.69355 4.24690 2.90347 2.73739 3.18146 2.89801 2.37887 2.77519 2.98518 4.58477 3.61503

0.00795 5.23378 5.95613 0.61958 0.77255 0.48576 0.95510

427 3.08329 4.46087 4.98888 4.38127 2.86196 4.25784 4.59415 1.58617 4.18338 1.95293 3.03690 2.71396 4.61779 4.32739 4.21609 3.56875 2.35011 1.72248 5.10227 3.92520 709 - -

2.68618 4.42225 2.77519 2.73123 3.46354 2.40513 3.72494 3.29354 2.67741 2.69355 4.24690 2.90347 2.73739 3.18146 2.89801 2.37887 2.77519 2.98518 4.58477 3.61503

0.00795 5.23378 5.95613 0.61958 0.77255 0.48576 0.95510

428 3.61254 4.91496 5.53017 4.96505 3.96796 4.93326 5.34377 1.29631 4.80455 1.65045 3.83174 5.05872 5.22186 2.65236 4.87685 4.27780 3.85064 1.23619 5.77272 4.61702 710 - -

2.68618 4.42225 2.77519 2.73123 3.46354 2.40513 3.72494 3.29354 2.67741 2.69355 4.24690 2.90347 2.73739 3.18146 2.89801 2.37887 2.77519 2.98518 4.58477 3.61503

0.00795 5.23378 5.95613 0.61958 0.77255 0.48576 0.95510

429 3.80679 5.03850 5.88648 5.37174 2.50546 5.30042 5.85257 1.55605 5.26369 2.55878 4.08458 5.46259 5.55048 5.44415 5.35201 4.68843 3.67941 0.68572 6.20770 5.01557 711 - -

2.68618 4.42225 2.77519 2.73123 3.46354 2.40513 3.72494 3.29354 2.67741 2.69355 4.24690 2.90347 2.73739 3.18146 2.89801 2.37887 2.77519 2.98518 4.58477 3.61503

0.00795 5.23378 5.95613 0.61958 0.77255 0.48576 0.95510

430 3.24901 4.65882 4.90756 4.31724 2.54686 4.39172 4.66411 2.93227 4.15711 1.47259 3.59412 4.44228 4.73350 1.71767 4.26210 3.69515 3.47860 1.59038 5.18766 3.65604 712 - -

2.68618 4.42225 2.77519 2.73123 3.46354 2.40513 3.72494 3.29354 2.67741 2.69355 4.24690 2.90347 2.73739 3.18146 2.89801 2.37887 2.77519 2.98518 4.58477 3.61503

0.00795 5.23378 5.95613 0.61958 0.77255 0.48576 0.95510

431 3.03303 5.53838 2.24917 2.54362 4.88646 3.31111 3.97707 4.36902 1.30522 3.84058 4.58193 3.25272 4.18676 3.07620 2.01631 2.63289 3.03859 3.93141 5.96469 4.55895 713 - -

2.68618 4.42225 2.77519 2.73123 3.46354 2.40513 3.72494 3.29354 2.67741 2.69355 4.24690 2.90347 2.73739 3.18146 2.89801 2.37887 2.77519 2.98518 4.58477 3.61503

0.00795 5.23378 5.95613 0.61958 0.77255 0.48576 0.95510

432 3.86338 6.58204 1.02319 2.86186 5.86720 4.01649 4.61344 5.44207 2.45939 4.87502 5.74642 1.10322 4.67720 3.78440 4.31782 3.70305 4.16500 4.96497 7.00666 5.43068 714 - -

2.68618 4.42225 2.77519 2.73123 3.46354 2.40513 3.72494 3.29354 2.67741 2.69355 4.24690 2.90347 2.73739 3.18146 2.89801 2.37887 2.77519 2.98518 4.58477 3.61503

0.00795 5.23378 5.95613 0.61958 0.77255 0.48576 0.95510

433 2.80668 4.87189 5.54029 4.98021 4.00435 4.89147 5.33661 1.04413 4.82703 1.25252 3.88055 5.04505 5.19961 4.98289 4.89036 4.24036 2.91282 2.15421 5.77394 4.60612 715 - -

2.68618 4.42225 2.77519 2.73123 3.46354 2.40513 3.72494 3.29354 2.67741 2.69355 4.24690 2.90347 2.73739 3.18146 2.89801 2.37887 2.77519 2.98518 4.58477 3.61503

0.00795 5.23378 5.95613 0.61958 0.77255 0.48576 0.95510

434 2.51671 5.54894 1.49992 2.06997 4.88720 3.78458 4.01104 4.37011 2.77114 3.85571 4.60350 3.25639 4.20319 2.98437 3.26988 2.06329 2.25496 3.70722 5.99411 4.58093 716 - -

2.68618 4.42225 2.77519 2.73123 3.46354 2.40513 3.72494 3.29354 2.67741 2.69355 4.24690 2.90347 2.73739 3.18146 2.89801 2.37887 2.77519 2.98518 4.58477 3.61503

0.00795 5.23378 5.95613 0.61958 0.77255 0.48576 0.95510

435 2.97956 5.47639 2.35203 2.17632 3.10278 2.98797 2.65658 4.29756 2.11072 3.19498 4.51858 3.22508 2.51589 3.04131 2.81702 2.64309 3.21072 3.86391 5.91254 3.86410 717 - -

2.68618 4.42225 2.77519 2.73123 3.46354 2.40513 3.72494 3.29354 2.67741 2.69355 4.24690 2.90347 2.73739 3.18146 2.89801 2.37887 2.77519 2.98518 4.58477 3.61503

0.00795 5.23378 5.95613 0.61958 0.77255 0.48576 0.95510

436 5.03213 6.17607 6.31057 6.08270 4.26401 5.68175 6.43848 3.61193 5.93356 0.12972 4.09688 6.31147 6.00364 5.93124 5.84445 5.70644 5.29975 3.94624 6.40738 5.40779 718 - -

2.68618 4.42225 2.77519 2.73123 3.46354 2.40513 3.72494 3.29354 2.67741 2.69355 4.24690 2.90347 2.73739 3.18146 2.89801 2.37887 2.77519 2.98518 4.58477 3.61503

0.00795 5.23378 5.95613 0.61958 0.77255 0.48576 0.95510

437 4.02888 6.02851 6.00847 5.90037 0.70128 5.56596 4.39686 4.56276 5.65539 3.74085 5.10195 5.22766 5.86455 5.38094 5.48328 4.99103 5.20984 4.53729 1.15938 2.73108 719 - -

2.68618 4.42225 2.77519 2.73123 3.46354 2.40513 3.72494 3.29354 2.67741 2.69355 4.24690 2.90347 2.73739 3.18146 2.89801 2.37887 2.77519 2.98518 4.58477 3.61503

0.00795 5.23378 5.95613 0.61958 0.77255 0.48576 0.95510

438 2.87209 5.49223 2.12792 2.07419 4.83790 3.75307 3.14173 4.32430 1.95832 3.79772 4.17496 2.99269 4.14682 2.37810 3.04682 2.01510 3.21127 3.88320 5.92447 3.69810 720 - -

2.68618 4.42225 2.77519 2.73123 3.46354 2.40513 3.72494 3.29354 2.67741 2.69355 4.24690 2.90347 2.73739 3.18146 2.89801 2.37887 2.77519 2.98518 4.58477 3.61503

0.00795 5.23378 5.95613 0.61958 0.77255 0.48576 0.95510

439 3.04594 2.09424 5.01290 4.39803 2.64541 4.21797 4.55250 1.87129 4.18306 2.05297 2.78820 4.38124 4.58013 4.31316 4.19047 3.52990 2.19976 2.22480 5.04336 3.01487 721 - -

2.68618 4.42225 2.77519 2.73123 3.46354 2.40513 3.72494 3.29354 2.67741 2.69355 4.24690 2.90347 2.73739 3.18146 2.89801 2.37887 2.77519 2.98518 4.58477 3.61503

0.00795 5.23378 5.95613 0.61958 0.77255 0.48576 0.95510

440 3.04763 5.55932 1.76344 2.60769 4.89729 2.57812 3.68274 4.38410 2.67899 3.86186 4.60513 2.82823 2.01166 2.95028 3.25342 2.08956 3.28225 3.69155 5.99403 4.57748 722 - -

2.68618 4.42225 2.77519 2.73123 3.46354 2.40513 3.72494 3.29354 2.67741 2.69355 4.24690 2.90347 2.73739 3.18146 2.89801 2.37887 2.77519 2.98518 4.58477 3.61503

0.00795 5.23378 5.95613 0.61958 0.77255 0.48576 0.95510

441 2.98314 5.43681 2.78176 2.25990 4.75933 3.76543 3.95424 3.86574 2.14819 2.01948 2.54837 3.12278 4.15827 2.19287 2.60324 2.85282 3.21414 3.81531 5.88282 4.48686 723 - -

2.68661 4.42268 2.77510 2.73133 3.46397 2.40352 3.72537 3.29397 2.67729 2.69398 4.24733 2.90301 2.73772 3.18148 2.89810 2.37893 2.77516 2.98561 4.58520 3.61546

0.49525 0.94677 5.95613 0.75786 0.63237 0.48576 0.95510

442 2.89574 4.83168 3.73061 3.16647 2.11937 2.79337 3.85529 2.59361 1.74501 2.61700 3.23625 3.07583 4.32396 3.45318 2.87735 3.03378 3.23539 2.97147 5.39841 4.14359 727 - -

2.68618 4.42225 2.77519 2.73123 3.46354 2.40513 3.72494 3.29354 2.67741 2.69355 4.24690 2.90347 2.73739 3.18146 2.89801 2.37887 2.77519 2.98518 4.58477 3.61503

0.46591 5.23378 1.00211 0.61958 0.77255 0.48576 0.95510

443 2.82993 4.31251 4.26174 3.68307 3.41406 3.92653 4.23260 2.52542 3.55501 2.50205 3.43095 3.54296 4.29967 3.51114 3.74327 3.21605 2.67456 1.16151 4.92786 2.61753 728 - -

2.68618 4.42225 2.77519 2.73123 3.46354 2.40513 3.72494 3.29354 2.67741 2.69355 4.24690 2.90347 2.73739 3.18146 2.89801 2.37887 2.77519 2.98518 4.58477 3.61503

0.01255 4.78042 5.50276 0.61958 0.77255 1.31804 0.31151

444 2.95635 4.39797 4.47577 3.89487 2.85907 4.09609 4.40929 2.08397 3.77269 1.86914 3.44198 1.72257 4.45001 3.98446 3.94233 3.39204 3.18994 1.87545 5.04473 3.84813 729 - -

2.68618 4.42225 2.77519 2.73123 3.46354 2.40513 3.72494 3.29354 2.67741 2.69355 4.24690 2.90347 2.73739 3.18146 2.89801 2.37887 2.77519 2.98518 4.58477 3.61503

0.04120 4.78042 3.44289 0.61958 0.77255 1.31804 0.31151

445 2.43518 4.81004 3.45346 3.04844 3.84562 1.17172 3.53758 3.53757 3.07457 3.19576 4.07974 3.47216 4.19927 3.40957 3.45463 3.03688 3.19783 3.25025 5.29562 2.42537 730 - -

2.68618 4.42225 2.77519 2.73123 3.46354 2.40513 3.72494 3.29354 2.67741 2.69355 4.24690 2.90347 2.73739 3.18146 2.89801 2.37887 2.77519 2.98518 4.58477 3.61503

0.01291 4.75212 5.47447 0.61958 0.77255 1.34515 0.30178

446 0.35247 4.68891 4.42786 4.33116 5.20637 2.95805 5.25742 4.60951 4.42855 4.36715 5.19793 4.15711 4.30651 4.63553 4.58332 2.99482 3.34777 3.91292 6.52228 5.44164 731 - -

2.68618 4.42225 2.77519 2.73123 3.46354 2.40513 3.72494 3.29354 2.67741 2.69355 4.24690 2.90347 2.73739 3.18146 2.89801 2.37887 2.77519 2.98518 4.58477 3.61503

0.01291 4.75212 5.47447 0.61958 0.77255 1.34515 0.30178

447 3.07918 4.71799 4.57736 4.25780 4.24259 3.95298 5.03557 2.78556 4.17285 3.06362 4.18433 4.33602 1.95071 4.47670 4.36622 3.42767 3.48632 0.71953 5.88217 4.67427 732 - -

2.68618 4.42225 2.77519 2.73123 3.46354 2.40513 3.72494 3.29354 2.67741 2.69355 4.24690 2.90347 2.73739 3.18146 2.89801 2.37887 2.77519 2.98518 4.58477 3.61503

0.01291 4.75212 5.47447 0.61958 0.77255 1.34515 0.30178

448 2.83330 5.13034 3.15600 1.30015 4.40298 3.62791 3.81546 3.80524 2.07419 3.14201 3.32732 3.13360 4.02092 2.94441 2.99568 2.83924 3.06227 3.10014 5.60304 4.25252 733 - -

2.68618 4.42225 2.77519 2.73123 3.46354 2.40513 3.72494 3.29354 2.67741 2.69355 4.24690 2.90347 2.73739 3.18146 2.89801 2.37887 2.77519 2.98518 4.58477 3.61503

0.01291 4.75212 5.47447 0.61958 0.77255 1.34515 0.30178

449 2.85801 5.04570 2.14905 2.66343 4.29443 3.61729 3.91355 3.75161 2.75299 3.35827 4.18052 3.18212 4.06185 3.07837 3.22173 2.44180 1.66228 3.41986 5.58059 2.36157 734 - -

2.68618 4.42225 2.77519 2.73123 3.46354 2.40513 3.72494 3.29354 2.67741 2.69355 4.24690 2.90347 2.73739 3.18146 2.89801 2.37887 2.77519 2.98518 4.58477 3.61503
0.01291 4.75212 5.47447 0.61958 0.77255 1.34515 0.30178
450 2.78039 0.44709 4.89872 4.76833 4.88284 3.49568 5.32636 4.25610 4.58150 4.11230 5.01829 4.30894 4.33317 4.82048 4.63024 2.52282 3.34196 3.70037 6.30908 5.14029 735 - -
2.68618 4.42225 2.77519 2.73123 3.46354 2.40513 3.72494 3.29354 2.67741 2.69355 4.24690 2.90347 2.73739 3.18146 2.89801 2.37887 2.77519 2.98518 4.58477 3.61503
0.01291 4.75212 5.47447 0.61958 0.77255 1.34515 0.30178
451 2.78652 4.61719 3.50988 2.94563 2.24595 2.98788 2.60917 3.14320 2.67254 2.49287 3.33547 2.99593 4.10549 3.23248 2.71925 2.95491 2.69157 2.90143 3.94507 3.92722 736 - -
2.68618 4.42225 2.77519 2.73123 3.46354 2.40513 3.72494 3.29354 2.67741 2.69355 4.24690 2.90347 2.73739 3.18146 2.89801 2.37887 2.77519 2.98518 4.58477 3.61503
0.01291 4.75212 5.47447 0.61958 0.77255 1.34515 0.30178
452 1.92208 2.36056 3.45647 1.94311 4.08690 2.41702 4.02808 3.45928 2.91586 3.13799 3.98967 3.36917 4.08514 3.24495 3.33371 2.88955 3.05266 2.73747 5.44464 4.16691 737 - -
2.68653 4.42260 2.77482 2.73023 3.46389 2.40490 3.72530 3.29355 2.67716 2.69367 4.24725 2.90362 2.73758 3.18182 2.89808 2.37894 2.77517 2.98529 4.58512 3.61538
0.47915 0.97684 5.47447 1.05355 0.42878 1.34515 0.30178
453 2.78665 3.76778 3.45126 2.89011 3.38294 3.70201 3.94000 3.21920 2.86188 2.90232 3.77156 2.24417 4.08966 2.81168 3.26791 1.95074 2.81330 2.96693 2.51035 3.96138 741 - -
2.68634 4.42148 2.77536 2.73140 3.46370 2.40529 3.72414 3.29370 2.67732 2.69371 4.24540 2.90363 2.73739 3.18163 2.89817 2.37851 2.77536 2.98488 4.58493 3.61492
0.38503 1.31883 2.95422 0.68196 0.70446 1.34515 0.30178
454 2.78900 4.40222 3.93475 3.38803 1.51372 3.24277 4.12368 2.92131 3.31991 2.64798 3.55974 3.69177 4.20647 3.58733 3.59904 2.33045 2.28420 2.70495 4.96314 3.20566 744 - -
2.68618 4.42225 2.77519 2.73123 3.46354 2.40513 3.72494 3.29354 2.67741 2.69355 4.24690 2.90347 2.73739 3.18146 2.89801 2.37887 2.77519 2.98518 4.58477 3.61503
0.01356 4.70301 5.42536 0.61958 0.77255 1.38884 0.28684
455 2.83393 5.18293 3.15373 2.58543 4.49143 3.61654 2.29437 3.93028 2.16258 3.25283 4.24191 3.11079 2.63592 2.89865 2.35927 2.82530 2.12208 3.55595 5.62774 4.27552 745 - -
2.68618 4.42225 2.77519 2.73123 3.46354 2.40513 3.72494 3.29354 2.67741 2.69355 4.24690 2.90347 2.73739 3.18146 2.89801 2.37887 2.77519 2.98518 4.58477 3.61503
0.01356 4.70301 5.42536 0.61958 0.77255 1.38884 0.28684
456 2.86176 5.30321 3.14388 2.56787 4.65830 3.62265 3.46918 4.11429 1.88509 3.29123 4.35008 2.94076 4.00828 2.15083 1.85206 2.38177 2.90442 3.70123 5.70943 4.34511 746 - -
2.68618 4.42225 2.77519 2.73123 3.46354 2.40513 3.72494 3.29354 2.67741 2.69355 4.24690 2.90347 2.73739 3.18146 2.89801 2.37887 2.77519 2.98518 4.58477 3.61503
0.01356 4.70301 5.42536 0.61958 0.77255 1.38884 0.28684
457 3.72623 4.99825 5.18841 4.76456 1.67632 4.70868 4.10676 3.26874 4.58211 2.25455 2.71678 4.56347 4.99960 4.55299 4.57302 4.04943 3.94505 3.23347 4.27338 0.92872 747 - -
2.68618 4.42225 2.77519 2.73123 3.46354 2.40513 3.72494 3.29354 2.67741 2.69355 4.24690 2.90347 2.73739 3.18146 2.89801 2.37887 2.77519 2.98518 4.58477 3.61503
0.01356 4.70301 5.42536 0.61958 0.77255 1.38884 0.28684
458 2.60347 5.18365 2.32045 2.49781 2.55209 3.22842 3.37820 3.95072 1.91754 3.47252 4.23537 3.03893 3.94934 2.85930 2.71191 2.41330 3.00102 3.55407 5.63951 4.25301 748 - -
2.68618 4.42225 2.77519 2.73123 3.46354 2.40513 3.72494 3.29354 2.67741 2.69355 4.24690 2.90347 2.73739 3.18146 2.89801 2.37887 2.77519 2.98518 4.58477 3.61503
0.01356 4.70301 5.42536 0.61958 0.77255 1.38884 0.28684
459 2.76932 5.15873 2.81933 2.51025 4.45782 3.56035 3.75288 2.58209 2.12991 3.44355 4.21256 2.76094 3.95336 2.53516 2.77435 2.43672 2.06393 3.52456 5.61954 4.23991 749 - -
2.68618 4.42225 2.77519 2.73123 3.46354 2.40513 3.72494 3.29354 2.67741 2.69355 4.24690 2.90347 2.73739 3.18146 2.89801 2.37887 2.77519 2.98518 4.58477 3.61503
0.01356 4.70301 5.42536 0.61958 0.77255 1.38884 0.28684
460 2.77254 4.75785 3.12707 2.77129 3.12639 2.40507 3.88012 3.33561 2.75746 2.99660 3.85043 3.25614 3.42820 3.09253 2.87894 2.45950 3.00599 3.06420 5.29366 1.97916 750 - -
2.68618 4.42225 2.77519 2.73123 3.46354 2.40513 3.72494 3.29354 2.67741 2.69355 4.24690 2.90347 2.73739 3.18146 2.89801 2.37887 2.77519 2.98518 4.58477 3.61503
0.01356 4.70301 5.42536 0.61958 0.77255 1.38884 0.28684

461 2.66491 4.20185 4.41854 3.82247 2.64567 3.01884 4.21607 2.32203 3.67105 1.73549 3.00281 3.95286 4.26788 3.85992 3.48196 3.00594 2.57155 2.45192 4.81767 2.38278 751 - -

2.68618 4.42225 2.77519 2.73123 3.46354 2.40513 3.72494 3.29354 2.67741 2.69355 4.24690 2.90347 2.73739 3.18146 2.89801 2.37887 2.77519 2.98518 4.58477 3.61503

0.01356 4.70301 5.42536 0.61958 0.77255 1.38884 0.28684

462 2.90532 2.31097 4.06193 3.57522 1.96020 3.82196 4.21000 3.13861 3.50558 2.85837 3.81407 1.44126 4.31546 3.77459 3.77410 3.16461 3.20889 2.91331 4.92896 3.49104 752 - -

2.68618 4.42225 2.77519 2.73123 3.46354 2.40513 3.72494 3.29354 2.67741 2.69355 4.24690 2.90347 2.73739 3.18146 2.89801 2.37887 2.77519 2.98518 4.58477 3.61503

0.01356 4.70301 5.42536 0.61958 0.77255 1.38884 0.28684

463 3.88836 5.19237 4.95937 4.56172 1.05840 4.71836 3.92416 3.73900 4.39828 3.17535 4.32567 4.42078 5.03243 2.05385 4.48059 4.05024 4.10555 3.60170 1.85541 2.17118 753 - -

2.68618 4.42225 2.77519 2.73123 3.46354 2.40513 3.72494 3.29354 2.67741 2.69355 4.24690 2.90347 2.73739 3.18146 2.89801 2.37887 2.77519 2.98518 4.58477 3.61503

0.01356 4.70301 5.42536 0.61958 0.77255 1.23061 0.34547

464 2.86961 4.95571 3.29762 2.78217 4.09259 3.68163 3.91611 3.60869 2.71688 2.79706 4.07622 3.27288 4.09856 2.29710 3.12753 1.22787 3.11290 3.31054 5.42664 3.60147 754 - -

2.68618 4.42225 2.77519 2.73123 3.46354 2.40513 3.72494 3.29354 2.67741 2.69355 4.24690 2.90347 2.73739 3.18146 2.89801 2.37887 2.77519 2.98518 4.58477 3.61503

0.01291 4.75212 5.47447 0.61958 0.77255 1.34515 0.30178

465 2.79876 3.05760 2.20752 2.43830 4.57074 3.56826 2.46795 4.04029 2.51648 3.54520 4.29972 2.17954 3.96821 2.87419 2.69847 2.48600 3.03107 3.62812 5.69807 4.30115 755 - -

2.68647 4.42254 2.77520 2.73105 3.46383 2.40542 3.72523 3.29383 2.67699 2.69384 4.24719 2.90375 2.73694 3.18161 2.89818 2.37916 2.77502 2.98437 4.58034 3.61532

0.17760 1.84183 5.47447 1.60799 0.22351 1.34515 0.30178

466 2.65393 5.18910 3.04399 2.44618 4.51805 3.61363 3.89501 3.95570 2.57165 3.51622 4.31513 3.14629 1.33140 2.72743 3.12920 2.56398 2.88421 3.58760 5.71624 4.35594 762 - -

2.68618 4.42225 2.77519 2.73123 3.46354 2.40513 3.72494 3.29354 2.67741 2.69355 4.24690 2.90347 2.73739 3.18146 2.89801 2.37887 2.77519 2.98518 4.58477 3.61503

0.01291 4.75212 5.47447 0.61958 0.77255 1.34515 0.30178

467 2.47359 4.70415 3.40919 2.53531 3.87587 3.43887 3.40411 3.25541 2.42475 1.90588 3.50800 3.31823 4.07635 3.15284 2.86347 2.77074 2.67641 2.64988 3.54459 3.98372 763 - -

2.68618 4.42225 2.77519 2.73123 3.46354 2.40513 3.72494 3.29354 2.67741 2.69355 4.24690 2.90347 2.73739 3.18146 2.89801 2.37887 2.77519 2.98518 4.58477 3.61503

0.01291 4.75212 5.47447 0.61958 0.77255 1.34515 0.30178

468 2.57709 4.38281 3.59527 2.82289 3.01177 3.82593 4.10158 1.70144 2.84554 2.09467 3.49239 3.39452 4.20080 3.53357 3.54920 2.92176 3.02036 2.34021 4.98082 3.48253 764 - -

2.68618 4.42225 2.77519 2.73123 3.46354 2.40513 3.72494 3.29354 2.67741 2.69355 4.24690 2.90347 2.73739 3.18146 2.89801 2.37887 2.77519 2.98518 4.58477 3.61503

0.01291 4.75212 5.47447 0.61958 0.77255 0.21030 1.66251

469 2.38373 2.27735 3.35405 2.62268 4.48074 3.80076 4.00233 3.90554 2.40208 3.49320 3.64566 3.19537 3.66081 3.14235 2.78577 1.52865 3.21163 3.37934 5.72144 4.37575 765 - -

2.68618 4.42225 2.77519 2.73123 3.46354 2.40513 3.72494 3.29354 2.67741 2.69355 4.24690 2.90347 2.73739 3.18146 2.89801 2.37887 2.77519 2.98518 4.58477 3.61503

0.00810 5.21590 5.93824 0.61958 0.77255 0.44929 1.01634

470 1.49331 5.40966 3.26729 2.15550 4.72138 2.66875 3.96186 3.83296 2.26739 2.77031 4.45970 3.25332 4.16408 2.87967 2.97200 2.50651 3.21570 3.78235 5.86275 4.47392 766 - -

2.68618 4.42225 2.77519 2.73123 3.46354 2.40513 3.72494 3.29354 2.67741 2.69355 4.24690 2.90347 2.73739 3.18146 2.89801 2.37887 2.77519 2.98518 4.58477 3.61503

0.00795 5.23378 5.95613 0.61958 0.77255 0.48576 0.95510

471 2.64651 5.28025 3.33244 2.77563 4.15492 2.90278 3.22855 3.98091 2.40931 3.38130 4.34358 1.77698 4.18839 3.12637 3.24651 2.57162 2.14064 2.47867 5.76423 4.40686 767 - -

2.68618 4.42225 2.77519 2.73123 3.46354 2.40513 3.72494 3.29354 2.67741 2.69355 4.24690 2.90347 2.73739 3.18146 2.89801 2.37887 2.77519 2.98518 4.58477 3.61503

0.00795 5.23378 5.95613 0.61958 0.77255 0.48576 0.95510

472 2.77362 4.52676 4.39768 3.57289 1.45165 2.23481 3.98101 2.86889 3.71170 1.78348 3.64203 4.05852 3.51517 3.94604 3.91942 3.23995 3.25736 2.78335 5.13531 3.93820 768 - -

2.68636 4.42253 2.77388 2.73141 3.46382 2.40505 3.72522 3.29382 2.67752 2.69372 4.24717 2.90287 2.73767 3.18174 2.89795 2.37873 2.77547 2.98524 4.58505 3.61531

0.49525 0.94677 5.95613 0.35705 1.20309 0.48576 0.95510

473 2.69182 5.41722 3.27365 2.71636 4.73156 3.77590 3.96372 2.80537 1.97300 3.70796 4.46705 2.13433 1.98139 2.78100 2.63870 2.96904 2.91650 3.56505 5.86779 4.47999 774 - -

2.68618 4.42225 2.77519 2.73123 3.46354 2.40513 3.72494 3.29354 2.67741 2.69355 4.24690 2.90347 2.73739 3.18146 2.89801 2.37887 2.77519 2.98518 4.58477 3.61503

0.00795 5.23378 5.95613 0.61958 0.77255 0.48576 0.95510

474 2.98502 5.49827 2.52193 2.29249 4.84397 3.37110 2.67994 4.33072 2.07101 3.80373 4.53902 1.82790 2.81713 3.04075 2.73010 2.79241 2.95213 3.88930 5.93027 4.51835 775 - -

2.68618 4.42225 2.77519 2.73123 3.46354 2.40513 3.72494 3.29354 2.67741 2.69355 4.24690 2.90347 2.73739 3.18146 2.89801 2.37887 2.77519 2.98518 4.58477 3.61503

0.00795 5.23378 5.95613 0.61958 0.77255 0.48576 0.95510

475 1.78612 5.40238 3.02781 2.42870 4.04767 3.13194 3.96491 4.17342 2.40662 3.69359 4.45470 3.25533 1.96497 3.07470 3.20244 2.22615 3.21594 3.18734 5.85931 4.47145 776 - -

2.68618 4.42225 2.77519 2.73123 3.46354 2.40513 3.72494 3.29354 2.67741 2.69355 4.24690 2.90347 2.73739 3.18146 2.89801 2.37887 2.77519 2.98518 4.58477 3.61503

0.00795 5.23378 5.95613 0.61958 0.77255 0.48576 0.95510

476 3.02621 1.30398 4.43122 2.55304 3.62839 4.11674 4.41993 2.96878 3.73427 2.33114 3.63670 4.07736 3.95903 3.96584 3.93369 3.40038 2.51432 2.28773 3.61846 3.93839 777 - -

2.68618 4.42225 2.77519 2.73123 3.46354 2.40513 3.72494 3.29354 2.67741 2.69355 4.24690 2.90347 2.73739 3.18146 2.89801 2.37887 2.77519 2.98518 4.58477 3.61503

0.00795 5.23378 5.95613 0.61958 0.77255 0.48576 0.95510

477 2.24953 4.52484 4.40435 3.82054 3.63156 2.65254 4.40882 2.09371 3.70949 1.88727 3.63907 4.06008 4.48270 3.94548 2.57473 3.39262 2.82925 2.77921 5.13403 2.03827 778 - -

2.68618 4.42225 2.77519 2.73123 3.46354 2.40513 3.72494 3.29354 2.67741 2.69355 4.24690 2.90347 2.73739 3.18146 2.89801 2.37887 2.77519 2.98518 4.58477 3.61503

0.00795 5.23378 5.95613 0.61958 0.77255 0.48576 0.95510

478 5.08582 6.05114 6.07465 5.98738 0.81478 5.66213 4.35477 4.60849 5.75362 2.16477 5.12999 5.23598 5.90952 5.39595 5.53505 5.06068 5.27920 4.57999 1.37473 2.27650 779 - -

2.68618 4.42225 2.77519 2.73123 3.46354 2.40513 3.72494 3.29354 2.67741 2.69355 4.24690 2.90347 2.73739 3.18146 2.89801 2.37887 2.77519 2.98518 4.58477 3.61503

0.00795 5.23378 5.95613 0.61958 0.77255 0.48576 0.95510

479 1.80758 5.14949 3.48088 2.94714 4.37332 3.86901 2.71292 3.79857 2.92143 3.12529 3.51511 1.26690 4.27728 3.28365 3.35818 3.10085 3.28083 3.49371 5.67873 4.35593 780 - -

2.68618 4.42225 2.77519 2.73123 3.46354 2.40513 3.72494 3.29354 2.67741 2.69355 4.24690 2.90347 2.73739 3.18146 2.89801 2.37887 2.77519 2.98518 4.58477 3.61503

0.00795 5.23378 5.95613 0.61958 0.77255 0.48576 0.95510

480 2.29442 4.66457 4.17154 3.61159 2.55355 4.03371 4.38372 3.18827 3.54382 2.91603 3.82257 3.92803 4.45113 3.81582 3.83476 1.51828 1.86790 2.96898 5.27027 2.42868 781 - -

2.68618 4.42225 2.77519 2.73123 3.46354 2.40513 3.72494 3.29354 2.67741 2.69355 4.24690 2.90347 2.73739 3.18146 2.89801 2.37887 2.77519 2.98518 4.58477 3.61503

0.00795 5.23378 5.95613 0.61958 0.77255 0.48576 0.95510

481 4.93323 6.40146 5.78358 5.85066 6.68996 0.04823 6.69126 6.75204 6.14149 6.12498 7.22189 5.95711 5.77909 6.37886 6.09390 5.16100 5.47638 6.07920 7.31103 6.87939 782 - -

2.68618 4.42225 2.77519 2.73123 3.46354 2.40513 3.72494 3.29354 2.67741 2.69355 4.24690 2.90347 2.73739 3.18146 2.89801 2.37887 2.77519 2.98518 4.58477 3.61503

0.00795 5.23378 5.95613 0.61958 0.77255 0.48576 0.95510

482 4.06620 5.28574 6.10439 5.55449 3.95897 5.51642 5.94511 2.09669 5.43062 1.51819 1.29717 5.68623 5.65251 5.42723 5.43150 4.90584 4.29377 1.26981 6.08592 5.04768 783 - -

2.68618 4.42225 2.77519 2.73123 3.46354 2.40513 3.72494 3.29354 2.67741 2.69355 4.24690 2.90347 2.73739 3.18146 2.89801 2.37887 2.77519 2.98518 4.58477 3.61503

0.00795 5.23378 5.95613 0.61958 0.77255 0.48576 0.95510

483 3.42489 4.87708 4.81741 4.27126 2.01671 4.50367 4.64015 3.12035 4.13848 2.33450 1.28718 1.65318 4.84967 4.33810 4.30472 3.80948 3.65796 3.03985 5.13843 3.75193 784 - -

2.68618 4.42225 2.77519 2.73123 3.46354 2.40513 3.72494 3.29354 2.67741 2.69355 4.24690 2.90347 2.73739 3.18146 2.89801 2.37887 2.77519 2.98518 4.58477 3.61503

0.00795 5.23378 5.95613 0.61958 0.77255 0.48576 0.95510

484 3.93255 5.15407 6.00029 5.47565 3.51725 5.42554 5.93924 2.02399 5.36747 2.18226 2.94995 5.58954 5.62217 5.47079 5.42372 4.81804 4.17360 0.54209 6.18883 5.06921 785 - -

2.68618 4.42225 2.77519 2.73123 3.46354 2.40513 3.72494 3.29354 2.67741 2.69355 4.24690 2.90347 2.73739 3.18146 2.89801 2.37887 2.77519 2.98518 4.58477 3.61503
0.00795 5.23378 5.95613 0.61958 0.77255 0.48576 0.95510
485 3.36579 4.70930 5.34009 4.73251 1.57013 4.57351 4.90159 1.79560 4.52920 1.68472 3.02049 4.73762 4.89001 4.61967 4.52631 3.89573 3.59450 1.72036 5.31425 3.28862 786 - -
2.68618 4.42225 2.77519 2.73123 3.46354 2.40513 3.72494 3.29354 2.67741 2.69355 4.24690 2.90347 2.73739 3.18146 2.89801 2.37887 2.77519 2.98518 4.58477 3.61503
0.00795 5.23378 5.95613 0.61958 0.77255 0.48576 0.95510
486 3.86124 6.61904 1.05107 1.06244 5.88129 3.61532 4.60980 5.46066 3.71755 4.89120 5.76017 3.44224 4.66883 2.32998 4.39285 3.69447 4.16289 4.97841 7.03237 5.43570 787 - -
2.68618 4.42225 2.77519 2.73123 3.46354 2.40513 3.72494 3.29354 2.67741 2.69355 4.24690 2.90347 2.73739 3.18146 2.89801 2.37887 2.77519 2.98518 4.58477 3.61503
0.00795 5.23378 5.95613 0.61958 0.77255 0.48576 0.95510
487 3.76352 5.31028 5.29323 4.90453 4.00820 4.70174 5.49408 3.22376 4.69506 0.96896 3.86947 5.01017 0.99965 4.93764 4.81800 4.15206 4.11472 3.07101 5.98300 4.84336 788 - -
2.68618 4.42225 2.77519 2.73123 3.46354 2.40513 3.72494 3.29354 2.67741 2.69355 4.24690 2.90347 2.73739 3.18146 2.89801 2.37887 2.77519 2.98518 4.58477 3.61503
0.00795 5.23378 5.95613 0.61958 0.77255 0.48576 0.95510
488 2.60406 3.70566 2.85206 2.33420 4.81437 3.54756 3.94403 4.29586 2.09346 3.77826 4.51813 2.44391 4.14953 2.91875 3.03523 1.50677 3.21149 3.22995 5.91224 4.50515 789 - -
2.68618 4.42225 2.77519 2.73123 3.46354 2.40513 3.72494 3.29354 2.67741 2.69355 4.24690 2.90347 2.73739 3.18146 2.89801 2.37887 2.77519 2.98518 4.58477 3.61503
0.00795 5.23378 5.95613 0.61958 0.77255 0.48576 0.95510
489 2.13597 5.41748 3.26088 1.70680 4.73263 3.76730 3.95757 4.19786 2.37209 2.87462 3.38132 2.36282 3.58610 3.06450 2.50450 2.95882 3.06909 3.09991 5.86842 4.47652 790 - -
2.68725 4.42203 2.77620 2.73160 3.46460 2.40578 3.72634 3.29260 2.67705 2.69442 4.24786 2.90311 2.73879 3.18196 2.89375 2.37999 2.77364 2.98587 4.56680 3.61468
0.49525 0.94677 5.95613 1.89146 0.16352 0.48576 0.95510
490 2.30284 2.70200 3.05590 1.63059 4.72808 2.94504 3.95822 4.19261 2.58149 3.05377 4.46325 3.24814 3.83966 2.95060 2.93417 2.24066 3.21247 3.40091 5.86607 4.47477 798 - -
2.68618 4.42225 2.77519 2.73123 3.46354 2.40513 3.72494 3.29354 2.67741 2.69355 4.24690 2.90347 2.73739 3.18146 2.89801 2.37887 2.77519 2.98518 4.58477 3.61503
0.00795 5.23378 5.95613 0.61958 0.77255 0.48576 0.95510
491 2.80598 4.63003 4.09262 3.22748 3.75117 4.04351 4.31472 2.55345 2.67051 2.07975 3.23639 3.86533 4.41905 3.38703 3.21921 3.30352 1.56797 2.02319 5.22551 4.01056 799 - -
2.68618 4.42225 2.77519 2.73123 3.46354 2.40513 3.72494 3.29354 2.67741 2.69355 4.24690 2.90347 2.73739 3.18146 2.89801 2.37887 2.77519 2.98518 4.58477 3.61503
0.00795 5.23378 5.95613 0.61958 0.77255 0.48576 0.95510
492 4.19020 5.42065 5.76741 5.37477 1.06652 5.19154 4.54336 2.52042 5.16992 2.56077 4.25319 5.06780 5.46901 5.07435 5.09467 4.54425 4.40817 3.28397 4.68299 1.07218 800 - -
2.68618 4.42225 2.77519 2.73123 3.46354 2.40513 3.72494 3.29354 2.67741 2.69355 4.24690 2.90347 2.73739 3.18146 2.89801 2.37887 2.77519 2.98518 4.58477 3.61503
0.00795 5.23378 5.95613 0.61958 0.77255 0.48576 0.95510
493 2.98040 5.48267 3.23238 1.84508 4.82456 3.75524 2.28752 3.57529 2.25436 3.43147 4.52425 2.42178 4.14858 2.31577 2.74198 2.76724 2.83992 3.87162 5.91707 3.64762 801 - -
2.68618 4.42225 2.77519 2.73123 3.46354 2.40513 3.72494 3.29354 2.67741 2.69355 4.24690 2.90347 2.73739 3.18146 2.89801 2.37887 2.77519 2.98518 4.58477 3.61503
0.00795 5.23378 5.95613 0.61958 0.77255 0.48576 0.95510
494 2.97985 5.46846 1.72704 2.45020 4.09926 3.75694 3.55682 4.28386 2.08916 2.81492 4.51156 2.87941 4.15025 2.94017 2.97230 2.56350 2.53754 3.85416 5.90667 3.03703 802 - -
2.68618 4.42225 2.77519 2.73123 3.46354 2.40513 3.72494 3.29354 2.67741 2.69355 4.24690 2.90347 2.73739 3.18146 2.89801 2.37887 2.77519 2.98518 4.58477 3.61503
0.00795 5.23378 5.95613 0.61958 0.77255 0.48576 0.95510
495 3.47008 4.81438 5.37307 4.77730 3.48970 4.65011 4.86096 2.57204 4.57597 1.41449 1.83154 4.78027 4.95423 4.64683 4.57398 3.97180 3.69517 2.95720 1.53680 2.74985 803 - -
2.68618 4.42225 2.77519 2.73123 3.46354 2.40513 3.72494 3.29354 2.67741 2.69355 4.24690 2.90347 2.73739 3.18146 2.89801 2.37887 2.77519 2.98518 4.58477 3.61503
0.00795 5.23378 5.95613 0.61958 0.77255 0.48576 0.95510

496 3.43349 4.82467 5.10322 4.51294 2.93348 4.58573 4.82784 2.99205 4.34949 1.33789 1.28280 4.63816 4.89663 2.29684 4.43780 3.89444 3.65873 2.97202 5.28287 3.30926 804 - -
2.68618 4.42225 2.77519 2.73123 3.46354 2.40513 3.72494 3.29354 2.67741 2.69355 4.24690 2.90347 2.73739 3.18146 2.89801 2.37887 2.77519 2.98518 4.58477 3.61503
0.20149 5.23378 1.73075 0.61958 0.77255 0.48576 0.95510
497 2.78851 5.40663 2.64305 1.92465 4.75190 3.66969 2.65805 4.23725 2.26894 3.34378 4.44751 3.13724 4.06350 2.49273 1.96468 2.56236 2.99627 3.79744 5.83861 4.42858 805 - -
2.68618 4.42225 2.77519 2.73123 3.46354 2.40513 3.72494 3.29354 2.67741 2.69355 4.24690 2.90347 2.73739 3.18146 2.89801 2.37887 2.77519 2.98518 4.58477 3.61503
0.00965 5.04194 5.76429 0.61958 0.77255 0.27081 1.43867
498 2.97950 5.47578 2.66895 1.84899 4.81484 3.75546 3.22481 3.01920 1.95728 3.10968 4.09205 2.29759 4.14881 2.71974 3.17189 2.94456 2.60237 3.47302 5.91206 4.50478 806 - -
2.68621 4.42228 2.77523 2.73127 3.46357 2.40516 3.72498 3.29357 2.67744 2.69358 4.24693 2.90306 2.73743 3.18150 2.89804 2.37878 2.77523 2.98522 4.58480 3.61506
0.07819 2.62252 5.95613 0.23359 1.56870 0.48576 0.95510
499 3.02739 4.49944 4.50719 3.91841 3.60174 4.13229 4.43526 2.35170 3.43493 1.40473 2.90375 4.11937 4.50128 2.53973 3.22044 2.81056 3.25930 1.85753 5.11122 3.92034 808 - -
2.68618 4.42225 2.77519 2.73123 3.46354 2.40513 3.72494 3.29354 2.67741 2.69355 4.24690 2.90347 2.73739 3.18146 2.89801 2.37887 2.77519 2.98518 4.58477 3.61503
0.00795 5.23378 5.95613 0.61958 0.77255 0.48576 0.95510
500 2.98791 5.49121 3.23455 2.34031 3.44602 3.18743 3.94659 4.31801 1.58560 3.79476 4.53285 1.95252 4.15402 2.37702 2.87163 2.46915 3.21877 3.88111 5.92380 4.51589 809 - -
2.68618 4.42225 2.77519 2.73123 3.46354 2.40513 3.72494 3.29354 2.67741 2.69355 4.24690 2.90347 2.73739 3.18146 2.89801 2.37887 2.77519 2.98518 4.58477 3.61503
0.00795 5.23378 5.95613 0.61958 0.77255 0.48576 0.95510
501 2.98581 5.36410 3.29064 1.74783 4.65874 3.78096 3.97335 3.05816 2.30563 3.64716 4.41867 3.27170 4.17299 2.80086 2.77432 2.28098 2.32245 2.24211 5.82787 4.45076 810 - -
2.68633 4.42240 2.77442 2.73138 3.46368 2.40498 3.72509 3.29369 2.67727 2.69370 4.24704 2.90281 2.73754 3.18161 2.89815 2.37901 2.77534 2.98533 4.58492 3.61518
0.34458 1.24170 5.95613 0.07835 2.58552 0.48576 0.95510
502 2.99340 5.05575 2.99513 2.77045 4.25828 2.90646 3.78526 2.32518 2.35674 2.10676 3.59289 3.43018 4.24608 3.25806 2.19704 3.07023 2.59890 2.76409 5.58451 4.28072 812 - -
2.68618 4.42225 2.77519 2.73123 3.46354 2.40513 3.72494 3.29354 2.67741 2.69355 4.24690 2.90347 2.73739 3.18146 2.89801 2.37887 2.77519 2.98518 4.58477 3.61503
0.00795 5.23378 5.95613 0.61958 0.77255 0.48576 0.95510
503 2.81851 5.45334 3.24462 2.07392 4.78296 3.40425 3.54197 4.25784 2.69003 3.75202 4.49844 3.23450 2.95376 2.85545 1.97137 2.20178 2.16246 2.87753 5.89560 4.49457 813 - -
2.68618 4.42225 2.77519 2.73123 3.46354 2.40513 3.72494 3.29354 2.67741 2.69355 4.24690 2.90347 2.73739 3.18146 2.89801 2.37887 2.77519 2.98518 4.58477 3.61503
0.46271 5.23378 1.00761 0.61958 0.77255 0.48576 0.95510
504 2.81245 4.47958 2.91761 3.20834 3.60636 3.81407 4.07642 2.07286 2.60369 1.55648 3.09657 3.58863 4.19167 3.44441 3.47760 2.71789 3.04434 2.73022 5.07093 3.84669 814 - -
2.68618 4.42225 2.77519 2.73123 3.46354 2.40513 3.72494 3.29354 2.67741 2.69355 4.24690 2.90347 2.73739 3.18146 2.89801 2.37887 2.77519 2.98518 4.58477 3.61503
0.01251 4.78358 5.50593 0.61958 0.77255 0.18383 1.78425
505 3.10820 5.04871 3.68358 3.15108 3.92850 3.97683 4.17618 3.64850 3.13144 3.29708 4.17229 2.52257 2.73914 3.46403 3.54353 1.56765 3.35030 3.38198 1.96081 2.50509 815 - -
2.68618 4.42225 2.77519 2.73123 3.46354 2.40513 3.72494 3.29354 2.67741 2.69355 4.24690 2.90347 2.73739 3.18146 2.89801 2.37887 2.77519 2.98518 4.58477 3.61503
0.00795 5.23378 5.95613 0.61958 0.77255 0.48576 0.95510
506 2.98792 5.48211 3.00001 2.12385 4.82021 3.76008 3.69064 4.30124 1.80823 3.78404 4.52500 3.23002 2.79959 2.70392 3.17804 2.85664 2.53948 3.86909 5.91836 1.96762 816 - -
2.68618 4.42225 2.77519 2.73123 3.46354 2.40513 3.72494 3.29354 2.67741 2.69355 4.24690 2.90347 2.73739 3.18146 2.89801 2.37887 2.77519 2.98518 4.58477 3.61503
0.00795 5.23378 5.95613 0.61958 0.77255 0.48576 0.95510
507 2.48727 5.21496 2.51573 2.82174 4.46088 3.81684 4.02489 3.88124 2.82205 1.71384 4.28696 2.14580 3.00596 3.17112 3.28886 2.90916 2.40244 3.55182 5.71628 3.77318 817 - -
2.68618 4.42225 2.77519 2.73123 3.46354 2.40513 3.72494 3.29354 2.67741 2.69355 4.24690 2.90347 2.73739 3.18146 2.89801 2.37887 2.77519 2.98518 4.58477 3.61503

0.00795 5.23378 5.95613 0.61958 0.77255 0.48576 0.95510
508 3.49729 5.13885 4.96829 4.64523 2.31459 0.51089 4.95317 2.99697 4.57826 3.30153 4.45320 4.64471 4.94967 4.77075 4.72698 3.77849 3.89477 3.48331 5.42251 3.89047 818 - -
2.68618 4.42225 2.77519 2.73123 3.46354 2.40513 3.72494 3.29354 2.67741 2.69355 4.24690 2.90347 2.73739 3.18146 2.89801 2.37887 2.77519 2.98518 4.58477 3.61503
0.00795 5.23378 5.95613 0.61958 0.77255 0.48576 0.95510
509 1.35205 4.96982 4.90111 4.60087 5.55277 1.27459 5.48466 5.02957 4.58027 4.69480 5.45532 4.42519 4.58211 4.79310 4.80784 1.75252 1.78010 4.25090 6.87421 5.75011 819 - -
2.68618 4.42225 2.77519 2.73123 3.46354 2.40513 3.72494 3.29354 2.67741 2.69355 4.24690 2.90347 2.73739 3.18146 2.89801 2.37887 2.77519 2.98518 4.58477 3.61503
0.00795 5.23378 5.95613 0.61958 0.77255 0.48576 0.95510
510 3.74070 6.20599 1.10491 2.07521 5.38527 4.02277 4.57174 4.84069 3.62498 1.18757 5.29522 3.48968 4.64553 3.75375 4.22297 3.64157 4.02766 4.50378 6.67020 5.19255 820 - -
2.68618 4.42225 2.77519 2.73123 3.46354 2.40513 3.72494 3.29354 2.67741 2.69355 4.24690 2.90347 2.73739 3.18146 2.89801 2.37887 2.77519 2.98518 4.58477 3.61503
0.00795 5.23378 5.95613 0.61958 0.77255 0.48576 0.95510
511 3.95335 5.97202 3.92949 3.79790 5.69541 4.36234 5.08105 5.37471 3.75830 4.82807 5.75791 4.24083 1.09651 0.76595 4.04112 4.00270 4.33419 4.90455 6.75756 5.54790 821 - -
2.68618 4.42225 2.77519 2.73123 3.46354 2.40513 3.72494 3.29354 2.67741 2.69355 4.24690 2.90347 2.73739 3.18146 2.89801 2.37887 2.77519 2.98518 4.58477 3.61503
0.02007 5.23378 4.23113 0.61958 0.77255 0.48576 0.95510
512 2.73358 5.23151 2.51358 3.03188 4.73039 1.39526 4.27603 3.62849 3.11022 3.75639 4.56436 3.51658 1.56766 3.43384 3.57305 3.12097 3.36888 3.32698 5.98791 4.65404 822 - -
2.68618 4.42225 2.77519 2.73123 3.46354 2.40513 3.72494 3.29354 2.67741 2.69355 4.24690 2.90347 2.73739 3.18146 2.89801 2.37887 2.77519 2.98518 4.58477 3.61503
0.00805 5.22177 5.94411 0.61958 0.77255 0.52744 0.89188
513 2.77288 4.62147 4.45792 3.91947 1.21931 1.99786 4.48675 3.15285 3.82736 2.88758 3.80800 4.12291 4.53186 4.05617 4.03595 2.97127 3.02476 2.94082 5.18611 2.39295 823 - -
2.68618 4.42225 2.77519 2.73123 3.46354 2.40513 3.72494 3.29354 2.67741 2.69355 4.24690 2.90347 2.73739 3.18146 2.89801 2.37887 2.77519 2.98518 4.58477 3.61503
0.00805 5.22177 5.94411 0.61958 0.77255 0.52744 0.89188
514 4.68501 5.85575 6.26762 5.83761 3.60175 5.73014 5.57623 3.33151 5.54384 0.24524 3.76560 5.86810 5.85425 5.48847 5.47259 5.29687 4.89265 3.66573 3.32199 4.18302 824 - -
2.68618 4.42225 2.77519 2.73123 3.46354 2.40513 3.72494 3.29354 2.67741 2.69355 4.24690 2.90347 2.73739 3.18146 2.89801 2.37887 2.77519 2.98518 4.58477 3.61503
0.00805 5.22177 5.94411 0.61958 0.77255 0.52744 0.89188
515 3.44839 4.81563 5.13750 4.57621 3.91097 4.68228 5.06397 1.68820 4.44125 1.54425 3.81959 1.42116 5.01146 4.64497 4.57920 4.00715 3.68862 1.89947 5.61643 4.43568 825 - -
2.68618 4.42225 2.77519 2.73123 3.46354 2.40513 3.72494 3.29354 2.67741 2.69355 4.24690 2.90347 2.73739 3.18146 2.89801 2.37887 2.77519 2.98518 4.58477 3.61503
0.00805 5.22177 5.94411 0.61958 0.77255 0.52744 0.89188
516 2.47194 4.91478 3.62277 1.98695 4.09151 3.90167 4.13487 3.14274 3.03899 3.14576 3.02536 3.53209 4.28865 3.36821 3.45493 1.92945 1.93760 2.39258 5.47339 4.20004 826 - -
2.68618 4.42225 2.77519 2.73123 3.46354 2.40513 3.72494 3.29354 2.67741 2.69355 4.24690 2.90347 2.73739 3.18146 2.89801 2.37887 2.77519 2.98518 4.58477 3.61503
0.00805 5.22177 5.94411 0.61958 0.77255 0.52744 0.89188
517 3.99997 5.26174 5.65247 5.22250 0.98953 5.04446 4.53465 2.18248 5.01907 2.99976 4.20183 4.97080 5.34421 4.97605 4.96781 4.39013 4.22382 2.67770 4.70322 1.33133 827 - -
2.68618 4.42225 2.77519 2.73123 3.46354 2.40513 3.72494 3.29354 2.67741 2.69355 4.24690 2.90347 2.73739 3.18146 2.89801 2.37887 2.77519 2.98518 4.58477 3.61503
0.00805 5.22177 5.94411 0.61958 0.77255 0.52744 0.89188
518 2.32998 4.64522 4.11727 3.54574 1.50218 3.73741 3.72901 3.12081 2.86031 2.84617 3.75467 3.88471 4.43744 3.22629 3.76487 3.19890 3.26850 2.91130 4.10869 1.89928 828 - -
2.68618 4.42225 2.77519 2.73123 3.46354 2.40513 3.72494 3.29354 2.67741 2.69355 4.24690 2.90347 2.73739 3.18146 2.89801 2.37887 2.77519 2.98518 4.58477 3.61503
0.00805 5.22177 5.94411 0.61958 0.77255 0.52744 0.89188
519 2.97630 5.45373 2.92812 2.60700 4.78542 1.93868 3.94364 4.26186 2.47064 3.75351 4.49855 2.82784 2.72979 3.04467 2.73604 2.15762 2.49515 2.75148 5.89507 4.49246 829 - -

2.68618 4.42225 2.77519 2.73123 3.46354 2.40513 3.72494 3.29354 2.67741 2.69355 4.24690 2.90347 2.73739 3.18146 2.89801 2.37887 2.77519 2.98518 4.58477 3.61503
0.00805 5.22177 5.94411 0.61958 0.77255 0.52744 0.89188
520 2.97922 5.44859 1.75983 2.62043 4.77738 3.75794 2.99229 4.25131 2.35875 2.55142 4.49382 2.87809 4.15135 2.52956 2.96919 2.94933 3.00792 3.83052 2.49545 4.49066 830 - -
2.68618 4.42225 2.77519 2.73123 3.46354 2.40513 3.72494 3.29354 2.67741 2.69355 4.24690 2.90347 2.73739 3.18146 2.89801 2.37887 2.77519 2.98518 4.58477 3.61503
0.00805 5.22177 5.94411 0.61958 0.77255 0.52744 0.89188
521 3.09192 4.46269 5.04138 4.42985 2.41478 4.26840 4.60071 2.11210 4.22110 2.02372 3.54014 4.42509 4.62502 4.35092 4.23476 3.58122 1.89927 1.80143 2.27669 3.91012 831 - -
2.68618 4.42225 2.77519 2.73123 3.46354 2.40513 3.72494 3.29354 2.67741 2.69355 4.24690 2.90347 2.73739 3.18146 2.89801 2.37887 2.77519 2.98518 4.58477 3.61503
0.00805 5.22177 5.94411 0.61958 0.77255 0.52744 0.89188
522 2.64367 5.26212 3.33492 2.37845 3.58336 3.79519 2.15238 3.95498 2.38152 2.48017 4.32581 3.30644 2.72490 2.54159 3.24481 2.99393 3.21228 3.60724 5.74763 2.52791 832 - -
2.68618 4.42225 2.77519 2.73123 3.46354 2.40513 3.72494 3.29354 2.67741 2.69355 4.24690 2.90347 2.73739 3.18146 2.89801 2.37887 2.77519 2.98518 4.58477 3.61503
0.00805 5.22177 5.94411 0.61958 0.77255 0.46056 0.99678
523 2.17280 5.48069 2.78287 2.67851 4.82149 3.75637 3.94436 3.92159 2.23702 3.78418 4.52312 2.19622 2.25145 3.04237 2.11348 2.63844 3.00725 3.86917 5.91633 4.50853 833 - -
2.68622 4.42229 2.77524 2.73127 3.46358 2.40517 3.72499 3.29358 2.67745 2.69359 4.24694 2.90351 2.73679 3.18150 2.89805 2.37891 2.77524 2.98522 4.58481 3.61507
0.09275 2.45357 5.95613 0.20805 1.67219 0.48576 0.95510
524 2.27317 4.57663 4.99179 4.39309 3.62338 4.36590 4.69873 1.51922 4.21932 1.37938 3.19081 4.46993 4.71371 2.67203 4.28914 3.67531 3.42313 2.21992 5.22131 4.05218 835 - -
2.68618 4.42225 2.77519 2.73123 3.46354 2.40513 3.72494 3.29354 2.67741 2.69355 4.24690 2.90347 2.73739 3.18146 2.89801 2.37887 2.77519 2.98518 4.58477 3.61503
0.23073 5.23378 1.60588 0.61958 0.77255 0.48576 0.95510
525 3.20451 5.77758 1.53052 2.03031 5.09925 3.74123 4.09745 4.60761 2.95067 4.07437 4.84483 2.60338 1.86105 2.94075 3.49035 2.26011 3.45705 4.15947 6.20850 4.74743 836 - -
2.68618 4.42225 2.77519 2.73123 3.46354 2.40513 3.72494 3.29354 2.67741 2.69355 4.24690 2.90347 2.73739 3.18146 2.89801 2.37887 2.77519 2.98518 4.58477 3.61503
0.00993 5.01298 5.73533 0.61958 0.77255 1.01979 0.44733
526 2.73755 5.38258 2.83663 2.59165 4.72236 3.66680 2.52488 4.20134 1.63729 3.29950 4.42568 3.13731 2.83735 2.95051 2.31232 2.42113 3.12322 3.77061 5.81671 4.41364 837 - -
2.68618 4.42225 2.77519 2.73123 3.46354 2.40513 3.72494 3.29354 2.67741 2.69355 4.24690 2.90347 2.73739 3.18146 2.89801 2.37887 2.77519 2.98518 4.58477 3.61503
0.00993 5.01298 5.73533 0.61958 0.77255 1.01979 0.44733
527 2.70409 5.24223 3.20922 2.65124 3.85638 3.69001 2.77775 3.97382 2.23193 3.52118 4.29949 3.18597 4.08140 2.87984 1.80301 2.09592 2.78408 2.82971 5.71063 4.34115 838 - -
2.68618 4.42225 2.77519 2.73123 3.46354 2.40513 3.72494 3.29354 2.67741 2.69355 4.24690 2.90347 2.73739 3.18146 2.89801 2.37887 2.77519 2.98518 4.58477 3.61503
0.00993 5.01298 5.73533 0.61958 0.77255 1.01979 0.44733
528 3.21581 4.58675 4.98191 4.41024 3.18711 4.34710 4.43264 2.92828 4.21767 2.34859 2.45497 4.41276 4.68988 4.33730 4.25971 3.66085 3.44514 2.19363 1.22229 2.27513 839 - -
2.68618 4.42225 2.77519 2.73123 3.46354 2.40513 3.72494 3.29354 2.67741 2.69355 4.24690 2.90347 2.73739 3.18146 2.89801 2.37887 2.77519 2.98518 4.58477 3.61503
0.00993 5.01298 5.73533 0.61958 0.77255 1.01979 0.44733
529 2.78170 5.56882 3.12010 2.94722 4.89966 3.83193 1.58120 4.60666 3.18430 4.13647 4.98000 0.99008 4.42279 3.54067 3.60693 3.31616 3.64094 4.17852 6.16158 4.72230 840 - -
2.68618 4.42225 2.77519 2.73123 3.46354 2.40513 3.72494 3.29354 2.67741 2.69355 4.24690 2.90347 2.73739 3.18146 2.89801 2.37887 2.77519 2.98518 4.58477 3.61503
0.00993 5.01298 5.73533 0.61958 0.77255 1.01979 0.44733
530 2.97377 4.36780 4.78764 4.18326 2.23109 4.13231 4.45788 2.42028 4.00207 1.73035 2.78232 4.24358 4.48976 2.43708 4.06235 3.43849 2.61823 1.78897 4.98629 3.81292 841 - -
2.68636 4.42243 2.77538 2.73141 3.46372 2.40531 3.72513 3.29352 2.67759 2.69229 4.24448 2.90365 2.73758 3.18164 2.89819 2.37905 2.77538 2.98456 4.58495 3.61521
1.66106 1.10978 0.73307 0.34297 1.23670 0.25563 1.48913

531 2.21622 5.28349 3.22384 2.65208 4.65545 3.66748 3.82326 4.09540 1.59459 3.59098 4.36736 3.16873 2.48580 2.93582 2.03210 2.72723 3.13708 3.69938 5.72311 4.38088 844 - -
2.68618 4.42225 2.77519 2.73123 3.46354 2.40513 3.72494 3.29354 2.67741 2.69355 4.24690 2.90347 2.73739 3.18146 2.89801 2.37887 2.77519 2.98518 4.58477 3.61503
0.01292 4.75145 5.47380 0.61958 0.77255 1.34578 0.30156
532 2.79919 4.29549 4.18881 3.60529 3.38224 3.88842 2.67072 2.74328 3.49178 1.90495 2.43869 3.03602 4.25847 3.72576 3.69444 3.17041 3.03109 1.83365 4.89005 2.81542 845 - -
2.68618 4.42225 2.77519 2.73123 3.46354 2.40513 3.72494 3.29354 2.67741 2.69355 4.24690 2.90347 2.73739 3.18146 2.89801 2.37887 2.77519 2.98518 4.58477 3.61503
0.01292 4.75145 5.47380 0.61958 0.77255 1.34578 0.30156
533 4.16976 5.36680 5.63923 5.26771 0.69806 5.19924 4.64591 3.01698 5.08500 1.44554 3.56607 5.11891 5.38078 4.96333 5.02684 4.62850 4.38950 3.21825 4.72625 3.11743 846 - -
2.68618 4.42225 2.77519 2.73123 3.46354 2.40513 3.72494 3.29354 2.67741 2.69355 4.24690 2.90347 2.73739 3.18146 2.89801 2.37887 2.77519 2.98518 4.58477 3.61503
0.01292 4.75145 5.47380 0.61958 0.77255 1.34578 0.30156
534 2.83998 5.13078 2.01000 2.59609 4.40504 3.19195 3.84399 3.85545 2.62503 3.42334 4.22074 3.12610 4.02505 2.98086 2.56607 2.41368 3.07910 3.49877 1.94965 4.26025 847 - -
2.68618 4.42225 2.77519 2.73123 3.46354 2.40513 3.72494 3.29354 2.67741 2.69355 4.24690 2.90347 2.73739 3.18146 2.89801 2.37887 2.77519 2.98518 4.58477 3.61503
0.01292 4.75145 5.47380 0.61958 0.77255 1.34578 0.30156
535 2.80263 5.20902 2.81745 2.12890 4.51591 3.57866 3.77913 3.10234 2.53103 3.49805 4.26449 3.06452 1.97405 2.89212 2.25039 2.46491 3.03484 3.57970 5.66730 4.28274 848 - -
2.68618 4.42225 2.77519 2.73123 3.46354 2.40513 3.72494 3.29354 2.67741 2.69355 4.24690 2.90347 2.73739 3.18146 2.89801 2.37887 2.77519 2.98518 4.58477 3.61503
0.01292 4.75145 5.47380 0.61958 0.77255 1.34578 0.30156
536 2.81575 5.28850 3.00766 2.34965 4.61869 2.57412 2.52530 4.09408 2.32133 3.58815 4.33893 2.64031 2.56215 2.88119 3.01360 2.07346 3.04911 3.67237 5.73225 4.32909 849 - -
2.68618 4.42225 2.77519 2.73123 3.46354 2.40513 3.72494 3.29354 2.67741 2.69355 4.24690 2.90347 2.73739 3.18146 2.89801 2.37887 2.77519 2.98518 4.58477 3.61503
0.01292 4.75145 5.47380 0.61958 0.77255 1.34578 0.30156
537 2.80336 5.09641 2.34944 2.59276 4.41312 3.58432 3.83746 3.84973 2.62487 3.41867 4.20950 2.76320 2.21691 2.96989 3.10217 2.26080 2.62744 2.32480 5.62656 4.26555 850 - -
2.68647 4.42403 2.77568 2.73015 3.46453 2.40608 3.72359 3.29429 2.67657 2.69344 4.24365 2.90353 2.73639 3.18324 2.89552 2.38038 2.77453 2.98434 4.58655 3.61681
1.39953 0.51233 1.86957 2.17447 0.12066 0.17772 1.81511
538 2.77449 5.03053 3.48542 2.91949 4.23687 3.42885 2.56313 3.63008 2.03056 1.42047 3.66404 3.41328 4.22542 3.22711 2.92427 2.76686 3.20782 3.34481 5.55329 4.25715 866 - -
2.68618 4.42225 2.77519 2.73123 3.46354 2.40513 3.72494 3.29354 2.67741 2.69355 4.24690 2.90347 2.73739 3.18146 2.89801 2.37887 2.77519 2.98518 4.58477 3.61503
0.21332 5.13661 1.68080 0.61958 0.77255 0.77085 0.62105
539 2.85012 4.81217 4.16461 4.00948 5.36391 0.76154 5.13605 4.86471 4.21306 4.50905 5.27855 4.04965 2.11090 4.42017 4.49520 1.79184 3.39332 4.08440 6.66102 5.49586 867 - -
2.68618 4.42225 2.77519 2.73123 3.46354 2.40513 3.72494 3.29354 2.67741 2.69355 4.24690 2.90347 2.73739 3.18146 2.89801 2.37887 2.77519 2.98518 4.58477 3.61503
0.01075 4.93404 5.65639 0.61958 0.77255 1.13946 0.38565
540 3.56822 5.06369 5.12450 4.70712 3.77071 4.55730 5.27668 2.89185 4.48566 0.54717 3.61144 4.82939 5.01725 4.72471 4.60133 3.99905 2.10308 2.94455 5.74492 4.63028 868 - -
2.68618 4.42225 2.77519 2.73123 3.46354 2.40513 3.72494 3.29354 2.67741 2.69355 4.24690 2.90347 2.73739 3.18146 2.89801 2.37887 2.77519 2.98518 4.58477 3.61503
0.01075 4.93404 5.65639 0.61958 0.77255 1.13946 0.38565
541 3.45479 5.22483 4.55253 4.38261 4.75629 0.46954 5.28135 4.19844 4.37489 1.86879 4.93525 4.51456 4.80282 4.71988 4.56904 3.67603 3.92571 3.92216 6.22405 5.01468 869 - -
2.68618 4.42225 2.77519 2.73123 3.46354 2.40513 3.72494 3.29354 2.67741 2.69355 4.24690 2.90347 2.73739 3.18146 2.89801 2.37887 2.77519 2.98518 4.58477 3.61503
0.01075 4.93404 5.65639 0.61958 0.77255 1.13946 0.38565
542 3.07525 4.97763 3.57729 3.11210 3.76848 2.27042 3.44491 3.59979 3.01602 3.23885 4.13109 3.55261 4.32430 3.41749 3.05289 2.86131 3.32336 3.33700 5.23268 1.11294 870 - -
2.68618 4.42225 2.77519 2.73123 3.46354 2.40513 3.72494 3.29354 2.67741 2.69355 4.24690 2.90347 2.73739 3.18146 2.89801 2.37887 2.77519 2.98518 4.58477 3.61503

0.01075 4.93404 5.65639 0.61958 0.77255 1.13946 0.38565
543 3.01650 5.55398 1.75067 2.28354 4.88794 3.66742 3.94061 4.38294 2.72776 3.85364 4.60666 1.80250 4.12072 1.96089 2.92624 2.95930 2.99173 3.94040 5.98370 4.55127 871 - -
2.68618 4.42225 2.77519 2.73123 3.46354 2.40513 3.72494 3.29354 2.67741 2.69355 4.24690 2.90347 2.73739 3.18146 2.89801 2.37887 2.77519 2.98518 4.58477 3.61503
0.03505 4.93404 3.60285 0.61958 0.77255 1.13946 0.38565
544 2.15298 5.12237 3.19684 2.50023 4.38386 3.65816 3.85853 3.81432 2.50888 3.39160 2.68545 2.26073 2.60007 2.99270 3.11129 2.25050 3.07626 3.46750 5.60978 3.48160 872 - -
2.68618 4.42225 2.77519 2.73123 3.46354 2.40513 3.72494 3.29354 2.67741 2.69355 4.24690 2.90347 2.73739 3.18146 2.89801 2.37887 2.77519 2.98518 4.58477 3.61503
0.01101 4.91000 5.63235 0.61958 0.77255 1.07450 0.41774
545 2.65155 5.00907 1.90594 2.72545 3.33561 2.71370 3.90321 3.24063 2.51496 3.10684 4.08355 3.01450 4.08514 3.06394 3.17494 2.42327 2.87183 2.66448 5.52078 3.45238 873 - -
2.68618 4.42225 2.77519 2.73123 3.46354 2.40513 3.72494 3.29354 2.67741 2.69355 4.24690 2.90347 2.73739 3.18146 2.89801 2.37887 2.77519 2.98518 4.58477 3.61503
0.01075 4.93404 5.65639 0.61958 0.77255 1.13946 0.38565
546 2.16401 4.50670 3.90313 2.99252 3.63242 3.88907 4.15606 2.25411 2.96517 2.37262 3.61476 3.69125 4.26529 3.55028 2.59464 3.14612 2.61648 1.71835 5.10158 3.88282 874 - -
2.68618 4.42225 2.77519 2.73123 3.46354 2.40513 3.72494 3.29354 2.67741 2.69355 4.24690 2.90347 2.73739 3.18146 2.89801 2.37887 2.77519 2.98518 4.58477 3.61503
0.01075 4.93404 5.65639 0.61958 0.77255 1.13946 0.38565
547 2.96634 5.46375 2.45329 2.57632 4.80636 1.86572 3.91449 4.29155 2.53867 3.77633 4.53021 2.23884 4.09660 3.02374 2.91615 1.76277 3.20719 3.85842 5.91525 4.49698 875 - -
2.68618 4.42225 2.77519 2.73123 3.46354 2.40513 3.72494 3.29354 2.67741 2.69355 4.24690 2.90347 2.73739 3.18146 2.89801 2.37887 2.77519 2.98518 4.58477 3.61503
0.01075 4.93404 5.65639 0.61958 0.77255 0.62496 0.76632
548 2.29942 4.78666 3.61379 3.05063 3.94709 3.85247 4.08691 3.13672 2.80652 2.01124 3.19143 2.97450 3.63762 2.83354 3.24018 2.52473 2.46124 2.88267 5.34866 2.53249 876 - -
2.68605 4.41809 2.77586 2.73155 3.46341 2.40579 3.72561 3.29420 2.67692 2.69179 4.24379 2.90278 2.73806 3.18185 2.89822 2.37933 2.77586 2.98584 4.58543 3.61277
0.64547 0.74959 5.80205 1.12673 0.39170 0.29442 1.36636
549 2.65980 5.38204 2.89089 2.07152 2.73822 3.51244 3.96622 4.14009 2.47118 3.66859 4.43439 3.26066 4.16633 2.81097 2.07617 2.79351 2.97162 3.44795 2.59213 3.37681 883 - -
2.68618 4.42225 2.77519 2.73123 3.46354 2.40513 3.72494 3.29354 2.67741 2.69355 4.24690 2.90347 2.73739 3.18146 2.89801 2.37887 2.77519 2.98518 4.58477 3.61503
0.05682 5.23378 2.99760 0.61958 0.77255 0.48576 0.95510
550 2.34292 4.58942 2.17314 2.43333 4.76363 2.78762 3.11990 3.69470 2.46372 3.73211 2.78839 2.26874 4.12962 3.02623 2.94174 2.66036 3.05704 3.48848 5.87441 4.47232 884 - -
2.68618 4.42225 2.77519 2.73123 3.46354 2.40513 3.72494 3.29354 2.67741 2.69355 4.24690 2.90347 2.73739 3.18146 2.89801 2.37887 2.77519 2.98518 4.58477 3.61503
0.00835 5.18531 5.90766 0.61958 0.77255 0.64154 0.74756
551 2.01197 4.51184 3.64515 4.15069 3.63900 4.24358 4.57623 1.39950 4.00909 1.91597 3.63737 3.64410 4.60688 4.20509 4.13773 3.54511 3.32654 1.78224 5.18117 3.99347 885 - -
2.68618 4.42225 2.77519 2.73123 3.46354 2.40513 3.72494 3.29354 2.67741 2.69355 4.24690 2.90347 2.73739 3.18146 2.89801 2.37887 2.77519 2.98518 4.58477 3.61503
0.00835 5.18531 5.90766 0.61958 0.77255 0.64154 0.74756
552 2.97477 5.48334 2.02173 1.56210 4.82718 3.73946 2.59766 4.31228 2.22868 3.04890 4.52517 3.20686 3.23678 3.02939 2.79860 2.93625 3.20629 3.87350 5.91539 4.50492 886 - -
2.68618 4.42225 2.77519 2.73123 3.46354 2.40513 3.72494 3.29354 2.67741 2.69355 4.24690 2.90347 2.73739 3.18146 2.89801 2.37887 2.77519 2.98518 4.58477 3.61503
0.00835 5.18531 5.90766 0.61958 0.77255 0.64154 0.74756
553 2.95926 3.59902 2.40599 2.66653 4.76124 3.73828 3.92718 4.23617 2.34464 2.67410 4.47665 2.36094 1.95906 2.70735 2.80053 2.54937 3.19042 3.81401 5.87368 4.47260 887 - -
2.68618 4.42225 2.77519 2.73123 3.46354 2.40513 3.72494 3.29354 2.67741 2.69355 4.24690 2.90347 2.73739 3.18146 2.89801 2.37887 2.77519 2.98518 4.58477 3.61503
0.00835 5.18531 5.90766 0.61958 0.77255 0.64154 0.74756
554 1.73800 5.54128 2.70895 2.22013 4.89567 2.07797 4.01743 4.37988 2.78658 3.86646 4.61659 3.08814 2.54683 3.12443 3.28769 2.16167 3.29536 3.94490 6.00578 4.59100 888 - -

2.68618 4.42225 2.77519 2.73123 3.46354 2.40513 3.72494 3.29354 2.67741 2.69355 4.24690 2.90347 2.73739 3.18146 2.89801 2.37887 2.77519 2.98518 4.58477 3.61503
0.34708 5.18531 1.24599 0.61958 0.77255 0.64154 0.74756
555 2.82712 5.23720 3.11276 2.47114 4.54513 3.61377 3.80356 3.26827 1.89783 3.52515 4.28897 2.60731 2.22089 2.29365 3.02698 2.68161 3.05783 2.96303 5.69175 4.30800 889 - -
2.68618 4.42225 2.77519 2.73123 3.46354 2.40513 3.72494 3.29354 2.67741 2.69355 4.24690 2.90347 2.73739 3.18146 2.89801 2.37887 2.77519 2.98518 4.58477 3.61503
0.01170 4.84993 5.57227 0.61958 0.77255 1.24451 0.33979
556 2.88930 4.46421 4.79871 4.22307 3.60381 4.28511 4.65343 2.07658 4.07657 1.42744 3.55567 2.99847 4.63371 4.27465 4.19823 3.60138 3.32014 1.29591 5.22253 4.03709 890 - -
2.68618 4.42225 2.77519 2.73123 3.46354 2.40513 3.72494 3.29354 2.67741 2.69355 4.24690 2.90347 2.73739 3.18146 2.89801 2.37887 2.77519 2.98518 4.58477 3.61503
0.01170 4.84993 5.57227 0.61958 0.77255 0.29989 1.35054
557 1.80032 5.39959 3.23597 2.37773 4.71380 3.42385 2.65522 4.17830 2.33213 3.69163 4.44959 3.09128 4.14375 3.05009 3.17621 1.88688 3.19851 3.77384 5.85138 2.67843 891 - -
2.68618 4.42225 2.77519 2.73123 3.46354 2.40513 3.72494 3.29354 2.67741 2.69355 4.24690 2.90347 2.73739 3.18146 2.89801 2.37887 2.77519 2.98518 4.58477 3.61503
0.00835 5.18531 5.90766 0.61958 0.77255 0.64154 0.74756
558 2.80122 4.70948 5.23258 4.67034 3.92385 3.12525 5.05023 2.28140 4.51662 1.99391 3.85677 4.74410 4.95928 4.70247 4.60675 3.93849 3.60726 0.73929 5.57424 4.38566 892 - -
2.68618 4.42225 2.77519 2.73123 3.46354 2.40513 3.72494 3.29354 2.67741 2.69355 4.24690 2.90347 2.73739 3.18146 2.89801 2.37887 2.77519 2.98518 4.58477 3.61503
0.00835 5.18531 5.90766 0.61958 0.77255 0.64154 0.74756
559 4.06339 5.23661 6.18729 5.70873 4.26509 5.70788 6.37295 1.07959 5.65332 1.23950 3.98188 5.88011 5.83635 5.76719 5.74564 5.15205 4.31077 1.33078 6.48994 5.38558 893 - -
2.68618 4.42225 2.77519 2.73123 3.46354 2.40513 3.72494 3.29354 2.67741 2.69355 4.24690 2.90347 2.73739 3.18146 2.89801 2.37887 2.77519 2.98518 4.58477 3.61503
0.00835 5.18531 5.90766 0.61958 0.77255 0.64154 0.74756
560 3.70651 5.60793 4.15544 4.02711 4.69503 4.23772 0.33209 4.98433 3.85864 4.51369 5.50329 4.32676 4.93506 4.42276 4.08344 3.82961 2.79819 4.54494 6.13278 4.64525 894 - -
2.68618 4.42225 2.77519 2.73123 3.46354 2.40513 3.72494 3.29354 2.67741 2.69355 4.24690 2.90347 2.73739 3.18146 2.89801 2.37887 2.77519 2.98518 4.58477 3.61503
0.00835 5.18531 5.90766 0.61958 0.77255 0.64154 0.74756
561 5.45078 6.23407 6.12305 6.16781 1.67166 5.77729 4.28037 4.97215 5.92333 4.03236 5.45670 5.24412 6.00951 5.45234 5.63902 5.19843 5.62477 4.94270 2.13453 0.50610 895 - -
2.68633 4.41839 2.77535 2.73138 3.46369 2.40500 3.72509 3.29369 2.67756 2.69370 4.24705 2.90362 2.73716 3.18161 2.89816 2.37902 2.77448 2.98533 4.58492 3.61518
0.30627 1.34289 5.90766 0.23636 1.55827 0.64154 0.74756
562 2.14765 5.23884 2.87970 2.82159 4.52762 3.79673 4.05185 3.95321 2.85571 1.75166 4.34974 1.90402 3.79588 3.19705 3.32656 2.12518 3.24975 3.60970 5.77440 4.42820 898 - -
2.68618 4.42225 2.77519 2.73123 3.46354 2.40513 3.72494 3.29354 2.67741 2.69355 4.24690 2.90347 2.73739 3.18146 2.89801 2.37887 2.77519 2.98518 4.58477 3.61503
0.00835 5.18531 5.90766 0.61958 0.77255 0.64154 0.74756
563 2.50309 5.04183 3.90220 3.55359 4.86165 0.63214 4.70534 4.26996 3.32784 3.93034 4.76285 3.29287 3.78040 3.93730 4.00725 3.21083 3.47798 2.83731 6.19360 4.94010 899 - -
2.68618 4.42225 2.77519 2.73123 3.46354 2.40513 3.72494 3.29354 2.67741 2.69355 4.24690 2.90347 2.73739 3.18146 2.89801 2.37887 2.77519 2.98518 4.58477 3.61503
0.00835 5.18531 5.90766 0.61958 0.77255 0.64154 0.74756
564 2.96410 5.18734 3.35119 2.79343 4.43169 2.67873 3.60221 3.46120 2.27545 2.86876 3.33498 2.20475 2.55740 2.87408 3.03098 2.74371 3.19540 3.26630 3.07920 3.49754 900 - -
2.68618 4.42225 2.77519 2.73123 3.46354 2.40513 3.72494 3.29354 2.67741 2.69355 4.24690 2.90347 2.73739 3.18146 2.89801 2.37887 2.77519 2.98518 4.58477 3.61503
0.15838 5.18531 1.95990 0.61958 0.77255 0.64154 0.74756
565 2.38903 4.54768 3.95798 3.38821 3.67459 2.46450 4.20771 3.02948 3.32524 1.74702 2.20419 3.12074 4.31175 3.60715 3.63412 2.32602 3.14891 2.81615 5.14461 3.40312 901 - -
2.68618 4.42225 2.77519 2.73123 3.46354 2.40513 3.72494 3.29354 2.67741 2.69355 4.24690 2.90347 2.73739 3.18146 2.89801 2.37887 2.77519 2.98518 4.58477 3.61503
0.00970 5.03663 5.75898 0.61958 0.77255 0.41238 1.08492

566 4.86097 6.47778 5.12299 4.65788 6.21781 4.97651 5.30060 5.84287 0.12569 5.15790 6.17512 4.96921 5.48903 4.51596 3.67503 4.90572 5.07428 5.51965 6.80614 5.93957 902 - -
2.68618 4.42225 2.77519 2.73123 3.46354 2.40513 3.72494 3.29354 2.67741 2.69355 4.24690 2.90347 2.73739 3.18146 2.89801 2.37887 2.77519 2.98518 4.58477 3.61503
0.00835 5.18531 5.90766 0.61958 0.77255 0.64154 0.74756
567 5.10765 6.46575 5.78612 5.83498 6.54574 5.09509 6.63739 6.62307 6.02366 5.97232 7.15228 6.00848 0.05010 6.34212 5.98889 5.34123 5.62565 6.09681 7.21598 6.71980 903 - -
2.68618 4.42225 2.77519 2.73123 3.46354 2.40513 3.72494 3.29354 2.67741 2.69355 4.24690 2.90347 2.73739 3.18146 2.89801 2.37887 2.77519 2.98518 4.58477 3.61503
0.00835 5.18531 5.90766 0.61958 0.77255 0.64154 0.74756
568 5.91720 6.71886 6.23790 6.26686 4.90518 5.43093 6.11837 6.30012 6.19088 5.42436 6.81578 6.34523 6.03937 6.47947 6.05523 6.14671 6.29246 6.16723 0.05432 4.87524 904 - -
2.68618 4.42225 2.77519 2.73123 3.46354 2.40513 3.72494 3.29354 2.67741 2.69355 4.24690 2.90347 2.73739 3.18146 2.89801 2.37887 2.77519 2.98518 4.58477 3.61503
0.00835 5.18531 5.90766 0.61958 0.77255 0.64154 0.74756
569 2.86441 4.65471 2.59040 3.38781 3.57421 3.99048 4.25239 3.03500 3.17735 1.29141 3.76023 3.76682 4.36800 3.62301 2.51233 3.24242 3.00486 2.35366 5.24357 3.13491 905 - -
2.68618 4.42225 2.77519 2.73123 3.46354 2.40513 3.72494 3.29354 2.67741 2.69355 4.24690 2.90347 2.73739 3.18146 2.89801 2.37887 2.77519 2.98518 4.58477 3.61503
0.00835 5.18531 5.90766 0.61958 0.77255 0.64154 0.74756
570 2.96526 2.42323 2.50309 2.05048 3.21874 3.38195 3.99777 3.83363 2.66131 3.43420 4.24478 3.32313 4.18455 3.14598 2.36623 2.99709 3.19656 3.50799 2.54294 3.46293 906 - -
2.68618 4.42225 2.77519 2.73123 3.46354 2.40513 3.72494 3.29354 2.67741 2.69355 4.24690 2.90347 2.73739 3.18146 2.89801 2.37887 2.77519 2.98518 4.58477 3.61503
0.00835 5.18531 5.90766 0.61958 0.77255 0.64154 0.74756
571 3.01200 4.39517 3.93861 4.30580 2.97892 4.17487 4.50117 1.81753 4.10356 1.70174 3.21651 4.31721 4.53838 4.24469 4.13199 3.48381 2.52644 2.64048 2.37364 2.33035 907 - -
2.68618 4.42225 2.77519 2.73123 3.46354 2.40513 3.72494 3.29354 2.67741 2.69355 4.24690 2.90347 2.73739 3.18146 2.89801 2.37887 2.77519 2.98518 4.58477 3.61503
0.00835 5.18531 5.90766 0.61958 0.77255 0.64154 0.74756
572 2.28942 5.45869 2.34448 2.52950 4.80115 2.01408 3.94446 4.28038 2.68828 3.76837 4.51271 3.21822 4.14386 3.04585 2.08354 2.44206 2.38695 3.85067 5.90699 4.50149 908 - -
2.68618 4.42225 2.77519 2.73123 3.46354 2.40513 3.72494 3.29354 2.67741 2.69355 4.24690 2.90347 2.73739 3.18146 2.89801 2.37887 2.77519 2.98518 4.58477 3.61503
0.00835 5.18531 5.90766 0.61958 0.77255 0.64154 0.74756
573 2.16276 5.13669 2.04063 2.36512 3.05492 3.80399 4.00997 2.58363 2.60422 3.39129 3.11899 3.07641 4.19333 3.02950 3.27908 2.58607 3.07997 3.46377 3.60414 4.31697 909 - -
2.68618 4.42225 2.77519 2.73123 3.46354 2.40513 3.72494 3.29354 2.67741 2.69355 4.24690 2.90347 2.73739 3.18146 2.89801 2.37887 2.77519 2.98518 4.58477 3.61503
0.05971 5.18531 2.94954 0.61958 0.77255 0.64154 0.74756
574 2.73998 5.41203 2.97435 1.91247 4.74318 3.14325 3.90232 4.21947 2.39516 3.71134 4.45641 2.81624 2.79182 3.00332 2.81250 2.60076 3.16612 3.79518 2.91404 2.36157 910 - -
2.68650 4.41554 2.77429 2.73137 3.46331 2.40555 3.72472 3.29403 2.67790 2.69404 4.24402 2.90181 2.73777 3.18195 2.89850 2.37936 2.77569 2.98567 4.58093 3.61478
0.34928 1.23119 5.85674 1.24086 0.34127 0.53418 0.88226
575 2.96070 5.47136 2.14581 2.38138 4.81684 3.73275 3.16925 4.30265 2.03511 2.55213 4.51215 2.28949 3.24203 2.70210 2.40595 2.92273 3.19180 3.86227 5.90333 4.49273 915 - -
2.68618 4.42225 2.77519 2.73123 3.46354 2.40513 3.72494 3.29354 2.67741 2.69355 4.24690 2.90347 2.73739 3.18146 2.89801 2.37887 2.77519 2.98518 4.58477 3.61503
0.00835 5.18531 5.90766 0.61958 0.77255 0.64154 0.74756
576 3.00108 4.44938 4.57524 3.98291 3.23322 4.12089 4.43073 2.63140 3.84374 2.21799 3.56504 4.14493 2.60536 4.04592 2.64911 2.86105 3.23317 1.58221 5.06259 2.07829 916 - -
2.68618 4.42225 2.77519 2.73123 3.46354 2.40513 3.72494 3.29354 2.67741 2.69355 4.24690 2.90347 2.73739 3.18146 2.89801 2.37887 2.77519 2.98518 4.58477 3.61503
0.00835 5.18531 5.90766 0.61958 0.77255 0.64154 0.74756
577 2.96466 2.83929 2.33619 2.16349 4.79875 3.73892 3.92496 4.27933 1.64780 3.07212 4.50130 3.20882 4.13235 2.86000 2.25220 2.69342 3.19540 3.84678 5.89337 4.48808 917 - -
2.68618 4.42225 2.77519 2.73123 3.46354 2.40513 3.72494 3.29354 2.67741 2.69355 4.24690 2.90347 2.73739 3.18146 2.89801 2.37887 2.77519 2.98518 4.58477 3.61503

0.34708 5.18531 1.24599 0.61958 0.77255 0.64154 0.74756

578 2.92506 5.26209 2.20281 2.12761 4.54271 3.64552 3.90935 2.54248 2.71653 3.54296 4.33915 3.14503 1.69563 3.04310 3.20631 2.91530 2.87907 3.60943 5.75003 4.37256 918 - -

2.68499 4.42352 2.77525 2.73143 3.46173 2.40612 3.72262 3.29273 2.67797 2.69277 4.24480 2.90473 2.73866 3.18181 2.89833 2.37916 2.77610 2.98244 4.58604 3.61515

1.49800 0.25797 5.57227 1.50935 0.24981 1.24451 0.33979

579 2.64182 5.27072 1.75099 2.27714 3.29782 3.60398 3.79615 4.05743 1.98281 3.56442 4.31889 3.08205 3.99888 2.90209 2.61939 2.79914 3.05596 3.04101 5.71866 4.32420 929 - -

2.68618 4.42225 2.77519 2.73123 3.46354 2.40513 3.72494 3.29354 2.67741 2.69355 4.24690 2.90347 2.73739 3.18146 2.89801 2.37887 2.77519 2.98518 4.58477 3.61503

0.01170 4.84993 5.57227 0.61958 0.77255 0.29989 1.35054

580 2.13038 5.44706 3.21609 2.66019 4.78351 3.39738 3.32309 4.26285 1.89781 3.74847 4.49034 2.90320 2.45233 3.02254 2.40882 2.28767 2.69464 3.83313 5.88494 4.06267 930 - -

2.68618 4.42225 2.77519 2.73123 3.46354 2.40513 3.72494 3.29354 2.67741 2.69355 4.24690 2.90347 2.73739 3.18146 2.89801 2.37887 2.77519 2.98518 4.58477 3.61503

0.00835 5.18531 5.90766 0.61958 0.77255 0.64154 0.74756

581 4.83978 5.88236 5.96647 5.80775 2.23285 5.52249 4.33107 4.37341 5.58060 1.52913 4.90989 5.15580 5.77930 5.28726 5.39974 4.90919 5.04056 4.34118 0.95090 1.67560 931 - -

2.68618 4.42225 2.77519 2.73123 3.46354 2.40513 3.72494 3.29354 2.67741 2.69355 4.24690 2.90347 2.73739 3.18146 2.89801 2.37887 2.77519 2.98518 4.58477 3.61503

0.00835 5.18531 5.90766 0.61958 0.77255 0.64154 0.74756

582 5.10579 6.23781 5.62371 5.45039 3.14052 5.37354 1.81745 5.10920 4.95058 4.27673 5.60384 5.23063 5.79623 5.26238 4.98615 5.07523 5.35422 5.00732 0.41821 3.06282 932 - -

2.68618 4.42225 2.77519 2.73123 3.46354 2.40513 3.72494 3.29354 2.67741 2.69355 4.24690 2.90347 2.73739 3.18146 2.89801 2.37887 2.77519 2.98518 4.58477 3.61503

0.00835 5.18531 5.90766 0.61958 0.77255 0.64154 0.74756

583 2.36501 5.45772 2.52799 2.14071 4.79873 3.73194 3.91943 4.28185 2.17989 3.76127 3.44366 2.83056 4.12533 3.01707 2.60268 2.08229 2.35201 3.58109 5.89283 3.83036 933 - -

2.68618 4.42225 2.77519 2.73123 3.46354 2.40513 3.72494 3.29354 2.67741 2.69355 4.24690 2.90347 2.73739 3.18146 2.89801 2.37887 2.77519 2.98518 4.58477 3.61503

0.00835 5.18531 5.90766 0.61958 0.77255 0.64154 0.74756

584 3.04012 5.01001 3.62035 3.04421 4.20396 3.91735 4.10994 1.90692 1.93780 3.23983 4.09802 3.51782 2.60668 2.78331 2.59562 3.14589 3.26644 2.02107 5.53992 4.26622 934 - -

2.68618 4.42225 2.77519 2.73123 3.46354 2.40513 3.72494 3.29354 2.67741 2.69355 4.24690 2.90347 2.73739 3.18146 2.89801 2.37887 2.77519 2.98518 4.58477 3.61503

0.00835 5.18531 5.90766 0.61958 0.77255 0.64154 0.74756

585 4.20336 5.88371 4.74923 4.15750 3.23659 4.82722 1.60409 4.59292 3.31814 3.93735 5.03474 4.37569 5.16585 4.05446 1.84946 4.20483 4.37895 4.39573 4.83964 0.92488 935 - -

2.68618 4.42225 2.77519 2.73123 3.46354 2.40513 3.72494 3.29354 2.67741 2.69355 4.24690 2.90347 2.73739 3.18146 2.89801 2.37887 2.77519 2.98518 4.58477 3.61503

0.00835 5.18531 5.90766 0.61958 0.77255 0.64154 0.74756

586 2.97129 5.19464 1.71490 2.55546 4.43959 3.79424 3.99892 3.44611 2.79352 2.87989 4.26399 2.80707 4.18611 3.14432 2.77882 2.99901 3.00507 1.75300 5.69339 4.35268 936 - -

2.68618 4.42225 2.77519 2.73123 3.46354 2.40513 3.72494 3.29354 2.67741 2.69355 4.24690 2.90347 2.73739 3.18146 2.89801 2.37887 2.77519 2.98518 4.58477 3.61503

0.05971 5.18531 2.94954 0.61958 0.77255 0.64154 0.74756

587 2.38928 5.44085 2.00016 2.48613 4.77891 3.71394 3.90675 4.26067 1.84047 2.53207 4.48405 2.59867 3.34254 3.00599 3.13926 2.42334 3.17645 3.82786 5.87751 4.46952 937 - -

2.68618 4.42225 2.77519 2.73123 3.46354 2.40513 3.72494 3.29354 2.67741 2.69355 4.24690 2.90347 2.73739 3.18146 2.89801 2.37887 2.77519 2.98518 4.58477 3.61503

0.00879 5.13439 5.85674 0.61958 0.77255 0.53418 0.88226

588 2.57630 4.61490 4.03933 2.75645 2.97072 4.01052 3.89111 3.09204 3.40308 2.04150 2.16645 3.44535 4.38661 3.68337 3.20467 2.63347 3.06991 2.88001 5.20881 2.03944 938 - -

2.68618 4.42225 2.77519 2.73123 3.46354 2.40513 3.72494 3.29354 2.67741 2.69355 4.24690 2.90347 2.73739 3.18146 2.89801 2.37887 2.77519 2.98518 4.58477 3.61503

0.05971 5.18531 2.94954 0.61958 0.77255 0.64154 0.74756

589 2.94291 5.45511 2.28745 1.77288 4.79988 3.52823 3.24183 4.28635 2.43080 3.76019 4.49627 3.17762 2.71955 2.88281 2.81798 1.90131 2.98343 3.84567 5.88751 4.47553 939 - -

2.68618 4.42225 2.77519 2.73123 3.46354 2.40513 3.72494 3.29354 2.67741 2.69355 4.24690 2.90347 2.73739 3.18146 2.89801 2.37887 2.77519 2.98518 4.58477 3.61503
0.00879 5.13439 5.85674 0.61958 0.77255 0.77623 0.61644
590 2.93456 5.43222 2.35118 1.86476 4.77176 3.70978 2.37548 4.25390 2.49936 2.53686 3.88663 2.71681 4.10341 2.64031 3.12669 2.49682 3.03128 3.81990 5.86817 4.06127 940 - -
2.68618 4.42225 2.77519 2.73123 3.46354 2.40513 3.72494 3.29354 2.67741 2.69355 4.24690 2.90347 2.73739 3.18146 2.89801 2.37887 2.77519 2.98518 4.58477 3.61503
0.00879 5.13439 5.85674 0.61958 0.77255 0.77623 0.61644
591 2.62876 5.44050 2.29997 2.18938 4.78405 3.70773 3.89496 3.70292 2.37383 3.74513 4.48156 2.83093 2.62926 2.65035 2.59163 2.57677 2.28286 3.45796 5.87409 4.11330 941 - -
2.68618 4.42225 2.77519 2.73123 3.46354 2.40513 3.72494 3.29354 2.67741 2.69355 4.24690 2.90347 2.73739 3.18146 2.89801 2.37887 2.77519 2.98518 4.58477 3.61503
0.00879 5.13439 5.85674 0.61958 0.77255 0.77623 0.61644
592 3.02066 4.43530 4.72353 4.12959 1.86647 4.15408 3.33737 2.66765 3.96237 1.44703 3.55193 4.22067 4.02909 4.13640 3.44832 3.28394 3.25245 2.68764 4.96273 2.08199 942 - -
2.68618 4.42225 2.77519 2.73123 3.46354 2.40513 3.72494 3.29354 2.67741 2.69355 4.24690 2.90347 2.73739 3.18146 2.89801 2.37887 2.77519 2.98518 4.58477 3.61503
0.00898 5.11355 5.83590 0.61958 0.77255 0.77623 0.61644
593 2.81912 5.17753 3.13205 2.58684 4.42893 3.75191 2.72892 2.92303 1.98179 2.25012 3.75596 3.27117 2.67361 2.64145 3.09223 2.95221 3.16176 2.81531 5.67068 4.32475 943 - -
2.68618 4.42225 2.77519 2.73123 3.46354 2.40513 3.72494 3.29354 2.67741 2.69355 4.24690 2.90347 2.73739 3.18146 2.89801 2.37887 2.77519 2.98518 4.58477 3.61503
0.00908 5.10246 5.82481 0.61958 0.77255 0.77623 0.61644
594 2.92086 5.39335 3.18486 2.62876 4.02755 2.28148 3.88884 3.87325 1.98299 3.27406 4.43817 2.74892 3.73277 2.13171 2.31984 2.50059 3.02032 3.77568 5.83523 4.01950 944 - -
2.68618 4.42225 2.77519 2.73123 3.46354 2.40513 3.72494 3.29354 2.67741 2.69355 4.24690 2.90347 2.73739 3.18146 2.89801 2.37887 2.77519 2.98518 4.58477 3.61503
0.13590 5.10246 2.11206 0.61958 0.77255 0.77623 0.61644
595 2.54325 5.19270 2.75946 2.50365 2.31537 3.67480 3.87110 3.59994 2.33697 3.11981 3.72320 2.88349 3.76404 2.67635 2.53595 2.61359 2.90748 3.36722 5.66957 3.20436 945 - -
2.68618 4.42225 2.77519 2.73123 3.46354 2.40513 3.72494 3.29354 2.67741 2.69355 4.24690 2.90347 2.73739 3.18146 2.89801 2.37887 2.77519 2.98518 4.58477 3.61503
0.01030 4.97686 5.69920 0.61958 0.77255 1.01979 0.44733
596 2.89062 1.33176 3.31705 2.71769 3.95709 3.79706 4.03411 3.16698 2.73091 3.01493 3.88303 3.23363 4.18611 3.10578 3.35243 2.75073 3.12355 2.55155 5.34530 4.07740 946 - -
2.68618 4.42225 2.77519 2.73123 3.46354 2.40513 3.72494 3.29354 2.67741 2.69355 4.24690 2.90347 2.73739 3.18146 2.89801 2.37887 2.77519 2.98518 4.58477 3.61503
0.01046 4.96089 5.68323 0.61958 0.77255 1.01979 0.44733
597 2.85968 5.35153 2.68604 2.28913 4.68873 2.93517 3.07414 3.86207 2.42318 3.11345 4.39426 2.02580 3.46277 2.92315 2.56276 2.62379 2.96730 3.49286 5.78894 3.17183 947 - -
2.68618 4.42225 2.77519 2.73123 3.46354 2.40513 3.72494 3.29354 2.67741 2.69355 4.24690 2.90347 2.73739 3.18146 2.89801 2.37887 2.77519 2.98518 4.58477 3.61503
0.01100 4.91133 5.63368 0.61958 0.77255 1.01979 0.44733
598 2.60900 4.29280 4.51792 3.92155 2.66959 3.68160 3.84106 1.89896 3.76875 1.47919 3.40947 3.41004 4.36127 3.40722 3.88470 3.29087 2.82972 2.25246 4.90909 3.72458 948 - -
2.68618 4.42225 2.77519 2.73123 3.46354 2.40513 3.72494 3.29354 2.67741 2.69355 4.24690 2.90347 2.73739 3.18146 2.89801 2.37887 2.77519 2.98518 4.58477 3.61503
0.02566 4.91133 4.01924 0.61958 0.77255 1.01979 0.44733
599 2.83456 4.43411 2.83510 2.34104 3.92685 3.05061 2.90366 4.07807 2.55187 3.16326 3.69084 2.18340 4.01051 2.65139 2.29025 2.62880 2.69304 3.44943 5.73418 4.33797 949 - -
2.68618 4.42225 2.77519 2.73123 3.46354 2.40513 3.72494 3.29354 2.67741 2.69355 4.24690 2.90347 2.73739 3.18146 2.89801 2.37887 2.77519 2.98518 4.58477 3.61503
0.01144 4.87188 5.59422 0.61958 0.77255 1.04133 0.43538
600 2.45755 5.31517 2.75174 1.99554 4.64854 3.37743 3.79637 4.12680 2.14725 2.53072 4.35953 2.84414 3.08053 2.89703 3.02912 2.09579 3.06289 3.69963 5.75491 4.35022 950 - -
2.68618 4.42225 2.77519 2.73123 3.46354 2.40513 3.72494 3.29354 2.67741 2.69355 4.24690 2.90347 2.73739 3.18146 2.89801 2.37887 2.77519 2.98518 4.58477 3.61503
0.16589 4.60994 1.94554 0.61958 0.77255 0.75298 0.63669

601 2.72874 5.16512 2.87498 2.27873 4.02268 3.51019 3.70171 3.94621 1.64533 3.07955 4.21423 2.68048 3.90452 2.68812 2.93151 2.53875 2.42628 3.54016 5.61491 4.22332 951 - -
2.68543 4.42356 2.77575 2.72960 3.46449 2.40632 3.72626 3.29302 2.67645 2.69375 4.24562 2.90400 2.73844 3.17985 2.89502 2.37989 2.77619 2.98604 4.57980 3.61551
0.86407 0.55621 5.26341 2.15022 0.12382 0.60444 0.79049
602 2.41102 4.80158 3.28725 2.62571 3.99774 3.64075 3.34126 2.76024 2.42804 2.46630 3.88546 2.74158 4.02787 3.05134 3.14959 2.64410 1.98522 2.95466 5.33547 4.03810 965 - -
2.68618 4.42225 2.77519 2.73123 3.46354 2.40513 3.72494 3.29354 2.67741 2.69355 4.24690 2.90347 2.73739 3.18146 2.89801 2.37887 2.77519 2.98518 4.58477 3.61503
0.23119 4.70889 1.62255 0.61958 0.77255 0.84494 0.56139
[truncated: 521,352 more chars]
